# Supplementary material for: RedundancyMiner: De-replication of redundant GO categories in microarray and proteomics analysis
Source: BMC Bioinformatics. 2011 Feb 10;12:52. doi: 10.1186/1471-2105-12-52 (PMC3223614; doi:10.1186/1471-2105-12-52)
Supplement: Additional file 8 — Retinal development HTGM download. compressed package of the results of running HTGM on the retinal development genes list. [file 1471-2105-12-52-S8.ZIP › SCENARIO_2_MODIFIED/total.txt.total.txt.dir/Exp1_BestClusterMap_LEIGS_KM_24.csv.join.8.txt.dir/Exp1_BestClusterMap_LEIGS_KM_24.csv.join.8.txt.change.gce.CIM.dir/cgi_user_x.html]

**X-axis Names**   
Cluster is based on euclidean distance  
Cluster method is: average  
plclust  
height plot  

1.GO:0007420\_brain\_development

2.GO:0007417\_central\_nervous\_system\_development

3.GO:0045785\_positive\_regulation\_of\_cell\_adhesion

4.GO:0030155\_regulation\_of\_cell\_adhesion

5.GO:0022610\_biological\_adhesion

6.GO:0007155\_cell\_adhesion

7.GO:0001501\_skeletal\_system\_development

8.GO:0045669\_positive\_regulation\_of\_osteoblast\_differentiation

9.GO:0001655\_urogenital\_system\_development

10.GO:0050880\_regulation\_of\_blood\_vessel\_size

11.GO:0003018\_vascular\_process\_in\_circulatory\_system

12.GO:0035150\_regulation\_of\_tube\_size

13.GO:0045787\_positive\_regulation\_of\_cell\_cycle

14.GO:0048514\_blood\_vessel\_morphogenesis

15.GO:0001945\_lymph\_vessel\_development

16.GO:0048844\_artery\_morphogenesis

17.GO:0060840\_artery\_development

18.GO:0006916\_anti-apoptosis

19.GO:0019800\_peptide\_cross-linking\_via\_chondroitin\_4-sulfate\_glycosaminoglycan

20.GO:0018149\_peptide\_cross-linking

21.GO:0050654\_chondroitin\_sulfate\_proteoglycan\_metabolic\_process

22.GO:0006022\_aminoglycan\_metabolic\_process

23.GO:0030203\_glycosaminoglycan\_metabolic\_process

24.GO:0007507\_heart\_development

25.GO:0048729\_tissue\_morphogenesis

26.GO:0048598\_embryonic\_morphogenesis
