# Supplementary material for: RedundancyMiner: De-replication of redundant GO categories in microarray and proteomics analysis
Source: BMC Bioinformatics. 2011 Feb 10;12:52. doi: 10.1186/1471-2105-12-52 (PMC3223614; doi:10.1186/1471-2105-12-52)
Supplement: Additional file 8 — Retinal development HTGM download. compressed package of the results of running HTGM on the retinal development genes list. [file 1471-2105-12-52-S8.ZIP › SCENARIO_2_MODIFIED/total.txt.total.txt.dir/Exp1_BestClusterMap_LEIGS_KM_24.csv.join.8.txt.dir/Exp1_BestClusterMap_LEIGS_KM_24.csv.join.8.txt.change.gce.CIM.dir/cgi_user_y.html]

**Y-axis Names**   
Cluster is based on euclidean distance  
Cluster method is: average  
plclust  
height plot  

1.FOXC1

2.A930038C07RIK

3.DCN

4.PROX1

5.PRRX1

6.APOE

7.ACTA2

8.PTPRM

9.PLEK

10.CCDC80

11.VAV3

12.BMP2

13.XRCC4

14.SEPT4

15.RORA

16.PRKG1

17.LRP8

18.PCDH18

19.WNT5A

20.ZEB2

21.TBX5

22.RSPO3

23.FOXP4

24.NRP2

25.RB1CC1

26.JUN

27.SGK3

28.ZC3HC1

29.RBP4

30.COL5A2

31.SCHIP1

32.CTNNB1

33.CITED2

34.TGFB2
