# Supplementary material for: RedundancyMiner: De-replication of redundant GO categories in microarray and proteomics analysis
Source: BMC Bioinformatics. 2011 Feb 10;12:52. doi: 10.1186/1471-2105-12-52 (PMC3223614; doi:10.1186/1471-2105-12-52)
Supplement: Additional file 8 — Retinal development HTGM download. compressed package of the results of running HTGM on the retinal development genes list. [file 1471-2105-12-52-S8.ZIP › SCENARIO_2_MODIFIED/total.txt.total.txt.dir/Exp1_BestClusterMap_LEIGS_KM_24.csv.join.8.txt.dir/Exp1_BestClusterMap_LEIGS_KM_24.csv.join.8.txt.change.gce.html]

Gene Category Report for Exp1\_BestClusterMap\_LEIGS\_KM\_24.csv.join.8.txt

# Gene Category Report for Exp1\_BestClusterMap\_LEIGS\_KM\_24.csv.join.8.txt

| HYPERLINKED GO CATEGORY | HYPERLINKED GENE NAME | TOTAL GENES | CHANGED GENES | ENRICHMENT | LOG10(p) | CUMULATIVE NUMBER OF CATEGORIES | CUMULATIVE RANDOMS MEAN | FALSE DISCOVERY RATE |  |
| --- | --- | --- | --- | --- | --- | --- | --- | --- | --- |
| GO:0048844\_artery\_morphogenesis | APOE | 14 | 4 | 24.824798 | -4.842229 | 2 | 0.0 | 0.000000 |  |
| GO:0048844\_artery\_morphogenesis | PRRX1 | 14 | 4 | 24.824798 | -4.842229 | 2 | 0.0 | 0.000000 |  |
| GO:0048844\_artery\_morphogenesis | FOXC1 | 14 | 4 | 24.824798 | -4.842229 | 2 | 0.0 | 0.000000 |  |
| GO:0048844\_artery\_morphogenesis | PROX1 | 14 | 4 | 24.824798 | -4.842229 | 2 | 0.0 | 0.000000 |  |
| GO:0060840\_artery\_development | APOE | 14 | 4 | 24.824798 | -4.842229 | 2 | 0.0 | 0.000000 |  |
| GO:0060840\_artery\_development | PRRX1 | 14 | 4 | 24.824798 | -4.842229 | 2 | 0.0 | 0.000000 |  |
| GO:0060840\_artery\_development | FOXC1 | 14 | 4 | 24.824798 | -4.842229 | 2 | 0.0 | 0.000000 |  |
| GO:0060840\_artery\_development | PROX1 | 14 | 4 | 24.824798 | -4.842229 | 2 | 0.0 | 0.000000 |  |
| GO:0007507\_heart\_development | NRP2 | 195 | 10 | 4.455733 | -4.233734 | 3 | 0.02 | 0.006667 |  |
| GO:0007507\_heart\_development | RBP4 | 195 | 10 | 4.455733 | -4.233734 | 3 | 0.02 | 0.006667 |  |
| GO:0007507\_heart\_development | RB1CC1 | 195 | 10 | 4.455733 | -4.233734 | 3 | 0.02 | 0.006667 |  |
| GO:0007507\_heart\_development | TBX5 | 195 | 10 | 4.455733 | -4.233734 | 3 | 0.02 | 0.006667 |  |
| GO:0007507\_heart\_development | FOXC1 | 195 | 10 | 4.455733 | -4.233734 | 3 | 0.02 | 0.006667 |  |
| GO:0007507\_heart\_development | FOXP4 | 195 | 10 | 4.455733 | -4.233734 | 3 | 0.02 | 0.006667 |  |
| GO:0007507\_heart\_development | PROX1 | 195 | 10 | 4.455733 | -4.233734 | 3 | 0.02 | 0.006667 |  |
| GO:0007507\_heart\_development | CTNNB1 | 195 | 10 | 4.455733 | -4.233734 | 3 | 0.02 | 0.006667 |  |
| GO:0007507\_heart\_development | TGFB2 | 195 | 10 | 4.455733 | -4.233734 | 3 | 0.02 | 0.006667 |  |
| GO:0007507\_heart\_development | CITED2 | 195 | 10 | 4.455733 | -4.233734 | 3 | 0.02 | 0.006667 |  |
| GO:0007417\_central\_nervous\_system\_development | WNT5A | 287 | 12 | 3.632897 | -4.141468 | 4 | 0.02 | 0.005000 |  |
| GO:0007417\_central\_nervous\_system\_development | XRCC4 | 287 | 12 | 3.632897 | -4.141468 | 4 | 0.02 | 0.005000 |  |
| GO:0007417\_central\_nervous\_system\_development | SEPT4 | 287 | 12 | 3.632897 | -4.141468 | 4 | 0.02 | 0.005000 |  |
| GO:0007417\_central\_nervous\_system\_development | BMP2 | 287 | 12 | 3.632897 | -4.141468 | 4 | 0.02 | 0.005000 |  |
| GO:0007417\_central\_nervous\_system\_development | FOXC1 | 287 | 12 | 3.632897 | -4.141468 | 4 | 0.02 | 0.005000 |  |
| GO:0007417\_central\_nervous\_system\_development | LRP8 | 287 | 12 | 3.632897 | -4.141468 | 4 | 0.02 | 0.005000 |  |
| GO:0007417\_central\_nervous\_system\_development | ZEB2 | 287 | 12 | 3.632897 | -4.141468 | 4 | 0.02 | 0.005000 |  |
| GO:0007417\_central\_nervous\_system\_development | RORA | 287 | 12 | 3.632897 | -4.141468 | 4 | 0.02 | 0.005000 |  |
| GO:0007417\_central\_nervous\_system\_development | PRKG1 | 287 | 12 | 3.632897 | -4.141468 | 4 | 0.02 | 0.005000 |  |
| GO:0007417\_central\_nervous\_system\_development | CITED2 | 287 | 12 | 3.632897 | -4.141468 | 4 | 0.02 | 0.005000 |  |
| GO:0007417\_central\_nervous\_system\_development | PCDH18 | 287 | 12 | 3.632897 | -4.141468 | 4 | 0.02 | 0.005000 |  |
| GO:0007417\_central\_nervous\_system\_development | CTNNB1 | 287 | 12 | 3.632897 | -4.141468 | 4 | 0.02 | 0.005000 |  |
| GO:0048513\_organ\_development | WNT5A | 1365 | 28 | 1.782293 | -3.494466 | 5 | 0.28 | 0.056000 |  |
| GO:0048513\_organ\_development | NRP2 | 1365 | 28 | 1.782293 | -3.494466 | 5 | 0.28 | 0.056000 |  |
| GO:0048513\_organ\_development | XRCC4 | 1365 | 28 | 1.782293 | -3.494466 | 5 | 0.28 | 0.056000 |  |
| GO:0048513\_organ\_development | RBP4 | 1365 | 28 | 1.782293 | -3.494466 | 5 | 0.28 | 0.056000 |  |
| GO:0048513\_organ\_development | SCHIP1 | 1365 | 28 | 1.782293 | -3.494466 | 5 | 0.28 | 0.056000 |  |
| GO:0048513\_organ\_development | SEPT4 | 1365 | 28 | 1.782293 | -3.494466 | 5 | 0.28 | 0.056000 |  |
| GO:0048513\_organ\_development | PRRX1 | 1365 | 28 | 1.782293 | -3.494466 | 5 | 0.28 | 0.056000 |  |
| GO:0048513\_organ\_development | ZEB2 | 1365 | 28 | 1.782293 | -3.494466 | 5 | 0.28 | 0.056000 |  |
| GO:0048513\_organ\_development | RORA | 1365 | 28 | 1.782293 | -3.494466 | 5 | 0.28 | 0.056000 |  |
| GO:0048513\_organ\_development | PRKG1 | 1365 | 28 | 1.782293 | -3.494466 | 5 | 0.28 | 0.056000 |  |
| GO:0048513\_organ\_development | TGFB2 | 1365 | 28 | 1.782293 | -3.494466 | 5 | 0.28 | 0.056000 |  |
| GO:0048513\_organ\_development | CTNNB1 | 1365 | 28 | 1.782293 | -3.494466 | 5 | 0.28 | 0.056000 |  |
| GO:0048513\_organ\_development | CITED2 | 1365 | 28 | 1.782293 | -3.494466 | 5 | 0.28 | 0.056000 |  |
| GO:0048513\_organ\_development | APOE | 1365 | 28 | 1.782293 | -3.494466 | 5 | 0.28 | 0.056000 |  |
| GO:0048513\_organ\_development | RSPO3 | 1365 | 28 | 1.782293 | -3.494466 | 5 | 0.28 | 0.056000 |  |
| GO:0048513\_organ\_development | RB1CC1 | 1365 | 28 | 1.782293 | -3.494466 | 5 | 0.28 | 0.056000 |  |
| GO:0048513\_organ\_development | HBA-X | 1365 | 28 | 1.782293 | -3.494466 | 5 | 0.28 | 0.056000 |  |
| GO:0048513\_organ\_development | EGR1 | 1365 | 28 | 1.782293 | -3.494466 | 5 | 0.28 | 0.056000 |  |
| GO:0048513\_organ\_development | INSM1 | 1365 | 28 | 1.782293 | -3.494466 | 5 | 0.28 | 0.056000 |  |
| GO:0048513\_organ\_development | BMP2 | 1365 | 28 | 1.782293 | -3.494466 | 5 | 0.28 | 0.056000 |  |
| GO:0048513\_organ\_development | TBX5 | 1365 | 28 | 1.782293 | -3.494466 | 5 | 0.28 | 0.056000 |  |
| GO:0048513\_organ\_development | DLL1 | 1365 | 28 | 1.782293 | -3.494466 | 5 | 0.28 | 0.056000 |  |
| GO:0048513\_organ\_development | FOXP4 | 1365 | 28 | 1.782293 | -3.494466 | 5 | 0.28 | 0.056000 |  |
| GO:0048513\_organ\_development | PROX1 | 1365 | 28 | 1.782293 | -3.494466 | 5 | 0.28 | 0.056000 |  |
| GO:0048513\_organ\_development | PCDH18 | 1365 | 28 | 1.782293 | -3.494466 | 5 | 0.28 | 0.056000 |  |
| GO:0048513\_organ\_development | JUN | 1365 | 28 | 1.782293 | -3.494466 | 5 | 0.28 | 0.056000 |  |
| GO:0048513\_organ\_development | FOXC1 | 1365 | 28 | 1.782293 | -3.494466 | 5 | 0.28 | 0.056000 |  |
| GO:0048513\_organ\_development | LRP8 | 1365 | 28 | 1.782293 | -3.494466 | 5 | 0.28 | 0.056000 |  |
| GO:0003018\_vascular\_process\_in\_circulatory\_system | PTPRM | 31 | 4 | 11.211199 | -3.407795 | 8 | 0.37 | 0.046250 |  |
| GO:0003018\_vascular\_process\_in\_circulatory\_system | APOE | 31 | 4 | 11.211199 | -3.407795 | 8 | 0.37 | 0.046250 |  |
| GO:0003018\_vascular\_process\_in\_circulatory\_system | ACTA2 | 31 | 4 | 11.211199 | -3.407795 | 8 | 0.37 | 0.046250 |  |
| GO:0003018\_vascular\_process\_in\_circulatory\_system | FOXC1 | 31 | 4 | 11.211199 | -3.407795 | 8 | 0.37 | 0.046250 |  |
| GO:0035150\_regulation\_of\_tube\_size | PTPRM | 31 | 4 | 11.211199 | -3.407795 | 8 | 0.37 | 0.046250 |  |
| GO:0035150\_regulation\_of\_tube\_size | APOE | 31 | 4 | 11.211199 | -3.407795 | 8 | 0.37 | 0.046250 |  |
| GO:0035150\_regulation\_of\_tube\_size | ACTA2 | 31 | 4 | 11.211199 | -3.407795 | 8 | 0.37 | 0.046250 |  |
| GO:0035150\_regulation\_of\_tube\_size | FOXC1 | 31 | 4 | 11.211199 | -3.407795 | 8 | 0.37 | 0.046250 |  |
| GO:0050880\_regulation\_of\_blood\_vessel\_size | PTPRM | 31 | 4 | 11.211199 | -3.407795 | 8 | 0.37 | 0.046250 |  |
| GO:0050880\_regulation\_of\_blood\_vessel\_size | APOE | 31 | 4 | 11.211199 | -3.407795 | 8 | 0.37 | 0.046250 |  |
| GO:0050880\_regulation\_of\_blood\_vessel\_size | ACTA2 | 31 | 4 | 11.211199 | -3.407795 | 8 | 0.37 | 0.046250 |  |
| GO:0050880\_regulation\_of\_blood\_vessel\_size | FOXC1 | 31 | 4 | 11.211199 | -3.407795 | 8 | 0.37 | 0.046250 |  |
| GO:0060157\_urinary\_bladder\_development | WNT5A | 4 | 2 |  |  |  |  |  |  |
| GO:0060157\_urinary\_bladder\_development | RBP4 | 4 | 2 |  |  |  |  |  |  |
| GO:0045785\_positive\_regulation\_of\_cell\_adhesion | A930038C07RIK | 37 | 4 | 9.393167 | -3.107952 | 9 | 0.72 | 0.080000 |  |
| GO:0045785\_positive\_regulation\_of\_cell\_adhesion | VAV3 | 37 | 4 | 9.393167 | -3.107952 | 9 | 0.72 | 0.080000 |  |
| GO:0045785\_positive\_regulation\_of\_cell\_adhesion | CCDC80 | 37 | 4 | 9.393167 | -3.107952 | 9 | 0.72 | 0.080000 |  |
| GO:0045785\_positive\_regulation\_of\_cell\_adhesion | CITED2 | 37 | 4 | 9.393167 | -3.107952 | 9 | 0.72 | 0.080000 |  |
| GO:0006022\_aminoglycan\_metabolic\_process | A930038C07RIK | 18 | 3 | 14.481132 | -2.983025 | 11 | 0.83 | 0.075455 |  |
| GO:0006022\_aminoglycan\_metabolic\_process | FOXC1 | 18 | 3 | 14.481132 | -2.983025 | 11 | 0.83 | 0.075455 |  |
| GO:0006022\_aminoglycan\_metabolic\_process | DCN | 18 | 3 | 14.481132 | -2.983025 | 11 | 0.83 | 0.075455 |  |
| GO:0030203\_glycosaminoglycan\_metabolic\_process | A930038C07RIK | 18 | 3 | 14.481132 | -2.983025 | 11 | 0.83 | 0.075455 |  |
| GO:0030203\_glycosaminoglycan\_metabolic\_process | FOXC1 | 18 | 3 | 14.481132 | -2.983025 | 11 | 0.83 | 0.075455 |  |
| GO:0030203\_glycosaminoglycan\_metabolic\_process | DCN | 18 | 3 | 14.481132 | -2.983025 | 11 | 0.83 | 0.075455 |  |
| GO:0007420\_brain\_development | WNT5A | 231 | 9 | 3.385200 | -2.958542 | 12 | 0.86 | 0.071667 |  |
| GO:0007420\_brain\_development | SEPT4 | 231 | 9 | 3.385200 | -2.958542 | 12 | 0.86 | 0.071667 |  |
| GO:0007420\_brain\_development | FOXC1 | 231 | 9 | 3.385200 | -2.958542 | 12 | 0.86 | 0.071667 |  |
| GO:0007420\_brain\_development | LRP8 | 231 | 9 | 3.385200 | -2.958542 | 12 | 0.86 | 0.071667 |  |
| GO:0007420\_brain\_development | ZEB2 | 231 | 9 | 3.385200 | -2.958542 | 12 | 0.86 | 0.071667 |  |
| GO:0007420\_brain\_development | RORA | 231 | 9 | 3.385200 | -2.958542 | 12 | 0.86 | 0.071667 |  |
| GO:0007420\_brain\_development | PRKG1 | 231 | 9 | 3.385200 | -2.958542 | 12 | 0.86 | 0.071667 |  |
| GO:0007420\_brain\_development | PCDH18 | 231 | 9 | 3.385200 | -2.958542 | 12 | 0.86 | 0.071667 |  |
| GO:0007420\_brain\_development | CTNNB1 | 231 | 9 | 3.385200 | -2.958542 | 12 | 0.86 | 0.071667 |  |
| GO:0001945\_lymph\_vessel\_development | FOXC1 | 5 | 2 | 34.754717 | -2.895738 | 13 | 1.33 | 0.102308 |  |
| GO:0001945\_lymph\_vessel\_development | PROX1 | 5 | 2 | 34.754717 | -2.895738 | 13 | 1.33 | 0.102308 |  |
| GO:0048522\_positive\_regulation\_of\_cellular\_process | RBP4 | 895 | 20 | 1.941604 | -2.850254 | 14 | 1.43 | 0.102143 |  |
| GO:0048522\_positive\_regulation\_of\_cellular\_process | XRCC4 | 895 | 20 | 1.941604 | -2.850254 | 14 | 1.43 | 0.102143 |  |
| GO:0048522\_positive\_regulation\_of\_cellular\_process | BMP2 | 895 | 20 | 1.941604 | -2.850254 | 14 | 1.43 | 0.102143 |  |
| GO:0048522\_positive\_regulation\_of\_cellular\_process | VAV3 | 895 | 20 | 1.941604 | -2.850254 | 14 | 1.43 | 0.102143 |  |
| GO:0048522\_positive\_regulation\_of\_cellular\_process | KLF12 | 895 | 20 | 1.941604 | -2.850254 | 14 | 1.43 | 0.102143 |  |
| GO:0048522\_positive\_regulation\_of\_cellular\_process | PLEK | 895 | 20 | 1.941604 | -2.850254 | 14 | 1.43 | 0.102143 |  |
| GO:0048522\_positive\_regulation\_of\_cellular\_process | TBX5 | 895 | 20 | 1.941604 | -2.850254 | 14 | 1.43 | 0.102143 |  |
| GO:0048522\_positive\_regulation\_of\_cellular\_process | CCDC80 | 895 | 20 | 1.941604 | -2.850254 | 14 | 1.43 | 0.102143 |  |
| GO:0048522\_positive\_regulation\_of\_cellular\_process | PRRX1 | 895 | 20 | 1.941604 | -2.850254 | 14 | 1.43 | 0.102143 |  |
| GO:0048522\_positive\_regulation\_of\_cellular\_process | ZEB2 | 895 | 20 | 1.941604 | -2.850254 | 14 | 1.43 | 0.102143 |  |
| GO:0048522\_positive\_regulation\_of\_cellular\_process | RORA | 895 | 20 | 1.941604 | -2.850254 | 14 | 1.43 | 0.102143 |  |
| GO:0048522\_positive\_regulation\_of\_cellular\_process | PROX1 | 895 | 20 | 1.941604 | -2.850254 | 14 | 1.43 | 0.102143 |  |
| GO:0048522\_positive\_regulation\_of\_cellular\_process | CTNNB1 | 895 | 20 | 1.941604 | -2.850254 | 14 | 1.43 | 0.102143 |  |
| GO:0048522\_positive\_regulation\_of\_cellular\_process | CITED2 | 895 | 20 | 1.941604 | -2.850254 | 14 | 1.43 | 0.102143 |  |
| GO:0048522\_positive\_regulation\_of\_cellular\_process | TGFB2 | 895 | 20 | 1.941604 | -2.850254 | 14 | 1.43 | 0.102143 |  |
| GO:0048522\_positive\_regulation\_of\_cellular\_process | A930038C07RIK | 895 | 20 | 1.941604 | -2.850254 | 14 | 1.43 | 0.102143 |  |
| GO:0048522\_positive\_regulation\_of\_cellular\_process | APOE | 895 | 20 | 1.941604 | -2.850254 | 14 | 1.43 | 0.102143 |  |
| GO:0048522\_positive\_regulation\_of\_cellular\_process | JUN | 895 | 20 | 1.941604 | -2.850254 | 14 | 1.43 | 0.102143 |  |
| GO:0048522\_positive\_regulation\_of\_cellular\_process | RB1CC1 | 895 | 20 | 1.941604 | -2.850254 | 14 | 1.43 | 0.102143 |  |
| GO:0048522\_positive\_regulation\_of\_cellular\_process | FOXC1 | 895 | 20 | 1.941604 | -2.850254 | 14 | 1.43 | 0.102143 |  |
| GO:0048598\_embryonic\_morphogenesis | WNT5A | 299 | 10 | 2.905913 | -2.737875 | 15 | 1.66 | 0.110667 |  |
| GO:0048598\_embryonic\_morphogenesis | RBP4 | 299 | 10 | 2.905913 | -2.737875 | 15 | 1.66 | 0.110667 |  |
| GO:0048598\_embryonic\_morphogenesis | RSPO3 | 299 | 10 | 2.905913 | -2.737875 | 15 | 1.66 | 0.110667 |  |
| GO:0048598\_embryonic\_morphogenesis | TBX5 | 299 | 10 | 2.905913 | -2.737875 | 15 | 1.66 | 0.110667 |  |
| GO:0048598\_embryonic\_morphogenesis | PRRX1 | 299 | 10 | 2.905913 | -2.737875 | 15 | 1.66 | 0.110667 |  |
| GO:0048598\_embryonic\_morphogenesis | ZEB2 | 299 | 10 | 2.905913 | -2.737875 | 15 | 1.66 | 0.110667 |  |
| GO:0048598\_embryonic\_morphogenesis | FOXC1 | 299 | 10 | 2.905913 | -2.737875 | 15 | 1.66 | 0.110667 |  |
| GO:0048598\_embryonic\_morphogenesis | FOXP4 | 299 | 10 | 2.905913 | -2.737875 | 15 | 1.66 | 0.110667 |  |
| GO:0048598\_embryonic\_morphogenesis | PROX1 | 299 | 10 | 2.905913 | -2.737875 | 15 | 1.66 | 0.110667 |  |
| GO:0048598\_embryonic\_morphogenesis | CTNNB1 | 299 | 10 | 2.905913 | -2.737875 | 15 | 1.66 | 0.110667 |  |
| GO:0009888\_tissue\_development | WNT5A | 525 | 14 | 2.316981 | -2.731649 | 16 | 1.66 | 0.103750 |  |
| GO:0009888\_tissue\_development | NRP2 | 525 | 14 | 2.316981 | -2.731649 | 16 | 1.66 | 0.103750 |  |
| GO:0009888\_tissue\_development | SCHIP1 | 525 | 14 | 2.316981 | -2.731649 | 16 | 1.66 | 0.103750 |  |
| GO:0009888\_tissue\_development | RBP4 | 525 | 14 | 2.316981 | -2.731649 | 16 | 1.66 | 0.103750 |  |
| GO:0009888\_tissue\_development | BMP2 | 525 | 14 | 2.316981 | -2.731649 | 16 | 1.66 | 0.103750 |  |
| GO:0009888\_tissue\_development | TBX5 | 525 | 14 | 2.316981 | -2.731649 | 16 | 1.66 | 0.103750 |  |
| GO:0009888\_tissue\_development | PRRX1 | 525 | 14 | 2.316981 | -2.731649 | 16 | 1.66 | 0.103750 |  |
| GO:0009888\_tissue\_development | ZEB2 | 525 | 14 | 2.316981 | -2.731649 | 16 | 1.66 | 0.103750 |  |
| GO:0009888\_tissue\_development | PROX1 | 525 | 14 | 2.316981 | -2.731649 | 16 | 1.66 | 0.103750 |  |
| GO:0009888\_tissue\_development | TGFB2 | 525 | 14 | 2.316981 | -2.731649 | 16 | 1.66 | 0.103750 |  |
| GO:0009888\_tissue\_development | CTNNB1 | 525 | 14 | 2.316981 | -2.731649 | 16 | 1.66 | 0.103750 |  |
| GO:0009888\_tissue\_development | CITED2 | 525 | 14 | 2.316981 | -2.731649 | 16 | 1.66 | 0.103750 |  |
| GO:0009888\_tissue\_development | JUN | 525 | 14 | 2.316981 | -2.731649 | 16 | 1.66 | 0.103750 |  |
| GO:0009888\_tissue\_development | FOXC1 | 525 | 14 | 2.316981 | -2.731649 | 16 | 1.66 | 0.103750 |  |
| GO:0045787\_positive\_regulation\_of\_cell\_cycle | PROX1 | 22 | 3 | 11.848199 | -2.721317 | 17 | 2.22 | 0.130588 |  |
| GO:0045787\_positive\_regulation\_of\_cell\_cycle | TGFB2 | 22 | 3 | 11.848199 | -2.721317 | 17 | 2.22 | 0.130588 |  |
| GO:0045787\_positive\_regulation\_of\_cell\_cycle | CITED2 | 22 | 3 | 11.848199 | -2.721317 | 17 | 2.22 | 0.130588 |  |
| GO:0048514\_blood\_vessel\_morphogenesis | APOE | 158 | 7 | 3.849415 | -2.698837 | 18 | 2.29 | 0.127222 |  |
| GO:0048514\_blood\_vessel\_morphogenesis | PRRX1 | 158 | 7 | 3.849415 | -2.698837 | 18 | 2.29 | 0.127222 |  |
| GO:0048514\_blood\_vessel\_morphogenesis | FOXC1 | 158 | 7 | 3.849415 | -2.698837 | 18 | 2.29 | 0.127222 |  |
| GO:0048514\_blood\_vessel\_morphogenesis | PROX1 | 158 | 7 | 3.849415 | -2.698837 | 18 | 2.29 | 0.127222 |  |
| GO:0048514\_blood\_vessel\_morphogenesis | TGFB2 | 158 | 7 | 3.849415 | -2.698837 | 18 | 2.29 | 0.127222 |  |
| GO:0048514\_blood\_vessel\_morphogenesis | CITED2 | 158 | 7 | 3.849415 | -2.698837 | 18 | 2.29 | 0.127222 |  |
| GO:0048514\_blood\_vessel\_morphogenesis | CTNNB1 | 158 | 7 | 3.849415 | -2.698837 | 18 | 2.29 | 0.127222 |  |
| GO:0048518\_positive\_regulation\_of\_biological\_process | RBP4 | 995 | 21 | 1.833792 | -2.668663 | 19 | 2.31 | 0.121579 |  |
| GO:0048518\_positive\_regulation\_of\_biological\_process | XRCC4 | 995 | 21 | 1.833792 | -2.668663 | 19 | 2.31 | 0.121579 |  |
| GO:0048518\_positive\_regulation\_of\_biological\_process | BMP2 | 995 | 21 | 1.833792 | -2.668663 | 19 | 2.31 | 0.121579 |  |
| GO:0048518\_positive\_regulation\_of\_biological\_process | PTPRM | 995 | 21 | 1.833792 | -2.668663 | 19 | 2.31 | 0.121579 |  |
| GO:0048518\_positive\_regulation\_of\_biological\_process | VAV3 | 995 | 21 | 1.833792 | -2.668663 | 19 | 2.31 | 0.121579 |  |
| GO:0048518\_positive\_regulation\_of\_biological\_process | KLF12 | 995 | 21 | 1.833792 | -2.668663 | 19 | 2.31 | 0.121579 |  |
| GO:0048518\_positive\_regulation\_of\_biological\_process | PLEK | 995 | 21 | 1.833792 | -2.668663 | 19 | 2.31 | 0.121579 |  |
| GO:0048518\_positive\_regulation\_of\_biological\_process | TBX5 | 995 | 21 | 1.833792 | -2.668663 | 19 | 2.31 | 0.121579 |  |
| GO:0048518\_positive\_regulation\_of\_biological\_process | CCDC80 | 995 | 21 | 1.833792 | -2.668663 | 19 | 2.31 | 0.121579 |  |
| GO:0048518\_positive\_regulation\_of\_biological\_process | PRRX1 | 995 | 21 | 1.833792 | -2.668663 | 19 | 2.31 | 0.121579 |  |
| GO:0048518\_positive\_regulation\_of\_biological\_process | ZEB2 | 995 | 21 | 1.833792 | -2.668663 | 19 | 2.31 | 0.121579 |  |
| GO:0048518\_positive\_regulation\_of\_biological\_process | RORA | 995 | 21 | 1.833792 | -2.668663 | 19 | 2.31 | 0.121579 |  |
| GO:0048518\_positive\_regulation\_of\_biological\_process | PROX1 | 995 | 21 | 1.833792 | -2.668663 | 19 | 2.31 | 0.121579 |  |
| GO:0048518\_positive\_regulation\_of\_biological\_process | TGFB2 | 995 | 21 | 1.833792 | -2.668663 | 19 | 2.31 | 0.121579 |  |
| GO:0048518\_positive\_regulation\_of\_biological\_process | CITED2 | 995 | 21 | 1.833792 | -2.668663 | 19 | 2.31 | 0.121579 |  |
| GO:0048518\_positive\_regulation\_of\_biological\_process | CTNNB1 | 995 | 21 | 1.833792 | -2.668663 | 19 | 2.31 | 0.121579 |  |
| GO:0048518\_positive\_regulation\_of\_biological\_process | A930038C07RIK | 995 | 21 | 1.833792 | -2.668663 | 19 | 2.31 | 0.121579 |  |
| GO:0048518\_positive\_regulation\_of\_biological\_process | APOE | 995 | 21 | 1.833792 | -2.668663 | 19 | 2.31 | 0.121579 |  |
| GO:0048518\_positive\_regulation\_of\_biological\_process | JUN | 995 | 21 | 1.833792 | -2.668663 | 19 | 2.31 | 0.121579 |  |
| GO:0048518\_positive\_regulation\_of\_biological\_process | RB1CC1 | 995 | 21 | 1.833792 | -2.668663 | 19 | 2.31 | 0.121579 |  |
| GO:0048518\_positive\_regulation\_of\_biological\_process | FOXC1 | 995 | 21 | 1.833792 | -2.668663 | 19 | 2.31 | 0.121579 |  |
| GO:0048729\_tissue\_morphogenesis | WNT5A | 255 | 9 | 3.066593 | -2.657764 | 20 | 2.32 | 0.116000 |  |
| GO:0048729\_tissue\_morphogenesis | BMP2 | 255 | 9 | 3.066593 | -2.657764 | 20 | 2.32 | 0.116000 |  |
| GO:0048729\_tissue\_morphogenesis | TBX5 | 255 | 9 | 3.066593 | -2.657764 | 20 | 2.32 | 0.116000 |  |
| GO:0048729\_tissue\_morphogenesis | JUN | 255 | 9 | 3.066593 | -2.657764 | 20 | 2.32 | 0.116000 |  |
| GO:0048729\_tissue\_morphogenesis | FOXC1 | 255 | 9 | 3.066593 | -2.657764 | 20 | 2.32 | 0.116000 |  |
| GO:0048729\_tissue\_morphogenesis | ZEB2 | 255 | 9 | 3.066593 | -2.657764 | 20 | 2.32 | 0.116000 |  |
| GO:0048729\_tissue\_morphogenesis | PROX1 | 255 | 9 | 3.066593 | -2.657764 | 20 | 2.32 | 0.116000 |  |
| GO:0048729\_tissue\_morphogenesis | TGFB2 | 255 | 9 | 3.066593 | -2.657764 | 20 | 2.32 | 0.116000 |  |
| GO:0048729\_tissue\_morphogenesis | CTNNB1 | 255 | 9 | 3.066593 | -2.657764 | 20 | 2.32 | 0.116000 |  |
| GO:0048856\_anatomical\_structure\_development | WNT5A | 1688 | 30 | 1.544197 | -2.635741 | 21 | 2.4 | 0.114286 |  |
| GO:0048856\_anatomical\_structure\_development | RHOJ | 1688 | 30 | 1.544197 | -2.635741 | 21 | 2.4 | 0.114286 |  |
| GO:0048856\_anatomical\_structure\_development | NRP2 | 1688 | 30 | 1.544197 | -2.635741 | 21 | 2.4 | 0.114286 |  |
| GO:0048856\_anatomical\_structure\_development | XRCC4 | 1688 | 30 | 1.544197 | -2.635741 | 21 | 2.4 | 0.114286 |  |
| GO:0048856\_anatomical\_structure\_development | SCHIP1 | 1688 | 30 | 1.544197 | -2.635741 | 21 | 2.4 | 0.114286 |  |
| GO:0048856\_anatomical\_structure\_development | RBP4 | 1688 | 30 | 1.544197 | -2.635741 | 21 | 2.4 | 0.114286 |  |
| GO:0048856\_anatomical\_structure\_development | SEPT4 | 1688 | 30 | 1.544197 | -2.635741 | 21 | 2.4 | 0.114286 |  |
| GO:0048856\_anatomical\_structure\_development | PRRX1 | 1688 | 30 | 1.544197 | -2.635741 | 21 | 2.4 | 0.114286 |  |
| GO:0048856\_anatomical\_structure\_development | ZEB2 | 1688 | 30 | 1.544197 | -2.635741 | 21 | 2.4 | 0.114286 |  |
| GO:0048856\_anatomical\_structure\_development | RORA | 1688 | 30 | 1.544197 | -2.635741 | 21 | 2.4 | 0.114286 |  |
| GO:0048856\_anatomical\_structure\_development | PRKG1 | 1688 | 30 | 1.544197 | -2.635741 | 21 | 2.4 | 0.114286 |  |
| GO:0048856\_anatomical\_structure\_development | TGFB2 | 1688 | 30 | 1.544197 | -2.635741 | 21 | 2.4 | 0.114286 |  |
| GO:0048856\_anatomical\_structure\_development | CTNNB1 | 1688 | 30 | 1.544197 | -2.635741 | 21 | 2.4 | 0.114286 |  |
| GO:0048856\_anatomical\_structure\_development | CITED2 | 1688 | 30 | 1.544197 | -2.635741 | 21 | 2.4 | 0.114286 |  |
| GO:0048856\_anatomical\_structure\_development | APOE | 1688 | 30 | 1.544197 | -2.635741 | 21 | 2.4 | 0.114286 |  |
| GO:0048856\_anatomical\_structure\_development | RSPO3 | 1688 | 30 | 1.544197 | -2.635741 | 21 | 2.4 | 0.114286 |  |
| GO:0048856\_anatomical\_structure\_development | RB1CC1 | 1688 | 30 | 1.544197 | -2.635741 | 21 | 2.4 | 0.114286 |  |
| GO:0048856\_anatomical\_structure\_development | HBA-X | 1688 | 30 | 1.544197 | -2.635741 | 21 | 2.4 | 0.114286 |  |
| GO:0048856\_anatomical\_structure\_development | EGR1 | 1688 | 30 | 1.544197 | -2.635741 | 21 | 2.4 | 0.114286 |  |
| GO:0048856\_anatomical\_structure\_development | INSM1 | 1688 | 30 | 1.544197 | -2.635741 | 21 | 2.4 | 0.114286 |  |
| GO:0048856\_anatomical\_structure\_development | BMP2 | 1688 | 30 | 1.544197 | -2.635741 | 21 | 2.4 | 0.114286 |  |
| GO:0048856\_anatomical\_structure\_development | TBX5 | 1688 | 30 | 1.544197 | -2.635741 | 21 | 2.4 | 0.114286 |  |
| GO:0048856\_anatomical\_structure\_development | DLL1 | 1688 | 30 | 1.544197 | -2.635741 | 21 | 2.4 | 0.114286 |  |
| GO:0048856\_anatomical\_structure\_development | COL5A2 | 1688 | 30 | 1.544197 | -2.635741 | 21 | 2.4 | 0.114286 |  |
| GO:0048856\_anatomical\_structure\_development | FOXP4 | 1688 | 30 | 1.544197 | -2.635741 | 21 | 2.4 | 0.114286 |  |
| GO:0048856\_anatomical\_structure\_development | PROX1 | 1688 | 30 | 1.544197 | -2.635741 | 21 | 2.4 | 0.114286 |  |
| GO:0048856\_anatomical\_structure\_development | PCDH18 | 1688 | 30 | 1.544197 | -2.635741 | 21 | 2.4 | 0.114286 |  |
| GO:0048856\_anatomical\_structure\_development | JUN | 1688 | 30 | 1.544197 | -2.635741 | 21 | 2.4 | 0.114286 |  |
| GO:0048856\_anatomical\_structure\_development | FOXC1 | 1688 | 30 | 1.544197 | -2.635741 | 21 | 2.4 | 0.114286 |  |
| GO:0048856\_anatomical\_structure\_development | LRP8 | 1688 | 30 | 1.544197 | -2.635741 | 21 | 2.4 | 0.114286 |  |
| GO:0048731\_system\_development | NRP2 | 1609 | 29 | 1.566014 | -2.626124 | 22 | 2.41 | 0.109545 |  |
| GO:0048731\_system\_development | WNT5A | 1609 | 29 | 1.566014 | -2.626124 | 22 | 2.41 | 0.109545 |  |
| GO:0048731\_system\_development | XRCC4 | 1609 | 29 | 1.566014 | -2.626124 | 22 | 2.41 | 0.109545 |  |
| GO:0048731\_system\_development | RBP4 | 1609 | 29 | 1.566014 | -2.626124 | 22 | 2.41 | 0.109545 |  |
| GO:0048731\_system\_development | SCHIP1 | 1609 | 29 | 1.566014 | -2.626124 | 22 | 2.41 | 0.109545 |  |
| GO:0048731\_system\_development | SEPT4 | 1609 | 29 | 1.566014 | -2.626124 | 22 | 2.41 | 0.109545 |  |
| GO:0048731\_system\_development | PRRX1 | 1609 | 29 | 1.566014 | -2.626124 | 22 | 2.41 | 0.109545 |  |
| GO:0048731\_system\_development | ZEB2 | 1609 | 29 | 1.566014 | -2.626124 | 22 | 2.41 | 0.109545 |  |
| GO:0048731\_system\_development | RORA | 1609 | 29 | 1.566014 | -2.626124 | 22 | 2.41 | 0.109545 |  |
| GO:0048731\_system\_development | PRKG1 | 1609 | 29 | 1.566014 | -2.626124 | 22 | 2.41 | 0.109545 |  |
| GO:0048731\_system\_development | CTNNB1 | 1609 | 29 | 1.566014 | -2.626124 | 22 | 2.41 | 0.109545 |  |
| GO:0048731\_system\_development | TGFB2 | 1609 | 29 | 1.566014 | -2.626124 | 22 | 2.41 | 0.109545 |  |
| GO:0048731\_system\_development | CITED2 | 1609 | 29 | 1.566014 | -2.626124 | 22 | 2.41 | 0.109545 |  |
| GO:0048731\_system\_development | APOE | 1609 | 29 | 1.566014 | -2.626124 | 22 | 2.41 | 0.109545 |  |
| GO:0048731\_system\_development | RB1CC1 | 1609 | 29 | 1.566014 | -2.626124 | 22 | 2.41 | 0.109545 |  |
| GO:0048731\_system\_development | RSPO3 | 1609 | 29 | 1.566014 | -2.626124 | 22 | 2.41 | 0.109545 |  |
| GO:0048731\_system\_development | HBA-X | 1609 | 29 | 1.566014 | -2.626124 | 22 | 2.41 | 0.109545 |  |
| GO:0048731\_system\_development | EGR1 | 1609 | 29 | 1.566014 | -2.626124 | 22 | 2.41 | 0.109545 |  |
| GO:0048731\_system\_development | INSM1 | 1609 | 29 | 1.566014 | -2.626124 | 22 | 2.41 | 0.109545 |  |
| GO:0048731\_system\_development | BMP2 | 1609 | 29 | 1.566014 | -2.626124 | 22 | 2.41 | 0.109545 |  |
| GO:0048731\_system\_development | TBX5 | 1609 | 29 | 1.566014 | -2.626124 | 22 | 2.41 | 0.109545 |  |
| GO:0048731\_system\_development | DLL1 | 1609 | 29 | 1.566014 | -2.626124 | 22 | 2.41 | 0.109545 |  |
| GO:0048731\_system\_development | FOXP4 | 1609 | 29 | 1.566014 | -2.626124 | 22 | 2.41 | 0.109545 |  |
| GO:0048731\_system\_development | COL5A2 | 1609 | 29 | 1.566014 | -2.626124 | 22 | 2.41 | 0.109545 |  |
| GO:0048731\_system\_development | PROX1 | 1609 | 29 | 1.566014 | -2.626124 | 22 | 2.41 | 0.109545 |  |
| GO:0048731\_system\_development | PCDH18 | 1609 | 29 | 1.566014 | -2.626124 | 22 | 2.41 | 0.109545 |  |
| GO:0048731\_system\_development | JUN | 1609 | 29 | 1.566014 | -2.626124 | 22 | 2.41 | 0.109545 |  |
| GO:0048731\_system\_development | FOXC1 | 1609 | 29 | 1.566014 | -2.626124 | 22 | 2.41 | 0.109545 |  |
| GO:0048731\_system\_development | LRP8 | 1609 | 29 | 1.566014 | -2.626124 | 22 | 2.41 | 0.109545 |  |
| GO:0019800\_peptide\_cross-linking\_via\_chondroitin\_4-sulfate\_glycosaminoglycan | A930038C07RIK | 7 | 2 | 24.824798 | -2.579941 | 23 | 3.09 | 0.134348 |  |
| GO:0019800\_peptide\_cross-linking\_via\_chondroitin\_4-sulfate\_glycosaminoglycan | DCN | 7 | 2 | 24.824798 | -2.579941 | 23 | 3.09 | 0.134348 |  |
| GO:0001655\_urogenital\_system\_development | WNT5A | 128 | 6 | 4.072818 | -2.491239 | 24 | 3.42 | 0.142500 |  |
| GO:0001655\_urogenital\_system\_development | RBP4 | 128 | 6 | 4.072818 | -2.491239 | 24 | 3.42 | 0.142500 |  |
| GO:0001655\_urogenital\_system\_development | SCHIP1 | 128 | 6 | 4.072818 | -2.491239 | 24 | 3.42 | 0.142500 |  |
| GO:0001655\_urogenital\_system\_development | BMP2 | 128 | 6 | 4.072818 | -2.491239 | 24 | 3.42 | 0.142500 |  |
| GO:0001655\_urogenital\_system\_development | FOXC1 | 128 | 6 | 4.072818 | -2.491239 | 24 | 3.42 | 0.142500 |  |
| GO:0001655\_urogenital\_system\_development | CTNNB1 | 128 | 6 | 4.072818 | -2.491239 | 24 | 3.42 | 0.142500 |  |
| GO:0007399\_nervous\_system\_development | WNT5A | 621 | 15 | 2.098715 | -2.480383 | 25 | 3.49 | 0.139600 |  |
| GO:0007399\_nervous\_system\_development | SEPT4 | 621 | 15 | 2.098715 | -2.480383 | 25 | 3.49 | 0.139600 |  |
| GO:0007399\_nervous\_system\_development | XRCC4 | 621 | 15 | 2.098715 | -2.480383 | 25 | 3.49 | 0.139600 |  |
| GO:0007399\_nervous\_system\_development | BMP2 | 621 | 15 | 2.098715 | -2.480383 | 25 | 3.49 | 0.139600 |  |
| GO:0007399\_nervous\_system\_development | ZEB2 | 621 | 15 | 2.098715 | -2.480383 | 25 | 3.49 | 0.139600 |  |
| GO:0007399\_nervous\_system\_development | DLL1 | 621 | 15 | 2.098715 | -2.480383 | 25 | 3.49 | 0.139600 |  |
| GO:0007399\_nervous\_system\_development | RORA | 621 | 15 | 2.098715 | -2.480383 | 25 | 3.49 | 0.139600 |  |
| GO:0007399\_nervous\_system\_development | PRKG1 | 621 | 15 | 2.098715 | -2.480383 | 25 | 3.49 | 0.139600 |  |
| GO:0007399\_nervous\_system\_development | TGFB2 | 621 | 15 | 2.098715 | -2.480383 | 25 | 3.49 | 0.139600 |  |
| GO:0007399\_nervous\_system\_development | CTNNB1 | 621 | 15 | 2.098715 | -2.480383 | 25 | 3.49 | 0.139600 |  |
| GO:0007399\_nervous\_system\_development | PCDH18 | 621 | 15 | 2.098715 | -2.480383 | 25 | 3.49 | 0.139600 |  |
| GO:0007399\_nervous\_system\_development | CITED2 | 621 | 15 | 2.098715 | -2.480383 | 25 | 3.49 | 0.139600 |  |
| GO:0007399\_nervous\_system\_development | APOE | 621 | 15 | 2.098715 | -2.480383 | 25 | 3.49 | 0.139600 |  |
| GO:0007399\_nervous\_system\_development | LRP8 | 621 | 15 | 2.098715 | -2.480383 | 25 | 3.49 | 0.139600 |  |
| GO:0007399\_nervous\_system\_development | FOXC1 | 621 | 15 | 2.098715 | -2.480383 | 25 | 3.49 | 0.139600 |  |
| GO:0030204\_chondroitin\_sulfate\_metabolic\_process | A930038C07RIK | 8 | 2 | 21.721698 | -2.458209 | 26 | 4.06 | 0.156154 |  |
| GO:0030204\_chondroitin\_sulfate\_metabolic\_process | DCN | 8 | 2 | 21.721698 | -2.458209 | 26 | 4.06 | 0.156154 |  |
| GO:0009790\_embryonic\_development | WNT5A | 567 | 14 | 2.145353 | -2.415966 | 27 | 4.18 | 0.154815 |  |
| GO:0009790\_embryonic\_development | XRCC4 | 567 | 14 | 2.145353 | -2.415966 | 27 | 4.18 | 0.154815 |  |
| GO:0009790\_embryonic\_development | RBP4 | 567 | 14 | 2.145353 | -2.415966 | 27 | 4.18 | 0.154815 |  |
| GO:0009790\_embryonic\_development | BMP2 | 567 | 14 | 2.145353 | -2.415966 | 27 | 4.18 | 0.154815 |  |
| GO:0009790\_embryonic\_development | TBX5 | 567 | 14 | 2.145353 | -2.415966 | 27 | 4.18 | 0.154815 |  |
| GO:0009790\_embryonic\_development | PRRX1 | 567 | 14 | 2.145353 | -2.415966 | 27 | 4.18 | 0.154815 |  |
| GO:0009790\_embryonic\_development | DLL1 | 567 | 14 | 2.145353 | -2.415966 | 27 | 4.18 | 0.154815 |  |
| GO:0009790\_embryonic\_development | ZEB2 | 567 | 14 | 2.145353 | -2.415966 | 27 | 4.18 | 0.154815 |  |
| GO:0009790\_embryonic\_development | PROX1 | 567 | 14 | 2.145353 | -2.415966 | 27 | 4.18 | 0.154815 |  |
| GO:0009790\_embryonic\_development | FOXP4 | 567 | 14 | 2.145353 | -2.415966 | 27 | 4.18 | 0.154815 |  |
| GO:0009790\_embryonic\_development | CITED2 | 567 | 14 | 2.145353 | -2.415966 | 27 | 4.18 | 0.154815 |  |
| GO:0009790\_embryonic\_development | CTNNB1 | 567 | 14 | 2.145353 | -2.415966 | 27 | 4.18 | 0.154815 |  |
| GO:0009790\_embryonic\_development | RSPO3 | 567 | 14 | 2.145353 | -2.415966 | 27 | 4.18 | 0.154815 |  |
| GO:0009790\_embryonic\_development | FOXC1 | 567 | 14 | 2.145353 | -2.415966 | 27 | 4.18 | 0.154815 |  |
| GO:0048646\_anatomical\_structure\_formation\_involved\_in\_morphogenesis | WNT5A | 277 | 9 | 2.823037 | -2.413873 | 28 | 4.25 | 0.151786 |  |
| GO:0048646\_anatomical\_structure\_formation\_involved\_in\_morphogenesis | RBP4 | 277 | 9 | 2.823037 | -2.413873 | 28 | 4.25 | 0.151786 |  |
| GO:0048646\_anatomical\_structure\_formation\_involved\_in\_morphogenesis | LRP8 | 277 | 9 | 2.823037 | -2.413873 | 28 | 4.25 | 0.151786 |  |
| GO:0048646\_anatomical\_structure\_formation\_involved\_in\_morphogenesis | ZEB2 | 277 | 9 | 2.823037 | -2.413873 | 28 | 4.25 | 0.151786 |  |
| GO:0048646\_anatomical\_structure\_formation\_involved\_in\_morphogenesis | FOXC1 | 277 | 9 | 2.823037 | -2.413873 | 28 | 4.25 | 0.151786 |  |
| GO:0048646\_anatomical\_structure\_formation\_involved\_in\_morphogenesis | RORA | 277 | 9 | 2.823037 | -2.413873 | 28 | 4.25 | 0.151786 |  |
| GO:0048646\_anatomical\_structure\_formation\_involved\_in\_morphogenesis | PROX1 | 277 | 9 | 2.823037 | -2.413873 | 28 | 4.25 | 0.151786 |  |
| GO:0048646\_anatomical\_structure\_formation\_involved\_in\_morphogenesis | CITED2 | 277 | 9 | 2.823037 | -2.413873 | 28 | 4.25 | 0.151786 |  |
| GO:0048646\_anatomical\_structure\_formation\_involved\_in\_morphogenesis | CTNNB1 | 277 | 9 | 2.823037 | -2.413873 | 28 | 4.25 | 0.151786 |  |
| GO:0051093\_negative\_regulation\_of\_developmental\_process | WNT5A | 331 | 10 | 2.624979 | -2.412540 | 29 | 4.25 | 0.146552 |  |
| GO:0051093\_negative\_regulation\_of\_developmental\_process | RBP4 | 331 | 10 | 2.624979 | -2.412540 | 29 | 4.25 | 0.146552 |  |
| GO:0051093\_negative\_regulation\_of\_developmental\_process | XRCC4 | 331 | 10 | 2.624979 | -2.412540 | 29 | 4.25 | 0.146552 |  |
| GO:0051093\_negative\_regulation\_of\_developmental\_process | ZC3HC1 | 331 | 10 | 2.624979 | -2.412540 | 29 | 4.25 | 0.146552 |  |
| GO:0051093\_negative\_regulation\_of\_developmental\_process | SGK3 | 331 | 10 | 2.624979 | -2.412540 | 29 | 4.25 | 0.146552 |  |
| GO:0051093\_negative\_regulation\_of\_developmental\_process | RB1CC1 | 331 | 10 | 2.624979 | -2.412540 | 29 | 4.25 | 0.146552 |  |
| GO:0051093\_negative\_regulation\_of\_developmental\_process | DLL1 | 331 | 10 | 2.624979 | -2.412540 | 29 | 4.25 | 0.146552 |  |
| GO:0051093\_negative\_regulation\_of\_developmental\_process | FOXC1 | 331 | 10 | 2.624979 | -2.412540 | 29 | 4.25 | 0.146552 |  |
| GO:0051093\_negative\_regulation\_of\_developmental\_process | CTNNB1 | 331 | 10 | 2.624979 | -2.412540 | 29 | 4.25 | 0.146552 |  |
| GO:0051093\_negative\_regulation\_of\_developmental\_process | CITED2 | 331 | 10 | 2.624979 | -2.412540 | 29 | 4.25 | 0.146552 |  |
| GO:0016055\_Wnt\_receptor\_signaling\_pathway | FZD10 | 59 | 4 | 5.890630 | -2.350705 | 30 | 5.23 | 0.174333 |  |
| GO:0016055\_Wnt\_receptor\_signaling\_pathway | RSPO3 | 59 | 4 | 5.890630 | -2.350705 | 30 | 5.23 | 0.174333 |  |
| GO:0016055\_Wnt\_receptor\_signaling\_pathway | ZEB2 | 59 | 4 | 5.890630 | -2.350705 | 30 | 5.23 | 0.174333 |  |
| GO:0016055\_Wnt\_receptor\_signaling\_pathway | CTNNB1 | 59 | 4 | 5.890630 | -2.350705 | 30 | 5.23 | 0.174333 |  |
| GO:0048523\_negative\_regulation\_of\_cellular\_process | WNT5A | 774 | 17 | 1.908366 | -2.343623 | 31 | 5.24 | 0.169032 |  |
| GO:0048523\_negative\_regulation\_of\_cellular\_process | EGR1 | 774 | 17 | 1.908366 | -2.343623 | 31 | 5.24 | 0.169032 |  |
| GO:0048523\_negative\_regulation\_of\_cellular\_process | XRCC4 | 774 | 17 | 1.908366 | -2.343623 | 31 | 5.24 | 0.169032 |  |
| GO:0048523\_negative\_regulation\_of\_cellular\_process | RBP4 | 774 | 17 | 1.908366 | -2.343623 | 31 | 5.24 | 0.169032 |  |
| GO:0048523\_negative\_regulation\_of\_cellular\_process | BMP2 | 774 | 17 | 1.908366 | -2.343623 | 31 | 5.24 | 0.169032 |  |
| GO:0048523\_negative\_regulation\_of\_cellular\_process | ZC3HC1 | 774 | 17 | 1.908366 | -2.343623 | 31 | 5.24 | 0.169032 |  |
| GO:0048523\_negative\_regulation\_of\_cellular\_process | SGK3 | 774 | 17 | 1.908366 | -2.343623 | 31 | 5.24 | 0.169032 |  |
| GO:0048523\_negative\_regulation\_of\_cellular\_process | DLL1 | 774 | 17 | 1.908366 | -2.343623 | 31 | 5.24 | 0.169032 |  |
| GO:0048523\_negative\_regulation\_of\_cellular\_process | PROX1 | 774 | 17 | 1.908366 | -2.343623 | 31 | 5.24 | 0.169032 |  |
| GO:0048523\_negative\_regulation\_of\_cellular\_process | FOXP4 | 774 | 17 | 1.908366 | -2.343623 | 31 | 5.24 | 0.169032 |  |
| GO:0048523\_negative\_regulation\_of\_cellular\_process | CTNNB1 | 774 | 17 | 1.908366 | -2.343623 | 31 | 5.24 | 0.169032 |  |
| GO:0048523\_negative\_regulation\_of\_cellular\_process | CITED2 | 774 | 17 | 1.908366 | -2.343623 | 31 | 5.24 | 0.169032 |  |
| GO:0048523\_negative\_regulation\_of\_cellular\_process | RB1CC1 | 774 | 17 | 1.908366 | -2.343623 | 31 | 5.24 | 0.169032 |  |
| GO:0048523\_negative\_regulation\_of\_cellular\_process | JUN | 774 | 17 | 1.908366 | -2.343623 | 31 | 5.24 | 0.169032 |  |
| GO:0048523\_negative\_regulation\_of\_cellular\_process | FOXC1 | 774 | 17 | 1.908366 | -2.343623 | 31 | 5.24 | 0.169032 |  |
| GO:0048523\_negative\_regulation\_of\_cellular\_process | HBA-X | 774 | 17 | 1.908366 | -2.343623 | 31 | 5.24 | 0.169032 |  |
| GO:0048523\_negative\_regulation\_of\_cellular\_process | C1D | 774 | 17 | 1.908366 | -2.343623 | 31 | 5.24 | 0.169032 |  |
| GO:0009887\_organ\_morphogenesis | WNT5A | 642 | 15 | 2.030065 | -2.339996 | 32 | 5.25 | 0.164062 |  |
| GO:0009887\_organ\_morphogenesis | RBP4 | 642 | 15 | 2.030065 | -2.339996 | 32 | 5.25 | 0.164062 |  |
| GO:0009887\_organ\_morphogenesis | SCHIP1 | 642 | 15 | 2.030065 | -2.339996 | 32 | 5.25 | 0.164062 |  |
| GO:0009887\_organ\_morphogenesis | BMP2 | 642 | 15 | 2.030065 | -2.339996 | 32 | 5.25 | 0.164062 |  |
| GO:0009887\_organ\_morphogenesis | TBX5 | 642 | 15 | 2.030065 | -2.339996 | 32 | 5.25 | 0.164062 |  |
| GO:0009887\_organ\_morphogenesis | PRRX1 | 642 | 15 | 2.030065 | -2.339996 | 32 | 5.25 | 0.164062 |  |
| GO:0009887\_organ\_morphogenesis | ZEB2 | 642 | 15 | 2.030065 | -2.339996 | 32 | 5.25 | 0.164062 |  |
| GO:0009887\_organ\_morphogenesis | PROX1 | 642 | 15 | 2.030065 | -2.339996 | 32 | 5.25 | 0.164062 |  |
| GO:0009887\_organ\_morphogenesis | FOXP4 | 642 | 15 | 2.030065 | -2.339996 | 32 | 5.25 | 0.164062 |  |
| GO:0009887\_organ\_morphogenesis | CTNNB1 | 642 | 15 | 2.030065 | -2.339996 | 32 | 5.25 | 0.164062 |  |
| GO:0009887\_organ\_morphogenesis | CITED2 | 642 | 15 | 2.030065 | -2.339996 | 32 | 5.25 | 0.164062 |  |
| GO:0009887\_organ\_morphogenesis | TGFB2 | 642 | 15 | 2.030065 | -2.339996 | 32 | 5.25 | 0.164062 |  |
| GO:0009887\_organ\_morphogenesis | APOE | 642 | 15 | 2.030065 | -2.339996 | 32 | 5.25 | 0.164062 |  |
| GO:0009887\_organ\_morphogenesis | JUN | 642 | 15 | 2.030065 | -2.339996 | 32 | 5.25 | 0.164062 |  |
| GO:0009887\_organ\_morphogenesis | FOXC1 | 642 | 15 | 2.030065 | -2.339996 | 32 | 5.25 | 0.164062 |  |
| GO:0032501\_multicellular\_organismal\_process | NRP2 | 2183 | 35 | 1.393054 | -2.336298 | 33 | 5.27 | 0.159697 |  |
| GO:0032501\_multicellular\_organismal\_process | WNT5A | 2183 | 35 | 1.393054 | -2.336298 | 33 | 5.27 | 0.159697 |  |
| GO:0032501\_multicellular\_organismal\_process | SEPT4 | 2183 | 35 | 1.393054 | -2.336298 | 33 | 5.27 | 0.159697 |  |
| GO:0032501\_multicellular\_organismal\_process | XRCC4 | 2183 | 35 | 1.393054 | -2.336298 | 33 | 5.27 | 0.159697 |  |
| GO:0032501\_multicellular\_organismal\_process | RBP4 | 2183 | 35 | 1.393054 | -2.336298 | 33 | 5.27 | 0.159697 |  |
| GO:0032501\_multicellular\_organismal\_process | SCHIP1 | 2183 | 35 | 1.393054 | -2.336298 | 33 | 5.27 | 0.159697 |  |
| GO:0032501\_multicellular\_organismal\_process | PRRX1 | 2183 | 35 | 1.393054 | -2.336298 | 33 | 5.27 | 0.159697 |  |
| GO:0032501\_multicellular\_organismal\_process | ZEB2 | 2183 | 35 | 1.393054 | -2.336298 | 33 | 5.27 | 0.159697 |  |
| GO:0032501\_multicellular\_organismal\_process | RORA | 2183 | 35 | 1.393054 | -2.336298 | 33 | 5.27 | 0.159697 |  |
| GO:0032501\_multicellular\_organismal\_process | PRKG1 | 2183 | 35 | 1.393054 | -2.336298 | 33 | 5.27 | 0.159697 |  |
| GO:0032501\_multicellular\_organismal\_process | CITED2 | 2183 | 35 | 1.393054 | -2.336298 | 33 | 5.27 | 0.159697 |  |
| GO:0032501\_multicellular\_organismal\_process | CTNNB1 | 2183 | 35 | 1.393054 | -2.336298 | 33 | 5.27 | 0.159697 |  |
| GO:0032501\_multicellular\_organismal\_process | TGFB2 | 2183 | 35 | 1.393054 | -2.336298 | 33 | 5.27 | 0.159697 |  |
| GO:0032501\_multicellular\_organismal\_process | APOE | 2183 | 35 | 1.393054 | -2.336298 | 33 | 5.27 | 0.159697 |  |
| GO:0032501\_multicellular\_organismal\_process | RB1CC1 | 2183 | 35 | 1.393054 | -2.336298 | 33 | 5.27 | 0.159697 |  |
| GO:0032501\_multicellular\_organismal\_process | RSPO3 | 2183 | 35 | 1.393054 | -2.336298 | 33 | 5.27 | 0.159697 |  |
| GO:0032501\_multicellular\_organismal\_process | HBA-X | 2183 | 35 | 1.393054 | -2.336298 | 33 | 5.27 | 0.159697 |  |
| GO:0032501\_multicellular\_organismal\_process | USP14 | 2183 | 35 | 1.393054 | -2.336298 | 33 | 5.27 | 0.159697 |  |
| GO:0032501\_multicellular\_organismal\_process | INSM1 | 2183 | 35 | 1.393054 | -2.336298 | 33 | 5.27 | 0.159697 |  |
| GO:0032501\_multicellular\_organismal\_process | EGR1 | 2183 | 35 | 1.393054 | -2.336298 | 33 | 5.27 | 0.159697 |  |
| GO:0032501\_multicellular\_organismal\_process | BMP2 | 2183 | 35 | 1.393054 | -2.336298 | 33 | 5.27 | 0.159697 |  |
| GO:0032501\_multicellular\_organismal\_process | PTPRM | 2183 | 35 | 1.393054 | -2.336298 | 33 | 5.27 | 0.159697 |  |
| GO:0032501\_multicellular\_organismal\_process | PLEK | 2183 | 35 | 1.393054 | -2.336298 | 33 | 5.27 | 0.159697 |  |
| GO:0032501\_multicellular\_organismal\_process | ACTA2 | 2183 | 35 | 1.393054 | -2.336298 | 33 | 5.27 | 0.159697 |  |
| GO:0032501\_multicellular\_organismal\_process | TBX5 | 2183 | 35 | 1.393054 | -2.336298 | 33 | 5.27 | 0.159697 |  |
| GO:0032501\_multicellular\_organismal\_process | DLL1 | 2183 | 35 | 1.393054 | -2.336298 | 33 | 5.27 | 0.159697 |  |
| GO:0032501\_multicellular\_organismal\_process | PROX1 | 2183 | 35 | 1.393054 | -2.336298 | 33 | 5.27 | 0.159697 |  |
| GO:0032501\_multicellular\_organismal\_process | FOXP4 | 2183 | 35 | 1.393054 | -2.336298 | 33 | 5.27 | 0.159697 |  |
| GO:0032501\_multicellular\_organismal\_process | COL5A2 | 2183 | 35 | 1.393054 | -2.336298 | 33 | 5.27 | 0.159697 |  |
| GO:0032501\_multicellular\_organismal\_process | RGS9BP | 2183 | 35 | 1.393054 | -2.336298 | 33 | 5.27 | 0.159697 |  |
| GO:0032501\_multicellular\_organismal\_process | PCDH18 | 2183 | 35 | 1.393054 | -2.336298 | 33 | 5.27 | 0.159697 |  |
| GO:0032501\_multicellular\_organismal\_process | BTG2 | 2183 | 35 | 1.393054 | -2.336298 | 33 | 5.27 | 0.159697 |  |
| GO:0032501\_multicellular\_organismal\_process | JUN | 2183 | 35 | 1.393054 | -2.336298 | 33 | 5.27 | 0.159697 |  |
| GO:0032501\_multicellular\_organismal\_process | LRP8 | 2183 | 35 | 1.393054 | -2.336298 | 33 | 5.27 | 0.159697 |  |
| GO:0032501\_multicellular\_organismal\_process | FOXC1 | 2183 | 35 | 1.393054 | -2.336298 | 33 | 5.27 | 0.159697 |  |
| GO:0014032\_neural\_crest\_cell\_development | NRP2 | 30 | 3 | 8.688679 | -2.328489 | 35 | 5.38 | 0.153714 |  |
| GO:0014032\_neural\_crest\_cell\_development | FOXC1 | 30 | 3 | 8.688679 | -2.328489 | 35 | 5.38 | 0.153714 |  |
| GO:0014032\_neural\_crest\_cell\_development | ZEB2 | 30 | 3 | 8.688679 | -2.328489 | 35 | 5.38 | 0.153714 |  |
| GO:0014033\_neural\_crest\_cell\_differentiation | NRP2 | 30 | 3 | 8.688679 | -2.328489 | 35 | 5.38 | 0.153714 |  |
| GO:0014033\_neural\_crest\_cell\_differentiation | FOXC1 | 30 | 3 | 8.688679 | -2.328489 | 35 | 5.38 | 0.153714 |  |
| GO:0014033\_neural\_crest\_cell\_differentiation | ZEB2 | 30 | 3 | 8.688679 | -2.328489 | 35 | 5.38 | 0.153714 |  |
| GO:0007275\_multicellular\_organismal\_development | NRP2 | 1760 | 30 | 1.481025 | -2.317847 | 36 | 5.42 | 0.150556 |  |
| GO:0007275\_multicellular\_organismal\_development | WNT5A | 1760 | 30 | 1.481025 | -2.317847 | 36 | 5.42 | 0.150556 |  |
| GO:0007275\_multicellular\_organismal\_development | RBP4 | 1760 | 30 | 1.481025 | -2.317847 | 36 | 5.42 | 0.150556 |  |
| GO:0007275\_multicellular\_organismal\_development | XRCC4 | 1760 | 30 | 1.481025 | -2.317847 | 36 | 5.42 | 0.150556 |  |
| GO:0007275\_multicellular\_organismal\_development | SCHIP1 | 1760 | 30 | 1.481025 | -2.317847 | 36 | 5.42 | 0.150556 |  |
| GO:0007275\_multicellular\_organismal\_development | SEPT4 | 1760 | 30 | 1.481025 | -2.317847 | 36 | 5.42 | 0.150556 |  |
| GO:0007275\_multicellular\_organismal\_development | PRRX1 | 1760 | 30 | 1.481025 | -2.317847 | 36 | 5.42 | 0.150556 |  |
| GO:0007275\_multicellular\_organismal\_development | ZEB2 | 1760 | 30 | 1.481025 | -2.317847 | 36 | 5.42 | 0.150556 |  |
| GO:0007275\_multicellular\_organismal\_development | RORA | 1760 | 30 | 1.481025 | -2.317847 | 36 | 5.42 | 0.150556 |  |
| GO:0007275\_multicellular\_organismal\_development | PRKG1 | 1760 | 30 | 1.481025 | -2.317847 | 36 | 5.42 | 0.150556 |  |
| GO:0007275\_multicellular\_organismal\_development | CTNNB1 | 1760 | 30 | 1.481025 | -2.317847 | 36 | 5.42 | 0.150556 |  |
| GO:0007275\_multicellular\_organismal\_development | CITED2 | 1760 | 30 | 1.481025 | -2.317847 | 36 | 5.42 | 0.150556 |  |
| GO:0007275\_multicellular\_organismal\_development | TGFB2 | 1760 | 30 | 1.481025 | -2.317847 | 36 | 5.42 | 0.150556 |  |
| GO:0007275\_multicellular\_organismal\_development | APOE | 1760 | 30 | 1.481025 | -2.317847 | 36 | 5.42 | 0.150556 |  |
| GO:0007275\_multicellular\_organismal\_development | RB1CC1 | 1760 | 30 | 1.481025 | -2.317847 | 36 | 5.42 | 0.150556 |  |
| GO:0007275\_multicellular\_organismal\_development | RSPO3 | 1760 | 30 | 1.481025 | -2.317847 | 36 | 5.42 | 0.150556 |  |
| GO:0007275\_multicellular\_organismal\_development | HBA-X | 1760 | 30 | 1.481025 | -2.317847 | 36 | 5.42 | 0.150556 |  |
| GO:0007275\_multicellular\_organismal\_development | INSM1 | 1760 | 30 | 1.481025 | -2.317847 | 36 | 5.42 | 0.150556 |  |
| GO:0007275\_multicellular\_organismal\_development | EGR1 | 1760 | 30 | 1.481025 | -2.317847 | 36 | 5.42 | 0.150556 |  |
| GO:0007275\_multicellular\_organismal\_development | BMP2 | 1760 | 30 | 1.481025 | -2.317847 | 36 | 5.42 | 0.150556 |  |
| GO:0007275\_multicellular\_organismal\_development | TBX5 | 1760 | 30 | 1.481025 | -2.317847 | 36 | 5.42 | 0.150556 |  |
| GO:0007275\_multicellular\_organismal\_development | DLL1 | 1760 | 30 | 1.481025 | -2.317847 | 36 | 5.42 | 0.150556 |  |
| GO:0007275\_multicellular\_organismal\_development | FOXP4 | 1760 | 30 | 1.481025 | -2.317847 | 36 | 5.42 | 0.150556 |  |
| GO:0007275\_multicellular\_organismal\_development | PROX1 | 1760 | 30 | 1.481025 | -2.317847 | 36 | 5.42 | 0.150556 |  |
| GO:0007275\_multicellular\_organismal\_development | COL5A2 | 1760 | 30 | 1.481025 | -2.317847 | 36 | 5.42 | 0.150556 |  |
| GO:0007275\_multicellular\_organismal\_development | PCDH18 | 1760 | 30 | 1.481025 | -2.317847 | 36 | 5.42 | 0.150556 |  |
| GO:0007275\_multicellular\_organismal\_development | BTG2 | 1760 | 30 | 1.481025 | -2.317847 | 36 | 5.42 | 0.150556 |  |
| GO:0007275\_multicellular\_organismal\_development | JUN | 1760 | 30 | 1.481025 | -2.317847 | 36 | 5.42 | 0.150556 |  |
| GO:0007275\_multicellular\_organismal\_development | FOXC1 | 1760 | 30 | 1.481025 | -2.317847 | 36 | 5.42 | 0.150556 |  |
| GO:0007275\_multicellular\_organismal\_development | LRP8 | 1760 | 30 | 1.481025 | -2.317847 | 36 | 5.42 | 0.150556 |  |
| GO:0050794\_regulation\_of\_cellular\_process | WNT5A | 2190 | 35 | 1.388602 | -2.308145 | 37 | 5.45 | 0.147297 |  |
| GO:0050794\_regulation\_of\_cellular\_process | RHOJ | 2190 | 35 | 1.388602 | -2.308145 | 37 | 5.45 | 0.147297 |  |
| GO:0050794\_regulation\_of\_cellular\_process | RBP4 | 2190 | 35 | 1.388602 | -2.308145 | 37 | 5.45 | 0.147297 |  |
| GO:0050794\_regulation\_of\_cellular\_process | SCHIP1 | 2190 | 35 | 1.388602 | -2.308145 | 37 | 5.45 | 0.147297 |  |
| GO:0050794\_regulation\_of\_cellular\_process | XRCC4 | 2190 | 35 | 1.388602 | -2.308145 | 37 | 5.45 | 0.147297 |  |
| GO:0050794\_regulation\_of\_cellular\_process | PRRX1 | 2190 | 35 | 1.388602 | -2.308145 | 37 | 5.45 | 0.147297 |  |
| GO:0050794\_regulation\_of\_cellular\_process | ZEB2 | 2190 | 35 | 1.388602 | -2.308145 | 37 | 5.45 | 0.147297 |  |
| GO:0050794\_regulation\_of\_cellular\_process | RORA | 2190 | 35 | 1.388602 | -2.308145 | 37 | 5.45 | 0.147297 |  |
| GO:0050794\_regulation\_of\_cellular\_process | PRKG1 | 2190 | 35 | 1.388602 | -2.308145 | 37 | 5.45 | 0.147297 |  |
| GO:0050794\_regulation\_of\_cellular\_process | CTNNB1 | 2190 | 35 | 1.388602 | -2.308145 | 37 | 5.45 | 0.147297 |  |
| GO:0050794\_regulation\_of\_cellular\_process | TGFB2 | 2190 | 35 | 1.388602 | -2.308145 | 37 | 5.45 | 0.147297 |  |
| GO:0050794\_regulation\_of\_cellular\_process | CITED2 | 2190 | 35 | 1.388602 | -2.308145 | 37 | 5.45 | 0.147297 |  |
| GO:0050794\_regulation\_of\_cellular\_process | A930038C07RIK | 2190 | 35 | 1.388602 | -2.308145 | 37 | 5.45 | 0.147297 |  |
| GO:0050794\_regulation\_of\_cellular\_process | APOE | 2190 | 35 | 1.388602 | -2.308145 | 37 | 5.45 | 0.147297 |  |
| GO:0050794\_regulation\_of\_cellular\_process | RSPO3 | 2190 | 35 | 1.388602 | -2.308145 | 37 | 5.45 | 0.147297 |  |
| GO:0050794\_regulation\_of\_cellular\_process | RB1CC1 | 2190 | 35 | 1.388602 | -2.308145 | 37 | 5.45 | 0.147297 |  |
| GO:0050794\_regulation\_of\_cellular\_process | CDC42EP4 | 2190 | 35 | 1.388602 | -2.308145 | 37 | 5.45 | 0.147297 |  |
| GO:0050794\_regulation\_of\_cellular\_process | HBA-X | 2190 | 35 | 1.388602 | -2.308145 | 37 | 5.45 | 0.147297 |  |
| GO:0050794\_regulation\_of\_cellular\_process | EGR1 | 2190 | 35 | 1.388602 | -2.308145 | 37 | 5.45 | 0.147297 |  |
| GO:0050794\_regulation\_of\_cellular\_process | BMP2 | 2190 | 35 | 1.388602 | -2.308145 | 37 | 5.45 | 0.147297 |  |
| GO:0050794\_regulation\_of\_cellular\_process | VAV3 | 2190 | 35 | 1.388602 | -2.308145 | 37 | 5.45 | 0.147297 |  |
| GO:0050794\_regulation\_of\_cellular\_process | ZC3HC1 | 2190 | 35 | 1.388602 | -2.308145 | 37 | 5.45 | 0.147297 |  |
| GO:0050794\_regulation\_of\_cellular\_process | KLF12 | 2190 | 35 | 1.388602 | -2.308145 | 37 | 5.45 | 0.147297 |  |
| GO:0050794\_regulation\_of\_cellular\_process | PLEK | 2190 | 35 | 1.388602 | -2.308145 | 37 | 5.45 | 0.147297 |  |
| GO:0050794\_regulation\_of\_cellular\_process | SGK3 | 2190 | 35 | 1.388602 | -2.308145 | 37 | 5.45 | 0.147297 |  |
| GO:0050794\_regulation\_of\_cellular\_process | TBX5 | 2190 | 35 | 1.388602 | -2.308145 | 37 | 5.45 | 0.147297 |  |
| GO:0050794\_regulation\_of\_cellular\_process | CCDC80 | 2190 | 35 | 1.388602 | -2.308145 | 37 | 5.45 | 0.147297 |  |
| GO:0050794\_regulation\_of\_cellular\_process | DLL1 | 2190 | 35 | 1.388602 | -2.308145 | 37 | 5.45 | 0.147297 |  |
| GO:0050794\_regulation\_of\_cellular\_process | FOXP4 | 2190 | 35 | 1.388602 | -2.308145 | 37 | 5.45 | 0.147297 |  |
| GO:0050794\_regulation\_of\_cellular\_process | PROX1 | 2190 | 35 | 1.388602 | -2.308145 | 37 | 5.45 | 0.147297 |  |
| GO:0050794\_regulation\_of\_cellular\_process | FZD10 | 2190 | 35 | 1.388602 | -2.308145 | 37 | 5.45 | 0.147297 |  |
| GO:0050794\_regulation\_of\_cellular\_process | JUN | 2190 | 35 | 1.388602 | -2.308145 | 37 | 5.45 | 0.147297 |  |
| GO:0050794\_regulation\_of\_cellular\_process | LRP8 | 2190 | 35 | 1.388602 | -2.308145 | 37 | 5.45 | 0.147297 |  |
| GO:0050794\_regulation\_of\_cellular\_process | FOXC1 | 2190 | 35 | 1.388602 | -2.308145 | 37 | 5.45 | 0.147297 |  |
| GO:0050794\_regulation\_of\_cellular\_process | C1D | 2190 | 35 | 1.388602 | -2.308145 | 37 | 5.45 | 0.147297 |  |
| GO:0007155\_cell\_adhesion | A930038C07RIK | 186 | 7 | 3.269933 | -2.301298 | 39 | 5.47 | 0.140256 |  |
| GO:0007155\_cell\_adhesion | VAV3 | 186 | 7 | 3.269933 | -2.301298 | 39 | 5.47 | 0.140256 |  |
| GO:0007155\_cell\_adhesion | PLEK | 186 | 7 | 3.269933 | -2.301298 | 39 | 5.47 | 0.140256 |  |
| GO:0007155\_cell\_adhesion | CCDC80 | 186 | 7 | 3.269933 | -2.301298 | 39 | 5.47 | 0.140256 |  |
| GO:0007155\_cell\_adhesion | CTNNB1 | 186 | 7 | 3.269933 | -2.301298 | 39 | 5.47 | 0.140256 |  |
| GO:0007155\_cell\_adhesion | CITED2 | 186 | 7 | 3.269933 | -2.301298 | 39 | 5.47 | 0.140256 |  |
| GO:0007155\_cell\_adhesion | TGFB2 | 186 | 7 | 3.269933 | -2.301298 | 39 | 5.47 | 0.140256 |  |
| GO:0022610\_biological\_adhesion | A930038C07RIK | 186 | 7 | 3.269933 | -2.301298 | 39 | 5.47 | 0.140256 |  |
| GO:0022610\_biological\_adhesion | VAV3 | 186 | 7 | 3.269933 | -2.301298 | 39 | 5.47 | 0.140256 |  |
| GO:0022610\_biological\_adhesion | PLEK | 186 | 7 | 3.269933 | -2.301298 | 39 | 5.47 | 0.140256 |  |
| GO:0022610\_biological\_adhesion | CCDC80 | 186 | 7 | 3.269933 | -2.301298 | 39 | 5.47 | 0.140256 |  |
| GO:0022610\_biological\_adhesion | CTNNB1 | 186 | 7 | 3.269933 | -2.301298 | 39 | 5.47 | 0.140256 |  |
| GO:0022610\_biological\_adhesion | CITED2 | 186 | 7 | 3.269933 | -2.301298 | 39 | 5.47 | 0.140256 |  |
| GO:0022610\_biological\_adhesion | TGFB2 | 186 | 7 | 3.269933 | -2.301298 | 39 | 5.47 | 0.140256 |  |
| GO:0001501\_skeletal\_system\_development | RBP4 | 236 | 8 | 2.945315 | -2.296501 | 40 | 5.51 | 0.137750 |  |
| GO:0001501\_skeletal\_system\_development | SCHIP1 | 236 | 8 | 2.945315 | -2.296501 | 40 | 5.51 | 0.137750 |  |
| GO:0001501\_skeletal\_system\_development | BMP2 | 236 | 8 | 2.945315 | -2.296501 | 40 | 5.51 | 0.137750 |  |
| GO:0001501\_skeletal\_system\_development | PRRX1 | 236 | 8 | 2.945315 | -2.296501 | 40 | 5.51 | 0.137750 |  |
| GO:0001501\_skeletal\_system\_development | FOXC1 | 236 | 8 | 2.945315 | -2.296501 | 40 | 5.51 | 0.137750 |  |
| GO:0001501\_skeletal\_system\_development | COL5A2 | 236 | 8 | 2.945315 | -2.296501 | 40 | 5.51 | 0.137750 |  |
| GO:0001501\_skeletal\_system\_development | CTNNB1 | 236 | 8 | 2.945315 | -2.296501 | 40 | 5.51 | 0.137750 |  |
| GO:0001501\_skeletal\_system\_development | TGFB2 | 236 | 8 | 2.945315 | -2.296501 | 40 | 5.51 | 0.137750 |  |
| GO:0006916\_anti-apoptosis | ZC3HC1 | 62 | 4 | 5.605600 | -2.273344 | 42 | 5.82 | 0.138571 |  |
| GO:0006916\_anti-apoptosis | SGK3 | 62 | 4 | 5.605600 | -2.273344 | 42 | 5.82 | 0.138571 |  |
| GO:0006916\_anti-apoptosis | FOXC1 | 62 | 4 | 5.605600 | -2.273344 | 42 | 5.82 | 0.138571 |  |
| GO:0006916\_anti-apoptosis | CITED2 | 62 | 4 | 5.605600 | -2.273344 | 42 | 5.82 | 0.138571 |  |
| GO:0030155\_regulation\_of\_cell\_adhesion | A930038C07RIK | 62 | 4 | 5.605600 | -2.273344 | 42 | 5.82 | 0.138571 |  |
| GO:0030155\_regulation\_of\_cell\_adhesion | VAV3 | 62 | 4 | 5.605600 | -2.273344 | 42 | 5.82 | 0.138571 |  |
| GO:0030155\_regulation\_of\_cell\_adhesion | CCDC80 | 62 | 4 | 5.605600 | -2.273344 | 42 | 5.82 | 0.138571 |  |
| GO:0030155\_regulation\_of\_cell\_adhesion | CITED2 | 62 | 4 | 5.605600 | -2.273344 | 42 | 5.82 | 0.138571 |  |
| GO:0048468\_cell\_development | NRP2 | 654 | 15 | 1.992816 | -2.263113 | 43 | 5.83 | 0.135581 |  |
| GO:0048468\_cell\_development | SEPT4 | 654 | 15 | 1.992816 | -2.263113 | 43 | 5.83 | 0.135581 |  |
| GO:0048468\_cell\_development | RBP4 | 654 | 15 | 1.992816 | -2.263113 | 43 | 5.83 | 0.135581 |  |
| GO:0048468\_cell\_development | XRCC4 | 654 | 15 | 1.992816 | -2.263113 | 43 | 5.83 | 0.135581 |  |
| GO:0048468\_cell\_development | BMP2 | 654 | 15 | 1.992816 | -2.263113 | 43 | 5.83 | 0.135581 |  |
| GO:0048468\_cell\_development | DLL1 | 654 | 15 | 1.992816 | -2.263113 | 43 | 5.83 | 0.135581 |  |
| GO:0048468\_cell\_development | ZEB2 | 654 | 15 | 1.992816 | -2.263113 | 43 | 5.83 | 0.135581 |  |
| GO:0048468\_cell\_development | RORA | 654 | 15 | 1.992816 | -2.263113 | 43 | 5.83 | 0.135581 |  |
| GO:0048468\_cell\_development | PRKG1 | 654 | 15 | 1.992816 | -2.263113 | 43 | 5.83 | 0.135581 |  |
| GO:0048468\_cell\_development | PROX1 | 654 | 15 | 1.992816 | -2.263113 | 43 | 5.83 | 0.135581 |  |
| GO:0048468\_cell\_development | TGFB2 | 654 | 15 | 1.992816 | -2.263113 | 43 | 5.83 | 0.135581 |  |
| GO:0048468\_cell\_development | CTNNB1 | 654 | 15 | 1.992816 | -2.263113 | 43 | 5.83 | 0.135581 |  |
| GO:0048468\_cell\_development | APOE | 654 | 15 | 1.992816 | -2.263113 | 43 | 5.83 | 0.135581 |  |
| GO:0048468\_cell\_development | FOXC1 | 654 | 15 | 1.992816 | -2.263113 | 43 | 5.83 | 0.135581 |  |
| GO:0048468\_cell\_development | HBA-X | 654 | 15 | 1.992816 | -2.263113 | 43 | 5.83 | 0.135581 |  |
| GO:0018149\_peptide\_cross-linking | A930038C07RIK | 10 | 2 | 17.377358 | -2.258562 | 46 | 6.54 | 0.142174 |  |
| GO:0018149\_peptide\_cross-linking | DCN | 10 | 2 | 17.377358 | -2.258562 | 46 | 6.54 | 0.142174 |  |
| GO:0045669\_positive\_regulation\_of\_osteoblast\_differentiation | BMP2 | 10 | 2 | 17.377358 | -2.258562 | 46 | 6.54 | 0.142174 |  |
| GO:0045669\_positive\_regulation\_of\_osteoblast\_differentiation | CTNNB1 | 10 | 2 | 17.377358 | -2.258562 | 46 | 6.54 | 0.142174 |  |
| GO:0050654\_chondroitin\_sulfate\_proteoglycan\_metabolic\_process | A930038C07RIK | 10 | 2 | 17.377358 | -2.258562 | 46 | 6.54 | 0.142174 |  |
| GO:0050654\_chondroitin\_sulfate\_proteoglycan\_metabolic\_process | DCN | 10 | 2 | 17.377358 | -2.258562 | 46 | 6.54 | 0.142174 |  |
| GO:0001756\_somitogenesis | FOXC1 | 35 | 3 | 7.447439 | -2.138668 | 48 | 7.84 | 0.163333 |  |
| GO:0001756\_somitogenesis | ZEB2 | 35 | 3 | 7.447439 | -2.138668 | 48 | 7.84 | 0.163333 |  |
| GO:0001756\_somitogenesis | DLL1 | 35 | 3 | 7.447439 | -2.138668 | 48 | 7.84 | 0.163333 |  |
| GO:0006869\_lipid\_transport | RBP4 | 35 | 3 | 7.447439 | -2.138668 | 48 | 7.84 | 0.163333 |  |
| GO:0006869\_lipid\_transport | APOE | 35 | 3 | 7.447439 | -2.138668 | 48 | 7.84 | 0.163333 |  |
| GO:0006869\_lipid\_transport | CFTR | 35 | 3 | 7.447439 | -2.138668 | 48 | 7.84 | 0.163333 |  |
| GO:0032502\_developmental\_process | RHOJ | 2060 | 33 | 1.391876 | -2.131284 | 49 | 7.88 | 0.160816 |  |
| GO:0032502\_developmental\_process | NRP2 | 2060 | 33 | 1.391876 | -2.131284 | 49 | 7.88 | 0.160816 |  |
| GO:0032502\_developmental\_process | WNT5A | 2060 | 33 | 1.391876 | -2.131284 | 49 | 7.88 | 0.160816 |  |
| GO:0032502\_developmental\_process | XRCC4 | 2060 | 33 | 1.391876 | -2.131284 | 49 | 7.88 | 0.160816 |  |
| GO:0032502\_developmental\_process | RBP4 | 2060 | 33 | 1.391876 | -2.131284 | 49 | 7.88 | 0.160816 |  |
| GO:0032502\_developmental\_process | SCHIP1 | 2060 | 33 | 1.391876 | -2.131284 | 49 | 7.88 | 0.160816 |  |
| GO:0032502\_developmental\_process | SEPT4 | 2060 | 33 | 1.391876 | -2.131284 | 49 | 7.88 | 0.160816 |  |
| GO:0032502\_developmental\_process | PRRX1 | 2060 | 33 | 1.391876 | -2.131284 | 49 | 7.88 | 0.160816 |  |
| GO:0032502\_developmental\_process | ZEB2 | 2060 | 33 | 1.391876 | -2.131284 | 49 | 7.88 | 0.160816 |  |
| GO:0032502\_developmental\_process | RORA | 2060 | 33 | 1.391876 | -2.131284 | 49 | 7.88 | 0.160816 |  |
| GO:0032502\_developmental\_process | PRKG1 | 2060 | 33 | 1.391876 | -2.131284 | 49 | 7.88 | 0.160816 |  |
| GO:0032502\_developmental\_process | CITED2 | 2060 | 33 | 1.391876 | -2.131284 | 49 | 7.88 | 0.160816 |  |
| GO:0032502\_developmental\_process | CTNNB1 | 2060 | 33 | 1.391876 | -2.131284 | 49 | 7.88 | 0.160816 |  |
| GO:0032502\_developmental\_process | TGFB2 | 2060 | 33 | 1.391876 | -2.131284 | 49 | 7.88 | 0.160816 |  |
| GO:0032502\_developmental\_process | APOE | 2060 | 33 | 1.391876 | -2.131284 | 49 | 7.88 | 0.160816 |  |
| GO:0032502\_developmental\_process | RB1CC1 | 2060 | 33 | 1.391876 | -2.131284 | 49 | 7.88 | 0.160816 |  |
| GO:0032502\_developmental\_process | RSPO3 | 2060 | 33 | 1.391876 | -2.131284 | 49 | 7.88 | 0.160816 |  |
| GO:0032502\_developmental\_process | HBA-X | 2060 | 33 | 1.391876 | -2.131284 | 49 | 7.88 | 0.160816 |  |
| GO:0032502\_developmental\_process | INSM1 | 2060 | 33 | 1.391876 | -2.131284 | 49 | 7.88 | 0.160816 |  |
| GO:0032502\_developmental\_process | EGR1 | 2060 | 33 | 1.391876 | -2.131284 | 49 | 7.88 | 0.160816 |  |
| GO:0032502\_developmental\_process | BMP2 | 2060 | 33 | 1.391876 | -2.131284 | 49 | 7.88 | 0.160816 |  |
| GO:0032502\_developmental\_process | ZC3HC1 | 2060 | 33 | 1.391876 | -2.131284 | 49 | 7.88 | 0.160816 |  |
| GO:0032502\_developmental\_process | SGK3 | 2060 | 33 | 1.391876 | -2.131284 | 49 | 7.88 | 0.160816 |  |
| GO:0032502\_developmental\_process | TBX5 | 2060 | 33 | 1.391876 | -2.131284 | 49 | 7.88 | 0.160816 |  |
| GO:0032502\_developmental\_process | DLL1 | 2060 | 33 | 1.391876 | -2.131284 | 49 | 7.88 | 0.160816 |  |
| GO:0032502\_developmental\_process | PROX1 | 2060 | 33 | 1.391876 | -2.131284 | 49 | 7.88 | 0.160816 |  |
| GO:0032502\_developmental\_process | FOXP4 | 2060 | 33 | 1.391876 | -2.131284 | 49 | 7.88 | 0.160816 |  |
| GO:0032502\_developmental\_process | COL5A2 | 2060 | 33 | 1.391876 | -2.131284 | 49 | 7.88 | 0.160816 |  |
| GO:0032502\_developmental\_process | PCDH18 | 2060 | 33 | 1.391876 | -2.131284 | 49 | 7.88 | 0.160816 |  |
| GO:0032502\_developmental\_process | BTG2 | 2060 | 33 | 1.391876 | -2.131284 | 49 | 7.88 | 0.160816 |  |
| GO:0032502\_developmental\_process | JUN | 2060 | 33 | 1.391876 | -2.131284 | 49 | 7.88 | 0.160816 |  |
| GO:0032502\_developmental\_process | FOXC1 | 2060 | 33 | 1.391876 | -2.131284 | 49 | 7.88 | 0.160816 |  |
| GO:0032502\_developmental\_process | LRP8 | 2060 | 33 | 1.391876 | -2.131284 | 49 | 7.88 | 0.160816 |  |
| GO:0009653\_anatomical\_structure\_morphogenesis | RHOJ | 958 | 19 | 1.723224 | -2.101751 | 50 | 8.13 | 0.162600 |  |
| GO:0009653\_anatomical\_structure\_morphogenesis | WNT5A | 958 | 19 | 1.723224 | -2.101751 | 50 | 8.13 | 0.162600 |  |
| GO:0009653\_anatomical\_structure\_morphogenesis | RBP4 | 958 | 19 | 1.723224 | -2.101751 | 50 | 8.13 | 0.162600 |  |
| GO:0009653\_anatomical\_structure\_morphogenesis | SCHIP1 | 958 | 19 | 1.723224 | -2.101751 | 50 | 8.13 | 0.162600 |  |
| GO:0009653\_anatomical\_structure\_morphogenesis | BMP2 | 958 | 19 | 1.723224 | -2.101751 | 50 | 8.13 | 0.162600 |  |
| GO:0009653\_anatomical\_structure\_morphogenesis | TBX5 | 958 | 19 | 1.723224 | -2.101751 | 50 | 8.13 | 0.162600 |  |
| GO:0009653\_anatomical\_structure\_morphogenesis | PRRX1 | 958 | 19 | 1.723224 | -2.101751 | 50 | 8.13 | 0.162600 |  |
| GO:0009653\_anatomical\_structure\_morphogenesis | ZEB2 | 958 | 19 | 1.723224 | -2.101751 | 50 | 8.13 | 0.162600 |  |
| GO:0009653\_anatomical\_structure\_morphogenesis | RORA | 958 | 19 | 1.723224 | -2.101751 | 50 | 8.13 | 0.162600 |  |
| GO:0009653\_anatomical\_structure\_morphogenesis | PROX1 | 958 | 19 | 1.723224 | -2.101751 | 50 | 8.13 | 0.162600 |  |
| GO:0009653\_anatomical\_structure\_morphogenesis | FOXP4 | 958 | 19 | 1.723224 | -2.101751 | 50 | 8.13 | 0.162600 |  |
| GO:0009653\_anatomical\_structure\_morphogenesis | CTNNB1 | 958 | 19 | 1.723224 | -2.101751 | 50 | 8.13 | 0.162600 |  |
| GO:0009653\_anatomical\_structure\_morphogenesis | CITED2 | 958 | 19 | 1.723224 | -2.101751 | 50 | 8.13 | 0.162600 |  |
| GO:0009653\_anatomical\_structure\_morphogenesis | TGFB2 | 958 | 19 | 1.723224 | -2.101751 | 50 | 8.13 | 0.162600 |  |
| GO:0009653\_anatomical\_structure\_morphogenesis | APOE | 958 | 19 | 1.723224 | -2.101751 | 50 | 8.13 | 0.162600 |  |
| GO:0009653\_anatomical\_structure\_morphogenesis | JUN | 958 | 19 | 1.723224 | -2.101751 | 50 | 8.13 | 0.162600 |  |
| GO:0009653\_anatomical\_structure\_morphogenesis | RSPO3 | 958 | 19 | 1.723224 | -2.101751 | 50 | 8.13 | 0.162600 |  |
| GO:0009653\_anatomical\_structure\_morphogenesis | LRP8 | 958 | 19 | 1.723224 | -2.101751 | 50 | 8.13 | 0.162600 |  |
| GO:0009653\_anatomical\_structure\_morphogenesis | FOXC1 | 958 | 19 | 1.723224 | -2.101751 | 50 | 8.13 | 0.162600 |  |
| GO:0001568\_blood\_vessel\_development | APOE | 203 | 7 | 2.996096 | -2.096388 | 51 | 8.84 | 0.173333 |  |
| GO:0001568\_blood\_vessel\_development | PRRX1 | 203 | 7 | 2.996096 | -2.096388 | 51 | 8.84 | 0.173333 |  |
| GO:0001568\_blood\_vessel\_development | FOXC1 | 203 | 7 | 2.996096 | -2.096388 | 51 | 8.84 | 0.173333 |  |
| GO:0001568\_blood\_vessel\_development | PROX1 | 203 | 7 | 2.996096 | -2.096388 | 51 | 8.84 | 0.173333 |  |
| GO:0001568\_blood\_vessel\_development | TGFB2 | 203 | 7 | 2.996096 | -2.096388 | 51 | 8.84 | 0.173333 |  |
| GO:0001568\_blood\_vessel\_development | CITED2 | 203 | 7 | 2.996096 | -2.096388 | 51 | 8.84 | 0.173333 |  |
| GO:0001568\_blood\_vessel\_development | CTNNB1 | 203 | 7 | 2.996096 | -2.096388 | 51 | 8.84 | 0.173333 |  |
| GO:0048869\_cellular\_developmental\_process | RHOJ | 1113 | 21 | 1.639373 | -2.059363 | 52 | 9.08 | 0.174615 |  |
| GO:0048869\_cellular\_developmental\_process | INSM1 | 1113 | 21 | 1.639373 | -2.059363 | 52 | 9.08 | 0.174615 |  |
| GO:0048869\_cellular\_developmental\_process | NRP2 | 1113 | 21 | 1.639373 | -2.059363 | 52 | 9.08 | 0.174615 |  |
| GO:0048869\_cellular\_developmental\_process | EGR1 | 1113 | 21 | 1.639373 | -2.059363 | 52 | 9.08 | 0.174615 |  |
| GO:0048869\_cellular\_developmental\_process | RBP4 | 1113 | 21 | 1.639373 | -2.059363 | 52 | 9.08 | 0.174615 |  |
| GO:0048869\_cellular\_developmental\_process | SEPT4 | 1113 | 21 | 1.639373 | -2.059363 | 52 | 9.08 | 0.174615 |  |
| GO:0048869\_cellular\_developmental\_process | XRCC4 | 1113 | 21 | 1.639373 | -2.059363 | 52 | 9.08 | 0.174615 |  |
| GO:0048869\_cellular\_developmental\_process | BMP2 | 1113 | 21 | 1.639373 | -2.059363 | 52 | 9.08 | 0.174615 |  |
| GO:0048869\_cellular\_developmental\_process | TBX5 | 1113 | 21 | 1.639373 | -2.059363 | 52 | 9.08 | 0.174615 |  |
| GO:0048869\_cellular\_developmental\_process | ZEB2 | 1113 | 21 | 1.639373 | -2.059363 | 52 | 9.08 | 0.174615 |  |
| GO:0048869\_cellular\_developmental\_process | DLL1 | 1113 | 21 | 1.639373 | -2.059363 | 52 | 9.08 | 0.174615 |  |
| GO:0048869\_cellular\_developmental\_process | RORA | 1113 | 21 | 1.639373 | -2.059363 | 52 | 9.08 | 0.174615 |  |
| GO:0048869\_cellular\_developmental\_process | PRKG1 | 1113 | 21 | 1.639373 | -2.059363 | 52 | 9.08 | 0.174615 |  |
| GO:0048869\_cellular\_developmental\_process | PROX1 | 1113 | 21 | 1.639373 | -2.059363 | 52 | 9.08 | 0.174615 |  |
| GO:0048869\_cellular\_developmental\_process | CTNNB1 | 1113 | 21 | 1.639373 | -2.059363 | 52 | 9.08 | 0.174615 |  |
| GO:0048869\_cellular\_developmental\_process | CITED2 | 1113 | 21 | 1.639373 | -2.059363 | 52 | 9.08 | 0.174615 |  |
| GO:0048869\_cellular\_developmental\_process | TGFB2 | 1113 | 21 | 1.639373 | -2.059363 | 52 | 9.08 | 0.174615 |  |
| GO:0048869\_cellular\_developmental\_process | APOE | 1113 | 21 | 1.639373 | -2.059363 | 52 | 9.08 | 0.174615 |  |
| GO:0048869\_cellular\_developmental\_process | JUN | 1113 | 21 | 1.639373 | -2.059363 | 52 | 9.08 | 0.174615 |  |
| GO:0048869\_cellular\_developmental\_process | FOXC1 | 1113 | 21 | 1.639373 | -2.059363 | 52 | 9.08 | 0.174615 |  |
| GO:0048869\_cellular\_developmental\_process | HBA-X | 1113 | 21 | 1.639373 | -2.059363 | 52 | 9.08 | 0.174615 |  |
| GO:0001944\_vasculature\_development | APOE | 208 | 7 | 2.924075 | -2.040477 | 53 | 9.31 | 0.175660 |  |
| GO:0001944\_vasculature\_development | PRRX1 | 208 | 7 | 2.924075 | -2.040477 | 53 | 9.31 | 0.175660 |  |
| GO:0001944\_vasculature\_development | FOXC1 | 208 | 7 | 2.924075 | -2.040477 | 53 | 9.31 | 0.175660 |  |
| GO:0001944\_vasculature\_development | PROX1 | 208 | 7 | 2.924075 | -2.040477 | 53 | 9.31 | 0.175660 |  |
| GO:0001944\_vasculature\_development | TGFB2 | 208 | 7 | 2.924075 | -2.040477 | 53 | 9.31 | 0.175660 |  |
| GO:0001944\_vasculature\_development | CITED2 | 208 | 7 | 2.924075 | -2.040477 | 53 | 9.31 | 0.175660 |  |
| GO:0001944\_vasculature\_development | CTNNB1 | 208 | 7 | 2.924075 | -2.040477 | 53 | 9.31 | 0.175660 |  |
| GO:0006357\_regulation\_of\_transcription\_from\_RNA\_polymerase\_II\_promoter | EGR1 | 435 | 11 | 2.197137 | -2.032691 | 54 | 9.63 | 0.178333 |  |
| GO:0006357\_regulation\_of\_transcription\_from\_RNA\_polymerase\_II\_promoter | BMP2 | 435 | 11 | 2.197137 | -2.032691 | 54 | 9.63 | 0.178333 |  |
| GO:0006357\_regulation\_of\_transcription\_from\_RNA\_polymerase\_II\_promoter | KLF12 | 435 | 11 | 2.197137 | -2.032691 | 54 | 9.63 | 0.178333 |  |
| GO:0006357\_regulation\_of\_transcription\_from\_RNA\_polymerase\_II\_promoter | JUN | 435 | 11 | 2.197137 | -2.032691 | 54 | 9.63 | 0.178333 |  |
| GO:0006357\_regulation\_of\_transcription\_from\_RNA\_polymerase\_II\_promoter | TBX5 | 435 | 11 | 2.197137 | -2.032691 | 54 | 9.63 | 0.178333 |  |
| GO:0006357\_regulation\_of\_transcription\_from\_RNA\_polymerase\_II\_promoter | FOXC1 | 435 | 11 | 2.197137 | -2.032691 | 54 | 9.63 | 0.178333 |  |
| GO:0006357\_regulation\_of\_transcription\_from\_RNA\_polymerase\_II\_promoter | RORA | 435 | 11 | 2.197137 | -2.032691 | 54 | 9.63 | 0.178333 |  |
| GO:0006357\_regulation\_of\_transcription\_from\_RNA\_polymerase\_II\_promoter | HBA-X | 435 | 11 | 2.197137 | -2.032691 | 54 | 9.63 | 0.178333 |  |
| GO:0006357\_regulation\_of\_transcription\_from\_RNA\_polymerase\_II\_promoter | FOXP4 | 435 | 11 | 2.197137 | -2.032691 | 54 | 9.63 | 0.178333 |  |
| GO:0006357\_regulation\_of\_transcription\_from\_RNA\_polymerase\_II\_promoter | CITED2 | 435 | 11 | 2.197137 | -2.032691 | 54 | 9.63 | 0.178333 |  |
| GO:0006357\_regulation\_of\_transcription\_from\_RNA\_polymerase\_II\_promoter | CTNNB1 | 435 | 11 | 2.197137 | -2.032691 | 54 | 9.63 | 0.178333 |  |
| GO:0007439\_ectodermal\_gut\_development | WNT5A | 13 | 2 | 13.367199 | -2.029272 | 59 | 10.28 | 0.174237 |  |
| GO:0007439\_ectodermal\_gut\_development | FOXP4 | 13 | 2 | 13.367199 | -2.029272 | 59 | 10.28 | 0.174237 |  |
| GO:0048567\_ectodermal\_gut\_morphogenesis | WNT5A | 13 | 2 | 13.367199 | -2.029272 | 59 | 10.28 | 0.174237 |  |
| GO:0048567\_ectodermal\_gut\_morphogenesis | FOXP4 | 13 | 2 | 13.367199 | -2.029272 | 59 | 10.28 | 0.174237 |  |
| GO:0051495\_positive\_regulation\_of\_cytoskeleton\_organization | PLEK | 13 | 2 | 13.367199 | -2.029272 | 59 | 10.28 | 0.174237 |  |
| GO:0051495\_positive\_regulation\_of\_cytoskeleton\_organization | PROX1 | 13 | 2 | 13.367199 | -2.029272 | 59 | 10.28 | 0.174237 |  |
| GO:0060038\_cardiac\_muscle\_cell\_proliferation | RBP4 | 13 | 2 | 13.367199 | -2.029272 | 59 | 10.28 | 0.174237 |  |
| GO:0060038\_cardiac\_muscle\_cell\_proliferation | FOXC1 | 13 | 2 | 13.367199 | -2.029272 | 59 | 10.28 | 0.174237 |  |
| GO:0060070\_Wnt\_receptor\_signaling\_pathway\_through\_beta-catenin | RSPO3 | 13 | 2 | 13.367199 | -2.029272 | 59 | 10.28 | 0.174237 |  |
| GO:0060070\_Wnt\_receptor\_signaling\_pathway\_through\_beta-catenin | CTNNB1 | 13 | 2 | 13.367199 | -2.029272 | 59 | 10.28 | 0.174237 |  |
| GO:0050789\_regulation\_of\_biological\_process | RHOJ | 2357 | 36 | 1.327079 | -2.010181 | 60 | 10.43 | 0.173833 |  |
| GO:0050789\_regulation\_of\_biological\_process | WNT5A | 2357 | 36 | 1.327079 | -2.010181 | 60 | 10.43 | 0.173833 |  |
| GO:0050789\_regulation\_of\_biological\_process | SCHIP1 | 2357 | 36 | 1.327079 | -2.010181 | 60 | 10.43 | 0.173833 |  |
| GO:0050789\_regulation\_of\_biological\_process | RBP4 | 2357 | 36 | 1.327079 | -2.010181 | 60 | 10.43 | 0.173833 |  |
| GO:0050789\_regulation\_of\_biological\_process | XRCC4 | 2357 | 36 | 1.327079 | -2.010181 | 60 | 10.43 | 0.173833 |  |
| GO:0050789\_regulation\_of\_biological\_process | PRRX1 | 2357 | 36 | 1.327079 | -2.010181 | 60 | 10.43 | 0.173833 |  |
| GO:0050789\_regulation\_of\_biological\_process | ZEB2 | 2357 | 36 | 1.327079 | -2.010181 | 60 | 10.43 | 0.173833 |  |
| GO:0050789\_regulation\_of\_biological\_process | RORA | 2357 | 36 | 1.327079 | -2.010181 | 60 | 10.43 | 0.173833 |  |
| GO:0050789\_regulation\_of\_biological\_process | PRKG1 | 2357 | 36 | 1.327079 | -2.010181 | 60 | 10.43 | 0.173833 |  |
| GO:0050789\_regulation\_of\_biological\_process | CITED2 | 2357 | 36 | 1.327079 | -2.010181 | 60 | 10.43 | 0.173833 |  |
| GO:0050789\_regulation\_of\_biological\_process | CTNNB1 | 2357 | 36 | 1.327079 | -2.010181 | 60 | 10.43 | 0.173833 |  |
| GO:0050789\_regulation\_of\_biological\_process | TGFB2 | 2357 | 36 | 1.327079 | -2.010181 | 60 | 10.43 | 0.173833 |  |
| GO:0050789\_regulation\_of\_biological\_process | A930038C07RIK | 2357 | 36 | 1.327079 | -2.010181 | 60 | 10.43 | 0.173833 |  |
| GO:0050789\_regulation\_of\_biological\_process | APOE | 2357 | 36 | 1.327079 | -2.010181 | 60 | 10.43 | 0.173833 |  |
| GO:0050789\_regulation\_of\_biological\_process | RSPO3 | 2357 | 36 | 1.327079 | -2.010181 | 60 | 10.43 | 0.173833 |  |
| GO:0050789\_regulation\_of\_biological\_process | RB1CC1 | 2357 | 36 | 1.327079 | -2.010181 | 60 | 10.43 | 0.173833 |  |
| GO:0050789\_regulation\_of\_biological\_process | CDC42EP4 | 2357 | 36 | 1.327079 | -2.010181 | 60 | 10.43 | 0.173833 |  |
| GO:0050789\_regulation\_of\_biological\_process | HBA-X | 2357 | 36 | 1.327079 | -2.010181 | 60 | 10.43 | 0.173833 |  |
| GO:0050789\_regulation\_of\_biological\_process | EGR1 | 2357 | 36 | 1.327079 | -2.010181 | 60 | 10.43 | 0.173833 |  |
| GO:0050789\_regulation\_of\_biological\_process | BMP2 | 2357 | 36 | 1.327079 | -2.010181 | 60 | 10.43 | 0.173833 |  |
| GO:0050789\_regulation\_of\_biological\_process | ZC3HC1 | 2357 | 36 | 1.327079 | -2.010181 | 60 | 10.43 | 0.173833 |  |
| GO:0050789\_regulation\_of\_biological\_process | PTPRM | 2357 | 36 | 1.327079 | -2.010181 | 60 | 10.43 | 0.173833 |  |
| GO:0050789\_regulation\_of\_biological\_process | VAV3 | 2357 | 36 | 1.327079 | -2.010181 | 60 | 10.43 | 0.173833 |  |
| GO:0050789\_regulation\_of\_biological\_process | SGK3 | 2357 | 36 | 1.327079 | -2.010181 | 60 | 10.43 | 0.173833 |  |
| GO:0050789\_regulation\_of\_biological\_process | KLF12 | 2357 | 36 | 1.327079 | -2.010181 | 60 | 10.43 | 0.173833 |  |
| GO:0050789\_regulation\_of\_biological\_process | PLEK | 2357 | 36 | 1.327079 | -2.010181 | 60 | 10.43 | 0.173833 |  |
| GO:0050789\_regulation\_of\_biological\_process | TBX5 | 2357 | 36 | 1.327079 | -2.010181 | 60 | 10.43 | 0.173833 |  |
| GO:0050789\_regulation\_of\_biological\_process | CCDC80 | 2357 | 36 | 1.327079 | -2.010181 | 60 | 10.43 | 0.173833 |  |
| GO:0050789\_regulation\_of\_biological\_process | DLL1 | 2357 | 36 | 1.327079 | -2.010181 | 60 | 10.43 | 0.173833 |  |
| GO:0050789\_regulation\_of\_biological\_process | FOXP4 | 2357 | 36 | 1.327079 | -2.010181 | 60 | 10.43 | 0.173833 |  |
| GO:0050789\_regulation\_of\_biological\_process | PROX1 | 2357 | 36 | 1.327079 | -2.010181 | 60 | 10.43 | 0.173833 |  |
| GO:0050789\_regulation\_of\_biological\_process | FZD10 | 2357 | 36 | 1.327079 | -2.010181 | 60 | 10.43 | 0.173833 |  |
| GO:0050789\_regulation\_of\_biological\_process | JUN | 2357 | 36 | 1.327079 | -2.010181 | 60 | 10.43 | 0.173833 |  |
| GO:0050789\_regulation\_of\_biological\_process | FOXC1 | 2357 | 36 | 1.327079 | -2.010181 | 60 | 10.43 | 0.173833 |  |
| GO:0050789\_regulation\_of\_biological\_process | LRP8 | 2357 | 36 | 1.327079 | -2.010181 | 60 | 10.43 | 0.173833 |  |
| GO:0050789\_regulation\_of\_biological\_process | C1D | 2357 | 36 | 1.327079 | -2.010181 | 60 | 10.43 | 0.173833 |  |
| GO:0005976\_polysaccharide\_metabolic\_process | A930038C07RIK | 39 | 3 | 6.683599 | -2.007703 | 61 | 10.6 | 0.173770 |  |
| GO:0005976\_polysaccharide\_metabolic\_process | FOXC1 | 39 | 3 | 6.683599 | -2.007703 | 61 | 10.6 | 0.173770 |  |
| GO:0005976\_polysaccharide\_metabolic\_process | DCN | 39 | 3 | 6.683599 | -2.007703 | 61 | 10.6 | 0.173770 |  |
| GO:0048771\_tissue\_remodeling | BMP2 | 74 | 4 | 4.696583 | -2.002925 | 62 | 10.72 | 0.172903 |  |
| GO:0048771\_tissue\_remodeling | FOXC1 | 74 | 4 | 4.696583 | -2.002925 | 62 | 10.72 | 0.172903 |  |
| GO:0048771\_tissue\_remodeling | CTNNB1 | 74 | 4 | 4.696583 | -2.002925 | 62 | 10.72 | 0.172903 |  |
| GO:0048771\_tissue\_remodeling | TGFB2 | 74 | 4 | 4.696583 | -2.002925 | 62 | 10.72 | 0.172903 |  |
| GO:0035295\_tube\_development | WNT5A | 212 | 7 | 2.868904 | -1.997054 | 63 | 10.76 | 0.170794 |  |
| GO:0035295\_tube\_development | RBP4 | 212 | 7 | 2.868904 | -1.997054 | 63 | 10.76 | 0.170794 |  |
| GO:0035295\_tube\_development | BMP2 | 212 | 7 | 2.868904 | -1.997054 | 63 | 10.76 | 0.170794 |  |
| GO:0035295\_tube\_development | TBX5 | 212 | 7 | 2.868904 | -1.997054 | 63 | 10.76 | 0.170794 |  |
| GO:0035295\_tube\_development | ZEB2 | 212 | 7 | 2.868904 | -1.997054 | 63 | 10.76 | 0.170794 |  |
| GO:0035295\_tube\_development | FOXC1 | 212 | 7 | 2.868904 | -1.997054 | 63 | 10.76 | 0.170794 |  |
| GO:0035295\_tube\_development | CTNNB1 | 212 | 7 | 2.868904 | -1.997054 | 63 | 10.76 | 0.170794 |  |
| GO:0031325\_positive\_regulation\_of\_cellular\_metabolic\_process | BMP2 | 442 | 11 | 2.162341 | -1.982454 | 64 | 10.86 | 0.169688 |  |
| GO:0031325\_positive\_regulation\_of\_cellular\_metabolic\_process | PLEK | 442 | 11 | 2.162341 | -1.982454 | 64 | 10.86 | 0.169688 |  |
| GO:0031325\_positive\_regulation\_of\_cellular\_metabolic\_process | KLF12 | 442 | 11 | 2.162341 | -1.982454 | 64 | 10.86 | 0.169688 |  |
| GO:0031325\_positive\_regulation\_of\_cellular\_metabolic\_process | APOE | 442 | 11 | 2.162341 | -1.982454 | 64 | 10.86 | 0.169688 |  |
| GO:0031325\_positive\_regulation\_of\_cellular\_metabolic\_process | JUN | 442 | 11 | 2.162341 | -1.982454 | 64 | 10.86 | 0.169688 |  |
| GO:0031325\_positive\_regulation\_of\_cellular\_metabolic\_process | TBX5 | 442 | 11 | 2.162341 | -1.982454 | 64 | 10.86 | 0.169688 |  |
| GO:0031325\_positive\_regulation\_of\_cellular\_metabolic\_process | RB1CC1 | 442 | 11 | 2.162341 | -1.982454 | 64 | 10.86 | 0.169688 |  |
| GO:0031325\_positive\_regulation\_of\_cellular\_metabolic\_process | FOXC1 | 442 | 11 | 2.162341 | -1.982454 | 64 | 10.86 | 0.169688 |  |
| GO:0031325\_positive\_regulation\_of\_cellular\_metabolic\_process | RORA | 442 | 11 | 2.162341 | -1.982454 | 64 | 10.86 | 0.169688 |  |
| GO:0031325\_positive\_regulation\_of\_cellular\_metabolic\_process | CTNNB1 | 442 | 11 | 2.162341 | -1.982454 | 64 | 10.86 | 0.169688 |  |
| GO:0031325\_positive\_regulation\_of\_cellular\_metabolic\_process | CITED2 | 442 | 11 | 2.162341 | -1.982454 | 64 | 10.86 | 0.169688 |  |
| GO:0014031\_mesenchymal\_cell\_development | NRP2 | 40 | 3 | 6.516509 | -1.977347 | 65 | 11.05 | 0.170000 |  |
| GO:0014031\_mesenchymal\_cell\_development | FOXC1 | 40 | 3 | 6.516509 | -1.977347 | 65 | 11.05 | 0.170000 |  |
| GO:0014031\_mesenchymal\_cell\_development | ZEB2 | 40 | 3 | 6.516509 | -1.977347 | 65 | 11.05 | 0.170000 |  |
| GO:0006366\_transcription\_from\_RNA\_polymerase\_II\_promoter | EGR1 | 444 | 11 | 2.152601 | -1.968330 | 66 | 11.15 | 0.168939 |  |
| GO:0006366\_transcription\_from\_RNA\_polymerase\_II\_promoter | BMP2 | 444 | 11 | 2.152601 | -1.968330 | 66 | 11.15 | 0.168939 |  |
| GO:0006366\_transcription\_from\_RNA\_polymerase\_II\_promoter | KLF12 | 444 | 11 | 2.152601 | -1.968330 | 66 | 11.15 | 0.168939 |  |
| GO:0006366\_transcription\_from\_RNA\_polymerase\_II\_promoter | JUN | 444 | 11 | 2.152601 | -1.968330 | 66 | 11.15 | 0.168939 |  |
| GO:0006366\_transcription\_from\_RNA\_polymerase\_II\_promoter | TBX5 | 444 | 11 | 2.152601 | -1.968330 | 66 | 11.15 | 0.168939 |  |
| GO:0006366\_transcription\_from\_RNA\_polymerase\_II\_promoter | FOXC1 | 444 | 11 | 2.152601 | -1.968330 | 66 | 11.15 | 0.168939 |  |
| GO:0006366\_transcription\_from\_RNA\_polymerase\_II\_promoter | RORA | 444 | 11 | 2.152601 | -1.968330 | 66 | 11.15 | 0.168939 |  |
| GO:0006366\_transcription\_from\_RNA\_polymerase\_II\_promoter | HBA-X | 444 | 11 | 2.152601 | -1.968330 | 66 | 11.15 | 0.168939 |  |
| GO:0006366\_transcription\_from\_RNA\_polymerase\_II\_promoter | FOXP4 | 444 | 11 | 2.152601 | -1.968330 | 66 | 11.15 | 0.168939 |  |
| GO:0006366\_transcription\_from\_RNA\_polymerase\_II\_promoter | CITED2 | 444 | 11 | 2.152601 | -1.968330 | 66 | 11.15 | 0.168939 |  |
| GO:0006366\_transcription\_from\_RNA\_polymerase\_II\_promoter | CTNNB1 | 444 | 11 | 2.152601 | -1.968330 | 66 | 11.15 | 0.168939 |  |
| GO:0014855\_striated\_muscle\_cell\_proliferation | RBP4 | 14 | 2 | 12.412399 | -1.965517 | 67 | 11.86 | 0.177015 |  |
| GO:0014855\_striated\_muscle\_cell\_proliferation | FOXC1 | 14 | 2 | 12.412399 | -1.965517 | 67 | 11.86 | 0.177015 |  |
| GO:0045944\_positive\_regulation\_of\_transcription\_from\_RNA\_polymerase\_II\_promoter | BMP2 | 269 | 8 | 2.583994 | -1.962275 | 68 | 11.9 | 0.175000 |  |
| GO:0045944\_positive\_regulation\_of\_transcription\_from\_RNA\_polymerase\_II\_promoter | KLF12 | 269 | 8 | 2.583994 | -1.962275 | 68 | 11.9 | 0.175000 |  |
| GO:0045944\_positive\_regulation\_of\_transcription\_from\_RNA\_polymerase\_II\_promoter | JUN | 269 | 8 | 2.583994 | -1.962275 | 68 | 11.9 | 0.175000 |  |
| GO:0045944\_positive\_regulation\_of\_transcription\_from\_RNA\_polymerase\_II\_promoter | TBX5 | 269 | 8 | 2.583994 | -1.962275 | 68 | 11.9 | 0.175000 |  |
| GO:0045944\_positive\_regulation\_of\_transcription\_from\_RNA\_polymerase\_II\_promoter | FOXC1 | 269 | 8 | 2.583994 | -1.962275 | 68 | 11.9 | 0.175000 |  |
| GO:0045944\_positive\_regulation\_of\_transcription\_from\_RNA\_polymerase\_II\_promoter | RORA | 269 | 8 | 2.583994 | -1.962275 | 68 | 11.9 | 0.175000 |  |
| GO:0045944\_positive\_regulation\_of\_transcription\_from\_RNA\_polymerase\_II\_promoter | CTNNB1 | 269 | 8 | 2.583994 | -1.962275 | 68 | 11.9 | 0.175000 |  |
| GO:0045944\_positive\_regulation\_of\_transcription\_from\_RNA\_polymerase\_II\_promoter | CITED2 | 269 | 8 | 2.583994 | -1.962275 | 68 | 11.9 | 0.175000 |  |
| GO:0030154\_cell\_differentiation | INSM1 | 1060 | 20 | 1.639373 | -1.961807 | 69 | 11.91 | 0.172609 |  |
| GO:0030154\_cell\_differentiation | NRP2 | 1060 | 20 | 1.639373 | -1.961807 | 69 | 11.91 | 0.172609 |  |
| GO:0030154\_cell\_differentiation | EGR1 | 1060 | 20 | 1.639373 | -1.961807 | 69 | 11.91 | 0.172609 |  |
| GO:0030154\_cell\_differentiation | RBP4 | 1060 | 20 | 1.639373 | -1.961807 | 69 | 11.91 | 0.172609 |  |
| GO:0030154\_cell\_differentiation | SEPT4 | 1060 | 20 | 1.639373 | -1.961807 | 69 | 11.91 | 0.172609 |  |
| GO:0030154\_cell\_differentiation | XRCC4 | 1060 | 20 | 1.639373 | -1.961807 | 69 | 11.91 | 0.172609 |  |
| GO:0030154\_cell\_differentiation | BMP2 | 1060 | 20 | 1.639373 | -1.961807 | 69 | 11.91 | 0.172609 |  |
| GO:0030154\_cell\_differentiation | TBX5 | 1060 | 20 | 1.639373 | -1.961807 | 69 | 11.91 | 0.172609 |  |
| GO:0030154\_cell\_differentiation | ZEB2 | 1060 | 20 | 1.639373 | -1.961807 | 69 | 11.91 | 0.172609 |  |
| GO:0030154\_cell\_differentiation | DLL1 | 1060 | 20 | 1.639373 | -1.961807 | 69 | 11.91 | 0.172609 |  |
| GO:0030154\_cell\_differentiation | RORA | 1060 | 20 | 1.639373 | -1.961807 | 69 | 11.91 | 0.172609 |  |
| GO:0030154\_cell\_differentiation | PRKG1 | 1060 | 20 | 1.639373 | -1.961807 | 69 | 11.91 | 0.172609 |  |
| GO:0030154\_cell\_differentiation | PROX1 | 1060 | 20 | 1.639373 | -1.961807 | 69 | 11.91 | 0.172609 |  |
| GO:0030154\_cell\_differentiation | CITED2 | 1060 | 20 | 1.639373 | -1.961807 | 69 | 11.91 | 0.172609 |  |
| GO:0030154\_cell\_differentiation | CTNNB1 | 1060 | 20 | 1.639373 | -1.961807 | 69 | 11.91 | 0.172609 |  |
| GO:0030154\_cell\_differentiation | TGFB2 | 1060 | 20 | 1.639373 | -1.961807 | 69 | 11.91 | 0.172609 |  |
| GO:0030154\_cell\_differentiation | APOE | 1060 | 20 | 1.639373 | -1.961807 | 69 | 11.91 | 0.172609 |  |
| GO:0030154\_cell\_differentiation | JUN | 1060 | 20 | 1.639373 | -1.961807 | 69 | 11.91 | 0.172609 |  |
| GO:0030154\_cell\_differentiation | FOXC1 | 1060 | 20 | 1.639373 | -1.961807 | 69 | 11.91 | 0.172609 |  |
| GO:0030154\_cell\_differentiation | HBA-X | 1060 | 20 | 1.639373 | -1.961807 | 69 | 11.91 | 0.172609 |  |
| GO:0007254\_JNK\_cascade | WNT5A | 41 | 3 | 6.357570 | -1.947847 | 70 | 12.16 | 0.173714 |  |
| GO:0007254\_JNK\_cascade | RB1CC1 | 41 | 3 | 6.357570 | -1.947847 | 70 | 12.16 | 0.173714 |  |
| GO:0007254\_JNK\_cascade | ZEB2 | 41 | 3 | 6.357570 | -1.947847 | 70 | 12.16 | 0.173714 |  |
| GO:0007284\_spermatogonial\_cell\_division | RBP4 | 1 | 1 |  |  |  |  |  |  |
| GO:0010543\_regulation\_of\_platelet\_activation | PLEK | 1 | 1 |  |  |  |  |  |  |
| GO:0010572\_positive\_regulation\_of\_platelet\_activation | PLEK | 1 | 1 |  |  |  |  |  |  |
| GO:0010873\_positive\_regulation\_of\_cholesterol\_esterification | APOE | 1 | 1 |  |  |  |  |  |  |
| GO:0030382\_sperm\_mitochondrion\_organization | SEPT4 | 1 | 1 |  |  |  |  |  |  |
| GO:0034372\_very-low-density\_lipoprotein\_particle\_remodeling | APOE | 1 | 1 |  |  |  |  |  |  |
| GO:0034633\_retinol\_transport | RBP4 | 1 | 1 |  |  |  |  |  |  |
| GO:0035026\_leading\_edge\_cell\_differentiation | JUN | 1 | 1 |  |  |  |  |  |  |
| GO:0045909\_positive\_regulation\_of\_vasodilation | PTPRM | 1 | 1 |  |  |  |  |  |  |
| GO:0046864\_isoprenoid\_transport | RBP4 | 1 | 1 |  |  |  |  |  |  |
| GO:0046865\_terpenoid\_transport | RBP4 | 1 | 1 |  |  |  |  |  |  |
| GO:0055005\_ventricular\_cardiac\_myofibril\_development | PROX1 | 1 | 1 |  |  |  |  |  |  |
| GO:0060059\_embryonic\_retina\_morphogenesis\_in\_camera-type\_eye | RBP4 | 1 | 1 |  |  |  |  |  |  |
| GO:0060297\_regulation\_of\_sarcomere\_organization | PROX1 | 1 | 1 |  |  |  |  |  |  |
| GO:0060298\_positive\_regulation\_of\_sarcomere\_organization | PROX1 | 1 | 1 |  |  |  |  |  |  |
| GO:0060414\_aorta\_smooth\_muscle\_tissue\_morphogenesis | PROX1 | 1 | 1 |  |  |  |  |  |  |
| GO:0060419\_heart\_growth | PROX1 | 1 | 1 |  |  |  |  |  |  |
| GO:0060420\_regulation\_of\_heart\_growth | PROX1 | 1 | 1 |  |  |  |  |  |  |
| GO:0060421\_positive\_regulation\_of\_heart\_growth | PROX1 | 1 | 1 |  |  |  |  |  |  |
| GO:0070493\_thrombin\_receptor\_signaling\_pathway | PLEK | 1 | 1 |  |  |  |  |  |  |
| GO:0070527\_platelet\_aggregation | PLEK | 1 | 1 |  |  |  |  |  |  |
| GO:0070528\_protein\_kinase\_C\_signaling\_cascade | PLEK | 1 | 1 |  |  |  |  |  |  |
| GO:0070560\_protein\_secretion\_by\_platelet | PLEK | 1 | 1 |  |  |  |  |  |  |
| GO:0030326\_embryonic\_limb\_morphogenesis | WNT5A | 78 | 4 | 4.455733 | -1.924248 | 72 | 12.46 | 0.173056 |  |
| GO:0030326\_embryonic\_limb\_morphogenesis | TBX5 | 78 | 4 | 4.455733 | -1.924248 | 72 | 12.46 | 0.173056 |  |
| GO:0030326\_embryonic\_limb\_morphogenesis | PRRX1 | 78 | 4 | 4.455733 | -1.924248 | 72 | 12.46 | 0.173056 |  |
| GO:0030326\_embryonic\_limb\_morphogenesis | CTNNB1 | 78 | 4 | 4.455733 | -1.924248 | 72 | 12.46 | 0.173056 |  |
| GO:0035113\_embryonic\_appendage\_morphogenesis | WNT5A | 78 | 4 | 4.455733 | -1.924248 | 72 | 12.46 | 0.173056 |  |
| GO:0035113\_embryonic\_appendage\_morphogenesis | TBX5 | 78 | 4 | 4.455733 | -1.924248 | 72 | 12.46 | 0.173056 |  |
| GO:0035113\_embryonic\_appendage\_morphogenesis | PRRX1 | 78 | 4 | 4.455733 | -1.924248 | 72 | 12.46 | 0.173056 |  |
| GO:0035113\_embryonic\_appendage\_morphogenesis | CTNNB1 | 78 | 4 | 4.455733 | -1.924248 | 72 | 12.46 | 0.173056 |  |
| GO:0055010\_ventricular\_cardiac\_muscle\_morphogenesis | FOXC1 | 15 | 2 | 11.584906 | -1.906559 | 73 | 13.66 | 0.187123 |  |
| GO:0055010\_ventricular\_cardiac\_muscle\_morphogenesis | PROX1 | 15 | 2 | 11.584906 | -1.906559 | 73 | 13.66 | 0.187123 |  |
| GO:0031098\_stress-activated\_protein\_kinase\_signaling\_pathway | WNT5A | 43 | 3 | 6.061869 | -1.891250 | 75 | 13.99 | 0.186533 |  |
| GO:0031098\_stress-activated\_protein\_kinase\_signaling\_pathway | RB1CC1 | 43 | 3 | 6.061869 | -1.891250 | 75 | 13.99 | 0.186533 |  |
| GO:0031098\_stress-activated\_protein\_kinase\_signaling\_pathway | ZEB2 | 43 | 3 | 6.061869 | -1.891250 | 75 | 13.99 | 0.186533 |  |
| GO:0048762\_mesenchymal\_cell\_differentiation | NRP2 | 43 | 3 | 6.061869 | -1.891250 | 75 | 13.99 | 0.186533 |  |
| GO:0048762\_mesenchymal\_cell\_differentiation | FOXC1 | 43 | 3 | 6.061869 | -1.891250 | 75 | 13.99 | 0.186533 |  |
| GO:0048762\_mesenchymal\_cell\_differentiation | ZEB2 | 43 | 3 | 6.061869 | -1.891250 | 75 | 13.99 | 0.186533 |  |
| GO:0048519\_negative\_regulation\_of\_biological\_process | EGR1 | 859 | 17 | 1.719529 | -1.883542 | 76 | 14.16 | 0.186316 |  |
| GO:0048519\_negative\_regulation\_of\_biological\_process | WNT5A | 859 | 17 | 1.719529 | -1.883542 | 76 | 14.16 | 0.186316 |  |
| GO:0048519\_negative\_regulation\_of\_biological\_process | XRCC4 | 859 | 17 | 1.719529 | -1.883542 | 76 | 14.16 | 0.186316 |  |
| GO:0048519\_negative\_regulation\_of\_biological\_process | RBP4 | 859 | 17 | 1.719529 | -1.883542 | 76 | 14.16 | 0.186316 |  |
| GO:0048519\_negative\_regulation\_of\_biological\_process | BMP2 | 859 | 17 | 1.719529 | -1.883542 | 76 | 14.16 | 0.186316 |  |
| GO:0048519\_negative\_regulation\_of\_biological\_process | ZC3HC1 | 859 | 17 | 1.719529 | -1.883542 | 76 | 14.16 | 0.186316 |  |
| GO:0048519\_negative\_regulation\_of\_biological\_process | SGK3 | 859 | 17 | 1.719529 | -1.883542 | 76 | 14.16 | 0.186316 |  |
| GO:0048519\_negative\_regulation\_of\_biological\_process | DLL1 | 859 | 17 | 1.719529 | -1.883542 | 76 | 14.16 | 0.186316 |  |
| GO:0048519\_negative\_regulation\_of\_biological\_process | FOXP4 | 859 | 17 | 1.719529 | -1.883542 | 76 | 14.16 | 0.186316 |  |
| GO:0048519\_negative\_regulation\_of\_biological\_process | PROX1 | 859 | 17 | 1.719529 | -1.883542 | 76 | 14.16 | 0.186316 |  |
| GO:0048519\_negative\_regulation\_of\_biological\_process | CTNNB1 | 859 | 17 | 1.719529 | -1.883542 | 76 | 14.16 | 0.186316 |  |
| GO:0048519\_negative\_regulation\_of\_biological\_process | CITED2 | 859 | 17 | 1.719529 | -1.883542 | 76 | 14.16 | 0.186316 |  |
| GO:0048519\_negative\_regulation\_of\_biological\_process | RB1CC1 | 859 | 17 | 1.719529 | -1.883542 | 76 | 14.16 | 0.186316 |  |
| GO:0048519\_negative\_regulation\_of\_biological\_process | JUN | 859 | 17 | 1.719529 | -1.883542 | 76 | 14.16 | 0.186316 |  |
| GO:0048519\_negative\_regulation\_of\_biological\_process | FOXC1 | 859 | 17 | 1.719529 | -1.883542 | 76 | 14.16 | 0.186316 |  |
| GO:0048519\_negative\_regulation\_of\_biological\_process | HBA-X | 859 | 17 | 1.719529 | -1.883542 | 76 | 14.16 | 0.186316 |  |
| GO:0048519\_negative\_regulation\_of\_biological\_process | C1D | 859 | 17 | 1.719529 | -1.883542 | 76 | 14.16 | 0.186316 |  |
| GO:0009893\_positive\_regulation\_of\_metabolic\_process | BMP2 | 458 | 11 | 2.086801 | -1.872226 | 77 | 14.32 | 0.185974 |  |
| GO:0009893\_positive\_regulation\_of\_metabolic\_process | PLEK | 458 | 11 | 2.086801 | -1.872226 | 77 | 14.32 | 0.185974 |  |
| GO:0009893\_positive\_regulation\_of\_metabolic\_process | KLF12 | 458 | 11 | 2.086801 | -1.872226 | 77 | 14.32 | 0.185974 |  |
| GO:0009893\_positive\_regulation\_of\_metabolic\_process | APOE | 458 | 11 | 2.086801 | -1.872226 | 77 | 14.32 | 0.185974 |  |
| GO:0009893\_positive\_regulation\_of\_metabolic\_process | RB1CC1 | 458 | 11 | 2.086801 | -1.872226 | 77 | 14.32 | 0.185974 |  |
| GO:0009893\_positive\_regulation\_of\_metabolic\_process | JUN | 458 | 11 | 2.086801 | -1.872226 | 77 | 14.32 | 0.185974 |  |
| GO:0009893\_positive\_regulation\_of\_metabolic\_process | TBX5 | 458 | 11 | 2.086801 | -1.872226 | 77 | 14.32 | 0.185974 |  |
| GO:0009893\_positive\_regulation\_of\_metabolic\_process | FOXC1 | 458 | 11 | 2.086801 | -1.872226 | 77 | 14.32 | 0.185974 |  |
| GO:0009893\_positive\_regulation\_of\_metabolic\_process | RORA | 458 | 11 | 2.086801 | -1.872226 | 77 | 14.32 | 0.185974 |  |
| GO:0009893\_positive\_regulation\_of\_metabolic\_process | CTNNB1 | 458 | 11 | 2.086801 | -1.872226 | 77 | 14.32 | 0.185974 |  |
| GO:0009893\_positive\_regulation\_of\_metabolic\_process | CITED2 | 458 | 11 | 2.086801 | -1.872226 | 77 | 14.32 | 0.185974 |  |
| GO:0035282\_segmentation | FOXC1 | 44 | 3 | 5.924099 | -1.864078 | 80 | 14.6 | 0.182500 |  |
| GO:0035282\_segmentation | ZEB2 | 44 | 3 | 5.924099 | -1.864078 | 80 | 14.6 | 0.182500 |  |
| GO:0035282\_segmentation | DLL1 | 44 | 3 | 5.924099 | -1.864078 | 80 | 14.6 | 0.182500 |  |
| GO:0048593\_camera-type\_eye\_morphogenesis | RBP4 | 44 | 3 | 5.924099 | -1.864078 | 80 | 14.6 | 0.182500 |  |
| GO:0048593\_camera-type\_eye\_morphogenesis | PROX1 | 44 | 3 | 5.924099 | -1.864078 | 80 | 14.6 | 0.182500 |  |
| GO:0048593\_camera-type\_eye\_morphogenesis | CTNNB1 | 44 | 3 | 5.924099 | -1.864078 | 80 | 14.6 | 0.182500 |  |
| GO:0060485\_mesenchyme\_development | NRP2 | 44 | 3 | 5.924099 | -1.864078 | 80 | 14.6 | 0.182500 |  |
| GO:0060485\_mesenchyme\_development | FOXC1 | 44 | 3 | 5.924099 | -1.864078 | 80 | 14.6 | 0.182500 |  |
| GO:0060485\_mesenchyme\_development | ZEB2 | 44 | 3 | 5.924099 | -1.864078 | 80 | 14.6 | 0.182500 |  |
| GO:0043062\_extracellular\_structure\_organization | A930038C07RIK | 125 | 5 | 3.475472 | -1.860682 | 81 | 14.65 | 0.180864 |  |
| GO:0043062\_extracellular\_structure\_organization | CCDC80 | 125 | 5 | 3.475472 | -1.860682 | 81 | 14.65 | 0.180864 |  |
| GO:0043062\_extracellular\_structure\_organization | FOXC1 | 125 | 5 | 3.475472 | -1.860682 | 81 | 14.65 | 0.180864 |  |
| GO:0043062\_extracellular\_structure\_organization | CTNNB1 | 125 | 5 | 3.475472 | -1.860682 | 81 | 14.65 | 0.180864 |  |
| GO:0043062\_extracellular\_structure\_organization | TGFB2 | 125 | 5 | 3.475472 | -1.860682 | 81 | 14.65 | 0.180864 |  |
| GO:0032956\_regulation\_of\_actin\_cytoskeleton\_organization | PLEK | 16 | 2 | 10.860849 | -1.851754 | 84 | 15.27 | 0.181786 |  |
| GO:0032956\_regulation\_of\_actin\_cytoskeleton\_organization | PROX1 | 16 | 2 | 10.860849 | -1.851754 | 84 | 15.27 | 0.181786 |  |
| GO:0042311\_vasodilation | PTPRM | 16 | 2 | 10.860849 | -1.851754 | 84 | 15.27 | 0.181786 |  |
| GO:0042311\_vasodilation | APOE | 16 | 2 | 10.860849 | -1.851754 | 84 | 15.27 | 0.181786 |  |
| GO:0055007\_cardiac\_muscle\_cell\_differentiation | TBX5 | 16 | 2 | 10.860849 | -1.851754 | 84 | 15.27 | 0.181786 |  |
| GO:0055007\_cardiac\_muscle\_cell\_differentiation | PROX1 | 16 | 2 | 10.860849 | -1.851754 | 84 | 15.27 | 0.181786 |  |
| GO:0030198\_extracellular\_matrix\_organization | A930038C07RIK | 83 | 4 | 4.187315 | -1.832518 | 85 | 15.61 | 0.183647 |  |
| GO:0030198\_extracellular\_matrix\_organization | CCDC80 | 83 | 4 | 4.187315 | -1.832518 | 85 | 15.61 | 0.183647 |  |
| GO:0030198\_extracellular\_matrix\_organization | FOXC1 | 83 | 4 | 4.187315 | -1.832518 | 85 | 15.61 | 0.183647 |  |
| GO:0030198\_extracellular\_matrix\_organization | TGFB2 | 83 | 4 | 4.187315 | -1.832518 | 85 | 15.61 | 0.183647 |  |
| GO:0043066\_negative\_regulation\_of\_apoptosis | XRCC4 | 176 | 6 | 2.962050 | -1.829827 | 86 | 15.65 | 0.181977 |  |
| GO:0043066\_negative\_regulation\_of\_apoptosis | ZC3HC1 | 176 | 6 | 2.962050 | -1.829827 | 86 | 15.65 | 0.181977 |  |
| GO:0043066\_negative\_regulation\_of\_apoptosis | SGK3 | 176 | 6 | 2.962050 | -1.829827 | 86 | 15.65 | 0.181977 |  |
| GO:0043066\_negative\_regulation\_of\_apoptosis | RB1CC1 | 176 | 6 | 2.962050 | -1.829827 | 86 | 15.65 | 0.181977 |  |
| GO:0043066\_negative\_regulation\_of\_apoptosis | FOXC1 | 176 | 6 | 2.962050 | -1.829827 | 86 | 15.65 | 0.181977 |  |
| GO:0043066\_negative\_regulation\_of\_apoptosis | CITED2 | 176 | 6 | 2.962050 | -1.829827 | 86 | 15.65 | 0.181977 |  |
| GO:0032970\_regulation\_of\_actin\_filament-based\_process | PLEK | 17 | 2 | 10.221976 | -1.800581 | 89 | 16.9 | 0.189888 |  |
| GO:0032970\_regulation\_of\_actin\_filament-based\_process | PROX1 | 17 | 2 | 10.221976 | -1.800581 | 89 | 16.9 | 0.189888 |  |
| GO:0035115\_embryonic\_forelimb\_morphogenesis | TBX5 | 17 | 2 | 10.221976 | -1.800581 | 89 | 16.9 | 0.189888 |  |
| GO:0035115\_embryonic\_forelimb\_morphogenesis | CTNNB1 | 17 | 2 | 10.221976 | -1.800581 | 89 | 16.9 | 0.189888 |  |
| GO:0045667\_regulation\_of\_osteoblast\_differentiation | BMP2 | 17 | 2 | 10.221976 | -1.800581 | 89 | 16.9 | 0.189888 |  |
| GO:0045667\_regulation\_of\_osteoblast\_differentiation | CTNNB1 | 17 | 2 | 10.221976 | -1.800581 | 89 | 16.9 | 0.189888 |  |
| GO:0043069\_negative\_regulation\_of\_programmed\_cell\_death | XRCC4 | 179 | 6 | 2.912406 | -1.796545 | 91 | 16.94 | 0.186154 |  |
| GO:0043069\_negative\_regulation\_of\_programmed\_cell\_death | ZC3HC1 | 179 | 6 | 2.912406 | -1.796545 | 91 | 16.94 | 0.186154 |  |
| GO:0043069\_negative\_regulation\_of\_programmed\_cell\_death | SGK3 | 179 | 6 | 2.912406 | -1.796545 | 91 | 16.94 | 0.186154 |  |
| GO:0043069\_negative\_regulation\_of\_programmed\_cell\_death | RB1CC1 | 179 | 6 | 2.912406 | -1.796545 | 91 | 16.94 | 0.186154 |  |
| GO:0043069\_negative\_regulation\_of\_programmed\_cell\_death | FOXC1 | 179 | 6 | 2.912406 | -1.796545 | 91 | 16.94 | 0.186154 |  |
| GO:0043069\_negative\_regulation\_of\_programmed\_cell\_death | CITED2 | 179 | 6 | 2.912406 | -1.796545 | 91 | 16.94 | 0.186154 |  |
| GO:0060548\_negative\_regulation\_of\_cell\_death | XRCC4 | 179 | 6 | 2.912406 | -1.796545 | 91 | 16.94 | 0.186154 |  |
| GO:0060548\_negative\_regulation\_of\_cell\_death | ZC3HC1 | 179 | 6 | 2.912406 | -1.796545 | 91 | 16.94 | 0.186154 |  |
| GO:0060548\_negative\_regulation\_of\_cell\_death | SGK3 | 179 | 6 | 2.912406 | -1.796545 | 91 | 16.94 | 0.186154 |  |
| GO:0060548\_negative\_regulation\_of\_cell\_death | RB1CC1 | 179 | 6 | 2.912406 | -1.796545 | 91 | 16.94 | 0.186154 |  |
| GO:0060548\_negative\_regulation\_of\_cell\_death | FOXC1 | 179 | 6 | 2.912406 | -1.796545 | 91 | 16.94 | 0.186154 |  |
| GO:0060548\_negative\_regulation\_of\_cell\_death | CITED2 | 179 | 6 | 2.912406 | -1.796545 | 91 | 16.94 | 0.186154 |  |
| GO:0016477\_cell\_migration | NRP2 | 234 | 7 | 2.599178 | -1.777051 | 92 | 17.42 | 0.189348 |  |
| GO:0016477\_cell\_migration | SCHIP1 | 234 | 7 | 2.599178 | -1.777051 | 92 | 17.42 | 0.189348 |  |
| GO:0016477\_cell\_migration | VAV3 | 234 | 7 | 2.599178 | -1.777051 | 92 | 17.42 | 0.189348 |  |
| GO:0016477\_cell\_migration | LRP8 | 234 | 7 | 2.599178 | -1.777051 | 92 | 17.42 | 0.189348 |  |
| GO:0016477\_cell\_migration | FOXC1 | 234 | 7 | 2.599178 | -1.777051 | 92 | 17.42 | 0.189348 |  |
| GO:0016477\_cell\_migration | ZEB2 | 234 | 7 | 2.599178 | -1.777051 | 92 | 17.42 | 0.189348 |  |
| GO:0016477\_cell\_migration | PRKG1 | 234 | 7 | 2.599178 | -1.777051 | 92 | 17.42 | 0.189348 |  |
| GO:0016337\_cell-cell\_adhesion | PLEK | 87 | 4 | 3.994795 | -1.763874 | 93 | 17.61 | 0.189355 |  |
| GO:0016337\_cell-cell\_adhesion | CTNNB1 | 87 | 4 | 3.994795 | -1.763874 | 93 | 17.61 | 0.189355 |  |
| GO:0016337\_cell-cell\_adhesion | CITED2 | 87 | 4 | 3.994795 | -1.763874 | 93 | 17.61 | 0.189355 |  |
| GO:0016337\_cell-cell\_adhesion | TGFB2 | 87 | 4 | 3.994795 | -1.763874 | 93 | 17.61 | 0.189355 |  |
| GO:0009952\_anterior\_posterior\_pattern\_formation | BTG2 | 133 | 5 | 3.266421 | -1.753778 | 94 | 17.78 | 0.189149 |  |
| GO:0009952\_anterior\_posterior\_pattern\_formation | FOXC1 | 133 | 5 | 3.266421 | -1.753778 | 94 | 17.78 | 0.189149 |  |
| GO:0009952\_anterior\_posterior\_pattern\_formation | ZEB2 | 133 | 5 | 3.266421 | -1.753778 | 94 | 17.78 | 0.189149 |  |
| GO:0009952\_anterior\_posterior\_pattern\_formation | DLL1 | 133 | 5 | 3.266421 | -1.753778 | 94 | 17.78 | 0.189149 |  |
| GO:0009952\_anterior\_posterior\_pattern\_formation | CTNNB1 | 133 | 5 | 3.266421 | -1.753778 | 94 | 17.78 | 0.189149 |  |
| GO:0001974\_blood\_vessel\_remodeling | FOXC1 | 18 | 2 | 9.654088 | -1.752610 | 99 | 18.73 | 0.189192 |  |
| GO:0001974\_blood\_vessel\_remodeling | TGFB2 | 18 | 2 | 9.654088 | -1.752610 | 99 | 18.73 | 0.189192 |  |
| GO:0035051\_cardiac\_cell\_differentiation | TBX5 | 18 | 2 | 9.654088 | -1.752610 | 99 | 18.73 | 0.189192 |  |
| GO:0035051\_cardiac\_cell\_differentiation | PROX1 | 18 | 2 | 9.654088 | -1.752610 | 99 | 18.73 | 0.189192 |  |
| GO:0046620\_regulation\_of\_organ\_growth | FOXC1 | 18 | 2 | 9.654088 | -1.752610 | 99 | 18.73 | 0.189192 |  |
| GO:0046620\_regulation\_of\_organ\_growth | PROX1 | 18 | 2 | 9.654088 | -1.752610 | 99 | 18.73 | 0.189192 |  |
| GO:0055008\_cardiac\_muscle\_tissue\_morphogenesis | FOXC1 | 18 | 2 | 9.654088 | -1.752610 | 99 | 18.73 | 0.189192 |  |
| GO:0055008\_cardiac\_muscle\_tissue\_morphogenesis | PROX1 | 18 | 2 | 9.654088 | -1.752610 | 99 | 18.73 | 0.189192 |  |
| GO:0060415\_muscle\_tissue\_morphogenesis | FOXC1 | 18 | 2 | 9.654088 | -1.752610 | 99 | 18.73 | 0.189192 |  |
| GO:0060415\_muscle\_tissue\_morphogenesis | PROX1 | 18 | 2 | 9.654088 | -1.752610 | 99 | 18.73 | 0.189192 |  |
| GO:0046660\_female\_sex\_differentiation | WNT5A | 49 | 3 | 5.319600 | -1.738170 | 100 | 19.03 | 0.190300 |  |
| GO:0046660\_female\_sex\_differentiation | RBP4 | 49 | 3 | 5.319600 | -1.738170 | 100 | 19.03 | 0.190300 |  |
| GO:0046660\_female\_sex\_differentiation | FOXC1 | 49 | 3 | 5.319600 | -1.738170 | 100 | 19.03 | 0.190300 |  |
| GO:0030324\_lung\_development | WNT5A | 90 | 4 | 3.861635 | -1.714898 | 101 | 19.25 | 0.190594 |  |
| GO:0030324\_lung\_development | RBP4 | 90 | 4 | 3.861635 | -1.714898 | 101 | 19.25 | 0.190594 |  |
| GO:0030324\_lung\_development | TBX5 | 90 | 4 | 3.861635 | -1.714898 | 101 | 19.25 | 0.190594 |  |
| GO:0030324\_lung\_development | CTNNB1 | 90 | 4 | 3.861635 | -1.714898 | 101 | 19.25 | 0.190594 |  |
| GO:0009584\_detection\_of\_visible\_light | RBP4 | 19 | 2 | 9.145978 | -1.707485 | 106 | 20.11 | 0.189717 |  |
| GO:0009584\_detection\_of\_visible\_light | RGS9BP | 19 | 2 | 9.145978 | -1.707485 | 106 | 20.11 | 0.189717 |  |
| GO:0033002\_muscle\_cell\_proliferation | RBP4 | 19 | 2 | 9.145978 | -1.707485 | 106 | 20.11 | 0.189717 |  |
| GO:0033002\_muscle\_cell\_proliferation | FOXC1 | 19 | 2 | 9.145978 | -1.707485 | 106 | 20.11 | 0.189717 |  |
| GO:0048547\_gut\_morphogenesis | WNT5A | 19 | 2 | 9.145978 | -1.707485 | 106 | 20.11 | 0.189717 |  |
| GO:0048547\_gut\_morphogenesis | FOXP4 | 19 | 2 | 9.145978 | -1.707485 | 106 | 20.11 | 0.189717 |  |
| GO:0050908\_detection\_of\_light\_stimulus\_involved\_in\_visual\_perception | RBP4 | 19 | 2 | 9.145978 | -1.707485 | 106 | 20.11 | 0.189717 |  |
| GO:0050908\_detection\_of\_light\_stimulus\_involved\_in\_visual\_perception | RGS9BP | 19 | 2 | 9.145978 | -1.707485 | 106 | 20.11 | 0.189717 |  |
| GO:0050962\_detection\_of\_light\_stimulus\_involved\_in\_sensory\_perception | RBP4 | 19 | 2 | 9.145978 | -1.707485 | 106 | 20.11 | 0.189717 |  |
| GO:0050962\_detection\_of\_light\_stimulus\_involved\_in\_sensory\_perception | RGS9BP | 19 | 2 | 9.145978 | -1.707485 | 106 | 20.11 | 0.189717 |  |
| GO:0030323\_respiratory\_tube\_development | WNT5A | 92 | 4 | 3.777687 | -1.683361 | 107 | 20.52 | 0.191776 |  |
| GO:0030323\_respiratory\_tube\_development | RBP4 | 92 | 4 | 3.777687 | -1.683361 | 107 | 20.52 | 0.191776 |  |
| GO:0030323\_respiratory\_tube\_development | TBX5 | 92 | 4 | 3.777687 | -1.683361 | 107 | 20.52 | 0.191776 |  |
| GO:0030323\_respiratory\_tube\_development | CTNNB1 | 92 | 4 | 3.777687 | -1.683361 | 107 | 20.52 | 0.191776 |  |
| GO:0035107\_appendage\_morphogenesis | WNT5A | 93 | 4 | 3.737066 | -1.667912 | 109 | 20.72 | 0.190092 |  |
| GO:0035107\_appendage\_morphogenesis | TBX5 | 93 | 4 | 3.737066 | -1.667912 | 109 | 20.72 | 0.190092 |  |
| GO:0035107\_appendage\_morphogenesis | PRRX1 | 93 | 4 | 3.737066 | -1.667912 | 109 | 20.72 | 0.190092 |  |
| GO:0035107\_appendage\_morphogenesis | CTNNB1 | 93 | 4 | 3.737066 | -1.667912 | 109 | 20.72 | 0.190092 |  |
| GO:0035108\_limb\_morphogenesis | WNT5A | 93 | 4 | 3.737066 | -1.667912 | 109 | 20.72 | 0.190092 |  |
| GO:0035108\_limb\_morphogenesis | TBX5 | 93 | 4 | 3.737066 | -1.667912 | 109 | 20.72 | 0.190092 |  |
| GO:0035108\_limb\_morphogenesis | PRRX1 | 93 | 4 | 3.737066 | -1.667912 | 109 | 20.72 | 0.190092 |  |
| GO:0035108\_limb\_morphogenesis | CTNNB1 | 93 | 4 | 3.737066 | -1.667912 | 109 | 20.72 | 0.190092 |  |
| GO:0018209\_peptidyl-serine\_modification | A930038C07RIK | 20 | 2 | 8.688679 | -1.664904 | 110 | 21.23 | 0.193000 |  |
| GO:0018209\_peptidyl-serine\_modification | DCN | 20 | 2 | 8.688679 | -1.664904 | 110 | 21.23 | 0.193000 |  |
| GO:0045893\_positive\_regulation\_of\_transcription\_\_DNA-dependent | BMP2 | 306 | 8 | 2.271550 | -1.651336 | 112 | 21.46 | 0.191607 |  |
| GO:0045893\_positive\_regulation\_of\_transcription\_\_DNA-dependent | KLF12 | 306 | 8 | 2.271550 | -1.651336 | 112 | 21.46 | 0.191607 |  |
| GO:0045893\_positive\_regulation\_of\_transcription\_\_DNA-dependent | JUN | 306 | 8 | 2.271550 | -1.651336 | 112 | 21.46 | 0.191607 |  |
| GO:0045893\_positive\_regulation\_of\_transcription\_\_DNA-dependent | TBX5 | 306 | 8 | 2.271550 | -1.651336 | 112 | 21.46 | 0.191607 |  |
| GO:0045893\_positive\_regulation\_of\_transcription\_\_DNA-dependent | FOXC1 | 306 | 8 | 2.271550 | -1.651336 | 112 | 21.46 | 0.191607 |  |
| GO:0045893\_positive\_regulation\_of\_transcription\_\_DNA-dependent | RORA | 306 | 8 | 2.271550 | -1.651336 | 112 | 21.46 | 0.191607 |  |
| GO:0045893\_positive\_regulation\_of\_transcription\_\_DNA-dependent | CTNNB1 | 306 | 8 | 2.271550 | -1.651336 | 112 | 21.46 | 0.191607 |  |
| GO:0045893\_positive\_regulation\_of\_transcription\_\_DNA-dependent | CITED2 | 306 | 8 | 2.271550 | -1.651336 | 112 | 21.46 | 0.191607 |  |
| GO:0051254\_positive\_regulation\_of\_RNA\_metabolic\_process | BMP2 | 306 | 8 | 2.271550 | -1.651336 | 112 | 21.46 | 0.191607 |  |
| GO:0051254\_positive\_regulation\_of\_RNA\_metabolic\_process | KLF12 | 306 | 8 | 2.271550 | -1.651336 | 112 | 21.46 | 0.191607 |  |
| GO:0051254\_positive\_regulation\_of\_RNA\_metabolic\_process | JUN | 306 | 8 | 2.271550 | -1.651336 | 112 | 21.46 | 0.191607 |  |
| GO:0051254\_positive\_regulation\_of\_RNA\_metabolic\_process | TBX5 | 306 | 8 | 2.271550 | -1.651336 | 112 | 21.46 | 0.191607 |  |
| GO:0051254\_positive\_regulation\_of\_RNA\_metabolic\_process | FOXC1 | 306 | 8 | 2.271550 | -1.651336 | 112 | 21.46 | 0.191607 |  |
| GO:0051254\_positive\_regulation\_of\_RNA\_metabolic\_process | RORA | 306 | 8 | 2.271550 | -1.651336 | 112 | 21.46 | 0.191607 |  |
| GO:0051254\_positive\_regulation\_of\_RNA\_metabolic\_process | CTNNB1 | 306 | 8 | 2.271550 | -1.651336 | 112 | 21.46 | 0.191607 |  |
| GO:0051254\_positive\_regulation\_of\_RNA\_metabolic\_process | CITED2 | 306 | 8 | 2.271550 | -1.651336 | 112 | 21.46 | 0.191607 |  |
| GO:0001743\_optic\_placode\_formation | PROX1 | 2 | 1 |  |  |  |  |  |  |
| GO:0002576\_platelet\_degranulation | PLEK | 2 | 1 |  |  |  |  |  |  |
| GO:0010872\_regulation\_of\_cholesterol\_esterification | APOE | 2 | 1 |  |  |  |  |  |  |
| GO:0014829\_vascular\_smooth\_muscle\_contraction | ACTA2 | 2 | 1 |  |  |  |  |  |  |
| GO:0014866\_skeletal\_myofibril\_assembly | PROX1 | 2 | 1 |  |  |  |  |  |  |
| GO:0030240\_muscle\_thin\_filament\_assembly | PROX1 | 2 | 1 |  |  |  |  |  |  |
| GO:0031952\_regulation\_of\_protein\_amino\_acid\_autophosphorylation | JUN | 2 | 1 |  |  |  |  |  |  |
| GO:0031953\_negative\_regulation\_of\_protein\_amino\_acid\_autophosphorylation | JUN | 2 | 1 |  |  |  |  |  |  |
| GO:0033622\_integrin\_activation | PLEK | 2 | 1 |  |  |  |  |  |  |
| GO:0033623\_regulation\_of\_integrin\_activation | PLEK | 2 | 1 |  |  |  |  |  |  |
| GO:0033625\_positive\_regulation\_of\_integrin\_activation | PLEK | 2 | 1 |  |  |  |  |  |  |
| GO:0034370\_triglyceride-rich\_lipoprotein\_particle\_remodeling | APOE | 2 | 1 |  |  |  |  |  |  |
| GO:0034374\_low-density\_lipoprotein\_particle\_remodeling | APOE | 2 | 1 |  |  |  |  |  |  |
| GO:0034433\_steroid\_esterification | APOE | 2 | 1 |  |  |  |  |  |  |
| GO:0034434\_sterol\_esterification | APOE | 2 | 1 |  |  |  |  |  |  |
| GO:0034435\_cholesterol\_esterification | APOE | 2 | 1 |  |  |  |  |  |  |
| GO:0035117\_embryonic\_arm\_morphogenesis | CTNNB1 | 2 | 1 |  |  |  |  |  |  |
| GO:0035140\_arm\_morphogenesis | CTNNB1 | 2 | 1 |  |  |  |  |  |  |
| GO:0042312\_regulation\_of\_vasodilation | PTPRM | 2 | 1 |  |  |  |  |  |  |
| GO:0045608\_negative\_regulation\_of\_auditory\_receptor\_cell\_differentiation | DLL1 | 2 | 1 |  |  |  |  |  |  |
| GO:0045632\_negative\_regulation\_of\_mechanoreceptor\_differentiation | DLL1 | 2 | 1 |  |  |  |  |  |  |
| GO:0046619\_optic\_placode\_formation\_involved\_in\_camera-type\_eye | PROX1 | 2 | 1 |  |  |  |  |  |  |
| GO:0048134\_germ-line\_cyst\_formation | RBP4 | 2 | 1 |  |  |  |  |  |  |
| GO:0048136\_male\_germ-line\_cyst\_formation | RBP4 | 2 | 1 |  |  |  |  |  |  |
| GO:0048711\_positive\_regulation\_of\_astrocyte\_differentiation | BMP2 | 2 | 1 |  |  |  |  |  |  |
| GO:0048807\_female\_genitalia\_morphogenesis | RBP4 | 2 | 1 |  |  |  |  |  |  |
| GO:0048850\_hypophysis\_morphogenesis | WNT5A | 2 | 1 |  |  |  |  |  |  |
| GO:0060044\_negative\_regulation\_of\_cardiac\_muscle\_cell\_proliferation | RBP4 | 2 | 1 |  |  |  |  |  |  |
| GO:0060214\_endocardium\_formation | PROX1 | 2 | 1 |  |  |  |  |  |  |
| GO:0060599\_lateral\_sprouting\_involved\_in\_mammary\_gland\_duct\_morphogenesis | WNT5A | 2 | 1 |  |  |  |  |  |  |
| GO:0060769\_positive\_regulation\_of\_epithelial\_cell\_proliferation\_involved\_in\_prostate\_gland\_development | CTNNB1 | 2 | 1 |  |  |  |  |  |  |
| GO:0007389\_pattern\_specification\_process | BTG2 | 250 | 7 | 2.432830 | -1.634697 | 113 | 21.82 | 0.193097 |  |
| GO:0007389\_pattern\_specification\_process | TBX5 | 250 | 7 | 2.432830 | -1.634697 | 113 | 21.82 | 0.193097 |  |
| GO:0007389\_pattern\_specification\_process | FOXC1 | 250 | 7 | 2.432830 | -1.634697 | 113 | 21.82 | 0.193097 |  |
| GO:0007389\_pattern\_specification\_process | ZEB2 | 250 | 7 | 2.432830 | -1.634697 | 113 | 21.82 | 0.193097 |  |
| GO:0007389\_pattern\_specification\_process | DLL1 | 250 | 7 | 2.432830 | -1.634697 | 113 | 21.82 | 0.193097 |  |
| GO:0007389\_pattern\_specification\_process | CITED2 | 250 | 7 | 2.432830 | -1.634697 | 113 | 21.82 | 0.193097 |  |
| GO:0007389\_pattern\_specification\_process | CTNNB1 | 250 | 7 | 2.432830 | -1.634697 | 113 | 21.82 | 0.193097 |  |
| GO:0016310\_phosphorylation | WNT5A | 309 | 8 | 2.249496 | -1.628591 | 114 | 21.94 | 0.192456 |  |
| GO:0016310\_phosphorylation | VAV3 | 309 | 8 | 2.249496 | -1.628591 | 114 | 21.94 | 0.192456 |  |
| GO:0016310\_phosphorylation | RB1CC1 | 309 | 8 | 2.249496 | -1.628591 | 114 | 21.94 | 0.192456 |  |
| GO:0016310\_phosphorylation | JUN | 309 | 8 | 2.249496 | -1.628591 | 114 | 21.94 | 0.192456 |  |
| GO:0016310\_phosphorylation | LRP8 | 309 | 8 | 2.249496 | -1.628591 | 114 | 21.94 | 0.192456 |  |
| GO:0016310\_phosphorylation | ZEB2 | 309 | 8 | 2.249496 | -1.628591 | 114 | 21.94 | 0.192456 |  |
| GO:0016310\_phosphorylation | PRKG1 | 309 | 8 | 2.249496 | -1.628591 | 114 | 21.94 | 0.192456 |  |
| GO:0016310\_phosphorylation | TGFB2 | 309 | 8 | 2.249496 | -1.628591 | 114 | 21.94 | 0.192456 |  |
| GO:0010604\_positive\_regulation\_of\_macromolecule\_metabolic\_process | BMP2 | 433 | 10 | 2.006623 | -1.624662 | 115 | 22.21 | 0.193130 |  |
| GO:0010604\_positive\_regulation\_of\_macromolecule\_metabolic\_process | PLEK | 433 | 10 | 2.006623 | -1.624662 | 115 | 22.21 | 0.193130 |  |
| GO:0010604\_positive\_regulation\_of\_macromolecule\_metabolic\_process | KLF12 | 433 | 10 | 2.006623 | -1.624662 | 115 | 22.21 | 0.193130 |  |
| GO:0010604\_positive\_regulation\_of\_macromolecule\_metabolic\_process | RB1CC1 | 433 | 10 | 2.006623 | -1.624662 | 115 | 22.21 | 0.193130 |  |
| GO:0010604\_positive\_regulation\_of\_macromolecule\_metabolic\_process | JUN | 433 | 10 | 2.006623 | -1.624662 | 115 | 22.21 | 0.193130 |  |
| GO:0010604\_positive\_regulation\_of\_macromolecule\_metabolic\_process | TBX5 | 433 | 10 | 2.006623 | -1.624662 | 115 | 22.21 | 0.193130 |  |
| GO:0010604\_positive\_regulation\_of\_macromolecule\_metabolic\_process | FOXC1 | 433 | 10 | 2.006623 | -1.624662 | 115 | 22.21 | 0.193130 |  |
| GO:0010604\_positive\_regulation\_of\_macromolecule\_metabolic\_process | RORA | 433 | 10 | 2.006623 | -1.624662 | 115 | 22.21 | 0.193130 |  |
| GO:0010604\_positive\_regulation\_of\_macromolecule\_metabolic\_process | CTNNB1 | 433 | 10 | 2.006623 | -1.624662 | 115 | 22.21 | 0.193130 |  |
| GO:0010604\_positive\_regulation\_of\_macromolecule\_metabolic\_process | CITED2 | 433 | 10 | 2.006623 | -1.624662 | 115 | 22.21 | 0.193130 |  |
| GO:0001709\_cell\_fate\_determination | PROX1 | 21 | 2 | 8.274933 | -1.624612 | 120 | 23.14 | 0.192833 |  |
| GO:0001709\_cell\_fate\_determination | CTNNB1 | 21 | 2 | 8.274933 | -1.624612 | 120 | 23.14 | 0.192833 |  |
| GO:0001755\_neural\_crest\_cell\_migration | NRP2 | 21 | 2 | 8.274933 | -1.624612 | 120 | 23.14 | 0.192833 |  |
| GO:0001755\_neural\_crest\_cell\_migration | ZEB2 | 21 | 2 | 8.274933 | -1.624612 | 120 | 23.14 | 0.192833 |  |
| GO:0009583\_detection\_of\_light\_stimulus | RBP4 | 21 | 2 | 8.274933 | -1.624612 | 120 | 23.14 | 0.192833 |  |
| GO:0009583\_detection\_of\_light\_stimulus | RGS9BP | 21 | 2 | 8.274933 | -1.624612 | 120 | 23.14 | 0.192833 |  |
| GO:0010552\_positive\_regulation\_of\_specific\_transcription\_from\_RNA\_polymerase\_II\_promoter | BMP2 | 21 | 2 | 8.274933 | -1.624612 | 120 | 23.14 | 0.192833 |  |
| GO:0010552\_positive\_regulation\_of\_specific\_transcription\_from\_RNA\_polymerase\_II\_promoter | CITED2 | 21 | 2 | 8.274933 | -1.624612 | 120 | 23.14 | 0.192833 |  |
| GO:0035136\_forelimb\_morphogenesis | TBX5 | 21 | 2 | 8.274933 | -1.624612 | 120 | 23.14 | 0.192833 |  |
| GO:0035136\_forelimb\_morphogenesis | CTNNB1 | 21 | 2 | 8.274933 | -1.624612 | 120 | 23.14 | 0.192833 |  |
| GO:0007165\_signal\_transduction | RHOJ | 915 | 17 | 1.614290 | -1.624086 | 121 | 23.15 | 0.191322 |  |
| GO:0007165\_signal\_transduction | WNT5A | 915 | 17 | 1.614290 | -1.624086 | 121 | 23.15 | 0.191322 |  |
| GO:0007165\_signal\_transduction | SCHIP1 | 915 | 17 | 1.614290 | -1.624086 | 121 | 23.15 | 0.191322 |  |
| GO:0007165\_signal\_transduction | BMP2 | 915 | 17 | 1.614290 | -1.624086 | 121 | 23.15 | 0.191322 |  |
| GO:0007165\_signal\_transduction | VAV3 | 915 | 17 | 1.614290 | -1.624086 | 121 | 23.15 | 0.191322 |  |
| GO:0007165\_signal\_transduction | PLEK | 915 | 17 | 1.614290 | -1.624086 | 121 | 23.15 | 0.191322 |  |
| GO:0007165\_signal\_transduction | PRRX1 | 915 | 17 | 1.614290 | -1.624086 | 121 | 23.15 | 0.191322 |  |
| GO:0007165\_signal\_transduction | ZEB2 | 915 | 17 | 1.614290 | -1.624086 | 121 | 23.15 | 0.191322 |  |
| GO:0007165\_signal\_transduction | PRKG1 | 915 | 17 | 1.614290 | -1.624086 | 121 | 23.15 | 0.191322 |  |
| GO:0007165\_signal\_transduction | CTNNB1 | 915 | 17 | 1.614290 | -1.624086 | 121 | 23.15 | 0.191322 |  |
| GO:0007165\_signal\_transduction | CITED2 | 915 | 17 | 1.614290 | -1.624086 | 121 | 23.15 | 0.191322 |  |
| GO:0007165\_signal\_transduction | TGFB2 | 915 | 17 | 1.614290 | -1.624086 | 121 | 23.15 | 0.191322 |  |
| GO:0007165\_signal\_transduction | FZD10 | 915 | 17 | 1.614290 | -1.624086 | 121 | 23.15 | 0.191322 |  |
| GO:0007165\_signal\_transduction | RB1CC1 | 915 | 17 | 1.614290 | -1.624086 | 121 | 23.15 | 0.191322 |  |
| GO:0007165\_signal\_transduction | RSPO3 | 915 | 17 | 1.614290 | -1.624086 | 121 | 23.15 | 0.191322 |  |
| GO:0007165\_signal\_transduction | FOXC1 | 915 | 17 | 1.614290 | -1.624086 | 121 | 23.15 | 0.191322 |  |
| GO:0007165\_signal\_transduction | CDC42EP4 | 915 | 17 | 1.614290 | -1.624086 | 121 | 23.15 | 0.191322 |  |
| GO:0048736\_appendage\_development | WNT5A | 96 | 4 | 3.620283 | -1.622786 | 123 | 23.25 | 0.189024 |  |
| GO:0048736\_appendage\_development | TBX5 | 96 | 4 | 3.620283 | -1.622786 | 123 | 23.25 | 0.189024 |  |
| GO:0048736\_appendage\_development | PRRX1 | 96 | 4 | 3.620283 | -1.622786 | 123 | 23.25 | 0.189024 |  |
| GO:0048736\_appendage\_development | CTNNB1 | 96 | 4 | 3.620283 | -1.622786 | 123 | 23.25 | 0.189024 |  |
| GO:0060173\_limb\_development | WNT5A | 96 | 4 | 3.620283 | -1.622786 | 123 | 23.25 | 0.189024 |  |
| GO:0060173\_limb\_development | TBX5 | 96 | 4 | 3.620283 | -1.622786 | 123 | 23.25 | 0.189024 |  |
| GO:0060173\_limb\_development | PRRX1 | 96 | 4 | 3.620283 | -1.622786 | 123 | 23.25 | 0.189024 |  |
| GO:0060173\_limb\_development | CTNNB1 | 96 | 4 | 3.620283 | -1.622786 | 123 | 23.25 | 0.189024 |  |
| GO:0033554\_cellular\_response\_to\_stress | WNT5A | 196 | 6 | 2.659800 | -1.621412 | 124 | 23.27 | 0.187661 |  |
| GO:0033554\_cellular\_response\_to\_stress | XRCC4 | 196 | 6 | 2.659800 | -1.621412 | 124 | 23.27 | 0.187661 |  |
| GO:0033554\_cellular\_response\_to\_stress | APOE | 196 | 6 | 2.659800 | -1.621412 | 124 | 23.27 | 0.187661 |  |
| GO:0033554\_cellular\_response\_to\_stress | RB1CC1 | 196 | 6 | 2.659800 | -1.621412 | 124 | 23.27 | 0.187661 |  |
| GO:0033554\_cellular\_response\_to\_stress | ZEB2 | 196 | 6 | 2.659800 | -1.621412 | 124 | 23.27 | 0.187661 |  |
| GO:0033554\_cellular\_response\_to\_stress | TCFEC | 196 | 6 | 2.659800 | -1.621412 | 124 | 23.27 | 0.187661 |  |
| GO:0050793\_regulation\_of\_developmental\_process | RHOJ | 703 | 14 | 1.730320 | -1.612408 | 125 | 23.36 | 0.186880 |  |
| GO:0050793\_regulation\_of\_developmental\_process | WNT5A | 703 | 14 | 1.730320 | -1.612408 | 125 | 23.36 | 0.186880 |  |
| GO:0050793\_regulation\_of\_developmental\_process | RBP4 | 703 | 14 | 1.730320 | -1.612408 | 125 | 23.36 | 0.186880 |  |
| GO:0050793\_regulation\_of\_developmental\_process | XRCC4 | 703 | 14 | 1.730320 | -1.612408 | 125 | 23.36 | 0.186880 |  |
| GO:0050793\_regulation\_of\_developmental\_process | BMP2 | 703 | 14 | 1.730320 | -1.612408 | 125 | 23.36 | 0.186880 |  |
| GO:0050793\_regulation\_of\_developmental\_process | ZC3HC1 | 703 | 14 | 1.730320 | -1.612408 | 125 | 23.36 | 0.186880 |  |
| GO:0050793\_regulation\_of\_developmental\_process | SGK3 | 703 | 14 | 1.730320 | -1.612408 | 125 | 23.36 | 0.186880 |  |
| GO:0050793\_regulation\_of\_developmental\_process | DLL1 | 703 | 14 | 1.730320 | -1.612408 | 125 | 23.36 | 0.186880 |  |
| GO:0050793\_regulation\_of\_developmental\_process | PROX1 | 703 | 14 | 1.730320 | -1.612408 | 125 | 23.36 | 0.186880 |  |
| GO:0050793\_regulation\_of\_developmental\_process | TGFB2 | 703 | 14 | 1.730320 | -1.612408 | 125 | 23.36 | 0.186880 |  |
| GO:0050793\_regulation\_of\_developmental\_process | CITED2 | 703 | 14 | 1.730320 | -1.612408 | 125 | 23.36 | 0.186880 |  |
| GO:0050793\_regulation\_of\_developmental\_process | CTNNB1 | 703 | 14 | 1.730320 | -1.612408 | 125 | 23.36 | 0.186880 |  |
| GO:0050793\_regulation\_of\_developmental\_process | RB1CC1 | 703 | 14 | 1.730320 | -1.612408 | 125 | 23.36 | 0.186880 |  |
| GO:0050793\_regulation\_of\_developmental\_process | FOXC1 | 703 | 14 | 1.730320 | -1.612408 | 125 | 23.36 | 0.186880 |  |
| GO:0002009\_morphogenesis\_of\_an\_epithelium | WNT5A | 198 | 6 | 2.632933 | -1.602197 | 127 | 23.75 | 0.187008 |  |
| GO:0002009\_morphogenesis\_of\_an\_epithelium | BMP2 | 198 | 6 | 2.632933 | -1.602197 | 127 | 23.75 | 0.187008 |  |
| GO:0002009\_morphogenesis\_of\_an\_epithelium | TBX5 | 198 | 6 | 2.632933 | -1.602197 | 127 | 23.75 | 0.187008 |  |
| GO:0002009\_morphogenesis\_of\_an\_epithelium | JUN | 198 | 6 | 2.632933 | -1.602197 | 127 | 23.75 | 0.187008 |  |
| GO:0002009\_morphogenesis\_of\_an\_epithelium | ZEB2 | 198 | 6 | 2.632933 | -1.602197 | 127 | 23.75 | 0.187008 |  |
| GO:0002009\_morphogenesis\_of\_an\_epithelium | CTNNB1 | 198 | 6 | 2.632933 | -1.602197 | 127 | 23.75 | 0.187008 |  |
| GO:0060429\_epithelium\_development | WNT5A | 198 | 6 | 2.632933 | -1.602197 | 127 | 23.75 | 0.187008 |  |
| GO:0060429\_epithelium\_development | BMP2 | 198 | 6 | 2.632933 | -1.602197 | 127 | 23.75 | 0.187008 |  |
| GO:0060429\_epithelium\_development | TBX5 | 198 | 6 | 2.632933 | -1.602197 | 127 | 23.75 | 0.187008 |  |
| GO:0060429\_epithelium\_development | JUN | 198 | 6 | 2.632933 | -1.602197 | 127 | 23.75 | 0.187008 |  |
| GO:0060429\_epithelium\_development | ZEB2 | 198 | 6 | 2.632933 | -1.602197 | 127 | 23.75 | 0.187008 |  |
| GO:0060429\_epithelium\_development | CTNNB1 | 198 | 6 | 2.632933 | -1.602197 | 127 | 23.75 | 0.187008 |  |
| GO:0030900\_forebrain\_development | WNT5A | 146 | 5 | 2.975575 | -1.596877 | 128 | 23.88 | 0.186562 |  |
| GO:0030900\_forebrain\_development | LRP8 | 146 | 5 | 2.975575 | -1.596877 | 128 | 23.88 | 0.186562 |  |
| GO:0030900\_forebrain\_development | ZEB2 | 146 | 5 | 2.975575 | -1.596877 | 128 | 23.88 | 0.186562 |  |
| GO:0030900\_forebrain\_development | PRKG1 | 146 | 5 | 2.975575 | -1.596877 | 128 | 23.88 | 0.186562 |  |
| GO:0030900\_forebrain\_development | CTNNB1 | 146 | 5 | 2.975575 | -1.596877 | 128 | 23.88 | 0.186562 |  |
| GO:0009967\_positive\_regulation\_of\_signal\_transduction | PRRX1 | 98 | 4 | 3.546400 | -1.593678 | 130 | 23.97 | 0.184385 |  |
| GO:0009967\_positive\_regulation\_of\_signal\_transduction | ZEB2 | 98 | 4 | 3.546400 | -1.593678 | 130 | 23.97 | 0.184385 |  |
| GO:0009967\_positive\_regulation\_of\_signal\_transduction | CITED2 | 98 | 4 | 3.546400 | -1.593678 | 130 | 23.97 | 0.184385 |  |
| GO:0009967\_positive\_regulation\_of\_signal\_transduction | CTNNB1 | 98 | 4 | 3.546400 | -1.593678 | 130 | 23.97 | 0.184385 |  |
| GO:0060541\_respiratory\_system\_development | WNT5A | 98 | 4 | 3.546400 | -1.593678 | 130 | 23.97 | 0.184385 |  |
| GO:0060541\_respiratory\_system\_development | RBP4 | 98 | 4 | 3.546400 | -1.593678 | 130 | 23.97 | 0.184385 |  |
| GO:0060541\_respiratory\_system\_development | TBX5 | 98 | 4 | 3.546400 | -1.593678 | 130 | 23.97 | 0.184385 |  |
| GO:0060541\_respiratory\_system\_development | CTNNB1 | 98 | 4 | 3.546400 | -1.593678 | 130 | 23.97 | 0.184385 |  |
| GO:0006029\_proteoglycan\_metabolic\_process | A930038C07RIK | 22 | 2 | 7.898799 | -1.586391 | 134 | 25.03 | 0.186791 |  |
| GO:0006029\_proteoglycan\_metabolic\_process | DCN | 22 | 2 | 7.898799 | -1.586391 | 134 | 25.03 | 0.186791 |  |
| GO:0015918\_sterol\_transport | APOE | 22 | 2 | 7.898799 | -1.586391 | 134 | 25.03 | 0.186791 |  |
| GO:0015918\_sterol\_transport | CFTR | 22 | 2 | 7.898799 | -1.586391 | 134 | 25.03 | 0.186791 |  |
| GO:0021766\_hippocampus\_development | LRP8 | 22 | 2 | 7.898799 | -1.586391 | 134 | 25.03 | 0.186791 |  |
| GO:0021766\_hippocampus\_development | ZEB2 | 22 | 2 | 7.898799 | -1.586391 | 134 | 25.03 | 0.186791 |  |
| GO:0030301\_cholesterol\_transport | APOE | 22 | 2 | 7.898799 | -1.586391 | 134 | 25.03 | 0.186791 |  |
| GO:0030301\_cholesterol\_transport | CFTR | 22 | 2 | 7.898799 | -1.586391 | 134 | 25.03 | 0.186791 |  |
| GO:0080090\_regulation\_of\_primary\_metabolic\_process | EGR1 | 926 | 17 | 1.595114 | -1.576754 | 135 | 25.62 | 0.189778 |  |
| GO:0080090\_regulation\_of\_primary\_metabolic\_process | BMP2 | 926 | 17 | 1.595114 | -1.576754 | 135 | 25.62 | 0.189778 |  |
| GO:0080090\_regulation\_of\_primary\_metabolic\_process | VAV3 | 926 | 17 | 1.595114 | -1.576754 | 135 | 25.62 | 0.189778 |  |
| GO:0080090\_regulation\_of\_primary\_metabolic\_process | PLEK | 926 | 17 | 1.595114 | -1.576754 | 135 | 25.62 | 0.189778 |  |
| GO:0080090\_regulation\_of\_primary\_metabolic\_process | KLF12 | 926 | 17 | 1.595114 | -1.576754 | 135 | 25.62 | 0.189778 |  |
| GO:0080090\_regulation\_of\_primary\_metabolic\_process | TBX5 | 926 | 17 | 1.595114 | -1.576754 | 135 | 25.62 | 0.189778 |  |
| GO:0080090\_regulation\_of\_primary\_metabolic\_process | ZEB2 | 926 | 17 | 1.595114 | -1.576754 | 135 | 25.62 | 0.189778 |  |
| GO:0080090\_regulation\_of\_primary\_metabolic\_process | RORA | 926 | 17 | 1.595114 | -1.576754 | 135 | 25.62 | 0.189778 |  |
| GO:0080090\_regulation\_of\_primary\_metabolic\_process | FOXP4 | 926 | 17 | 1.595114 | -1.576754 | 135 | 25.62 | 0.189778 |  |
| GO:0080090\_regulation\_of\_primary\_metabolic\_process | CITED2 | 926 | 17 | 1.595114 | -1.576754 | 135 | 25.62 | 0.189778 |  |
| GO:0080090\_regulation\_of\_primary\_metabolic\_process | CTNNB1 | 926 | 17 | 1.595114 | -1.576754 | 135 | 25.62 | 0.189778 |  |
| GO:0080090\_regulation\_of\_primary\_metabolic\_process | APOE | 926 | 17 | 1.595114 | -1.576754 | 135 | 25.62 | 0.189778 |  |
| GO:0080090\_regulation\_of\_primary\_metabolic\_process | RB1CC1 | 926 | 17 | 1.595114 | -1.576754 | 135 | 25.62 | 0.189778 |  |
| GO:0080090\_regulation\_of\_primary\_metabolic\_process | JUN | 926 | 17 | 1.595114 | -1.576754 | 135 | 25.62 | 0.189778 |  |
| GO:0080090\_regulation\_of\_primary\_metabolic\_process | FOXC1 | 926 | 17 | 1.595114 | -1.576754 | 135 | 25.62 | 0.189778 |  |
| GO:0080090\_regulation\_of\_primary\_metabolic\_process | HBA-X | 926 | 17 | 1.595114 | -1.576754 | 135 | 25.62 | 0.189778 |  |
| GO:0080090\_regulation\_of\_primary\_metabolic\_process | C1D | 926 | 17 | 1.595114 | -1.576754 | 135 | 25.62 | 0.189778 |  |
| GO:0048870\_cell\_motility | NRP2 | 257 | 7 | 2.366566 | -1.576519 | 136 | 25.63 | 0.188456 |  |
| GO:0048870\_cell\_motility | SCHIP1 | 257 | 7 | 2.366566 | -1.576519 | 136 | 25.63 | 0.188456 |  |
| GO:0048870\_cell\_motility | VAV3 | 257 | 7 | 2.366566 | -1.576519 | 136 | 25.63 | 0.188456 |  |
| GO:0048870\_cell\_motility | LRP8 | 257 | 7 | 2.366566 | -1.576519 | 136 | 25.63 | 0.188456 |  |
| GO:0048870\_cell\_motility | FOXC1 | 257 | 7 | 2.366566 | -1.576519 | 136 | 25.63 | 0.188456 |  |
| GO:0048870\_cell\_motility | ZEB2 | 257 | 7 | 2.366566 | -1.576519 | 136 | 25.63 | 0.188456 |  |
| GO:0048870\_cell\_motility | PRKG1 | 257 | 7 | 2.366566 | -1.576519 | 136 | 25.63 | 0.188456 |  |
| GO:0010926\_anatomical\_structure\_formation | WNT5A | 447 | 10 | 1.943776 | -1.539128 | 137 | 26.91 | 0.196423 |  |
| GO:0010926\_anatomical\_structure\_formation | RBP4 | 447 | 10 | 1.943776 | -1.539128 | 137 | 26.91 | 0.196423 |  |
| GO:0010926\_anatomical\_structure\_formation | VAV3 | 447 | 10 | 1.943776 | -1.539128 | 137 | 26.91 | 0.196423 |  |
| GO:0010926\_anatomical\_structure\_formation | LRP8 | 447 | 10 | 1.943776 | -1.539128 | 137 | 26.91 | 0.196423 |  |
| GO:0010926\_anatomical\_structure\_formation | ZEB2 | 447 | 10 | 1.943776 | -1.539128 | 137 | 26.91 | 0.196423 |  |
| GO:0010926\_anatomical\_structure\_formation | FOXC1 | 447 | 10 | 1.943776 | -1.539128 | 137 | 26.91 | 0.196423 |  |
| GO:0010926\_anatomical\_structure\_formation | RORA | 447 | 10 | 1.943776 | -1.539128 | 137 | 26.91 | 0.196423 |  |
| GO:0010926\_anatomical\_structure\_formation | PROX1 | 447 | 10 | 1.943776 | -1.539128 | 137 | 26.91 | 0.196423 |  |
| GO:0010926\_anatomical\_structure\_formation | CITED2 | 447 | 10 | 1.943776 | -1.539128 | 137 | 26.91 | 0.196423 |  |
| GO:0010926\_anatomical\_structure\_formation | CTNNB1 | 447 | 10 | 1.943776 | -1.539128 | 137 | 26.91 | 0.196423 |  |
| GO:0043687\_post-translational\_protein\_modification | WNT5A | 384 | 9 | 2.036409 | -1.533559 | 138 | 27.11 | 0.196449 |  |
| GO:0043687\_post-translational\_protein\_modification | A930038C07RIK | 384 | 9 | 2.036409 | -1.533559 | 138 | 27.11 | 0.196449 |  |
| GO:0043687\_post-translational\_protein\_modification | BTG2 | 384 | 9 | 2.036409 | -1.533559 | 138 | 27.11 | 0.196449 |  |
| GO:0043687\_post-translational\_protein\_modification | RB1CC1 | 384 | 9 | 2.036409 | -1.533559 | 138 | 27.11 | 0.196449 |  |
| GO:0043687\_post-translational\_protein\_modification | JUN | 384 | 9 | 2.036409 | -1.533559 | 138 | 27.11 | 0.196449 |  |
| GO:0043687\_post-translational\_protein\_modification | ZEB2 | 384 | 9 | 2.036409 | -1.533559 | 138 | 27.11 | 0.196449 |  |
| GO:0043687\_post-translational\_protein\_modification | DCN | 384 | 9 | 2.036409 | -1.533559 | 138 | 27.11 | 0.196449 |  |
| GO:0043687\_post-translational\_protein\_modification | PRKG1 | 384 | 9 | 2.036409 | -1.533559 | 138 | 27.11 | 0.196449 |  |
| GO:0043687\_post-translational\_protein\_modification | TGFB2 | 384 | 9 | 2.036409 | -1.533559 | 138 | 27.11 | 0.196449 |  |
| GO:0035270\_endocrine\_system\_development | INSM1 | 59 | 3 | 4.417972 | -1.526345 | 140 | 27.31 | 0.195071 |  |
| GO:0035270\_endocrine\_system\_development | WNT5A | 59 | 3 | 4.417972 | -1.526345 | 140 | 27.31 | 0.195071 |  |
| GO:0035270\_endocrine\_system\_development | CITED2 | 59 | 3 | 4.417972 | -1.526345 | 140 | 27.31 | 0.195071 |  |
| GO:0048469\_cell\_maturation | SEPT4 | 59 | 3 | 4.417972 | -1.526345 | 140 | 27.31 | 0.195071 |  |
| GO:0048469\_cell\_maturation | HBA-X | 59 | 3 | 4.417972 | -1.526345 | 140 | 27.31 | 0.195071 |  |
| GO:0048469\_cell\_maturation | CTNNB1 | 59 | 3 | 4.417972 | -1.526345 | 140 | 27.31 | 0.195071 |  |
| GO:0003013\_circulatory\_system\_process | PTPRM | 103 | 4 | 3.374244 | -1.524090 | 142 | 27.44 | 0.193239 |  |
| GO:0003013\_circulatory\_system\_process | APOE | 103 | 4 | 3.374244 | -1.524090 | 142 | 27.44 | 0.193239 |  |
| GO:0003013\_circulatory\_system\_process | ACTA2 | 103 | 4 | 3.374244 | -1.524090 | 142 | 27.44 | 0.193239 |  |
| GO:0003013\_circulatory\_system\_process | FOXC1 | 103 | 4 | 3.374244 | -1.524090 | 142 | 27.44 | 0.193239 |  |
| GO:0008015\_blood\_circulation | PTPRM | 103 | 4 | 3.374244 | -1.524090 | 142 | 27.44 | 0.193239 |  |
| GO:0008015\_blood\_circulation | APOE | 103 | 4 | 3.374244 | -1.524090 | 142 | 27.44 | 0.193239 |  |
| GO:0008015\_blood\_circulation | ACTA2 | 103 | 4 | 3.374244 | -1.524090 | 142 | 27.44 | 0.193239 |  |
| GO:0008015\_blood\_circulation | FOXC1 | 103 | 4 | 3.374244 | -1.524090 | 142 | 27.44 | 0.193239 |  |
| GO:0065007\_biological\_regulation | RHOJ | 2593 | 37 | 1.239804 | -1.518326 | 143 | 27.65 | 0.193357 |  |
| GO:0065007\_biological\_regulation | WNT5A | 2593 | 37 | 1.239804 | -1.518326 | 143 | 27.65 | 0.193357 |  |
| GO:0065007\_biological\_regulation | SCHIP1 | 2593 | 37 | 1.239804 | -1.518326 | 143 | 27.65 | 0.193357 |  |
| GO:0065007\_biological\_regulation | RBP4 | 2593 | 37 | 1.239804 | -1.518326 | 143 | 27.65 | 0.193357 |  |
| GO:0065007\_biological\_regulation | XRCC4 | 2593 | 37 | 1.239804 | -1.518326 | 143 | 27.65 | 0.193357 |  |
| GO:0065007\_biological\_regulation | PRRX1 | 2593 | 37 | 1.239804 | -1.518326 | 143 | 27.65 | 0.193357 |  |
| GO:0065007\_biological\_regulation | ZEB2 | 2593 | 37 | 1.239804 | -1.518326 | 143 | 27.65 | 0.193357 |  |
| GO:0065007\_biological\_regulation | RORA | 2593 | 37 | 1.239804 | -1.518326 | 143 | 27.65 | 0.193357 |  |
| GO:0065007\_biological\_regulation | PRKG1 | 2593 | 37 | 1.239804 | -1.518326 | 143 | 27.65 | 0.193357 |  |
| GO:0065007\_biological\_regulation | CITED2 | 2593 | 37 | 1.239804 | -1.518326 | 143 | 27.65 | 0.193357 |  |
| GO:0065007\_biological\_regulation | CTNNB1 | 2593 | 37 | 1.239804 | -1.518326 | 143 | 27.65 | 0.193357 |  |
| GO:0065007\_biological\_regulation | TGFB2 | 2593 | 37 | 1.239804 | -1.518326 | 143 | 27.65 | 0.193357 |  |
| GO:0065007\_biological\_regulation | A930038C07RIK | 2593 | 37 | 1.239804 | -1.518326 | 143 | 27.65 | 0.193357 |  |
| GO:0065007\_biological\_regulation | APOE | 2593 | 37 | 1.239804 | -1.518326 | 143 | 27.65 | 0.193357 |  |
| GO:0065007\_biological\_regulation | RSPO3 | 2593 | 37 | 1.239804 | -1.518326 | 143 | 27.65 | 0.193357 |  |
| GO:0065007\_biological\_regulation | RB1CC1 | 2593 | 37 | 1.239804 | -1.518326 | 143 | 27.65 | 0.193357 |  |
| GO:0065007\_biological\_regulation | CDC42EP4 | 2593 | 37 | 1.239804 | -1.518326 | 143 | 27.65 | 0.193357 |  |
| GO:0065007\_biological\_regulation | HBA-X | 2593 | 37 | 1.239804 | -1.518326 | 143 | 27.65 | 0.193357 |  |
| GO:0065007\_biological\_regulation | EGR1 | 2593 | 37 | 1.239804 | -1.518326 | 143 | 27.65 | 0.193357 |  |
| GO:0065007\_biological\_regulation | BMP2 | 2593 | 37 | 1.239804 | -1.518326 | 143 | 27.65 | 0.193357 |  |
| GO:0065007\_biological\_regulation | ZC3HC1 | 2593 | 37 | 1.239804 | -1.518326 | 143 | 27.65 | 0.193357 |  |
| GO:0065007\_biological\_regulation | PTPRM | 2593 | 37 | 1.239804 | -1.518326 | 143 | 27.65 | 0.193357 |  |
| GO:0065007\_biological\_regulation | VAV3 | 2593 | 37 | 1.239804 | -1.518326 | 143 | 27.65 | 0.193357 |  |
| GO:0065007\_biological\_regulation | SGK3 | 2593 | 37 | 1.239804 | -1.518326 | 143 | 27.65 | 0.193357 |  |
| GO:0065007\_biological\_regulation | KLF12 | 2593 | 37 | 1.239804 | -1.518326 | 143 | 27.65 | 0.193357 |  |
| GO:0065007\_biological\_regulation | PLEK | 2593 | 37 | 1.239804 | -1.518326 | 143 | 27.65 | 0.193357 |  |
| GO:0065007\_biological\_regulation | ACTA2 | 2593 | 37 | 1.239804 | -1.518326 | 143 | 27.65 | 0.193357 |  |
| GO:0065007\_biological\_regulation | TBX5 | 2593 | 37 | 1.239804 | -1.518326 | 143 | 27.65 | 0.193357 |  |
| GO:0065007\_biological\_regulation | CCDC80 | 2593 | 37 | 1.239804 | -1.518326 | 143 | 27.65 | 0.193357 |  |
| GO:0065007\_biological\_regulation | DLL1 | 2593 | 37 | 1.239804 | -1.518326 | 143 | 27.65 | 0.193357 |  |
| GO:0065007\_biological\_regulation | FOXP4 | 2593 | 37 | 1.239804 | -1.518326 | 143 | 27.65 | 0.193357 |  |
| GO:0065007\_biological\_regulation | PROX1 | 2593 | 37 | 1.239804 | -1.518326 | 143 | 27.65 | 0.193357 |  |
| GO:0065007\_biological\_regulation | FZD10 | 2593 | 37 | 1.239804 | -1.518326 | 143 | 27.65 | 0.193357 |  |
| GO:0065007\_biological\_regulation | JUN | 2593 | 37 | 1.239804 | -1.518326 | 143 | 27.65 | 0.193357 |  |
| GO:0065007\_biological\_regulation | FOXC1 | 2593 | 37 | 1.239804 | -1.518326 | 143 | 27.65 | 0.193357 |  |
| GO:0065007\_biological\_regulation | LRP8 | 2593 | 37 | 1.239804 | -1.518326 | 143 | 27.65 | 0.193357 |  |
| GO:0065007\_biological\_regulation | C1D | 2593 | 37 | 1.239804 | -1.518326 | 143 | 27.65 | 0.193357 |  |
| GO:0031323\_regulation\_of\_cellular\_metabolic\_process | EGR1 | 1015 | 18 | 1.540850 | -1.516122 | 144 | 27.66 | 0.192083 |  |
| GO:0031323\_regulation\_of\_cellular\_metabolic\_process | BMP2 | 1015 | 18 | 1.540850 | -1.516122 | 144 | 27.66 | 0.192083 |  |
| GO:0031323\_regulation\_of\_cellular\_metabolic\_process | VAV3 | 1015 | 18 | 1.540850 | -1.516122 | 144 | 27.66 | 0.192083 |  |
| GO:0031323\_regulation\_of\_cellular\_metabolic\_process | PLEK | 1015 | 18 | 1.540850 | -1.516122 | 144 | 27.66 | 0.192083 |  |
| GO:0031323\_regulation\_of\_cellular\_metabolic\_process | KLF12 | 1015 | 18 | 1.540850 | -1.516122 | 144 | 27.66 | 0.192083 |  |
| GO:0031323\_regulation\_of\_cellular\_metabolic\_process | TBX5 | 1015 | 18 | 1.540850 | -1.516122 | 144 | 27.66 | 0.192083 |  |
| GO:0031323\_regulation\_of\_cellular\_metabolic\_process | ZEB2 | 1015 | 18 | 1.540850 | -1.516122 | 144 | 27.66 | 0.192083 |  |
| GO:0031323\_regulation\_of\_cellular\_metabolic\_process | RORA | 1015 | 18 | 1.540850 | -1.516122 | 144 | 27.66 | 0.192083 |  |
| GO:0031323\_regulation\_of\_cellular\_metabolic\_process | FOXP4 | 1015 | 18 | 1.540850 | -1.516122 | 144 | 27.66 | 0.192083 |  |
| GO:0031323\_regulation\_of\_cellular\_metabolic\_process | CITED2 | 1015 | 18 | 1.540850 | -1.516122 | 144 | 27.66 | 0.192083 |  |
| GO:0031323\_regulation\_of\_cellular\_metabolic\_process | CTNNB1 | 1015 | 18 | 1.540850 | -1.516122 | 144 | 27.66 | 0.192083 |  |
| GO:0031323\_regulation\_of\_cellular\_metabolic\_process | APOE | 1015 | 18 | 1.540850 | -1.516122 | 144 | 27.66 | 0.192083 |  |
| GO:0031323\_regulation\_of\_cellular\_metabolic\_process | JUN | 1015 | 18 | 1.540850 | -1.516122 | 144 | 27.66 | 0.192083 |  |
| GO:0031323\_regulation\_of\_cellular\_metabolic\_process | RB1CC1 | 1015 | 18 | 1.540850 | -1.516122 | 144 | 27.66 | 0.192083 |  |
| GO:0031323\_regulation\_of\_cellular\_metabolic\_process | LRP8 | 1015 | 18 | 1.540850 | -1.516122 | 144 | 27.66 | 0.192083 |  |
| GO:0031323\_regulation\_of\_cellular\_metabolic\_process | FOXC1 | 1015 | 18 | 1.540850 | -1.516122 | 144 | 27.66 | 0.192083 |  |
| GO:0031323\_regulation\_of\_cellular\_metabolic\_process | HBA-X | 1015 | 18 | 1.540850 | -1.516122 | 144 | 27.66 | 0.192083 |  |
| GO:0031323\_regulation\_of\_cellular\_metabolic\_process | C1D | 1015 | 18 | 1.540850 | -1.516122 | 144 | 27.66 | 0.192083 |  |
| GO:0007266\_Rho\_protein\_signal\_transduction | RHOJ | 24 | 2 | 7.240566 | -1.515429 | 146 | 28.63 | 0.196096 |  |
| GO:0007266\_Rho\_protein\_signal\_transduction | CDC42EP4 | 24 | 2 | 7.240566 | -1.515429 | 146 | 28.63 | 0.196096 |  |
| GO:0048546\_digestive\_tract\_morphogenesis | WNT5A | 24 | 2 | 7.240566 | -1.515429 | 146 | 28.63 | 0.196096 |  |
| GO:0048546\_digestive\_tract\_morphogenesis | FOXP4 | 24 | 2 | 7.240566 | -1.515429 | 146 | 28.63 | 0.196096 |  |
| GO:0031328\_positive\_regulation\_of\_cellular\_biosynthetic\_process | BMP2 | 387 | 9 | 2.020623 | -1.514401 | 147 | 28.63 | 0.194762 |  |
| GO:0031328\_positive\_regulation\_of\_cellular\_biosynthetic\_process | KLF12 | 387 | 9 | 2.020623 | -1.514401 | 147 | 28.63 | 0.194762 |  |
| GO:0031328\_positive\_regulation\_of\_cellular\_biosynthetic\_process | APOE | 387 | 9 | 2.020623 | -1.514401 | 147 | 28.63 | 0.194762 |  |
| GO:0031328\_positive\_regulation\_of\_cellular\_biosynthetic\_process | JUN | 387 | 9 | 2.020623 | -1.514401 | 147 | 28.63 | 0.194762 |  |
| GO:0031328\_positive\_regulation\_of\_cellular\_biosynthetic\_process | TBX5 | 387 | 9 | 2.020623 | -1.514401 | 147 | 28.63 | 0.194762 |  |
| GO:0031328\_positive\_regulation\_of\_cellular\_biosynthetic\_process | FOXC1 | 387 | 9 | 2.020623 | -1.514401 | 147 | 28.63 | 0.194762 |  |
| GO:0031328\_positive\_regulation\_of\_cellular\_biosynthetic\_process | RORA | 387 | 9 | 2.020623 | -1.514401 | 147 | 28.63 | 0.194762 |  |
| GO:0031328\_positive\_regulation\_of\_cellular\_biosynthetic\_process | CTNNB1 | 387 | 9 | 2.020623 | -1.514401 | 147 | 28.63 | 0.194762 |  |
| GO:0031328\_positive\_regulation\_of\_cellular\_biosynthetic\_process | CITED2 | 387 | 9 | 2.020623 | -1.514401 | 147 | 28.63 | 0.194762 |  |
| GO:0009891\_positive\_regulation\_of\_biosynthetic\_process | BMP2 | 388 | 9 | 2.015415 | -1.508069 | 148 | 28.75 | 0.194257 |  |
| GO:0009891\_positive\_regulation\_of\_biosynthetic\_process | KLF12 | 388 | 9 | 2.015415 | -1.508069 | 148 | 28.75 | 0.194257 |  |
| GO:0009891\_positive\_regulation\_of\_biosynthetic\_process | APOE | 388 | 9 | 2.015415 | -1.508069 | 148 | 28.75 | 0.194257 |  |
| GO:0009891\_positive\_regulation\_of\_biosynthetic\_process | JUN | 388 | 9 | 2.015415 | -1.508069 | 148 | 28.75 | 0.194257 |  |
| GO:0009891\_positive\_regulation\_of\_biosynthetic\_process | TBX5 | 388 | 9 | 2.015415 | -1.508069 | 148 | 28.75 | 0.194257 |  |
| GO:0009891\_positive\_regulation\_of\_biosynthetic\_process | FOXC1 | 388 | 9 | 2.015415 | -1.508069 | 148 | 28.75 | 0.194257 |  |
| GO:0009891\_positive\_regulation\_of\_biosynthetic\_process | RORA | 388 | 9 | 2.015415 | -1.508069 | 148 | 28.75 | 0.194257 |  |
| GO:0009891\_positive\_regulation\_of\_biosynthetic\_process | CTNNB1 | 388 | 9 | 2.015415 | -1.508069 | 148 | 28.75 | 0.194257 |  |
| GO:0009891\_positive\_regulation\_of\_biosynthetic\_process | CITED2 | 388 | 9 | 2.015415 | -1.508069 | 148 | 28.75 | 0.194257 |  |
| GO:0008285\_negative\_regulation\_of\_cell\_proliferation | WNT5A | 155 | 5 | 2.802800 | -1.498788 | 149 | 28.91 | 0.194027 |  |
| GO:0008285\_negative\_regulation\_of\_cell\_proliferation | RBP4 | 155 | 5 | 2.802800 | -1.498788 | 149 | 28.91 | 0.194027 |  |
| GO:0008285\_negative\_regulation\_of\_cell\_proliferation | BMP2 | 155 | 5 | 2.802800 | -1.498788 | 149 | 28.91 | 0.194027 |  |
| GO:0008285\_negative\_regulation\_of\_cell\_proliferation | JUN | 155 | 5 | 2.802800 | -1.498788 | 149 | 28.91 | 0.194027 |  |
| GO:0008285\_negative\_regulation\_of\_cell\_proliferation | PROX1 | 155 | 5 | 2.802800 | -1.498788 | 149 | 28.91 | 0.194027 |  |
| GO:0010811\_positive\_regulation\_of\_cell-substrate\_adhesion | A930038C07RIK | 25 | 2 | 6.950943 | -1.482381 | 151 | 29.96 | 0.198411 |  |
| GO:0010811\_positive\_regulation\_of\_cell-substrate\_adhesion | CCDC80 | 25 | 2 | 6.950943 | -1.482381 | 151 | 29.96 | 0.198411 |  |
| GO:0055123\_digestive\_system\_development | WNT5A | 25 | 2 | 6.950943 | -1.482381 | 151 | 29.96 | 0.198411 |  |
| GO:0055123\_digestive\_system\_development | FOXP4 | 25 | 2 | 6.950943 | -1.482381 | 151 | 29.96 | 0.198411 |  |
| GO:0006928\_cell\_motion | NRP2 | 330 | 8 | 2.106346 | -1.478400 | 153 | 30.09 | 0.196667 |  |
| GO:0006928\_cell\_motion | SCHIP1 | 330 | 8 | 2.106346 | -1.478400 | 153 | 30.09 | 0.196667 |  |
| GO:0006928\_cell\_motion | VAV3 | 330 | 8 | 2.106346 | -1.478400 | 153 | 30.09 | 0.196667 |  |
| GO:0006928\_cell\_motion | LRP8 | 330 | 8 | 2.106346 | -1.478400 | 153 | 30.09 | 0.196667 |  |
| GO:0006928\_cell\_motion | FOXC1 | 330 | 8 | 2.106346 | -1.478400 | 153 | 30.09 | 0.196667 |  |
| GO:0006928\_cell\_motion | ZEB2 | 330 | 8 | 2.106346 | -1.478400 | 153 | 30.09 | 0.196667 |  |
| GO:0006928\_cell\_motion | PRKG1 | 330 | 8 | 2.106346 | -1.478400 | 153 | 30.09 | 0.196667 |  |
| GO:0006928\_cell\_motion | TGFB2 | 330 | 8 | 2.106346 | -1.478400 | 153 | 30.09 | 0.196667 |  |
| GO:0051674\_localization\_of\_cell | NRP2 | 330 | 8 | 2.106346 | -1.478400 | 153 | 30.09 | 0.196667 |  |
| GO:0051674\_localization\_of\_cell | SCHIP1 | 330 | 8 | 2.106346 | -1.478400 | 153 | 30.09 | 0.196667 |  |
| GO:0051674\_localization\_of\_cell | VAV3 | 330 | 8 | 2.106346 | -1.478400 | 153 | 30.09 | 0.196667 |  |
| GO:0051674\_localization\_of\_cell | LRP8 | 330 | 8 | 2.106346 | -1.478400 | 153 | 30.09 | 0.196667 |  |
| GO:0051674\_localization\_of\_cell | FOXC1 | 330 | 8 | 2.106346 | -1.478400 | 153 | 30.09 | 0.196667 |  |
| GO:0051674\_localization\_of\_cell | ZEB2 | 330 | 8 | 2.106346 | -1.478400 | 153 | 30.09 | 0.196667 |  |
| GO:0051674\_localization\_of\_cell | PRKG1 | 330 | 8 | 2.106346 | -1.478400 | 153 | 30.09 | 0.196667 |  |
| GO:0051674\_localization\_of\_cell | TGFB2 | 330 | 8 | 2.106346 | -1.478400 | 153 | 30.09 | 0.196667 |  |
| GO:0001711\_endodermal\_cell\_fate\_commitment | CTNNB1 | 3 | 1 |  |  |  |  |  |  |
| GO:0001757\_somite\_specification | DLL1 | 3 | 1 |  |  |  |  |  |  |
| GO:0006266\_DNA\_ligation | XRCC4 | 3 | 1 |  |  |  |  |  |  |
| GO:0006904\_vesicle\_docking\_during\_exocytosis | PLEK | 3 | 1 |  |  |  |  |  |  |
| GO:0007403\_glial\_cell\_fate\_determination | CTNNB1 | 3 | 1 |  |  |  |  |  |  |
| GO:0015988\_energy\_coupled\_proton\_transport\_\_against\_electrochemical\_gradient | ATP6V1E1 | 3 | 1 |  |  |  |  |  |  |
| GO:0015991\_ATP\_hydrolysis\_coupled\_proton\_transport | ATP6V1E1 | 3 | 1 |  |  |  |  |  |  |
| GO:0021819\_layer\_formation\_in\_the\_cerebral\_cortex | LRP8 | 3 | 1 |  |  |  |  |  |  |
| GO:0022406\_membrane\_docking | PLEK | 3 | 1 |  |  |  |  |  |  |
| GO:0030540\_female\_genitalia\_development | RBP4 | 3 | 1 |  |  |  |  |  |  |
| GO:0030836\_positive\_regulation\_of\_actin\_filament\_depolymerization | PLEK | 3 | 1 |  |  |  |  |  |  |
| GO:0043243\_positive\_regulation\_of\_protein\_complex\_disassembly | PLEK | 3 | 1 |  |  |  |  |  |  |
| GO:0043249\_erythrocyte\_maturation | HBA-X | 3 | 1 |  |  |  |  |  |  |
| GO:0045607\_regulation\_of\_auditory\_receptor\_cell\_differentiation | DLL1 | 3 | 1 |  |  |  |  |  |  |
| GO:0045631\_regulation\_of\_mechanoreceptor\_differentiation | DLL1 | 3 | 1 |  |  |  |  |  |  |
| GO:0048278\_vesicle\_docking | PLEK | 3 | 1 |  |  |  |  |  |  |
| GO:0048845\_venous\_blood\_vessel\_morphogenesis | PROX1 | 3 | 1 |  |  |  |  |  |  |
| GO:0048852\_diencephalon\_morphogenesis | WNT5A | 3 | 1 |  |  |  |  |  |  |
| GO:0051153\_regulation\_of\_striated\_muscle\_cell\_differentiation | PROX1 | 3 | 1 |  |  |  |  |  |  |
| GO:0060686\_negative\_regulation\_of\_prostatic\_bud\_formation | WNT5A | 3 | 1 |  |  |  |  |  |  |
| GO:0060750\_epithelial\_cell\_proliferation\_involved\_in\_mammary\_gland\_duct\_elongation | WNT5A | 3 | 1 |  |  |  |  |  |  |
| GO:0060841\_venous\_blood\_vessel\_development | PROX1 | 3 | 1 |  |  |  |  |  |  |
| GO:0070307\_lens\_fiber\_cell\_development | PROX1 | 3 | 1 |  |  |  |  |  |  |
| GO:0070309\_lens\_fiber\_cell\_morphogenesis | PROX1 | 3 | 1 |  |  |  |  |  |  |
| GO:0051216\_cartilage\_development | PRRX1 | 63 | 3 | 4.137466 | -1.453311 | 154 | 31.08 | 0.201818 |  |
| GO:0051216\_cartilage\_development | CTNNB1 | 63 | 3 | 4.137466 | -1.453311 | 154 | 31.08 | 0.201818 |  |
| GO:0051216\_cartilage\_development | TGFB2 | 63 | 3 | 4.137466 | -1.453311 | 154 | 31.08 | 0.201818 |  |
| GO:0007166\_cell\_surface\_receptor\_linked\_signal\_transduction | SCHIP1 | 597 | 12 | 1.746468 | -1.451696 | 155 | 31.13 | 0.200839 |  |
| GO:0007166\_cell\_surface\_receptor\_linked\_signal\_transduction | BMP2 | 597 | 12 | 1.746468 | -1.451696 | 155 | 31.13 | 0.200839 |  |
| GO:0007166\_cell\_surface\_receptor\_linked\_signal\_transduction | FZD10 | 597 | 12 | 1.746468 | -1.451696 | 155 | 31.13 | 0.200839 |  |
| GO:0007166\_cell\_surface\_receptor\_linked\_signal\_transduction | VAV3 | 597 | 12 | 1.746468 | -1.451696 | 155 | 31.13 | 0.200839 |  |
| GO:0007166\_cell\_surface\_receptor\_linked\_signal\_transduction | PLEK | 597 | 12 | 1.746468 | -1.451696 | 155 | 31.13 | 0.200839 |  |
| GO:0007166\_cell\_surface\_receptor\_linked\_signal\_transduction | RSPO3 | 597 | 12 | 1.746468 | -1.451696 | 155 | 31.13 | 0.200839 |  |
| GO:0007166\_cell\_surface\_receptor\_linked\_signal\_transduction | PRRX1 | 597 | 12 | 1.746468 | -1.451696 | 155 | 31.13 | 0.200839 |  |
| GO:0007166\_cell\_surface\_receptor\_linked\_signal\_transduction | FOXC1 | 597 | 12 | 1.746468 | -1.451696 | 155 | 31.13 | 0.200839 |  |
| GO:0007166\_cell\_surface\_receptor\_linked\_signal\_transduction | ZEB2 | 597 | 12 | 1.746468 | -1.451696 | 155 | 31.13 | 0.200839 |  |
| GO:0007166\_cell\_surface\_receptor\_linked\_signal\_transduction | TGFB2 | 597 | 12 | 1.746468 | -1.451696 | 155 | 31.13 | 0.200839 |  |
| GO:0007166\_cell\_surface\_receptor\_linked\_signal\_transduction | CITED2 | 597 | 12 | 1.746468 | -1.451696 | 155 | 31.13 | 0.200839 |  |
| GO:0007166\_cell\_surface\_receptor\_linked\_signal\_transduction | CTNNB1 | 597 | 12 | 1.746468 | -1.451696 | 155 | 31.13 | 0.200839 |  |
| GO:0010647\_positive\_regulation\_of\_cell\_communication | PRRX1 | 110 | 4 | 3.159520 | -1.433628 | 157 | 32.38 | 0.206242 |  |
| GO:0010647\_positive\_regulation\_of\_cell\_communication | ZEB2 | 110 | 4 | 3.159520 | -1.433628 | 157 | 32.38 | 0.206242 |  |
| GO:0010647\_positive\_regulation\_of\_cell\_communication | CITED2 | 110 | 4 | 3.159520 | -1.433628 | 157 | 32.38 | 0.206242 |  |
| GO:0010647\_positive\_regulation\_of\_cell\_communication | CTNNB1 | 110 | 4 | 3.159520 | -1.433628 | 157 | 32.38 | 0.206242 |  |
| GO:0043010\_camera-type\_eye\_development | RBP4 | 110 | 4 | 3.159520 | -1.433628 | 157 | 32.38 | 0.206242 |  |
| GO:0043010\_camera-type\_eye\_development | FOXC1 | 110 | 4 | 3.159520 | -1.433628 | 157 | 32.38 | 0.206242 |  |
| GO:0043010\_camera-type\_eye\_development | PROX1 | 110 | 4 | 3.159520 | -1.433628 | 157 | 32.38 | 0.206242 |  |
| GO:0043010\_camera-type\_eye\_development | CTNNB1 | 110 | 4 | 3.159520 | -1.433628 | 157 | 32.38 | 0.206242 |  |
| GO:0045941\_positive\_regulation\_of\_transcription | BMP2 | 338 | 8 | 2.056492 | -1.425066 | 158 | 32.48 | 0.205570 |  |
| GO:0045941\_positive\_regulation\_of\_transcription | KLF12 | 338 | 8 | 2.056492 | -1.425066 | 158 | 32.48 | 0.205570 |  |
| GO:0045941\_positive\_regulation\_of\_transcription | JUN | 338 | 8 | 2.056492 | -1.425066 | 158 | 32.48 | 0.205570 |  |
| GO:0045941\_positive\_regulation\_of\_transcription | TBX5 | 338 | 8 | 2.056492 | -1.425066 | 158 | 32.48 | 0.205570 |  |
| GO:0045941\_positive\_regulation\_of\_transcription | FOXC1 | 338 | 8 | 2.056492 | -1.425066 | 158 | 32.48 | 0.205570 |  |
| GO:0045941\_positive\_regulation\_of\_transcription | RORA | 338 | 8 | 2.056492 | -1.425066 | 158 | 32.48 | 0.205570 |  |
| GO:0045941\_positive\_regulation\_of\_transcription | CTNNB1 | 338 | 8 | 2.056492 | -1.425066 | 158 | 32.48 | 0.205570 |  |
| GO:0045941\_positive\_regulation\_of\_transcription | CITED2 | 338 | 8 | 2.056492 | -1.425066 | 158 | 32.48 | 0.205570 |  |
| GO:0010638\_positive\_regulation\_of\_organelle\_organization | PLEK | 27 | 2 | 6.436059 | -1.420515 | 160 | 33.37 | 0.208562 |  |
| GO:0010638\_positive\_regulation\_of\_organelle\_organization | PROX1 | 27 | 2 | 6.436059 | -1.420515 | 160 | 33.37 | 0.208562 |  |
| GO:0031016\_pancreas\_development | INSM1 | 27 | 2 | 6.436059 | -1.420515 | 160 | 33.37 | 0.208562 |  |
| GO:0031016\_pancreas\_development | CTNNB1 | 27 | 2 | 6.436059 | -1.420515 | 160 | 33.37 | 0.208562 |  |
| GO:0007423\_sensory\_organ\_development | RBP4 | 219 | 6 | 2.380460 | -1.415830 | 161 | 33.57 | 0.208509 |  |
| GO:0007423\_sensory\_organ\_development | PRRX1 | 219 | 6 | 2.380460 | -1.415830 | 161 | 33.57 | 0.208509 |  |
| GO:0007423\_sensory\_organ\_development | DLL1 | 219 | 6 | 2.380460 | -1.415830 | 161 | 33.57 | 0.208509 |  |
| GO:0007423\_sensory\_organ\_development | FOXC1 | 219 | 6 | 2.380460 | -1.415830 | 161 | 33.57 | 0.208509 |  |
| GO:0007423\_sensory\_organ\_development | PROX1 | 219 | 6 | 2.380460 | -1.415830 | 161 | 33.57 | 0.208509 |  |
| GO:0007423\_sensory\_organ\_development | CTNNB1 | 219 | 6 | 2.380460 | -1.415830 | 161 | 33.57 | 0.208509 |  |
| GO:0006793\_phosphorus\_metabolic\_process | WNT5A | 340 | 8 | 2.044395 | -1.412046 | 163 | 33.66 | 0.206503 |  |
| GO:0006793\_phosphorus\_metabolic\_process | VAV3 | 340 | 8 | 2.044395 | -1.412046 | 163 | 33.66 | 0.206503 |  |
| GO:0006793\_phosphorus\_metabolic\_process | RB1CC1 | 340 | 8 | 2.044395 | -1.412046 | 163 | 33.66 | 0.206503 |  |
| GO:0006793\_phosphorus\_metabolic\_process | JUN | 340 | 8 | 2.044395 | -1.412046 | 163 | 33.66 | 0.206503 |  |
| GO:0006793\_phosphorus\_metabolic\_process | LRP8 | 340 | 8 | 2.044395 | -1.412046 | 163 | 33.66 | 0.206503 |  |
| GO:0006793\_phosphorus\_metabolic\_process | ZEB2 | 340 | 8 | 2.044395 | -1.412046 | 163 | 33.66 | 0.206503 |  |
| GO:0006793\_phosphorus\_metabolic\_process | PRKG1 | 340 | 8 | 2.044395 | -1.412046 | 163 | 33.66 | 0.206503 |  |
| GO:0006793\_phosphorus\_metabolic\_process | TGFB2 | 340 | 8 | 2.044395 | -1.412046 | 163 | 33.66 | 0.206503 |  |
| GO:0006796\_phosphate\_metabolic\_process | WNT5A | 340 | 8 | 2.044395 | -1.412046 | 163 | 33.66 | 0.206503 |  |
| GO:0006796\_phosphate\_metabolic\_process | VAV3 | 340 | 8 | 2.044395 | -1.412046 | 163 | 33.66 | 0.206503 |  |
| GO:0006796\_phosphate\_metabolic\_process | RB1CC1 | 340 | 8 | 2.044395 | -1.412046 | 163 | 33.66 | 0.206503 |  |
| GO:0006796\_phosphate\_metabolic\_process | JUN | 340 | 8 | 2.044395 | -1.412046 | 163 | 33.66 | 0.206503 |  |
| GO:0006796\_phosphate\_metabolic\_process | LRP8 | 340 | 8 | 2.044395 | -1.412046 | 163 | 33.66 | 0.206503 |  |
| GO:0006796\_phosphate\_metabolic\_process | ZEB2 | 340 | 8 | 2.044395 | -1.412046 | 163 | 33.66 | 0.206503 |  |
| GO:0006796\_phosphate\_metabolic\_process | PRKG1 | 340 | 8 | 2.044395 | -1.412046 | 163 | 33.66 | 0.206503 |  |
| GO:0006796\_phosphate\_metabolic\_process | TGFB2 | 340 | 8 | 2.044395 | -1.412046 | 163 | 33.66 | 0.206503 |  |
| GO:0042325\_regulation\_of\_phosphorylation | VAV3 | 164 | 5 | 2.648988 | -1.408174 | 164 | 33.79 | 0.206037 |  |
| GO:0042325\_regulation\_of\_phosphorylation | RB1CC1 | 164 | 5 | 2.648988 | -1.408174 | 164 | 33.79 | 0.206037 |  |
| GO:0042325\_regulation\_of\_phosphorylation | JUN | 164 | 5 | 2.648988 | -1.408174 | 164 | 33.79 | 0.206037 |  |
| GO:0042325\_regulation\_of\_phosphorylation | LRP8 | 164 | 5 | 2.648988 | -1.408174 | 164 | 33.79 | 0.206037 |  |
| GO:0042325\_regulation\_of\_phosphorylation | ZEB2 | 164 | 5 | 2.648988 | -1.408174 | 164 | 33.79 | 0.206037 |  |
| GO:0031589\_cell-substrate\_adhesion | A930038C07RIK | 66 | 3 | 3.949400 | -1.402123 | 165 | 34.2 | 0.207273 |  |
| GO:0031589\_cell-substrate\_adhesion | CCDC80 | 66 | 3 | 3.949400 | -1.402123 | 165 | 34.2 | 0.207273 |  |
| GO:0031589\_cell-substrate\_adhesion | CTNNB1 | 66 | 3 | 3.949400 | -1.402123 | 165 | 34.2 | 0.207273 |  |
| GO:0019220\_regulation\_of\_phosphate\_metabolic\_process | VAV3 | 165 | 5 | 2.632933 | -1.398530 | 167 | 34.34 | 0.205629 |  |
| GO:0019220\_regulation\_of\_phosphate\_metabolic\_process | RB1CC1 | 165 | 5 | 2.632933 | -1.398530 | 167 | 34.34 | 0.205629 |  |
| GO:0019220\_regulation\_of\_phosphate\_metabolic\_process | JUN | 165 | 5 | 2.632933 | -1.398530 | 167 | 34.34 | 0.205629 |  |
| GO:0019220\_regulation\_of\_phosphate\_metabolic\_process | LRP8 | 165 | 5 | 2.632933 | -1.398530 | 167 | 34.34 | 0.205629 |  |
| GO:0019220\_regulation\_of\_phosphate\_metabolic\_process | ZEB2 | 165 | 5 | 2.632933 | -1.398530 | 167 | 34.34 | 0.205629 |  |
| GO:0051174\_regulation\_of\_phosphorus\_metabolic\_process | VAV3 | 165 | 5 | 2.632933 | -1.398530 | 167 | 34.34 | 0.205629 |  |
| GO:0051174\_regulation\_of\_phosphorus\_metabolic\_process | JUN | 165 | 5 | 2.632933 | -1.398530 | 167 | 34.34 | 0.205629 |  |
| GO:0051174\_regulation\_of\_phosphorus\_metabolic\_process | RB1CC1 | 165 | 5 | 2.632933 | -1.398530 | 167 | 34.34 | 0.205629 |  |
| GO:0051174\_regulation\_of\_phosphorus\_metabolic\_process | LRP8 | 165 | 5 | 2.632933 | -1.398530 | 167 | 34.34 | 0.205629 |  |
| GO:0051174\_regulation\_of\_phosphorus\_metabolic\_process | ZEB2 | 165 | 5 | 2.632933 | -1.398530 | 167 | 34.34 | 0.205629 |  |
| GO:0043193\_positive\_regulation\_of\_gene-specific\_transcription | BMP2 | 28 | 2 | 6.206199 | -1.391486 | 168 | 34.97 | 0.208155 |  |
| GO:0043193\_positive\_regulation\_of\_gene-specific\_transcription | CITED2 | 28 | 2 | 6.206199 | -1.391486 | 168 | 34.97 | 0.208155 |  |
| GO:0003007\_heart\_morphogenesis | RBP4 | 67 | 3 | 3.890453 | -1.385688 | 169 | 35.22 | 0.208402 |  |
| GO:0003007\_heart\_morphogenesis | FOXC1 | 67 | 3 | 3.890453 | -1.385688 | 169 | 35.22 | 0.208402 |  |
| GO:0003007\_heart\_morphogenesis | PROX1 | 67 | 3 | 3.890453 | -1.385688 | 169 | 35.22 | 0.208402 |  |
| GO:0000165\_MAPKKK\_cascade | WNT5A | 114 | 4 | 3.048659 | -1.385225 | 170 | 35.31 | 0.207706 |  |
| GO:0000165\_MAPKKK\_cascade | RB1CC1 | 114 | 4 | 3.048659 | -1.385225 | 170 | 35.31 | 0.207706 |  |
| GO:0000165\_MAPKKK\_cascade | ZEB2 | 114 | 4 | 3.048659 | -1.385225 | 170 | 35.31 | 0.207706 |  |
| GO:0000165\_MAPKKK\_cascade | CTNNB1 | 114 | 4 | 3.048659 | -1.385225 | 170 | 35.31 | 0.207706 |  |
| GO:0010628\_positive\_regulation\_of\_gene\_expression | BMP2 | 346 | 8 | 2.008943 | -1.373714 | 171 | 35.64 | 0.208421 |  |
| GO:0010628\_positive\_regulation\_of\_gene\_expression | KLF12 | 346 | 8 | 2.008943 | -1.373714 | 171 | 35.64 | 0.208421 |  |
| GO:0010628\_positive\_regulation\_of\_gene\_expression | JUN | 346 | 8 | 2.008943 | -1.373714 | 171 | 35.64 | 0.208421 |  |
| GO:0010628\_positive\_regulation\_of\_gene\_expression | TBX5 | 346 | 8 | 2.008943 | -1.373714 | 171 | 35.64 | 0.208421 |  |
| GO:0010628\_positive\_regulation\_of\_gene\_expression | FOXC1 | 346 | 8 | 2.008943 | -1.373714 | 171 | 35.64 | 0.208421 |  |
| GO:0010628\_positive\_regulation\_of\_gene\_expression | RORA | 346 | 8 | 2.008943 | -1.373714 | 171 | 35.64 | 0.208421 |  |
| GO:0010628\_positive\_regulation\_of\_gene\_expression | CTNNB1 | 346 | 8 | 2.008943 | -1.373714 | 171 | 35.64 | 0.208421 |  |
| GO:0010628\_positive\_regulation\_of\_gene\_expression | CITED2 | 346 | 8 | 2.008943 | -1.373714 | 171 | 35.64 | 0.208421 |  |
| GO:0021761\_limbic\_system\_development | LRP8 | 29 | 2 | 5.992193 | -1.363605 | 174 | 36.81 | 0.211552 |  |
| GO:0021761\_limbic\_system\_development | ZEB2 | 29 | 2 | 5.992193 | -1.363605 | 174 | 36.81 | 0.211552 |  |
| GO:0050769\_positive\_regulation\_of\_neurogenesis | XRCC4 | 29 | 2 | 5.992193 | -1.363605 | 174 | 36.81 | 0.211552 |  |
| GO:0050769\_positive\_regulation\_of\_neurogenesis | BMP2 | 29 | 2 | 5.992193 | -1.363605 | 174 | 36.81 | 0.211552 |  |
| GO:0051301\_cell\_division | RBP4 | 29 | 2 | 5.992193 | -1.363605 | 174 | 36.81 | 0.211552 |  |
| GO:0051301\_cell\_division | TGFB2 | 29 | 2 | 5.992193 | -1.363605 | 174 | 36.81 | 0.211552 |  |
| GO:0048608\_reproductive\_structure\_development | WNT5A | 116 | 4 | 2.996096 | -1.361851 | 175 | 36.91 | 0.210914 |  |
| GO:0048608\_reproductive\_structure\_development | RBP4 | 116 | 4 | 2.996096 | -1.361851 | 175 | 36.91 | 0.210914 |  |
| GO:0048608\_reproductive\_structure\_development | FOXC1 | 116 | 4 | 2.996096 | -1.361851 | 175 | 36.91 | 0.210914 |  |
| GO:0048608\_reproductive\_structure\_development | CTNNB1 | 116 | 4 | 2.996096 | -1.361851 | 175 | 36.91 | 0.210914 |  |
| GO:0044260\_cellular\_macromolecule\_metabolic\_process | WNT5A | 1447 | 23 | 1.381062 | -1.361378 | 176 | 36.95 | 0.209943 |  |
| GO:0044260\_cellular\_macromolecule\_metabolic\_process | EGR1 | 1447 | 23 | 1.381062 | -1.361378 | 176 | 36.95 | 0.209943 |  |
| GO:0044260\_cellular\_macromolecule\_metabolic\_process | XRCC4 | 1447 | 23 | 1.381062 | -1.361378 | 176 | 36.95 | 0.209943 |  |
| GO:0044260\_cellular\_macromolecule\_metabolic\_process | BMP2 | 1447 | 23 | 1.381062 | -1.361378 | 176 | 36.95 | 0.209943 |  |
| GO:0044260\_cellular\_macromolecule\_metabolic\_process | PLEK | 1447 | 23 | 1.381062 | -1.361378 | 176 | 36.95 | 0.209943 |  |
| GO:0044260\_cellular\_macromolecule\_metabolic\_process | KLF12 | 1447 | 23 | 1.381062 | -1.361378 | 176 | 36.95 | 0.209943 |  |
| GO:0044260\_cellular\_macromolecule\_metabolic\_process | TBX5 | 1447 | 23 | 1.381062 | -1.361378 | 176 | 36.95 | 0.209943 |  |
| GO:0044260\_cellular\_macromolecule\_metabolic\_process | ZEB2 | 1447 | 23 | 1.381062 | -1.361378 | 176 | 36.95 | 0.209943 |  |
| GO:0044260\_cellular\_macromolecule\_metabolic\_process | DCN | 1447 | 23 | 1.381062 | -1.361378 | 176 | 36.95 | 0.209943 |  |
| GO:0044260\_cellular\_macromolecule\_metabolic\_process | RORA | 1447 | 23 | 1.381062 | -1.361378 | 176 | 36.95 | 0.209943 |  |
| GO:0044260\_cellular\_macromolecule\_metabolic\_process | PRKG1 | 1447 | 23 | 1.381062 | -1.361378 | 176 | 36.95 | 0.209943 |  |
| GO:0044260\_cellular\_macromolecule\_metabolic\_process | FOXP4 | 1447 | 23 | 1.381062 | -1.361378 | 176 | 36.95 | 0.209943 |  |
| GO:0044260\_cellular\_macromolecule\_metabolic\_process | TGFB2 | 1447 | 23 | 1.381062 | -1.361378 | 176 | 36.95 | 0.209943 |  |
| GO:0044260\_cellular\_macromolecule\_metabolic\_process | CTNNB1 | 1447 | 23 | 1.381062 | -1.361378 | 176 | 36.95 | 0.209943 |  |
| GO:0044260\_cellular\_macromolecule\_metabolic\_process | CITED2 | 1447 | 23 | 1.381062 | -1.361378 | 176 | 36.95 | 0.209943 |  |
| GO:0044260\_cellular\_macromolecule\_metabolic\_process | A930038C07RIK | 1447 | 23 | 1.381062 | -1.361378 | 176 | 36.95 | 0.209943 |  |
| GO:0044260\_cellular\_macromolecule\_metabolic\_process | BTG2 | 1447 | 23 | 1.381062 | -1.361378 | 176 | 36.95 | 0.209943 |  |
| GO:0044260\_cellular\_macromolecule\_metabolic\_process | APOE | 1447 | 23 | 1.381062 | -1.361378 | 176 | 36.95 | 0.209943 |  |
| GO:0044260\_cellular\_macromolecule\_metabolic\_process | RB1CC1 | 1447 | 23 | 1.381062 | -1.361378 | 176 | 36.95 | 0.209943 |  |
| GO:0044260\_cellular\_macromolecule\_metabolic\_process | JUN | 1447 | 23 | 1.381062 | -1.361378 | 176 | 36.95 | 0.209943 |  |
| GO:0044260\_cellular\_macromolecule\_metabolic\_process | FOXC1 | 1447 | 23 | 1.381062 | -1.361378 | 176 | 36.95 | 0.209943 |  |
| GO:0044260\_cellular\_macromolecule\_metabolic\_process | HBA-X | 1447 | 23 | 1.381062 | -1.361378 | 176 | 36.95 | 0.209943 |  |
| GO:0044260\_cellular\_macromolecule\_metabolic\_process | C1D | 1447 | 23 | 1.381062 | -1.361378 | 176 | 36.95 | 0.209943 |  |
| GO:0001932\_regulation\_of\_protein\_amino\_acid\_phosphorylation | RB1CC1 | 69 | 3 | 3.777687 | -1.353700 | 177 | 37.15 | 0.209887 |  |
| GO:0001932\_regulation\_of\_protein\_amino\_acid\_phosphorylation | JUN | 69 | 3 | 3.777687 | -1.353700 | 177 | 37.15 | 0.209887 |  |
| GO:0001932\_regulation\_of\_protein\_amino\_acid\_phosphorylation | ZEB2 | 69 | 3 | 3.777687 | -1.353700 | 177 | 37.15 | 0.209887 |  |
| GO:0006707\_cholesterol\_catabolic\_process | APOE | 4 | 1 |  |  |  |  |  |  |
| GO:0006906\_vesicle\_fusion | VAV3 | 4 | 1 |  |  |  |  |  |  |
| GO:0007386\_compartment\_specification | DLL1 | 4 | 1 |  |  |  |  |  |  |
| GO:0016127\_sterol\_catabolic\_process | APOE | 4 | 1 |  |  |  |  |  |  |
| GO:0021801\_cerebral\_cortex\_radial\_glia\_guided\_migration | LRP8 | 4 | 1 |  |  |  |  |  |  |
| GO:0030858\_positive\_regulation\_of\_epithelial\_cell\_differentiation | CTNNB1 | 4 | 1 |  |  |  |  |  |  |
| GO:0032808\_lacrimal\_gland\_development | FOXC1 | 4 | 1 |  |  |  |  |  |  |
| GO:0045940\_positive\_regulation\_of\_steroid\_metabolic\_process | APOE | 4 | 1 |  |  |  |  |  |  |
| GO:0048240\_sperm\_capacitation | SEPT4 | 4 | 1 |  |  |  |  |  |  |
| GO:0048341\_paraxial\_mesoderm\_formation | FOXC1 | 4 | 1 |  |  |  |  |  |  |
| GO:0048639\_positive\_regulation\_of\_developmental\_growth | PROX1 | 4 | 1 |  |  |  |  |  |  |
| GO:0048710\_regulation\_of\_astrocyte\_differentiation | BMP2 | 4 | 1 |  |  |  |  |  |  |
| GO:0050919\_negative\_chemotaxis | NRP2 | 4 | 1 |  |  |  |  |  |  |
| GO:0051024\_positive\_regulation\_of\_immunoglobulin\_secretion | RBP4 | 4 | 1 |  |  |  |  |  |  |
| GO:0055009\_atrial\_cardiac\_muscle\_morphogenesis | PROX1 | 4 | 1 |  |  |  |  |  |  |
| GO:0060065\_uterus\_development | RBP4 | 4 | 1 |  |  |  |  |  |  |
| GO:0060412\_ventricular\_septum\_morphogenesis | PROX1 | 4 | 1 |  |  |  |  |  |  |
| GO:0060751\_mammary\_gland\_duct\_branch\_elongation | WNT5A | 4 | 1 |  |  |  |  |  |  |
| GO:0060900\_embryonic\_camera-type\_eye\_formation | PROX1 | 4 | 1 |  |  |  |  |  |  |
| GO:0008406\_gonad\_development | WNT5A | 70 | 3 | 3.723720 | -1.338131 | 179 | 37.67 | 0.210447 |  |
| GO:0008406\_gonad\_development | RBP4 | 70 | 3 | 3.723720 | -1.338131 | 179 | 37.67 | 0.210447 |  |
| GO:0008406\_gonad\_development | FOXC1 | 70 | 3 | 3.723720 | -1.338131 | 179 | 37.67 | 0.210447 |  |
| GO:0048592\_eye\_morphogenesis | RBP4 | 70 | 3 | 3.723720 | -1.338131 | 179 | 37.67 | 0.210447 |  |
| GO:0048592\_eye\_morphogenesis | PROX1 | 70 | 3 | 3.723720 | -1.338131 | 179 | 37.67 | 0.210447 |  |
| GO:0048592\_eye\_morphogenesis | CTNNB1 | 70 | 3 | 3.723720 | -1.338131 | 179 | 37.67 | 0.210447 |  |
| GO:0035265\_organ\_growth | FOXC1 | 30 | 2 | 5.792453 | -1.336792 | 183 | 38.85 | 0.212295 |  |
| GO:0035265\_organ\_growth | PROX1 | 30 | 2 | 5.792453 | -1.336792 | 183 | 38.85 | 0.212295 |  |
| GO:0048565\_gut\_development | WNT5A | 30 | 2 | 5.792453 | -1.336792 | 183 | 38.85 | 0.212295 |  |
| GO:0048565\_gut\_development | FOXP4 | 30 | 2 | 5.792453 | -1.336792 | 183 | 38.85 | 0.212295 |  |
| GO:0051146\_striated\_muscle\_cell\_differentiation | TBX5 | 30 | 2 | 5.792453 | -1.336792 | 183 | 38.85 | 0.212295 |  |
| GO:0051146\_striated\_muscle\_cell\_differentiation | PROX1 | 30 | 2 | 5.792453 | -1.336792 | 183 | 38.85 | 0.212295 |  |
| GO:0060021\_palate\_development | SCHIP1 | 30 | 2 | 5.792453 | -1.336792 | 183 | 38.85 | 0.212295 |  |
| GO:0060021\_palate\_development | PRRX1 | 30 | 2 | 5.792453 | -1.336792 | 183 | 38.85 | 0.212295 |  |
| GO:0045935\_positive\_regulation\_of\_nucleobase\_\_nucleoside\_\_nucleotide\_and\_nucleic\_acid\_metabolic\_process | BMP2 | 352 | 8 | 1.974700 | -1.336446 | 184 | 38.9 | 0.211413 |  |
| GO:0045935\_positive\_regulation\_of\_nucleobase\_\_nucleoside\_\_nucleotide\_and\_nucleic\_acid\_metabolic\_process | KLF12 | 352 | 8 | 1.974700 | -1.336446 | 184 | 38.9 | 0.211413 |  |
| GO:0045935\_positive\_regulation\_of\_nucleobase\_\_nucleoside\_\_nucleotide\_and\_nucleic\_acid\_metabolic\_process | JUN | 352 | 8 | 1.974700 | -1.336446 | 184 | 38.9 | 0.211413 |  |
| GO:0045935\_positive\_regulation\_of\_nucleobase\_\_nucleoside\_\_nucleotide\_and\_nucleic\_acid\_metabolic\_process | TBX5 | 352 | 8 | 1.974700 | -1.336446 | 184 | 38.9 | 0.211413 |  |
| GO:0045935\_positive\_regulation\_of\_nucleobase\_\_nucleoside\_\_nucleotide\_and\_nucleic\_acid\_metabolic\_process | FOXC1 | 352 | 8 | 1.974700 | -1.336446 | 184 | 38.9 | 0.211413 |  |
| GO:0045935\_positive\_regulation\_of\_nucleobase\_\_nucleoside\_\_nucleotide\_and\_nucleic\_acid\_metabolic\_process | RORA | 352 | 8 | 1.974700 | -1.336446 | 184 | 38.9 | 0.211413 |  |
| GO:0045935\_positive\_regulation\_of\_nucleobase\_\_nucleoside\_\_nucleotide\_and\_nucleic\_acid\_metabolic\_process | CTNNB1 | 352 | 8 | 1.974700 | -1.336446 | 184 | 38.9 | 0.211413 |  |
| GO:0045935\_positive\_regulation\_of\_nucleobase\_\_nucleoside\_\_nucleotide\_and\_nucleic\_acid\_metabolic\_process | CITED2 | 352 | 8 | 1.974700 | -1.336446 | 184 | 38.9 | 0.211413 |  |
| GO:0016331\_morphogenesis\_of\_embryonic\_epithelium | WNT5A | 71 | 3 | 3.671273 | -1.322834 | 186 | 39.49 | 0.212312 |  |
| GO:0016331\_morphogenesis\_of\_embryonic\_epithelium | ZEB2 | 71 | 3 | 3.671273 | -1.322834 | 186 | 39.49 | 0.212312 |  |
| GO:0016331\_morphogenesis\_of\_embryonic\_epithelium | CTNNB1 | 71 | 3 | 3.671273 | -1.322834 | 186 | 39.49 | 0.212312 |  |
| GO:0033674\_positive\_regulation\_of\_kinase\_activity | VAV3 | 71 | 3 | 3.671273 | -1.322834 | 186 | 39.49 | 0.212312 |  |
| GO:0033674\_positive\_regulation\_of\_kinase\_activity | LRP8 | 71 | 3 | 3.671273 | -1.322834 | 186 | 39.49 | 0.212312 |  |
| GO:0033674\_positive\_regulation\_of\_kinase\_activity | ZEB2 | 71 | 3 | 3.671273 | -1.322834 | 186 | 39.49 | 0.212312 |  |
| GO:0009306\_protein\_secretion | RBP4 | 31 | 2 | 5.605600 | -1.310976 | 188 | 41.17 | 0.218989 |  |
| GO:0009306\_protein\_secretion | PLEK | 31 | 2 | 5.605600 | -1.310976 | 188 | 41.17 | 0.218989 |  |
| GO:0048562\_embryonic\_organ\_morphogenesis | RBP4 | 31 | 2 | 5.605600 | -1.310976 | 188 | 41.17 | 0.218989 |  |
| GO:0048562\_embryonic\_organ\_morphogenesis | PROX1 | 31 | 2 | 5.605600 | -1.310976 | 188 | 41.17 | 0.218989 |  |
| GO:0007264\_small\_GTPase\_mediated\_signal\_transduction | RHOJ | 72 | 3 | 3.620283 | -1.307802 | 190 | 41.81 | 0.220053 |  |
| GO:0007264\_small\_GTPase\_mediated\_signal\_transduction | VAV3 | 72 | 3 | 3.620283 | -1.307802 | 190 | 41.81 | 0.220053 |  |
| GO:0007264\_small\_GTPase\_mediated\_signal\_transduction | CDC42EP4 | 72 | 3 | 3.620283 | -1.307802 | 190 | 41.81 | 0.220053 |  |
| GO:0051347\_positive\_regulation\_of\_transferase\_activity | VAV3 | 72 | 3 | 3.620283 | -1.307802 | 190 | 41.81 | 0.220053 |  |
| GO:0051347\_positive\_regulation\_of\_transferase\_activity | LRP8 | 72 | 3 | 3.620283 | -1.307802 | 190 | 41.81 | 0.220053 |  |
| GO:0051347\_positive\_regulation\_of\_transferase\_activity | ZEB2 | 72 | 3 | 3.620283 | -1.307802 | 190 | 41.81 | 0.220053 |  |
| GO:0051726\_regulation\_of\_cell\_cycle | JUN | 121 | 4 | 2.872291 | -1.305677 | 191 | 42.01 | 0.219948 |  |
| GO:0051726\_regulation\_of\_cell\_cycle | PROX1 | 121 | 4 | 2.872291 | -1.305677 | 191 | 42.01 | 0.219948 |  |
| GO:0051726\_regulation\_of\_cell\_cycle | TGFB2 | 121 | 4 | 2.872291 | -1.305677 | 191 | 42.01 | 0.219948 |  |
| GO:0051726\_regulation\_of\_cell\_cycle | CITED2 | 121 | 4 | 2.872291 | -1.305677 | 191 | 42.01 | 0.219948 |  |
| GO:0040011\_locomotion | NRP2 | 295 | 7 | 2.061720 | -1.297878 | 192 | 42.32 | 0.220417 |  |
| GO:0040011\_locomotion | SCHIP1 | 295 | 7 | 2.061720 | -1.297878 | 192 | 42.32 | 0.220417 |  |
| GO:0040011\_locomotion | VAV3 | 295 | 7 | 2.061720 | -1.297878 | 192 | 42.32 | 0.220417 |  |
| GO:0040011\_locomotion | LRP8 | 295 | 7 | 2.061720 | -1.297878 | 192 | 42.32 | 0.220417 |  |
| GO:0040011\_locomotion | FOXC1 | 295 | 7 | 2.061720 | -1.297878 | 192 | 42.32 | 0.220417 |  |
| GO:0040011\_locomotion | ZEB2 | 295 | 7 | 2.061720 | -1.297878 | 192 | 42.32 | 0.220417 |  |
| GO:0040011\_locomotion | PRKG1 | 295 | 7 | 2.061720 | -1.297878 | 192 | 42.32 | 0.220417 |  |
| GO:0060284\_regulation\_of\_cell\_development | XRCC4 | 122 | 4 | 2.848747 | -1.294813 | 193 | 42.66 | 0.221036 |  |
| GO:0060284\_regulation\_of\_cell\_development | BMP2 | 122 | 4 | 2.848747 | -1.294813 | 193 | 42.66 | 0.221036 |  |
| GO:0060284\_regulation\_of\_cell\_development | DLL1 | 122 | 4 | 2.848747 | -1.294813 | 193 | 42.66 | 0.221036 |  |
| GO:0060284\_regulation\_of\_cell\_development | PROX1 | 122 | 4 | 2.848747 | -1.294813 | 193 | 42.66 | 0.221036 |  |
| GO:0051493\_regulation\_of\_cytoskeleton\_organization | PLEK | 32 | 2 | 5.430425 | -1.286093 | 194 | 43.8 | 0.225773 |  |
| GO:0051493\_regulation\_of\_cytoskeleton\_organization | PROX1 | 32 | 2 | 5.430425 | -1.286093 | 194 | 43.8 | 0.225773 |  |
| GO:0051173\_positive\_regulation\_of\_nitrogen\_compound\_metabolic\_process | BMP2 | 361 | 8 | 1.925469 | -1.282456 | 195 | 43.92 | 0.225231 |  |
| GO:0051173\_positive\_regulation\_of\_nitrogen\_compound\_metabolic\_process | KLF12 | 361 | 8 | 1.925469 | -1.282456 | 195 | 43.92 | 0.225231 |  |
| GO:0051173\_positive\_regulation\_of\_nitrogen\_compound\_metabolic\_process | JUN | 361 | 8 | 1.925469 | -1.282456 | 195 | 43.92 | 0.225231 |  |
| GO:0051173\_positive\_regulation\_of\_nitrogen\_compound\_metabolic\_process | TBX5 | 361 | 8 | 1.925469 | -1.282456 | 195 | 43.92 | 0.225231 |  |
| GO:0051173\_positive\_regulation\_of\_nitrogen\_compound\_metabolic\_process | FOXC1 | 361 | 8 | 1.925469 | -1.282456 | 195 | 43.92 | 0.225231 |  |
| GO:0051173\_positive\_regulation\_of\_nitrogen\_compound\_metabolic\_process | RORA | 361 | 8 | 1.925469 | -1.282456 | 195 | 43.92 | 0.225231 |  |
| GO:0051173\_positive\_regulation\_of\_nitrogen\_compound\_metabolic\_process | CTNNB1 | 361 | 8 | 1.925469 | -1.282456 | 195 | 43.92 | 0.225231 |  |
| GO:0051173\_positive\_regulation\_of\_nitrogen\_compound\_metabolic\_process | CITED2 | 361 | 8 | 1.925469 | -1.282456 | 195 | 43.92 | 0.225231 |  |
| GO:0006468\_protein\_amino\_acid\_phosphorylation | WNT5A | 237 | 6 | 2.199666 | -1.275717 | 196 | 44.27 | 0.225867 |  |
| GO:0006468\_protein\_amino\_acid\_phosphorylation | RB1CC1 | 237 | 6 | 2.199666 | -1.275717 | 196 | 44.27 | 0.225867 |  |
| GO:0006468\_protein\_amino\_acid\_phosphorylation | JUN | 237 | 6 | 2.199666 | -1.275717 | 196 | 44.27 | 0.225867 |  |
| GO:0006468\_protein\_amino\_acid\_phosphorylation | ZEB2 | 237 | 6 | 2.199666 | -1.275717 | 196 | 44.27 | 0.225867 |  |
| GO:0006468\_protein\_amino\_acid\_phosphorylation | PRKG1 | 237 | 6 | 2.199666 | -1.275717 | 196 | 44.27 | 0.225867 |  |
| GO:0006468\_protein\_amino\_acid\_phosphorylation | TGFB2 | 237 | 6 | 2.199666 | -1.275717 | 196 | 44.27 | 0.225867 |  |
| GO:0009308\_amine\_metabolic\_process | A930038C07RIK | 124 | 4 | 2.802800 | -1.273437 | 197 | 44.32 | 0.224975 |  |
| GO:0009308\_amine\_metabolic\_process | FOXC1 | 124 | 4 | 2.802800 | -1.273437 | 197 | 44.32 | 0.224975 |  |
| GO:0009308\_amine\_metabolic\_process | DCN | 124 | 4 | 2.802800 | -1.273437 | 197 | 44.32 | 0.224975 |  |
| GO:0009308\_amine\_metabolic\_process | TGFB2 | 124 | 4 | 2.802800 | -1.273437 | 197 | 44.32 | 0.224975 |  |
| GO:0034960\_cellular\_biopolymer\_metabolic\_process | WNT5A | 1395 | 22 | 1.370258 | -1.271074 | 198 | 44.5 | 0.224747 |  |
| GO:0034960\_cellular\_biopolymer\_metabolic\_process | EGR1 | 1395 | 22 | 1.370258 | -1.271074 | 198 | 44.5 | 0.224747 |  |
| GO:0034960\_cellular\_biopolymer\_metabolic\_process | XRCC4 | 1395 | 22 | 1.370258 | -1.271074 | 198 | 44.5 | 0.224747 |  |
| GO:0034960\_cellular\_biopolymer\_metabolic\_process | BMP2 | 1395 | 22 | 1.370258 | -1.271074 | 198 | 44.5 | 0.224747 |  |
| GO:0034960\_cellular\_biopolymer\_metabolic\_process | PLEK | 1395 | 22 | 1.370258 | -1.271074 | 198 | 44.5 | 0.224747 |  |
| GO:0034960\_cellular\_biopolymer\_metabolic\_process | KLF12 | 1395 | 22 | 1.370258 | -1.271074 | 198 | 44.5 | 0.224747 |  |
| GO:0034960\_cellular\_biopolymer\_metabolic\_process | TBX5 | 1395 | 22 | 1.370258 | -1.271074 | 198 | 44.5 | 0.224747 |  |
| GO:0034960\_cellular\_biopolymer\_metabolic\_process | ZEB2 | 1395 | 22 | 1.370258 | -1.271074 | 198 | 44.5 | 0.224747 |  |
| GO:0034960\_cellular\_biopolymer\_metabolic\_process | DCN | 1395 | 22 | 1.370258 | -1.271074 | 198 | 44.5 | 0.224747 |  |
| GO:0034960\_cellular\_biopolymer\_metabolic\_process | RORA | 1395 | 22 | 1.370258 | -1.271074 | 198 | 44.5 | 0.224747 |  |
| GO:0034960\_cellular\_biopolymer\_metabolic\_process | PRKG1 | 1395 | 22 | 1.370258 | -1.271074 | 198 | 44.5 | 0.224747 |  |
| GO:0034960\_cellular\_biopolymer\_metabolic\_process | FOXP4 | 1395 | 22 | 1.370258 | -1.271074 | 198 | 44.5 | 0.224747 |  |
| GO:0034960\_cellular\_biopolymer\_metabolic\_process | TGFB2 | 1395 | 22 | 1.370258 | -1.271074 | 198 | 44.5 | 0.224747 |  |
| GO:0034960\_cellular\_biopolymer\_metabolic\_process | CTNNB1 | 1395 | 22 | 1.370258 | -1.271074 | 198 | 44.5 | 0.224747 |  |
| GO:0034960\_cellular\_biopolymer\_metabolic\_process | CITED2 | 1395 | 22 | 1.370258 | -1.271074 | 198 | 44.5 | 0.224747 |  |
| GO:0034960\_cellular\_biopolymer\_metabolic\_process | A930038C07RIK | 1395 | 22 | 1.370258 | -1.271074 | 198 | 44.5 | 0.224747 |  |
| GO:0034960\_cellular\_biopolymer\_metabolic\_process | BTG2 | 1395 | 22 | 1.370258 | -1.271074 | 198 | 44.5 | 0.224747 |  |
| GO:0034960\_cellular\_biopolymer\_metabolic\_process | RB1CC1 | 1395 | 22 | 1.370258 | -1.271074 | 198 | 44.5 | 0.224747 |  |
| GO:0034960\_cellular\_biopolymer\_metabolic\_process | JUN | 1395 | 22 | 1.370258 | -1.271074 | 198 | 44.5 | 0.224747 |  |
| GO:0034960\_cellular\_biopolymer\_metabolic\_process | FOXC1 | 1395 | 22 | 1.370258 | -1.271074 | 198 | 44.5 | 0.224747 |  |
| GO:0034960\_cellular\_biopolymer\_metabolic\_process | HBA-X | 1395 | 22 | 1.370258 | -1.271074 | 198 | 44.5 | 0.224747 |  |
| GO:0034960\_cellular\_biopolymer\_metabolic\_process | C1D | 1395 | 22 | 1.370258 | -1.271074 | 198 | 44.5 | 0.224747 |  |
| GO:0001763\_morphogenesis\_of\_a\_branching\_structure | WNT5A | 125 | 4 | 2.780377 | -1.262922 | 199 | 45.01 | 0.226181 |  |
| GO:0001763\_morphogenesis\_of\_a\_branching\_structure | BMP2 | 125 | 4 | 2.780377 | -1.262922 | 199 | 45.01 | 0.226181 |  |
| GO:0001763\_morphogenesis\_of\_a\_branching\_structure | RSPO3 | 125 | 4 | 2.780377 | -1.262922 | 199 | 45.01 | 0.226181 |  |
| GO:0001763\_morphogenesis\_of\_a\_branching\_structure | CTNNB1 | 125 | 4 | 2.780377 | -1.262922 | 199 | 45.01 | 0.226181 |  |
| GO:0008584\_male\_gonad\_development | WNT5A | 33 | 2 | 5.265866 | -1.262083 | 200 | 45.65 | 0.228250 |  |
| GO:0008584\_male\_gonad\_development | RBP4 | 33 | 2 | 5.265866 | -1.262083 | 200 | 45.65 | 0.228250 |  |
| GO:0043009\_chordate\_embryonic\_development | RBP4 | 365 | 8 | 1.904368 | -1.259171 | 201 | 45.81 | 0.227910 |  |
| GO:0043009\_chordate\_embryonic\_development | XRCC4 | 365 | 8 | 1.904368 | -1.259171 | 201 | 45.81 | 0.227910 |  |
| GO:0043009\_chordate\_embryonic\_development | RSPO3 | 365 | 8 | 1.904368 | -1.259171 | 201 | 45.81 | 0.227910 |  |
| GO:0043009\_chordate\_embryonic\_development | PRRX1 | 365 | 8 | 1.904368 | -1.259171 | 201 | 45.81 | 0.227910 |  |
| GO:0043009\_chordate\_embryonic\_development | ZEB2 | 365 | 8 | 1.904368 | -1.259171 | 201 | 45.81 | 0.227910 |  |
| GO:0043009\_chordate\_embryonic\_development | DLL1 | 365 | 8 | 1.904368 | -1.259171 | 201 | 45.81 | 0.227910 |  |
| GO:0043009\_chordate\_embryonic\_development | FOXC1 | 365 | 8 | 1.904368 | -1.259171 | 201 | 45.81 | 0.227910 |  |
| GO:0043009\_chordate\_embryonic\_development | CITED2 | 365 | 8 | 1.904368 | -1.259171 | 201 | 45.81 | 0.227910 |  |
| GO:0000726\_non-recombinational\_repair | XRCC4 | 5 | 1 | 17.377358 | -1.249796 | 233 | 62.02 | 0.266180 |  |
| GO:0001706\_endoderm\_formation | CTNNB1 | 5 | 1 | 17.377358 | -1.249796 | 233 | 62.02 | 0.266180 |  |
| GO:0002328\_pro-B\_cell\_differentiation | XRCC4 | 5 | 1 | 17.377358 | -1.249796 | 233 | 62.02 | 0.266180 |  |
| GO:0006303\_double-strand\_break\_repair\_via\_nonhomologous\_end\_joining | XRCC4 | 5 | 1 | 17.377358 | -1.249796 | 233 | 62.02 | 0.266180 |  |
| GO:0006924\_activation-induced\_cell\_death\_of\_T\_cells | TGFB2 | 5 | 1 | 17.377358 | -1.249796 | 233 | 62.02 | 0.266180 |  |
| GO:0009950\_dorsal\_ventral\_axis\_specification | CTNNB1 | 5 | 1 | 17.377358 | -1.249796 | 233 | 62.02 | 0.266180 |  |
| GO:0010761\_fibroblast\_migration | SCHIP1 | 5 | 1 | 17.377358 | -1.249796 | 233 | 62.02 | 0.266180 |  |
| GO:0014015\_positive\_regulation\_of\_gliogenesis | BMP2 | 5 | 1 | 17.377358 | -1.249796 | 233 | 62.02 | 0.266180 |  |
| GO:0030042\_actin\_filament\_depolymerization | PLEK | 5 | 1 | 17.377358 | -1.249796 | 233 | 62.02 | 0.266180 |  |
| GO:0030177\_positive\_regulation\_of\_Wnt\_receptor\_signaling\_pathway | ZEB2 | 5 | 1 | 17.377358 | -1.249796 | 233 | 62.02 | 0.266180 |  |
| GO:0030834\_regulation\_of\_actin\_filament\_depolymerization | PLEK | 5 | 1 | 17.377358 | -1.249796 | 233 | 62.02 | 0.266180 |  |
| GO:0033152\_immunoglobulin\_V(D)J\_recombination | XRCC4 | 5 | 1 | 17.377358 | -1.249796 | 233 | 62.02 | 0.266180 |  |
| GO:0034109\_homotypic\_cell-cell\_adhesion | PLEK | 5 | 1 | 17.377358 | -1.249796 | 233 | 62.02 | 0.266180 |  |
| GO:0034367\_macromolecular\_complex\_remodeling | APOE | 5 | 1 | 17.377358 | -1.249796 | 233 | 62.02 | 0.266180 |  |
| GO:0034368\_protein-lipid\_complex\_remodeling | APOE | 5 | 1 | 17.377358 | -1.249796 | 233 | 62.02 | 0.266180 |  |
| GO:0034369\_plasma\_lipoprotein\_particle\_remodeling | APOE | 5 | 1 | 17.377358 | -1.249796 | 233 | 62.02 | 0.266180 |  |
| GO:0042159\_lipoprotein\_catabolic\_process | APOE | 5 | 1 | 17.377358 | -1.249796 | 233 | 62.02 | 0.266180 |  |
| GO:0042416\_dopamine\_biosynthetic\_process | TGFB2 | 5 | 1 | 17.377358 | -1.249796 | 233 | 62.02 | 0.266180 |  |
| GO:0042574\_retinal\_metabolic\_process | RBP4 | 5 | 1 | 17.377358 | -1.249796 | 233 | 62.02 | 0.266180 |  |
| GO:0042695\_thelarche | WNT5A | 5 | 1 | 17.377358 | -1.249796 | 233 | 62.02 | 0.266180 |  |
| GO:0043030\_regulation\_of\_macrophage\_activation | RORA | 5 | 1 | 17.377358 | -1.249796 | 233 | 62.02 | 0.266180 |  |
| GO:0043288\_apocarotenoid\_metabolic\_process | RBP4 | 5 | 1 | 17.377358 | -1.249796 | 233 | 62.02 | 0.266180 |  |
| GO:0043550\_regulation\_of\_lipid\_kinase\_activity | VAV3 | 5 | 1 | 17.377358 | -1.249796 | 233 | 62.02 | 0.266180 |  |
| GO:0043551\_regulation\_of\_phosphoinositide\_3-kinase\_activity | VAV3 | 5 | 1 | 17.377358 | -1.249796 | 233 | 62.02 | 0.266180 |  |
| GO:0043552\_positive\_regulation\_of\_phosphoinositide\_3-kinase\_activity | VAV3 | 5 | 1 | 17.377358 | -1.249796 | 233 | 62.02 | 0.266180 |  |
| GO:0045687\_positive\_regulation\_of\_glial\_cell\_differentiation | BMP2 | 5 | 1 | 17.377358 | -1.249796 | 233 | 62.02 | 0.266180 |  |
| GO:0045793\_positive\_regulation\_of\_cell\_size | RB1CC1 | 5 | 1 | 17.377358 | -1.249796 | 233 | 62.02 | 0.266180 |  |
| GO:0046697\_decidualization | CITED2 | 5 | 1 | 17.377358 | -1.249796 | 233 | 62.02 | 0.266180 |  |
| GO:0055003\_cardiac\_myofibril\_assembly | PROX1 | 5 | 1 | 17.377358 | -1.249796 | 233 | 62.02 | 0.266180 |  |
| GO:0055015\_ventricular\_cardiac\_muscle\_cell\_development | PROX1 | 5 | 1 | 17.377358 | -1.249796 | 233 | 62.02 | 0.266180 |  |
| GO:0060744\_mammary\_gland\_branching\_involved\_in\_thelarche | WNT5A | 5 | 1 | 17.377358 | -1.249796 | 233 | 62.02 | 0.266180 |  |
| GO:0060762\_regulation\_of\_branching\_involved\_in\_mammary\_gland\_duct\_morphogenesis | WNT5A | 5 | 1 | 17.377358 | -1.249796 | 233 | 62.02 | 0.266180 |  |
| GO:0019222\_regulation\_of\_metabolic\_process | EGR1 | 1088 | 18 | 1.437465 | -1.246316 | 234 | 62.14 | 0.265556 |  |
| GO:0019222\_regulation\_of\_metabolic\_process | BMP2 | 1088 | 18 | 1.437465 | -1.246316 | 234 | 62.14 | 0.265556 |  |
| GO:0019222\_regulation\_of\_metabolic\_process | VAV3 | 1088 | 18 | 1.437465 | -1.246316 | 234 | 62.14 | 0.265556 |  |
| GO:0019222\_regulation\_of\_metabolic\_process | PLEK | 1088 | 18 | 1.437465 | -1.246316 | 234 | 62.14 | 0.265556 |  |
| GO:0019222\_regulation\_of\_metabolic\_process | KLF12 | 1088 | 18 | 1.437465 | -1.246316 | 234 | 62.14 | 0.265556 |  |
| GO:0019222\_regulation\_of\_metabolic\_process | TBX5 | 1088 | 18 | 1.437465 | -1.246316 | 234 | 62.14 | 0.265556 |  |
| GO:0019222\_regulation\_of\_metabolic\_process | ZEB2 | 1088 | 18 | 1.437465 | -1.246316 | 234 | 62.14 | 0.265556 |  |
| GO:0019222\_regulation\_of\_metabolic\_process | RORA | 1088 | 18 | 1.437465 | -1.246316 | 234 | 62.14 | 0.265556 |  |
| GO:0019222\_regulation\_of\_metabolic\_process | FOXP4 | 1088 | 18 | 1.437465 | -1.246316 | 234 | 62.14 | 0.265556 |  |
| GO:0019222\_regulation\_of\_metabolic\_process | CTNNB1 | 1088 | 18 | 1.437465 | -1.246316 | 234 | 62.14 | 0.265556 |  |
| GO:0019222\_regulation\_of\_metabolic\_process | CITED2 | 1088 | 18 | 1.437465 | -1.246316 | 234 | 62.14 | 0.265556 |  |
| GO:0019222\_regulation\_of\_metabolic\_process | APOE | 1088 | 18 | 1.437465 | -1.246316 | 234 | 62.14 | 0.265556 |  |
| GO:0019222\_regulation\_of\_metabolic\_process | RB1CC1 | 1088 | 18 | 1.437465 | -1.246316 | 234 | 62.14 | 0.265556 |  |
| GO:0019222\_regulation\_of\_metabolic\_process | JUN | 1088 | 18 | 1.437465 | -1.246316 | 234 | 62.14 | 0.265556 |  |
| GO:0019222\_regulation\_of\_metabolic\_process | LRP8 | 1088 | 18 | 1.437465 | -1.246316 | 234 | 62.14 | 0.265556 |  |
| GO:0019222\_regulation\_of\_metabolic\_process | FOXC1 | 1088 | 18 | 1.437465 | -1.246316 | 234 | 62.14 | 0.265556 |  |
| GO:0019222\_regulation\_of\_metabolic\_process | HBA-X | 1088 | 18 | 1.437465 | -1.246316 | 234 | 62.14 | 0.265556 |  |
| GO:0019222\_regulation\_of\_metabolic\_process | C1D | 1088 | 18 | 1.437465 | -1.246316 | 234 | 62.14 | 0.265556 |  |
| GO:0060255\_regulation\_of\_macromolecule\_metabolic\_process | EGR1 | 936 | 16 | 1.485244 | -1.243974 | 235 | 62.19 | 0.264638 |  |
| GO:0060255\_regulation\_of\_macromolecule\_metabolic\_process | BMP2 | 936 | 16 | 1.485244 | -1.243974 | 235 | 62.19 | 0.264638 |  |
| GO:0060255\_regulation\_of\_macromolecule\_metabolic\_process | PLEK | 936 | 16 | 1.485244 | -1.243974 | 235 | 62.19 | 0.264638 |  |
| GO:0060255\_regulation\_of\_macromolecule\_metabolic\_process | KLF12 | 936 | 16 | 1.485244 | -1.243974 | 235 | 62.19 | 0.264638 |  |
| GO:0060255\_regulation\_of\_macromolecule\_metabolic\_process | TBX5 | 936 | 16 | 1.485244 | -1.243974 | 235 | 62.19 | 0.264638 |  |
| GO:0060255\_regulation\_of\_macromolecule\_metabolic\_process | ZEB2 | 936 | 16 | 1.485244 | -1.243974 | 235 | 62.19 | 0.264638 |  |
| GO:0060255\_regulation\_of\_macromolecule\_metabolic\_process | RORA | 936 | 16 | 1.485244 | -1.243974 | 235 | 62.19 | 0.264638 |  |
| GO:0060255\_regulation\_of\_macromolecule\_metabolic\_process | FOXP4 | 936 | 16 | 1.485244 | -1.243974 | 235 | 62.19 | 0.264638 |  |
| GO:0060255\_regulation\_of\_macromolecule\_metabolic\_process | CITED2 | 936 | 16 | 1.485244 | -1.243974 | 235 | 62.19 | 0.264638 |  |
| GO:0060255\_regulation\_of\_macromolecule\_metabolic\_process | CTNNB1 | 936 | 16 | 1.485244 | -1.243974 | 235 | 62.19 | 0.264638 |  |
| GO:0060255\_regulation\_of\_macromolecule\_metabolic\_process | APOE | 936 | 16 | 1.485244 | -1.243974 | 235 | 62.19 | 0.264638 |  |
| GO:0060255\_regulation\_of\_macromolecule\_metabolic\_process | RB1CC1 | 936 | 16 | 1.485244 | -1.243974 | 235 | 62.19 | 0.264638 |  |
| GO:0060255\_regulation\_of\_macromolecule\_metabolic\_process | JUN | 936 | 16 | 1.485244 | -1.243974 | 235 | 62.19 | 0.264638 |  |
| GO:0060255\_regulation\_of\_macromolecule\_metabolic\_process | FOXC1 | 936 | 16 | 1.485244 | -1.243974 | 235 | 62.19 | 0.264638 |  |
| GO:0060255\_regulation\_of\_macromolecule\_metabolic\_process | HBA-X | 936 | 16 | 1.485244 | -1.243974 | 235 | 62.19 | 0.264638 |  |
| GO:0060255\_regulation\_of\_macromolecule\_metabolic\_process | C1D | 936 | 16 | 1.485244 | -1.243974 | 235 | 62.19 | 0.264638 |  |
| GO:0009792\_embryonic\_development\_ending\_in\_birth\_or\_egg\_hatching | RBP4 | 368 | 8 | 1.888843 | -1.241986 | 236 | 62.29 | 0.263941 |  |
| GO:0009792\_embryonic\_development\_ending\_in\_birth\_or\_egg\_hatching | XRCC4 | 368 | 8 | 1.888843 | -1.241986 | 236 | 62.29 | 0.263941 |  |
| GO:0009792\_embryonic\_development\_ending\_in\_birth\_or\_egg\_hatching | RSPO3 | 368 | 8 | 1.888843 | -1.241986 | 236 | 62.29 | 0.263941 |  |
| GO:0009792\_embryonic\_development\_ending\_in\_birth\_or\_egg\_hatching | PRRX1 | 368 | 8 | 1.888843 | -1.241986 | 236 | 62.29 | 0.263941 |  |
| GO:0009792\_embryonic\_development\_ending\_in\_birth\_or\_egg\_hatching | ZEB2 | 368 | 8 | 1.888843 | -1.241986 | 236 | 62.29 | 0.263941 |  |
| GO:0009792\_embryonic\_development\_ending\_in\_birth\_or\_egg\_hatching | DLL1 | 368 | 8 | 1.888843 | -1.241986 | 236 | 62.29 | 0.263941 |  |
| GO:0009792\_embryonic\_development\_ending\_in\_birth\_or\_egg\_hatching | FOXC1 | 368 | 8 | 1.888843 | -1.241986 | 236 | 62.29 | 0.263941 |  |
| GO:0009792\_embryonic\_development\_ending\_in\_birth\_or\_egg\_hatching | CITED2 | 368 | 8 | 1.888843 | -1.241986 | 236 | 62.29 | 0.263941 |  |
| GO:0010720\_positive\_regulation\_of\_cell\_development | XRCC4 | 34 | 2 | 5.110988 | -1.238894 | 237 | 63.27 | 0.266962 |  |
| GO:0010720\_positive\_regulation\_of\_cell\_development | BMP2 | 34 | 2 | 5.110988 | -1.238894 | 237 | 63.27 | 0.266962 |  |
| GO:0060537\_muscle\_tissue\_development | SCHIP1 | 128 | 4 | 2.715212 | -1.232048 | 238 | 63.62 | 0.267311 |  |
| GO:0060537\_muscle\_tissue\_development | RBP4 | 128 | 4 | 2.715212 | -1.232048 | 238 | 63.62 | 0.267311 |  |
| GO:0060537\_muscle\_tissue\_development | TBX5 | 128 | 4 | 2.715212 | -1.232048 | 238 | 63.62 | 0.267311 |  |
| GO:0060537\_muscle\_tissue\_development | PROX1 | 128 | 4 | 2.715212 | -1.232048 | 238 | 63.62 | 0.267311 |  |
| GO:0006355\_regulation\_of\_transcription\_\_DNA-dependent | EGR1 | 575 | 11 | 1.662182 | -1.226881 | 239 | 63.96 | 0.267615 |  |
| GO:0006355\_regulation\_of\_transcription\_\_DNA-dependent | BMP2 | 575 | 11 | 1.662182 | -1.226881 | 239 | 63.96 | 0.267615 |  |
| GO:0006355\_regulation\_of\_transcription\_\_DNA-dependent | KLF12 | 575 | 11 | 1.662182 | -1.226881 | 239 | 63.96 | 0.267615 |  |
| GO:0006355\_regulation\_of\_transcription\_\_DNA-dependent | JUN | 575 | 11 | 1.662182 | -1.226881 | 239 | 63.96 | 0.267615 |  |
| GO:0006355\_regulation\_of\_transcription\_\_DNA-dependent | TBX5 | 575 | 11 | 1.662182 | -1.226881 | 239 | 63.96 | 0.267615 |  |
| GO:0006355\_regulation\_of\_transcription\_\_DNA-dependent | FOXC1 | 575 | 11 | 1.662182 | -1.226881 | 239 | 63.96 | 0.267615 |  |
| GO:0006355\_regulation\_of\_transcription\_\_DNA-dependent | RORA | 575 | 11 | 1.662182 | -1.226881 | 239 | 63.96 | 0.267615 |  |
| GO:0006355\_regulation\_of\_transcription\_\_DNA-dependent | HBA-X | 575 | 11 | 1.662182 | -1.226881 | 239 | 63.96 | 0.267615 |  |
| GO:0006355\_regulation\_of\_transcription\_\_DNA-dependent | FOXP4 | 575 | 11 | 1.662182 | -1.226881 | 239 | 63.96 | 0.267615 |  |
| GO:0006355\_regulation\_of\_transcription\_\_DNA-dependent | CITED2 | 575 | 11 | 1.662182 | -1.226881 | 239 | 63.96 | 0.267615 |  |
| GO:0006355\_regulation\_of\_transcription\_\_DNA-dependent | CTNNB1 | 575 | 11 | 1.662182 | -1.226881 | 239 | 63.96 | 0.267615 |  |
| GO:0010557\_positive\_regulation\_of\_macromolecule\_biosynthetic\_process | BMP2 | 371 | 8 | 1.873570 | -1.225034 | 240 | 64.05 | 0.266875 |  |
| GO:0010557\_positive\_regulation\_of\_macromolecule\_biosynthetic\_process | KLF12 | 371 | 8 | 1.873570 | -1.225034 | 240 | 64.05 | 0.266875 |  |
| GO:0010557\_positive\_regulation\_of\_macromolecule\_biosynthetic\_process | JUN | 371 | 8 | 1.873570 | -1.225034 | 240 | 64.05 | 0.266875 |  |
| GO:0010557\_positive\_regulation\_of\_macromolecule\_biosynthetic\_process | TBX5 | 371 | 8 | 1.873570 | -1.225034 | 240 | 64.05 | 0.266875 |  |
| GO:0010557\_positive\_regulation\_of\_macromolecule\_biosynthetic\_process | FOXC1 | 371 | 8 | 1.873570 | -1.225034 | 240 | 64.05 | 0.266875 |  |
| GO:0010557\_positive\_regulation\_of\_macromolecule\_biosynthetic\_process | RORA | 371 | 8 | 1.873570 | -1.225034 | 240 | 64.05 | 0.266875 |  |
| GO:0010557\_positive\_regulation\_of\_macromolecule\_biosynthetic\_process | CTNNB1 | 371 | 8 | 1.873570 | -1.225034 | 240 | 64.05 | 0.266875 |  |
| GO:0010557\_positive\_regulation\_of\_macromolecule\_biosynthetic\_process | CITED2 | 371 | 8 | 1.873570 | -1.225034 | 240 | 64.05 | 0.266875 |  |
| GO:0007154\_cell\_communication | RHOJ | 1096 | 18 | 1.426973 | -1.219198 | 241 | 64.48 | 0.267552 |  |
| GO:0007154\_cell\_communication | WNT5A | 1096 | 18 | 1.426973 | -1.219198 | 241 | 64.48 | 0.267552 |  |
| GO:0007154\_cell\_communication | SCHIP1 | 1096 | 18 | 1.426973 | -1.219198 | 241 | 64.48 | 0.267552 |  |
| GO:0007154\_cell\_communication | BMP2 | 1096 | 18 | 1.426973 | -1.219198 | 241 | 64.48 | 0.267552 |  |
| GO:0007154\_cell\_communication | VAV3 | 1096 | 18 | 1.426973 | -1.219198 | 241 | 64.48 | 0.267552 |  |
| GO:0007154\_cell\_communication | PLEK | 1096 | 18 | 1.426973 | -1.219198 | 241 | 64.48 | 0.267552 |  |
| GO:0007154\_cell\_communication | PRRX1 | 1096 | 18 | 1.426973 | -1.219198 | 241 | 64.48 | 0.267552 |  |
| GO:0007154\_cell\_communication | ZEB2 | 1096 | 18 | 1.426973 | -1.219198 | 241 | 64.48 | 0.267552 |  |
| GO:0007154\_cell\_communication | PRKG1 | 1096 | 18 | 1.426973 | -1.219198 | 241 | 64.48 | 0.267552 |  |
| GO:0007154\_cell\_communication | CTNNB1 | 1096 | 18 | 1.426973 | -1.219198 | 241 | 64.48 | 0.267552 |  |
| GO:0007154\_cell\_communication | CITED2 | 1096 | 18 | 1.426973 | -1.219198 | 241 | 64.48 | 0.267552 |  |
| GO:0007154\_cell\_communication | TGFB2 | 1096 | 18 | 1.426973 | -1.219198 | 241 | 64.48 | 0.267552 |  |
| GO:0007154\_cell\_communication | FZD10 | 1096 | 18 | 1.426973 | -1.219198 | 241 | 64.48 | 0.267552 |  |
| GO:0007154\_cell\_communication | RB1CC1 | 1096 | 18 | 1.426973 | -1.219198 | 241 | 64.48 | 0.267552 |  |
| GO:0007154\_cell\_communication | RSPO3 | 1096 | 18 | 1.426973 | -1.219198 | 241 | 64.48 | 0.267552 |  |
| GO:0007154\_cell\_communication | FOXC1 | 1096 | 18 | 1.426973 | -1.219198 | 241 | 64.48 | 0.267552 |  |
| GO:0007154\_cell\_communication | CDC42EP4 | 1096 | 18 | 1.426973 | -1.219198 | 241 | 64.48 | 0.267552 |  |
| GO:0007154\_cell\_communication | USP14 | 1096 | 18 | 1.426973 | -1.219198 | 241 | 64.48 | 0.267552 |  |
| GO:0006464\_protein\_modification\_process | WNT5A | 439 | 9 | 1.781278 | -1.217541 | 242 | 64.53 | 0.266653 |  |
| GO:0006464\_protein\_modification\_process | A930038C07RIK | 439 | 9 | 1.781278 | -1.217541 | 242 | 64.53 | 0.266653 |  |
| GO:0006464\_protein\_modification\_process | BTG2 | 439 | 9 | 1.781278 | -1.217541 | 242 | 64.53 | 0.266653 |  |
| GO:0006464\_protein\_modification\_process | RB1CC1 | 439 | 9 | 1.781278 | -1.217541 | 242 | 64.53 | 0.266653 |  |
| GO:0006464\_protein\_modification\_process | JUN | 439 | 9 | 1.781278 | -1.217541 | 242 | 64.53 | 0.266653 |  |
| GO:0006464\_protein\_modification\_process | ZEB2 | 439 | 9 | 1.781278 | -1.217541 | 242 | 64.53 | 0.266653 |  |
| GO:0006464\_protein\_modification\_process | DCN | 439 | 9 | 1.781278 | -1.217541 | 242 | 64.53 | 0.266653 |  |
| GO:0006464\_protein\_modification\_process | PRKG1 | 439 | 9 | 1.781278 | -1.217541 | 242 | 64.53 | 0.266653 |  |
| GO:0006464\_protein\_modification\_process | TGFB2 | 439 | 9 | 1.781278 | -1.217541 | 242 | 64.53 | 0.266653 |  |
| GO:0010810\_regulation\_of\_cell-substrate\_adhesion | A930038C07RIK | 35 | 2 | 4.964960 | -1.216477 | 243 | 65.24 | 0.268477 |  |
| GO:0010810\_regulation\_of\_cell-substrate\_adhesion | CCDC80 | 35 | 2 | 4.964960 | -1.216477 | 243 | 65.24 | 0.268477 |  |
| GO:0045165\_cell\_fate\_commitment | BMP2 | 130 | 4 | 2.673440 | -1.212005 | 244 | 65.69 | 0.269221 |  |
| GO:0045165\_cell\_fate\_commitment | PROX1 | 130 | 4 | 2.673440 | -1.212005 | 244 | 65.69 | 0.269221 |  |
| GO:0045165\_cell\_fate\_commitment | TGFB2 | 130 | 4 | 2.673440 | -1.212005 | 244 | 65.69 | 0.269221 |  |
| GO:0045165\_cell\_fate\_commitment | CTNNB1 | 130 | 4 | 2.673440 | -1.212005 | 244 | 65.69 | 0.269221 |  |
| GO:0001704\_formation\_of\_primary\_germ\_layer | FOXC1 | 36 | 2 | 4.827044 | -1.194788 | 248 | 67.38 | 0.271694 |  |
| GO:0001704\_formation\_of\_primary\_germ\_layer | CTNNB1 | 36 | 2 | 4.827044 | -1.194788 | 248 | 67.38 | 0.271694 |  |
| GO:0001889\_liver\_development | RB1CC1 | 36 | 2 | 4.827044 | -1.194788 | 248 | 67.38 | 0.271694 |  |
| GO:0001889\_liver\_development | CITED2 | 36 | 2 | 4.827044 | -1.194788 | 248 | 67.38 | 0.271694 |  |
| GO:0007368\_determination\_of\_left\_right\_symmetry | DLL1 | 36 | 2 | 4.827044 | -1.194788 | 248 | 67.38 | 0.271694 |  |
| GO:0007368\_determination\_of\_left\_right\_symmetry | CITED2 | 36 | 2 | 4.827044 | -1.194788 | 248 | 67.38 | 0.271694 |  |
| GO:0030278\_regulation\_of\_ossification | BMP2 | 36 | 2 | 4.827044 | -1.194788 | 248 | 67.38 | 0.271694 |  |
| GO:0030278\_regulation\_of\_ossification | CTNNB1 | 36 | 2 | 4.827044 | -1.194788 | 248 | 67.38 | 0.271694 |  |
| GO:0021700\_developmental\_maturation | SEPT4 | 81 | 3 | 3.218029 | -1.183269 | 249 | 67.77 | 0.272169 |  |
| GO:0021700\_developmental\_maturation | HBA-X | 81 | 3 | 3.218029 | -1.183269 | 249 | 67.77 | 0.272169 |  |
| GO:0021700\_developmental\_maturation | CTNNB1 | 81 | 3 | 3.218029 | -1.183269 | 249 | 67.77 | 0.272169 |  |
| GO:0009799\_determination\_of\_symmetry | DLL1 | 37 | 2 | 4.696583 | -1.173786 | 252 | 68.91 | 0.273452 |  |
| GO:0009799\_determination\_of\_symmetry | CITED2 | 37 | 2 | 4.696583 | -1.173786 | 252 | 68.91 | 0.273452 |  |
| GO:0009855\_determination\_of\_bilateral\_symmetry | DLL1 | 37 | 2 | 4.696583 | -1.173786 | 252 | 68.91 | 0.273452 |  |
| GO:0009855\_determination\_of\_bilateral\_symmetry | CITED2 | 37 | 2 | 4.696583 | -1.173786 | 252 | 68.91 | 0.273452 |  |
| GO:0050906\_detection\_of\_stimulus\_involved\_in\_sensory\_perception | RBP4 | 37 | 2 | 4.696583 | -1.173786 | 252 | 68.91 | 0.273452 |  |
| GO:0050906\_detection\_of\_stimulus\_involved\_in\_sensory\_perception | RGS9BP | 37 | 2 | 4.696583 | -1.173786 | 252 | 68.91 | 0.273452 |  |
| GO:0006706\_steroid\_catabolic\_process | APOE | 6 | 1 | 14.481132 | -1.173057 | 268 | 81.39 | 0.303694 |  |
| GO:0007442\_hindgut\_morphogenesis | WNT5A | 6 | 1 | 14.481132 | -1.173057 | 268 | 81.39 | 0.303694 |  |
| GO:0022409\_positive\_regulation\_of\_cell-cell\_adhesion | CITED2 | 6 | 1 | 14.481132 | -1.173057 | 268 | 81.39 | 0.303694 |  |
| GO:0032331\_negative\_regulation\_of\_chondrocyte\_differentiation | CTNNB1 | 6 | 1 | 14.481132 | -1.173057 | 268 | 81.39 | 0.303694 |  |
| GO:0045778\_positive\_regulation\_of\_ossification | BMP2 | 6 | 1 | 14.481132 | -1.173057 | 268 | 81.39 | 0.303694 |  |
| GO:0046852\_positive\_regulation\_of\_bone\_remodeling | BMP2 | 6 | 1 | 14.481132 | -1.173057 | 268 | 81.39 | 0.303694 |  |
| GO:0048103\_somatic\_stem\_cell\_division | TGFB2 | 6 | 1 | 14.481132 | -1.173057 | 268 | 81.39 | 0.303694 |  |
| GO:0048340\_paraxial\_mesoderm\_morphogenesis | FOXC1 | 6 | 1 | 14.481132 | -1.173057 | 268 | 81.39 | 0.303694 |  |
| GO:0048617\_embryonic\_foregut\_morphogenesis | FOXP4 | 6 | 1 | 14.481132 | -1.173057 | 268 | 81.39 | 0.303694 |  |
| GO:0048853\_forebrain\_morphogenesis | WNT5A | 6 | 1 | 14.481132 | -1.173057 | 268 | 81.39 | 0.303694 |  |
| GO:0051180\_vitamin\_transport | RBP4 | 6 | 1 | 14.481132 | -1.173057 | 268 | 81.39 | 0.303694 |  |
| GO:0060068\_vagina\_development | RBP4 | 6 | 1 | 14.481132 | -1.173057 | 268 | 81.39 | 0.303694 |  |
| GO:0060136\_embryonic\_process\_involved\_in\_female\_pregnancy | CITED2 | 6 | 1 | 14.481132 | -1.173057 | 268 | 81.39 | 0.303694 |  |
| GO:0060389\_pathway-restricted\_SMAD\_protein\_phosphorylation | TGFB2 | 6 | 1 | 14.481132 | -1.173057 | 268 | 81.39 | 0.303694 |  |
| GO:0060411\_heart\_septum\_morphogenesis | PROX1 | 6 | 1 | 14.481132 | -1.173057 | 268 | 81.39 | 0.303694 |  |
| GO:0060685\_regulation\_of\_prostatic\_bud\_formation | WNT5A | 6 | 1 | 14.481132 | -1.173057 | 268 | 81.39 | 0.303694 |  |
| GO:0051252\_regulation\_of\_RNA\_metabolic\_process | EGR1 | 590 | 11 | 1.619923 | -1.160971 | 269 | 82.11 | 0.305242 |  |
| GO:0051252\_regulation\_of\_RNA\_metabolic\_process | BMP2 | 590 | 11 | 1.619923 | -1.160971 | 269 | 82.11 | 0.305242 |  |
| GO:0051252\_regulation\_of\_RNA\_metabolic\_process | KLF12 | 590 | 11 | 1.619923 | -1.160971 | 269 | 82.11 | 0.305242 |  |
| GO:0051252\_regulation\_of\_RNA\_metabolic\_process | JUN | 590 | 11 | 1.619923 | -1.160971 | 269 | 82.11 | 0.305242 |  |
| GO:0051252\_regulation\_of\_RNA\_metabolic\_process | TBX5 | 590 | 11 | 1.619923 | -1.160971 | 269 | 82.11 | 0.305242 |  |
| GO:0051252\_regulation\_of\_RNA\_metabolic\_process | FOXC1 | 590 | 11 | 1.619923 | -1.160971 | 269 | 82.11 | 0.305242 |  |
| GO:0051252\_regulation\_of\_RNA\_metabolic\_process | RORA | 590 | 11 | 1.619923 | -1.160971 | 269 | 82.11 | 0.305242 |  |
| GO:0051252\_regulation\_of\_RNA\_metabolic\_process | HBA-X | 590 | 11 | 1.619923 | -1.160971 | 269 | 82.11 | 0.305242 |  |
| GO:0051252\_regulation\_of\_RNA\_metabolic\_process | FOXP4 | 590 | 11 | 1.619923 | -1.160971 | 269 | 82.11 | 0.305242 |  |
| GO:0051252\_regulation\_of\_RNA\_metabolic\_process | CITED2 | 590 | 11 | 1.619923 | -1.160971 | 269 | 82.11 | 0.305242 |  |
| GO:0051252\_regulation\_of\_RNA\_metabolic\_process | CTNNB1 | 590 | 11 | 1.619923 | -1.160971 | 269 | 82.11 | 0.305242 |  |
| GO:0001654\_eye\_development | RBP4 | 136 | 4 | 2.555494 | -1.154323 | 270 | 82.54 | 0.305704 |  |
| GO:0001654\_eye\_development | FOXC1 | 136 | 4 | 2.555494 | -1.154323 | 270 | 82.54 | 0.305704 |  |
| GO:0001654\_eye\_development | PROX1 | 136 | 4 | 2.555494 | -1.154323 | 270 | 82.54 | 0.305704 |  |
| GO:0001654\_eye\_development | CTNNB1 | 136 | 4 | 2.555494 | -1.154323 | 270 | 82.54 | 0.305704 |  |
| GO:0001649\_osteoblast\_differentiation | BMP2 | 38 | 2 | 4.572989 | -1.153434 | 272 | 83.86 | 0.308309 |  |
| GO:0001649\_osteoblast\_differentiation | CTNNB1 | 38 | 2 | 4.572989 | -1.153434 | 272 | 83.86 | 0.308309 |  |
| GO:0001657\_ureteric\_bud\_development | BMP2 | 38 | 2 | 4.572989 | -1.153434 | 272 | 83.86 | 0.308309 |  |
| GO:0001657\_ureteric\_bud\_development | FOXC1 | 38 | 2 | 4.572989 | -1.153434 | 272 | 83.86 | 0.308309 |  |
| GO:0045137\_development\_of\_primary\_sexual\_characteristics | WNT5A | 84 | 3 | 3.103100 | -1.145596 | 273 | 84.14 | 0.308205 |  |
| GO:0045137\_development\_of\_primary\_sexual\_characteristics | RBP4 | 84 | 3 | 3.103100 | -1.145596 | 273 | 84.14 | 0.308205 |  |
| GO:0045137\_development\_of\_primary\_sexual\_characteristics | FOXC1 | 84 | 3 | 3.103100 | -1.145596 | 273 | 84.14 | 0.308205 |  |
| GO:0006351\_transcription\_\_DNA-dependent | EGR1 | 594 | 11 | 1.609015 | -1.143938 | 274 | 84.28 | 0.307591 |  |
| GO:0006351\_transcription\_\_DNA-dependent | BMP2 | 594 | 11 | 1.609015 | -1.143938 | 274 | 84.28 | 0.307591 |  |
| GO:0006351\_transcription\_\_DNA-dependent | KLF12 | 594 | 11 | 1.609015 | -1.143938 | 274 | 84.28 | 0.307591 |  |
| GO:0006351\_transcription\_\_DNA-dependent | JUN | 594 | 11 | 1.609015 | -1.143938 | 274 | 84.28 | 0.307591 |  |
| GO:0006351\_transcription\_\_DNA-dependent | TBX5 | 594 | 11 | 1.609015 | -1.143938 | 274 | 84.28 | 0.307591 |  |
| GO:0006351\_transcription\_\_DNA-dependent | FOXC1 | 594 | 11 | 1.609015 | -1.143938 | 274 | 84.28 | 0.307591 |  |
| GO:0006351\_transcription\_\_DNA-dependent | RORA | 594 | 11 | 1.609015 | -1.143938 | 274 | 84.28 | 0.307591 |  |
| GO:0006351\_transcription\_\_DNA-dependent | HBA-X | 594 | 11 | 1.609015 | -1.143938 | 274 | 84.28 | 0.307591 |  |
| GO:0006351\_transcription\_\_DNA-dependent | FOXP4 | 594 | 11 | 1.609015 | -1.143938 | 274 | 84.28 | 0.307591 |  |
| GO:0006351\_transcription\_\_DNA-dependent | CITED2 | 594 | 11 | 1.609015 | -1.143938 | 274 | 84.28 | 0.307591 |  |
| GO:0006351\_transcription\_\_DNA-dependent | CTNNB1 | 594 | 11 | 1.609015 | -1.143938 | 274 | 84.28 | 0.307591 |  |
| GO:0003002\_regionalization | BTG2 | 195 | 5 | 2.227866 | -1.142928 | 275 | 84.39 | 0.306873 |  |
| GO:0003002\_regionalization | FOXC1 | 195 | 5 | 2.227866 | -1.142928 | 275 | 84.39 | 0.306873 |  |
| GO:0003002\_regionalization | ZEB2 | 195 | 5 | 2.227866 | -1.142928 | 275 | 84.39 | 0.306873 |  |
| GO:0003002\_regionalization | DLL1 | 195 | 5 | 2.227866 | -1.142928 | 275 | 84.39 | 0.306873 |  |
| GO:0003002\_regionalization | CTNNB1 | 195 | 5 | 2.227866 | -1.142928 | 275 | 84.39 | 0.306873 |  |
| GO:0032774\_RNA\_biosynthetic\_process | EGR1 | 595 | 11 | 1.606310 | -1.139715 | 276 | 84.41 | 0.305833 |  |
| GO:0032774\_RNA\_biosynthetic\_process | BMP2 | 595 | 11 | 1.606310 | -1.139715 | 276 | 84.41 | 0.305833 |  |
| GO:0032774\_RNA\_biosynthetic\_process | KLF12 | 595 | 11 | 1.606310 | -1.139715 | 276 | 84.41 | 0.305833 |  |
| GO:0032774\_RNA\_biosynthetic\_process | JUN | 595 | 11 | 1.606310 | -1.139715 | 276 | 84.41 | 0.305833 |  |
| GO:0032774\_RNA\_biosynthetic\_process | TBX5 | 595 | 11 | 1.606310 | -1.139715 | 276 | 84.41 | 0.305833 |  |
| GO:0032774\_RNA\_biosynthetic\_process | FOXC1 | 595 | 11 | 1.606310 | -1.139715 | 276 | 84.41 | 0.305833 |  |
| GO:0032774\_RNA\_biosynthetic\_process | RORA | 595 | 11 | 1.606310 | -1.139715 | 276 | 84.41 | 0.305833 |  |
| GO:0032774\_RNA\_biosynthetic\_process | HBA-X | 595 | 11 | 1.606310 | -1.139715 | 276 | 84.41 | 0.305833 |  |
| GO:0032774\_RNA\_biosynthetic\_process | FOXP4 | 595 | 11 | 1.606310 | -1.139715 | 276 | 84.41 | 0.305833 |  |
| GO:0032774\_RNA\_biosynthetic\_process | CITED2 | 595 | 11 | 1.606310 | -1.139715 | 276 | 84.41 | 0.305833 |  |
| GO:0032774\_RNA\_biosynthetic\_process | CTNNB1 | 595 | 11 | 1.606310 | -1.139715 | 276 | 84.41 | 0.305833 |  |
| GO:0042475\_odontogenesis\_of\_dentine-containing\_tooth | BMP2 | 39 | 2 | 4.455733 | -1.133697 | 277 | 85.66 | 0.309242 |  |
| GO:0042475\_odontogenesis\_of\_dentine-containing\_tooth | CTNNB1 | 39 | 2 | 4.455733 | -1.133697 | 277 | 85.66 | 0.309242 |  |
| GO:0043412\_biopolymer\_modification | WNT5A | 458 | 9 | 1.707382 | -1.123791 | 278 | 86.28 | 0.310360 |  |
| GO:0043412\_biopolymer\_modification | A930038C07RIK | 458 | 9 | 1.707382 | -1.123791 | 278 | 86.28 | 0.310360 |  |
| GO:0043412\_biopolymer\_modification | BTG2 | 458 | 9 | 1.707382 | -1.123791 | 278 | 86.28 | 0.310360 |  |
| GO:0043412\_biopolymer\_modification | RB1CC1 | 458 | 9 | 1.707382 | -1.123791 | 278 | 86.28 | 0.310360 |  |
| GO:0043412\_biopolymer\_modification | JUN | 458 | 9 | 1.707382 | -1.123791 | 278 | 86.28 | 0.310360 |  |
| GO:0043412\_biopolymer\_modification | ZEB2 | 458 | 9 | 1.707382 | -1.123791 | 278 | 86.28 | 0.310360 |  |
| GO:0043412\_biopolymer\_modification | DCN | 458 | 9 | 1.707382 | -1.123791 | 278 | 86.28 | 0.310360 |  |
| GO:0043412\_biopolymer\_modification | PRKG1 | 458 | 9 | 1.707382 | -1.123791 | 278 | 86.28 | 0.310360 |  |
| GO:0043412\_biopolymer\_modification | TGFB2 | 458 | 9 | 1.707382 | -1.123791 | 278 | 86.28 | 0.310360 |  |
| GO:0008203\_cholesterol\_metabolic\_process | APOE | 40 | 2 | 4.344340 | -1.114545 | 280 | 87.76 | 0.313429 |  |
| GO:0008203\_cholesterol\_metabolic\_process | CFTR | 40 | 2 | 4.344340 | -1.114545 | 280 | 87.76 | 0.313429 |  |
| GO:0046850\_regulation\_of\_bone\_remodeling | BMP2 | 40 | 2 | 4.344340 | -1.114545 | 280 | 87.76 | 0.313429 |  |
| GO:0046850\_regulation\_of\_bone\_remodeling | CTNNB1 | 40 | 2 | 4.344340 | -1.114545 | 280 | 87.76 | 0.313429 |  |
| GO:0001822\_kidney\_development | SCHIP1 | 87 | 3 | 2.996096 | -1.109611 | 282 | 88.35 | 0.313298 |  |
| GO:0001822\_kidney\_development | BMP2 | 87 | 3 | 2.996096 | -1.109611 | 282 | 88.35 | 0.313298 |  |
| GO:0001822\_kidney\_development | FOXC1 | 87 | 3 | 2.996096 | -1.109611 | 282 | 88.35 | 0.313298 |  |
| GO:0007178\_transmembrane\_receptor\_protein\_serine\_threonine\_kinase\_signaling\_pathway | BMP2 | 87 | 3 | 2.996096 | -1.109611 | 282 | 88.35 | 0.313298 |  |
| GO:0007178\_transmembrane\_receptor\_protein\_serine\_threonine\_kinase\_signaling\_pathway | TGFB2 | 87 | 3 | 2.996096 | -1.109611 | 282 | 88.35 | 0.313298 |  |
| GO:0007178\_transmembrane\_receptor\_protein\_serine\_threonine\_kinase\_signaling\_pathway | CITED2 | 87 | 3 | 2.996096 | -1.109611 | 282 | 88.35 | 0.313298 |  |
| GO:0003006\_reproductive\_developmental\_process | WNT5A | 141 | 4 | 2.464874 | -1.108880 | 283 | 88.53 | 0.312827 |  |
| GO:0003006\_reproductive\_developmental\_process | RBP4 | 141 | 4 | 2.464874 | -1.108880 | 283 | 88.53 | 0.312827 |  |
| GO:0003006\_reproductive\_developmental\_process | FOXC1 | 141 | 4 | 2.464874 | -1.108880 | 283 | 88.53 | 0.312827 |  |
| GO:0003006\_reproductive\_developmental\_process | CTNNB1 | 141 | 4 | 2.464874 | -1.108880 | 283 | 88.53 | 0.312827 |  |
| GO:0006818\_hydrogen\_transport | ATP6V1E1 | 7 | 1 | 12.412399 | -1.108548 | 294 | 102.76 | 0.349524 |  |
| GO:0007440\_foregut\_morphogenesis | FOXP4 | 7 | 1 | 12.412399 | -1.108548 | 294 | 102.76 | 0.349524 |  |
| GO:0015992\_proton\_transport | ATP6V1E1 | 7 | 1 | 12.412399 | -1.108548 | 294 | 102.76 | 0.349524 |  |
| GO:0022407\_regulation\_of\_cell-cell\_adhesion | CITED2 | 7 | 1 | 12.412399 | -1.108548 | 294 | 102.76 | 0.349524 |  |
| GO:0042572\_retinol\_metabolic\_process | RBP4 | 7 | 1 | 12.412399 | -1.108548 | 294 | 102.76 | 0.349524 |  |
| GO:0043507\_positive\_regulation\_of\_JUN\_kinase\_activity | ZEB2 | 7 | 1 | 12.412399 | -1.108548 | 294 | 102.76 | 0.349524 |  |
| GO:0045880\_positive\_regulation\_of\_smoothened\_signaling\_pathway | PRRX1 | 7 | 1 | 12.412399 | -1.108548 | 294 | 102.76 | 0.349524 |  |
| GO:0046543\_development\_of\_secondary\_female\_sexual\_characteristics | WNT5A | 7 | 1 | 12.412399 | -1.108548 | 294 | 102.76 | 0.349524 |  |
| GO:0046622\_positive\_regulation\_of\_organ\_growth | PROX1 | 7 | 1 | 12.412399 | -1.108548 | 294 | 102.76 | 0.349524 |  |
| GO:0060788\_ectodermal\_placode\_formation | PROX1 | 7 | 1 | 12.412399 | -1.108548 | 294 | 102.76 | 0.349524 |  |
| GO:0060795\_cell\_fate\_commitment\_involved\_in\_the\_formation\_of\_primary\_germ\_layers | CTNNB1 | 7 | 1 | 12.412399 | -1.108548 | 294 | 102.76 | 0.349524 |  |
| GO:0042127\_regulation\_of\_cell\_proliferation | WNT5A | 393 | 8 | 1.768688 | -1.107524 | 295 | 102.81 | 0.348508 |  |
| GO:0042127\_regulation\_of\_cell\_proliferation | RBP4 | 393 | 8 | 1.768688 | -1.107524 | 295 | 102.81 | 0.348508 |  |
| GO:0042127\_regulation\_of\_cell\_proliferation | XRCC4 | 393 | 8 | 1.768688 | -1.107524 | 295 | 102.81 | 0.348508 |  |
| GO:0042127\_regulation\_of\_cell\_proliferation | BMP2 | 393 | 8 | 1.768688 | -1.107524 | 295 | 102.81 | 0.348508 |  |
| GO:0042127\_regulation\_of\_cell\_proliferation | JUN | 393 | 8 | 1.768688 | -1.107524 | 295 | 102.81 | 0.348508 |  |
| GO:0042127\_regulation\_of\_cell\_proliferation | PRRX1 | 393 | 8 | 1.768688 | -1.107524 | 295 | 102.81 | 0.348508 |  |
| GO:0042127\_regulation\_of\_cell\_proliferation | PROX1 | 393 | 8 | 1.768688 | -1.107524 | 295 | 102.81 | 0.348508 |  |
| GO:0042127\_regulation\_of\_cell\_proliferation | CTNNB1 | 393 | 8 | 1.768688 | -1.107524 | 295 | 102.81 | 0.348508 |  |
| GO:0045449\_regulation\_of\_transcription | EGR1 | 676 | 12 | 1.542369 | -1.104571 | 296 | 102.95 | 0.347804 |  |
| GO:0045449\_regulation\_of\_transcription | BMP2 | 676 | 12 | 1.542369 | -1.104571 | 296 | 102.95 | 0.347804 |  |
| GO:0045449\_regulation\_of\_transcription | KLF12 | 676 | 12 | 1.542369 | -1.104571 | 296 | 102.95 | 0.347804 |  |
| GO:0045449\_regulation\_of\_transcription | JUN | 676 | 12 | 1.542369 | -1.104571 | 296 | 102.95 | 0.347804 |  |
| GO:0045449\_regulation\_of\_transcription | TBX5 | 676 | 12 | 1.542369 | -1.104571 | 296 | 102.95 | 0.347804 |  |
| GO:0045449\_regulation\_of\_transcription | FOXC1 | 676 | 12 | 1.542369 | -1.104571 | 296 | 102.95 | 0.347804 |  |
| GO:0045449\_regulation\_of\_transcription | RORA | 676 | 12 | 1.542369 | -1.104571 | 296 | 102.95 | 0.347804 |  |
| GO:0045449\_regulation\_of\_transcription | HBA-X | 676 | 12 | 1.542369 | -1.104571 | 296 | 102.95 | 0.347804 |  |
| GO:0045449\_regulation\_of\_transcription | FOXP4 | 676 | 12 | 1.542369 | -1.104571 | 296 | 102.95 | 0.347804 |  |
| GO:0045449\_regulation\_of\_transcription | C1D | 676 | 12 | 1.542369 | -1.104571 | 296 | 102.95 | 0.347804 |  |
| GO:0045449\_regulation\_of\_transcription | CITED2 | 676 | 12 | 1.542369 | -1.104571 | 296 | 102.95 | 0.347804 |  |
| GO:0045449\_regulation\_of\_transcription | CTNNB1 | 676 | 12 | 1.542369 | -1.104571 | 296 | 102.95 | 0.347804 |  |
| GO:0001503\_ossification | BMP2 | 88 | 3 | 2.962050 | -1.097970 | 298 | 103.27 | 0.346544 |  |
| GO:0001503\_ossification | FOXC1 | 88 | 3 | 2.962050 | -1.097970 | 298 | 103.27 | 0.346544 |  |
| GO:0001503\_ossification | CTNNB1 | 88 | 3 | 2.962050 | -1.097970 | 298 | 103.27 | 0.346544 |  |
| GO:0048754\_branching\_morphogenesis\_of\_a\_tube | WNT5A | 88 | 3 | 2.962050 | -1.097970 | 298 | 103.27 | 0.346544 |  |
| GO:0048754\_branching\_morphogenesis\_of\_a\_tube | BMP2 | 88 | 3 | 2.962050 | -1.097970 | 298 | 103.27 | 0.346544 |  |
| GO:0048754\_branching\_morphogenesis\_of\_a\_tube | CTNNB1 | 88 | 3 | 2.962050 | -1.097970 | 298 | 103.27 | 0.346544 |  |
| GO:0010551\_regulation\_of\_specific\_transcription\_from\_RNA\_polymerase\_II\_promoter | BMP2 | 41 | 2 | 4.238380 | -1.095946 | 302 | 104.72 | 0.346755 |  |
| GO:0010551\_regulation\_of\_specific\_transcription\_from\_RNA\_polymerase\_II\_promoter | CITED2 | 41 | 2 | 4.238380 | -1.095946 | 302 | 104.72 | 0.346755 |  |
| GO:0019216\_regulation\_of\_lipid\_metabolic\_process | VAV3 | 41 | 2 | 4.238380 | -1.095946 | 302 | 104.72 | 0.346755 |  |
| GO:0019216\_regulation\_of\_lipid\_metabolic\_process | APOE | 41 | 2 | 4.238380 | -1.095946 | 302 | 104.72 | 0.346755 |  |
| GO:0032569\_specific\_transcription\_from\_RNA\_polymerase\_II\_promoter | BMP2 | 41 | 2 | 4.238380 | -1.095946 | 302 | 104.72 | 0.346755 |  |
| GO:0032569\_specific\_transcription\_from\_RNA\_polymerase\_II\_promoter | CITED2 | 41 | 2 | 4.238380 | -1.095946 | 302 | 104.72 | 0.346755 |  |
| GO:0033077\_T\_cell\_differentiation\_in\_the\_thymus | XRCC4 | 41 | 2 | 4.238380 | -1.095946 | 302 | 104.72 | 0.346755 |  |
| GO:0033077\_T\_cell\_differentiation\_in\_the\_thymus | CTNNB1 | 41 | 2 | 4.238380 | -1.095946 | 302 | 104.72 | 0.346755 |  |
| GO:0035239\_tube\_morphogenesis | WNT5A | 143 | 4 | 2.430400 | -1.091328 | 303 | 104.86 | 0.346073 |  |
| GO:0035239\_tube\_morphogenesis | BMP2 | 143 | 4 | 2.430400 | -1.091328 | 303 | 104.86 | 0.346073 |  |
| GO:0035239\_tube\_morphogenesis | ZEB2 | 143 | 4 | 2.430400 | -1.091328 | 303 | 104.86 | 0.346073 |  |
| GO:0035239\_tube\_morphogenesis | CTNNB1 | 143 | 4 | 2.430400 | -1.091328 | 303 | 104.86 | 0.346073 |  |
| GO:0016125\_sterol\_metabolic\_process | APOE | 42 | 2 | 4.137466 | -1.077875 | 305 | 106.8 | 0.350164 |  |
| GO:0016125\_sterol\_metabolic\_process | CFTR | 42 | 2 | 4.137466 | -1.077875 | 305 | 106.8 | 0.350164 |  |
| GO:0042476\_odontogenesis | BMP2 | 42 | 2 | 4.137466 | -1.077875 | 305 | 106.8 | 0.350164 |  |
| GO:0042476\_odontogenesis | CTNNB1 | 42 | 2 | 4.137466 | -1.077875 | 305 | 106.8 | 0.350164 |  |
| GO:0007243\_protein\_kinase\_cascade | WNT5A | 205 | 5 | 2.119190 | -1.070163 | 306 | 107.38 | 0.350915 |  |
| GO:0007243\_protein\_kinase\_cascade | PLEK | 205 | 5 | 2.119190 | -1.070163 | 306 | 107.38 | 0.350915 |  |
| GO:0007243\_protein\_kinase\_cascade | RB1CC1 | 205 | 5 | 2.119190 | -1.070163 | 306 | 107.38 | 0.350915 |  |
| GO:0007243\_protein\_kinase\_cascade | ZEB2 | 205 | 5 | 2.119190 | -1.070163 | 306 | 107.38 | 0.350915 |  |
| GO:0007243\_protein\_kinase\_cascade | CTNNB1 | 205 | 5 | 2.119190 | -1.070163 | 306 | 107.38 | 0.350915 |  |
| GO:0005975\_carbohydrate\_metabolic\_process | RBP4 | 146 | 4 | 2.380460 | -1.065640 | 307 | 107.56 | 0.350358 |  |
| GO:0005975\_carbohydrate\_metabolic\_process | A930038C07RIK | 146 | 4 | 2.380460 | -1.065640 | 307 | 107.56 | 0.350358 |  |
| GO:0005975\_carbohydrate\_metabolic\_process | FOXC1 | 146 | 4 | 2.380460 | -1.065640 | 307 | 107.56 | 0.350358 |  |
| GO:0005975\_carbohydrate\_metabolic\_process | DCN | 146 | 4 | 2.380460 | -1.065640 | 307 | 107.56 | 0.350358 |  |
| GO:0031399\_regulation\_of\_protein\_modification\_process | RB1CC1 | 91 | 3 | 2.864400 | -1.064055 | 308 | 107.76 | 0.349870 |  |
| GO:0031399\_regulation\_of\_protein\_modification\_process | JUN | 91 | 3 | 2.864400 | -1.064055 | 308 | 107.76 | 0.349870 |  |
| GO:0031399\_regulation\_of\_protein\_modification\_process | ZEB2 | 91 | 3 | 2.864400 | -1.064055 | 308 | 107.76 | 0.349870 |  |
| GO:0009582\_detection\_of\_abiotic\_stimulus | RBP4 | 43 | 2 | 4.041246 | -1.060306 | 310 | 109.21 | 0.352290 |  |
| GO:0009582\_detection\_of\_abiotic\_stimulus | RGS9BP | 43 | 2 | 4.041246 | -1.060306 | 310 | 109.21 | 0.352290 |  |
| GO:0010001\_glial\_cell\_differentiation | BMP2 | 43 | 2 | 4.041246 | -1.060306 | 310 | 109.21 | 0.352290 |  |
| GO:0010001\_glial\_cell\_differentiation | CTNNB1 | 43 | 2 | 4.041246 | -1.060306 | 310 | 109.21 | 0.352290 |  |
| GO:0022603\_regulation\_of\_anatomical\_structure\_morphogenesis | WNT5A | 147 | 4 | 2.364266 | -1.057243 | 311 | 109.3 | 0.351447 |  |
| GO:0022603\_regulation\_of\_anatomical\_structure\_morphogenesis | RHOJ | 147 | 4 | 2.364266 | -1.057243 | 311 | 109.3 | 0.351447 |  |
| GO:0022603\_regulation\_of\_anatomical\_structure\_morphogenesis | PROX1 | 147 | 4 | 2.364266 | -1.057243 | 311 | 109.3 | 0.351447 |  |
| GO:0022603\_regulation\_of\_anatomical\_structure\_morphogenesis | CTNNB1 | 147 | 4 | 2.364266 | -1.057243 | 311 | 109.3 | 0.351447 |  |
| GO:0006807\_nitrogen\_compound\_metabolic\_process | EGR1 | 1147 | 18 | 1.363524 | -1.056828 | 312 | 109.37 | 0.350545 |  |
| GO:0006807\_nitrogen\_compound\_metabolic\_process | SCHIP1 | 1147 | 18 | 1.363524 | -1.056828 | 312 | 109.37 | 0.350545 |  |
| GO:0006807\_nitrogen\_compound\_metabolic\_process | XRCC4 | 1147 | 18 | 1.363524 | -1.056828 | 312 | 109.37 | 0.350545 |  |
| GO:0006807\_nitrogen\_compound\_metabolic\_process | ADSSL1 | 1147 | 18 | 1.363524 | -1.056828 | 312 | 109.37 | 0.350545 |  |
| GO:0006807\_nitrogen\_compound\_metabolic\_process | BMP2 | 1147 | 18 | 1.363524 | -1.056828 | 312 | 109.37 | 0.350545 |  |
| GO:0006807\_nitrogen\_compound\_metabolic\_process | KLF12 | 1147 | 18 | 1.363524 | -1.056828 | 312 | 109.37 | 0.350545 |  |
| GO:0006807\_nitrogen\_compound\_metabolic\_process | TBX5 | 1147 | 18 | 1.363524 | -1.056828 | 312 | 109.37 | 0.350545 |  |
| GO:0006807\_nitrogen\_compound\_metabolic\_process | DCN | 1147 | 18 | 1.363524 | -1.056828 | 312 | 109.37 | 0.350545 |  |
| GO:0006807\_nitrogen\_compound\_metabolic\_process | RORA | 1147 | 18 | 1.363524 | -1.056828 | 312 | 109.37 | 0.350545 |  |
| GO:0006807\_nitrogen\_compound\_metabolic\_process | FOXP4 | 1147 | 18 | 1.363524 | -1.056828 | 312 | 109.37 | 0.350545 |  |
| GO:0006807\_nitrogen\_compound\_metabolic\_process | TGFB2 | 1147 | 18 | 1.363524 | -1.056828 | 312 | 109.37 | 0.350545 |  |
| GO:0006807\_nitrogen\_compound\_metabolic\_process | CTNNB1 | 1147 | 18 | 1.363524 | -1.056828 | 312 | 109.37 | 0.350545 |  |
| GO:0006807\_nitrogen\_compound\_metabolic\_process | CITED2 | 1147 | 18 | 1.363524 | -1.056828 | 312 | 109.37 | 0.350545 |  |
| GO:0006807\_nitrogen\_compound\_metabolic\_process | A930038C07RIK | 1147 | 18 | 1.363524 | -1.056828 | 312 | 109.37 | 0.350545 |  |
| GO:0006807\_nitrogen\_compound\_metabolic\_process | JUN | 1147 | 18 | 1.363524 | -1.056828 | 312 | 109.37 | 0.350545 |  |
| GO:0006807\_nitrogen\_compound\_metabolic\_process | FOXC1 | 1147 | 18 | 1.363524 | -1.056828 | 312 | 109.37 | 0.350545 |  |
| GO:0006807\_nitrogen\_compound\_metabolic\_process | HBA-X | 1147 | 18 | 1.363524 | -1.056828 | 312 | 109.37 | 0.350545 |  |
| GO:0006807\_nitrogen\_compound\_metabolic\_process | C1D | 1147 | 18 | 1.363524 | -1.056828 | 312 | 109.37 | 0.350545 |  |
| GO:0008283\_cell\_proliferation | WNT5A | 544 | 10 | 1.597184 | -1.053792 | 313 | 109.4 | 0.349521 |  |
| GO:0008283\_cell\_proliferation | RBP4 | 544 | 10 | 1.597184 | -1.053792 | 313 | 109.4 | 0.349521 |  |
| GO:0008283\_cell\_proliferation | XRCC4 | 544 | 10 | 1.597184 | -1.053792 | 313 | 109.4 | 0.349521 |  |
| GO:0008283\_cell\_proliferation | BMP2 | 544 | 10 | 1.597184 | -1.053792 | 313 | 109.4 | 0.349521 |  |
| GO:0008283\_cell\_proliferation | JUN | 544 | 10 | 1.597184 | -1.053792 | 313 | 109.4 | 0.349521 |  |
| GO:0008283\_cell\_proliferation | PRRX1 | 544 | 10 | 1.597184 | -1.053792 | 313 | 109.4 | 0.349521 |  |
| GO:0008283\_cell\_proliferation | FOXC1 | 544 | 10 | 1.597184 | -1.053792 | 313 | 109.4 | 0.349521 |  |
| GO:0008283\_cell\_proliferation | ZEB2 | 544 | 10 | 1.597184 | -1.053792 | 313 | 109.4 | 0.349521 |  |
| GO:0008283\_cell\_proliferation | PROX1 | 544 | 10 | 1.597184 | -1.053792 | 313 | 109.4 | 0.349521 |  |
| GO:0008283\_cell\_proliferation | CTNNB1 | 544 | 10 | 1.597184 | -1.053792 | 313 | 109.4 | 0.349521 |  |
| GO:0030217\_T\_cell\_differentiation | EGR1 | 92 | 3 | 2.833265 | -1.053074 | 314 | 109.7 | 0.349363 |  |
| GO:0030217\_T\_cell\_differentiation | XRCC4 | 92 | 3 | 2.833265 | -1.053074 | 314 | 109.7 | 0.349363 |  |
| GO:0030217\_T\_cell\_differentiation | CTNNB1 | 92 | 3 | 2.833265 | -1.053074 | 314 | 109.7 | 0.349363 |  |
| GO:0001893\_maternal\_placenta\_development | CITED2 | 8 | 1 | 10.860849 | -1.052990 | 332 | 120.78 | 0.363795 |  |
| GO:0002320\_lymphoid\_progenitor\_cell\_differentiation | XRCC4 | 8 | 1 | 10.860849 | -1.052990 | 332 | 120.78 | 0.363795 |  |
| GO:0021692\_cerebellar\_Purkinje\_cell\_layer\_morphogenesis | RORA | 8 | 1 | 10.860849 | -1.052990 | 332 | 120.78 | 0.363795 |  |
| GO:0021694\_cerebellar\_Purkinje\_cell\_layer\_formation | RORA | 8 | 1 | 10.860849 | -1.052990 | 332 | 120.78 | 0.363795 |  |
| GO:0021702\_cerebellar\_Purkinje\_cell\_differentiation | RORA | 8 | 1 | 10.860849 | -1.052990 | 332 | 120.78 | 0.363795 |  |
| GO:0021781\_glial\_cell\_fate\_commitment | CTNNB1 | 8 | 1 | 10.860849 | -1.052990 | 332 | 120.78 | 0.363795 |  |
| GO:0021799\_cerebral\_cortex\_radially\_oriented\_cell\_migration | LRP8 | 8 | 1 | 10.860849 | -1.052990 | 332 | 120.78 | 0.363795 |  |
| GO:0030193\_regulation\_of\_blood\_coagulation | PLEK | 8 | 1 | 10.860849 | -1.052990 | 332 | 120.78 | 0.363795 |  |
| GO:0030511\_positive\_regulation\_of\_transforming\_growth\_factor\_beta\_receptor\_signaling\_pathway | CITED2 | 8 | 1 | 10.860849 | -1.052990 | 332 | 120.78 | 0.363795 |  |
| GO:0031102\_neuron\_projection\_regeneration | APOE | 8 | 1 | 10.860849 | -1.052990 | 332 | 120.78 | 0.363795 |  |
| GO:0031103\_axon\_regeneration | APOE | 8 | 1 | 10.860849 | -1.052990 | 332 | 120.78 | 0.363795 |  |
| GO:0035112\_genitalia\_morphogenesis | RBP4 | 8 | 1 | 10.860849 | -1.052990 | 332 | 120.78 | 0.363795 |  |
| GO:0042423\_catecholamine\_biosynthetic\_process | TGFB2 | 8 | 1 | 10.860849 | -1.052990 | 332 | 120.78 | 0.363795 |  |
| GO:0048638\_regulation\_of\_developmental\_growth | PROX1 | 8 | 1 | 10.860849 | -1.052990 | 332 | 120.78 | 0.363795 |  |
| GO:0060043\_regulation\_of\_cardiac\_muscle\_cell\_proliferation | RBP4 | 8 | 1 | 10.860849 | -1.052990 | 332 | 120.78 | 0.363795 |  |
| GO:0060347\_heart\_trabecula\_formation | RBP4 | 8 | 1 | 10.860849 | -1.052990 | 332 | 120.78 | 0.363795 |  |
| GO:0060670\_branching\_involved\_in\_embryonic\_placenta\_morphogenesis | RSPO3 | 8 | 1 | 10.860849 | -1.052990 | 332 | 120.78 | 0.363795 |  |
| GO:0070231\_T\_cell\_apoptosis | TGFB2 | 8 | 1 | 10.860849 | -1.052990 | 332 | 120.78 | 0.363795 |  |
| GO:0002377\_immunoglobulin\_production | RBP4 | 44 | 2 | 3.949400 | -1.043216 | 334 | 122.98 | 0.368204 |  |
| GO:0002377\_immunoglobulin\_production | XRCC4 | 44 | 2 | 3.949400 | -1.043216 | 334 | 122.98 | 0.368204 |  |
| GO:0046545\_development\_of\_primary\_female\_sexual\_characteristics | RBP4 | 44 | 2 | 3.949400 | -1.043216 | 334 | 122.98 | 0.368204 |  |
| GO:0046545\_development\_of\_primary\_female\_sexual\_characteristics | FOXC1 | 44 | 2 | 3.949400 | -1.043216 | 334 | 122.98 | 0.368204 |  |
| GO:0051716\_cellular\_response\_to\_stimulus | WNT5A | 273 | 6 | 1.909600 | -1.039089 | 335 | 123.42 | 0.368418 |  |
| GO:0051716\_cellular\_response\_to\_stimulus | XRCC4 | 273 | 6 | 1.909600 | -1.039089 | 335 | 123.42 | 0.368418 |  |
| GO:0051716\_cellular\_response\_to\_stimulus | APOE | 273 | 6 | 1.909600 | -1.039089 | 335 | 123.42 | 0.368418 |  |
| GO:0051716\_cellular\_response\_to\_stimulus | RB1CC1 | 273 | 6 | 1.909600 | -1.039089 | 335 | 123.42 | 0.368418 |  |
| GO:0051716\_cellular\_response\_to\_stimulus | ZEB2 | 273 | 6 | 1.909600 | -1.039089 | 335 | 123.42 | 0.368418 |  |
| GO:0051716\_cellular\_response\_to\_stimulus | TCFEC | 273 | 6 | 1.909600 | -1.039089 | 335 | 123.42 | 0.368418 |  |
| GO:0009605\_response\_to\_external\_stimulus | NRP2 | 339 | 7 | 1.794123 | -1.038820 | 336 | 123.47 | 0.367470 |  |
| GO:0009605\_response\_to\_external\_stimulus | RBP4 | 339 | 7 | 1.794123 | -1.038820 | 336 | 123.47 | 0.367470 |  |
| GO:0009605\_response\_to\_external\_stimulus | BMP2 | 339 | 7 | 1.794123 | -1.038820 | 336 | 123.47 | 0.367470 |  |
| GO:0009605\_response\_to\_external\_stimulus | PLEK | 339 | 7 | 1.794123 | -1.038820 | 336 | 123.47 | 0.367470 |  |
| GO:0009605\_response\_to\_external\_stimulus | APOE | 339 | 7 | 1.794123 | -1.038820 | 336 | 123.47 | 0.367470 |  |
| GO:0009605\_response\_to\_external\_stimulus | GDAP1 | 339 | 7 | 1.794123 | -1.038820 | 336 | 123.47 | 0.367470 |  |
| GO:0009605\_response\_to\_external\_stimulus | RGS9BP | 339 | 7 | 1.794123 | -1.038820 | 336 | 123.47 | 0.367470 |  |
| GO:0044237\_cellular\_metabolic\_process | WNT5A | 1974 | 28 | 1.232437 | -1.038345 | 337 | 123.51 | 0.366499 |  |
| GO:0044237\_cellular\_metabolic\_process | XRCC4 | 1974 | 28 | 1.232437 | -1.038345 | 337 | 123.51 | 0.366499 |  |
| GO:0044237\_cellular\_metabolic\_process | RBP4 | 1974 | 28 | 1.232437 | -1.038345 | 337 | 123.51 | 0.366499 |  |
| GO:0044237\_cellular\_metabolic\_process | ZEB2 | 1974 | 28 | 1.232437 | -1.038345 | 337 | 123.51 | 0.366499 |  |
| GO:0044237\_cellular\_metabolic\_process | RORA | 1974 | 28 | 1.232437 | -1.038345 | 337 | 123.51 | 0.366499 |  |
| GO:0044237\_cellular\_metabolic\_process | DCN | 1974 | 28 | 1.232437 | -1.038345 | 337 | 123.51 | 0.366499 |  |
| GO:0044237\_cellular\_metabolic\_process | PRKG1 | 1974 | 28 | 1.232437 | -1.038345 | 337 | 123.51 | 0.366499 |  |
| GO:0044237\_cellular\_metabolic\_process | CITED2 | 1974 | 28 | 1.232437 | -1.038345 | 337 | 123.51 | 0.366499 |  |
| GO:0044237\_cellular\_metabolic\_process | CTNNB1 | 1974 | 28 | 1.232437 | -1.038345 | 337 | 123.51 | 0.366499 |  |
| GO:0044237\_cellular\_metabolic\_process | TGFB2 | 1974 | 28 | 1.232437 | -1.038345 | 337 | 123.51 | 0.366499 |  |
| GO:0044237\_cellular\_metabolic\_process | A930038C07RIK | 1974 | 28 | 1.232437 | -1.038345 | 337 | 123.51 | 0.366499 |  |
| GO:0044237\_cellular\_metabolic\_process | APOE | 1974 | 28 | 1.232437 | -1.038345 | 337 | 123.51 | 0.366499 |  |
| GO:0044237\_cellular\_metabolic\_process | RB1CC1 | 1974 | 28 | 1.232437 | -1.038345 | 337 | 123.51 | 0.366499 |  |
| GO:0044237\_cellular\_metabolic\_process | HBA-X | 1974 | 28 | 1.232437 | -1.038345 | 337 | 123.51 | 0.366499 |  |
| GO:0044237\_cellular\_metabolic\_process | EGR1 | 1974 | 28 | 1.232437 | -1.038345 | 337 | 123.51 | 0.366499 |  |
| GO:0044237\_cellular\_metabolic\_process | BMP2 | 1974 | 28 | 1.232437 | -1.038345 | 337 | 123.51 | 0.366499 |  |
| GO:0044237\_cellular\_metabolic\_process | ADSSL1 | 1974 | 28 | 1.232437 | -1.038345 | 337 | 123.51 | 0.366499 |  |
| GO:0044237\_cellular\_metabolic\_process | VAV3 | 1974 | 28 | 1.232437 | -1.038345 | 337 | 123.51 | 0.366499 |  |
| GO:0044237\_cellular\_metabolic\_process | PLEK | 1974 | 28 | 1.232437 | -1.038345 | 337 | 123.51 | 0.366499 |  |
| GO:0044237\_cellular\_metabolic\_process | KLF12 | 1974 | 28 | 1.232437 | -1.038345 | 337 | 123.51 | 0.366499 |  |
| GO:0044237\_cellular\_metabolic\_process | TBX5 | 1974 | 28 | 1.232437 | -1.038345 | 337 | 123.51 | 0.366499 |  |
| GO:0044237\_cellular\_metabolic\_process | CFTR | 1974 | 28 | 1.232437 | -1.038345 | 337 | 123.51 | 0.366499 |  |
| GO:0044237\_cellular\_metabolic\_process | FOXP4 | 1974 | 28 | 1.232437 | -1.038345 | 337 | 123.51 | 0.366499 |  |
| GO:0044237\_cellular\_metabolic\_process | BTG2 | 1974 | 28 | 1.232437 | -1.038345 | 337 | 123.51 | 0.366499 |  |
| GO:0044237\_cellular\_metabolic\_process | JUN | 1974 | 28 | 1.232437 | -1.038345 | 337 | 123.51 | 0.366499 |  |
| GO:0044237\_cellular\_metabolic\_process | FOXC1 | 1974 | 28 | 1.232437 | -1.038345 | 337 | 123.51 | 0.366499 |  |
| GO:0044237\_cellular\_metabolic\_process | LRP8 | 1974 | 28 | 1.232437 | -1.038345 | 337 | 123.51 | 0.366499 |  |
| GO:0044237\_cellular\_metabolic\_process | C1D | 1974 | 28 | 1.232437 | -1.038345 | 337 | 123.51 | 0.366499 |  |
| GO:0034103\_regulation\_of\_tissue\_remodeling | BMP2 | 45 | 2 | 3.861635 | -1.026582 | 339 | 125.12 | 0.369086 |  |
| GO:0034103\_regulation\_of\_tissue\_remodeling | CTNNB1 | 45 | 2 | 3.861635 | -1.026582 | 339 | 125.12 | 0.369086 |  |
| GO:0046546\_development\_of\_primary\_male\_sexual\_characteristics | WNT5A | 45 | 2 | 3.861635 | -1.026582 | 339 | 125.12 | 0.369086 |  |
| GO:0046546\_development\_of\_primary\_male\_sexual\_characteristics | RBP4 | 45 | 2 | 3.861635 | -1.026582 | 339 | 125.12 | 0.369086 |  |
| GO:0007242\_intracellular\_signaling\_cascade | RHOJ | 411 | 8 | 1.691227 | -1.019624 | 340 | 125.54 | 0.369235 |  |
| GO:0007242\_intracellular\_signaling\_cascade | WNT5A | 411 | 8 | 1.691227 | -1.019624 | 340 | 125.54 | 0.369235 |  |
| GO:0007242\_intracellular\_signaling\_cascade | VAV3 | 411 | 8 | 1.691227 | -1.019624 | 340 | 125.54 | 0.369235 |  |
| GO:0007242\_intracellular\_signaling\_cascade | PLEK | 411 | 8 | 1.691227 | -1.019624 | 340 | 125.54 | 0.369235 |  |
| GO:0007242\_intracellular\_signaling\_cascade | RB1CC1 | 411 | 8 | 1.691227 | -1.019624 | 340 | 125.54 | 0.369235 |  |
| GO:0007242\_intracellular\_signaling\_cascade | ZEB2 | 411 | 8 | 1.691227 | -1.019624 | 340 | 125.54 | 0.369235 |  |
| GO:0007242\_intracellular\_signaling\_cascade | CDC42EP4 | 411 | 8 | 1.691227 | -1.019624 | 340 | 125.54 | 0.369235 |  |
| GO:0007242\_intracellular\_signaling\_cascade | CTNNB1 | 411 | 8 | 1.691227 | -1.019624 | 340 | 125.54 | 0.369235 |  |
| GO:0032268\_regulation\_of\_cellular\_protein\_metabolic\_process | PLEK | 152 | 4 | 2.286495 | -1.016447 | 341 | 125.61 | 0.368358 |  |
| GO:0032268\_regulation\_of\_cellular\_protein\_metabolic\_process | RB1CC1 | 152 | 4 | 2.286495 | -1.016447 | 341 | 125.61 | 0.368358 |  |
| GO:0032268\_regulation\_of\_cellular\_protein\_metabolic\_process | JUN | 152 | 4 | 2.286495 | -1.016447 | 341 | 125.61 | 0.368358 |  |
| GO:0032268\_regulation\_of\_cellular\_protein\_metabolic\_process | ZEB2 | 152 | 4 | 2.286495 | -1.016447 | 341 | 125.61 | 0.368358 |  |
| GO:0006350\_transcription | EGR1 | 701 | 12 | 1.487363 | -1.010985 | 342 | 125.78 | 0.367778 |  |
| GO:0006350\_transcription | BMP2 | 701 | 12 | 1.487363 | -1.010985 | 342 | 125.78 | 0.367778 |  |
| GO:0006350\_transcription | KLF12 | 701 | 12 | 1.487363 | -1.010985 | 342 | 125.78 | 0.367778 |  |
| GO:0006350\_transcription | JUN | 701 | 12 | 1.487363 | -1.010985 | 342 | 125.78 | 0.367778 |  |
| GO:0006350\_transcription | TBX5 | 701 | 12 | 1.487363 | -1.010985 | 342 | 125.78 | 0.367778 |  |
| GO:0006350\_transcription | FOXC1 | 701 | 12 | 1.487363 | -1.010985 | 342 | 125.78 | 0.367778 |  |
| GO:0006350\_transcription | RORA | 701 | 12 | 1.487363 | -1.010985 | 342 | 125.78 | 0.367778 |  |
| GO:0006350\_transcription | HBA-X | 701 | 12 | 1.487363 | -1.010985 | 342 | 125.78 | 0.367778 |  |
| GO:0006350\_transcription | FOXP4 | 701 | 12 | 1.487363 | -1.010985 | 342 | 125.78 | 0.367778 |  |
| GO:0006350\_transcription | C1D | 701 | 12 | 1.487363 | -1.010985 | 342 | 125.78 | 0.367778 |  |
| GO:0006350\_transcription | CITED2 | 701 | 12 | 1.487363 | -1.010985 | 342 | 125.78 | 0.367778 |  |
| GO:0006350\_transcription | CTNNB1 | 701 | 12 | 1.487363 | -1.010985 | 342 | 125.78 | 0.367778 |  |
| GO:0009581\_detection\_of\_external\_stimulus | RBP4 | 46 | 2 | 3.777687 | -1.010384 | 345 | 127.12 | 0.368464 |  |
| GO:0009581\_detection\_of\_external\_stimulus | RGS9BP | 46 | 2 | 3.777687 | -1.010384 | 345 | 127.12 | 0.368464 |  |
| GO:0030850\_prostate\_gland\_development | WNT5A | 46 | 2 | 3.777687 | -1.010384 | 345 | 127.12 | 0.368464 |  |
| GO:0030850\_prostate\_gland\_development | CTNNB1 | 46 | 2 | 3.777687 | -1.010384 | 345 | 127.12 | 0.368464 |  |
| GO:0042063\_gliogenesis | BMP2 | 46 | 2 | 3.777687 | -1.010384 | 345 | 127.12 | 0.368464 |  |
| GO:0042063\_gliogenesis | CTNNB1 | 46 | 2 | 3.777687 | -1.010384 | 345 | 127.12 | 0.368464 |  |
| GO:0007517\_muscle\_organ\_development | SCHIP1 | 153 | 4 | 2.271550 | -1.008519 | 346 | 127.17 | 0.367543 |  |
| GO:0007517\_muscle\_organ\_development | RBP4 | 153 | 4 | 2.271550 | -1.008519 | 346 | 127.17 | 0.367543 |  |
| GO:0007517\_muscle\_organ\_development | TBX5 | 153 | 4 | 2.271550 | -1.008519 | 346 | 127.17 | 0.367543 |  |
| GO:0007517\_muscle\_organ\_development | PROX1 | 153 | 4 | 2.271550 | -1.008519 | 346 | 127.17 | 0.367543 |  |
| GO:0001667\_ameboidal\_cell\_migration | SCHIP1 | 9 | 1 | 9.654088 | -1.004267 | 368 | 138.47 | 0.376277 |  |
| GO:0002021\_response\_to\_dietary\_excess | APOE | 9 | 1 | 9.654088 | -1.004267 | 368 | 138.47 | 0.376277 |  |
| GO:0007379\_segment\_specification | DLL1 | 9 | 1 | 9.654088 | -1.004267 | 368 | 138.47 | 0.376277 |  |
| GO:0010165\_response\_to\_X-ray | XRCC4 | 9 | 1 | 9.654088 | -1.004267 | 368 | 138.47 | 0.376277 |  |
| GO:0017145\_stem\_cell\_division | TGFB2 | 9 | 1 | 9.654088 | -1.004267 | 368 | 138.47 | 0.376277 |  |
| GO:0030325\_adrenal\_gland\_development | CITED2 | 9 | 1 | 9.654088 | -1.004267 | 368 | 138.47 | 0.376277 |  |
| GO:0033151\_V(D)J\_recombination | XRCC4 | 9 | 1 | 9.654088 | -1.004267 | 368 | 138.47 | 0.376277 |  |
| GO:0033344\_cholesterol\_efflux | APOE | 9 | 1 | 9.654088 | -1.004267 | 368 | 138.47 | 0.376277 |  |
| GO:0034605\_cellular\_response\_to\_heat | TCFEC | 9 | 1 | 9.654088 | -1.004267 | 368 | 138.47 | 0.376277 |  |
| GO:0045136\_development\_of\_secondary\_sexual\_characteristics | WNT5A | 9 | 1 | 9.654088 | -1.004267 | 368 | 138.47 | 0.376277 |  |
| GO:0045214\_sarcomere\_organization | PROX1 | 9 | 1 | 9.654088 | -1.004267 | 368 | 138.47 | 0.376277 |  |
| GO:0045671\_negative\_regulation\_of\_osteoclast\_differentiation | CTNNB1 | 9 | 1 | 9.654088 | -1.004267 | 368 | 138.47 | 0.376277 |  |
| GO:0048146\_positive\_regulation\_of\_fibroblast\_proliferation | XRCC4 | 9 | 1 | 9.654088 | -1.004267 | 368 | 138.47 | 0.376277 |  |
| GO:0048284\_organelle\_fusion | VAV3 | 9 | 1 | 9.654088 | -1.004267 | 368 | 138.47 | 0.376277 |  |
| GO:0048708\_astrocyte\_differentiation | BMP2 | 9 | 1 | 9.654088 | -1.004267 | 368 | 138.47 | 0.376277 |  |
| GO:0051023\_regulation\_of\_immunoglobulin\_secretion | RBP4 | 9 | 1 | 9.654088 | -1.004267 | 368 | 138.47 | 0.376277 |  |
| GO:0055012\_ventricular\_cardiac\_muscle\_cell\_differentiation | PROX1 | 9 | 1 | 9.654088 | -1.004267 | 368 | 138.47 | 0.376277 |  |
| GO:0055013\_cardiac\_muscle\_cell\_development | PROX1 | 9 | 1 | 9.654088 | -1.004267 | 368 | 138.47 | 0.376277 |  |
| GO:0060325\_face\_morphogenesis | SCHIP1 | 9 | 1 | 9.654088 | -1.004267 | 368 | 138.47 | 0.376277 |  |
| GO:0060513\_prostatic\_bud\_formation | WNT5A | 9 | 1 | 9.654088 | -1.004267 | 368 | 138.47 | 0.376277 |  |
| GO:0060602\_branch\_elongation\_of\_an\_epithelium | WNT5A | 9 | 1 | 9.654088 | -1.004267 | 368 | 138.47 | 0.376277 |  |
| GO:0070306\_lens\_fiber\_cell\_differentiation | PROX1 | 9 | 1 | 9.654088 | -1.004267 | 368 | 138.47 | 0.376277 |  |
| GO:0010468\_regulation\_of\_gene\_expression | EGR1 | 778 | 13 | 1.451836 | -1.003521 | 369 | 138.54 | 0.375447 |  |
| GO:0010468\_regulation\_of\_gene\_expression | BMP2 | 778 | 13 | 1.451836 | -1.003521 | 369 | 138.54 | 0.375447 |  |
| GO:0010468\_regulation\_of\_gene\_expression | KLF12 | 778 | 13 | 1.451836 | -1.003521 | 369 | 138.54 | 0.375447 |  |
| GO:0010468\_regulation\_of\_gene\_expression | TBX5 | 778 | 13 | 1.451836 | -1.003521 | 369 | 138.54 | 0.375447 |  |
| GO:0010468\_regulation\_of\_gene\_expression | RORA | 778 | 13 | 1.451836 | -1.003521 | 369 | 138.54 | 0.375447 |  |
| GO:0010468\_regulation\_of\_gene\_expression | FOXP4 | 778 | 13 | 1.451836 | -1.003521 | 369 | 138.54 | 0.375447 |  |
| GO:0010468\_regulation\_of\_gene\_expression | CITED2 | 778 | 13 | 1.451836 | -1.003521 | 369 | 138.54 | 0.375447 |  |
| GO:0010468\_regulation\_of\_gene\_expression | CTNNB1 | 778 | 13 | 1.451836 | -1.003521 | 369 | 138.54 | 0.375447 |  |
| GO:0010468\_regulation\_of\_gene\_expression | APOE | 778 | 13 | 1.451836 | -1.003521 | 369 | 138.54 | 0.375447 |  |
| GO:0010468\_regulation\_of\_gene\_expression | JUN | 778 | 13 | 1.451836 | -1.003521 | 369 | 138.54 | 0.375447 |  |
| GO:0010468\_regulation\_of\_gene\_expression | FOXC1 | 778 | 13 | 1.451836 | -1.003521 | 369 | 138.54 | 0.375447 |  |
| GO:0010468\_regulation\_of\_gene\_expression | HBA-X | 778 | 13 | 1.451836 | -1.003521 | 369 | 138.54 | 0.375447 |  |
| GO:0010468\_regulation\_of\_gene\_expression | C1D | 778 | 13 | 1.451836 | -1.003521 | 369 | 138.54 | 0.375447 |  |
| GO:0044238\_primary\_metabolic\_process | WNT5A | 1905 | 27 | 1.231466 | -0.998594 | 370 | 139.02 | 0.375730 |  |
| GO:0044238\_primary\_metabolic\_process | XRCC4 | 1905 | 27 | 1.231466 | -0.998594 | 370 | 139.02 | 0.375730 |  |
| GO:0044238\_primary\_metabolic\_process | RBP4 | 1905 | 27 | 1.231466 | -0.998594 | 370 | 139.02 | 0.375730 |  |
| GO:0044238\_primary\_metabolic\_process | ZEB2 | 1905 | 27 | 1.231466 | -0.998594 | 370 | 139.02 | 0.375730 |  |
| GO:0044238\_primary\_metabolic\_process | RORA | 1905 | 27 | 1.231466 | -0.998594 | 370 | 139.02 | 0.375730 |  |
| GO:0044238\_primary\_metabolic\_process | DCN | 1905 | 27 | 1.231466 | -0.998594 | 370 | 139.02 | 0.375730 |  |
| GO:0044238\_primary\_metabolic\_process | PRKG1 | 1905 | 27 | 1.231466 | -0.998594 | 370 | 139.02 | 0.375730 |  |
| GO:0044238\_primary\_metabolic\_process | CITED2 | 1905 | 27 | 1.231466 | -0.998594 | 370 | 139.02 | 0.375730 |  |
| GO:0044238\_primary\_metabolic\_process | CTNNB1 | 1905 | 27 | 1.231466 | -0.998594 | 370 | 139.02 | 0.375730 |  |
| GO:0044238\_primary\_metabolic\_process | TGFB2 | 1905 | 27 | 1.231466 | -0.998594 | 370 | 139.02 | 0.375730 |  |
| GO:0044238\_primary\_metabolic\_process | A930038C07RIK | 1905 | 27 | 1.231466 | -0.998594 | 370 | 139.02 | 0.375730 |  |
| GO:0044238\_primary\_metabolic\_process | APOE | 1905 | 27 | 1.231466 | -0.998594 | 370 | 139.02 | 0.375730 |  |
| GO:0044238\_primary\_metabolic\_process | RB1CC1 | 1905 | 27 | 1.231466 | -0.998594 | 370 | 139.02 | 0.375730 |  |
| GO:0044238\_primary\_metabolic\_process | HBA-X | 1905 | 27 | 1.231466 | -0.998594 | 370 | 139.02 | 0.375730 |  |
| GO:0044238\_primary\_metabolic\_process | EGR1 | 1905 | 27 | 1.231466 | -0.998594 | 370 | 139.02 | 0.375730 |  |
| GO:0044238\_primary\_metabolic\_process | BMP2 | 1905 | 27 | 1.231466 | -0.998594 | 370 | 139.02 | 0.375730 |  |
| GO:0044238\_primary\_metabolic\_process | ADSSL1 | 1905 | 27 | 1.231466 | -0.998594 | 370 | 139.02 | 0.375730 |  |
| GO:0044238\_primary\_metabolic\_process | VAV3 | 1905 | 27 | 1.231466 | -0.998594 | 370 | 139.02 | 0.375730 |  |
| GO:0044238\_primary\_metabolic\_process | PLEK | 1905 | 27 | 1.231466 | -0.998594 | 370 | 139.02 | 0.375730 |  |
| GO:0044238\_primary\_metabolic\_process | KLF12 | 1905 | 27 | 1.231466 | -0.998594 | 370 | 139.02 | 0.375730 |  |
| GO:0044238\_primary\_metabolic\_process | TBX5 | 1905 | 27 | 1.231466 | -0.998594 | 370 | 139.02 | 0.375730 |  |
| GO:0044238\_primary\_metabolic\_process | CFTR | 1905 | 27 | 1.231466 | -0.998594 | 370 | 139.02 | 0.375730 |  |
| GO:0044238\_primary\_metabolic\_process | FOXP4 | 1905 | 27 | 1.231466 | -0.998594 | 370 | 139.02 | 0.375730 |  |
| GO:0044238\_primary\_metabolic\_process | BTG2 | 1905 | 27 | 1.231466 | -0.998594 | 370 | 139.02 | 0.375730 |  |
| GO:0044238\_primary\_metabolic\_process | JUN | 1905 | 27 | 1.231466 | -0.998594 | 370 | 139.02 | 0.375730 |  |
| GO:0044238\_primary\_metabolic\_process | FOXC1 | 1905 | 27 | 1.231466 | -0.998594 | 370 | 139.02 | 0.375730 |  |
| GO:0044238\_primary\_metabolic\_process | C1D | 1905 | 27 | 1.231466 | -0.998594 | 370 | 139.02 | 0.375730 |  |
| GO:0043283\_biopolymer\_metabolic\_process | EGR1 | 1490 | 22 | 1.282892 | -0.995879 | 371 | 139.15 | 0.375067 |  |
| GO:0043283\_biopolymer\_metabolic\_process | WNT5A | 1490 | 22 | 1.282892 | -0.995879 | 371 | 139.15 | 0.375067 |  |
| GO:0043283\_biopolymer\_metabolic\_process | XRCC4 | 1490 | 22 | 1.282892 | -0.995879 | 371 | 139.15 | 0.375067 |  |
| GO:0043283\_biopolymer\_metabolic\_process | BMP2 | 1490 | 22 | 1.282892 | -0.995879 | 371 | 139.15 | 0.375067 |  |
| GO:0043283\_biopolymer\_metabolic\_process | KLF12 | 1490 | 22 | 1.282892 | -0.995879 | 371 | 139.15 | 0.375067 |  |
| GO:0043283\_biopolymer\_metabolic\_process | PLEK | 1490 | 22 | 1.282892 | -0.995879 | 371 | 139.15 | 0.375067 |  |
| GO:0043283\_biopolymer\_metabolic\_process | TBX5 | 1490 | 22 | 1.282892 | -0.995879 | 371 | 139.15 | 0.375067 |  |
| GO:0043283\_biopolymer\_metabolic\_process | ZEB2 | 1490 | 22 | 1.282892 | -0.995879 | 371 | 139.15 | 0.375067 |  |
| GO:0043283\_biopolymer\_metabolic\_process | RORA | 1490 | 22 | 1.282892 | -0.995879 | 371 | 139.15 | 0.375067 |  |
| GO:0043283\_biopolymer\_metabolic\_process | DCN | 1490 | 22 | 1.282892 | -0.995879 | 371 | 139.15 | 0.375067 |  |
| GO:0043283\_biopolymer\_metabolic\_process | PRKG1 | 1490 | 22 | 1.282892 | -0.995879 | 371 | 139.15 | 0.375067 |  |
| GO:0043283\_biopolymer\_metabolic\_process | FOXP4 | 1490 | 22 | 1.282892 | -0.995879 | 371 | 139.15 | 0.375067 |  |
| GO:0043283\_biopolymer\_metabolic\_process | CITED2 | 1490 | 22 | 1.282892 | -0.995879 | 371 | 139.15 | 0.375067 |  |
| GO:0043283\_biopolymer\_metabolic\_process | CTNNB1 | 1490 | 22 | 1.282892 | -0.995879 | 371 | 139.15 | 0.375067 |  |
| GO:0043283\_biopolymer\_metabolic\_process | TGFB2 | 1490 | 22 | 1.282892 | -0.995879 | 371 | 139.15 | 0.375067 |  |
| GO:0043283\_biopolymer\_metabolic\_process | A930038C07RIK | 1490 | 22 | 1.282892 | -0.995879 | 371 | 139.15 | 0.375067 |  |
| GO:0043283\_biopolymer\_metabolic\_process | BTG2 | 1490 | 22 | 1.282892 | -0.995879 | 371 | 139.15 | 0.375067 |  |
| GO:0043283\_biopolymer\_metabolic\_process | JUN | 1490 | 22 | 1.282892 | -0.995879 | 371 | 139.15 | 0.375067 |  |
| GO:0043283\_biopolymer\_metabolic\_process | RB1CC1 | 1490 | 22 | 1.282892 | -0.995879 | 371 | 139.15 | 0.375067 |  |
| GO:0043283\_biopolymer\_metabolic\_process | FOXC1 | 1490 | 22 | 1.282892 | -0.995879 | 371 | 139.15 | 0.375067 |  |
| GO:0043283\_biopolymer\_metabolic\_process | HBA-X | 1490 | 22 | 1.282892 | -0.995879 | 371 | 139.15 | 0.375067 |  |
| GO:0043283\_biopolymer\_metabolic\_process | C1D | 1490 | 22 | 1.282892 | -0.995879 | 371 | 139.15 | 0.375067 |  |
| GO:0044267\_cellular\_protein\_metabolic\_process | WNT5A | 559 | 10 | 1.554325 | -0.992803 | 372 | 140.33 | 0.377231 |  |
| GO:0044267\_cellular\_protein\_metabolic\_process | A930038C07RIK | 559 | 10 | 1.554325 | -0.992803 | 372 | 140.33 | 0.377231 |  |
| GO:0044267\_cellular\_protein\_metabolic\_process | PLEK | 559 | 10 | 1.554325 | -0.992803 | 372 | 140.33 | 0.377231 |  |
| GO:0044267\_cellular\_protein\_metabolic\_process | BTG2 | 559 | 10 | 1.554325 | -0.992803 | 372 | 140.33 | 0.377231 |  |
| GO:0044267\_cellular\_protein\_metabolic\_process | RB1CC1 | 559 | 10 | 1.554325 | -0.992803 | 372 | 140.33 | 0.377231 |  |
| GO:0044267\_cellular\_protein\_metabolic\_process | JUN | 559 | 10 | 1.554325 | -0.992803 | 372 | 140.33 | 0.377231 |  |
| GO:0044267\_cellular\_protein\_metabolic\_process | ZEB2 | 559 | 10 | 1.554325 | -0.992803 | 372 | 140.33 | 0.377231 |  |
| GO:0044267\_cellular\_protein\_metabolic\_process | DCN | 559 | 10 | 1.554325 | -0.992803 | 372 | 140.33 | 0.377231 |  |
| GO:0044267\_cellular\_protein\_metabolic\_process | PRKG1 | 559 | 10 | 1.554325 | -0.992803 | 372 | 140.33 | 0.377231 |  |
| GO:0044267\_cellular\_protein\_metabolic\_process | TGFB2 | 559 | 10 | 1.554325 | -0.992803 | 372 | 140.33 | 0.377231 |  |
| GO:0007548\_sex\_differentiation | WNT5A | 98 | 3 | 2.659800 | -0.990334 | 374 | 140.77 | 0.376390 |  |
| GO:0007548\_sex\_differentiation | RBP4 | 98 | 3 | 2.659800 | -0.990334 | 374 | 140.77 | 0.376390 |  |
| GO:0007548\_sex\_differentiation | FOXC1 | 98 | 3 | 2.659800 | -0.990334 | 374 | 140.77 | 0.376390 |  |
| GO:0009314\_response\_to\_radiation | XRCC4 | 98 | 3 | 2.659800 | -0.990334 | 374 | 140.77 | 0.376390 |  |
| GO:0009314\_response\_to\_radiation | RBP4 | 98 | 3 | 2.659800 | -0.990334 | 374 | 140.77 | 0.376390 |  |
| GO:0009314\_response\_to\_radiation | RGS9BP | 98 | 3 | 2.659800 | -0.990334 | 374 | 140.77 | 0.376390 |  |
| GO:0043170\_macromolecule\_metabolic\_process | WNT5A | 1576 | 23 | 1.268018 | -0.985708 | 375 | 141.08 | 0.376213 |  |
| GO:0043170\_macromolecule\_metabolic\_process | EGR1 | 1576 | 23 | 1.268018 | -0.985708 | 375 | 141.08 | 0.376213 |  |
| GO:0043170\_macromolecule\_metabolic\_process | XRCC4 | 1576 | 23 | 1.268018 | -0.985708 | 375 | 141.08 | 0.376213 |  |
| GO:0043170\_macromolecule\_metabolic\_process | BMP2 | 1576 | 23 | 1.268018 | -0.985708 | 375 | 141.08 | 0.376213 |  |
| GO:0043170\_macromolecule\_metabolic\_process | PLEK | 1576 | 23 | 1.268018 | -0.985708 | 375 | 141.08 | 0.376213 |  |
| GO:0043170\_macromolecule\_metabolic\_process | KLF12 | 1576 | 23 | 1.268018 | -0.985708 | 375 | 141.08 | 0.376213 |  |
| GO:0043170\_macromolecule\_metabolic\_process | TBX5 | 1576 | 23 | 1.268018 | -0.985708 | 375 | 141.08 | 0.376213 |  |
| GO:0043170\_macromolecule\_metabolic\_process | ZEB2 | 1576 | 23 | 1.268018 | -0.985708 | 375 | 141.08 | 0.376213 |  |
| GO:0043170\_macromolecule\_metabolic\_process | DCN | 1576 | 23 | 1.268018 | -0.985708 | 375 | 141.08 | 0.376213 |  |
| GO:0043170\_macromolecule\_metabolic\_process | RORA | 1576 | 23 | 1.268018 | -0.985708 | 375 | 141.08 | 0.376213 |  |
| GO:0043170\_macromolecule\_metabolic\_process | PRKG1 | 1576 | 23 | 1.268018 | -0.985708 | 375 | 141.08 | 0.376213 |  |
| GO:0043170\_macromolecule\_metabolic\_process | FOXP4 | 1576 | 23 | 1.268018 | -0.985708 | 375 | 141.08 | 0.376213 |  |
| GO:0043170\_macromolecule\_metabolic\_process | TGFB2 | 1576 | 23 | 1.268018 | -0.985708 | 375 | 141.08 | 0.376213 |  |
| GO:0043170\_macromolecule\_metabolic\_process | CITED2 | 1576 | 23 | 1.268018 | -0.985708 | 375 | 141.08 | 0.376213 |  |
| GO:0043170\_macromolecule\_metabolic\_process | CTNNB1 | 1576 | 23 | 1.268018 | -0.985708 | 375 | 141.08 | 0.376213 |  |
| GO:0043170\_macromolecule\_metabolic\_process | A930038C07RIK | 1576 | 23 | 1.268018 | -0.985708 | 375 | 141.08 | 0.376213 |  |
| GO:0043170\_macromolecule\_metabolic\_process | BTG2 | 1576 | 23 | 1.268018 | -0.985708 | 375 | 141.08 | 0.376213 |  |
| GO:0043170\_macromolecule\_metabolic\_process | APOE | 1576 | 23 | 1.268018 | -0.985708 | 375 | 141.08 | 0.376213 |  |
| GO:0043170\_macromolecule\_metabolic\_process | RB1CC1 | 1576 | 23 | 1.268018 | -0.985708 | 375 | 141.08 | 0.376213 |  |
| GO:0043170\_macromolecule\_metabolic\_process | JUN | 1576 | 23 | 1.268018 | -0.985708 | 375 | 141.08 | 0.376213 |  |
| GO:0043170\_macromolecule\_metabolic\_process | FOXC1 | 1576 | 23 | 1.268018 | -0.985708 | 375 | 141.08 | 0.376213 |  |
| GO:0043170\_macromolecule\_metabolic\_process | HBA-X | 1576 | 23 | 1.268018 | -0.985708 | 375 | 141.08 | 0.376213 |  |
| GO:0043170\_macromolecule\_metabolic\_process | C1D | 1576 | 23 | 1.268018 | -0.985708 | 375 | 141.08 | 0.376213 |  |
| GO:0060348\_bone\_development | BMP2 | 99 | 3 | 2.632933 | -0.980374 | 377 | 141.58 | 0.375544 |  |
| GO:0060348\_bone\_development | FOXC1 | 99 | 3 | 2.632933 | -0.980374 | 377 | 141.58 | 0.375544 |  |
| GO:0060348\_bone\_development | CTNNB1 | 99 | 3 | 2.632933 | -0.980374 | 377 | 141.58 | 0.375544 |  |
| GO:0060562\_epithelial\_tube\_morphogenesis | WNT5A | 99 | 3 | 2.632933 | -0.980374 | 377 | 141.58 | 0.375544 |  |
| GO:0060562\_epithelial\_tube\_morphogenesis | BMP2 | 99 | 3 | 2.632933 | -0.980374 | 377 | 141.58 | 0.375544 |  |
| GO:0060562\_epithelial\_tube\_morphogenesis | ZEB2 | 99 | 3 | 2.632933 | -0.980374 | 377 | 141.58 | 0.375544 |  |
| GO:0046849\_bone\_remodeling | BMP2 | 48 | 2 | 3.620283 | -0.979221 | 378 | 142.3 | 0.376455 |  |
| GO:0046849\_bone\_remodeling | CTNNB1 | 48 | 2 | 3.620283 | -0.979221 | 378 | 142.3 | 0.376455 |  |
| GO:0006066\_alcohol\_metabolic\_process | RBP4 | 158 | 4 | 2.199666 | -0.969974 | 379 | 143.25 | 0.377968 |  |
| GO:0006066\_alcohol\_metabolic\_process | APOE | 158 | 4 | 2.199666 | -0.969974 | 379 | 143.25 | 0.377968 |  |
| GO:0006066\_alcohol\_metabolic\_process | CFTR | 158 | 4 | 2.199666 | -0.969974 | 379 | 143.25 | 0.377968 |  |
| GO:0006066\_alcohol\_metabolic\_process | TGFB2 | 158 | 4 | 2.199666 | -0.969974 | 379 | 143.25 | 0.377968 |  |
| GO:0022008\_neurogenesis | XRCC4 | 423 | 8 | 1.643249 | -0.964789 | 380 | 143.3 | 0.377105 |  |
| GO:0022008\_neurogenesis | BMP2 | 423 | 8 | 1.643249 | -0.964789 | 380 | 143.3 | 0.377105 |  |
| GO:0022008\_neurogenesis | APOE | 423 | 8 | 1.643249 | -0.964789 | 380 | 143.3 | 0.377105 |  |
| GO:0022008\_neurogenesis | DLL1 | 423 | 8 | 1.643249 | -0.964789 | 380 | 143.3 | 0.377105 |  |
| GO:0022008\_neurogenesis | RORA | 423 | 8 | 1.643249 | -0.964789 | 380 | 143.3 | 0.377105 |  |
| GO:0022008\_neurogenesis | PRKG1 | 423 | 8 | 1.643249 | -0.964789 | 380 | 143.3 | 0.377105 |  |
| GO:0022008\_neurogenesis | TGFB2 | 423 | 8 | 1.643249 | -0.964789 | 380 | 143.3 | 0.377105 |  |
| GO:0022008\_neurogenesis | CTNNB1 | 423 | 8 | 1.643249 | -0.964789 | 380 | 143.3 | 0.377105 |  |
| GO:0002440\_production\_of\_molecular\_mediator\_of\_immune\_response | RBP4 | 49 | 2 | 3.546400 | -0.964222 | 383 | 144.58 | 0.377493 |  |
| GO:0002440\_production\_of\_molecular\_mediator\_of\_immune\_response | XRCC4 | 49 | 2 | 3.546400 | -0.964222 | 383 | 144.58 | 0.377493 |  |
| GO:0021543\_pallium\_development | LRP8 | 49 | 2 | 3.546400 | -0.964222 | 383 | 144.58 | 0.377493 |  |
| GO:0021543\_pallium\_development | ZEB2 | 49 | 2 | 3.546400 | -0.964222 | 383 | 144.58 | 0.377493 |  |
| GO:0046661\_male\_sex\_differentiation | WNT5A | 49 | 2 | 3.546400 | -0.964222 | 383 | 144.58 | 0.377493 |  |
| GO:0046661\_male\_sex\_differentiation | RBP4 | 49 | 2 | 3.546400 | -0.964222 | 383 | 144.58 | 0.377493 |  |
| GO:0002762\_negative\_regulation\_of\_myeloid\_leukocyte\_differentiation | CTNNB1 | 10 | 1 | 8.688679 | -0.960934 | 403 | 157.34 | 0.390422 |  |
| GO:0006081\_cellular\_aldehyde\_metabolic\_process | RBP4 | 10 | 1 | 8.688679 | -0.960934 | 403 | 157.34 | 0.390422 |  |
| GO:0009948\_anterior\_posterior\_axis\_specification | CTNNB1 | 10 | 1 | 8.688679 | -0.960934 | 403 | 157.34 | 0.390422 |  |
| GO:0030168\_platelet\_activation | PLEK | 10 | 1 | 8.688679 | -0.960934 | 403 | 157.34 | 0.390422 |  |
| GO:0031018\_endocrine\_pancreas\_development | INSM1 | 10 | 1 | 8.688679 | -0.960934 | 403 | 157.34 | 0.390422 |  |
| GO:0034105\_positive\_regulation\_of\_tissue\_remodeling | BMP2 | 10 | 1 | 8.688679 | -0.960934 | 403 | 157.34 | 0.390422 |  |
| GO:0042116\_macrophage\_activation | RORA | 10 | 1 | 8.688679 | -0.960934 | 403 | 157.34 | 0.390422 |  |
| GO:0043506\_regulation\_of\_JUN\_kinase\_activity | ZEB2 | 10 | 1 | 8.688679 | -0.960934 | 403 | 157.34 | 0.390422 |  |
| GO:0045446\_endothelial\_cell\_differentiation | PROX1 | 10 | 1 | 8.688679 | -0.960934 | 403 | 157.34 | 0.390422 |  |
| GO:0048339\_paraxial\_mesoderm\_development | FOXC1 | 10 | 1 | 8.688679 | -0.960934 | 403 | 157.34 | 0.390422 |  |
| GO:0048596\_embryonic\_camera-type\_eye\_morphogenesis | PROX1 | 10 | 1 | 8.688679 | -0.960934 | 403 | 157.34 | 0.390422 |  |
| GO:0048738\_cardiac\_muscle\_tissue\_development | RBP4 | 10 | 1 | 8.688679 | -0.960934 | 403 | 157.34 | 0.390422 |  |
| GO:0050714\_positive\_regulation\_of\_protein\_secretion | RBP4 | 10 | 1 | 8.688679 | -0.960934 | 403 | 157.34 | 0.390422 |  |
| GO:0051147\_regulation\_of\_muscle\_cell\_differentiation | PROX1 | 10 | 1 | 8.688679 | -0.960934 | 403 | 157.34 | 0.390422 |  |
| GO:0051651\_maintenance\_of\_location\_in\_cell | APOE | 10 | 1 | 8.688679 | -0.960934 | 403 | 157.34 | 0.390422 |  |
| GO:0060323\_head\_morphogenesis | SCHIP1 | 10 | 1 | 8.688679 | -0.960934 | 403 | 157.34 | 0.390422 |  |
| GO:0060343\_trabecula\_formation | RBP4 | 10 | 1 | 8.688679 | -0.960934 | 403 | 157.34 | 0.390422 |  |
| GO:0060601\_lateral\_sprouting\_from\_an\_epithelium | WNT5A | 10 | 1 | 8.688679 | -0.960934 | 403 | 157.34 | 0.390422 |  |
| GO:0060669\_embryonic\_placenta\_morphogenesis | RSPO3 | 10 | 1 | 8.688679 | -0.960934 | 403 | 157.34 | 0.390422 |  |
| GO:0060768\_regulation\_of\_epithelial\_cell\_proliferation\_involved\_in\_prostate\_gland\_development | CTNNB1 | 10 | 1 | 8.688679 | -0.960934 | 403 | 157.34 | 0.390422 |  |
| GO:0030036\_actin\_cytoskeleton\_organization | RHOJ | 102 | 3 | 2.555494 | -0.951284 | 404 | 157.84 | 0.390693 |  |
| GO:0030036\_actin\_cytoskeleton\_organization | PLEK | 102 | 3 | 2.555494 | -0.951284 | 404 | 157.84 | 0.390693 |  |
| GO:0030036\_actin\_cytoskeleton\_organization | PROX1 | 102 | 3 | 2.555494 | -0.951284 | 404 | 157.84 | 0.390693 |  |
| GO:0001656\_metanephros\_development | BMP2 | 50 | 2 | 3.475472 | -0.949589 | 407 | 158.63 | 0.389754 |  |
| GO:0001656\_metanephros\_development | FOXC1 | 50 | 2 | 3.475472 | -0.949589 | 407 | 158.63 | 0.389754 |  |
| GO:0007015\_actin\_filament\_organization | PLEK | 50 | 2 | 3.475472 | -0.949589 | 407 | 158.63 | 0.389754 |  |
| GO:0007015\_actin\_filament\_organization | PROX1 | 50 | 2 | 3.475472 | -0.949589 | 407 | 158.63 | 0.389754 |  |
| GO:0051606\_detection\_of\_stimulus | RBP4 | 50 | 2 | 3.475472 | -0.949589 | 407 | 158.63 | 0.389754 |  |
| GO:0051606\_detection\_of\_stimulus | RGS9BP | 50 | 2 | 3.475472 | -0.949589 | 407 | 158.63 | 0.389754 |  |
| GO:0006915\_apoptosis | XRCC4 | 427 | 8 | 1.627856 | -0.947143 | 408 | 158.77 | 0.389142 |  |
| GO:0006915\_apoptosis | ZC3HC1 | 427 | 8 | 1.627856 | -0.947143 | 408 | 158.77 | 0.389142 |  |
| GO:0006915\_apoptosis | SGK3 | 427 | 8 | 1.627856 | -0.947143 | 408 | 158.77 | 0.389142 |  |
| GO:0006915\_apoptosis | RB1CC1 | 427 | 8 | 1.627856 | -0.947143 | 408 | 158.77 | 0.389142 |  |
| GO:0006915\_apoptosis | FOXC1 | 427 | 8 | 1.627856 | -0.947143 | 408 | 158.77 | 0.389142 |  |
| GO:0006915\_apoptosis | CTNNB1 | 427 | 8 | 1.627856 | -0.947143 | 408 | 158.77 | 0.389142 |  |
| GO:0006915\_apoptosis | CITED2 | 427 | 8 | 1.627856 | -0.947143 | 408 | 158.77 | 0.389142 |  |
| GO:0006915\_apoptosis | TGFB2 | 427 | 8 | 1.627856 | -0.947143 | 408 | 158.77 | 0.389142 |  |
| GO:0009628\_response\_to\_abiotic\_stimulus | XRCC4 | 162 | 4 | 2.145353 | -0.940398 | 409 | 159.55 | 0.390098 |  |
| GO:0009628\_response\_to\_abiotic\_stimulus | RBP4 | 162 | 4 | 2.145353 | -0.940398 | 409 | 159.55 | 0.390098 |  |
| GO:0009628\_response\_to\_abiotic\_stimulus | TCFEC | 162 | 4 | 2.145353 | -0.940398 | 409 | 159.55 | 0.390098 |  |
| GO:0009628\_response\_to\_abiotic\_stimulus | RGS9BP | 162 | 4 | 2.145353 | -0.940398 | 409 | 159.55 | 0.390098 |  |
| GO:0007601\_visual\_perception | RBP4 | 51 | 2 | 3.407325 | -0.935309 | 412 | 160.36 | 0.389223 |  |
| GO:0007601\_visual\_perception | RGS9BP | 51 | 2 | 3.407325 | -0.935309 | 412 | 160.36 | 0.389223 |  |
| GO:0032583\_regulation\_of\_gene-specific\_transcription | BMP2 | 51 | 2 | 3.407325 | -0.935309 | 412 | 160.36 | 0.389223 |  |
| GO:0032583\_regulation\_of\_gene-specific\_transcription | CITED2 | 51 | 2 | 3.407325 | -0.935309 | 412 | 160.36 | 0.389223 |  |
| GO:0043408\_regulation\_of\_MAPKKK\_cascade | ZEB2 | 51 | 2 | 3.407325 | -0.935309 | 412 | 160.36 | 0.389223 |  |
| GO:0043408\_regulation\_of\_MAPKKK\_cascade | CTNNB1 | 51 | 2 | 3.407325 | -0.935309 | 412 | 160.36 | 0.389223 |  |
| GO:0042981\_regulation\_of\_apoptosis | XRCC4 | 360 | 7 | 1.689465 | -0.934282 | 413 | 160.41 | 0.388402 |  |
| GO:0042981\_regulation\_of\_apoptosis | ZC3HC1 | 360 | 7 | 1.689465 | -0.934282 | 413 | 160.41 | 0.388402 |  |
| GO:0042981\_regulation\_of\_apoptosis | SGK3 | 360 | 7 | 1.689465 | -0.934282 | 413 | 160.41 | 0.388402 |  |
| GO:0042981\_regulation\_of\_apoptosis | RB1CC1 | 360 | 7 | 1.689465 | -0.934282 | 413 | 160.41 | 0.388402 |  |
| GO:0042981\_regulation\_of\_apoptosis | FOXC1 | 360 | 7 | 1.689465 | -0.934282 | 413 | 160.41 | 0.388402 |  |
| GO:0042981\_regulation\_of\_apoptosis | CITED2 | 360 | 7 | 1.689465 | -0.934282 | 413 | 160.41 | 0.388402 |  |
| GO:0042981\_regulation\_of\_apoptosis | TGFB2 | 360 | 7 | 1.689465 | -0.934282 | 413 | 160.41 | 0.388402 |  |
| GO:0050767\_regulation\_of\_neurogenesis | XRCC4 | 104 | 3 | 2.506350 | -0.932525 | 414 | 160.76 | 0.388309 |  |
| GO:0050767\_regulation\_of\_neurogenesis | BMP2 | 104 | 3 | 2.506350 | -0.932525 | 414 | 160.76 | 0.388309 |  |
| GO:0050767\_regulation\_of\_neurogenesis | DLL1 | 104 | 3 | 2.506350 | -0.932525 | 414 | 160.76 | 0.388309 |  |
| GO:0007229\_integrin-mediated\_signaling\_pathway | VAV3 | 11 | 1 | 7.898799 | -0.921962 | 425 | 170.45 | 0.401059 |  |
| GO:0008354\_germ\_cell\_migration | FOXC1 | 11 | 1 | 7.898799 | -0.921962 | 425 | 170.45 | 0.401059 |  |
| GO:0014013\_regulation\_of\_gliogenesis | BMP2 | 11 | 1 | 7.898799 | -0.921962 | 425 | 170.45 | 0.401059 |  |
| GO:0021846\_cell\_proliferation\_in\_forebrain | ZEB2 | 11 | 1 | 7.898799 | -0.921962 | 425 | 170.45 | 0.401059 |  |
| GO:0030856\_regulation\_of\_epithelial\_cell\_differentiation | CTNNB1 | 11 | 1 | 7.898799 | -0.921962 | 425 | 170.45 | 0.401059 |  |
| GO:0042401\_biogenic\_amine\_biosynthetic\_process | TGFB2 | 11 | 1 | 7.898799 | -0.921962 | 425 | 170.45 | 0.401059 |  |
| GO:0045685\_regulation\_of\_glial\_cell\_differentiation | BMP2 | 11 | 1 | 7.898799 | -0.921962 | 425 | 170.45 | 0.401059 |  |
| GO:0045834\_positive\_regulation\_of\_lipid\_metabolic\_process | APOE | 11 | 1 | 7.898799 | -0.921962 | 425 | 170.45 | 0.401059 |  |
| GO:0048678\_response\_to\_axon\_injury | APOE | 11 | 1 | 7.898799 | -0.921962 | 425 | 170.45 | 0.401059 |  |
| GO:0048745\_smooth\_muscle\_tissue\_development | SCHIP1 | 11 | 1 | 7.898799 | -0.921962 | 425 | 170.45 | 0.401059 |  |
| GO:0060767\_epithelial\_cell\_proliferation\_involved\_in\_prostate\_gland\_development | CTNNB1 | 11 | 1 | 7.898799 | -0.921962 | 425 | 170.45 | 0.401059 |  |
| GO:0050953\_sensory\_perception\_of\_light\_stimulus | RBP4 | 52 | 2 | 3.341800 | -0.921366 | 426 | 171.12 | 0.401690 |  |
| GO:0050953\_sensory\_perception\_of\_light\_stimulus | RGS9BP | 52 | 2 | 3.341800 | -0.921366 | 426 | 171.12 | 0.401690 |  |
| GO:0012501\_programmed\_cell\_death | XRCC4 | 433 | 8 | 1.605299 | -0.921245 | 427 | 171.29 | 0.401148 |  |
| GO:0012501\_programmed\_cell\_death | ZC3HC1 | 433 | 8 | 1.605299 | -0.921245 | 427 | 171.29 | 0.401148 |  |
| GO:0012501\_programmed\_cell\_death | SGK3 | 433 | 8 | 1.605299 | -0.921245 | 427 | 171.29 | 0.401148 |  |
| GO:0012501\_programmed\_cell\_death | RB1CC1 | 433 | 8 | 1.605299 | -0.921245 | 427 | 171.29 | 0.401148 |  |
| GO:0012501\_programmed\_cell\_death | FOXC1 | 433 | 8 | 1.605299 | -0.921245 | 427 | 171.29 | 0.401148 |  |
| GO:0012501\_programmed\_cell\_death | CTNNB1 | 433 | 8 | 1.605299 | -0.921245 | 427 | 171.29 | 0.401148 |  |
| GO:0012501\_programmed\_cell\_death | CITED2 | 433 | 8 | 1.605299 | -0.921245 | 427 | 171.29 | 0.401148 |  |
| GO:0012501\_programmed\_cell\_death | TGFB2 | 433 | 8 | 1.605299 | -0.921245 | 427 | 171.29 | 0.401148 |  |
| GO:0007167\_enzyme\_linked\_receptor\_protein\_signaling\_pathway | SCHIP1 | 229 | 5 | 1.897092 | -0.915798 | 428 | 172.03 | 0.401939 |  |
| GO:0007167\_enzyme\_linked\_receptor\_protein\_signaling\_pathway | BMP2 | 229 | 5 | 1.897092 | -0.915798 | 428 | 172.03 | 0.401939 |  |
| GO:0007167\_enzyme\_linked\_receptor\_protein\_signaling\_pathway | FOXC1 | 229 | 5 | 1.897092 | -0.915798 | 428 | 172.03 | 0.401939 |  |
| GO:0007167\_enzyme\_linked\_receptor\_protein\_signaling\_pathway | TGFB2 | 229 | 5 | 1.897092 | -0.915798 | 428 | 172.03 | 0.401939 |  |
| GO:0007167\_enzyme\_linked\_receptor\_protein\_signaling\_pathway | CITED2 | 229 | 5 | 1.897092 | -0.915798 | 428 | 172.03 | 0.401939 |  |
| GO:0010941\_regulation\_of\_cell\_death | XRCC4 | 365 | 7 | 1.666322 | -0.910964 | 430 | 172.79 | 0.401837 |  |
| GO:0010941\_regulation\_of\_cell\_death | ZC3HC1 | 365 | 7 | 1.666322 | -0.910964 | 430 | 172.79 | 0.401837 |  |
| GO:0010941\_regulation\_of\_cell\_death | SGK3 | 365 | 7 | 1.666322 | -0.910964 | 430 | 172.79 | 0.401837 |  |
| GO:0010941\_regulation\_of\_cell\_death | RB1CC1 | 365 | 7 | 1.666322 | -0.910964 | 430 | 172.79 | 0.401837 |  |
| GO:0010941\_regulation\_of\_cell\_death | FOXC1 | 365 | 7 | 1.666322 | -0.910964 | 430 | 172.79 | 0.401837 |  |
| GO:0010941\_regulation\_of\_cell\_death | CITED2 | 365 | 7 | 1.666322 | -0.910964 | 430 | 172.79 | 0.401837 |  |
| GO:0010941\_regulation\_of\_cell\_death | TGFB2 | 365 | 7 | 1.666322 | -0.910964 | 430 | 172.79 | 0.401837 |  |
| GO:0043067\_regulation\_of\_programmed\_cell\_death | XRCC4 | 365 | 7 | 1.666322 | -0.910964 | 430 | 172.79 | 0.401837 |  |
| GO:0043067\_regulation\_of\_programmed\_cell\_death | ZC3HC1 | 365 | 7 | 1.666322 | -0.910964 | 430 | 172.79 | 0.401837 |  |
| GO:0043067\_regulation\_of\_programmed\_cell\_death | SGK3 | 365 | 7 | 1.666322 | -0.910964 | 430 | 172.79 | 0.401837 |  |
| GO:0043067\_regulation\_of\_programmed\_cell\_death | RB1CC1 | 365 | 7 | 1.666322 | -0.910964 | 430 | 172.79 | 0.401837 |  |
| GO:0043067\_regulation\_of\_programmed\_cell\_death | FOXC1 | 365 | 7 | 1.666322 | -0.910964 | 430 | 172.79 | 0.401837 |  |
| GO:0043067\_regulation\_of\_programmed\_cell\_death | CITED2 | 365 | 7 | 1.666322 | -0.910964 | 430 | 172.79 | 0.401837 |  |
| GO:0043067\_regulation\_of\_programmed\_cell\_death | TGFB2 | 365 | 7 | 1.666322 | -0.910964 | 430 | 172.79 | 0.401837 |  |
| GO:0016070\_RNA\_metabolic\_process | EGR1 | 658 | 11 | 1.452515 | -0.899739 | 431 | 174.12 | 0.403991 |  |
| GO:0016070\_RNA\_metabolic\_process | BMP2 | 658 | 11 | 1.452515 | -0.899739 | 431 | 174.12 | 0.403991 |  |
| GO:0016070\_RNA\_metabolic\_process | KLF12 | 658 | 11 | 1.452515 | -0.899739 | 431 | 174.12 | 0.403991 |  |
| GO:0016070\_RNA\_metabolic\_process | JUN | 658 | 11 | 1.452515 | -0.899739 | 431 | 174.12 | 0.403991 |  |
| GO:0016070\_RNA\_metabolic\_process | TBX5 | 658 | 11 | 1.452515 | -0.899739 | 431 | 174.12 | 0.403991 |  |
| GO:0016070\_RNA\_metabolic\_process | FOXC1 | 658 | 11 | 1.452515 | -0.899739 | 431 | 174.12 | 0.403991 |  |
| GO:0016070\_RNA\_metabolic\_process | RORA | 658 | 11 | 1.452515 | -0.899739 | 431 | 174.12 | 0.403991 |  |
| GO:0016070\_RNA\_metabolic\_process | HBA-X | 658 | 11 | 1.452515 | -0.899739 | 431 | 174.12 | 0.403991 |  |
| GO:0016070\_RNA\_metabolic\_process | FOXP4 | 658 | 11 | 1.452515 | -0.899739 | 431 | 174.12 | 0.403991 |  |
| GO:0016070\_RNA\_metabolic\_process | CITED2 | 658 | 11 | 1.452515 | -0.899739 | 431 | 174.12 | 0.403991 |  |
| GO:0016070\_RNA\_metabolic\_process | CTNNB1 | 658 | 11 | 1.452515 | -0.899739 | 431 | 174.12 | 0.403991 |  |
| GO:0007265\_Ras\_protein\_signal\_transduction | RHOJ | 54 | 2 | 3.218029 | -0.894443 | 433 | 175.58 | 0.405497 |  |
| GO:0007265\_Ras\_protein\_signal\_transduction | CDC42EP4 | 54 | 2 | 3.218029 | -0.894443 | 433 | 175.58 | 0.405497 |  |
| GO:0044271\_nitrogen\_compound\_biosynthetic\_process | RORA | 54 | 2 | 3.218029 | -0.894443 | 433 | 175.58 | 0.405497 |  |
| GO:0044271\_nitrogen\_compound\_biosynthetic\_process | TGFB2 | 54 | 2 | 3.218029 | -0.894443 | 433 | 175.58 | 0.405497 |  |
| GO:0031326\_regulation\_of\_cellular\_biosynthetic\_process | EGR1 | 812 | 13 | 1.391045 | -0.892665 | 434 | 175.63 | 0.404677 |  |
| GO:0031326\_regulation\_of\_cellular\_biosynthetic\_process | BMP2 | 812 | 13 | 1.391045 | -0.892665 | 434 | 175.63 | 0.404677 |  |
| GO:0031326\_regulation\_of\_cellular\_biosynthetic\_process | KLF12 | 812 | 13 | 1.391045 | -0.892665 | 434 | 175.63 | 0.404677 |  |
| GO:0031326\_regulation\_of\_cellular\_biosynthetic\_process | TBX5 | 812 | 13 | 1.391045 | -0.892665 | 434 | 175.63 | 0.404677 |  |
| GO:0031326\_regulation\_of\_cellular\_biosynthetic\_process | RORA | 812 | 13 | 1.391045 | -0.892665 | 434 | 175.63 | 0.404677 |  |
| GO:0031326\_regulation\_of\_cellular\_biosynthetic\_process | FOXP4 | 812 | 13 | 1.391045 | -0.892665 | 434 | 175.63 | 0.404677 |  |
| GO:0031326\_regulation\_of\_cellular\_biosynthetic\_process | CTNNB1 | 812 | 13 | 1.391045 | -0.892665 | 434 | 175.63 | 0.404677 |  |
| GO:0031326\_regulation\_of\_cellular\_biosynthetic\_process | CITED2 | 812 | 13 | 1.391045 | -0.892665 | 434 | 175.63 | 0.404677 |  |
| GO:0031326\_regulation\_of\_cellular\_biosynthetic\_process | APOE | 812 | 13 | 1.391045 | -0.892665 | 434 | 175.63 | 0.404677 |  |
| GO:0031326\_regulation\_of\_cellular\_biosynthetic\_process | JUN | 812 | 13 | 1.391045 | -0.892665 | 434 | 175.63 | 0.404677 |  |
| GO:0031326\_regulation\_of\_cellular\_biosynthetic\_process | FOXC1 | 812 | 13 | 1.391045 | -0.892665 | 434 | 175.63 | 0.404677 |  |
| GO:0031326\_regulation\_of\_cellular\_biosynthetic\_process | HBA-X | 812 | 13 | 1.391045 | -0.892665 | 434 | 175.63 | 0.404677 |  |
| GO:0031326\_regulation\_of\_cellular\_biosynthetic\_process | C1D | 812 | 13 | 1.391045 | -0.892665 | 434 | 175.63 | 0.404677 |  |
| GO:0030029\_actin\_filament-based\_process | RHOJ | 109 | 3 | 2.391380 | -0.887704 | 435 | 176.1 | 0.404828 |  |
| GO:0030029\_actin\_filament-based\_process | PLEK | 109 | 3 | 2.391380 | -0.887704 | 435 | 176.1 | 0.404828 |  |
| GO:0030029\_actin\_filament-based\_process | PROX1 | 109 | 3 | 2.391380 | -0.887704 | 435 | 176.1 | 0.404828 |  |
| GO:0051239\_regulation\_of\_multicellular\_organismal\_process | WNT5A | 587 | 10 | 1.480184 | -0.887326 | 436 | 176.15 | 0.404014 |  |
| GO:0051239\_regulation\_of\_multicellular\_organismal\_process | XRCC4 | 587 | 10 | 1.480184 | -0.887326 | 436 | 176.15 | 0.404014 |  |
| GO:0051239\_regulation\_of\_multicellular\_organismal\_process | RBP4 | 587 | 10 | 1.480184 | -0.887326 | 436 | 176.15 | 0.404014 |  |
| GO:0051239\_regulation\_of\_multicellular\_organismal\_process | BMP2 | 587 | 10 | 1.480184 | -0.887326 | 436 | 176.15 | 0.404014 |  |
| GO:0051239\_regulation\_of\_multicellular\_organismal\_process | PTPRM | 587 | 10 | 1.480184 | -0.887326 | 436 | 176.15 | 0.404014 |  |
| GO:0051239\_regulation\_of\_multicellular\_organismal\_process | PLEK | 587 | 10 | 1.480184 | -0.887326 | 436 | 176.15 | 0.404014 |  |
| GO:0051239\_regulation\_of\_multicellular\_organismal\_process | APOE | 587 | 10 | 1.480184 | -0.887326 | 436 | 176.15 | 0.404014 |  |
| GO:0051239\_regulation\_of\_multicellular\_organismal\_process | DLL1 | 587 | 10 | 1.480184 | -0.887326 | 436 | 176.15 | 0.404014 |  |
| GO:0051239\_regulation\_of\_multicellular\_organismal\_process | PROX1 | 587 | 10 | 1.480184 | -0.887326 | 436 | 176.15 | 0.404014 |  |
| GO:0051239\_regulation\_of\_multicellular\_organismal\_process | CTNNB1 | 587 | 10 | 1.480184 | -0.887326 | 436 | 176.15 | 0.404014 |  |
| GO:0002089\_lens\_morphogenesis\_in\_camera-type\_eye | PROX1 | 12 | 1 | 7.240566 | -0.886590 | 454 | 186.74 | 0.411322 |  |
| GO:0002244\_hemopoietic\_progenitor\_cell\_differentiation | XRCC4 | 12 | 1 | 7.240566 | -0.886590 | 454 | 186.74 | 0.411322 |  |
| GO:0006094\_gluconeogenesis | RBP4 | 12 | 1 | 7.240566 | -0.886590 | 454 | 186.74 | 0.411322 |  |
| GO:0019319\_hexose\_biosynthetic\_process | RBP4 | 12 | 1 | 7.240566 | -0.886590 | 454 | 186.74 | 0.411322 |  |
| GO:0021680\_cerebellar\_Purkinje\_cell\_layer\_development | RORA | 12 | 1 | 7.240566 | -0.886590 | 454 | 186.74 | 0.411322 |  |
| GO:0021697\_cerebellar\_cortex\_formation | RORA | 12 | 1 | 7.240566 | -0.886590 | 454 | 186.74 | 0.411322 |  |
| GO:0030239\_myofibril\_assembly | PROX1 | 12 | 1 | 7.240566 | -0.886590 | 454 | 186.74 | 0.411322 |  |
| GO:0033598\_mammary\_gland\_epithelial\_cell\_proliferation | WNT5A | 12 | 1 | 7.240566 | -0.886590 | 454 | 186.74 | 0.411322 |  |
| GO:0035050\_embryonic\_heart\_tube\_development | FOXC1 | 12 | 1 | 7.240566 | -0.886590 | 454 | 186.74 | 0.411322 |  |
| GO:0043624\_cellular\_protein\_complex\_disassembly | PLEK | 12 | 1 | 7.240566 | -0.886590 | 454 | 186.74 | 0.411322 |  |
| GO:0045995\_regulation\_of\_embryonic\_development | WNT5A | 12 | 1 | 7.240566 | -0.886590 | 454 | 186.74 | 0.411322 |  |
| GO:0046068\_cGMP\_metabolic\_process | RORA | 12 | 1 | 7.240566 | -0.886590 | 454 | 186.74 | 0.411322 |  |
| GO:0048821\_erythrocyte\_development | HBA-X | 12 | 1 | 7.240566 | -0.886590 | 454 | 186.74 | 0.411322 |  |
| GO:0048854\_brain\_morphogenesis | WNT5A | 12 | 1 | 7.240566 | -0.886590 | 454 | 186.74 | 0.411322 |  |
| GO:0051261\_protein\_depolymerization | PLEK | 12 | 1 | 7.240566 | -0.886590 | 454 | 186.74 | 0.411322 |  |
| GO:0055002\_striated\_muscle\_cell\_development | PROX1 | 12 | 1 | 7.240566 | -0.886590 | 454 | 186.74 | 0.411322 |  |
| GO:0060042\_retina\_morphogenesis\_in\_camera-type\_eye | RBP4 | 12 | 1 | 7.240566 | -0.886590 | 454 | 186.74 | 0.411322 |  |
| GO:0060572\_morphogenesis\_of\_an\_epithelial\_bud | WNT5A | 12 | 1 | 7.240566 | -0.886590 | 454 | 186.74 | 0.411322 |  |
| GO:0051246\_regulation\_of\_protein\_metabolic\_process | PLEK | 170 | 4 | 2.044395 | -0.884370 | 455 | 186.88 | 0.410725 |  |
| GO:0051246\_regulation\_of\_protein\_metabolic\_process | RB1CC1 | 170 | 4 | 2.044395 | -0.884370 | 455 | 186.88 | 0.410725 |  |
| GO:0051246\_regulation\_of\_protein\_metabolic\_process | JUN | 170 | 4 | 2.044395 | -0.884370 | 455 | 186.88 | 0.410725 |  |
| GO:0051246\_regulation\_of\_protein\_metabolic\_process | ZEB2 | 170 | 4 | 2.044395 | -0.884370 | 455 | 186.88 | 0.410725 |  |
| GO:0009889\_regulation\_of\_biosynthetic\_process | EGR1 | 815 | 13 | 1.385924 | -0.883390 | 456 | 187.06 | 0.410219 |  |
| GO:0009889\_regulation\_of\_biosynthetic\_process | BMP2 | 815 | 13 | 1.385924 | -0.883390 | 456 | 187.06 | 0.410219 |  |
| GO:0009889\_regulation\_of\_biosynthetic\_process | KLF12 | 815 | 13 | 1.385924 | -0.883390 | 456 | 187.06 | 0.410219 |  |
| GO:0009889\_regulation\_of\_biosynthetic\_process | TBX5 | 815 | 13 | 1.385924 | -0.883390 | 456 | 187.06 | 0.410219 |  |
| GO:0009889\_regulation\_of\_biosynthetic\_process | RORA | 815 | 13 | 1.385924 | -0.883390 | 456 | 187.06 | 0.410219 |  |
| GO:0009889\_regulation\_of\_biosynthetic\_process | FOXP4 | 815 | 13 | 1.385924 | -0.883390 | 456 | 187.06 | 0.410219 |  |
| GO:0009889\_regulation\_of\_biosynthetic\_process | CTNNB1 | 815 | 13 | 1.385924 | -0.883390 | 456 | 187.06 | 0.410219 |  |
| GO:0009889\_regulation\_of\_biosynthetic\_process | CITED2 | 815 | 13 | 1.385924 | -0.883390 | 456 | 187.06 | 0.410219 |  |
| GO:0009889\_regulation\_of\_biosynthetic\_process | APOE | 815 | 13 | 1.385924 | -0.883390 | 456 | 187.06 | 0.410219 |  |
| GO:0009889\_regulation\_of\_biosynthetic\_process | JUN | 815 | 13 | 1.385924 | -0.883390 | 456 | 187.06 | 0.410219 |  |
| GO:0009889\_regulation\_of\_biosynthetic\_process | FOXC1 | 815 | 13 | 1.385924 | -0.883390 | 456 | 187.06 | 0.410219 |  |
| GO:0009889\_regulation\_of\_biosynthetic\_process | HBA-X | 815 | 13 | 1.385924 | -0.883390 | 456 | 187.06 | 0.410219 |  |
| GO:0009889\_regulation\_of\_biosynthetic\_process | C1D | 815 | 13 | 1.385924 | -0.883390 | 456 | 187.06 | 0.410219 |  |
| GO:0048568\_embryonic\_organ\_development | RBP4 | 55 | 2 | 3.159520 | -0.881438 | 457 | 187.82 | 0.410985 |  |
| GO:0048568\_embryonic\_organ\_development | PROX1 | 55 | 2 | 3.159520 | -0.881438 | 457 | 187.82 | 0.410985 |  |
| GO:0008219\_cell\_death | XRCC4 | 444 | 8 | 1.565528 | -0.875487 | 458 | 188.58 | 0.411747 |  |
| GO:0008219\_cell\_death | ZC3HC1 | 444 | 8 | 1.565528 | -0.875487 | 458 | 188.58 | 0.411747 |  |
| GO:0008219\_cell\_death | SGK3 | 444 | 8 | 1.565528 | -0.875487 | 458 | 188.58 | 0.411747 |  |
| GO:0008219\_cell\_death | RB1CC1 | 444 | 8 | 1.565528 | -0.875487 | 458 | 188.58 | 0.411747 |  |
| GO:0008219\_cell\_death | FOXC1 | 444 | 8 | 1.565528 | -0.875487 | 458 | 188.58 | 0.411747 |  |
| GO:0008219\_cell\_death | CTNNB1 | 444 | 8 | 1.565528 | -0.875487 | 458 | 188.58 | 0.411747 |  |
| GO:0008219\_cell\_death | CITED2 | 444 | 8 | 1.565528 | -0.875487 | 458 | 188.58 | 0.411747 |  |
| GO:0008219\_cell\_death | TGFB2 | 444 | 8 | 1.565528 | -0.875487 | 458 | 188.58 | 0.411747 |  |
| GO:0048705\_skeletal\_system\_morphogenesis | SCHIP1 | 111 | 3 | 2.348292 | -0.870559 | 459 | 188.87 | 0.411481 |  |
| GO:0048705\_skeletal\_system\_morphogenesis | PRRX1 | 111 | 3 | 2.348292 | -0.870559 | 459 | 188.87 | 0.411481 |  |
| GO:0048705\_skeletal\_system\_morphogenesis | TGFB2 | 111 | 3 | 2.348292 | -0.870559 | 459 | 188.87 | 0.411481 |  |
| GO:0006790\_sulfur\_metabolic\_process | A930038C07RIK | 56 | 2 | 3.103100 | -0.868723 | 461 | 190.17 | 0.412516 |  |
| GO:0006790\_sulfur\_metabolic\_process | DCN | 56 | 2 | 3.103100 | -0.868723 | 461 | 190.17 | 0.412516 |  |
| GO:0050678\_regulation\_of\_epithelial\_cell\_proliferation | WNT5A | 56 | 2 | 3.103100 | -0.868723 | 461 | 190.17 | 0.412516 |  |
| GO:0050678\_regulation\_of\_epithelial\_cell\_proliferation | CTNNB1 | 56 | 2 | 3.103100 | -0.868723 | 461 | 190.17 | 0.412516 |  |
| GO:0008152\_metabolic\_process | WNT5A | 2133 | 29 | 1.181302 | -0.863563 | 462 | 190.54 | 0.412424 |  |
| GO:0008152\_metabolic\_process | XRCC4 | 2133 | 29 | 1.181302 | -0.863563 | 462 | 190.54 | 0.412424 |  |
| GO:0008152\_metabolic\_process | RBP4 | 2133 | 29 | 1.181302 | -0.863563 | 462 | 190.54 | 0.412424 |  |
| GO:0008152\_metabolic\_process | SCHIP1 | 2133 | 29 | 1.181302 | -0.863563 | 462 | 190.54 | 0.412424 |  |
| GO:0008152\_metabolic\_process | ZEB2 | 2133 | 29 | 1.181302 | -0.863563 | 462 | 190.54 | 0.412424 |  |
| GO:0008152\_metabolic\_process | RORA | 2133 | 29 | 1.181302 | -0.863563 | 462 | 190.54 | 0.412424 |  |
| GO:0008152\_metabolic\_process | DCN | 2133 | 29 | 1.181302 | -0.863563 | 462 | 190.54 | 0.412424 |  |
| GO:0008152\_metabolic\_process | PRKG1 | 2133 | 29 | 1.181302 | -0.863563 | 462 | 190.54 | 0.412424 |  |
| GO:0008152\_metabolic\_process | CITED2 | 2133 | 29 | 1.181302 | -0.863563 | 462 | 190.54 | 0.412424 |  |
| GO:0008152\_metabolic\_process | CTNNB1 | 2133 | 29 | 1.181302 | -0.863563 | 462 | 190.54 | 0.412424 |  |
| GO:0008152\_metabolic\_process | TGFB2 | 2133 | 29 | 1.181302 | -0.863563 | 462 | 190.54 | 0.412424 |  |
| GO:0008152\_metabolic\_process | A930038C07RIK | 2133 | 29 | 1.181302 | -0.863563 | 462 | 190.54 | 0.412424 |  |
| GO:0008152\_metabolic\_process | APOE | 2133 | 29 | 1.181302 | -0.863563 | 462 | 190.54 | 0.412424 |  |
| GO:0008152\_metabolic\_process | RB1CC1 | 2133 | 29 | 1.181302 | -0.863563 | 462 | 190.54 | 0.412424 |  |
| GO:0008152\_metabolic\_process | HBA-X | 2133 | 29 | 1.181302 | -0.863563 | 462 | 190.54 | 0.412424 |  |
| GO:0008152\_metabolic\_process | EGR1 | 2133 | 29 | 1.181302 | -0.863563 | 462 | 190.54 | 0.412424 |  |
| GO:0008152\_metabolic\_process | BMP2 | 2133 | 29 | 1.181302 | -0.863563 | 462 | 190.54 | 0.412424 |  |
| GO:0008152\_metabolic\_process | ADSSL1 | 2133 | 29 | 1.181302 | -0.863563 | 462 | 190.54 | 0.412424 |  |
| GO:0008152\_metabolic\_process | VAV3 | 2133 | 29 | 1.181302 | -0.863563 | 462 | 190.54 | 0.412424 |  |
| GO:0008152\_metabolic\_process | KLF12 | 2133 | 29 | 1.181302 | -0.863563 | 462 | 190.54 | 0.412424 |  |
| GO:0008152\_metabolic\_process | PLEK | 2133 | 29 | 1.181302 | -0.863563 | 462 | 190.54 | 0.412424 |  |
| GO:0008152\_metabolic\_process | TBX5 | 2133 | 29 | 1.181302 | -0.863563 | 462 | 190.54 | 0.412424 |  |
| GO:0008152\_metabolic\_process | CFTR | 2133 | 29 | 1.181302 | -0.863563 | 462 | 190.54 | 0.412424 |  |
| GO:0008152\_metabolic\_process | FOXP4 | 2133 | 29 | 1.181302 | -0.863563 | 462 | 190.54 | 0.412424 |  |
| GO:0008152\_metabolic\_process | BTG2 | 2133 | 29 | 1.181302 | -0.863563 | 462 | 190.54 | 0.412424 |  |
| GO:0008152\_metabolic\_process | JUN | 2133 | 29 | 1.181302 | -0.863563 | 462 | 190.54 | 0.412424 |  |
| GO:0008152\_metabolic\_process | LRP8 | 2133 | 29 | 1.181302 | -0.863563 | 462 | 190.54 | 0.412424 |  |
| GO:0008152\_metabolic\_process | FOXC1 | 2133 | 29 | 1.181302 | -0.863563 | 462 | 190.54 | 0.412424 |  |
| GO:0008152\_metabolic\_process | C1D | 2133 | 29 | 1.181302 | -0.863563 | 462 | 190.54 | 0.412424 |  |
| GO:0010556\_regulation\_of\_macromolecule\_biosynthetic\_process | EGR1 | 745 | 12 | 1.399519 | -0.862601 | 463 | 190.75 | 0.411987 |  |
| GO:0010556\_regulation\_of\_macromolecule\_biosynthetic\_process | BMP2 | 745 | 12 | 1.399519 | -0.862601 | 463 | 190.75 | 0.411987 |  |
| GO:0010556\_regulation\_of\_macromolecule\_biosynthetic\_process | KLF12 | 745 | 12 | 1.399519 | -0.862601 | 463 | 190.75 | 0.411987 |  |
| GO:0010556\_regulation\_of\_macromolecule\_biosynthetic\_process | JUN | 745 | 12 | 1.399519 | -0.862601 | 463 | 190.75 | 0.411987 |  |
| GO:0010556\_regulation\_of\_macromolecule\_biosynthetic\_process | TBX5 | 745 | 12 | 1.399519 | -0.862601 | 463 | 190.75 | 0.411987 |  |
| GO:0010556\_regulation\_of\_macromolecule\_biosynthetic\_process | FOXC1 | 745 | 12 | 1.399519 | -0.862601 | 463 | 190.75 | 0.411987 |  |
| GO:0010556\_regulation\_of\_macromolecule\_biosynthetic\_process | RORA | 745 | 12 | 1.399519 | -0.862601 | 463 | 190.75 | 0.411987 |  |
| GO:0010556\_regulation\_of\_macromolecule\_biosynthetic\_process | HBA-X | 745 | 12 | 1.399519 | -0.862601 | 463 | 190.75 | 0.411987 |  |
| GO:0010556\_regulation\_of\_macromolecule\_biosynthetic\_process | FOXP4 | 745 | 12 | 1.399519 | -0.862601 | 463 | 190.75 | 0.411987 |  |
| GO:0010556\_regulation\_of\_macromolecule\_biosynthetic\_process | C1D | 745 | 12 | 1.399519 | -0.862601 | 463 | 190.75 | 0.411987 |  |
| GO:0010556\_regulation\_of\_macromolecule\_biosynthetic\_process | CTNNB1 | 745 | 12 | 1.399519 | -0.862601 | 463 | 190.75 | 0.411987 |  |
| GO:0010556\_regulation\_of\_macromolecule\_biosynthetic\_process | CITED2 | 745 | 12 | 1.399519 | -0.862601 | 463 | 190.75 | 0.411987 |  |
| GO:0043549\_regulation\_of\_kinase\_activity | VAV3 | 112 | 3 | 2.327325 | -0.862147 | 464 | 191.02 | 0.411681 |  |
| GO:0043549\_regulation\_of\_kinase\_activity | LRP8 | 112 | 3 | 2.327325 | -0.862147 | 464 | 191.02 | 0.411681 |  |
| GO:0043549\_regulation\_of\_kinase\_activity | ZEB2 | 112 | 3 | 2.327325 | -0.862147 | 464 | 191.02 | 0.411681 |  |
| GO:0022414\_reproductive\_process | WNT5A | 376 | 7 | 1.617573 | -0.861644 | 465 | 191.08 | 0.410925 |  |
| GO:0022414\_reproductive\_process | SEPT4 | 376 | 7 | 1.617573 | -0.861644 | 465 | 191.08 | 0.410925 |  |
| GO:0022414\_reproductive\_process | RBP4 | 376 | 7 | 1.617573 | -0.861644 | 465 | 191.08 | 0.410925 |  |
| GO:0022414\_reproductive\_process | SPACA3 | 376 | 7 | 1.617573 | -0.861644 | 465 | 191.08 | 0.410925 |  |
| GO:0022414\_reproductive\_process | FOXC1 | 376 | 7 | 1.617573 | -0.861644 | 465 | 191.08 | 0.410925 |  |
| GO:0022414\_reproductive\_process | CITED2 | 376 | 7 | 1.617573 | -0.861644 | 465 | 191.08 | 0.410925 |  |
| GO:0022414\_reproductive\_process | CTNNB1 | 376 | 7 | 1.617573 | -0.861644 | 465 | 191.08 | 0.410925 |  |
| GO:0001892\_embryonic\_placenta\_development | RSPO3 | 57 | 2 | 3.048659 | -0.856288 | 466 | 192.82 | 0.413777 |  |
| GO:0001892\_embryonic\_placenta\_development | CITED2 | 57 | 2 | 3.048659 | -0.856288 | 466 | 192.82 | 0.413777 |  |
| GO:0006090\_pyruvate\_metabolic\_process | RBP4 | 13 | 1 | 6.683599 | -0.854239 | 478 | 202.83 | 0.424331 |  |
| GO:0021533\_cell\_differentiation\_in\_hindbrain | RORA | 13 | 1 | 6.683599 | -0.854239 | 478 | 202.83 | 0.424331 |  |
| GO:0031032\_actomyosin\_structure\_organization | PROX1 | 13 | 1 | 6.683599 | -0.854239 | 478 | 202.83 | 0.424331 |  |
| GO:0032330\_regulation\_of\_chondrocyte\_differentiation | CTNNB1 | 13 | 1 | 6.683599 | -0.854239 | 478 | 202.83 | 0.424331 |  |
| GO:0042474\_middle\_ear\_morphogenesis | PRRX1 | 13 | 1 | 6.683599 | -0.854239 | 478 | 202.83 | 0.424331 |  |
| GO:0043241\_protein\_complex\_disassembly | PLEK | 13 | 1 | 6.683599 | -0.854239 | 478 | 202.83 | 0.424331 |  |
| GO:0043244\_regulation\_of\_protein\_complex\_disassembly | PLEK | 13 | 1 | 6.683599 | -0.854239 | 478 | 202.83 | 0.424331 |  |
| GO:0048305\_immunoglobulin\_secretion | RBP4 | 13 | 1 | 6.683599 | -0.854239 | 478 | 202.83 | 0.424331 |  |
| GO:0050818\_regulation\_of\_coagulation | PLEK | 13 | 1 | 6.683599 | -0.854239 | 478 | 202.83 | 0.424331 |  |
| GO:0060324\_face\_development | SCHIP1 | 13 | 1 | 6.683599 | -0.854239 | 478 | 202.83 | 0.424331 |  |
| GO:0060560\_developmental\_growth\_involved\_in\_morphogenesis | WNT5A | 13 | 1 | 6.683599 | -0.854239 | 478 | 202.83 | 0.424331 |  |
| GO:0060742\_epithelial\_cell\_differentiation\_involved\_in\_prostate\_gland\_development | CTNNB1 | 13 | 1 | 6.683599 | -0.854239 | 478 | 202.83 | 0.424331 |  |
| GO:0040008\_regulation\_of\_growth | APOE | 113 | 3 | 2.306729 | -0.853838 | 479 | 203.02 | 0.423841 |  |
| GO:0040008\_regulation\_of\_growth | FOXC1 | 113 | 3 | 2.306729 | -0.853838 | 479 | 203.02 | 0.423841 |  |
| GO:0040008\_regulation\_of\_growth | PROX1 | 113 | 3 | 2.306729 | -0.853838 | 479 | 203.02 | 0.423841 |  |
| GO:0016265\_death | XRCC4 | 450 | 8 | 1.544654 | -0.851433 | 480 | 203.23 | 0.423396 |  |
| GO:0016265\_death | ZC3HC1 | 450 | 8 | 1.544654 | -0.851433 | 480 | 203.23 | 0.423396 |  |
| GO:0016265\_death | SGK3 | 450 | 8 | 1.544654 | -0.851433 | 480 | 203.23 | 0.423396 |  |
| GO:0016265\_death | RB1CC1 | 450 | 8 | 1.544654 | -0.851433 | 480 | 203.23 | 0.423396 |  |
| GO:0016265\_death | FOXC1 | 450 | 8 | 1.544654 | -0.851433 | 480 | 203.23 | 0.423396 |  |
| GO:0016265\_death | CTNNB1 | 450 | 8 | 1.544654 | -0.851433 | 480 | 203.23 | 0.423396 |  |
| GO:0016265\_death | CITED2 | 450 | 8 | 1.544654 | -0.851433 | 480 | 203.23 | 0.423396 |  |
| GO:0016265\_death | TGFB2 | 450 | 8 | 1.544654 | -0.851433 | 480 | 203.23 | 0.423396 |  |
| GO:0000122\_negative\_regulation\_of\_transcription\_from\_RNA\_polymerase\_II\_promoter | EGR1 | 175 | 4 | 1.985984 | -0.851323 | 481 | 203.61 | 0.423306 |  |
| GO:0000122\_negative\_regulation\_of\_transcription\_from\_RNA\_polymerase\_II\_promoter | HBA-X | 175 | 4 | 1.985984 | -0.851323 | 481 | 203.61 | 0.423306 |  |
| GO:0000122\_negative\_regulation\_of\_transcription\_from\_RNA\_polymerase\_II\_promoter | FOXP4 | 175 | 4 | 1.985984 | -0.851323 | 481 | 203.61 | 0.423306 |  |
| GO:0000122\_negative\_regulation\_of\_transcription\_from\_RNA\_polymerase\_II\_promoter | CTNNB1 | 175 | 4 | 1.985984 | -0.851323 | 481 | 203.61 | 0.423306 |  |
| GO:0000003\_reproduction | WNT5A | 379 | 7 | 1.604769 | -0.848648 | 482 | 203.67 | 0.422552 |  |
| GO:0000003\_reproduction | RBP4 | 379 | 7 | 1.604769 | -0.848648 | 482 | 203.67 | 0.422552 |  |
| GO:0000003\_reproduction | SEPT4 | 379 | 7 | 1.604769 | -0.848648 | 482 | 203.67 | 0.422552 |  |
| GO:0000003\_reproduction | SPACA3 | 379 | 7 | 1.604769 | -0.848648 | 482 | 203.67 | 0.422552 |  |
| GO:0000003\_reproduction | FOXC1 | 379 | 7 | 1.604769 | -0.848648 | 482 | 203.67 | 0.422552 |  |
| GO:0000003\_reproduction | CITED2 | 379 | 7 | 1.604769 | -0.848648 | 482 | 203.67 | 0.422552 |  |
| GO:0000003\_reproduction | CTNNB1 | 379 | 7 | 1.604769 | -0.848648 | 482 | 203.67 | 0.422552 |  |
| GO:0033043\_regulation\_of\_organelle\_organization | PLEK | 58 | 2 | 2.996096 | -0.844122 | 483 | 205.04 | 0.424513 |  |
| GO:0033043\_regulation\_of\_organelle\_organization | PROX1 | 58 | 2 | 2.996096 | -0.844122 | 483 | 205.04 | 0.424513 |  |
| GO:0051338\_regulation\_of\_transferase\_activity | VAV3 | 115 | 3 | 2.266612 | -0.837525 | 484 | 205.39 | 0.424360 |  |
| GO:0051338\_regulation\_of\_transferase\_activity | LRP8 | 115 | 3 | 2.266612 | -0.837525 | 484 | 205.39 | 0.424360 |  |
| GO:0051338\_regulation\_of\_transferase\_activity | ZEB2 | 115 | 3 | 2.266612 | -0.837525 | 484 | 205.39 | 0.424360 |  |
| GO:0048732\_gland\_development | WNT5A | 179 | 4 | 1.941604 | -0.825905 | 485 | 207.21 | 0.427237 |  |
| GO:0048732\_gland\_development | FOXC1 | 179 | 4 | 1.941604 | -0.825905 | 485 | 207.21 | 0.427237 |  |
| GO:0048732\_gland\_development | CTNNB1 | 179 | 4 | 1.941604 | -0.825905 | 485 | 207.21 | 0.427237 |  |
| GO:0048732\_gland\_development | CITED2 | 179 | 4 | 1.941604 | -0.825905 | 485 | 207.21 | 0.427237 |  |
| GO:0019219\_regulation\_of\_nucleobase\_\_nucleoside\_\_nucleotide\_and\_nucleic\_acid\_metabolic\_process | EGR1 | 757 | 12 | 1.377334 | -0.825474 | 486 | 207.26 | 0.426461 |  |
| GO:0019219\_regulation\_of\_nucleobase\_\_nucleoside\_\_nucleotide\_and\_nucleic\_acid\_metabolic\_process | BMP2 | 757 | 12 | 1.377334 | -0.825474 | 486 | 207.26 | 0.426461 |  |
| GO:0019219\_regulation\_of\_nucleobase\_\_nucleoside\_\_nucleotide\_and\_nucleic\_acid\_metabolic\_process | KLF12 | 757 | 12 | 1.377334 | -0.825474 | 486 | 207.26 | 0.426461 |  |
| GO:0019219\_regulation\_of\_nucleobase\_\_nucleoside\_\_nucleotide\_and\_nucleic\_acid\_metabolic\_process | JUN | 757 | 12 | 1.377334 | -0.825474 | 486 | 207.26 | 0.426461 |  |
| GO:0019219\_regulation\_of\_nucleobase\_\_nucleoside\_\_nucleotide\_and\_nucleic\_acid\_metabolic\_process | TBX5 | 757 | 12 | 1.377334 | -0.825474 | 486 | 207.26 | 0.426461 |  |
| GO:0019219\_regulation\_of\_nucleobase\_\_nucleoside\_\_nucleotide\_and\_nucleic\_acid\_metabolic\_process | FOXC1 | 757 | 12 | 1.377334 | -0.825474 | 486 | 207.26 | 0.426461 |  |
| GO:0019219\_regulation\_of\_nucleobase\_\_nucleoside\_\_nucleotide\_and\_nucleic\_acid\_metabolic\_process | RORA | 757 | 12 | 1.377334 | -0.825474 | 486 | 207.26 | 0.426461 |  |
| GO:0019219\_regulation\_of\_nucleobase\_\_nucleoside\_\_nucleotide\_and\_nucleic\_acid\_metabolic\_process | HBA-X | 757 | 12 | 1.377334 | -0.825474 | 486 | 207.26 | 0.426461 |  |
| GO:0019219\_regulation\_of\_nucleobase\_\_nucleoside\_\_nucleotide\_and\_nucleic\_acid\_metabolic\_process | FOXP4 | 757 | 12 | 1.377334 | -0.825474 | 486 | 207.26 | 0.426461 |  |
| GO:0019219\_regulation\_of\_nucleobase\_\_nucleoside\_\_nucleotide\_and\_nucleic\_acid\_metabolic\_process | C1D | 757 | 12 | 1.377334 | -0.825474 | 486 | 207.26 | 0.426461 |  |
| GO:0019219\_regulation\_of\_nucleobase\_\_nucleoside\_\_nucleotide\_and\_nucleic\_acid\_metabolic\_process | CTNNB1 | 757 | 12 | 1.377334 | -0.825474 | 486 | 207.26 | 0.426461 |  |
| GO:0019219\_regulation\_of\_nucleobase\_\_nucleoside\_\_nucleotide\_and\_nucleic\_acid\_metabolic\_process | CITED2 | 757 | 12 | 1.377334 | -0.825474 | 486 | 207.26 | 0.426461 |  |
| GO:0001502\_cartilage\_condensation | TGFB2 | 14 | 1 | 6.206199 | -0.824462 | 501 | 216.68 | 0.432495 |  |
| GO:0001829\_trophectodermal\_cell\_differentiation | CITED2 | 14 | 1 | 6.206199 | -0.824462 | 501 | 216.68 | 0.432495 |  |
| GO:0006695\_cholesterol\_biosynthetic\_process | CFTR | 14 | 1 | 6.206199 | -0.824462 | 501 | 216.68 | 0.432495 |  |
| GO:0006809\_nitric\_oxide\_biosynthetic\_process | RORA | 14 | 1 | 6.206199 | -0.824462 | 501 | 216.68 | 0.432495 |  |
| GO:0008064\_regulation\_of\_actin\_polymerization\_or\_depolymerization | PLEK | 14 | 1 | 6.206199 | -0.824462 | 501 | 216.68 | 0.432495 |  |
| GO:0010332\_response\_to\_gamma\_radiation | XRCC4 | 14 | 1 | 6.206199 | -0.824462 | 501 | 216.68 | 0.432495 |  |
| GO:0030032\_lamellipodium\_assembly | VAV3 | 14 | 1 | 6.206199 | -0.824462 | 501 | 216.68 | 0.432495 |  |
| GO:0030832\_regulation\_of\_actin\_filament\_length | PLEK | 14 | 1 | 6.206199 | -0.824462 | 501 | 216.68 | 0.432495 |  |
| GO:0031099\_regeneration | APOE | 14 | 1 | 6.206199 | -0.824462 | 501 | 216.68 | 0.432495 |  |
| GO:0034623\_cellular\_macromolecular\_complex\_disassembly | PLEK | 14 | 1 | 6.206199 | -0.824462 | 501 | 216.68 | 0.432495 |  |
| GO:0035036\_sperm-egg\_recognition | SPACA3 | 14 | 1 | 6.206199 | -0.824462 | 501 | 216.68 | 0.432495 |  |
| GO:0042310\_vasoconstriction | ACTA2 | 14 | 1 | 6.206199 | -0.824462 | 501 | 216.68 | 0.432495 |  |
| GO:0045453\_bone\_resorption | CTNNB1 | 14 | 1 | 6.206199 | -0.824462 | 501 | 216.68 | 0.432495 |  |
| GO:0046209\_nitric\_oxide\_metabolic\_process | RORA | 14 | 1 | 6.206199 | -0.824462 | 501 | 216.68 | 0.432495 |  |
| GO:0048048\_embryonic\_eye\_morphogenesis | PROX1 | 14 | 1 | 6.206199 | -0.824462 | 501 | 216.68 | 0.432495 |  |
| GO:0051960\_regulation\_of\_nervous\_system\_development | XRCC4 | 118 | 3 | 2.208986 | -0.813789 | 502 | 217.68 | 0.433625 |  |
| GO:0051960\_regulation\_of\_nervous\_system\_development | BMP2 | 118 | 3 | 2.208986 | -0.813789 | 502 | 217.68 | 0.433625 |  |
| GO:0051960\_regulation\_of\_nervous\_system\_development | DLL1 | 118 | 3 | 2.208986 | -0.813789 | 502 | 217.68 | 0.433625 |  |
| GO:0032270\_positive\_regulation\_of\_cellular\_protein\_metabolic\_process | PLEK | 61 | 2 | 2.848747 | -0.809148 | 503 | 219.16 | 0.435706 |  |
| GO:0032270\_positive\_regulation\_of\_cellular\_protein\_metabolic\_process | RB1CC1 | 61 | 2 | 2.848747 | -0.809148 | 503 | 219.16 | 0.435706 |  |
| GO:0014706\_striated\_muscle\_tissue\_development | RBP4 | 120 | 3 | 2.172170 | -0.798434 | 504 | 220.04 | 0.436587 |  |
| GO:0014706\_striated\_muscle\_tissue\_development | TBX5 | 120 | 3 | 2.172170 | -0.798434 | 504 | 220.04 | 0.436587 |  |
| GO:0014706\_striated\_muscle\_tissue\_development | PROX1 | 120 | 3 | 2.172170 | -0.798434 | 504 | 220.04 | 0.436587 |  |
| GO:0021537\_telencephalon\_development | LRP8 | 62 | 2 | 2.802800 | -0.797970 | 506 | 220.98 | 0.436719 |  |
| GO:0021537\_telencephalon\_development | ZEB2 | 62 | 2 | 2.802800 | -0.797970 | 506 | 220.98 | 0.436719 |  |
| GO:0030855\_epithelial\_cell\_differentiation | JUN | 62 | 2 | 2.802800 | -0.797970 | 506 | 220.98 | 0.436719 |  |
| GO:0030855\_epithelial\_cell\_differentiation | CTNNB1 | 62 | 2 | 2.802800 | -0.797970 | 506 | 220.98 | 0.436719 |  |
| GO:0010171\_body\_morphogenesis | SCHIP1 | 15 | 1 | 5.792453 | -0.796902 | 518 | 229.67 | 0.443378 |  |
| GO:0021795\_cerebral\_cortex\_cell\_migration | LRP8 | 15 | 1 | 5.792453 | -0.796902 | 518 | 229.67 | 0.443378 |  |
| GO:0031069\_hair\_follicle\_morphogenesis | TGFB2 | 15 | 1 | 5.792453 | -0.796902 | 518 | 229.67 | 0.443378 |  |
| GO:0031076\_embryonic\_camera-type\_eye\_development | PROX1 | 15 | 1 | 5.792453 | -0.796902 | 518 | 229.67 | 0.443378 |  |
| GO:0035116\_embryonic\_hindlimb\_morphogenesis | CTNNB1 | 15 | 1 | 5.792453 | -0.796902 | 518 | 229.67 | 0.443378 |  |
| GO:0048008\_platelet-derived\_growth\_factor\_receptor\_signaling\_pathway | SCHIP1 | 15 | 1 | 5.792453 | -0.796902 | 518 | 229.67 | 0.443378 |  |
| GO:0048010\_vascular\_endothelial\_growth\_factor\_receptor\_signaling\_pathway | FOXC1 | 15 | 1 | 5.792453 | -0.796902 | 518 | 229.67 | 0.443378 |  |
| GO:0048144\_fibroblast\_proliferation | XRCC4 | 15 | 1 | 5.792453 | -0.796902 | 518 | 229.67 | 0.443378 |  |
| GO:0048145\_regulation\_of\_fibroblast\_proliferation | XRCC4 | 15 | 1 | 5.792453 | -0.796902 | 518 | 229.67 | 0.443378 |  |
| GO:0048610\_reproductive\_cellular\_process | SEPT4 | 15 | 1 | 5.792453 | -0.796902 | 518 | 229.67 | 0.443378 |  |
| GO:0060322\_head\_development | SCHIP1 | 15 | 1 | 5.792453 | -0.796902 | 518 | 229.67 | 0.443378 |  |
| GO:0070227\_lymphocyte\_apoptosis | TGFB2 | 15 | 1 | 5.792453 | -0.796902 | 518 | 229.67 | 0.443378 |  |
| GO:0008104\_protein\_localization | RBP4 | 251 | 5 | 1.730813 | -0.795393 | 519 | 229.87 | 0.442909 |  |
| GO:0008104\_protein\_localization | PLEK | 251 | 5 | 1.730813 | -0.795393 | 519 | 229.87 | 0.442909 |  |
| GO:0008104\_protein\_localization | APOE | 251 | 5 | 1.730813 | -0.795393 | 519 | 229.87 | 0.442909 |  |
| GO:0008104\_protein\_localization | OPTN | 251 | 5 | 1.730813 | -0.795393 | 519 | 229.87 | 0.442909 |  |
| GO:0008104\_protein\_localization | CTNNB1 | 251 | 5 | 1.730813 | -0.795393 | 519 | 229.87 | 0.442909 |  |
| GO:0007369\_gastrulation | FOXC1 | 63 | 2 | 2.758311 | -0.787019 | 520 | 231.69 | 0.445558 |  |
| GO:0007369\_gastrulation | CTNNB1 | 63 | 2 | 2.758311 | -0.787019 | 520 | 231.69 | 0.445558 |  |
| GO:0065008\_regulation\_of\_biological\_quality | RHOJ | 693 | 11 | 1.379155 | -0.786355 | 521 | 231.77 | 0.444856 |  |
| GO:0065008\_regulation\_of\_biological\_quality | RBP4 | 693 | 11 | 1.379155 | -0.786355 | 521 | 231.77 | 0.444856 |  |
| GO:0065008\_regulation\_of\_biological\_quality | PTPRM | 693 | 11 | 1.379155 | -0.786355 | 521 | 231.77 | 0.444856 |  |
| GO:0065008\_regulation\_of\_biological\_quality | PLEK | 693 | 11 | 1.379155 | -0.786355 | 521 | 231.77 | 0.444856 |  |
| GO:0065008\_regulation\_of\_biological\_quality | APOE | 693 | 11 | 1.379155 | -0.786355 | 521 | 231.77 | 0.444856 |  |
| GO:0065008\_regulation\_of\_biological\_quality | ACTA2 | 693 | 11 | 1.379155 | -0.786355 | 521 | 231.77 | 0.444856 |  |
| GO:0065008\_regulation\_of\_biological\_quality | RB1CC1 | 693 | 11 | 1.379155 | -0.786355 | 521 | 231.77 | 0.444856 |  |
| GO:0065008\_regulation\_of\_biological\_quality | FOXC1 | 693 | 11 | 1.379155 | -0.786355 | 521 | 231.77 | 0.444856 |  |
| GO:0065008\_regulation\_of\_biological\_quality | HBA-X | 693 | 11 | 1.379155 | -0.786355 | 521 | 231.77 | 0.444856 |  |
| GO:0065008\_regulation\_of\_biological\_quality | TGFB2 | 693 | 11 | 1.379155 | -0.786355 | 521 | 231.77 | 0.444856 |  |
| GO:0065008\_regulation\_of\_biological\_quality | CTNNB1 | 693 | 11 | 1.379155 | -0.786355 | 521 | 231.77 | 0.444856 |  |
| GO:0016481\_negative\_regulation\_of\_transcription | EGR1 | 253 | 5 | 1.717130 | -0.785313 | 523 | 232.03 | 0.443652 |  |
| GO:0016481\_negative\_regulation\_of\_transcription | HBA-X | 253 | 5 | 1.717130 | -0.785313 | 523 | 232.03 | 0.443652 |  |
| GO:0016481\_negative\_regulation\_of\_transcription | FOXP4 | 253 | 5 | 1.717130 | -0.785313 | 523 | 232.03 | 0.443652 |  |
| GO:0016481\_negative\_regulation\_of\_transcription | C1D | 253 | 5 | 1.717130 | -0.785313 | 523 | 232.03 | 0.443652 |  |
| GO:0016481\_negative\_regulation\_of\_transcription | CTNNB1 | 253 | 5 | 1.717130 | -0.785313 | 523 | 232.03 | 0.443652 |  |
| GO:0030097\_hemopoiesis | EGR1 | 253 | 5 | 1.717130 | -0.785313 | 523 | 232.03 | 0.443652 |  |
| GO:0030097\_hemopoiesis | XRCC4 | 253 | 5 | 1.717130 | -0.785313 | 523 | 232.03 | 0.443652 |  |
| GO:0030097\_hemopoiesis | HBA-X | 253 | 5 | 1.717130 | -0.785313 | 523 | 232.03 | 0.443652 |  |
| GO:0030097\_hemopoiesis | TGFB2 | 253 | 5 | 1.717130 | -0.785313 | 523 | 232.03 | 0.443652 |  |
| GO:0030097\_hemopoiesis | CTNNB1 | 253 | 5 | 1.717130 | -0.785313 | 523 | 232.03 | 0.443652 |  |
| GO:0051171\_regulation\_of\_nitrogen\_compound\_metabolic\_process | EGR1 | 771 | 12 | 1.352324 | -0.783853 | 524 | 232.09 | 0.442920 |  |
| GO:0051171\_regulation\_of\_nitrogen\_compound\_metabolic\_process | BMP2 | 771 | 12 | 1.352324 | -0.783853 | 524 | 232.09 | 0.442920 |  |
| GO:0051171\_regulation\_of\_nitrogen\_compound\_metabolic\_process | KLF12 | 771 | 12 | 1.352324 | -0.783853 | 524 | 232.09 | 0.442920 |  |
| GO:0051171\_regulation\_of\_nitrogen\_compound\_metabolic\_process | JUN | 771 | 12 | 1.352324 | -0.783853 | 524 | 232.09 | 0.442920 |  |
| GO:0051171\_regulation\_of\_nitrogen\_compound\_metabolic\_process | TBX5 | 771 | 12 | 1.352324 | -0.783853 | 524 | 232.09 | 0.442920 |  |
| GO:0051171\_regulation\_of\_nitrogen\_compound\_metabolic\_process | FOXC1 | 771 | 12 | 1.352324 | -0.783853 | 524 | 232.09 | 0.442920 |  |
| GO:0051171\_regulation\_of\_nitrogen\_compound\_metabolic\_process | RORA | 771 | 12 | 1.352324 | -0.783853 | 524 | 232.09 | 0.442920 |  |
| GO:0051171\_regulation\_of\_nitrogen\_compound\_metabolic\_process | HBA-X | 771 | 12 | 1.352324 | -0.783853 | 524 | 232.09 | 0.442920 |  |
| GO:0051171\_regulation\_of\_nitrogen\_compound\_metabolic\_process | FOXP4 | 771 | 12 | 1.352324 | -0.783853 | 524 | 232.09 | 0.442920 |  |
| GO:0051171\_regulation\_of\_nitrogen\_compound\_metabolic\_process | C1D | 771 | 12 | 1.352324 | -0.783853 | 524 | 232.09 | 0.442920 |  |
| GO:0051171\_regulation\_of\_nitrogen\_compound\_metabolic\_process | CTNNB1 | 771 | 12 | 1.352324 | -0.783853 | 524 | 232.09 | 0.442920 |  |
| GO:0051171\_regulation\_of\_nitrogen\_compound\_metabolic\_process | CITED2 | 771 | 12 | 1.352324 | -0.783853 | 524 | 232.09 | 0.442920 |  |
| GO:0048699\_generation\_of\_neurons | XRCC4 | 396 | 7 | 1.535878 | -0.778492 | 525 | 233.47 | 0.444705 |  |
| GO:0048699\_generation\_of\_neurons | BMP2 | 396 | 7 | 1.535878 | -0.778492 | 525 | 233.47 | 0.444705 |  |
| GO:0048699\_generation\_of\_neurons | APOE | 396 | 7 | 1.535878 | -0.778492 | 525 | 233.47 | 0.444705 |  |
| GO:0048699\_generation\_of\_neurons | DLL1 | 396 | 7 | 1.535878 | -0.778492 | 525 | 233.47 | 0.444705 |  |
| GO:0048699\_generation\_of\_neurons | RORA | 396 | 7 | 1.535878 | -0.778492 | 525 | 233.47 | 0.444705 |  |
| GO:0048699\_generation\_of\_neurons | PRKG1 | 396 | 7 | 1.535878 | -0.778492 | 525 | 233.47 | 0.444705 |  |
| GO:0048699\_generation\_of\_neurons | TGFB2 | 396 | 7 | 1.535878 | -0.778492 | 525 | 233.47 | 0.444705 |  |
| GO:0001933\_negative\_regulation\_of\_protein\_amino\_acid\_phosphorylation | JUN | 16 | 1 | 5.430425 | -0.771273 | 532 | 242.4 | 0.455639 |  |
| GO:0009988\_cell-cell\_recognition | SPACA3 | 16 | 1 | 5.430425 | -0.771273 | 532 | 242.4 | 0.455639 |  |
| GO:0016126\_sterol\_biosynthetic\_process | CFTR | 16 | 1 | 5.430425 | -0.771273 | 532 | 242.4 | 0.455639 |  |
| GO:0021696\_cerebellar\_cortex\_morphogenesis | RORA | 16 | 1 | 5.430425 | -0.771273 | 532 | 242.4 | 0.455639 |  |
| GO:0043087\_regulation\_of\_GTPase\_activity | VAV3 | 16 | 1 | 5.430425 | -0.771273 | 532 | 242.4 | 0.455639 |  |
| GO:0046364\_monosaccharide\_biosynthetic\_process | RBP4 | 16 | 1 | 5.430425 | -0.771273 | 532 | 242.4 | 0.455639 |  |
| GO:0060713\_labyrinthine\_layer\_morphogenesis | RSPO3 | 16 | 1 | 5.430425 | -0.771273 | 532 | 242.4 | 0.455639 |  |
| GO:0030098\_lymphocyte\_differentiation | EGR1 | 124 | 3 | 2.102100 | -0.768792 | 533 | 242.64 | 0.455235 |  |
| GO:0030098\_lymphocyte\_differentiation | XRCC4 | 124 | 3 | 2.102100 | -0.768792 | 533 | 242.64 | 0.455235 |  |
| GO:0030098\_lymphocyte\_differentiation | CTNNB1 | 124 | 3 | 2.102100 | -0.768792 | 533 | 242.64 | 0.455235 |  |
| GO:0007179\_transforming\_growth\_factor\_beta\_receptor\_signaling\_pathway | BMP2 | 66 | 2 | 2.632933 | -0.755455 | 536 | 245.18 | 0.457425 |  |
| GO:0007179\_transforming\_growth\_factor\_beta\_receptor\_signaling\_pathway | CITED2 | 66 | 2 | 2.632933 | -0.755455 | 536 | 245.18 | 0.457425 |  |
| GO:0045860\_positive\_regulation\_of\_protein\_kinase\_activity | LRP8 | 66 | 2 | 2.632933 | -0.755455 | 536 | 245.18 | 0.457425 |  |
| GO:0045860\_positive\_regulation\_of\_protein\_kinase\_activity | ZEB2 | 66 | 2 | 2.632933 | -0.755455 | 536 | 245.18 | 0.457425 |  |
| GO:0051130\_positive\_regulation\_of\_cellular\_component\_organization | PLEK | 66 | 2 | 2.632933 | -0.755455 | 536 | 245.18 | 0.457425 |  |
| GO:0051130\_positive\_regulation\_of\_cellular\_component\_organization | PROX1 | 66 | 2 | 2.632933 | -0.755455 | 536 | 245.18 | 0.457425 |  |
| GO:0010605\_negative\_regulation\_of\_macromolecule\_metabolic\_process | EGR1 | 331 | 6 | 1.574987 | -0.749576 | 537 | 245.75 | 0.457635 |  |
| GO:0010605\_negative\_regulation\_of\_macromolecule\_metabolic\_process | JUN | 331 | 6 | 1.574987 | -0.749576 | 537 | 245.75 | 0.457635 |  |
| GO:0010605\_negative\_regulation\_of\_macromolecule\_metabolic\_process | HBA-X | 331 | 6 | 1.574987 | -0.749576 | 537 | 245.75 | 0.457635 |  |
| GO:0010605\_negative\_regulation\_of\_macromolecule\_metabolic\_process | FOXP4 | 331 | 6 | 1.574987 | -0.749576 | 537 | 245.75 | 0.457635 |  |
| GO:0010605\_negative\_regulation\_of\_macromolecule\_metabolic\_process | C1D | 331 | 6 | 1.574987 | -0.749576 | 537 | 245.75 | 0.457635 |  |
| GO:0010605\_negative\_regulation\_of\_macromolecule\_metabolic\_process | CTNNB1 | 331 | 6 | 1.574987 | -0.749576 | 537 | 245.75 | 0.457635 |  |
| GO:0009408\_response\_to\_heat | TCFEC | 17 | 1 | 5.110988 | -0.747338 | 545 | 253.92 | 0.465908 |  |
| GO:0022029\_telencephalon\_cell\_migration | LRP8 | 17 | 1 | 5.110988 | -0.747338 | 545 | 253.92 | 0.465908 |  |
| GO:0032535\_regulation\_of\_cellular\_component\_size | PLEK | 17 | 1 | 5.110988 | -0.747338 | 545 | 253.92 | 0.465908 |  |
| GO:0042398\_cellular\_amino\_acid\_derivative\_biosynthetic\_process | TGFB2 | 17 | 1 | 5.110988 | -0.747338 | 545 | 253.92 | 0.465908 |  |
| GO:0042417\_dopamine\_metabolic\_process | TGFB2 | 17 | 1 | 5.110988 | -0.747338 | 545 | 253.92 | 0.465908 |  |
| GO:0045670\_regulation\_of\_osteoclast\_differentiation | CTNNB1 | 17 | 1 | 5.110988 | -0.747338 | 545 | 253.92 | 0.465908 |  |
| GO:0050708\_regulation\_of\_protein\_secretion | RBP4 | 17 | 1 | 5.110988 | -0.747338 | 545 | 253.92 | 0.465908 |  |
| GO:0055001\_muscle\_cell\_development | PROX1 | 17 | 1 | 5.110988 | -0.747338 | 545 | 253.92 | 0.465908 |  |
| GO:0031324\_negative\_regulation\_of\_cellular\_metabolic\_process | EGR1 | 332 | 6 | 1.570243 | -0.745371 | 546 | 254.01 | 0.465220 |  |
| GO:0031324\_negative\_regulation\_of\_cellular\_metabolic\_process | JUN | 332 | 6 | 1.570243 | -0.745371 | 546 | 254.01 | 0.465220 |  |
| GO:0031324\_negative\_regulation\_of\_cellular\_metabolic\_process | HBA-X | 332 | 6 | 1.570243 | -0.745371 | 546 | 254.01 | 0.465220 |  |
| GO:0031324\_negative\_regulation\_of\_cellular\_metabolic\_process | FOXP4 | 332 | 6 | 1.570243 | -0.745371 | 546 | 254.01 | 0.465220 |  |
| GO:0031324\_negative\_regulation\_of\_cellular\_metabolic\_process | C1D | 332 | 6 | 1.570243 | -0.745371 | 546 | 254.01 | 0.465220 |  |
| GO:0031324\_negative\_regulation\_of\_cellular\_metabolic\_process | CTNNB1 | 332 | 6 | 1.570243 | -0.745371 | 546 | 254.01 | 0.465220 |  |
| GO:0051247\_positive\_regulation\_of\_protein\_metabolic\_process | PLEK | 67 | 2 | 2.593636 | -0.745342 | 547 | 254.82 | 0.465850 |  |
| GO:0051247\_positive\_regulation\_of\_protein\_metabolic\_process | RB1CC1 | 67 | 2 | 2.593636 | -0.745342 | 547 | 254.82 | 0.465850 |  |
| GO:0001775\_cell\_activation | EGR1 | 262 | 5 | 1.658145 | -0.741575 | 549 | 255.25 | 0.464936 |  |
| GO:0001775\_cell\_activation | XRCC4 | 262 | 5 | 1.658145 | -0.741575 | 549 | 255.25 | 0.464936 |  |
| GO:0001775\_cell\_activation | PLEK | 262 | 5 | 1.658145 | -0.741575 | 549 | 255.25 | 0.464936 |  |
| GO:0001775\_cell\_activation | RORA | 262 | 5 | 1.658145 | -0.741575 | 549 | 255.25 | 0.464936 |  |
| GO:0001775\_cell\_activation | CTNNB1 | 262 | 5 | 1.658145 | -0.741575 | 549 | 255.25 | 0.464936 |  |
| GO:0010629\_negative\_regulation\_of\_gene\_expression | EGR1 | 262 | 5 | 1.658145 | -0.741575 | 549 | 255.25 | 0.464936 |  |
| GO:0010629\_negative\_regulation\_of\_gene\_expression | HBA-X | 262 | 5 | 1.658145 | -0.741575 | 549 | 255.25 | 0.464936 |  |
| GO:0010629\_negative\_regulation\_of\_gene\_expression | FOXP4 | 262 | 5 | 1.658145 | -0.741575 | 549 | 255.25 | 0.464936 |  |
| GO:0010629\_negative\_regulation\_of\_gene\_expression | C1D | 262 | 5 | 1.658145 | -0.741575 | 549 | 255.25 | 0.464936 |  |
| GO:0010629\_negative\_regulation\_of\_gene\_expression | CTNNB1 | 262 | 5 | 1.658145 | -0.741575 | 549 | 255.25 | 0.464936 |  |
| GO:0045597\_positive\_regulation\_of\_cell\_differentiation | XRCC4 | 128 | 3 | 2.036409 | -0.740498 | 550 | 255.66 | 0.464836 |  |
| GO:0045597\_positive\_regulation\_of\_cell\_differentiation | BMP2 | 128 | 3 | 2.036409 | -0.740498 | 550 | 255.66 | 0.464836 |  |
| GO:0045597\_positive\_regulation\_of\_cell\_differentiation | CTNNB1 | 128 | 3 | 2.036409 | -0.740498 | 550 | 255.66 | 0.464836 |  |
| GO:0042692\_muscle\_cell\_differentiation | TBX5 | 68 | 2 | 2.555494 | -0.735422 | 551 | 256.44 | 0.465408 |  |
| GO:0042692\_muscle\_cell\_differentiation | PROX1 | 68 | 2 | 2.555494 | -0.735422 | 551 | 256.44 | 0.465408 |  |
| GO:0001825\_blastocyst\_formation | CITED2 | 18 | 1 | 4.827044 | -0.724905 | 561 | 268.15 | 0.477986 |  |
| GO:0008589\_regulation\_of\_smoothened\_signaling\_pathway | PRRX1 | 18 | 1 | 4.827044 | -0.724905 | 561 | 268.15 | 0.477986 |  |
| GO:0021885\_forebrain\_cell\_migration | LRP8 | 18 | 1 | 4.827044 | -0.724905 | 561 | 268.15 | 0.477986 |  |
| GO:0030282\_bone\_mineralization | BMP2 | 18 | 1 | 4.827044 | -0.724905 | 561 | 268.15 | 0.477986 |  |
| GO:0032984\_macromolecular\_complex\_disassembly | PLEK | 18 | 1 | 4.827044 | -0.724905 | 561 | 268.15 | 0.477986 |  |
| GO:0043029\_T\_cell\_homeostasis | TGFB2 | 18 | 1 | 4.827044 | -0.724905 | 561 | 268.15 | 0.477986 |  |
| GO:0045638\_negative\_regulation\_of\_myeloid\_cell\_differentiation | CTNNB1 | 18 | 1 | 4.827044 | -0.724905 | 561 | 268.15 | 0.477986 |  |
| GO:0048730\_epidermis\_morphogenesis | TGFB2 | 18 | 1 | 4.827044 | -0.724905 | 561 | 268.15 | 0.477986 |  |
| GO:0051222\_positive\_regulation\_of\_protein\_transport | RBP4 | 18 | 1 | 4.827044 | -0.724905 | 561 | 268.15 | 0.477986 |  |
| GO:0060571\_morphogenesis\_of\_an\_epithelial\_fold | WNT5A | 18 | 1 | 4.827044 | -0.724905 | 561 | 268.15 | 0.477986 |  |
| GO:0000904\_cell\_morphogenesis\_involved\_in\_differentiation | APOE | 199 | 4 | 1.746468 | -0.710986 | 562 | 270.54 | 0.481388 |  |
| GO:0000904\_cell\_morphogenesis\_involved\_in\_differentiation | PROX1 | 199 | 4 | 1.746468 | -0.710986 | 562 | 270.54 | 0.481388 |  |
| GO:0000904\_cell\_morphogenesis\_involved\_in\_differentiation | CTNNB1 | 199 | 4 | 1.746468 | -0.710986 | 562 | 270.54 | 0.481388 |  |
| GO:0000904\_cell\_morphogenesis\_involved\_in\_differentiation | TGFB2 | 199 | 4 | 1.746468 | -0.710986 | 562 | 270.54 | 0.481388 |  |
| GO:0009100\_glycoprotein\_metabolic\_process | A930038C07RIK | 71 | 2 | 2.447515 | -0.706768 | 563 | 271.9 | 0.482948 |  |
| GO:0009100\_glycoprotein\_metabolic\_process | DCN | 71 | 2 | 2.447515 | -0.706768 | 563 | 271.9 | 0.482948 |  |
| GO:0045934\_negative\_regulation\_of\_nucleobase\_\_nucleoside\_\_nucleotide\_and\_nucleic\_acid\_metabolic\_process | EGR1 | 270 | 5 | 1.609015 | -0.704817 | 564 | 272.1 | 0.482447 |  |
| GO:0045934\_negative\_regulation\_of\_nucleobase\_\_nucleoside\_\_nucleotide\_and\_nucleic\_acid\_metabolic\_process | HBA-X | 270 | 5 | 1.609015 | -0.704817 | 564 | 272.1 | 0.482447 |  |
| GO:0045934\_negative\_regulation\_of\_nucleobase\_\_nucleoside\_\_nucleotide\_and\_nucleic\_acid\_metabolic\_process | FOXP4 | 270 | 5 | 1.609015 | -0.704817 | 564 | 272.1 | 0.482447 |  |
| GO:0045934\_negative\_regulation\_of\_nucleobase\_\_nucleoside\_\_nucleotide\_and\_nucleic\_acid\_metabolic\_process | C1D | 270 | 5 | 1.609015 | -0.704817 | 564 | 272.1 | 0.482447 |  |
| GO:0045934\_negative\_regulation\_of\_nucleobase\_\_nucleoside\_\_nucleotide\_and\_nucleic\_acid\_metabolic\_process | CTNNB1 | 270 | 5 | 1.609015 | -0.704817 | 564 | 272.1 | 0.482447 |  |
| GO:0006776\_vitamin\_A\_metabolic\_process | RBP4 | 19 | 1 | 4.572989 | -0.703810 | 575 | 278.5 | 0.484348 |  |
| GO:0009798\_axis\_specification | CTNNB1 | 19 | 1 | 4.572989 | -0.703810 | 575 | 278.5 | 0.484348 |  |
| GO:0019218\_regulation\_of\_steroid\_metabolic\_process | APOE | 19 | 1 | 4.572989 | -0.703810 | 575 | 278.5 | 0.484348 |  |
| GO:0021587\_cerebellum\_morphogenesis | RORA | 19 | 1 | 4.572989 | -0.703810 | 575 | 278.5 | 0.484348 |  |
| GO:0030199\_collagen\_fibril\_organization | FOXC1 | 19 | 1 | 4.572989 | -0.703810 | 575 | 278.5 | 0.484348 |  |
| GO:0032526\_response\_to\_retinoic\_acid | GDAP1 | 19 | 1 | 4.572989 | -0.703810 | 575 | 278.5 | 0.484348 |  |
| GO:0033189\_response\_to\_vitamin\_A | GDAP1 | 19 | 1 | 4.572989 | -0.703810 | 575 | 278.5 | 0.484348 |  |
| GO:0042491\_auditory\_receptor\_cell\_differentiation | DLL1 | 19 | 1 | 4.572989 | -0.703810 | 575 | 278.5 | 0.484348 |  |
| GO:0046165\_alcohol\_biosynthetic\_process | RBP4 | 19 | 1 | 4.572989 | -0.703810 | 575 | 278.5 | 0.484348 |  |
| GO:0048701\_embryonic\_cranial\_skeleton\_morphogenesis | PRRX1 | 19 | 1 | 4.572989 | -0.703810 | 575 | 278.5 | 0.484348 |  |
| GO:0060444\_branching\_involved\_in\_mammary\_gland\_duct\_morphogenesis | WNT5A | 19 | 1 | 4.572989 | -0.703810 | 575 | 278.5 | 0.484348 |  |
| GO:0051172\_negative\_regulation\_of\_nitrogen\_compound\_metabolic\_process | EGR1 | 271 | 5 | 1.603077 | -0.700356 | 576 | 279.0 | 0.484375 |  |
| GO:0051172\_negative\_regulation\_of\_nitrogen\_compound\_metabolic\_process | HBA-X | 271 | 5 | 1.603077 | -0.700356 | 576 | 279.0 | 0.484375 |  |
| GO:0051172\_negative\_regulation\_of\_nitrogen\_compound\_metabolic\_process | FOXP4 | 271 | 5 | 1.603077 | -0.700356 | 576 | 279.0 | 0.484375 |  |
| GO:0051172\_negative\_regulation\_of\_nitrogen\_compound\_metabolic\_process | C1D | 271 | 5 | 1.603077 | -0.700356 | 576 | 279.0 | 0.484375 |  |
| GO:0051172\_negative\_regulation\_of\_nitrogen\_compound\_metabolic\_process | CTNNB1 | 271 | 5 | 1.603077 | -0.700356 | 576 | 279.0 | 0.484375 |  |
| GO:0048839\_inner\_ear\_development | PRRX1 | 72 | 2 | 2.413522 | -0.697567 | 578 | 280.76 | 0.485744 |  |
| GO:0048839\_inner\_ear\_development | DLL1 | 72 | 2 | 2.413522 | -0.697567 | 578 | 280.76 | 0.485744 |  |
| GO:0050673\_epithelial\_cell\_proliferation | WNT5A | 72 | 2 | 2.413522 | -0.697567 | 578 | 280.76 | 0.485744 |  |
| GO:0050673\_epithelial\_cell\_proliferation | CTNNB1 | 72 | 2 | 2.413522 | -0.697567 | 578 | 280.76 | 0.485744 |  |
| GO:0034961\_cellular\_biopolymer\_biosynthetic\_process | EGR1 | 804 | 12 | 1.296818 | -0.692578 | 579 | 280.85 | 0.485060 |  |
| GO:0034961\_cellular\_biopolymer\_biosynthetic\_process | BMP2 | 804 | 12 | 1.296818 | -0.692578 | 579 | 280.85 | 0.485060 |  |
| GO:0034961\_cellular\_biopolymer\_biosynthetic\_process | KLF12 | 804 | 12 | 1.296818 | -0.692578 | 579 | 280.85 | 0.485060 |  |
| GO:0034961\_cellular\_biopolymer\_biosynthetic\_process | JUN | 804 | 12 | 1.296818 | -0.692578 | 579 | 280.85 | 0.485060 |  |
| GO:0034961\_cellular\_biopolymer\_biosynthetic\_process | TBX5 | 804 | 12 | 1.296818 | -0.692578 | 579 | 280.85 | 0.485060 |  |
| GO:0034961\_cellular\_biopolymer\_biosynthetic\_process | FOXC1 | 804 | 12 | 1.296818 | -0.692578 | 579 | 280.85 | 0.485060 |  |
| GO:0034961\_cellular\_biopolymer\_biosynthetic\_process | RORA | 804 | 12 | 1.296818 | -0.692578 | 579 | 280.85 | 0.485060 |  |
| GO:0034961\_cellular\_biopolymer\_biosynthetic\_process | HBA-X | 804 | 12 | 1.296818 | -0.692578 | 579 | 280.85 | 0.485060 |  |
| GO:0034961\_cellular\_biopolymer\_biosynthetic\_process | FOXP4 | 804 | 12 | 1.296818 | -0.692578 | 579 | 280.85 | 0.485060 |  |
| GO:0034961\_cellular\_biopolymer\_biosynthetic\_process | C1D | 804 | 12 | 1.296818 | -0.692578 | 579 | 280.85 | 0.485060 |  |
| GO:0034961\_cellular\_biopolymer\_biosynthetic\_process | CITED2 | 804 | 12 | 1.296818 | -0.692578 | 579 | 280.85 | 0.485060 |  |
| GO:0034961\_cellular\_biopolymer\_biosynthetic\_process | CTNNB1 | 804 | 12 | 1.296818 | -0.692578 | 579 | 280.85 | 0.485060 |  |
| GO:0006163\_purine\_nucleotide\_metabolic\_process | ADSSL1 | 73 | 2 | 2.380460 | -0.688533 | 581 | 282.17 | 0.485663 |  |
| GO:0006163\_purine\_nucleotide\_metabolic\_process | RORA | 73 | 2 | 2.380460 | -0.688533 | 581 | 282.17 | 0.485663 |  |
| GO:0048706\_embryonic\_skeletal\_system\_development | RBP4 | 73 | 2 | 2.380460 | -0.688533 | 581 | 282.17 | 0.485663 |  |
| GO:0048706\_embryonic\_skeletal\_system\_development | PRRX1 | 73 | 2 | 2.380460 | -0.688533 | 581 | 282.17 | 0.485663 |  |
| GO:0010558\_negative\_regulation\_of\_macromolecule\_biosynthetic\_process | EGR1 | 274 | 5 | 1.585525 | -0.687146 | 583 | 282.53 | 0.484614 |  |
| GO:0010558\_negative\_regulation\_of\_macromolecule\_biosynthetic\_process | HBA-X | 274 | 5 | 1.585525 | -0.687146 | 583 | 282.53 | 0.484614 |  |
| GO:0010558\_negative\_regulation\_of\_macromolecule\_biosynthetic\_process | FOXP4 | 274 | 5 | 1.585525 | -0.687146 | 583 | 282.53 | 0.484614 |  |
| GO:0010558\_negative\_regulation\_of\_macromolecule\_biosynthetic\_process | C1D | 274 | 5 | 1.585525 | -0.687146 | 583 | 282.53 | 0.484614 |  |
| GO:0010558\_negative\_regulation\_of\_macromolecule\_biosynthetic\_process | CTNNB1 | 274 | 5 | 1.585525 | -0.687146 | 583 | 282.53 | 0.484614 |  |
| GO:0033036\_macromolecule\_localization | RBP4 | 274 | 5 | 1.585525 | -0.687146 | 583 | 282.53 | 0.484614 |  |
| GO:0033036\_macromolecule\_localization | PLEK | 274 | 5 | 1.585525 | -0.687146 | 583 | 282.53 | 0.484614 |  |
| GO:0033036\_macromolecule\_localization | APOE | 274 | 5 | 1.585525 | -0.687146 | 583 | 282.53 | 0.484614 |  |
| GO:0033036\_macromolecule\_localization | OPTN | 274 | 5 | 1.585525 | -0.687146 | 583 | 282.53 | 0.484614 |  |
| GO:0033036\_macromolecule\_localization | CTNNB1 | 274 | 5 | 1.585525 | -0.687146 | 583 | 282.53 | 0.484614 |  |
| GO:0043284\_biopolymer\_biosynthetic\_process | EGR1 | 807 | 12 | 1.291997 | -0.684734 | 584 | 282.79 | 0.484229 |  |
| GO:0043284\_biopolymer\_biosynthetic\_process | BMP2 | 807 | 12 | 1.291997 | -0.684734 | 584 | 282.79 | 0.484229 |  |
| GO:0043284\_biopolymer\_biosynthetic\_process | KLF12 | 807 | 12 | 1.291997 | -0.684734 | 584 | 282.79 | 0.484229 |  |
| GO:0043284\_biopolymer\_biosynthetic\_process | JUN | 807 | 12 | 1.291997 | -0.684734 | 584 | 282.79 | 0.484229 |  |
| GO:0043284\_biopolymer\_biosynthetic\_process | TBX5 | 807 | 12 | 1.291997 | -0.684734 | 584 | 282.79 | 0.484229 |  |
| GO:0043284\_biopolymer\_biosynthetic\_process | FOXC1 | 807 | 12 | 1.291997 | -0.684734 | 584 | 282.79 | 0.484229 |  |
| GO:0043284\_biopolymer\_biosynthetic\_process | RORA | 807 | 12 | 1.291997 | -0.684734 | 584 | 282.79 | 0.484229 |  |
| GO:0043284\_biopolymer\_biosynthetic\_process | HBA-X | 807 | 12 | 1.291997 | -0.684734 | 584 | 282.79 | 0.484229 |  |
| GO:0043284\_biopolymer\_biosynthetic\_process | FOXP4 | 807 | 12 | 1.291997 | -0.684734 | 584 | 282.79 | 0.484229 |  |
| GO:0043284\_biopolymer\_biosynthetic\_process | C1D | 807 | 12 | 1.291997 | -0.684734 | 584 | 282.79 | 0.484229 |  |
| GO:0043284\_biopolymer\_biosynthetic\_process | CITED2 | 807 | 12 | 1.291997 | -0.684734 | 584 | 282.79 | 0.484229 |  |
| GO:0043284\_biopolymer\_biosynthetic\_process | CTNNB1 | 807 | 12 | 1.291997 | -0.684734 | 584 | 282.79 | 0.484229 |  |
| GO:0006584\_catecholamine\_metabolic\_process | TGFB2 | 20 | 1 | 4.344340 | -0.683915 | 594 | 288.71 | 0.486044 |  |
| GO:0008360\_regulation\_of\_cell\_shape | RHOJ | 20 | 1 | 4.344340 | -0.683915 | 594 | 288.71 | 0.486044 |  |
| GO:0009712\_catechol\_metabolic\_process | TGFB2 | 20 | 1 | 4.344340 | -0.683915 | 594 | 288.71 | 0.486044 |  |
| GO:0010927\_cellular\_component\_assembly\_involved\_in\_morphogenesis | PROX1 | 20 | 1 | 4.344340 | -0.683915 | 594 | 288.71 | 0.486044 |  |
| GO:0018958\_phenol\_metabolic\_process | TGFB2 | 20 | 1 | 4.344340 | -0.683915 | 594 | 288.71 | 0.486044 |  |
| GO:0021695\_cerebellar\_cortex\_development | RORA | 20 | 1 | 4.344340 | -0.683915 | 594 | 288.71 | 0.486044 |  |
| GO:0031214\_biomineral\_formation | BMP2 | 20 | 1 | 4.344340 | -0.683915 | 594 | 288.71 | 0.486044 |  |
| GO:0034311\_diol\_metabolic\_process | TGFB2 | 20 | 1 | 4.344340 | -0.683915 | 594 | 288.71 | 0.486044 |  |
| GO:0042326\_negative\_regulation\_of\_phosphorylation | JUN | 20 | 1 | 4.344340 | -0.683915 | 594 | 288.71 | 0.486044 |  |
| GO:0048806\_genitalia\_development | RBP4 | 20 | 1 | 4.344340 | -0.683915 | 594 | 288.71 | 0.486044 |  |
| GO:0009892\_negative\_regulation\_of\_metabolic\_process | EGR1 | 348 | 6 | 1.498048 | -0.681155 | 595 | 288.98 | 0.485681 |  |
| GO:0009892\_negative\_regulation\_of\_metabolic\_process | JUN | 348 | 6 | 1.498048 | -0.681155 | 595 | 288.98 | 0.485681 |  |
| GO:0009892\_negative\_regulation\_of\_metabolic\_process | HBA-X | 348 | 6 | 1.498048 | -0.681155 | 595 | 288.98 | 0.485681 |  |
| GO:0009892\_negative\_regulation\_of\_metabolic\_process | FOXP4 | 348 | 6 | 1.498048 | -0.681155 | 595 | 288.98 | 0.485681 |  |
| GO:0009892\_negative\_regulation\_of\_metabolic\_process | C1D | 348 | 6 | 1.498048 | -0.681155 | 595 | 288.98 | 0.485681 |  |
| GO:0009892\_negative\_regulation\_of\_metabolic\_process | CTNNB1 | 348 | 6 | 1.498048 | -0.681155 | 595 | 288.98 | 0.485681 |  |
| GO:0009416\_response\_to\_light\_stimulus | RBP4 | 74 | 2 | 2.348292 | -0.679662 | 596 | 289.63 | 0.485956 |  |
| GO:0009416\_response\_to\_light\_stimulus | RGS9BP | 74 | 2 | 2.348292 | -0.679662 | 596 | 289.63 | 0.485956 |  |
| GO:0048534\_hemopoietic\_or\_lymphoid\_organ\_development | EGR1 | 277 | 5 | 1.568354 | -0.674189 | 597 | 290.09 | 0.485913 |  |
| GO:0048534\_hemopoietic\_or\_lymphoid\_organ\_development | XRCC4 | 277 | 5 | 1.568354 | -0.674189 | 597 | 290.09 | 0.485913 |  |
| GO:0048534\_hemopoietic\_or\_lymphoid\_organ\_development | HBA-X | 277 | 5 | 1.568354 | -0.674189 | 597 | 290.09 | 0.485913 |  |
| GO:0048534\_hemopoietic\_or\_lymphoid\_organ\_development | TGFB2 | 277 | 5 | 1.568354 | -0.674189 | 597 | 290.09 | 0.485913 |  |
| GO:0048534\_hemopoietic\_or\_lymphoid\_organ\_development | CTNNB1 | 277 | 5 | 1.568354 | -0.674189 | 597 | 290.09 | 0.485913 |  |
| GO:0019538\_protein\_metabolic\_process | WNT5A | 655 | 10 | 1.326516 | -0.670967 | 598 | 290.38 | 0.485585 |  |
| GO:0019538\_protein\_metabolic\_process | A930038C07RIK | 655 | 10 | 1.326516 | -0.670967 | 598 | 290.38 | 0.485585 |  |
| GO:0019538\_protein\_metabolic\_process | PLEK | 655 | 10 | 1.326516 | -0.670967 | 598 | 290.38 | 0.485585 |  |
| GO:0019538\_protein\_metabolic\_process | BTG2 | 655 | 10 | 1.326516 | -0.670967 | 598 | 290.38 | 0.485585 |  |
| GO:0019538\_protein\_metabolic\_process | RB1CC1 | 655 | 10 | 1.326516 | -0.670967 | 598 | 290.38 | 0.485585 |  |
| GO:0019538\_protein\_metabolic\_process | JUN | 655 | 10 | 1.326516 | -0.670967 | 598 | 290.38 | 0.485585 |  |
| GO:0019538\_protein\_metabolic\_process | ZEB2 | 655 | 10 | 1.326516 | -0.670967 | 598 | 290.38 | 0.485585 |  |
| GO:0019538\_protein\_metabolic\_process | DCN | 655 | 10 | 1.326516 | -0.670967 | 598 | 290.38 | 0.485585 |  |
| GO:0019538\_protein\_metabolic\_process | PRKG1 | 655 | 10 | 1.326516 | -0.670967 | 598 | 290.38 | 0.485585 |  |
| GO:0019538\_protein\_metabolic\_process | TGFB2 | 655 | 10 | 1.326516 | -0.670967 | 598 | 290.38 | 0.485585 |  |
| GO:0007281\_germ\_cell\_development | RBP4 | 75 | 2 | 2.316981 | -0.670948 | 600 | 290.97 | 0.484950 |  |
| GO:0007281\_germ\_cell\_development | SEPT4 | 75 | 2 | 2.316981 | -0.670948 | 600 | 290.97 | 0.484950 |  |
| GO:0048589\_developmental\_growth | WNT5A | 75 | 2 | 2.316981 | -0.670948 | 600 | 290.97 | 0.484950 |  |
| GO:0048589\_developmental\_growth | PROX1 | 75 | 2 | 2.316981 | -0.670948 | 600 | 290.97 | 0.484950 |  |
| GO:0001702\_gastrulation\_with\_mouth\_forming\_second | CTNNB1 | 21 | 1 | 4.137466 | -0.665102 | 609 | 299.68 | 0.492085 |  |
| GO:0002053\_positive\_regulation\_of\_mesenchymal\_cell\_proliferation | PRRX1 | 21 | 1 | 4.137466 | -0.665102 | 609 | 299.68 | 0.492085 |  |
| GO:0006944\_membrane\_fusion | VAV3 | 21 | 1 | 4.137466 | -0.665102 | 609 | 299.68 | 0.492085 |  |
| GO:0008154\_actin\_polymerization\_or\_depolymerization | PLEK | 21 | 1 | 4.137466 | -0.665102 | 609 | 299.68 | 0.492085 |  |
| GO:0010563\_negative\_regulation\_of\_phosphorus\_metabolic\_process | JUN | 21 | 1 | 4.137466 | -0.665102 | 609 | 299.68 | 0.492085 |  |
| GO:0016202\_regulation\_of\_striated\_muscle\_tissue\_development | PROX1 | 21 | 1 | 4.137466 | -0.665102 | 609 | 299.68 | 0.492085 |  |
| GO:0045936\_negative\_regulation\_of\_phosphate\_metabolic\_process | JUN | 21 | 1 | 4.137466 | -0.665102 | 609 | 299.68 | 0.492085 |  |
| GO:0048538\_thymus\_development | CTNNB1 | 21 | 1 | 4.137466 | -0.665102 | 609 | 299.68 | 0.492085 |  |
| GO:0048634\_regulation\_of\_muscle\_development | PROX1 | 21 | 1 | 4.137466 | -0.665102 | 609 | 299.68 | 0.492085 |  |
| GO:0051179\_localization | NRP2 | 1058 | 15 | 1.231854 | -0.659274 | 610 | 301.1 | 0.493607 |  |
| GO:0051179\_localization | RBP4 | 1058 | 15 | 1.231854 | -0.659274 | 610 | 301.1 | 0.493607 |  |
| GO:0051179\_localization | SCHIP1 | 1058 | 15 | 1.231854 | -0.659274 | 610 | 301.1 | 0.493607 |  |
| GO:0051179\_localization | VAV3 | 1058 | 15 | 1.231854 | -0.659274 | 610 | 301.1 | 0.493607 |  |
| GO:0051179\_localization | PLEK | 1058 | 15 | 1.231854 | -0.659274 | 610 | 301.1 | 0.493607 |  |
| GO:0051179\_localization | CFTR | 1058 | 15 | 1.231854 | -0.659274 | 610 | 301.1 | 0.493607 |  |
| GO:0051179\_localization | ZEB2 | 1058 | 15 | 1.231854 | -0.659274 | 610 | 301.1 | 0.493607 |  |
| GO:0051179\_localization | OPTN | 1058 | 15 | 1.231854 | -0.659274 | 610 | 301.1 | 0.493607 |  |
| GO:0051179\_localization | PRKG1 | 1058 | 15 | 1.231854 | -0.659274 | 610 | 301.1 | 0.493607 |  |
| GO:0051179\_localization | CTNNB1 | 1058 | 15 | 1.231854 | -0.659274 | 610 | 301.1 | 0.493607 |  |
| GO:0051179\_localization | TGFB2 | 1058 | 15 | 1.231854 | -0.659274 | 610 | 301.1 | 0.493607 |  |
| GO:0051179\_localization | APOE | 1058 | 15 | 1.231854 | -0.659274 | 610 | 301.1 | 0.493607 |  |
| GO:0051179\_localization | ATP6V1E1 | 1058 | 15 | 1.231854 | -0.659274 | 610 | 301.1 | 0.493607 |  |
| GO:0051179\_localization | LRP8 | 1058 | 15 | 1.231854 | -0.659274 | 610 | 301.1 | 0.493607 |  |
| GO:0051179\_localization | FOXC1 | 1058 | 15 | 1.231854 | -0.659274 | 610 | 301.1 | 0.493607 |  |
| GO:0001890\_placenta\_development | RSPO3 | 77 | 2 | 2.256800 | -0.653978 | 611 | 301.91 | 0.494124 |  |
| GO:0001890\_placenta\_development | CITED2 | 77 | 2 | 2.256800 | -0.653978 | 611 | 301.91 | 0.494124 |  |
| GO:0031327\_negative\_regulation\_of\_cellular\_biosynthetic\_process | EGR1 | 282 | 5 | 1.540546 | -0.653145 | 612 | 302.04 | 0.493529 |  |
| GO:0031327\_negative\_regulation\_of\_cellular\_biosynthetic\_process | HBA-X | 282 | 5 | 1.540546 | -0.653145 | 612 | 302.04 | 0.493529 |  |
| GO:0031327\_negative\_regulation\_of\_cellular\_biosynthetic\_process | FOXP4 | 282 | 5 | 1.540546 | -0.653145 | 612 | 302.04 | 0.493529 |  |
| GO:0031327\_negative\_regulation\_of\_cellular\_biosynthetic\_process | C1D | 282 | 5 | 1.540546 | -0.653145 | 612 | 302.04 | 0.493529 |  |
| GO:0031327\_negative\_regulation\_of\_cellular\_biosynthetic\_process | CTNNB1 | 282 | 5 | 1.540546 | -0.653145 | 612 | 302.04 | 0.493529 |  |
| GO:0034645\_cellular\_macromolecule\_biosynthetic\_process | EGR1 | 901 | 13 | 1.253639 | -0.649025 | 613 | 302.28 | 0.493116 |  |
| GO:0034645\_cellular\_macromolecule\_biosynthetic\_process | BMP2 | 901 | 13 | 1.253639 | -0.649025 | 613 | 302.28 | 0.493116 |  |
| GO:0034645\_cellular\_macromolecule\_biosynthetic\_process | KLF12 | 901 | 13 | 1.253639 | -0.649025 | 613 | 302.28 | 0.493116 |  |
| GO:0034645\_cellular\_macromolecule\_biosynthetic\_process | TBX5 | 901 | 13 | 1.253639 | -0.649025 | 613 | 302.28 | 0.493116 |  |
| GO:0034645\_cellular\_macromolecule\_biosynthetic\_process | RORA | 901 | 13 | 1.253639 | -0.649025 | 613 | 302.28 | 0.493116 |  |
| GO:0034645\_cellular\_macromolecule\_biosynthetic\_process | FOXP4 | 901 | 13 | 1.253639 | -0.649025 | 613 | 302.28 | 0.493116 |  |
| GO:0034645\_cellular\_macromolecule\_biosynthetic\_process | CTNNB1 | 901 | 13 | 1.253639 | -0.649025 | 613 | 302.28 | 0.493116 |  |
| GO:0034645\_cellular\_macromolecule\_biosynthetic\_process | CITED2 | 901 | 13 | 1.253639 | -0.649025 | 613 | 302.28 | 0.493116 |  |
| GO:0034645\_cellular\_macromolecule\_biosynthetic\_process | APOE | 901 | 13 | 1.253639 | -0.649025 | 613 | 302.28 | 0.493116 |  |
| GO:0034645\_cellular\_macromolecule\_biosynthetic\_process | JUN | 901 | 13 | 1.253639 | -0.649025 | 613 | 302.28 | 0.493116 |  |
| GO:0034645\_cellular\_macromolecule\_biosynthetic\_process | FOXC1 | 901 | 13 | 1.253639 | -0.649025 | 613 | 302.28 | 0.493116 |  |
| GO:0034645\_cellular\_macromolecule\_biosynthetic\_process | HBA-X | 901 | 13 | 1.253639 | -0.649025 | 613 | 302.28 | 0.493116 |  |
| GO:0034645\_cellular\_macromolecule\_biosynthetic\_process | C1D | 901 | 13 | 1.253639 | -0.649025 | 613 | 302.28 | 0.493116 |  |
| GO:0000902\_cell\_morphogenesis | RHOJ | 283 | 5 | 1.535102 | -0.649017 | 614 | 302.42 | 0.492541 |  |
| GO:0000902\_cell\_morphogenesis | APOE | 283 | 5 | 1.535102 | -0.649017 | 614 | 302.42 | 0.492541 |  |
| GO:0000902\_cell\_morphogenesis | PROX1 | 283 | 5 | 1.535102 | -0.649017 | 614 | 302.42 | 0.492541 |  |
| GO:0000902\_cell\_morphogenesis | CTNNB1 | 283 | 5 | 1.535102 | -0.649017 | 614 | 302.42 | 0.492541 |  |
| GO:0000902\_cell\_morphogenesis | TGFB2 | 283 | 5 | 1.535102 | -0.649017 | 614 | 302.42 | 0.492541 |  |
| GO:0001523\_retinoid\_metabolic\_process | RBP4 | 22 | 1 | 3.949400 | -0.647272 | 628 | 311.32 | 0.495732 |  |
| GO:0001569\_patterning\_of\_blood\_vessels | CTNNB1 | 22 | 1 | 3.949400 | -0.647272 | 628 | 311.32 | 0.495732 |  |
| GO:0002637\_regulation\_of\_immunoglobulin\_production | RBP4 | 22 | 1 | 3.949400 | -0.647272 | 628 | 311.32 | 0.495732 |  |
| GO:0006721\_terpenoid\_metabolic\_process | RBP4 | 22 | 1 | 3.949400 | -0.647272 | 628 | 311.32 | 0.495732 |  |
| GO:0009309\_amine\_biosynthetic\_process | TGFB2 | 22 | 1 | 3.949400 | -0.647272 | 628 | 311.32 | 0.495732 |  |
| GO:0010463\_mesenchymal\_cell\_proliferation | PRRX1 | 22 | 1 | 3.949400 | -0.647272 | 628 | 311.32 | 0.495732 |  |
| GO:0010464\_regulation\_of\_mesenchymal\_cell\_proliferation | PRRX1 | 22 | 1 | 3.949400 | -0.647272 | 628 | 311.32 | 0.495732 |  |
| GO:0016101\_diterpenoid\_metabolic\_process | RBP4 | 22 | 1 | 3.949400 | -0.647272 | 628 | 311.32 | 0.495732 |  |
| GO:0021575\_hindbrain\_morphogenesis | RORA | 22 | 1 | 3.949400 | -0.647272 | 628 | 311.32 | 0.495732 |  |
| GO:0030258\_lipid\_modification | APOE | 22 | 1 | 3.949400 | -0.647272 | 628 | 311.32 | 0.495732 |  |
| GO:0030316\_osteoclast\_differentiation | CTNNB1 | 22 | 1 | 3.949400 | -0.647272 | 628 | 311.32 | 0.495732 |  |
| GO:0033273\_response\_to\_vitamin | GDAP1 | 22 | 1 | 3.949400 | -0.647272 | 628 | 311.32 | 0.495732 |  |
| GO:0042733\_embryonic\_digit\_morphogenesis | CTNNB1 | 22 | 1 | 3.949400 | -0.647272 | 628 | 311.32 | 0.495732 |  |
| GO:0048489\_synaptic\_vesicle\_transport | CTNNB1 | 22 | 1 | 3.949400 | -0.647272 | 628 | 311.32 | 0.495732 |  |
| GO:0009890\_negative\_regulation\_of\_biosynthetic\_process | EGR1 | 284 | 5 | 1.529697 | -0.644915 | 629 | 313.0 | 0.497615 |  |
| GO:0009890\_negative\_regulation\_of\_biosynthetic\_process | HBA-X | 284 | 5 | 1.529697 | -0.644915 | 629 | 313.0 | 0.497615 |  |
| GO:0009890\_negative\_regulation\_of\_biosynthetic\_process | FOXP4 | 284 | 5 | 1.529697 | -0.644915 | 629 | 313.0 | 0.497615 |  |
| GO:0009890\_negative\_regulation\_of\_biosynthetic\_process | C1D | 284 | 5 | 1.529697 | -0.644915 | 629 | 313.0 | 0.497615 |  |
| GO:0009890\_negative\_regulation\_of\_biosynthetic\_process | CTNNB1 | 284 | 5 | 1.529697 | -0.644915 | 629 | 313.0 | 0.497615 |  |
| GO:0010467\_gene\_expression | EGR1 | 905 | 13 | 1.248098 | -0.639496 | 630 | 313.76 | 0.498032 |  |
| GO:0010467\_gene\_expression | BMP2 | 905 | 13 | 1.248098 | -0.639496 | 630 | 313.76 | 0.498032 |  |
| GO:0010467\_gene\_expression | KLF12 | 905 | 13 | 1.248098 | -0.639496 | 630 | 313.76 | 0.498032 |  |
| GO:0010467\_gene\_expression | TBX5 | 905 | 13 | 1.248098 | -0.639496 | 630 | 313.76 | 0.498032 |  |
| GO:0010467\_gene\_expression | RORA | 905 | 13 | 1.248098 | -0.639496 | 630 | 313.76 | 0.498032 |  |
| GO:0010467\_gene\_expression | FOXP4 | 905 | 13 | 1.248098 | -0.639496 | 630 | 313.76 | 0.498032 |  |
| GO:0010467\_gene\_expression | CTNNB1 | 905 | 13 | 1.248098 | -0.639496 | 630 | 313.76 | 0.498032 |  |
| GO:0010467\_gene\_expression | CITED2 | 905 | 13 | 1.248098 | -0.639496 | 630 | 313.76 | 0.498032 |  |
| GO:0010467\_gene\_expression | APOE | 905 | 13 | 1.248098 | -0.639496 | 630 | 313.76 | 0.498032 |  |
| GO:0010467\_gene\_expression | JUN | 905 | 13 | 1.248098 | -0.639496 | 630 | 313.76 | 0.498032 |  |
| GO:0010467\_gene\_expression | FOXC1 | 905 | 13 | 1.248098 | -0.639496 | 630 | 313.76 | 0.498032 |  |
| GO:0010467\_gene\_expression | HBA-X | 905 | 13 | 1.248098 | -0.639496 | 630 | 313.76 | 0.498032 |  |
| GO:0010467\_gene\_expression | C1D | 905 | 13 | 1.248098 | -0.639496 | 630 | 313.76 | 0.498032 |  |
| GO:0002204\_somatic\_recombination\_of\_immunoglobulin\_genes\_during\_immune\_response | XRCC4 | 23 | 1 | 3.777687 | -0.630335 | 635 | 320.27 | 0.504362 |  |
| GO:0002208\_somatic\_diversification\_of\_immunoglobulins\_during\_immune\_response | XRCC4 | 23 | 1 | 3.777687 | -0.630335 | 635 | 320.27 | 0.504362 |  |
| GO:0007584\_response\_to\_nutrient | GDAP1 | 23 | 1 | 3.777687 | -0.630335 | 635 | 320.27 | 0.504362 |  |
| GO:0009954\_proximal\_distal\_pattern\_formation | CTNNB1 | 23 | 1 | 3.777687 | -0.630335 | 635 | 320.27 | 0.504362 |  |
| GO:0045190\_isotype\_switching | XRCC4 | 23 | 1 | 3.777687 | -0.630335 | 635 | 320.27 | 0.504362 |  |
| GO:0009059\_macromolecule\_biosynthetic\_process | EGR1 | 910 | 13 | 1.241240 | -0.627743 | 636 | 321.39 | 0.505330 |  |
| GO:0009059\_macromolecule\_biosynthetic\_process | BMP2 | 910 | 13 | 1.241240 | -0.627743 | 636 | 321.39 | 0.505330 |  |
| GO:0009059\_macromolecule\_biosynthetic\_process | KLF12 | 910 | 13 | 1.241240 | -0.627743 | 636 | 321.39 | 0.505330 |  |
| GO:0009059\_macromolecule\_biosynthetic\_process | TBX5 | 910 | 13 | 1.241240 | -0.627743 | 636 | 321.39 | 0.505330 |  |
| GO:0009059\_macromolecule\_biosynthetic\_process | RORA | 910 | 13 | 1.241240 | -0.627743 | 636 | 321.39 | 0.505330 |  |
| GO:0009059\_macromolecule\_biosynthetic\_process | FOXP4 | 910 | 13 | 1.241240 | -0.627743 | 636 | 321.39 | 0.505330 |  |
| GO:0009059\_macromolecule\_biosynthetic\_process | CITED2 | 910 | 13 | 1.241240 | -0.627743 | 636 | 321.39 | 0.505330 |  |
| GO:0009059\_macromolecule\_biosynthetic\_process | CTNNB1 | 910 | 13 | 1.241240 | -0.627743 | 636 | 321.39 | 0.505330 |  |
| GO:0009059\_macromolecule\_biosynthetic\_process | APOE | 910 | 13 | 1.241240 | -0.627743 | 636 | 321.39 | 0.505330 |  |
| GO:0009059\_macromolecule\_biosynthetic\_process | JUN | 910 | 13 | 1.241240 | -0.627743 | 636 | 321.39 | 0.505330 |  |
| GO:0009059\_macromolecule\_biosynthetic\_process | FOXC1 | 910 | 13 | 1.241240 | -0.627743 | 636 | 321.39 | 0.505330 |  |
| GO:0009059\_macromolecule\_biosynthetic\_process | HBA-X | 910 | 13 | 1.241240 | -0.627743 | 636 | 321.39 | 0.505330 |  |
| GO:0009059\_macromolecule\_biosynthetic\_process | C1D | 910 | 13 | 1.241240 | -0.627743 | 636 | 321.39 | 0.505330 |  |
| GO:0003008\_system\_process | RBP4 | 516 | 8 | 1.347082 | -0.624033 | 637 | 322.08 | 0.505620 |  |
| GO:0003008\_system\_process | PTPRM | 516 | 8 | 1.347082 | -0.624033 | 637 | 322.08 | 0.505620 |  |
| GO:0003008\_system\_process | APOE | 516 | 8 | 1.347082 | -0.624033 | 637 | 322.08 | 0.505620 |  |
| GO:0003008\_system\_process | ACTA2 | 516 | 8 | 1.347082 | -0.624033 | 637 | 322.08 | 0.505620 |  |
| GO:0003008\_system\_process | FOXC1 | 516 | 8 | 1.347082 | -0.624033 | 637 | 322.08 | 0.505620 |  |
| GO:0003008\_system\_process | RGS9BP | 516 | 8 | 1.347082 | -0.624033 | 637 | 322.08 | 0.505620 |  |
| GO:0003008\_system\_process | USP14 | 516 | 8 | 1.347082 | -0.624033 | 637 | 322.08 | 0.505620 |  |
| GO:0003008\_system\_process | CTNNB1 | 516 | 8 | 1.347082 | -0.624033 | 637 | 322.08 | 0.505620 |  |
| GO:0040007\_growth | WNT5A | 217 | 4 | 1.601600 | -0.622469 | 638 | 322.42 | 0.505361 |  |
| GO:0040007\_growth | APOE | 217 | 4 | 1.601600 | -0.622469 | 638 | 322.42 | 0.505361 |  |
| GO:0040007\_growth | FOXC1 | 217 | 4 | 1.601600 | -0.622469 | 638 | 322.42 | 0.505361 |  |
| GO:0040007\_growth | PROX1 | 217 | 4 | 1.601600 | -0.622469 | 638 | 322.42 | 0.505361 |  |
| GO:0045892\_negative\_regulation\_of\_transcription\_\_DNA-dependent | EGR1 | 218 | 4 | 1.594253 | -0.617913 | 639 | 323.45 | 0.506182 |  |
| GO:0045892\_negative\_regulation\_of\_transcription\_\_DNA-dependent | HBA-X | 218 | 4 | 1.594253 | -0.617913 | 639 | 323.45 | 0.506182 |  |
| GO:0045892\_negative\_regulation\_of\_transcription\_\_DNA-dependent | FOXP4 | 218 | 4 | 1.594253 | -0.617913 | 639 | 323.45 | 0.506182 |  |
| GO:0045892\_negative\_regulation\_of\_transcription\_\_DNA-dependent | CTNNB1 | 218 | 4 | 1.594253 | -0.617913 | 639 | 323.45 | 0.506182 |  |
| GO:0043085\_positive\_regulation\_of\_catalytic\_activity | VAV3 | 148 | 3 | 1.761219 | -0.616483 | 640 | 323.94 | 0.506156 |  |
| GO:0043085\_positive\_regulation\_of\_catalytic\_activity | LRP8 | 148 | 3 | 1.761219 | -0.616483 | 640 | 323.94 | 0.506156 |  |
| GO:0043085\_positive\_regulation\_of\_catalytic\_activity | ZEB2 | 148 | 3 | 1.761219 | -0.616483 | 640 | 323.94 | 0.506156 |  |
| GO:0001541\_ovarian\_follicle\_development | FOXC1 | 24 | 1 | 3.620283 | -0.614216 | 648 | 330.36 | 0.509815 |  |
| GO:0002381\_immunoglobulin\_production\_during\_immune\_response | XRCC4 | 24 | 1 | 3.620283 | -0.614216 | 648 | 330.36 | 0.509815 |  |
| GO:0042158\_lipoprotein\_biosynthetic\_process | APOE | 24 | 1 | 3.620283 | -0.614216 | 648 | 330.36 | 0.509815 |  |
| GO:0042632\_cholesterol\_homeostasis | APOE | 24 | 1 | 3.620283 | -0.614216 | 648 | 330.36 | 0.509815 |  |
| GO:0043410\_positive\_regulation\_of\_MAPKKK\_cascade | CTNNB1 | 24 | 1 | 3.620283 | -0.614216 | 648 | 330.36 | 0.509815 |  |
| GO:0050679\_positive\_regulation\_of\_epithelial\_cell\_proliferation | CTNNB1 | 24 | 1 | 3.620283 | -0.614216 | 648 | 330.36 | 0.509815 |  |
| GO:0055092\_sterol\_homeostasis | APOE | 24 | 1 | 3.620283 | -0.614216 | 648 | 330.36 | 0.509815 |  |
| GO:0060113\_inner\_ear\_receptor\_cell\_differentiation | DLL1 | 24 | 1 | 3.620283 | -0.614216 | 648 | 330.36 | 0.509815 |  |
| GO:0008202\_steroid\_metabolic\_process | APOE | 82 | 2 | 2.119190 | -0.614034 | 650 | 331.34 | 0.509754 |  |
| GO:0008202\_steroid\_metabolic\_process | CFTR | 82 | 2 | 2.119190 | -0.614034 | 650 | 331.34 | 0.509754 |  |
| GO:0010627\_regulation\_of\_protein\_kinase\_cascade | ZEB2 | 82 | 2 | 2.119190 | -0.614034 | 650 | 331.34 | 0.509754 |  |
| GO:0010627\_regulation\_of\_protein\_kinase\_cascade | CTNNB1 | 82 | 2 | 2.119190 | -0.614034 | 650 | 331.34 | 0.509754 |  |
| GO:0051253\_negative\_regulation\_of\_RNA\_metabolic\_process | EGR1 | 220 | 4 | 1.579760 | -0.608906 | 651 | 332.05 | 0.510061 |  |
| GO:0051253\_negative\_regulation\_of\_RNA\_metabolic\_process | HBA-X | 220 | 4 | 1.579760 | -0.608906 | 651 | 332.05 | 0.510061 |  |
| GO:0051253\_negative\_regulation\_of\_RNA\_metabolic\_process | FOXP4 | 220 | 4 | 1.579760 | -0.608906 | 651 | 332.05 | 0.510061 |  |
| GO:0051253\_negative\_regulation\_of\_RNA\_metabolic\_process | CTNNB1 | 220 | 4 | 1.579760 | -0.608906 | 651 | 332.05 | 0.510061 |  |
| GO:0001701\_in\_utero\_embryonic\_development | XRCC4 | 221 | 4 | 1.572612 | -0.604455 | 652 | 333.64 | 0.511718 |  |
| GO:0001701\_in\_utero\_embryonic\_development | RSPO3 | 221 | 4 | 1.572612 | -0.604455 | 652 | 333.64 | 0.511718 |  |
| GO:0001701\_in\_utero\_embryonic\_development | FOXC1 | 221 | 4 | 1.572612 | -0.604455 | 652 | 333.64 | 0.511718 |  |
| GO:0001701\_in\_utero\_embryonic\_development | CITED2 | 221 | 4 | 1.572612 | -0.604455 | 652 | 333.64 | 0.511718 |  |
| GO:0006139\_nucleobase\_\_nucleoside\_\_nucleotide\_and\_nucleic\_acid\_metabolic\_process | EGR1 | 1002 | 14 | 1.213987 | -0.602938 | 653 | 333.77 | 0.511133 |  |
| GO:0006139\_nucleobase\_\_nucleoside\_\_nucleotide\_and\_nucleic\_acid\_metabolic\_process | XRCC4 | 1002 | 14 | 1.213987 | -0.602938 | 653 | 333.77 | 0.511133 |  |
| GO:0006139\_nucleobase\_\_nucleoside\_\_nucleotide\_and\_nucleic\_acid\_metabolic\_process | ADSSL1 | 1002 | 14 | 1.213987 | -0.602938 | 653 | 333.77 | 0.511133 |  |
| GO:0006139\_nucleobase\_\_nucleoside\_\_nucleotide\_and\_nucleic\_acid\_metabolic\_process | BMP2 | 1002 | 14 | 1.213987 | -0.602938 | 653 | 333.77 | 0.511133 |  |
| GO:0006139\_nucleobase\_\_nucleoside\_\_nucleotide\_and\_nucleic\_acid\_metabolic\_process | KLF12 | 1002 | 14 | 1.213987 | -0.602938 | 653 | 333.77 | 0.511133 |  |
| GO:0006139\_nucleobase\_\_nucleoside\_\_nucleotide\_and\_nucleic\_acid\_metabolic\_process | TBX5 | 1002 | 14 | 1.213987 | -0.602938 | 653 | 333.77 | 0.511133 |  |
| GO:0006139\_nucleobase\_\_nucleoside\_\_nucleotide\_and\_nucleic\_acid\_metabolic\_process | RORA | 1002 | 14 | 1.213987 | -0.602938 | 653 | 333.77 | 0.511133 |  |
| GO:0006139\_nucleobase\_\_nucleoside\_\_nucleotide\_and\_nucleic\_acid\_metabolic\_process | FOXP4 | 1002 | 14 | 1.213987 | -0.602938 | 653 | 333.77 | 0.511133 |  |
| GO:0006139\_nucleobase\_\_nucleoside\_\_nucleotide\_and\_nucleic\_acid\_metabolic\_process | CTNNB1 | 1002 | 14 | 1.213987 | -0.602938 | 653 | 333.77 | 0.511133 |  |
| GO:0006139\_nucleobase\_\_nucleoside\_\_nucleotide\_and\_nucleic\_acid\_metabolic\_process | CITED2 | 1002 | 14 | 1.213987 | -0.602938 | 653 | 333.77 | 0.511133 |  |
| GO:0006139\_nucleobase\_\_nucleoside\_\_nucleotide\_and\_nucleic\_acid\_metabolic\_process | JUN | 1002 | 14 | 1.213987 | -0.602938 | 653 | 333.77 | 0.511133 |  |
| GO:0006139\_nucleobase\_\_nucleoside\_\_nucleotide\_and\_nucleic\_acid\_metabolic\_process | FOXC1 | 1002 | 14 | 1.213987 | -0.602938 | 653 | 333.77 | 0.511133 |  |
| GO:0006139\_nucleobase\_\_nucleoside\_\_nucleotide\_and\_nucleic\_acid\_metabolic\_process | HBA-X | 1002 | 14 | 1.213987 | -0.602938 | 653 | 333.77 | 0.511133 |  |
| GO:0006139\_nucleobase\_\_nucleoside\_\_nucleotide\_and\_nucleic\_acid\_metabolic\_process | C1D | 1002 | 14 | 1.213987 | -0.602938 | 653 | 333.77 | 0.511133 |  |
| GO:0002520\_immune\_system\_development | EGR1 | 295 | 5 | 1.472657 | -0.601478 | 655 | 334.28 | 0.510351 |  |
| GO:0002520\_immune\_system\_development | XRCC4 | 295 | 5 | 1.472657 | -0.601478 | 655 | 334.28 | 0.510351 |  |
| GO:0002520\_immune\_system\_development | HBA-X | 295 | 5 | 1.472657 | -0.601478 | 655 | 334.28 | 0.510351 |  |
| GO:0002520\_immune\_system\_development | TGFB2 | 295 | 5 | 1.472657 | -0.601478 | 655 | 334.28 | 0.510351 |  |
| GO:0002520\_immune\_system\_development | CTNNB1 | 295 | 5 | 1.472657 | -0.601478 | 655 | 334.28 | 0.510351 |  |
| GO:0045595\_regulation\_of\_cell\_differentiation | XRCC4 | 295 | 5 | 1.472657 | -0.601478 | 655 | 334.28 | 0.510351 |  |
| GO:0045595\_regulation\_of\_cell\_differentiation | BMP2 | 295 | 5 | 1.472657 | -0.601478 | 655 | 334.28 | 0.510351 |  |
| GO:0045595\_regulation\_of\_cell\_differentiation | DLL1 | 295 | 5 | 1.472657 | -0.601478 | 655 | 334.28 | 0.510351 |  |
| GO:0045595\_regulation\_of\_cell\_differentiation | PROX1 | 295 | 5 | 1.472657 | -0.601478 | 655 | 334.28 | 0.510351 |  |
| GO:0045595\_regulation\_of\_cell\_differentiation | CTNNB1 | 295 | 5 | 1.472657 | -0.601478 | 655 | 334.28 | 0.510351 |  |
| GO:0002700\_regulation\_of\_production\_of\_molecular\_mediator\_of\_immune\_response | RBP4 | 25 | 1 | 3.475472 | -0.598847 | 664 | 340.79 | 0.513238 |  |
| GO:0006302\_double-strand\_break\_repair | XRCC4 | 25 | 1 | 3.475472 | -0.598847 | 664 | 340.79 | 0.513238 |  |
| GO:0006775\_fat-soluble\_vitamin\_metabolic\_process | RBP4 | 25 | 1 | 3.475472 | -0.598847 | 664 | 340.79 | 0.513238 |  |
| GO:0007492\_endoderm\_development | CTNNB1 | 25 | 1 | 3.475472 | -0.598847 | 664 | 340.79 | 0.513238 |  |
| GO:0021983\_pituitary\_gland\_development | WNT5A | 25 | 1 | 3.475472 | -0.598847 | 664 | 340.79 | 0.513238 |  |
| GO:0031400\_negative\_regulation\_of\_protein\_modification\_process | JUN | 25 | 1 | 3.475472 | -0.598847 | 664 | 340.79 | 0.513238 |  |
| GO:0035137\_hindlimb\_morphogenesis | CTNNB1 | 25 | 1 | 3.475472 | -0.598847 | 664 | 340.79 | 0.513238 |  |
| GO:0060603\_mammary\_gland\_duct\_morphogenesis | WNT5A | 25 | 1 | 3.475472 | -0.598847 | 664 | 340.79 | 0.513238 |  |
| GO:0060688\_regulation\_of\_morphogenesis\_of\_a\_branching\_structure | WNT5A | 25 | 1 | 3.475472 | -0.598847 | 664 | 340.79 | 0.513238 |  |
| GO:0032504\_multicellular\_organism\_reproduction | FOXC1 | 86 | 2 | 2.020623 | -0.584403 | 667 | 343.57 | 0.515097 |  |
| GO:0032504\_multicellular\_organism\_reproduction | CITED2 | 86 | 2 | 2.020623 | -0.584403 | 667 | 343.57 | 0.515097 |  |
| GO:0034641\_cellular\_nitrogen\_compound\_metabolic\_process | RORA | 86 | 2 | 2.020623 | -0.584403 | 667 | 343.57 | 0.515097 |  |
| GO:0034641\_cellular\_nitrogen\_compound\_metabolic\_process | TGFB2 | 86 | 2 | 2.020623 | -0.584403 | 667 | 343.57 | 0.515097 |  |
| GO:0048609\_reproductive\_process\_in\_a\_multicellular\_organism | FOXC1 | 86 | 2 | 2.020623 | -0.584403 | 667 | 343.57 | 0.515097 |  |
| GO:0048609\_reproductive\_process\_in\_a\_multicellular\_organism | CITED2 | 86 | 2 | 2.020623 | -0.584403 | 667 | 343.57 | 0.515097 |  |
| GO:0001658\_branching\_involved\_in\_ureteric\_bud\_morphogenesis | BMP2 | 26 | 1 | 3.341800 | -0.584169 | 676 | 349.07 | 0.516376 |  |
| GO:0006720\_isoprenoid\_metabolic\_process | RBP4 | 26 | 1 | 3.341800 | -0.584169 | 676 | 349.07 | 0.516376 |  |
| GO:0009880\_embryonic\_pattern\_specification | DLL1 | 26 | 1 | 3.341800 | -0.584169 | 676 | 349.07 | 0.516376 |  |
| GO:0010212\_response\_to\_ionizing\_radiation | XRCC4 | 26 | 1 | 3.341800 | -0.584169 | 676 | 349.07 | 0.516376 |  |
| GO:0045665\_negative\_regulation\_of\_neuron\_differentiation | DLL1 | 26 | 1 | 3.341800 | -0.584169 | 676 | 349.07 | 0.516376 |  |
| GO:0048645\_organ\_formation | WNT5A | 26 | 1 | 3.341800 | -0.584169 | 676 | 349.07 | 0.516376 |  |
| GO:0050680\_negative\_regulation\_of\_epithelial\_cell\_proliferation | WNT5A | 26 | 1 | 3.341800 | -0.584169 | 676 | 349.07 | 0.516376 |  |
| GO:0051235\_maintenance\_of\_location | APOE | 26 | 1 | 3.341800 | -0.584169 | 676 | 349.07 | 0.516376 |  |
| GO:0060675\_ureteric\_bud\_morphogenesis | BMP2 | 26 | 1 | 3.341800 | -0.584169 | 676 | 349.07 | 0.516376 |  |
| GO:0009058\_biosynthetic\_process | EGR1 | 1175 | 16 | 1.183139 | -0.583979 | 677 | 349.27 | 0.515908 |  |
| GO:0009058\_biosynthetic\_process | RBP4 | 1175 | 16 | 1.183139 | -0.583979 | 677 | 349.27 | 0.515908 |  |
| GO:0009058\_biosynthetic\_process | BMP2 | 1175 | 16 | 1.183139 | -0.583979 | 677 | 349.27 | 0.515908 |  |
| GO:0009058\_biosynthetic\_process | KLF12 | 1175 | 16 | 1.183139 | -0.583979 | 677 | 349.27 | 0.515908 |  |
| GO:0009058\_biosynthetic\_process | TBX5 | 1175 | 16 | 1.183139 | -0.583979 | 677 | 349.27 | 0.515908 |  |
| GO:0009058\_biosynthetic\_process | CFTR | 1175 | 16 | 1.183139 | -0.583979 | 677 | 349.27 | 0.515908 |  |
| GO:0009058\_biosynthetic\_process | RORA | 1175 | 16 | 1.183139 | -0.583979 | 677 | 349.27 | 0.515908 |  |
| GO:0009058\_biosynthetic\_process | FOXP4 | 1175 | 16 | 1.183139 | -0.583979 | 677 | 349.27 | 0.515908 |  |
| GO:0009058\_biosynthetic\_process | TGFB2 | 1175 | 16 | 1.183139 | -0.583979 | 677 | 349.27 | 0.515908 |  |
| GO:0009058\_biosynthetic\_process | CITED2 | 1175 | 16 | 1.183139 | -0.583979 | 677 | 349.27 | 0.515908 |  |
| GO:0009058\_biosynthetic\_process | CTNNB1 | 1175 | 16 | 1.183139 | -0.583979 | 677 | 349.27 | 0.515908 |  |
| GO:0009058\_biosynthetic\_process | APOE | 1175 | 16 | 1.183139 | -0.583979 | 677 | 349.27 | 0.515908 |  |
| GO:0009058\_biosynthetic\_process | JUN | 1175 | 16 | 1.183139 | -0.583979 | 677 | 349.27 | 0.515908 |  |
| GO:0009058\_biosynthetic\_process | FOXC1 | 1175 | 16 | 1.183139 | -0.583979 | 677 | 349.27 | 0.515908 |  |
| GO:0009058\_biosynthetic\_process | HBA-X | 1175 | 16 | 1.183139 | -0.583979 | 677 | 349.27 | 0.515908 |  |
| GO:0009058\_biosynthetic\_process | C1D | 1175 | 16 | 1.183139 | -0.583979 | 677 | 349.27 | 0.515908 |  |
| GO:0043583\_ear\_development | PRRX1 | 87 | 2 | 1.997398 | -0.577292 | 678 | 351.18 | 0.517965 |  |
| GO:0043583\_ear\_development | DLL1 | 87 | 2 | 1.997398 | -0.577292 | 678 | 351.18 | 0.517965 |  |
| GO:0019953\_sexual\_reproduction | RBP4 | 228 | 4 | 1.524330 | -0.574245 | 679 | 351.82 | 0.518144 |  |
| GO:0019953\_sexual\_reproduction | SEPT4 | 228 | 4 | 1.524330 | -0.574245 | 679 | 351.82 | 0.518144 |  |
| GO:0019953\_sexual\_reproduction | SPACA3 | 228 | 4 | 1.524330 | -0.574245 | 679 | 351.82 | 0.518144 |  |
| GO:0019953\_sexual\_reproduction | FOXC1 | 228 | 4 | 1.524330 | -0.574245 | 679 | 351.82 | 0.518144 |  |
| GO:0002761\_regulation\_of\_myeloid\_leukocyte\_differentiation | CTNNB1 | 27 | 1 | 3.218029 | -0.570129 | 683 | 356.79 | 0.522387 |  |
| GO:0006479\_protein\_amino\_acid\_methylation | BTG2 | 27 | 1 | 3.218029 | -0.570129 | 683 | 356.79 | 0.522387 |  |
| GO:0008213\_protein\_amino\_acid\_alkylation | BTG2 | 27 | 1 | 3.218029 | -0.570129 | 683 | 356.79 | 0.522387 |  |
| GO:0016050\_vesicle\_organization | VAV3 | 27 | 1 | 3.218029 | -0.570129 | 683 | 356.79 | 0.522387 |  |
| GO:0032989\_cellular\_component\_morphogenesis | RHOJ | 307 | 5 | 1.415094 | -0.557416 | 684 | 359.05 | 0.524927 |  |
| GO:0032989\_cellular\_component\_morphogenesis | APOE | 307 | 5 | 1.415094 | -0.557416 | 684 | 359.05 | 0.524927 |  |
| GO:0032989\_cellular\_component\_morphogenesis | PROX1 | 307 | 5 | 1.415094 | -0.557416 | 684 | 359.05 | 0.524927 |  |
| GO:0032989\_cellular\_component\_morphogenesis | CTNNB1 | 307 | 5 | 1.415094 | -0.557416 | 684 | 359.05 | 0.524927 |  |
| GO:0032989\_cellular\_component\_morphogenesis | TGFB2 | 307 | 5 | 1.415094 | -0.557416 | 684 | 359.05 | 0.524927 |  |
| GO:0002062\_chondrocyte\_differentiation | CTNNB1 | 28 | 1 | 3.103100 | -0.556682 | 689 | 363.3 | 0.527286 |  |
| GO:0002088\_lens\_development\_in\_camera-type\_eye | PROX1 | 28 | 1 | 3.103100 | -0.556682 | 689 | 363.3 | 0.527286 |  |
| GO:0021549\_cerebellum\_development | RORA | 28 | 1 | 3.103100 | -0.556682 | 689 | 363.3 | 0.527286 |  |
| GO:0030111\_regulation\_of\_Wnt\_receptor\_signaling\_pathway | ZEB2 | 28 | 1 | 3.103100 | -0.556682 | 689 | 363.3 | 0.527286 |  |
| GO:0046328\_regulation\_of\_JNK\_cascade | ZEB2 | 28 | 1 | 3.103100 | -0.556682 | 689 | 363.3 | 0.527286 |  |
| GO:0051094\_positive\_regulation\_of\_developmental\_process | XRCC4 | 308 | 5 | 1.410500 | -0.553892 | 690 | 364.36 | 0.528058 |  |
| GO:0051094\_positive\_regulation\_of\_developmental\_process | BMP2 | 308 | 5 | 1.410500 | -0.553892 | 690 | 364.36 | 0.528058 |  |
| GO:0051094\_positive\_regulation\_of\_developmental\_process | PROX1 | 308 | 5 | 1.410500 | -0.553892 | 690 | 364.36 | 0.528058 |  |
| GO:0051094\_positive\_regulation\_of\_developmental\_process | CTNNB1 | 308 | 5 | 1.410500 | -0.553892 | 690 | 364.36 | 0.528058 |  |
| GO:0051094\_positive\_regulation\_of\_developmental\_process | TGFB2 | 308 | 5 | 1.410500 | -0.553892 | 690 | 364.36 | 0.528058 |  |
| GO:0051128\_regulation\_of\_cellular\_component\_organization | RHOJ | 160 | 3 | 1.629127 | -0.553760 | 691 | 364.59 | 0.527627 |  |
| GO:0051128\_regulation\_of\_cellular\_component\_organization | PLEK | 160 | 3 | 1.629127 | -0.553760 | 691 | 364.59 | 0.527627 |  |
| GO:0051128\_regulation\_of\_cellular\_component\_organization | PROX1 | 160 | 3 | 1.629127 | -0.553760 | 691 | 364.59 | 0.527627 |  |
| GO:0002521\_leukocyte\_differentiation | EGR1 | 161 | 3 | 1.619009 | -0.548870 | 692 | 366.23 | 0.529234 |  |
| GO:0002521\_leukocyte\_differentiation | XRCC4 | 161 | 3 | 1.619009 | -0.548870 | 692 | 366.23 | 0.529234 |  |
| GO:0002521\_leukocyte\_differentiation | CTNNB1 | 161 | 3 | 1.619009 | -0.548870 | 692 | 366.23 | 0.529234 |  |
| GO:0001934\_positive\_regulation\_of\_protein\_amino\_acid\_phosphorylation | RB1CC1 | 29 | 1 | 2.996096 | -0.543784 | 698 | 372.27 | 0.533338 |  |
| GO:0016447\_somatic\_recombination\_of\_immunoglobulin\_gene\_segments | XRCC4 | 29 | 1 | 2.996096 | -0.543784 | 698 | 372.27 | 0.533338 |  |
| GO:0042490\_mechanoreceptor\_differentiation | DLL1 | 29 | 1 | 2.996096 | -0.543784 | 698 | 372.27 | 0.533338 |  |
| GO:0044087\_regulation\_of\_cellular\_component\_biogenesis | PROX1 | 29 | 1 | 2.996096 | -0.543784 | 698 | 372.27 | 0.533338 |  |
| GO:0060041\_retina\_development\_in\_camera-type\_eye | RBP4 | 29 | 1 | 2.996096 | -0.543784 | 698 | 372.27 | 0.533338 |  |
| GO:0070302\_regulation\_of\_stress-activated\_protein\_kinase\_signaling\_pathway | ZEB2 | 29 | 1 | 2.996096 | -0.543784 | 698 | 372.27 | 0.533338 |  |
| GO:0042110\_T\_cell\_activation | EGR1 | 163 | 3 | 1.599143 | -0.539237 | 699 | 373.41 | 0.534206 |  |
| GO:0042110\_T\_cell\_activation | XRCC4 | 163 | 3 | 1.599143 | -0.539237 | 699 | 373.41 | 0.534206 |  |
| GO:0042110\_T\_cell\_activation | CTNNB1 | 163 | 3 | 1.599143 | -0.539237 | 699 | 373.41 | 0.534206 |  |
| GO:0007049\_cell\_cycle | JUN | 238 | 4 | 1.460282 | -0.533808 | 700 | 375.11 | 0.535871 |  |
| GO:0007049\_cell\_cycle | PROX1 | 238 | 4 | 1.460282 | -0.533808 | 700 | 375.11 | 0.535871 |  |
| GO:0007049\_cell\_cycle | TGFB2 | 238 | 4 | 1.460282 | -0.533808 | 700 | 375.11 | 0.535871 |  |
| GO:0007049\_cell\_cycle | CITED2 | 238 | 4 | 1.460282 | -0.533808 | 700 | 375.11 | 0.535871 |  |
| GO:0006950\_response\_to\_stress | WNT5A | 549 | 8 | 1.266110 | -0.532265 | 701 | 375.21 | 0.535250 |  |
| GO:0006950\_response\_to\_stress | XRCC4 | 549 | 8 | 1.266110 | -0.532265 | 701 | 375.21 | 0.535250 |  |
| GO:0006950\_response\_to\_stress | BMP2 | 549 | 8 | 1.266110 | -0.532265 | 701 | 375.21 | 0.535250 |  |
| GO:0006950\_response\_to\_stress | PLEK | 549 | 8 | 1.266110 | -0.532265 | 701 | 375.21 | 0.535250 |  |
| GO:0006950\_response\_to\_stress | APOE | 549 | 8 | 1.266110 | -0.532265 | 701 | 375.21 | 0.535250 |  |
| GO:0006950\_response\_to\_stress | RB1CC1 | 549 | 8 | 1.266110 | -0.532265 | 701 | 375.21 | 0.535250 |  |
| GO:0006950\_response\_to\_stress | ZEB2 | 549 | 8 | 1.266110 | -0.532265 | 701 | 375.21 | 0.535250 |  |
| GO:0006950\_response\_to\_stress | TCFEC | 549 | 8 | 1.266110 | -0.532265 | 701 | 375.21 | 0.535250 |  |
| GO:0002260\_lymphocyte\_homeostasis | TGFB2 | 30 | 1 | 2.896226 | -0.531399 | 707 | 381.22 | 0.539208 |  |
| GO:0007219\_Notch\_signaling\_pathway | FOXC1 | 30 | 1 | 2.896226 | -0.531399 | 707 | 381.22 | 0.539208 |  |
| GO:0009266\_response\_to\_temperature\_stimulus | TCFEC | 30 | 1 | 2.896226 | -0.531399 | 707 | 381.22 | 0.539208 |  |
| GO:0016445\_somatic\_diversification\_of\_immunoglobulins | XRCC4 | 30 | 1 | 2.896226 | -0.531399 | 707 | 381.22 | 0.539208 |  |
| GO:0022411\_cellular\_component\_disassembly | PLEK | 30 | 1 | 2.896226 | -0.531399 | 707 | 381.22 | 0.539208 |  |
| GO:0060740\_prostate\_gland\_epithelium\_morphogenesis | WNT5A | 30 | 1 | 2.896226 | -0.531399 | 707 | 381.22 | 0.539208 |  |
| GO:0006753\_nucleoside\_phosphate\_metabolic\_process | ADSSL1 | 94 | 2 | 1.848655 | -0.530540 | 709 | 382.42 | 0.539379 |  |
| GO:0006753\_nucleoside\_phosphate\_metabolic\_process | RORA | 94 | 2 | 1.848655 | -0.530540 | 709 | 382.42 | 0.539379 |  |
| GO:0009117\_nucleotide\_metabolic\_process | ADSSL1 | 94 | 2 | 1.848655 | -0.530540 | 709 | 382.42 | 0.539379 |  |
| GO:0009117\_nucleotide\_metabolic\_process | RORA | 94 | 2 | 1.848655 | -0.530540 | 709 | 382.42 | 0.539379 |  |
| GO:0006694\_steroid\_biosynthetic\_process | CFTR | 31 | 1 | 2.802800 | -0.519492 | 717 | 392.58 | 0.547531 |  |
| GO:0006939\_smooth\_muscle\_contraction | ACTA2 | 31 | 1 | 2.802800 | -0.519492 | 717 | 392.58 | 0.547531 |  |
| GO:0010562\_positive\_regulation\_of\_phosphorus\_metabolic\_process | RB1CC1 | 31 | 1 | 2.802800 | -0.519492 | 717 | 392.58 | 0.547531 |  |
| GO:0042157\_lipoprotein\_metabolic\_process | APOE | 31 | 1 | 2.802800 | -0.519492 | 717 | 392.58 | 0.547531 |  |
| GO:0042327\_positive\_regulation\_of\_phosphorylation | RB1CC1 | 31 | 1 | 2.802800 | -0.519492 | 717 | 392.58 | 0.547531 |  |
| GO:0045937\_positive\_regulation\_of\_phosphate\_metabolic\_process | RB1CC1 | 31 | 1 | 2.802800 | -0.519492 | 717 | 392.58 | 0.547531 |  |
| GO:0055088\_lipid\_homeostasis | APOE | 31 | 1 | 2.802800 | -0.519492 | 717 | 392.58 | 0.547531 |  |
| GO:0060512\_prostate\_gland\_morphogenesis | WNT5A | 31 | 1 | 2.802800 | -0.519492 | 717 | 392.58 | 0.547531 |  |
| GO:0018193\_peptidyl-amino\_acid\_modification | A930038C07RIK | 97 | 2 | 1.791480 | -0.511995 | 718 | 394.4 | 0.549304 |  |
| GO:0018193\_peptidyl-amino\_acid\_modification | DCN | 97 | 2 | 1.791480 | -0.511995 | 718 | 394.4 | 0.549304 |  |
| GO:0001707\_mesoderm\_formation | FOXC1 | 32 | 1 | 2.715212 | -0.508033 | 721 | 398.72 | 0.553010 |  |
| GO:0002274\_myeloid\_leukocyte\_activation | RORA | 32 | 1 | 2.715212 | -0.508033 | 721 | 398.72 | 0.553010 |  |
| GO:0048332\_mesoderm\_morphogenesis | FOXC1 | 32 | 1 | 2.715212 | -0.508033 | 721 | 398.72 | 0.553010 |  |
| GO:0007398\_ectoderm\_development | CTNNB1 | 99 | 2 | 1.755289 | -0.500086 | 722 | 400.96 | 0.555346 |  |
| GO:0007398\_ectoderm\_development | TGFB2 | 99 | 2 | 1.755289 | -0.500086 | 722 | 400.96 | 0.555346 |  |
| GO:0009611\_response\_to\_wounding | BMP2 | 172 | 3 | 1.515467 | -0.498169 | 723 | 401.35 | 0.555118 |  |
| GO:0009611\_response\_to\_wounding | PLEK | 172 | 3 | 1.515467 | -0.498169 | 723 | 401.35 | 0.555118 |  |
| GO:0009611\_response\_to\_wounding | APOE | 172 | 3 | 1.515467 | -0.498169 | 723 | 401.35 | 0.555118 |  |
| GO:0001843\_neural\_tube\_closure | ZEB2 | 33 | 1 | 2.632933 | -0.496993 | 731 | 405.36 | 0.554528 |  |
| GO:0002562\_somatic\_diversification\_of\_immune\_receptors\_via\_germline\_recombination\_within\_a\_single\_locus | XRCC4 | 33 | 1 | 2.632933 | -0.496993 | 731 | 405.36 | 0.554528 |  |
| GO:0007565\_female\_pregnancy | CITED2 | 33 | 1 | 2.632933 | -0.496993 | 731 | 405.36 | 0.554528 |  |
| GO:0016444\_somatic\_cell\_DNA\_recombination | XRCC4 | 33 | 1 | 2.632933 | -0.496993 | 731 | 405.36 | 0.554528 |  |
| GO:0021536\_diencephalon\_development | WNT5A | 33 | 1 | 2.632933 | -0.496993 | 731 | 405.36 | 0.554528 |  |
| GO:0021987\_cerebral\_cortex\_development | LRP8 | 33 | 1 | 2.632933 | -0.496993 | 731 | 405.36 | 0.554528 |  |
| GO:0022037\_metencephalon\_development | RORA | 33 | 1 | 2.632933 | -0.496993 | 731 | 405.36 | 0.554528 |  |
| GO:0060606\_tube\_closure | ZEB2 | 33 | 1 | 2.632933 | -0.496993 | 731 | 405.36 | 0.554528 |  |
| GO:0045321\_leukocyte\_activation | EGR1 | 248 | 4 | 1.401400 | -0.496316 | 732 | 405.77 | 0.554331 |  |
| GO:0045321\_leukocyte\_activation | XRCC4 | 248 | 4 | 1.401400 | -0.496316 | 732 | 405.77 | 0.554331 |  |
| GO:0045321\_leukocyte\_activation | RORA | 248 | 4 | 1.401400 | -0.496316 | 732 | 405.77 | 0.554331 |  |
| GO:0045321\_leukocyte\_activation | CTNNB1 | 248 | 4 | 1.401400 | -0.496316 | 732 | 405.77 | 0.554331 |  |
| GO:0044093\_positive\_regulation\_of\_molecular\_function | VAV3 | 173 | 3 | 1.506707 | -0.493824 | 733 | 406.53 | 0.554611 |  |
| GO:0044093\_positive\_regulation\_of\_molecular\_function | LRP8 | 173 | 3 | 1.506707 | -0.493824 | 733 | 406.53 | 0.554611 |  |
| GO:0044093\_positive\_regulation\_of\_molecular\_function | ZEB2 | 173 | 3 | 1.506707 | -0.493824 | 733 | 406.53 | 0.554611 |  |
| GO:0002200\_somatic\_diversification\_of\_immune\_receptors | XRCC4 | 34 | 1 | 2.555494 | -0.486349 | 740 | 412.34 | 0.557216 |  |
| GO:0007338\_single\_fertilization | SPACA3 | 34 | 1 | 2.555494 | -0.486349 | 740 | 412.34 | 0.557216 |  |
| GO:0030509\_BMP\_signaling\_pathway | BMP2 | 34 | 1 | 2.555494 | -0.486349 | 740 | 412.34 | 0.557216 |  |
| GO:0045927\_positive\_regulation\_of\_growth | PROX1 | 34 | 1 | 2.555494 | -0.486349 | 740 | 412.34 | 0.557216 |  |
| GO:0051047\_positive\_regulation\_of\_secretion | RBP4 | 34 | 1 | 2.555494 | -0.486349 | 740 | 412.34 | 0.557216 |  |
| GO:0060443\_mammary\_gland\_morphogenesis | WNT5A | 34 | 1 | 2.555494 | -0.486349 | 740 | 412.34 | 0.557216 |  |
| GO:0060711\_labyrinthine\_layer\_development | RSPO3 | 34 | 1 | 2.555494 | -0.486349 | 740 | 412.34 | 0.557216 |  |
| GO:0042221\_response\_to\_chemical\_stimulus | EGR1 | 409 | 6 | 1.274623 | -0.481663 | 741 | 414.3 | 0.559109 |  |
| GO:0042221\_response\_to\_chemical\_stimulus | NRP2 | 409 | 6 | 1.274623 | -0.481663 | 741 | 414.3 | 0.559109 |  |
| GO:0042221\_response\_to\_chemical\_stimulus | RBP4 | 409 | 6 | 1.274623 | -0.481663 | 741 | 414.3 | 0.559109 |  |
| GO:0042221\_response\_to\_chemical\_stimulus | APOE | 409 | 6 | 1.274623 | -0.481663 | 741 | 414.3 | 0.559109 |  |
| GO:0042221\_response\_to\_chemical\_stimulus | JUN | 409 | 6 | 1.274623 | -0.481663 | 741 | 414.3 | 0.559109 |  |
| GO:0042221\_response\_to\_chemical\_stimulus | GDAP1 | 409 | 6 | 1.274623 | -0.481663 | 741 | 414.3 | 0.559109 |  |
| GO:0016051\_carbohydrate\_biosynthetic\_process | RBP4 | 35 | 1 | 2.482480 | -0.476076 | 744 | 421.25 | 0.566196 |  |
| GO:0043406\_positive\_regulation\_of\_MAP\_kinase\_activity | ZEB2 | 35 | 1 | 2.482480 | -0.476076 | 744 | 421.25 | 0.566196 |  |
| GO:0044242\_cellular\_lipid\_catabolic\_process | APOE | 35 | 1 | 2.482480 | -0.476076 | 744 | 421.25 | 0.566196 |  |
| GO:0044249\_cellular\_biosynthetic\_process | EGR1 | 1150 | 15 | 1.133306 | -0.474377 | 745 | 421.69 | 0.566027 |  |
| GO:0044249\_cellular\_biosynthetic\_process | BMP2 | 1150 | 15 | 1.133306 | -0.474377 | 745 | 421.69 | 0.566027 |  |
| GO:0044249\_cellular\_biosynthetic\_process | KLF12 | 1150 | 15 | 1.133306 | -0.474377 | 745 | 421.69 | 0.566027 |  |
| GO:0044249\_cellular\_biosynthetic\_process | TBX5 | 1150 | 15 | 1.133306 | -0.474377 | 745 | 421.69 | 0.566027 |  |
| GO:0044249\_cellular\_biosynthetic\_process | CFTR | 1150 | 15 | 1.133306 | -0.474377 | 745 | 421.69 | 0.566027 |  |
| GO:0044249\_cellular\_biosynthetic\_process | RORA | 1150 | 15 | 1.133306 | -0.474377 | 745 | 421.69 | 0.566027 |  |
| GO:0044249\_cellular\_biosynthetic\_process | FOXP4 | 1150 | 15 | 1.133306 | -0.474377 | 745 | 421.69 | 0.566027 |  |
| GO:0044249\_cellular\_biosynthetic\_process | TGFB2 | 1150 | 15 | 1.133306 | -0.474377 | 745 | 421.69 | 0.566027 |  |
| GO:0044249\_cellular\_biosynthetic\_process | CTNNB1 | 1150 | 15 | 1.133306 | -0.474377 | 745 | 421.69 | 0.566027 |  |
| GO:0044249\_cellular\_biosynthetic\_process | CITED2 | 1150 | 15 | 1.133306 | -0.474377 | 745 | 421.69 | 0.566027 |  |
| GO:0044249\_cellular\_biosynthetic\_process | APOE | 1150 | 15 | 1.133306 | -0.474377 | 745 | 421.69 | 0.566027 |  |
| GO:0044249\_cellular\_biosynthetic\_process | JUN | 1150 | 15 | 1.133306 | -0.474377 | 745 | 421.69 | 0.566027 |  |
| GO:0044249\_cellular\_biosynthetic\_process | FOXC1 | 1150 | 15 | 1.133306 | -0.474377 | 745 | 421.69 | 0.566027 |  |
| GO:0044249\_cellular\_biosynthetic\_process | HBA-X | 1150 | 15 | 1.133306 | -0.474377 | 745 | 421.69 | 0.566027 |  |
| GO:0044249\_cellular\_biosynthetic\_process | C1D | 1150 | 15 | 1.133306 | -0.474377 | 745 | 421.69 | 0.566027 |  |
| GO:0055086\_nucleobase\_\_nucleoside\_and\_nucleotide\_metabolic\_process | ADSSL1 | 104 | 2 | 1.670900 | -0.471796 | 746 | 422.4 | 0.566220 |  |
| GO:0055086\_nucleobase\_\_nucleoside\_and\_nucleotide\_metabolic\_process | RORA | 104 | 2 | 1.670900 | -0.471796 | 746 | 422.4 | 0.566220 |  |
| GO:0009966\_regulation\_of\_signal\_transduction | PRRX1 | 256 | 4 | 1.357606 | -0.468273 | 747 | 423.67 | 0.567162 |  |
| GO:0009966\_regulation\_of\_signal\_transduction | ZEB2 | 256 | 4 | 1.357606 | -0.468273 | 747 | 423.67 | 0.567162 |  |
| GO:0009966\_regulation\_of\_signal\_transduction | CITED2 | 256 | 4 | 1.357606 | -0.468273 | 747 | 423.67 | 0.567162 |  |
| GO:0009966\_regulation\_of\_signal\_transduction | CTNNB1 | 256 | 4 | 1.357606 | -0.468273 | 747 | 423.67 | 0.567162 |  |
| GO:0048872\_homeostasis\_of\_number\_of\_cells | HBA-X | 105 | 2 | 1.654987 | -0.466379 | 748 | 424.01 | 0.566858 |  |
| GO:0048872\_homeostasis\_of\_number\_of\_cells | TGFB2 | 105 | 2 | 1.654987 | -0.466379 | 748 | 424.01 | 0.566858 |  |
| GO:0014020\_primary\_neural\_tube\_formation | ZEB2 | 36 | 1 | 2.413522 | -0.466153 | 751 | 429.24 | 0.571558 |  |
| GO:0022602\_ovulation\_cycle\_process | FOXC1 | 36 | 1 | 2.413522 | -0.466153 | 751 | 429.24 | 0.571558 |  |
| GO:0051223\_regulation\_of\_protein\_transport | RBP4 | 36 | 1 | 2.413522 | -0.466153 | 751 | 429.24 | 0.571558 |  |
| GO:0042698\_ovulation\_cycle | FOXC1 | 37 | 1 | 2.348292 | -0.456561 | 752 | 434.28 | 0.577500 |  |
| GO:0045859\_regulation\_of\_protein\_kinase\_activity | LRP8 | 107 | 2 | 1.624052 | -0.455772 | 753 | 434.9 | 0.577556 |  |
| GO:0045859\_regulation\_of\_protein\_kinase\_activity | ZEB2 | 107 | 2 | 1.624052 | -0.455772 | 753 | 434.9 | 0.577556 |  |
| GO:0030099\_myeloid\_cell\_differentiation | HBA-X | 108 | 2 | 1.609015 | -0.450579 | 755 | 436.66 | 0.578358 |  |
| GO:0030099\_myeloid\_cell\_differentiation | CTNNB1 | 108 | 2 | 1.609015 | -0.450579 | 755 | 436.66 | 0.578358 |  |
| GO:0051240\_positive\_regulation\_of\_multicellular\_organismal\_process | BMP2 | 108 | 2 | 1.609015 | -0.450579 | 755 | 436.66 | 0.578358 |  |
| GO:0051240\_positive\_regulation\_of\_multicellular\_organismal\_process | PTPRM | 108 | 2 | 1.609015 | -0.450579 | 755 | 436.66 | 0.578358 |  |
| GO:0016192\_vesicle-mediated\_transport | VAV3 | 184 | 3 | 1.416632 | -0.448690 | 756 | 437.06 | 0.578122 |  |
| GO:0016192\_vesicle-mediated\_transport | PLEK | 184 | 3 | 1.416632 | -0.448690 | 756 | 437.06 | 0.578122 |  |
| GO:0016192\_vesicle-mediated\_transport | CTNNB1 | 184 | 3 | 1.416632 | -0.448690 | 756 | 437.06 | 0.578122 |  |
| GO:0001570\_vasculogenesis | CITED2 | 38 | 1 | 2.286495 | -0.447282 | 764 | 443.21 | 0.580118 |  |
| GO:0007596\_blood\_coagulation | PLEK | 38 | 1 | 2.286495 | -0.447282 | 764 | 443.21 | 0.580118 |  |
| GO:0016042\_lipid\_catabolic\_process | APOE | 38 | 1 | 2.286495 | -0.447282 | 764 | 443.21 | 0.580118 |  |
| GO:0031401\_positive\_regulation\_of\_protein\_modification\_process | RB1CC1 | 38 | 1 | 2.286495 | -0.447282 | 764 | 443.21 | 0.580118 |  |
| GO:0032259\_methylation | BTG2 | 38 | 1 | 2.286495 | -0.447282 | 764 | 443.21 | 0.580118 |  |
| GO:0042493\_response\_to\_drug | JUN | 38 | 1 | 2.286495 | -0.447282 | 764 | 443.21 | 0.580118 |  |
| GO:0043414\_biopolymer\_methylation | BTG2 | 38 | 1 | 2.286495 | -0.447282 | 764 | 443.21 | 0.580118 |  |
| GO:0046777\_protein\_amino\_acid\_autophosphorylation | JUN | 38 | 1 | 2.286495 | -0.447282 | 764 | 443.21 | 0.580118 |  |
| GO:0030030\_cell\_projection\_organization | VAV3 | 263 | 4 | 1.321472 | -0.445058 | 765 | 443.99 | 0.580379 |  |
| GO:0030030\_cell\_projection\_organization | APOE | 263 | 4 | 1.321472 | -0.445058 | 765 | 443.99 | 0.580379 |  |
| GO:0030030\_cell\_projection\_organization | PRKG1 | 263 | 4 | 1.321472 | -0.445058 | 765 | 443.99 | 0.580379 |  |
| GO:0030030\_cell\_projection\_organization | TGFB2 | 263 | 4 | 1.321472 | -0.445058 | 765 | 443.99 | 0.580379 |  |
| GO:0007010\_cytoskeleton\_organization | RHOJ | 185 | 3 | 1.408975 | -0.444815 | 766 | 444.24 | 0.579948 |  |
| GO:0007010\_cytoskeleton\_organization | PLEK | 185 | 3 | 1.408975 | -0.444815 | 766 | 444.24 | 0.579948 |  |
| GO:0007010\_cytoskeleton\_organization | PROX1 | 185 | 3 | 1.408975 | -0.444815 | 766 | 444.24 | 0.579948 |  |
| GO:0002376\_immune\_system\_process | EGR1 | 505 | 7 | 1.204371 | -0.442160 | 767 | 444.53 | 0.579570 |  |
| GO:0002376\_immune\_system\_process | RBP4 | 505 | 7 | 1.204371 | -0.442160 | 767 | 444.53 | 0.579570 |  |
| GO:0002376\_immune\_system\_process | XRCC4 | 505 | 7 | 1.204371 | -0.442160 | 767 | 444.53 | 0.579570 |  |
| GO:0002376\_immune\_system\_process | RORA | 505 | 7 | 1.204371 | -0.442160 | 767 | 444.53 | 0.579570 |  |
| GO:0002376\_immune\_system\_process | HBA-X | 505 | 7 | 1.204371 | -0.442160 | 767 | 444.53 | 0.579570 |  |
| GO:0002376\_immune\_system\_process | CTNNB1 | 505 | 7 | 1.204371 | -0.442160 | 767 | 444.53 | 0.579570 |  |
| GO:0002376\_immune\_system\_process | TGFB2 | 505 | 7 | 1.204371 | -0.442160 | 767 | 444.53 | 0.579570 |  |
| GO:0006730\_one-carbon\_metabolic\_process | BTG2 | 39 | 1 | 2.227866 | -0.438300 | 775 | 450.96 | 0.581884 |  |
| GO:0007160\_cell-matrix\_adhesion | CTNNB1 | 39 | 1 | 2.227866 | -0.438300 | 775 | 450.96 | 0.581884 |  |
| GO:0007286\_spermatid\_development | SEPT4 | 39 | 1 | 2.227866 | -0.438300 | 775 | 450.96 | 0.581884 |  |
| GO:0008037\_cell\_recognition | SPACA3 | 39 | 1 | 2.227866 | -0.438300 | 775 | 450.96 | 0.581884 |  |
| GO:0035148\_tube\_lumen\_formation | ZEB2 | 39 | 1 | 2.227866 | -0.438300 | 775 | 450.96 | 0.581884 |  |
| GO:0043524\_negative\_regulation\_of\_neuron\_apoptosis | XRCC4 | 39 | 1 | 2.227866 | -0.438300 | 775 | 450.96 | 0.581884 |  |
| GO:0048663\_neuron\_fate\_commitment | TGFB2 | 39 | 1 | 2.227866 | -0.438300 | 775 | 450.96 | 0.581884 |  |
| GO:0070201\_regulation\_of\_establishment\_of\_protein\_localization | RBP4 | 39 | 1 | 2.227866 | -0.438300 | 775 | 450.96 | 0.581884 |  |
| GO:0007276\_gamete\_generation | RBP4 | 188 | 3 | 1.386491 | -0.433408 | 776 | 452.32 | 0.582887 |  |
| GO:0007276\_gamete\_generation | SEPT4 | 188 | 3 | 1.386491 | -0.433408 | 776 | 452.32 | 0.582887 |  |
| GO:0007276\_gamete\_generation | FOXC1 | 188 | 3 | 1.386491 | -0.433408 | 776 | 452.32 | 0.582887 |  |
| GO:0009987\_cellular\_process | NRP2 | 3868 | 46 | 1.033297 | -0.432032 | 777 | 452.46 | 0.582317 |  |
| GO:0009987\_cellular\_process | RHOJ | 3868 | 46 | 1.033297 | -0.432032 | 777 | 452.46 | 0.582317 |  |
| GO:0009987\_cellular\_process | WNT5A | 3868 | 46 | 1.033297 | -0.432032 | 777 | 452.46 | 0.582317 |  |
| GO:0009987\_cellular\_process | RBP4 | 3868 | 46 | 1.033297 | -0.432032 | 777 | 452.46 | 0.582317 |  |
| GO:0009987\_cellular\_process | SCHIP1 | 3868 | 46 | 1.033297 | -0.432032 | 777 | 452.46 | 0.582317 |  |
| GO:0009987\_cellular\_process | XRCC4 | 3868 | 46 | 1.033297 | -0.432032 | 777 | 452.46 | 0.582317 |  |
| GO:0009987\_cellular\_process | SEPT4 | 3868 | 46 | 1.033297 | -0.432032 | 777 | 452.46 | 0.582317 |  |
| GO:0009987\_cellular\_process | PRRX1 | 3868 | 46 | 1.033297 | -0.432032 | 777 | 452.46 | 0.582317 |  |
| GO:0009987\_cellular\_process | ZEB2 | 3868 | 46 | 1.033297 | -0.432032 | 777 | 452.46 | 0.582317 |  |
| GO:0009987\_cellular\_process | DCN | 3868 | 46 | 1.033297 | -0.432032 | 777 | 452.46 | 0.582317 |  |
| GO:0009987\_cellular\_process | RORA | 3868 | 46 | 1.033297 | -0.432032 | 777 | 452.46 | 0.582317 |  |
| GO:0009987\_cellular\_process | PRKG1 | 3868 | 46 | 1.033297 | -0.432032 | 777 | 452.46 | 0.582317 |  |
| GO:0009987\_cellular\_process | CITED2 | 3868 | 46 | 1.033297 | -0.432032 | 777 | 452.46 | 0.582317 |  |
| GO:0009987\_cellular\_process | TGFB2 | 3868 | 46 | 1.033297 | -0.432032 | 777 | 452.46 | 0.582317 |  |
| GO:0009987\_cellular\_process | CTNNB1 | 3868 | 46 | 1.033297 | -0.432032 | 777 | 452.46 | 0.582317 |  |
| GO:0009987\_cellular\_process | A930038C07RIK | 3868 | 46 | 1.033297 | -0.432032 | 777 | 452.46 | 0.582317 |  |
| GO:0009987\_cellular\_process | APOE | 3868 | 46 | 1.033297 | -0.432032 | 777 | 452.46 | 0.582317 |  |
| GO:0009987\_cellular\_process | RB1CC1 | 3868 | 46 | 1.033297 | -0.432032 | 777 | 452.46 | 0.582317 |  |
| GO:0009987\_cellular\_process | RSPO3 | 3868 | 46 | 1.033297 | -0.432032 | 777 | 452.46 | 0.582317 |  |
| GO:0009987\_cellular\_process | CDC42EP4 | 3868 | 46 | 1.033297 | -0.432032 | 777 | 452.46 | 0.582317 |  |
| GO:0009987\_cellular\_process | TCFEC | 3868 | 46 | 1.033297 | -0.432032 | 777 | 452.46 | 0.582317 |  |
| GO:0009987\_cellular\_process | HBA-X | 3868 | 46 | 1.033297 | -0.432032 | 777 | 452.46 | 0.582317 |  |
| GO:0009987\_cellular\_process | USP14 | 3868 | 46 | 1.033297 | -0.432032 | 777 | 452.46 | 0.582317 |  |
| GO:0009987\_cellular\_process | INSM1 | 3868 | 46 | 1.033297 | -0.432032 | 777 | 452.46 | 0.582317 |  |
| GO:0009987\_cellular\_process | EGR1 | 3868 | 46 | 1.033297 | -0.432032 | 777 | 452.46 | 0.582317 |  |
| GO:0009987\_cellular\_process | ADSSL1 | 3868 | 46 | 1.033297 | -0.432032 | 777 | 452.46 | 0.582317 |  |
| GO:0009987\_cellular\_process | SPACA3 | 3868 | 46 | 1.033297 | -0.432032 | 777 | 452.46 | 0.582317 |  |
| GO:0009987\_cellular\_process | BMP2 | 3868 | 46 | 1.033297 | -0.432032 | 777 | 452.46 | 0.582317 |  |
| GO:0009987\_cellular\_process | ZC3HC1 | 3868 | 46 | 1.033297 | -0.432032 | 777 | 452.46 | 0.582317 |  |
| GO:0009987\_cellular\_process | VAV3 | 3868 | 46 | 1.033297 | -0.432032 | 777 | 452.46 | 0.582317 |  |
| GO:0009987\_cellular\_process | KLF12 | 3868 | 46 | 1.033297 | -0.432032 | 777 | 452.46 | 0.582317 |  |
| GO:0009987\_cellular\_process | SGK3 | 3868 | 46 | 1.033297 | -0.432032 | 777 | 452.46 | 0.582317 |  |
| GO:0009987\_cellular\_process | PLEK | 3868 | 46 | 1.033297 | -0.432032 | 777 | 452.46 | 0.582317 |  |
| GO:0009987\_cellular\_process | TBX5 | 3868 | 46 | 1.033297 | -0.432032 | 777 | 452.46 | 0.582317 |  |
| GO:0009987\_cellular\_process | CCDC80 | 3868 | 46 | 1.033297 | -0.432032 | 777 | 452.46 | 0.582317 |  |
| GO:0009987\_cellular\_process | DLL1 | 3868 | 46 | 1.033297 | -0.432032 | 777 | 452.46 | 0.582317 |  |
| GO:0009987\_cellular\_process | CFTR | 3868 | 46 | 1.033297 | -0.432032 | 777 | 452.46 | 0.582317 |  |
| GO:0009987\_cellular\_process | OPTN | 3868 | 46 | 1.033297 | -0.432032 | 777 | 452.46 | 0.582317 |  |
| GO:0009987\_cellular\_process | FOXP4 | 3868 | 46 | 1.033297 | -0.432032 | 777 | 452.46 | 0.582317 |  |
| GO:0009987\_cellular\_process | PROX1 | 3868 | 46 | 1.033297 | -0.432032 | 777 | 452.46 | 0.582317 |  |
| GO:0009987\_cellular\_process | FZD10 | 3868 | 46 | 1.033297 | -0.432032 | 777 | 452.46 | 0.582317 |  |
| GO:0009987\_cellular\_process | BTG2 | 3868 | 46 | 1.033297 | -0.432032 | 777 | 452.46 | 0.582317 |  |
| GO:0009987\_cellular\_process | JUN | 3868 | 46 | 1.033297 | -0.432032 | 777 | 452.46 | 0.582317 |  |
| GO:0009987\_cellular\_process | FOXC1 | 3868 | 46 | 1.033297 | -0.432032 | 777 | 452.46 | 0.582317 |  |
| GO:0009987\_cellular\_process | LRP8 | 3868 | 46 | 1.033297 | -0.432032 | 777 | 452.46 | 0.582317 |  |
| GO:0009987\_cellular\_process | C1D | 3868 | 46 | 1.033297 | -0.432032 | 777 | 452.46 | 0.582317 |  |
| GO:0001824\_blastocyst\_development | CITED2 | 40 | 1 | 2.172170 | -0.429598 | 782 | 457.43 | 0.584949 |  |
| GO:0007599\_hemostasis | PLEK | 40 | 1 | 2.172170 | -0.429598 | 782 | 457.43 | 0.584949 |  |
| GO:0016358\_dendrite\_development | PRKG1 | 40 | 1 | 2.172170 | -0.429598 | 782 | 457.43 | 0.584949 |  |
| GO:0017015\_regulation\_of\_transforming\_growth\_factor\_beta\_receptor\_signaling\_pathway | CITED2 | 40 | 1 | 2.172170 | -0.429598 | 782 | 457.43 | 0.584949 |  |
| GO:0035272\_exocrine\_system\_development | FOXC1 | 40 | 1 | 2.172170 | -0.429598 | 782 | 457.43 | 0.584949 |  |
| GO:0001776\_leukocyte\_homeostasis | TGFB2 | 41 | 1 | 2.119190 | -0.421164 | 786 | 465.56 | 0.592316 |  |
| GO:0006979\_response\_to\_oxidative\_stress | APOE | 41 | 1 | 2.119190 | -0.421164 | 786 | 465.56 | 0.592316 |  |
| GO:0008585\_female\_gonad\_development | FOXC1 | 41 | 1 | 2.119190 | -0.421164 | 786 | 465.56 | 0.592316 |  |
| GO:0019748\_secondary\_metabolic\_process | RBP4 | 41 | 1 | 2.119190 | -0.421164 | 786 | 465.56 | 0.592316 |  |
| GO:0006006\_glucose\_metabolic\_process | RBP4 | 42 | 1 | 2.068733 | -0.412984 | 794 | 474.25 | 0.597292 |  |
| GO:0008361\_regulation\_of\_cell\_size | RB1CC1 | 42 | 1 | 2.068733 | -0.412984 | 794 | 474.25 | 0.597292 |  |
| GO:0010740\_positive\_regulation\_of\_protein\_kinase\_cascade | CTNNB1 | 42 | 1 | 2.068733 | -0.412984 | 794 | 474.25 | 0.597292 |  |
| GO:0015672\_monovalent\_inorganic\_cation\_transport | ATP6V1E1 | 42 | 1 | 2.068733 | -0.412984 | 794 | 474.25 | 0.597292 |  |
| GO:0045637\_regulation\_of\_myeloid\_cell\_differentiation | CTNNB1 | 42 | 1 | 2.068733 | -0.412984 | 794 | 474.25 | 0.597292 |  |
| GO:0048515\_spermatid\_differentiation | SEPT4 | 42 | 1 | 2.068733 | -0.412984 | 794 | 474.25 | 0.597292 |  |
| GO:0050817\_coagulation | PLEK | 42 | 1 | 2.068733 | -0.412984 | 794 | 474.25 | 0.597292 |  |
| GO:0080135\_regulation\_of\_cellular\_response\_to\_stress | ZEB2 | 42 | 1 | 2.068733 | -0.412984 | 794 | 474.25 | 0.597292 |  |
| GO:0046483\_heterocycle\_metabolic\_process | ADSSL1 | 116 | 2 | 1.498048 | -0.411522 | 796 | 475.0 | 0.596734 |  |
| GO:0046483\_heterocycle\_metabolic\_process | RORA | 116 | 2 | 1.498048 | -0.411522 | 796 | 475.0 | 0.596734 |  |
| GO:0080134\_regulation\_of\_response\_to\_stress | PLEK | 116 | 2 | 1.498048 | -0.411522 | 796 | 475.0 | 0.596734 |  |
| GO:0080134\_regulation\_of\_response\_to\_stress | ZEB2 | 116 | 2 | 1.498048 | -0.411522 | 796 | 475.0 | 0.596734 |  |
| GO:0030182\_neuron\_differentiation | APOE | 356 | 5 | 1.220320 | -0.407979 | 797 | 476.25 | 0.597553 |  |
| GO:0030182\_neuron\_differentiation | DLL1 | 356 | 5 | 1.220320 | -0.407979 | 797 | 476.25 | 0.597553 |  |
| GO:0030182\_neuron\_differentiation | RORA | 356 | 5 | 1.220320 | -0.407979 | 797 | 476.25 | 0.597553 |  |
| GO:0030182\_neuron\_differentiation | PRKG1 | 356 | 5 | 1.220320 | -0.407979 | 797 | 476.25 | 0.597553 |  |
| GO:0030182\_neuron\_differentiation | TGFB2 | 356 | 5 | 1.220320 | -0.407979 | 797 | 476.25 | 0.597553 |  |
| GO:0043933\_macromolecular\_complex\_subunit\_organization | PLEK | 117 | 2 | 1.485244 | -0.406931 | 798 | 476.48 | 0.597093 |  |
| GO:0043933\_macromolecular\_complex\_subunit\_organization | APOE | 117 | 2 | 1.485244 | -0.406931 | 798 | 476.48 | 0.597093 |  |
| GO:0050896\_response\_to\_stimulus | EGR1 | 1107 | 14 | 1.098839 | -0.405373 | 799 | 476.66 | 0.596571 |  |
| GO:0050896\_response\_to\_stimulus | WNT5A | 1107 | 14 | 1.098839 | -0.405373 | 799 | 476.66 | 0.596571 |  |
| GO:0050896\_response\_to\_stimulus | NRP2 | 1107 | 14 | 1.098839 | -0.405373 | 799 | 476.66 | 0.596571 |  |
| GO:0050896\_response\_to\_stimulus | XRCC4 | 1107 | 14 | 1.098839 | -0.405373 | 799 | 476.66 | 0.596571 |  |
| GO:0050896\_response\_to\_stimulus | RBP4 | 1107 | 14 | 1.098839 | -0.405373 | 799 | 476.66 | 0.596571 |  |
| GO:0050896\_response\_to\_stimulus | BMP2 | 1107 | 14 | 1.098839 | -0.405373 | 799 | 476.66 | 0.596571 |  |
| GO:0050896\_response\_to\_stimulus | PLEK | 1107 | 14 | 1.098839 | -0.405373 | 799 | 476.66 | 0.596571 |  |
| GO:0050896\_response\_to\_stimulus | GDAP1 | 1107 | 14 | 1.098839 | -0.405373 | 799 | 476.66 | 0.596571 |  |
| GO:0050896\_response\_to\_stimulus | ZEB2 | 1107 | 14 | 1.098839 | -0.405373 | 799 | 476.66 | 0.596571 |  |
| GO:0050896\_response\_to\_stimulus | RGS9BP | 1107 | 14 | 1.098839 | -0.405373 | 799 | 476.66 | 0.596571 |  |
| GO:0050896\_response\_to\_stimulus | APOE | 1107 | 14 | 1.098839 | -0.405373 | 799 | 476.66 | 0.596571 |  |
| GO:0050896\_response\_to\_stimulus | RB1CC1 | 1107 | 14 | 1.098839 | -0.405373 | 799 | 476.66 | 0.596571 |  |
| GO:0050896\_response\_to\_stimulus | JUN | 1107 | 14 | 1.098839 | -0.405373 | 799 | 476.66 | 0.596571 |  |
| GO:0050896\_response\_to\_stimulus | TCFEC | 1107 | 14 | 1.098839 | -0.405373 | 799 | 476.66 | 0.596571 |  |
| GO:0001841\_neural\_tube\_formation | ZEB2 | 43 | 1 | 2.020623 | -0.405046 | 805 | 481.63 | 0.598298 |  |
| GO:0001894\_tissue\_homeostasis | CTNNB1 | 43 | 1 | 2.020623 | -0.405046 | 805 | 481.63 | 0.598298 |  |
| GO:0006766\_vitamin\_metabolic\_process | RBP4 | 43 | 1 | 2.020623 | -0.405046 | 805 | 481.63 | 0.598298 |  |
| GO:0007224\_smoothened\_signaling\_pathway | PRRX1 | 43 | 1 | 2.020623 | -0.405046 | 805 | 481.63 | 0.598298 |  |
| GO:0032868\_response\_to\_insulin\_stimulus | RBP4 | 43 | 1 | 2.020623 | -0.405046 | 805 | 481.63 | 0.598298 |  |
| GO:0051789\_response\_to\_protein\_stimulus | EGR1 | 43 | 1 | 2.020623 | -0.405046 | 805 | 481.63 | 0.598298 |  |
| GO:0031175\_neuron\_projection\_development | APOE | 197 | 3 | 1.323149 | -0.401030 | 806 | 483.77 | 0.600211 |  |
| GO:0031175\_neuron\_projection\_development | PRKG1 | 197 | 3 | 1.323149 | -0.401030 | 806 | 483.77 | 0.600211 |  |
| GO:0031175\_neuron\_projection\_development | TGFB2 | 197 | 3 | 1.323149 | -0.401030 | 806 | 483.77 | 0.600211 |  |
| GO:0001942\_hair\_follicle\_development | TGFB2 | 44 | 1 | 1.974700 | -0.397339 | 813 | 491.47 | 0.604514 |  |
| GO:0016064\_immunoglobulin\_mediated\_immune\_response | XRCC4 | 44 | 1 | 1.974700 | -0.397339 | 813 | 491.47 | 0.604514 |  |
| GO:0022404\_molting\_cycle\_process | TGFB2 | 44 | 1 | 1.974700 | -0.397339 | 813 | 491.47 | 0.604514 |  |
| GO:0022405\_hair\_cycle\_process | TGFB2 | 44 | 1 | 1.974700 | -0.397339 | 813 | 491.47 | 0.604514 |  |
| GO:0042303\_molting\_cycle | TGFB2 | 44 | 1 | 1.974700 | -0.397339 | 813 | 491.47 | 0.604514 |  |
| GO:0042633\_hair\_cycle | TGFB2 | 44 | 1 | 1.974700 | -0.397339 | 813 | 491.47 | 0.604514 |  |
| GO:0050808\_synapse\_organization | CTNNB1 | 44 | 1 | 1.974700 | -0.397339 | 813 | 491.47 | 0.604514 |  |
| GO:0001838\_embryonic\_epithelial\_tube\_formation | ZEB2 | 45 | 1 | 1.930818 | -0.389851 | 814 | 495.11 | 0.608243 |  |
| GO:0002252\_immune\_effector\_process | RBP4 | 122 | 2 | 1.424374 | -0.384869 | 816 | 498.7 | 0.611152 |  |
| GO:0002252\_immune\_effector\_process | XRCC4 | 122 | 2 | 1.424374 | -0.384869 | 816 | 498.7 | 0.611152 |  |
| GO:0050865\_regulation\_of\_cell\_activation | PLEK | 122 | 2 | 1.424374 | -0.384869 | 816 | 498.7 | 0.611152 |  |
| GO:0050865\_regulation\_of\_cell\_activation | RORA | 122 | 2 | 1.424374 | -0.384869 | 816 | 498.7 | 0.611152 |  |
| GO:0008217\_regulation\_of\_blood\_pressure | ACTA2 | 46 | 1 | 1.888843 | -0.382574 | 819 | 501.65 | 0.612515 |  |
| GO:0019724\_B\_cell\_mediated\_immunity | XRCC4 | 46 | 1 | 1.888843 | -0.382574 | 819 | 501.65 | 0.612515 |  |
| GO:0030218\_erythrocyte\_differentiation | HBA-X | 46 | 1 | 1.888843 | -0.382574 | 819 | 501.65 | 0.612515 |  |
| GO:0006629\_lipid\_metabolic\_process | RBP4 | 285 | 4 | 1.219464 | -0.379345 | 820 | 502.84 | 0.613220 |  |
| GO:0006629\_lipid\_metabolic\_process | VAV3 | 285 | 4 | 1.219464 | -0.379345 | 820 | 502.84 | 0.613220 |  |
| GO:0006629\_lipid\_metabolic\_process | APOE | 285 | 4 | 1.219464 | -0.379345 | 820 | 502.84 | 0.613220 |  |
| GO:0006629\_lipid\_metabolic\_process | CFTR | 285 | 4 | 1.219464 | -0.379345 | 820 | 502.84 | 0.613220 |  |
| GO:0031667\_response\_to\_nutrient\_levels | GDAP1 | 47 | 1 | 1.848655 | -0.375497 | 823 | 507.56 | 0.616719 |  |
| GO:0034754\_cellular\_hormone\_metabolic\_process | RBP4 | 47 | 1 | 1.848655 | -0.375497 | 823 | 507.56 | 0.616719 |  |
| GO:0048871\_multicellular\_organismal\_homeostasis | CTNNB1 | 47 | 1 | 1.848655 | -0.375497 | 823 | 507.56 | 0.616719 |  |
| GO:0051641\_cellular\_localization | RBP4 | 370 | 5 | 1.174146 | -0.372886 | 824 | 508.35 | 0.616930 |  |
| GO:0051641\_cellular\_localization | PLEK | 370 | 5 | 1.174146 | -0.372886 | 824 | 508.35 | 0.616930 |  |
| GO:0051641\_cellular\_localization | APOE | 370 | 5 | 1.174146 | -0.372886 | 824 | 508.35 | 0.616930 |  |
| GO:0051641\_cellular\_localization | OPTN | 370 | 5 | 1.174146 | -0.372886 | 824 | 508.35 | 0.616930 |  |
| GO:0051641\_cellular\_localization | CTNNB1 | 370 | 5 | 1.174146 | -0.372886 | 824 | 508.35 | 0.616930 |  |
| GO:0007498\_mesoderm\_development | FOXC1 | 48 | 1 | 1.810142 | -0.368613 | 827 | 511.48 | 0.618476 |  |
| GO:0019318\_hexose\_metabolic\_process | RBP4 | 48 | 1 | 1.810142 | -0.368613 | 827 | 511.48 | 0.618476 |  |
| GO:0032269\_negative\_regulation\_of\_cellular\_protein\_metabolic\_process | JUN | 48 | 1 | 1.810142 | -0.368613 | 827 | 511.48 | 0.618476 |  |
| GO:0008284\_positive\_regulation\_of\_cell\_proliferation | XRCC4 | 208 | 3 | 1.253175 | -0.364910 | 828 | 511.93 | 0.618273 |  |
| GO:0008284\_positive\_regulation\_of\_cell\_proliferation | PRRX1 | 208 | 3 | 1.253175 | -0.364910 | 828 | 511.93 | 0.618273 |  |
| GO:0008284\_positive\_regulation\_of\_cell\_proliferation | CTNNB1 | 208 | 3 | 1.253175 | -0.364910 | 828 | 511.93 | 0.618273 |  |
| GO:0016043\_cellular\_component\_organization | RHOJ | 964 | 12 | 1.081578 | -0.363675 | 829 | 512.09 | 0.617720 |  |
| GO:0016043\_cellular\_component\_organization | A930038C07RIK | 964 | 12 | 1.081578 | -0.363675 | 829 | 512.09 | 0.617720 |  |
| GO:0016043\_cellular\_component\_organization | SEPT4 | 964 | 12 | 1.081578 | -0.363675 | 829 | 512.09 | 0.617720 |  |
| GO:0016043\_cellular\_component\_organization | VAV3 | 964 | 12 | 1.081578 | -0.363675 | 829 | 512.09 | 0.617720 |  |
| GO:0016043\_cellular\_component\_organization | PLEK | 964 | 12 | 1.081578 | -0.363675 | 829 | 512.09 | 0.617720 |  |
| GO:0016043\_cellular\_component\_organization | APOE | 964 | 12 | 1.081578 | -0.363675 | 829 | 512.09 | 0.617720 |  |
| GO:0016043\_cellular\_component\_organization | CCDC80 | 964 | 12 | 1.081578 | -0.363675 | 829 | 512.09 | 0.617720 |  |
| GO:0016043\_cellular\_component\_organization | FOXC1 | 964 | 12 | 1.081578 | -0.363675 | 829 | 512.09 | 0.617720 |  |
| GO:0016043\_cellular\_component\_organization | PRKG1 | 964 | 12 | 1.081578 | -0.363675 | 829 | 512.09 | 0.617720 |  |
| GO:0016043\_cellular\_component\_organization | PROX1 | 964 | 12 | 1.081578 | -0.363675 | 829 | 512.09 | 0.617720 |  |
| GO:0016043\_cellular\_component\_organization | CTNNB1 | 964 | 12 | 1.081578 | -0.363675 | 829 | 512.09 | 0.617720 |  |
| GO:0016043\_cellular\_component\_organization | TGFB2 | 964 | 12 | 1.081578 | -0.363675 | 829 | 512.09 | 0.617720 |  |
| GO:0006725\_cellular\_aromatic\_compound\_metabolic\_process | TGFB2 | 49 | 1 | 1.773200 | -0.361913 | 831 | 516.42 | 0.621444 |  |
| GO:0034101\_erythrocyte\_homeostasis | HBA-X | 49 | 1 | 1.773200 | -0.361913 | 831 | 516.42 | 0.621444 |  |
| GO:0002573\_myeloid\_leukocyte\_differentiation | CTNNB1 | 50 | 1 | 1.737736 | -0.355389 | 832 | 520.67 | 0.625805 |  |
| GO:0006887\_exocytosis | PLEK | 51 | 1 | 1.703663 | -0.349035 | 834 | 526.57 | 0.631379 |  |
| GO:0032880\_regulation\_of\_protein\_localization | RBP4 | 51 | 1 | 1.703663 | -0.349035 | 834 | 526.57 | 0.631379 |  |
| GO:0048583\_regulation\_of\_response\_to\_stimulus | RBP4 | 217 | 3 | 1.201200 | -0.337898 | 835 | 531.03 | 0.635964 |  |
| GO:0048583\_regulation\_of\_response\_to\_stimulus | PLEK | 217 | 3 | 1.201200 | -0.337898 | 835 | 531.03 | 0.635964 |  |
| GO:0048583\_regulation\_of\_response\_to\_stimulus | ZEB2 | 217 | 3 | 1.201200 | -0.337898 | 835 | 531.03 | 0.635964 |  |
| GO:0007283\_spermatogenesis | SEPT4 | 134 | 2 | 1.296818 | -0.337388 | 837 | 531.71 | 0.635257 |  |
| GO:0007283\_spermatogenesis | RBP4 | 134 | 2 | 1.296818 | -0.337388 | 837 | 531.71 | 0.635257 |  |
| GO:0048232\_male\_gamete\_generation | SEPT4 | 134 | 2 | 1.296818 | -0.337388 | 837 | 531.71 | 0.635257 |  |
| GO:0048232\_male\_gamete\_generation | RBP4 | 134 | 2 | 1.296818 | -0.337388 | 837 | 531.71 | 0.635257 |  |
| GO:0006576\_biogenic\_amine\_metabolic\_process | TGFB2 | 53 | 1 | 1.639373 | -0.336809 | 842 | 534.46 | 0.634751 |  |
| GO:0006935\_chemotaxis | NRP2 | 53 | 1 | 1.639373 | -0.336809 | 842 | 534.46 | 0.634751 |  |
| GO:0030031\_cell\_projection\_assembly | VAV3 | 53 | 1 | 1.639373 | -0.336809 | 842 | 534.46 | 0.634751 |  |
| GO:0042330\_taxis | NRP2 | 53 | 1 | 1.639373 | -0.336809 | 842 | 534.46 | 0.634751 |  |
| GO:0051248\_negative\_regulation\_of\_protein\_metabolic\_process | JUN | 53 | 1 | 1.639373 | -0.336809 | 842 | 534.46 | 0.634751 |  |
| GO:0009566\_fertilization | SPACA3 | 54 | 1 | 1.609015 | -0.330924 | 844 | 539.16 | 0.638815 |  |
| GO:0043405\_regulation\_of\_MAP\_kinase\_activity | ZEB2 | 54 | 1 | 1.609015 | -0.330924 | 844 | 539.16 | 0.638815 |  |
| GO:0006310\_DNA\_recombination | XRCC4 | 55 | 1 | 1.579760 | -0.325183 | 847 | 543.64 | 0.641842 |  |
| GO:0043434\_response\_to\_peptide\_hormone\_stimulus | RBP4 | 55 | 1 | 1.579760 | -0.325183 | 847 | 543.64 | 0.641842 |  |
| GO:0048704\_embryonic\_skeletal\_system\_morphogenesis | PRRX1 | 55 | 1 | 1.579760 | -0.325183 | 847 | 543.64 | 0.641842 |  |
| GO:0007169\_transmembrane\_receptor\_protein\_tyrosine\_kinase\_signaling\_pathway | SCHIP1 | 139 | 2 | 1.250170 | -0.319618 | 849 | 544.93 | 0.641849 |  |
| GO:0007169\_transmembrane\_receptor\_protein\_tyrosine\_kinase\_signaling\_pathway | FOXC1 | 139 | 2 | 1.250170 | -0.319618 | 849 | 544.93 | 0.641849 |  |
| GO:0034613\_cellular\_protein\_localization | OPTN | 139 | 2 | 1.250170 | -0.319618 | 849 | 544.93 | 0.641849 |  |
| GO:0034613\_cellular\_protein\_localization | CTNNB1 | 139 | 2 | 1.250170 | -0.319618 | 849 | 544.93 | 0.641849 |  |
| GO:0001708\_cell\_fate\_specification | CTNNB1 | 56 | 1 | 1.551550 | -0.319582 | 851 | 548.18 | 0.644160 |  |
| GO:0009187\_cyclic\_nucleotide\_metabolic\_process | RORA | 56 | 1 | 1.551550 | -0.319582 | 851 | 548.18 | 0.644160 |  |
| GO:0001764\_neuron\_migration | PRKG1 | 57 | 1 | 1.524330 | -0.314115 | 856 | 553.12 | 0.646168 |  |
| GO:0009953\_dorsal\_ventral\_pattern\_formation | CTNNB1 | 57 | 1 | 1.524330 | -0.314115 | 856 | 553.12 | 0.646168 |  |
| GO:0042472\_inner\_ear\_morphogenesis | PRRX1 | 57 | 1 | 1.524330 | -0.314115 | 856 | 553.12 | 0.646168 |  |
| GO:0043523\_regulation\_of\_neuron\_apoptosis | XRCC4 | 57 | 1 | 1.524330 | -0.314115 | 856 | 553.12 | 0.646168 |  |
| GO:0050878\_regulation\_of\_body\_fluid\_levels | PLEK | 57 | 1 | 1.524330 | -0.314115 | 856 | 553.12 | 0.646168 |  |
| GO:0070727\_cellular\_macromolecule\_localization | OPTN | 141 | 2 | 1.232437 | -0.312809 | 857 | 553.68 | 0.646068 |  |
| GO:0070727\_cellular\_macromolecule\_localization | CTNNB1 | 141 | 2 | 1.232437 | -0.312809 | 857 | 553.68 | 0.646068 |  |
| GO:0030902\_hindbrain\_development | RORA | 58 | 1 | 1.498048 | -0.308777 | 858 | 555.49 | 0.647424 |  |
| GO:0002682\_regulation\_of\_immune\_system\_process | RBP4 | 228 | 3 | 1.143247 | -0.307671 | 860 | 556.47 | 0.647058 |  |
| GO:0002682\_regulation\_of\_immune\_system\_process | RORA | 228 | 3 | 1.143247 | -0.307671 | 860 | 556.47 | 0.647058 |  |
| GO:0002682\_regulation\_of\_immune\_system\_process | CTNNB1 | 228 | 3 | 1.143247 | -0.307671 | 860 | 556.47 | 0.647058 |  |
| GO:0046649\_lymphocyte\_activation | EGR1 | 228 | 3 | 1.143247 | -0.307671 | 860 | 556.47 | 0.647058 |  |
| GO:0046649\_lymphocyte\_activation | XRCC4 | 228 | 3 | 1.143247 | -0.307671 | 860 | 556.47 | 0.647058 |  |
| GO:0046649\_lymphocyte\_activation | CTNNB1 | 228 | 3 | 1.143247 | -0.307671 | 860 | 556.47 | 0.647058 |  |
| GO:0045596\_negative\_regulation\_of\_cell\_differentiation | DLL1 | 144 | 2 | 1.206761 | -0.302900 | 861 | 559.79 | 0.650163 |  |
| GO:0045596\_negative\_regulation\_of\_cell\_differentiation | CTNNB1 | 144 | 2 | 1.206761 | -0.302900 | 861 | 559.79 | 0.650163 |  |
| GO:0009123\_nucleoside\_monophosphate\_metabolic\_process | RORA | 60 | 1 | 1.448113 | -0.298472 | 862 | 560.64 | 0.650394 |  |
| GO:0050790\_regulation\_of\_catalytic\_activity | VAV3 | 233 | 3 | 1.118714 | -0.294863 | 863 | 562.43 | 0.651715 |  |
| GO:0050790\_regulation\_of\_catalytic\_activity | LRP8 | 233 | 3 | 1.118714 | -0.294863 | 863 | 562.43 | 0.651715 |  |
| GO:0050790\_regulation\_of\_catalytic\_activity | ZEB2 | 233 | 3 | 1.118714 | -0.294863 | 863 | 562.43 | 0.651715 |  |
| GO:0006874\_cellular\_calcium\_ion\_homeostasis | APOE | 61 | 1 | 1.424374 | -0.293495 | 866 | 564.48 | 0.651824 |  |
| GO:0007005\_mitochondrion\_organization | SEPT4 | 61 | 1 | 1.424374 | -0.293495 | 866 | 564.48 | 0.651824 |  |
| GO:0009991\_response\_to\_extracellular\_stimulus | GDAP1 | 61 | 1 | 1.424374 | -0.293495 | 866 | 564.48 | 0.651824 |  |
| GO:0022604\_regulation\_of\_cell\_morphogenesis | RHOJ | 62 | 1 | 1.401400 | -0.288632 | 868 | 568.95 | 0.655472 |  |
| GO:0040014\_regulation\_of\_multicellular\_organism\_growth | APOE | 62 | 1 | 1.401400 | -0.288632 | 868 | 568.95 | 0.655472 |  |
| GO:0032940\_secretion\_by\_cell | RBP4 | 149 | 2 | 1.166266 | -0.287156 | 869 | 569.56 | 0.655420 |  |
| GO:0032940\_secretion\_by\_cell | PLEK | 149 | 2 | 1.166266 | -0.287156 | 869 | 569.56 | 0.655420 |  |
| GO:0006875\_cellular\_metal\_ion\_homeostasis | APOE | 64 | 1 | 1.357606 | -0.279226 | 872 | 575.42 | 0.659885 |  |
| GO:0042060\_wound\_healing | PLEK | 64 | 1 | 1.357606 | -0.279226 | 872 | 575.42 | 0.659885 |  |
| GO:0055074\_calcium\_ion\_homeostasis | APOE | 64 | 1 | 1.357606 | -0.279226 | 872 | 575.42 | 0.659885 |  |
| GO:0042471\_ear\_morphogenesis | PRRX1 | 65 | 1 | 1.336720 | -0.274677 | 874 | 577.35 | 0.660584 |  |
| GO:0048511\_rhythmic\_process | FOXC1 | 65 | 1 | 1.336720 | -0.274677 | 874 | 577.35 | 0.660584 |  |
| GO:0010646\_regulation\_of\_cell\_communication | PRRX1 | 330 | 4 | 1.053173 | -0.273195 | 875 | 578.11 | 0.660697 |  |
| GO:0010646\_regulation\_of\_cell\_communication | ZEB2 | 330 | 4 | 1.053173 | -0.273195 | 875 | 578.11 | 0.660697 |  |
| GO:0010646\_regulation\_of\_cell\_communication | CITED2 | 330 | 4 | 1.053173 | -0.273195 | 875 | 578.11 | 0.660697 |  |
| GO:0010646\_regulation\_of\_cell\_communication | CTNNB1 | 330 | 4 | 1.053173 | -0.273195 | 875 | 578.11 | 0.660697 |  |
| GO:0007268\_synaptic\_transmission | USP14 | 154 | 2 | 1.128400 | -0.272315 | 876 | 578.43 | 0.660308 |  |
| GO:0007268\_synaptic\_transmission | CTNNB1 | 154 | 2 | 1.128400 | -0.272315 | 876 | 578.43 | 0.660308 |  |
| GO:0051402\_neuron\_apoptosis | XRCC4 | 66 | 1 | 1.316467 | -0.270227 | 877 | 581.66 | 0.663238 |  |
| GO:0009791\_post-embryonic\_development | SCHIP1 | 67 | 1 | 1.296818 | -0.265873 | 879 | 584.75 | 0.665245 |  |
| GO:0042445\_hormone\_metabolic\_process | RBP4 | 67 | 1 | 1.296818 | -0.265873 | 879 | 584.75 | 0.665245 |  |
| GO:0002697\_regulation\_of\_immune\_effector\_process | RBP4 | 68 | 1 | 1.277747 | -0.261610 | 880 | 586.85 | 0.666875 |  |
| GO:0007409\_axonogenesis | APOE | 158 | 2 | 1.099833 | -0.261049 | 881 | 587.68 | 0.667060 |  |
| GO:0007409\_axonogenesis | TGFB2 | 158 | 2 | 1.099833 | -0.261049 | 881 | 587.68 | 0.667060 |  |
| GO:0005996\_monosaccharide\_metabolic\_process | RBP4 | 69 | 1 | 1.259229 | -0.257438 | 884 | 590.31 | 0.667771 |  |
| GO:0032101\_regulation\_of\_response\_to\_external\_stimulus | PLEK | 69 | 1 | 1.259229 | -0.257438 | 884 | 590.31 | 0.667771 |  |
| GO:0055065\_metal\_ion\_homeostasis | APOE | 69 | 1 | 1.259229 | -0.257438 | 884 | 590.31 | 0.667771 |  |
| GO:0007267\_cell-cell\_signaling | WNT5A | 252 | 3 | 1.034367 | -0.250922 | 885 | 594.8 | 0.672090 |  |
| GO:0007267\_cell-cell\_signaling | USP14 | 252 | 3 | 1.034367 | -0.250922 | 885 | 594.8 | 0.672090 |  |
| GO:0007267\_cell-cell\_signaling | CTNNB1 | 252 | 3 | 1.034367 | -0.250922 | 885 | 594.8 | 0.672090 |  |
| GO:0006281\_DNA\_repair | XRCC4 | 71 | 1 | 1.223758 | -0.249350 | 886 | 597.01 | 0.673826 |  |
| GO:0021915\_neural\_tube\_development | ZEB2 | 72 | 1 | 1.206761 | -0.245430 | 888 | 602.83 | 0.678863 |  |
| GO:0030879\_mammary\_gland\_development | WNT5A | 72 | 1 | 1.206761 | -0.245430 | 888 | 602.83 | 0.678863 |  |
| GO:0006936\_muscle\_contraction | ACTA2 | 73 | 1 | 1.190230 | -0.241590 | 890 | 607.54 | 0.682629 |  |
| GO:0051336\_regulation\_of\_hydrolase\_activity | VAV3 | 73 | 1 | 1.190230 | -0.241590 | 890 | 607.54 | 0.682629 |  |
| GO:0044265\_cellular\_macromolecule\_catabolic\_process | APOE | 75 | 1 | 1.158491 | -0.234138 | 892 | 611.66 | 0.685717 |  |
| GO:0051050\_positive\_regulation\_of\_transport | RBP4 | 75 | 1 | 1.158491 | -0.234138 | 892 | 611.66 | 0.685717 |  |
| GO:0003012\_muscle\_system\_process | ACTA2 | 76 | 1 | 1.143247 | -0.230523 | 895 | 613.73 | 0.685732 |  |
| GO:0009725\_response\_to\_hormone\_stimulus | RBP4 | 76 | 1 | 1.143247 | -0.230523 | 895 | 613.73 | 0.685732 |  |
| GO:0034621\_cellular\_macromolecular\_complex\_subunit\_organization | PLEK | 76 | 1 | 1.143247 | -0.230523 | 895 | 613.73 | 0.685732 |  |
| GO:0048666\_neuron\_development | APOE | 262 | 3 | 0.994887 | -0.230498 | 896 | 614.38 | 0.685692 |  |
| GO:0048666\_neuron\_development | PRKG1 | 262 | 3 | 0.994887 | -0.230498 | 896 | 614.38 | 0.685692 |  |
| GO:0048666\_neuron\_development | TGFB2 | 262 | 3 | 0.994887 | -0.230498 | 896 | 614.38 | 0.685692 |  |
| GO:0048812\_neuron\_projection\_morphogenesis | APOE | 170 | 2 | 1.022198 | -0.230189 | 897 | 614.99 | 0.685608 |  |
| GO:0048812\_neuron\_projection\_morphogenesis | TGFB2 | 170 | 2 | 1.022198 | -0.230189 | 897 | 614.99 | 0.685608 |  |
| GO:0051241\_negative\_regulation\_of\_multicellular\_organismal\_process | RBP4 | 77 | 1 | 1.128400 | -0.226978 | 898 | 616.31 | 0.686314 |  |
| GO:0044255\_cellular\_lipid\_metabolic\_process | RBP4 | 264 | 3 | 0.987350 | -0.226616 | 899 | 616.49 | 0.685751 |  |
| GO:0044255\_cellular\_lipid\_metabolic\_process | APOE | 264 | 3 | 0.987350 | -0.226616 | 899 | 616.49 | 0.685751 |  |
| GO:0044255\_cellular\_lipid\_metabolic\_process | CFTR | 264 | 3 | 0.987350 | -0.226616 | 899 | 616.49 | 0.685751 |  |
| GO:0007600\_sensory\_perception | RBP4 | 172 | 2 | 1.010312 | -0.225440 | 900 | 617.29 | 0.685878 |  |
| GO:0007600\_sensory\_perception | RGS9BP | 172 | 2 | 1.010312 | -0.225440 | 900 | 617.29 | 0.685878 |  |
| GO:0048667\_cell\_morphogenesis\_involved\_in\_neuron\_differentiation | APOE | 173 | 2 | 1.004472 | -0.223105 | 901 | 621.06 | 0.689301 |  |
| GO:0048667\_cell\_morphogenesis\_involved\_in\_neuron\_differentiation | TGFB2 | 173 | 2 | 1.004472 | -0.223105 | 901 | 621.06 | 0.689301 |  |
| GO:0006996\_organelle\_organization | RHOJ | 449 | 5 | 0.967559 | -0.222055 | 902 | 621.58 | 0.689113 |  |
| GO:0006996\_organelle\_organization | SEPT4 | 449 | 5 | 0.967559 | -0.222055 | 902 | 621.58 | 0.689113 |  |
| GO:0006996\_organelle\_organization | VAV3 | 449 | 5 | 0.967559 | -0.222055 | 902 | 621.58 | 0.689113 |  |
| GO:0006996\_organelle\_organization | PLEK | 449 | 5 | 0.967559 | -0.222055 | 902 | 621.58 | 0.689113 |  |
| GO:0006996\_organelle\_organization | PROX1 | 449 | 5 | 0.967559 | -0.222055 | 902 | 621.58 | 0.689113 |  |
| GO:0051046\_regulation\_of\_secretion | RBP4 | 79 | 1 | 1.099833 | -0.220094 | 903 | 622.52 | 0.689391 |  |
| GO:0015031\_protein\_transport | RBP4 | 175 | 2 | 0.992992 | -0.218511 | 905 | 623.36 | 0.688796 |  |
| GO:0015031\_protein\_transport | PLEK | 175 | 2 | 0.992992 | -0.218511 | 905 | 623.36 | 0.688796 |  |
| GO:0046903\_secretion | RBP4 | 175 | 2 | 0.992992 | -0.218511 | 905 | 623.36 | 0.688796 |  |
| GO:0046903\_secretion | PLEK | 175 | 2 | 0.992992 | -0.218511 | 905 | 623.36 | 0.688796 |  |
| GO:0002250\_adaptive\_immune\_response | XRCC4 | 80 | 1 | 1.086085 | -0.216751 | 907 | 625.74 | 0.689901 |  |
| GO:0002460\_adaptive\_immune\_response\_based\_on\_somatic\_recombination\_of\_immune\_receptors\_built\_from\_immunoglobulin\_superfamily\_domains | XRCC4 | 80 | 1 | 1.086085 | -0.216751 | 907 | 625.74 | 0.689901 |  |
| GO:0048858\_cell\_projection\_morphogenesis | APOE | 176 | 2 | 0.987350 | -0.216253 | 908 | 626.62 | 0.690110 |  |
| GO:0048858\_cell\_projection\_morphogenesis | TGFB2 | 176 | 2 | 0.987350 | -0.216253 | 908 | 626.62 | 0.690110 |  |
| GO:0007411\_axon\_guidance | TGFB2 | 82 | 1 | 1.059595 | -0.210253 | 910 | 630.85 | 0.693242 |  |
| GO:0045664\_regulation\_of\_neuron\_differentiation | DLL1 | 82 | 1 | 1.059595 | -0.210253 | 910 | 630.85 | 0.693242 |  |
| GO:0045184\_establishment\_of\_protein\_localization | RBP4 | 180 | 2 | 0.965409 | -0.207463 | 911 | 633.63 | 0.695532 |  |
| GO:0045184\_establishment\_of\_protein\_localization | PLEK | 180 | 2 | 0.965409 | -0.207463 | 911 | 633.63 | 0.695532 |  |
| GO:0006575\_cellular\_amino\_acid\_derivative\_metabolic\_process | TGFB2 | 83 | 1 | 1.046829 | -0.207095 | 913 | 636.48 | 0.697130 |  |
| GO:0050867\_positive\_regulation\_of\_cell\_activation | PLEK | 83 | 1 | 1.046829 | -0.207095 | 913 | 636.48 | 0.697130 |  |
| GO:0030005\_cellular\_di-\_\_tri-valent\_inorganic\_cation\_homeostasis | APOE | 84 | 1 | 1.034367 | -0.203996 | 914 | 638.51 | 0.698589 |  |
| GO:0002449\_lymphocyte\_mediated\_immunity | XRCC4 | 85 | 1 | 1.022198 | -0.200954 | 915 | 641.19 | 0.700754 |  |
| GO:0065009\_regulation\_of\_molecular\_function | VAV3 | 279 | 3 | 0.934267 | -0.199488 | 916 | 641.94 | 0.700808 |  |
| GO:0065009\_regulation\_of\_molecular\_function | LRP8 | 279 | 3 | 0.934267 | -0.199488 | 916 | 641.94 | 0.700808 |  |
| GO:0065009\_regulation\_of\_molecular\_function | ZEB2 | 279 | 3 | 0.934267 | -0.199488 | 916 | 641.94 | 0.700808 |  |
| GO:0032990\_cell\_part\_morphogenesis | APOE | 184 | 2 | 0.944422 | -0.199051 | 917 | 642.53 | 0.700687 |  |
| GO:0032990\_cell\_part\_morphogenesis | TGFB2 | 184 | 2 | 0.944422 | -0.199051 | 917 | 642.53 | 0.700687 |  |
| GO:0022612\_gland\_morphogenesis | WNT5A | 87 | 1 | 0.998699 | -0.195037 | 918 | 648.21 | 0.706111 |  |
| GO:0019226\_transmission\_of\_nerve\_impulse | USP14 | 189 | 2 | 0.919437 | -0.189038 | 919 | 653.03 | 0.710588 |  |
| GO:0019226\_transmission\_of\_nerve\_impulse | CTNNB1 | 189 | 2 | 0.919437 | -0.189038 | 919 | 653.03 | 0.710588 |  |
| GO:0030003\_cellular\_cation\_homeostasis | APOE | 90 | 1 | 0.965409 | -0.186555 | 922 | 654.91 | 0.710315 |  |
| GO:0035264\_multicellular\_organism\_growth | APOE | 90 | 1 | 0.965409 | -0.186555 | 922 | 654.91 | 0.710315 |  |
| GO:0042113\_B\_cell\_activation | XRCC4 | 90 | 1 | 0.965409 | -0.186555 | 922 | 654.91 | 0.710315 |  |
| GO:0002443\_leukocyte\_mediated\_immunity | XRCC4 | 91 | 1 | 0.954800 | -0.183829 | 924 | 656.36 | 0.710346 |  |
| GO:0008544\_epidermis\_development | TGFB2 | 91 | 1 | 0.954800 | -0.183829 | 924 | 656.36 | 0.710346 |  |
| GO:0009719\_response\_to\_endogenous\_stimulus | RBP4 | 92 | 1 | 0.944422 | -0.181150 | 925 | 657.91 | 0.711254 |  |
| GO:0055066\_di-\_\_tri-valent\_inorganic\_cation\_homeostasis | APOE | 93 | 1 | 0.934267 | -0.178518 | 926 | 660.27 | 0.713035 |  |
| GO:0008610\_lipid\_biosynthetic\_process | CFTR | 94 | 1 | 0.924328 | -0.175932 | 928 | 663.77 | 0.715269 |  |
| GO:0034984\_cellular\_response\_to\_DNA\_damage\_stimulus | XRCC4 | 94 | 1 | 0.924328 | -0.175932 | 928 | 663.77 | 0.715269 |  |
| GO:0050877\_neurological\_system\_process | RBP4 | 390 | 4 | 0.891147 | -0.174975 | 929 | 664.21 | 0.714973 |  |
| GO:0050877\_neurological\_system\_process | RGS9BP | 390 | 4 | 0.891147 | -0.174975 | 929 | 664.21 | 0.714973 |  |
| GO:0050877\_neurological\_system\_process | USP14 | 390 | 4 | 0.891147 | -0.174975 | 929 | 664.21 | 0.714973 |  |
| GO:0050877\_neurological\_system\_process | CTNNB1 | 390 | 4 | 0.891147 | -0.174975 | 929 | 664.21 | 0.714973 |  |
| GO:0006954\_inflammatory\_response | BMP2 | 96 | 1 | 0.905071 | -0.170894 | 931 | 669.19 | 0.718786 |  |
| GO:0060249\_anatomical\_structure\_homeostasis | CTNNB1 | 96 | 1 | 0.905071 | -0.170894 | 931 | 669.19 | 0.718786 |  |
| GO:0060341\_regulation\_of\_cellular\_localization | RBP4 | 97 | 1 | 0.895740 | -0.168439 | 932 | 670.37 | 0.719281 |  |
| GO:0022607\_cellular\_component\_assembly | VAV3 | 204 | 2 | 0.851831 | -0.162033 | 933 | 675.55 | 0.724062 |  |
| GO:0022607\_cellular\_component\_assembly | PROX1 | 204 | 2 | 0.851831 | -0.162033 | 933 | 675.55 | 0.724062 |  |
| GO:0001525\_angiogenesis | CTNNB1 | 100 | 1 | 0.868868 | -0.161324 | 934 | 676.0 | 0.723769 |  |
| GO:0006955\_immune\_response | RBP4 | 205 | 2 | 0.847676 | -0.160381 | 935 | 676.7 | 0.723743 |  |
| GO:0006955\_immune\_response | XRCC4 | 205 | 2 | 0.847676 | -0.160381 | 935 | 676.7 | 0.723743 |  |
| GO:0010817\_regulation\_of\_hormone\_levels | RBP4 | 106 | 1 | 0.819687 | -0.148128 | 936 | 683.14 | 0.729850 |  |
| GO:0010033\_response\_to\_organic\_substance | EGR1 | 216 | 2 | 0.804507 | -0.143313 | 937 | 684.69 | 0.730726 |  |
| GO:0010033\_response\_to\_organic\_substance | RBP4 | 216 | 2 | 0.804507 | -0.143313 | 937 | 684.69 | 0.730726 |  |
| GO:0042592\_homeostatic\_process | APOE | 419 | 4 | 0.829468 | -0.140403 | 938 | 685.96 | 0.731301 |  |
| GO:0042592\_homeostatic\_process | HBA-X | 419 | 4 | 0.829468 | -0.140403 | 938 | 685.96 | 0.731301 |  |
| GO:0042592\_homeostatic\_process | TGFB2 | 419 | 4 | 0.829468 | -0.140403 | 938 | 685.96 | 0.731301 |  |
| GO:0042592\_homeostatic\_process | CTNNB1 | 419 | 4 | 0.829468 | -0.140403 | 938 | 685.96 | 0.731301 |  |
| GO:0055080\_cation\_homeostasis | APOE | 110 | 1 | 0.789880 | -0.140029 | 939 | 687.44 | 0.732098 |  |
| GO:0006974\_response\_to\_DNA\_damage\_stimulus | XRCC4 | 113 | 1 | 0.768910 | -0.134289 | 940 | 690.61 | 0.734691 |  |
| GO:0006810\_transport | RBP4 | 718 | 7 | 0.847086 | -0.130100 | 941 | 693.49 | 0.736971 |  |
| GO:0006810\_transport | VAV3 | 718 | 7 | 0.847086 | -0.130100 | 941 | 693.49 | 0.736971 |  |
| GO:0006810\_transport | PLEK | 718 | 7 | 0.847086 | -0.130100 | 941 | 693.49 | 0.736971 |  |
| GO:0006810\_transport | APOE | 718 | 7 | 0.847086 | -0.130100 | 941 | 693.49 | 0.736971 |  |
| GO:0006810\_transport | ATP6V1E1 | 718 | 7 | 0.847086 | -0.130100 | 941 | 693.49 | 0.736971 |  |
| GO:0006810\_transport | CFTR | 718 | 7 | 0.847086 | -0.130100 | 941 | 693.49 | 0.736971 |  |
| GO:0006810\_transport | CTNNB1 | 718 | 7 | 0.847086 | -0.130100 | 941 | 693.49 | 0.736971 |  |
| GO:0006519\_cellular\_amino\_acid\_and\_derivative\_metabolic\_process | TGFB2 | 118 | 1 | 0.736329 | -0.125312 | 942 | 699.14 | 0.742187 |  |
| GO:0051234\_establishment\_of\_localization | RBP4 | 729 | 7 | 0.834304 | -0.121428 | 943 | 700.56 | 0.742906 |  |
| GO:0051234\_establishment\_of\_localization | VAV3 | 729 | 7 | 0.834304 | -0.121428 | 943 | 700.56 | 0.742906 |  |
| GO:0051234\_establishment\_of\_localization | PLEK | 729 | 7 | 0.834304 | -0.121428 | 943 | 700.56 | 0.742906 |  |
| GO:0051234\_establishment\_of\_localization | APOE | 729 | 7 | 0.834304 | -0.121428 | 943 | 700.56 | 0.742906 |  |
| GO:0051234\_establishment\_of\_localization | ATP6V1E1 | 729 | 7 | 0.834304 | -0.121428 | 943 | 700.56 | 0.742906 |  |
| GO:0051234\_establishment\_of\_localization | CFTR | 729 | 7 | 0.834304 | -0.121428 | 943 | 700.56 | 0.742906 |  |
| GO:0051234\_establishment\_of\_localization | CTNNB1 | 729 | 7 | 0.834304 | -0.121428 | 943 | 700.56 | 0.742906 |  |
| GO:0050890\_cognition | RBP4 | 233 | 2 | 0.745809 | -0.120493 | 944 | 700.93 | 0.742511 |  |
| GO:0050890\_cognition | RGS9BP | 233 | 2 | 0.745809 | -0.120493 | 944 | 700.93 | 0.742511 |  |
| GO:0002694\_regulation\_of\_leukocyte\_activation | RORA | 121 | 1 | 0.718073 | -0.120253 | 947 | 702.86 | 0.742196 |  |
| GO:0006917\_induction\_of\_apoptosis | TGFB2 | 121 | 1 | 0.718073 | -0.120253 | 947 | 702.86 | 0.742196 |  |
| GO:0012502\_induction\_of\_programmed\_cell\_death | TGFB2 | 121 | 1 | 0.718073 | -0.120253 | 947 | 702.86 | 0.742196 |  |
| GO:0044085\_cellular\_component\_biogenesis | VAV3 | 237 | 2 | 0.733222 | -0.115680 | 948 | 707.59 | 0.746403 |  |
| GO:0044085\_cellular\_component\_biogenesis | PROX1 | 237 | 2 | 0.733222 | -0.115680 | 948 | 707.59 | 0.746403 |  |
| GO:0032787\_monocarboxylic\_acid\_metabolic\_process | RBP4 | 130 | 1 | 0.668360 | -0.106404 | 950 | 714.43 | 0.752032 |  |
| GO:0050776\_regulation\_of\_immune\_response | RBP4 | 130 | 1 | 0.668360 | -0.106404 | 950 | 714.43 | 0.752032 |  |
| GO:0044057\_regulation\_of\_system\_process | PTPRM | 133 | 1 | 0.653284 | -0.102190 | 951 | 716.75 | 0.753680 |  |
| GO:0009057\_macromolecule\_catabolic\_process | APOE | 137 | 1 | 0.634210 | -0.096855 | 952 | 720.24 | 0.756555 |  |
| GO:0016044\_membrane\_organization | VAV3 | 140 | 1 | 0.620620 | -0.093055 | 953 | 722.85 | 0.758499 |  |
| GO:0007186\_G-protein\_coupled\_receptor\_protein\_signaling\_pathway | PLEK | 144 | 1 | 0.603381 | -0.088240 | 954 | 727.0 | 0.762055 |  |
| GO:0006812\_cation\_transport | ATP6V1E1 | 146 | 1 | 0.595115 | -0.085935 | 955 | 728.31 | 0.762628 |  |
| GO:0051704\_multi-organism\_process | CITED2 | 157 | 1 | 0.553419 | -0.074366 | 956 | 736.43 | 0.770324 |  |
| GO:0007626\_locomotory\_behavior | NRP2 | 163 | 1 | 0.533048 | -0.068771 | 957 | 741.12 | 0.774420 |  |
| GO:0006259\_DNA\_metabolic\_process | XRCC4 | 165 | 1 | 0.526587 | -0.067006 | 958 | 742.41 | 0.774958 |  |
| GO:0043065\_positive\_regulation\_of\_apoptosis | TGFB2 | 166 | 1 | 0.523414 | -0.066142 | 959 | 742.96 | 0.774724 |  |
| GO:0010942\_positive\_regulation\_of\_cell\_death | TGFB2 | 167 | 1 | 0.520280 | -0.065290 | 962 | 744.09 | 0.773482 |  |
| GO:0043068\_positive\_regulation\_of\_programmed\_cell\_death | TGFB2 | 167 | 1 | 0.520280 | -0.065290 | 962 | 744.09 | 0.773482 |  |
| GO:0051049\_regulation\_of\_transport | RBP4 | 167 | 1 | 0.520280 | -0.065290 | 962 | 744.09 | 0.773482 |  |
| GO:0044248\_cellular\_catabolic\_process | APOE | 173 | 1 | 0.502236 | -0.060415 | 963 | 747.91 | 0.776646 |  |
| GO:0006873\_cellular\_ion\_homeostasis | APOE | 176 | 1 | 0.493675 | -0.058123 | 964 | 749.79 | 0.777790 |  |
| GO:0019752\_carboxylic\_acid\_metabolic\_process | RBP4 | 181 | 1 | 0.480038 | -0.054503 | 967 | 752.9 | 0.778594 |  |
| GO:0043436\_oxoacid\_metabolic\_process | RBP4 | 181 | 1 | 0.480038 | -0.054503 | 967 | 752.9 | 0.778594 |  |
| GO:0055082\_cellular\_chemical\_homeostasis | APOE | 181 | 1 | 0.480038 | -0.054503 | 967 | 752.9 | 0.778594 |  |
| GO:0006082\_organic\_acid\_metabolic\_process | RBP4 | 182 | 1 | 0.477400 | -0.053808 | 968 | 753.14 | 0.778037 |  |
| GO:0042180\_cellular\_ketone\_metabolic\_process | RBP4 | 183 | 1 | 0.474791 | -0.053122 | 969 | 753.42 | 0.777523 |  |
| GO:0006811\_ion\_transport | ATP6V1E1 | 186 | 1 | 0.467133 | -0.051119 | 970 | 755.28 | 0.778639 |  |
| GO:0006952\_defense\_response | BMP2 | 187 | 1 | 0.464635 | -0.050469 | 971 | 755.94 | 0.778517 |  |
| GO:0019725\_cellular\_homeostasis | APOE | 195 | 1 | 0.445573 | -0.045567 | 972 | 758.81 | 0.780669 |  |
| GO:0050801\_ion\_homeostasis | APOE | 197 | 1 | 0.441050 | -0.044421 | 973 | 760.15 | 0.781244 |  |
| GO:0051649\_establishment\_of\_localization\_in\_cell | RBP4 | 342 | 2 | 0.508110 | -0.039373 | 974 | 763.69 | 0.784076 |  |
| GO:0051649\_establishment\_of\_localization\_in\_cell | PLEK | 342 | 2 | 0.508110 | -0.039373 | 974 | 763.69 | 0.784076 |  |
| GO:0009056\_catabolic\_process | APOE | 243 | 1 | 0.357559 | -0.024832 | 975 | 773.2 | 0.793026 |  |
| GO:0032879\_regulation\_of\_localization | RBP4 | 248 | 1 | 0.350350 | -0.023319 | 976 | 774.07 | 0.793105 |  |
| GO:0048878\_chemical\_homeostasis | APOE | 254 | 1 | 0.342074 | -0.021625 | 977 | 775.44 | 0.793695 |  |
| GO:0007610\_behavior | NRP2 | 279 | 1 | 0.311422 | -0.015799 | 978 | 779.26 | 0.796789 |  |
| GO:0008150\_biological\_process | NRP2 | 4605 | 53 | 1.000000 | 0.000000 | 2749 | 2748.64 | 0.999869 |  |
| GO:0008150\_biological\_process | RHOJ | 4605 | 53 | 1.000000 | 0.000000 | 2749 | 2748.64 | 0.999869 |  |
| GO:0008150\_biological\_process | SCHIP1 | 4605 | 53 | 1.000000 | 0.000000 | 2749 | 2748.64 | 0.999869 |  |
| GO:0008150\_biological\_process | SEPT4 | 4605 | 53 | 1.000000 | 0.000000 | 2749 | 2748.64 | 0.999869 |  |
| GO:0008150\_biological\_process | XRCC4 | 4605 | 53 | 1.000000 | 0.000000 | 2749 | 2748.64 | 0.999869 |  |
| GO:0008150\_biological\_process | PRRX1 | 4605 | 53 | 1.000000 | 0.000000 | 2749 | 2748.64 | 0.999869 |  |
| GO:0008150\_biological\_process | ZEB2 | 4605 | 53 | 1.000000 | 0.000000 | 2749 | 2748.64 | 0.999869 |  |
| GO:0008150\_biological\_process | RORA | 4605 | 53 | 1.000000 | 0.000000 | 2749 | 2748.64 | 0.999869 |  |
| GO:0008150\_biological\_process | PRKG1 | 4605 | 53 | 1.000000 | 0.000000 | 2749 | 2748.64 | 0.999869 |  |
| GO:0008150\_biological\_process | CTNNB1 | 4605 | 53 | 1.000000 | 0.000000 | 2749 | 2748.64 | 0.999869 |  |
| GO:0008150\_biological\_process | CITED2 | 4605 | 53 | 1.000000 | 0.000000 | 2749 | 2748.64 | 0.999869 |  |
| GO:0008150\_biological\_process | TGFB2 | 4605 | 53 | 1.000000 | 0.000000 | 2749 | 2748.64 | 0.999869 |  |
| GO:0008150\_biological\_process | APOE | 4605 | 53 | 1.000000 | 0.000000 | 2749 | 2748.64 | 0.999869 |  |
| GO:0008150\_biological\_process | RSPO3 | 4605 | 53 | 1.000000 | 0.000000 | 2749 | 2748.64 | 0.999869 |  |
| GO:0008150\_biological\_process | USP14 | 4605 | 53 | 1.000000 | 0.000000 | 2749 | 2748.64 | 0.999869 |  |
| GO:0008150\_biological\_process | EGR1 | 4605 | 53 | 1.000000 | 0.000000 | 2749 | 2748.64 | 0.999869 |  |
| GO:0008150\_biological\_process | SPACA3 | 4605 | 53 | 1.000000 | 0.000000 | 2749 | 2748.64 | 0.999869 |  |
| GO:0008150\_biological\_process | ZC3HC1 | 4605 | 53 | 1.000000 | 0.000000 | 2749 | 2748.64 | 0.999869 |  |
| GO:0008150\_biological\_process | PTPRM | 4605 | 53 | 1.000000 | 0.000000 | 2749 | 2748.64 | 0.999869 |  |
| GO:0008150\_biological\_process | SGK3 | 4605 | 53 | 1.000000 | 0.000000 | 2749 | 2748.64 | 0.999869 |  |
| GO:0008150\_biological\_process | ACTA2 | 4605 | 53 | 1.000000 | 0.000000 | 2749 | 2748.64 | 0.999869 |  |
| GO:0008150\_biological\_process | CFTR | 4605 | 53 | 1.000000 | 0.000000 | 2749 | 2748.64 | 0.999869 |  |
| GO:0008150\_biological\_process | DLL1 | 4605 | 53 | 1.000000 | 0.000000 | 2749 | 2748.64 | 0.999869 |  |
| GO:0008150\_biological\_process | OPTN | 4605 | 53 | 1.000000 | 0.000000 | 2749 | 2748.64 | 0.999869 |  |
| GO:0008150\_biological\_process | PROX1 | 4605 | 53 | 1.000000 | 0.000000 | 2749 | 2748.64 | 0.999869 |  |
| GO:0008150\_biological\_process | BTG2 | 4605 | 53 | 1.000000 | 0.000000 | 2749 | 2748.64 | 0.999869 |  |
| GO:0008150\_biological\_process | JUN | 4605 | 53 | 1.000000 | 0.000000 | 2749 | 2748.64 | 0.999869 |  |
| GO:0008150\_biological\_process | FOXC1 | 4605 | 53 | 1.000000 | 0.000000 | 2749 | 2748.64 | 0.999869 |  |
| GO:0008150\_biological\_process | C1D | 4605 | 53 | 1.000000 | 0.000000 | 2749 | 2748.64 | 0.999869 |  |
| GO:0008150\_biological\_process | WNT5A | 4605 | 53 | 1.000000 | 0.000000 | 2749 | 2748.64 | 0.999869 |  |
| GO:0008150\_biological\_process | RBP4 | 4605 | 53 | 1.000000 | 0.000000 | 2749 | 2748.64 | 0.999869 |  |
| GO:0008150\_biological\_process | GDAP1 | 4605 | 53 | 1.000000 | 0.000000 | 2749 | 2748.64 | 0.999869 |  |
| GO:0008150\_biological\_process | DCN | 4605 | 53 | 1.000000 | 0.000000 | 2749 | 2748.64 | 0.999869 |  |
| GO:0008150\_biological\_process | A930038C07RIK | 4605 | 53 | 1.000000 | 0.000000 | 2749 | 2748.64 | 0.999869 |  |
| GO:0008150\_biological\_process | RB1CC1 | 4605 | 53 | 1.000000 | 0.000000 | 2749 | 2748.64 | 0.999869 |  |
| GO:0008150\_biological\_process | CDC42EP4 | 4605 | 53 | 1.000000 | 0.000000 | 2749 | 2748.64 | 0.999869 |  |
| GO:0008150\_biological\_process | HBA-X | 4605 | 53 | 1.000000 | 0.000000 | 2749 | 2748.64 | 0.999869 |  |
| GO:0008150\_biological\_process | TCFEC | 4605 | 53 | 1.000000 | 0.000000 | 2749 | 2748.64 | 0.999869 |  |
| GO:0008150\_biological\_process | INSM1 | 4605 | 53 | 1.000000 | 0.000000 | 2749 | 2748.64 | 0.999869 |  |
| GO:0008150\_biological\_process | ADSSL1 | 4605 | 53 | 1.000000 | 0.000000 | 2749 | 2748.64 | 0.999869 |  |
| GO:0008150\_biological\_process | BMP2 | 4605 | 53 | 1.000000 | 0.000000 | 2749 | 2748.64 | 0.999869 |  |
| GO:0008150\_biological\_process | VAV3 | 4605 | 53 | 1.000000 | 0.000000 | 2749 | 2748.64 | 0.999869 |  |
| GO:0008150\_biological\_process | PLEK | 4605 | 53 | 1.000000 | 0.000000 | 2749 | 2748.64 | 0.999869 |  |
| GO:0008150\_biological\_process | KLF12 | 4605 | 53 | 1.000000 | 0.000000 | 2749 | 2748.64 | 0.999869 |  |
| GO:0008150\_biological\_process | TBX5 | 4605 | 53 | 1.000000 | 0.000000 | 2749 | 2748.64 | 0.999869 |  |
| GO:0008150\_biological\_process | CCDC80 | 4605 | 53 | 1.000000 | 0.000000 | 2749 | 2748.64 | 0.999869 |  |
| GO:0008150\_biological\_process | COL5A2 | 4605 | 53 | 1.000000 | 0.000000 | 2749 | 2748.64 | 0.999869 |  |
| GO:0008150\_biological\_process | FOXP4 | 4605 | 53 | 1.000000 | 0.000000 | 2749 | 2748.64 | 0.999869 |  |
| GO:0008150\_biological\_process | RGS9BP | 4605 | 53 | 1.000000 | 0.000000 | 2749 | 2748.64 | 0.999869 |  |
| GO:0008150\_biological\_process | PCDH18 | 4605 | 53 | 1.000000 | 0.000000 | 2749 | 2748.64 | 0.999869 |  |
| GO:0008150\_biological\_process | FZD10 | 4605 | 53 | 1.000000 | 0.000000 | 2749 | 2748.64 | 0.999869 |  |
| GO:0008150\_biological\_process | ATP6V1E1 | 4605 | 53 | 1.000000 | 0.000000 | 2749 | 2748.64 | 0.999869 |  |
| GO:0008150\_biological\_process | LRP8 | 4605 | 53 | 1.000000 | 0.000000 | 2749 | 2748.64 | 0.999869 |  |
