# Supplementary material for: RedundancyMiner: De-replication of redundant GO categories in microarray and proteomics analysis
Source: BMC Bioinformatics. 2011 Feb 10;12:52. doi: 10.1186/1471-2105-12-52 (PMC3223614; doi:10.1186/1471-2105-12-52)
Supplement: Additional file 8 — Retinal development HTGM download. compressed package of the results of running HTGM on the retinal development genes list. [file 1471-2105-12-52-S8.ZIP › SCENARIO_2_MODIFIED/total.txt.total.txt.dir/Exp1_BestClusterMap_LEIGS_KM_24.csv.join.8.txt.dir/Exp1_BestClusterMap_LEIGS_KM_24.csv.join.8.txt.change.html]

Category Summary Report for Exp1\_BestClusterMap\_LEIGS\_KM\_24.csv.join.8.txt

# Category Summary Report for Exp1\_BestClusterMap\_LEIGS\_KM\_24.csv.join.8.txt

| HYPERLINKED GO CATEGORY | TOTAL GENES | CHANGED GENES | ENRICHMENT | LOG10(p) | CUMULATIVE NUMBER OF CATEGORIES | CUMULATIVE RANDOMS LOWER BOUND | CUMULATIVE RANDOMS MEAN | CUMULATIVE RANDOMS UPPER BOUND | FALSE DISCOVERY RATE |  |
| --- | --- | --- | --- | --- | --- | --- | --- | --- | --- | --- |
| GO:0048844\_artery\_morphogenesis | 14 | 4 | 24.824798 | -4.842229 | 2 | 0.000000 | 0.0 | 0.000000 | 0.000000 |  |
| GO:0060840\_artery\_development | 14 | 4 | 24.824798 | -4.842229 | 2 | 0.000000 | 0.0 | 0.000000 | 0.000000 |  |
| GO:0007507\_heart\_development | 195 | 10 | 4.455733 | -4.233734 | 3 | -0.120705 | 0.02 | 0.160705 | 0.006667 |  |
| GO:0007417\_central\_nervous\_system\_development | 287 | 12 | 3.632897 | -4.141468 | 4 | -0.120705 | 0.02 | 0.160705 | 0.005000 |  |
| GO:0048513\_organ\_development | 1365 | 28 | 1.782293 | -3.494466 | 5 | -0.561835 | 0.28 | 1.121835 | 0.056000 |  |
| GO:0003018\_vascular\_process\_in\_circulatory\_system | 31 | 4 | 11.211199 | -3.407795 | 8 | -0.699834 | 0.37 | 1.439834 | 0.046250 |  |
| GO:0035150\_regulation\_of\_tube\_size | 31 | 4 | 11.211199 | -3.407795 | 8 | -0.699834 | 0.37 | 1.439834 | 0.046250 |  |
| GO:0050880\_regulation\_of\_blood\_vessel\_size | 31 | 4 | 11.211199 | -3.407795 | 8 | -0.699834 | 0.37 | 1.439834 | 0.046250 |  |
| GO:0060157\_urinary\_bladder\_development | 4 | 2 |  |  |  |  |  |  |  |  |
| GO:0045785\_positive\_regulation\_of\_cell\_adhesion | 37 | 4 | 9.393167 | -3.107952 | 9 | -1.280404 | 0.72 | 2.720404 | 0.080000 |  |
| GO:0006022\_aminoglycan\_metabolic\_process | 18 | 3 | 14.481132 | -2.983025 | 11 | -1.311721 | 0.83 | 2.971721 | 0.075455 |  |
| GO:0030203\_glycosaminoglycan\_metabolic\_process | 18 | 3 | 14.481132 | -2.983025 | 11 | -1.311721 | 0.83 | 2.971721 | 0.075455 |  |
| GO:0007420\_brain\_development | 231 | 9 | 3.385200 | -2.958542 | 12 | -1.318963 | 0.86 | 3.038963 | 0.071667 |  |
| GO:0001945\_lymph\_vessel\_development | 5 | 2 | 34.754717 | -2.895738 | 13 | -1.287173 | 1.33 | 3.947173 | 0.102308 |  |
| GO:0048522\_positive\_regulation\_of\_cellular\_process | 895 | 20 | 1.941604 | -2.850254 | 14 | -1.293838 | 1.43 | 4.153838 | 0.102143 |  |
| GO:0048598\_embryonic\_morphogenesis | 299 | 10 | 2.905913 | -2.737875 | 15 | -1.441710 | 1.66 | 4.761710 | 0.110667 |  |
| GO:0009888\_tissue\_development | 525 | 14 | 2.316981 | -2.731649 | 16 | -1.441710 | 1.66 | 4.761710 | 0.103750 |  |
| GO:0045787\_positive\_regulation\_of\_cell\_cycle | 22 | 3 | 11.848199 | -2.721317 | 17 | -1.213583 | 2.22 | 5.653583 | 0.130588 |  |
| GO:0048514\_blood\_vessel\_morphogenesis | 158 | 7 | 3.849415 | -2.698837 | 18 | -1.284022 | 2.29 | 5.864022 | 0.127222 |  |
| GO:0048518\_positive\_regulation\_of\_biological\_process | 995 | 21 | 1.833792 | -2.668663 | 19 | -1.267977 | 2.31 | 5.887977 | 0.121579 |  |
| GO:0048729\_tissue\_morphogenesis | 255 | 9 | 3.066593 | -2.657764 | 20 | -1.255675 | 2.32 | 5.895675 | 0.116000 |  |
| GO:0048856\_anatomical\_structure\_development | 1688 | 30 | 1.544197 | -2.635741 | 21 | -1.206952 | 2.4 | 6.006952 | 0.114286 |  |
| GO:0048731\_system\_development | 1609 | 29 | 1.566014 | -2.626124 | 22 | -1.191613 | 2.41 | 6.011613 | 0.109545 |  |
| GO:0019800\_peptide\_cross-linking\_via\_chondroitin\_4-sulfate\_glycosaminoglycan | 7 | 2 | 24.824798 | -2.579941 | 23 | -0.957933 | 3.09 | 7.137933 | 0.134348 |  |
| GO:0001655\_urogenital\_system\_development | 128 | 6 | 4.072818 | -2.491239 | 24 | -0.879824 | 3.42 | 7.719824 | 0.142500 |  |
| GO:0007399\_nervous\_system\_development | 621 | 15 | 2.098715 | -2.480383 | 25 | -0.840990 | 3.49 | 7.820990 | 0.139600 |  |
| GO:0030204\_chondroitin\_sulfate\_metabolic\_process | 8 | 2 | 21.721698 | -2.458209 | 26 | -0.717515 | 4.06 | 8.837515 | 0.156154 |  |
| GO:0009790\_embryonic\_development | 567 | 14 | 2.145353 | -2.415966 | 27 | -0.730060 | 4.18 | 9.090060 | 0.154815 |  |
| GO:0048646\_anatomical\_structure\_formation\_involved\_in\_morphogenesis | 277 | 9 | 2.823037 | -2.413873 | 28 | -0.715284 | 4.25 | 9.215284 | 0.151786 |  |
| GO:0051093\_negative\_regulation\_of\_developmental\_process | 331 | 10 | 2.624979 | -2.412540 | 29 | -0.715284 | 4.25 | 9.215284 | 0.146552 |  |
| GO:0016055\_Wnt\_receptor\_signaling\_pathway | 59 | 4 | 5.890630 | -2.350705 | 30 | -0.458647 | 5.23 | 10.918647 | 0.174333 |  |
| GO:0048523\_negative\_regulation\_of\_cellular\_process | 774 | 17 | 1.908366 | -2.343623 | 31 | -0.457988 | 5.24 | 10.937988 | 0.169032 |  |
| GO:0009887\_organ\_morphogenesis | 642 | 15 | 2.030065 | -2.339996 | 32 | -0.464371 | 5.25 | 10.964371 | 0.164062 |  |
| GO:0032501\_multicellular\_organismal\_process | 2183 | 35 | 1.393054 | -2.336298 | 33 | -0.459341 | 5.27 | 10.999341 | 0.159697 |  |
| GO:0014032\_neural\_crest\_cell\_development | 30 | 3 | 8.688679 | -2.328489 | 35 | -0.485117 | 5.38 | 11.245117 | 0.153714 |  |
| GO:0014033\_neural\_crest\_cell\_differentiation | 30 | 3 | 8.688679 | -2.328489 | 35 | -0.485117 | 5.38 | 11.245117 | 0.153714 |  |
| GO:0007275\_multicellular\_organismal\_development | 1760 | 30 | 1.481025 | -2.317847 | 36 | -0.476721 | 5.42 | 11.316721 | 0.150556 |  |
| GO:0050794\_regulation\_of\_cellular\_process | 2190 | 35 | 1.388602 | -2.308145 | 37 | -0.460742 | 5.45 | 11.360742 | 0.147297 |  |
| GO:0007155\_cell\_adhesion | 186 | 7 | 3.269933 | -2.301298 | 39 | -0.439169 | 5.47 | 11.379169 | 0.140256 |  |
| GO:0022610\_biological\_adhesion | 186 | 7 | 3.269933 | -2.301298 | 39 | -0.439169 | 5.47 | 11.379169 | 0.140256 |  |
| GO:0001501\_skeletal\_system\_development | 236 | 8 | 2.945315 | -2.296501 | 40 | -0.494199 | 5.51 | 11.514199 | 0.137750 |  |
| GO:0006916\_anti-apoptosis | 62 | 4 | 5.605600 | -2.273344 | 42 | -0.530662 | 5.82 | 12.170662 | 0.138571 |  |
| GO:0030155\_regulation\_of\_cell\_adhesion | 62 | 4 | 5.605600 | -2.273344 | 42 | -0.530662 | 5.82 | 12.170662 | 0.138571 |  |
| GO:0048468\_cell\_development | 654 | 15 | 1.992816 | -2.263113 | 43 | -0.545545 | 5.83 | 12.205545 | 0.135581 |  |
| GO:0018149\_peptide\_cross-linking | 10 | 2 | 17.377358 | -2.258562 | 46 | -0.143947 | 6.54 | 13.223947 | 0.142174 |  |
| GO:0045669\_positive\_regulation\_of\_osteoblast\_differentiation | 10 | 2 | 17.377358 | -2.258562 | 46 | -0.143947 | 6.54 | 13.223947 | 0.142174 |  |
| GO:0050654\_chondroitin\_sulfate\_proteoglycan\_metabolic\_process | 10 | 2 | 17.377358 | -2.258562 | 46 | -0.143947 | 6.54 | 13.223947 | 0.142174 |  |
| GO:0001756\_somitogenesis | 35 | 3 | 7.447439 | -2.138668 | 48 | 0.083973 | 7.84 | 15.596027 | 0.163333 |  |
| GO:0006869\_lipid\_transport | 35 | 3 | 7.447439 | -2.138668 | 48 | 0.083973 | 7.84 | 15.596027 | 0.163333 |  |
| GO:0032502\_developmental\_process | 2060 | 33 | 1.391876 | -2.131284 | 49 | 0.073916 | 7.88 | 15.686084 | 0.160816 |  |
| GO:0009653\_anatomical\_structure\_morphogenesis | 958 | 19 | 1.723224 | -2.101751 | 50 | 0.053779 | 8.13 | 16.206221 | 0.162600 |  |
| GO:0001568\_blood\_vessel\_development | 203 | 7 | 2.996096 | -2.096388 | 51 | 0.198171 | 8.84 | 17.481829 | 0.173333 |  |
| GO:0048869\_cellular\_developmental\_process | 1113 | 21 | 1.639373 | -2.059363 | 52 | 0.155309 | 9.08 | 18.004691 | 0.174615 |  |
| GO:0001944\_vasculature\_development | 208 | 7 | 2.924075 | -2.040477 | 53 | 0.204895 | 9.31 | 18.415105 | 0.175660 |  |
| GO:0006357\_regulation\_of\_transcription\_from\_RNA\_polymerase\_II\_promoter | 435 | 11 | 2.197137 | -2.032691 | 54 | 0.188361 | 9.63 | 19.071639 | 0.178333 |  |
| GO:0007439\_ectodermal\_gut\_development | 13 | 2 | 13.367199 | -2.029272 | 59 | 0.605416 | 10.28 | 19.954584 | 0.174237 |  |
| GO:0048567\_ectodermal\_gut\_morphogenesis | 13 | 2 | 13.367199 | -2.029272 | 59 | 0.605416 | 10.28 | 19.954584 | 0.174237 |  |
| GO:0051495\_positive\_regulation\_of\_cytoskeleton\_organization | 13 | 2 | 13.367199 | -2.029272 | 59 | 0.605416 | 10.28 | 19.954584 | 0.174237 |  |
| GO:0060038\_cardiac\_muscle\_cell\_proliferation | 13 | 2 | 13.367199 | -2.029272 | 59 | 0.605416 | 10.28 | 19.954584 | 0.174237 |  |
| GO:0060070\_Wnt\_receptor\_signaling\_pathway\_through\_beta-catenin | 13 | 2 | 13.367199 | -2.029272 | 59 | 0.605416 | 10.28 | 19.954584 | 0.174237 |  |
| GO:0050789\_regulation\_of\_biological\_process | 2357 | 36 | 1.327079 | -2.010181 | 60 | 0.560902 | 10.43 | 20.299098 | 0.173833 |  |
| GO:0005976\_polysaccharide\_metabolic\_process | 39 | 3 | 6.683599 | -2.007703 | 61 | 0.479514 | 10.6 | 20.720486 | 0.173770 |  |
| GO:0048771\_tissue\_remodeling | 74 | 4 | 4.696583 | -2.002925 | 62 | 0.492211 | 10.72 | 20.947789 | 0.172903 |  |
| GO:0035295\_tube\_development | 212 | 7 | 2.868904 | -1.997054 | 63 | 0.524273 | 10.76 | 20.995727 | 0.170794 |  |
| GO:0031325\_positive\_regulation\_of\_cellular\_metabolic\_process | 442 | 11 | 2.162341 | -1.982454 | 64 | 0.557485 | 10.86 | 21.162515 | 0.169688 |  |
| GO:0014031\_mesenchymal\_cell\_development | 40 | 3 | 6.516509 | -1.977347 | 65 | 0.660247 | 11.05 | 21.439753 | 0.170000 |  |
| GO:0006366\_transcription\_from\_RNA\_polymerase\_II\_promoter | 444 | 11 | 2.152601 | -1.968330 | 66 | 0.566637 | 11.15 | 21.733363 | 0.168939 |  |
| GO:0014855\_striated\_muscle\_cell\_proliferation | 14 | 2 | 12.412399 | -1.965517 | 67 | 0.909219 | 11.86 | 22.810781 | 0.177015 |  |
| GO:0045944\_positive\_regulation\_of\_transcription\_from\_RNA\_polymerase\_II\_promoter | 269 | 8 | 2.583994 | -1.962275 | 68 | 0.879816 | 11.9 | 22.920184 | 0.175000 |  |
| GO:0030154\_cell\_differentiation | 1060 | 20 | 1.639373 | -1.961807 | 69 | 0.896606 | 11.91 | 22.923394 | 0.172609 |  |
| GO:0007254\_JNK\_cascade | 41 | 3 | 6.357570 | -1.947847 | 70 | 0.801988 | 12.16 | 23.518012 | 0.173714 |  |
| GO:0007284\_spermatogonial\_cell\_division | 1 | 1 |  |  |  |  |  |  |  |  |
| GO:0010543\_regulation\_of\_platelet\_activation | 1 | 1 |  |  |  |  |  |  |  |  |
| GO:0010572\_positive\_regulation\_of\_platelet\_activation | 1 | 1 |  |  |  |  |  |  |  |  |
| GO:0010873\_positive\_regulation\_of\_cholesterol\_esterification | 1 | 1 |  |  |  |  |  |  |  |  |
| GO:0030382\_sperm\_mitochondrion\_organization | 1 | 1 |  |  |  |  |  |  |  |  |
| GO:0034372\_very-low-density\_lipoprotein\_particle\_remodeling | 1 | 1 |  |  |  |  |  |  |  |  |
| GO:0034633\_retinol\_transport | 1 | 1 |  |  |  |  |  |  |  |  |
| GO:0035026\_leading\_edge\_cell\_differentiation | 1 | 1 |  |  |  |  |  |  |  |  |
| GO:0045909\_positive\_regulation\_of\_vasodilation | 1 | 1 |  |  |  |  |  |  |  |  |
| GO:0046864\_isoprenoid\_transport | 1 | 1 |  |  |  |  |  |  |  |  |
| GO:0046865\_terpenoid\_transport | 1 | 1 |  |  |  |  |  |  |  |  |
| GO:0055005\_ventricular\_cardiac\_myofibril\_development | 1 | 1 |  |  |  |  |  |  |  |  |
| GO:0060059\_embryonic\_retina\_morphogenesis\_in\_camera-type\_eye | 1 | 1 |  |  |  |  |  |  |  |  |
| GO:0060297\_regulation\_of\_sarcomere\_organization | 1 | 1 |  |  |  |  |  |  |  |  |
| GO:0060298\_positive\_regulation\_of\_sarcomere\_organization | 1 | 1 |  |  |  |  |  |  |  |  |
| GO:0060414\_aorta\_smooth\_muscle\_tissue\_morphogenesis | 1 | 1 |  |  |  |  |  |  |  |  |
| GO:0060419\_heart\_growth | 1 | 1 |  |  |  |  |  |  |  |  |
| GO:0060420\_regulation\_of\_heart\_growth | 1 | 1 |  |  |  |  |  |  |  |  |
| GO:0060421\_positive\_regulation\_of\_heart\_growth | 1 | 1 |  |  |  |  |  |  |  |  |
| GO:0070493\_thrombin\_receptor\_signaling\_pathway | 1 | 1 |  |  |  |  |  |  |  |  |
| GO:0070527\_platelet\_aggregation | 1 | 1 |  |  |  |  |  |  |  |  |
| GO:0070528\_protein\_kinase\_C\_signaling\_cascade | 1 | 1 |  |  |  |  |  |  |  |  |
| GO:0070560\_protein\_secretion\_by\_platelet | 1 | 1 |  |  |  |  |  |  |  |  |
| GO:0030326\_embryonic\_limb\_morphogenesis | 78 | 4 | 4.455733 | -1.924248 | 72 | 0.785613 | 12.46 | 24.134387 | 0.173056 |  |
| GO:0035113\_embryonic\_appendage\_morphogenesis | 78 | 4 | 4.455733 | -1.924248 | 72 | 0.785613 | 12.46 | 24.134387 | 0.173056 |  |
| GO:0055010\_ventricular\_cardiac\_muscle\_morphogenesis | 15 | 2 | 11.584906 | -1.906559 | 73 | 1.452021 | 13.66 | 25.867979 | 0.187123 |  |
| GO:0031098\_stress-activated\_protein\_kinase\_signaling\_pathway | 43 | 3 | 6.061869 | -1.891250 | 75 | 1.495561 | 13.99 | 26.484439 | 0.186533 |  |
| GO:0048762\_mesenchymal\_cell\_differentiation | 43 | 3 | 6.061869 | -1.891250 | 75 | 1.495561 | 13.99 | 26.484439 | 0.186533 |  |
| GO:0048519\_negative\_regulation\_of\_biological\_process | 859 | 17 | 1.719529 | -1.883542 | 76 | 1.399803 | 14.16 | 26.920197 | 0.186316 |  |
| GO:0009893\_positive\_regulation\_of\_metabolic\_process | 458 | 11 | 2.086801 | -1.872226 | 77 | 1.388260 | 14.32 | 27.251740 | 0.185974 |  |
| GO:0035282\_segmentation | 44 | 3 | 5.924099 | -1.864078 | 80 | 1.445134 | 14.6 | 27.754866 | 0.182500 |  |
| GO:0048593\_camera-type\_eye\_morphogenesis | 44 | 3 | 5.924099 | -1.864078 | 80 | 1.445134 | 14.6 | 27.754866 | 0.182500 |  |
| GO:0060485\_mesenchyme\_development | 44 | 3 | 5.924099 | -1.864078 | 80 | 1.445134 | 14.6 | 27.754866 | 0.182500 |  |
| GO:0043062\_extracellular\_structure\_organization | 125 | 5 | 3.475472 | -1.860682 | 81 | 1.470298 | 14.65 | 27.829702 | 0.180864 |  |
| GO:0032956\_regulation\_of\_actin\_cytoskeleton\_organization | 16 | 2 | 10.860849 | -1.851754 | 84 | 1.703959 | 15.27 | 28.836041 | 0.181786 |  |
| GO:0042311\_vasodilation | 16 | 2 | 10.860849 | -1.851754 | 84 | 1.703959 | 15.27 | 28.836041 | 0.181786 |  |
| GO:0055007\_cardiac\_muscle\_cell\_differentiation | 16 | 2 | 10.860849 | -1.851754 | 84 | 1.703959 | 15.27 | 28.836041 | 0.181786 |  |
| GO:0030198\_extracellular\_matrix\_organization | 83 | 4 | 4.187315 | -1.832518 | 85 | 1.785782 | 15.61 | 29.434218 | 0.183647 |  |
| GO:0043066\_negative\_regulation\_of\_apoptosis | 176 | 6 | 2.962050 | -1.829827 | 86 | 1.778020 | 15.65 | 29.521980 | 0.181977 |  |
| GO:0032970\_regulation\_of\_actin\_filament-based\_process | 17 | 2 | 10.221976 | -1.800581 | 89 | 2.380421 | 16.9 | 31.419579 | 0.189888 |  |
| GO:0035115\_embryonic\_forelimb\_morphogenesis | 17 | 2 | 10.221976 | -1.800581 | 89 | 2.380421 | 16.9 | 31.419579 | 0.189888 |  |
| GO:0045667\_regulation\_of\_osteoblast\_differentiation | 17 | 2 | 10.221976 | -1.800581 | 89 | 2.380421 | 16.9 | 31.419579 | 0.189888 |  |
| GO:0043069\_negative\_regulation\_of\_programmed\_cell\_death | 179 | 6 | 2.912406 | -1.796545 | 91 | 2.364651 | 16.94 | 31.515349 | 0.186154 |  |
| GO:0060548\_negative\_regulation\_of\_cell\_death | 179 | 6 | 2.912406 | -1.796545 | 91 | 2.364651 | 16.94 | 31.515349 | 0.186154 |  |
| GO:0016477\_cell\_migration | 234 | 7 | 2.599178 | -1.777051 | 92 | 2.589524 | 17.42 | 32.250476 | 0.189348 |  |
| GO:0016337\_cell-cell\_adhesion | 87 | 4 | 3.994795 | -1.763874 | 93 | 2.642766 | 17.61 | 32.577234 | 0.189355 |  |
| GO:0009952\_anterior\_posterior\_pattern\_formation | 133 | 5 | 3.266421 | -1.753778 | 94 | 2.647868 | 17.78 | 32.912132 | 0.189149 |  |
| GO:0001974\_blood\_vessel\_remodeling | 18 | 2 | 9.654088 | -1.752610 | 99 | 3.160115 | 18.73 | 34.299885 | 0.189192 |  |
| GO:0035051\_cardiac\_cell\_differentiation | 18 | 2 | 9.654088 | -1.752610 | 99 | 3.160115 | 18.73 | 34.299885 | 0.189192 |  |
| GO:0046620\_regulation\_of\_organ\_growth | 18 | 2 | 9.654088 | -1.752610 | 99 | 3.160115 | 18.73 | 34.299885 | 0.189192 |  |
| GO:0055008\_cardiac\_muscle\_tissue\_morphogenesis | 18 | 2 | 9.654088 | -1.752610 | 99 | 3.160115 | 18.73 | 34.299885 | 0.189192 |  |
| GO:0060415\_muscle\_tissue\_morphogenesis | 18 | 2 | 9.654088 | -1.752610 | 99 | 3.160115 | 18.73 | 34.299885 | 0.189192 |  |
| GO:0046660\_female\_sex\_differentiation | 49 | 3 | 5.319600 | -1.738170 | 100 | 3.278486 | 19.03 | 34.781514 | 0.190300 |  |
| GO:0030324\_lung\_development | 90 | 4 | 3.861635 | -1.714898 | 101 | 3.327603 | 19.25 | 35.172397 | 0.190594 |  |
| GO:0009584\_detection\_of\_visible\_light | 19 | 2 | 9.145978 | -1.707485 | 106 | 3.971149 | 20.11 | 36.248851 | 0.189717 |  |
| GO:0033002\_muscle\_cell\_proliferation | 19 | 2 | 9.145978 | -1.707485 | 106 | 3.971149 | 20.11 | 36.248851 | 0.189717 |  |
| GO:0048547\_gut\_morphogenesis | 19 | 2 | 9.145978 | -1.707485 | 106 | 3.971149 | 20.11 | 36.248851 | 0.189717 |  |
| GO:0050908\_detection\_of\_light\_stimulus\_involved\_in\_visual\_perception | 19 | 2 | 9.145978 | -1.707485 | 106 | 3.971149 | 20.11 | 36.248851 | 0.189717 |  |
| GO:0050962\_detection\_of\_light\_stimulus\_involved\_in\_sensory\_perception | 19 | 2 | 9.145978 | -1.707485 | 106 | 3.971149 | 20.11 | 36.248851 | 0.189717 |  |
| GO:0030323\_respiratory\_tube\_development | 92 | 4 | 3.777687 | -1.683361 | 107 | 3.977749 | 20.52 | 37.062251 | 0.191776 |  |
| GO:0035107\_appendage\_morphogenesis | 93 | 4 | 3.737066 | -1.667912 | 109 | 3.941539 | 20.72 | 37.498461 | 0.190092 |  |
| GO:0035108\_limb\_morphogenesis | 93 | 4 | 3.737066 | -1.667912 | 109 | 3.941539 | 20.72 | 37.498461 | 0.190092 |  |
| GO:0018209\_peptidyl-serine\_modification | 20 | 2 | 8.688679 | -1.664904 | 110 | 4.169291 | 21.23 | 38.290709 | 0.193000 |  |
| GO:0045893\_positive\_regulation\_of\_transcription\_\_DNA-dependent | 306 | 8 | 2.271550 | -1.651336 | 112 | 4.122926 | 21.46 | 38.797074 | 0.191607 |  |
| GO:0051254\_positive\_regulation\_of\_RNA\_metabolic\_process | 306 | 8 | 2.271550 | -1.651336 | 112 | 4.122926 | 21.46 | 38.797074 | 0.191607 |  |
| GO:0001743\_optic\_placode\_formation | 2 | 1 |  |  |  |  |  |  |  |  |
| GO:0002576\_platelet\_degranulation | 2 | 1 |  |  |  |  |  |  |  |  |
| GO:0010872\_regulation\_of\_cholesterol\_esterification | 2 | 1 |  |  |  |  |  |  |  |  |
| GO:0014829\_vascular\_smooth\_muscle\_contraction | 2 | 1 |  |  |  |  |  |  |  |  |
| GO:0014866\_skeletal\_myofibril\_assembly | 2 | 1 |  |  |  |  |  |  |  |  |
| GO:0030240\_muscle\_thin\_filament\_assembly | 2 | 1 |  |  |  |  |  |  |  |  |
| GO:0031952\_regulation\_of\_protein\_amino\_acid\_autophosphorylation | 2 | 1 |  |  |  |  |  |  |  |  |
| GO:0031953\_negative\_regulation\_of\_protein\_amino\_acid\_autophosphorylation | 2 | 1 |  |  |  |  |  |  |  |  |
| GO:0033622\_integrin\_activation | 2 | 1 |  |  |  |  |  |  |  |  |
| GO:0033623\_regulation\_of\_integrin\_activation | 2 | 1 |  |  |  |  |  |  |  |  |
| GO:0033625\_positive\_regulation\_of\_integrin\_activation | 2 | 1 |  |  |  |  |  |  |  |  |
| GO:0034370\_triglyceride-rich\_lipoprotein\_particle\_remodeling | 2 | 1 |  |  |  |  |  |  |  |  |
| GO:0034374\_low-density\_lipoprotein\_particle\_remodeling | 2 | 1 |  |  |  |  |  |  |  |  |
| GO:0034433\_steroid\_esterification | 2 | 1 |  |  |  |  |  |  |  |  |
| GO:0034434\_sterol\_esterification | 2 | 1 |  |  |  |  |  |  |  |  |
| GO:0034435\_cholesterol\_esterification | 2 | 1 |  |  |  |  |  |  |  |  |
| GO:0035117\_embryonic\_arm\_morphogenesis | 2 | 1 |  |  |  |  |  |  |  |  |
| GO:0035140\_arm\_morphogenesis | 2 | 1 |  |  |  |  |  |  |  |  |
| GO:0042312\_regulation\_of\_vasodilation | 2 | 1 |  |  |  |  |  |  |  |  |
| GO:0045608\_negative\_regulation\_of\_auditory\_receptor\_cell\_differentiation | 2 | 1 |  |  |  |  |  |  |  |  |
| GO:0045632\_negative\_regulation\_of\_mechanoreceptor\_differentiation | 2 | 1 |  |  |  |  |  |  |  |  |
| GO:0046619\_optic\_placode\_formation\_involved\_in\_camera-type\_eye | 2 | 1 |  |  |  |  |  |  |  |  |
| GO:0048134\_germ-line\_cyst\_formation | 2 | 1 |  |  |  |  |  |  |  |  |
| GO:0048136\_male\_germ-line\_cyst\_formation | 2 | 1 |  |  |  |  |  |  |  |  |
| GO:0048711\_positive\_regulation\_of\_astrocyte\_differentiation | 2 | 1 |  |  |  |  |  |  |  |  |
| GO:0048807\_female\_genitalia\_morphogenesis | 2 | 1 |  |  |  |  |  |  |  |  |
| GO:0048850\_hypophysis\_morphogenesis | 2 | 1 |  |  |  |  |  |  |  |  |
| GO:0060044\_negative\_regulation\_of\_cardiac\_muscle\_cell\_proliferation | 2 | 1 |  |  |  |  |  |  |  |  |
| GO:0060214\_endocardium\_formation | 2 | 1 |  |  |  |  |  |  |  |  |
| GO:0060599\_lateral\_sprouting\_involved\_in\_mammary\_gland\_duct\_morphogenesis | 2 | 1 |  |  |  |  |  |  |  |  |
| GO:0060769\_positive\_regulation\_of\_epithelial\_cell\_proliferation\_involved\_in\_prostate\_gland\_development | 2 | 1 |  |  |  |  |  |  |  |  |
| GO:0007389\_pattern\_specification\_process | 250 | 7 | 2.432830 | -1.634697 | 113 | 4.184877 | 21.82 | 39.455123 | 0.193097 |  |
| GO:0016310\_phosphorylation | 309 | 8 | 2.249496 | -1.628591 | 114 | 4.250866 | 21.94 | 39.629134 | 0.192456 |  |
| GO:0010604\_positive\_regulation\_of\_macromolecule\_metabolic\_process | 433 | 10 | 2.006623 | -1.624662 | 115 | 4.347283 | 22.21 | 40.072717 | 0.193130 |  |
| GO:0001709\_cell\_fate\_determination | 21 | 2 | 8.274933 | -1.624612 | 120 | 4.869297 | 23.14 | 41.410703 | 0.192833 |  |
| GO:0001755\_neural\_crest\_cell\_migration | 21 | 2 | 8.274933 | -1.624612 | 120 | 4.869297 | 23.14 | 41.410703 | 0.192833 |  |
| GO:0009583\_detection\_of\_light\_stimulus | 21 | 2 | 8.274933 | -1.624612 | 120 | 4.869297 | 23.14 | 41.410703 | 0.192833 |  |
| GO:0010552\_positive\_regulation\_of\_specific\_transcription\_from\_RNA\_polymerase\_II\_promoter | 21 | 2 | 8.274933 | -1.624612 | 120 | 4.869297 | 23.14 | 41.410703 | 0.192833 |  |
| GO:0035136\_forelimb\_morphogenesis | 21 | 2 | 8.274933 | -1.624612 | 120 | 4.869297 | 23.14 | 41.410703 | 0.192833 |  |
| GO:0007165\_signal\_transduction | 915 | 17 | 1.614290 | -1.624086 | 121 | 4.874679 | 23.15 | 41.425321 | 0.191322 |  |
| GO:0048736\_appendage\_development | 96 | 4 | 3.620283 | -1.622786 | 123 | 4.822213 | 23.25 | 41.677787 | 0.189024 |  |
| GO:0060173\_limb\_development | 96 | 4 | 3.620283 | -1.622786 | 123 | 4.822213 | 23.25 | 41.677787 | 0.189024 |  |
| GO:0033554\_cellular\_response\_to\_stress | 196 | 6 | 2.659800 | -1.621412 | 124 | 4.847980 | 23.27 | 41.692020 | 0.187661 |  |
| GO:0050793\_regulation\_of\_developmental\_process | 703 | 14 | 1.730320 | -1.612408 | 125 | 4.845185 | 23.36 | 41.874815 | 0.186880 |  |
| GO:0002009\_morphogenesis\_of\_an\_epithelium | 198 | 6 | 2.632933 | -1.602197 | 127 | 5.033414 | 23.75 | 42.466586 | 0.187008 |  |
| GO:0060429\_epithelium\_development | 198 | 6 | 2.632933 | -1.602197 | 127 | 5.033414 | 23.75 | 42.466586 | 0.187008 |  |
| GO:0030900\_forebrain\_development | 146 | 5 | 2.975575 | -1.596877 | 128 | 5.092362 | 23.88 | 42.667638 | 0.186562 |  |
| GO:0009967\_positive\_regulation\_of\_signal\_transduction | 98 | 4 | 3.546400 | -1.593678 | 130 | 5.190334 | 23.97 | 42.749666 | 0.184385 |  |
| GO:0060541\_respiratory\_system\_development | 98 | 4 | 3.546400 | -1.593678 | 130 | 5.190334 | 23.97 | 42.749666 | 0.184385 |  |
| GO:0006029\_proteoglycan\_metabolic\_process | 22 | 2 | 7.898799 | -1.586391 | 134 | 5.776007 | 25.03 | 44.283993 | 0.186791 |  |
| GO:0015918\_sterol\_transport | 22 | 2 | 7.898799 | -1.586391 | 134 | 5.776007 | 25.03 | 44.283993 | 0.186791 |  |
| GO:0021766\_hippocampus\_development | 22 | 2 | 7.898799 | -1.586391 | 134 | 5.776007 | 25.03 | 44.283993 | 0.186791 |  |
| GO:0030301\_cholesterol\_transport | 22 | 2 | 7.898799 | -1.586391 | 134 | 5.776007 | 25.03 | 44.283993 | 0.186791 |  |
| GO:0080090\_regulation\_of\_primary\_metabolic\_process | 926 | 17 | 1.595114 | -1.576754 | 135 | 5.835413 | 25.62 | 45.404587 | 0.189778 |  |
| GO:0048870\_cell\_motility | 257 | 7 | 2.366566 | -1.576519 | 136 | 5.844455 | 25.63 | 45.415545 | 0.188456 |  |
| GO:0010926\_anatomical\_structure\_formation | 447 | 10 | 1.943776 | -1.539128 | 137 | 6.633579 | 26.91 | 47.186421 | 0.196423 |  |
| GO:0043687\_post-translational\_protein\_modification | 384 | 9 | 2.036409 | -1.533559 | 138 | 6.625024 | 27.11 | 47.594976 | 0.196449 |  |
| GO:0035270\_endocrine\_system\_development | 59 | 3 | 4.417972 | -1.526345 | 140 | 6.658150 | 27.31 | 47.961850 | 0.195071 |  |
| GO:0048469\_cell\_maturation | 59 | 3 | 4.417972 | -1.526345 | 140 | 6.658150 | 27.31 | 47.961850 | 0.195071 |  |
| GO:0003013\_circulatory\_system\_process | 103 | 4 | 3.374244 | -1.524090 | 142 | 6.577623 | 27.44 | 48.302377 | 0.193239 |  |
| GO:0008015\_blood\_circulation | 103 | 4 | 3.374244 | -1.524090 | 142 | 6.577623 | 27.44 | 48.302377 | 0.193239 |  |
| GO:0065007\_biological\_regulation | 2593 | 37 | 1.239804 | -1.518326 | 143 | 6.624582 | 27.65 | 48.675418 | 0.193357 |  |
| GO:0031323\_regulation\_of\_cellular\_metabolic\_process | 1015 | 18 | 1.540850 | -1.516122 | 144 | 6.640903 | 27.66 | 48.679097 | 0.192083 |  |
| GO:0007266\_Rho\_protein\_signal\_transduction | 24 | 2 | 7.240566 | -1.515429 | 146 | 7.231116 | 28.63 | 50.028884 | 0.196096 |  |
| GO:0048546\_digestive\_tract\_morphogenesis | 24 | 2 | 7.240566 | -1.515429 | 146 | 7.231116 | 28.63 | 50.028884 | 0.196096 |  |
| GO:0031328\_positive\_regulation\_of\_cellular\_biosynthetic\_process | 387 | 9 | 2.020623 | -1.514401 | 147 | 7.231116 | 28.63 | 50.028884 | 0.194762 |  |
| GO:0009891\_positive\_regulation\_of\_biosynthetic\_process | 388 | 9 | 2.015415 | -1.508069 | 148 | 7.238727 | 28.75 | 50.261273 | 0.194257 |  |
| GO:0008285\_negative\_regulation\_of\_cell\_proliferation | 155 | 5 | 2.802800 | -1.498788 | 149 | 7.321051 | 28.91 | 50.498949 | 0.194027 |  |
| GO:0010811\_positive\_regulation\_of\_cell-substrate\_adhesion | 25 | 2 | 6.950943 | -1.482381 | 151 | 7.678096 | 29.96 | 52.241904 | 0.198411 |  |
| GO:0055123\_digestive\_system\_development | 25 | 2 | 6.950943 | -1.482381 | 151 | 7.678096 | 29.96 | 52.241904 | 0.198411 |  |
| GO:0006928\_cell\_motion | 330 | 8 | 2.106346 | -1.478400 | 153 | 7.691364 | 30.09 | 52.488636 | 0.196667 |  |
| GO:0051674\_localization\_of\_cell | 330 | 8 | 2.106346 | -1.478400 | 153 | 7.691364 | 30.09 | 52.488636 | 0.196667 |  |
| GO:0001711\_endodermal\_cell\_fate\_commitment | 3 | 1 |  |  |  |  |  |  |  |  |
| GO:0001757\_somite\_specification | 3 | 1 |  |  |  |  |  |  |  |  |
| GO:0006266\_DNA\_ligation | 3 | 1 |  |  |  |  |  |  |  |  |
| GO:0006904\_vesicle\_docking\_during\_exocytosis | 3 | 1 |  |  |  |  |  |  |  |  |
| GO:0007403\_glial\_cell\_fate\_determination | 3 | 1 |  |  |  |  |  |  |  |  |
| GO:0015988\_energy\_coupled\_proton\_transport\_\_against\_electrochemical\_gradient | 3 | 1 |  |  |  |  |  |  |  |  |
| GO:0015991\_ATP\_hydrolysis\_coupled\_proton\_transport | 3 | 1 |  |  |  |  |  |  |  |  |
| GO:0021819\_layer\_formation\_in\_the\_cerebral\_cortex | 3 | 1 |  |  |  |  |  |  |  |  |
| GO:0022406\_membrane\_docking | 3 | 1 |  |  |  |  |  |  |  |  |
| GO:0030540\_female\_genitalia\_development | 3 | 1 |  |  |  |  |  |  |  |  |
| GO:0030836\_positive\_regulation\_of\_actin\_filament\_depolymerization | 3 | 1 |  |  |  |  |  |  |  |  |
| GO:0043243\_positive\_regulation\_of\_protein\_complex\_disassembly | 3 | 1 |  |  |  |  |  |  |  |  |
| GO:0043249\_erythrocyte\_maturation | 3 | 1 |  |  |  |  |  |  |  |  |
| GO:0045607\_regulation\_of\_auditory\_receptor\_cell\_differentiation | 3 | 1 |  |  |  |  |  |  |  |  |
| GO:0045631\_regulation\_of\_mechanoreceptor\_differentiation | 3 | 1 |  |  |  |  |  |  |  |  |
| GO:0048278\_vesicle\_docking | 3 | 1 |  |  |  |  |  |  |  |  |
| GO:0048845\_venous\_blood\_vessel\_morphogenesis | 3 | 1 |  |  |  |  |  |  |  |  |
| GO:0048852\_diencephalon\_morphogenesis | 3 | 1 |  |  |  |  |  |  |  |  |
| GO:0051153\_regulation\_of\_striated\_muscle\_cell\_differentiation | 3 | 1 |  |  |  |  |  |  |  |  |
| GO:0060686\_negative\_regulation\_of\_prostatic\_bud\_formation | 3 | 1 |  |  |  |  |  |  |  |  |
| GO:0060750\_epithelial\_cell\_proliferation\_involved\_in\_mammary\_gland\_duct\_elongation | 3 | 1 |  |  |  |  |  |  |  |  |
| GO:0060841\_venous\_blood\_vessel\_development | 3 | 1 |  |  |  |  |  |  |  |  |
| GO:0070307\_lens\_fiber\_cell\_development | 3 | 1 |  |  |  |  |  |  |  |  |
| GO:0070309\_lens\_fiber\_cell\_morphogenesis | 3 | 1 |  |  |  |  |  |  |  |  |
| GO:0051216\_cartilage\_development | 63 | 3 | 4.137466 | -1.453311 | 154 | 8.114642 | 31.08 | 54.045358 | 0.201818 |  |
| GO:0007166\_cell\_surface\_receptor\_linked\_signal\_transduction | 597 | 12 | 1.746468 | -1.451696 | 155 | 8.081235 | 31.13 | 54.178765 | 0.200839 |  |
| GO:0010647\_positive\_regulation\_of\_cell\_communication | 110 | 4 | 3.159520 | -1.433628 | 157 | 8.710226 | 32.38 | 56.049774 | 0.206242 |  |
| GO:0043010\_camera-type\_eye\_development | 110 | 4 | 3.159520 | -1.433628 | 157 | 8.710226 | 32.38 | 56.049774 | 0.206242 |  |
| GO:0045941\_positive\_regulation\_of\_transcription | 338 | 8 | 2.056492 | -1.425066 | 158 | 8.741747 | 32.48 | 56.218253 | 0.205570 |  |
| GO:0010638\_positive\_regulation\_of\_organelle\_organization | 27 | 2 | 6.436059 | -1.420515 | 160 | 9.286117 | 33.37 | 57.453883 | 0.208562 |  |
| GO:0031016\_pancreas\_development | 27 | 2 | 6.436059 | -1.420515 | 160 | 9.286117 | 33.37 | 57.453883 | 0.208562 |  |
| GO:0007423\_sensory\_organ\_development | 219 | 6 | 2.380460 | -1.415830 | 161 | 9.344521 | 33.57 | 57.795479 | 0.208509 |  |
| GO:0006793\_phosphorus\_metabolic\_process | 340 | 8 | 2.044395 | -1.412046 | 163 | 9.444545 | 33.66 | 57.875455 | 0.206503 |  |
| GO:0006796\_phosphate\_metabolic\_process | 340 | 8 | 2.044395 | -1.412046 | 163 | 9.444545 | 33.66 | 57.875455 | 0.206503 |  |
| GO:0042325\_regulation\_of\_phosphorylation | 164 | 5 | 2.648988 | -1.408174 | 164 | 9.489983 | 33.79 | 58.090017 | 0.206037 |  |
| GO:0031589\_cell-substrate\_adhesion | 66 | 3 | 3.949400 | -1.402123 | 165 | 9.566115 | 34.2 | 58.833885 | 0.207273 |  |
| GO:0019220\_regulation\_of\_phosphate\_metabolic\_process | 165 | 5 | 2.632933 | -1.398530 | 167 | 9.622109 | 34.34 | 59.057891 | 0.205629 |  |
| GO:0051174\_regulation\_of\_phosphorus\_metabolic\_process | 165 | 5 | 2.632933 | -1.398530 | 167 | 9.622109 | 34.34 | 59.057891 | 0.205629 |  |
| GO:0043193\_positive\_regulation\_of\_gene-specific\_transcription | 28 | 2 | 6.206199 | -1.391486 | 168 | 9.939330 | 34.97 | 60.000670 | 0.208155 |  |
| GO:0003007\_heart\_morphogenesis | 67 | 3 | 3.890453 | -1.385688 | 169 | 10.019960 | 35.22 | 60.420040 | 0.208402 |  |
| GO:0000165\_MAPKKK\_cascade | 114 | 4 | 3.048659 | -1.385225 | 170 | 10.047062 | 35.31 | 60.572938 | 0.207706 |  |
| GO:0010628\_positive\_regulation\_of\_gene\_expression | 346 | 8 | 2.008943 | -1.373714 | 171 | 10.137962 | 35.64 | 61.142038 | 0.208421 |  |
| GO:0021761\_limbic\_system\_development | 29 | 2 | 5.992193 | -1.363605 | 174 | 10.789924 | 36.81 | 62.830076 | 0.211552 |  |
| GO:0050769\_positive\_regulation\_of\_neurogenesis | 29 | 2 | 5.992193 | -1.363605 | 174 | 10.789924 | 36.81 | 62.830076 | 0.211552 |  |
| GO:0051301\_cell\_division | 29 | 2 | 5.992193 | -1.363605 | 174 | 10.789924 | 36.81 | 62.830076 | 0.211552 |  |
| GO:0048608\_reproductive\_structure\_development | 116 | 4 | 2.996096 | -1.361851 | 175 | 10.819600 | 36.91 | 63.000400 | 0.210914 |  |
| GO:0044260\_cellular\_macromolecule\_metabolic\_process | 1447 | 23 | 1.381062 | -1.361378 | 176 | 10.817326 | 36.95 | 63.082674 | 0.209943 |  |
| GO:0001932\_regulation\_of\_protein\_amino\_acid\_phosphorylation | 69 | 3 | 3.777687 | -1.353700 | 177 | 10.880082 | 37.15 | 63.419918 | 0.209887 |  |
| GO:0006707\_cholesterol\_catabolic\_process | 4 | 1 |  |  |  |  |  |  |  |  |
| GO:0006906\_vesicle\_fusion | 4 | 1 |  |  |  |  |  |  |  |  |
| GO:0007386\_compartment\_specification | 4 | 1 |  |  |  |  |  |  |  |  |
| GO:0016127\_sterol\_catabolic\_process | 4 | 1 |  |  |  |  |  |  |  |  |
| GO:0021801\_cerebral\_cortex\_radial\_glia\_guided\_migration | 4 | 1 |  |  |  |  |  |  |  |  |
| GO:0030858\_positive\_regulation\_of\_epithelial\_cell\_differentiation | 4 | 1 |  |  |  |  |  |  |  |  |
| GO:0032808\_lacrimal\_gland\_development | 4 | 1 |  |  |  |  |  |  |  |  |
| GO:0045940\_positive\_regulation\_of\_steroid\_metabolic\_process | 4 | 1 |  |  |  |  |  |  |  |  |
| GO:0048240\_sperm\_capacitation | 4 | 1 |  |  |  |  |  |  |  |  |
| GO:0048341\_paraxial\_mesoderm\_formation | 4 | 1 |  |  |  |  |  |  |  |  |
| GO:0048639\_positive\_regulation\_of\_developmental\_growth | 4 | 1 |  |  |  |  |  |  |  |  |
| GO:0048710\_regulation\_of\_astrocyte\_differentiation | 4 | 1 |  |  |  |  |  |  |  |  |
| GO:0050919\_negative\_chemotaxis | 4 | 1 |  |  |  |  |  |  |  |  |
| GO:0051024\_positive\_regulation\_of\_immunoglobulin\_secretion | 4 | 1 |  |  |  |  |  |  |  |  |
| GO:0055009\_atrial\_cardiac\_muscle\_morphogenesis | 4 | 1 |  |  |  |  |  |  |  |  |
| GO:0060065\_uterus\_development | 4 | 1 |  |  |  |  |  |  |  |  |
| GO:0060412\_ventricular\_septum\_morphogenesis | 4 | 1 |  |  |  |  |  |  |  |  |
| GO:0060751\_mammary\_gland\_duct\_branch\_elongation | 4 | 1 |  |  |  |  |  |  |  |  |
| GO:0060900\_embryonic\_camera-type\_eye\_formation | 4 | 1 |  |  |  |  |  |  |  |  |
| GO:0008406\_gonad\_development | 70 | 3 | 3.723720 | -1.338131 | 179 | 11.059053 | 37.67 | 64.280947 | 0.210447 |  |
| GO:0048592\_eye\_morphogenesis | 70 | 3 | 3.723720 | -1.338131 | 179 | 11.059053 | 37.67 | 64.280947 | 0.210447 |  |
| GO:0035265\_organ\_growth | 30 | 2 | 5.792453 | -1.336792 | 183 | 11.985441 | 38.85 | 65.714559 | 0.212295 |  |
| GO:0048565\_gut\_development | 30 | 2 | 5.792453 | -1.336792 | 183 | 11.985441 | 38.85 | 65.714559 | 0.212295 |  |
| GO:0051146\_striated\_muscle\_cell\_differentiation | 30 | 2 | 5.792453 | -1.336792 | 183 | 11.985441 | 38.85 | 65.714559 | 0.212295 |  |
| GO:0060021\_palate\_development | 30 | 2 | 5.792453 | -1.336792 | 183 | 11.985441 | 38.85 | 65.714559 | 0.212295 |  |
| GO:0045935\_positive\_regulation\_of\_nucleobase\_\_nucleoside\_\_nucleotide\_and\_nucleic\_acid\_metabolic\_process | 352 | 8 | 1.974700 | -1.336446 | 184 | 12.047428 | 38.9 | 65.752572 | 0.211413 |  |
| GO:0016331\_morphogenesis\_of\_embryonic\_epithelium | 71 | 3 | 3.671273 | -1.322834 | 186 | 12.237535 | 39.49 | 66.742465 | 0.212312 |  |
| GO:0033674\_positive\_regulation\_of\_kinase\_activity | 71 | 3 | 3.671273 | -1.322834 | 186 | 12.237535 | 39.49 | 66.742465 | 0.212312 |  |
| GO:0009306\_protein\_secretion | 31 | 2 | 5.605600 | -1.310976 | 188 | 13.005600 | 41.17 | 69.334400 | 0.218989 |  |
| GO:0048562\_embryonic\_organ\_morphogenesis | 31 | 2 | 5.605600 | -1.310976 | 188 | 13.005600 | 41.17 | 69.334400 | 0.218989 |  |
| GO:0007264\_small\_GTPase\_mediated\_signal\_transduction | 72 | 3 | 3.620283 | -1.307802 | 190 | 13.252882 | 41.81 | 70.367118 | 0.220053 |  |
| GO:0051347\_positive\_regulation\_of\_transferase\_activity | 72 | 3 | 3.620283 | -1.307802 | 190 | 13.252882 | 41.81 | 70.367118 | 0.220053 |  |
| GO:0051726\_regulation\_of\_cell\_cycle | 121 | 4 | 2.872291 | -1.305677 | 191 | 13.375594 | 42.01 | 70.644406 | 0.219948 |  |
| GO:0040011\_locomotion | 295 | 7 | 2.061720 | -1.297878 | 192 | 13.528559 | 42.32 | 71.111441 | 0.220417 |  |
| GO:0060284\_regulation\_of\_cell\_development | 122 | 4 | 2.848747 | -1.294813 | 193 | 13.470885 | 42.66 | 71.849115 | 0.221036 |  |
| GO:0051493\_regulation\_of\_cytoskeleton\_organization | 32 | 2 | 5.430425 | -1.286093 | 194 | 14.189737 | 43.8 | 73.410263 | 0.225773 |  |
| GO:0051173\_positive\_regulation\_of\_nitrogen\_compound\_metabolic\_process | 361 | 8 | 1.925469 | -1.282456 | 195 | 14.277797 | 43.92 | 73.562203 | 0.225231 |  |
| GO:0006468\_protein\_amino\_acid\_phosphorylation | 237 | 6 | 2.199666 | -1.275717 | 196 | 14.358395 | 44.27 | 74.181605 | 0.225867 |  |
| GO:0009308\_amine\_metabolic\_process | 124 | 4 | 2.802800 | -1.273437 | 197 | 14.357438 | 44.32 | 74.282562 | 0.224975 |  |
| GO:0034960\_cellular\_biopolymer\_metabolic\_process | 1395 | 22 | 1.370258 | -1.271074 | 198 | 14.369811 | 44.5 | 74.630189 | 0.224747 |  |
| GO:0001763\_morphogenesis\_of\_a\_branching\_structure | 125 | 4 | 2.780377 | -1.262922 | 199 | 14.553044 | 45.01 | 75.466956 | 0.226181 |  |
| GO:0008584\_male\_gonad\_development | 33 | 2 | 5.265866 | -1.262083 | 200 | 15.046516 | 45.65 | 76.253484 | 0.228250 |  |
| GO:0043009\_chordate\_embryonic\_development | 365 | 8 | 1.904368 | -1.259171 | 201 | 15.104262 | 45.81 | 76.515738 | 0.227910 |  |
| GO:0000726\_non-recombinational\_repair | 5 | 1 | 17.377358 | -1.249796 | 233 | 27.272431 | 62.02 | 96.767569 | 0.266180 |  |
| GO:0001706\_endoderm\_formation | 5 | 1 | 17.377358 | -1.249796 | 233 | 27.272431 | 62.02 | 96.767569 | 0.266180 |  |
| GO:0002328\_pro-B\_cell\_differentiation | 5 | 1 | 17.377358 | -1.249796 | 233 | 27.272431 | 62.02 | 96.767569 | 0.266180 |  |
| GO:0006303\_double-strand\_break\_repair\_via\_nonhomologous\_end\_joining | 5 | 1 | 17.377358 | -1.249796 | 233 | 27.272431 | 62.02 | 96.767569 | 0.266180 |  |
| GO:0006924\_activation-induced\_cell\_death\_of\_T\_cells | 5 | 1 | 17.377358 | -1.249796 | 233 | 27.272431 | 62.02 | 96.767569 | 0.266180 |  |
| GO:0009950\_dorsal\_ventral\_axis\_specification | 5 | 1 | 17.377358 | -1.249796 | 233 | 27.272431 | 62.02 | 96.767569 | 0.266180 |  |
| GO:0010761\_fibroblast\_migration | 5 | 1 | 17.377358 | -1.249796 | 233 | 27.272431 | 62.02 | 96.767569 | 0.266180 |  |
| GO:0014015\_positive\_regulation\_of\_gliogenesis | 5 | 1 | 17.377358 | -1.249796 | 233 | 27.272431 | 62.02 | 96.767569 | 0.266180 |  |
| GO:0030042\_actin\_filament\_depolymerization | 5 | 1 | 17.377358 | -1.249796 | 233 | 27.272431 | 62.02 | 96.767569 | 0.266180 |  |
| GO:0030177\_positive\_regulation\_of\_Wnt\_receptor\_signaling\_pathway | 5 | 1 | 17.377358 | -1.249796 | 233 | 27.272431 | 62.02 | 96.767569 | 0.266180 |  |
| GO:0030834\_regulation\_of\_actin\_filament\_depolymerization | 5 | 1 | 17.377358 | -1.249796 | 233 | 27.272431 | 62.02 | 96.767569 | 0.266180 |  |
| GO:0033152\_immunoglobulin\_V(D)J\_recombination | 5 | 1 | 17.377358 | -1.249796 | 233 | 27.272431 | 62.02 | 96.767569 | 0.266180 |  |
| GO:0034109\_homotypic\_cell-cell\_adhesion | 5 | 1 | 17.377358 | -1.249796 | 233 | 27.272431 | 62.02 | 96.767569 | 0.266180 |  |
| GO:0034367\_macromolecular\_complex\_remodeling | 5 | 1 | 17.377358 | -1.249796 | 233 | 27.272431 | 62.02 | 96.767569 | 0.266180 |  |
| GO:0034368\_protein-lipid\_complex\_remodeling | 5 | 1 | 17.377358 | -1.249796 | 233 | 27.272431 | 62.02 | 96.767569 | 0.266180 |  |
| GO:0034369\_plasma\_lipoprotein\_particle\_remodeling | 5 | 1 | 17.377358 | -1.249796 | 233 | 27.272431 | 62.02 | 96.767569 | 0.266180 |  |
| GO:0042159\_lipoprotein\_catabolic\_process | 5 | 1 | 17.377358 | -1.249796 | 233 | 27.272431 | 62.02 | 96.767569 | 0.266180 |  |
| GO:0042416\_dopamine\_biosynthetic\_process | 5 | 1 | 17.377358 | -1.249796 | 233 | 27.272431 | 62.02 | 96.767569 | 0.266180 |  |
| GO:0042574\_retinal\_metabolic\_process | 5 | 1 | 17.377358 | -1.249796 | 233 | 27.272431 | 62.02 | 96.767569 | 0.266180 |  |
| GO:0042695\_thelarche | 5 | 1 | 17.377358 | -1.249796 | 233 | 27.272431 | 62.02 | 96.767569 | 0.266180 |  |
| GO:0043030\_regulation\_of\_macrophage\_activation | 5 | 1 | 17.377358 | -1.249796 | 233 | 27.272431 | 62.02 | 96.767569 | 0.266180 |  |
| GO:0043288\_apocarotenoid\_metabolic\_process | 5 | 1 | 17.377358 | -1.249796 | 233 | 27.272431 | 62.02 | 96.767569 | 0.266180 |  |
| GO:0043550\_regulation\_of\_lipid\_kinase\_activity | 5 | 1 | 17.377358 | -1.249796 | 233 | 27.272431 | 62.02 | 96.767569 | 0.266180 |  |
| GO:0043551\_regulation\_of\_phosphoinositide\_3-kinase\_activity | 5 | 1 | 17.377358 | -1.249796 | 233 | 27.272431 | 62.02 | 96.767569 | 0.266180 |  |
| GO:0043552\_positive\_regulation\_of\_phosphoinositide\_3-kinase\_activity | 5 | 1 | 17.377358 | -1.249796 | 233 | 27.272431 | 62.02 | 96.767569 | 0.266180 |  |
| GO:0045687\_positive\_regulation\_of\_glial\_cell\_differentiation | 5 | 1 | 17.377358 | -1.249796 | 233 | 27.272431 | 62.02 | 96.767569 | 0.266180 |  |
| GO:0045793\_positive\_regulation\_of\_cell\_size | 5 | 1 | 17.377358 | -1.249796 | 233 | 27.272431 | 62.02 | 96.767569 | 0.266180 |  |
| GO:0046697\_decidualization | 5 | 1 | 17.377358 | -1.249796 | 233 | 27.272431 | 62.02 | 96.767569 | 0.266180 |  |
| GO:0055003\_cardiac\_myofibril\_assembly | 5 | 1 | 17.377358 | -1.249796 | 233 | 27.272431 | 62.02 | 96.767569 | 0.266180 |  |
| GO:0055015\_ventricular\_cardiac\_muscle\_cell\_development | 5 | 1 | 17.377358 | -1.249796 | 233 | 27.272431 | 62.02 | 96.767569 | 0.266180 |  |
| GO:0060744\_mammary\_gland\_branching\_involved\_in\_thelarche | 5 | 1 | 17.377358 | -1.249796 | 233 | 27.272431 | 62.02 | 96.767569 | 0.266180 |  |
| GO:0060762\_regulation\_of\_branching\_involved\_in\_mammary\_gland\_duct\_morphogenesis | 5 | 1 | 17.377358 | -1.249796 | 233 | 27.272431 | 62.02 | 96.767569 | 0.266180 |  |
| GO:0019222\_regulation\_of\_metabolic\_process | 1088 | 18 | 1.437465 | -1.246316 | 234 | 27.265037 | 62.14 | 97.014963 | 0.265556 |  |
| GO:0060255\_regulation\_of\_macromolecule\_metabolic\_process | 936 | 16 | 1.485244 | -1.243974 | 235 | 27.232105 | 62.19 | 97.147895 | 0.264638 |  |
| GO:0009792\_embryonic\_development\_ending\_in\_birth\_or\_egg\_hatching | 368 | 8 | 1.888843 | -1.241986 | 236 | 27.306513 | 62.29 | 97.273487 | 0.263941 |  |
| GO:0010720\_positive\_regulation\_of\_cell\_development | 34 | 2 | 5.110988 | -1.238894 | 237 | 27.931277 | 63.27 | 98.608723 | 0.266962 |  |
| GO:0060537\_muscle\_tissue\_development | 128 | 4 | 2.715212 | -1.232048 | 238 | 28.138669 | 63.62 | 99.101331 | 0.267311 |  |
| GO:0006355\_regulation\_of\_transcription\_\_DNA-dependent | 575 | 11 | 1.662182 | -1.226881 | 239 | 28.218526 | 63.96 | 99.701474 | 0.267615 |  |
| GO:0010557\_positive\_regulation\_of\_macromolecule\_biosynthetic\_process | 371 | 8 | 1.873570 | -1.225034 | 240 | 28.224278 | 64.05 | 99.875722 | 0.266875 |  |
| GO:0007154\_cell\_communication | 1096 | 18 | 1.426973 | -1.219198 | 241 | 28.238810 | 64.48 | 100.721190 | 0.267552 |  |
| GO:0006464\_protein\_modification\_process | 439 | 9 | 1.781278 | -1.217541 | 242 | 28.228386 | 64.53 | 100.831614 | 0.266653 |  |
| GO:0010810\_regulation\_of\_cell-substrate\_adhesion | 35 | 2 | 4.964960 | -1.216477 | 243 | 28.834561 | 65.24 | 101.645439 | 0.268477 |  |
| GO:0045165\_cell\_fate\_commitment | 130 | 4 | 2.673440 | -1.212005 | 244 | 29.007718 | 65.69 | 102.372282 | 0.269221 |  |
| GO:0001704\_formation\_of\_primary\_germ\_layer | 36 | 2 | 4.827044 | -1.194788 | 248 | 29.943784 | 67.38 | 104.816216 | 0.271694 |  |
| GO:0001889\_liver\_development | 36 | 2 | 4.827044 | -1.194788 | 248 | 29.943784 | 67.38 | 104.816216 | 0.271694 |  |
| GO:0007368\_determination\_of\_left\_right\_symmetry | 36 | 2 | 4.827044 | -1.194788 | 248 | 29.943784 | 67.38 | 104.816216 | 0.271694 |  |
| GO:0030278\_regulation\_of\_ossification | 36 | 2 | 4.827044 | -1.194788 | 248 | 29.943784 | 67.38 | 104.816216 | 0.271694 |  |
| GO:0021700\_developmental\_maturation | 81 | 3 | 3.218029 | -1.183269 | 249 | 30.104579 | 67.77 | 105.435421 | 0.272169 |  |
| GO:0009799\_determination\_of\_symmetry | 37 | 2 | 4.696583 | -1.173786 | 252 | 30.854194 | 68.91 | 106.965806 | 0.273452 |  |
| GO:0009855\_determination\_of\_bilateral\_symmetry | 37 | 2 | 4.696583 | -1.173786 | 252 | 30.854194 | 68.91 | 106.965806 | 0.273452 |  |
| GO:0050906\_detection\_of\_stimulus\_involved\_in\_sensory\_perception | 37 | 2 | 4.696583 | -1.173786 | 252 | 30.854194 | 68.91 | 106.965806 | 0.273452 |  |
| GO:0006706\_steroid\_catabolic\_process | 6 | 1 | 14.481132 | -1.173057 | 268 | 40.686863 | 81.39 | 122.093137 | 0.303694 |  |
| GO:0007442\_hindgut\_morphogenesis | 6 | 1 | 14.481132 | -1.173057 | 268 | 40.686863 | 81.39 | 122.093137 | 0.303694 |  |
| GO:0022409\_positive\_regulation\_of\_cell-cell\_adhesion | 6 | 1 | 14.481132 | -1.173057 | 268 | 40.686863 | 81.39 | 122.093137 | 0.303694 |  |
| GO:0032331\_negative\_regulation\_of\_chondrocyte\_differentiation | 6 | 1 | 14.481132 | -1.173057 | 268 | 40.686863 | 81.39 | 122.093137 | 0.303694 |  |
| GO:0045778\_positive\_regulation\_of\_ossification | 6 | 1 | 14.481132 | -1.173057 | 268 | 40.686863 | 81.39 | 122.093137 | 0.303694 |  |
| GO:0046852\_positive\_regulation\_of\_bone\_remodeling | 6 | 1 | 14.481132 | -1.173057 | 268 | 40.686863 | 81.39 | 122.093137 | 0.303694 |  |
| GO:0048103\_somatic\_stem\_cell\_division | 6 | 1 | 14.481132 | -1.173057 | 268 | 40.686863 | 81.39 | 122.093137 | 0.303694 |  |
| GO:0048340\_paraxial\_mesoderm\_morphogenesis | 6 | 1 | 14.481132 | -1.173057 | 268 | 40.686863 | 81.39 | 122.093137 | 0.303694 |  |
| GO:0048617\_embryonic\_foregut\_morphogenesis | 6 | 1 | 14.481132 | -1.173057 | 268 | 40.686863 | 81.39 | 122.093137 | 0.303694 |  |
| GO:0048853\_forebrain\_morphogenesis | 6 | 1 | 14.481132 | -1.173057 | 268 | 40.686863 | 81.39 | 122.093137 | 0.303694 |  |
| GO:0051180\_vitamin\_transport | 6 | 1 | 14.481132 | -1.173057 | 268 | 40.686863 | 81.39 | 122.093137 | 0.303694 |  |
| GO:0060068\_vagina\_development | 6 | 1 | 14.481132 | -1.173057 | 268 | 40.686863 | 81.39 | 122.093137 | 0.303694 |  |
| GO:0060136\_embryonic\_process\_involved\_in\_female\_pregnancy | 6 | 1 | 14.481132 | -1.173057 | 268 | 40.686863 | 81.39 | 122.093137 | 0.303694 |  |
| GO:0060389\_pathway-restricted\_SMAD\_protein\_phosphorylation | 6 | 1 | 14.481132 | -1.173057 | 268 | 40.686863 | 81.39 | 122.093137 | 0.303694 |  |
| GO:0060411\_heart\_septum\_morphogenesis | 6 | 1 | 14.481132 | -1.173057 | 268 | 40.686863 | 81.39 | 122.093137 | 0.303694 |  |
| GO:0060685\_regulation\_of\_prostatic\_bud\_formation | 6 | 1 | 14.481132 | -1.173057 | 268 | 40.686863 | 81.39 | 122.093137 | 0.303694 |  |
| GO:0051252\_regulation\_of\_RNA\_metabolic\_process | 590 | 11 | 1.619923 | -1.160971 | 269 | 41.038027 | 82.11 | 123.181973 | 0.305242 |  |
| GO:0001654\_eye\_development | 136 | 4 | 2.555494 | -1.154323 | 270 | 41.271620 | 82.54 | 123.808380 | 0.305704 |  |
| GO:0001649\_osteoblast\_differentiation | 38 | 2 | 4.572989 | -1.153434 | 272 | 42.014923 | 83.86 | 125.705077 | 0.308309 |  |
| GO:0001657\_ureteric\_bud\_development | 38 | 2 | 4.572989 | -1.153434 | 272 | 42.014923 | 83.86 | 125.705077 | 0.308309 |  |
| GO:0045137\_development\_of\_primary\_sexual\_characteristics | 84 | 3 | 3.103100 | -1.145596 | 273 | 42.224978 | 84.14 | 126.055022 | 0.308205 |  |
| GO:0006351\_transcription\_\_DNA-dependent | 594 | 11 | 1.609015 | -1.143938 | 274 | 42.245603 | 84.28 | 126.314397 | 0.307591 |  |
| GO:0003002\_regionalization | 195 | 5 | 2.227866 | -1.142928 | 275 | 42.299934 | 84.39 | 126.480066 | 0.306873 |  |
| GO:0032774\_RNA\_biosynthetic\_process | 595 | 11 | 1.606310 | -1.139715 | 276 | 42.290858 | 84.41 | 126.529142 | 0.305833 |  |
| GO:0042475\_odontogenesis\_of\_dentine-containing\_tooth | 39 | 2 | 4.455733 | -1.133697 | 277 | 43.246637 | 85.66 | 128.073363 | 0.309242 |  |
| GO:0043412\_biopolymer\_modification | 458 | 9 | 1.707382 | -1.123791 | 278 | 43.418447 | 86.28 | 129.141553 | 0.310360 |  |
| GO:0008203\_cholesterol\_metabolic\_process | 40 | 2 | 4.344340 | -1.114545 | 280 | 44.244504 | 87.76 | 131.275496 | 0.313429 |  |
| GO:0046850\_regulation\_of\_bone\_remodeling | 40 | 2 | 4.344340 | -1.114545 | 280 | 44.244504 | 87.76 | 131.275496 | 0.313429 |  |
| GO:0001822\_kidney\_development | 87 | 3 | 2.996096 | -1.109611 | 282 | 44.640358 | 88.35 | 132.059642 | 0.313298 |  |
| GO:0007178\_transmembrane\_receptor\_protein\_serine\_threonine\_kinase\_signaling\_pathway | 87 | 3 | 2.996096 | -1.109611 | 282 | 44.640358 | 88.35 | 132.059642 | 0.313298 |  |
| GO:0003006\_reproductive\_developmental\_process | 141 | 4 | 2.464874 | -1.108880 | 283 | 44.778762 | 88.53 | 132.281238 | 0.312827 |  |
| GO:0006818\_hydrogen\_transport | 7 | 1 | 12.412399 | -1.108548 | 294 | 56.332883 | 102.76 | 149.187117 | 0.349524 |  |
| GO:0007440\_foregut\_morphogenesis | 7 | 1 | 12.412399 | -1.108548 | 294 | 56.332883 | 102.76 | 149.187117 | 0.349524 |  |
| GO:0015992\_proton\_transport | 7 | 1 | 12.412399 | -1.108548 | 294 | 56.332883 | 102.76 | 149.187117 | 0.349524 |  |
| GO:0022407\_regulation\_of\_cell-cell\_adhesion | 7 | 1 | 12.412399 | -1.108548 | 294 | 56.332883 | 102.76 | 149.187117 | 0.349524 |  |
| GO:0042572\_retinol\_metabolic\_process | 7 | 1 | 12.412399 | -1.108548 | 294 | 56.332883 | 102.76 | 149.187117 | 0.349524 |  |
| GO:0043507\_positive\_regulation\_of\_JUN\_kinase\_activity | 7 | 1 | 12.412399 | -1.108548 | 294 | 56.332883 | 102.76 | 149.187117 | 0.349524 |  |
| GO:0045880\_positive\_regulation\_of\_smoothened\_signaling\_pathway | 7 | 1 | 12.412399 | -1.108548 | 294 | 56.332883 | 102.76 | 149.187117 | 0.349524 |  |
| GO:0046543\_development\_of\_secondary\_female\_sexual\_characteristics | 7 | 1 | 12.412399 | -1.108548 | 294 | 56.332883 | 102.76 | 149.187117 | 0.349524 |  |
| GO:0046622\_positive\_regulation\_of\_organ\_growth | 7 | 1 | 12.412399 | -1.108548 | 294 | 56.332883 | 102.76 | 149.187117 | 0.349524 |  |
| GO:0060788\_ectodermal\_placode\_formation | 7 | 1 | 12.412399 | -1.108548 | 294 | 56.332883 | 102.76 | 149.187117 | 0.349524 |  |
| GO:0060795\_cell\_fate\_commitment\_involved\_in\_the\_formation\_of\_primary\_germ\_layers | 7 | 1 | 12.412399 | -1.108548 | 294 | 56.332883 | 102.76 | 149.187117 | 0.349524 |  |
| GO:0042127\_regulation\_of\_cell\_proliferation | 393 | 8 | 1.768688 | -1.107524 | 295 | 56.379930 | 102.81 | 149.240070 | 0.348508 |  |
| GO:0045449\_regulation\_of\_transcription | 676 | 12 | 1.542369 | -1.104571 | 296 | 56.423723 | 102.95 | 149.476277 | 0.347804 |  |
| GO:0001503\_ossification | 88 | 3 | 2.962050 | -1.097970 | 298 | 56.611158 | 103.27 | 149.928842 | 0.346544 |  |
| GO:0048754\_branching\_morphogenesis\_of\_a\_tube | 88 | 3 | 2.962050 | -1.097970 | 298 | 56.611158 | 103.27 | 149.928842 | 0.346544 |  |
| GO:0010551\_regulation\_of\_specific\_transcription\_from\_RNA\_polymerase\_II\_promoter | 41 | 2 | 4.238380 | -1.095946 | 302 | 57.380035 | 104.72 | 152.059965 | 0.346755 |  |
| GO:0019216\_regulation\_of\_lipid\_metabolic\_process | 41 | 2 | 4.238380 | -1.095946 | 302 | 57.380035 | 104.72 | 152.059965 | 0.346755 |  |
| GO:0032569\_specific\_transcription\_from\_RNA\_polymerase\_II\_promoter | 41 | 2 | 4.238380 | -1.095946 | 302 | 57.380035 | 104.72 | 152.059965 | 0.346755 |  |
| GO:0033077\_T\_cell\_differentiation\_in\_the\_thymus | 41 | 2 | 4.238380 | -1.095946 | 302 | 57.380035 | 104.72 | 152.059965 | 0.346755 |  |
| GO:0035239\_tube\_morphogenesis | 143 | 4 | 2.430400 | -1.091328 | 303 | 57.444574 | 104.86 | 152.275426 | 0.346073 |  |
| GO:0016125\_sterol\_metabolic\_process | 42 | 2 | 4.137466 | -1.077875 | 305 | 58.465726 | 106.8 | 155.134274 | 0.350164 |  |
| GO:0042476\_odontogenesis | 42 | 2 | 4.137466 | -1.077875 | 305 | 58.465726 | 106.8 | 155.134274 | 0.350164 |  |
| GO:0007243\_protein\_kinase\_cascade | 205 | 5 | 2.119190 | -1.070163 | 306 | 58.668568 | 107.38 | 156.091432 | 0.350915 |  |
| GO:0005975\_carbohydrate\_metabolic\_process | 146 | 4 | 2.380460 | -1.065640 | 307 | 58.810109 | 107.56 | 156.309891 | 0.350358 |  |
| GO:0031399\_regulation\_of\_protein\_modification\_process | 91 | 3 | 2.864400 | -1.064055 | 308 | 58.900664 | 107.76 | 156.619336 | 0.349870 |  |
| GO:0009582\_detection\_of\_abiotic\_stimulus | 43 | 2 | 4.041246 | -1.060306 | 310 | 59.836696 | 109.21 | 158.583304 | 0.352290 |  |
| GO:0010001\_glial\_cell\_differentiation | 43 | 2 | 4.041246 | -1.060306 | 310 | 59.836696 | 109.21 | 158.583304 | 0.352290 |  |
| GO:0022603\_regulation\_of\_anatomical\_structure\_morphogenesis | 147 | 4 | 2.364266 | -1.057243 | 311 | 59.869199 | 109.3 | 158.730801 | 0.351447 |  |
| GO:0006807\_nitrogen\_compound\_metabolic\_process | 1147 | 18 | 1.363524 | -1.056828 | 312 | 59.953474 | 109.37 | 158.786526 | 0.350545 |  |
| GO:0008283\_cell\_proliferation | 544 | 10 | 1.597184 | -1.053792 | 313 | 59.944582 | 109.4 | 158.855418 | 0.349521 |  |
| GO:0030217\_T\_cell\_differentiation | 92 | 3 | 2.833265 | -1.053074 | 314 | 60.040045 | 109.7 | 159.359955 | 0.349363 |  |
| GO:0001893\_maternal\_placenta\_development | 8 | 1 | 10.860849 | -1.052990 | 332 | 68.204786 | 120.78 | 173.355214 | 0.363795 |  |
| GO:0002320\_lymphoid\_progenitor\_cell\_differentiation | 8 | 1 | 10.860849 | -1.052990 | 332 | 68.204786 | 120.78 | 173.355214 | 0.363795 |  |
| GO:0021692\_cerebellar\_Purkinje\_cell\_layer\_morphogenesis | 8 | 1 | 10.860849 | -1.052990 | 332 | 68.204786 | 120.78 | 173.355214 | 0.363795 |  |
| GO:0021694\_cerebellar\_Purkinje\_cell\_layer\_formation | 8 | 1 | 10.860849 | -1.052990 | 332 | 68.204786 | 120.78 | 173.355214 | 0.363795 |  |
| GO:0021702\_cerebellar\_Purkinje\_cell\_differentiation | 8 | 1 | 10.860849 | -1.052990 | 332 | 68.204786 | 120.78 | 173.355214 | 0.363795 |  |
| GO:0021781\_glial\_cell\_fate\_commitment | 8 | 1 | 10.860849 | -1.052990 | 332 | 68.204786 | 120.78 | 173.355214 | 0.363795 |  |
| GO:0021799\_cerebral\_cortex\_radially\_oriented\_cell\_migration | 8 | 1 | 10.860849 | -1.052990 | 332 | 68.204786 | 120.78 | 173.355214 | 0.363795 |  |
| GO:0030193\_regulation\_of\_blood\_coagulation | 8 | 1 | 10.860849 | -1.052990 | 332 | 68.204786 | 120.78 | 173.355214 | 0.363795 |  |
| GO:0030511\_positive\_regulation\_of\_transforming\_growth\_factor\_beta\_receptor\_signaling\_pathway | 8 | 1 | 10.860849 | -1.052990 | 332 | 68.204786 | 120.78 | 173.355214 | 0.363795 |  |
| GO:0031102\_neuron\_projection\_regeneration | 8 | 1 | 10.860849 | -1.052990 | 332 | 68.204786 | 120.78 | 173.355214 | 0.363795 |  |
| GO:0031103\_axon\_regeneration | 8 | 1 | 10.860849 | -1.052990 | 332 | 68.204786 | 120.78 | 173.355214 | 0.363795 |  |
| GO:0035112\_genitalia\_morphogenesis | 8 | 1 | 10.860849 | -1.052990 | 332 | 68.204786 | 120.78 | 173.355214 | 0.363795 |  |
| GO:0042423\_catecholamine\_biosynthetic\_process | 8 | 1 | 10.860849 | -1.052990 | 332 | 68.204786 | 120.78 | 173.355214 | 0.363795 |  |
| GO:0048638\_regulation\_of\_developmental\_growth | 8 | 1 | 10.860849 | -1.052990 | 332 | 68.204786 | 120.78 | 173.355214 | 0.363795 |  |
| GO:0060043\_regulation\_of\_cardiac\_muscle\_cell\_proliferation | 8 | 1 | 10.860849 | -1.052990 | 332 | 68.204786 | 120.78 | 173.355214 | 0.363795 |  |
| GO:0060347\_heart\_trabecula\_formation | 8 | 1 | 10.860849 | -1.052990 | 332 | 68.204786 | 120.78 | 173.355214 | 0.363795 |  |
| GO:0060670\_branching\_involved\_in\_embryonic\_placenta\_morphogenesis | 8 | 1 | 10.860849 | -1.052990 | 332 | 68.204786 | 120.78 | 173.355214 | 0.363795 |  |
| GO:0070231\_T\_cell\_apoptosis | 8 | 1 | 10.860849 | -1.052990 | 332 | 68.204786 | 120.78 | 173.355214 | 0.363795 |  |
| GO:0002377\_immunoglobulin\_production | 44 | 2 | 3.949400 | -1.043216 | 334 | 69.364274 | 122.98 | 176.595726 | 0.368204 |  |
| GO:0046545\_development\_of\_primary\_female\_sexual\_characteristics | 44 | 2 | 3.949400 | -1.043216 | 334 | 69.364274 | 122.98 | 176.595726 | 0.368204 |  |
| GO:0051716\_cellular\_response\_to\_stimulus | 273 | 6 | 1.909600 | -1.039089 | 335 | 69.632506 | 123.42 | 177.207494 | 0.368418 |  |
| GO:0009605\_response\_to\_external\_stimulus | 339 | 7 | 1.794123 | -1.038820 | 336 | 69.616578 | 123.47 | 177.323422 | 0.367470 |  |
| GO:0044237\_cellular\_metabolic\_process | 1974 | 28 | 1.232437 | -1.038345 | 337 | 69.635193 | 123.51 | 177.384807 | 0.366499 |  |
| GO:0034103\_regulation\_of\_tissue\_remodeling | 45 | 2 | 3.861635 | -1.026582 | 339 | 70.377392 | 125.12 | 179.862608 | 0.369086 |  |
| GO:0046546\_development\_of\_primary\_male\_sexual\_characteristics | 45 | 2 | 3.861635 | -1.026582 | 339 | 70.377392 | 125.12 | 179.862608 | 0.369086 |  |
| GO:0007242\_intracellular\_signaling\_cascade | 411 | 8 | 1.691227 | -1.019624 | 340 | 70.594587 | 125.54 | 180.485413 | 0.369235 |  |
| GO:0032268\_regulation\_of\_cellular\_protein\_metabolic\_process | 152 | 4 | 2.286495 | -1.016447 | 341 | 70.641709 | 125.61 | 180.578291 | 0.368358 |  |
| GO:0006350\_transcription | 701 | 12 | 1.487363 | -1.010985 | 342 | 70.741982 | 125.78 | 180.818018 | 0.367778 |  |
| GO:0009581\_detection\_of\_external\_stimulus | 46 | 2 | 3.777687 | -1.010384 | 345 | 71.652969 | 127.12 | 182.587031 | 0.368464 |  |
| GO:0030850\_prostate\_gland\_development | 46 | 2 | 3.777687 | -1.010384 | 345 | 71.652969 | 127.12 | 182.587031 | 0.368464 |  |
| GO:0042063\_gliogenesis | 46 | 2 | 3.777687 | -1.010384 | 345 | 71.652969 | 127.12 | 182.587031 | 0.368464 |  |
| GO:0007517\_muscle\_organ\_development | 153 | 4 | 2.271550 | -1.008519 | 346 | 71.664235 | 127.17 | 182.675765 | 0.367543 |  |
| GO:0001667\_ameboidal\_cell\_migration | 9 | 1 | 9.654088 | -1.004267 | 368 | 80.394908 | 138.47 | 196.545092 | 0.376277 |  |
| GO:0002021\_response\_to\_dietary\_excess | 9 | 1 | 9.654088 | -1.004267 | 368 | 80.394908 | 138.47 | 196.545092 | 0.376277 |  |
| GO:0007379\_segment\_specification | 9 | 1 | 9.654088 | -1.004267 | 368 | 80.394908 | 138.47 | 196.545092 | 0.376277 |  |
| GO:0010165\_response\_to\_X-ray | 9 | 1 | 9.654088 | -1.004267 | 368 | 80.394908 | 138.47 | 196.545092 | 0.376277 |  |
| GO:0017145\_stem\_cell\_division | 9 | 1 | 9.654088 | -1.004267 | 368 | 80.394908 | 138.47 | 196.545092 | 0.376277 |  |
| GO:0030325\_adrenal\_gland\_development | 9 | 1 | 9.654088 | -1.004267 | 368 | 80.394908 | 138.47 | 196.545092 | 0.376277 |  |
| GO:0033151\_V(D)J\_recombination | 9 | 1 | 9.654088 | -1.004267 | 368 | 80.394908 | 138.47 | 196.545092 | 0.376277 |  |
| GO:0033344\_cholesterol\_efflux | 9 | 1 | 9.654088 | -1.004267 | 368 | 80.394908 | 138.47 | 196.545092 | 0.376277 |  |
| GO:0034605\_cellular\_response\_to\_heat | 9 | 1 | 9.654088 | -1.004267 | 368 | 80.394908 | 138.47 | 196.545092 | 0.376277 |  |
| GO:0045136\_development\_of\_secondary\_sexual\_characteristics | 9 | 1 | 9.654088 | -1.004267 | 368 | 80.394908 | 138.47 | 196.545092 | 0.376277 |  |
| GO:0045214\_sarcomere\_organization | 9 | 1 | 9.654088 | -1.004267 | 368 | 80.394908 | 138.47 | 196.545092 | 0.376277 |  |
| GO:0045671\_negative\_regulation\_of\_osteoclast\_differentiation | 9 | 1 | 9.654088 | -1.004267 | 368 | 80.394908 | 138.47 | 196.545092 | 0.376277 |  |
| GO:0048146\_positive\_regulation\_of\_fibroblast\_proliferation | 9 | 1 | 9.654088 | -1.004267 | 368 | 80.394908 | 138.47 | 196.545092 | 0.376277 |  |
| GO:0048284\_organelle\_fusion | 9 | 1 | 9.654088 | -1.004267 | 368 | 80.394908 | 138.47 | 196.545092 | 0.376277 |  |
| GO:0048708\_astrocyte\_differentiation | 9 | 1 | 9.654088 | -1.004267 | 368 | 80.394908 | 138.47 | 196.545092 | 0.376277 |  |
| GO:0051023\_regulation\_of\_immunoglobulin\_secretion | 9 | 1 | 9.654088 | -1.004267 | 368 | 80.394908 | 138.47 | 196.545092 | 0.376277 |  |
| GO:0055012\_ventricular\_cardiac\_muscle\_cell\_differentiation | 9 | 1 | 9.654088 | -1.004267 | 368 | 80.394908 | 138.47 | 196.545092 | 0.376277 |  |
| GO:0055013\_cardiac\_muscle\_cell\_development | 9 | 1 | 9.654088 | -1.004267 | 368 | 80.394908 | 138.47 | 196.545092 | 0.376277 |  |
| GO:0060325\_face\_morphogenesis | 9 | 1 | 9.654088 | -1.004267 | 368 | 80.394908 | 138.47 | 196.545092 | 0.376277 |  |
| GO:0060513\_prostatic\_bud\_formation | 9 | 1 | 9.654088 | -1.004267 | 368 | 80.394908 | 138.47 | 196.545092 | 0.376277 |  |
| GO:0060602\_branch\_elongation\_of\_an\_epithelium | 9 | 1 | 9.654088 | -1.004267 | 368 | 80.394908 | 138.47 | 196.545092 | 0.376277 |  |
| GO:0070306\_lens\_fiber\_cell\_differentiation | 9 | 1 | 9.654088 | -1.004267 | 368 | 80.394908 | 138.47 | 196.545092 | 0.376277 |  |
| GO:0010468\_regulation\_of\_gene\_expression | 778 | 13 | 1.451836 | -1.003521 | 369 | 80.421970 | 138.54 | 196.658030 | 0.375447 |  |
| GO:0044238\_primary\_metabolic\_process | 1905 | 27 | 1.231466 | -0.998594 | 370 | 80.723825 | 139.02 | 197.316175 | 0.375730 |  |
| GO:0043283\_biopolymer\_metabolic\_process | 1490 | 22 | 1.282892 | -0.995879 | 371 | 80.751789 | 139.15 | 197.548211 | 0.375067 |  |
| GO:0044267\_cellular\_protein\_metabolic\_process | 559 | 10 | 1.554325 | -0.992803 | 372 | 81.431002 | 140.33 | 199.228998 | 0.377231 |  |
| GO:0007548\_sex\_differentiation | 98 | 3 | 2.659800 | -0.990334 | 374 | 81.630061 | 140.77 | 199.909939 | 0.376390 |  |
| GO:0009314\_response\_to\_radiation | 98 | 3 | 2.659800 | -0.990334 | 374 | 81.630061 | 140.77 | 199.909939 | 0.376390 |  |
| GO:0043170\_macromolecule\_metabolic\_process | 1576 | 23 | 1.268018 | -0.985708 | 375 | 81.728168 | 141.08 | 200.431832 | 0.376213 |  |
| GO:0060348\_bone\_development | 99 | 3 | 2.632933 | -0.980374 | 377 | 81.944715 | 141.58 | 201.215285 | 0.375544 |  |
| GO:0060562\_epithelial\_tube\_morphogenesis | 99 | 3 | 2.632933 | -0.980374 | 377 | 81.944715 | 141.58 | 201.215285 | 0.375544 |  |
| GO:0046849\_bone\_remodeling | 48 | 2 | 3.620283 | -0.979221 | 378 | 82.430529 | 142.3 | 202.169471 | 0.376455 |  |
| GO:0006066\_alcohol\_metabolic\_process | 158 | 4 | 2.199666 | -0.969974 | 379 | 83.051276 | 143.25 | 203.448724 | 0.377968 |  |
| GO:0022008\_neurogenesis | 423 | 8 | 1.643249 | -0.964789 | 380 | 83.073240 | 143.3 | 203.526760 | 0.377105 |  |
| GO:0002440\_production\_of\_molecular\_mediator\_of\_immune\_response | 49 | 2 | 3.546400 | -0.964222 | 383 | 83.779252 | 144.58 | 205.380748 | 0.377493 |  |
| GO:0021543\_pallium\_development | 49 | 2 | 3.546400 | -0.964222 | 383 | 83.779252 | 144.58 | 205.380748 | 0.377493 |  |
| GO:0046661\_male\_sex\_differentiation | 49 | 2 | 3.546400 | -0.964222 | 383 | 83.779252 | 144.58 | 205.380748 | 0.377493 |  |
| GO:0002762\_negative\_regulation\_of\_myeloid\_leukocyte\_differentiation | 10 | 1 | 8.688679 | -0.960934 | 403 | 93.709561 | 157.34 | 220.970439 | 0.390422 |  |
| GO:0006081\_cellular\_aldehyde\_metabolic\_process | 10 | 1 | 8.688679 | -0.960934 | 403 | 93.709561 | 157.34 | 220.970439 | 0.390422 |  |
| GO:0009948\_anterior\_posterior\_axis\_specification | 10 | 1 | 8.688679 | -0.960934 | 403 | 93.709561 | 157.34 | 220.970439 | 0.390422 |  |
| GO:0030168\_platelet\_activation | 10 | 1 | 8.688679 | -0.960934 | 403 | 93.709561 | 157.34 | 220.970439 | 0.390422 |  |
| GO:0031018\_endocrine\_pancreas\_development | 10 | 1 | 8.688679 | -0.960934 | 403 | 93.709561 | 157.34 | 220.970439 | 0.390422 |  |
| GO:0034105\_positive\_regulation\_of\_tissue\_remodeling | 10 | 1 | 8.688679 | -0.960934 | 403 | 93.709561 | 157.34 | 220.970439 | 0.390422 |  |
| GO:0042116\_macrophage\_activation | 10 | 1 | 8.688679 | -0.960934 | 403 | 93.709561 | 157.34 | 220.970439 | 0.390422 |  |
| GO:0043506\_regulation\_of\_JUN\_kinase\_activity | 10 | 1 | 8.688679 | -0.960934 | 403 | 93.709561 | 157.34 | 220.970439 | 0.390422 |  |
| GO:0045446\_endothelial\_cell\_differentiation | 10 | 1 | 8.688679 | -0.960934 | 403 | 93.709561 | 157.34 | 220.970439 | 0.390422 |  |
| GO:0048339\_paraxial\_mesoderm\_development | 10 | 1 | 8.688679 | -0.960934 | 403 | 93.709561 | 157.34 | 220.970439 | 0.390422 |  |
| GO:0048596\_embryonic\_camera-type\_eye\_morphogenesis | 10 | 1 | 8.688679 | -0.960934 | 403 | 93.709561 | 157.34 | 220.970439 | 0.390422 |  |
| GO:0048738\_cardiac\_muscle\_tissue\_development | 10 | 1 | 8.688679 | -0.960934 | 403 | 93.709561 | 157.34 | 220.970439 | 0.390422 |  |
| GO:0050714\_positive\_regulation\_of\_protein\_secretion | 10 | 1 | 8.688679 | -0.960934 | 403 | 93.709561 | 157.34 | 220.970439 | 0.390422 |  |
| GO:0051147\_regulation\_of\_muscle\_cell\_differentiation | 10 | 1 | 8.688679 | -0.960934 | 403 | 93.709561 | 157.34 | 220.970439 | 0.390422 |  |
| GO:0051651\_maintenance\_of\_location\_in\_cell | 10 | 1 | 8.688679 | -0.960934 | 403 | 93.709561 | 157.34 | 220.970439 | 0.390422 |  |
| GO:0060323\_head\_morphogenesis | 10 | 1 | 8.688679 | -0.960934 | 403 | 93.709561 | 157.34 | 220.970439 | 0.390422 |  |
| GO:0060343\_trabecula\_formation | 10 | 1 | 8.688679 | -0.960934 | 403 | 93.709561 | 157.34 | 220.970439 | 0.390422 |  |
| GO:0060601\_lateral\_sprouting\_from\_an\_epithelium | 10 | 1 | 8.688679 | -0.960934 | 403 | 93.709561 | 157.34 | 220.970439 | 0.390422 |  |
| GO:0060669\_embryonic\_placenta\_morphogenesis | 10 | 1 | 8.688679 | -0.960934 | 403 | 93.709561 | 157.34 | 220.970439 | 0.390422 |  |
| GO:0060768\_regulation\_of\_epithelial\_cell\_proliferation\_involved\_in\_prostate\_gland\_development | 10 | 1 | 8.688679 | -0.960934 | 403 | 93.709561 | 157.34 | 220.970439 | 0.390422 |  |
| GO:0030036\_actin\_cytoskeleton\_organization | 102 | 3 | 2.555494 | -0.951284 | 404 | 93.954572 | 157.84 | 221.725428 | 0.390693 |  |
| GO:0001656\_metanephros\_development | 50 | 2 | 3.475472 | -0.949589 | 407 | 94.529597 | 158.63 | 222.730403 | 0.389754 |  |
| GO:0007015\_actin\_filament\_organization | 50 | 2 | 3.475472 | -0.949589 | 407 | 94.529597 | 158.63 | 222.730403 | 0.389754 |  |
| GO:0051606\_detection\_of\_stimulus | 50 | 2 | 3.475472 | -0.949589 | 407 | 94.529597 | 158.63 | 222.730403 | 0.389754 |  |
| GO:0006915\_apoptosis | 427 | 8 | 1.627856 | -0.947143 | 408 | 94.525540 | 158.77 | 223.014460 | 0.389142 |  |
| GO:0009628\_response\_to\_abiotic\_stimulus | 162 | 4 | 2.145353 | -0.940398 | 409 | 95.018217 | 159.55 | 224.081783 | 0.390098 |  |
| GO:0007601\_visual\_perception | 51 | 2 | 3.407325 | -0.935309 | 412 | 95.527579 | 160.36 | 225.192421 | 0.389223 |  |
| GO:0032583\_regulation\_of\_gene-specific\_transcription | 51 | 2 | 3.407325 | -0.935309 | 412 | 95.527579 | 160.36 | 225.192421 | 0.389223 |  |
| GO:0043408\_regulation\_of\_MAPKKK\_cascade | 51 | 2 | 3.407325 | -0.935309 | 412 | 95.527579 | 160.36 | 225.192421 | 0.389223 |  |
| GO:0042981\_regulation\_of\_apoptosis | 360 | 7 | 1.689465 | -0.934282 | 413 | 95.510218 | 160.41 | 225.309782 | 0.388402 |  |
| GO:0050767\_regulation\_of\_neurogenesis | 104 | 3 | 2.506350 | -0.932525 | 414 | 95.772492 | 160.76 | 225.747508 | 0.388309 |  |
| GO:0007229\_integrin-mediated\_signaling\_pathway | 11 | 1 | 7.898799 | -0.921962 | 425 | 103.878192 | 170.45 | 237.021808 | 0.401059 |  |
| GO:0008354\_germ\_cell\_migration | 11 | 1 | 7.898799 | -0.921962 | 425 | 103.878192 | 170.45 | 237.021808 | 0.401059 |  |
| GO:0014013\_regulation\_of\_gliogenesis | 11 | 1 | 7.898799 | -0.921962 | 425 | 103.878192 | 170.45 | 237.021808 | 0.401059 |  |
| GO:0021846\_cell\_proliferation\_in\_forebrain | 11 | 1 | 7.898799 | -0.921962 | 425 | 103.878192 | 170.45 | 237.021808 | 0.401059 |  |
| GO:0030856\_regulation\_of\_epithelial\_cell\_differentiation | 11 | 1 | 7.898799 | -0.921962 | 425 | 103.878192 | 170.45 | 237.021808 | 0.401059 |  |
| GO:0042401\_biogenic\_amine\_biosynthetic\_process | 11 | 1 | 7.898799 | -0.921962 | 425 | 103.878192 | 170.45 | 237.021808 | 0.401059 |  |
| GO:0045685\_regulation\_of\_glial\_cell\_differentiation | 11 | 1 | 7.898799 | -0.921962 | 425 | 103.878192 | 170.45 | 237.021808 | 0.401059 |  |
| GO:0045834\_positive\_regulation\_of\_lipid\_metabolic\_process | 11 | 1 | 7.898799 | -0.921962 | 425 | 103.878192 | 170.45 | 237.021808 | 0.401059 |  |
| GO:0048678\_response\_to\_axon\_injury | 11 | 1 | 7.898799 | -0.921962 | 425 | 103.878192 | 170.45 | 237.021808 | 0.401059 |  |
| GO:0048745\_smooth\_muscle\_tissue\_development | 11 | 1 | 7.898799 | -0.921962 | 425 | 103.878192 | 170.45 | 237.021808 | 0.401059 |  |
| GO:0060767\_epithelial\_cell\_proliferation\_involved\_in\_prostate\_gland\_development | 11 | 1 | 7.898799 | -0.921962 | 425 | 103.878192 | 170.45 | 237.021808 | 0.401059 |  |
| GO:0050953\_sensory\_perception\_of\_light\_stimulus | 52 | 2 | 3.341800 | -0.921366 | 426 | 104.202098 | 171.12 | 238.037902 | 0.401690 |  |
| GO:0012501\_programmed\_cell\_death | 433 | 8 | 1.605299 | -0.921245 | 427 | 104.254310 | 171.29 | 238.325690 | 0.401148 |  |
| GO:0007167\_enzyme\_linked\_receptor\_protein\_signaling\_pathway | 229 | 5 | 1.897092 | -0.915798 | 428 | 104.533430 | 172.03 | 239.526570 | 0.401939 |  |
| GO:0010941\_regulation\_of\_cell\_death | 365 | 7 | 1.666322 | -0.910964 | 430 | 104.770648 | 172.79 | 240.809352 | 0.401837 |  |
| GO:0043067\_regulation\_of\_programmed\_cell\_death | 365 | 7 | 1.666322 | -0.910964 | 430 | 104.770648 | 172.79 | 240.809352 | 0.401837 |  |
| GO:0016070\_RNA\_metabolic\_process | 658 | 11 | 1.452515 | -0.899739 | 431 | 105.606007 | 174.12 | 242.633993 | 0.403991 |  |
| GO:0007265\_Ras\_protein\_signal\_transduction | 54 | 2 | 3.218029 | -0.894443 | 433 | 106.407959 | 175.58 | 244.752041 | 0.405497 |  |
| GO:0044271\_nitrogen\_compound\_biosynthetic\_process | 54 | 2 | 3.218029 | -0.894443 | 433 | 106.407959 | 175.58 | 244.752041 | 0.405497 |  |
| GO:0031326\_regulation\_of\_cellular\_biosynthetic\_process | 812 | 13 | 1.391045 | -0.892665 | 434 | 106.379955 | 175.63 | 244.880045 | 0.404677 |  |
| GO:0030029\_actin\_filament-based\_process | 109 | 3 | 2.391380 | -0.887704 | 435 | 106.772130 | 176.1 | 245.427870 | 0.404828 |  |
| GO:0051239\_regulation\_of\_multicellular\_organismal\_process | 587 | 10 | 1.480184 | -0.887326 | 436 | 106.775832 | 176.15 | 245.524168 | 0.404014 |  |
| GO:0002089\_lens\_morphogenesis\_in\_camera-type\_eye | 12 | 1 | 7.240566 | -0.886590 | 454 | 114.863616 | 186.74 | 258.616384 | 0.411322 |  |
| GO:0002244\_hemopoietic\_progenitor\_cell\_differentiation | 12 | 1 | 7.240566 | -0.886590 | 454 | 114.863616 | 186.74 | 258.616384 | 0.411322 |  |
| GO:0006094\_gluconeogenesis | 12 | 1 | 7.240566 | -0.886590 | 454 | 114.863616 | 186.74 | 258.616384 | 0.411322 |  |
| GO:0019319\_hexose\_biosynthetic\_process | 12 | 1 | 7.240566 | -0.886590 | 454 | 114.863616 | 186.74 | 258.616384 | 0.411322 |  |
| GO:0021680\_cerebellar\_Purkinje\_cell\_layer\_development | 12 | 1 | 7.240566 | -0.886590 | 454 | 114.863616 | 186.74 | 258.616384 | 0.411322 |  |
| GO:0021697\_cerebellar\_cortex\_formation | 12 | 1 | 7.240566 | -0.886590 | 454 | 114.863616 | 186.74 | 258.616384 | 0.411322 |  |
| GO:0030239\_myofibril\_assembly | 12 | 1 | 7.240566 | -0.886590 | 454 | 114.863616 | 186.74 | 258.616384 | 0.411322 |  |
| GO:0033598\_mammary\_gland\_epithelial\_cell\_proliferation | 12 | 1 | 7.240566 | -0.886590 | 454 | 114.863616 | 186.74 | 258.616384 | 0.411322 |  |
| GO:0035050\_embryonic\_heart\_tube\_development | 12 | 1 | 7.240566 | -0.886590 | 454 | 114.863616 | 186.74 | 258.616384 | 0.411322 |  |
| GO:0043624\_cellular\_protein\_complex\_disassembly | 12 | 1 | 7.240566 | -0.886590 | 454 | 114.863616 | 186.74 | 258.616384 | 0.411322 |  |
| GO:0045995\_regulation\_of\_embryonic\_development | 12 | 1 | 7.240566 | -0.886590 | 454 | 114.863616 | 186.74 | 258.616384 | 0.411322 |  |
| GO:0046068\_cGMP\_metabolic\_process | 12 | 1 | 7.240566 | -0.886590 | 454 | 114.863616 | 186.74 | 258.616384 | 0.411322 |  |
| GO:0048821\_erythrocyte\_development | 12 | 1 | 7.240566 | -0.886590 | 454 | 114.863616 | 186.74 | 258.616384 | 0.411322 |  |
| GO:0048854\_brain\_morphogenesis | 12 | 1 | 7.240566 | -0.886590 | 454 | 114.863616 | 186.74 | 258.616384 | 0.411322 |  |
| GO:0051261\_protein\_depolymerization | 12 | 1 | 7.240566 | -0.886590 | 454 | 114.863616 | 186.74 | 258.616384 | 0.411322 |  |
| GO:0055002\_striated\_muscle\_cell\_development | 12 | 1 | 7.240566 | -0.886590 | 454 | 114.863616 | 186.74 | 258.616384 | 0.411322 |  |
| GO:0060042\_retina\_morphogenesis\_in\_camera-type\_eye | 12 | 1 | 7.240566 | -0.886590 | 454 | 114.863616 | 186.74 | 258.616384 | 0.411322 |  |
| GO:0060572\_morphogenesis\_of\_an\_epithelial\_bud | 12 | 1 | 7.240566 | -0.886590 | 454 | 114.863616 | 186.74 | 258.616384 | 0.411322 |  |
| GO:0051246\_regulation\_of\_protein\_metabolic\_process | 170 | 4 | 2.044395 | -0.884370 | 455 | 114.983852 | 186.88 | 258.776148 | 0.410725 |  |
| GO:0009889\_regulation\_of\_biosynthetic\_process | 815 | 13 | 1.385924 | -0.883390 | 456 | 114.953482 | 187.06 | 259.166518 | 0.410219 |  |
| GO:0048568\_embryonic\_organ\_development | 55 | 2 | 3.159520 | -0.881438 | 457 | 115.541585 | 187.82 | 260.098415 | 0.410985 |  |
| GO:0008219\_cell\_death | 444 | 8 | 1.565528 | -0.875487 | 458 | 115.890515 | 188.58 | 261.269485 | 0.411747 |  |
| GO:0048705\_skeletal\_system\_morphogenesis | 111 | 3 | 2.348292 | -0.870559 | 459 | 116.078940 | 188.87 | 261.661060 | 0.411481 |  |
| GO:0006790\_sulfur\_metabolic\_process | 56 | 2 | 3.103100 | -0.868723 | 461 | 116.869035 | 190.17 | 263.470965 | 0.412516 |  |
| GO:0050678\_regulation\_of\_epithelial\_cell\_proliferation | 56 | 2 | 3.103100 | -0.868723 | 461 | 116.869035 | 190.17 | 263.470965 | 0.412516 |  |
| GO:0008152\_metabolic\_process | 2133 | 29 | 1.181302 | -0.863563 | 462 | 117.097874 | 190.54 | 263.982126 | 0.412424 |  |
| GO:0010556\_regulation\_of\_macromolecule\_biosynthetic\_process | 745 | 12 | 1.399519 | -0.862601 | 463 | 117.150843 | 190.75 | 264.349157 | 0.411987 |  |
| GO:0043549\_regulation\_of\_kinase\_activity | 112 | 3 | 2.327325 | -0.862147 | 464 | 117.260771 | 191.02 | 264.779229 | 0.411681 |  |
| GO:0022414\_reproductive\_process | 376 | 7 | 1.617573 | -0.861644 | 465 | 117.286036 | 191.08 | 264.873964 | 0.410925 |  |
| GO:0001892\_embryonic\_placenta\_development | 57 | 2 | 3.048659 | -0.856288 | 466 | 118.380359 | 192.82 | 267.259641 | 0.413777 |  |
| GO:0006090\_pyruvate\_metabolic\_process | 13 | 1 | 6.683599 | -0.854239 | 478 | 126.684730 | 202.83 | 278.975270 | 0.424331 |  |
| GO:0021533\_cell\_differentiation\_in\_hindbrain | 13 | 1 | 6.683599 | -0.854239 | 478 | 126.684730 | 202.83 | 278.975270 | 0.424331 |  |
| GO:0031032\_actomyosin\_structure\_organization | 13 | 1 | 6.683599 | -0.854239 | 478 | 126.684730 | 202.83 | 278.975270 | 0.424331 |  |
| GO:0032330\_regulation\_of\_chondrocyte\_differentiation | 13 | 1 | 6.683599 | -0.854239 | 478 | 126.684730 | 202.83 | 278.975270 | 0.424331 |  |
| GO:0042474\_middle\_ear\_morphogenesis | 13 | 1 | 6.683599 | -0.854239 | 478 | 126.684730 | 202.83 | 278.975270 | 0.424331 |  |
| GO:0043241\_protein\_complex\_disassembly | 13 | 1 | 6.683599 | -0.854239 | 478 | 126.684730 | 202.83 | 278.975270 | 0.424331 |  |
| GO:0043244\_regulation\_of\_protein\_complex\_disassembly | 13 | 1 | 6.683599 | -0.854239 | 478 | 126.684730 | 202.83 | 278.975270 | 0.424331 |  |
| GO:0048305\_immunoglobulin\_secretion | 13 | 1 | 6.683599 | -0.854239 | 478 | 126.684730 | 202.83 | 278.975270 | 0.424331 |  |
| GO:0050818\_regulation\_of\_coagulation | 13 | 1 | 6.683599 | -0.854239 | 478 | 126.684730 | 202.83 | 278.975270 | 0.424331 |  |
| GO:0060324\_face\_development | 13 | 1 | 6.683599 | -0.854239 | 478 | 126.684730 | 202.83 | 278.975270 | 0.424331 |  |
| GO:0060560\_developmental\_growth\_involved\_in\_morphogenesis | 13 | 1 | 6.683599 | -0.854239 | 478 | 126.684730 | 202.83 | 278.975270 | 0.424331 |  |
| GO:0060742\_epithelial\_cell\_differentiation\_involved\_in\_prostate\_gland\_development | 13 | 1 | 6.683599 | -0.854239 | 478 | 126.684730 | 202.83 | 278.975270 | 0.424331 |  |
| GO:0040008\_regulation\_of\_growth | 113 | 3 | 2.306729 | -0.853838 | 479 | 126.843307 | 203.02 | 279.196693 | 0.423841 |  |
| GO:0016265\_death | 450 | 8 | 1.544654 | -0.851433 | 480 | 126.878493 | 203.23 | 279.581507 | 0.423396 |  |
| GO:0000122\_negative\_regulation\_of\_transcription\_from\_RNA\_polymerase\_II\_promoter | 175 | 4 | 1.985984 | -0.851323 | 481 | 127.115741 | 203.61 | 280.104259 | 0.423306 |  |
| GO:0000003\_reproduction | 379 | 7 | 1.604769 | -0.848648 | 482 | 127.139415 | 203.67 | 280.200585 | 0.422552 |  |
| GO:0033043\_regulation\_of\_organelle\_organization | 58 | 2 | 2.996096 | -0.844122 | 483 | 128.185695 | 205.04 | 281.894305 | 0.424513 |  |
| GO:0051338\_regulation\_of\_transferase\_activity | 115 | 3 | 2.266612 | -0.837525 | 484 | 128.363454 | 205.39 | 282.416546 | 0.424360 |  |
| GO:0048732\_gland\_development | 179 | 4 | 1.941604 | -0.825905 | 485 | 129.450166 | 207.21 | 284.969834 | 0.427237 |  |
| GO:0019219\_regulation\_of\_nucleobase\_\_nucleoside\_\_nucleotide\_and\_nucleic\_acid\_metabolic\_process | 757 | 12 | 1.377334 | -0.825474 | 486 | 129.466876 | 207.26 | 285.053124 | 0.426461 |  |
| GO:0001502\_cartilage\_condensation | 14 | 1 | 6.206199 | -0.824462 | 501 | 136.910018 | 216.68 | 296.449982 | 0.432495 |  |
| GO:0001829\_trophectodermal\_cell\_differentiation | 14 | 1 | 6.206199 | -0.824462 | 501 | 136.910018 | 216.68 | 296.449982 | 0.432495 |  |
| GO:0006695\_cholesterol\_biosynthetic\_process | 14 | 1 | 6.206199 | -0.824462 | 501 | 136.910018 | 216.68 | 296.449982 | 0.432495 |  |
| GO:0006809\_nitric\_oxide\_biosynthetic\_process | 14 | 1 | 6.206199 | -0.824462 | 501 | 136.910018 | 216.68 | 296.449982 | 0.432495 |  |
| GO:0008064\_regulation\_of\_actin\_polymerization\_or\_depolymerization | 14 | 1 | 6.206199 | -0.824462 | 501 | 136.910018 | 216.68 | 296.449982 | 0.432495 |  |
| GO:0010332\_response\_to\_gamma\_radiation | 14 | 1 | 6.206199 | -0.824462 | 501 | 136.910018 | 216.68 | 296.449982 | 0.432495 |  |
| GO:0030032\_lamellipodium\_assembly | 14 | 1 | 6.206199 | -0.824462 | 501 | 136.910018 | 216.68 | 296.449982 | 0.432495 |  |
| GO:0030832\_regulation\_of\_actin\_filament\_length | 14 | 1 | 6.206199 | -0.824462 | 501 | 136.910018 | 216.68 | 296.449982 | 0.432495 |  |
| GO:0031099\_regeneration | 14 | 1 | 6.206199 | -0.824462 | 501 | 136.910018 | 216.68 | 296.449982 | 0.432495 |  |
| GO:0034623\_cellular\_macromolecular\_complex\_disassembly | 14 | 1 | 6.206199 | -0.824462 | 501 | 136.910018 | 216.68 | 296.449982 | 0.432495 |  |
| GO:0035036\_sperm-egg\_recognition | 14 | 1 | 6.206199 | -0.824462 | 501 | 136.910018 | 216.68 | 296.449982 | 0.432495 |  |
| GO:0042310\_vasoconstriction | 14 | 1 | 6.206199 | -0.824462 | 501 | 136.910018 | 216.68 | 296.449982 | 0.432495 |  |
| GO:0045453\_bone\_resorption | 14 | 1 | 6.206199 | -0.824462 | 501 | 136.910018 | 216.68 | 296.449982 | 0.432495 |  |
| GO:0046209\_nitric\_oxide\_metabolic\_process | 14 | 1 | 6.206199 | -0.824462 | 501 | 136.910018 | 216.68 | 296.449982 | 0.432495 |  |
| GO:0048048\_embryonic\_eye\_morphogenesis | 14 | 1 | 6.206199 | -0.824462 | 501 | 136.910018 | 216.68 | 296.449982 | 0.432495 |  |
| GO:0051960\_regulation\_of\_nervous\_system\_development | 118 | 3 | 2.208986 | -0.813789 | 502 | 137.414421 | 217.68 | 297.945579 | 0.433625 |  |
| GO:0032270\_positive\_regulation\_of\_cellular\_protein\_metabolic\_process | 61 | 2 | 2.848747 | -0.809148 | 503 | 138.448803 | 219.16 | 299.871197 | 0.435706 |  |
| GO:0014706\_striated\_muscle\_tissue\_development | 120 | 3 | 2.172170 | -0.798434 | 504 | 139.172242 | 220.04 | 300.907758 | 0.436587 |  |
| GO:0021537\_telencephalon\_development | 62 | 2 | 2.802800 | -0.797970 | 506 | 139.801685 | 220.98 | 302.158315 | 0.436719 |  |
| GO:0030855\_epithelial\_cell\_differentiation | 62 | 2 | 2.802800 | -0.797970 | 506 | 139.801685 | 220.98 | 302.158315 | 0.436719 |  |
| GO:0010171\_body\_morphogenesis | 15 | 1 | 5.792453 | -0.796902 | 518 | 146.740304 | 229.67 | 312.599696 | 0.443378 |  |
| GO:0021795\_cerebral\_cortex\_cell\_migration | 15 | 1 | 5.792453 | -0.796902 | 518 | 146.740304 | 229.67 | 312.599696 | 0.443378 |  |
| GO:0031069\_hair\_follicle\_morphogenesis | 15 | 1 | 5.792453 | -0.796902 | 518 | 146.740304 | 229.67 | 312.599696 | 0.443378 |  |
| GO:0031076\_embryonic\_camera-type\_eye\_development | 15 | 1 | 5.792453 | -0.796902 | 518 | 146.740304 | 229.67 | 312.599696 | 0.443378 |  |
| GO:0035116\_embryonic\_hindlimb\_morphogenesis | 15 | 1 | 5.792453 | -0.796902 | 518 | 146.740304 | 229.67 | 312.599696 | 0.443378 |  |
| GO:0048008\_platelet-derived\_growth\_factor\_receptor\_signaling\_pathway | 15 | 1 | 5.792453 | -0.796902 | 518 | 146.740304 | 229.67 | 312.599696 | 0.443378 |  |
| GO:0048010\_vascular\_endothelial\_growth\_factor\_receptor\_signaling\_pathway | 15 | 1 | 5.792453 | -0.796902 | 518 | 146.740304 | 229.67 | 312.599696 | 0.443378 |  |
| GO:0048144\_fibroblast\_proliferation | 15 | 1 | 5.792453 | -0.796902 | 518 | 146.740304 | 229.67 | 312.599696 | 0.443378 |  |
| GO:0048145\_regulation\_of\_fibroblast\_proliferation | 15 | 1 | 5.792453 | -0.796902 | 518 | 146.740304 | 229.67 | 312.599696 | 0.443378 |  |
| GO:0048610\_reproductive\_cellular\_process | 15 | 1 | 5.792453 | -0.796902 | 518 | 146.740304 | 229.67 | 312.599696 | 0.443378 |  |
| GO:0060322\_head\_development | 15 | 1 | 5.792453 | -0.796902 | 518 | 146.740304 | 229.67 | 312.599696 | 0.443378 |  |
| GO:0070227\_lymphocyte\_apoptosis | 15 | 1 | 5.792453 | -0.796902 | 518 | 146.740304 | 229.67 | 312.599696 | 0.443378 |  |
| GO:0008104\_protein\_localization | 251 | 5 | 1.730813 | -0.795393 | 519 | 146.807938 | 229.87 | 312.932062 | 0.442909 |  |
| GO:0007369\_gastrulation | 63 | 2 | 2.758311 | -0.787019 | 520 | 147.862532 | 231.69 | 315.517468 | 0.445558 |  |
| GO:0065008\_regulation\_of\_biological\_quality | 693 | 11 | 1.379155 | -0.786355 | 521 | 147.860133 | 231.77 | 315.679867 | 0.444856 |  |
| GO:0016481\_negative\_regulation\_of\_transcription | 253 | 5 | 1.717130 | -0.785313 | 523 | 147.958667 | 232.03 | 316.101333 | 0.443652 |  |
| GO:0030097\_hemopoiesis | 253 | 5 | 1.717130 | -0.785313 | 523 | 147.958667 | 232.03 | 316.101333 | 0.443652 |  |
| GO:0051171\_regulation\_of\_nitrogen\_compound\_metabolic\_process | 771 | 12 | 1.352324 | -0.783853 | 524 | 147.991922 | 232.09 | 316.188078 | 0.442920 |  |
| GO:0048699\_generation\_of\_neurons | 396 | 7 | 1.535878 | -0.778492 | 525 | 148.957559 | 233.47 | 317.982441 | 0.444705 |  |
| GO:0001933\_negative\_regulation\_of\_protein\_amino\_acid\_phosphorylation | 16 | 1 | 5.430425 | -0.771273 | 532 | 155.717717 | 242.4 | 329.082283 | 0.455639 |  |
| GO:0009988\_cell-cell\_recognition | 16 | 1 | 5.430425 | -0.771273 | 532 | 155.717717 | 242.4 | 329.082283 | 0.455639 |  |
| GO:0016126\_sterol\_biosynthetic\_process | 16 | 1 | 5.430425 | -0.771273 | 532 | 155.717717 | 242.4 | 329.082283 | 0.455639 |  |
| GO:0021696\_cerebellar\_cortex\_morphogenesis | 16 | 1 | 5.430425 | -0.771273 | 532 | 155.717717 | 242.4 | 329.082283 | 0.455639 |  |
| GO:0043087\_regulation\_of\_GTPase\_activity | 16 | 1 | 5.430425 | -0.771273 | 532 | 155.717717 | 242.4 | 329.082283 | 0.455639 |  |
| GO:0046364\_monosaccharide\_biosynthetic\_process | 16 | 1 | 5.430425 | -0.771273 | 532 | 155.717717 | 242.4 | 329.082283 | 0.455639 |  |
| GO:0060713\_labyrinthine\_layer\_morphogenesis | 16 | 1 | 5.430425 | -0.771273 | 532 | 155.717717 | 242.4 | 329.082283 | 0.455639 |  |
| GO:0030098\_lymphocyte\_differentiation | 124 | 3 | 2.102100 | -0.768792 | 533 | 155.829685 | 242.64 | 329.450315 | 0.455235 |  |
| GO:0007179\_transforming\_growth\_factor\_beta\_receptor\_signaling\_pathway | 66 | 2 | 2.632933 | -0.755455 | 536 | 157.508738 | 245.18 | 332.851262 | 0.457425 |  |
| GO:0045860\_positive\_regulation\_of\_protein\_kinase\_activity | 66 | 2 | 2.632933 | -0.755455 | 536 | 157.508738 | 245.18 | 332.851262 | 0.457425 |  |
| GO:0051130\_positive\_regulation\_of\_cellular\_component\_organization | 66 | 2 | 2.632933 | -0.755455 | 536 | 157.508738 | 245.18 | 332.851262 | 0.457425 |  |
| GO:0010605\_negative\_regulation\_of\_macromolecule\_metabolic\_process | 331 | 6 | 1.574987 | -0.749576 | 537 | 157.833330 | 245.75 | 333.666670 | 0.457635 |  |
| GO:0009408\_response\_to\_heat | 17 | 1 | 5.110988 | -0.747338 | 545 | 165.051390 | 253.92 | 342.788610 | 0.465908 |  |
| GO:0022029\_telencephalon\_cell\_migration | 17 | 1 | 5.110988 | -0.747338 | 545 | 165.051390 | 253.92 | 342.788610 | 0.465908 |  |
| GO:0032535\_regulation\_of\_cellular\_component\_size | 17 | 1 | 5.110988 | -0.747338 | 545 | 165.051390 | 253.92 | 342.788610 | 0.465908 |  |
| GO:0042398\_cellular\_amino\_acid\_derivative\_biosynthetic\_process | 17 | 1 | 5.110988 | -0.747338 | 545 | 165.051390 | 253.92 | 342.788610 | 0.465908 |  |
| GO:0042417\_dopamine\_metabolic\_process | 17 | 1 | 5.110988 | -0.747338 | 545 | 165.051390 | 253.92 | 342.788610 | 0.465908 |  |
| GO:0045670\_regulation\_of\_osteoclast\_differentiation | 17 | 1 | 5.110988 | -0.747338 | 545 | 165.051390 | 253.92 | 342.788610 | 0.465908 |  |
| GO:0050708\_regulation\_of\_protein\_secretion | 17 | 1 | 5.110988 | -0.747338 | 545 | 165.051390 | 253.92 | 342.788610 | 0.465908 |  |
| GO:0055001\_muscle\_cell\_development | 17 | 1 | 5.110988 | -0.747338 | 545 | 165.051390 | 253.92 | 342.788610 | 0.465908 |  |
| GO:0031324\_negative\_regulation\_of\_cellular\_metabolic\_process | 332 | 6 | 1.570243 | -0.745371 | 546 | 165.138797 | 254.01 | 342.881203 | 0.465220 |  |
| GO:0051247\_positive\_regulation\_of\_protein\_metabolic\_process | 67 | 2 | 2.593636 | -0.745342 | 547 | 165.754778 | 254.82 | 343.885222 | 0.465850 |  |
| GO:0001775\_cell\_activation | 262 | 5 | 1.658145 | -0.741575 | 549 | 166.001694 | 255.25 | 344.498306 | 0.464936 |  |
| GO:0010629\_negative\_regulation\_of\_gene\_expression | 262 | 5 | 1.658145 | -0.741575 | 549 | 166.001694 | 255.25 | 344.498306 | 0.464936 |  |
| GO:0045597\_positive\_regulation\_of\_cell\_differentiation | 128 | 3 | 2.036409 | -0.740498 | 550 | 166.191963 | 255.66 | 345.128037 | 0.464836 |  |
| GO:0042692\_muscle\_cell\_differentiation | 68 | 2 | 2.555494 | -0.735422 | 551 | 166.723801 | 256.44 | 346.156199 | 0.465408 |  |
| GO:0001825\_blastocyst\_formation | 18 | 1 | 4.827044 | -0.724905 | 561 | 176.139200 | 268.15 | 360.160800 | 0.477986 |  |
| GO:0008589\_regulation\_of\_smoothened\_signaling\_pathway | 18 | 1 | 4.827044 | -0.724905 | 561 | 176.139200 | 268.15 | 360.160800 | 0.477986 |  |
| GO:0021885\_forebrain\_cell\_migration | 18 | 1 | 4.827044 | -0.724905 | 561 | 176.139200 | 268.15 | 360.160800 | 0.477986 |  |
| GO:0030282\_bone\_mineralization | 18 | 1 | 4.827044 | -0.724905 | 561 | 176.139200 | 268.15 | 360.160800 | 0.477986 |  |
| GO:0032984\_macromolecular\_complex\_disassembly | 18 | 1 | 4.827044 | -0.724905 | 561 | 176.139200 | 268.15 | 360.160800 | 0.477986 |  |
| GO:0043029\_T\_cell\_homeostasis | 18 | 1 | 4.827044 | -0.724905 | 561 | 176.139200 | 268.15 | 360.160800 | 0.477986 |  |
| GO:0045638\_negative\_regulation\_of\_myeloid\_cell\_differentiation | 18 | 1 | 4.827044 | -0.724905 | 561 | 176.139200 | 268.15 | 360.160800 | 0.477986 |  |
| GO:0048730\_epidermis\_morphogenesis | 18 | 1 | 4.827044 | -0.724905 | 561 | 176.139200 | 268.15 | 360.160800 | 0.477986 |  |
| GO:0051222\_positive\_regulation\_of\_protein\_transport | 18 | 1 | 4.827044 | -0.724905 | 561 | 176.139200 | 268.15 | 360.160800 | 0.477986 |  |
| GO:0060571\_morphogenesis\_of\_an\_epithelial\_fold | 18 | 1 | 4.827044 | -0.724905 | 561 | 176.139200 | 268.15 | 360.160800 | 0.477986 |  |
| GO:0000904\_cell\_morphogenesis\_involved\_in\_differentiation | 199 | 4 | 1.746468 | -0.710986 | 562 | 177.836779 | 270.54 | 363.243221 | 0.481388 |  |
| GO:0009100\_glycoprotein\_metabolic\_process | 71 | 2 | 2.447515 | -0.706768 | 563 | 179.178793 | 271.9 | 364.621207 | 0.482948 |  |
| GO:0045934\_negative\_regulation\_of\_nucleobase\_\_nucleoside\_\_nucleotide\_and\_nucleic\_acid\_metabolic\_process | 270 | 5 | 1.609015 | -0.704817 | 564 | 179.242936 | 272.1 | 364.957064 | 0.482447 |  |
| GO:0006776\_vitamin\_A\_metabolic\_process | 19 | 1 | 4.572989 | -0.703810 | 575 | 184.210150 | 278.5 | 372.789850 | 0.484348 |  |
| GO:0009798\_axis\_specification | 19 | 1 | 4.572989 | -0.703810 | 575 | 184.210150 | 278.5 | 372.789850 | 0.484348 |  |
| GO:0019218\_regulation\_of\_steroid\_metabolic\_process | 19 | 1 | 4.572989 | -0.703810 | 575 | 184.210150 | 278.5 | 372.789850 | 0.484348 |  |
| GO:0021587\_cerebellum\_morphogenesis | 19 | 1 | 4.572989 | -0.703810 | 575 | 184.210150 | 278.5 | 372.789850 | 0.484348 |  |
| GO:0030199\_collagen\_fibril\_organization | 19 | 1 | 4.572989 | -0.703810 | 575 | 184.210150 | 278.5 | 372.789850 | 0.484348 |  |
| GO:0032526\_response\_to\_retinoic\_acid | 19 | 1 | 4.572989 | -0.703810 | 575 | 184.210150 | 278.5 | 372.789850 | 0.484348 |  |
| GO:0033189\_response\_to\_vitamin\_A | 19 | 1 | 4.572989 | -0.703810 | 575 | 184.210150 | 278.5 | 372.789850 | 0.484348 |  |
| GO:0042491\_auditory\_receptor\_cell\_differentiation | 19 | 1 | 4.572989 | -0.703810 | 575 | 184.210150 | 278.5 | 372.789850 | 0.484348 |  |
| GO:0046165\_alcohol\_biosynthetic\_process | 19 | 1 | 4.572989 | -0.703810 | 575 | 184.210150 | 278.5 | 372.789850 | 0.484348 |  |
| GO:0048701\_embryonic\_cranial\_skeleton\_morphogenesis | 19 | 1 | 4.572989 | -0.703810 | 575 | 184.210150 | 278.5 | 372.789850 | 0.484348 |  |
| GO:0060444\_branching\_involved\_in\_mammary\_gland\_duct\_morphogenesis | 19 | 1 | 4.572989 | -0.703810 | 575 | 184.210150 | 278.5 | 372.789850 | 0.484348 |  |
| GO:0051172\_negative\_regulation\_of\_nitrogen\_compound\_metabolic\_process | 271 | 5 | 1.603077 | -0.700356 | 576 | 184.590512 | 279.0 | 373.409488 | 0.484375 |  |
| GO:0048839\_inner\_ear\_development | 72 | 2 | 2.413522 | -0.697567 | 578 | 185.978367 | 280.76 | 375.541633 | 0.485744 |  |
| GO:0050673\_epithelial\_cell\_proliferation | 72 | 2 | 2.413522 | -0.697567 | 578 | 185.978367 | 280.76 | 375.541633 | 0.485744 |  |
| GO:0034961\_cellular\_biopolymer\_biosynthetic\_process | 804 | 12 | 1.296818 | -0.692578 | 579 | 186.038398 | 280.85 | 375.661602 | 0.485060 |  |
| GO:0006163\_purine\_nucleotide\_metabolic\_process | 73 | 2 | 2.380460 | -0.688533 | 581 | 187.190996 | 282.17 | 377.149004 | 0.485663 |  |
| GO:0048706\_embryonic\_skeletal\_system\_development | 73 | 2 | 2.380460 | -0.688533 | 581 | 187.190996 | 282.17 | 377.149004 | 0.485663 |  |
| GO:0010558\_negative\_regulation\_of\_macromolecule\_biosynthetic\_process | 274 | 5 | 1.585525 | -0.687146 | 583 | 187.411525 | 282.53 | 377.648475 | 0.484614 |  |
| GO:0033036\_macromolecule\_localization | 274 | 5 | 1.585525 | -0.687146 | 583 | 187.411525 | 282.53 | 377.648475 | 0.484614 |  |
| GO:0043284\_biopolymer\_biosynthetic\_process | 807 | 12 | 1.291997 | -0.684734 | 584 | 187.606361 | 282.79 | 377.973639 | 0.484229 |  |
| GO:0006584\_catecholamine\_metabolic\_process | 20 | 1 | 4.344340 | -0.683915 | 594 | 192.184053 | 288.71 | 385.235947 | 0.486044 |  |
| GO:0008360\_regulation\_of\_cell\_shape | 20 | 1 | 4.344340 | -0.683915 | 594 | 192.184053 | 288.71 | 385.235947 | 0.486044 |  |
| GO:0009712\_catechol\_metabolic\_process | 20 | 1 | 4.344340 | -0.683915 | 594 | 192.184053 | 288.71 | 385.235947 | 0.486044 |  |
| GO:0010927\_cellular\_component\_assembly\_involved\_in\_morphogenesis | 20 | 1 | 4.344340 | -0.683915 | 594 | 192.184053 | 288.71 | 385.235947 | 0.486044 |  |
| GO:0018958\_phenol\_metabolic\_process | 20 | 1 | 4.344340 | -0.683915 | 594 | 192.184053 | 288.71 | 385.235947 | 0.486044 |  |
| GO:0021695\_cerebellar\_cortex\_development | 20 | 1 | 4.344340 | -0.683915 | 594 | 192.184053 | 288.71 | 385.235947 | 0.486044 |  |
| GO:0031214\_biomineral\_formation | 20 | 1 | 4.344340 | -0.683915 | 594 | 192.184053 | 288.71 | 385.235947 | 0.486044 |  |
| GO:0034311\_diol\_metabolic\_process | 20 | 1 | 4.344340 | -0.683915 | 594 | 192.184053 | 288.71 | 385.235947 | 0.486044 |  |
| GO:0042326\_negative\_regulation\_of\_phosphorylation | 20 | 1 | 4.344340 | -0.683915 | 594 | 192.184053 | 288.71 | 385.235947 | 0.486044 |  |
| GO:0048806\_genitalia\_development | 20 | 1 | 4.344340 | -0.683915 | 594 | 192.184053 | 288.71 | 385.235947 | 0.486044 |  |
| GO:0009892\_negative\_regulation\_of\_metabolic\_process | 348 | 6 | 1.498048 | -0.681155 | 595 | 192.437762 | 288.98 | 385.522238 | 0.485681 |  |
| GO:0009416\_response\_to\_light\_stimulus | 74 | 2 | 2.348292 | -0.679662 | 596 | 192.934951 | 289.63 | 386.325049 | 0.485956 |  |
| GO:0048534\_hemopoietic\_or\_lymphoid\_organ\_development | 277 | 5 | 1.568354 | -0.674189 | 597 | 193.166403 | 290.09 | 387.013597 | 0.485913 |  |
| GO:0019538\_protein\_metabolic\_process | 655 | 10 | 1.326516 | -0.670967 | 598 | 193.277037 | 290.38 | 387.482963 | 0.485585 |  |
| GO:0007281\_germ\_cell\_development | 75 | 2 | 2.316981 | -0.670948 | 600 | 193.674921 | 290.97 | 388.265079 | 0.484950 |  |
| GO:0048589\_developmental\_growth | 75 | 2 | 2.316981 | -0.670948 | 600 | 193.674921 | 290.97 | 388.265079 | 0.484950 |  |
| GO:0001702\_gastrulation\_with\_mouth\_forming\_second | 21 | 1 | 4.137466 | -0.665102 | 609 | 200.007721 | 299.68 | 399.352279 | 0.492085 |  |
| GO:0002053\_positive\_regulation\_of\_mesenchymal\_cell\_proliferation | 21 | 1 | 4.137466 | -0.665102 | 609 | 200.007721 | 299.68 | 399.352279 | 0.492085 |  |
| GO:0006944\_membrane\_fusion | 21 | 1 | 4.137466 | -0.665102 | 609 | 200.007721 | 299.68 | 399.352279 | 0.492085 |  |
| GO:0008154\_actin\_polymerization\_or\_depolymerization | 21 | 1 | 4.137466 | -0.665102 | 609 | 200.007721 | 299.68 | 399.352279 | 0.492085 |  |
| GO:0010563\_negative\_regulation\_of\_phosphorus\_metabolic\_process | 21 | 1 | 4.137466 | -0.665102 | 609 | 200.007721 | 299.68 | 399.352279 | 0.492085 |  |
| GO:0016202\_regulation\_of\_striated\_muscle\_tissue\_development | 21 | 1 | 4.137466 | -0.665102 | 609 | 200.007721 | 299.68 | 399.352279 | 0.492085 |  |
| GO:0045936\_negative\_regulation\_of\_phosphate\_metabolic\_process | 21 | 1 | 4.137466 | -0.665102 | 609 | 200.007721 | 299.68 | 399.352279 | 0.492085 |  |
| GO:0048538\_thymus\_development | 21 | 1 | 4.137466 | -0.665102 | 609 | 200.007721 | 299.68 | 399.352279 | 0.492085 |  |
| GO:0048634\_regulation\_of\_muscle\_development | 21 | 1 | 4.137466 | -0.665102 | 609 | 200.007721 | 299.68 | 399.352279 | 0.492085 |  |
| GO:0051179\_localization | 1058 | 15 | 1.231854 | -0.659274 | 610 | 201.064602 | 301.1 | 401.135398 | 0.493607 |  |
| GO:0001890\_placenta\_development | 77 | 2 | 2.256800 | -0.653978 | 611 | 201.561205 | 301.91 | 402.258795 | 0.494124 |  |
| GO:0031327\_negative\_regulation\_of\_cellular\_biosynthetic\_process | 282 | 5 | 1.540546 | -0.653145 | 612 | 201.670489 | 302.04 | 402.409511 | 0.493529 |  |
| GO:0034645\_cellular\_macromolecule\_biosynthetic\_process | 901 | 13 | 1.253639 | -0.649025 | 613 | 201.742949 | 302.28 | 402.817051 | 0.493116 |  |
| GO:0000902\_cell\_morphogenesis | 283 | 5 | 1.535102 | -0.649017 | 614 | 201.906452 | 302.42 | 402.933548 | 0.492541 |  |
| GO:0001523\_retinoid\_metabolic\_process | 22 | 1 | 3.949400 | -0.647272 | 628 | 209.320309 | 311.32 | 413.319691 | 0.495732 |  |
| GO:0001569\_patterning\_of\_blood\_vessels | 22 | 1 | 3.949400 | -0.647272 | 628 | 209.320309 | 311.32 | 413.319691 | 0.495732 |  |
| GO:0002637\_regulation\_of\_immunoglobulin\_production | 22 | 1 | 3.949400 | -0.647272 | 628 | 209.320309 | 311.32 | 413.319691 | 0.495732 |  |
| GO:0006721\_terpenoid\_metabolic\_process | 22 | 1 | 3.949400 | -0.647272 | 628 | 209.320309 | 311.32 | 413.319691 | 0.495732 |  |
| GO:0009309\_amine\_biosynthetic\_process | 22 | 1 | 3.949400 | -0.647272 | 628 | 209.320309 | 311.32 | 413.319691 | 0.495732 |  |
| GO:0010463\_mesenchymal\_cell\_proliferation | 22 | 1 | 3.949400 | -0.647272 | 628 | 209.320309 | 311.32 | 413.319691 | 0.495732 |  |
| GO:0010464\_regulation\_of\_mesenchymal\_cell\_proliferation | 22 | 1 | 3.949400 | -0.647272 | 628 | 209.320309 | 311.32 | 413.319691 | 0.495732 |  |
| GO:0016101\_diterpenoid\_metabolic\_process | 22 | 1 | 3.949400 | -0.647272 | 628 | 209.320309 | 311.32 | 413.319691 | 0.495732 |  |
| GO:0021575\_hindbrain\_morphogenesis | 22 | 1 | 3.949400 | -0.647272 | 628 | 209.320309 | 311.32 | 413.319691 | 0.495732 |  |
| GO:0030258\_lipid\_modification | 22 | 1 | 3.949400 | -0.647272 | 628 | 209.320309 | 311.32 | 413.319691 | 0.495732 |  |
| GO:0030316\_osteoclast\_differentiation | 22 | 1 | 3.949400 | -0.647272 | 628 | 209.320309 | 311.32 | 413.319691 | 0.495732 |  |
| GO:0033273\_response\_to\_vitamin | 22 | 1 | 3.949400 | -0.647272 | 628 | 209.320309 | 311.32 | 413.319691 | 0.495732 |  |
| GO:0042733\_embryonic\_digit\_morphogenesis | 22 | 1 | 3.949400 | -0.647272 | 628 | 209.320309 | 311.32 | 413.319691 | 0.495732 |  |
| GO:0048489\_synaptic\_vesicle\_transport | 22 | 1 | 3.949400 | -0.647272 | 628 | 209.320309 | 311.32 | 413.319691 | 0.495732 |  |
| GO:0009890\_negative\_regulation\_of\_biosynthetic\_process | 284 | 5 | 1.529697 | -0.644915 | 629 | 210.515313 | 313.0 | 415.484687 | 0.497615 |  |
| GO:0010467\_gene\_expression | 905 | 13 | 1.248098 | -0.639496 | 630 | 211.104538 | 313.76 | 416.415462 | 0.498032 |  |
| GO:0002204\_somatic\_recombination\_of\_immunoglobulin\_genes\_during\_immune\_response | 23 | 1 | 3.777687 | -0.630335 | 635 | 215.906043 | 320.27 | 424.633957 | 0.504362 |  |
| GO:0002208\_somatic\_diversification\_of\_immunoglobulins\_during\_immune\_response | 23 | 1 | 3.777687 | -0.630335 | 635 | 215.906043 | 320.27 | 424.633957 | 0.504362 |  |
| GO:0007584\_response\_to\_nutrient | 23 | 1 | 3.777687 | -0.630335 | 635 | 215.906043 | 320.27 | 424.633957 | 0.504362 |  |
| GO:0009954\_proximal\_distal\_pattern\_formation | 23 | 1 | 3.777687 | -0.630335 | 635 | 215.906043 | 320.27 | 424.633957 | 0.504362 |  |
| GO:0045190\_isotype\_switching | 23 | 1 | 3.777687 | -0.630335 | 635 | 215.906043 | 320.27 | 424.633957 | 0.504362 |  |
| GO:0009059\_macromolecule\_biosynthetic\_process | 910 | 13 | 1.241240 | -0.627743 | 636 | 216.692466 | 321.39 | 426.087534 | 0.505330 |  |
| GO:0003008\_system\_process | 516 | 8 | 1.347082 | -0.624033 | 637 | 217.208199 | 322.08 | 426.951801 | 0.505620 |  |
| GO:0040007\_growth | 217 | 4 | 1.601600 | -0.622469 | 638 | 217.489125 | 322.42 | 427.350875 | 0.505361 |  |
| GO:0045892\_negative\_regulation\_of\_transcription\_\_DNA-dependent | 218 | 4 | 1.594253 | -0.617913 | 639 | 218.306195 | 323.45 | 428.593805 | 0.506182 |  |
| GO:0043085\_positive\_regulation\_of\_catalytic\_activity | 148 | 3 | 1.761219 | -0.616483 | 640 | 218.551942 | 323.94 | 429.328058 | 0.506156 |  |
| GO:0001541\_ovarian\_follicle\_development | 24 | 1 | 3.620283 | -0.614216 | 648 | 223.481657 | 330.36 | 437.238343 | 0.509815 |  |
| GO:0002381\_immunoglobulin\_production\_during\_immune\_response | 24 | 1 | 3.620283 | -0.614216 | 648 | 223.481657 | 330.36 | 437.238343 | 0.509815 |  |
| GO:0042158\_lipoprotein\_biosynthetic\_process | 24 | 1 | 3.620283 | -0.614216 | 648 | 223.481657 | 330.36 | 437.238343 | 0.509815 |  |
| GO:0042632\_cholesterol\_homeostasis | 24 | 1 | 3.620283 | -0.614216 | 648 | 223.481657 | 330.36 | 437.238343 | 0.509815 |  |
| GO:0043410\_positive\_regulation\_of\_MAPKKK\_cascade | 24 | 1 | 3.620283 | -0.614216 | 648 | 223.481657 | 330.36 | 437.238343 | 0.509815 |  |
| GO:0050679\_positive\_regulation\_of\_epithelial\_cell\_proliferation | 24 | 1 | 3.620283 | -0.614216 | 648 | 223.481657 | 330.36 | 437.238343 | 0.509815 |  |
| GO:0055092\_sterol\_homeostasis | 24 | 1 | 3.620283 | -0.614216 | 648 | 223.481657 | 330.36 | 437.238343 | 0.509815 |  |
| GO:0060113\_inner\_ear\_receptor\_cell\_differentiation | 24 | 1 | 3.620283 | -0.614216 | 648 | 223.481657 | 330.36 | 437.238343 | 0.509815 |  |
| GO:0008202\_steroid\_metabolic\_process | 82 | 2 | 2.119190 | -0.614034 | 650 | 224.185319 | 331.34 | 438.494681 | 0.509754 |  |
| GO:0010627\_regulation\_of\_protein\_kinase\_cascade | 82 | 2 | 2.119190 | -0.614034 | 650 | 224.185319 | 331.34 | 438.494681 | 0.509754 |  |
| GO:0051253\_negative\_regulation\_of\_RNA\_metabolic\_process | 220 | 4 | 1.579760 | -0.608906 | 651 | 224.583268 | 332.05 | 439.516732 | 0.510061 |  |
| GO:0001701\_in\_utero\_embryonic\_development | 221 | 4 | 1.572612 | -0.604455 | 652 | 225.817613 | 333.64 | 441.462387 | 0.511718 |  |
| GO:0006139\_nucleobase\_\_nucleoside\_\_nucleotide\_and\_nucleic\_acid\_metabolic\_process | 1002 | 14 | 1.213987 | -0.602938 | 653 | 225.939057 | 333.77 | 441.600943 | 0.511133 |  |
| GO:0002520\_immune\_system\_development | 295 | 5 | 1.472657 | -0.601478 | 655 | 226.370191 | 334.28 | 442.189809 | 0.510351 |  |
| GO:0045595\_regulation\_of\_cell\_differentiation | 295 | 5 | 1.472657 | -0.601478 | 655 | 226.370191 | 334.28 | 442.189809 | 0.510351 |  |
| GO:0002700\_regulation\_of\_production\_of\_molecular\_mediator\_of\_immune\_response | 25 | 1 | 3.475472 | -0.598847 | 664 | 231.445461 | 340.79 | 450.134539 | 0.513238 |  |
| GO:0006302\_double-strand\_break\_repair | 25 | 1 | 3.475472 | -0.598847 | 664 | 231.445461 | 340.79 | 450.134539 | 0.513238 |  |
| GO:0006775\_fat-soluble\_vitamin\_metabolic\_process | 25 | 1 | 3.475472 | -0.598847 | 664 | 231.445461 | 340.79 | 450.134539 | 0.513238 |  |
| GO:0007492\_endoderm\_development | 25 | 1 | 3.475472 | -0.598847 | 664 | 231.445461 | 340.79 | 450.134539 | 0.513238 |  |
| GO:0021983\_pituitary\_gland\_development | 25 | 1 | 3.475472 | -0.598847 | 664 | 231.445461 | 340.79 | 450.134539 | 0.513238 |  |
| GO:0031400\_negative\_regulation\_of\_protein\_modification\_process | 25 | 1 | 3.475472 | -0.598847 | 664 | 231.445461 | 340.79 | 450.134539 | 0.513238 |  |
| GO:0035137\_hindlimb\_morphogenesis | 25 | 1 | 3.475472 | -0.598847 | 664 | 231.445461 | 340.79 | 450.134539 | 0.513238 |  |
| GO:0060603\_mammary\_gland\_duct\_morphogenesis | 25 | 1 | 3.475472 | -0.598847 | 664 | 231.445461 | 340.79 | 450.134539 | 0.513238 |  |
| GO:0060688\_regulation\_of\_morphogenesis\_of\_a\_branching\_structure | 25 | 1 | 3.475472 | -0.598847 | 664 | 231.445461 | 340.79 | 450.134539 | 0.513238 |  |
| GO:0032504\_multicellular\_organism\_reproduction | 86 | 2 | 2.020623 | -0.584403 | 667 | 233.387575 | 343.57 | 453.752425 | 0.515097 |  |
| GO:0034641\_cellular\_nitrogen\_compound\_metabolic\_process | 86 | 2 | 2.020623 | -0.584403 | 667 | 233.387575 | 343.57 | 453.752425 | 0.515097 |  |
| GO:0048609\_reproductive\_process\_in\_a\_multicellular\_organism | 86 | 2 | 2.020623 | -0.584403 | 667 | 233.387575 | 343.57 | 453.752425 | 0.515097 |  |
| GO:0001658\_branching\_involved\_in\_ureteric\_bud\_morphogenesis | 26 | 1 | 3.341800 | -0.584169 | 676 | 237.922791 | 349.07 | 460.217209 | 0.516376 |  |
| GO:0006720\_isoprenoid\_metabolic\_process | 26 | 1 | 3.341800 | -0.584169 | 676 | 237.922791 | 349.07 | 460.217209 | 0.516376 |  |
| GO:0009880\_embryonic\_pattern\_specification | 26 | 1 | 3.341800 | -0.584169 | 676 | 237.922791 | 349.07 | 460.217209 | 0.516376 |  |
| GO:0010212\_response\_to\_ionizing\_radiation | 26 | 1 | 3.341800 | -0.584169 | 676 | 237.922791 | 349.07 | 460.217209 | 0.516376 |  |
| GO:0045665\_negative\_regulation\_of\_neuron\_differentiation | 26 | 1 | 3.341800 | -0.584169 | 676 | 237.922791 | 349.07 | 460.217209 | 0.516376 |  |
| GO:0048645\_organ\_formation | 26 | 1 | 3.341800 | -0.584169 | 676 | 237.922791 | 349.07 | 460.217209 | 0.516376 |  |
| GO:0050680\_negative\_regulation\_of\_epithelial\_cell\_proliferation | 26 | 1 | 3.341800 | -0.584169 | 676 | 237.922791 | 349.07 | 460.217209 | 0.516376 |  |
| GO:0051235\_maintenance\_of\_location | 26 | 1 | 3.341800 | -0.584169 | 676 | 237.922791 | 349.07 | 460.217209 | 0.516376 |  |
| GO:0060675\_ureteric\_bud\_morphogenesis | 26 | 1 | 3.341800 | -0.584169 | 676 | 237.922791 | 349.07 | 460.217209 | 0.516376 |  |
| GO:0009058\_biosynthetic\_process | 1175 | 16 | 1.183139 | -0.583979 | 677 | 238.091841 | 349.27 | 460.448159 | 0.515908 |  |
| GO:0043583\_ear\_development | 87 | 2 | 1.997398 | -0.577292 | 678 | 239.491004 | 351.18 | 462.868996 | 0.517965 |  |
| GO:0019953\_sexual\_reproduction | 228 | 4 | 1.524330 | -0.574245 | 679 | 240.012501 | 351.82 | 463.627499 | 0.518144 |  |
| GO:0002761\_regulation\_of\_myeloid\_leukocyte\_differentiation | 27 | 1 | 3.218029 | -0.570129 | 683 | 244.099372 | 356.79 | 469.480628 | 0.522387 |  |
| GO:0006479\_protein\_amino\_acid\_methylation | 27 | 1 | 3.218029 | -0.570129 | 683 | 244.099372 | 356.79 | 469.480628 | 0.522387 |  |
| GO:0008213\_protein\_amino\_acid\_alkylation | 27 | 1 | 3.218029 | -0.570129 | 683 | 244.099372 | 356.79 | 469.480628 | 0.522387 |  |
| GO:0016050\_vesicle\_organization | 27 | 1 | 3.218029 | -0.570129 | 683 | 244.099372 | 356.79 | 469.480628 | 0.522387 |  |
| GO:0032989\_cellular\_component\_morphogenesis | 307 | 5 | 1.415094 | -0.557416 | 684 | 245.822565 | 359.05 | 472.277435 | 0.524927 |  |
| GO:0002062\_chondrocyte\_differentiation | 28 | 1 | 3.103100 | -0.556682 | 689 | 249.228916 | 363.3 | 477.371084 | 0.527286 |  |
| GO:0002088\_lens\_development\_in\_camera-type\_eye | 28 | 1 | 3.103100 | -0.556682 | 689 | 249.228916 | 363.3 | 477.371084 | 0.527286 |  |
| GO:0021549\_cerebellum\_development | 28 | 1 | 3.103100 | -0.556682 | 689 | 249.228916 | 363.3 | 477.371084 | 0.527286 |  |
| GO:0030111\_regulation\_of\_Wnt\_receptor\_signaling\_pathway | 28 | 1 | 3.103100 | -0.556682 | 689 | 249.228916 | 363.3 | 477.371084 | 0.527286 |  |
| GO:0046328\_regulation\_of\_JNK\_cascade | 28 | 1 | 3.103100 | -0.556682 | 689 | 249.228916 | 363.3 | 477.371084 | 0.527286 |  |
| GO:0051094\_positive\_regulation\_of\_developmental\_process | 308 | 5 | 1.410500 | -0.553892 | 690 | 250.047517 | 364.36 | 478.672483 | 0.528058 |  |
| GO:0051128\_regulation\_of\_cellular\_component\_organization | 160 | 3 | 1.629127 | -0.553760 | 691 | 250.259530 | 364.59 | 478.920470 | 0.527627 |  |
| GO:0002521\_leukocyte\_differentiation | 161 | 3 | 1.619009 | -0.548870 | 692 | 251.357224 | 366.23 | 481.102776 | 0.529234 |  |
| GO:0001934\_positive\_regulation\_of\_protein\_amino\_acid\_phosphorylation | 29 | 1 | 2.996096 | -0.543784 | 698 | 256.706134 | 372.27 | 487.833866 | 0.533338 |  |
| GO:0016447\_somatic\_recombination\_of\_immunoglobulin\_gene\_segments | 29 | 1 | 2.996096 | -0.543784 | 698 | 256.706134 | 372.27 | 487.833866 | 0.533338 |  |
| GO:0042490\_mechanoreceptor\_differentiation | 29 | 1 | 2.996096 | -0.543784 | 698 | 256.706134 | 372.27 | 487.833866 | 0.533338 |  |
| GO:0044087\_regulation\_of\_cellular\_component\_biogenesis | 29 | 1 | 2.996096 | -0.543784 | 698 | 256.706134 | 372.27 | 487.833866 | 0.533338 |  |
| GO:0060041\_retina\_development\_in\_camera-type\_eye | 29 | 1 | 2.996096 | -0.543784 | 698 | 256.706134 | 372.27 | 487.833866 | 0.533338 |  |
| GO:0070302\_regulation\_of\_stress-activated\_protein\_kinase\_signaling\_pathway | 29 | 1 | 2.996096 | -0.543784 | 698 | 256.706134 | 372.27 | 487.833866 | 0.533338 |  |
| GO:0042110\_T\_cell\_activation | 163 | 3 | 1.599143 | -0.539237 | 699 | 257.479849 | 373.41 | 489.340151 | 0.534206 |  |
| GO:0007049\_cell\_cycle | 238 | 4 | 1.460282 | -0.533808 | 700 | 258.767516 | 375.11 | 491.452484 | 0.535871 |  |
| GO:0006950\_response\_to\_stress | 549 | 8 | 1.266110 | -0.532265 | 701 | 258.873385 | 375.21 | 491.546615 | 0.535250 |  |
| GO:0002260\_lymphocyte\_homeostasis | 30 | 1 | 2.896226 | -0.531399 | 707 | 264.235534 | 381.22 | 498.204466 | 0.539208 |  |
| GO:0007219\_Notch\_signaling\_pathway | 30 | 1 | 2.896226 | -0.531399 | 707 | 264.235534 | 381.22 | 498.204466 | 0.539208 |  |
| GO:0009266\_response\_to\_temperature\_stimulus | 30 | 1 | 2.896226 | -0.531399 | 707 | 264.235534 | 381.22 | 498.204466 | 0.539208 |  |
| GO:0016445\_somatic\_diversification\_of\_immunoglobulins | 30 | 1 | 2.896226 | -0.531399 | 707 | 264.235534 | 381.22 | 498.204466 | 0.539208 |  |
| GO:0022411\_cellular\_component\_disassembly | 30 | 1 | 2.896226 | -0.531399 | 707 | 264.235534 | 381.22 | 498.204466 | 0.539208 |  |
| GO:0060740\_prostate\_gland\_epithelium\_morphogenesis | 30 | 1 | 2.896226 | -0.531399 | 707 | 264.235534 | 381.22 | 498.204466 | 0.539208 |  |
| GO:0006753\_nucleoside\_phosphate\_metabolic\_process | 94 | 2 | 1.848655 | -0.530540 | 709 | 265.269214 | 382.42 | 499.570786 | 0.539379 |  |
| GO:0009117\_nucleotide\_metabolic\_process | 94 | 2 | 1.848655 | -0.530540 | 709 | 265.269214 | 382.42 | 499.570786 | 0.539379 |  |
| GO:0006694\_steroid\_biosynthetic\_process | 31 | 1 | 2.802800 | -0.519492 | 717 | 273.893572 | 392.58 | 511.266428 | 0.547531 |  |
| GO:0006939\_smooth\_muscle\_contraction | 31 | 1 | 2.802800 | -0.519492 | 717 | 273.893572 | 392.58 | 511.266428 | 0.547531 |  |
| GO:0010562\_positive\_regulation\_of\_phosphorus\_metabolic\_process | 31 | 1 | 2.802800 | -0.519492 | 717 | 273.893572 | 392.58 | 511.266428 | 0.547531 |  |
| GO:0042157\_lipoprotein\_metabolic\_process | 31 | 1 | 2.802800 | -0.519492 | 717 | 273.893572 | 392.58 | 511.266428 | 0.547531 |  |
| GO:0042327\_positive\_regulation\_of\_phosphorylation | 31 | 1 | 2.802800 | -0.519492 | 717 | 273.893572 | 392.58 | 511.266428 | 0.547531 |  |
| GO:0045937\_positive\_regulation\_of\_phosphate\_metabolic\_process | 31 | 1 | 2.802800 | -0.519492 | 717 | 273.893572 | 392.58 | 511.266428 | 0.547531 |  |
| GO:0055088\_lipid\_homeostasis | 31 | 1 | 2.802800 | -0.519492 | 717 | 273.893572 | 392.58 | 511.266428 | 0.547531 |  |
| GO:0060512\_prostate\_gland\_morphogenesis | 31 | 1 | 2.802800 | -0.519492 | 717 | 273.893572 | 392.58 | 511.266428 | 0.547531 |  |
| GO:0018193\_peptidyl-amino\_acid\_modification | 97 | 2 | 1.791480 | -0.511995 | 718 | 275.308405 | 394.4 | 513.491595 | 0.549304 |  |
| GO:0001707\_mesoderm\_formation | 32 | 1 | 2.715212 | -0.508033 | 721 | 279.233381 | 398.72 | 518.206619 | 0.553010 |  |
| GO:0002274\_myeloid\_leukocyte\_activation | 32 | 1 | 2.715212 | -0.508033 | 721 | 279.233381 | 398.72 | 518.206619 | 0.553010 |  |
| GO:0048332\_mesoderm\_morphogenesis | 32 | 1 | 2.715212 | -0.508033 | 721 | 279.233381 | 398.72 | 518.206619 | 0.553010 |  |
| GO:0007398\_ectoderm\_development | 99 | 2 | 1.755289 | -0.500086 | 722 | 281.072854 | 400.96 | 520.847146 | 0.555346 |  |
| GO:0009611\_response\_to\_wounding | 172 | 3 | 1.515467 | -0.498169 | 723 | 281.427098 | 401.35 | 521.272902 | 0.555118 |  |
| GO:0001843\_neural\_tube\_closure | 33 | 1 | 2.632933 | -0.496993 | 731 | 284.620651 | 405.36 | 526.099349 | 0.554528 |  |
| GO:0002562\_somatic\_diversification\_of\_immune\_receptors\_via\_germline\_recombination\_within\_a\_single\_locus | 33 | 1 | 2.632933 | -0.496993 | 731 | 284.620651 | 405.36 | 526.099349 | 0.554528 |  |
| GO:0007565\_female\_pregnancy | 33 | 1 | 2.632933 | -0.496993 | 731 | 284.620651 | 405.36 | 526.099349 | 0.554528 |  |
| GO:0016444\_somatic\_cell\_DNA\_recombination | 33 | 1 | 2.632933 | -0.496993 | 731 | 284.620651 | 405.36 | 526.099349 | 0.554528 |  |
| GO:0021536\_diencephalon\_development | 33 | 1 | 2.632933 | -0.496993 | 731 | 284.620651 | 405.36 | 526.099349 | 0.554528 |  |
| GO:0021987\_cerebral\_cortex\_development | 33 | 1 | 2.632933 | -0.496993 | 731 | 284.620651 | 405.36 | 526.099349 | 0.554528 |  |
| GO:0022037\_metencephalon\_development | 33 | 1 | 2.632933 | -0.496993 | 731 | 284.620651 | 405.36 | 526.099349 | 0.554528 |  |
| GO:0060606\_tube\_closure | 33 | 1 | 2.632933 | -0.496993 | 731 | 284.620651 | 405.36 | 526.099349 | 0.554528 |  |
| GO:0045321\_leukocyte\_activation | 248 | 4 | 1.401400 | -0.496316 | 732 | 284.904196 | 405.77 | 526.635804 | 0.554331 |  |
| GO:0044093\_positive\_regulation\_of\_molecular\_function | 173 | 3 | 1.506707 | -0.493824 | 733 | 285.454228 | 406.53 | 527.605772 | 0.554611 |  |
| GO:0002200\_somatic\_diversification\_of\_immune\_receptors | 34 | 1 | 2.555494 | -0.486349 | 740 | 290.389503 | 412.34 | 534.290497 | 0.557216 |  |
| GO:0007338\_single\_fertilization | 34 | 1 | 2.555494 | -0.486349 | 740 | 290.389503 | 412.34 | 534.290497 | 0.557216 |  |
| GO:0030509\_BMP\_signaling\_pathway | 34 | 1 | 2.555494 | -0.486349 | 740 | 290.389503 | 412.34 | 534.290497 | 0.557216 |  |
| GO:0045927\_positive\_regulation\_of\_growth | 34 | 1 | 2.555494 | -0.486349 | 740 | 290.389503 | 412.34 | 534.290497 | 0.557216 |  |
| GO:0051047\_positive\_regulation\_of\_secretion | 34 | 1 | 2.555494 | -0.486349 | 740 | 290.389503 | 412.34 | 534.290497 | 0.557216 |  |
| GO:0060443\_mammary\_gland\_morphogenesis | 34 | 1 | 2.555494 | -0.486349 | 740 | 290.389503 | 412.34 | 534.290497 | 0.557216 |  |
| GO:0060711\_labyrinthine\_layer\_development | 34 | 1 | 2.555494 | -0.486349 | 740 | 290.389503 | 412.34 | 534.290497 | 0.557216 |  |
| GO:0042221\_response\_to\_chemical\_stimulus | 409 | 6 | 1.274623 | -0.481663 | 741 | 292.125551 | 414.3 | 536.474449 | 0.559109 |  |
| GO:0016051\_carbohydrate\_biosynthetic\_process | 35 | 1 | 2.482480 | -0.476076 | 744 | 298.179488 | 421.25 | 544.320512 | 0.566196 |  |
| GO:0043406\_positive\_regulation\_of\_MAP\_kinase\_activity | 35 | 1 | 2.482480 | -0.476076 | 744 | 298.179488 | 421.25 | 544.320512 | 0.566196 |  |
| GO:0044242\_cellular\_lipid\_catabolic\_process | 35 | 1 | 2.482480 | -0.476076 | 744 | 298.179488 | 421.25 | 544.320512 | 0.566196 |  |
| GO:0044249\_cellular\_biosynthetic\_process | 1150 | 15 | 1.133306 | -0.474377 | 745 | 298.475176 | 421.69 | 544.904824 | 0.566027 |  |
| GO:0055086\_nucleobase\_\_nucleoside\_and\_nucleotide\_metabolic\_process | 104 | 2 | 1.670900 | -0.471796 | 746 | 299.051516 | 422.4 | 545.748484 | 0.566220 |  |
| GO:0009966\_regulation\_of\_signal\_transduction | 256 | 4 | 1.357606 | -0.468273 | 747 | 300.065543 | 423.67 | 547.274457 | 0.567162 |  |
| GO:0048872\_homeostasis\_of\_number\_of\_cells | 105 | 2 | 1.654987 | -0.466379 | 748 | 300.330837 | 424.01 | 547.689163 | 0.566858 |  |
| GO:0014020\_primary\_neural\_tube\_formation | 36 | 1 | 2.413522 | -0.466153 | 751 | 304.985852 | 429.24 | 553.494148 | 0.571558 |  |
| GO:0022602\_ovulation\_cycle\_process | 36 | 1 | 2.413522 | -0.466153 | 751 | 304.985852 | 429.24 | 553.494148 | 0.571558 |  |
| GO:0051223\_regulation\_of\_protein\_transport | 36 | 1 | 2.413522 | -0.466153 | 751 | 304.985852 | 429.24 | 553.494148 | 0.571558 |  |
| GO:0042698\_ovulation\_cycle | 37 | 1 | 2.348292 | -0.456561 | 752 | 309.369359 | 434.28 | 559.190641 | 0.577500 |  |
| GO:0045859\_regulation\_of\_protein\_kinase\_activity | 107 | 2 | 1.624052 | -0.455772 | 753 | 309.893697 | 434.9 | 559.906303 | 0.577556 |  |
| GO:0030099\_myeloid\_cell\_differentiation | 108 | 2 | 1.609015 | -0.450579 | 755 | 311.381867 | 436.66 | 561.938133 | 0.578358 |  |
| GO:0051240\_positive\_regulation\_of\_multicellular\_organismal\_process | 108 | 2 | 1.609015 | -0.450579 | 755 | 311.381867 | 436.66 | 561.938133 | 0.578358 |  |
| GO:0016192\_vesicle-mediated\_transport | 184 | 3 | 1.416632 | -0.448690 | 756 | 311.796414 | 437.06 | 562.323586 | 0.578122 |  |
| GO:0001570\_vasculogenesis | 38 | 1 | 2.286495 | -0.447282 | 764 | 317.429102 | 443.21 | 568.990898 | 0.580118 |  |
| GO:0007596\_blood\_coagulation | 38 | 1 | 2.286495 | -0.447282 | 764 | 317.429102 | 443.21 | 568.990898 | 0.580118 |  |
| GO:0016042\_lipid\_catabolic\_process | 38 | 1 | 2.286495 | -0.447282 | 764 | 317.429102 | 443.21 | 568.990898 | 0.580118 |  |
| GO:0031401\_positive\_regulation\_of\_protein\_modification\_process | 38 | 1 | 2.286495 | -0.447282 | 764 | 317.429102 | 443.21 | 568.990898 | 0.580118 |  |
| GO:0032259\_methylation | 38 | 1 | 2.286495 | -0.447282 | 764 | 317.429102 | 443.21 | 568.990898 | 0.580118 |  |
| GO:0042493\_response\_to\_drug | 38 | 1 | 2.286495 | -0.447282 | 764 | 317.429102 | 443.21 | 568.990898 | 0.580118 |  |
| GO:0043414\_biopolymer\_methylation | 38 | 1 | 2.286495 | -0.447282 | 764 | 317.429102 | 443.21 | 568.990898 | 0.580118 |  |
| GO:0046777\_protein\_amino\_acid\_autophosphorylation | 38 | 1 | 2.286495 | -0.447282 | 764 | 317.429102 | 443.21 | 568.990898 | 0.580118 |  |
| GO:0030030\_cell\_projection\_organization | 263 | 4 | 1.321472 | -0.445058 | 765 | 318.110107 | 443.99 | 569.869893 | 0.580379 |  |
| GO:0007010\_cytoskeleton\_organization | 185 | 3 | 1.408975 | -0.444815 | 766 | 318.329248 | 444.24 | 570.150752 | 0.579948 |  |
| GO:0002376\_immune\_system\_process | 505 | 7 | 1.204371 | -0.442160 | 767 | 318.610236 | 444.53 | 570.449764 | 0.579570 |  |
| GO:0006730\_one-carbon\_metabolic\_process | 39 | 1 | 2.227866 | -0.438300 | 775 | 324.581067 | 450.96 | 577.338933 | 0.581884 |  |
| GO:0007160\_cell-matrix\_adhesion | 39 | 1 | 2.227866 | -0.438300 | 775 | 324.581067 | 450.96 | 577.338933 | 0.581884 |  |
| GO:0007286\_spermatid\_development | 39 | 1 | 2.227866 | -0.438300 | 775 | 324.581067 | 450.96 | 577.338933 | 0.581884 |  |
| GO:0008037\_cell\_recognition | 39 | 1 | 2.227866 | -0.438300 | 775 | 324.581067 | 450.96 | 577.338933 | 0.581884 |  |
| GO:0035148\_tube\_lumen\_formation | 39 | 1 | 2.227866 | -0.438300 | 775 | 324.581067 | 450.96 | 577.338933 | 0.581884 |  |
| GO:0043524\_negative\_regulation\_of\_neuron\_apoptosis | 39 | 1 | 2.227866 | -0.438300 | 775 | 324.581067 | 450.96 | 577.338933 | 0.581884 |  |
| GO:0048663\_neuron\_fate\_commitment | 39 | 1 | 2.227866 | -0.438300 | 775 | 324.581067 | 450.96 | 577.338933 | 0.581884 |  |
| GO:0070201\_regulation\_of\_establishment\_of\_protein\_localization | 39 | 1 | 2.227866 | -0.438300 | 775 | 324.581067 | 450.96 | 577.338933 | 0.581884 |  |
| GO:0007276\_gamete\_generation | 188 | 3 | 1.386491 | -0.433408 | 776 | 325.747078 | 452.32 | 578.892922 | 0.582887 |  |
| GO:0009987\_cellular\_process | 3868 | 46 | 1.033297 | -0.432032 | 777 | 325.831902 | 452.46 | 579.088098 | 0.582317 |  |
| GO:0001824\_blastocyst\_development | 40 | 1 | 2.172170 | -0.429598 | 782 | 330.341925 | 457.43 | 584.518075 | 0.584949 |  |
| GO:0007599\_hemostasis | 40 | 1 | 2.172170 | -0.429598 | 782 | 330.341925 | 457.43 | 584.518075 | 0.584949 |  |
| GO:0016358\_dendrite\_development | 40 | 1 | 2.172170 | -0.429598 | 782 | 330.341925 | 457.43 | 584.518075 | 0.584949 |  |
| GO:0017015\_regulation\_of\_transforming\_growth\_factor\_beta\_receptor\_signaling\_pathway | 40 | 1 | 2.172170 | -0.429598 | 782 | 330.341925 | 457.43 | 584.518075 | 0.584949 |  |
| GO:0035272\_exocrine\_system\_development | 40 | 1 | 2.172170 | -0.429598 | 782 | 330.341925 | 457.43 | 584.518075 | 0.584949 |  |
| GO:0001776\_leukocyte\_homeostasis | 41 | 1 | 2.119190 | -0.421164 | 786 | 337.347793 | 465.56 | 593.772207 | 0.592316 |  |
| GO:0006979\_response\_to\_oxidative\_stress | 41 | 1 | 2.119190 | -0.421164 | 786 | 337.347793 | 465.56 | 593.772207 | 0.592316 |  |
| GO:0008585\_female\_gonad\_development | 41 | 1 | 2.119190 | -0.421164 | 786 | 337.347793 | 465.56 | 593.772207 | 0.592316 |  |
| GO:0019748\_secondary\_metabolic\_process | 41 | 1 | 2.119190 | -0.421164 | 786 | 337.347793 | 465.56 | 593.772207 | 0.592316 |  |
| GO:0006006\_glucose\_metabolic\_process | 42 | 1 | 2.068733 | -0.412984 | 794 | 344.961319 | 474.25 | 603.538681 | 0.597292 |  |
| GO:0008361\_regulation\_of\_cell\_size | 42 | 1 | 2.068733 | -0.412984 | 794 | 344.961319 | 474.25 | 603.538681 | 0.597292 |  |
| GO:0010740\_positive\_regulation\_of\_protein\_kinase\_cascade | 42 | 1 | 2.068733 | -0.412984 | 794 | 344.961319 | 474.25 | 603.538681 | 0.597292 |  |
| GO:0015672\_monovalent\_inorganic\_cation\_transport | 42 | 1 | 2.068733 | -0.412984 | 794 | 344.961319 | 474.25 | 603.538681 | 0.597292 |  |
| GO:0045637\_regulation\_of\_myeloid\_cell\_differentiation | 42 | 1 | 2.068733 | -0.412984 | 794 | 344.961319 | 474.25 | 603.538681 | 0.597292 |  |
| GO:0048515\_spermatid\_differentiation | 42 | 1 | 2.068733 | -0.412984 | 794 | 344.961319 | 474.25 | 603.538681 | 0.597292 |  |
| GO:0050817\_coagulation | 42 | 1 | 2.068733 | -0.412984 | 794 | 344.961319 | 474.25 | 603.538681 | 0.597292 |  |
| GO:0080135\_regulation\_of\_cellular\_response\_to\_stress | 42 | 1 | 2.068733 | -0.412984 | 794 | 344.961319 | 474.25 | 603.538681 | 0.597292 |  |
| GO:0046483\_heterocycle\_metabolic\_process | 116 | 2 | 1.498048 | -0.411522 | 796 | 345.713457 | 475.0 | 604.286543 | 0.596734 |  |
| GO:0080134\_regulation\_of\_response\_to\_stress | 116 | 2 | 1.498048 | -0.411522 | 796 | 345.713457 | 475.0 | 604.286543 | 0.596734 |  |
| GO:0030182\_neuron\_differentiation | 356 | 5 | 1.220320 | -0.407979 | 797 | 346.862135 | 476.25 | 605.637865 | 0.597553 |  |
| GO:0043933\_macromolecular\_complex\_subunit\_organization | 117 | 2 | 1.485244 | -0.406931 | 798 | 347.087130 | 476.48 | 605.872870 | 0.597093 |  |
| GO:0050896\_response\_to\_stimulus | 1107 | 14 | 1.098839 | -0.405373 | 799 | 347.217121 | 476.66 | 606.102879 | 0.596571 |  |
| GO:0001841\_neural\_tube\_formation | 43 | 1 | 2.020623 | -0.405046 | 805 | 351.444689 | 481.63 | 611.815311 | 0.598298 |  |
| GO:0001894\_tissue\_homeostasis | 43 | 1 | 2.020623 | -0.405046 | 805 | 351.444689 | 481.63 | 611.815311 | 0.598298 |  |
| GO:0006766\_vitamin\_metabolic\_process | 43 | 1 | 2.020623 | -0.405046 | 805 | 351.444689 | 481.63 | 611.815311 | 0.598298 |  |
| GO:0007224\_smoothened\_signaling\_pathway | 43 | 1 | 2.020623 | -0.405046 | 805 | 351.444689 | 481.63 | 611.815311 | 0.598298 |  |
| GO:0032868\_response\_to\_insulin\_stimulus | 43 | 1 | 2.020623 | -0.405046 | 805 | 351.444689 | 481.63 | 611.815311 | 0.598298 |  |
| GO:0051789\_response\_to\_protein\_stimulus | 43 | 1 | 2.020623 | -0.405046 | 805 | 351.444689 | 481.63 | 611.815311 | 0.598298 |  |
| GO:0031175\_neuron\_projection\_development | 197 | 3 | 1.323149 | -0.401030 | 806 | 353.340495 | 483.77 | 614.199505 | 0.600211 |  |
| GO:0001942\_hair\_follicle\_development | 44 | 1 | 1.974700 | -0.397339 | 813 | 359.948641 | 491.47 | 622.991359 | 0.604514 |  |
| GO:0016064\_immunoglobulin\_mediated\_immune\_response | 44 | 1 | 1.974700 | -0.397339 | 813 | 359.948641 | 491.47 | 622.991359 | 0.604514 |  |
| GO:0022404\_molting\_cycle\_process | 44 | 1 | 1.974700 | -0.397339 | 813 | 359.948641 | 491.47 | 622.991359 | 0.604514 |  |
| GO:0022405\_hair\_cycle\_process | 44 | 1 | 1.974700 | -0.397339 | 813 | 359.948641 | 491.47 | 622.991359 | 0.604514 |  |
| GO:0042303\_molting\_cycle | 44 | 1 | 1.974700 | -0.397339 | 813 | 359.948641 | 491.47 | 622.991359 | 0.604514 |  |
| GO:0042633\_hair\_cycle | 44 | 1 | 1.974700 | -0.397339 | 813 | 359.948641 | 491.47 | 622.991359 | 0.604514 |  |
| GO:0050808\_synapse\_organization | 44 | 1 | 1.974700 | -0.397339 | 813 | 359.948641 | 491.47 | 622.991359 | 0.604514 |  |
| GO:0001838\_embryonic\_epithelial\_tube\_formation | 45 | 1 | 1.930818 | -0.389851 | 814 | 363.275937 | 495.11 | 626.944063 | 0.608243 |  |
| GO:0002252\_immune\_effector\_process | 122 | 2 | 1.424374 | -0.384869 | 816 | 366.165918 | 498.7 | 631.234082 | 0.611152 |  |
| GO:0050865\_regulation\_of\_cell\_activation | 122 | 2 | 1.424374 | -0.384869 | 816 | 366.165918 | 498.7 | 631.234082 | 0.611152 |  |
| GO:0008217\_regulation\_of\_blood\_pressure | 46 | 1 | 1.888843 | -0.382574 | 819 | 368.725447 | 501.65 | 634.574553 | 0.612515 |  |
| GO:0019724\_B\_cell\_mediated\_immunity | 46 | 1 | 1.888843 | -0.382574 | 819 | 368.725447 | 501.65 | 634.574553 | 0.612515 |  |
| GO:0030218\_erythrocyte\_differentiation | 46 | 1 | 1.888843 | -0.382574 | 819 | 368.725447 | 501.65 | 634.574553 | 0.612515 |  |
| GO:0006629\_lipid\_metabolic\_process | 285 | 4 | 1.219464 | -0.379345 | 820 | 369.714805 | 502.84 | 635.965195 | 0.613220 |  |
| GO:0031667\_response\_to\_nutrient\_levels | 47 | 1 | 1.848655 | -0.375497 | 823 | 373.826561 | 507.56 | 641.293439 | 0.616719 |  |
| GO:0034754\_cellular\_hormone\_metabolic\_process | 47 | 1 | 1.848655 | -0.375497 | 823 | 373.826561 | 507.56 | 641.293439 | 0.616719 |  |
| GO:0048871\_multicellular\_organismal\_homeostasis | 47 | 1 | 1.848655 | -0.375497 | 823 | 373.826561 | 507.56 | 641.293439 | 0.616719 |  |
| GO:0051641\_cellular\_localization | 370 | 5 | 1.174146 | -0.372886 | 824 | 374.420848 | 508.35 | 642.279152 | 0.616930 |  |
| GO:0007498\_mesoderm\_development | 48 | 1 | 1.810142 | -0.368613 | 827 | 377.199540 | 511.48 | 645.760460 | 0.618476 |  |
| GO:0019318\_hexose\_metabolic\_process | 48 | 1 | 1.810142 | -0.368613 | 827 | 377.199540 | 511.48 | 645.760460 | 0.618476 |  |
| GO:0032269\_negative\_regulation\_of\_cellular\_protein\_metabolic\_process | 48 | 1 | 1.810142 | -0.368613 | 827 | 377.199540 | 511.48 | 645.760460 | 0.618476 |  |
| GO:0008284\_positive\_regulation\_of\_cell\_proliferation | 208 | 3 | 1.253175 | -0.364910 | 828 | 377.600896 | 511.93 | 646.259104 | 0.618273 |  |
| GO:0016043\_cellular\_component\_organization | 964 | 12 | 1.081578 | -0.363675 | 829 | 377.725195 | 512.09 | 646.454805 | 0.617720 |  |
| GO:0006725\_cellular\_aromatic\_compound\_metabolic\_process | 49 | 1 | 1.773200 | -0.361913 | 831 | 381.410746 | 516.42 | 651.429254 | 0.621444 |  |
| GO:0034101\_erythrocyte\_homeostasis | 49 | 1 | 1.773200 | -0.361913 | 831 | 381.410746 | 516.42 | 651.429254 | 0.621444 |  |
| GO:0002573\_myeloid\_leukocyte\_differentiation | 50 | 1 | 1.737736 | -0.355389 | 832 | 385.171217 | 520.67 | 656.168783 | 0.625805 |  |
| GO:0006887\_exocytosis | 51 | 1 | 1.703663 | -0.349035 | 834 | 390.628074 | 526.57 | 662.511926 | 0.631379 |  |
| GO:0032880\_regulation\_of\_protein\_localization | 51 | 1 | 1.703663 | -0.349035 | 834 | 390.628074 | 526.57 | 662.511926 | 0.631379 |  |
| GO:0048583\_regulation\_of\_response\_to\_stimulus | 217 | 3 | 1.201200 | -0.337898 | 835 | 394.596981 | 531.03 | 667.463019 | 0.635964 |  |
| GO:0007283\_spermatogenesis | 134 | 2 | 1.296818 | -0.337388 | 837 | 395.501952 | 531.71 | 667.918048 | 0.635257 |  |
| GO:0048232\_male\_gamete\_generation | 134 | 2 | 1.296818 | -0.337388 | 837 | 395.501952 | 531.71 | 667.918048 | 0.635257 |  |
| GO:0006576\_biogenic\_amine\_metabolic\_process | 53 | 1 | 1.639373 | -0.336809 | 842 | 398.032018 | 534.46 | 670.887982 | 0.634751 |  |
| GO:0006935\_chemotaxis | 53 | 1 | 1.639373 | -0.336809 | 842 | 398.032018 | 534.46 | 670.887982 | 0.634751 |  |
| GO:0030031\_cell\_projection\_assembly | 53 | 1 | 1.639373 | -0.336809 | 842 | 398.032018 | 534.46 | 670.887982 | 0.634751 |  |
| GO:0042330\_taxis | 53 | 1 | 1.639373 | -0.336809 | 842 | 398.032018 | 534.46 | 670.887982 | 0.634751 |  |
| GO:0051248\_negative\_regulation\_of\_protein\_metabolic\_process | 53 | 1 | 1.639373 | -0.336809 | 842 | 398.032018 | 534.46 | 670.887982 | 0.634751 |  |
| GO:0009566\_fertilization | 54 | 1 | 1.609015 | -0.330924 | 844 | 402.564252 | 539.16 | 675.755748 | 0.638815 |  |
| GO:0043405\_regulation\_of\_MAP\_kinase\_activity | 54 | 1 | 1.609015 | -0.330924 | 844 | 402.564252 | 539.16 | 675.755748 | 0.638815 |  |
| GO:0006310\_DNA\_recombination | 55 | 1 | 1.579760 | -0.325183 | 847 | 407.017352 | 543.64 | 680.262648 | 0.641842 |  |
| GO:0043434\_response\_to\_peptide\_hormone\_stimulus | 55 | 1 | 1.579760 | -0.325183 | 847 | 407.017352 | 543.64 | 680.262648 | 0.641842 |  |
| GO:0048704\_embryonic\_skeletal\_system\_morphogenesis | 55 | 1 | 1.579760 | -0.325183 | 847 | 407.017352 | 543.64 | 680.262648 | 0.641842 |  |
| GO:0007169\_transmembrane\_receptor\_protein\_tyrosine\_kinase\_signaling\_pathway | 139 | 2 | 1.250170 | -0.319618 | 849 | 408.112400 | 544.93 | 681.747600 | 0.641849 |  |
| GO:0034613\_cellular\_protein\_localization | 139 | 2 | 1.250170 | -0.319618 | 849 | 408.112400 | 544.93 | 681.747600 | 0.641849 |  |
| GO:0001708\_cell\_fate\_specification | 56 | 1 | 1.551550 | -0.319582 | 851 | 411.085004 | 548.18 | 685.274996 | 0.644160 |  |
| GO:0009187\_cyclic\_nucleotide\_metabolic\_process | 56 | 1 | 1.551550 | -0.319582 | 851 | 411.085004 | 548.18 | 685.274996 | 0.644160 |  |
| GO:0001764\_neuron\_migration | 57 | 1 | 1.524330 | -0.314115 | 856 | 415.987808 | 553.12 | 690.252192 | 0.646168 |  |
| GO:0009953\_dorsal\_ventral\_pattern\_formation | 57 | 1 | 1.524330 | -0.314115 | 856 | 415.987808 | 553.12 | 690.252192 | 0.646168 |  |
| GO:0042472\_inner\_ear\_morphogenesis | 57 | 1 | 1.524330 | -0.314115 | 856 | 415.987808 | 553.12 | 690.252192 | 0.646168 |  |
| GO:0043523\_regulation\_of\_neuron\_apoptosis | 57 | 1 | 1.524330 | -0.314115 | 856 | 415.987808 | 553.12 | 690.252192 | 0.646168 |  |
| GO:0050878\_regulation\_of\_body\_fluid\_levels | 57 | 1 | 1.524330 | -0.314115 | 856 | 415.987808 | 553.12 | 690.252192 | 0.646168 |  |
| GO:0070727\_cellular\_macromolecule\_localization | 141 | 2 | 1.232437 | -0.312809 | 857 | 416.492531 | 553.68 | 690.867469 | 0.646068 |  |
| GO:0030902\_hindbrain\_development | 58 | 1 | 1.498048 | -0.308777 | 858 | 418.089053 | 555.49 | 692.890947 | 0.647424 |  |
| GO:0002682\_regulation\_of\_immune\_system\_process | 228 | 3 | 1.143247 | -0.307671 | 860 | 418.864470 | 556.47 | 694.075530 | 0.647058 |  |
| GO:0046649\_lymphocyte\_activation | 228 | 3 | 1.143247 | -0.307671 | 860 | 418.864470 | 556.47 | 694.075530 | 0.647058 |  |
| GO:0045596\_negative\_regulation\_of\_cell\_differentiation | 144 | 2 | 1.206761 | -0.302900 | 861 | 421.888022 | 559.79 | 697.691978 | 0.650163 |  |
| GO:0009123\_nucleoside\_monophosphate\_metabolic\_process | 60 | 1 | 1.448113 | -0.298472 | 862 | 422.829376 | 560.64 | 698.450624 | 0.650394 |  |
| GO:0050790\_regulation\_of\_catalytic\_activity | 233 | 3 | 1.118714 | -0.294863 | 863 | 424.451722 | 562.43 | 700.408278 | 0.651715 |  |
| GO:0006874\_cellular\_calcium\_ion\_homeostasis | 61 | 1 | 1.424374 | -0.293495 | 866 | 426.353394 | 564.48 | 702.606606 | 0.651824 |  |
| GO:0007005\_mitochondrion\_organization | 61 | 1 | 1.424374 | -0.293495 | 866 | 426.353394 | 564.48 | 702.606606 | 0.651824 |  |
| GO:0009991\_response\_to\_extracellular\_stimulus | 61 | 1 | 1.424374 | -0.293495 | 866 | 426.353394 | 564.48 | 702.606606 | 0.651824 |  |
| GO:0022604\_regulation\_of\_cell\_morphogenesis | 62 | 1 | 1.401400 | -0.288632 | 868 | 430.917477 | 568.95 | 706.982523 | 0.655472 |  |
| GO:0040014\_regulation\_of\_multicellular\_organism\_growth | 62 | 1 | 1.401400 | -0.288632 | 868 | 430.917477 | 568.95 | 706.982523 | 0.655472 |  |
| GO:0032940\_secretion\_by\_cell | 149 | 2 | 1.166266 | -0.287156 | 869 | 431.582010 | 569.56 | 707.537990 | 0.655420 |  |
| GO:0006875\_cellular\_metal\_ion\_homeostasis | 64 | 1 | 1.357606 | -0.279226 | 872 | 437.543890 | 575.42 | 713.296110 | 0.659885 |  |
| GO:0042060\_wound\_healing | 64 | 1 | 1.357606 | -0.279226 | 872 | 437.543890 | 575.42 | 713.296110 | 0.659885 |  |
| GO:0055074\_calcium\_ion\_homeostasis | 64 | 1 | 1.357606 | -0.279226 | 872 | 437.543890 | 575.42 | 713.296110 | 0.659885 |  |
| GO:0042471\_ear\_morphogenesis | 65 | 1 | 1.336720 | -0.274677 | 874 | 439.448016 | 577.35 | 715.251984 | 0.660584 |  |
| GO:0048511\_rhythmic\_process | 65 | 1 | 1.336720 | -0.274677 | 874 | 439.448016 | 577.35 | 715.251984 | 0.660584 |  |
| GO:0010646\_regulation\_of\_cell\_communication | 330 | 4 | 1.053173 | -0.273195 | 875 | 440.260434 | 578.11 | 715.959566 | 0.660697 |  |
| GO:0007268\_synaptic\_transmission | 154 | 2 | 1.128400 | -0.272315 | 876 | 440.592352 | 578.43 | 716.267648 | 0.660308 |  |
| GO:0051402\_neuron\_apoptosis | 66 | 1 | 1.316467 | -0.270227 | 877 | 443.511476 | 581.66 | 719.808524 | 0.663238 |  |
| GO:0009791\_post-embryonic\_development | 67 | 1 | 1.296818 | -0.265873 | 879 | 446.396524 | 584.75 | 723.103476 | 0.665245 |  |
| GO:0042445\_hormone\_metabolic\_process | 67 | 1 | 1.296818 | -0.265873 | 879 | 446.396524 | 584.75 | 723.103476 | 0.665245 |  |
| GO:0002697\_regulation\_of\_immune\_effector\_process | 68 | 1 | 1.277747 | -0.261610 | 880 | 448.326518 | 586.85 | 725.373482 | 0.666875 |  |
| GO:0007409\_axonogenesis | 158 | 2 | 1.099833 | -0.261049 | 881 | 448.993748 | 587.68 | 726.366252 | 0.667060 |  |
| GO:0005996\_monosaccharide\_metabolic\_process | 69 | 1 | 1.259229 | -0.257438 | 884 | 451.608395 | 590.31 | 729.011605 | 0.667771 |  |
| GO:0032101\_regulation\_of\_response\_to\_external\_stimulus | 69 | 1 | 1.259229 | -0.257438 | 884 | 451.608395 | 590.31 | 729.011605 | 0.667771 |  |
| GO:0055065\_metal\_ion\_homeostasis | 69 | 1 | 1.259229 | -0.257438 | 884 | 451.608395 | 590.31 | 729.011605 | 0.667771 |  |
| GO:0007267\_cell-cell\_signaling | 252 | 3 | 1.034367 | -0.250922 | 885 | 456.155042 | 594.8 | 733.444958 | 0.672090 |  |
| GO:0006281\_DNA\_repair | 71 | 1 | 1.223758 | -0.249350 | 886 | 458.252782 | 597.01 | 735.767218 | 0.673826 |  |
| GO:0021915\_neural\_tube\_development | 72 | 1 | 1.206761 | -0.245430 | 888 | 464.332503 | 602.83 | 741.327497 | 0.678863 |  |
| GO:0030879\_mammary\_gland\_development | 72 | 1 | 1.206761 | -0.245430 | 888 | 464.332503 | 602.83 | 741.327497 | 0.678863 |  |
| GO:0006936\_muscle\_contraction | 73 | 1 | 1.190230 | -0.241590 | 890 | 469.004557 | 607.54 | 746.075443 | 0.682629 |  |
| GO:0051336\_regulation\_of\_hydrolase\_activity | 73 | 1 | 1.190230 | -0.241590 | 890 | 469.004557 | 607.54 | 746.075443 | 0.682629 |  |
| GO:0044265\_cellular\_macromolecule\_catabolic\_process | 75 | 1 | 1.158491 | -0.234138 | 892 | 472.835991 | 611.66 | 750.484009 | 0.685717 |  |
| GO:0051050\_positive\_regulation\_of\_transport | 75 | 1 | 1.158491 | -0.234138 | 892 | 472.835991 | 611.66 | 750.484009 | 0.685717 |  |
| GO:0003012\_muscle\_system\_process | 76 | 1 | 1.143247 | -0.230523 | 895 | 474.761225 | 613.73 | 752.698775 | 0.685732 |  |
| GO:0009725\_response\_to\_hormone\_stimulus | 76 | 1 | 1.143247 | -0.230523 | 895 | 474.761225 | 613.73 | 752.698775 | 0.685732 |  |
| GO:0034621\_cellular\_macromolecular\_complex\_subunit\_organization | 76 | 1 | 1.143247 | -0.230523 | 895 | 474.761225 | 613.73 | 752.698775 | 0.685732 |  |
| GO:0048666\_neuron\_development | 262 | 3 | 0.994887 | -0.230498 | 896 | 475.397494 | 614.38 | 753.362506 | 0.685692 |  |
| GO:0048812\_neuron\_projection\_morphogenesis | 170 | 2 | 1.022198 | -0.230189 | 897 | 475.981934 | 614.99 | 753.998066 | 0.685608 |  |
| GO:0051241\_negative\_regulation\_of\_multicellular\_organismal\_process | 77 | 1 | 1.128400 | -0.226978 | 898 | 477.401434 | 616.31 | 755.218566 | 0.686314 |  |
| GO:0044255\_cellular\_lipid\_metabolic\_process | 264 | 3 | 0.987350 | -0.226616 | 899 | 477.630832 | 616.49 | 755.349168 | 0.685751 |  |
| GO:0007600\_sensory\_perception | 172 | 2 | 1.010312 | -0.225440 | 900 | 478.454199 | 617.29 | 756.125801 | 0.685878 |  |
| GO:0048667\_cell\_morphogenesis\_involved\_in\_neuron\_differentiation | 173 | 2 | 1.004472 | -0.223105 | 901 | 482.380235 | 621.06 | 759.739765 | 0.689301 |  |
| GO:0006996\_organelle\_organization | 449 | 5 | 0.967559 | -0.222055 | 902 | 482.943262 | 621.58 | 760.216738 | 0.689113 |  |
| GO:0051046\_regulation\_of\_secretion | 79 | 1 | 1.099833 | -0.220094 | 903 | 483.876392 | 622.52 | 761.163608 | 0.689391 |  |
| GO:0015031\_protein\_transport | 175 | 2 | 0.992992 | -0.218511 | 905 | 484.891062 | 623.36 | 761.828938 | 0.688796 |  |
| GO:0046903\_secretion | 175 | 2 | 0.992992 | -0.218511 | 905 | 484.891062 | 623.36 | 761.828938 | 0.688796 |  |
| GO:0002250\_adaptive\_immune\_response | 80 | 1 | 1.086085 | -0.216751 | 907 | 487.223063 | 625.74 | 764.256937 | 0.689901 |  |
| GO:0002460\_adaptive\_immune\_response\_based\_on\_somatic\_recombination\_of\_immune\_receptors\_built\_from\_immunoglobulin\_superfamily\_domains | 80 | 1 | 1.086085 | -0.216751 | 907 | 487.223063 | 625.74 | 764.256937 | 0.689901 |  |
| GO:0048858\_cell\_projection\_morphogenesis | 176 | 2 | 0.987350 | -0.216253 | 908 | 488.110052 | 626.62 | 765.129948 | 0.690110 |  |
| GO:0007411\_axon\_guidance | 82 | 1 | 1.059595 | -0.210253 | 910 | 492.416238 | 630.85 | 769.283762 | 0.693242 |  |
| GO:0045664\_regulation\_of\_neuron\_differentiation | 82 | 1 | 1.059595 | -0.210253 | 910 | 492.416238 | 630.85 | 769.283762 | 0.693242 |  |
| GO:0045184\_establishment\_of\_protein\_localization | 180 | 2 | 0.965409 | -0.207463 | 911 | 495.244894 | 633.63 | 772.015106 | 0.695532 |  |
| GO:0006575\_cellular\_amino\_acid\_derivative\_metabolic\_process | 83 | 1 | 1.046829 | -0.207095 | 913 | 498.024123 | 636.48 | 774.935877 | 0.697130 |  |
| GO:0050867\_positive\_regulation\_of\_cell\_activation | 83 | 1 | 1.046829 | -0.207095 | 913 | 498.024123 | 636.48 | 774.935877 | 0.697130 |  |
| GO:0030005\_cellular\_di-\_\_tri-valent\_inorganic\_cation\_homeostasis | 84 | 1 | 1.034367 | -0.203996 | 914 | 500.207850 | 638.51 | 776.812150 | 0.698589 |  |
| GO:0002449\_lymphocyte\_mediated\_immunity | 85 | 1 | 1.022198 | -0.200954 | 915 | 502.912670 | 641.19 | 779.467330 | 0.700754 |  |
| GO:0065009\_regulation\_of\_molecular\_function | 279 | 3 | 0.934267 | -0.199488 | 916 | 503.660688 | 641.94 | 780.219312 | 0.700808 |  |
| GO:0032990\_cell\_part\_morphogenesis | 184 | 2 | 0.944422 | -0.199051 | 917 | 504.211129 | 642.53 | 780.848871 | 0.700687 |  |
| GO:0022612\_gland\_morphogenesis | 87 | 1 | 0.998699 | -0.195037 | 918 | 510.031935 | 648.21 | 786.388065 | 0.706111 |  |
| GO:0019226\_transmission\_of\_nerve\_impulse | 189 | 2 | 0.919437 | -0.189038 | 919 | 515.115925 | 653.03 | 790.944075 | 0.710588 |  |
| GO:0030003\_cellular\_cation\_homeostasis | 90 | 1 | 0.965409 | -0.186555 | 922 | 517.133861 | 654.91 | 792.686139 | 0.710315 |  |
| GO:0035264\_multicellular\_organism\_growth | 90 | 1 | 0.965409 | -0.186555 | 922 | 517.133861 | 654.91 | 792.686139 | 0.710315 |  |
| GO:0042113\_B\_cell\_activation | 90 | 1 | 0.965409 | -0.186555 | 922 | 517.133861 | 654.91 | 792.686139 | 0.710315 |  |
| GO:0002443\_leukocyte\_mediated\_immunity | 91 | 1 | 0.954800 | -0.183829 | 924 | 518.539262 | 656.36 | 794.180738 | 0.710346 |  |
| GO:0008544\_epidermis\_development | 91 | 1 | 0.954800 | -0.183829 | 924 | 518.539262 | 656.36 | 794.180738 | 0.710346 |  |
| GO:0009719\_response\_to\_endogenous\_stimulus | 92 | 1 | 0.944422 | -0.181150 | 925 | 520.076174 | 657.91 | 795.743826 | 0.711254 |  |
| GO:0055066\_di-\_\_tri-valent\_inorganic\_cation\_homeostasis | 93 | 1 | 0.934267 | -0.178518 | 926 | 522.474818 | 660.27 | 798.065182 | 0.713035 |  |
| GO:0008610\_lipid\_biosynthetic\_process | 94 | 1 | 0.924328 | -0.175932 | 928 | 526.233165 | 663.77 | 801.306835 | 0.715269 |  |
| GO:0034984\_cellular\_response\_to\_DNA\_damage\_stimulus | 94 | 1 | 0.924328 | -0.175932 | 928 | 526.233165 | 663.77 | 801.306835 | 0.715269 |  |
| GO:0050877\_neurological\_system\_process | 390 | 4 | 0.891147 | -0.174975 | 929 | 526.693404 | 664.21 | 801.726596 | 0.714973 |  |
| GO:0006954\_inflammatory\_response | 96 | 1 | 0.905071 | -0.170894 | 931 | 532.024267 | 669.19 | 806.355733 | 0.718786 |  |
| GO:0060249\_anatomical\_structure\_homeostasis | 96 | 1 | 0.905071 | -0.170894 | 931 | 532.024267 | 669.19 | 806.355733 | 0.718786 |  |
| GO:0060341\_regulation\_of\_cellular\_localization | 97 | 1 | 0.895740 | -0.168439 | 932 | 533.349198 | 670.37 | 807.390802 | 0.719281 |  |
| GO:0022607\_cellular\_component\_assembly | 204 | 2 | 0.851831 | -0.162033 | 933 | 538.815104 | 675.55 | 812.284896 | 0.724062 |  |
| GO:0001525\_angiogenesis | 100 | 1 | 0.868868 | -0.161324 | 934 | 539.161675 | 676.0 | 812.838325 | 0.723769 |  |
| GO:0006955\_immune\_response | 205 | 2 | 0.847676 | -0.160381 | 935 | 539.983416 | 676.7 | 813.416584 | 0.723743 |  |
| GO:0010817\_regulation\_of\_hormone\_levels | 106 | 1 | 0.819687 | -0.148128 | 936 | 547.111407 | 683.14 | 819.168593 | 0.729850 |  |
| GO:0010033\_response\_to\_organic\_substance | 216 | 2 | 0.804507 | -0.143313 | 937 | 548.849504 | 684.69 | 820.530496 | 0.730726 |  |
| GO:0042592\_homeostatic\_process | 419 | 4 | 0.829468 | -0.140403 | 938 | 550.271191 | 685.96 | 821.648809 | 0.731301 |  |
| GO:0055080\_cation\_homeostasis | 110 | 1 | 0.789880 | -0.140029 | 939 | 551.824358 | 687.44 | 823.055642 | 0.732098 |  |
| GO:0006974\_response\_to\_DNA\_damage\_stimulus | 113 | 1 | 0.768910 | -0.134289 | 940 | 555.506008 | 690.61 | 825.713992 | 0.734691 |  |
| GO:0006810\_transport | 718 | 7 | 0.847086 | -0.130100 | 941 | 558.821946 | 693.49 | 828.158054 | 0.736971 |  |
| GO:0006519\_cellular\_amino\_acid\_and\_derivative\_metabolic\_process | 118 | 1 | 0.736329 | -0.125312 | 942 | 565.258625 | 699.14 | 833.021375 | 0.742187 |  |
| GO:0051234\_establishment\_of\_localization | 729 | 7 | 0.834304 | -0.121428 | 943 | 566.924183 | 700.56 | 834.195817 | 0.742906 |  |
| GO:0050890\_cognition | 233 | 2 | 0.745809 | -0.120493 | 944 | 567.418509 | 700.93 | 834.441491 | 0.742511 |  |
| GO:0002694\_regulation\_of\_leukocyte\_activation | 121 | 1 | 0.718073 | -0.120253 | 947 | 569.516060 | 702.86 | 836.203940 | 0.742196 |  |
| GO:0006917\_induction\_of\_apoptosis | 121 | 1 | 0.718073 | -0.120253 | 947 | 569.516060 | 702.86 | 836.203940 | 0.742196 |  |
| GO:0012502\_induction\_of\_programmed\_cell\_death | 121 | 1 | 0.718073 | -0.120253 | 947 | 569.516060 | 702.86 | 836.203940 | 0.742196 |  |
| GO:0044085\_cellular\_component\_biogenesis | 237 | 2 | 0.733222 | -0.115680 | 948 | 575.029048 | 707.59 | 840.150952 | 0.746403 |  |
| GO:0032787\_monocarboxylic\_acid\_metabolic\_process | 130 | 1 | 0.668360 | -0.106404 | 950 | 583.147187 | 714.43 | 845.712813 | 0.752032 |  |
| GO:0050776\_regulation\_of\_immune\_response | 130 | 1 | 0.668360 | -0.106404 | 950 | 583.147187 | 714.43 | 845.712813 | 0.752032 |  |
| GO:0044057\_regulation\_of\_system\_process | 133 | 1 | 0.653284 | -0.102190 | 951 | 585.927013 | 716.75 | 847.572987 | 0.753680 |  |
| GO:0009057\_macromolecule\_catabolic\_process | 137 | 1 | 0.634210 | -0.096855 | 952 | 589.904681 | 720.24 | 850.575319 | 0.756555 |  |
| GO:0016044\_membrane\_organization | 140 | 1 | 0.620620 | -0.093055 | 953 | 592.898076 | 722.85 | 852.801924 | 0.758499 |  |
| GO:0007186\_G-protein\_coupled\_receptor\_protein\_signaling\_pathway | 144 | 1 | 0.603381 | -0.088240 | 954 | 597.960737 | 727.0 | 856.039263 | 0.762055 |  |
| GO:0006812\_cation\_transport | 146 | 1 | 0.595115 | -0.085935 | 955 | 599.437132 | 728.31 | 857.182868 | 0.762628 |  |
| GO:0051704\_multi-organism\_process | 157 | 1 | 0.553419 | -0.074366 | 956 | 610.139470 | 736.43 | 862.720530 | 0.770324 |  |
| GO:0007626\_locomotory\_behavior | 163 | 1 | 0.533048 | -0.068771 | 957 | 616.076712 | 741.12 | 866.163288 | 0.774420 |  |
| GO:0006259\_DNA\_metabolic\_process | 165 | 1 | 0.526587 | -0.067006 | 958 | 617.659575 | 742.41 | 867.160425 | 0.774958 |  |
| GO:0043065\_positive\_regulation\_of\_apoptosis | 166 | 1 | 0.523414 | -0.066142 | 959 | 618.364013 | 742.96 | 867.555987 | 0.774724 |  |
| GO:0010942\_positive\_regulation\_of\_cell\_death | 167 | 1 | 0.520280 | -0.065290 | 962 | 619.695702 | 744.09 | 868.484298 | 0.773482 |  |
| GO:0043068\_positive\_regulation\_of\_programmed\_cell\_death | 167 | 1 | 0.520280 | -0.065290 | 962 | 619.695702 | 744.09 | 868.484298 | 0.773482 |  |
| GO:0051049\_regulation\_of\_transport | 167 | 1 | 0.520280 | -0.065290 | 962 | 619.695702 | 744.09 | 868.484298 | 0.773482 |  |
| GO:0044248\_cellular\_catabolic\_process | 173 | 1 | 0.502236 | -0.060415 | 963 | 624.609669 | 747.91 | 871.210331 | 0.776646 |  |
| GO:0006873\_cellular\_ion\_homeostasis | 176 | 1 | 0.493675 | -0.058123 | 964 | 626.907590 | 749.79 | 872.672410 | 0.777790 |  |
| GO:0019752\_carboxylic\_acid\_metabolic\_process | 181 | 1 | 0.480038 | -0.054503 | 967 | 630.883566 | 752.9 | 874.916434 | 0.778594 |  |
| GO:0043436\_oxoacid\_metabolic\_process | 181 | 1 | 0.480038 | -0.054503 | 967 | 630.883566 | 752.9 | 874.916434 | 0.778594 |  |
| GO:0055082\_cellular\_chemical\_homeostasis | 181 | 1 | 0.480038 | -0.054503 | 967 | 630.883566 | 752.9 | 874.916434 | 0.778594 |  |
| GO:0006082\_organic\_acid\_metabolic\_process | 182 | 1 | 0.477400 | -0.053808 | 968 | 631.140992 | 753.14 | 875.139008 | 0.778037 |  |
| GO:0042180\_cellular\_ketone\_metabolic\_process | 183 | 1 | 0.474791 | -0.053122 | 969 | 631.447311 | 753.42 | 875.392689 | 0.777523 |  |
| GO:0006811\_ion\_transport | 186 | 1 | 0.467133 | -0.051119 | 970 | 633.597927 | 755.28 | 876.962073 | 0.778639 |  |
| GO:0006952\_defense\_response | 187 | 1 | 0.464635 | -0.050469 | 971 | 634.578047 | 755.94 | 877.301953 | 0.778517 |  |
| GO:0019725\_cellular\_homeostasis | 195 | 1 | 0.445573 | -0.045567 | 972 | 638.262956 | 758.81 | 879.357044 | 0.780669 |  |
| GO:0050801\_ion\_homeostasis | 197 | 1 | 0.441050 | -0.044421 | 973 | 640.018223 | 760.15 | 880.281777 | 0.781244 |  |
| GO:0051649\_establishment\_of\_localization\_in\_cell | 342 | 2 | 0.508110 | -0.039373 | 974 | 644.779568 | 763.69 | 882.600432 | 0.784076 |  |
| GO:0009056\_catabolic\_process | 243 | 1 | 0.357559 | -0.024832 | 975 | 658.352187 | 773.2 | 888.047813 | 0.793026 |  |
| GO:0032879\_regulation\_of\_localization | 248 | 1 | 0.350350 | -0.023319 | 976 | 659.789002 | 774.07 | 888.350998 | 0.793105 |  |
| GO:0048878\_chemical\_homeostasis | 254 | 1 | 0.342074 | -0.021625 | 977 | 661.618740 | 775.44 | 889.261260 | 0.793695 |  |
| GO:0007610\_behavior | 279 | 1 | 0.311422 | -0.015799 | 978 | 667.033172 | 779.26 | 891.486828 | 0.796789 |  |
| GO:0000012\_single\_strand\_break\_repair | 2 | 0 |  |  |  |  |  |  |  |  |
| GO:0000019\_regulation\_of\_mitotic\_recombination | 2 | 0 |  |  |  |  |  |  |  |  |
| GO:0000076\_DNA\_replication\_checkpoint | 2 | 0 |  |  |  |  |  |  |  |  |
| GO:0000080\_G1\_phase\_of\_mitotic\_cell\_cycle | 2 | 0 |  |  |  |  |  |  |  |  |
| GO:0000083\_regulation\_of\_transcription\_of\_G1\_S-phase\_of\_mitotic\_cell\_cycle | 2 | 0 |  |  |  |  |  |  |  |  |
| GO:0000085\_G2\_phase\_of\_mitotic\_cell\_cycle | 2 | 0 |  |  |  |  |  |  |  |  |
| GO:0000289\_nuclear-transcribed\_mRNA\_poly(A)\_tail\_shortening | 2 | 0 |  |  |  |  |  |  |  |  |
| GO:0000381\_regulation\_of\_alternative\_nuclear\_mRNA\_splicing\_\_via\_spliceosome | 2 | 0 |  |  |  |  |  |  |  |  |
| GO:0000712\_resolution\_of\_meiotic\_joint\_molecules\_as\_recombinants | 2 | 0 |  |  |  |  |  |  |  |  |
| GO:0000720\_pyrimidine\_dimer\_repair\_by\_nucleotide-excision\_repair | 2 | 0 |  |  |  |  |  |  |  |  |
| GO:0001302\_replicative\_cell\_aging | 2 | 0 |  |  |  |  |  |  |  |  |
| GO:0001306\_age-dependent\_response\_to\_oxidative\_stress | 2 | 0 |  |  |  |  |  |  |  |  |
| GO:0001514\_selenocysteine\_incorporation | 2 | 0 |  |  |  |  |  |  |  |  |
| GO:0001522\_pseudouridine\_synthesis | 2 | 0 |  |  |  |  |  |  |  |  |
| GO:0001543\_ovarian\_follicle\_rupture | 2 | 0 |  |  |  |  |  |  |  |  |
| GO:0001561\_fatty\_acid\_alpha-oxidation | 2 | 0 |  |  |  |  |  |  |  |  |
| GO:0001675\_acrosome\_assembly | 2 | 0 |  |  |  |  |  |  |  |  |
| GO:0001767\_establishment\_of\_lymphocyte\_polarity | 2 | 0 |  |  |  |  |  |  |  |  |
| GO:0001768\_establishment\_of\_T\_cell\_polarity | 2 | 0 |  |  |  |  |  |  |  |  |
| GO:0001771\_formation\_of\_immunological\_synapse | 2 | 0 |  |  |  |  |  |  |  |  |
| GO:0001774\_microglial\_cell\_activation | 2 | 0 |  |  |  |  |  |  |  |  |
| GO:0001781\_neutrophil\_apoptosis | 2 | 0 |  |  |  |  |  |  |  |  |
| GO:0001787\_natural\_killer\_cell\_proliferation | 2 | 0 |  |  |  |  |  |  |  |  |
| GO:0001788\_antibody-dependent\_cellular\_cytotoxicity | 2 | 0 |  |  |  |  |  |  |  |  |
| GO:0001806\_type\_IV\_hypersensitivity | 2 | 0 |  |  |  |  |  |  |  |  |
| GO:0001807\_regulation\_of\_type\_IV\_hypersensitivity | 2 | 0 |  |  |  |  |  |  |  |  |
| GO:0001808\_negative\_regulation\_of\_type\_IV\_hypersensitivity | 2 | 0 |  |  |  |  |  |  |  |  |
| GO:0001823\_mesonephros\_development | 2 | 0 |  |  |  |  |  |  |  |  |
| GO:0001845\_phagolysosome\_formation | 2 | 0 |  |  |  |  |  |  |  |  |
| GO:0001866\_NK\_T\_cell\_proliferation | 2 | 0 |  |  |  |  |  |  |  |  |
| GO:0001879\_detection\_of\_yeast | 2 | 0 |  |  |  |  |  |  |  |  |
| GO:0001886\_endothelial\_cell\_morphogenesis | 2 | 0 |  |  |  |  |  |  |  |  |
| GO:0001919\_regulation\_of\_receptor\_recycling | 2 | 0 |  |  |  |  |  |  |  |  |
| GO:0001954\_positive\_regulation\_of\_cell-matrix\_adhesion | 2 | 0 |  |  |  |  |  |  |  |  |
| GO:0001977\_renal\_system\_process\_involved\_in\_regulation\_of\_blood\_volume | 2 | 0 |  |  |  |  |  |  |  |  |
| GO:0001982\_baroreceptor\_response\_to\_decreased\_systemic\_arterial\_blood\_pressure | 2 | 0 |  |  |  |  |  |  |  |  |
| GO:0001983\_baroreceptor\_response\_to\_increased\_systemic\_arterial\_blood\_pressure | 2 | 0 |  |  |  |  |  |  |  |  |
| GO:0001992\_regulation\_of\_systemic\_arterial\_blood\_pressure\_by\_vasopressin | 2 | 0 |  |  |  |  |  |  |  |  |
| GO:0001997\_positive\_regulation\_of\_the\_force\_of\_heart\_contraction\_by\_epinephrine-norepinephrine | 2 | 0 |  |  |  |  |  |  |  |  |
| GO:0001998\_angiotensin\_mediated\_vasoconstriction\_involved\_in\_regulation\_of\_systemic\_arterial\_blood\_pressure | 2 | 0 |  |  |  |  |  |  |  |  |
| GO:0001999\_renal\_response\_to\_blood\_flow\_during\_renin-angiotensin\_regulation\_of\_systemic\_arterial\_blood\_pressure | 2 | 0 |  |  |  |  |  |  |  |  |
| GO:0002018\_renin-angiotensin\_regulation\_of\_aldosterone\_production | 2 | 0 |  |  |  |  |  |  |  |  |
| GO:0002019\_regulation\_of\_renal\_output\_by\_angiotensin | 2 | 0 |  |  |  |  |  |  |  |  |
| GO:0002024\_diet\_induced\_thermogenesis | 2 | 0 |  |  |  |  |  |  |  |  |
| GO:0002025\_vasodilation\_by\_norepinephrine-epinephrine\_involved\_in\_regulation\_of\_systemic\_arterial\_blood\_pressure | 2 | 0 |  |  |  |  |  |  |  |  |
| GO:0002029\_desensitization\_of\_G-protein\_coupled\_receptor\_protein\_signaling\_pathway | 2 | 0 |  |  |  |  |  |  |  |  |
| GO:0002033\_vasodilation\_by\_angiotensin\_involved\_in\_regulation\_of\_systemic\_arterial\_blood\_pressure | 2 | 0 |  |  |  |  |  |  |  |  |
| GO:0002066\_columnar\_cuboidal\_epithelial\_cell\_development | 2 | 0 |  |  |  |  |  |  |  |  |
| GO:0002072\_optic\_cup\_morphogenesis\_involved\_in\_camera-type\_eye\_development | 2 | 0 |  |  |  |  |  |  |  |  |
| GO:0002074\_extraocular\_skeletal\_muscle\_development | 2 | 0 |  |  |  |  |  |  |  |  |
| GO:0002138\_retinoic\_acid\_biosynthetic\_process | 2 | 0 |  |  |  |  |  |  |  |  |
| GO:0002223\_stimulatory\_C-type\_lectin\_receptor\_signaling\_pathway | 2 | 0 |  |  |  |  |  |  |  |  |
| GO:0002246\_healing\_during\_inflammatory\_response | 2 | 0 |  |  |  |  |  |  |  |  |
| GO:0002251\_organ\_or\_tissue\_specific\_immune\_response | 2 | 0 |  |  |  |  |  |  |  |  |
| GO:0002266\_follicular\_dendritic\_cell\_activation | 2 | 0 |  |  |  |  |  |  |  |  |
| GO:0002268\_follicular\_dendritic\_cell\_differentiation | 2 | 0 |  |  |  |  |  |  |  |  |
| GO:0002327\_immature\_B\_cell\_differentiation | 2 | 0 |  |  |  |  |  |  |  |  |
| GO:0002329\_pre-B\_cell\_differentiation | 2 | 0 |  |  |  |  |  |  |  |  |
| GO:0002339\_B\_cell\_selection | 2 | 0 |  |  |  |  |  |  |  |  |
| GO:0002352\_B\_cell\_negative\_selection | 2 | 0 |  |  |  |  |  |  |  |  |
| GO:0002358\_B\_cell\_homeostatic\_proliferation | 2 | 0 |  |  |  |  |  |  |  |  |
| GO:0002385\_mucosal\_immune\_response | 2 | 0 |  |  |  |  |  |  |  |  |
| GO:0002514\_B\_cell\_tolerance\_induction | 2 | 0 |  |  |  |  |  |  |  |  |
| GO:0002523\_leukocyte\_migration\_during\_inflammatory\_response | 2 | 0 |  |  |  |  |  |  |  |  |
| GO:0002536\_respiratory\_burst\_during\_acute\_inflammatory\_response | 2 | 0 |  |  |  |  |  |  |  |  |
| GO:0002537\_production\_of\_nitric\_oxide\_during\_acute\_inflammatory\_response | 2 | 0 |  |  |  |  |  |  |  |  |
| GO:0002639\_positive\_regulation\_of\_immunoglobulin\_production | 2 | 0 |  |  |  |  |  |  |  |  |
| GO:0002661\_regulation\_of\_B\_cell\_tolerance\_induction | 2 | 0 |  |  |  |  |  |  |  |  |
| GO:0002663\_positive\_regulation\_of\_B\_cell\_tolerance\_induction | 2 | 0 |  |  |  |  |  |  |  |  |
| GO:0002676\_regulation\_of\_chronic\_inflammatory\_response | 2 | 0 |  |  |  |  |  |  |  |  |
| GO:0002679\_respiratory\_burst\_during\_defense\_response | 2 | 0 |  |  |  |  |  |  |  |  |
| GO:0002686\_negative\_regulation\_of\_leukocyte\_migration | 2 | 0 |  |  |  |  |  |  |  |  |
| GO:0002720\_positive\_regulation\_of\_cytokine\_production\_during\_immune\_response | 2 | 0 |  |  |  |  |  |  |  |  |
| GO:0002752\_cell\_surface\_pattern\_recognition\_receptor\_signaling\_pathway | 2 | 0 |  |  |  |  |  |  |  |  |
| GO:0002755\_MyD88-dependent\_toll-like\_receptor\_signaling\_pathway | 2 | 0 |  |  |  |  |  |  |  |  |
| GO:0002765\_immune\_response-inhibiting\_signal\_transduction | 2 | 0 |  |  |  |  |  |  |  |  |
| GO:0002921\_negative\_regulation\_of\_humoral\_immune\_response | 2 | 0 |  |  |  |  |  |  |  |  |
| GO:0002922\_positive\_regulation\_of\_humoral\_immune\_response | 2 | 0 |  |  |  |  |  |  |  |  |
| GO:0002924\_negative\_regulation\_of\_humoral\_immune\_response\_mediated\_by\_circulating\_immunoglobulin | 2 | 0 |  |  |  |  |  |  |  |  |
| GO:0002925\_positive\_regulation\_of\_humoral\_immune\_response\_mediated\_by\_circulating\_immunoglobulin | 2 | 0 |  |  |  |  |  |  |  |  |
| GO:0003057\_regulation\_of\_the\_force\_of\_heart\_contraction\_by\_chemical\_signal | 2 | 0 |  |  |  |  |  |  |  |  |
| GO:0003099\_positive\_regulation\_of\_the\_force\_of\_heart\_contraction\_by\_chemical\_signal | 2 | 0 |  |  |  |  |  |  |  |  |
| GO:0005981\_regulation\_of\_glycogen\_catabolic\_process | 2 | 0 |  |  |  |  |  |  |  |  |
| GO:0006021\_inositol\_biosynthetic\_process | 2 | 0 |  |  |  |  |  |  |  |  |
| GO:0006042\_glucosamine\_biosynthetic\_process | 2 | 0 |  |  |  |  |  |  |  |  |
| GO:0006045\_N-acetylglucosamine\_biosynthetic\_process | 2 | 0 |  |  |  |  |  |  |  |  |
| GO:0006048\_UDP-N-acetylglucosamine\_biosynthetic\_process | 2 | 0 |  |  |  |  |  |  |  |  |
| GO:0006054\_N-acetylneuraminate\_metabolic\_process | 2 | 0 |  |  |  |  |  |  |  |  |
| GO:0006059\_hexitol\_metabolic\_process | 2 | 0 |  |  |  |  |  |  |  |  |
| GO:0006063\_uronic\_acid\_metabolic\_process | 2 | 0 |  |  |  |  |  |  |  |  |
| GO:0006068\_ethanol\_catabolic\_process | 2 | 0 |  |  |  |  |  |  |  |  |
| GO:0006083\_acetate\_metabolic\_process | 2 | 0 |  |  |  |  |  |  |  |  |
| GO:0006089\_lactate\_metabolic\_process | 2 | 0 |  |  |  |  |  |  |  |  |
| GO:0006105\_succinate\_metabolic\_process | 2 | 0 |  |  |  |  |  |  |  |  |
| GO:0006106\_fumarate\_metabolic\_process | 2 | 0 |  |  |  |  |  |  |  |  |
| GO:0006110\_regulation\_of\_glycolysis | 2 | 0 |  |  |  |  |  |  |  |  |
| GO:0006113\_fermentation | 2 | 0 |  |  |  |  |  |  |  |  |
| GO:0006114\_glycerol\_biosynthetic\_process | 2 | 0 |  |  |  |  |  |  |  |  |
| GO:0006122\_mitochondrial\_electron\_transport\_\_ubiquinol\_to\_cytochrome\_c | 2 | 0 |  |  |  |  |  |  |  |  |
| GO:0006152\_purine\_nucleoside\_catabolic\_process | 2 | 0 |  |  |  |  |  |  |  |  |
| GO:0006168\_adenine\_salvage | 2 | 0 |  |  |  |  |  |  |  |  |
| GO:0006200\_ATP\_catabolic\_process | 2 | 0 |  |  |  |  |  |  |  |  |
| GO:0006206\_pyrimidine\_base\_metabolic\_process | 2 | 0 |  |  |  |  |  |  |  |  |
| GO:0006213\_pyrimidine\_nucleoside\_metabolic\_process | 2 | 0 |  |  |  |  |  |  |  |  |
| GO:0006265\_DNA\_topological\_change | 2 | 0 |  |  |  |  |  |  |  |  |
| GO:0006278\_RNA-dependent\_DNA\_replication | 2 | 0 |  |  |  |  |  |  |  |  |
| GO:0006312\_mitotic\_recombination | 2 | 0 |  |  |  |  |  |  |  |  |
| GO:0006398\_histone\_mRNA\_3'-end\_processing | 2 | 0 |  |  |  |  |  |  |  |  |
| GO:0006418\_tRNA\_aminoacylation\_for\_protein\_translation | 2 | 0 |  |  |  |  |  |  |  |  |
| GO:0006451\_translational\_readthrough | 2 | 0 |  |  |  |  |  |  |  |  |
| GO:0006477\_protein\_amino\_acid\_sulfation | 2 | 0 |  |  |  |  |  |  |  |  |
| GO:0006482\_protein\_amino\_acid\_demethylation | 2 | 0 |  |  |  |  |  |  |  |  |
| GO:0006499\_N-terminal\_protein\_myristoylation | 2 | 0 |  |  |  |  |  |  |  |  |
| GO:0006525\_arginine\_metabolic\_process | 2 | 0 |  |  |  |  |  |  |  |  |
| GO:0006527\_arginine\_catabolic\_process | 2 | 0 |  |  |  |  |  |  |  |  |
| GO:0006532\_aspartate\_biosynthetic\_process | 2 | 0 |  |  |  |  |  |  |  |  |
| GO:0006538\_glutamate\_catabolic\_process | 2 | 0 |  |  |  |  |  |  |  |  |
| GO:0006558\_L-phenylalanine\_metabolic\_process | 2 | 0 |  |  |  |  |  |  |  |  |
| GO:0006563\_L-serine\_metabolic\_process | 2 | 0 |  |  |  |  |  |  |  |  |
| GO:0006566\_threonine\_metabolic\_process | 2 | 0 |  |  |  |  |  |  |  |  |
| GO:0006568\_tryptophan\_metabolic\_process | 2 | 0 |  |  |  |  |  |  |  |  |
| GO:0006583\_melanin\_biosynthetic\_process\_from\_tyrosine | 2 | 0 |  |  |  |  |  |  |  |  |
| GO:0006600\_creatine\_metabolic\_process | 2 | 0 |  |  |  |  |  |  |  |  |
| GO:0006603\_phosphocreatine\_metabolic\_process | 2 | 0 |  |  |  |  |  |  |  |  |
| GO:0006610\_ribosomal\_protein\_import\_into\_nucleus | 2 | 0 |  |  |  |  |  |  |  |  |
| GO:0006642\_triglyceride\_mobilization | 2 | 0 |  |  |  |  |  |  |  |  |
| GO:0006649\_phospholipid\_transfer\_to\_membrane | 2 | 0 |  |  |  |  |  |  |  |  |
| GO:0006681\_galactosylceramide\_metabolic\_process | 2 | 0 |  |  |  |  |  |  |  |  |
| GO:0006686\_sphingomyelin\_biosynthetic\_process | 2 | 0 |  |  |  |  |  |  |  |  |
| GO:0006702\_androgen\_biosynthetic\_process | 2 | 0 |  |  |  |  |  |  |  |  |
| GO:0006750\_glutathione\_biosynthetic\_process | 2 | 0 |  |  |  |  |  |  |  |  |
| GO:0006760\_folic\_acid\_and\_derivative\_metabolic\_process | 2 | 0 |  |  |  |  |  |  |  |  |
| GO:0006808\_regulation\_of\_nitrogen\_utilization | 2 | 0 |  |  |  |  |  |  |  |  |
| GO:0006868\_glutamine\_transport | 2 | 0 |  |  |  |  |  |  |  |  |
| GO:0006907\_pinocytosis | 2 | 0 |  |  |  |  |  |  |  |  |
| GO:0006925\_inflammatory\_cell\_apoptosis | 2 | 0 |  |  |  |  |  |  |  |  |
| GO:0006977\_DNA\_damage\_response\_\_signal\_transduction\_by\_p53\_class\_mediator\_resulting\_in\_cell\_cycle\_arrest | 2 | 0 |  |  |  |  |  |  |  |  |
| GO:0006991\_response\_to\_sterol\_depletion | 2 | 0 |  |  |  |  |  |  |  |  |
| GO:0007004\_telomere\_maintenance\_via\_telomerase | 2 | 0 |  |  |  |  |  |  |  |  |
| GO:0007020\_microtubule\_nucleation | 2 | 0 |  |  |  |  |  |  |  |  |
| GO:0007030\_Golgi\_organization | 2 | 0 |  |  |  |  |  |  |  |  |
| GO:0007035\_vacuolar\_acidification | 2 | 0 |  |  |  |  |  |  |  |  |
| GO:0007042\_lysosomal\_lumen\_acidification | 2 | 0 |  |  |  |  |  |  |  |  |
| GO:0007060\_male\_meiosis\_chromosome\_segregation | 2 | 0 |  |  |  |  |  |  |  |  |
| GO:0007089\_traversing\_start\_control\_point\_of\_mitotic\_cell\_cycle | 2 | 0 |  |  |  |  |  |  |  |  |
| GO:0007094\_mitotic\_cell\_cycle\_spindle\_assembly\_checkpoint | 2 | 0 |  |  |  |  |  |  |  |  |
| GO:0007097\_nuclear\_migration | 2 | 0 |  |  |  |  |  |  |  |  |
| GO:0007100\_mitotic\_centrosome\_separation | 2 | 0 |  |  |  |  |  |  |  |  |
| GO:0007132\_meiotic\_metaphase\_I | 2 | 0 |  |  |  |  |  |  |  |  |
| GO:0007171\_activation\_of\_transmembrane\_receptor\_protein\_tyrosine\_kinase\_activity | 2 | 0 |  |  |  |  |  |  |  |  |
| GO:0007182\_common-partner\_SMAD\_protein\_phosphorylation | 2 | 0 |  |  |  |  |  |  |  |  |
| GO:0007185\_transmembrane\_receptor\_protein\_tyrosine\_phosphatase\_signaling\_pathway | 2 | 0 |  |  |  |  |  |  |  |  |
| GO:0007205\_activation\_of\_protein\_kinase\_C\_activity\_by\_G-protein\_coupled\_receptor\_protein\_signaling\_pathway | 2 | 0 |  |  |  |  |  |  |  |  |
| GO:0007210\_serotonin\_receptor\_signaling\_pathway | 2 | 0 |  |  |  |  |  |  |  |  |
| GO:0007220\_Notch\_receptor\_processing | 2 | 0 |  |  |  |  |  |  |  |  |
| GO:0007256\_activation\_of\_JNKK\_activity | 2 | 0 |  |  |  |  |  |  |  |  |
| GO:0007258\_JUN\_phosphorylation | 2 | 0 |  |  |  |  |  |  |  |  |
| GO:0007263\_nitric\_oxide\_mediated\_signal\_transduction | 2 | 0 |  |  |  |  |  |  |  |  |
| GO:0007289\_spermatid\_nucleus\_differentiation | 2 | 0 |  |  |  |  |  |  |  |  |
| GO:0007343\_egg\_activation | 2 | 0 |  |  |  |  |  |  |  |  |
| GO:0007351\_tripartite\_regional\_subdivision | 2 | 0 |  |  |  |  |  |  |  |  |
| GO:0007418\_ventral\_midline\_development | 2 | 0 |  |  |  |  |  |  |  |  |
| GO:0007494\_midgut\_development | 2 | 0 |  |  |  |  |  |  |  |  |
| GO:0007527\_adult\_somatic\_muscle\_development | 2 | 0 |  |  |  |  |  |  |  |  |
| GO:0007549\_dosage\_compensation | 2 | 0 |  |  |  |  |  |  |  |  |
| GO:0007571\_age-dependent\_general\_metabolic\_decline | 2 | 0 |  |  |  |  |  |  |  |  |
| GO:0007603\_phototransduction\_\_visible\_light | 2 | 0 |  |  |  |  |  |  |  |  |
| GO:0007619\_courtship\_behavior | 2 | 0 |  |  |  |  |  |  |  |  |
| GO:0008065\_establishment\_of\_blood-nerve\_barrier | 2 | 0 |  |  |  |  |  |  |  |  |
| GO:0008089\_anterograde\_axon\_cargo\_transport | 2 | 0 |  |  |  |  |  |  |  |  |
| GO:0008210\_estrogen\_metabolic\_process | 2 | 0 |  |  |  |  |  |  |  |  |
| GO:0008212\_mineralocorticoid\_metabolic\_process | 2 | 0 |  |  |  |  |  |  |  |  |
| GO:0008214\_protein\_amino\_acid\_dealkylation | 2 | 0 |  |  |  |  |  |  |  |  |
| GO:0008228\_opsonization | 2 | 0 |  |  |  |  |  |  |  |  |
| GO:0008272\_sulfate\_transport | 2 | 0 |  |  |  |  |  |  |  |  |
| GO:0008291\_acetylcholine\_metabolic\_process | 2 | 0 |  |  |  |  |  |  |  |  |
| GO:0008298\_intracellular\_mRNA\_localization | 2 | 0 |  |  |  |  |  |  |  |  |
| GO:0008334\_histone\_mRNA\_metabolic\_process | 2 | 0 |  |  |  |  |  |  |  |  |
| GO:0008356\_asymmetric\_cell\_division | 2 | 0 |  |  |  |  |  |  |  |  |
| GO:0008582\_regulation\_of\_synaptic\_growth\_at\_neuromuscular\_junction | 2 | 0 |  |  |  |  |  |  |  |  |
| GO:0008594\_photoreceptor\_cell\_morphogenesis | 2 | 0 |  |  |  |  |  |  |  |  |
| GO:0008595\_determination\_of\_anterior\_posterior\_axis\_\_embryo | 2 | 0 |  |  |  |  |  |  |  |  |
| GO:0008608\_attachment\_of\_spindle\_microtubules\_to\_kinetochore | 2 | 0 |  |  |  |  |  |  |  |  |
| GO:0008616\_queuosine\_biosynthetic\_process | 2 | 0 |  |  |  |  |  |  |  |  |
| GO:0008617\_guanosine\_metabolic\_process | 2 | 0 |  |  |  |  |  |  |  |  |
| GO:0008618\_7-methylguanosine\_metabolic\_process | 2 | 0 |  |  |  |  |  |  |  |  |
| GO:0008634\_negative\_regulation\_of\_survival\_gene\_product\_expression | 2 | 0 |  |  |  |  |  |  |  |  |
| GO:0009048\_dosage\_compensation\_\_by\_inactivation\_of\_X\_chromosome | 2 | 0 |  |  |  |  |  |  |  |  |
| GO:0009070\_serine\_family\_amino\_acid\_biosynthetic\_process | 2 | 0 |  |  |  |  |  |  |  |  |
| GO:0009071\_serine\_family\_amino\_acid\_catabolic\_process | 2 | 0 |  |  |  |  |  |  |  |  |
| GO:0009074\_aromatic\_amino\_acid\_family\_catabolic\_process | 2 | 0 |  |  |  |  |  |  |  |  |
| GO:0009083\_branched\_chain\_family\_amino\_acid\_catabolic\_process | 2 | 0 |  |  |  |  |  |  |  |  |
| GO:0009093\_cysteine\_catabolic\_process | 2 | 0 |  |  |  |  |  |  |  |  |
| GO:0009120\_deoxyribonucleoside\_metabolic\_process | 2 | 0 |  |  |  |  |  |  |  |  |
| GO:0009125\_nucleoside\_monophosphate\_catabolic\_process | 2 | 0 |  |  |  |  |  |  |  |  |
| GO:0009126\_purine\_nucleoside\_monophosphate\_metabolic\_process | 2 | 0 |  |  |  |  |  |  |  |  |
| GO:0009142\_nucleoside\_triphosphate\_biosynthetic\_process | 2 | 0 |  |  |  |  |  |  |  |  |
| GO:0009161\_ribonucleoside\_monophosphate\_metabolic\_process | 2 | 0 |  |  |  |  |  |  |  |  |
| GO:0009164\_nucleoside\_catabolic\_process | 2 | 0 |  |  |  |  |  |  |  |  |
| GO:0009167\_purine\_ribonucleoside\_monophosphate\_metabolic\_process | 2 | 0 |  |  |  |  |  |  |  |  |
| GO:0009202\_deoxyribonucleoside\_triphosphate\_biosynthetic\_process | 2 | 0 |  |  |  |  |  |  |  |  |
| GO:0009203\_ribonucleoside\_triphosphate\_catabolic\_process | 2 | 0 |  |  |  |  |  |  |  |  |
| GO:0009207\_purine\_ribonucleoside\_triphosphate\_catabolic\_process | 2 | 0 |  |  |  |  |  |  |  |  |
| GO:0009219\_pyrimidine\_deoxyribonucleotide\_metabolic\_process | 2 | 0 |  |  |  |  |  |  |  |  |
| GO:0009265\_2'-deoxyribonucleotide\_biosynthetic\_process | 2 | 0 |  |  |  |  |  |  |  |  |
| GO:0009268\_response\_to\_pH | 2 | 0 |  |  |  |  |  |  |  |  |
| GO:0009313\_oligosaccharide\_catabolic\_process | 2 | 0 |  |  |  |  |  |  |  |  |
| GO:0009395\_phospholipid\_catabolic\_process | 2 | 0 |  |  |  |  |  |  |  |  |
| GO:0009435\_NAD\_biosynthetic\_process | 2 | 0 |  |  |  |  |  |  |  |  |
| GO:0009608\_response\_to\_symbiont | 2 | 0 |  |  |  |  |  |  |  |  |
| GO:0009609\_response\_to\_symbiotic\_bacterium | 2 | 0 |  |  |  |  |  |  |  |  |
| GO:0009649\_entrainment\_of\_circadian\_clock | 2 | 0 |  |  |  |  |  |  |  |  |
| GO:0009996\_negative\_regulation\_of\_cell\_fate\_specification | 2 | 0 |  |  |  |  |  |  |  |  |
| GO:0010002\_cardioblast\_differentiation | 2 | 0 |  |  |  |  |  |  |  |  |
| GO:0010149\_senescence | 2 | 0 |  |  |  |  |  |  |  |  |
| GO:0010225\_response\_to\_UV-C | 2 | 0 |  |  |  |  |  |  |  |  |
| GO:0010389\_regulation\_of\_G2\_M\_transition\_of\_mitotic\_cell\_cycle | 2 | 0 |  |  |  |  |  |  |  |  |
| GO:0010458\_exit\_from\_mitosis | 2 | 0 |  |  |  |  |  |  |  |  |
| GO:0010459\_negative\_regulation\_of\_heart\_rate | 2 | 0 |  |  |  |  |  |  |  |  |
| GO:0010559\_regulation\_of\_glycoprotein\_biosynthetic\_process | 2 | 0 |  |  |  |  |  |  |  |  |
| GO:0010633\_negative\_regulation\_of\_epithelial\_cell\_migration | 2 | 0 |  |  |  |  |  |  |  |  |
| GO:0010677\_negative\_regulation\_of\_cellular\_carbohydrate\_metabolic\_process | 2 | 0 |  |  |  |  |  |  |  |  |
| GO:0010718\_positive\_regulation\_of\_epithelial\_to\_mesenchymal\_transition | 2 | 0 |  |  |  |  |  |  |  |  |
| GO:0010742\_foam\_cell\_differentiation | 2 | 0 |  |  |  |  |  |  |  |  |
| GO:0010743\_regulation\_of\_foam\_cell\_differentiation | 2 | 0 |  |  |  |  |  |  |  |  |
| GO:0010744\_positive\_regulation\_of\_foam\_cell\_differentiation | 2 | 0 |  |  |  |  |  |  |  |  |
| GO:0010765\_positive\_regulation\_of\_sodium\_ion\_transport | 2 | 0 |  |  |  |  |  |  |  |  |
| GO:0010766\_negative\_regulation\_of\_sodium\_ion\_transport | 2 | 0 |  |  |  |  |  |  |  |  |
| GO:0010770\_positive\_regulation\_of\_cell\_morphogenesis\_involved\_in\_differentiation | 2 | 0 |  |  |  |  |  |  |  |  |
| GO:0010771\_negative\_regulation\_of\_cell\_morphogenesis\_involved\_in\_differentiation | 2 | 0 |  |  |  |  |  |  |  |  |
| GO:0010824\_regulation\_of\_centrosome\_duplication | 2 | 0 |  |  |  |  |  |  |  |  |
| GO:0010833\_telomere\_maintenance\_via\_telomere\_lengthening | 2 | 0 |  |  |  |  |  |  |  |  |
| GO:0010862\_positive\_regulation\_of\_pathway-restricted\_SMAD\_protein\_phosphorylation | 2 | 0 |  |  |  |  |  |  |  |  |
| GO:0010878\_cholesterol\_storage | 2 | 0 |  |  |  |  |  |  |  |  |
| GO:0010885\_regulation\_of\_cholesterol\_storage | 2 | 0 |  |  |  |  |  |  |  |  |
| GO:0010886\_positive\_regulation\_of\_cholesterol\_storage | 2 | 0 |  |  |  |  |  |  |  |  |
| GO:0010891\_negative\_regulation\_of\_sequestering\_of\_triglyceride | 2 | 0 |  |  |  |  |  |  |  |  |
| GO:0010896\_regulation\_of\_triglyceride\_catabolic\_process | 2 | 0 |  |  |  |  |  |  |  |  |
| GO:0010898\_positive\_regulation\_of\_triglyceride\_catabolic\_process | 2 | 0 |  |  |  |  |  |  |  |  |
| GO:0010907\_positive\_regulation\_of\_glucose\_metabolic\_process | 2 | 0 |  |  |  |  |  |  |  |  |
| GO:0014028\_notochord\_formation | 2 | 0 |  |  |  |  |  |  |  |  |
| GO:0014048\_regulation\_of\_glutamate\_secretion | 2 | 0 |  |  |  |  |  |  |  |  |
| GO:0014052\_regulation\_of\_gamma-aminobutyric\_acid\_secretion | 2 | 0 |  |  |  |  |  |  |  |  |
| GO:0014054\_positive\_regulation\_of\_gamma-aminobutyric\_acid\_secretion | 2 | 0 |  |  |  |  |  |  |  |  |
| GO:0014055\_acetylcholine\_secretion | 2 | 0 |  |  |  |  |  |  |  |  |
| GO:0014056\_regulation\_of\_acetylcholine\_secretion | 2 | 0 |  |  |  |  |  |  |  |  |
| GO:0014067\_negative\_regulation\_of\_phosphoinositide\_3-kinase\_cascade | 2 | 0 |  |  |  |  |  |  |  |  |
| GO:0014745\_negative\_regulation\_of\_muscle\_adaptation | 2 | 0 |  |  |  |  |  |  |  |  |
| GO:0014850\_response\_to\_muscle\_activity | 2 | 0 |  |  |  |  |  |  |  |  |
| GO:0014888\_striated\_muscle\_adaptation | 2 | 0 |  |  |  |  |  |  |  |  |
| GO:0014916\_regulation\_of\_lung\_blood\_pressure | 2 | 0 |  |  |  |  |  |  |  |  |
| GO:0015671\_oxygen\_transport | 2 | 0 |  |  |  |  |  |  |  |  |
| GO:0015696\_ammonium\_transport | 2 | 0 |  |  |  |  |  |  |  |  |
| GO:0015732\_prostaglandin\_transport | 2 | 0 |  |  |  |  |  |  |  |  |
| GO:0015819\_lysine\_transport | 2 | 0 |  |  |  |  |  |  |  |  |
| GO:0015840\_urea\_transport | 2 | 0 |  |  |  |  |  |  |  |  |
| GO:0015860\_purine\_nucleoside\_transport | 2 | 0 |  |  |  |  |  |  |  |  |
| GO:0015870\_acetylcholine\_transport | 2 | 0 |  |  |  |  |  |  |  |  |
| GO:0015937\_coenzyme\_A\_biosynthetic\_process | 2 | 0 |  |  |  |  |  |  |  |  |
| GO:0016045\_detection\_of\_bacterium | 2 | 0 |  |  |  |  |  |  |  |  |
| GO:0016046\_detection\_of\_fungus | 2 | 0 |  |  |  |  |  |  |  |  |
| GO:0016080\_synaptic\_vesicle\_targeting | 2 | 0 |  |  |  |  |  |  |  |  |
| GO:0016199\_axon\_midline\_choice\_point\_recognition | 2 | 0 |  |  |  |  |  |  |  |  |
| GO:0016226\_iron-sulfur\_cluster\_assembly | 2 | 0 |  |  |  |  |  |  |  |  |
| GO:0016233\_telomere\_capping | 2 | 0 |  |  |  |  |  |  |  |  |
| GO:0016242\_negative\_regulation\_of\_macroautophagy | 2 | 0 |  |  |  |  |  |  |  |  |
| GO:0016441\_posttranscriptional\_gene\_silencing | 2 | 0 |  |  |  |  |  |  |  |  |
| GO:0016540\_protein\_autoprocessing | 2 | 0 |  |  |  |  |  |  |  |  |
| GO:0016558\_protein\_import\_into\_peroxisome\_matrix | 2 | 0 |  |  |  |  |  |  |  |  |
| GO:0016572\_histone\_phosphorylation | 2 | 0 |  |  |  |  |  |  |  |  |
| GO:0016577\_histone\_demethylation | 2 | 0 |  |  |  |  |  |  |  |  |
| GO:0016584\_nucleosome\_positioning | 2 | 0 |  |  |  |  |  |  |  |  |
| GO:0016926\_protein\_desumoylation | 2 | 0 |  |  |  |  |  |  |  |  |
| GO:0017014\_protein\_amino\_acid\_nitrosylation | 2 | 0 |  |  |  |  |  |  |  |  |
| GO:0017144\_drug\_metabolic\_process | 2 | 0 |  |  |  |  |  |  |  |  |
| GO:0018094\_protein\_polyglycylation | 2 | 0 |  |  |  |  |  |  |  |  |
| GO:0018119\_peptidyl-cysteine\_S-nitrosylation | 2 | 0 |  |  |  |  |  |  |  |  |
| GO:0018125\_peptidyl-cysteine\_methylation | 2 | 0 |  |  |  |  |  |  |  |  |
| GO:0018205\_peptidyl-lysine\_modification | 2 | 0 |  |  |  |  |  |  |  |  |
| GO:0018319\_protein\_amino\_acid\_myristoylation | 2 | 0 |  |  |  |  |  |  |  |  |
| GO:0018377\_protein\_myristoylation | 2 | 0 |  |  |  |  |  |  |  |  |
| GO:0018401\_peptidyl-proline\_hydroxylation\_to\_4-hydroxy-L-proline | 2 | 0 |  |  |  |  |  |  |  |  |
| GO:0018993\_somatic\_sex\_determination | 2 | 0 |  |  |  |  |  |  |  |  |
| GO:0019067\_viral\_assembly\_\_maturation\_\_egress\_\_and\_release | 2 | 0 |  |  |  |  |  |  |  |  |
| GO:0019322\_pentose\_biosynthetic\_process | 2 | 0 |  |  |  |  |  |  |  |  |
| GO:0019370\_leukotriene\_biosynthetic\_process | 2 | 0 |  |  |  |  |  |  |  |  |
| GO:0019374\_galactolipid\_metabolic\_process | 2 | 0 |  |  |  |  |  |  |  |  |
| GO:0019401\_alditol\_biosynthetic\_process | 2 | 0 |  |  |  |  |  |  |  |  |
| GO:0019448\_L-cysteine\_catabolic\_process | 2 | 0 |  |  |  |  |  |  |  |  |
| GO:0019452\_L-cysteine\_catabolic\_process\_to\_taurine | 2 | 0 |  |  |  |  |  |  |  |  |
| GO:0019471\_4-hydroxyproline\_metabolic\_process | 2 | 0 |  |  |  |  |  |  |  |  |
| GO:0019511\_peptidyl-proline\_hydroxylation | 2 | 0 |  |  |  |  |  |  |  |  |
| GO:0019550\_glutamate\_catabolic\_process\_to\_aspartate | 2 | 0 |  |  |  |  |  |  |  |  |
| GO:0019551\_glutamate\_catabolic\_process\_to\_2-oxoglutarate | 2 | 0 |  |  |  |  |  |  |  |  |
| GO:0019585\_glucuronate\_metabolic\_process | 2 | 0 |  |  |  |  |  |  |  |  |
| GO:0019730\_antimicrobial\_humoral\_response | 2 | 0 |  |  |  |  |  |  |  |  |
| GO:0019740\_nitrogen\_utilization | 2 | 0 |  |  |  |  |  |  |  |  |
| GO:0019853\_L-ascorbic\_acid\_biosynthetic\_process | 2 | 0 |  |  |  |  |  |  |  |  |
| GO:0021506\_anterior\_neuropore\_closure | 2 | 0 |  |  |  |  |  |  |  |  |
| GO:0021524\_visceral\_motor\_neuron\_differentiation | 2 | 0 |  |  |  |  |  |  |  |  |
| GO:0021526\_medial\_motor\_column\_neuron\_differentiation | 2 | 0 |  |  |  |  |  |  |  |  |
| GO:0021557\_oculomotor\_nerve\_development | 2 | 0 |  |  |  |  |  |  |  |  |
| GO:0021558\_trochlear\_nerve\_development | 2 | 0 |  |  |  |  |  |  |  |  |
| GO:0021562\_vestibulocochlear\_nerve\_development | 2 | 0 |  |  |  |  |  |  |  |  |
| GO:0021568\_rhombomere\_2\_development | 2 | 0 |  |  |  |  |  |  |  |  |
| GO:0021578\_hindbrain\_maturation | 2 | 0 |  |  |  |  |  |  |  |  |
| GO:0021593\_rhombomere\_morphogenesis | 2 | 0 |  |  |  |  |  |  |  |  |
| GO:0021626\_central\_nervous\_system\_maturation | 2 | 0 |  |  |  |  |  |  |  |  |
| GO:0021658\_rhombomere\_3\_morphogenesis | 2 | 0 |  |  |  |  |  |  |  |  |
| GO:0021754\_facial\_nucleus\_development | 2 | 0 |  |  |  |  |  |  |  |  |
| GO:0021775\_smoothened\_signaling\_pathway\_involved\_in\_ventral\_spinal\_cord\_interneuron\_specification | 2 | 0 |  |  |  |  |  |  |  |  |
| GO:0021776\_smoothened\_signaling\_pathway\_involved\_in\_spinal\_cord\_motor\_neuron\_cell\_fate\_specification | 2 | 0 |  |  |  |  |  |  |  |  |
| GO:0021796\_cerebral\_cortex\_regionalization | 2 | 0 |  |  |  |  |  |  |  |  |
| GO:0021831\_embryonic\_olfactory\_bulb\_interneuron\_precursor\_migration | 2 | 0 |  |  |  |  |  |  |  |  |
| GO:0021869\_forebrain\_ventricular\_zone\_progenitor\_cell\_division | 2 | 0 |  |  |  |  |  |  |  |  |
| GO:0021873\_forebrain\_neuroblast\_division | 2 | 0 |  |  |  |  |  |  |  |  |
| GO:0021882\_regulation\_of\_transcription\_from\_RNA\_polymerase\_II\_promoter\_involved\_in\_forebrain\_neuron\_fate\_commitment | 2 | 0 |  |  |  |  |  |  |  |  |
| GO:0021893\_cerebral\_cortex\_GABAergic\_interneuron\_fate\_commitment | 2 | 0 |  |  |  |  |  |  |  |  |
| GO:0021898\_commitment\_of\_multipotent\_stem\_cells\_to\_the\_neuronal\_lineage\_in\_the\_forebrain | 2 | 0 |  |  |  |  |  |  |  |  |
| GO:0021932\_hindbrain\_radial\_glia\_guided\_cell\_migration | 2 | 0 |  |  |  |  |  |  |  |  |
| GO:0021965\_spinal\_cord\_ventral\_commissure\_morphogenesis | 2 | 0 |  |  |  |  |  |  |  |  |
| GO:0021985\_neurohypophysis\_development | 2 | 0 |  |  |  |  |  |  |  |  |
| GO:0021990\_neural\_plate\_formation | 2 | 0 |  |  |  |  |  |  |  |  |
| GO:0021995\_neuropore\_closure | 2 | 0 |  |  |  |  |  |  |  |  |
| GO:0022028\_tangential\_migration\_from\_the\_subventricular\_zone\_to\_the\_olfactory\_bulb | 2 | 0 |  |  |  |  |  |  |  |  |
| GO:0022401\_adaptation\_of\_signaling\_pathway | 2 | 0 |  |  |  |  |  |  |  |  |
| GO:0022408\_negative\_regulation\_of\_cell-cell\_adhesion | 2 | 0 |  |  |  |  |  |  |  |  |
| GO:0022410\_circadian\_sleep\_wake\_cycle\_process | 2 | 0 |  |  |  |  |  |  |  |  |
| GO:0030046\_parallel\_actin\_filament\_bundle\_formation | 2 | 0 |  |  |  |  |  |  |  |  |
| GO:0030049\_muscle\_filament\_sliding | 2 | 0 |  |  |  |  |  |  |  |  |
| GO:0030050\_vesicle\_transport\_along\_actin\_filament | 2 | 0 |  |  |  |  |  |  |  |  |
| GO:0030071\_regulation\_of\_mitotic\_metaphase\_anaphase\_transition | 2 | 0 |  |  |  |  |  |  |  |  |
| GO:0030147\_natriuresis | 2 | 0 |  |  |  |  |  |  |  |  |
| GO:0030174\_regulation\_of\_DNA\_replication\_initiation | 2 | 0 |  |  |  |  |  |  |  |  |
| GO:0030202\_heparin\_metabolic\_process | 2 | 0 |  |  |  |  |  |  |  |  |
| GO:0030219\_megakaryocyte\_differentiation | 2 | 0 |  |  |  |  |  |  |  |  |
| GO:0030223\_neutrophil\_differentiation | 2 | 0 |  |  |  |  |  |  |  |  |
| GO:0030259\_lipid\_glycosylation | 2 | 0 |  |  |  |  |  |  |  |  |
| GO:0030397\_membrane\_disassembly | 2 | 0 |  |  |  |  |  |  |  |  |
| GO:0030502\_negative\_regulation\_of\_bone\_mineralization | 2 | 0 |  |  |  |  |  |  |  |  |
| GO:0030644\_cellular\_chloride\_ion\_homeostasis | 2 | 0 |  |  |  |  |  |  |  |  |
| GO:0030825\_positive\_regulation\_of\_cGMP\_metabolic\_process | 2 | 0 |  |  |  |  |  |  |  |  |
| GO:0030828\_positive\_regulation\_of\_cGMP\_biosynthetic\_process | 2 | 0 |  |  |  |  |  |  |  |  |
| GO:0030835\_negative\_regulation\_of\_actin\_filament\_depolymerization | 2 | 0 |  |  |  |  |  |  |  |  |
| GO:0030837\_negative\_regulation\_of\_actin\_filament\_polymerization | 2 | 0 |  |  |  |  |  |  |  |  |
| GO:0030852\_regulation\_of\_granulocyte\_differentiation | 2 | 0 |  |  |  |  |  |  |  |  |
| GO:0030885\_regulation\_of\_myeloid\_dendritic\_cell\_activation | 2 | 0 |  |  |  |  |  |  |  |  |
| GO:0030910\_olfactory\_placode\_formation | 2 | 0 |  |  |  |  |  |  |  |  |
| GO:0030948\_negative\_regulation\_of\_vascular\_endothelial\_growth\_factor\_receptor\_signaling\_pathway | 2 | 0 |  |  |  |  |  |  |  |  |
| GO:0030953\_spindle\_astral\_microtubule\_organization | 2 | 0 |  |  |  |  |  |  |  |  |
| GO:0031050\_dsRNA\_fragmentation | 2 | 0 |  |  |  |  |  |  |  |  |
| GO:0031061\_negative\_regulation\_of\_histone\_methylation | 2 | 0 |  |  |  |  |  |  |  |  |
| GO:0031119\_tRNA\_pseudouridine\_synthesis | 2 | 0 |  |  |  |  |  |  |  |  |
| GO:0031163\_metallo-sulfur\_cluster\_assembly | 2 | 0 |  |  |  |  |  |  |  |  |
| GO:0031223\_auditory\_behavior | 2 | 0 |  |  |  |  |  |  |  |  |
| GO:0031296\_B\_cell\_costimulation | 2 | 0 |  |  |  |  |  |  |  |  |
| GO:0031338\_regulation\_of\_vesicle\_fusion | 2 | 0 |  |  |  |  |  |  |  |  |
| GO:0031573\_intra-S\_DNA\_damage\_checkpoint | 2 | 0 |  |  |  |  |  |  |  |  |
| GO:0031577\_spindle\_checkpoint | 2 | 0 |  |  |  |  |  |  |  |  |
| GO:0031629\_synaptic\_vesicle\_fusion\_to\_presynaptic\_membrane | 2 | 0 |  |  |  |  |  |  |  |  |
| GO:0031630\_regulation\_of\_synaptic\_vesicle\_fusion\_to\_presynaptic\_membrane | 2 | 0 |  |  |  |  |  |  |  |  |
| GO:0031664\_regulation\_of\_lipopolysaccharide-mediated\_signaling\_pathway | 2 | 0 |  |  |  |  |  |  |  |  |
| GO:0031670\_cellular\_response\_to\_nutrient | 2 | 0 |  |  |  |  |  |  |  |  |
| GO:0031848\_protection\_from\_non-homologous\_end\_joining\_at\_telomere | 2 | 0 |  |  |  |  |  |  |  |  |
| GO:0031946\_regulation\_of\_glucocorticoid\_biosynthetic\_process | 2 | 0 |  |  |  |  |  |  |  |  |
| GO:0031958\_corticosteroid\_receptor\_signaling\_pathway | 2 | 0 |  |  |  |  |  |  |  |  |
| GO:0031987\_locomotion\_involved\_in\_locomotory\_behavior | 2 | 0 |  |  |  |  |  |  |  |  |
| GO:0032096\_negative\_regulation\_of\_response\_to\_food | 2 | 0 |  |  |  |  |  |  |  |  |
| GO:0032099\_negative\_regulation\_of\_appetite | 2 | 0 |  |  |  |  |  |  |  |  |
| GO:0032106\_positive\_regulation\_of\_response\_to\_extracellular\_stimulus | 2 | 0 |  |  |  |  |  |  |  |  |
| GO:0032109\_positive\_regulation\_of\_response\_to\_nutrient\_levels | 2 | 0 |  |  |  |  |  |  |  |  |
| GO:0032226\_positive\_regulation\_of\_synaptic\_transmission\_\_dopaminergic | 2 | 0 |  |  |  |  |  |  |  |  |
| GO:0032230\_positive\_regulation\_of\_synaptic\_transmission\_\_GABAergic | 2 | 0 |  |  |  |  |  |  |  |  |
| GO:0032234\_regulation\_of\_calcium\_ion\_transport\_via\_store-operated\_calcium\_channel\_activity | 2 | 0 |  |  |  |  |  |  |  |  |
| GO:0032236\_positive\_regulation\_of\_calcium\_ion\_transport\_via\_store-operated\_calcium\_channel\_activity | 2 | 0 |  |  |  |  |  |  |  |  |
| GO:0032297\_negative\_regulation\_of\_DNA\_replication\_initiation | 2 | 0 |  |  |  |  |  |  |  |  |
| GO:0032309\_icosanoid\_secretion | 2 | 0 |  |  |  |  |  |  |  |  |
| GO:0032328\_alanine\_transport | 2 | 0 |  |  |  |  |  |  |  |  |
| GO:0032341\_aldosterone\_metabolic\_process | 2 | 0 |  |  |  |  |  |  |  |  |
| GO:0032351\_negative\_regulation\_of\_hormone\_metabolic\_process | 2 | 0 |  |  |  |  |  |  |  |  |
| GO:0032353\_negative\_regulation\_of\_hormone\_biosynthetic\_process | 2 | 0 |  |  |  |  |  |  |  |  |
| GO:0032435\_negative\_regulation\_of\_proteasomal\_ubiquitin-dependent\_protein\_catabolic\_process | 2 | 0 |  |  |  |  |  |  |  |  |
| GO:0032471\_reduction\_of\_endoplasmic\_reticulum\_calcium\_ion\_concentration | 2 | 0 |  |  |  |  |  |  |  |  |
| GO:0032481\_positive\_regulation\_of\_type\_I\_interferon\_production | 2 | 0 |  |  |  |  |  |  |  |  |
| GO:0032488\_Cdc42\_protein\_signal\_transduction | 2 | 0 |  |  |  |  |  |  |  |  |
| GO:0032489\_regulation\_of\_Cdc42\_protein\_signal\_transduction | 2 | 0 |  |  |  |  |  |  |  |  |
| GO:0032495\_response\_to\_muramyl\_dipeptide | 2 | 0 |  |  |  |  |  |  |  |  |
| GO:0032604\_granulocyte\_macrophage\_colony-stimulating\_factor\_production | 2 | 0 |  |  |  |  |  |  |  |  |
| GO:0032616\_interleukin-13\_production | 2 | 0 |  |  |  |  |  |  |  |  |
| GO:0032645\_regulation\_of\_granulocyte\_macrophage\_colony-stimulating\_factor\_production | 2 | 0 |  |  |  |  |  |  |  |  |
| GO:0032672\_regulation\_of\_interleukin-3\_production | 2 | 0 |  |  |  |  |  |  |  |  |
| GO:0032695\_negative\_regulation\_of\_interleukin-12\_production | 2 | 0 |  |  |  |  |  |  |  |  |
| GO:0032714\_negative\_regulation\_of\_interleukin-5\_production | 2 | 0 |  |  |  |  |  |  |  |  |
| GO:0032722\_positive\_regulation\_of\_chemokine\_production | 2 | 0 |  |  |  |  |  |  |  |  |
| GO:0032743\_positive\_regulation\_of\_interleukin-2\_production | 2 | 0 |  |  |  |  |  |  |  |  |
| GO:0032762\_mast\_cell\_cytokine\_production | 2 | 0 |  |  |  |  |  |  |  |  |
| GO:0032763\_regulation\_of\_mast\_cell\_cytokine\_production | 2 | 0 |  |  |  |  |  |  |  |  |
| GO:0032768\_regulation\_of\_monooxygenase\_activity | 2 | 0 |  |  |  |  |  |  |  |  |
| GO:0032788\_saturated\_monocarboxylic\_acid\_metabolic\_process | 2 | 0 |  |  |  |  |  |  |  |  |
| GO:0032789\_unsaturated\_monocarboxylic\_acid\_metabolic\_process | 2 | 0 |  |  |  |  |  |  |  |  |
| GO:0032796\_uropod\_organization | 2 | 0 |  |  |  |  |  |  |  |  |
| GO:0032800\_receptor\_biosynthetic\_process | 2 | 0 |  |  |  |  |  |  |  |  |
| GO:0032801\_receptor\_catabolic\_process | 2 | 0 |  |  |  |  |  |  |  |  |
| GO:0032829\_regulation\_of\_CD4-positive\_\_CD25-positive\_\_alpha-beta\_regulatory\_T\_cell\_differentiation | 2 | 0 |  |  |  |  |  |  |  |  |
| GO:0032831\_positive\_regulation\_of\_CD4-positive\_\_CD25-positive\_\_alpha-beta\_regulatory\_T\_cell\_differentiation | 2 | 0 |  |  |  |  |  |  |  |  |
| GO:0032892\_positive\_regulation\_of\_organic\_acid\_transport | 2 | 0 |  |  |  |  |  |  |  |  |
| GO:0032905\_transforming\_growth\_factor-beta1\_production | 2 | 0 |  |  |  |  |  |  |  |  |
| GO:0032908\_regulation\_of\_transforming\_growth\_factor-beta1\_production | 2 | 0 |  |  |  |  |  |  |  |  |
| GO:0032914\_positive\_regulation\_of\_transforming\_growth\_factor-beta1\_production | 2 | 0 |  |  |  |  |  |  |  |  |
| GO:0032933\_SREBP-mediated\_signaling\_pathway | 2 | 0 |  |  |  |  |  |  |  |  |
| GO:0032957\_inositol\_trisphosphate\_metabolic\_process | 2 | 0 |  |  |  |  |  |  |  |  |
| GO:0032958\_inositol\_phosphate\_biosynthetic\_process | 2 | 0 |  |  |  |  |  |  |  |  |
| GO:0032959\_inositol\_trisphosphate\_biosynthetic\_process | 2 | 0 |  |  |  |  |  |  |  |  |
| GO:0033092\_positive\_regulation\_of\_immature\_T\_cell\_proliferation\_in\_the\_thymus | 2 | 0 |  |  |  |  |  |  |  |  |
| GO:0033119\_negative\_regulation\_of\_RNA\_splicing | 2 | 0 |  |  |  |  |  |  |  |  |
| GO:0033136\_serine\_phosphorylation\_of\_STAT3\_protein | 2 | 0 |  |  |  |  |  |  |  |  |
| GO:0033145\_positive\_regulation\_of\_steroid\_hormone\_receptor\_signaling\_pathway | 2 | 0 |  |  |  |  |  |  |  |  |
| GO:0033147\_negative\_regulation\_of\_estrogen\_receptor\_signaling\_pathway | 2 | 0 |  |  |  |  |  |  |  |  |
| GO:0033148\_positive\_regulation\_of\_estrogen\_receptor\_signaling\_pathway | 2 | 0 |  |  |  |  |  |  |  |  |
| GO:0033194\_response\_to\_hydroperoxide | 2 | 0 |  |  |  |  |  |  |  |  |
| GO:0033275\_actin-myosin\_filament\_sliding | 2 | 0 |  |  |  |  |  |  |  |  |
| GO:0033280\_response\_to\_vitamin\_D | 2 | 0 |  |  |  |  |  |  |  |  |
| GO:0033364\_mast\_cell\_secretory\_granule\_organization | 2 | 0 |  |  |  |  |  |  |  |  |
| GO:0033504\_floor\_plate\_development | 2 | 0 |  |  |  |  |  |  |  |  |
| GO:0033603\_positive\_regulation\_of\_dopamine\_secretion | 2 | 0 |  |  |  |  |  |  |  |  |
| GO:0033605\_positive\_regulation\_of\_catecholamine\_secretion | 2 | 0 |  |  |  |  |  |  |  |  |
| GO:0033700\_phospholipid\_efflux | 2 | 0 |  |  |  |  |  |  |  |  |
| GO:0034142\_toll-like\_receptor\_4\_signaling\_pathway | 2 | 0 |  |  |  |  |  |  |  |  |
| GO:0034310\_monohydric\_alcohol\_catabolic\_process | 2 | 0 |  |  |  |  |  |  |  |  |
| GO:0034341\_response\_to\_interferon-gamma | 2 | 0 |  |  |  |  |  |  |  |  |
| GO:0034377\_plasma\_lipoprotein\_particle\_assembly | 2 | 0 |  |  |  |  |  |  |  |  |
| GO:0034384\_high-density\_lipoprotein\_particle\_clearance | 2 | 0 |  |  |  |  |  |  |  |  |
| GO:0034453\_microtubule\_anchoring | 2 | 0 |  |  |  |  |  |  |  |  |
| GO:0034644\_cellular\_response\_to\_UV | 2 | 0 |  |  |  |  |  |  |  |  |
| GO:0034755\_iron\_ion\_transmembrane\_transport | 2 | 0 |  |  |  |  |  |  |  |  |
| GO:0034764\_positive\_regulation\_of\_transmembrane\_transport | 2 | 0 |  |  |  |  |  |  |  |  |
| GO:0035021\_negative\_regulation\_of\_Rac\_protein\_signal\_transduction | 2 | 0 |  |  |  |  |  |  |  |  |
| GO:0035054\_embryonic\_heart\_tube\_anterior\_posterior\_pattern\_formation | 2 | 0 |  |  |  |  |  |  |  |  |
| GO:0035092\_sperm\_chromatin\_condensation | 2 | 0 |  |  |  |  |  |  |  |  |
| GO:0035110\_leg\_morphogenesis | 2 | 0 |  |  |  |  |  |  |  |  |
| GO:0035120\_post-embryonic\_appendage\_morphogenesis | 2 | 0 |  |  |  |  |  |  |  |  |
| GO:0035127\_post-embryonic\_limb\_morphogenesis | 2 | 0 |  |  |  |  |  |  |  |  |
| GO:0035129\_post-embryonic\_hindlimb\_morphogenesis | 2 | 0 |  |  |  |  |  |  |  |  |
| GO:0035194\_posttranscriptional\_gene\_silencing\_by\_RNA | 2 | 0 |  |  |  |  |  |  |  |  |
| GO:0035195\_gene\_silencing\_by\_miRNA | 2 | 0 |  |  |  |  |  |  |  |  |
| GO:0035196\_gene\_silencing\_by\_miRNA\_\_production\_of\_miRNAs | 2 | 0 |  |  |  |  |  |  |  |  |
| GO:0035315\_hair\_cell\_differentiation | 2 | 0 |  |  |  |  |  |  |  |  |
| GO:0040009\_regulation\_of\_growth\_rate | 2 | 0 |  |  |  |  |  |  |  |  |
| GO:0040037\_negative\_regulation\_of\_fibroblast\_growth\_factor\_receptor\_signaling\_pathway | 2 | 0 |  |  |  |  |  |  |  |  |
| GO:0042119\_neutrophil\_activation | 2 | 0 |  |  |  |  |  |  |  |  |
| GO:0042147\_retrograde\_transport\_\_endosome\_to\_Golgi | 2 | 0 |  |  |  |  |  |  |  |  |
| GO:0042223\_interleukin-3\_biosynthetic\_process | 2 | 0 |  |  |  |  |  |  |  |  |
| GO:0042249\_establishment\_of\_polarity\_of\_embryonic\_epithelium | 2 | 0 |  |  |  |  |  |  |  |  |
| GO:0042253\_granulocyte\_macrophage\_colony-stimulating\_factor\_biosynthetic\_process | 2 | 0 |  |  |  |  |  |  |  |  |
| GO:0042270\_protection\_from\_natural\_killer\_cell\_mediated\_cytotoxicity | 2 | 0 |  |  |  |  |  |  |  |  |
| GO:0042274\_ribosomal\_small\_subunit\_biogenesis | 2 | 0 |  |  |  |  |  |  |  |  |
| GO:0042346\_positive\_regulation\_of\_NF-kappaB\_import\_into\_nucleus | 2 | 0 |  |  |  |  |  |  |  |  |
| GO:0042396\_phosphagen\_biosynthetic\_process | 2 | 0 |  |  |  |  |  |  |  |  |
| GO:0042454\_ribonucleoside\_catabolic\_process | 2 | 0 |  |  |  |  |  |  |  |  |
| GO:0042482\_positive\_regulation\_of\_odontogenesis | 2 | 0 |  |  |  |  |  |  |  |  |
| GO:0042483\_negative\_regulation\_of\_odontogenesis | 2 | 0 |  |  |  |  |  |  |  |  |
| GO:0042488\_positive\_regulation\_of\_odontogenesis\_of\_dentine-containing\_tooth | 2 | 0 |  |  |  |  |  |  |  |  |
| GO:0042501\_serine\_phosphorylation\_of\_STAT\_protein | 2 | 0 |  |  |  |  |  |  |  |  |
| GO:0042517\_positive\_regulation\_of\_tyrosine\_phosphorylation\_of\_Stat3\_protein | 2 | 0 |  |  |  |  |  |  |  |  |
| GO:0042532\_negative\_regulation\_of\_tyrosine\_phosphorylation\_of\_STAT\_protein | 2 | 0 |  |  |  |  |  |  |  |  |
| GO:0042559\_pteridine\_and\_derivative\_biosynthetic\_process | 2 | 0 |  |  |  |  |  |  |  |  |
| GO:0042730\_fibrinolysis | 2 | 0 |  |  |  |  |  |  |  |  |
| GO:0042749\_regulation\_of\_circadian\_sleep\_wake\_cycle | 2 | 0 |  |  |  |  |  |  |  |  |
| GO:0042886\_amide\_transport | 2 | 0 |  |  |  |  |  |  |  |  |
| GO:0042921\_glucocorticoid\_receptor\_signaling\_pathway | 2 | 0 |  |  |  |  |  |  |  |  |
| GO:0042987\_amyloid\_precursor\_protein\_catabolic\_process | 2 | 0 |  |  |  |  |  |  |  |  |
| GO:0042993\_positive\_regulation\_of\_transcription\_factor\_import\_into\_nucleus | 2 | 0 |  |  |  |  |  |  |  |  |
| GO:0042994\_cytoplasmic\_sequestering\_of\_transcription\_factor | 2 | 0 |  |  |  |  |  |  |  |  |
| GO:0043032\_positive\_regulation\_of\_macrophage\_activation | 2 | 0 |  |  |  |  |  |  |  |  |
| GO:0043038\_amino\_acid\_activation | 2 | 0 |  |  |  |  |  |  |  |  |
| GO:0043039\_tRNA\_aminoacylation | 2 | 0 |  |  |  |  |  |  |  |  |
| GO:0043084\_penile\_erection | 2 | 0 |  |  |  |  |  |  |  |  |
| GO:0043088\_regulation\_of\_Cdc42\_GTPase\_activity | 2 | 0 |  |  |  |  |  |  |  |  |
| GO:0043089\_positive\_regulation\_of\_Cdc42\_GTPase\_activity | 2 | 0 |  |  |  |  |  |  |  |  |
| GO:0043096\_purine\_base\_salvage | 2 | 0 |  |  |  |  |  |  |  |  |
| GO:0043247\_telomere\_maintenance\_in\_response\_to\_DNA\_damage | 2 | 0 |  |  |  |  |  |  |  |  |
| GO:0043297\_apical\_junction\_assembly | 2 | 0 |  |  |  |  |  |  |  |  |
| GO:0043312\_neutrophil\_degranulation | 2 | 0 |  |  |  |  |  |  |  |  |
| GO:0043320\_natural\_killer\_cell\_degranulation | 2 | 0 |  |  |  |  |  |  |  |  |
| GO:0043366\_beta\_selection | 2 | 0 |  |  |  |  |  |  |  |  |
| GO:0043450\_alkene\_biosynthetic\_process | 2 | 0 |  |  |  |  |  |  |  |  |
| GO:0043476\_pigment\_accumulation | 2 | 0 |  |  |  |  |  |  |  |  |
| GO:0043490\_malate-aspartate\_shuttle | 2 | 0 |  |  |  |  |  |  |  |  |
| GO:0043502\_regulation\_of\_muscle\_adaptation | 2 | 0 |  |  |  |  |  |  |  |  |
| GO:0043516\_regulation\_of\_DNA\_damage\_response\_\_signal\_transduction\_by\_p53\_class\_mediator | 2 | 0 |  |  |  |  |  |  |  |  |
| GO:0043568\_positive\_regulation\_of\_insulin-like\_growth\_factor\_receptor\_signaling\_pathway | 2 | 0 |  |  |  |  |  |  |  |  |
| GO:0043589\_skin\_morphogenesis | 2 | 0 |  |  |  |  |  |  |  |  |
| GO:0043618\_regulation\_of\_transcription\_from\_RNA\_polymerase\_II\_promoter\_in\_response\_to\_stress | 2 | 0 |  |  |  |  |  |  |  |  |
| GO:0043619\_regulation\_of\_transcription\_from\_RNA\_polymerase\_II\_promoter\_in\_response\_to\_oxidative\_stress | 2 | 0 |  |  |  |  |  |  |  |  |
| GO:0043620\_regulation\_of\_transcription\_in\_response\_to\_stress | 2 | 0 |  |  |  |  |  |  |  |  |
| GO:0043647\_inositol\_phosphate\_metabolic\_process | 2 | 0 |  |  |  |  |  |  |  |  |
| GO:0043654\_recognition\_of\_apoptotic\_cell | 2 | 0 |  |  |  |  |  |  |  |  |
| GO:0043966\_histone\_H3\_acetylation | 2 | 0 |  |  |  |  |  |  |  |  |
| GO:0043967\_histone\_H4\_acetylation | 2 | 0 |  |  |  |  |  |  |  |  |
| GO:0044070\_regulation\_of\_anion\_transport | 2 | 0 |  |  |  |  |  |  |  |  |
| GO:0044246\_regulation\_of\_multicellular\_organismal\_metabolic\_process | 2 | 0 |  |  |  |  |  |  |  |  |
| GO:0044253\_positive\_regulation\_of\_multicellular\_organismal\_metabolic\_process | 2 | 0 |  |  |  |  |  |  |  |  |
| GO:0044268\_multicellular\_organismal\_protein\_metabolic\_process | 2 | 0 |  |  |  |  |  |  |  |  |
| GO:0045005\_maintenance\_of\_fidelity\_during\_DNA-dependent\_DNA\_replication | 2 | 0 |  |  |  |  |  |  |  |  |
| GO:0045010\_actin\_nucleation | 2 | 0 |  |  |  |  |  |  |  |  |
| GO:0045065\_cytotoxic\_T\_cell\_differentiation | 2 | 0 |  |  |  |  |  |  |  |  |
| GO:0045077\_negative\_regulation\_of\_interferon-gamma\_biosynthetic\_process | 2 | 0 |  |  |  |  |  |  |  |  |
| GO:0045079\_negative\_regulation\_of\_chemokine\_biosynthetic\_process | 2 | 0 |  |  |  |  |  |  |  |  |
| GO:0045116\_protein\_neddylation | 2 | 0 |  |  |  |  |  |  |  |  |
| GO:0045187\_regulation\_of\_circadian\_sleep\_wake\_cycle\_\_sleep | 2 | 0 |  |  |  |  |  |  |  |  |
| GO:0045212\_neurotransmitter\_receptor\_biosynthetic\_process | 2 | 0 |  |  |  |  |  |  |  |  |
| GO:0045399\_regulation\_of\_interleukin-3\_biosynthetic\_process | 2 | 0 |  |  |  |  |  |  |  |  |
| GO:0045401\_positive\_regulation\_of\_interleukin-3\_biosynthetic\_process | 2 | 0 |  |  |  |  |  |  |  |  |
| GO:0045409\_negative\_regulation\_of\_interleukin-6\_biosynthetic\_process | 2 | 0 |  |  |  |  |  |  |  |  |
| GO:0045423\_regulation\_of\_granulocyte\_macrophage\_colony-stimulating\_factor\_biosynthetic\_process | 2 | 0 |  |  |  |  |  |  |  |  |
| GO:0045425\_positive\_regulation\_of\_granulocyte\_macrophage\_colony-stimulating\_factor\_biosynthetic\_process | 2 | 0 |  |  |  |  |  |  |  |  |
| GO:0045475\_locomotor\_rhythm | 2 | 0 |  |  |  |  |  |  |  |  |
| GO:0045578\_negative\_regulation\_of\_B\_cell\_differentiation | 2 | 0 |  |  |  |  |  |  |  |  |
| GO:0045589\_regulation\_of\_regulatory\_T\_cell\_differentiation | 2 | 0 |  |  |  |  |  |  |  |  |
| GO:0045591\_positive\_regulation\_of\_regulatory\_T\_cell\_differentiation | 2 | 0 |  |  |  |  |  |  |  |  |
| GO:0045627\_positive\_regulation\_of\_T-helper\_1\_cell\_differentiation | 2 | 0 |  |  |  |  |  |  |  |  |
| GO:0045629\_negative\_regulation\_of\_T-helper\_2\_cell\_differentiation | 2 | 0 |  |  |  |  |  |  |  |  |
| GO:0045630\_positive\_regulation\_of\_T-helper\_2\_cell\_differentiation | 2 | 0 |  |  |  |  |  |  |  |  |
| GO:0045636\_positive\_regulation\_of\_melanocyte\_differentiation | 2 | 0 |  |  |  |  |  |  |  |  |
| GO:0045655\_regulation\_of\_monocyte\_differentiation | 2 | 0 |  |  |  |  |  |  |  |  |
| GO:0045658\_regulation\_of\_neutrophil\_differentiation | 2 | 0 |  |  |  |  |  |  |  |  |
| GO:0045662\_negative\_regulation\_of\_myoblast\_differentiation | 2 | 0 |  |  |  |  |  |  |  |  |
| GO:0045663\_positive\_regulation\_of\_myoblast\_differentiation | 2 | 0 |  |  |  |  |  |  |  |  |
| GO:0045683\_negative\_regulation\_of\_epidermis\_development | 2 | 0 |  |  |  |  |  |  |  |  |
| GO:0045737\_positive\_regulation\_of\_cyclin-dependent\_protein\_kinase\_activity | 2 | 0 |  |  |  |  |  |  |  |  |
| GO:0045739\_positive\_regulation\_of\_DNA\_repair | 2 | 0 |  |  |  |  |  |  |  |  |
| GO:0045741\_positive\_regulation\_of\_epidermal\_growth\_factor\_receptor\_activity | 2 | 0 |  |  |  |  |  |  |  |  |
| GO:0045743\_positive\_regulation\_of\_fibroblast\_growth\_factor\_receptor\_signaling\_pathway | 2 | 0 |  |  |  |  |  |  |  |  |
| GO:0045749\_negative\_regulation\_of\_S\_phase\_of\_mitotic\_cell\_cycle | 2 | 0 |  |  |  |  |  |  |  |  |
| GO:0045819\_positive\_regulation\_of\_glycogen\_catabolic\_process | 2 | 0 |  |  |  |  |  |  |  |  |
| GO:0045821\_positive\_regulation\_of\_glycolysis | 2 | 0 |  |  |  |  |  |  |  |  |
| GO:0045835\_negative\_regulation\_of\_meiosis | 2 | 0 |  |  |  |  |  |  |  |  |
| GO:0045836\_positive\_regulation\_of\_meiosis | 2 | 0 |  |  |  |  |  |  |  |  |
| GO:0045839\_negative\_regulation\_of\_mitosis | 2 | 0 |  |  |  |  |  |  |  |  |
| GO:0045841\_negative\_regulation\_of\_mitotic\_metaphase\_anaphase\_transition | 2 | 0 |  |  |  |  |  |  |  |  |
| GO:0045872\_positive\_regulation\_of\_rhodopsin\_gene\_expression | 2 | 0 |  |  |  |  |  |  |  |  |
| GO:0045912\_negative\_regulation\_of\_carbohydrate\_metabolic\_process | 2 | 0 |  |  |  |  |  |  |  |  |
| GO:0045948\_positive\_regulation\_of\_translational\_initiation | 2 | 0 |  |  |  |  |  |  |  |  |
| GO:0045950\_negative\_regulation\_of\_mitotic\_recombination | 2 | 0 |  |  |  |  |  |  |  |  |
| GO:0046033\_AMP\_metabolic\_process | 2 | 0 |  |  |  |  |  |  |  |  |
| GO:0046060\_dATP\_metabolic\_process | 2 | 0 |  |  |  |  |  |  |  |  |
| GO:0046070\_dGTP\_metabolic\_process | 2 | 0 |  |  |  |  |  |  |  |  |
| GO:0046083\_adenine\_metabolic\_process | 2 | 0 |  |  |  |  |  |  |  |  |
| GO:0046085\_adenosine\_metabolic\_process | 2 | 0 |  |  |  |  |  |  |  |  |
| GO:0046100\_hypoxanthine\_metabolic\_process | 2 | 0 |  |  |  |  |  |  |  |  |
| GO:0046114\_guanosine\_biosynthetic\_process | 2 | 0 |  |  |  |  |  |  |  |  |
| GO:0046116\_queuosine\_metabolic\_process | 2 | 0 |  |  |  |  |  |  |  |  |
| GO:0046118\_7-methylguanosine\_biosynthetic\_process | 2 | 0 |  |  |  |  |  |  |  |  |
| GO:0046130\_purine\_ribonucleoside\_catabolic\_process | 2 | 0 |  |  |  |  |  |  |  |  |
| GO:0046146\_tetrahydrobiopterin\_metabolic\_process | 2 | 0 |  |  |  |  |  |  |  |  |
| GO:0046185\_aldehyde\_catabolic\_process | 2 | 0 |  |  |  |  |  |  |  |  |
| GO:0046208\_spermine\_catabolic\_process | 2 | 0 |  |  |  |  |  |  |  |  |
| GO:0046349\_amino\_sugar\_biosynthetic\_process | 2 | 0 |  |  |  |  |  |  |  |  |
| GO:0046439\_L-cysteine\_metabolic\_process | 2 | 0 |  |  |  |  |  |  |  |  |
| GO:0046500\_S-adenosylmethionine\_metabolic\_process | 2 | 0 |  |  |  |  |  |  |  |  |
| GO:0046501\_protoporphyrinogen\_IX\_metabolic\_process | 2 | 0 |  |  |  |  |  |  |  |  |
| GO:0046514\_ceramide\_catabolic\_process | 2 | 0 |  |  |  |  |  |  |  |  |
| GO:0046521\_sphingoid\_catabolic\_process | 2 | 0 |  |  |  |  |  |  |  |  |
| GO:0046532\_regulation\_of\_photoreceptor\_cell\_differentiation | 2 | 0 |  |  |  |  |  |  |  |  |
| GO:0046533\_negative\_regulation\_of\_photoreceptor\_cell\_differentiation | 2 | 0 |  |  |  |  |  |  |  |  |
| GO:0046544\_development\_of\_secondary\_male\_sexual\_characteristics | 2 | 0 |  |  |  |  |  |  |  |  |
| GO:0046950\_cellular\_ketone\_body\_metabolic\_process | 2 | 0 |  |  |  |  |  |  |  |  |
| GO:0046984\_regulation\_of\_hemoglobin\_biosynthetic\_process | 2 | 0 |  |  |  |  |  |  |  |  |
| GO:0047484\_regulation\_of\_response\_to\_osmotic\_stress | 2 | 0 |  |  |  |  |  |  |  |  |
| GO:0048025\_negative\_regulation\_of\_nuclear\_mRNA\_splicing\_\_via\_spliceosome | 2 | 0 |  |  |  |  |  |  |  |  |
| GO:0048172\_regulation\_of\_short-term\_neuronal\_synaptic\_plasticity | 2 | 0 |  |  |  |  |  |  |  |  |
| GO:0048295\_positive\_regulation\_of\_isotype\_switching\_to\_IgE\_isotypes | 2 | 0 |  |  |  |  |  |  |  |  |
| GO:0048342\_paraxial\_mesodermal\_cell\_differentiation | 2 | 0 |  |  |  |  |  |  |  |  |
| GO:0048343\_paraxial\_mesodermal\_cell\_fate\_commitment | 2 | 0 |  |  |  |  |  |  |  |  |
| GO:0048382\_mesendoderm\_development | 2 | 0 |  |  |  |  |  |  |  |  |
| GO:0048552\_regulation\_of\_metalloenzyme\_activity | 2 | 0 |  |  |  |  |  |  |  |  |
| GO:0048554\_positive\_regulation\_of\_metalloenzyme\_activity | 2 | 0 |  |  |  |  |  |  |  |  |
| GO:0048619\_embryonic\_hindgut\_morphogenesis | 2 | 0 |  |  |  |  |  |  |  |  |
| GO:0048625\_myoblast\_cell\_fate\_commitment | 2 | 0 |  |  |  |  |  |  |  |  |
| GO:0048627\_myoblast\_development | 2 | 0 |  |  |  |  |  |  |  |  |
| GO:0048643\_positive\_regulation\_of\_skeletal\_muscle\_tissue\_development | 2 | 0 |  |  |  |  |  |  |  |  |
| GO:0048661\_positive\_regulation\_of\_smooth\_muscle\_cell\_proliferation | 2 | 0 |  |  |  |  |  |  |  |  |
| GO:0048670\_regulation\_of\_collateral\_sprouting | 2 | 0 |  |  |  |  |  |  |  |  |
| GO:0048671\_negative\_regulation\_of\_collateral\_sprouting | 2 | 0 |  |  |  |  |  |  |  |  |
| GO:0048677\_axon\_extension\_involved\_in\_regeneration | 2 | 0 |  |  |  |  |  |  |  |  |
| GO:0048679\_regulation\_of\_axon\_regeneration | 2 | 0 |  |  |  |  |  |  |  |  |
| GO:0048682\_sprouting\_of\_injured\_axon | 2 | 0 |  |  |  |  |  |  |  |  |
| GO:0048702\_embryonic\_neurocranium\_morphogenesis | 2 | 0 |  |  |  |  |  |  |  |  |
| GO:0048712\_negative\_regulation\_of\_astrocyte\_differentiation | 2 | 0 |  |  |  |  |  |  |  |  |
| GO:0048739\_cardiac\_muscle\_fiber\_development | 2 | 0 |  |  |  |  |  |  |  |  |
| GO:0048808\_male\_genitalia\_morphogenesis | 2 | 0 |  |  |  |  |  |  |  |  |
| GO:0048840\_otolith\_development | 2 | 0 |  |  |  |  |  |  |  |  |
| GO:0048867\_stem\_cell\_fate\_determination | 2 | 0 |  |  |  |  |  |  |  |  |
| GO:0050000\_chromosome\_localization | 2 | 0 |  |  |  |  |  |  |  |  |
| GO:0050686\_negative\_regulation\_of\_mRNA\_processing | 2 | 0 |  |  |  |  |  |  |  |  |
| GO:0050688\_regulation\_of\_defense\_response\_to\_virus | 2 | 0 |  |  |  |  |  |  |  |  |
| GO:0050746\_regulation\_of\_lipoprotein\_metabolic\_process | 2 | 0 |  |  |  |  |  |  |  |  |
| GO:0050779\_RNA\_destabilization | 2 | 0 |  |  |  |  |  |  |  |  |
| GO:0050792\_regulation\_of\_viral\_reproduction | 2 | 0 |  |  |  |  |  |  |  |  |
| GO:0050802\_circadian\_sleep\_wake\_cycle\_\_sleep | 2 | 0 |  |  |  |  |  |  |  |  |
| GO:0050847\_progesterone\_receptor\_signaling\_pathway | 2 | 0 |  |  |  |  |  |  |  |  |
| GO:0050855\_regulation\_of\_B\_cell\_receptor\_signaling\_pathway | 2 | 0 |  |  |  |  |  |  |  |  |
| GO:0050883\_musculoskeletal\_movement\_\_spinal\_reflex\_action | 2 | 0 |  |  |  |  |  |  |  |  |
| GO:0050901\_leukocyte\_tethering\_or\_rolling | 2 | 0 |  |  |  |  |  |  |  |  |
| GO:0050907\_detection\_of\_chemical\_stimulus\_involved\_in\_sensory\_perception | 2 | 0 |  |  |  |  |  |  |  |  |
| GO:0050917\_sensory\_perception\_of\_umami\_taste | 2 | 0 |  |  |  |  |  |  |  |  |
| GO:0050942\_positive\_regulation\_of\_pigment\_cell\_differentiation | 2 | 0 |  |  |  |  |  |  |  |  |
| GO:0050955\_thermoception | 2 | 0 |  |  |  |  |  |  |  |  |
| GO:0050968\_detection\_of\_chemical\_stimulus\_involved\_in\_sensory\_perception\_of\_pain | 2 | 0 |  |  |  |  |  |  |  |  |
| GO:0050973\_detection\_of\_mechanical\_stimulus\_involved\_in\_equilibrioception | 2 | 0 |  |  |  |  |  |  |  |  |
| GO:0050999\_regulation\_of\_nitric-oxide\_synthase\_activity | 2 | 0 |  |  |  |  |  |  |  |  |
| GO:0051004\_regulation\_of\_lipoprotein\_lipase\_activity | 2 | 0 |  |  |  |  |  |  |  |  |
| GO:0051014\_actin\_filament\_severing | 2 | 0 |  |  |  |  |  |  |  |  |
| GO:0051026\_chiasma\_formation | 2 | 0 |  |  |  |  |  |  |  |  |
| GO:0051081\_nuclear\_envelope\_disassembly | 2 | 0 |  |  |  |  |  |  |  |  |
| GO:0051132\_NK\_T\_cell\_activation | 2 | 0 |  |  |  |  |  |  |  |  |
| GO:0051133\_regulation\_of\_NK\_T\_cell\_activation | 2 | 0 |  |  |  |  |  |  |  |  |
| GO:0051135\_positive\_regulation\_of\_NK\_T\_cell\_activation | 2 | 0 |  |  |  |  |  |  |  |  |
| GO:0051150\_regulation\_of\_smooth\_muscle\_cell\_differentiation | 2 | 0 |  |  |  |  |  |  |  |  |
| GO:0051220\_cytoplasmic\_sequestering\_of\_protein | 2 | 0 |  |  |  |  |  |  |  |  |
| GO:0051279\_regulation\_of\_release\_of\_sequestered\_calcium\_ion\_into\_cytosol | 2 | 0 |  |  |  |  |  |  |  |  |
| GO:0051293\_establishment\_of\_spindle\_localization | 2 | 0 |  |  |  |  |  |  |  |  |
| GO:0051295\_establishment\_of\_meiotic\_spindle\_localization | 2 | 0 |  |  |  |  |  |  |  |  |
| GO:0051299\_centrosome\_separation | 2 | 0 |  |  |  |  |  |  |  |  |
| GO:0051303\_establishment\_of\_chromosome\_localization | 2 | 0 |  |  |  |  |  |  |  |  |
| GO:0051304\_chromosome\_separation | 2 | 0 |  |  |  |  |  |  |  |  |
| GO:0051307\_meiotic\_chromosome\_separation | 2 | 0 |  |  |  |  |  |  |  |  |
| GO:0051313\_attachment\_of\_spindle\_microtubules\_to\_chromosome | 2 | 0 |  |  |  |  |  |  |  |  |
| GO:0051318\_G1\_phase | 2 | 0 |  |  |  |  |  |  |  |  |
| GO:0051319\_G2\_phase | 2 | 0 |  |  |  |  |  |  |  |  |
| GO:0051353\_positive\_regulation\_of\_oxidoreductase\_activity | 2 | 0 |  |  |  |  |  |  |  |  |
| GO:0051451\_myoblast\_migration | 2 | 0 |  |  |  |  |  |  |  |  |
| GO:0051489\_regulation\_of\_filopodium\_assembly | 2 | 0 |  |  |  |  |  |  |  |  |
| GO:0051491\_positive\_regulation\_of\_filopodium\_assembly | 2 | 0 |  |  |  |  |  |  |  |  |
| GO:0051541\_elastin\_metabolic\_process | 2 | 0 |  |  |  |  |  |  |  |  |
| GO:0051546\_keratinocyte\_migration | 2 | 0 |  |  |  |  |  |  |  |  |
| GO:0051563\_smooth\_endoplasmic\_reticulum\_calcium\_ion\_homeostasis | 2 | 0 |  |  |  |  |  |  |  |  |
| GO:0051590\_positive\_regulation\_of\_neurotransmitter\_transport | 2 | 0 |  |  |  |  |  |  |  |  |
| GO:0051602\_response\_to\_electrical\_stimulus | 2 | 0 |  |  |  |  |  |  |  |  |
| GO:0051608\_histamine\_transport | 2 | 0 |  |  |  |  |  |  |  |  |
| GO:0051643\_ER\_localization | 2 | 0 |  |  |  |  |  |  |  |  |
| GO:0051653\_spindle\_localization | 2 | 0 |  |  |  |  |  |  |  |  |
| GO:0051657\_maintenance\_of\_organelle\_location | 2 | 0 |  |  |  |  |  |  |  |  |
| GO:0051702\_interaction\_with\_symbiont | 2 | 0 |  |  |  |  |  |  |  |  |
| GO:0051781\_positive\_regulation\_of\_cell\_division | 2 | 0 |  |  |  |  |  |  |  |  |
| GO:0051784\_negative\_regulation\_of\_nuclear\_division | 2 | 0 |  |  |  |  |  |  |  |  |
| GO:0051890\_regulation\_of\_cardioblast\_differentiation | 2 | 0 |  |  |  |  |  |  |  |  |
| GO:0051891\_positive\_regulation\_of\_cardioblast\_differentiation | 2 | 0 |  |  |  |  |  |  |  |  |
| GO:0051923\_sulfation | 2 | 0 |  |  |  |  |  |  |  |  |
| GO:0051938\_L-glutamate\_import | 2 | 0 |  |  |  |  |  |  |  |  |
| GO:0051957\_positive\_regulation\_of\_amino\_acid\_transport | 2 | 0 |  |  |  |  |  |  |  |  |
| GO:0051988\_regulation\_of\_attachment\_of\_spindle\_microtubules\_to\_kinetochore | 2 | 0 |  |  |  |  |  |  |  |  |
| GO:0055057\_neuroblast\_division | 2 | 0 |  |  |  |  |  |  |  |  |
| GO:0055064\_chloride\_ion\_homeostasis | 2 | 0 |  |  |  |  |  |  |  |  |
| GO:0055075\_potassium\_ion\_homeostasis | 2 | 0 |  |  |  |  |  |  |  |  |
| GO:0055090\_acylglycerol\_homeostasis | 2 | 0 |  |  |  |  |  |  |  |  |
| GO:0055091\_phospholipid\_homeostasis | 2 | 0 |  |  |  |  |  |  |  |  |
| GO:0060012\_synaptic\_transmission\_\_glycinergic | 2 | 0 |  |  |  |  |  |  |  |  |
| GO:0060023\_soft\_palate\_development | 2 | 0 |  |  |  |  |  |  |  |  |
| GO:0060032\_notochord\_regression | 2 | 0 |  |  |  |  |  |  |  |  |
| GO:0060039\_pericardium\_development | 2 | 0 |  |  |  |  |  |  |  |  |
| GO:0060060\_post-embryonic\_retina\_morphogenesis\_in\_camera-type\_eye | 2 | 0 |  |  |  |  |  |  |  |  |
| GO:0060083\_smooth\_muscle\_contraction\_involved\_in\_micturition | 2 | 0 |  |  |  |  |  |  |  |  |
| GO:0060124\_positive\_regulation\_of\_growth\_hormone\_secretion | 2 | 0 |  |  |  |  |  |  |  |  |
| GO:0060133\_somatotropin\_secreting\_cell\_development | 2 | 0 |  |  |  |  |  |  |  |  |
| GO:0060155\_platelet\_dense\_granule\_organization | 2 | 0 |  |  |  |  |  |  |  |  |
| GO:0060159\_regulation\_of\_dopamine\_receptor\_signaling\_pathway | 2 | 0 |  |  |  |  |  |  |  |  |
| GO:0060160\_negative\_regulation\_of\_dopamine\_receptor\_signaling\_pathway | 2 | 0 |  |  |  |  |  |  |  |  |
| GO:0060166\_olfactory\_pit\_development | 2 | 0 |  |  |  |  |  |  |  |  |
| GO:0060179\_male\_mating\_behavior | 2 | 0 |  |  |  |  |  |  |  |  |
| GO:0060180\_female\_mating\_behavior | 2 | 0 |  |  |  |  |  |  |  |  |
| GO:0060218\_hemopoietic\_stem\_cell\_differentiation | 2 | 0 |  |  |  |  |  |  |  |  |
| GO:0060259\_regulation\_of\_feeding\_behavior | 2 | 0 |  |  |  |  |  |  |  |  |
| GO:0060260\_regulation\_of\_transcription\_initiation\_from\_RNA\_polymerase\_II\_promoter | 2 | 0 |  |  |  |  |  |  |  |  |
| GO:0060292\_long\_term\_synaptic\_depression | 2 | 0 |  |  |  |  |  |  |  |  |
| GO:0060318\_definitive\_erythrocyte\_differentiation | 2 | 0 |  |  |  |  |  |  |  |  |
| GO:0060346\_bone\_trabecula\_formation | 2 | 0 |  |  |  |  |  |  |  |  |
| GO:0060363\_cranial\_suture\_morphogenesis | 2 | 0 |  |  |  |  |  |  |  |  |
| GO:0060393\_regulation\_of\_pathway-restricted\_SMAD\_protein\_phosphorylation | 2 | 0 |  |  |  |  |  |  |  |  |
| GO:0060397\_JAK-STAT\_cascade\_involved\_in\_growth\_hormone\_signaling\_pathway | 2 | 0 |  |  |  |  |  |  |  |  |
| GO:0060426\_lung\_vasculature\_development | 2 | 0 |  |  |  |  |  |  |  |  |
| GO:0060430\_lung\_saccule\_development | 2 | 0 |  |  |  |  |  |  |  |  |
| GO:0060434\_bronchus\_morphogenesis | 2 | 0 |  |  |  |  |  |  |  |  |
| GO:0060439\_trachea\_morphogenesis | 2 | 0 |  |  |  |  |  |  |  |  |
| GO:0060458\_right\_lung\_development | 2 | 0 |  |  |  |  |  |  |  |  |
| GO:0060462\_lung\_lobe\_development | 2 | 0 |  |  |  |  |  |  |  |  |
| GO:0060463\_lung\_lobe\_morphogenesis | 2 | 0 |  |  |  |  |  |  |  |  |
| GO:0060479\_lung\_cell\_differentiation | 2 | 0 |  |  |  |  |  |  |  |  |
| GO:0060487\_lung\_epithelial\_cell\_differentiation | 2 | 0 |  |  |  |  |  |  |  |  |
| GO:0060516\_primary\_prostatic\_bud\_elongation | 2 | 0 |  |  |  |  |  |  |  |  |
| GO:0060529\_squamous\_basal\_epithelial\_stem\_cell\_differentiation\_involved\_in\_prostate\_gland\_acinus\_development | 2 | 0 |  |  |  |  |  |  |  |  |
| GO:0060534\_trachea\_cartilage\_development | 2 | 0 |  |  |  |  |  |  |  |  |
| GO:0060612\_adipose\_tissue\_development | 2 | 0 |  |  |  |  |  |  |  |  |
| GO:0060615\_mammary\_gland\_bud\_formation | 2 | 0 |  |  |  |  |  |  |  |  |
| GO:0060667\_branch\_elongation\_involved\_in\_salivary\_gland\_morphogenesis | 2 | 0 |  |  |  |  |  |  |  |  |
| GO:0060690\_epithelial\_cell\_differentiation\_involved\_in\_salivary\_gland\_development | 2 | 0 |  |  |  |  |  |  |  |  |
| GO:0060738\_epithelial-mesenchymal\_signaling\_involved\_in\_prostate\_gland\_development | 2 | 0 |  |  |  |  |  |  |  |  |
| GO:0060741\_prostate\_gland\_stromal\_morphogenesis | 2 | 0 |  |  |  |  |  |  |  |  |
| GO:0060763\_mammary\_duct\_terminal\_end\_bud\_growth | 2 | 0 |  |  |  |  |  |  |  |  |
| GO:0060765\_regulation\_of\_androgen\_receptor\_signaling\_pathway | 2 | 0 |  |  |  |  |  |  |  |  |
| GO:0060766\_negative\_regulation\_of\_androgen\_receptor\_signaling\_pathway | 2 | 0 |  |  |  |  |  |  |  |  |
| GO:0065005\_protein-lipid\_complex\_assembly | 2 | 0 |  |  |  |  |  |  |  |  |
| GO:0070076\_histone\_lysine\_demethylation | 2 | 0 |  |  |  |  |  |  |  |  |
| GO:0070168\_negative\_regulation\_of\_biomineral\_formation | 2 | 0 |  |  |  |  |  |  |  |  |
| GO:0070252\_actin-mediated\_cell\_contraction | 2 | 0 |  |  |  |  |  |  |  |  |
| GO:0070256\_negative\_regulation\_of\_mucus\_secretion | 2 | 0 |  |  |  |  |  |  |  |  |
| GO:0070257\_positive\_regulation\_of\_mucus\_secretion | 2 | 0 |  |  |  |  |  |  |  |  |
| GO:0070570\_regulation\_of\_neuron\_projection\_regeneration | 2 | 0 |  |  |  |  |  |  |  |  |
| GO:0070723\_response\_to\_cholesterol | 2 | 0 |  |  |  |  |  |  |  |  |
| GO:0090030\_regulation\_of\_steroid\_hormone\_biosynthetic\_process | 2 | 0 |  |  |  |  |  |  |  |  |
| GO:0006643\_membrane\_lipid\_metabolic\_process | 33 | 0 | 0.000000 | -0.000000 | 984 | 692.740159 | 797.94 | 903.139841 | 0.810915 |  |
| GO:0007188\_G-protein\_signaling\_\_coupled\_to\_cAMP\_nucleotide\_second\_messenger | 33 | 0 | 0.000000 | -0.000000 | 984 | 692.740159 | 797.94 | 903.139841 | 0.810915 |  |
| GO:0007270\_nerve-nerve\_synaptic\_transmission | 33 | 0 | 0.000000 | -0.000000 | 984 | 692.740159 | 797.94 | 903.139841 | 0.810915 |  |
| GO:0007431\_salivary\_gland\_development | 33 | 0 | 0.000000 | -0.000000 | 984 | 692.740159 | 797.94 | 903.139841 | 0.810915 |  |
| GO:0008643\_carbohydrate\_transport | 33 | 0 | 0.000000 | -0.000000 | 984 | 692.740159 | 797.94 | 903.139841 | 0.810915 |  |
| GO:0042108\_positive\_regulation\_of\_cytokine\_biosynthetic\_process | 33 | 0 | 0.000000 | -0.000000 | 984 | 692.740159 | 797.94 | 903.139841 | 0.810915 |  |
| GO:0000086\_G2\_M\_transition\_of\_mitotic\_cell\_cycle | 4 | 0 |  |  |  |  |  |  |  |  |
| GO:0000305\_response\_to\_oxygen\_radical | 4 | 0 |  |  |  |  |  |  |  |  |
| GO:0001661\_conditioned\_taste\_aversion | 4 | 0 |  |  |  |  |  |  |  |  |
| GO:0001678\_cellular\_glucose\_homeostasis | 4 | 0 |  |  |  |  |  |  |  |  |
| GO:0001777\_T\_cell\_homeostatic\_proliferation | 4 | 0 |  |  |  |  |  |  |  |  |
| GO:0001794\_type\_IIa\_hypersensitivity | 4 | 0 |  |  |  |  |  |  |  |  |
| GO:0001796\_regulation\_of\_type\_IIa\_hypersensitivity | 4 | 0 |  |  |  |  |  |  |  |  |
| GO:0001798\_positive\_regulation\_of\_type\_IIa\_hypersensitivity | 4 | 0 |  |  |  |  |  |  |  |  |
| GO:0001810\_regulation\_of\_type\_I\_hypersensitivity | 4 | 0 |  |  |  |  |  |  |  |  |
| GO:0001820\_serotonin\_secretion | 4 | 0 |  |  |  |  |  |  |  |  |
| GO:0001835\_blastocyst\_hatching | 4 | 0 |  |  |  |  |  |  |  |  |
| GO:0001842\_neural\_fold\_formation | 4 | 0 |  |  |  |  |  |  |  |  |
| GO:0001881\_receptor\_recycling | 4 | 0 |  |  |  |  |  |  |  |  |
| GO:0001938\_positive\_regulation\_of\_endothelial\_cell\_proliferation | 4 | 0 |  |  |  |  |  |  |  |  |
| GO:0001978\_regulation\_of\_systemic\_arterial\_blood\_pressure\_by\_carotid\_sinus\_baroreceptor\_feedback | 4 | 0 |  |  |  |  |  |  |  |  |
| GO:0002035\_brain\_renin-angiotensin\_system | 4 | 0 |  |  |  |  |  |  |  |  |
| GO:0002051\_osteoblast\_fate\_commitment | 4 | 0 |  |  |  |  |  |  |  |  |
| GO:0002220\_innate\_immune\_response\_activating\_cell\_surface\_receptor\_signaling\_pathway | 4 | 0 |  |  |  |  |  |  |  |  |
| GO:0002249\_lymphocyte\_anergy | 4 | 0 |  |  |  |  |  |  |  |  |
| GO:0002312\_B\_cell\_activation\_during\_immune\_response | 4 | 0 |  |  |  |  |  |  |  |  |
| GO:0002313\_mature\_B\_cell\_differentiation\_during\_immune\_response | 4 | 0 |  |  |  |  |  |  |  |  |
| GO:0002318\_myeloid\_progenitor\_cell\_differentiation | 4 | 0 |  |  |  |  |  |  |  |  |
| GO:0002326\_B\_cell\_lineage\_commitment | 4 | 0 |  |  |  |  |  |  |  |  |
| GO:0002347\_response\_to\_tumor\_cell | 4 | 0 |  |  |  |  |  |  |  |  |
| GO:0002418\_immune\_response\_to\_tumor\_cell | 4 | 0 |  |  |  |  |  |  |  |  |
| GO:0002445\_type\_II\_hypersensitivity | 4 | 0 |  |  |  |  |  |  |  |  |
| GO:0002544\_chronic\_inflammatory\_response | 4 | 0 |  |  |  |  |  |  |  |  |
| GO:0002636\_positive\_regulation\_of\_germinal\_center\_formation | 4 | 0 |  |  |  |  |  |  |  |  |
| GO:0002667\_regulation\_of\_T\_cell\_anergy | 4 | 0 |  |  |  |  |  |  |  |  |
| GO:0002669\_positive\_regulation\_of\_T\_cell\_anergy | 4 | 0 |  |  |  |  |  |  |  |  |
| GO:0002687\_positive\_regulation\_of\_leukocyte\_migration | 4 | 0 |  |  |  |  |  |  |  |  |
| GO:0002702\_positive\_regulation\_of\_production\_of\_molecular\_mediator\_of\_immune\_response | 4 | 0 |  |  |  |  |  |  |  |  |
| GO:0002718\_regulation\_of\_cytokine\_production\_during\_immune\_response | 4 | 0 |  |  |  |  |  |  |  |  |
| GO:0002829\_negative\_regulation\_of\_T-helper\_2\_type\_immune\_response | 4 | 0 |  |  |  |  |  |  |  |  |
| GO:0002833\_positive\_regulation\_of\_response\_to\_biotic\_stimulus | 4 | 0 |  |  |  |  |  |  |  |  |
| GO:0002834\_regulation\_of\_response\_to\_tumor\_cell | 4 | 0 |  |  |  |  |  |  |  |  |
| GO:0002836\_positive\_regulation\_of\_response\_to\_tumor\_cell | 4 | 0 |  |  |  |  |  |  |  |  |
| GO:0002837\_regulation\_of\_immune\_response\_to\_tumor\_cell | 4 | 0 |  |  |  |  |  |  |  |  |
| GO:0002839\_positive\_regulation\_of\_immune\_response\_to\_tumor\_cell | 4 | 0 |  |  |  |  |  |  |  |  |
| GO:0002870\_T\_cell\_anergy | 4 | 0 |  |  |  |  |  |  |  |  |
| GO:0002888\_positive\_regulation\_of\_myeloid\_leukocyte\_mediated\_immunity | 4 | 0 |  |  |  |  |  |  |  |  |
| GO:0002892\_regulation\_of\_type\_II\_hypersensitivity | 4 | 0 |  |  |  |  |  |  |  |  |
| GO:0002894\_positive\_regulation\_of\_type\_II\_hypersensitivity | 4 | 0 |  |  |  |  |  |  |  |  |
| GO:0002911\_regulation\_of\_lymphocyte\_anergy | 4 | 0 |  |  |  |  |  |  |  |  |
| GO:0002913\_positive\_regulation\_of\_lymphocyte\_anergy | 4 | 0 |  |  |  |  |  |  |  |  |
| GO:0002923\_regulation\_of\_humoral\_immune\_response\_mediated\_by\_circulating\_immunoglobulin | 4 | 0 |  |  |  |  |  |  |  |  |
| GO:0003025\_regulation\_of\_systemic\_arterial\_blood\_pressure\_by\_baroreceptor\_feedback | 4 | 0 |  |  |  |  |  |  |  |  |
| GO:0003091\_renal\_water\_homeostasis | 4 | 0 |  |  |  |  |  |  |  |  |
| GO:0005978\_glycogen\_biosynthetic\_process | 4 | 0 |  |  |  |  |  |  |  |  |
| GO:0006012\_galactose\_metabolic\_process | 4 | 0 |  |  |  |  |  |  |  |  |
| GO:0006085\_acetyl-CoA\_biosynthetic\_process | 4 | 0 |  |  |  |  |  |  |  |  |
| GO:0006111\_regulation\_of\_gluconeogenesis | 4 | 0 |  |  |  |  |  |  |  |  |
| GO:0006144\_purine\_base\_metabolic\_process | 4 | 0 |  |  |  |  |  |  |  |  |
| GO:0006290\_pyrimidine\_dimer\_repair | 4 | 0 |  |  |  |  |  |  |  |  |
| GO:0006334\_nucleosome\_assembly | 4 | 0 |  |  |  |  |  |  |  |  |
| GO:0006534\_cysteine\_metabolic\_process | 4 | 0 |  |  |  |  |  |  |  |  |
| GO:0006547\_histidine\_metabolic\_process | 4 | 0 |  |  |  |  |  |  |  |  |
| GO:0006548\_histidine\_catabolic\_process | 4 | 0 |  |  |  |  |  |  |  |  |
| GO:0006555\_methionine\_metabolic\_process | 4 | 0 |  |  |  |  |  |  |  |  |
| GO:0006599\_phosphagen\_metabolic\_process | 4 | 0 |  |  |  |  |  |  |  |  |
| GO:0006623\_protein\_targeting\_to\_vacuole | 4 | 0 |  |  |  |  |  |  |  |  |
| GO:0006626\_protein\_targeting\_to\_mitochondrion | 4 | 0 |  |  |  |  |  |  |  |  |
| GO:0006684\_sphingomyelin\_metabolic\_process | 4 | 0 |  |  |  |  |  |  |  |  |
| GO:0006688\_glycosphingolipid\_biosynthetic\_process | 4 | 0 |  |  |  |  |  |  |  |  |
| GO:0006739\_NADP\_metabolic\_process | 4 | 0 |  |  |  |  |  |  |  |  |
| GO:0006835\_dicarboxylic\_acid\_transport | 4 | 0 |  |  |  |  |  |  |  |  |
| GO:0006837\_serotonin\_transport | 4 | 0 |  |  |  |  |  |  |  |  |
| GO:0006888\_ER\_to\_Golgi\_vesicle-mediated\_transport | 4 | 0 |  |  |  |  |  |  |  |  |
| GO:0006927\_transformed\_cell\_apoptosis | 4 | 0 |  |  |  |  |  |  |  |  |
| GO:0006972\_hyperosmotic\_response | 4 | 0 |  |  |  |  |  |  |  |  |
| GO:0007028\_cytoplasm\_organization | 4 | 0 |  |  |  |  |  |  |  |  |
| GO:0007031\_peroxisome\_organization | 4 | 0 |  |  |  |  |  |  |  |  |
| GO:0007066\_female\_meiosis\_sister\_chromatid\_cohesion | 4 | 0 |  |  |  |  |  |  |  |  |
| GO:0007144\_female\_meiosis\_I | 4 | 0 |  |  |  |  |  |  |  |  |
| GO:0007184\_SMAD\_protein\_nuclear\_translocation | 4 | 0 |  |  |  |  |  |  |  |  |
| GO:0007216\_metabotropic\_glutamate\_receptor\_signaling\_pathway | 4 | 0 |  |  |  |  |  |  |  |  |
| GO:0007342\_fusion\_of\_sperm\_to\_egg\_plasma\_membrane | 4 | 0 |  |  |  |  |  |  |  |  |
| GO:0008053\_mitochondrial\_fusion | 4 | 0 |  |  |  |  |  |  |  |  |
| GO:0008207\_C21-steroid\_hormone\_metabolic\_process | 4 | 0 |  |  |  |  |  |  |  |  |
| GO:0008215\_spermine\_metabolic\_process | 4 | 0 |  |  |  |  |  |  |  |  |
| GO:0009065\_glutamine\_family\_amino\_acid\_catabolic\_process | 4 | 0 |  |  |  |  |  |  |  |  |
| GO:0009075\_histidine\_family\_amino\_acid\_metabolic\_process | 4 | 0 |  |  |  |  |  |  |  |  |
| GO:0009077\_histidine\_family\_amino\_acid\_catabolic\_process | 4 | 0 |  |  |  |  |  |  |  |  |
| GO:0009134\_nucleoside\_diphosphate\_catabolic\_process | 4 | 0 |  |  |  |  |  |  |  |  |
| GO:0009163\_nucleoside\_biosynthetic\_process | 4 | 0 |  |  |  |  |  |  |  |  |
| GO:0009225\_nucleotide-sugar\_metabolic\_process | 4 | 0 |  |  |  |  |  |  |  |  |
| GO:0009250\_glucan\_biosynthetic\_process | 4 | 0 |  |  |  |  |  |  |  |  |
| GO:0009404\_toxin\_metabolic\_process | 4 | 0 |  |  |  |  |  |  |  |  |
| GO:0009593\_detection\_of\_chemical\_stimulus | 4 | 0 |  |  |  |  |  |  |  |  |
| GO:0009595\_detection\_of\_biotic\_stimulus | 4 | 0 |  |  |  |  |  |  |  |  |
| GO:0009755\_hormone-mediated\_signaling | 4 | 0 |  |  |  |  |  |  |  |  |
| GO:0009912\_auditory\_receptor\_cell\_fate\_commitment | 4 | 0 |  |  |  |  |  |  |  |  |
| GO:0010224\_response\_to\_UV-B | 4 | 0 |  |  |  |  |  |  |  |  |
| GO:0010453\_regulation\_of\_cell\_fate\_commitment | 4 | 0 |  |  |  |  |  |  |  |  |
| GO:0010506\_regulation\_of\_autophagy | 4 | 0 |  |  |  |  |  |  |  |  |
| GO:0010631\_epithelial\_cell\_migration | 4 | 0 |  |  |  |  |  |  |  |  |
| GO:0010812\_negative\_regulation\_of\_cell-substrate\_adhesion | 4 | 0 |  |  |  |  |  |  |  |  |
| GO:0010829\_negative\_regulation\_of\_glucose\_transport | 4 | 0 |  |  |  |  |  |  |  |  |
| GO:0014002\_astrocyte\_development | 4 | 0 |  |  |  |  |  |  |  |  |
| GO:0014832\_urinary\_bladder\_smooth\_muscle\_contraction | 4 | 0 |  |  |  |  |  |  |  |  |
| GO:0014848\_urinary\_tract\_smooth\_muscle\_contraction | 4 | 0 |  |  |  |  |  |  |  |  |
| GO:0015701\_bicarbonate\_transport | 4 | 0 |  |  |  |  |  |  |  |  |
| GO:0015809\_arginine\_transport | 4 | 0 |  |  |  |  |  |  |  |  |
| GO:0015850\_organic\_alcohol\_transport | 4 | 0 |  |  |  |  |  |  |  |  |
| GO:0015858\_nucleoside\_transport | 4 | 0 |  |  |  |  |  |  |  |  |
| GO:0016068\_type\_I\_hypersensitivity | 4 | 0 |  |  |  |  |  |  |  |  |
| GO:0016198\_axon\_choice\_point\_recognition | 4 | 0 |  |  |  |  |  |  |  |  |
| GO:0016338\_calcium-independent\_cell-cell\_adhesion | 4 | 0 |  |  |  |  |  |  |  |  |
| GO:0018198\_peptidyl-cysteine\_modification | 4 | 0 |  |  |  |  |  |  |  |  |
| GO:0018409\_peptide\_or\_protein\_amino-terminal\_blocking | 4 | 0 |  |  |  |  |  |  |  |  |
| GO:0019377\_glycolipid\_catabolic\_process | 4 | 0 |  |  |  |  |  |  |  |  |
| GO:0019432\_triglyceride\_biosynthetic\_process | 4 | 0 |  |  |  |  |  |  |  |  |
| GO:0019530\_taurine\_metabolic\_process | 4 | 0 |  |  |  |  |  |  |  |  |
| GO:0021523\_somatic\_motor\_neuron\_differentiation | 4 | 0 |  |  |  |  |  |  |  |  |
| GO:0021535\_cell\_migration\_in\_hindbrain | 4 | 0 |  |  |  |  |  |  |  |  |
| GO:0021542\_dentate\_gyrus\_development | 4 | 0 |  |  |  |  |  |  |  |  |
| GO:0021561\_facial\_nerve\_development | 4 | 0 |  |  |  |  |  |  |  |  |
| GO:0021569\_rhombomere\_3\_development | 4 | 0 |  |  |  |  |  |  |  |  |
| GO:0021571\_rhombomere\_5\_development | 4 | 0 |  |  |  |  |  |  |  |  |
| GO:0021604\_cranial\_nerve\_structural\_organization | 4 | 0 |  |  |  |  |  |  |  |  |
| GO:0021610\_facial\_nerve\_morphogenesis | 4 | 0 |  |  |  |  |  |  |  |  |
| GO:0021612\_facial\_nerve\_structural\_organization | 4 | 0 |  |  |  |  |  |  |  |  |
| GO:0021631\_optic\_nerve\_morphogenesis | 4 | 0 |  |  |  |  |  |  |  |  |
| GO:0021681\_cerebellar\_granular\_layer\_development | 4 | 0 |  |  |  |  |  |  |  |  |
| GO:0021683\_cerebellar\_granular\_layer\_morphogenesis | 4 | 0 |  |  |  |  |  |  |  |  |
| GO:0021684\_cerebellar\_granular\_layer\_formation | 4 | 0 |  |  |  |  |  |  |  |  |
| GO:0021707\_cerebellar\_granule\_cell\_differentiation | 4 | 0 |  |  |  |  |  |  |  |  |
| GO:0021778\_oligodendrocyte\_cell\_fate\_specification | 4 | 0 |  |  |  |  |  |  |  |  |
| GO:0021779\_oligodendrocyte\_cell\_fate\_commitment | 4 | 0 |  |  |  |  |  |  |  |  |
| GO:0021780\_glial\_cell\_fate\_specification | 4 | 0 |  |  |  |  |  |  |  |  |
| GO:0021830\_interneuron\_migration\_from\_the\_subpallium\_to\_the\_cortex | 4 | 0 |  |  |  |  |  |  |  |  |
| GO:0021853\_cerebral\_cortex\_GABAergic\_interneuron\_migration | 4 | 0 |  |  |  |  |  |  |  |  |
| GO:0021877\_forebrain\_neuron\_fate\_commitment | 4 | 0 |  |  |  |  |  |  |  |  |
| GO:0021894\_cerebral\_cortex\_GABAergic\_interneuron\_development | 4 | 0 |  |  |  |  |  |  |  |  |
| GO:0021910\_smoothened\_signaling\_pathway\_involved\_in\_ventral\_spinal\_cord\_patterning | 4 | 0 |  |  |  |  |  |  |  |  |
| GO:0021913\_regulation\_of\_transcription\_from\_RNA\_polymerase\_II\_promoter\_involved\_in\_ventral\_spinal\_cord\_interneuron\_specification | 4 | 0 |  |  |  |  |  |  |  |  |
| GO:0021938\_smoothened\_signaling\_pathway\_involved\_in\_regulation\_of\_granule\_cell\_precursor\_cell\_proliferation | 4 | 0 |  |  |  |  |  |  |  |  |
| GO:0021978\_telencephalon\_regionalization | 4 | 0 |  |  |  |  |  |  |  |  |
| GO:0022011\_myelination\_in\_the\_peripheral\_nervous\_system | 4 | 0 |  |  |  |  |  |  |  |  |
| GO:0030146\_diuresis | 4 | 0 |  |  |  |  |  |  |  |  |
| GO:0030300\_regulation\_of\_intestinal\_cholesterol\_absorption | 4 | 0 |  |  |  |  |  |  |  |  |
| GO:0030800\_negative\_regulation\_of\_cyclic\_nucleotide\_metabolic\_process | 4 | 0 |  |  |  |  |  |  |  |  |
| GO:0030803\_negative\_regulation\_of\_cyclic\_nucleotide\_biosynthetic\_process | 4 | 0 |  |  |  |  |  |  |  |  |
| GO:0030809\_negative\_regulation\_of\_nucleotide\_biosynthetic\_process | 4 | 0 |  |  |  |  |  |  |  |  |
| GO:0030815\_negative\_regulation\_of\_cAMP\_metabolic\_process | 4 | 0 |  |  |  |  |  |  |  |  |
| GO:0030816\_positive\_regulation\_of\_cAMP\_metabolic\_process | 4 | 0 |  |  |  |  |  |  |  |  |
| GO:0030818\_negative\_regulation\_of\_cAMP\_biosynthetic\_process | 4 | 0 |  |  |  |  |  |  |  |  |
| GO:0030819\_positive\_regulation\_of\_cAMP\_biosynthetic\_process | 4 | 0 |  |  |  |  |  |  |  |  |
| GO:0030826\_regulation\_of\_cGMP\_biosynthetic\_process | 4 | 0 |  |  |  |  |  |  |  |  |
| GO:0030859\_polarized\_epithelial\_cell\_differentiation | 4 | 0 |  |  |  |  |  |  |  |  |
| GO:0030949\_positive\_regulation\_of\_vascular\_endothelial\_growth\_factor\_receptor\_signaling\_pathway | 4 | 0 |  |  |  |  |  |  |  |  |
| GO:0031113\_regulation\_of\_microtubule\_polymerization | 4 | 0 |  |  |  |  |  |  |  |  |
| GO:0031365\_N-terminal\_protein\_amino\_acid\_modification | 4 | 0 |  |  |  |  |  |  |  |  |
| GO:0031424\_keratinization | 4 | 0 |  |  |  |  |  |  |  |  |
| GO:0031557\_induction\_of\_programmed\_cell\_death\_in\_response\_to\_chemical\_stimulus | 4 | 0 |  |  |  |  |  |  |  |  |
| GO:0031558\_induction\_of\_apoptosis\_in\_response\_to\_chemical\_stimulus | 4 | 0 |  |  |  |  |  |  |  |  |
| GO:0031623\_receptor\_internalization | 4 | 0 |  |  |  |  |  |  |  |  |
| GO:0032088\_negative\_regulation\_of\_NF-kappaB\_transcription\_factor\_activity | 4 | 0 |  |  |  |  |  |  |  |  |
| GO:0032098\_regulation\_of\_appetite | 4 | 0 |  |  |  |  |  |  |  |  |
| GO:0032105\_negative\_regulation\_of\_response\_to\_extracellular\_stimulus | 4 | 0 |  |  |  |  |  |  |  |  |
| GO:0032108\_negative\_regulation\_of\_response\_to\_nutrient\_levels | 4 | 0 |  |  |  |  |  |  |  |  |
| GO:0032225\_regulation\_of\_synaptic\_transmission\_\_dopaminergic | 4 | 0 |  |  |  |  |  |  |  |  |
| GO:0032292\_ensheathment\_of\_axons\_in\_the\_peripheral\_nervous\_system | 4 | 0 |  |  |  |  |  |  |  |  |
| GO:0032321\_positive\_regulation\_of\_Rho\_GTPase\_activity | 4 | 0 |  |  |  |  |  |  |  |  |
| GO:0032371\_regulation\_of\_sterol\_transport | 4 | 0 |  |  |  |  |  |  |  |  |
| GO:0032374\_regulation\_of\_cholesterol\_transport | 4 | 0 |  |  |  |  |  |  |  |  |
| GO:0032401\_establishment\_of\_melanosome\_localization | 4 | 0 |  |  |  |  |  |  |  |  |
| GO:0032608\_interferon-beta\_production | 4 | 0 |  |  |  |  |  |  |  |  |
| GO:0032611\_interleukin-1\_beta\_production | 4 | 0 |  |  |  |  |  |  |  |  |
| GO:0032612\_interleukin-1\_production | 4 | 0 |  |  |  |  |  |  |  |  |
| GO:0032648\_regulation\_of\_interferon-beta\_production | 4 | 0 |  |  |  |  |  |  |  |  |
| GO:0032651\_regulation\_of\_interleukin-1\_beta\_production | 4 | 0 |  |  |  |  |  |  |  |  |
| GO:0032652\_regulation\_of\_interleukin-1\_production | 4 | 0 |  |  |  |  |  |  |  |  |
| GO:0032689\_negative\_regulation\_of\_interferon-gamma\_production | 4 | 0 |  |  |  |  |  |  |  |  |
| GO:0032713\_negative\_regulation\_of\_interleukin-4\_production | 4 | 0 |  |  |  |  |  |  |  |  |
| GO:0032715\_negative\_regulation\_of\_interleukin-6\_production | 4 | 0 |  |  |  |  |  |  |  |  |
| GO:0032733\_positive\_regulation\_of\_interleukin-10\_production | 4 | 0 |  |  |  |  |  |  |  |  |
| GO:0032835\_glomerulus\_development | 4 | 0 |  |  |  |  |  |  |  |  |
| GO:0032872\_regulation\_of\_stress-activated\_MAPK\_cascade | 4 | 0 |  |  |  |  |  |  |  |  |
| GO:0032922\_circadian\_regulation\_of\_gene\_expression | 4 | 0 |  |  |  |  |  |  |  |  |
| GO:0033026\_negative\_regulation\_of\_mast\_cell\_apoptosis | 4 | 0 |  |  |  |  |  |  |  |  |
| GO:0033079\_immature\_T\_cell\_proliferation | 4 | 0 |  |  |  |  |  |  |  |  |
| GO:0033083\_regulation\_of\_immature\_T\_cell\_proliferation | 4 | 0 |  |  |  |  |  |  |  |  |
| GO:0033089\_positive\_regulation\_of\_T\_cell\_differentiation\_in\_the\_thymus | 4 | 0 |  |  |  |  |  |  |  |  |
| GO:0033135\_regulation\_of\_peptidyl-serine\_phosphorylation | 4 | 0 |  |  |  |  |  |  |  |  |
| GO:0033299\_secretion\_of\_lysosomal\_enzymes | 4 | 0 |  |  |  |  |  |  |  |  |
| GO:0033327\_Leydig\_cell\_differentiation | 4 | 0 |  |  |  |  |  |  |  |  |
| GO:0033363\_secretory\_granule\_organization | 4 | 0 |  |  |  |  |  |  |  |  |
| GO:0033599\_regulation\_of\_mammary\_gland\_epithelial\_cell\_proliferation | 4 | 0 |  |  |  |  |  |  |  |  |
| GO:0033865\_nucleoside\_bisphosphate\_metabolic\_process | 4 | 0 |  |  |  |  |  |  |  |  |
| GO:0034204\_lipid\_translocation | 4 | 0 |  |  |  |  |  |  |  |  |
| GO:0034404\_nucleobase\_\_nucleoside\_and\_nucleotide\_biosynthetic\_process | 4 | 0 |  |  |  |  |  |  |  |  |
| GO:0034587\_piRNA\_metabolic\_process | 4 | 0 |  |  |  |  |  |  |  |  |
| GO:0034614\_cellular\_response\_to\_reactive\_oxygen\_species | 4 | 0 |  |  |  |  |  |  |  |  |
| GO:0034654\_nucleobase\_\_nucleoside\_\_nucleotide\_and\_nucleic\_acid\_biosynthetic\_process | 4 | 0 |  |  |  |  |  |  |  |  |
| GO:0035020\_regulation\_of\_Rac\_protein\_signal\_transduction | 4 | 0 |  |  |  |  |  |  |  |  |
| GO:0035082\_axoneme\_assembly | 4 | 0 |  |  |  |  |  |  |  |  |
| GO:0035188\_hatching | 4 | 0 |  |  |  |  |  |  |  |  |
| GO:0035235\_ionotropic\_glutamate\_receptor\_signaling\_pathway | 4 | 0 |  |  |  |  |  |  |  |  |
| GO:0042345\_regulation\_of\_NF-kappaB\_import\_into\_nucleus | 4 | 0 |  |  |  |  |  |  |  |  |
| GO:0042348\_NF-kappaB\_import\_into\_nucleus | 4 | 0 |  |  |  |  |  |  |  |  |
| GO:0042359\_vitamin\_D\_metabolic\_process | 4 | 0 |  |  |  |  |  |  |  |  |
| GO:0042428\_serotonin\_metabolic\_process | 4 | 0 |  |  |  |  |  |  |  |  |
| GO:0042451\_purine\_nucleoside\_biosynthetic\_process | 4 | 0 |  |  |  |  |  |  |  |  |
| GO:0042455\_ribonucleoside\_biosynthetic\_process | 4 | 0 |  |  |  |  |  |  |  |  |
| GO:0042473\_outer\_ear\_morphogenesis | 4 | 0 |  |  |  |  |  |  |  |  |
| GO:0042522\_regulation\_of\_tyrosine\_phosphorylation\_of\_Stat5\_protein | 4 | 0 |  |  |  |  |  |  |  |  |
| GO:0042535\_positive\_regulation\_of\_tumor\_necrosis\_factor\_biosynthetic\_process | 4 | 0 |  |  |  |  |  |  |  |  |
| GO:0042541\_hemoglobin\_biosynthetic\_process | 4 | 0 |  |  |  |  |  |  |  |  |
| GO:0042558\_pteridine\_and\_derivative\_metabolic\_process | 4 | 0 |  |  |  |  |  |  |  |  |
| GO:0042634\_regulation\_of\_hair\_cycle | 4 | 0 |  |  |  |  |  |  |  |  |
| GO:0042744\_hydrogen\_peroxide\_catabolic\_process | 4 | 0 |  |  |  |  |  |  |  |  |
| GO:0042773\_ATP\_synthesis\_coupled\_electron\_transport | 4 | 0 |  |  |  |  |  |  |  |  |
| GO:0042775\_mitochondrial\_ATP\_synthesis\_coupled\_electron\_transport | 4 | 0 |  |  |  |  |  |  |  |  |
| GO:0042832\_defense\_response\_to\_protozoan | 4 | 0 |  |  |  |  |  |  |  |  |
| GO:0042982\_amyloid\_precursor\_protein\_metabolic\_process | 4 | 0 |  |  |  |  |  |  |  |  |
| GO:0042992\_negative\_regulation\_of\_transcription\_factor\_import\_into\_nucleus | 4 | 0 |  |  |  |  |  |  |  |  |
| GO:0043043\_peptide\_biosynthetic\_process | 4 | 0 |  |  |  |  |  |  |  |  |
| GO:0043129\_surfactant\_homeostasis | 4 | 0 |  |  |  |  |  |  |  |  |
| GO:0043374\_CD8-positive\_\_alpha-beta\_T\_cell\_differentiation | 4 | 0 |  |  |  |  |  |  |  |  |
| GO:0043470\_regulation\_of\_carbohydrate\_catabolic\_process | 4 | 0 |  |  |  |  |  |  |  |  |
| GO:0043471\_regulation\_of\_cellular\_carbohydrate\_catabolic\_process | 4 | 0 |  |  |  |  |  |  |  |  |
| GO:0043484\_regulation\_of\_RNA\_splicing | 4 | 0 |  |  |  |  |  |  |  |  |
| GO:0043500\_muscle\_adaptation | 4 | 0 |  |  |  |  |  |  |  |  |
| GO:0043534\_blood\_vessel\_endothelial\_cell\_migration | 4 | 0 |  |  |  |  |  |  |  |  |
| GO:0043691\_reverse\_cholesterol\_transport | 4 | 0 |  |  |  |  |  |  |  |  |
| GO:0044243\_multicellular\_organismal\_catabolic\_process | 4 | 0 |  |  |  |  |  |  |  |  |
| GO:0044403\_symbiosis\_\_encompassing\_mutualism\_through\_parasitism | 4 | 0 |  |  |  |  |  |  |  |  |
| GO:0044419\_interspecies\_interaction\_between\_organisms | 4 | 0 |  |  |  |  |  |  |  |  |
| GO:0045066\_regulatory\_T\_cell\_differentiation | 4 | 0 |  |  |  |  |  |  |  |  |
| GO:0045078\_positive\_regulation\_of\_interferon-gamma\_biosynthetic\_process | 4 | 0 |  |  |  |  |  |  |  |  |
| GO:0045332\_phospholipid\_translocation | 4 | 0 |  |  |  |  |  |  |  |  |
| GO:0045346\_regulation\_of\_MHC\_class\_II\_biosynthetic\_process | 4 | 0 |  |  |  |  |  |  |  |  |
| GO:0045350\_interferon-beta\_biosynthetic\_process | 4 | 0 |  |  |  |  |  |  |  |  |
| GO:0045357\_regulation\_of\_interferon-beta\_biosynthetic\_process | 4 | 0 |  |  |  |  |  |  |  |  |
| GO:0045359\_positive\_regulation\_of\_interferon-beta\_biosynthetic\_process | 4 | 0 |  |  |  |  |  |  |  |  |
| GO:0045600\_positive\_regulation\_of\_fat\_cell\_differentiation | 4 | 0 |  |  |  |  |  |  |  |  |
| GO:0045616\_regulation\_of\_keratinocyte\_differentiation | 4 | 0 |  |  |  |  |  |  |  |  |
| GO:0045624\_positive\_regulation\_of\_T-helper\_cell\_differentiation | 4 | 0 |  |  |  |  |  |  |  |  |
| GO:0045628\_regulation\_of\_T-helper\_2\_cell\_differentiation | 4 | 0 |  |  |  |  |  |  |  |  |
| GO:0045634\_regulation\_of\_melanocyte\_differentiation | 4 | 0 |  |  |  |  |  |  |  |  |
| GO:0045647\_negative\_regulation\_of\_erythrocyte\_differentiation | 4 | 0 |  |  |  |  |  |  |  |  |
| GO:0045672\_positive\_regulation\_of\_osteoclast\_differentiation | 4 | 0 |  |  |  |  |  |  |  |  |
| GO:0045684\_positive\_regulation\_of\_epidermis\_development | 4 | 0 |  |  |  |  |  |  |  |  |
| GO:0045736\_negative\_regulation\_of\_cyclin-dependent\_protein\_kinase\_activity | 4 | 0 |  |  |  |  |  |  |  |  |
| GO:0045742\_positive\_regulation\_of\_epidermal\_growth\_factor\_receptor\_signaling\_pathway | 4 | 0 |  |  |  |  |  |  |  |  |
| GO:0045747\_positive\_regulation\_of\_Notch\_signaling\_pathway | 4 | 0 |  |  |  |  |  |  |  |  |
| GO:0045767\_regulation\_of\_anti-apoptosis | 4 | 0 |  |  |  |  |  |  |  |  |
| GO:0045779\_negative\_regulation\_of\_bone\_resorption | 4 | 0 |  |  |  |  |  |  |  |  |
| GO:0045923\_positive\_regulation\_of\_fatty\_acid\_metabolic\_process | 4 | 0 |  |  |  |  |  |  |  |  |
| GO:0045930\_negative\_regulation\_of\_mitotic\_cell\_cycle | 4 | 0 |  |  |  |  |  |  |  |  |
| GO:0045980\_negative\_regulation\_of\_nucleotide\_metabolic\_process | 4 | 0 |  |  |  |  |  |  |  |  |
| GO:0046129\_purine\_ribonucleoside\_biosynthetic\_process | 4 | 0 |  |  |  |  |  |  |  |  |
| GO:0046173\_polyol\_biosynthetic\_process | 4 | 0 |  |  |  |  |  |  |  |  |
| GO:0046541\_saliva\_secretion | 4 | 0 |  |  |  |  |  |  |  |  |
| GO:0046548\_retinal\_rod\_cell\_development | 4 | 0 |  |  |  |  |  |  |  |  |
| GO:0046579\_positive\_regulation\_of\_Ras\_protein\_signal\_transduction | 4 | 0 |  |  |  |  |  |  |  |  |
| GO:0046639\_negative\_regulation\_of\_alpha-beta\_T\_cell\_differentiation | 4 | 0 |  |  |  |  |  |  |  |  |
| GO:0046642\_negative\_regulation\_of\_alpha-beta\_T\_cell\_proliferation | 4 | 0 |  |  |  |  |  |  |  |  |
| GO:0046668\_regulation\_of\_retinal\_cell\_programmed\_cell\_death | 4 | 0 |  |  |  |  |  |  |  |  |
| GO:0046686\_response\_to\_cadmium\_ion | 4 | 0 |  |  |  |  |  |  |  |  |
| GO:0046835\_carbohydrate\_phosphorylation | 4 | 0 |  |  |  |  |  |  |  |  |
| GO:0046902\_regulation\_of\_mitochondrial\_membrane\_permeability | 4 | 0 |  |  |  |  |  |  |  |  |
| GO:0047496\_vesicle\_transport\_along\_microtubule | 4 | 0 |  |  |  |  |  |  |  |  |
| GO:0048011\_nerve\_growth\_factor\_receptor\_signaling\_pathway | 4 | 0 |  |  |  |  |  |  |  |  |
| GO:0048024\_regulation\_of\_nuclear\_mRNA\_splicing\_\_via\_spliceosome | 4 | 0 |  |  |  |  |  |  |  |  |
| GO:0048484\_enteric\_nervous\_system\_development | 4 | 0 |  |  |  |  |  |  |  |  |
| GO:0048512\_circadian\_behavior | 4 | 0 |  |  |  |  |  |  |  |  |
| GO:0048558\_embryonic\_gut\_morphogenesis | 4 | 0 |  |  |  |  |  |  |  |  |
| GO:0048841\_regulation\_of\_axon\_extension\_involved\_in\_axon\_guidance | 4 | 0 |  |  |  |  |  |  |  |  |
| GO:0048843\_negative\_regulation\_of\_axon\_extension\_involved\_in\_axon\_guidance | 4 | 0 |  |  |  |  |  |  |  |  |
| GO:0048846\_axon\_extension\_involved\_in\_axon\_guidance | 4 | 0 |  |  |  |  |  |  |  |  |
| GO:0048875\_chemical\_homeostasis\_within\_a\_tissue | 4 | 0 |  |  |  |  |  |  |  |  |
| GO:0048935\_peripheral\_nervous\_system\_neuron\_development | 4 | 0 |  |  |  |  |  |  |  |  |
| GO:0050702\_interleukin-1\_beta\_secretion | 4 | 0 |  |  |  |  |  |  |  |  |
| GO:0050704\_regulation\_of\_interleukin-1\_secretion | 4 | 0 |  |  |  |  |  |  |  |  |
| GO:0050706\_regulation\_of\_interleukin-1\_beta\_secretion | 4 | 0 |  |  |  |  |  |  |  |  |
| GO:0050716\_positive\_regulation\_of\_interleukin-1\_secretion | 4 | 0 |  |  |  |  |  |  |  |  |
| GO:0050718\_positive\_regulation\_of\_interleukin-1\_beta\_secretion | 4 | 0 |  |  |  |  |  |  |  |  |
| GO:0050820\_positive\_regulation\_of\_coagulation | 4 | 0 |  |  |  |  |  |  |  |  |
| GO:0050891\_multicellular\_organismal\_water\_homeostasis | 4 | 0 |  |  |  |  |  |  |  |  |
| GO:0050932\_regulation\_of\_pigment\_cell\_differentiation | 4 | 0 |  |  |  |  |  |  |  |  |
| GO:0050961\_detection\_of\_temperature\_stimulus\_involved\_in\_sensory\_perception | 4 | 0 |  |  |  |  |  |  |  |  |
| GO:0050965\_detection\_of\_temperature\_stimulus\_involved\_in\_sensory\_perception\_of\_pain | 4 | 0 |  |  |  |  |  |  |  |  |
| GO:0050994\_regulation\_of\_lipid\_catabolic\_process | 4 | 0 |  |  |  |  |  |  |  |  |
| GO:0051055\_negative\_regulation\_of\_lipid\_biosynthetic\_process | 4 | 0 |  |  |  |  |  |  |  |  |
| GO:0051124\_synaptic\_growth\_at\_neuromuscular\_junction | 4 | 0 |  |  |  |  |  |  |  |  |
| GO:0051148\_negative\_regulation\_of\_muscle\_cell\_differentiation | 4 | 0 |  |  |  |  |  |  |  |  |
| GO:0051205\_protein\_insertion\_into\_membrane | 4 | 0 |  |  |  |  |  |  |  |  |
| GO:0051225\_spindle\_assembly | 4 | 0 |  |  |  |  |  |  |  |  |
| GO:0051341\_regulation\_of\_oxidoreductase\_activity | 4 | 0 |  |  |  |  |  |  |  |  |
| GO:0051452\_intracellular\_pH\_reduction | 4 | 0 |  |  |  |  |  |  |  |  |
| GO:0051567\_histone\_H3-K9\_methylation | 4 | 0 |  |  |  |  |  |  |  |  |
| GO:0051642\_centrosome\_localization | 4 | 0 |  |  |  |  |  |  |  |  |
| GO:0051797\_regulation\_of\_hair\_follicle\_development | 4 | 0 |  |  |  |  |  |  |  |  |
| GO:0051897\_positive\_regulation\_of\_protein\_kinase\_B\_signaling\_cascade | 4 | 0 |  |  |  |  |  |  |  |  |
| GO:0051904\_pigment\_granule\_transport | 4 | 0 |  |  |  |  |  |  |  |  |
| GO:0060008\_Sertoli\_cell\_differentiation | 4 | 0 |  |  |  |  |  |  |  |  |
| GO:0060011\_Sertoli\_cell\_proliferation | 4 | 0 |  |  |  |  |  |  |  |  |
| GO:0060057\_apoptosis\_involved\_in\_mammary\_gland\_involution | 4 | 0 |  |  |  |  |  |  |  |  |
| GO:0060058\_positive\_regulation\_of\_apoptosis\_involved\_in\_mammary\_gland\_involution | 4 | 0 |  |  |  |  |  |  |  |  |
| GO:0060087\_relaxation\_of\_vascular\_smooth\_muscle | 4 | 0 |  |  |  |  |  |  |  |  |
| GO:0060120\_inner\_ear\_receptor\_cell\_fate\_commitment | 4 | 0 |  |  |  |  |  |  |  |  |
| GO:0060158\_activation\_of\_phospholipase\_C\_activity\_by\_dopamine\_receptor\_signaling\_pathway | 4 | 0 |  |  |  |  |  |  |  |  |
| GO:0060164\_regulation\_of\_timing\_of\_neuron\_differentiation | 4 | 0 |  |  |  |  |  |  |  |  |
| GO:0060235\_lens\_induction\_in\_camera-type\_eye | 4 | 0 |  |  |  |  |  |  |  |  |
| GO:0060291\_long-term\_synaptic\_potentiation | 4 | 0 |  |  |  |  |  |  |  |  |
| GO:0060459\_left\_lung\_development | 4 | 0 |  |  |  |  |  |  |  |  |
| GO:0060528\_secretory\_columnal\_luminar\_epithelial\_cell\_differentiation\_involved\_in\_prostate\_glandular\_acinus\_development | 4 | 0 |  |  |  |  |  |  |  |  |
| GO:0060561\_apoptosis\_involved\_in\_morphogenesis | 4 | 0 |  |  |  |  |  |  |  |  |
| GO:0060592\_mammary\_gland\_formation | 4 | 0 |  |  |  |  |  |  |  |  |
| GO:0060644\_mammary\_gland\_epithelial\_cell\_differentiation | 4 | 0 |  |  |  |  |  |  |  |  |
| GO:0060666\_dichotomous\_subdivision\_of\_terminal\_units\_involved\_in\_salivary\_gland\_branching | 4 | 0 |  |  |  |  |  |  |  |  |
| GO:0060737\_prostate\_gland\_morphogenetic\_growth | 4 | 0 |  |  |  |  |  |  |  |  |
| GO:0060743\_epithelial\_cell\_maturation\_involved\_in\_prostate\_gland\_development | 4 | 0 |  |  |  |  |  |  |  |  |
| GO:0070059\_apoptosis\_in\_response\_to\_endoplasmic\_reticulum\_stress | 4 | 0 |  |  |  |  |  |  |  |  |
| GO:0070254\_mucus\_secretion | 4 | 0 |  |  |  |  |  |  |  |  |
| GO:0070255\_regulation\_of\_mucus\_secretion | 4 | 0 |  |  |  |  |  |  |  |  |
| GO:0070301\_cellular\_response\_to\_hydrogen\_peroxide | 4 | 0 |  |  |  |  |  |  |  |  |
| GO:0070585\_protein\_localization\_in\_mitochondrion | 4 | 0 |  |  |  |  |  |  |  |  |
| GO:0000082\_G1\_S\_transition\_of\_mitotic\_cell\_cycle | 23 | 0 | 0.000000 | -0.000000 | 1003 | 714.698906 | 818.15 | 921.601094 | 0.815703 |  |
| GO:0002228\_natural\_killer\_cell\_mediated\_immunity | 23 | 0 | 0.000000 | -0.000000 | 1003 | 714.698906 | 818.15 | 921.601094 | 0.815703 |  |
| GO:0002821\_positive\_regulation\_of\_adaptive\_immune\_response | 23 | 0 | 0.000000 | -0.000000 | 1003 | 714.698906 | 818.15 | 921.601094 | 0.815703 |  |
| GO:0002824\_positive\_regulation\_of\_adaptive\_immune\_response\_based\_on\_somatic\_recombination\_of\_immune\_receptors\_built\_from\_immunoglobulin\_superfamily\_domains | 23 | 0 | 0.000000 | -0.000000 | 1003 | 714.698906 | 818.15 | 921.601094 | 0.815703 |  |
| GO:0003073\_regulation\_of\_systemic\_arterial\_blood\_pressure | 23 | 0 | 0.000000 | -0.000000 | 1003 | 714.698906 | 818.15 | 921.601094 | 0.815703 |  |
| GO:0006397\_mRNA\_processing | 23 | 0 | 0.000000 | -0.000000 | 1003 | 714.698906 | 818.15 | 921.601094 | 0.815703 |  |
| GO:0007018\_microtubule-based\_movement | 23 | 0 | 0.000000 | -0.000000 | 1003 | 714.698906 | 818.15 | 921.601094 | 0.815703 |  |
| GO:0007163\_establishment\_or\_maintenance\_of\_cell\_polarity | 23 | 0 | 0.000000 | -0.000000 | 1003 | 714.698906 | 818.15 | 921.601094 | 0.815703 |  |
| GO:0008542\_visual\_learning | 23 | 0 | 0.000000 | -0.000000 | 1003 | 714.698906 | 818.15 | 921.601094 | 0.815703 |  |
| GO:0015698\_inorganic\_anion\_transport | 23 | 0 | 0.000000 | -0.000000 | 1003 | 714.698906 | 818.15 | 921.601094 | 0.815703 |  |
| GO:0022613\_ribonucleoprotein\_complex\_biogenesis | 23 | 0 | 0.000000 | -0.000000 | 1003 | 714.698906 | 818.15 | 921.601094 | 0.815703 |  |
| GO:0030512\_negative\_regulation\_of\_transforming\_growth\_factor\_beta\_receptor\_signaling\_pathway | 23 | 0 | 0.000000 | -0.000000 | 1003 | 714.698906 | 818.15 | 921.601094 | 0.815703 |  |
| GO:0032635\_interleukin-6\_production | 23 | 0 | 0.000000 | -0.000000 | 1003 | 714.698906 | 818.15 | 921.601094 | 0.815703 |  |
| GO:0032675\_regulation\_of\_interleukin-6\_production | 23 | 0 | 0.000000 | -0.000000 | 1003 | 714.698906 | 818.15 | 921.601094 | 0.815703 |  |
| GO:0042267\_natural\_killer\_cell\_mediated\_cytotoxicity | 23 | 0 | 0.000000 | -0.000000 | 1003 | 714.698906 | 818.15 | 921.601094 | 0.815703 |  |
| GO:0043388\_positive\_regulation\_of\_DNA\_binding | 23 | 0 | 0.000000 | -0.000000 | 1003 | 714.698906 | 818.15 | 921.601094 | 0.815703 |  |
| GO:0051705\_behavioral\_interaction\_between\_organisms | 23 | 0 | 0.000000 | -0.000000 | 1003 | 714.698906 | 818.15 | 921.601094 | 0.815703 |  |
| GO:0060349\_bone\_morphogenesis | 23 | 0 | 0.000000 | -0.000000 | 1003 | 714.698906 | 818.15 | 921.601094 | 0.815703 |  |
| GO:0060445\_branching\_involved\_in\_salivary\_gland\_morphogenesis | 23 | 0 | 0.000000 | -0.000000 | 1003 | 714.698906 | 818.15 | 921.601094 | 0.815703 |  |
| GO:0002683\_negative\_regulation\_of\_immune\_system\_process | 56 | 0 | 0.000000 | -0.000000 | 1009 | 720.759890 | 823.33 | 925.900110 | 0.815986 |  |
| GO:0002703\_regulation\_of\_leukocyte\_mediated\_immunity | 56 | 0 | 0.000000 | -0.000000 | 1009 | 720.759890 | 823.33 | 925.900110 | 0.815986 |  |
| GO:0042089\_cytokine\_biosynthetic\_process | 56 | 0 | 0.000000 | -0.000000 | 1009 | 720.759890 | 823.33 | 925.900110 | 0.815986 |  |
| GO:0042107\_cytokine\_metabolic\_process | 56 | 0 | 0.000000 | -0.000000 | 1009 | 720.759890 | 823.33 | 925.900110 | 0.815986 |  |
| GO:0046486\_glycerolipid\_metabolic\_process | 56 | 0 | 0.000000 | -0.000000 | 1009 | 720.759890 | 823.33 | 925.900110 | 0.815986 |  |
| GO:0051321\_meiotic\_cell\_cycle | 56 | 0 | 0.000000 | -0.000000 | 1009 | 720.759890 | 823.33 | 925.900110 | 0.815986 |  |
| GO:0002757\_immune\_response-activating\_signal\_transduction | 47 | 0 | 0.000000 | -0.000000 | 1018 | 730.270339 | 831.57 | 932.869661 | 0.816866 |  |
| GO:0006140\_regulation\_of\_nucleotide\_metabolic\_process | 47 | 0 | 0.000000 | -0.000000 | 1018 | 730.270339 | 831.57 | 932.869661 | 0.816866 |  |
| GO:0006396\_RNA\_processing | 47 | 0 | 0.000000 | -0.000000 | 1018 | 730.270339 | 831.57 | 932.869661 | 0.816866 |  |
| GO:0016570\_histone\_modification | 47 | 0 | 0.000000 | -0.000000 | 1018 | 730.270339 | 831.57 | 932.869661 | 0.816866 |  |
| GO:0030183\_B\_cell\_differentiation | 47 | 0 | 0.000000 | -0.000000 | 1018 | 730.270339 | 831.57 | 932.869661 | 0.816866 |  |
| GO:0030799\_regulation\_of\_cyclic\_nucleotide\_metabolic\_process | 47 | 0 | 0.000000 | -0.000000 | 1018 | 730.270339 | 831.57 | 932.869661 | 0.816866 |  |
| GO:0045087\_innate\_immune\_response | 47 | 0 | 0.000000 | -0.000000 | 1018 | 730.270339 | 831.57 | 932.869661 | 0.816866 |  |
| GO:0045619\_regulation\_of\_lymphocyte\_differentiation | 47 | 0 | 0.000000 | -0.000000 | 1018 | 730.270339 | 831.57 | 932.869661 | 0.816866 |  |
| GO:0060627\_regulation\_of\_vesicle-mediated\_transport | 47 | 0 | 0.000000 | -0.000000 | 1018 | 730.270339 | 831.57 | 932.869661 | 0.816866 |  |
| GO:0000060\_protein\_import\_into\_nucleus\_\_translocation | 14 | 0 | 0.000000 | -0.000000 | 1065 | 787.477901 | 886.42 | 985.362099 | 0.832319 |  |
| GO:0000077\_DNA\_damage\_checkpoint | 14 | 0 | 0.000000 | -0.000000 | 1065 | 787.477901 | 886.42 | 985.362099 | 0.832319 |  |
| GO:0002027\_regulation\_of\_heart\_rate | 14 | 0 | 0.000000 | -0.000000 | 1065 | 787.477901 | 886.42 | 985.362099 | 0.832319 |  |
| GO:0002262\_myeloid\_cell\_homeostasis | 14 | 0 | 0.000000 | -0.000000 | 1065 | 787.477901 | 886.42 | 985.362099 | 0.832319 |  |
| GO:0002698\_negative\_regulation\_of\_immune\_effector\_process | 14 | 0 | 0.000000 | -0.000000 | 1065 | 787.477901 | 886.42 | 985.362099 | 0.832319 |  |
| GO:0006304\_DNA\_modification | 14 | 0 | 0.000000 | -0.000000 | 1065 | 787.477901 | 886.42 | 985.362099 | 0.832319 |  |
| GO:0006305\_DNA\_alkylation | 14 | 0 | 0.000000 | -0.000000 | 1065 | 787.477901 | 886.42 | 985.362099 | 0.832319 |  |
| GO:0006306\_DNA\_methylation | 14 | 0 | 0.000000 | -0.000000 | 1065 | 787.477901 | 886.42 | 985.362099 | 0.832319 |  |
| GO:0006914\_autophagy | 14 | 0 | 0.000000 | -0.000000 | 1065 | 787.477901 | 886.42 | 985.362099 | 0.832319 |  |
| GO:0006970\_response\_to\_osmotic\_stress | 14 | 0 | 0.000000 | -0.000000 | 1065 | 787.477901 | 886.42 | 985.362099 | 0.832319 |  |
| GO:0007157\_heterophilic\_cell\_adhesion | 14 | 0 | 0.000000 | -0.000000 | 1065 | 787.477901 | 886.42 | 985.362099 | 0.832319 |  |
| GO:0007530\_sex\_determination | 14 | 0 | 0.000000 | -0.000000 | 1065 | 787.477901 | 886.42 | 985.362099 | 0.832319 |  |
| GO:0007589\_body\_fluid\_secretion | 14 | 0 | 0.000000 | -0.000000 | 1065 | 787.477901 | 886.42 | 985.362099 | 0.832319 |  |
| GO:0008306\_associative\_learning | 14 | 0 | 0.000000 | -0.000000 | 1065 | 787.477901 | 886.42 | 985.362099 | 0.832319 |  |
| GO:0008630\_DNA\_damage\_response\_\_signal\_transduction\_resulting\_in\_induction\_of\_apoptosis | 14 | 0 | 0.000000 | -0.000000 | 1065 | 787.477901 | 886.42 | 985.362099 | 0.832319 |  |
| GO:0009108\_coenzyme\_biosynthetic\_process | 14 | 0 | 0.000000 | -0.000000 | 1065 | 787.477901 | 886.42 | 985.362099 | 0.832319 |  |
| GO:0009267\_cellular\_response\_to\_starvation | 14 | 0 | 0.000000 | -0.000000 | 1065 | 787.477901 | 886.42 | 985.362099 | 0.832319 |  |
| GO:0009895\_negative\_regulation\_of\_catabolic\_process | 14 | 0 | 0.000000 | -0.000000 | 1065 | 787.477901 | 886.42 | 985.362099 | 0.832319 |  |
| GO:0016573\_histone\_acetylation | 14 | 0 | 0.000000 | -0.000000 | 1065 | 787.477901 | 886.42 | 985.362099 | 0.832319 |  |
| GO:0018130\_heterocycle\_biosynthetic\_process | 14 | 0 | 0.000000 | -0.000000 | 1065 | 787.477901 | 886.42 | 985.362099 | 0.832319 |  |
| GO:0019217\_regulation\_of\_fatty\_acid\_metabolic\_process | 14 | 0 | 0.000000 | -0.000000 | 1065 | 787.477901 | 886.42 | 985.362099 | 0.832319 |  |
| GO:0021782\_glial\_cell\_development | 14 | 0 | 0.000000 | -0.000000 | 1065 | 787.477901 | 886.42 | 985.362099 | 0.832319 |  |
| GO:0021904\_dorsal\_ventral\_neural\_tube\_patterning | 14 | 0 | 0.000000 | -0.000000 | 1065 | 787.477901 | 886.42 | 985.362099 | 0.832319 |  |
| GO:0030148\_sphingolipid\_biosynthetic\_process | 14 | 0 | 0.000000 | -0.000000 | 1065 | 787.477901 | 886.42 | 985.362099 | 0.832319 |  |
| GO:0030162\_regulation\_of\_proteolysis | 14 | 0 | 0.000000 | -0.000000 | 1065 | 787.477901 | 886.42 | 985.362099 | 0.832319 |  |
| GO:0031346\_positive\_regulation\_of\_cell\_projection\_organization | 14 | 0 | 0.000000 | -0.000000 | 1065 | 787.477901 | 886.42 | 985.362099 | 0.832319 |  |
| GO:0031663\_lipopolysaccharide-mediated\_signaling\_pathway | 14 | 0 | 0.000000 | -0.000000 | 1065 | 787.477901 | 886.42 | 985.362099 | 0.832319 |  |
| GO:0032271\_regulation\_of\_protein\_polymerization | 14 | 0 | 0.000000 | -0.000000 | 1065 | 787.477901 | 886.42 | 985.362099 | 0.832319 |  |
| GO:0033044\_regulation\_of\_chromosome\_organization | 14 | 0 | 0.000000 | -0.000000 | 1065 | 787.477901 | 886.42 | 985.362099 | 0.832319 |  |
| GO:0034104\_negative\_regulation\_of\_tissue\_remodeling | 14 | 0 | 0.000000 | -0.000000 | 1065 | 787.477901 | 886.42 | 985.362099 | 0.832319 |  |
| GO:0042573\_retinoic\_acid\_metabolic\_process | 14 | 0 | 0.000000 | -0.000000 | 1065 | 787.477901 | 886.42 | 985.362099 | 0.832319 |  |
| GO:0043123\_positive\_regulation\_of\_I-kappaB\_kinase\_NF-kappaB\_cascade | 14 | 0 | 0.000000 | -0.000000 | 1065 | 787.477901 | 886.42 | 985.362099 | 0.832319 |  |
| GO:0043254\_regulation\_of\_protein\_complex\_assembly | 14 | 0 | 0.000000 | -0.000000 | 1065 | 787.477901 | 886.42 | 985.362099 | 0.832319 |  |
| GO:0043491\_protein\_kinase\_B\_signaling\_cascade | 14 | 0 | 0.000000 | -0.000000 | 1065 | 787.477901 | 886.42 | 985.362099 | 0.832319 |  |
| GO:0044236\_multicellular\_organismal\_metabolic\_process | 14 | 0 | 0.000000 | -0.000000 | 1065 | 787.477901 | 886.42 | 985.362099 | 0.832319 |  |
| GO:0045061\_thymic\_T\_cell\_selection | 14 | 0 | 0.000000 | -0.000000 | 1065 | 787.477901 | 886.42 | 985.362099 | 0.832319 |  |
| GO:0045598\_regulation\_of\_fat\_cell\_differentiation | 14 | 0 | 0.000000 | -0.000000 | 1065 | 787.477901 | 886.42 | 985.362099 | 0.832319 |  |
| GO:0045732\_positive\_regulation\_of\_protein\_catabolic\_process | 14 | 0 | 0.000000 | -0.000000 | 1065 | 787.477901 | 886.42 | 985.362099 | 0.832319 |  |
| GO:0048545\_response\_to\_steroid\_hormone\_stimulus | 14 | 0 | 0.000000 | -0.000000 | 1065 | 787.477901 | 886.42 | 985.362099 | 0.832319 |  |
| GO:0048665\_neuron\_fate\_specification | 14 | 0 | 0.000000 | -0.000000 | 1065 | 787.477901 | 886.42 | 985.362099 | 0.832319 |  |
| GO:0050810\_regulation\_of\_steroid\_biosynthetic\_process | 14 | 0 | 0.000000 | -0.000000 | 1065 | 787.477901 | 886.42 | 985.362099 | 0.832319 |  |
| GO:0051017\_actin\_filament\_bundle\_formation | 14 | 0 | 0.000000 | -0.000000 | 1065 | 787.477901 | 886.42 | 985.362099 | 0.832319 |  |
| GO:0051053\_negative\_regulation\_of\_DNA\_metabolic\_process | 14 | 0 | 0.000000 | -0.000000 | 1065 | 787.477901 | 886.42 | 985.362099 | 0.832319 |  |
| GO:0051054\_positive\_regulation\_of\_DNA\_metabolic\_process | 14 | 0 | 0.000000 | -0.000000 | 1065 | 787.477901 | 886.42 | 985.362099 | 0.832319 |  |
| GO:0051100\_negative\_regulation\_of\_binding | 14 | 0 | 0.000000 | -0.000000 | 1065 | 787.477901 | 886.42 | 985.362099 | 0.832319 |  |
| GO:0051952\_regulation\_of\_amine\_transport | 14 | 0 | 0.000000 | -0.000000 | 1065 | 787.477901 | 886.42 | 985.362099 | 0.832319 |  |
| GO:0060716\_labyrinthine\_layer\_blood\_vessel\_development | 14 | 0 | 0.000000 | -0.000000 | 1065 | 787.477901 | 886.42 | 985.362099 | 0.832319 |  |
| GO:0007346\_regulation\_of\_mitotic\_cell\_cycle | 40 | 0 | 0.000000 | -0.000000 | 1070 | 796.381346 | 894.4 | 992.418654 | 0.835888 |  |
| GO:0016071\_mRNA\_metabolic\_process | 40 | 0 | 0.000000 | -0.000000 | 1070 | 796.381346 | 894.4 | 992.418654 | 0.835888 |  |
| GO:0016485\_protein\_processing | 40 | 0 | 0.000000 | -0.000000 | 1070 | 796.381346 | 894.4 | 992.418654 | 0.835888 |  |
| GO:0019935\_cyclic-nucleotide-mediated\_signaling | 40 | 0 | 0.000000 | -0.000000 | 1070 | 796.381346 | 894.4 | 992.418654 | 0.835888 |  |
| GO:0051129\_negative\_regulation\_of\_cellular\_component\_organization | 40 | 0 | 0.000000 | -0.000000 | 1070 | 796.381346 | 894.4 | 992.418654 | 0.835888 |  |
| GO:0000910\_cytokinesis | 8 | 0 | 0.000000 | -0.000000 | 1183 | 919.897767 | 1014.9 | 1109.902233 | 0.857904 |  |
| GO:0001783\_B\_cell\_apoptosis | 8 | 0 | 0.000000 | -0.000000 | 1183 | 919.897767 | 1014.9 | 1109.902233 | 0.857904 |  |
| GO:0001833\_inner\_cell\_mass\_cell\_proliferation | 8 | 0 | 0.000000 | -0.000000 | 1183 | 919.897767 | 1014.9 | 1109.902233 | 0.857904 |  |
| GO:0001840\_neural\_plate\_development | 8 | 0 | 0.000000 | -0.000000 | 1183 | 919.897767 | 1014.9 | 1109.902233 | 0.857904 |  |
| GO:0001911\_negative\_regulation\_of\_leukocyte\_mediated\_cytotoxicity | 8 | 0 | 0.000000 | -0.000000 | 1183 | 919.897767 | 1014.9 | 1109.902233 | 0.857904 |  |
| GO:0001916\_positive\_regulation\_of\_T\_cell\_mediated\_cytotoxicity | 8 | 0 | 0.000000 | -0.000000 | 1183 | 919.897767 | 1014.9 | 1109.902233 | 0.857904 |  |
| GO:0002065\_columnar\_cuboidal\_epithelial\_cell\_differentiation | 8 | 0 | 0.000000 | -0.000000 | 1183 | 919.897767 | 1014.9 | 1109.902233 | 0.857904 |  |
| GO:0002438\_acute\_inflammatory\_response\_to\_antigenic\_stimulus | 8 | 0 | 0.000000 | -0.000000 | 1183 | 919.897767 | 1014.9 | 1109.902233 | 0.857904 |  |
| GO:0002524\_hypersensitivity | 8 | 0 | 0.000000 | -0.000000 | 1183 | 919.897767 | 1014.9 | 1109.902233 | 0.857904 |  |
| GO:0002566\_somatic\_diversification\_of\_immune\_receptors\_via\_somatic\_mutation | 8 | 0 | 0.000000 | -0.000000 | 1183 | 919.897767 | 1014.9 | 1109.902233 | 0.857904 |  |
| GO:0002864\_regulation\_of\_acute\_inflammatory\_response\_to\_antigenic\_stimulus | 8 | 0 | 0.000000 | -0.000000 | 1183 | 919.897767 | 1014.9 | 1109.902233 | 0.857904 |  |
| GO:0002883\_regulation\_of\_hypersensitivity | 8 | 0 | 0.000000 | -0.000000 | 1183 | 919.897767 | 1014.9 | 1109.902233 | 0.857904 |  |
| GO:0003081\_regulation\_of\_systemic\_arterial\_blood\_pressure\_by\_renin-angiotensin | 8 | 0 | 0.000000 | -0.000000 | 1183 | 919.897767 | 1014.9 | 1109.902233 | 0.857904 |  |
| GO:0006020\_inositol\_metabolic\_process | 8 | 0 | 0.000000 | -0.000000 | 1183 | 919.897767 | 1014.9 | 1109.902233 | 0.857904 |  |
| GO:0006195\_purine\_nucleotide\_catabolic\_process | 8 | 0 | 0.000000 | -0.000000 | 1183 | 919.897767 | 1014.9 | 1109.902233 | 0.857904 |  |
| GO:0006284\_base-excision\_repair | 8 | 0 | 0.000000 | -0.000000 | 1183 | 919.897767 | 1014.9 | 1109.902233 | 0.857904 |  |
| GO:0006349\_genetic\_imprinting | 8 | 0 | 0.000000 | -0.000000 | 1183 | 919.897767 | 1014.9 | 1109.902233 | 0.857904 |  |
| GO:0006360\_transcription\_from\_RNA\_polymerase\_I\_promoter | 8 | 0 | 0.000000 | -0.000000 | 1183 | 919.897767 | 1014.9 | 1109.902233 | 0.857904 |  |
| GO:0006399\_tRNA\_metabolic\_process | 8 | 0 | 0.000000 | -0.000000 | 1183 | 919.897767 | 1014.9 | 1109.902233 | 0.857904 |  |
| GO:0006458\_'de\_novo'\_protein\_folding | 8 | 0 | 0.000000 | -0.000000 | 1183 | 919.897767 | 1014.9 | 1109.902233 | 0.857904 |  |
| GO:0006493\_protein\_amino\_acid\_O-linked\_glycosylation | 8 | 0 | 0.000000 | -0.000000 | 1183 | 919.897767 | 1014.9 | 1109.902233 | 0.857904 |  |
| GO:0006582\_melanin\_metabolic\_process | 8 | 0 | 0.000000 | -0.000000 | 1183 | 919.897767 | 1014.9 | 1109.902233 | 0.857904 |  |
| GO:0006733\_oxidoreduction\_coenzyme\_metabolic\_process | 8 | 0 | 0.000000 | -0.000000 | 1183 | 919.897767 | 1014.9 | 1109.902233 | 0.857904 |  |
| GO:0006829\_zinc\_ion\_transport | 8 | 0 | 0.000000 | -0.000000 | 1183 | 919.897767 | 1014.9 | 1109.902233 | 0.857904 |  |
| GO:0007009\_plasma\_membrane\_organization | 8 | 0 | 0.000000 | -0.000000 | 1183 | 919.897767 | 1014.9 | 1109.902233 | 0.857904 |  |
| GO:0007098\_centrosome\_cycle | 8 | 0 | 0.000000 | -0.000000 | 1183 | 919.897767 | 1014.9 | 1109.902233 | 0.857904 |  |
| GO:0007131\_reciprocal\_meiotic\_recombination | 8 | 0 | 0.000000 | -0.000000 | 1183 | 919.897767 | 1014.9 | 1109.902233 | 0.857904 |  |
| GO:0007141\_male\_meiosis\_I | 8 | 0 | 0.000000 | -0.000000 | 1183 | 919.897767 | 1014.9 | 1109.902233 | 0.857904 |  |
| GO:0007625\_grooming\_behavior | 8 | 0 | 0.000000 | -0.000000 | 1183 | 919.897767 | 1014.9 | 1109.902233 | 0.857904 |  |
| GO:0008105\_asymmetric\_protein\_localization | 8 | 0 | 0.000000 | -0.000000 | 1183 | 919.897767 | 1014.9 | 1109.902233 | 0.857904 |  |
| GO:0008593\_regulation\_of\_Notch\_signaling\_pathway | 8 | 0 | 0.000000 | -0.000000 | 1183 | 919.897767 | 1014.9 | 1109.902233 | 0.857904 |  |
| GO:0009072\_aromatic\_amino\_acid\_family\_metabolic\_process | 8 | 0 | 0.000000 | -0.000000 | 1183 | 919.897767 | 1014.9 | 1109.902233 | 0.857904 |  |
| GO:0009144\_purine\_nucleoside\_triphosphate\_metabolic\_process | 8 | 0 | 0.000000 | -0.000000 | 1183 | 919.897767 | 1014.9 | 1109.902233 | 0.857904 |  |
| GO:0009746\_response\_to\_hexose\_stimulus | 8 | 0 | 0.000000 | -0.000000 | 1183 | 919.897767 | 1014.9 | 1109.902233 | 0.857904 |  |
| GO:0009749\_response\_to\_glucose\_stimulus | 8 | 0 | 0.000000 | -0.000000 | 1183 | 919.897767 | 1014.9 | 1109.902233 | 0.857904 |  |
| GO:0014014\_negative\_regulation\_of\_gliogenesis | 8 | 0 | 0.000000 | -0.000000 | 1183 | 919.897767 | 1014.9 | 1109.902233 | 0.857904 |  |
| GO:0014046\_dopamine\_secretion | 8 | 0 | 0.000000 | -0.000000 | 1183 | 919.897767 | 1014.9 | 1109.902233 | 0.857904 |  |
| GO:0014059\_regulation\_of\_dopamine\_secretion | 8 | 0 | 0.000000 | -0.000000 | 1183 | 919.897767 | 1014.9 | 1109.902233 | 0.857904 |  |
| GO:0014065\_phosphoinositide\_3-kinase\_cascade | 8 | 0 | 0.000000 | -0.000000 | 1183 | 919.897767 | 1014.9 | 1109.902233 | 0.857904 |  |
| GO:0015800\_acidic\_amino\_acid\_transport | 8 | 0 | 0.000000 | -0.000000 | 1183 | 919.897767 | 1014.9 | 1109.902233 | 0.857904 |  |
| GO:0015804\_neutral\_amino\_acid\_transport | 8 | 0 | 0.000000 | -0.000000 | 1183 | 919.897767 | 1014.9 | 1109.902233 | 0.857904 |  |
| GO:0016236\_macroautophagy | 8 | 0 | 0.000000 | -0.000000 | 1183 | 919.897767 | 1014.9 | 1109.902233 | 0.857904 |  |
| GO:0016446\_somatic\_hypermutation\_of\_immunoglobulin\_genes | 8 | 0 | 0.000000 | -0.000000 | 1183 | 919.897767 | 1014.9 | 1109.902233 | 0.857904 |  |
| GO:0018107\_peptidyl-threonine\_phosphorylation | 8 | 0 | 0.000000 | -0.000000 | 1183 | 919.897767 | 1014.9 | 1109.902233 | 0.857904 |  |
| GO:0018210\_peptidyl-threonine\_modification | 8 | 0 | 0.000000 | -0.000000 | 1183 | 919.897767 | 1014.9 | 1109.902233 | 0.857904 |  |
| GO:0018345\_protein\_palmitoylation | 8 | 0 | 0.000000 | -0.000000 | 1183 | 919.897767 | 1014.9 | 1109.902233 | 0.857904 |  |
| GO:0019229\_regulation\_of\_vasoconstriction | 8 | 0 | 0.000000 | -0.000000 | 1183 | 919.897767 | 1014.9 | 1109.902233 | 0.857904 |  |
| GO:0019400\_alditol\_metabolic\_process | 8 | 0 | 0.000000 | -0.000000 | 1183 | 919.897767 | 1014.9 | 1109.902233 | 0.857904 |  |
| GO:0022898\_regulation\_of\_transmembrane\_transporter\_activity | 8 | 0 | 0.000000 | -0.000000 | 1183 | 919.897767 | 1014.9 | 1109.902233 | 0.857904 |  |
| GO:0030035\_microspike\_assembly | 8 | 0 | 0.000000 | -0.000000 | 1183 | 919.897767 | 1014.9 | 1109.902233 | 0.857904 |  |
| GO:0030500\_regulation\_of\_bone\_mineralization | 8 | 0 | 0.000000 | -0.000000 | 1183 | 919.897767 | 1014.9 | 1109.902233 | 0.857904 |  |
| GO:0031111\_negative\_regulation\_of\_microtubule\_polymerization\_or\_depolymerization | 8 | 0 | 0.000000 | -0.000000 | 1183 | 919.897767 | 1014.9 | 1109.902233 | 0.857904 |  |
| GO:0031123\_RNA\_3'-end\_processing | 8 | 0 | 0.000000 | -0.000000 | 1183 | 919.897767 | 1014.9 | 1109.902233 | 0.857904 |  |
| GO:0031294\_lymphocyte\_costimulation | 8 | 0 | 0.000000 | -0.000000 | 1183 | 919.897767 | 1014.9 | 1109.902233 | 0.857904 |  |
| GO:0031295\_T\_cell\_costimulation | 8 | 0 | 0.000000 | -0.000000 | 1183 | 919.897767 | 1014.9 | 1109.902233 | 0.857904 |  |
| GO:0031334\_positive\_regulation\_of\_protein\_complex\_assembly | 8 | 0 | 0.000000 | -0.000000 | 1183 | 919.897767 | 1014.9 | 1109.902233 | 0.857904 |  |
| GO:0031342\_negative\_regulation\_of\_cell\_killing | 8 | 0 | 0.000000 | -0.000000 | 1183 | 919.897767 | 1014.9 | 1109.902233 | 0.857904 |  |
| GO:0031396\_regulation\_of\_protein\_ubiquitination | 8 | 0 | 0.000000 | -0.000000 | 1183 | 919.897767 | 1014.9 | 1109.902233 | 0.857904 |  |
| GO:0032094\_response\_to\_food | 8 | 0 | 0.000000 | -0.000000 | 1183 | 919.897767 | 1014.9 | 1109.902233 | 0.857904 |  |
| GO:0032273\_positive\_regulation\_of\_protein\_polymerization | 8 | 0 | 0.000000 | -0.000000 | 1183 | 919.897767 | 1014.9 | 1109.902233 | 0.857904 |  |
| GO:0032409\_regulation\_of\_transporter\_activity | 8 | 0 | 0.000000 | -0.000000 | 1183 | 919.897767 | 1014.9 | 1109.902233 | 0.857904 |  |
| GO:0032412\_regulation\_of\_ion\_transmembrane\_transporter\_activity | 8 | 0 | 0.000000 | -0.000000 | 1183 | 919.897767 | 1014.9 | 1109.902233 | 0.857904 |  |
| GO:0032613\_interleukin-10\_production | 8 | 0 | 0.000000 | -0.000000 | 1183 | 919.897767 | 1014.9 | 1109.902233 | 0.857904 |  |
| GO:0033198\_response\_to\_ATP | 8 | 0 | 0.000000 | -0.000000 | 1183 | 919.897767 | 1014.9 | 1109.902233 | 0.857904 |  |
| GO:0034284\_response\_to\_monosaccharide\_stimulus | 8 | 0 | 0.000000 | -0.000000 | 1183 | 919.897767 | 1014.9 | 1109.902233 | 0.857904 |  |
| GO:0034728\_nucleosome\_organization | 8 | 0 | 0.000000 | -0.000000 | 1183 | 919.897767 | 1014.9 | 1109.902233 | 0.857904 |  |
| GO:0035023\_regulation\_of\_Rho\_protein\_signal\_transduction | 8 | 0 | 0.000000 | -0.000000 | 1183 | 919.897767 | 1014.9 | 1109.902233 | 0.857904 |  |
| GO:0040017\_positive\_regulation\_of\_locomotion | 8 | 0 | 0.000000 | -0.000000 | 1183 | 919.897767 | 1014.9 | 1109.902233 | 0.857904 |  |
| GO:0040034\_regulation\_of\_development\_\_heterochronic | 8 | 0 | 0.000000 | -0.000000 | 1183 | 919.897767 | 1014.9 | 1109.902233 | 0.857904 |  |
| GO:0042074\_cell\_migration\_involved\_in\_gastrulation | 8 | 0 | 0.000000 | -0.000000 | 1183 | 919.897767 | 1014.9 | 1109.902233 | 0.857904 |  |
| GO:0042090\_interleukin-12\_biosynthetic\_process | 8 | 0 | 0.000000 | -0.000000 | 1183 | 919.897767 | 1014.9 | 1109.902233 | 0.857904 |  |
| GO:0042092\_T-helper\_2\_type\_immune\_response | 8 | 0 | 0.000000 | -0.000000 | 1183 | 919.897767 | 1014.9 | 1109.902233 | 0.857904 |  |
| GO:0042095\_interferon-gamma\_biosynthetic\_process | 8 | 0 | 0.000000 | -0.000000 | 1183 | 919.897767 | 1014.9 | 1109.902233 | 0.857904 |  |
| GO:0042104\_positive\_regulation\_of\_activated\_T\_cell\_proliferation | 8 | 0 | 0.000000 | -0.000000 | 1183 | 919.897767 | 1014.9 | 1109.902233 | 0.857904 |  |
| GO:0042226\_interleukin-6\_biosynthetic\_process | 8 | 0 | 0.000000 | -0.000000 | 1183 | 919.897767 | 1014.9 | 1109.902233 | 0.857904 |  |
| GO:0042304\_regulation\_of\_fatty\_acid\_biosynthetic\_process | 8 | 0 | 0.000000 | -0.000000 | 1183 | 919.897767 | 1014.9 | 1109.902233 | 0.857904 |  |
| GO:0042771\_DNA\_damage\_response\_\_signal\_transduction\_by\_p53\_class\_mediator\_resulting\_in\_induction\_of\_apoptosis | 8 | 0 | 0.000000 | -0.000000 | 1183 | 919.897767 | 1014.9 | 1109.902233 | 0.857904 |  |
| GO:0042990\_regulation\_of\_transcription\_factor\_import\_into\_nucleus | 8 | 0 | 0.000000 | -0.000000 | 1183 | 919.897767 | 1014.9 | 1109.902233 | 0.857904 |  |
| GO:0042991\_transcription\_factor\_import\_into\_nucleus | 8 | 0 | 0.000000 | -0.000000 | 1183 | 919.897767 | 1014.9 | 1109.902233 | 0.857904 |  |
| GO:0043011\_myeloid\_dendritic\_cell\_differentiation | 8 | 0 | 0.000000 | -0.000000 | 1183 | 919.897767 | 1014.9 | 1109.902233 | 0.857904 |  |
| GO:0043368\_positive\_T\_cell\_selection | 8 | 0 | 0.000000 | -0.000000 | 1183 | 919.897767 | 1014.9 | 1109.902233 | 0.857904 |  |
| GO:0043370\_regulation\_of\_CD4-positive\_\_alpha\_beta\_T\_cell\_differentiation | 8 | 0 | 0.000000 | -0.000000 | 1183 | 919.897767 | 1014.9 | 1109.902233 | 0.857904 |  |
| GO:0043542\_endothelial\_cell\_migration | 8 | 0 | 0.000000 | -0.000000 | 1183 | 919.897767 | 1014.9 | 1109.902233 | 0.857904 |  |
| GO:0043616\_keratinocyte\_proliferation | 8 | 0 | 0.000000 | -0.000000 | 1183 | 919.897767 | 1014.9 | 1109.902233 | 0.857904 |  |
| GO:0045075\_regulation\_of\_interleukin-12\_biosynthetic\_process | 8 | 0 | 0.000000 | -0.000000 | 1183 | 919.897767 | 1014.9 | 1109.902233 | 0.857904 |  |
| GO:0045086\_positive\_regulation\_of\_interleukin-2\_biosynthetic\_process | 8 | 0 | 0.000000 | -0.000000 | 1183 | 919.897767 | 1014.9 | 1109.902233 | 0.857904 |  |
| GO:0045351\_type\_I\_interferon\_biosynthetic\_process | 8 | 0 | 0.000000 | -0.000000 | 1183 | 919.897767 | 1014.9 | 1109.902233 | 0.857904 |  |
| GO:0045408\_regulation\_of\_interleukin-6\_biosynthetic\_process | 8 | 0 | 0.000000 | -0.000000 | 1183 | 919.897767 | 1014.9 | 1109.902233 | 0.857904 |  |
| GO:0045429\_positive\_regulation\_of\_nitric\_oxide\_biosynthetic\_process | 8 | 0 | 0.000000 | -0.000000 | 1183 | 919.897767 | 1014.9 | 1109.902233 | 0.857904 |  |
| GO:0045494\_photoreceptor\_cell\_maintenance | 8 | 0 | 0.000000 | -0.000000 | 1183 | 919.897767 | 1014.9 | 1109.902233 | 0.857904 |  |
| GO:0045686\_negative\_regulation\_of\_glial\_cell\_differentiation | 8 | 0 | 0.000000 | -0.000000 | 1183 | 919.897767 | 1014.9 | 1109.902233 | 0.857904 |  |
| GO:0045910\_negative\_regulation\_of\_DNA\_recombination | 8 | 0 | 0.000000 | -0.000000 | 1183 | 919.897767 | 1014.9 | 1109.902233 | 0.857904 |  |
| GO:0045921\_positive\_regulation\_of\_exocytosis | 8 | 0 | 0.000000 | -0.000000 | 1183 | 919.897767 | 1014.9 | 1109.902233 | 0.857904 |  |
| GO:0045932\_negative\_regulation\_of\_muscle\_contraction | 8 | 0 | 0.000000 | -0.000000 | 1183 | 919.897767 | 1014.9 | 1109.902233 | 0.857904 |  |
| GO:0046470\_phosphatidylcholine\_metabolic\_process | 8 | 0 | 0.000000 | -0.000000 | 1183 | 919.897767 | 1014.9 | 1109.902233 | 0.857904 |  |
| GO:0048266\_behavioral\_response\_to\_pain | 8 | 0 | 0.000000 | -0.000000 | 1183 | 919.897767 | 1014.9 | 1109.902233 | 0.857904 |  |
| GO:0048505\_regulation\_of\_timing\_of\_cell\_differentiation | 8 | 0 | 0.000000 | -0.000000 | 1183 | 919.897767 | 1014.9 | 1109.902233 | 0.857904 |  |
| GO:0048520\_positive\_regulation\_of\_behavior | 8 | 0 | 0.000000 | -0.000000 | 1183 | 919.897767 | 1014.9 | 1109.902233 | 0.857904 |  |
| GO:0048557\_embryonic\_digestive\_tract\_morphogenesis | 8 | 0 | 0.000000 | -0.000000 | 1183 | 919.897767 | 1014.9 | 1109.902233 | 0.857904 |  |
| GO:0048742\_regulation\_of\_skeletal\_muscle\_fiber\_development | 8 | 0 | 0.000000 | -0.000000 | 1183 | 919.897767 | 1014.9 | 1109.902233 | 0.857904 |  |
| GO:0050707\_regulation\_of\_cytokine\_secretion | 8 | 0 | 0.000000 | -0.000000 | 1183 | 919.897767 | 1014.9 | 1109.902233 | 0.857904 |  |
| GO:0050909\_sensory\_perception\_of\_taste | 8 | 0 | 0.000000 | -0.000000 | 1183 | 919.897767 | 1014.9 | 1109.902233 | 0.857904 |  |
| GO:0050920\_regulation\_of\_chemotaxis | 8 | 0 | 0.000000 | -0.000000 | 1183 | 919.897767 | 1014.9 | 1109.902233 | 0.857904 |  |
| GO:0050921\_positive\_regulation\_of\_chemotaxis | 8 | 0 | 0.000000 | -0.000000 | 1183 | 919.897767 | 1014.9 | 1109.902233 | 0.857904 |  |
| GO:0050926\_regulation\_of\_positive\_chemotaxis | 8 | 0 | 0.000000 | -0.000000 | 1183 | 919.897767 | 1014.9 | 1109.902233 | 0.857904 |  |
| GO:0050927\_positive\_regulation\_of\_positive\_chemotaxis | 8 | 0 | 0.000000 | -0.000000 | 1183 | 919.897767 | 1014.9 | 1109.902233 | 0.857904 |  |
| GO:0050930\_induction\_of\_positive\_chemotaxis | 8 | 0 | 0.000000 | -0.000000 | 1183 | 919.897767 | 1014.9 | 1109.902233 | 0.857904 |  |
| GO:0051084\_'de\_novo'\_posttranslational\_protein\_folding | 8 | 0 | 0.000000 | -0.000000 | 1183 | 919.897767 | 1014.9 | 1109.902233 | 0.857904 |  |
| GO:0051181\_cofactor\_transport | 8 | 0 | 0.000000 | -0.000000 | 1183 | 919.897767 | 1014.9 | 1109.902233 | 0.857904 |  |
| GO:0060712\_spongiotrophoblast\_layer\_development | 8 | 0 | 0.000000 | -0.000000 | 1183 | 919.897767 | 1014.9 | 1109.902233 | 0.857904 |  |
| GO:0070167\_regulation\_of\_biomineral\_formation | 8 | 0 | 0.000000 | -0.000000 | 1183 | 919.897767 | 1014.9 | 1109.902233 | 0.857904 |  |
| GO:0070193\_synaptonemal\_complex\_organization | 8 | 0 | 0.000000 | -0.000000 | 1183 | 919.897767 | 1014.9 | 1109.902233 | 0.857904 |  |
| GO:0070584\_mitochondrion\_morphogenesis | 8 | 0 | 0.000000 | -0.000000 | 1183 | 919.897767 | 1014.9 | 1109.902233 | 0.857904 |  |
| GO:0006816\_calcium\_ion\_transport | 69 | 0 | 0.000000 | -0.000000 | 1184 | 922.561035 | 1017.07 | 1111.578965 | 0.859012 |  |
| GO:0000280\_nuclear\_division | 24 | 0 | 0.000000 | -0.000000 | 1203 | 946.169345 | 1038.95 | 1131.730655 | 0.863633 |  |
| GO:0006650\_glycerophospholipid\_metabolic\_process | 24 | 0 | 0.000000 | -0.000000 | 1203 | 946.169345 | 1038.95 | 1131.730655 | 0.863633 |  |
| GO:0006941\_striated\_muscle\_contraction | 24 | 0 | 0.000000 | -0.000000 | 1203 | 946.169345 | 1038.95 | 1131.730655 | 0.863633 |  |
| GO:0006959\_humoral\_immune\_response | 24 | 0 | 0.000000 | -0.000000 | 1203 | 946.169345 | 1038.95 | 1131.730655 | 0.863633 |  |
| GO:0007050\_cell\_cycle\_arrest | 24 | 0 | 0.000000 | -0.000000 | 1203 | 946.169345 | 1038.95 | 1131.730655 | 0.863633 |  |
| GO:0007067\_mitosis | 24 | 0 | 0.000000 | -0.000000 | 1203 | 946.169345 | 1038.95 | 1131.730655 | 0.863633 |  |
| GO:0007204\_elevation\_of\_cytosolic\_calcium\_ion\_concentration | 24 | 0 | 0.000000 | -0.000000 | 1203 | 946.169345 | 1038.95 | 1131.730655 | 0.863633 |  |
| GO:0007259\_JAK-STAT\_cascade | 24 | 0 | 0.000000 | -0.000000 | 1203 | 946.169345 | 1038.95 | 1131.730655 | 0.863633 |  |
| GO:0007632\_visual\_behavior | 24 | 0 | 0.000000 | -0.000000 | 1203 | 946.169345 | 1038.95 | 1131.730655 | 0.863633 |  |
| GO:0008629\_induction\_of\_apoptosis\_by\_intracellular\_signals | 24 | 0 | 0.000000 | -0.000000 | 1203 | 946.169345 | 1038.95 | 1131.730655 | 0.863633 |  |
| GO:0009612\_response\_to\_mechanical\_stimulus | 24 | 0 | 0.000000 | -0.000000 | 1203 | 946.169345 | 1038.95 | 1131.730655 | 0.863633 |  |
| GO:0014070\_response\_to\_organic\_cyclic\_substance | 24 | 0 | 0.000000 | -0.000000 | 1203 | 946.169345 | 1038.95 | 1131.730655 | 0.863633 |  |
| GO:0021515\_cell\_differentiation\_in\_spinal\_cord | 24 | 0 | 0.000000 | -0.000000 | 1203 | 946.169345 | 1038.95 | 1131.730655 | 0.863633 |  |
| GO:0032386\_regulation\_of\_intracellular\_transport | 24 | 0 | 0.000000 | -0.000000 | 1203 | 946.169345 | 1038.95 | 1131.730655 | 0.863633 |  |
| GO:0043588\_skin\_development | 24 | 0 | 0.000000 | -0.000000 | 1203 | 946.169345 | 1038.95 | 1131.730655 | 0.863633 |  |
| GO:0048002\_antigen\_processing\_and\_presentation\_of\_peptide\_antigen | 24 | 0 | 0.000000 | -0.000000 | 1203 | 946.169345 | 1038.95 | 1131.730655 | 0.863633 |  |
| GO:0051099\_positive\_regulation\_of\_binding | 24 | 0 | 0.000000 | -0.000000 | 1203 | 946.169345 | 1038.95 | 1131.730655 | 0.863633 |  |
| GO:0060078\_regulation\_of\_postsynaptic\_membrane\_potential | 24 | 0 | 0.000000 | -0.000000 | 1203 | 946.169345 | 1038.95 | 1131.730655 | 0.863633 |  |
| GO:0070667\_negative\_regulation\_of\_mast\_cell\_proliferation | 24 | 0 | 0.000000 | -0.000000 | 1203 | 946.169345 | 1038.95 | 1131.730655 | 0.863633 |  |
| GO:0002237\_response\_to\_molecule\_of\_bacterial\_origin | 34 | 0 | 0.000000 | -0.000000 | 1213 | 959.187670 | 1050.87 | 1142.552330 | 0.866340 |  |
| GO:0002699\_positive\_regulation\_of\_immune\_effector\_process | 34 | 0 | 0.000000 | -0.000000 | 1213 | 959.187670 | 1050.87 | 1142.552330 | 0.866340 |  |
| GO:0007269\_neurotransmitter\_secretion | 34 | 0 | 0.000000 | -0.000000 | 1213 | 959.187670 | 1050.87 | 1142.552330 | 0.866340 |  |
| GO:0007568\_aging | 34 | 0 | 0.000000 | -0.000000 | 1213 | 959.187670 | 1050.87 | 1142.552330 | 0.866340 |  |
| GO:0010721\_negative\_regulation\_of\_cell\_development | 34 | 0 | 0.000000 | -0.000000 | 1213 | 959.187670 | 1050.87 | 1142.552330 | 0.866340 |  |
| GO:0016054\_organic\_acid\_catabolic\_process | 34 | 0 | 0.000000 | -0.000000 | 1213 | 959.187670 | 1050.87 | 1142.552330 | 0.866340 |  |
| GO:0019882\_antigen\_processing\_and\_presentation | 34 | 0 | 0.000000 | -0.000000 | 1213 | 959.187670 | 1050.87 | 1142.552330 | 0.866340 |  |
| GO:0046395\_carboxylic\_acid\_catabolic\_process | 34 | 0 | 0.000000 | -0.000000 | 1213 | 959.187670 | 1050.87 | 1142.552330 | 0.866340 |  |
| GO:0050730\_regulation\_of\_peptidyl-tyrosine\_phosphorylation | 34 | 0 | 0.000000 | -0.000000 | 1213 | 959.187670 | 1050.87 | 1142.552330 | 0.866340 |  |
| GO:0051052\_regulation\_of\_DNA\_metabolic\_process | 34 | 0 | 0.000000 | -0.000000 | 1213 | 959.187670 | 1050.87 | 1142.552330 | 0.866340 |  |
| GO:0006417\_regulation\_of\_translation | 29 | 0 | 0.000000 | -0.000000 | 1226 | 975.849454 | 1066.46 | 1157.070546 | 0.869869 |  |
| GO:0006641\_triglyceride\_metabolic\_process | 29 | 0 | 0.000000 | -0.000000 | 1226 | 975.849454 | 1066.46 | 1157.070546 | 0.869869 |  |
| GO:0006909\_phagocytosis | 29 | 0 | 0.000000 | -0.000000 | 1226 | 975.849454 | 1066.46 | 1157.070546 | 0.869869 |  |
| GO:0007190\_activation\_of\_adenylate\_cyclase\_activity | 29 | 0 | 0.000000 | -0.000000 | 1226 | 975.849454 | 1066.46 | 1157.070546 | 0.869869 |  |
| GO:0010564\_regulation\_of\_cell\_cycle\_process | 29 | 0 | 0.000000 | -0.000000 | 1226 | 975.849454 | 1066.46 | 1157.070546 | 0.869869 |  |
| GO:0042176\_regulation\_of\_protein\_catabolic\_process | 29 | 0 | 0.000000 | -0.000000 | 1226 | 975.849454 | 1066.46 | 1157.070546 | 0.869869 |  |
| GO:0042770\_DNA\_damage\_response\_\_signal\_transduction | 29 | 0 | 0.000000 | -0.000000 | 1226 | 975.849454 | 1066.46 | 1157.070546 | 0.869869 |  |
| GO:0043281\_regulation\_of\_caspase\_activity | 29 | 0 | 0.000000 | -0.000000 | 1226 | 975.849454 | 1066.46 | 1157.070546 | 0.869869 |  |
| GO:0044270\_nitrogen\_compound\_catabolic\_process | 29 | 0 | 0.000000 | -0.000000 | 1226 | 975.849454 | 1066.46 | 1157.070546 | 0.869869 |  |
| GO:0045621\_positive\_regulation\_of\_lymphocyte\_differentiation | 29 | 0 | 0.000000 | -0.000000 | 1226 | 975.849454 | 1066.46 | 1157.070546 | 0.869869 |  |
| GO:0046634\_regulation\_of\_alpha-beta\_T\_cell\_activation | 29 | 0 | 0.000000 | -0.000000 | 1226 | 975.849454 | 1066.46 | 1157.070546 | 0.869869 |  |
| GO:0048066\_pigmentation\_during\_development | 29 | 0 | 0.000000 | -0.000000 | 1226 | 975.849454 | 1066.46 | 1157.070546 | 0.869869 |  |
| GO:0052548\_regulation\_of\_endopeptidase\_activity | 29 | 0 | 0.000000 | -0.000000 | 1226 | 975.849454 | 1066.46 | 1157.070546 | 0.869869 |  |
| GO:0001818\_negative\_regulation\_of\_cytokine\_production | 18 | 0 | 0.000000 | -0.000000 | 1262 | 1020.549732 | 1109.15 | 1197.750268 | 0.878883 |  |
| GO:0002064\_epithelial\_cell\_development | 18 | 0 | 0.000000 | -0.000000 | 1262 | 1020.549732 | 1109.15 | 1197.750268 | 0.878883 |  |
| GO:0002285\_lymphocyte\_activation\_during\_immune\_response | 18 | 0 | 0.000000 | -0.000000 | 1262 | 1020.549732 | 1109.15 | 1197.750268 | 0.878883 |  |
| GO:0002715\_regulation\_of\_natural\_killer\_cell\_mediated\_immunity | 18 | 0 | 0.000000 | -0.000000 | 1262 | 1020.549732 | 1109.15 | 1197.750268 | 0.878883 |  |
| GO:0003014\_renal\_system\_process | 18 | 0 | 0.000000 | -0.000000 | 1262 | 1020.549732 | 1109.15 | 1197.750268 | 0.878883 |  |
| GO:0006457\_protein\_folding | 18 | 0 | 0.000000 | -0.000000 | 1262 | 1020.549732 | 1109.15 | 1197.750268 | 0.878883 |  |
| GO:0006940\_regulation\_of\_smooth\_muscle\_contraction | 18 | 0 | 0.000000 | -0.000000 | 1262 | 1020.549732 | 1109.15 | 1197.750268 | 0.878883 |  |
| GO:0007140\_male\_meiosis | 18 | 0 | 0.000000 | -0.000000 | 1262 | 1020.549732 | 1109.15 | 1197.750268 | 0.878883 |  |
| GO:0007608\_sensory\_perception\_of\_smell | 18 | 0 | 0.000000 | -0.000000 | 1262 | 1020.549732 | 1109.15 | 1197.750268 | 0.878883 |  |
| GO:0009063\_cellular\_amino\_acid\_catabolic\_process | 18 | 0 | 0.000000 | -0.000000 | 1262 | 1020.549732 | 1109.15 | 1197.750268 | 0.878883 |  |
| GO:0010498\_proteasomal\_protein\_catabolic\_process | 18 | 0 | 0.000000 | -0.000000 | 1262 | 1020.549732 | 1109.15 | 1197.750268 | 0.878883 |  |
| GO:0010553\_negative\_regulation\_of\_specific\_transcription\_from\_RNA\_polymerase\_II\_promoter | 18 | 0 | 0.000000 | -0.000000 | 1262 | 1020.549732 | 1109.15 | 1197.750268 | 0.878883 |  |
| GO:0015711\_organic\_anion\_transport | 18 | 0 | 0.000000 | -0.000000 | 1262 | 1020.549732 | 1109.15 | 1197.750268 | 0.878883 |  |
| GO:0016458\_gene\_silencing | 18 | 0 | 0.000000 | -0.000000 | 1262 | 1020.549732 | 1109.15 | 1197.750268 | 0.878883 |  |
| GO:0021517\_ventral\_spinal\_cord\_development | 18 | 0 | 0.000000 | -0.000000 | 1262 | 1020.549732 | 1109.15 | 1197.750268 | 0.878883 |  |
| GO:0030178\_negative\_regulation\_of\_Wnt\_receptor\_signaling\_pathway | 18 | 0 | 0.000000 | -0.000000 | 1262 | 1020.549732 | 1109.15 | 1197.750268 | 0.878883 |  |
| GO:0030318\_melanocyte\_differentiation | 18 | 0 | 0.000000 | -0.000000 | 1262 | 1020.549732 | 1109.15 | 1197.750268 | 0.878883 |  |
| GO:0030336\_negative\_regulation\_of\_cell\_migration | 18 | 0 | 0.000000 | -0.000000 | 1262 | 1020.549732 | 1109.15 | 1197.750268 | 0.878883 |  |
| GO:0030510\_regulation\_of\_BMP\_signaling\_pathway | 18 | 0 | 0.000000 | -0.000000 | 1262 | 1020.549732 | 1109.15 | 1197.750268 | 0.878883 |  |
| GO:0030901\_midbrain\_development | 18 | 0 | 0.000000 | -0.000000 | 1262 | 1020.549732 | 1109.15 | 1197.750268 | 0.878883 |  |
| GO:0032623\_interleukin-2\_production | 18 | 0 | 0.000000 | -0.000000 | 1262 | 1020.549732 | 1109.15 | 1197.750268 | 0.878883 |  |
| GO:0033157\_regulation\_of\_intracellular\_protein\_transport | 18 | 0 | 0.000000 | -0.000000 | 1262 | 1020.549732 | 1109.15 | 1197.750268 | 0.878883 |  |
| GO:0042269\_regulation\_of\_natural\_killer\_cell\_mediated\_cytotoxicity | 18 | 0 | 0.000000 | -0.000000 | 1262 | 1020.549732 | 1109.15 | 1197.750268 | 0.878883 |  |
| GO:0043161\_proteasomal\_ubiquitin-dependent\_protein\_catabolic\_process | 18 | 0 | 0.000000 | -0.000000 | 1262 | 1020.549732 | 1109.15 | 1197.750268 | 0.878883 |  |
| GO:0044272\_sulfur\_compound\_biosynthetic\_process | 18 | 0 | 0.000000 | -0.000000 | 1262 | 1020.549732 | 1109.15 | 1197.750268 | 0.878883 |  |
| GO:0045058\_T\_cell\_selection | 18 | 0 | 0.000000 | -0.000000 | 1262 | 1020.549732 | 1109.15 | 1197.750268 | 0.878883 |  |
| GO:0045103\_intermediate\_filament-based\_process | 18 | 0 | 0.000000 | -0.000000 | 1262 | 1020.549732 | 1109.15 | 1197.750268 | 0.878883 |  |
| GO:0045807\_positive\_regulation\_of\_endocytosis | 18 | 0 | 0.000000 | -0.000000 | 1262 | 1020.549732 | 1109.15 | 1197.750268 | 0.878883 |  |
| GO:0046578\_regulation\_of\_Ras\_protein\_signal\_transduction | 18 | 0 | 0.000000 | -0.000000 | 1262 | 1020.549732 | 1109.15 | 1197.750268 | 0.878883 |  |
| GO:0048535\_lymph\_node\_development | 18 | 0 | 0.000000 | -0.000000 | 1262 | 1020.549732 | 1109.15 | 1197.750268 | 0.878883 |  |
| GO:0048813\_dendrite\_morphogenesis | 18 | 0 | 0.000000 | -0.000000 | 1262 | 1020.549732 | 1109.15 | 1197.750268 | 0.878883 |  |
| GO:0050731\_positive\_regulation\_of\_peptidyl-tyrosine\_phosphorylation | 18 | 0 | 0.000000 | -0.000000 | 1262 | 1020.549732 | 1109.15 | 1197.750268 | 0.878883 |  |
| GO:0050982\_detection\_of\_mechanical\_stimulus | 18 | 0 | 0.000000 | -0.000000 | 1262 | 1020.549732 | 1109.15 | 1197.750268 | 0.878883 |  |
| GO:0051168\_nuclear\_export | 18 | 0 | 0.000000 | -0.000000 | 1262 | 1020.549732 | 1109.15 | 1197.750268 | 0.878883 |  |
| GO:0051924\_regulation\_of\_calcium\_ion\_transport | 18 | 0 | 0.000000 | -0.000000 | 1262 | 1020.549732 | 1109.15 | 1197.750268 | 0.878883 |  |
| GO:0060674\_placenta\_blood\_vessel\_development | 18 | 0 | 0.000000 | -0.000000 | 1262 | 1020.549732 | 1109.15 | 1197.750268 | 0.878883 |  |
| GO:0002764\_immune\_response-regulating\_signal\_transduction | 51 | 0 | 0.000000 | -0.000000 | 1267 | 1026.286623 | 1114.23 | 1202.173377 | 0.879424 |  |
| GO:0006520\_cellular\_amino\_acid\_metabolic\_process | 51 | 0 | 0.000000 | -0.000000 | 1267 | 1026.286623 | 1114.23 | 1202.173377 | 0.879424 |  |
| GO:0016569\_covalent\_chromatin\_modification | 51 | 0 | 0.000000 | -0.000000 | 1267 | 1026.286623 | 1114.23 | 1202.173377 | 0.879424 |  |
| GO:0044106\_cellular\_amine\_metabolic\_process | 51 | 0 | 0.000000 | -0.000000 | 1267 | 1026.286623 | 1114.23 | 1202.173377 | 0.879424 |  |
| GO:0048747\_muscle\_fiber\_development | 51 | 0 | 0.000000 | -0.000000 | 1267 | 1026.286623 | 1114.23 | 1202.173377 | 0.879424 |  |
| GO:0000079\_regulation\_of\_cyclin-dependent\_protein\_kinase\_activity | 7 | 0 | 0.000000 | -0.000000 | 1437 | 1196.912260 | 1281.51 | 1366.107740 | 0.891795 |  |
| GO:0000188\_inactivation\_of\_MAPK\_activity | 7 | 0 | 0.000000 | -0.000000 | 1437 | 1196.912260 | 1281.51 | 1366.107740 | 0.891795 |  |
| GO:0001504\_neurotransmitter\_uptake | 7 | 0 | 0.000000 | -0.000000 | 1437 | 1196.912260 | 1281.51 | 1366.107740 | 0.891795 |  |
| GO:0001556\_oocyte\_maturation | 7 | 0 | 0.000000 | -0.000000 | 1437 | 1196.912260 | 1281.51 | 1366.107740 | 0.891795 |  |
| GO:0001573\_ganglioside\_metabolic\_process | 7 | 0 | 0.000000 | -0.000000 | 1437 | 1196.912260 | 1281.51 | 1366.107740 | 0.891795 |  |
| GO:0001736\_establishment\_of\_planar\_polarity | 7 | 0 | 0.000000 | -0.000000 | 1437 | 1196.912260 | 1281.51 | 1366.107740 | 0.891795 |  |
| GO:0001839\_neural\_plate\_morphogenesis | 7 | 0 | 0.000000 | -0.000000 | 1437 | 1196.912260 | 1281.51 | 1366.107740 | 0.891795 |  |
| GO:0001936\_regulation\_of\_endothelial\_cell\_proliferation | 7 | 0 | 0.000000 | -0.000000 | 1437 | 1196.912260 | 1281.51 | 1366.107740 | 0.891795 |  |
| GO:0001967\_suckling\_behavior | 7 | 0 | 0.000000 | -0.000000 | 1437 | 1196.912260 | 1281.51 | 1366.107740 | 0.891795 |  |
| GO:0002011\_morphogenesis\_of\_an\_epithelial\_sheet | 7 | 0 | 0.000000 | -0.000000 | 1437 | 1196.912260 | 1281.51 | 1366.107740 | 0.891795 |  |
| GO:0002052\_positive\_regulation\_of\_neuroblast\_proliferation | 7 | 0 | 0.000000 | -0.000000 | 1437 | 1196.912260 | 1281.51 | 1366.107740 | 0.891795 |  |
| GO:0002063\_chondrocyte\_development | 7 | 0 | 0.000000 | -0.000000 | 1437 | 1196.912260 | 1281.51 | 1366.107740 | 0.891795 |  |
| GO:0002067\_glandular\_epithelial\_cell\_differentiation | 7 | 0 | 0.000000 | -0.000000 | 1437 | 1196.912260 | 1281.51 | 1366.107740 | 0.891795 |  |
| GO:0002076\_osteoblast\_development | 7 | 0 | 0.000000 | -0.000000 | 1437 | 1196.912260 | 1281.51 | 1366.107740 | 0.891795 |  |
| GO:0002087\_regulation\_of\_respiratory\_gaseous\_exchange\_by\_neurological\_system\_process | 7 | 0 | 0.000000 | -0.000000 | 1437 | 1196.912260 | 1281.51 | 1366.107740 | 0.891795 |  |
| GO:0002093\_auditory\_receptor\_cell\_morphogenesis | 7 | 0 | 0.000000 | -0.000000 | 1437 | 1196.912260 | 1281.51 | 1366.107740 | 0.891795 |  |
| GO:0002224\_toll-like\_receptor\_signaling\_pathway | 7 | 0 | 0.000000 | -0.000000 | 1437 | 1196.912260 | 1281.51 | 1366.107740 | 0.891795 |  |
| GO:0002455\_humoral\_immune\_response\_mediated\_by\_circulating\_immunoglobulin | 7 | 0 | 0.000000 | -0.000000 | 1437 | 1196.912260 | 1281.51 | 1366.107740 | 0.891795 |  |
| GO:0002643\_regulation\_of\_tolerance\_induction | 7 | 0 | 0.000000 | -0.000000 | 1437 | 1196.912260 | 1281.51 | 1366.107740 | 0.891795 |  |
| GO:0002645\_positive\_regulation\_of\_tolerance\_induction | 7 | 0 | 0.000000 | -0.000000 | 1437 | 1196.912260 | 1281.51 | 1366.107740 | 0.891795 |  |
| GO:0002714\_positive\_regulation\_of\_B\_cell\_mediated\_immunity | 7 | 0 | 0.000000 | -0.000000 | 1437 | 1196.912260 | 1281.51 | 1366.107740 | 0.891795 |  |
| GO:0002792\_negative\_regulation\_of\_peptide\_secretion | 7 | 0 | 0.000000 | -0.000000 | 1437 | 1196.912260 | 1281.51 | 1366.107740 | 0.891795 |  |
| GO:0002793\_positive\_regulation\_of\_peptide\_secretion | 7 | 0 | 0.000000 | -0.000000 | 1437 | 1196.912260 | 1281.51 | 1366.107740 | 0.891795 |  |
| GO:0002828\_regulation\_of\_T-helper\_2\_type\_immune\_response | 7 | 0 | 0.000000 | -0.000000 | 1437 | 1196.912260 | 1281.51 | 1366.107740 | 0.891795 |  |
| GO:0002863\_positive\_regulation\_of\_inflammatory\_response\_to\_antigenic\_stimulus | 7 | 0 | 0.000000 | -0.000000 | 1437 | 1196.912260 | 1281.51 | 1366.107740 | 0.891795 |  |
| GO:0002891\_positive\_regulation\_of\_immunoglobulin\_mediated\_immune\_response | 7 | 0 | 0.000000 | -0.000000 | 1437 | 1196.912260 | 1281.51 | 1366.107740 | 0.891795 |  |
| GO:0003084\_positive\_regulation\_of\_systemic\_arterial\_blood\_pressure | 7 | 0 | 0.000000 | -0.000000 | 1437 | 1196.912260 | 1281.51 | 1366.107740 | 0.891795 |  |
| GO:0003085\_negative\_regulation\_of\_systemic\_arterial\_blood\_pressure | 7 | 0 | 0.000000 | -0.000000 | 1437 | 1196.912260 | 1281.51 | 1366.107740 | 0.891795 |  |
| GO:0006014\_D-ribose\_metabolic\_process | 7 | 0 | 0.000000 | -0.000000 | 1437 | 1196.912260 | 1281.51 | 1366.107740 | 0.891795 |  |
| GO:0006041\_glucosamine\_metabolic\_process | 7 | 0 | 0.000000 | -0.000000 | 1437 | 1196.912260 | 1281.51 | 1366.107740 | 0.891795 |  |
| GO:0006044\_N-acetylglucosamine\_metabolic\_process | 7 | 0 | 0.000000 | -0.000000 | 1437 | 1196.912260 | 1281.51 | 1366.107740 | 0.891795 |  |
| GO:0006096\_glycolysis | 7 | 0 | 0.000000 | -0.000000 | 1437 | 1196.912260 | 1281.51 | 1366.107740 | 0.891795 |  |
| GO:0006119\_oxidative\_phosphorylation | 7 | 0 | 0.000000 | -0.000000 | 1437 | 1196.912260 | 1281.51 | 1366.107740 | 0.891795 |  |
| GO:0006275\_regulation\_of\_DNA\_replication | 7 | 0 | 0.000000 | -0.000000 | 1437 | 1196.912260 | 1281.51 | 1366.107740 | 0.891795 |  |
| GO:0006298\_mismatch\_repair | 7 | 0 | 0.000000 | -0.000000 | 1437 | 1196.912260 | 1281.51 | 1366.107740 | 0.891795 |  |
| GO:0006352\_transcription\_initiation | 7 | 0 | 0.000000 | -0.000000 | 1437 | 1196.912260 | 1281.51 | 1366.107740 | 0.891795 |  |
| GO:0006401\_RNA\_catabolic\_process | 7 | 0 | 0.000000 | -0.000000 | 1437 | 1196.912260 | 1281.51 | 1366.107740 | 0.891795 |  |
| GO:0006406\_mRNA\_export\_from\_nucleus | 7 | 0 | 0.000000 | -0.000000 | 1437 | 1196.912260 | 1281.51 | 1366.107740 | 0.891795 |  |
| GO:0006505\_GPI\_anchor\_metabolic\_process | 7 | 0 | 0.000000 | -0.000000 | 1437 | 1196.912260 | 1281.51 | 1366.107740 | 0.891795 |  |
| GO:0006516\_glycoprotein\_catabolic\_process | 7 | 0 | 0.000000 | -0.000000 | 1437 | 1196.912260 | 1281.51 | 1366.107740 | 0.891795 |  |
| GO:0006612\_protein\_targeting\_to\_membrane | 7 | 0 | 0.000000 | -0.000000 | 1437 | 1196.912260 | 1281.51 | 1366.107740 | 0.891795 |  |
| GO:0006769\_nicotinamide\_metabolic\_process | 7 | 0 | 0.000000 | -0.000000 | 1437 | 1196.912260 | 1281.51 | 1366.107740 | 0.891795 |  |
| GO:0006783\_heme\_biosynthetic\_process | 7 | 0 | 0.000000 | -0.000000 | 1437 | 1196.912260 | 1281.51 | 1366.107740 | 0.891795 |  |
| GO:0006878\_cellular\_copper\_ion\_homeostasis | 7 | 0 | 0.000000 | -0.000000 | 1437 | 1196.912260 | 1281.51 | 1366.107740 | 0.891795 |  |
| GO:0006884\_cell\_volume\_homeostasis | 7 | 0 | 0.000000 | -0.000000 | 1437 | 1196.912260 | 1281.51 | 1366.107740 | 0.891795 |  |
| GO:0006949\_syncytium\_formation | 7 | 0 | 0.000000 | -0.000000 | 1437 | 1196.912260 | 1281.51 | 1366.107740 | 0.891795 |  |
| GO:0007019\_microtubule\_depolymerization | 7 | 0 | 0.000000 | -0.000000 | 1437 | 1196.912260 | 1281.51 | 1366.107740 | 0.891795 |  |
| GO:0007026\_negative\_regulation\_of\_microtubule\_depolymerization | 7 | 0 | 0.000000 | -0.000000 | 1437 | 1196.912260 | 1281.51 | 1366.107740 | 0.891795 |  |
| GO:0007034\_vacuolar\_transport | 7 | 0 | 0.000000 | -0.000000 | 1437 | 1196.912260 | 1281.51 | 1366.107740 | 0.891795 |  |
| GO:0007062\_sister\_chromatid\_cohesion | 7 | 0 | 0.000000 | -0.000000 | 1437 | 1196.912260 | 1281.51 | 1366.107740 | 0.891795 |  |
| GO:0007130\_synaptonemal\_complex\_assembly | 7 | 0 | 0.000000 | -0.000000 | 1437 | 1196.912260 | 1281.51 | 1366.107740 | 0.891795 |  |
| GO:0007164\_establishment\_of\_tissue\_polarity | 7 | 0 | 0.000000 | -0.000000 | 1437 | 1196.912260 | 1281.51 | 1366.107740 | 0.891795 |  |
| GO:0007191\_activation\_of\_adenylate\_cyclase\_activity\_by\_dopamine\_receptor\_signaling\_pathway | 7 | 0 | 0.000000 | -0.000000 | 1437 | 1196.912260 | 1281.51 | 1366.107740 | 0.891795 |  |
| GO:0007271\_synaptic\_transmission\_\_cholinergic | 7 | 0 | 0.000000 | -0.000000 | 1437 | 1196.912260 | 1281.51 | 1366.107740 | 0.891795 |  |
| GO:0007413\_axonal\_fasciculation | 7 | 0 | 0.000000 | -0.000000 | 1437 | 1196.912260 | 1281.51 | 1366.107740 | 0.891795 |  |
| GO:0007616\_long-term\_memory | 7 | 0 | 0.000000 | -0.000000 | 1437 | 1196.912260 | 1281.51 | 1366.107740 | 0.891795 |  |
| GO:0008033\_tRNA\_processing | 7 | 0 | 0.000000 | -0.000000 | 1437 | 1196.912260 | 1281.51 | 1366.107740 | 0.891795 |  |
| GO:0008299\_isoprenoid\_biosynthetic\_process | 7 | 0 | 0.000000 | -0.000000 | 1437 | 1196.912260 | 1281.51 | 1366.107740 | 0.891795 |  |
| GO:0008340\_determination\_of\_adult\_lifespan | 7 | 0 | 0.000000 | -0.000000 | 1437 | 1196.912260 | 1281.51 | 1366.107740 | 0.891795 |  |
| GO:0009150\_purine\_ribonucleotide\_metabolic\_process | 7 | 0 | 0.000000 | -0.000000 | 1437 | 1196.912260 | 1281.51 | 1366.107740 | 0.891795 |  |
| GO:0009200\_deoxyribonucleoside\_triphosphate\_metabolic\_process | 7 | 0 | 0.000000 | -0.000000 | 1437 | 1196.912260 | 1281.51 | 1366.107740 | 0.891795 |  |
| GO:0009259\_ribonucleotide\_metabolic\_process | 7 | 0 | 0.000000 | -0.000000 | 1437 | 1196.912260 | 1281.51 | 1366.107740 | 0.891795 |  |
| GO:0009311\_oligosaccharide\_metabolic\_process | 7 | 0 | 0.000000 | -0.000000 | 1437 | 1196.912260 | 1281.51 | 1366.107740 | 0.891795 |  |
| GO:0009394\_2'-deoxyribonucleotide\_metabolic\_process | 7 | 0 | 0.000000 | -0.000000 | 1437 | 1196.912260 | 1281.51 | 1366.107740 | 0.891795 |  |
| GO:0009820\_alkaloid\_metabolic\_process | 7 | 0 | 0.000000 | -0.000000 | 1437 | 1196.912260 | 1281.51 | 1366.107740 | 0.891795 |  |
| GO:0010469\_regulation\_of\_receptor\_activity | 7 | 0 | 0.000000 | -0.000000 | 1437 | 1196.912260 | 1281.51 | 1366.107740 | 0.891795 |  |
| GO:0010948\_negative\_regulation\_of\_cell\_cycle\_process | 7 | 0 | 0.000000 | -0.000000 | 1437 | 1196.912260 | 1281.51 | 1366.107740 | 0.891795 |  |
| GO:0014047\_glutamate\_secretion | 7 | 0 | 0.000000 | -0.000000 | 1437 | 1196.912260 | 1281.51 | 1366.107740 | 0.891795 |  |
| GO:0014066\_regulation\_of\_phosphoinositide\_3-kinase\_cascade | 7 | 0 | 0.000000 | -0.000000 | 1437 | 1196.912260 | 1281.51 | 1366.107740 | 0.891795 |  |
| GO:0014821\_phasic\_smooth\_muscle\_contraction | 7 | 0 | 0.000000 | -0.000000 | 1437 | 1196.912260 | 1281.51 | 1366.107740 | 0.891795 |  |
| GO:0015697\_quaternary\_ammonium\_group\_transport | 7 | 0 | 0.000000 | -0.000000 | 1437 | 1196.912260 | 1281.51 | 1366.107740 | 0.891795 |  |
| GO:0015813\_L-glutamate\_transport | 7 | 0 | 0.000000 | -0.000000 | 1437 | 1196.912260 | 1281.51 | 1366.107740 | 0.891795 |  |
| GO:0015908\_fatty\_acid\_transport | 7 | 0 | 0.000000 | -0.000000 | 1437 | 1196.912260 | 1281.51 | 1366.107740 | 0.891795 |  |
| GO:0015914\_phospholipid\_transport | 7 | 0 | 0.000000 | -0.000000 | 1437 | 1196.912260 | 1281.51 | 1366.107740 | 0.891795 |  |
| GO:0016339\_calcium-dependent\_cell-cell\_adhesion | 7 | 0 | 0.000000 | -0.000000 | 1437 | 1196.912260 | 1281.51 | 1366.107740 | 0.891795 |  |
| GO:0016575\_histone\_deacetylation | 7 | 0 | 0.000000 | -0.000000 | 1437 | 1196.912260 | 1281.51 | 1366.107740 | 0.891795 |  |
| GO:0019362\_pyridine\_nucleotide\_metabolic\_process | 7 | 0 | 0.000000 | -0.000000 | 1437 | 1196.912260 | 1281.51 | 1366.107740 | 0.891795 |  |
| GO:0019692\_deoxyribose\_phosphate\_metabolic\_process | 7 | 0 | 0.000000 | -0.000000 | 1437 | 1196.912260 | 1281.51 | 1366.107740 | 0.891795 |  |
| GO:0020027\_hemoglobin\_metabolic\_process | 7 | 0 | 0.000000 | -0.000000 | 1437 | 1196.912260 | 1281.51 | 1366.107740 | 0.891795 |  |
| GO:0021514\_ventral\_spinal\_cord\_interneuron\_differentiation | 7 | 0 | 0.000000 | -0.000000 | 1437 | 1196.912260 | 1281.51 | 1366.107740 | 0.891795 |  |
| GO:0021516\_dorsal\_spinal\_cord\_development | 7 | 0 | 0.000000 | -0.000000 | 1437 | 1196.912260 | 1281.51 | 1366.107740 | 0.891795 |  |
| GO:0021520\_spinal\_cord\_motor\_neuron\_cell\_fate\_specification | 7 | 0 | 0.000000 | -0.000000 | 1437 | 1196.912260 | 1281.51 | 1366.107740 | 0.891795 |  |
| GO:0021521\_ventral\_spinal\_cord\_interneuron\_specification | 7 | 0 | 0.000000 | -0.000000 | 1437 | 1196.912260 | 1281.51 | 1366.107740 | 0.891795 |  |
| GO:0021546\_rhombomere\_development | 7 | 0 | 0.000000 | -0.000000 | 1437 | 1196.912260 | 1281.51 | 1366.107740 | 0.891795 |  |
| GO:0021756\_striatum\_development | 7 | 0 | 0.000000 | -0.000000 | 1437 | 1196.912260 | 1281.51 | 1366.107740 | 0.891795 |  |
| GO:0021884\_forebrain\_neuron\_development | 7 | 0 | 0.000000 | -0.000000 | 1437 | 1196.912260 | 1281.51 | 1366.107740 | 0.891795 |  |
| GO:0021903\_rostrocaudal\_neural\_tube\_patterning | 7 | 0 | 0.000000 | -0.000000 | 1437 | 1196.912260 | 1281.51 | 1366.107740 | 0.891795 |  |
| GO:0021984\_adenohypophysis\_development | 7 | 0 | 0.000000 | -0.000000 | 1437 | 1196.912260 | 1281.51 | 1366.107740 | 0.891795 |  |
| GO:0022618\_ribonucleoprotein\_complex\_assembly | 7 | 0 | 0.000000 | -0.000000 | 1437 | 1196.912260 | 1281.51 | 1366.107740 | 0.891795 |  |
| GO:0030104\_water\_homeostasis | 7 | 0 | 0.000000 | -0.000000 | 1437 | 1196.912260 | 1281.51 | 1366.107740 | 0.891795 |  |
| GO:0030201\_heparan\_sulfate\_proteoglycan\_metabolic\_process | 7 | 0 | 0.000000 | -0.000000 | 1437 | 1196.912260 | 1281.51 | 1366.107740 | 0.891795 |  |
| GO:0030432\_peristalsis | 7 | 0 | 0.000000 | -0.000000 | 1437 | 1196.912260 | 1281.51 | 1366.107740 | 0.891795 |  |
| GO:0030517\_negative\_regulation\_of\_axon\_extension | 7 | 0 | 0.000000 | -0.000000 | 1437 | 1196.912260 | 1281.51 | 1366.107740 | 0.891795 |  |
| GO:0030520\_estrogen\_receptor\_signaling\_pathway | 7 | 0 | 0.000000 | -0.000000 | 1437 | 1196.912260 | 1281.51 | 1366.107740 | 0.891795 |  |
| GO:0030521\_androgen\_receptor\_signaling\_pathway | 7 | 0 | 0.000000 | -0.000000 | 1437 | 1196.912260 | 1281.51 | 1366.107740 | 0.891795 |  |
| GO:0030903\_notochord\_development | 7 | 0 | 0.000000 | -0.000000 | 1437 | 1196.912260 | 1281.51 | 1366.107740 | 0.891795 |  |
| GO:0031017\_exocrine\_pancreas\_development | 7 | 0 | 0.000000 | -0.000000 | 1437 | 1196.912260 | 1281.51 | 1366.107740 | 0.891795 |  |
| GO:0031114\_regulation\_of\_microtubule\_depolymerization | 7 | 0 | 0.000000 | -0.000000 | 1437 | 1196.912260 | 1281.51 | 1366.107740 | 0.891795 |  |
| GO:0031124\_mRNA\_3'-end\_processing | 7 | 0 | 0.000000 | -0.000000 | 1437 | 1196.912260 | 1281.51 | 1366.107740 | 0.891795 |  |
| GO:0031497\_chromatin\_assembly | 7 | 0 | 0.000000 | -0.000000 | 1437 | 1196.912260 | 1281.51 | 1366.107740 | 0.891795 |  |
| GO:0032104\_regulation\_of\_response\_to\_extracellular\_stimulus | 7 | 0 | 0.000000 | -0.000000 | 1437 | 1196.912260 | 1281.51 | 1366.107740 | 0.891795 |  |
| GO:0032107\_regulation\_of\_response\_to\_nutrient\_levels | 7 | 0 | 0.000000 | -0.000000 | 1437 | 1196.912260 | 1281.51 | 1366.107740 | 0.891795 |  |
| GO:0032228\_regulation\_of\_synaptic\_transmission\_\_GABAergic | 7 | 0 | 0.000000 | -0.000000 | 1437 | 1196.912260 | 1281.51 | 1366.107740 | 0.891795 |  |
| GO:0032319\_regulation\_of\_Rho\_GTPase\_activity | 7 | 0 | 0.000000 | -0.000000 | 1437 | 1196.912260 | 1281.51 | 1366.107740 | 0.891795 |  |
| GO:0032387\_negative\_regulation\_of\_intracellular\_transport | 7 | 0 | 0.000000 | -0.000000 | 1437 | 1196.912260 | 1281.51 | 1366.107740 | 0.891795 |  |
| GO:0032507\_maintenance\_of\_protein\_location\_in\_cell | 7 | 0 | 0.000000 | -0.000000 | 1437 | 1196.912260 | 1281.51 | 1366.107740 | 0.891795 |  |
| GO:0033032\_regulation\_of\_myeloid\_cell\_apoptosis | 7 | 0 | 0.000000 | -0.000000 | 1437 | 1196.912260 | 1281.51 | 1366.107740 | 0.891795 |  |
| GO:0033057\_reproductive\_behavior\_in\_a\_multicellular\_organism | 7 | 0 | 0.000000 | -0.000000 | 1437 | 1196.912260 | 1281.51 | 1366.107740 | 0.891795 |  |
| GO:0034599\_cellular\_response\_to\_oxidative\_stress | 7 | 0 | 0.000000 | -0.000000 | 1437 | 1196.912260 | 1281.51 | 1366.107740 | 0.891795 |  |
| GO:0042033\_chemokine\_biosynthetic\_process | 7 | 0 | 0.000000 | -0.000000 | 1437 | 1196.912260 | 1281.51 | 1366.107740 | 0.891795 |  |
| GO:0042133\_neurotransmitter\_metabolic\_process | 7 | 0 | 0.000000 | -0.000000 | 1437 | 1196.912260 | 1281.51 | 1366.107740 | 0.891795 |  |
| GO:0042168\_heme\_metabolic\_process | 7 | 0 | 0.000000 | -0.000000 | 1437 | 1196.912260 | 1281.51 | 1366.107740 | 0.891795 |  |
| GO:0042415\_norepinephrine\_metabolic\_process | 7 | 0 | 0.000000 | -0.000000 | 1437 | 1196.912260 | 1281.51 | 1366.107740 | 0.891795 |  |
| GO:0042438\_melanin\_biosynthetic\_process | 7 | 0 | 0.000000 | -0.000000 | 1437 | 1196.912260 | 1281.51 | 1366.107740 | 0.891795 |  |
| GO:0042503\_tyrosine\_phosphorylation\_of\_Stat3\_protein | 7 | 0 | 0.000000 | -0.000000 | 1437 | 1196.912260 | 1281.51 | 1366.107740 | 0.891795 |  |
| GO:0043353\_enucleate\_erythrocyte\_differentiation | 7 | 0 | 0.000000 | -0.000000 | 1437 | 1196.912260 | 1281.51 | 1366.107740 | 0.891795 |  |
| GO:0043372\_positive\_regulation\_of\_CD4-positive\_\_alpha\_beta\_T\_cell\_differentiation | 7 | 0 | 0.000000 | -0.000000 | 1437 | 1196.912260 | 1281.51 | 1366.107740 | 0.891795 |  |
| GO:0043449\_cellular\_alkene\_metabolic\_process | 7 | 0 | 0.000000 | -0.000000 | 1437 | 1196.912260 | 1281.51 | 1366.107740 | 0.891795 |  |
| GO:0043567\_regulation\_of\_insulin-like\_growth\_factor\_receptor\_signaling\_pathway | 7 | 0 | 0.000000 | -0.000000 | 1437 | 1196.912260 | 1281.51 | 1366.107740 | 0.891795 |  |
| GO:0043584\_nose\_development | 7 | 0 | 0.000000 | -0.000000 | 1437 | 1196.912260 | 1281.51 | 1366.107740 | 0.891795 |  |
| GO:0044065\_regulation\_of\_respiratory\_system\_process | 7 | 0 | 0.000000 | -0.000000 | 1437 | 1196.912260 | 1281.51 | 1366.107740 | 0.891795 |  |
| GO:0044275\_cellular\_carbohydrate\_catabolic\_process | 7 | 0 | 0.000000 | -0.000000 | 1437 | 1196.912260 | 1281.51 | 1366.107740 | 0.891795 |  |
| GO:0045059\_positive\_thymic\_T\_cell\_selection | 7 | 0 | 0.000000 | -0.000000 | 1437 | 1196.912260 | 1281.51 | 1366.107740 | 0.891795 |  |
| GO:0045073\_regulation\_of\_chemokine\_biosynthetic\_process | 7 | 0 | 0.000000 | -0.000000 | 1437 | 1196.912260 | 1281.51 | 1366.107740 | 0.891795 |  |
| GO:0045581\_negative\_regulation\_of\_T\_cell\_differentiation | 7 | 0 | 0.000000 | -0.000000 | 1437 | 1196.912260 | 1281.51 | 1366.107740 | 0.891795 |  |
| GO:0045599\_negative\_regulation\_of\_fat\_cell\_differentiation | 7 | 0 | 0.000000 | -0.000000 | 1437 | 1196.912260 | 1281.51 | 1366.107740 | 0.891795 |  |
| GO:0045604\_regulation\_of\_epidermal\_cell\_differentiation | 7 | 0 | 0.000000 | -0.000000 | 1437 | 1196.912260 | 1281.51 | 1366.107740 | 0.891795 |  |
| GO:0045668\_negative\_regulation\_of\_osteoblast\_differentiation | 7 | 0 | 0.000000 | -0.000000 | 1437 | 1196.912260 | 1281.51 | 1366.107740 | 0.891795 |  |
| GO:0045823\_positive\_regulation\_of\_heart\_contraction | 7 | 0 | 0.000000 | -0.000000 | 1437 | 1196.912260 | 1281.51 | 1366.107740 | 0.891795 |  |
| GO:0045840\_positive\_regulation\_of\_mitosis | 7 | 0 | 0.000000 | -0.000000 | 1437 | 1196.912260 | 1281.51 | 1366.107740 | 0.891795 |  |
| GO:0045862\_positive\_regulation\_of\_proteolysis | 7 | 0 | 0.000000 | -0.000000 | 1437 | 1196.912260 | 1281.51 | 1366.107740 | 0.891795 |  |
| GO:0045879\_negative\_regulation\_of\_smoothened\_signaling\_pathway | 7 | 0 | 0.000000 | -0.000000 | 1437 | 1196.912260 | 1281.51 | 1366.107740 | 0.891795 |  |
| GO:0045986\_negative\_regulation\_of\_smooth\_muscle\_contraction | 7 | 0 | 0.000000 | -0.000000 | 1437 | 1196.912260 | 1281.51 | 1366.107740 | 0.891795 |  |
| GO:0046496\_nicotinamide\_nucleotide\_metabolic\_process | 7 | 0 | 0.000000 | -0.000000 | 1437 | 1196.912260 | 1281.51 | 1366.107740 | 0.891795 |  |
| GO:0046504\_glycerol\_ether\_biosynthetic\_process | 7 | 0 | 0.000000 | -0.000000 | 1437 | 1196.912260 | 1281.51 | 1366.107740 | 0.891795 |  |
| GO:0046513\_ceramide\_biosynthetic\_process | 7 | 0 | 0.000000 | -0.000000 | 1437 | 1196.912260 | 1281.51 | 1366.107740 | 0.891795 |  |
| GO:0046520\_sphingoid\_biosynthetic\_process | 7 | 0 | 0.000000 | -0.000000 | 1437 | 1196.912260 | 1281.51 | 1366.107740 | 0.891795 |  |
| GO:0046626\_regulation\_of\_insulin\_receptor\_signaling\_pathway | 7 | 0 | 0.000000 | -0.000000 | 1437 | 1196.912260 | 1281.51 | 1366.107740 | 0.891795 |  |
| GO:0046676\_negative\_regulation\_of\_insulin\_secretion | 7 | 0 | 0.000000 | -0.000000 | 1437 | 1196.912260 | 1281.51 | 1366.107740 | 0.891795 |  |
| GO:0046677\_response\_to\_antibiotic | 7 | 0 | 0.000000 | -0.000000 | 1437 | 1196.912260 | 1281.51 | 1366.107740 | 0.891795 |  |
| GO:0046823\_negative\_regulation\_of\_nucleocytoplasmic\_transport | 7 | 0 | 0.000000 | -0.000000 | 1437 | 1196.912260 | 1281.51 | 1366.107740 | 0.891795 |  |
| GO:0046824\_positive\_regulation\_of\_nucleocytoplasmic\_transport | 7 | 0 | 0.000000 | -0.000000 | 1437 | 1196.912260 | 1281.51 | 1366.107740 | 0.891795 |  |
| GO:0046847\_filopodium\_assembly | 7 | 0 | 0.000000 | -0.000000 | 1437 | 1196.912260 | 1281.51 | 1366.107740 | 0.891795 |  |
| GO:0048148\_behavioral\_response\_to\_cocaine | 7 | 0 | 0.000000 | -0.000000 | 1437 | 1196.912260 | 1281.51 | 1366.107740 | 0.891795 |  |
| GO:0048304\_positive\_regulation\_of\_isotype\_switching\_to\_IgG\_isotypes | 7 | 0 | 0.000000 | -0.000000 | 1437 | 1196.912260 | 1281.51 | 1366.107740 | 0.891795 |  |
| GO:0048486\_parasympathetic\_nervous\_system\_development | 7 | 0 | 0.000000 | -0.000000 | 1437 | 1196.912260 | 1281.51 | 1366.107740 | 0.891795 |  |
| GO:0048537\_mucosal-associated\_lymphoid\_tissue\_development | 7 | 0 | 0.000000 | -0.000000 | 1437 | 1196.912260 | 1281.51 | 1366.107740 | 0.891795 |  |
| GO:0048753\_pigment\_granule\_organization | 7 | 0 | 0.000000 | -0.000000 | 1437 | 1196.912260 | 1281.51 | 1366.107740 | 0.891795 |  |
| GO:0048814\_regulation\_of\_dendrite\_morphogenesis | 7 | 0 | 0.000000 | -0.000000 | 1437 | 1196.912260 | 1281.51 | 1366.107740 | 0.891795 |  |
| GO:0048857\_neural\_nucleus\_development | 7 | 0 | 0.000000 | -0.000000 | 1437 | 1196.912260 | 1281.51 | 1366.107740 | 0.891795 |  |
| GO:0050755\_chemokine\_metabolic\_process | 7 | 0 | 0.000000 | -0.000000 | 1437 | 1196.912260 | 1281.51 | 1366.107740 | 0.891795 |  |
| GO:0050773\_regulation\_of\_dendrite\_development | 7 | 0 | 0.000000 | -0.000000 | 1437 | 1196.912260 | 1281.51 | 1366.107740 | 0.891795 |  |
| GO:0051028\_mRNA\_transport | 7 | 0 | 0.000000 | -0.000000 | 1437 | 1196.912260 | 1281.51 | 1366.107740 | 0.891795 |  |
| GO:0051785\_positive\_regulation\_of\_nuclear\_division | 7 | 0 | 0.000000 | -0.000000 | 1437 | 1196.912260 | 1281.51 | 1366.107740 | 0.891795 |  |
| GO:0051928\_positive\_regulation\_of\_calcium\_ion\_transport | 7 | 0 | 0.000000 | -0.000000 | 1437 | 1196.912260 | 1281.51 | 1366.107740 | 0.891795 |  |
| GO:0055069\_zinc\_ion\_homeostasis | 7 | 0 | 0.000000 | -0.000000 | 1437 | 1196.912260 | 1281.51 | 1366.107740 | 0.891795 |  |
| GO:0055070\_copper\_ion\_homeostasis | 7 | 0 | 0.000000 | -0.000000 | 1437 | 1196.912260 | 1281.51 | 1366.107740 | 0.891795 |  |
| GO:0060037\_pharyngeal\_system\_development | 7 | 0 | 0.000000 | -0.000000 | 1437 | 1196.912260 | 1281.51 | 1366.107740 | 0.891795 |  |
| GO:0060080\_regulation\_of\_inhibitory\_postsynaptic\_membrane\_potential | 7 | 0 | 0.000000 | -0.000000 | 1437 | 1196.912260 | 1281.51 | 1366.107740 | 0.891795 |  |
| GO:0060088\_auditory\_receptor\_cell\_stereocilium\_organization | 7 | 0 | 0.000000 | -0.000000 | 1437 | 1196.912260 | 1281.51 | 1366.107740 | 0.891795 |  |
| GO:0060117\_auditory\_receptor\_cell\_development | 7 | 0 | 0.000000 | -0.000000 | 1437 | 1196.912260 | 1281.51 | 1366.107740 | 0.891795 |  |
| GO:0060441\_branching\_involved\_in\_lung\_morphogenesis | 7 | 0 | 0.000000 | -0.000000 | 1437 | 1196.912260 | 1281.51 | 1366.107740 | 0.891795 |  |
| GO:0060526\_prostate\_glandular\_acinus\_morphogenesis | 7 | 0 | 0.000000 | -0.000000 | 1437 | 1196.912260 | 1281.51 | 1366.107740 | 0.891795 |  |
| GO:0060527\_prostate\_epithelial\_cord\_arborization\_involved\_in\_prostate\_glandular\_acinus\_morphogenesis | 7 | 0 | 0.000000 | -0.000000 | 1437 | 1196.912260 | 1281.51 | 1366.107740 | 0.891795 |  |
| GO:0060579\_ventral\_spinal\_cord\_interneuron\_fate\_commitment | 7 | 0 | 0.000000 | -0.000000 | 1437 | 1196.912260 | 1281.51 | 1366.107740 | 0.891795 |  |
| GO:0060664\_epithelial\_cell\_proliferation\_involved\_in\_salivary\_gland\_morphogenesis | 7 | 0 | 0.000000 | -0.000000 | 1437 | 1196.912260 | 1281.51 | 1366.107740 | 0.891795 |  |
| GO:0060687\_regulation\_of\_branching\_involved\_in\_prostate\_gland\_morphogenesis | 7 | 0 | 0.000000 | -0.000000 | 1437 | 1196.912260 | 1281.51 | 1366.107740 | 0.891795 |  |
| GO:0060770\_negative\_regulation\_of\_epithelial\_cell\_proliferation\_involved\_in\_prostate\_gland\_development | 7 | 0 | 0.000000 | -0.000000 | 1437 | 1196.912260 | 1281.51 | 1366.107740 | 0.891795 |  |
| GO:0070228\_regulation\_of\_lymphocyte\_apoptosis | 7 | 0 | 0.000000 | -0.000000 | 1437 | 1196.912260 | 1281.51 | 1366.107740 | 0.891795 |  |
| GO:0070646\_protein\_modification\_by\_small\_protein\_removal | 7 | 0 | 0.000000 | -0.000000 | 1437 | 1196.912260 | 1281.51 | 1366.107740 | 0.891795 |  |
| GO:0003015\_heart\_process | 49 | 0 | 0.000000 | -0.000000 | 1443 | 1204.764149 | 1288.19 | 1371.615851 | 0.892717 |  |
| GO:0007606\_sensory\_perception\_of\_chemical\_stimulus | 49 | 0 | 0.000000 | -0.000000 | 1443 | 1204.764149 | 1288.19 | 1371.615851 | 0.892717 |  |
| GO:0042035\_regulation\_of\_cytokine\_biosynthetic\_process | 49 | 0 | 0.000000 | -0.000000 | 1443 | 1204.764149 | 1288.19 | 1371.615851 | 0.892717 |  |
| GO:0043473\_pigmentation | 49 | 0 | 0.000000 | -0.000000 | 1443 | 1204.764149 | 1288.19 | 1371.615851 | 0.892717 |  |
| GO:0048741\_skeletal\_muscle\_fiber\_development | 49 | 0 | 0.000000 | -0.000000 | 1443 | 1204.764149 | 1288.19 | 1371.615851 | 0.892717 |  |
| GO:0060047\_heart\_contraction | 49 | 0 | 0.000000 | -0.000000 | 1443 | 1204.764149 | 1288.19 | 1371.615851 | 0.892717 |  |
| GO:0000002\_mitochondrial\_genome\_maintenance | 9 | 0 | 0.000000 | -0.000000 | 1541 | 1315.438496 | 1396.33 | 1477.221504 | 0.906119 |  |
| GO:0000186\_activation\_of\_MAPKK\_activity | 9 | 0 | 0.000000 | -0.000000 | 1541 | 1315.438496 | 1396.33 | 1477.221504 | 0.906119 |  |
| GO:0001539\_ciliary\_or\_flagellar\_motility | 9 | 0 | 0.000000 | -0.000000 | 1541 | 1315.438496 | 1396.33 | 1477.221504 | 0.906119 |  |
| GO:0001542\_ovulation\_from\_ovarian\_follicle | 9 | 0 | 0.000000 | -0.000000 | 1541 | 1315.438496 | 1396.33 | 1477.221504 | 0.906119 |  |
| GO:0001676\_long-chain\_fatty\_acid\_metabolic\_process | 9 | 0 | 0.000000 | -0.000000 | 1541 | 1315.438496 | 1396.33 | 1477.221504 | 0.906119 |  |
| GO:0001935\_endothelial\_cell\_proliferation | 9 | 0 | 0.000000 | -0.000000 | 1541 | 1315.438496 | 1396.33 | 1477.221504 | 0.906119 |  |
| GO:0002028\_regulation\_of\_sodium\_ion\_transport | 9 | 0 | 0.000000 | -0.000000 | 1541 | 1315.438496 | 1396.33 | 1477.221504 | 0.906119 |  |
| GO:0002221\_pattern\_recognition\_receptor\_signaling\_pathway | 9 | 0 | 0.000000 | -0.000000 | 1541 | 1315.438496 | 1396.33 | 1477.221504 | 0.906119 |  |
| GO:0002292\_T\_cell\_differentiation\_during\_immune\_response | 9 | 0 | 0.000000 | -0.000000 | 1541 | 1315.438496 | 1396.33 | 1477.221504 | 0.906119 |  |
| GO:0002293\_alpha-beta\_T\_cell\_differentiation\_during\_immune\_response | 9 | 0 | 0.000000 | -0.000000 | 1541 | 1315.438496 | 1396.33 | 1477.221504 | 0.906119 |  |
| GO:0002294\_CD4-positive\_\_alpha-beta\_T\_cell\_differentiation\_during\_immune\_response | 9 | 0 | 0.000000 | -0.000000 | 1541 | 1315.438496 | 1396.33 | 1477.221504 | 0.906119 |  |
| GO:0002507\_tolerance\_induction | 9 | 0 | 0.000000 | -0.000000 | 1541 | 1315.438496 | 1396.33 | 1477.221504 | 0.906119 |  |
| GO:0002886\_regulation\_of\_myeloid\_leukocyte\_mediated\_immunity | 9 | 0 | 0.000000 | -0.000000 | 1541 | 1315.438496 | 1396.33 | 1477.221504 | 0.906119 |  |
| GO:0006007\_glucose\_catabolic\_process | 9 | 0 | 0.000000 | -0.000000 | 1541 | 1315.438496 | 1396.33 | 1477.221504 | 0.906119 |  |
| GO:0006182\_cGMP\_biosynthetic\_process | 9 | 0 | 0.000000 | -0.000000 | 1541 | 1315.438496 | 1396.33 | 1477.221504 | 0.906119 |  |
| GO:0006309\_DNA\_fragmentation\_involved\_in\_apoptosis | 9 | 0 | 0.000000 | -0.000000 | 1541 | 1315.438496 | 1396.33 | 1477.221504 | 0.906119 |  |
| GO:0006364\_rRNA\_processing | 9 | 0 | 0.000000 | -0.000000 | 1541 | 1315.438496 | 1396.33 | 1477.221504 | 0.906119 |  |
| GO:0006476\_protein\_amino\_acid\_deacetylation | 9 | 0 | 0.000000 | -0.000000 | 1541 | 1315.438496 | 1396.33 | 1477.221504 | 0.906119 |  |
| GO:0006595\_polyamine\_metabolic\_process | 9 | 0 | 0.000000 | -0.000000 | 1541 | 1315.438496 | 1396.33 | 1477.221504 | 0.906119 |  |
| GO:0006611\_protein\_export\_from\_nucleus | 9 | 0 | 0.000000 | -0.000000 | 1541 | 1315.438496 | 1396.33 | 1477.221504 | 0.906119 |  |
| GO:0006910\_phagocytosis\_\_recognition | 9 | 0 | 0.000000 | -0.000000 | 1541 | 1315.438496 | 1396.33 | 1477.221504 | 0.906119 |  |
| GO:0006911\_phagocytosis\_\_engulfment | 9 | 0 | 0.000000 | -0.000000 | 1541 | 1315.438496 | 1396.33 | 1477.221504 | 0.906119 |  |
| GO:0007128\_meiotic\_prophase\_I | 9 | 0 | 0.000000 | -0.000000 | 1541 | 1315.438496 | 1396.33 | 1477.221504 | 0.906119 |  |
| GO:0007193\_inhibition\_of\_adenylate\_cyclase\_activity\_by\_G-protein\_signaling | 9 | 0 | 0.000000 | -0.000000 | 1541 | 1315.438496 | 1396.33 | 1477.221504 | 0.906119 |  |
| GO:0007617\_mating\_behavior | 9 | 0 | 0.000000 | -0.000000 | 1541 | 1315.438496 | 1396.33 | 1477.221504 | 0.906119 |  |
| GO:0009451\_RNA\_modification | 9 | 0 | 0.000000 | -0.000000 | 1541 | 1315.438496 | 1396.33 | 1477.221504 | 0.906119 |  |
| GO:0010675\_regulation\_of\_cellular\_carbohydrate\_metabolic\_process | 9 | 0 | 0.000000 | -0.000000 | 1541 | 1315.438496 | 1396.33 | 1477.221504 | 0.906119 |  |
| GO:0014037\_Schwann\_cell\_differentiation | 9 | 0 | 0.000000 | -0.000000 | 1541 | 1315.438496 | 1396.33 | 1477.221504 | 0.906119 |  |
| GO:0014073\_response\_to\_tropane | 9 | 0 | 0.000000 | -0.000000 | 1541 | 1315.438496 | 1396.33 | 1477.221504 | 0.906119 |  |
| GO:0015695\_organic\_cation\_transport | 9 | 0 | 0.000000 | -0.000000 | 1541 | 1315.438496 | 1396.33 | 1477.221504 | 0.906119 |  |
| GO:0016072\_rRNA\_metabolic\_process | 9 | 0 | 0.000000 | -0.000000 | 1541 | 1315.438496 | 1396.33 | 1477.221504 | 0.906119 |  |
| GO:0016601\_Rac\_protein\_signal\_transduction | 9 | 0 | 0.000000 | -0.000000 | 1541 | 1315.438496 | 1396.33 | 1477.221504 | 0.906119 |  |
| GO:0019320\_hexose\_catabolic\_process | 9 | 0 | 0.000000 | -0.000000 | 1541 | 1315.438496 | 1396.33 | 1477.221504 | 0.906119 |  |
| GO:0021544\_subpallium\_development | 9 | 0 | 0.000000 | -0.000000 | 1541 | 1315.438496 | 1396.33 | 1477.221504 | 0.906119 |  |
| GO:0021936\_regulation\_of\_granule\_cell\_precursor\_proliferation | 9 | 0 | 0.000000 | -0.000000 | 1541 | 1315.438496 | 1396.33 | 1477.221504 | 0.906119 |  |
| GO:0021940\_positive\_regulation\_of\_granule\_cell\_precursor\_proliferation | 9 | 0 | 0.000000 | -0.000000 | 1541 | 1315.438496 | 1396.33 | 1477.221504 | 0.906119 |  |
| GO:0030048\_actin\_filament-based\_movement | 9 | 0 | 0.000000 | -0.000000 | 1541 | 1315.438496 | 1396.33 | 1477.221504 | 0.906119 |  |
| GO:0030279\_negative\_regulation\_of\_ossification | 9 | 0 | 0.000000 | -0.000000 | 1541 | 1315.438496 | 1396.33 | 1477.221504 | 0.906119 |  |
| GO:0030728\_ovulation | 9 | 0 | 0.000000 | -0.000000 | 1541 | 1315.438496 | 1396.33 | 1477.221504 | 0.906119 |  |
| GO:0031023\_microtubule\_organizing\_center\_organization | 9 | 0 | 0.000000 | -0.000000 | 1541 | 1315.438496 | 1396.33 | 1477.221504 | 0.906119 |  |
| GO:0032388\_positive\_regulation\_of\_intracellular\_transport | 9 | 0 | 0.000000 | -0.000000 | 1541 | 1315.438496 | 1396.33 | 1477.221504 | 0.906119 |  |
| GO:0032606\_type\_I\_interferon\_production | 9 | 0 | 0.000000 | -0.000000 | 1541 | 1315.438496 | 1396.33 | 1477.221504 | 0.906119 |  |
| GO:0032814\_regulation\_of\_natural\_killer\_cell\_activation | 9 | 0 | 0.000000 | -0.000000 | 1541 | 1315.438496 | 1396.33 | 1477.221504 | 0.906119 |  |
| GO:0032816\_positive\_regulation\_of\_natural\_killer\_cell\_activation | 9 | 0 | 0.000000 | -0.000000 | 1541 | 1315.438496 | 1396.33 | 1477.221504 | 0.906119 |  |
| GO:0032963\_collagen\_metabolic\_process | 9 | 0 | 0.000000 | -0.000000 | 1541 | 1315.438496 | 1396.33 | 1477.221504 | 0.906119 |  |
| GO:0033028\_myeloid\_cell\_apoptosis | 9 | 0 | 0.000000 | -0.000000 | 1541 | 1315.438496 | 1396.33 | 1477.221504 | 0.906119 |  |
| GO:0033143\_regulation\_of\_steroid\_hormone\_receptor\_signaling\_pathway | 9 | 0 | 0.000000 | -0.000000 | 1541 | 1315.438496 | 1396.33 | 1477.221504 | 0.906119 |  |
| GO:0035088\_establishment\_or\_maintenance\_of\_apical\_basal\_cell\_polarity | 9 | 0 | 0.000000 | -0.000000 | 1541 | 1315.438496 | 1396.33 | 1477.221504 | 0.906119 |  |
| GO:0035162\_embryonic\_hemopoiesis | 9 | 0 | 0.000000 | -0.000000 | 1541 | 1315.438496 | 1396.33 | 1477.221504 | 0.906119 |  |
| GO:0040020\_regulation\_of\_meiosis | 9 | 0 | 0.000000 | -0.000000 | 1541 | 1315.438496 | 1396.33 | 1477.221504 | 0.906119 |  |
| GO:0042058\_regulation\_of\_epidermal\_growth\_factor\_receptor\_signaling\_pathway | 9 | 0 | 0.000000 | -0.000000 | 1541 | 1315.438496 | 1396.33 | 1477.221504 | 0.906119 |  |
| GO:0042093\_T-helper\_cell\_differentiation | 9 | 0 | 0.000000 | -0.000000 | 1541 | 1315.438496 | 1396.33 | 1477.221504 | 0.906119 |  |
| GO:0042220\_response\_to\_cocaine | 9 | 0 | 0.000000 | -0.000000 | 1541 | 1315.438496 | 1396.33 | 1477.221504 | 0.906119 |  |
| GO:0042402\_biogenic\_amine\_catabolic\_process | 9 | 0 | 0.000000 | -0.000000 | 1541 | 1315.438496 | 1396.33 | 1477.221504 | 0.906119 |  |
| GO:0042509\_regulation\_of\_tyrosine\_phosphorylation\_of\_STAT\_protein | 9 | 0 | 0.000000 | -0.000000 | 1541 | 1315.438496 | 1396.33 | 1477.221504 | 0.906119 |  |
| GO:0042640\_anagen | 9 | 0 | 0.000000 | -0.000000 | 1541 | 1315.438496 | 1396.33 | 1477.221504 | 0.906119 |  |
| GO:0043242\_negative\_regulation\_of\_protein\_complex\_disassembly | 9 | 0 | 0.000000 | -0.000000 | 1541 | 1315.438496 | 1396.33 | 1477.221504 | 0.906119 |  |
| GO:0043299\_leukocyte\_degranulation | 9 | 0 | 0.000000 | -0.000000 | 1541 | 1315.438496 | 1396.33 | 1477.221504 | 0.906119 |  |
| GO:0043383\_negative\_T\_cell\_selection | 9 | 0 | 0.000000 | -0.000000 | 1541 | 1315.438496 | 1396.33 | 1477.221504 | 0.906119 |  |
| GO:0043409\_negative\_regulation\_of\_MAPKKK\_cascade | 9 | 0 | 0.000000 | -0.000000 | 1541 | 1315.438496 | 1396.33 | 1477.221504 | 0.906119 |  |
| GO:0043433\_negative\_regulation\_of\_transcription\_factor\_activity | 9 | 0 | 0.000000 | -0.000000 | 1541 | 1315.438496 | 1396.33 | 1477.221504 | 0.906119 |  |
| GO:0043603\_cellular\_amide\_metabolic\_process | 9 | 0 | 0.000000 | -0.000000 | 1541 | 1315.438496 | 1396.33 | 1477.221504 | 0.906119 |  |
| GO:0045060\_negative\_thymic\_T\_cell\_selection | 9 | 0 | 0.000000 | -0.000000 | 1541 | 1315.438496 | 1396.33 | 1477.221504 | 0.906119 |  |
| GO:0045109\_intermediate\_filament\_organization | 9 | 0 | 0.000000 | -0.000000 | 1541 | 1315.438496 | 1396.33 | 1477.221504 | 0.906119 |  |
| GO:0045185\_maintenance\_of\_protein\_location | 9 | 0 | 0.000000 | -0.000000 | 1541 | 1315.438496 | 1396.33 | 1477.221504 | 0.906119 |  |
| GO:0045428\_regulation\_of\_nitric\_oxide\_biosynthetic\_process | 9 | 0 | 0.000000 | -0.000000 | 1541 | 1315.438496 | 1396.33 | 1477.221504 | 0.906119 |  |
| GO:0045620\_negative\_regulation\_of\_lymphocyte\_differentiation | 9 | 0 | 0.000000 | -0.000000 | 1541 | 1315.438496 | 1396.33 | 1477.221504 | 0.906119 |  |
| GO:0045646\_regulation\_of\_erythrocyte\_differentiation | 9 | 0 | 0.000000 | -0.000000 | 1541 | 1315.438496 | 1396.33 | 1477.221504 | 0.906119 |  |
| GO:0045766\_positive\_regulation\_of\_angiogenesis | 9 | 0 | 0.000000 | -0.000000 | 1541 | 1315.438496 | 1396.33 | 1477.221504 | 0.906119 |  |
| GO:0045830\_positive\_regulation\_of\_isotype\_switching | 9 | 0 | 0.000000 | -0.000000 | 1541 | 1315.438496 | 1396.33 | 1477.221504 | 0.906119 |  |
| GO:0045884\_regulation\_of\_survival\_gene\_product\_expression | 9 | 0 | 0.000000 | -0.000000 | 1541 | 1315.438496 | 1396.33 | 1477.221504 | 0.906119 |  |
| GO:0046006\_regulation\_of\_activated\_T\_cell\_proliferation | 9 | 0 | 0.000000 | -0.000000 | 1541 | 1315.438496 | 1396.33 | 1477.221504 | 0.906119 |  |
| GO:0046324\_regulation\_of\_glucose\_import | 9 | 0 | 0.000000 | -0.000000 | 1541 | 1315.438496 | 1396.33 | 1477.221504 | 0.906119 |  |
| GO:0046365\_monosaccharide\_catabolic\_process | 9 | 0 | 0.000000 | -0.000000 | 1541 | 1315.438496 | 1396.33 | 1477.221504 | 0.906119 |  |
| GO:0046636\_negative\_regulation\_of\_alpha-beta\_T\_cell\_activation | 9 | 0 | 0.000000 | -0.000000 | 1541 | 1315.438496 | 1396.33 | 1477.221504 | 0.906119 |  |
| GO:0046641\_positive\_regulation\_of\_alpha-beta\_T\_cell\_proliferation | 9 | 0 | 0.000000 | -0.000000 | 1541 | 1315.438496 | 1396.33 | 1477.221504 | 0.906119 |  |
| GO:0046888\_negative\_regulation\_of\_hormone\_secretion | 9 | 0 | 0.000000 | -0.000000 | 1541 | 1315.438496 | 1396.33 | 1477.221504 | 0.906119 |  |
| GO:0048070\_regulation\_of\_pigmentation\_during\_development | 9 | 0 | 0.000000 | -0.000000 | 1541 | 1315.438496 | 1396.33 | 1477.221504 | 0.906119 |  |
| GO:0048488\_synaptic\_vesicle\_endocytosis | 9 | 0 | 0.000000 | -0.000000 | 1541 | 1315.438496 | 1396.33 | 1477.221504 | 0.906119 |  |
| GO:0048569\_post-embryonic\_organ\_development | 9 | 0 | 0.000000 | -0.000000 | 1541 | 1315.438496 | 1396.33 | 1477.221504 | 0.906119 |  |
| GO:0050433\_regulation\_of\_catecholamine\_secretion | 9 | 0 | 0.000000 | -0.000000 | 1541 | 1315.438496 | 1396.33 | 1477.221504 | 0.906119 |  |
| GO:0050856\_regulation\_of\_T\_cell\_receptor\_signaling\_pathway | 9 | 0 | 0.000000 | -0.000000 | 1541 | 1315.438496 | 1396.33 | 1477.221504 | 0.906119 |  |
| GO:0050884\_neuromuscular\_process\_controlling\_posture | 9 | 0 | 0.000000 | -0.000000 | 1541 | 1315.438496 | 1396.33 | 1477.221504 | 0.906119 |  |
| GO:0050910\_detection\_of\_mechanical\_stimulus\_involved\_in\_sensory\_perception\_of\_sound | 9 | 0 | 0.000000 | -0.000000 | 1541 | 1315.438496 | 1396.33 | 1477.221504 | 0.906119 |  |
| GO:0050918\_positive\_chemotaxis | 9 | 0 | 0.000000 | -0.000000 | 1541 | 1315.438496 | 1396.33 | 1477.221504 | 0.906119 |  |
| GO:0051297\_centrosome\_organization | 9 | 0 | 0.000000 | -0.000000 | 1541 | 1315.438496 | 1396.33 | 1477.221504 | 0.906119 |  |
| GO:0051324\_prophase | 9 | 0 | 0.000000 | -0.000000 | 1541 | 1315.438496 | 1396.33 | 1477.221504 | 0.906119 |  |
| GO:0051607\_defense\_response\_to\_virus | 9 | 0 | 0.000000 | -0.000000 | 1541 | 1315.438496 | 1396.33 | 1477.221504 | 0.906119 |  |
| GO:0051647\_nucleus\_localization | 9 | 0 | 0.000000 | -0.000000 | 1541 | 1315.438496 | 1396.33 | 1477.221504 | 0.906119 |  |
| GO:0051896\_regulation\_of\_protein\_kinase\_B\_signaling\_cascade | 9 | 0 | 0.000000 | -0.000000 | 1541 | 1315.438496 | 1396.33 | 1477.221504 | 0.906119 |  |
| GO:0051932\_synaptic\_transmission\_\_GABAergic | 9 | 0 | 0.000000 | -0.000000 | 1541 | 1315.438496 | 1396.33 | 1477.221504 | 0.906119 |  |
| GO:0051963\_regulation\_of\_synaptogenesis | 9 | 0 | 0.000000 | -0.000000 | 1541 | 1315.438496 | 1396.33 | 1477.221504 | 0.906119 |  |
| GO:0060052\_neurofilament\_cytoskeleton\_organization | 9 | 0 | 0.000000 | -0.000000 | 1541 | 1315.438496 | 1396.33 | 1477.221504 | 0.906119 |  |
| GO:0060081\_membrane\_hyperpolarization | 9 | 0 | 0.000000 | -0.000000 | 1541 | 1315.438496 | 1396.33 | 1477.221504 | 0.906119 |  |
| GO:0060119\_inner\_ear\_receptor\_cell\_development | 9 | 0 | 0.000000 | -0.000000 | 1541 | 1315.438496 | 1396.33 | 1477.221504 | 0.906119 |  |
| GO:0060122\_inner\_ear\_receptor\_stereocilium\_organization | 9 | 0 | 0.000000 | -0.000000 | 1541 | 1315.438496 | 1396.33 | 1477.221504 | 0.906119 |  |
| GO:0060693\_regulation\_of\_branching\_involved\_in\_salivary\_gland\_morphogenesis | 9 | 0 | 0.000000 | -0.000000 | 1541 | 1315.438496 | 1396.33 | 1477.221504 | 0.906119 |  |
| GO:0090048\_negative\_regulation\_of\_transcription\_regulator\_activity | 9 | 0 | 0.000000 | -0.000000 | 1541 | 1315.438496 | 1396.33 | 1477.221504 | 0.906119 |  |
| GO:0009165\_nucleotide\_biosynthetic\_process | 63 | 0 | 0.000000 | -0.000000 | 1545 | 1318.902244 | 1399.16 | 1479.417756 | 0.905605 |  |
| GO:0051186\_cofactor\_metabolic\_process | 63 | 0 | 0.000000 | -0.000000 | 1545 | 1318.902244 | 1399.16 | 1479.417756 | 0.905605 |  |
| GO:0070662\_mast\_cell\_proliferation | 63 | 0 | 0.000000 | -0.000000 | 1545 | 1318.902244 | 1399.16 | 1479.417756 | 0.905605 |  |
| GO:0070666\_regulation\_of\_mast\_cell\_proliferation | 63 | 0 | 0.000000 | -0.000000 | 1545 | 1318.902244 | 1399.16 | 1479.417756 | 0.905605 |  |
| GO:0009190\_cyclic\_nucleotide\_biosynthetic\_process | 50 | 0 | 0.000000 | -0.000000 | 1549 | 1323.938803 | 1403.7 | 1483.461197 | 0.906198 |  |
| GO:0017038\_protein\_import | 50 | 0 | 0.000000 | -0.000000 | 1549 | 1323.938803 | 1403.7 | 1483.461197 | 0.906198 |  |
| GO:0042129\_regulation\_of\_T\_cell\_proliferation | 50 | 0 | 0.000000 | -0.000000 | 1549 | 1323.938803 | 1403.7 | 1483.461197 | 0.906198 |  |
| GO:0070647\_protein\_modification\_by\_small\_protein\_conjugation\_or\_removal | 50 | 0 | 0.000000 | -0.000000 | 1549 | 1323.938803 | 1403.7 | 1483.461197 | 0.906198 |  |
| GO:0016568\_chromatin\_modification | 72 | 0 | 0.000000 | -0.000000 | 1554 | 1329.209429 | 1408.32 | 1487.430571 | 0.906255 |  |
| GO:0040012\_regulation\_of\_locomotion | 72 | 0 | 0.000000 | -0.000000 | 1554 | 1329.209429 | 1408.32 | 1487.430571 | 0.906255 |  |
| GO:0042098\_T\_cell\_proliferation | 72 | 0 | 0.000000 | -0.000000 | 1554 | 1329.209429 | 1408.32 | 1487.430571 | 0.906255 |  |
| GO:0044262\_cellular\_carbohydrate\_metabolic\_process | 72 | 0 | 0.000000 | -0.000000 | 1554 | 1329.209429 | 1408.32 | 1487.430571 | 0.906255 |  |
| GO:0051169\_nuclear\_transport | 72 | 0 | 0.000000 | -0.000000 | 1554 | 1329.209429 | 1408.32 | 1487.430571 | 0.906255 |  |
| GO:0046942\_carboxylic\_acid\_transport | 53 | 0 | 0.000000 | -0.000000 | 1557 | 1334.109250 | 1412.63 | 1491.150750 | 0.907277 |  |
| GO:0050905\_neuromuscular\_process | 53 | 0 | 0.000000 | -0.000000 | 1557 | 1334.109250 | 1412.63 | 1491.150750 | 0.907277 |  |
| GO:0055085\_transmembrane\_transport | 53 | 0 | 0.000000 | -0.000000 | 1557 | 1334.109250 | 1412.63 | 1491.150750 | 0.907277 |  |
| GO:0002429\_immune\_response-activating\_cell\_surface\_receptor\_signaling\_pathway | 41 | 0 | 0.000000 | -0.000000 | 1568 | 1348.036937 | 1425.0 | 1501.963063 | 0.908801 |  |
| GO:0006260\_DNA\_replication | 41 | 0 | 0.000000 | -0.000000 | 1568 | 1348.036937 | 1425.0 | 1501.963063 | 0.908801 |  |
| GO:0006836\_neurotransmitter\_transport | 41 | 0 | 0.000000 | -0.000000 | 1568 | 1348.036937 | 1425.0 | 1501.963063 | 0.908801 |  |
| GO:0006865\_amino\_acid\_transport | 41 | 0 | 0.000000 | -0.000000 | 1568 | 1348.036937 | 1425.0 | 1501.963063 | 0.908801 |  |
| GO:0009894\_regulation\_of\_catabolic\_process | 41 | 0 | 0.000000 | -0.000000 | 1568 | 1348.036937 | 1425.0 | 1501.963063 | 0.908801 |  |
| GO:0015833\_peptide\_transport | 41 | 0 | 0.000000 | -0.000000 | 1568 | 1348.036937 | 1425.0 | 1501.963063 | 0.908801 |  |
| GO:0015980\_energy\_derivation\_by\_oxidation\_of\_organic\_compounds | 41 | 0 | 0.000000 | -0.000000 | 1568 | 1348.036937 | 1425.0 | 1501.963063 | 0.908801 |  |
| GO:0030817\_regulation\_of\_cAMP\_biosynthetic\_process | 41 | 0 | 0.000000 | -0.000000 | 1568 | 1348.036937 | 1425.0 | 1501.963063 | 0.908801 |  |
| GO:0031344\_regulation\_of\_cell\_projection\_organization | 41 | 0 | 0.000000 | -0.000000 | 1568 | 1348.036937 | 1425.0 | 1501.963063 | 0.908801 |  |
| GO:0032844\_regulation\_of\_homeostatic\_process | 41 | 0 | 0.000000 | -0.000000 | 1568 | 1348.036937 | 1425.0 | 1501.963063 | 0.908801 |  |
| GO:0050864\_regulation\_of\_B\_cell\_activation | 41 | 0 | 0.000000 | -0.000000 | 1568 | 1348.036937 | 1425.0 | 1501.963063 | 0.908801 |  |
| GO:0000723\_telomere\_maintenance | 13 | 0 | 0.000000 | -0.000000 | 1626 | 1414.670327 | 1489.34 | 1564.009673 | 0.915953 |  |
| GO:0001836\_release\_of\_cytochrome\_c\_from\_mitochondria | 13 | 0 | 0.000000 | -0.000000 | 1626 | 1414.670327 | 1489.34 | 1564.009673 | 0.915953 |  |
| GO:0001958\_endochondral\_ossification | 13 | 0 | 0.000000 | -0.000000 | 1626 | 1414.670327 | 1489.34 | 1564.009673 | 0.915953 |  |
| GO:0001975\_response\_to\_amphetamine | 13 | 0 | 0.000000 | -0.000000 | 1626 | 1414.670327 | 1489.34 | 1564.009673 | 0.915953 |  |
| GO:0001976\_neurological\_system\_process\_involved\_in\_regulation\_of\_systemic\_arterial\_blood\_pressure | 13 | 0 | 0.000000 | -0.000000 | 1626 | 1414.670327 | 1489.34 | 1564.009673 | 0.915953 |  |
| GO:0002704\_negative\_regulation\_of\_leukocyte\_mediated\_immunity | 13 | 0 | 0.000000 | -0.000000 | 1626 | 1414.670327 | 1489.34 | 1564.009673 | 0.915953 |  |
| GO:0002707\_negative\_regulation\_of\_lymphocyte\_mediated\_immunity | 13 | 0 | 0.000000 | -0.000000 | 1626 | 1414.670327 | 1489.34 | 1564.009673 | 0.915953 |  |
| GO:0002717\_positive\_regulation\_of\_natural\_killer\_cell\_mediated\_immunity | 13 | 0 | 0.000000 | -0.000000 | 1626 | 1414.670327 | 1489.34 | 1564.009673 | 0.915953 |  |
| GO:0003016\_respiratory\_system\_process | 13 | 0 | 0.000000 | -0.000000 | 1626 | 1414.670327 | 1489.34 | 1564.009673 | 0.915953 |  |
| GO:0006687\_glycosphingolipid\_metabolic\_process | 13 | 0 | 0.000000 | -0.000000 | 1626 | 1414.670327 | 1489.34 | 1564.009673 | 0.915953 |  |
| GO:0006778\_porphyrin\_metabolic\_process | 13 | 0 | 0.000000 | -0.000000 | 1626 | 1414.670327 | 1489.34 | 1564.009673 | 0.915953 |  |
| GO:0006833\_water\_transport | 13 | 0 | 0.000000 | -0.000000 | 1626 | 1414.670327 | 1489.34 | 1564.009673 | 0.915953 |  |
| GO:0006898\_receptor-mediated\_endocytosis | 13 | 0 | 0.000000 | -0.000000 | 1626 | 1414.670327 | 1489.34 | 1564.009673 | 0.915953 |  |
| GO:0006986\_response\_to\_unfolded\_protein | 13 | 0 | 0.000000 | -0.000000 | 1626 | 1414.670327 | 1489.34 | 1564.009673 | 0.915953 |  |
| GO:0007129\_synapsis | 13 | 0 | 0.000000 | -0.000000 | 1626 | 1414.670327 | 1489.34 | 1564.009673 | 0.915953 |  |
| GO:0007212\_dopamine\_receptor\_signaling\_pathway | 13 | 0 | 0.000000 | -0.000000 | 1626 | 1414.670327 | 1489.34 | 1564.009673 | 0.915953 |  |
| GO:0007274\_neuromuscular\_synaptic\_transmission | 13 | 0 | 0.000000 | -0.000000 | 1626 | 1414.670327 | 1489.34 | 1564.009673 | 0.915953 |  |
| GO:0007339\_binding\_of\_sperm\_to\_zona\_pellucida | 13 | 0 | 0.000000 | -0.000000 | 1626 | 1414.670327 | 1489.34 | 1564.009673 | 0.915953 |  |
| GO:0007512\_adult\_heart\_development | 13 | 0 | 0.000000 | -0.000000 | 1626 | 1414.670327 | 1489.34 | 1564.009673 | 0.915953 |  |
| GO:0007566\_embryo\_implantation | 13 | 0 | 0.000000 | -0.000000 | 1626 | 1414.670327 | 1489.34 | 1564.009673 | 0.915953 |  |
| GO:0009119\_ribonucleoside\_metabolic\_process | 13 | 0 | 0.000000 | -0.000000 | 1626 | 1414.670327 | 1489.34 | 1564.009673 | 0.915953 |  |
| GO:0009410\_response\_to\_xenobiotic\_stimulus | 13 | 0 | 0.000000 | -0.000000 | 1626 | 1414.670327 | 1489.34 | 1564.009673 | 0.915953 |  |
| GO:0009994\_oocyte\_differentiation | 13 | 0 | 0.000000 | -0.000000 | 1626 | 1414.670327 | 1489.34 | 1564.009673 | 0.915953 |  |
| GO:0010623\_developmental\_programmed\_cell\_death | 13 | 0 | 0.000000 | -0.000000 | 1626 | 1414.670327 | 1489.34 | 1564.009673 | 0.915953 |  |
| GO:0010970\_microtubule-based\_transport | 13 | 0 | 0.000000 | -0.000000 | 1626 | 1414.670327 | 1489.34 | 1564.009673 | 0.915953 |  |
| GO:0016525\_negative\_regulation\_of\_angiogenesis | 13 | 0 | 0.000000 | -0.000000 | 1626 | 1414.670327 | 1489.34 | 1564.009673 | 0.915953 |  |
| GO:0018105\_peptidyl-serine\_phosphorylation | 13 | 0 | 0.000000 | -0.000000 | 1626 | 1414.670327 | 1489.34 | 1564.009673 | 0.915953 |  |
| GO:0019098\_reproductive\_behavior | 13 | 0 | 0.000000 | -0.000000 | 1626 | 1414.670327 | 1489.34 | 1564.009673 | 0.915953 |  |
| GO:0021511\_spinal\_cord\_patterning | 13 | 0 | 0.000000 | -0.000000 | 1626 | 1414.670327 | 1489.34 | 1564.009673 | 0.915953 |  |
| GO:0021879\_forebrain\_neuron\_differentiation | 13 | 0 | 0.000000 | -0.000000 | 1626 | 1414.670327 | 1489.34 | 1564.009673 | 0.915953 |  |
| GO:0021955\_central\_nervous\_system\_neuron\_axonogenesis | 13 | 0 | 0.000000 | -0.000000 | 1626 | 1414.670327 | 1489.34 | 1564.009673 | 0.915953 |  |
| GO:0030384\_phosphoinositide\_metabolic\_process | 13 | 0 | 0.000000 | -0.000000 | 1626 | 1414.670327 | 1489.34 | 1564.009673 | 0.915953 |  |
| GO:0030516\_regulation\_of\_axon\_extension | 13 | 0 | 0.000000 | -0.000000 | 1626 | 1414.670327 | 1489.34 | 1564.009673 | 0.915953 |  |
| GO:0030539\_male\_genitalia\_development | 13 | 0 | 0.000000 | -0.000000 | 1626 | 1414.670327 | 1489.34 | 1564.009673 | 0.915953 |  |
| GO:0031290\_retinal\_ganglion\_cell\_axon\_guidance | 13 | 0 | 0.000000 | -0.000000 | 1626 | 1414.670327 | 1489.34 | 1564.009673 | 0.915953 |  |
| GO:0032200\_telomere\_organization | 13 | 0 | 0.000000 | -0.000000 | 1626 | 1414.670327 | 1489.34 | 1564.009673 | 0.915953 |  |
| GO:0032615\_interleukin-12\_production | 13 | 0 | 0.000000 | -0.000000 | 1626 | 1414.670327 | 1489.34 | 1564.009673 | 0.915953 |  |
| GO:0032729\_positive\_regulation\_of\_interferon-gamma\_production | 13 | 0 | 0.000000 | -0.000000 | 1626 | 1414.670327 | 1489.34 | 1564.009673 | 0.915953 |  |
| GO:0033013\_tetrapyrrole\_metabolic\_process | 13 | 0 | 0.000000 | -0.000000 | 1626 | 1414.670327 | 1489.34 | 1564.009673 | 0.915953 |  |
| GO:0034329\_cell\_junction\_assembly | 13 | 0 | 0.000000 | -0.000000 | 1626 | 1414.670327 | 1489.34 | 1564.009673 | 0.915953 |  |
| GO:0042044\_fluid\_transport | 13 | 0 | 0.000000 | -0.000000 | 1626 | 1414.670327 | 1489.34 | 1564.009673 | 0.915953 |  |
| GO:0042094\_interleukin-2\_biosynthetic\_process | 13 | 0 | 0.000000 | -0.000000 | 1626 | 1414.670327 | 1489.34 | 1564.009673 | 0.915953 |  |
| GO:0045191\_regulation\_of\_isotype\_switching | 13 | 0 | 0.000000 | -0.000000 | 1626 | 1414.670327 | 1489.34 | 1564.009673 | 0.915953 |  |
| GO:0045577\_regulation\_of\_B\_cell\_differentiation | 13 | 0 | 0.000000 | -0.000000 | 1626 | 1414.670327 | 1489.34 | 1564.009673 | 0.915953 |  |
| GO:0045682\_regulation\_of\_epidermis\_development | 13 | 0 | 0.000000 | -0.000000 | 1626 | 1414.670327 | 1489.34 | 1564.009673 | 0.915953 |  |
| GO:0045954\_positive\_regulation\_of\_natural\_killer\_cell\_mediated\_cytotoxicity | 13 | 0 | 0.000000 | -0.000000 | 1626 | 1414.670327 | 1489.34 | 1564.009673 | 0.915953 |  |
| GO:0046474\_glycerophospholipid\_biosynthetic\_process | 13 | 0 | 0.000000 | -0.000000 | 1626 | 1414.670327 | 1489.34 | 1564.009673 | 0.915953 |  |
| GO:0046640\_regulation\_of\_alpha-beta\_T\_cell\_proliferation | 13 | 0 | 0.000000 | -0.000000 | 1626 | 1414.670327 | 1489.34 | 1564.009673 | 0.915953 |  |
| GO:0046851\_negative\_regulation\_of\_bone\_remodeling | 13 | 0 | 0.000000 | -0.000000 | 1626 | 1414.670327 | 1489.34 | 1564.009673 | 0.915953 |  |
| GO:0048566\_embryonic\_gut\_development | 13 | 0 | 0.000000 | -0.000000 | 1626 | 1414.670327 | 1489.34 | 1564.009673 | 0.915953 |  |
| GO:0048599\_oocyte\_development | 13 | 0 | 0.000000 | -0.000000 | 1626 | 1414.670327 | 1489.34 | 1564.009673 | 0.915953 |  |
| GO:0050764\_regulation\_of\_phagocytosis | 13 | 0 | 0.000000 | -0.000000 | 1626 | 1414.670327 | 1489.34 | 1564.009673 | 0.915953 |  |
| GO:0050766\_positive\_regulation\_of\_phagocytosis | 13 | 0 | 0.000000 | -0.000000 | 1626 | 1414.670327 | 1489.34 | 1564.009673 | 0.915953 |  |
| GO:0050771\_negative\_regulation\_of\_axonogenesis | 13 | 0 | 0.000000 | -0.000000 | 1626 | 1414.670327 | 1489.34 | 1564.009673 | 0.915953 |  |
| GO:0051346\_negative\_regulation\_of\_hydrolase\_activity | 13 | 0 | 0.000000 | -0.000000 | 1626 | 1414.670327 | 1489.34 | 1564.009673 | 0.915953 |  |
| GO:0060401\_cytosolic\_calcium\_ion\_transport | 13 | 0 | 0.000000 | -0.000000 | 1626 | 1414.670327 | 1489.34 | 1564.009673 | 0.915953 |  |
| GO:0060402\_calcium\_ion\_transport\_into\_cytosol | 13 | 0 | 0.000000 | -0.000000 | 1626 | 1414.670327 | 1489.34 | 1564.009673 | 0.915953 |  |
| GO:0070192\_chromosome\_organization\_involved\_in\_meiosis | 13 | 0 | 0.000000 | -0.000000 | 1626 | 1414.670327 | 1489.34 | 1564.009673 | 0.915953 |  |
| GO:0015674\_di-\_\_tri-valent\_inorganic\_cation\_transport | 79 | 0 | 0.000000 | -0.000000 | 1627 | 1415.645797 | 1490.15 | 1564.654203 | 0.915888 |  |
| GO:0000278\_mitotic\_cell\_cycle | 80 | 0 | 0.000000 | -0.000000 | 1630 | 1417.990135 | 1492.14 | 1566.289865 | 0.915423 |  |
| GO:0006631\_fatty\_acid\_metabolic\_process | 80 | 0 | 0.000000 | -0.000000 | 1630 | 1417.990135 | 1492.14 | 1566.289865 | 0.915423 |  |
| GO:0044092\_negative\_regulation\_of\_molecular\_function | 80 | 0 | 0.000000 | -0.000000 | 1630 | 1417.990135 | 1492.14 | 1566.289865 | 0.915423 |  |
| GO:0000038\_very-long-chain\_fatty\_acid\_metabolic\_process | 6 | 0 | 0.000000 | -0.000000 | 1801 | 1595.218363 | 1666.34 | 1737.461637 | 0.925230 |  |
| GO:0000245\_spliceosome\_assembly | 6 | 0 | 0.000000 | -0.000000 | 1801 | 1595.218363 | 1666.34 | 1737.461637 | 0.925230 |  |
| GO:0000768\_syncytium\_formation\_by\_plasma\_membrane\_fusion | 6 | 0 | 0.000000 | -0.000000 | 1801 | 1595.218363 | 1666.34 | 1737.461637 | 0.925230 |  |
| GO:0001710\_mesodermal\_cell\_fate\_commitment | 6 | 0 | 0.000000 | -0.000000 | 1801 | 1595.218363 | 1666.34 | 1737.461637 | 0.925230 |  |
| GO:0001779\_natural\_killer\_cell\_differentiation | 6 | 0 | 0.000000 | -0.000000 | 1801 | 1595.218363 | 1666.34 | 1737.461637 | 0.925230 |  |
| GO:0001885\_endothelial\_cell\_development | 6 | 0 | 0.000000 | -0.000000 | 1801 | 1595.218363 | 1666.34 | 1737.461637 | 0.925230 |  |
| GO:0002016\_regulation\_of\_blood\_volume\_by\_renin-angiotensin | 6 | 0 | 0.000000 | -0.000000 | 1801 | 1595.218363 | 1666.34 | 1737.461637 | 0.925230 |  |
| GO:0002335\_mature\_B\_cell\_differentiation | 6 | 0 | 0.000000 | -0.000000 | 1801 | 1595.218363 | 1666.34 | 1737.461637 | 0.925230 |  |
| GO:0002360\_T\_cell\_lineage\_commitment | 6 | 0 | 0.000000 | -0.000000 | 1801 | 1595.218363 | 1666.34 | 1737.461637 | 0.925230 |  |
| GO:0002367\_cytokine\_production\_during\_immune\_response | 6 | 0 | 0.000000 | -0.000000 | 1801 | 1595.218363 | 1666.34 | 1737.461637 | 0.925230 |  |
| GO:0002474\_antigen\_processing\_and\_presentation\_of\_peptide\_antigen\_via\_MHC\_class\_I | 6 | 0 | 0.000000 | -0.000000 | 1801 | 1595.218363 | 1666.34 | 1737.461637 | 0.925230 |  |
| GO:0002475\_antigen\_processing\_and\_presentation\_via\_MHC\_class\_Ib | 6 | 0 | 0.000000 | -0.000000 | 1801 | 1595.218363 | 1666.34 | 1737.461637 | 0.925230 |  |
| GO:0002532\_production\_of\_molecular\_mediator\_of\_acute\_inflammatory\_response | 6 | 0 | 0.000000 | -0.000000 | 1801 | 1595.218363 | 1666.34 | 1737.461637 | 0.925230 |  |
| GO:0002541\_activation\_of\_plasma\_proteins\_involved\_in\_acute\_inflammatory\_response | 6 | 0 | 0.000000 | -0.000000 | 1801 | 1595.218363 | 1666.34 | 1737.461637 | 0.925230 |  |
| GO:0002675\_positive\_regulation\_of\_acute\_inflammatory\_response | 6 | 0 | 0.000000 | -0.000000 | 1801 | 1595.218363 | 1666.34 | 1737.461637 | 0.925230 |  |
| GO:0002685\_regulation\_of\_leukocyte\_migration | 6 | 0 | 0.000000 | -0.000000 | 1801 | 1595.218363 | 1666.34 | 1737.461637 | 0.925230 |  |
| GO:0002831\_regulation\_of\_response\_to\_biotic\_stimulus | 6 | 0 | 0.000000 | -0.000000 | 1801 | 1595.218363 | 1666.34 | 1737.461637 | 0.925230 |  |
| GO:0002920\_regulation\_of\_humoral\_immune\_response | 6 | 0 | 0.000000 | -0.000000 | 1801 | 1595.218363 | 1666.34 | 1737.461637 | 0.925230 |  |
| GO:0006071\_glycerol\_metabolic\_process | 6 | 0 | 0.000000 | -0.000000 | 1801 | 1595.218363 | 1666.34 | 1737.461637 | 0.925230 |  |
| GO:0006084\_acetyl-CoA\_metabolic\_process | 6 | 0 | 0.000000 | -0.000000 | 1801 | 1595.218363 | 1666.34 | 1737.461637 | 0.925230 |  |
| GO:0006264\_mitochondrial\_DNA\_replication | 6 | 0 | 0.000000 | -0.000000 | 1801 | 1595.218363 | 1666.34 | 1737.461637 | 0.925230 |  |
| GO:0006402\_mRNA\_catabolic\_process | 6 | 0 | 0.000000 | -0.000000 | 1801 | 1595.218363 | 1666.34 | 1737.461637 | 0.925230 |  |
| GO:0006471\_protein\_amino\_acid\_ADP-ribosylation | 6 | 0 | 0.000000 | -0.000000 | 1801 | 1595.218363 | 1666.34 | 1737.461637 | 0.925230 |  |
| GO:0006536\_glutamate\_metabolic\_process | 6 | 0 | 0.000000 | -0.000000 | 1801 | 1595.218363 | 1666.34 | 1737.461637 | 0.925230 |  |
| GO:0006656\_phosphatidylcholine\_biosynthetic\_process | 6 | 0 | 0.000000 | -0.000000 | 1801 | 1595.218363 | 1666.34 | 1737.461637 | 0.925230 |  |
| GO:0006692\_prostanoid\_metabolic\_process | 6 | 0 | 0.000000 | -0.000000 | 1801 | 1595.218363 | 1666.34 | 1737.461637 | 0.925230 |  |
| GO:0006693\_prostaglandin\_metabolic\_process | 6 | 0 | 0.000000 | -0.000000 | 1801 | 1595.218363 | 1666.34 | 1737.461637 | 0.925230 |  |
| GO:0006752\_group\_transfer\_coenzyme\_metabolic\_process | 6 | 0 | 0.000000 | -0.000000 | 1801 | 1595.218363 | 1666.34 | 1737.461637 | 0.925230 |  |
| GO:0006882\_cellular\_zinc\_ion\_homeostasis | 6 | 0 | 0.000000 | -0.000000 | 1801 | 1595.218363 | 1666.34 | 1737.461637 | 0.925230 |  |
| GO:0006942\_regulation\_of\_striated\_muscle\_contraction | 6 | 0 | 0.000000 | -0.000000 | 1801 | 1595.218363 | 1666.34 | 1737.461637 | 0.925230 |  |
| GO:0006956\_complement\_activation | 6 | 0 | 0.000000 | -0.000000 | 1801 | 1595.218363 | 1666.34 | 1737.461637 | 0.925230 |  |
| GO:0006998\_nuclear\_envelope\_organization | 6 | 0 | 0.000000 | -0.000000 | 1801 | 1595.218363 | 1666.34 | 1737.461637 | 0.925230 |  |
| GO:0007032\_endosome\_organization | 6 | 0 | 0.000000 | -0.000000 | 1801 | 1595.218363 | 1666.34 | 1737.461637 | 0.925230 |  |
| GO:0007176\_regulation\_of\_epidermal\_growth\_factor\_receptor\_activity | 6 | 0 | 0.000000 | -0.000000 | 1801 | 1595.218363 | 1666.34 | 1737.461637 | 0.925230 |  |
| GO:0007214\_gamma-aminobutyric\_acid\_signaling\_pathway | 6 | 0 | 0.000000 | -0.000000 | 1801 | 1595.218363 | 1666.34 | 1737.461637 | 0.925230 |  |
| GO:0007257\_activation\_of\_JUN\_kinase\_activity | 6 | 0 | 0.000000 | -0.000000 | 1801 | 1595.218363 | 1666.34 | 1737.461637 | 0.925230 |  |
| GO:0007341\_penetration\_of\_zona\_pellucida | 6 | 0 | 0.000000 | -0.000000 | 1801 | 1595.218363 | 1666.34 | 1737.461637 | 0.925230 |  |
| GO:0007406\_negative\_regulation\_of\_neuroblast\_proliferation | 6 | 0 | 0.000000 | -0.000000 | 1801 | 1595.218363 | 1666.34 | 1737.461637 | 0.925230 |  |
| GO:0007520\_myoblast\_fusion | 6 | 0 | 0.000000 | -0.000000 | 1801 | 1595.218363 | 1666.34 | 1737.461637 | 0.925230 |  |
| GO:0007620\_copulation | 6 | 0 | 0.000000 | -0.000000 | 1801 | 1595.218363 | 1666.34 | 1737.461637 | 0.925230 |  |
| GO:0008156\_negative\_regulation\_of\_DNA\_replication | 6 | 0 | 0.000000 | -0.000000 | 1801 | 1595.218363 | 1666.34 | 1737.461637 | 0.925230 |  |
| GO:0008209\_androgen\_metabolic\_process | 6 | 0 | 0.000000 | -0.000000 | 1801 | 1595.218363 | 1666.34 | 1737.461637 | 0.925230 |  |
| GO:0008625\_induction\_of\_apoptosis\_via\_death\_domain\_receptors | 6 | 0 | 0.000000 | -0.000000 | 1801 | 1595.218363 | 1666.34 | 1737.461637 | 0.925230 |  |
| GO:0009067\_aspartate\_family\_amino\_acid\_biosynthetic\_process | 6 | 0 | 0.000000 | -0.000000 | 1801 | 1595.218363 | 1666.34 | 1737.461637 | 0.925230 |  |
| GO:0009069\_serine\_family\_amino\_acid\_metabolic\_process | 6 | 0 | 0.000000 | -0.000000 | 1801 | 1595.218363 | 1666.34 | 1737.461637 | 0.925230 |  |
| GO:0009112\_nucleobase\_metabolic\_process | 6 | 0 | 0.000000 | -0.000000 | 1801 | 1595.218363 | 1666.34 | 1737.461637 | 0.925230 |  |
| GO:0009143\_nucleoside\_triphosphate\_catabolic\_process | 6 | 0 | 0.000000 | -0.000000 | 1801 | 1595.218363 | 1666.34 | 1737.461637 | 0.925230 |  |
| GO:0009247\_glycolipid\_biosynthetic\_process | 6 | 0 | 0.000000 | -0.000000 | 1801 | 1595.218363 | 1666.34 | 1737.461637 | 0.925230 |  |
| GO:0009650\_UV\_protection | 6 | 0 | 0.000000 | -0.000000 | 1801 | 1595.218363 | 1666.34 | 1737.461637 | 0.925230 |  |
| GO:0009651\_response\_to\_salt\_stress | 6 | 0 | 0.000000 | -0.000000 | 1801 | 1595.218363 | 1666.34 | 1737.461637 | 0.925230 |  |
| GO:0010466\_negative\_regulation\_of\_peptidase\_activity | 6 | 0 | 0.000000 | -0.000000 | 1801 | 1595.218363 | 1666.34 | 1737.461637 | 0.925230 |  |
| GO:0010883\_regulation\_of\_lipid\_storage | 6 | 0 | 0.000000 | -0.000000 | 1801 | 1595.218363 | 1666.34 | 1737.461637 | 0.925230 |  |
| GO:0010906\_regulation\_of\_glucose\_metabolic\_process | 6 | 0 | 0.000000 | -0.000000 | 1801 | 1595.218363 | 1666.34 | 1737.461637 | 0.925230 |  |
| GO:0014003\_oligodendrocyte\_development | 6 | 0 | 0.000000 | -0.000000 | 1801 | 1595.218363 | 1666.34 | 1737.461637 | 0.925230 |  |
| GO:0014051\_gamma-aminobutyric\_acid\_secretion | 6 | 0 | 0.000000 | -0.000000 | 1801 | 1595.218363 | 1666.34 | 1737.461637 | 0.925230 |  |
| GO:0014072\_response\_to\_isoquinoline\_alkaloid | 6 | 0 | 0.000000 | -0.000000 | 1801 | 1595.218363 | 1666.34 | 1737.461637 | 0.925230 |  |
| GO:0014812\_muscle\_cell\_migration | 6 | 0 | 0.000000 | -0.000000 | 1801 | 1595.218363 | 1666.34 | 1737.461637 | 0.925230 |  |
| GO:0014823\_response\_to\_activity | 6 | 0 | 0.000000 | -0.000000 | 1801 | 1595.218363 | 1666.34 | 1737.461637 | 0.925230 |  |
| GO:0015012\_heparan\_sulfate\_proteoglycan\_biosynthetic\_process | 6 | 0 | 0.000000 | -0.000000 | 1801 | 1595.218363 | 1666.34 | 1737.461637 | 0.925230 |  |
| GO:0015812\_gamma-aminobutyric\_acid\_transport | 6 | 0 | 0.000000 | -0.000000 | 1801 | 1595.218363 | 1666.34 | 1737.461637 | 0.925230 |  |
| GO:0016032\_viral\_reproduction | 6 | 0 | 0.000000 | -0.000000 | 1801 | 1595.218363 | 1666.34 | 1737.461637 | 0.925230 |  |
| GO:0016574\_histone\_ubiquitination | 6 | 0 | 0.000000 | -0.000000 | 1801 | 1595.218363 | 1666.34 | 1737.461637 | 0.925230 |  |
| GO:0016925\_protein\_sumoylation | 6 | 0 | 0.000000 | -0.000000 | 1801 | 1595.218363 | 1666.34 | 1737.461637 | 0.925230 |  |
| GO:0019433\_triglyceride\_catabolic\_process | 6 | 0 | 0.000000 | -0.000000 | 1801 | 1595.218363 | 1666.34 | 1737.461637 | 0.925230 |  |
| GO:0019835\_cytolysis | 6 | 0 | 0.000000 | -0.000000 | 1801 | 1595.218363 | 1666.34 | 1737.461637 | 0.925230 |  |
| GO:0021548\_pons\_development | 6 | 0 | 0.000000 | -0.000000 | 1801 | 1595.218363 | 1666.34 | 1737.461637 | 0.925230 |  |
| GO:0021783\_preganglionic\_parasympathetic\_nervous\_system\_development | 6 | 0 | 0.000000 | -0.000000 | 1801 | 1595.218363 | 1666.34 | 1737.461637 | 0.925230 |  |
| GO:0021892\_cerebral\_cortex\_GABAergic\_interneuron\_differentiation | 6 | 0 | 0.000000 | -0.000000 | 1801 | 1595.218363 | 1666.34 | 1737.461637 | 0.925230 |  |
| GO:0021937\_Purkinje\_cell-granule\_cell\_precursor\_cell\_signaling\_involved\_in\_regulation\_of\_granule\_cell\_precursor\_cell\_proliferation | 6 | 0 | 0.000000 | -0.000000 | 1801 | 1595.218363 | 1666.34 | 1737.461637 | 0.925230 |  |
| GO:0030002\_cellular\_anion\_homeostasis | 6 | 0 | 0.000000 | -0.000000 | 1801 | 1595.218363 | 1666.34 | 1737.461637 | 0.925230 |  |
| GO:0030149\_sphingolipid\_catabolic\_process | 6 | 0 | 0.000000 | -0.000000 | 1801 | 1595.218363 | 1666.34 | 1737.461637 | 0.925230 |  |
| GO:0030252\_growth\_hormone\_secretion | 6 | 0 | 0.000000 | -0.000000 | 1801 | 1595.218363 | 1666.34 | 1737.461637 | 0.925230 |  |
| GO:0030865\_cortical\_cytoskeleton\_organization | 6 | 0 | 0.000000 | -0.000000 | 1801 | 1595.218363 | 1666.34 | 1737.461637 | 0.925230 |  |
| GO:0030947\_regulation\_of\_vascular\_endothelial\_growth\_factor\_receptor\_signaling\_pathway | 6 | 0 | 0.000000 | -0.000000 | 1801 | 1595.218363 | 1666.34 | 1737.461637 | 0.925230 |  |
| GO:0031077\_post-embryonic\_camera-type\_eye\_development | 6 | 0 | 0.000000 | -0.000000 | 1801 | 1595.218363 | 1666.34 | 1737.461637 | 0.925230 |  |
| GO:0031330\_negative\_regulation\_of\_cellular\_catabolic\_process | 6 | 0 | 0.000000 | -0.000000 | 1801 | 1595.218363 | 1666.34 | 1737.461637 | 0.925230 |  |
| GO:0031575\_G1\_S\_transition\_checkpoint | 6 | 0 | 0.000000 | -0.000000 | 1801 | 1595.218363 | 1666.34 | 1737.461637 | 0.925230 |  |
| GO:0031960\_response\_to\_corticosteroid\_stimulus | 6 | 0 | 0.000000 | -0.000000 | 1801 | 1595.218363 | 1666.34 | 1737.461637 | 0.925230 |  |
| GO:0032042\_mitochondrial\_DNA\_metabolic\_process | 6 | 0 | 0.000000 | -0.000000 | 1801 | 1595.218363 | 1666.34 | 1737.461637 | 0.925230 |  |
| GO:0032392\_DNA\_geometric\_change | 6 | 0 | 0.000000 | -0.000000 | 1801 | 1595.218363 | 1666.34 | 1737.461637 | 0.925230 |  |
| GO:0032438\_melanosome\_organization | 6 | 0 | 0.000000 | -0.000000 | 1801 | 1595.218363 | 1666.34 | 1737.461637 | 0.925230 |  |
| GO:0032469\_endoplasmic\_reticulum\_calcium\_ion\_homeostasis | 6 | 0 | 0.000000 | -0.000000 | 1801 | 1595.218363 | 1666.34 | 1737.461637 | 0.925230 |  |
| GO:0032653\_regulation\_of\_interleukin-10\_production | 6 | 0 | 0.000000 | -0.000000 | 1801 | 1595.218363 | 1666.34 | 1737.461637 | 0.925230 |  |
| GO:0033238\_regulation\_of\_cellular\_amine\_metabolic\_process | 6 | 0 | 0.000000 | -0.000000 | 1801 | 1595.218363 | 1666.34 | 1737.461637 | 0.925230 |  |
| GO:0034968\_histone\_lysine\_methylation | 6 | 0 | 0.000000 | -0.000000 | 1801 | 1595.218363 | 1666.34 | 1737.461637 | 0.925230 |  |
| GO:0035019\_somatic\_stem\_cell\_maintenance | 6 | 0 | 0.000000 | -0.000000 | 1801 | 1595.218363 | 1666.34 | 1737.461637 | 0.925230 |  |
| GO:0035094\_response\_to\_nicotine | 6 | 0 | 0.000000 | -0.000000 | 1801 | 1595.218363 | 1666.34 | 1737.461637 | 0.925230 |  |
| GO:0035121\_tail\_morphogenesis | 6 | 0 | 0.000000 | -0.000000 | 1801 | 1595.218363 | 1666.34 | 1737.461637 | 0.925230 |  |
| GO:0040016\_embryonic\_cleavage | 6 | 0 | 0.000000 | -0.000000 | 1801 | 1595.218363 | 1666.34 | 1737.461637 | 0.925230 |  |
| GO:0040023\_establishment\_of\_nucleus\_localization | 6 | 0 | 0.000000 | -0.000000 | 1801 | 1595.218363 | 1666.34 | 1737.461637 | 0.925230 |  |
| GO:0040036\_regulation\_of\_fibroblast\_growth\_factor\_receptor\_signaling\_pathway | 6 | 0 | 0.000000 | -0.000000 | 1801 | 1595.218363 | 1666.34 | 1737.461637 | 0.925230 |  |
| GO:0042053\_regulation\_of\_dopamine\_metabolic\_process | 6 | 0 | 0.000000 | -0.000000 | 1801 | 1595.218363 | 1666.34 | 1737.461637 | 0.925230 |  |
| GO:0042069\_regulation\_of\_catecholamine\_metabolic\_process | 6 | 0 | 0.000000 | -0.000000 | 1801 | 1595.218363 | 1666.34 | 1737.461637 | 0.925230 |  |
| GO:0042246\_tissue\_regeneration | 6 | 0 | 0.000000 | -0.000000 | 1801 | 1595.218363 | 1666.34 | 1737.461637 | 0.925230 |  |
| GO:0042307\_positive\_regulation\_of\_protein\_import\_into\_nucleus | 6 | 0 | 0.000000 | -0.000000 | 1801 | 1595.218363 | 1666.34 | 1737.461637 | 0.925230 |  |
| GO:0042308\_negative\_regulation\_of\_protein\_import\_into\_nucleus | 6 | 0 | 0.000000 | -0.000000 | 1801 | 1595.218363 | 1666.34 | 1737.461637 | 0.925230 |  |
| GO:0042403\_thyroid\_hormone\_metabolic\_process | 6 | 0 | 0.000000 | -0.000000 | 1801 | 1595.218363 | 1666.34 | 1737.461637 | 0.925230 |  |
| GO:0042481\_regulation\_of\_odontogenesis | 6 | 0 | 0.000000 | -0.000000 | 1801 | 1595.218363 | 1666.34 | 1737.461637 | 0.925230 |  |
| GO:0042492\_gamma-delta\_T\_cell\_differentiation | 6 | 0 | 0.000000 | -0.000000 | 1801 | 1595.218363 | 1666.34 | 1737.461637 | 0.925230 |  |
| GO:0042953\_lipoprotein\_transport | 6 | 0 | 0.000000 | -0.000000 | 1801 | 1595.218363 | 1666.34 | 1737.461637 | 0.925230 |  |
| GO:0043064\_flagellum\_organization | 6 | 0 | 0.000000 | -0.000000 | 1801 | 1595.218363 | 1666.34 | 1737.461637 | 0.925230 |  |
| GO:0043154\_negative\_regulation\_of\_caspase\_activity | 6 | 0 | 0.000000 | -0.000000 | 1801 | 1595.218363 | 1666.34 | 1737.461637 | 0.925230 |  |
| GO:0043255\_regulation\_of\_carbohydrate\_biosynthetic\_process | 6 | 0 | 0.000000 | -0.000000 | 1801 | 1595.218363 | 1666.34 | 1737.461637 | 0.925230 |  |
| GO:0043271\_negative\_regulation\_of\_ion\_transport | 6 | 0 | 0.000000 | -0.000000 | 1801 | 1595.218363 | 1666.34 | 1737.461637 | 0.925230 |  |
| GO:0043278\_response\_to\_morphine | 6 | 0 | 0.000000 | -0.000000 | 1801 | 1595.218363 | 1666.34 | 1737.461637 | 0.925230 |  |
| GO:0043300\_regulation\_of\_leukocyte\_degranulation | 6 | 0 | 0.000000 | -0.000000 | 1801 | 1595.218363 | 1666.34 | 1737.461637 | 0.925230 |  |
| GO:0043467\_regulation\_of\_generation\_of\_precursor\_metabolites\_and\_energy | 6 | 0 | 0.000000 | -0.000000 | 1801 | 1595.218363 | 1666.34 | 1737.461637 | 0.925230 |  |
| GO:0043547\_positive\_regulation\_of\_GTPase\_activity | 6 | 0 | 0.000000 | -0.000000 | 1801 | 1595.218363 | 1666.34 | 1737.461637 | 0.925230 |  |
| GO:0043627\_response\_to\_estrogen\_stimulus | 6 | 0 | 0.000000 | -0.000000 | 1801 | 1595.218363 | 1666.34 | 1737.461637 | 0.925230 |  |
| GO:0044269\_glycerol\_ether\_catabolic\_process | 6 | 0 | 0.000000 | -0.000000 | 1801 | 1595.218363 | 1666.34 | 1737.461637 | 0.925230 |  |
| GO:0045072\_regulation\_of\_interferon-gamma\_biosynthetic\_process | 6 | 0 | 0.000000 | -0.000000 | 1801 | 1595.218363 | 1666.34 | 1737.461637 | 0.925230 |  |
| GO:0045084\_positive\_regulation\_of\_interleukin-12\_biosynthetic\_process | 6 | 0 | 0.000000 | -0.000000 | 1801 | 1595.218363 | 1666.34 | 1737.461637 | 0.925230 |  |
| GO:0045124\_regulation\_of\_bone\_resorption | 6 | 0 | 0.000000 | -0.000000 | 1801 | 1595.218363 | 1666.34 | 1737.461637 | 0.925230 |  |
| GO:0045176\_apical\_protein\_localization | 6 | 0 | 0.000000 | -0.000000 | 1801 | 1595.218363 | 1666.34 | 1737.461637 | 0.925230 |  |
| GO:0045540\_regulation\_of\_cholesterol\_biosynthetic\_process | 6 | 0 | 0.000000 | -0.000000 | 1801 | 1595.218363 | 1666.34 | 1737.461637 | 0.925230 |  |
| GO:0045579\_positive\_regulation\_of\_B\_cell\_differentiation | 6 | 0 | 0.000000 | -0.000000 | 1801 | 1595.218363 | 1666.34 | 1737.461637 | 0.925230 |  |
| GO:0045649\_regulation\_of\_macrophage\_differentiation | 6 | 0 | 0.000000 | -0.000000 | 1801 | 1595.218363 | 1666.34 | 1737.461637 | 0.925230 |  |
| GO:0045727\_positive\_regulation\_of\_translation | 6 | 0 | 0.000000 | -0.000000 | 1801 | 1595.218363 | 1666.34 | 1737.461637 | 0.925230 |  |
| GO:0045822\_negative\_regulation\_of\_heart\_contraction | 6 | 0 | 0.000000 | -0.000000 | 1801 | 1595.218363 | 1666.34 | 1737.461637 | 0.925230 |  |
| GO:0045824\_negative\_regulation\_of\_innate\_immune\_response | 6 | 0 | 0.000000 | -0.000000 | 1801 | 1595.218363 | 1666.34 | 1737.461637 | 0.925230 |  |
| GO:0045833\_negative\_regulation\_of\_lipid\_metabolic\_process | 6 | 0 | 0.000000 | -0.000000 | 1801 | 1595.218363 | 1666.34 | 1737.461637 | 0.925230 |  |
| GO:0045843\_negative\_regulation\_of\_striated\_muscle\_development | 6 | 0 | 0.000000 | -0.000000 | 1801 | 1595.218363 | 1666.34 | 1737.461637 | 0.925230 |  |
| GO:0045861\_negative\_regulation\_of\_proteolysis | 6 | 0 | 0.000000 | -0.000000 | 1801 | 1595.218363 | 1666.34 | 1737.461637 | 0.925230 |  |
| GO:0045913\_positive\_regulation\_of\_carbohydrate\_metabolic\_process | 6 | 0 | 0.000000 | -0.000000 | 1801 | 1595.218363 | 1666.34 | 1737.461637 | 0.925230 |  |
| GO:0045931\_positive\_regulation\_of\_mitotic\_cell\_cycle | 6 | 0 | 0.000000 | -0.000000 | 1801 | 1595.218363 | 1666.34 | 1737.461637 | 0.925230 |  |
| GO:0045933\_positive\_regulation\_of\_muscle\_contraction | 6 | 0 | 0.000000 | -0.000000 | 1801 | 1595.218363 | 1666.34 | 1737.461637 | 0.925230 |  |
| GO:0046427\_positive\_regulation\_of\_JAK-STAT\_cascade | 6 | 0 | 0.000000 | -0.000000 | 1801 | 1595.218363 | 1666.34 | 1737.461637 | 0.925230 |  |
| GO:0046460\_neutral\_lipid\_biosynthetic\_process | 6 | 0 | 0.000000 | -0.000000 | 1801 | 1595.218363 | 1666.34 | 1737.461637 | 0.925230 |  |
| GO:0046461\_neutral\_lipid\_catabolic\_process | 6 | 0 | 0.000000 | -0.000000 | 1801 | 1595.218363 | 1666.34 | 1737.461637 | 0.925230 |  |
| GO:0046463\_acylglycerol\_biosynthetic\_process | 6 | 0 | 0.000000 | -0.000000 | 1801 | 1595.218363 | 1666.34 | 1737.461637 | 0.925230 |  |
| GO:0046464\_acylglycerol\_catabolic\_process | 6 | 0 | 0.000000 | -0.000000 | 1801 | 1595.218363 | 1666.34 | 1737.461637 | 0.925230 |  |
| GO:0046466\_membrane\_lipid\_catabolic\_process | 6 | 0 | 0.000000 | -0.000000 | 1801 | 1595.218363 | 1666.34 | 1737.461637 | 0.925230 |  |
| GO:0046503\_glycerolipid\_catabolic\_process | 6 | 0 | 0.000000 | -0.000000 | 1801 | 1595.218363 | 1666.34 | 1737.461637 | 0.925230 |  |
| GO:0046580\_negative\_regulation\_of\_Ras\_protein\_signal\_transduction | 6 | 0 | 0.000000 | -0.000000 | 1801 | 1595.218363 | 1666.34 | 1737.461637 | 0.925230 |  |
| GO:0046627\_negative\_regulation\_of\_insulin\_receptor\_signaling\_pathway | 6 | 0 | 0.000000 | -0.000000 | 1801 | 1595.218363 | 1666.34 | 1737.461637 | 0.925230 |  |
| GO:0046629\_gamma-delta\_T\_cell\_activation | 6 | 0 | 0.000000 | -0.000000 | 1801 | 1595.218363 | 1666.34 | 1737.461637 | 0.925230 |  |
| GO:0046666\_retinal\_cell\_programmed\_cell\_death | 6 | 0 | 0.000000 | -0.000000 | 1801 | 1595.218363 | 1666.34 | 1737.461637 | 0.925230 |  |
| GO:0046889\_positive\_regulation\_of\_lipid\_biosynthetic\_process | 6 | 0 | 0.000000 | -0.000000 | 1801 | 1595.218363 | 1666.34 | 1737.461637 | 0.925230 |  |
| GO:0048041\_focal\_adhesion\_formation | 6 | 0 | 0.000000 | -0.000000 | 1801 | 1595.218363 | 1666.34 | 1737.461637 | 0.925230 |  |
| GO:0048147\_negative\_regulation\_of\_fibroblast\_proliferation | 6 | 0 | 0.000000 | -0.000000 | 1801 | 1595.218363 | 1666.34 | 1737.461637 | 0.925230 |  |
| GO:0048333\_mesodermal\_cell\_differentiation | 6 | 0 | 0.000000 | -0.000000 | 1801 | 1595.218363 | 1666.34 | 1737.461637 | 0.925230 |  |
| GO:0048541\_Peyer's\_patch\_development | 6 | 0 | 0.000000 | -0.000000 | 1801 | 1595.218363 | 1666.34 | 1737.461637 | 0.925230 |  |
| GO:0048563\_post-embryonic\_organ\_morphogenesis | 6 | 0 | 0.000000 | -0.000000 | 1801 | 1595.218363 | 1666.34 | 1737.461637 | 0.925230 |  |
| GO:0048635\_negative\_regulation\_of\_muscle\_development | 6 | 0 | 0.000000 | -0.000000 | 1801 | 1595.218363 | 1666.34 | 1737.461637 | 0.925230 |  |
| GO:0048644\_muscle\_organ\_morphogenesis | 6 | 0 | 0.000000 | -0.000000 | 1801 | 1595.218363 | 1666.34 | 1737.461637 | 0.925230 |  |
| GO:0048703\_embryonic\_viscerocranium\_morphogenesis | 6 | 0 | 0.000000 | -0.000000 | 1801 | 1595.218363 | 1666.34 | 1737.461637 | 0.925230 |  |
| GO:0048713\_regulation\_of\_oligodendrocyte\_differentiation | 6 | 0 | 0.000000 | -0.000000 | 1801 | 1595.218363 | 1666.34 | 1737.461637 | 0.925230 |  |
| GO:0050684\_regulation\_of\_mRNA\_processing | 6 | 0 | 0.000000 | -0.000000 | 1801 | 1595.218363 | 1666.34 | 1737.461637 | 0.925230 |  |
| GO:0050732\_negative\_regulation\_of\_peptidyl-tyrosine\_phosphorylation | 6 | 0 | 0.000000 | -0.000000 | 1801 | 1595.218363 | 1666.34 | 1737.461637 | 0.925230 |  |
| GO:0050805\_negative\_regulation\_of\_synaptic\_transmission | 6 | 0 | 0.000000 | -0.000000 | 1801 | 1595.218363 | 1666.34 | 1737.461637 | 0.925230 |  |
| GO:0050821\_protein\_stabilization | 6 | 0 | 0.000000 | -0.000000 | 1801 | 1595.218363 | 1666.34 | 1737.461637 | 0.925230 |  |
| GO:0050829\_defense\_response\_to\_Gram-negative\_bacterium | 6 | 0 | 0.000000 | -0.000000 | 1801 | 1595.218363 | 1666.34 | 1737.461637 | 0.925230 |  |
| GO:0050872\_white\_fat\_cell\_differentiation | 6 | 0 | 0.000000 | -0.000000 | 1801 | 1595.218363 | 1666.34 | 1737.461637 | 0.925230 |  |
| GO:0050951\_sensory\_perception\_of\_temperature\_stimulus | 6 | 0 | 0.000000 | -0.000000 | 1801 | 1595.218363 | 1666.34 | 1737.461637 | 0.925230 |  |
| GO:0050966\_detection\_of\_mechanical\_stimulus\_involved\_in\_sensory\_perception\_of\_pain | 6 | 0 | 0.000000 | -0.000000 | 1801 | 1595.218363 | 1666.34 | 1737.461637 | 0.925230 |  |
| GO:0051058\_negative\_regulation\_of\_small\_GTPase\_mediated\_signal\_transduction | 6 | 0 | 0.000000 | -0.000000 | 1801 | 1595.218363 | 1666.34 | 1737.461637 | 0.925230 |  |
| GO:0051085\_chaperone\_mediated\_protein\_folding\_requiring\_cofactor | 6 | 0 | 0.000000 | -0.000000 | 1801 | 1595.218363 | 1666.34 | 1737.461637 | 0.925230 |  |
| GO:0051384\_response\_to\_glucocorticoid\_stimulus | 6 | 0 | 0.000000 | -0.000000 | 1801 | 1595.218363 | 1666.34 | 1737.461637 | 0.925230 |  |
| GO:0051592\_response\_to\_calcium\_ion | 6 | 0 | 0.000000 | -0.000000 | 1801 | 1595.218363 | 1666.34 | 1737.461637 | 0.925230 |  |
| GO:0051875\_pigment\_granule\_localization | 6 | 0 | 0.000000 | -0.000000 | 1801 | 1595.218363 | 1666.34 | 1737.461637 | 0.925230 |  |
| GO:0051881\_regulation\_of\_mitochondrial\_membrane\_potential | 6 | 0 | 0.000000 | -0.000000 | 1801 | 1595.218363 | 1666.34 | 1737.461637 | 0.925230 |  |
| GO:0051970\_negative\_regulation\_of\_transmission\_of\_nerve\_impulse | 6 | 0 | 0.000000 | -0.000000 | 1801 | 1595.218363 | 1666.34 | 1737.461637 | 0.925230 |  |
| GO:0055081\_anion\_homeostasis | 6 | 0 | 0.000000 | -0.000000 | 1801 | 1595.218363 | 1666.34 | 1737.461637 | 0.925230 |  |
| GO:0060013\_righting\_reflex | 6 | 0 | 0.000000 | -0.000000 | 1801 | 1595.218363 | 1666.34 | 1737.461637 | 0.925230 |  |
| GO:0060017\_parathyroid\_gland\_development | 6 | 0 | 0.000000 | -0.000000 | 1801 | 1595.218363 | 1666.34 | 1737.461637 | 0.925230 |  |
| GO:0060056\_mammary\_gland\_involution | 6 | 0 | 0.000000 | -0.000000 | 1801 | 1595.218363 | 1666.34 | 1737.461637 | 0.925230 |  |
| GO:0060134\_prepulse\_inhibition | 6 | 0 | 0.000000 | -0.000000 | 1801 | 1595.218363 | 1666.34 | 1737.461637 | 0.925230 |  |
| GO:0060271\_cilium\_morphogenesis | 6 | 0 | 0.000000 | -0.000000 | 1801 | 1595.218363 | 1666.34 | 1737.461637 | 0.925230 |  |
| GO:0060638\_mesenchymal-epithelial\_cell\_signaling | 6 | 0 | 0.000000 | -0.000000 | 1801 | 1595.218363 | 1666.34 | 1737.461637 | 0.925230 |  |
| GO:0060710\_chorio-allantoic\_fusion | 6 | 0 | 0.000000 | -0.000000 | 1801 | 1595.218363 | 1666.34 | 1737.461637 | 0.925230 |  |
| GO:0065004\_protein-DNA\_complex\_assembly | 6 | 0 | 0.000000 | -0.000000 | 1801 | 1595.218363 | 1666.34 | 1737.461637 | 0.925230 |  |
| GO:0002253\_activation\_of\_immune\_response | 54 | 0 | 0.000000 | -0.000000 | 1806 | 1600.425589 | 1671.0 | 1741.574411 | 0.925249 |  |
| GO:0006091\_generation\_of\_precursor\_metabolites\_and\_energy | 54 | 0 | 0.000000 | -0.000000 | 1806 | 1600.425589 | 1671.0 | 1741.574411 | 0.925249 |  |
| GO:0006164\_purine\_nucleotide\_biosynthetic\_process | 54 | 0 | 0.000000 | -0.000000 | 1806 | 1600.425589 | 1671.0 | 1741.574411 | 0.925249 |  |
| GO:0006412\_translation | 54 | 0 | 0.000000 | -0.000000 | 1806 | 1600.425589 | 1671.0 | 1741.574411 | 0.925249 |  |
| GO:0015849\_organic\_acid\_transport | 54 | 0 | 0.000000 | -0.000000 | 1806 | 1600.425589 | 1671.0 | 1741.574411 | 0.925249 |  |
| GO:0000027\_ribosomal\_large\_subunit\_assembly | 1 | 0 |  |  |  |  |  |  |  |  |
| GO:0000042\_protein\_targeting\_to\_Golgi | 1 | 0 |  |  |  |  |  |  |  |  |
| GO:0000046\_autophagic\_vacuole\_fusion | 1 | 0 |  |  |  |  |  |  |  |  |
| GO:0000050\_urea\_cycle | 1 | 0 |  |  |  |  |  |  |  |  |
| GO:0000054\_ribosome\_export\_from\_nucleus | 1 | 0 |  |  |  |  |  |  |  |  |
| GO:0000055\_ribosomal\_large\_subunit\_export\_from\_nucleus | 1 | 0 |  |  |  |  |  |  |  |  |
| GO:0000056\_ribosomal\_small\_subunit\_export\_from\_nucleus | 1 | 0 |  |  |  |  |  |  |  |  |
| GO:0000072\_M\_phase\_specific\_microtubule\_process | 1 | 0 |  |  |  |  |  |  |  |  |
| GO:0000101\_sulfur\_amino\_acid\_transport | 1 | 0 |  |  |  |  |  |  |  |  |
| GO:0000147\_actin\_cortical\_patch\_assembly | 1 | 0 |  |  |  |  |  |  |  |  |
| GO:0000154\_rRNA\_modification | 1 | 0 |  |  |  |  |  |  |  |  |
| GO:0000183\_chromatin\_silencing\_at\_rDNA | 1 | 0 |  |  |  |  |  |  |  |  |
| GO:0000185\_activation\_of\_MAPKKK\_activity | 1 | 0 |  |  |  |  |  |  |  |  |
| GO:0000238\_zygotene | 1 | 0 |  |  |  |  |  |  |  |  |
| GO:0000255\_allantoin\_metabolic\_process | 1 | 0 |  |  |  |  |  |  |  |  |
| GO:0000266\_mitochondrial\_fission | 1 | 0 |  |  |  |  |  |  |  |  |
| GO:0000273\_lipoic\_acid\_metabolic\_process | 1 | 0 |  |  |  |  |  |  |  |  |
| GO:0000301\_retrograde\_transport\_\_vesicle\_recycling\_within\_Golgi | 1 | 0 |  |  |  |  |  |  |  |  |
| GO:0000394\_RNA\_splicing\_\_via\_endonucleolytic\_cleavage\_and\_ligation | 1 | 0 |  |  |  |  |  |  |  |  |
| GO:0000429\_regulation\_of\_transcription\_from\_RNA\_polymerase\_II\_promoter\_by\_carbon\_catabolites | 1 | 0 |  |  |  |  |  |  |  |  |
| GO:0000430\_regulation\_of\_transcription\_from\_RNA\_polymerase\_II\_promoter\_by\_glucose | 1 | 0 |  |  |  |  |  |  |  |  |
| GO:0000432\_positive\_regulation\_of\_transcription\_from\_RNA\_polymerase\_II\_promoter\_by\_glucose | 1 | 0 |  |  |  |  |  |  |  |  |
| GO:0000436\_positive\_regulation\_of\_transcription\_from\_RNA\_polymerase\_II\_promoter\_by\_carbon\_catabolites | 1 | 0 |  |  |  |  |  |  |  |  |
| GO:0000448\_cleavage\_in\_ITS2\_between\_5.8S\_rRNA\_and\_LSU-rRNA\_of\_tricistronic\_rRNA\_transcript\_(SSU-rRNA\_\_5.8S\_rRNA\_\_LSU-rRNA) | 1 | 0 |  |  |  |  |  |  |  |  |
| GO:0000460\_maturation\_of\_5.8S\_rRNA | 1 | 0 |  |  |  |  |  |  |  |  |
| GO:0000463\_maturation\_of\_LSU-rRNA\_from\_tricistronic\_rRNA\_transcript\_(SSU-rRNA\_\_5.8S\_rRNA\_\_LSU-rRNA) | 1 | 0 |  |  |  |  |  |  |  |  |
| GO:0000466\_maturation\_of\_5.8S\_rRNA\_from\_tricistronic\_rRNA\_transcript\_(SSU-rRNA\_\_5.8S\_rRNA\_\_LSU-rRNA) | 1 | 0 |  |  |  |  |  |  |  |  |
| GO:0000469\_cleavages\_during\_rRNA\_processing | 1 | 0 |  |  |  |  |  |  |  |  |
| GO:0000470\_maturation\_of\_LSU-rRNA | 1 | 0 |  |  |  |  |  |  |  |  |
| GO:0000478\_endonucleolytic\_cleavages\_during\_rRNA\_processing | 1 | 0 |  |  |  |  |  |  |  |  |
| GO:0000479\_endonucleolytic\_cleavage\_of\_tricistronic\_rRNA\_transcript\_(SSU-rRNA\_\_5.8S\_rRNA\_\_LSU-rRNA) | 1 | 0 |  |  |  |  |  |  |  |  |
| GO:0000705\_achiasmate\_meiosis\_I | 1 | 0 |  |  |  |  |  |  |  |  |
| GO:0000966\_RNA\_5'-end\_processing | 1 | 0 |  |  |  |  |  |  |  |  |
| GO:0001300\_chronological\_cell\_aging | 1 | 0 |  |  |  |  |  |  |  |  |
| GO:0001547\_antral\_ovarian\_follicle\_growth | 1 | 0 |  |  |  |  |  |  |  |  |
| GO:0001555\_oocyte\_growth | 1 | 0 |  |  |  |  |  |  |  |  |
| GO:0001560\_regulation\_of\_cell\_growth\_by\_extracellular\_stimulus | 1 | 0 |  |  |  |  |  |  |  |  |
| GO:0001660\_fever | 1 | 0 |  |  |  |  |  |  |  |  |
| GO:0001696\_gastric\_acid\_secretion | 1 | 0 |  |  |  |  |  |  |  |  |
| GO:0001712\_ectodermal\_cell\_fate\_commitment | 1 | 0 |  |  |  |  |  |  |  |  |
| GO:0001714\_endodermal\_cell\_fate\_specification | 1 | 0 |  |  |  |  |  |  |  |  |
| GO:0001762\_beta-alanine\_transport | 1 | 0 |  |  |  |  |  |  |  |  |
| GO:0001766\_membrane\_raft\_polarization | 1 | 0 |  |  |  |  |  |  |  |  |
| GO:0001811\_negative\_regulation\_of\_type\_I\_hypersensitivity | 1 | 0 |  |  |  |  |  |  |  |  |
| GO:0001821\_histamine\_secretion | 1 | 0 |  |  |  |  |  |  |  |  |
| GO:0001826\_inner\_cell\_mass\_cell\_differentiation | 1 | 0 |  |  |  |  |  |  |  |  |
| GO:0001830\_trophectodermal\_cell\_fate\_commitment | 1 | 0 |  |  |  |  |  |  |  |  |
| GO:0001834\_trophectodermal\_cell\_proliferation | 1 | 0 |  |  |  |  |  |  |  |  |
| GO:0001867\_complement\_activation\_\_lectin\_pathway | 1 | 0 |  |  |  |  |  |  |  |  |
| GO:0001880\_Mullerian\_duct\_regression | 1 | 0 |  |  |  |  |  |  |  |  |
| GO:0001887\_selenium\_metabolic\_process | 1 | 0 |  |  |  |  |  |  |  |  |
| GO:0001922\_B-1\_B\_cell\_homeostasis | 1 | 0 |  |  |  |  |  |  |  |  |
| GO:0001923\_B-1\_B\_cell\_differentiation | 1 | 0 |  |  |  |  |  |  |  |  |
| GO:0001941\_postsynaptic\_membrane\_organization | 1 | 0 |  |  |  |  |  |  |  |  |
| GO:0001946\_lymphangiogenesis | 1 | 0 |  |  |  |  |  |  |  |  |
| GO:0001956\_positive\_regulation\_of\_neurotransmitter\_secretion | 1 | 0 |  |  |  |  |  |  |  |  |
| GO:0001961\_positive\_regulation\_of\_cytokine-mediated\_signaling\_pathway | 1 | 0 |  |  |  |  |  |  |  |  |
| GO:0001979\_regulation\_of\_systemic\_arterial\_blood\_pressure\_by\_chemoreceptor\_signaling | 1 | 0 |  |  |  |  |  |  |  |  |
| GO:0001980\_regulation\_of\_systemic\_arterial\_blood\_pressure\_by\_ischemic\_conditions | 1 | 0 |  |  |  |  |  |  |  |  |
| GO:0001984\_vasodilation\_of\_artery\_during\_baroreceptor\_response\_to\_increased\_systemic\_arterial\_blood\_pressure | 1 | 0 |  |  |  |  |  |  |  |  |
| GO:0001985\_negative\_regulation\_of\_heart\_rate\_in\_baroreceptor\_response\_to\_increased\_systemic\_arterial\_blood\_pressure | 1 | 0 |  |  |  |  |  |  |  |  |
| GO:0001987\_vasoconstriction\_of\_artery\_involved\_in\_baroreceptor\_response\_to\_lowering\_of\_systemic\_arterial\_blood\_pressure | 1 | 0 |  |  |  |  |  |  |  |  |
| GO:0001988\_positive\_regulation\_of\_heart\_rate\_in\_baroreceptor\_response\_to\_decreased\_systemic\_arterial\_blood\_pressure | 1 | 0 |  |  |  |  |  |  |  |  |
| GO:0001994\_norepinephrine-epinephrine\_vasoconstriction\_involved\_in\_regulation\_of\_systemic\_arterial\_blood\_pressure | 1 | 0 |  |  |  |  |  |  |  |  |
| GO:0002001\_renin\_secretion\_into\_blood\_stream | 1 | 0 |  |  |  |  |  |  |  |  |
| GO:0002002\_regulation\_of\_angiotensin\_levels\_in\_blood | 1 | 0 |  |  |  |  |  |  |  |  |
| GO:0002003\_angiotensin\_maturation | 1 | 0 |  |  |  |  |  |  |  |  |
| GO:0002007\_detection\_of\_hypoxic\_conditions\_in\_blood\_by\_chemoreceptor\_signaling | 1 | 0 |  |  |  |  |  |  |  |  |
| GO:0002017\_regulation\_of\_blood\_volume\_by\_renal\_aldosterone | 1 | 0 |  |  |  |  |  |  |  |  |
| GO:0002023\_reduction\_of\_food\_intake\_in\_response\_to\_dietary\_excess | 1 | 0 |  |  |  |  |  |  |  |  |
| GO:0002031\_G-protein\_coupled\_receptor\_internalization | 1 | 0 |  |  |  |  |  |  |  |  |
| GO:0002036\_regulation\_of\_L-glutamate\_transport | 1 | 0 |  |  |  |  |  |  |  |  |
| GO:0002040\_sprouting\_angiogenesis | 1 | 0 |  |  |  |  |  |  |  |  |
| GO:0002041\_intussusceptive\_angiogenesis | 1 | 0 |  |  |  |  |  |  |  |  |
| GO:0002068\_glandular\_epithelial\_cell\_development | 1 | 0 |  |  |  |  |  |  |  |  |
| GO:0002069\_columnar\_cuboidal\_epithelial\_cell\_maturation | 1 | 0 |  |  |  |  |  |  |  |  |
| GO:0002071\_glandular\_epithelial\_cell\_maturation | 1 | 0 |  |  |  |  |  |  |  |  |
| GO:0002082\_regulation\_of\_oxidative\_phosphorylation | 1 | 0 |  |  |  |  |  |  |  |  |
| GO:0002084\_protein\_depalmitoylation | 1 | 0 |  |  |  |  |  |  |  |  |
| GO:0002085\_inhibition\_of\_neuroepithelial\_cell\_differentiation | 1 | 0 |  |  |  |  |  |  |  |  |
| GO:0002086\_diaphragm\_contraction | 1 | 0 |  |  |  |  |  |  |  |  |
| GO:0002118\_aggressive\_behavior | 1 | 0 |  |  |  |  |  |  |  |  |
| GO:0002121\_inter-male\_aggressive\_behavior | 1 | 0 |  |  |  |  |  |  |  |  |
| GO:0002124\_territorial\_aggressive\_behavior | 1 | 0 |  |  |  |  |  |  |  |  |
| GO:0002227\_innate\_immune\_response\_in\_mucosa | 1 | 0 |  |  |  |  |  |  |  |  |
| GO:0002232\_leukocyte\_chemotaxis\_during\_inflammatory\_response | 1 | 0 |  |  |  |  |  |  |  |  |
| GO:0002248\_connective\_tissue\_replacement\_during\_inflammatory\_response | 1 | 0 |  |  |  |  |  |  |  |  |
| GO:0002282\_microglial\_cell\_activation\_during\_immune\_response | 1 | 0 |  |  |  |  |  |  |  |  |
| GO:0002287\_alpha-beta\_T\_cell\_activation\_during\_immune\_response | 1 | 0 |  |  |  |  |  |  |  |  |
| GO:0002314\_germinal\_center\_B\_cell\_differentiation | 1 | 0 |  |  |  |  |  |  |  |  |
| GO:0002315\_marginal\_zone\_B\_cell\_differentiation | 1 | 0 |  |  |  |  |  |  |  |  |
| GO:0002316\_follicular\_B\_cell\_differentiation | 1 | 0 |  |  |  |  |  |  |  |  |
| GO:0002317\_plasma\_cell\_differentiation | 1 | 0 |  |  |  |  |  |  |  |  |
| GO:0002349\_histamine\_production\_during\_acute\_inflammatory\_response | 1 | 0 |  |  |  |  |  |  |  |  |
| GO:0002351\_serotonin\_production\_during\_acute\_inflammatory\_response | 1 | 0 |  |  |  |  |  |  |  |  |
| GO:0002355\_detection\_of\_tumor\_cell | 1 | 0 |  |  |  |  |  |  |  |  |
| GO:0002370\_natural\_killer\_cell\_cytokine\_production | 1 | 0 |  |  |  |  |  |  |  |  |
| GO:0002371\_dendritic\_cell\_cytokine\_production | 1 | 0 |  |  |  |  |  |  |  |  |
| GO:0002380\_immunoglobulin\_secretion\_during\_immune\_response | 1 | 0 |  |  |  |  |  |  |  |  |
| GO:0002396\_MHC\_protein\_complex\_assembly | 1 | 0 |  |  |  |  |  |  |  |  |
| GO:0002397\_MHC\_class\_I\_protein\_complex\_assembly | 1 | 0 |  |  |  |  |  |  |  |  |
| GO:0002420\_natural\_killer\_cell\_mediated\_cytotoxicity\_directed\_against\_tumor\_cell\_target | 1 | 0 |  |  |  |  |  |  |  |  |
| GO:0002423\_natural\_killer\_cell\_mediated\_immune\_response\_to\_tumor\_cell | 1 | 0 |  |  |  |  |  |  |  |  |
| GO:0002424\_T\_cell\_mediated\_immune\_response\_to\_tumor\_cell | 1 | 0 |  |  |  |  |  |  |  |  |
| GO:0002426\_immunoglobulin\_production\_in\_mucosal\_tissue | 1 | 0 |  |  |  |  |  |  |  |  |
| GO:0002431\_Fc\_receptor\_mediated\_stimulatory\_signaling\_pathway | 1 | 0 |  |  |  |  |  |  |  |  |
| GO:0002432\_granuloma\_formation | 1 | 0 |  |  |  |  |  |  |  |  |
| GO:0002441\_histamine\_secretion\_during\_acute\_inflammatory\_response | 1 | 0 |  |  |  |  |  |  |  |  |
| GO:0002442\_serotonin\_secretion\_during\_acute\_inflammatory\_response | 1 | 0 |  |  |  |  |  |  |  |  |
| GO:0002457\_T\_cell\_antigen\_processing\_and\_presentation | 1 | 0 |  |  |  |  |  |  |  |  |
| GO:0002458\_peripheral\_T\_cell\_tolerance\_induction | 1 | 0 |  |  |  |  |  |  |  |  |
| GO:0002461\_tolerance\_induction\_dependent\_upon\_immune\_response | 1 | 0 |  |  |  |  |  |  |  |  |
| GO:0002465\_peripheral\_tolerance\_induction | 1 | 0 |  |  |  |  |  |  |  |  |
| GO:0002468\_dendritic\_cell\_antigen\_processing\_and\_presentation | 1 | 0 |  |  |  |  |  |  |  |  |
| GO:0002476\_antigen\_processing\_and\_presentation\_of\_endogenous\_peptide\_antigen\_via\_MHC\_class\_Ib | 1 | 0 |  |  |  |  |  |  |  |  |
| GO:0002479\_antigen\_processing\_and\_presentation\_of\_exogenous\_peptide\_antigen\_via\_MHC\_class\_I\_\_TAP-dependent | 1 | 0 |  |  |  |  |  |  |  |  |
| GO:0002483\_antigen\_processing\_and\_presentation\_of\_endogenous\_peptide\_antigen | 1 | 0 |  |  |  |  |  |  |  |  |
| GO:0002501\_peptide\_antigen\_assembly\_with\_MHC\_protein\_complex | 1 | 0 |  |  |  |  |  |  |  |  |
| GO:0002502\_peptide\_antigen\_assembly\_with\_MHC\_class\_I\_protein\_complex | 1 | 0 |  |  |  |  |  |  |  |  |
| GO:0002508\_central\_tolerance\_induction | 1 | 0 |  |  |  |  |  |  |  |  |
| GO:0002510\_central\_B\_cell\_tolerance\_induction | 1 | 0 |  |  |  |  |  |  |  |  |
| GO:0002545\_chronic\_inflammatory\_response\_to\_non-antigenic\_stimulus | 1 | 0 |  |  |  |  |  |  |  |  |
| GO:0002553\_histamine\_secretion\_by\_mast\_cell | 1 | 0 |  |  |  |  |  |  |  |  |
| GO:0002554\_serotonin\_secretion\_by\_platelet | 1 | 0 |  |  |  |  |  |  |  |  |
| GO:0002572\_pro-T\_cell\_differentiation | 1 | 0 |  |  |  |  |  |  |  |  |
| GO:0002577\_regulation\_of\_antigen\_processing\_and\_presentation | 1 | 0 |  |  |  |  |  |  |  |  |
| GO:0002579\_positive\_regulation\_of\_antigen\_processing\_and\_presentation | 1 | 0 |  |  |  |  |  |  |  |  |
| GO:0002604\_regulation\_of\_dendritic\_cell\_antigen\_processing\_and\_presentation | 1 | 0 |  |  |  |  |  |  |  |  |
| GO:0002606\_positive\_regulation\_of\_dendritic\_cell\_antigen\_processing\_and\_presentation | 1 | 0 |  |  |  |  |  |  |  |  |
| GO:0002635\_negative\_regulation\_of\_germinal\_center\_formation | 1 | 0 |  |  |  |  |  |  |  |  |
| GO:0002646\_regulation\_of\_central\_tolerance\_induction | 1 | 0 |  |  |  |  |  |  |  |  |
| GO:0002648\_positive\_regulation\_of\_central\_tolerance\_induction | 1 | 0 |  |  |  |  |  |  |  |  |
| GO:0002649\_regulation\_of\_tolerance\_induction\_to\_self\_antigen | 1 | 0 |  |  |  |  |  |  |  |  |
| GO:0002651\_positive\_regulation\_of\_tolerance\_induction\_to\_self\_antigen | 1 | 0 |  |  |  |  |  |  |  |  |
| GO:0002652\_regulation\_of\_tolerance\_induction\_dependent\_upon\_immune\_response | 1 | 0 |  |  |  |  |  |  |  |  |
| GO:0002654\_positive\_regulation\_of\_tolerance\_induction\_dependent\_upon\_immune\_response | 1 | 0 |  |  |  |  |  |  |  |  |
| GO:0002658\_regulation\_of\_peripheral\_tolerance\_induction | 1 | 0 |  |  |  |  |  |  |  |  |
| GO:0002660\_positive\_regulation\_of\_peripheral\_tolerance\_induction | 1 | 0 |  |  |  |  |  |  |  |  |
| GO:0002677\_negative\_regulation\_of\_chronic\_inflammatory\_response | 1 | 0 |  |  |  |  |  |  |  |  |
| GO:0002678\_positive\_regulation\_of\_chronic\_inflammatory\_response | 1 | 0 |  |  |  |  |  |  |  |  |
| GO:0002701\_negative\_regulation\_of\_production\_of\_molecular\_mediator\_of\_immune\_response | 1 | 0 |  |  |  |  |  |  |  |  |
| GO:0002719\_negative\_regulation\_of\_cytokine\_production\_during\_immune\_response | 1 | 0 |  |  |  |  |  |  |  |  |
| GO:0002724\_regulation\_of\_T\_cell\_cytokine\_production | 1 | 0 |  |  |  |  |  |  |  |  |
| GO:0002727\_regulation\_of\_natural\_killer\_cell\_cytokine\_production | 1 | 0 |  |  |  |  |  |  |  |  |
| GO:0002729\_positive\_regulation\_of\_natural\_killer\_cell\_cytokine\_production | 1 | 0 |  |  |  |  |  |  |  |  |
| GO:0002730\_regulation\_of\_dendritic\_cell\_cytokine\_production | 1 | 0 |  |  |  |  |  |  |  |  |
| GO:0002756\_MyD88-independent\_toll-like\_receptor\_signaling\_pathway | 1 | 0 |  |  |  |  |  |  |  |  |
| GO:0002767\_immune\_response-inhibiting\_cell\_surface\_receptor\_signaling\_pathway | 1 | 0 |  |  |  |  |  |  |  |  |
| GO:0002769\_natural\_killer\_cell\_inhibitory\_signaling\_pathway | 1 | 0 |  |  |  |  |  |  |  |  |
| GO:0002840\_regulation\_of\_T\_cell\_mediated\_immune\_response\_to\_tumor\_cell | 1 | 0 |  |  |  |  |  |  |  |  |
| GO:0002842\_positive\_regulation\_of\_T\_cell\_mediated\_immune\_response\_to\_tumor\_cell | 1 | 0 |  |  |  |  |  |  |  |  |
| GO:0002849\_regulation\_of\_peripheral\_T\_cell\_tolerance\_induction | 1 | 0 |  |  |  |  |  |  |  |  |
| GO:0002851\_positive\_regulation\_of\_peripheral\_T\_cell\_tolerance\_induction | 1 | 0 |  |  |  |  |  |  |  |  |
| GO:0002855\_regulation\_of\_natural\_killer\_cell\_mediated\_immune\_response\_to\_tumor\_cell | 1 | 0 |  |  |  |  |  |  |  |  |
| GO:0002857\_positive\_regulation\_of\_natural\_killer\_cell\_mediated\_immune\_response\_to\_tumor\_cell | 1 | 0 |  |  |  |  |  |  |  |  |
| GO:0002858\_regulation\_of\_natural\_killer\_cell\_mediated\_cytotoxicity\_directed\_against\_tumor\_cell\_target | 1 | 0 |  |  |  |  |  |  |  |  |
| GO:0002860\_positive\_regulation\_of\_natural\_killer\_cell\_mediated\_cytotoxicity\_directed\_against\_tumor\_cell\_target | 1 | 0 |  |  |  |  |  |  |  |  |
| GO:0002880\_regulation\_of\_chronic\_inflammatory\_response\_to\_non-antigenic\_stimulus | 1 | 0 |  |  |  |  |  |  |  |  |
| GO:0002882\_positive\_regulation\_of\_chronic\_inflammatory\_response\_to\_non-antigenic\_stimulus | 1 | 0 |  |  |  |  |  |  |  |  |
| GO:0002895\_regulation\_of\_central\_B\_cell\_tolerance\_induction | 1 | 0 |  |  |  |  |  |  |  |  |
| GO:0002897\_positive\_regulation\_of\_central\_B\_cell\_tolerance\_induction | 1 | 0 |  |  |  |  |  |  |  |  |
| GO:0002901\_mature\_B\_cell\_apoptosis | 1 | 0 |  |  |  |  |  |  |  |  |
| GO:0002903\_negative\_regulation\_of\_B\_cell\_apoptosis | 1 | 0 |  |  |  |  |  |  |  |  |
| GO:0002905\_regulation\_of\_mature\_B\_cell\_apoptosis | 1 | 0 |  |  |  |  |  |  |  |  |
| GO:0002906\_negative\_regulation\_of\_mature\_B\_cell\_apoptosis | 1 | 0 |  |  |  |  |  |  |  |  |
| GO:0003011\_involuntary\_skeletal\_muscle\_contraction | 1 | 0 |  |  |  |  |  |  |  |  |
| GO:0003027\_regulation\_of\_systemic\_arterial\_blood\_pressure\_by\_carotid\_body\_chemoreceptor\_signaling | 1 | 0 |  |  |  |  |  |  |  |  |
| GO:0003029\_detection\_of\_hypoxic\_conditions\_in\_blood\_by\_carotid\_body\_chemoreceptor\_signaling | 1 | 0 |  |  |  |  |  |  |  |  |
| GO:0003032\_detection\_of\_oxygen | 1 | 0 |  |  |  |  |  |  |  |  |
| GO:0003056\_regulation\_of\_vascular\_smooth\_muscle\_contraction | 1 | 0 |  |  |  |  |  |  |  |  |
| GO:0003062\_regulation\_of\_heart\_rate\_by\_chemical\_signal | 1 | 0 |  |  |  |  |  |  |  |  |
| GO:0003065\_positive\_regulation\_of\_heart\_rate\_by\_epinephrine | 1 | 0 |  |  |  |  |  |  |  |  |
| GO:0003068\_regulation\_of\_systemic\_arterial\_blood\_pressure\_by\_acetylcholine | 1 | 0 |  |  |  |  |  |  |  |  |
| GO:0003069\_vasodilation\_by\_acetylcholine\_involved\_in\_regulation\_of\_systemic\_arterial\_blood\_pressure | 1 | 0 |  |  |  |  |  |  |  |  |
| GO:0003070\_regulation\_of\_systemic\_arterial\_blood\_pressure\_by\_neurotransmitter | 1 | 0 |  |  |  |  |  |  |  |  |
| GO:0003097\_renal\_water\_transport | 1 | 0 |  |  |  |  |  |  |  |  |
| GO:0005979\_regulation\_of\_glycogen\_biosynthetic\_process | 1 | 0 |  |  |  |  |  |  |  |  |
| GO:0005984\_disaccharide\_metabolic\_process | 1 | 0 |  |  |  |  |  |  |  |  |
| GO:0005988\_lactose\_metabolic\_process | 1 | 0 |  |  |  |  |  |  |  |  |
| GO:0005989\_lactose\_biosynthetic\_process | 1 | 0 |  |  |  |  |  |  |  |  |
| GO:0005997\_xylulose\_metabolic\_process | 1 | 0 |  |  |  |  |  |  |  |  |
| GO:0006000\_fructose\_metabolic\_process | 1 | 0 |  |  |  |  |  |  |  |  |
| GO:0006002\_fructose\_6-phosphate\_metabolic\_process | 1 | 0 |  |  |  |  |  |  |  |  |
| GO:0006004\_fucose\_metabolic\_process | 1 | 0 |  |  |  |  |  |  |  |  |
| GO:0006013\_mannose\_metabolic\_process | 1 | 0 |  |  |  |  |  |  |  |  |
| GO:0006060\_sorbitol\_metabolic\_process | 1 | 0 |  |  |  |  |  |  |  |  |
| GO:0006064\_glucuronate\_catabolic\_process | 1 | 0 |  |  |  |  |  |  |  |  |
| GO:0006086\_acetyl-CoA\_biosynthetic\_process\_from\_pyruvate | 1 | 0 |  |  |  |  |  |  |  |  |
| GO:0006098\_pentose-phosphate\_shunt | 1 | 0 |  |  |  |  |  |  |  |  |
| GO:0006101\_citrate\_metabolic\_process | 1 | 0 |  |  |  |  |  |  |  |  |
| GO:0006104\_succinyl-CoA\_metabolic\_process | 1 | 0 |  |  |  |  |  |  |  |  |
| GO:0006116\_NADH\_oxidation | 1 | 0 |  |  |  |  |  |  |  |  |
| GO:0006120\_mitochondrial\_electron\_transport\_\_NADH\_to\_ubiquinone | 1 | 0 |  |  |  |  |  |  |  |  |
| GO:0006154\_adenosine\_catabolic\_process | 1 | 0 |  |  |  |  |  |  |  |  |
| GO:0006157\_deoxyadenosine\_catabolic\_process | 1 | 0 |  |  |  |  |  |  |  |  |
| GO:0006167\_AMP\_biosynthetic\_process | 1 | 0 |  |  |  |  |  |  |  |  |
| GO:0006175\_dATP\_biosynthetic\_process | 1 | 0 |  |  |  |  |  |  |  |  |
| GO:0006178\_guanine\_salvage | 1 | 0 |  |  |  |  |  |  |  |  |
| GO:0006196\_AMP\_catabolic\_process | 1 | 0 |  |  |  |  |  |  |  |  |
| GO:0006203\_dGTP\_catabolic\_process | 1 | 0 |  |  |  |  |  |  |  |  |
| GO:0006208\_pyrimidine\_base\_catabolic\_process | 1 | 0 |  |  |  |  |  |  |  |  |
| GO:0006221\_pyrimidine\_nucleotide\_biosynthetic\_process | 1 | 0 |  |  |  |  |  |  |  |  |
| GO:0006235\_dTTP\_biosynthetic\_process | 1 | 0 |  |  |  |  |  |  |  |  |
| GO:0006244\_pyrimidine\_nucleotide\_catabolic\_process | 1 | 0 |  |  |  |  |  |  |  |  |
| GO:0006269\_DNA\_replication\_\_synthesis\_of\_RNA\_primer | 1 | 0 |  |  |  |  |  |  |  |  |
| GO:0006283\_transcription-coupled\_nucleotide-excision\_repair | 1 | 0 |  |  |  |  |  |  |  |  |
| GO:0006296\_nucleotide-excision\_repair\_\_DNA\_incision\_\_5'-to\_lesion | 1 | 0 |  |  |  |  |  |  |  |  |
| GO:0006307\_DNA\_dealkylation | 1 | 0 |  |  |  |  |  |  |  |  |
| GO:0006337\_nucleosome\_disassembly | 1 | 0 |  |  |  |  |  |  |  |  |
| GO:0006344\_maintenance\_of\_chromatin\_silencing | 1 | 0 |  |  |  |  |  |  |  |  |
| GO:0006356\_regulation\_of\_transcription\_from\_RNA\_polymerase\_I\_promoter | 1 | 0 |  |  |  |  |  |  |  |  |
| GO:0006388\_tRNA\_splicing\_\_via\_endonucleolytic\_cleavage\_and\_ligation | 1 | 0 |  |  |  |  |  |  |  |  |
| GO:0006407\_rRNA\_export\_from\_nucleus | 1 | 0 |  |  |  |  |  |  |  |  |
| GO:0006419\_alanyl-tRNA\_aminoacylation | 1 | 0 |  |  |  |  |  |  |  |  |
| GO:0006434\_seryl-tRNA\_aminoacylation | 1 | 0 |  |  |  |  |  |  |  |  |
| GO:0006447\_regulation\_of\_translational\_initiation\_by\_iron | 1 | 0 |  |  |  |  |  |  |  |  |
| GO:0006463\_steroid\_hormone\_receptor\_complex\_assembly | 1 | 0 |  |  |  |  |  |  |  |  |
| GO:0006467\_protein\_thiol-disulfide\_exchange | 1 | 0 |  |  |  |  |  |  |  |  |
| GO:0006474\_N-terminal\_protein\_amino\_acid\_acetylation | 1 | 0 |  |  |  |  |  |  |  |  |
| GO:0006481\_C-terminal\_protein\_amino\_acid\_methylation | 1 | 0 |  |  |  |  |  |  |  |  |
| GO:0006488\_dolichol-linked\_oligosaccharide\_biosynthetic\_process | 1 | 0 |  |  |  |  |  |  |  |  |
| GO:0006494\_protein\_amino\_acid\_terminal\_glycosylation | 1 | 0 |  |  |  |  |  |  |  |  |
| GO:0006496\_protein\_amino\_acid\_terminal\_N-glycosylation | 1 | 0 |  |  |  |  |  |  |  |  |
| GO:0006500\_N-terminal\_protein\_palmitoylation | 1 | 0 |  |  |  |  |  |  |  |  |
| GO:0006507\_GPI\_anchor\_release | 1 | 0 |  |  |  |  |  |  |  |  |
| GO:0006537\_glutamate\_biosynthetic\_process | 1 | 0 |  |  |  |  |  |  |  |  |
| GO:0006544\_glycine\_metabolic\_process | 1 | 0 |  |  |  |  |  |  |  |  |
| GO:0006549\_isoleucine\_metabolic\_process | 1 | 0 |  |  |  |  |  |  |  |  |
| GO:0006553\_lysine\_metabolic\_process | 1 | 0 |  |  |  |  |  |  |  |  |
| GO:0006554\_lysine\_catabolic\_process | 1 | 0 |  |  |  |  |  |  |  |  |
| GO:0006556\_S-adenosylmethionine\_biosynthetic\_process | 1 | 0 |  |  |  |  |  |  |  |  |
| GO:0006559\_L-phenylalanine\_catabolic\_process | 1 | 0 |  |  |  |  |  |  |  |  |
| GO:0006569\_tryptophan\_catabolic\_process | 1 | 0 |  |  |  |  |  |  |  |  |
| GO:0006572\_tyrosine\_catabolic\_process | 1 | 0 |  |  |  |  |  |  |  |  |
| GO:0006573\_valine\_metabolic\_process | 1 | 0 |  |  |  |  |  |  |  |  |
| GO:0006581\_acetylcholine\_catabolic\_process | 1 | 0 |  |  |  |  |  |  |  |  |
| GO:0006585\_dopamine\_biosynthetic\_process\_from\_tyrosine | 1 | 0 |  |  |  |  |  |  |  |  |
| GO:0006590\_thyroid\_hormone\_generation | 1 | 0 |  |  |  |  |  |  |  |  |
| GO:0006591\_ornithine\_metabolic\_process | 1 | 0 |  |  |  |  |  |  |  |  |
| GO:0006596\_polyamine\_biosynthetic\_process | 1 | 0 |  |  |  |  |  |  |  |  |
| GO:0006597\_spermine\_biosynthetic\_process | 1 | 0 |  |  |  |  |  |  |  |  |
| GO:0006601\_creatine\_biosynthetic\_process | 1 | 0 |  |  |  |  |  |  |  |  |
| GO:0006613\_cotranslational\_protein\_targeting\_to\_membrane | 1 | 0 |  |  |  |  |  |  |  |  |
| GO:0006622\_protein\_targeting\_to\_lysosome | 1 | 0 |  |  |  |  |  |  |  |  |
| GO:0006627\_mitochondrial\_protein\_processing\_during\_import | 1 | 0 |  |  |  |  |  |  |  |  |
| GO:0006653\_lecithin\_metabolic\_process | 1 | 0 |  |  |  |  |  |  |  |  |
| GO:0006654\_phosphatidic\_acid\_biosynthetic\_process | 1 | 0 |  |  |  |  |  |  |  |  |
| GO:0006658\_phosphatidylserine\_metabolic\_process | 1 | 0 |  |  |  |  |  |  |  |  |
| GO:0006659\_phosphatidylserine\_biosynthetic\_process | 1 | 0 |  |  |  |  |  |  |  |  |
| GO:0006667\_sphinganine\_metabolic\_process | 1 | 0 |  |  |  |  |  |  |  |  |
| GO:0006668\_sphinganine-1-phosphate\_metabolic\_process | 1 | 0 |  |  |  |  |  |  |  |  |
| GO:0006678\_glucosylceramide\_metabolic\_process | 1 | 0 |  |  |  |  |  |  |  |  |
| GO:0006682\_galactosylceramide\_biosynthetic\_process | 1 | 0 |  |  |  |  |  |  |  |  |
| GO:0006685\_sphingomyelin\_catabolic\_process | 1 | 0 |  |  |  |  |  |  |  |  |
| GO:0006700\_C21-steroid\_hormone\_biosynthetic\_process | 1 | 0 |  |  |  |  |  |  |  |  |
| GO:0006705\_mineralocorticoid\_biosynthetic\_process | 1 | 0 |  |  |  |  |  |  |  |  |
| GO:0006709\_progesterone\_catabolic\_process | 1 | 0 |  |  |  |  |  |  |  |  |
| GO:0006729\_tetrahydrobiopterin\_biosynthetic\_process | 1 | 0 |  |  |  |  |  |  |  |  |
| GO:0006734\_NADH\_metabolic\_process | 1 | 0 |  |  |  |  |  |  |  |  |
| GO:0006740\_NADPH\_regeneration | 1 | 0 |  |  |  |  |  |  |  |  |
| GO:0006741\_NADP\_biosynthetic\_process | 1 | 0 |  |  |  |  |  |  |  |  |
| GO:0006743\_ubiquinone\_metabolic\_process | 1 | 0 |  |  |  |  |  |  |  |  |
| GO:0006744\_ubiquinone\_biosynthetic\_process | 1 | 0 |  |  |  |  |  |  |  |  |
| GO:0006772\_thiamin\_metabolic\_process | 1 | 0 |  |  |  |  |  |  |  |  |
| GO:0006784\_heme\_a\_biosynthetic\_process | 1 | 0 |  |  |  |  |  |  |  |  |
| GO:0006797\_polyphosphate\_metabolic\_process | 1 | 0 |  |  |  |  |  |  |  |  |
| GO:0006798\_polyphosphate\_catabolic\_process | 1 | 0 |  |  |  |  |  |  |  |  |
| GO:0006824\_cobalt\_ion\_transport | 1 | 0 |  |  |  |  |  |  |  |  |
| GO:0006842\_tricarboxylic\_acid\_transport | 1 | 0 |  |  |  |  |  |  |  |  |
| GO:0006844\_acyl\_carnitine\_transport | 1 | 0 |  |  |  |  |  |  |  |  |
| GO:0006855\_multidrug\_transport | 1 | 0 |  |  |  |  |  |  |  |  |
| GO:0006863\_purine\_transport | 1 | 0 |  |  |  |  |  |  |  |  |
| GO:0006890\_retrograde\_vesicle-mediated\_transport\_\_Golgi\_to\_ER | 1 | 0 |  |  |  |  |  |  |  |  |
| GO:0006891\_intra-Golgi\_vesicle-mediated\_transport | 1 | 0 |  |  |  |  |  |  |  |  |
| GO:0006893\_Golgi\_to\_plasma\_membrane\_transport | 1 | 0 |  |  |  |  |  |  |  |  |
| GO:0006895\_Golgi\_to\_endosome\_transport | 1 | 0 |  |  |  |  |  |  |  |  |
| GO:0006896\_Golgi\_to\_vacuole\_transport | 1 | 0 |  |  |  |  |  |  |  |  |
| GO:0006900\_membrane\_budding | 1 | 0 |  |  |  |  |  |  |  |  |
| GO:0006930\_substrate-bound\_cell\_migration\_\_cell\_extension | 1 | 0 |  |  |  |  |  |  |  |  |
| GO:0006931\_substrate-bound\_cell\_migration\_\_cell\_attachment\_to\_substrate | 1 | 0 |  |  |  |  |  |  |  |  |
| GO:0006933\_negative\_regulation\_of\_cell\_adhesion\_involved\_in\_substrate-bound\_cell\_migration | 1 | 0 |  |  |  |  |  |  |  |  |
| GO:0006957\_complement\_activation\_\_alternative\_pathway | 1 | 0 |  |  |  |  |  |  |  |  |
| GO:0006958\_complement\_activation\_\_classical\_pathway | 1 | 0 |  |  |  |  |  |  |  |  |
| GO:0006978\_DNA\_damage\_response\_\_signal\_transduction\_by\_p53\_class\_mediator\_resulting\_in\_transcription\_of\_p21\_class\_mediator | 1 | 0 |  |  |  |  |  |  |  |  |
| GO:0007016\_cytoskeletal\_anchoring\_at\_plasma\_membrane | 1 | 0 |  |  |  |  |  |  |  |  |
| GO:0007021\_tubulin\_complex\_assembly | 1 | 0 |  |  |  |  |  |  |  |  |
| GO:0007052\_mitotic\_spindle\_organization | 1 | 0 |  |  |  |  |  |  |  |  |
| GO:0007056\_spindle\_assembly\_involved\_in\_female\_meiosis | 1 | 0 |  |  |  |  |  |  |  |  |
| GO:0007057\_spindle\_assembly\_involved\_in\_female\_meiosis\_I | 1 | 0 |  |  |  |  |  |  |  |  |
| GO:0007063\_regulation\_of\_sister\_chromatid\_cohesion | 1 | 0 |  |  |  |  |  |  |  |  |
| GO:0007065\_male\_meiosis\_sister\_chromatid\_cohesion | 1 | 0 |  |  |  |  |  |  |  |  |
| GO:0007076\_mitotic\_chromosome\_condensation | 1 | 0 |  |  |  |  |  |  |  |  |
| GO:0007095\_mitotic\_cell\_cycle\_G2\_M\_transition\_DNA\_damage\_checkpoint | 1 | 0 |  |  |  |  |  |  |  |  |
| GO:0007096\_regulation\_of\_exit\_from\_mitosis | 1 | 0 |  |  |  |  |  |  |  |  |
| GO:0007158\_neuron\_adhesion | 1 | 0 |  |  |  |  |  |  |  |  |
| GO:0007168\_receptor\_guanylyl\_cyclase\_signaling\_pathway | 1 | 0 |  |  |  |  |  |  |  |  |
| GO:0007197\_inhibition\_of\_adenylate\_cyclase\_activity\_by\_muscarinic\_acetylcholine\_receptor\_signaling\_pathway | 1 | 0 |  |  |  |  |  |  |  |  |
| GO:0007207\_activation\_of\_phospholipase\_C\_activity\_by\_muscarinic\_acetylcholine\_receptor\_signaling\_pathway | 1 | 0 |  |  |  |  |  |  |  |  |
| GO:0007208\_activation\_of\_phospholipase\_C\_activity\_by\_serotonin\_receptor\_signaling\_pathway | 1 | 0 |  |  |  |  |  |  |  |  |
| GO:0007217\_tachykinin\_receptor\_signaling\_pathway | 1 | 0 |  |  |  |  |  |  |  |  |
| GO:0007221\_positive\_regulation\_of\_transcription\_of\_Notch\_receptor\_target | 1 | 0 |  |  |  |  |  |  |  |  |
| GO:0007223\_Wnt\_receptor\_signaling\_pathway\_\_calcium\_modulating\_pathway | 1 | 0 |  |  |  |  |  |  |  |  |
| GO:0007225\_patched\_ligand\_processing | 1 | 0 |  |  |  |  |  |  |  |  |
| GO:0007227\_signal\_transduction\_downstream\_of\_smoothened | 1 | 0 |  |  |  |  |  |  |  |  |
| GO:0007228\_positive\_regulation\_of\_hh\_target\_transcription\_factor\_activity | 1 | 0 |  |  |  |  |  |  |  |  |
| GO:0007231\_osmosensory\_signaling\_pathway | 1 | 0 |  |  |  |  |  |  |  |  |
| GO:0007290\_spermatid\_nucleus\_elongation | 1 | 0 |  |  |  |  |  |  |  |  |
| GO:0007296\_vitellogenesis | 1 | 0 |  |  |  |  |  |  |  |  |
| GO:0007321\_sperm\_displacement | 1 | 0 |  |  |  |  |  |  |  |  |
| GO:0007380\_specification\_of\_segmental\_identity\_\_head | 1 | 0 |  |  |  |  |  |  |  |  |
| GO:0007382\_specification\_of\_segmental\_identity\_\_maxillary\_segment | 1 | 0 |  |  |  |  |  |  |  |  |
| GO:0007400\_neuroblast\_fate\_determination | 1 | 0 |  |  |  |  |  |  |  |  |
| GO:0007402\_ganglion\_mother\_cell\_fate\_determination | 1 | 0 |  |  |  |  |  |  |  |  |
| GO:0007495\_visceral\_mesoderm-endoderm\_interaction\_involved\_in\_midgut\_development | 1 | 0 |  |  |  |  |  |  |  |  |
| GO:0007497\_posterior\_midgut\_development | 1 | 0 |  |  |  |  |  |  |  |  |
| GO:0007499\_ectoderm\_and\_mesoderm\_interaction | 1 | 0 |  |  |  |  |  |  |  |  |
| GO:0007500\_mesodermal\_cell\_fate\_determination | 1 | 0 |  |  |  |  |  |  |  |  |
| GO:0007509\_mesoderm\_migration | 1 | 0 |  |  |  |  |  |  |  |  |
| GO:0007518\_myoblast\_cell\_fate\_determination | 1 | 0 |  |  |  |  |  |  |  |  |
| GO:0007521\_muscle\_cell\_fate\_determination | 1 | 0 |  |  |  |  |  |  |  |  |
| GO:0007522\_visceral\_muscle\_development | 1 | 0 |  |  |  |  |  |  |  |  |
| GO:0007529\_establishment\_of\_synaptic\_specificity\_at\_neuromuscular\_junction | 1 | 0 |  |  |  |  |  |  |  |  |
| GO:0007538\_primary\_sex\_determination | 1 | 0 |  |  |  |  |  |  |  |  |
| GO:0007542\_primary\_sex\_determination\_\_germ-line | 1 | 0 |  |  |  |  |  |  |  |  |
| GO:0007567\_parturition | 1 | 0 |  |  |  |  |  |  |  |  |
| GO:0007614\_short-term\_memory | 1 | 0 |  |  |  |  |  |  |  |  |
| GO:0007621\_negative\_regulation\_of\_female\_receptivity | 1 | 0 |  |  |  |  |  |  |  |  |
| GO:0008049\_male\_courtship\_behavior | 1 | 0 |  |  |  |  |  |  |  |  |
| GO:0008050\_female\_courtship\_behavior | 1 | 0 |  |  |  |  |  |  |  |  |
| GO:0008052\_sensory\_organ\_boundary\_specification | 1 | 0 |  |  |  |  |  |  |  |  |
| GO:0008054\_cyclin\_catabolic\_process | 1 | 0 |  |  |  |  |  |  |  |  |
| GO:0008057\_eye\_pigment\_granule\_organization | 1 | 0 |  |  |  |  |  |  |  |  |
| GO:0008078\_mesodermal\_cell\_migration | 1 | 0 |  |  |  |  |  |  |  |  |
| GO:0008208\_C21-steroid\_hormone\_catabolic\_process | 1 | 0 |  |  |  |  |  |  |  |  |
| GO:0008216\_spermidine\_metabolic\_process | 1 | 0 |  |  |  |  |  |  |  |  |
| GO:0008292\_acetylcholine\_biosynthetic\_process | 1 | 0 |  |  |  |  |  |  |  |  |
| GO:0008295\_spermidine\_biosynthetic\_process | 1 | 0 |  |  |  |  |  |  |  |  |
| GO:0008300\_isoprenoid\_catabolic\_process | 1 | 0 |  |  |  |  |  |  |  |  |
| GO:0008333\_endosome\_to\_lysosome\_transport | 1 | 0 |  |  |  |  |  |  |  |  |
| GO:0008355\_olfactory\_learning | 1 | 0 |  |  |  |  |  |  |  |  |
| GO:0008611\_ether\_lipid\_biosynthetic\_process | 1 | 0 |  |  |  |  |  |  |  |  |
| GO:0008626\_induction\_of\_apoptosis\_by\_granzyme | 1 | 0 |  |  |  |  |  |  |  |  |
| GO:0008633\_activation\_of\_pro-apoptotic\_gene\_products | 1 | 0 |  |  |  |  |  |  |  |  |
| GO:0008653\_lipopolysaccharide\_metabolic\_process | 1 | 0 |  |  |  |  |  |  |  |  |
| GO:0009068\_aspartate\_family\_amino\_acid\_catabolic\_process | 1 | 0 |  |  |  |  |  |  |  |  |
| GO:0009084\_glutamine\_family\_amino\_acid\_biosynthetic\_process | 1 | 0 |  |  |  |  |  |  |  |  |
| GO:0009088\_threonine\_biosynthetic\_process | 1 | 0 |  |  |  |  |  |  |  |  |
| GO:0009105\_lipoic\_acid\_biosynthetic\_process | 1 | 0 |  |  |  |  |  |  |  |  |
| GO:0009109\_coenzyme\_catabolic\_process | 1 | 0 |  |  |  |  |  |  |  |  |
| GO:0009111\_vitamin\_catabolic\_process | 1 | 0 |  |  |  |  |  |  |  |  |
| GO:0009113\_purine\_base\_biosynthetic\_process | 1 | 0 |  |  |  |  |  |  |  |  |
| GO:0009127\_purine\_nucleoside\_monophosphate\_biosynthetic\_process | 1 | 0 |  |  |  |  |  |  |  |  |
| GO:0009128\_purine\_nucleoside\_monophosphate\_catabolic\_process | 1 | 0 |  |  |  |  |  |  |  |  |
| GO:0009129\_pyrimidine\_nucleoside\_monophosphate\_metabolic\_process | 1 | 0 |  |  |  |  |  |  |  |  |
| GO:0009131\_pyrimidine\_nucleoside\_monophosphate\_catabolic\_process | 1 | 0 |  |  |  |  |  |  |  |  |
| GO:0009133\_nucleoside\_diphosphate\_biosynthetic\_process | 1 | 0 |  |  |  |  |  |  |  |  |
| GO:0009145\_purine\_nucleoside\_triphosphate\_biosynthetic\_process | 1 | 0 |  |  |  |  |  |  |  |  |
| GO:0009147\_pyrimidine\_nucleoside\_triphosphate\_metabolic\_process | 1 | 0 |  |  |  |  |  |  |  |  |
| GO:0009148\_pyrimidine\_nucleoside\_triphosphate\_biosynthetic\_process | 1 | 0 |  |  |  |  |  |  |  |  |
| GO:0009152\_purine\_ribonucleotide\_biosynthetic\_process | 1 | 0 |  |  |  |  |  |  |  |  |
| GO:0009153\_purine\_deoxyribonucleotide\_biosynthetic\_process | 1 | 0 |  |  |  |  |  |  |  |  |
| GO:0009156\_ribonucleoside\_monophosphate\_biosynthetic\_process | 1 | 0 |  |  |  |  |  |  |  |  |
| GO:0009158\_ribonucleoside\_monophosphate\_catabolic\_process | 1 | 0 |  |  |  |  |  |  |  |  |
| GO:0009159\_deoxyribonucleoside\_monophosphate\_catabolic\_process | 1 | 0 |  |  |  |  |  |  |  |  |
| GO:0009162\_deoxyribonucleoside\_monophosphate\_metabolic\_process | 1 | 0 |  |  |  |  |  |  |  |  |
| GO:0009168\_purine\_ribonucleoside\_monophosphate\_biosynthetic\_process | 1 | 0 |  |  |  |  |  |  |  |  |
| GO:0009169\_purine\_ribonucleoside\_monophosphate\_catabolic\_process | 1 | 0 |  |  |  |  |  |  |  |  |
| GO:0009176\_pyrimidine\_deoxyribonucleoside\_monophosphate\_metabolic\_process | 1 | 0 |  |  |  |  |  |  |  |  |
| GO:0009178\_pyrimidine\_deoxyribonucleoside\_monophosphate\_catabolic\_process | 1 | 0 |  |  |  |  |  |  |  |  |
| GO:0009211\_pyrimidine\_deoxyribonucleoside\_triphosphate\_metabolic\_process | 1 | 0 |  |  |  |  |  |  |  |  |
| GO:0009212\_pyrimidine\_deoxyribonucleoside\_triphosphate\_biosynthetic\_process | 1 | 0 |  |  |  |  |  |  |  |  |
| GO:0009216\_purine\_deoxyribonucleoside\_triphosphate\_biosynthetic\_process | 1 | 0 |  |  |  |  |  |  |  |  |
| GO:0009221\_pyrimidine\_deoxyribonucleotide\_biosynthetic\_process | 1 | 0 |  |  |  |  |  |  |  |  |
| GO:0009223\_pyrimidine\_deoxyribonucleotide\_catabolic\_process | 1 | 0 |  |  |  |  |  |  |  |  |
| GO:0009260\_ribonucleotide\_biosynthetic\_process | 1 | 0 |  |  |  |  |  |  |  |  |
| GO:0009405\_pathogenesis | 1 | 0 |  |  |  |  |  |  |  |  |
| GO:0009414\_response\_to\_water\_deprivation | 1 | 0 |  |  |  |  |  |  |  |  |
| GO:0009415\_response\_to\_water | 1 | 0 |  |  |  |  |  |  |  |  |
| GO:0009449\_gamma-aminobutyric\_acid\_biosynthetic\_process | 1 | 0 |  |  |  |  |  |  |  |  |
| GO:0009450\_gamma-aminobutyric\_acid\_catabolic\_process | 1 | 0 |  |  |  |  |  |  |  |  |
| GO:0009589\_detection\_of\_UV | 1 | 0 |  |  |  |  |  |  |  |  |
| GO:0009590\_detection\_of\_gravity | 1 | 0 |  |  |  |  |  |  |  |  |
| GO:0009624\_response\_to\_nematode | 1 | 0 |  |  |  |  |  |  |  |  |
| GO:0009629\_response\_to\_gravity | 1 | 0 |  |  |  |  |  |  |  |  |
| GO:0009648\_photoperiodism | 1 | 0 |  |  |  |  |  |  |  |  |
| GO:0009690\_cytokinin\_metabolic\_process | 1 | 0 |  |  |  |  |  |  |  |  |
| GO:0009691\_cytokinin\_biosynthetic\_process | 1 | 0 |  |  |  |  |  |  |  |  |
| GO:0009786\_regulation\_of\_asymmetric\_cell\_division | 1 | 0 |  |  |  |  |  |  |  |  |
| GO:0009794\_regulation\_of\_mitotic\_cell\_cycle\_\_embryonic | 1 | 0 |  |  |  |  |  |  |  |  |
| GO:0009956\_radial\_pattern\_formation | 1 | 0 |  |  |  |  |  |  |  |  |
| GO:0009957\_epidermal\_cell\_fate\_specification | 1 | 0 |  |  |  |  |  |  |  |  |
| GO:0009992\_cellular\_water\_homeostasis | 1 | 0 |  |  |  |  |  |  |  |  |
| GO:0010032\_meiotic\_chromosome\_condensation | 1 | 0 |  |  |  |  |  |  |  |  |
| GO:0010039\_response\_to\_iron\_ion | 1 | 0 |  |  |  |  |  |  |  |  |
| GO:0010042\_response\_to\_manganese\_ion | 1 | 0 |  |  |  |  |  |  |  |  |
| GO:0010045\_response\_to\_nickel\_ion | 1 | 0 |  |  |  |  |  |  |  |  |
| GO:0010046\_response\_to\_mycotoxin | 1 | 0 |  |  |  |  |  |  |  |  |
| GO:0010107\_potassium\_ion\_import | 1 | 0 |  |  |  |  |  |  |  |  |
| GO:0010155\_regulation\_of\_proton\_transport | 1 | 0 |  |  |  |  |  |  |  |  |
| GO:0010160\_formation\_of\_organ\_boundary | 1 | 0 |  |  |  |  |  |  |  |  |
| GO:0010260\_organ\_senescence | 1 | 0 |  |  |  |  |  |  |  |  |
| GO:0010310\_regulation\_of\_hydrogen\_peroxide\_metabolic\_process | 1 | 0 |  |  |  |  |  |  |  |  |
| GO:0010447\_response\_to\_acidity | 1 | 0 |  |  |  |  |  |  |  |  |
| GO:0010452\_histone\_H3-K36\_methylation | 1 | 0 |  |  |  |  |  |  |  |  |
| GO:0010455\_positive\_regulation\_of\_cell\_fate\_commitment | 1 | 0 |  |  |  |  |  |  |  |  |
| GO:0010470\_regulation\_of\_gastrulation | 1 | 0 |  |  |  |  |  |  |  |  |
| GO:0010508\_positive\_regulation\_of\_autophagy | 1 | 0 |  |  |  |  |  |  |  |  |
| GO:0010519\_negative\_regulation\_of\_phospholipase\_activity | 1 | 0 |  |  |  |  |  |  |  |  |
| GO:0010520\_regulation\_of\_reciprocal\_meiotic\_recombination | 1 | 0 |  |  |  |  |  |  |  |  |
| GO:0010523\_negative\_regulation\_of\_calcium\_ion\_transport\_into\_cytosol | 1 | 0 |  |  |  |  |  |  |  |  |
| GO:0010561\_negative\_regulation\_of\_glycoprotein\_biosynthetic\_process | 1 | 0 |  |  |  |  |  |  |  |  |
| GO:0010569\_regulation\_of\_double-strand\_break\_repair\_via\_homologous\_recombination | 1 | 0 |  |  |  |  |  |  |  |  |
| GO:0010594\_regulation\_of\_endothelial\_cell\_migration | 1 | 0 |  |  |  |  |  |  |  |  |
| GO:0010596\_negative\_regulation\_of\_endothelial\_cell\_migration | 1 | 0 |  |  |  |  |  |  |  |  |
| GO:0010611\_regulation\_of\_cardiac\_muscle\_hypertrophy | 1 | 0 |  |  |  |  |  |  |  |  |
| GO:0010612\_regulation\_of\_cardiac\_muscle\_adaptation | 1 | 0 |  |  |  |  |  |  |  |  |
| GO:0010614\_negative\_regulation\_of\_cardiac\_muscle\_hypertrophy | 1 | 0 |  |  |  |  |  |  |  |  |
| GO:0010616\_negative\_regulation\_of\_cardiac\_muscle\_adaptation | 1 | 0 |  |  |  |  |  |  |  |  |
| GO:0010634\_positive\_regulation\_of\_epithelial\_cell\_migration | 1 | 0 |  |  |  |  |  |  |  |  |
| GO:0010656\_negative\_regulation\_of\_muscle\_cell\_apoptosis | 1 | 0 |  |  |  |  |  |  |  |  |
| GO:0010657\_muscle\_cell\_apoptosis | 1 | 0 |  |  |  |  |  |  |  |  |
| GO:0010658\_striated\_muscle\_cell\_apoptosis | 1 | 0 |  |  |  |  |  |  |  |  |
| GO:0010659\_cardiac\_muscle\_cell\_apoptosis | 1 | 0 |  |  |  |  |  |  |  |  |
| GO:0010660\_regulation\_of\_muscle\_cell\_apoptosis | 1 | 0 |  |  |  |  |  |  |  |  |
| GO:0010662\_regulation\_of\_striated\_muscle\_cell\_apoptosis | 1 | 0 |  |  |  |  |  |  |  |  |
| GO:0010664\_negative\_regulation\_of\_striated\_muscle\_cell\_apoptosis | 1 | 0 |  |  |  |  |  |  |  |  |
| GO:0010665\_regulation\_of\_cardiac\_muscle\_cell\_apoptosis | 1 | 0 |  |  |  |  |  |  |  |  |
| GO:0010667\_negative\_regulation\_of\_cardiac\_muscle\_cell\_apoptosis | 1 | 0 |  |  |  |  |  |  |  |  |
| GO:0010668\_ectodermal\_cell\_differentiation | 1 | 0 |  |  |  |  |  |  |  |  |
| GO:0010671\_negative\_regulation\_of\_oxygen\_and\_reactive\_oxygen\_species\_metabolic\_process | 1 | 0 |  |  |  |  |  |  |  |  |
| GO:0010719\_negative\_regulation\_of\_epithelial\_to\_mesenchymal\_transition | 1 | 0 |  |  |  |  |  |  |  |  |
| GO:0010735\_positive\_regulation\_of\_transcription\_via\_serum\_response\_element\_binding | 1 | 0 |  |  |  |  |  |  |  |  |
| GO:0010825\_positive\_regulation\_of\_centrosome\_duplication | 1 | 0 |  |  |  |  |  |  |  |  |
| GO:0010845\_positive\_regulation\_of\_reciprocal\_meiotic\_recombination | 1 | 0 |  |  |  |  |  |  |  |  |
| GO:0010850\_chemoreceptor\_signaling\_pathway\_involved\_in\_regulation\_of\_blood\_pressure | 1 | 0 |  |  |  |  |  |  |  |  |
| GO:0010880\_regulation\_of\_release\_of\_sequestered\_calcium\_ion\_into\_cytosol\_by\_sarcoplasmic\_reticulum | 1 | 0 |  |  |  |  |  |  |  |  |
| GO:0010881\_regulation\_of\_cardiac\_muscle\_contraction\_by\_regulation\_of\_the\_release\_of\_sequestered\_calcium\_ion | 1 | 0 |  |  |  |  |  |  |  |  |
| GO:0010882\_regulation\_of\_cardiac\_muscle\_contraction\_by\_calcium\_ion\_signaling | 1 | 0 |  |  |  |  |  |  |  |  |
| GO:0010890\_positive\_regulation\_of\_sequestering\_of\_triglyceride | 1 | 0 |  |  |  |  |  |  |  |  |
| GO:0010919\_regulation\_of\_inositol\_phosphate\_biosynthetic\_process | 1 | 0 |  |  |  |  |  |  |  |  |
| GO:0010931\_macrophage\_tolerance\_induction | 1 | 0 |  |  |  |  |  |  |  |  |
| GO:0010932\_regulation\_of\_macrophage\_tolerance\_induction | 1 | 0 |  |  |  |  |  |  |  |  |
| GO:0010933\_positive\_regulation\_of\_macrophage\_tolerance\_induction | 1 | 0 |  |  |  |  |  |  |  |  |
| GO:0010934\_macrophage\_cytokine\_production | 1 | 0 |  |  |  |  |  |  |  |  |
| GO:0010935\_regulation\_of\_macrophage\_cytokine\_production | 1 | 0 |  |  |  |  |  |  |  |  |
| GO:0010936\_negative\_regulation\_of\_macrophage\_cytokine\_production | 1 | 0 |  |  |  |  |  |  |  |  |
| GO:0010953\_regulation\_of\_protein\_maturation\_by\_peptide\_bond\_cleavage | 1 | 0 |  |  |  |  |  |  |  |  |
| GO:0010962\_regulation\_of\_glucan\_biosynthetic\_process | 1 | 0 |  |  |  |  |  |  |  |  |
| GO:0010966\_regulation\_of\_phosphate\_transport | 1 | 0 |  |  |  |  |  |  |  |  |
| GO:0014012\_axon\_regeneration\_in\_the\_peripheral\_nervous\_system | 1 | 0 |  |  |  |  |  |  |  |  |
| GO:0014016\_neuroblast\_differentiation | 1 | 0 |  |  |  |  |  |  |  |  |
| GO:0014017\_neuroblast\_fate\_commitment | 1 | 0 |  |  |  |  |  |  |  |  |
| GO:0014041\_regulation\_of\_neuron\_maturation | 1 | 0 |  |  |  |  |  |  |  |  |
| GO:0014042\_positive\_regulation\_of\_neuron\_maturation | 1 | 0 |  |  |  |  |  |  |  |  |
| GO:0014049\_positive\_regulation\_of\_glutamate\_secretion | 1 | 0 |  |  |  |  |  |  |  |  |
| GO:0014061\_regulation\_of\_norepinephrine\_secretion | 1 | 0 |  |  |  |  |  |  |  |  |
| GO:0014071\_response\_to\_cycloalkane | 1 | 0 |  |  |  |  |  |  |  |  |
| GO:0014707\_branchiomeric\_skeletal\_muscle\_development | 1 | 0 |  |  |  |  |  |  |  |  |
| GO:0014738\_regulation\_of\_muscle\_hyperplasia | 1 | 0 |  |  |  |  |  |  |  |  |
| GO:0014740\_negative\_regulation\_of\_muscle\_hyperplasia | 1 | 0 |  |  |  |  |  |  |  |  |
| GO:0014741\_negative\_regulation\_of\_muscle\_hypertrophy | 1 | 0 |  |  |  |  |  |  |  |  |
| GO:0014743\_regulation\_of\_muscle\_hypertrophy | 1 | 0 |  |  |  |  |  |  |  |  |
| GO:0014805\_smooth\_muscle\_adaptation | 1 | 0 |  |  |  |  |  |  |  |  |
| GO:0014806\_smooth\_muscle\_hyperplasia | 1 | 0 |  |  |  |  |  |  |  |  |
| GO:0014807\_regulation\_of\_somitogenesis | 1 | 0 |  |  |  |  |  |  |  |  |
| GO:0014808\_release\_of\_sequestered\_calcium\_ion\_into\_cytosol\_by\_sarcoplasmic\_reticulum | 1 | 0 |  |  |  |  |  |  |  |  |
| GO:0014813\_satellite\_cell\_commitment | 1 | 0 |  |  |  |  |  |  |  |  |
| GO:0014816\_satellite\_cell\_differentiation | 1 | 0 |  |  |  |  |  |  |  |  |
| GO:0014819\_regulation\_of\_skeletal\_muscle\_contraction | 1 | 0 |  |  |  |  |  |  |  |  |
| GO:0014852\_regulation\_of\_skeletal\_muscle\_contraction\_by\_neural\_stimulation\_via\_neuromuscular\_junction | 1 | 0 |  |  |  |  |  |  |  |  |
| GO:0014853\_regulation\_of\_excitatory\_postsynaptic\_membrane\_potential\_involved\_in\_skeletal\_muscle\_contraction | 1 | 0 |  |  |  |  |  |  |  |  |
| GO:0014856\_skeletal\_muscle\_cell\_proliferation | 1 | 0 |  |  |  |  |  |  |  |  |
| GO:0014857\_regulation\_of\_skeletal\_muscle\_cell\_proliferation | 1 | 0 |  |  |  |  |  |  |  |  |
| GO:0014858\_positive\_regulation\_of\_skeletal\_muscle\_cell\_proliferation | 1 | 0 |  |  |  |  |  |  |  |  |
| GO:0014887\_cardiac\_muscle\_adaptation | 1 | 0 |  |  |  |  |  |  |  |  |
| GO:0014889\_muscle\_atrophy | 1 | 0 |  |  |  |  |  |  |  |  |
| GO:0014896\_muscle\_hypertrophy | 1 | 0 |  |  |  |  |  |  |  |  |
| GO:0014897\_striated\_muscle\_hypertrophy | 1 | 0 |  |  |  |  |  |  |  |  |
| GO:0014898\_cardiac\_muscle\_hypertrophy | 1 | 0 |  |  |  |  |  |  |  |  |
| GO:0014900\_muscle\_hyperplasia | 1 | 0 |  |  |  |  |  |  |  |  |
| GO:0014910\_regulation\_of\_smooth\_muscle\_cell\_migration | 1 | 0 |  |  |  |  |  |  |  |  |
| GO:0014911\_positive\_regulation\_of\_smooth\_muscle\_cell\_migration | 1 | 0 |  |  |  |  |  |  |  |  |
| GO:0015014\_heparan\_sulfate\_proteoglycan\_biosynthetic\_process\_\_polysaccharide\_chain\_biosynthetic\_process | 1 | 0 |  |  |  |  |  |  |  |  |
| GO:0015074\_DNA\_integration | 1 | 0 |  |  |  |  |  |  |  |  |
| GO:0015670\_carbon\_dioxide\_transport | 1 | 0 |  |  |  |  |  |  |  |  |
| GO:0015677\_copper\_ion\_import | 1 | 0 |  |  |  |  |  |  |  |  |
| GO:0015680\_intracellular\_copper\_ion\_transport | 1 | 0 |  |  |  |  |  |  |  |  |
| GO:0015684\_ferrous\_iron\_transport | 1 | 0 |  |  |  |  |  |  |  |  |
| GO:0015707\_nitrite\_transport | 1 | 0 |  |  |  |  |  |  |  |  |
| GO:0015724\_formate\_transport | 1 | 0 |  |  |  |  |  |  |  |  |
| GO:0015734\_taurine\_transport | 1 | 0 |  |  |  |  |  |  |  |  |
| GO:0015740\_C4-dicarboxylate\_transport | 1 | 0 |  |  |  |  |  |  |  |  |
| GO:0015744\_succinate\_transport | 1 | 0 |  |  |  |  |  |  |  |  |
| GO:0015746\_citrate\_transport | 1 | 0 |  |  |  |  |  |  |  |  |
| GO:0015747\_urate\_transport | 1 | 0 |  |  |  |  |  |  |  |  |
| GO:0015791\_polyol\_transport | 1 | 0 |  |  |  |  |  |  |  |  |
| GO:0015798\_myo-inositol\_transport | 1 | 0 |  |  |  |  |  |  |  |  |
| GO:0015808\_L-alanine\_transport | 1 | 0 |  |  |  |  |  |  |  |  |
| GO:0015810\_aspartate\_transport | 1 | 0 |  |  |  |  |  |  |  |  |
| GO:0015811\_L-cystine\_transport | 1 | 0 |  |  |  |  |  |  |  |  |
| GO:0015817\_histidine\_transport | 1 | 0 |  |  |  |  |  |  |  |  |
| GO:0015822\_ornithine\_transport | 1 | 0 |  |  |  |  |  |  |  |  |
| GO:0015824\_proline\_transport | 1 | 0 |  |  |  |  |  |  |  |  |
| GO:0015851\_nucleobase\_transport | 1 | 0 |  |  |  |  |  |  |  |  |
| GO:0015864\_pyrimidine\_nucleoside\_transport | 1 | 0 |  |  |  |  |  |  |  |  |
| GO:0015874\_norepinephrine\_transport | 1 | 0 |  |  |  |  |  |  |  |  |
| GO:0015881\_creatine\_transport | 1 | 0 |  |  |  |  |  |  |  |  |
| GO:0015884\_folic\_acid\_transport | 1 | 0 |  |  |  |  |  |  |  |  |
| GO:0015886\_heme\_transport | 1 | 0 |  |  |  |  |  |  |  |  |
| GO:0015888\_thiamin\_transport | 1 | 0 |  |  |  |  |  |  |  |  |
| GO:0015938\_coenzyme\_A\_catabolic\_process | 1 | 0 |  |  |  |  |  |  |  |  |
| GO:0015939\_pantothenate\_metabolic\_process | 1 | 0 |  |  |  |  |  |  |  |  |
| GO:0016073\_snRNA\_metabolic\_process | 1 | 0 |  |  |  |  |  |  |  |  |
| GO:0016074\_snoRNA\_metabolic\_process | 1 | 0 |  |  |  |  |  |  |  |  |
| GO:0016082\_synaptic\_vesicle\_priming | 1 | 0 |  |  |  |  |  |  |  |  |
| GO:0016090\_prenol\_metabolic\_process | 1 | 0 |  |  |  |  |  |  |  |  |
| GO:0016093\_polyprenol\_metabolic\_process | 1 | 0 |  |  |  |  |  |  |  |  |
| GO:0016180\_snRNA\_processing | 1 | 0 |  |  |  |  |  |  |  |  |
| GO:0016239\_positive\_regulation\_of\_macroautophagy | 1 | 0 |  |  |  |  |  |  |  |  |
| GO:0016246\_RNA\_interference | 1 | 0 |  |  |  |  |  |  |  |  |
| GO:0016255\_attachment\_of\_GPI\_anchor\_to\_protein | 1 | 0 |  |  |  |  |  |  |  |  |
| GO:0016333\_morphogenesis\_of\_follicular\_epithelium | 1 | 0 |  |  |  |  |  |  |  |  |
| GO:0016340\_calcium-dependent\_cell-matrix\_adhesion | 1 | 0 |  |  |  |  |  |  |  |  |
| GO:0016344\_meiotic\_chromosome\_movement\_towards\_spindle\_pole | 1 | 0 |  |  |  |  |  |  |  |  |
| GO:0016482\_cytoplasmic\_transport | 1 | 0 |  |  |  |  |  |  |  |  |
| GO:0016553\_base\_conversion\_or\_substitution\_editing | 1 | 0 |  |  |  |  |  |  |  |  |
| GO:0016554\_cytidine\_to\_uridine\_editing | 1 | 0 |  |  |  |  |  |  |  |  |
| GO:0016560\_protein\_import\_into\_peroxisome\_matrix\_\_docking | 1 | 0 |  |  |  |  |  |  |  |  |
| GO:0016578\_histone\_deubiquitination | 1 | 0 |  |  |  |  |  |  |  |  |
| GO:0016598\_protein\_arginylation | 1 | 0 |  |  |  |  |  |  |  |  |
| GO:0017004\_cytochrome\_complex\_assembly | 1 | 0 |  |  |  |  |  |  |  |  |
| GO:0018022\_peptidyl-lysine\_methylation | 1 | 0 |  |  |  |  |  |  |  |  |
| GO:0018023\_peptidyl-lysine\_trimethylation | 1 | 0 |  |  |  |  |  |  |  |  |
| GO:0018120\_peptidyl-arginine\_ADP-ribosylation | 1 | 0 |  |  |  |  |  |  |  |  |
| GO:0018126\_protein\_amino\_acid\_hydroxylation | 1 | 0 |  |  |  |  |  |  |  |  |
| GO:0018146\_keratan\_sulfate\_biosynthetic\_process | 1 | 0 |  |  |  |  |  |  |  |  |
| GO:0018158\_protein\_amino\_acid\_oxidation | 1 | 0 |  |  |  |  |  |  |  |  |
| GO:0018195\_peptidyl-arginine\_modification | 1 | 0 |  |  |  |  |  |  |  |  |
| GO:0018197\_peptidyl-aspartic\_acid\_modification | 1 | 0 |  |  |  |  |  |  |  |  |
| GO:0018282\_metal\_incorporation\_into\_metallo-sulfur\_cluster | 1 | 0 |  |  |  |  |  |  |  |  |
| GO:0018283\_iron\_incorporation\_into\_metallo-sulfur\_cluster | 1 | 0 |  |  |  |  |  |  |  |  |
| GO:0018318\_protein\_amino\_acid\_palmitoylation | 1 | 0 |  |  |  |  |  |  |  |  |
| GO:0018342\_protein\_prenylation | 1 | 0 |  |  |  |  |  |  |  |  |
| GO:0018344\_protein\_geranylgeranylation | 1 | 0 |  |  |  |  |  |  |  |  |
| GO:0018410\_peptide\_or\_protein\_carboxyl-terminal\_blocking | 1 | 0 |  |  |  |  |  |  |  |  |
| GO:0018916\_nitrobenzene\_metabolic\_process | 1 | 0 |  |  |  |  |  |  |  |  |
| GO:0018931\_naphthalene\_metabolic\_process | 1 | 0 |  |  |  |  |  |  |  |  |
| GO:0018992\_germ-line\_sex\_determination | 1 | 0 |  |  |  |  |  |  |  |  |
| GO:0019042\_latent\_virus\_infection | 1 | 0 |  |  |  |  |  |  |  |  |
| GO:0019046\_reactivation\_of\_latent\_virus | 1 | 0 |  |  |  |  |  |  |  |  |
| GO:0019047\_provirus\_integration | 1 | 0 |  |  |  |  |  |  |  |  |
| GO:0019076\_release\_of\_virus\_from\_host | 1 | 0 |  |  |  |  |  |  |  |  |
| GO:0019079\_viral\_genome\_replication | 1 | 0 |  |  |  |  |  |  |  |  |
| GO:0019100\_male\_germ-line\_sex\_determination | 1 | 0 |  |  |  |  |  |  |  |  |
| GO:0019101\_female\_somatic\_sex\_determination | 1 | 0 |  |  |  |  |  |  |  |  |
| GO:0019102\_male\_somatic\_sex\_determination | 1 | 0 |  |  |  |  |  |  |  |  |
| GO:0019255\_glucose\_1-phosphate\_metabolic\_process | 1 | 0 |  |  |  |  |  |  |  |  |
| GO:0019276\_UDP-N-acetylgalactosamine\_metabolic\_process | 1 | 0 |  |  |  |  |  |  |  |  |
| GO:0019344\_cysteine\_biosynthetic\_process | 1 | 0 |  |  |  |  |  |  |  |  |
| GO:0019348\_dolichol\_metabolic\_process | 1 | 0 |  |  |  |  |  |  |  |  |
| GO:0019375\_galactolipid\_biosynthetic\_process | 1 | 0 |  |  |  |  |  |  |  |  |
| GO:0019402\_galactitol\_metabolic\_process | 1 | 0 |  |  |  |  |  |  |  |  |
| GO:0019441\_tryptophan\_catabolic\_process\_to\_kynurenine | 1 | 0 |  |  |  |  |  |  |  |  |
| GO:0019477\_L-lysine\_catabolic\_process | 1 | 0 |  |  |  |  |  |  |  |  |
| GO:0019510\_S-adenosylhomocysteine\_catabolic\_process | 1 | 0 |  |  |  |  |  |  |  |  |
| GO:0019532\_oxalate\_transport | 1 | 0 |  |  |  |  |  |  |  |  |
| GO:0019626\_short-chain\_fatty\_acid\_catabolic\_process | 1 | 0 |  |  |  |  |  |  |  |  |
| GO:0019627\_urea\_metabolic\_process | 1 | 0 |  |  |  |  |  |  |  |  |
| GO:0019676\_ammonia\_assimilation\_cycle | 1 | 0 |  |  |  |  |  |  |  |  |
| GO:0019682\_glyceraldehyde-3-phosphate\_metabolic\_process | 1 | 0 |  |  |  |  |  |  |  |  |
| GO:0019695\_choline\_metabolic\_process | 1 | 0 |  |  |  |  |  |  |  |  |
| GO:0019731\_antibacterial\_humoral\_response | 1 | 0 |  |  |  |  |  |  |  |  |
| GO:0019794\_nonprotein\_amino\_acid\_metabolic\_process | 1 | 0 |  |  |  |  |  |  |  |  |
| GO:0019858\_cytosine\_metabolic\_process | 1 | 0 |  |  |  |  |  |  |  |  |
| GO:0019883\_antigen\_processing\_and\_presentation\_of\_endogenous\_antigen | 1 | 0 |  |  |  |  |  |  |  |  |
| GO:0019889\_pteridine\_metabolic\_process | 1 | 0 |  |  |  |  |  |  |  |  |
| GO:0019896\_axon\_transport\_of\_mitochondrion | 1 | 0 |  |  |  |  |  |  |  |  |
| GO:0021508\_floor\_plate\_formation | 1 | 0 |  |  |  |  |  |  |  |  |
| GO:0021528\_commissural\_neuron\_differentiation\_in\_the\_spinal\_cord | 1 | 0 |  |  |  |  |  |  |  |  |
| GO:0021572\_rhombomere\_6\_development | 1 | 0 |  |  |  |  |  |  |  |  |
| GO:0021577\_hindbrain\_structural\_organization | 1 | 0 |  |  |  |  |  |  |  |  |
| GO:0021586\_pons\_maturation | 1 | 0 |  |  |  |  |  |  |  |  |
| GO:0021589\_cerebellum\_structural\_organization | 1 | 0 |  |  |  |  |  |  |  |  |
| GO:0021590\_cerebellum\_maturation | 1 | 0 |  |  |  |  |  |  |  |  |
| GO:0021592\_fourth\_ventricle\_development | 1 | 0 |  |  |  |  |  |  |  |  |
| GO:0021594\_rhombomere\_formation | 1 | 0 |  |  |  |  |  |  |  |  |
| GO:0021660\_rhombomere\_3\_formation | 1 | 0 |  |  |  |  |  |  |  |  |
| GO:0021664\_rhombomere\_5\_morphogenesis | 1 | 0 |  |  |  |  |  |  |  |  |
| GO:0021666\_rhombomere\_5\_formation | 1 | 0 |  |  |  |  |  |  |  |  |
| GO:0021670\_lateral\_ventricle\_development | 1 | 0 |  |  |  |  |  |  |  |  |
| GO:0021678\_third\_ventricle\_development | 1 | 0 |  |  |  |  |  |  |  |  |
| GO:0021679\_cerebellar\_molecular\_layer\_development | 1 | 0 |  |  |  |  |  |  |  |  |
| GO:0021703\_locus\_ceruleus\_development | 1 | 0 |  |  |  |  |  |  |  |  |
| GO:0021732\_midbrain-hindbrain\_boundary\_maturation | 1 | 0 |  |  |  |  |  |  |  |  |
| GO:0021747\_cochlear\_nucleus\_development | 1 | 0 |  |  |  |  |  |  |  |  |
| GO:0021750\_vestibular\_nucleus\_development | 1 | 0 |  |  |  |  |  |  |  |  |
| GO:0021759\_globus\_pallidus\_development | 1 | 0 |  |  |  |  |  |  |  |  |
| GO:0021768\_nucleus\_accumbens\_development | 1 | 0 |  |  |  |  |  |  |  |  |
| GO:0021771\_lateral\_geniculate\_nucleus\_development | 1 | 0 |  |  |  |  |  |  |  |  |
| GO:0021812\_neuronal-glial\_interaction\_involved\_in\_cerebral\_cortex\_radial\_glia\_guided\_migration | 1 | 0 |  |  |  |  |  |  |  |  |
| GO:0021813\_cell-cell\_adhesion\_involved\_in\_neuronal-glial\_interactions\_involved\_in\_cerebral\_cortex\_radial\_glia\_guided\_migration | 1 | 0 |  |  |  |  |  |  |  |  |
| GO:0021870\_Cajal-Retzius\_cell\_differentiation | 1 | 0 |  |  |  |  |  |  |  |  |
| GO:0021874\_Wnt\_receptor\_signaling\_pathway\_in\_forebrain\_neuroblast\_division | 1 | 0 |  |  |  |  |  |  |  |  |
| GO:0021896\_forebrain\_astrocyte\_differentiation | 1 | 0 |  |  |  |  |  |  |  |  |
| GO:0021897\_forebrain\_astrocyte\_development | 1 | 0 |  |  |  |  |  |  |  |  |
| GO:0021902\_commitment\_of\_a\_neuronal\_cell\_to\_a\_specific\_type\_of\_neuron\_in\_the\_forebrain | 1 | 0 |  |  |  |  |  |  |  |  |
| GO:0021905\_forebrain-midbrain\_boundary\_formation | 1 | 0 |  |  |  |  |  |  |  |  |
| GO:0021914\_negative\_regulation\_of\_smoothened\_signaling\_pathway\_involved\_in\_ventral\_spinal\_cord\_patterning | 1 | 0 |  |  |  |  |  |  |  |  |
| GO:0021917\_somatic\_motor\_neuron\_fate\_commitment | 1 | 0 |  |  |  |  |  |  |  |  |
| GO:0021918\_regulation\_of\_transcription\_from\_RNA\_polymerase\_II\_promoter\_involved\_in\_somatic\_motor\_neuron\_fate\_commitment | 1 | 0 |  |  |  |  |  |  |  |  |
| GO:0021933\_radial\_glia\_guided\_migration\_of\_granule\_cell | 1 | 0 |  |  |  |  |  |  |  |  |
| GO:0021934\_hindbrain\_tangential\_cell\_migration | 1 | 0 |  |  |  |  |  |  |  |  |
| GO:0021935\_granule\_cell\_precursor\_tangential\_migration | 1 | 0 |  |  |  |  |  |  |  |  |
| GO:0021942\_radial\_glia\_guided\_migration\_of\_Purkinje\_cell | 1 | 0 |  |  |  |  |  |  |  |  |
| GO:0021960\_anterior\_commissure\_morphogenesis | 1 | 0 |  |  |  |  |  |  |  |  |
| GO:0021997\_neural\_plate\_axis\_specification | 1 | 0 |  |  |  |  |  |  |  |  |
| GO:0021999\_neural\_plate\_anterior\_posterior\_pattern\_formation | 1 | 0 |  |  |  |  |  |  |  |  |
| GO:0022004\_midbrain-hindbrain\_boundary\_maturation\_during\_brain\_development | 1 | 0 |  |  |  |  |  |  |  |  |
| GO:0022038\_corpus\_callosum\_development | 1 | 0 |  |  |  |  |  |  |  |  |
| GO:0022605\_oogenesis\_stage | 1 | 0 |  |  |  |  |  |  |  |  |
| GO:0030011\_maintenance\_of\_cell\_polarity | 1 | 0 |  |  |  |  |  |  |  |  |
| GO:0030069\_lysogeny | 1 | 0 |  |  |  |  |  |  |  |  |
| GO:0030070\_insulin\_processing | 1 | 0 |  |  |  |  |  |  |  |  |
| GO:0030092\_regulation\_of\_flagellum\_assembly | 1 | 0 |  |  |  |  |  |  |  |  |
| GO:0030103\_vasopressin\_secretion | 1 | 0 |  |  |  |  |  |  |  |  |
| GO:0030194\_positive\_regulation\_of\_blood\_coagulation | 1 | 0 |  |  |  |  |  |  |  |  |
| GO:0030206\_chondroitin\_sulfate\_biosynthetic\_process | 1 | 0 |  |  |  |  |  |  |  |  |
| GO:0030210\_heparin\_biosynthetic\_process | 1 | 0 |  |  |  |  |  |  |  |  |
| GO:0030220\_platelet\_formation | 1 | 0 |  |  |  |  |  |  |  |  |
| GO:0030222\_eosinophil\_differentiation | 1 | 0 |  |  |  |  |  |  |  |  |
| GO:0030237\_female\_sex\_determination | 1 | 0 |  |  |  |  |  |  |  |  |
| GO:0030264\_nuclear\_fragmentation\_during\_apoptosis | 1 | 0 |  |  |  |  |  |  |  |  |
| GO:0030322\_stabilization\_of\_membrane\_potential | 1 | 0 |  |  |  |  |  |  |  |  |
| GO:0030327\_prenylated\_protein\_catabolic\_process | 1 | 0 |  |  |  |  |  |  |  |  |
| GO:0030328\_prenylcysteine\_catabolic\_process | 1 | 0 |  |  |  |  |  |  |  |  |
| GO:0030329\_prenylcysteine\_metabolic\_process | 1 | 0 |  |  |  |  |  |  |  |  |
| GO:0030389\_fructosamine\_metabolic\_process | 1 | 0 |  |  |  |  |  |  |  |  |
| GO:0030422\_RNA\_interference\_\_production\_of\_siRNA | 1 | 0 |  |  |  |  |  |  |  |  |
| GO:0030449\_regulation\_of\_complement\_activation | 1 | 0 |  |  |  |  |  |  |  |  |
| GO:0030497\_fatty\_acid\_elongation | 1 | 0 |  |  |  |  |  |  |  |  |
| GO:0030575\_nuclear\_body\_organization | 1 | 0 |  |  |  |  |  |  |  |  |
| GO:0030578\_PML\_body\_organization | 1 | 0 |  |  |  |  |  |  |  |  |
| GO:0030853\_negative\_regulation\_of\_granulocyte\_differentiation | 1 | 0 |  |  |  |  |  |  |  |  |
| GO:0030854\_positive\_regulation\_of\_granulocyte\_differentiation | 1 | 0 |  |  |  |  |  |  |  |  |
| GO:0030886\_negative\_regulation\_of\_myeloid\_dendritic\_cell\_activation | 1 | 0 |  |  |  |  |  |  |  |  |
| GO:0030913\_paranodal\_junction\_assembly | 1 | 0 |  |  |  |  |  |  |  |  |
| GO:0031033\_myosin\_filament\_assembly\_or\_disassembly | 1 | 0 |  |  |  |  |  |  |  |  |
| GO:0031034\_myosin\_filament\_assembly | 1 | 0 |  |  |  |  |  |  |  |  |
| GO:0031055\_chromatin\_remodeling\_at\_centromere | 1 | 0 |  |  |  |  |  |  |  |  |
| GO:0031062\_positive\_regulation\_of\_histone\_methylation | 1 | 0 |  |  |  |  |  |  |  |  |
| GO:0031115\_negative\_regulation\_of\_microtubule\_polymerization | 1 | 0 |  |  |  |  |  |  |  |  |
| GO:0031129\_inductive\_cell-cell\_signaling | 1 | 0 |  |  |  |  |  |  |  |  |
| GO:0031284\_positive\_regulation\_of\_guanylate\_cyclase\_activity | 1 | 0 |  |  |  |  |  |  |  |  |
| GO:0031498\_chromatin\_disassembly | 1 | 0 |  |  |  |  |  |  |  |  |
| GO:0031507\_heterochromatin\_formation | 1 | 0 |  |  |  |  |  |  |  |  |
| GO:0031508\_centromeric\_heterochromatin\_formation | 1 | 0 |  |  |  |  |  |  |  |  |
| GO:0031529\_ruffle\_organization | 1 | 0 |  |  |  |  |  |  |  |  |
| GO:0031536\_positive\_regulation\_of\_exit\_from\_mitosis | 1 | 0 |  |  |  |  |  |  |  |  |
| GO:0031572\_G2\_M\_transition\_DNA\_damage\_checkpoint | 1 | 0 |  |  |  |  |  |  |  |  |
| GO:0031576\_G2\_M\_transition\_checkpoint | 1 | 0 |  |  |  |  |  |  |  |  |
| GO:0031580\_membrane\_raft\_distribution | 1 | 0 |  |  |  |  |  |  |  |  |
| GO:0031583\_activation\_of\_phospholipase\_D\_activity\_by\_G-protein\_coupled\_receptor\_protein\_signaling\_pathway | 1 | 0 |  |  |  |  |  |  |  |  |
| GO:0031584\_activation\_of\_phospholipase\_D\_activity | 1 | 0 |  |  |  |  |  |  |  |  |
| GO:0031585\_regulation\_of\_inositol-1\_4\_5-triphosphate\_receptor\_activity | 1 | 0 |  |  |  |  |  |  |  |  |
| GO:0031639\_plasminogen\_activation | 1 | 0 |  |  |  |  |  |  |  |  |
| GO:0031648\_protein\_destabilization | 1 | 0 |  |  |  |  |  |  |  |  |
| GO:0031665\_negative\_regulation\_of\_lipopolysaccharide-mediated\_signaling\_pathway | 1 | 0 |  |  |  |  |  |  |  |  |
| GO:0031914\_negative\_regulation\_of\_synaptic\_plasticity | 1 | 0 |  |  |  |  |  |  |  |  |
| GO:0031944\_negative\_regulation\_of\_glucocorticoid\_metabolic\_process | 1 | 0 |  |  |  |  |  |  |  |  |
| GO:0031947\_negative\_regulation\_of\_glucocorticoid\_biosynthetic\_process | 1 | 0 |  |  |  |  |  |  |  |  |
| GO:0032025\_response\_to\_cobalt\_ion | 1 | 0 |  |  |  |  |  |  |  |  |
| GO:0032026\_response\_to\_magnesium\_ion | 1 | 0 |  |  |  |  |  |  |  |  |
| GO:0032048\_cardiolipin\_metabolic\_process | 1 | 0 |  |  |  |  |  |  |  |  |
| GO:0032066\_nucleolus\_to\_nucleoplasm\_transport | 1 | 0 |  |  |  |  |  |  |  |  |
| GO:0032091\_negative\_regulation\_of\_protein\_binding | 1 | 0 |  |  |  |  |  |  |  |  |
| GO:0032092\_positive\_regulation\_of\_protein\_binding | 1 | 0 |  |  |  |  |  |  |  |  |
| GO:0032097\_positive\_regulation\_of\_response\_to\_food | 1 | 0 |  |  |  |  |  |  |  |  |
| GO:0032100\_positive\_regulation\_of\_appetite | 1 | 0 |  |  |  |  |  |  |  |  |
| GO:0032204\_regulation\_of\_telomere\_maintenance | 1 | 0 |  |  |  |  |  |  |  |  |
| GO:0032206\_positive\_regulation\_of\_telomere\_maintenance | 1 | 0 |  |  |  |  |  |  |  |  |
| GO:0032222\_regulation\_of\_synaptic\_transmission\_\_cholinergic | 1 | 0 |  |  |  |  |  |  |  |  |
| GO:0032224\_positive\_regulation\_of\_synaptic\_transmission\_\_cholinergic | 1 | 0 |  |  |  |  |  |  |  |  |
| GO:0032229\_negative\_regulation\_of\_synaptic\_transmission\_\_GABAergic | 1 | 0 |  |  |  |  |  |  |  |  |
| GO:0032237\_activation\_of\_store-operated\_calcium\_channel\_activity | 1 | 0 |  |  |  |  |  |  |  |  |
| GO:0032239\_regulation\_of\_nucleobase\_\_nucleoside\_\_nucleotide\_and\_nucleic\_acid\_transport | 1 | 0 |  |  |  |  |  |  |  |  |
| GO:0032252\_secretory\_granule\_localization | 1 | 0 |  |  |  |  |  |  |  |  |
| GO:0032274\_gonadotropin\_secretion | 1 | 0 |  |  |  |  |  |  |  |  |
| GO:0032275\_luteinizing\_hormone\_secretion | 1 | 0 |  |  |  |  |  |  |  |  |
| GO:0032287\_myelin\_maintenance\_in\_the\_peripheral\_nervous\_system | 1 | 0 |  |  |  |  |  |  |  |  |
| GO:0032289\_myelin\_formation\_in\_the\_central\_nervous\_system | 1 | 0 |  |  |  |  |  |  |  |  |
| GO:0032303\_regulation\_of\_icosanoid\_secretion | 1 | 0 |  |  |  |  |  |  |  |  |
| GO:0032305\_positive\_regulation\_of\_icosanoid\_secretion | 1 | 0 |  |  |  |  |  |  |  |  |
| GO:0032306\_regulation\_of\_prostaglandin\_secretion | 1 | 0 |  |  |  |  |  |  |  |  |
| GO:0032308\_positive\_regulation\_of\_prostaglandin\_secretion | 1 | 0 |  |  |  |  |  |  |  |  |
| GO:0032310\_prostaglandin\_secretion | 1 | 0 |  |  |  |  |  |  |  |  |
| GO:0032313\_regulation\_of\_Rab\_GTPase\_activity | 1 | 0 |  |  |  |  |  |  |  |  |
| GO:0032314\_regulation\_of\_Rac\_GTPase\_activity | 1 | 0 |  |  |  |  |  |  |  |  |
| GO:0032317\_regulation\_of\_Rap\_GTPase\_activity | 1 | 0 |  |  |  |  |  |  |  |  |
| GO:0032324\_molybdopterin\_cofactor\_biosynthetic\_process | 1 | 0 |  |  |  |  |  |  |  |  |
| GO:0032329\_serine\_transport | 1 | 0 |  |  |  |  |  |  |  |  |
| GO:0032342\_aldosterone\_biosynthetic\_process | 1 | 0 |  |  |  |  |  |  |  |  |
| GO:0032344\_regulation\_of\_aldosterone\_metabolic\_process | 1 | 0 |  |  |  |  |  |  |  |  |
| GO:0032365\_intracellular\_lipid\_transport | 1 | 0 |  |  |  |  |  |  |  |  |
| GO:0032366\_intracellular\_sterol\_transport | 1 | 0 |  |  |  |  |  |  |  |  |
| GO:0032367\_intracellular\_cholesterol\_transport | 1 | 0 |  |  |  |  |  |  |  |  |
| GO:0032370\_positive\_regulation\_of\_lipid\_transport | 1 | 0 |  |  |  |  |  |  |  |  |
| GO:0032410\_negative\_regulation\_of\_transporter\_activity | 1 | 0 |  |  |  |  |  |  |  |  |
| GO:0032413\_negative\_regulation\_of\_ion\_transmembrane\_transporter\_activity | 1 | 0 |  |  |  |  |  |  |  |  |
| GO:0032429\_regulation\_of\_phospholipase\_A2\_activity | 1 | 0 |  |  |  |  |  |  |  |  |
| GO:0032474\_otolith\_morphogenesis | 1 | 0 |  |  |  |  |  |  |  |  |
| GO:0032482\_Rab\_protein\_signal\_transduction | 1 | 0 |  |  |  |  |  |  |  |  |
| GO:0032483\_regulation\_of\_Rab\_protein\_signal\_transduction | 1 | 0 |  |  |  |  |  |  |  |  |
| GO:0032486\_Rap\_protein\_signal\_transduction | 1 | 0 |  |  |  |  |  |  |  |  |
| GO:0032487\_regulation\_of\_Rap\_protein\_signal\_transduction | 1 | 0 |  |  |  |  |  |  |  |  |
| GO:0032594\_protein\_transport\_within\_lipid\_bilayer | 1 | 0 |  |  |  |  |  |  |  |  |
| GO:0032599\_protein\_transport\_out\_of\_membrane\_raft | 1 | 0 |  |  |  |  |  |  |  |  |
| GO:0032600\_chemokine\_receptor\_transport\_out\_of\_membrane\_raft | 1 | 0 |  |  |  |  |  |  |  |  |
| GO:0032607\_interferon-alpha\_production | 1 | 0 |  |  |  |  |  |  |  |  |
| GO:0032621\_interleukin-18\_production | 1 | 0 |  |  |  |  |  |  |  |  |
| GO:0032647\_regulation\_of\_interferon-alpha\_production | 1 | 0 |  |  |  |  |  |  |  |  |
| GO:0032656\_regulation\_of\_interleukin-13\_production | 1 | 0 |  |  |  |  |  |  |  |  |
| GO:0032682\_negative\_regulation\_of\_chemokine\_production | 1 | 0 |  |  |  |  |  |  |  |  |
| GO:0032691\_negative\_regulation\_of\_interleukin-1\_beta\_production | 1 | 0 |  |  |  |  |  |  |  |  |
| GO:0032692\_negative\_regulation\_of\_interleukin-1\_production | 1 | 0 |  |  |  |  |  |  |  |  |
| GO:0032693\_negative\_regulation\_of\_interleukin-10\_production | 1 | 0 |  |  |  |  |  |  |  |  |
| GO:0032696\_negative\_regulation\_of\_interleukin-13\_production | 1 | 0 |  |  |  |  |  |  |  |  |
| GO:0032727\_positive\_regulation\_of\_interferon-alpha\_production | 1 | 0 |  |  |  |  |  |  |  |  |
| GO:0032731\_positive\_regulation\_of\_interleukin-1\_beta\_production | 1 | 0 |  |  |  |  |  |  |  |  |
| GO:0032732\_positive\_regulation\_of\_interleukin-1\_production | 1 | 0 |  |  |  |  |  |  |  |  |
| GO:0032735\_positive\_regulation\_of\_interleukin-12\_production | 1 | 0 |  |  |  |  |  |  |  |  |
| GO:0032764\_negative\_regulation\_of\_mast\_cell\_cytokine\_production | 1 | 0 |  |  |  |  |  |  |  |  |
| GO:0032765\_positive\_regulation\_of\_mast\_cell\_cytokine\_production | 1 | 0 |  |  |  |  |  |  |  |  |
| GO:0032769\_negative\_regulation\_of\_monooxygenase\_activity | 1 | 0 |  |  |  |  |  |  |  |  |
| GO:0032781\_positive\_regulation\_of\_ATPase\_activity | 1 | 0 |  |  |  |  |  |  |  |  |
| GO:0032790\_ribosome\_disassembly | 1 | 0 |  |  |  |  |  |  |  |  |
| GO:0032799\_low-density\_lipoprotein\_receptor\_metabolic\_process | 1 | 0 |  |  |  |  |  |  |  |  |
| GO:0032802\_low-density\_lipoprotein\_receptor\_catabolic\_process | 1 | 0 |  |  |  |  |  |  |  |  |
| GO:0032803\_regulation\_of\_low-density\_lipoprotein\_receptor\_catabolic\_process | 1 | 0 |  |  |  |  |  |  |  |  |
| GO:0032817\_regulation\_of\_natural\_killer\_cell\_proliferation | 1 | 0 |  |  |  |  |  |  |  |  |
| GO:0032819\_positive\_regulation\_of\_natural\_killer\_cell\_proliferation | 1 | 0 |  |  |  |  |  |  |  |  |
| GO:0032836\_glomerular\_basement\_membrane\_development | 1 | 0 |  |  |  |  |  |  |  |  |
| GO:0032855\_positive\_regulation\_of\_Rac\_GTPase\_activity | 1 | 0 |  |  |  |  |  |  |  |  |
| GO:0032863\_activation\_of\_Rac\_GTPase\_activity | 1 | 0 |  |  |  |  |  |  |  |  |
| GO:0032864\_activation\_of\_Cdc42\_GTPase\_activity | 1 | 0 |  |  |  |  |  |  |  |  |
| GO:0032885\_regulation\_of\_polysaccharide\_biosynthetic\_process | 1 | 0 |  |  |  |  |  |  |  |  |
| GO:0032907\_transforming\_growth\_factor-beta3\_production | 1 | 0 |  |  |  |  |  |  |  |  |
| GO:0032910\_regulation\_of\_transforming\_growth\_factor-beta3\_production | 1 | 0 |  |  |  |  |  |  |  |  |
| GO:0032913\_negative\_regulation\_of\_transforming\_growth\_factor-beta3\_production | 1 | 0 |  |  |  |  |  |  |  |  |
| GO:0032924\_activin\_receptor\_signaling\_pathway | 1 | 0 |  |  |  |  |  |  |  |  |
| GO:0032925\_regulation\_of\_activin\_receptor\_signaling\_pathway | 1 | 0 |  |  |  |  |  |  |  |  |
| GO:0032960\_regulation\_of\_inositol\_trisphosphate\_biosynthetic\_process | 1 | 0 |  |  |  |  |  |  |  |  |
| GO:0032962\_positive\_regulation\_of\_inositol\_trisphosphate\_biosynthetic\_process | 1 | 0 |  |  |  |  |  |  |  |  |
| GO:0032964\_collagen\_biosynthetic\_process | 1 | 0 |  |  |  |  |  |  |  |  |
| GO:0032971\_regulation\_of\_muscle\_filament\_sliding | 1 | 0 |  |  |  |  |  |  |  |  |
| GO:0032972\_regulation\_of\_muscle\_filament\_sliding\_speed | 1 | 0 |  |  |  |  |  |  |  |  |
| GO:0032986\_protein-DNA\_complex\_disassembly | 1 | 0 |  |  |  |  |  |  |  |  |
| GO:0032988\_ribonucleoprotein\_complex\_disassembly | 1 | 0 |  |  |  |  |  |  |  |  |
| GO:0033037\_polysaccharide\_localization | 1 | 0 |  |  |  |  |  |  |  |  |
| GO:0033078\_extrathymic\_T\_cell\_differentiation | 1 | 0 |  |  |  |  |  |  |  |  |
| GO:0033085\_negative\_regulation\_of\_T\_cell\_differentiation\_in\_the\_thymus | 1 | 0 |  |  |  |  |  |  |  |  |
| GO:0033087\_negative\_regulation\_of\_immature\_T\_cell\_proliferation | 1 | 0 |  |  |  |  |  |  |  |  |
| GO:0033088\_negative\_regulation\_of\_immature\_T\_cell\_proliferation\_in\_the\_thymus | 1 | 0 |  |  |  |  |  |  |  |  |
| GO:0033108\_mitochondrial\_respiratory\_chain\_complex\_assembly | 1 | 0 |  |  |  |  |  |  |  |  |
| GO:0033127\_regulation\_of\_histone\_phosphorylation | 1 | 0 |  |  |  |  |  |  |  |  |
| GO:0033128\_negative\_regulation\_of\_histone\_phosphorylation | 1 | 0 |  |  |  |  |  |  |  |  |
| GO:0033138\_positive\_regulation\_of\_peptidyl-serine\_phosphorylation | 1 | 0 |  |  |  |  |  |  |  |  |
| GO:0033158\_regulation\_of\_protein\_import\_into\_nucleus\_\_translocation | 1 | 0 |  |  |  |  |  |  |  |  |
| GO:0033160\_positive\_regulation\_of\_protein\_import\_into\_nucleus\_\_translocation | 1 | 0 |  |  |  |  |  |  |  |  |
| GO:0033169\_histone\_H3-K9\_demethylation | 1 | 0 |  |  |  |  |  |  |  |  |
| GO:0033206\_cytokinesis\_after\_meiosis | 1 | 0 |  |  |  |  |  |  |  |  |
| GO:0033240\_positive\_regulation\_of\_cellular\_amine\_metabolic\_process | 1 | 0 |  |  |  |  |  |  |  |  |
| GO:0033313\_meiotic\_cell\_cycle\_checkpoint | 1 | 0 |  |  |  |  |  |  |  |  |
| GO:0033315\_meiotic\_cell\_cycle\_DNA\_replication\_checkpoint | 1 | 0 |  |  |  |  |  |  |  |  |
| GO:0033326\_cerebrospinal\_fluid\_secretion | 1 | 0 |  |  |  |  |  |  |  |  |
| GO:0033366\_protein\_localization\_in\_secretory\_granule | 1 | 0 |  |  |  |  |  |  |  |  |
| GO:0033367\_protein\_localization\_in\_mast\_cell\_secretory\_granule | 1 | 0 |  |  |  |  |  |  |  |  |
| GO:0033368\_protease\_localization\_in\_mast\_cell\_secretory\_granule | 1 | 0 |  |  |  |  |  |  |  |  |
| GO:0033370\_maintenance\_of\_protein\_location\_in\_mast\_cell\_secretory\_granule | 1 | 0 |  |  |  |  |  |  |  |  |
| GO:0033371\_T\_cell\_secretory\_granule\_organization | 1 | 0 |  |  |  |  |  |  |  |  |
| GO:0033373\_maintenance\_of\_protease\_location\_in\_mast\_cell\_secretory\_granule | 1 | 0 |  |  |  |  |  |  |  |  |
| GO:0033374\_protein\_localization\_in\_T\_cell\_secretory\_granule | 1 | 0 |  |  |  |  |  |  |  |  |
| GO:0033375\_protease\_localization\_in\_T\_cell\_secretory\_granule | 1 | 0 |  |  |  |  |  |  |  |  |
| GO:0033377\_maintenance\_of\_protein\_location\_in\_T\_cell\_secretory\_granule | 1 | 0 |  |  |  |  |  |  |  |  |
| GO:0033379\_maintenance\_of\_protease\_location\_in\_T\_cell\_secretory\_granule | 1 | 0 |  |  |  |  |  |  |  |  |
| GO:0033380\_granzyme\_B\_localization\_in\_T\_cell\_secretory\_granule | 1 | 0 |  |  |  |  |  |  |  |  |
| GO:0033382\_maintenance\_of\_granzyme\_B\_location\_in\_T\_cell\_secretory\_granule | 1 | 0 |  |  |  |  |  |  |  |  |
| GO:0033483\_gas\_homeostasis | 1 | 0 |  |  |  |  |  |  |  |  |
| GO:0033484\_nitric\_oxide\_homeostasis | 1 | 0 |  |  |  |  |  |  |  |  |
| GO:0033505\_floor\_plate\_morphogenesis | 1 | 0 |  |  |  |  |  |  |  |  |
| GO:0033522\_histone\_H2A\_ubiquitination | 1 | 0 |  |  |  |  |  |  |  |  |
| GO:0033523\_histone\_H2B\_ubiquitination | 1 | 0 |  |  |  |  |  |  |  |  |
| GO:0033574\_response\_to\_testosterone\_stimulus | 1 | 0 |  |  |  |  |  |  |  |  |
| GO:0033606\_chemokine\_receptor\_transport\_within\_lipid\_bilayer | 1 | 0 |  |  |  |  |  |  |  |  |
| GO:0033628\_regulation\_of\_cell\_adhesion\_mediated\_by\_integrin | 1 | 0 |  |  |  |  |  |  |  |  |
| GO:0033630\_positive\_regulation\_of\_cell\_adhesion\_mediated\_by\_integrin | 1 | 0 |  |  |  |  |  |  |  |  |
| GO:0033632\_regulation\_of\_cell-cell\_adhesion\_mediated\_by\_integrin | 1 | 0 |  |  |  |  |  |  |  |  |
| GO:0033634\_positive\_regulation\_of\_cell-cell\_adhesion\_mediated\_by\_integrin | 1 | 0 |  |  |  |  |  |  |  |  |
| GO:0033683\_nucleotide-excision\_repair\_\_DNA\_incision | 1 | 0 |  |  |  |  |  |  |  |  |
| GO:0033687\_osteoblast\_proliferation | 1 | 0 |  |  |  |  |  |  |  |  |
| GO:0033688\_regulation\_of\_osteoblast\_proliferation | 1 | 0 |  |  |  |  |  |  |  |  |
| GO:0033689\_negative\_regulation\_of\_osteoblast\_proliferation | 1 | 0 |  |  |  |  |  |  |  |  |
| GO:0033750\_ribosome\_localization | 1 | 0 |  |  |  |  |  |  |  |  |
| GO:0033753\_establishment\_of\_ribosome\_localization | 1 | 0 |  |  |  |  |  |  |  |  |
| GO:0033866\_nucleoside\_bisphosphate\_biosynthetic\_process | 1 | 0 |  |  |  |  |  |  |  |  |
| GO:0033875\_ribonucleoside\_bisphosphate\_metabolic\_process | 1 | 0 |  |  |  |  |  |  |  |  |
| GO:0034030\_ribonucleoside\_bisphosphate\_biosynthetic\_process | 1 | 0 |  |  |  |  |  |  |  |  |
| GO:0034032\_purine\_nucleoside\_bisphosphate\_metabolic\_process | 1 | 0 |  |  |  |  |  |  |  |  |
| GO:0034033\_purine\_nucleoside\_bisphosphate\_biosynthetic\_process | 1 | 0 |  |  |  |  |  |  |  |  |
| GO:0034035\_purine\_ribonucleoside\_bisphosphate\_metabolic\_process | 1 | 0 |  |  |  |  |  |  |  |  |
| GO:0034036\_purine\_ribonucleoside\_bisphosphate\_biosynthetic\_process | 1 | 0 |  |  |  |  |  |  |  |  |
| GO:0034067\_protein\_localization\_in\_Golgi\_apparatus | 1 | 0 |  |  |  |  |  |  |  |  |
| GO:0034102\_erythrocyte\_clearance | 1 | 0 |  |  |  |  |  |  |  |  |
| GO:0034106\_regulation\_of\_erythrocyte\_clearance | 1 | 0 |  |  |  |  |  |  |  |  |
| GO:0034107\_negative\_regulation\_of\_erythrocyte\_clearance | 1 | 0 |  |  |  |  |  |  |  |  |
| GO:0034110\_regulation\_of\_homotypic\_cell-cell\_adhesion | 1 | 0 |  |  |  |  |  |  |  |  |
| GO:0034111\_negative\_regulation\_of\_homotypic\_cell-cell\_adhesion | 1 | 0 |  |  |  |  |  |  |  |  |
| GO:0034113\_heterotypic\_cell-cell\_adhesion | 1 | 0 |  |  |  |  |  |  |  |  |
| GO:0034117\_erythrocyte\_aggregation | 1 | 0 |  |  |  |  |  |  |  |  |
| GO:0034118\_regulation\_of\_erythrocyte\_aggregation | 1 | 0 |  |  |  |  |  |  |  |  |
| GO:0034119\_negative\_regulation\_of\_erythrocyte\_aggregation | 1 | 0 |  |  |  |  |  |  |  |  |
| GO:0034121\_regulation\_of\_toll-like\_receptor\_signaling\_pathway | 1 | 0 |  |  |  |  |  |  |  |  |
| GO:0034122\_negative\_regulation\_of\_toll-like\_receptor\_signaling\_pathway | 1 | 0 |  |  |  |  |  |  |  |  |
| GO:0034230\_enkephalin\_processing | 1 | 0 |  |  |  |  |  |  |  |  |
| GO:0034379\_very-low-density\_lipoprotein\_particle\_assembly | 1 | 0 |  |  |  |  |  |  |  |  |
| GO:0034380\_high-density\_lipoprotein\_particle\_assembly | 1 | 0 |  |  |  |  |  |  |  |  |
| GO:0034394\_protein\_localization\_at\_cell\_surface | 1 | 0 |  |  |  |  |  |  |  |  |
| GO:0034405\_response\_to\_fluid\_shear\_stress | 1 | 0 |  |  |  |  |  |  |  |  |
| GO:0034472\_snRNA\_3'-end\_processing | 1 | 0 |  |  |  |  |  |  |  |  |
| GO:0034474\_U2\_snRNA\_3'-end\_processing | 1 | 0 |  |  |  |  |  |  |  |  |
| GO:0034502\_protein\_localization\_to\_chromosome | 1 | 0 |  |  |  |  |  |  |  |  |
| GO:0034505\_tooth\_mineralization | 1 | 0 |  |  |  |  |  |  |  |  |
| GO:0034508\_centromere\_complex\_assembly | 1 | 0 |  |  |  |  |  |  |  |  |
| GO:0034643\_mitochondrion\_localization\_\_microtubule-mediated | 1 | 0 |  |  |  |  |  |  |  |  |
| GO:0034969\_histone\_arginine\_methylation | 1 | 0 |  |  |  |  |  |  |  |  |
| GO:0034982\_mitochondrial\_protein\_processing | 1 | 0 |  |  |  |  |  |  |  |  |
| GO:0035022\_positive\_regulation\_of\_Rac\_protein\_signal\_transduction | 1 | 0 |  |  |  |  |  |  |  |  |
| GO:0035024\_negative\_regulation\_of\_Rho\_protein\_signal\_transduction | 1 | 0 |  |  |  |  |  |  |  |  |
| GO:0035037\_sperm\_entry | 1 | 0 |  |  |  |  |  |  |  |  |
| GO:0035039\_male\_pronucleus\_formation | 1 | 0 |  |  |  |  |  |  |  |  |
| GO:0035066\_positive\_regulation\_of\_histone\_acetylation | 1 | 0 |  |  |  |  |  |  |  |  |
| GO:0035083\_cilium\_axoneme\_assembly | 1 | 0 |  |  |  |  |  |  |  |  |
| GO:0035090\_maintenance\_of\_apical\_basal\_cell\_polarity | 1 | 0 |  |  |  |  |  |  |  |  |
| GO:0035106\_operant\_conditioning | 1 | 0 |  |  |  |  |  |  |  |  |
| GO:0035172\_hemocyte\_proliferation | 1 | 0 |  |  |  |  |  |  |  |  |
| GO:0035227\_regulation\_of\_glutamate-cysteine\_ligase\_activity | 1 | 0 |  |  |  |  |  |  |  |  |
| GO:0035229\_positive\_regulation\_of\_glutamate-cysteine\_ligase\_activity | 1 | 0 |  |  |  |  |  |  |  |  |
| GO:0035260\_internal\_genitalia\_morphogenesis | 1 | 0 |  |  |  |  |  |  |  |  |
| GO:0035262\_gonad\_morphogenesis | 1 | 0 |  |  |  |  |  |  |  |  |
| GO:0035287\_head\_segmentation | 1 | 0 |  |  |  |  |  |  |  |  |
| GO:0035289\_posterior\_head\_segmentation | 1 | 0 |  |  |  |  |  |  |  |  |
| GO:0035303\_regulation\_of\_dephosphorylation | 1 | 0 |  |  |  |  |  |  |  |  |
| GO:0035304\_regulation\_of\_protein\_amino\_acid\_dephosphorylation | 1 | 0 |  |  |  |  |  |  |  |  |
| GO:0035305\_negative\_regulation\_of\_dephosphorylation | 1 | 0 |  |  |  |  |  |  |  |  |
| GO:0035308\_negative\_regulation\_of\_protein\_amino\_acid\_dephosphorylation | 1 | 0 |  |  |  |  |  |  |  |  |
| GO:0035313\_wound\_healing\_\_spreading\_of\_epidermal\_cells | 1 | 0 |  |  |  |  |  |  |  |  |
| GO:0040013\_negative\_regulation\_of\_locomotion | 1 | 0 |  |  |  |  |  |  |  |  |
| GO:0040019\_positive\_regulation\_of\_embryonic\_development | 1 | 0 |  |  |  |  |  |  |  |  |
| GO:0040032\_post-embryonic\_body\_morphogenesis | 1 | 0 |  |  |  |  |  |  |  |  |
| GO:0040038\_polar\_body\_extrusion\_after\_meiotic\_divisions | 1 | 0 |  |  |  |  |  |  |  |  |
| GO:0042026\_protein\_refolding | 1 | 0 |  |  |  |  |  |  |  |  |
| GO:0042048\_olfactory\_behavior | 1 | 0 |  |  |  |  |  |  |  |  |
| GO:0042059\_negative\_regulation\_of\_epidermal\_growth\_factor\_receptor\_signaling\_pathway | 1 | 0 |  |  |  |  |  |  |  |  |
| GO:0042073\_intraflagellar\_transport | 1 | 0 |  |  |  |  |  |  |  |  |
| GO:0042078\_germ-line\_stem\_cell\_division | 1 | 0 |  |  |  |  |  |  |  |  |
| GO:0042091\_interleukin-10\_biosynthetic\_process | 1 | 0 |  |  |  |  |  |  |  |  |
| GO:0042103\_positive\_regulation\_of\_T\_cell\_homeostatic\_proliferation | 1 | 0 |  |  |  |  |  |  |  |  |
| GO:0042136\_neurotransmitter\_biosynthetic\_process | 1 | 0 |  |  |  |  |  |  |  |  |
| GO:0042137\_sequestering\_of\_neurotransmitter | 1 | 0 |  |  |  |  |  |  |  |  |
| GO:0042138\_meiotic\_DNA\_double-strand\_break\_formation | 1 | 0 |  |  |  |  |  |  |  |  |
| GO:0042178\_xenobiotic\_catabolic\_process | 1 | 0 |  |  |  |  |  |  |  |  |
| GO:0042225\_interleukin-5\_biosynthetic\_process | 1 | 0 |  |  |  |  |  |  |  |  |
| GO:0042231\_interleukin-13\_biosynthetic\_process | 1 | 0 |  |  |  |  |  |  |  |  |
| GO:0042255\_ribosome\_assembly | 1 | 0 |  |  |  |  |  |  |  |  |
| GO:0042257\_ribosomal\_subunit\_assembly | 1 | 0 |  |  |  |  |  |  |  |  |
| GO:0042264\_peptidyl-aspartic\_acid\_hydroxylation | 1 | 0 |  |  |  |  |  |  |  |  |
| GO:0042276\_error-prone\_postreplication\_DNA\_repair | 1 | 0 |  |  |  |  |  |  |  |  |
| GO:0042297\_vocal\_learning | 1 | 0 |  |  |  |  |  |  |  |  |
| GO:0042309\_homoiothermy | 1 | 0 |  |  |  |  |  |  |  |  |
| GO:0042320\_regulation\_of\_circadian\_sleep\_wake\_cycle\_\_REM\_sleep | 1 | 0 |  |  |  |  |  |  |  |  |
| GO:0042339\_keratan\_sulfate\_metabolic\_process | 1 | 0 |  |  |  |  |  |  |  |  |
| GO:0042347\_negative\_regulation\_of\_NF-kappaB\_import\_into\_nucleus | 1 | 0 |  |  |  |  |  |  |  |  |
| GO:0042360\_vitamin\_E\_metabolic\_process | 1 | 0 |  |  |  |  |  |  |  |  |
| GO:0042363\_fat-soluble\_vitamin\_catabolic\_process | 1 | 0 |  |  |  |  |  |  |  |  |
| GO:0042369\_vitamin\_D\_catabolic\_process | 1 | 0 |  |  |  |  |  |  |  |  |
| GO:0042373\_vitamin\_K\_metabolic\_process | 1 | 0 |  |  |  |  |  |  |  |  |
| GO:0042404\_thyroid\_hormone\_catabolic\_process | 1 | 0 |  |  |  |  |  |  |  |  |
| GO:0042414\_epinephrine\_metabolic\_process | 1 | 0 |  |  |  |  |  |  |  |  |
| GO:0042436\_indole\_derivative\_catabolic\_process | 1 | 0 |  |  |  |  |  |  |  |  |
| GO:0042489\_negative\_regulation\_of\_odontogenesis\_of\_dentine-containing\_tooth | 1 | 0 |  |  |  |  |  |  |  |  |
| GO:0042508\_tyrosine\_phosphorylation\_of\_Stat1\_protein | 1 | 0 |  |  |  |  |  |  |  |  |
| GO:0042518\_negative\_regulation\_of\_tyrosine\_phosphorylation\_of\_Stat3\_protein | 1 | 0 |  |  |  |  |  |  |  |  |
| GO:0042524\_negative\_regulation\_of\_tyrosine\_phosphorylation\_of\_Stat5\_protein | 1 | 0 |  |  |  |  |  |  |  |  |
| GO:0042536\_negative\_regulation\_of\_tumor\_necrosis\_factor\_biosynthetic\_process | 1 | 0 |  |  |  |  |  |  |  |  |
| GO:0042538\_hyperosmotic\_salinity\_response | 1 | 0 |  |  |  |  |  |  |  |  |
| GO:0042628\_mating\_plug\_formation | 1 | 0 |  |  |  |  |  |  |  |  |
| GO:0042631\_cellular\_response\_to\_water\_deprivation | 1 | 0 |  |  |  |  |  |  |  |  |
| GO:0042637\_catagen | 1 | 0 |  |  |  |  |  |  |  |  |
| GO:0042660\_positive\_regulation\_of\_cell\_fate\_specification | 1 | 0 |  |  |  |  |  |  |  |  |
| GO:0042663\_regulation\_of\_endodermal\_cell\_fate\_specification | 1 | 0 |  |  |  |  |  |  |  |  |
| GO:0042664\_negative\_regulation\_of\_endodermal\_cell\_fate\_specification | 1 | 0 |  |  |  |  |  |  |  |  |
| GO:0042667\_auditory\_receptor\_cell\_fate\_specification | 1 | 0 |  |  |  |  |  |  |  |  |
| GO:0042694\_muscle\_cell\_fate\_specification | 1 | 0 |  |  |  |  |  |  |  |  |
| GO:0042706\_eye\_photoreceptor\_cell\_fate\_commitment | 1 | 0 |  |  |  |  |  |  |  |  |
| GO:0042713\_sperm\_ejaculation | 1 | 0 |  |  |  |  |  |  |  |  |
| GO:0042723\_thiamin\_and\_derivative\_metabolic\_process | 1 | 0 |  |  |  |  |  |  |  |  |
| GO:0042737\_drug\_catabolic\_process | 1 | 0 |  |  |  |  |  |  |  |  |
| GO:0042738\_exogenous\_drug\_catabolic\_process | 1 | 0 |  |  |  |  |  |  |  |  |
| GO:0042747\_circadian\_sleep\_wake\_cycle\_\_REM\_sleep | 1 | 0 |  |  |  |  |  |  |  |  |
| GO:0042748\_circadian\_sleep\_wake\_cycle\_\_non-REM\_sleep | 1 | 0 |  |  |  |  |  |  |  |  |
| GO:0042772\_DNA\_damage\_response\_\_signal\_transduction\_resulting\_in\_transcription | 1 | 0 |  |  |  |  |  |  |  |  |
| GO:0042790\_transcription\_of\_nuclear\_rRNA\_large\_RNA\_polymerase\_I\_transcript | 1 | 0 |  |  |  |  |  |  |  |  |
| GO:0042839\_D-glucuronate\_metabolic\_process | 1 | 0 |  |  |  |  |  |  |  |  |
| GO:0042840\_D-glucuronate\_catabolic\_process | 1 | 0 |  |  |  |  |  |  |  |  |
| GO:0042891\_antibiotic\_transport | 1 | 0 |  |  |  |  |  |  |  |  |
| GO:0042892\_chloramphenicol\_transport | 1 | 0 |  |  |  |  |  |  |  |  |
| GO:0042940\_D-amino\_acid\_transport | 1 | 0 |  |  |  |  |  |  |  |  |
| GO:0042941\_D-alanine\_transport | 1 | 0 |  |  |  |  |  |  |  |  |
| GO:0042942\_D-serine\_transport | 1 | 0 |  |  |  |  |  |  |  |  |
| GO:0042983\_amyloid\_precursor\_protein\_biosynthetic\_process | 1 | 0 |  |  |  |  |  |  |  |  |
| GO:0042984\_regulation\_of\_amyloid\_precursor\_protein\_biosynthetic\_process | 1 | 0 |  |  |  |  |  |  |  |  |
| GO:0042985\_negative\_regulation\_of\_amyloid\_precursor\_protein\_biosynthetic\_process | 1 | 0 |  |  |  |  |  |  |  |  |
| GO:0042989\_sequestering\_of\_actin\_monomers | 1 | 0 |  |  |  |  |  |  |  |  |
| GO:0043044\_ATP-dependent\_chromatin\_remodeling | 1 | 0 |  |  |  |  |  |  |  |  |
| GO:0043056\_forward\_locomotion | 1 | 0 |  |  |  |  |  |  |  |  |
| GO:0043060\_meiotic\_metaphase\_I\_plate\_congression | 1 | 0 |  |  |  |  |  |  |  |  |
| GO:0043091\_L-arginine\_import | 1 | 0 |  |  |  |  |  |  |  |  |
| GO:0043124\_negative\_regulation\_of\_I-kappaB\_kinase\_NF-kappaB\_cascade | 1 | 0 |  |  |  |  |  |  |  |  |
| GO:0043132\_NAD\_transport | 1 | 0 |  |  |  |  |  |  |  |  |
| GO:0043153\_entrainment\_of\_circadian\_clock\_by\_photoperiod | 1 | 0 |  |  |  |  |  |  |  |  |
| GO:0043171\_peptide\_catabolic\_process | 1 | 0 |  |  |  |  |  |  |  |  |
| GO:0043179\_rhythmic\_excitation | 1 | 0 |  |  |  |  |  |  |  |  |
| GO:0043206\_fibril\_organization | 1 | 0 |  |  |  |  |  |  |  |  |
| GO:0043217\_myelin\_maintenance | 1 | 0 |  |  |  |  |  |  |  |  |
| GO:0043313\_regulation\_of\_neutrophil\_degranulation | 1 | 0 |  |  |  |  |  |  |  |  |
| GO:0043316\_cytotoxic\_T\_cell\_degranulation | 1 | 0 |  |  |  |  |  |  |  |  |
| GO:0043369\_CD4-positive\_or\_CD8-positive\_\_alpha-beta\_T\_cell\_lineage\_commitment | 1 | 0 |  |  |  |  |  |  |  |  |
| GO:0043375\_CD8-positive\_\_alpha-beta\_T\_cell\_lineage\_commitment | 1 | 0 |  |  |  |  |  |  |  |  |
| GO:0043379\_memory\_T\_cell\_differentiation | 1 | 0 |  |  |  |  |  |  |  |  |
| GO:0043380\_regulation\_of\_memory\_T\_cell\_differentiation | 1 | 0 |  |  |  |  |  |  |  |  |
| GO:0043400\_cortisol\_secretion | 1 | 0 |  |  |  |  |  |  |  |  |
| GO:0043415\_positive\_regulation\_of\_skeletal\_muscle\_regeneration | 1 | 0 |  |  |  |  |  |  |  |  |
| GO:0043416\_regulation\_of\_skeletal\_muscle\_regeneration | 1 | 0 |  |  |  |  |  |  |  |  |
| GO:0043437\_butanoic\_acid\_metabolic\_process | 1 | 0 |  |  |  |  |  |  |  |  |
| GO:0043438\_acetoacetic\_acid\_metabolic\_process | 1 | 0 |  |  |  |  |  |  |  |  |
| GO:0043480\_pigment\_accumulation\_in\_tissues | 1 | 0 |  |  |  |  |  |  |  |  |
| GO:0043482\_cellular\_pigment\_accumulation | 1 | 0 |  |  |  |  |  |  |  |  |
| GO:0043486\_histone\_exchange | 1 | 0 |  |  |  |  |  |  |  |  |
| GO:0043496\_regulation\_of\_protein\_homodimerization\_activity | 1 | 0 |  |  |  |  |  |  |  |  |
| GO:0043501\_skeletal\_muscle\_adaptation | 1 | 0 |  |  |  |  |  |  |  |  |
| GO:0043508\_negative\_regulation\_of\_JUN\_kinase\_activity | 1 | 0 |  |  |  |  |  |  |  |  |
| GO:0043517\_positive\_regulation\_of\_DNA\_damage\_response\_\_signal\_transduction\_by\_p53\_class\_mediator | 1 | 0 |  |  |  |  |  |  |  |  |
| GO:0043535\_regulation\_of\_blood\_vessel\_endothelial\_cell\_migration | 1 | 0 |  |  |  |  |  |  |  |  |
| GO:0043537\_negative\_regulation\_of\_blood\_vessel\_endothelial\_cell\_migration | 1 | 0 |  |  |  |  |  |  |  |  |
| GO:0043545\_molybdopterin\_cofactor\_metabolic\_process | 1 | 0 |  |  |  |  |  |  |  |  |
| GO:0043587\_tongue\_morphogenesis | 1 | 0 |  |  |  |  |  |  |  |  |
| GO:0043604\_amide\_biosynthetic\_process | 1 | 0 |  |  |  |  |  |  |  |  |
| GO:0043628\_ncRNA\_3'-end\_processing | 1 | 0 |  |  |  |  |  |  |  |  |
| GO:0044254\_multicellular\_organismal\_protein\_catabolic\_process | 1 | 0 |  |  |  |  |  |  |  |  |
| GO:0044256\_protein\_digestion | 1 | 0 |  |  |  |  |  |  |  |  |
| GO:0044266\_multicellular\_organismal\_macromolecule\_catabolic\_process | 1 | 0 |  |  |  |  |  |  |  |  |
| GO:0045004\_DNA\_replication\_proofreading | 1 | 0 |  |  |  |  |  |  |  |  |
| GO:0045019\_negative\_regulation\_of\_nitric\_oxide\_biosynthetic\_process | 1 | 0 |  |  |  |  |  |  |  |  |
| GO:0045020\_error-prone\_DNA\_repair | 1 | 0 |  |  |  |  |  |  |  |  |
| GO:0045022\_early\_endosome\_to\_late\_endosome\_transport | 1 | 0 |  |  |  |  |  |  |  |  |
| GO:0045062\_extrathymic\_T\_cell\_selection | 1 | 0 |  |  |  |  |  |  |  |  |
| GO:0045069\_regulation\_of\_viral\_genome\_replication | 1 | 0 |  |  |  |  |  |  |  |  |
| GO:0045074\_regulation\_of\_interleukin-10\_biosynthetic\_process | 1 | 0 |  |  |  |  |  |  |  |  |
| GO:0045082\_positive\_regulation\_of\_interleukin-10\_biosynthetic\_process | 1 | 0 |  |  |  |  |  |  |  |  |
| GO:0045083\_negative\_regulation\_of\_interleukin-12\_biosynthetic\_process | 1 | 0 |  |  |  |  |  |  |  |  |
| GO:0045112\_integrin\_biosynthetic\_process | 1 | 0 |  |  |  |  |  |  |  |  |
| GO:0045113\_regulation\_of\_integrin\_biosynthetic\_process | 1 | 0 |  |  |  |  |  |  |  |  |
| GO:0045188\_regulation\_of\_circadian\_sleep\_wake\_cycle\_\_non-REM\_sleep | 1 | 0 |  |  |  |  |  |  |  |  |
| GO:0045210\_FasL\_biosynthetic\_process | 1 | 0 |  |  |  |  |  |  |  |  |
| GO:0045297\_post-mating\_behavior | 1 | 0 |  |  |  |  |  |  |  |  |
| GO:0045299\_otolith\_mineralization | 1 | 0 |  |  |  |  |  |  |  |  |
| GO:0045329\_carnitine\_biosynthetic\_process | 1 | 0 |  |  |  |  |  |  |  |  |
| GO:0045341\_MHC\_class\_I\_biosynthetic\_process | 1 | 0 |  |  |  |  |  |  |  |  |
| GO:0045343\_regulation\_of\_MHC\_class\_I\_biosynthetic\_process | 1 | 0 |  |  |  |  |  |  |  |  |
| GO:0045347\_negative\_regulation\_of\_MHC\_class\_II\_biosynthetic\_process | 1 | 0 |  |  |  |  |  |  |  |  |
| GO:0045405\_regulation\_of\_interleukin-5\_biosynthetic\_process | 1 | 0 |  |  |  |  |  |  |  |  |
| GO:0045407\_positive\_regulation\_of\_interleukin-5\_biosynthetic\_process | 1 | 0 |  |  |  |  |  |  |  |  |
| GO:0045426\_quinone\_cofactor\_biosynthetic\_process | 1 | 0 |  |  |  |  |  |  |  |  |
| GO:0045448\_mitotic\_cell\_cycle\_\_embryonic | 1 | 0 |  |  |  |  |  |  |  |  |
| GO:0045454\_cell\_redox\_homeostasis | 1 | 0 |  |  |  |  |  |  |  |  |
| GO:0045583\_regulation\_of\_cytotoxic\_T\_cell\_differentiation | 1 | 0 |  |  |  |  |  |  |  |  |
| GO:0045585\_positive\_regulation\_of\_cytotoxic\_T\_cell\_differentiation | 1 | 0 |  |  |  |  |  |  |  |  |
| GO:0045601\_regulation\_of\_endothelial\_cell\_differentiation | 1 | 0 |  |  |  |  |  |  |  |  |
| GO:0045602\_negative\_regulation\_of\_endothelial\_cell\_differentiation | 1 | 0 |  |  |  |  |  |  |  |  |
| GO:0045605\_negative\_regulation\_of\_epidermal\_cell\_differentiation | 1 | 0 |  |  |  |  |  |  |  |  |
| GO:0045606\_positive\_regulation\_of\_epidermal\_cell\_differentiation | 1 | 0 |  |  |  |  |  |  |  |  |
| GO:0045609\_positive\_regulation\_of\_auditory\_receptor\_cell\_differentiation | 1 | 0 |  |  |  |  |  |  |  |  |
| GO:0045617\_negative\_regulation\_of\_keratinocyte\_differentiation | 1 | 0 |  |  |  |  |  |  |  |  |
| GO:0045618\_positive\_regulation\_of\_keratinocyte\_differentiation | 1 | 0 |  |  |  |  |  |  |  |  |
| GO:0045626\_negative\_regulation\_of\_T-helper\_1\_cell\_differentiation | 1 | 0 |  |  |  |  |  |  |  |  |
| GO:0045633\_positive\_regulation\_of\_mechanoreceptor\_differentiation | 1 | 0 |  |  |  |  |  |  |  |  |
| GO:0045650\_negative\_regulation\_of\_macrophage\_differentiation | 1 | 0 |  |  |  |  |  |  |  |  |
| GO:0045656\_negative\_regulation\_of\_monocyte\_differentiation | 1 | 0 |  |  |  |  |  |  |  |  |
| GO:0045657\_positive\_regulation\_of\_monocyte\_differentiation | 1 | 0 |  |  |  |  |  |  |  |  |
| GO:0045659\_negative\_regulation\_of\_neutrophil\_differentiation | 1 | 0 |  |  |  |  |  |  |  |  |
| GO:0045660\_positive\_regulation\_of\_neutrophil\_differentiation | 1 | 0 |  |  |  |  |  |  |  |  |
| GO:0045721\_negative\_regulation\_of\_gluconeogenesis | 1 | 0 |  |  |  |  |  |  |  |  |
| GO:0045724\_positive\_regulation\_of\_flagellum\_assembly | 1 | 0 |  |  |  |  |  |  |  |  |
| GO:0045725\_positive\_regulation\_of\_glycogen\_biosynthetic\_process | 1 | 0 |  |  |  |  |  |  |  |  |
| GO:0045740\_positive\_regulation\_of\_DNA\_replication | 1 | 0 |  |  |  |  |  |  |  |  |
| GO:0045759\_negative\_regulation\_of\_action\_potential | 1 | 0 |  |  |  |  |  |  |  |  |
| GO:0045768\_positive\_regulation\_of\_anti-apoptosis | 1 | 0 |  |  |  |  |  |  |  |  |
| GO:0045769\_negative\_regulation\_of\_asymmetric\_cell\_division | 1 | 0 |  |  |  |  |  |  |  |  |
| GO:0045794\_negative\_regulation\_of\_cell\_volume | 1 | 0 |  |  |  |  |  |  |  |  |
| GO:0045815\_positive\_regulation\_of\_gene\_expression\_\_epigenetic | 1 | 0 |  |  |  |  |  |  |  |  |
| GO:0045818\_negative\_regulation\_of\_glycogen\_catabolic\_process | 1 | 0 |  |  |  |  |  |  |  |  |
| GO:0045842\_positive\_regulation\_of\_mitotic\_metaphase\_anaphase\_transition | 1 | 0 |  |  |  |  |  |  |  |  |
| GO:0045875\_negative\_regulation\_of\_sister\_chromatid\_cohesion | 1 | 0 |  |  |  |  |  |  |  |  |
| GO:0045898\_regulation\_of\_transcriptional\_preinitiation\_complex\_assembly | 1 | 0 |  |  |  |  |  |  |  |  |
| GO:0045899\_positive\_regulation\_of\_transcriptional\_preinitiation\_complex\_assembly | 1 | 0 |  |  |  |  |  |  |  |  |
| GO:0045906\_negative\_regulation\_of\_vasoconstriction | 1 | 0 |  |  |  |  |  |  |  |  |
| GO:0045908\_negative\_regulation\_of\_vasodilation | 1 | 0 |  |  |  |  |  |  |  |  |
| GO:0045915\_positive\_regulation\_of\_catecholamine\_metabolic\_process | 1 | 0 |  |  |  |  |  |  |  |  |
| GO:0045920\_negative\_regulation\_of\_exocytosis | 1 | 0 |  |  |  |  |  |  |  |  |
| GO:0045924\_regulation\_of\_female\_receptivity | 1 | 0 |  |  |  |  |  |  |  |  |
| GO:0045947\_negative\_regulation\_of\_translational\_initiation | 1 | 0 |  |  |  |  |  |  |  |  |
| GO:0045955\_negative\_regulation\_of\_calcium\_ion-dependent\_exocytosis | 1 | 0 |  |  |  |  |  |  |  |  |
| GO:0045956\_positive\_regulation\_of\_calcium\_ion-dependent\_exocytosis | 1 | 0 |  |  |  |  |  |  |  |  |
| GO:0045964\_positive\_regulation\_of\_dopamine\_metabolic\_process | 1 | 0 |  |  |  |  |  |  |  |  |
| GO:0045988\_negative\_regulation\_of\_striated\_muscle\_contraction | 1 | 0 |  |  |  |  |  |  |  |  |
| GO:0045989\_positive\_regulation\_of\_striated\_muscle\_contraction | 1 | 0 |  |  |  |  |  |  |  |  |
| GO:0045990\_regulation\_of\_transcription\_by\_carbon\_catabolites | 1 | 0 |  |  |  |  |  |  |  |  |
| GO:0045991\_positive\_regulation\_of\_transcription\_by\_carbon\_catabolites | 1 | 0 |  |  |  |  |  |  |  |  |
| GO:0045994\_positive\_regulation\_of\_translational\_initiation\_by\_iron | 1 | 0 |  |  |  |  |  |  |  |  |
| GO:0046007\_negative\_regulation\_of\_activated\_T\_cell\_proliferation | 1 | 0 |  |  |  |  |  |  |  |  |
| GO:0046014\_negative\_regulation\_of\_T\_cell\_homeostatic\_proliferation | 1 | 0 |  |  |  |  |  |  |  |  |
| GO:0046015\_regulation\_of\_transcription\_by\_glucose | 1 | 0 |  |  |  |  |  |  |  |  |
| GO:0046016\_positive\_regulation\_of\_transcription\_by\_glucose | 1 | 0 |  |  |  |  |  |  |  |  |
| GO:0046031\_ADP\_metabolic\_process | 1 | 0 |  |  |  |  |  |  |  |  |
| GO:0046032\_ADP\_catabolic\_process | 1 | 0 |  |  |  |  |  |  |  |  |
| GO:0046061\_dATP\_catabolic\_process | 1 | 0 |  |  |  |  |  |  |  |  |
| GO:0046075\_dTTP\_metabolic\_process | 1 | 0 |  |  |  |  |  |  |  |  |
| GO:0046078\_dUMP\_metabolic\_process | 1 | 0 |  |  |  |  |  |  |  |  |
| GO:0046079\_dUMP\_catabolic\_process | 1 | 0 |  |  |  |  |  |  |  |  |
| GO:0046086\_adenosine\_biosynthetic\_process | 1 | 0 |  |  |  |  |  |  |  |  |
| GO:0046090\_deoxyadenosine\_metabolic\_process | 1 | 0 |  |  |  |  |  |  |  |  |
| GO:0046098\_guanine\_metabolic\_process | 1 | 0 |  |  |  |  |  |  |  |  |
| GO:0046101\_hypoxanthine\_biosynthetic\_process | 1 | 0 |  |  |  |  |  |  |  |  |
| GO:0046102\_inosine\_metabolic\_process | 1 | 0 |  |  |  |  |  |  |  |  |
| GO:0046103\_inosine\_biosynthetic\_process | 1 | 0 |  |  |  |  |  |  |  |  |
| GO:0046108\_uridine\_metabolic\_process | 1 | 0 |  |  |  |  |  |  |  |  |
| GO:0046110\_xanthine\_metabolic\_process | 1 | 0 |  |  |  |  |  |  |  |  |
| GO:0046111\_xanthine\_biosynthetic\_process | 1 | 0 |  |  |  |  |  |  |  |  |
| GO:0046112\_nucleobase\_biosynthetic\_process | 1 | 0 |  |  |  |  |  |  |  |  |
| GO:0046113\_nucleobase\_catabolic\_process | 1 | 0 |  |  |  |  |  |  |  |  |
| GO:0046121\_deoxyribonucleoside\_catabolic\_process | 1 | 0 |  |  |  |  |  |  |  |  |
| GO:0046122\_purine\_deoxyribonucleoside\_metabolic\_process | 1 | 0 |  |  |  |  |  |  |  |  |
| GO:0046124\_purine\_deoxyribonucleoside\_catabolic\_process | 1 | 0 |  |  |  |  |  |  |  |  |
| GO:0046125\_pyrimidine\_deoxyribonucleoside\_metabolic\_process | 1 | 0 |  |  |  |  |  |  |  |  |
| GO:0046131\_pyrimidine\_ribonucleoside\_metabolic\_process | 1 | 0 |  |  |  |  |  |  |  |  |
| GO:0046160\_heme\_a\_metabolic\_process | 1 | 0 |  |  |  |  |  |  |  |  |
| GO:0046218\_indolalkylamine\_catabolic\_process | 1 | 0 |  |  |  |  |  |  |  |  |
| GO:0046292\_formaldehyde\_metabolic\_process | 1 | 0 |  |  |  |  |  |  |  |  |
| GO:0046294\_formaldehyde\_catabolic\_process | 1 | 0 |  |  |  |  |  |  |  |  |
| GO:0046314\_phosphocreatine\_biosynthetic\_process | 1 | 0 |  |  |  |  |  |  |  |  |
| GO:0046327\_glycerol\_biosynthetic\_process\_from\_pyruvate | 1 | 0 |  |  |  |  |  |  |  |  |
| GO:0046329\_negative\_regulation\_of\_JNK\_cascade | 1 | 0 |  |  |  |  |  |  |  |  |
| GO:0046340\_diacylglycerol\_catabolic\_process | 1 | 0 |  |  |  |  |  |  |  |  |
| GO:0046351\_disaccharide\_biosynthetic\_process | 1 | 0 |  |  |  |  |  |  |  |  |
| GO:0046356\_acetyl-CoA\_catabolic\_process | 1 | 0 |  |  |  |  |  |  |  |  |
| GO:0046358\_butyrate\_biosynthetic\_process | 1 | 0 |  |  |  |  |  |  |  |  |
| GO:0046359\_butyrate\_catabolic\_process | 1 | 0 |  |  |  |  |  |  |  |  |
| GO:0046381\_CMP-N-acetylneuraminate\_metabolic\_process | 1 | 0 |  |  |  |  |  |  |  |  |
| GO:0046415\_urate\_metabolic\_process | 1 | 0 |  |  |  |  |  |  |  |  |
| GO:0046416\_D-amino\_acid\_metabolic\_process | 1 | 0 |  |  |  |  |  |  |  |  |
| GO:0046434\_organophosphate\_catabolic\_process | 1 | 0 |  |  |  |  |  |  |  |  |
| GO:0046437\_D-amino\_acid\_biosynthetic\_process | 1 | 0 |  |  |  |  |  |  |  |  |
| GO:0046440\_L-lysine\_metabolic\_process | 1 | 0 |  |  |  |  |  |  |  |  |
| GO:0046449\_creatinine\_metabolic\_process | 1 | 0 |  |  |  |  |  |  |  |  |
| GO:0046471\_phosphatidylglycerol\_metabolic\_process | 1 | 0 |  |  |  |  |  |  |  |  |
| GO:0046473\_phosphatidic\_acid\_metabolic\_process | 1 | 0 |  |  |  |  |  |  |  |  |
| GO:0046476\_glycosylceramide\_biosynthetic\_process | 1 | 0 |  |  |  |  |  |  |  |  |
| GO:0046477\_glycosylceramide\_catabolic\_process | 1 | 0 |  |  |  |  |  |  |  |  |
| GO:0046485\_ether\_lipid\_metabolic\_process | 1 | 0 |  |  |  |  |  |  |  |  |
| GO:0046487\_glyoxylate\_metabolic\_process | 1 | 0 |  |  |  |  |  |  |  |  |
| GO:0046498\_S-adenosylhomocysteine\_metabolic\_process | 1 | 0 |  |  |  |  |  |  |  |  |
| GO:0046552\_photoreceptor\_cell\_fate\_commitment | 1 | 0 |  |  |  |  |  |  |  |  |
| GO:0046586\_regulation\_of\_calcium-dependent\_cell-cell\_adhesion | 1 | 0 |  |  |  |  |  |  |  |  |
| GO:0046587\_positive\_regulation\_of\_calcium-dependent\_cell-cell\_adhesion | 1 | 0 |  |  |  |  |  |  |  |  |
| GO:0046602\_regulation\_of\_mitotic\_centrosome\_separation | 1 | 0 |  |  |  |  |  |  |  |  |
| GO:0046604\_positive\_regulation\_of\_mitotic\_centrosome\_separation | 1 | 0 |  |  |  |  |  |  |  |  |
| GO:0046607\_positive\_regulation\_of\_centrosome\_cycle | 1 | 0 |  |  |  |  |  |  |  |  |
| GO:0046655\_folic\_acid\_metabolic\_process | 1 | 0 |  |  |  |  |  |  |  |  |
| GO:0046671\_negative\_regulation\_of\_retinal\_cell\_programmed\_cell\_death | 1 | 0 |  |  |  |  |  |  |  |  |
| GO:0046685\_response\_to\_arsenic | 1 | 0 |  |  |  |  |  |  |  |  |
| GO:0046692\_sperm\_competition | 1 | 0 |  |  |  |  |  |  |  |  |
| GO:0046707\_IDP\_metabolic\_process | 1 | 0 |  |  |  |  |  |  |  |  |
| GO:0046709\_IDP\_catabolic\_process | 1 | 0 |  |  |  |  |  |  |  |  |
| GO:0046724\_oxalic\_acid\_secretion | 1 | 0 |  |  |  |  |  |  |  |  |
| GO:0046753\_non-lytic\_viral\_release | 1 | 0 |  |  |  |  |  |  |  |  |
| GO:0046755\_non-lytic\_virus\_budding | 1 | 0 |  |  |  |  |  |  |  |  |
| GO:0046826\_negative\_regulation\_of\_protein\_export\_from\_nucleus | 1 | 0 |  |  |  |  |  |  |  |  |
| GO:0046827\_positive\_regulation\_of\_protein\_export\_from\_nucleus | 1 | 0 |  |  |  |  |  |  |  |  |
| GO:0046831\_regulation\_of\_RNA\_export\_from\_nucleus | 1 | 0 |  |  |  |  |  |  |  |  |
| GO:0046834\_lipid\_phosphorylation | 1 | 0 |  |  |  |  |  |  |  |  |
| GO:0046853\_inositol\_and\_derivative\_phosphorylation | 1 | 0 |  |  |  |  |  |  |  |  |
| GO:0046877\_regulation\_of\_saliva\_secretion | 1 | 0 |  |  |  |  |  |  |  |  |
| GO:0046878\_positive\_regulation\_of\_saliva\_secretion | 1 | 0 |  |  |  |  |  |  |  |  |
| GO:0046884\_follicle-stimulating\_hormone\_secretion | 1 | 0 |  |  |  |  |  |  |  |  |
| GO:0046898\_response\_to\_cycloheximide | 1 | 0 |  |  |  |  |  |  |  |  |
| GO:0046929\_negative\_regulation\_of\_neurotransmitter\_secretion | 1 | 0 |  |  |  |  |  |  |  |  |
| GO:0046931\_pore\_complex\_biogenesis | 1 | 0 |  |  |  |  |  |  |  |  |
| GO:0046949\_acyl-CoA\_biosynthetic\_process | 1 | 0 |  |  |  |  |  |  |  |  |
| GO:0046958\_nonassociative\_learning | 1 | 0 |  |  |  |  |  |  |  |  |
| GO:0046960\_sensitization | 1 | 0 |  |  |  |  |  |  |  |  |
| GO:0046986\_negative\_regulation\_of\_hemoglobin\_biosynthetic\_process | 1 | 0 |  |  |  |  |  |  |  |  |
| GO:0047497\_mitochondrion\_transport\_along\_microtubule | 1 | 0 |  |  |  |  |  |  |  |  |
| GO:0048047\_mating\_behavior\_\_sex\_discrimination | 1 | 0 |  |  |  |  |  |  |  |  |
| GO:0048133\_male\_germ-line\_stem\_cell\_division | 1 | 0 |  |  |  |  |  |  |  |  |
| GO:0048137\_spermatocyte\_division | 1 | 0 |  |  |  |  |  |  |  |  |
| GO:0048143\_astrocyte\_activation | 1 | 0 |  |  |  |  |  |  |  |  |
| GO:0048170\_positive\_regulation\_of\_long-term\_neuronal\_synaptic\_plasticity | 1 | 0 |  |  |  |  |  |  |  |  |
| GO:0048199\_vesicle\_targeting\_\_to\_\_from\_or\_within\_Golgi | 1 | 0 |  |  |  |  |  |  |  |  |
| GO:0048241\_epinephrine\_transport | 1 | 0 |  |  |  |  |  |  |  |  |
| GO:0048242\_epinephrine\_secretion | 1 | 0 |  |  |  |  |  |  |  |  |
| GO:0048243\_norepinephrine\_secretion | 1 | 0 |  |  |  |  |  |  |  |  |
| GO:0048247\_lymphocyte\_chemotaxis | 1 | 0 |  |  |  |  |  |  |  |  |
| GO:0048250\_mitochondrial\_iron\_ion\_transport | 1 | 0 |  |  |  |  |  |  |  |  |
| GO:0048259\_regulation\_of\_receptor-mediated\_endocytosis | 1 | 0 |  |  |  |  |  |  |  |  |
| GO:0048260\_positive\_regulation\_of\_receptor-mediated\_endocytosis | 1 | 0 |  |  |  |  |  |  |  |  |
| GO:0048290\_isotype\_switching\_to\_IgA\_isotypes | 1 | 0 |  |  |  |  |  |  |  |  |
| GO:0048296\_regulation\_of\_isotype\_switching\_to\_IgA\_isotypes | 1 | 0 |  |  |  |  |  |  |  |  |
| GO:0048298\_positive\_regulation\_of\_isotype\_switching\_to\_IgA\_isotypes | 1 | 0 |  |  |  |  |  |  |  |  |
| GO:0048319\_axial\_mesoderm\_morphogenesis | 1 | 0 |  |  |  |  |  |  |  |  |
| GO:0048320\_axial\_mesoderm\_formation | 1 | 0 |  |  |  |  |  |  |  |  |
| GO:0048385\_regulation\_of\_retinoic\_acid\_receptor\_signaling\_pathway | 1 | 0 |  |  |  |  |  |  |  |  |
| GO:0048387\_negative\_regulation\_of\_retinoic\_acid\_receptor\_signaling\_pathway | 1 | 0 |  |  |  |  |  |  |  |  |
| GO:0048388\_endosomal\_lumen\_acidification | 1 | 0 |  |  |  |  |  |  |  |  |
| GO:0048389\_intermediate\_mesoderm\_development | 1 | 0 |  |  |  |  |  |  |  |  |
| GO:0048478\_replication\_fork\_protection | 1 | 0 |  |  |  |  |  |  |  |  |
| GO:0048496\_maintenance\_of\_organ\_identity | 1 | 0 |  |  |  |  |  |  |  |  |
| GO:0048525\_negative\_regulation\_of\_viral\_reproduction | 1 | 0 |  |  |  |  |  |  |  |  |
| GO:0048539\_bone\_marrow\_development | 1 | 0 |  |  |  |  |  |  |  |  |
| GO:0048548\_regulation\_of\_pinocytosis | 1 | 0 |  |  |  |  |  |  |  |  |
| GO:0048549\_positive\_regulation\_of\_pinocytosis | 1 | 0 |  |  |  |  |  |  |  |  |
| GO:0048553\_negative\_regulation\_of\_metalloenzyme\_activity | 1 | 0 |  |  |  |  |  |  |  |  |
| GO:0048588\_developmental\_cell\_growth | 1 | 0 |  |  |  |  |  |  |  |  |
| GO:0048601\_oocyte\_morphogenesis | 1 | 0 |  |  |  |  |  |  |  |  |
| GO:0048621\_post-embryonic\_gut\_morphogenesis | 1 | 0 |  |  |  |  |  |  |  |  |
| GO:0048640\_negative\_regulation\_of\_developmental\_growth | 1 | 0 |  |  |  |  |  |  |  |  |
| GO:0048642\_negative\_regulation\_of\_skeletal\_muscle\_tissue\_development | 1 | 0 |  |  |  |  |  |  |  |  |
| GO:0048669\_collateral\_sprouting\_in\_the\_absence\_of\_injury | 1 | 0 |  |  |  |  |  |  |  |  |
| GO:0048680\_positive\_regulation\_of\_axon\_regeneration | 1 | 0 |  |  |  |  |  |  |  |  |
| GO:0048681\_negative\_regulation\_of\_axon\_regeneration | 1 | 0 |  |  |  |  |  |  |  |  |
| GO:0048686\_regulation\_of\_sprouting\_of\_injured\_axon | 1 | 0 |  |  |  |  |  |  |  |  |
| GO:0048687\_positive\_regulation\_of\_sprouting\_of\_injured\_axon | 1 | 0 |  |  |  |  |  |  |  |  |
| GO:0048690\_regulation\_of\_axon\_extension\_involved\_in\_regeneration | 1 | 0 |  |  |  |  |  |  |  |  |
| GO:0048691\_positive\_regulation\_of\_axon\_extension\_involved\_in\_regeneration | 1 | 0 |  |  |  |  |  |  |  |  |
| GO:0048714\_positive\_regulation\_of\_oligodendrocyte\_differentiation | 1 | 0 |  |  |  |  |  |  |  |  |
| GO:0048733\_sebaceous\_gland\_development | 1 | 0 |  |  |  |  |  |  |  |  |
| GO:0048743\_positive\_regulation\_of\_skeletal\_muscle\_fiber\_development | 1 | 0 |  |  |  |  |  |  |  |  |
| GO:0048752\_semicircular\_canal\_morphogenesis | 1 | 0 |  |  |  |  |  |  |  |  |
| GO:0048773\_erythrophore\_differentiation | 1 | 0 |  |  |  |  |  |  |  |  |
| GO:0048790\_maintenance\_of\_presynaptic\_active\_zone\_structure | 1 | 0 |  |  |  |  |  |  |  |  |
| GO:0048791\_calcium\_ion-dependent\_exocytosis\_of\_neurotransmitter | 1 | 0 |  |  |  |  |  |  |  |  |
| GO:0048822\_enucleate\_erythrocyte\_development | 1 | 0 |  |  |  |  |  |  |  |  |
| GO:0048866\_stem\_cell\_fate\_specification | 1 | 0 |  |  |  |  |  |  |  |  |
| GO:0048936\_peripheral\_nervous\_system\_neuron\_axonogenesis | 1 | 0 |  |  |  |  |  |  |  |  |
| GO:0050427\_3'-phosphoadenosine\_5'-phosphosulfate\_metabolic\_process | 1 | 0 |  |  |  |  |  |  |  |  |
| GO:0050428\_3'-phosphoadenosine\_5'-phosphosulfate\_biosynthetic\_process | 1 | 0 |  |  |  |  |  |  |  |  |
| GO:0050482\_arachidonic\_acid\_secretion | 1 | 0 |  |  |  |  |  |  |  |  |
| GO:0050667\_homocysteine\_metabolic\_process | 1 | 0 |  |  |  |  |  |  |  |  |
| GO:0050674\_urothelial\_cell\_proliferation | 1 | 0 |  |  |  |  |  |  |  |  |
| GO:0050675\_regulation\_of\_urothelial\_cell\_proliferation | 1 | 0 |  |  |  |  |  |  |  |  |
| GO:0050677\_positive\_regulation\_of\_urothelial\_cell\_proliferation | 1 | 0 |  |  |  |  |  |  |  |  |
| GO:0050691\_regulation\_of\_defense\_response\_to\_virus\_by\_host | 1 | 0 |  |  |  |  |  |  |  |  |
| GO:0050748\_negative\_regulation\_of\_lipoprotein\_metabolic\_process | 1 | 0 |  |  |  |  |  |  |  |  |
| GO:0050757\_thymidylate\_synthase\_biosynthetic\_process | 1 | 0 |  |  |  |  |  |  |  |  |
| GO:0050758\_regulation\_of\_thymidylate\_synthase\_biosynthetic\_process | 1 | 0 |  |  |  |  |  |  |  |  |
| GO:0050760\_negative\_regulation\_of\_thymidylate\_synthase\_biosynthetic\_process | 1 | 0 |  |  |  |  |  |  |  |  |
| GO:0050812\_regulation\_of\_acyl-CoA\_biosynthetic\_process | 1 | 0 |  |  |  |  |  |  |  |  |
| GO:0050832\_defense\_response\_to\_fungus | 1 | 0 |  |  |  |  |  |  |  |  |
| GO:0050861\_positive\_regulation\_of\_B\_cell\_receptor\_signaling\_pathway | 1 | 0 |  |  |  |  |  |  |  |  |
| GO:0050862\_positive\_regulation\_of\_T\_cell\_receptor\_signaling\_pathway | 1 | 0 |  |  |  |  |  |  |  |  |
| GO:0050916\_sensory\_perception\_of\_sweet\_taste | 1 | 0 |  |  |  |  |  |  |  |  |
| GO:0050975\_sensory\_perception\_of\_touch | 1 | 0 |  |  |  |  |  |  |  |  |
| GO:0050995\_negative\_regulation\_of\_lipid\_catabolic\_process | 1 | 0 |  |  |  |  |  |  |  |  |
| GO:0051001\_negative\_regulation\_of\_nitric-oxide\_synthase\_activity | 1 | 0 |  |  |  |  |  |  |  |  |
| GO:0051005\_negative\_regulation\_of\_lipoprotein\_lipase\_activity | 1 | 0 |  |  |  |  |  |  |  |  |
| GO:0051006\_positive\_regulation\_of\_lipoprotein\_lipase\_activity | 1 | 0 |  |  |  |  |  |  |  |  |
| GO:0051016\_barbed-end\_actin\_filament\_capping | 1 | 0 |  |  |  |  |  |  |  |  |
| GO:0051029\_rRNA\_transport | 1 | 0 |  |  |  |  |  |  |  |  |
| GO:0051043\_regulation\_of\_membrane\_protein\_ectodomain\_proteolysis | 1 | 0 |  |  |  |  |  |  |  |  |
| GO:0051044\_positive\_regulation\_of\_membrane\_protein\_ectodomain\_proteolysis | 1 | 0 |  |  |  |  |  |  |  |  |
| GO:0051088\_PMA-inducible\_membrane\_protein\_ectodomain\_proteolysis | 1 | 0 |  |  |  |  |  |  |  |  |
| GO:0051102\_DNA\_ligation\_during\_DNA\_recombination | 1 | 0 |  |  |  |  |  |  |  |  |
| GO:0051103\_DNA\_ligation\_during\_DNA\_repair | 1 | 0 |  |  |  |  |  |  |  |  |
| GO:0051123\_transcriptional\_preinitiation\_complex\_assembly | 1 | 0 |  |  |  |  |  |  |  |  |
| GO:0051125\_regulation\_of\_actin\_nucleation | 1 | 0 |  |  |  |  |  |  |  |  |
| GO:0051127\_positive\_regulation\_of\_actin\_nucleation | 1 | 0 |  |  |  |  |  |  |  |  |
| GO:0051151\_negative\_regulation\_of\_smooth\_muscle\_cell\_differentiation | 1 | 0 |  |  |  |  |  |  |  |  |
| GO:0051154\_negative\_regulation\_of\_striated\_muscle\_cell\_differentiation | 1 | 0 |  |  |  |  |  |  |  |  |
| GO:0051155\_positive\_regulation\_of\_striated\_muscle\_cell\_differentiation | 1 | 0 |  |  |  |  |  |  |  |  |
| GO:0051156\_glucose\_6-phosphate\_metabolic\_process | 1 | 0 |  |  |  |  |  |  |  |  |
| GO:0051187\_cofactor\_catabolic\_process | 1 | 0 |  |  |  |  |  |  |  |  |
| GO:0051189\_prosthetic\_group\_metabolic\_process | 1 | 0 |  |  |  |  |  |  |  |  |
| GO:0051193\_regulation\_of\_cofactor\_metabolic\_process | 1 | 0 |  |  |  |  |  |  |  |  |
| GO:0051196\_regulation\_of\_coenzyme\_metabolic\_process | 1 | 0 |  |  |  |  |  |  |  |  |
| GO:0051255\_spindle\_midzone\_assembly | 1 | 0 |  |  |  |  |  |  |  |  |
| GO:0051257\_spindle\_midzone\_assembly\_involved\_in\_meiosis | 1 | 0 |  |  |  |  |  |  |  |  |
| GO:0051281\_positive\_regulation\_of\_release\_of\_sequestered\_calcium\_ion\_into\_cytosol | 1 | 0 |  |  |  |  |  |  |  |  |
| GO:0051290\_protein\_heterotetramerization | 1 | 0 |  |  |  |  |  |  |  |  |
| GO:0051305\_chromosome\_movement\_towards\_spindle\_pole | 1 | 0 |  |  |  |  |  |  |  |  |
| GO:0051310\_metaphase\_plate\_congression | 1 | 0 |  |  |  |  |  |  |  |  |
| GO:0051311\_meiotic\_metaphase\_plate\_congression | 1 | 0 |  |  |  |  |  |  |  |  |
| GO:0051340\_regulation\_of\_ligase\_activity | 1 | 0 |  |  |  |  |  |  |  |  |
| GO:0051351\_positive\_regulation\_of\_ligase\_activity | 1 | 0 |  |  |  |  |  |  |  |  |
| GO:0051354\_negative\_regulation\_of\_oxidoreductase\_activity | 1 | 0 |  |  |  |  |  |  |  |  |
| GO:0051355\_proprioception\_during\_equilibrioception | 1 | 0 |  |  |  |  |  |  |  |  |
| GO:0051383\_kinetochore\_organization | 1 | 0 |  |  |  |  |  |  |  |  |
| GO:0051386\_regulation\_of\_nerve\_growth\_factor\_receptor\_signaling\_pathway | 1 | 0 |  |  |  |  |  |  |  |  |
| GO:0051409\_response\_to\_nitrosative\_stress | 1 | 0 |  |  |  |  |  |  |  |  |
| GO:0051457\_maintenance\_of\_protein\_location\_in\_nucleus | 1 | 0 |  |  |  |  |  |  |  |  |
| GO:0051462\_regulation\_of\_cortisol\_secretion | 1 | 0 |  |  |  |  |  |  |  |  |
| GO:0051463\_negative\_regulation\_of\_cortisol\_secretion | 1 | 0 |  |  |  |  |  |  |  |  |
| GO:0051481\_reduction\_of\_cytosolic\_calcium\_ion\_concentration | 1 | 0 |  |  |  |  |  |  |  |  |
| GO:0051482\_elevation\_of\_cytosolic\_calcium\_ion\_concentration\_during\_G-protein\_signaling\_\_coupled\_to\_IP3\_second\_messenger\_(phospholipase\_C\_activating) | 1 | 0 |  |  |  |  |  |  |  |  |
| GO:0051542\_elastin\_biosynthetic\_process | 1 | 0 |  |  |  |  |  |  |  |  |
| GO:0051568\_histone\_H3-K4\_methylation | 1 | 0 |  |  |  |  |  |  |  |  |
| GO:0051569\_regulation\_of\_histone\_H3-K4\_methylation | 1 | 0 |  |  |  |  |  |  |  |  |
| GO:0051570\_regulation\_of\_histone\_H3-K9\_methylation | 1 | 0 |  |  |  |  |  |  |  |  |
| GO:0051573\_negative\_regulation\_of\_histone\_H3-K9\_methylation | 1 | 0 |  |  |  |  |  |  |  |  |
| GO:0051580\_regulation\_of\_neurotransmitter\_uptake | 1 | 0 |  |  |  |  |  |  |  |  |
| GO:0051582\_positive\_regulation\_of\_neurotransmitter\_uptake | 1 | 0 |  |  |  |  |  |  |  |  |
| GO:0051584\_regulation\_of\_dopamine\_uptake | 1 | 0 |  |  |  |  |  |  |  |  |
| GO:0051586\_positive\_regulation\_of\_dopamine\_uptake | 1 | 0 |  |  |  |  |  |  |  |  |
| GO:0051589\_negative\_regulation\_of\_neurotransmitter\_transport | 1 | 0 |  |  |  |  |  |  |  |  |
| GO:0051593\_response\_to\_folic\_acid | 1 | 0 |  |  |  |  |  |  |  |  |
| GO:0051615\_histamine\_uptake | 1 | 0 |  |  |  |  |  |  |  |  |
| GO:0051646\_mitochondrion\_localization | 1 | 0 |  |  |  |  |  |  |  |  |
| GO:0051654\_establishment\_of\_mitochondrion\_localization | 1 | 0 |  |  |  |  |  |  |  |  |
| GO:0051661\_maintenance\_of\_centrosome\_location | 1 | 0 |  |  |  |  |  |  |  |  |
| GO:0051665\_membrane\_raft\_localization | 1 | 0 |  |  |  |  |  |  |  |  |
| GO:0051685\_maintenance\_of\_ER\_location | 1 | 0 |  |  |  |  |  |  |  |  |
| GO:0051693\_actin\_filament\_capping | 1 | 0 |  |  |  |  |  |  |  |  |
| GO:0051701\_interaction\_with\_host | 1 | 0 |  |  |  |  |  |  |  |  |
| GO:0051754\_meiotic\_sister\_chromatid\_cohesion\_\_centromeric | 1 | 0 |  |  |  |  |  |  |  |  |
| GO:0051782\_negative\_regulation\_of\_cell\_division | 1 | 0 |  |  |  |  |  |  |  |  |
| GO:0051790\_short-chain\_fatty\_acid\_biosynthetic\_process | 1 | 0 |  |  |  |  |  |  |  |  |
| GO:0051799\_negative\_regulation\_of\_hair\_follicle\_development | 1 | 0 |  |  |  |  |  |  |  |  |
| GO:0051823\_regulation\_of\_synapse\_structural\_plasticity | 1 | 0 |  |  |  |  |  |  |  |  |
| GO:0051865\_protein\_autoubiquitination | 1 | 0 |  |  |  |  |  |  |  |  |
| GO:0051901\_positive\_regulation\_of\_mitochondrial\_depolarization | 1 | 0 |  |  |  |  |  |  |  |  |
| GO:0051917\_regulation\_of\_fibrinolysis | 1 | 0 |  |  |  |  |  |  |  |  |
| GO:0051918\_negative\_regulation\_of\_fibrinolysis | 1 | 0 |  |  |  |  |  |  |  |  |
| GO:0051929\_positive\_regulation\_of\_calcium\_ion\_transport\_via\_voltage-gated\_calcium\_channel\_activity | 1 | 0 |  |  |  |  |  |  |  |  |
| GO:0051933\_amino\_acid\_uptake\_during\_transmission\_of\_nerve\_impulse | 1 | 0 |  |  |  |  |  |  |  |  |
| GO:0051935\_glutamate\_uptake\_during\_transmission\_of\_nerve\_impulse | 1 | 0 |  |  |  |  |  |  |  |  |
| GO:0051940\_regulation\_of\_catecholamine\_uptake\_during\_transmission\_of\_nerve\_impulse | 1 | 0 |  |  |  |  |  |  |  |  |
| GO:0051944\_positive\_regulation\_of\_catecholamine\_uptake\_during\_transmission\_of\_nerve\_impulse | 1 | 0 |  |  |  |  |  |  |  |  |
| GO:0051961\_negative\_regulation\_of\_nervous\_system\_development | 1 | 0 |  |  |  |  |  |  |  |  |
| GO:0051964\_negative\_regulation\_of\_synaptogenesis | 1 | 0 |  |  |  |  |  |  |  |  |
| GO:0051968\_positive\_regulation\_of\_synaptic\_transmission\_\_glutamatergic | 1 | 0 |  |  |  |  |  |  |  |  |
| GO:0051984\_positive\_regulation\_of\_chromosome\_segregation | 1 | 0 |  |  |  |  |  |  |  |  |
| GO:0051987\_positive\_regulation\_of\_attachment\_of\_spindle\_microtubules\_to\_kinetochore | 1 | 0 |  |  |  |  |  |  |  |  |
| GO:0052173\_response\_to\_defenses\_of\_other\_organism\_during\_symbiotic\_interaction | 1 | 0 |  |  |  |  |  |  |  |  |
| GO:0052200\_response\_to\_host\_defenses | 1 | 0 |  |  |  |  |  |  |  |  |
| GO:0052551\_response\_to\_defense-related\_nitric\_oxide\_production\_by\_other\_organism\_during\_symbiotic\_interaction | 1 | 0 |  |  |  |  |  |  |  |  |
| GO:0052564\_response\_to\_immune\_response\_of\_other\_organism\_during\_symbiotic\_interaction | 1 | 0 |  |  |  |  |  |  |  |  |
| GO:0052565\_response\_to\_defense-related\_host\_nitric\_oxide\_production | 1 | 0 |  |  |  |  |  |  |  |  |
| GO:0052572\_response\_to\_host\_immune\_response | 1 | 0 |  |  |  |  |  |  |  |  |
| GO:0055011\_atrial\_cardiac\_muscle\_cell\_differentiation | 1 | 0 |  |  |  |  |  |  |  |  |
| GO:0055014\_atrial\_cardiac\_muscle\_cell\_development | 1 | 0 |  |  |  |  |  |  |  |  |
| GO:0055078\_sodium\_ion\_homeostasis | 1 | 0 |  |  |  |  |  |  |  |  |
| GO:0055089\_fatty\_acid\_homeostasis | 1 | 0 |  |  |  |  |  |  |  |  |
| GO:0055093\_response\_to\_hyperoxia | 1 | 0 |  |  |  |  |  |  |  |  |
| GO:0060003\_copper\_ion\_export | 1 | 0 |  |  |  |  |  |  |  |  |
| GO:0060005\_vestibular\_reflex | 1 | 0 |  |  |  |  |  |  |  |  |
| GO:0060014\_granulosa\_cell\_differentiation | 1 | 0 |  |  |  |  |  |  |  |  |
| GO:0060018\_astrocyte\_fate\_commitment | 1 | 0 |  |  |  |  |  |  |  |  |
| GO:0060020\_Bergmann\_glial\_cell\_differentiation | 1 | 0 |  |  |  |  |  |  |  |  |
| GO:0060022\_hard\_palate\_development | 1 | 0 |  |  |  |  |  |  |  |  |
| GO:0060034\_notochord\_cell\_differentiation | 1 | 0 |  |  |  |  |  |  |  |  |
| GO:0060035\_notochord\_cell\_development | 1 | 0 |  |  |  |  |  |  |  |  |
| GO:0060046\_regulation\_of\_acrosome\_reaction | 1 | 0 |  |  |  |  |  |  |  |  |
| GO:0060054\_positive\_regulation\_of\_epithelial\_cell\_proliferation\_involved\_in\_wound\_healing | 1 | 0 |  |  |  |  |  |  |  |  |
| GO:0060061\_Spemann\_organizer\_formation | 1 | 0 |  |  |  |  |  |  |  |  |
| GO:0060064\_Spemann\_organizer\_formation\_at\_the\_anterior\_end\_of\_the\_primitive\_streak | 1 | 0 |  |  |  |  |  |  |  |  |
| GO:0060071\_Wnt\_receptor\_signaling\_pathway\_\_planar\_cell\_polarity\_pathway | 1 | 0 |  |  |  |  |  |  |  |  |
| GO:0060075\_regulation\_of\_resting\_membrane\_potential | 1 | 0 |  |  |  |  |  |  |  |  |
| GO:0060082\_eye\_blink\_reflex | 1 | 0 |  |  |  |  |  |  |  |  |
| GO:0060112\_generation\_of\_ovulation\_cycle\_rhythm | 1 | 0 |  |  |  |  |  |  |  |  |
| GO:0060125\_negative\_regulation\_of\_growth\_hormone\_secretion | 1 | 0 |  |  |  |  |  |  |  |  |
| GO:0060151\_peroxisome\_localization | 1 | 0 |  |  |  |  |  |  |  |  |
| GO:0060152\_microtubule-based\_peroxisome\_localization | 1 | 0 |  |  |  |  |  |  |  |  |
| GO:0060161\_positive\_regulation\_of\_dopamine\_receptor\_signaling\_pathway | 1 | 0 |  |  |  |  |  |  |  |  |
| GO:0060163\_subpallium\_neuron\_fate\_commitment | 1 | 0 |  |  |  |  |  |  |  |  |
| GO:0060165\_regulation\_of\_timing\_of\_subpallium\_neuron\_differentiation | 1 | 0 |  |  |  |  |  |  |  |  |
| GO:0060174\_limb\_bud\_formation | 1 | 0 |  |  |  |  |  |  |  |  |
| GO:0060177\_regulation\_of\_angiotensin\_metabolic\_process | 1 | 0 |  |  |  |  |  |  |  |  |
| GO:0060197\_cloacal\_septation | 1 | 0 |  |  |  |  |  |  |  |  |
| GO:0060215\_primitive\_hemopoiesis | 1 | 0 |  |  |  |  |  |  |  |  |
| GO:0060231\_mesenchymal\_to\_epithelial\_transition | 1 | 0 |  |  |  |  |  |  |  |  |
| GO:0060254\_regulation\_of\_N-terminal\_protein\_palmitoylation | 1 | 0 |  |  |  |  |  |  |  |  |
| GO:0060261\_positive\_regulation\_of\_transcription\_initiation\_from\_RNA\_polymerase\_II\_promoter | 1 | 0 |  |  |  |  |  |  |  |  |
| GO:0060262\_negative\_regulation\_of\_N-terminal\_protein\_palmitoylation | 1 | 0 |  |  |  |  |  |  |  |  |
| GO:0060263\_regulation\_of\_respiratory\_burst | 1 | 0 |  |  |  |  |  |  |  |  |
| GO:0060264\_regulation\_of\_respiratory\_burst\_during\_acute\_inflammatory\_response | 1 | 0 |  |  |  |  |  |  |  |  |
| GO:0060265\_positive\_regulation\_of\_respiratory\_burst\_during\_acute\_inflammatory\_response | 1 | 0 |  |  |  |  |  |  |  |  |
| GO:0060267\_positive\_regulation\_of\_respiratory\_burst | 1 | 0 |  |  |  |  |  |  |  |  |
| GO:0060272\_embryonic\_skeletal\_joint\_morphogenesis | 1 | 0 |  |  |  |  |  |  |  |  |
| GO:0060315\_negative\_regulation\_of\_ryanodine-sensitive\_calcium-release\_channel\_activity | 1 | 0 |  |  |  |  |  |  |  |  |
| GO:0060319\_primitive\_erythrocyte\_differentiation | 1 | 0 |  |  |  |  |  |  |  |  |
| GO:0060371\_regulation\_of\_atrial\_cardiomyocyte\_membrane\_depolarization | 1 | 0 |  |  |  |  |  |  |  |  |
| GO:0060374\_mast\_cell\_differentiation | 1 | 0 |  |  |  |  |  |  |  |  |
| GO:0060375\_regulation\_of\_mast\_cell\_differentiation | 1 | 0 |  |  |  |  |  |  |  |  |
| GO:0060376\_positive\_regulation\_of\_mast\_cell\_differentiation | 1 | 0 |  |  |  |  |  |  |  |  |
| GO:0060390\_regulation\_of\_SMAD\_protein\_nuclear\_translocation | 1 | 0 |  |  |  |  |  |  |  |  |
| GO:0060391\_positive\_regulation\_of\_SMAD\_protein\_nuclear\_translocation | 1 | 0 |  |  |  |  |  |  |  |  |
| GO:0060398\_regulation\_of\_growth\_hormone\_receptor\_signaling\_pathway | 1 | 0 |  |  |  |  |  |  |  |  |
| GO:0060399\_positive\_regulation\_of\_growth\_hormone\_receptor\_signaling\_pathway | 1 | 0 |  |  |  |  |  |  |  |  |
| GO:0060405\_regulation\_of\_penile\_erection | 1 | 0 |  |  |  |  |  |  |  |  |
| GO:0060407\_negative\_regulation\_of\_penile\_erection | 1 | 0 |  |  |  |  |  |  |  |  |
| GO:0060413\_atrial\_septum\_morphogenesis | 1 | 0 |  |  |  |  |  |  |  |  |
| GO:0060431\_primary\_lung\_bud\_formation | 1 | 0 |  |  |  |  |  |  |  |  |
| GO:0060436\_bronchiole\_morphogenesis | 1 | 0 |  |  |  |  |  |  |  |  |
| GO:0060440\_trachea\_formation | 1 | 0 |  |  |  |  |  |  |  |  |
| GO:0060449\_bud\_elongation\_involved\_in\_lung\_branching | 1 | 0 |  |  |  |  |  |  |  |  |
| GO:0060456\_positive\_regulation\_of\_digestive\_system\_process | 1 | 0 |  |  |  |  |  |  |  |  |
| GO:0060461\_right\_lung\_morphogenesis | 1 | 0 |  |  |  |  |  |  |  |  |
| GO:0060481\_lobar\_bronchus\_epithelium\_development | 1 | 0 |  |  |  |  |  |  |  |  |
| GO:0060482\_lobar\_bronchus\_development | 1 | 0 |  |  |  |  |  |  |  |  |
| GO:0060484\_lung-associated\_mesenchyme\_development | 1 | 0 |  |  |  |  |  |  |  |  |
| GO:0060486\_Clara\_cell\_differentiation | 1 | 0 |  |  |  |  |  |  |  |  |
| GO:0060510\_Type\_II\_pneumocyte\_differentiation | 1 | 0 |  |  |  |  |  |  |  |  |
| GO:0060514\_prostate\_induction | 1 | 0 |  |  |  |  |  |  |  |  |
| GO:0060515\_prostate\_field\_specification | 1 | 0 |  |  |  |  |  |  |  |  |
| GO:0060517\_epithelial\_cell\_proliferation\_involved\_in\_prostatic\_bud\_elongation | 1 | 0 |  |  |  |  |  |  |  |  |
| GO:0060520\_activation\_of\_prostate\_induction\_by\_androgen\_receptor\_signaling\_pathway | 1 | 0 |  |  |  |  |  |  |  |  |
| GO:0060535\_trachea\_cartilage\_morphogenesis | 1 | 0 |  |  |  |  |  |  |  |  |
| GO:0060536\_cartilage\_morphogenesis | 1 | 0 |  |  |  |  |  |  |  |  |
| GO:0060563\_neuroepithelial\_cell\_differentiation | 1 | 0 |  |  |  |  |  |  |  |  |
| GO:0060577\_pulmonary\_vein\_morphogenesis | 1 | 0 |  |  |  |  |  |  |  |  |
| GO:0060578\_superior\_vena\_cava\_morphogenesis | 1 | 0 |  |  |  |  |  |  |  |  |
| GO:0060584\_regulation\_of\_prostaglandin-endoperoxide\_synthase\_activity | 1 | 0 |  |  |  |  |  |  |  |  |
| GO:0060585\_positive\_regulation\_of\_prostaglandin-endoperoxidase\_synthase\_activity | 1 | 0 |  |  |  |  |  |  |  |  |
| GO:0060598\_dichotomous\_subdivision\_of\_terminal\_units\_involved\_in\_mammary\_gland\_duct\_morphogenesis | 1 | 0 |  |  |  |  |  |  |  |  |
| GO:0060611\_mammary\_gland\_fat\_development | 1 | 0 |  |  |  |  |  |  |  |  |
| GO:0060618\_nipple\_development | 1 | 0 |  |  |  |  |  |  |  |  |
| GO:0060631\_regulation\_of\_meiosis\_I | 1 | 0 |  |  |  |  |  |  |  |  |
| GO:0060649\_mammary\_gland\_bud\_elongation | 1 | 0 |  |  |  |  |  |  |  |  |
| GO:0060658\_nipple\_morphogenesis | 1 | 0 |  |  |  |  |  |  |  |  |
| GO:0060659\_nipple\_sheath\_formation | 1 | 0 |  |  |  |  |  |  |  |  |
| GO:0060668\_regulation\_of\_branching\_involved\_in\_salivary\_gland\_morphogenesis\_by\_extracellular\_matrix-epithelial\_cell\_signaling | 1 | 0 |  |  |  |  |  |  |  |  |
| GO:0060683\_regulation\_of\_branching\_involved\_in\_salivary\_gland\_morphogenesis\_by\_epithelial-mesenchymal\_signaling | 1 | 0 |  |  |  |  |  |  |  |  |
| GO:0060691\_epithelial\_cell\_maturation\_involved\_in\_salivary\_gland\_development | 1 | 0 |  |  |  |  |  |  |  |  |
| GO:0060709\_glycogen\_cell\_development\_involved\_in\_embryonic\_placenta\_development | 1 | 0 |  |  |  |  |  |  |  |  |
| GO:0060732\_positive\_regulation\_of\_inositol\_phosphate\_biosynthetic\_process | 1 | 0 |  |  |  |  |  |  |  |  |
| GO:0060739\_mesenchymal-epithelial\_cell\_signaling\_involved\_in\_prostate\_gland\_development | 1 | 0 |  |  |  |  |  |  |  |  |
| GO:0060781\_mesenchymal\_cell\_proliferation\_involved\_in\_prostate\_gland\_development | 1 | 0 |  |  |  |  |  |  |  |  |
| GO:0060782\_regulation\_of\_mesenchymal\_cell\_proliferation\_involved\_in\_prostate\_gland\_development | 1 | 0 |  |  |  |  |  |  |  |  |
| GO:0060783\_mesenchymal\_smoothened\_signaling\_pathway\_involved\_in\_prostate\_gland\_development | 1 | 0 |  |  |  |  |  |  |  |  |
| GO:0060872\_semicircular\_canal\_development | 1 | 0 |  |  |  |  |  |  |  |  |
| GO:0060896\_neural\_plate\_pattern\_specification | 1 | 0 |  |  |  |  |  |  |  |  |
| GO:0070091\_glucagon\_secretion | 1 | 0 |  |  |  |  |  |  |  |  |
| GO:0070162\_adiponectin\_secretion | 1 | 0 |  |  |  |  |  |  |  |  |
| GO:0070163\_regulation\_of\_adiponectin\_secretion | 1 | 0 |  |  |  |  |  |  |  |  |
| GO:0070164\_negative\_regulation\_of\_adiponectin\_secretion | 1 | 0 |  |  |  |  |  |  |  |  |
| GO:0070178\_D-serine\_metabolic\_process | 1 | 0 |  |  |  |  |  |  |  |  |
| GO:0070179\_D-serine\_biosynthetic\_process | 1 | 0 |  |  |  |  |  |  |  |  |
| GO:0070296\_sarcoplasmic\_reticulum\_calcium\_ion\_transport | 1 | 0 |  |  |  |  |  |  |  |  |
| GO:0070303\_negative\_regulation\_of\_stress-activated\_protein\_kinase\_signaling\_pathway | 1 | 0 |  |  |  |  |  |  |  |  |
| GO:0070328\_triglyceride\_homeostasis | 1 | 0 |  |  |  |  |  |  |  |  |
| GO:0070365\_hepatocyte\_differentiation | 1 | 0 |  |  |  |  |  |  |  |  |
| GO:0070384\_Harderian\_gland\_development | 1 | 0 |  |  |  |  |  |  |  |  |
| GO:0070391\_response\_to\_lipoteichoic\_acid | 1 | 0 |  |  |  |  |  |  |  |  |
| GO:0070424\_regulation\_of\_nucleotide-binding\_oligomerization\_domain\_containing\_signaling\_pathway | 1 | 0 |  |  |  |  |  |  |  |  |
| GO:0070426\_positive\_regulation\_of\_nucleotide-binding\_oligomerization\_domain\_containing\_signaling\_pathway | 1 | 0 |  |  |  |  |  |  |  |  |
| GO:0070428\_regulation\_of\_nucleotide-binding\_oligomerization\_domain\_containing\_1\_signaling\_pathway | 1 | 0 |  |  |  |  |  |  |  |  |
| GO:0070430\_positive\_regulation\_of\_nucleotide-binding\_oligomerization\_domain\_containing\_1\_signaling\_pathway | 1 | 0 |  |  |  |  |  |  |  |  |
| GO:0070432\_regulation\_of\_nucleotide-binding\_oligomerization\_domain\_containing\_2\_signaling\_pathway | 1 | 0 |  |  |  |  |  |  |  |  |
| GO:0070434\_positive\_regulation\_of\_nucleotide-binding\_oligomerization\_domain\_containing\_2\_signaling\_pathway | 1 | 0 |  |  |  |  |  |  |  |  |
| GO:0070508\_cholesterol\_import | 1 | 0 |  |  |  |  |  |  |  |  |
| GO:0070555\_response\_to\_interleukin-1 | 1 | 0 |  |  |  |  |  |  |  |  |
| GO:0070561\_vitamin\_D\_receptor\_signaling\_pathway | 1 | 0 |  |  |  |  |  |  |  |  |
| GO:0070562\_regulation\_of\_vitamin\_D\_receptor\_signaling\_pathway | 1 | 0 |  |  |  |  |  |  |  |  |
| GO:0070571\_negative\_regulation\_of\_neuron\_projection\_regeneration | 1 | 0 |  |  |  |  |  |  |  |  |
| GO:0070572\_positive\_regulation\_of\_neuron\_projection\_regeneration | 1 | 0 |  |  |  |  |  |  |  |  |
| GO:0070613\_regulation\_of\_protein\_processing | 1 | 0 |  |  |  |  |  |  |  |  |
| GO:0070627\_ferrous\_iron\_import | 1 | 0 |  |  |  |  |  |  |  |  |
| GO:0070669\_response\_to\_interleukin-2 | 1 | 0 |  |  |  |  |  |  |  |  |
| GO:0070670\_response\_to\_interleukin-4 | 1 | 0 |  |  |  |  |  |  |  |  |
| GO:0070671\_response\_to\_interleukin-12 | 1 | 0 |  |  |  |  |  |  |  |  |
| GO:0070672\_response\_to\_interleukin-15 | 1 | 0 |  |  |  |  |  |  |  |  |
| GO:0070673\_response\_to\_interleukin-18 | 1 | 0 |  |  |  |  |  |  |  |  |
| GO:0070828\_heterochromatin\_organization | 1 | 0 |  |  |  |  |  |  |  |  |
| GO:0070874\_negative\_regulation\_of\_glycogen\_metabolic\_process | 1 | 0 |  |  |  |  |  |  |  |  |
| GO:0075136\_response\_to\_host | 1 | 0 |  |  |  |  |  |  |  |  |
| GO:0080010\_regulation\_of\_oxygen\_and\_reactive\_oxygen\_species\_metabolic\_process | 1 | 0 |  |  |  |  |  |  |  |  |
| GO:0090032\_negative\_regulation\_of\_steroid\_hormone\_biosynthetic\_process | 1 | 0 |  |  |  |  |  |  |  |  |
| GO:0001505\_regulation\_of\_neurotransmitter\_levels | 48 | 0 | 0.000000 | -0.000000 | 1809 | 1604.966915 | 1674.9 | 1744.833085 | 0.925871 |  |
| GO:0009101\_glycoprotein\_biosynthetic\_process | 48 | 0 | 0.000000 | -0.000000 | 1809 | 1604.966915 | 1674.9 | 1744.833085 | 0.925871 |  |
| GO:0034504\_protein\_localization\_in\_nucleus | 48 | 0 | 0.000000 | -0.000000 | 1809 | 1604.966915 | 1674.9 | 1744.833085 | 0.925871 |  |
| GO:0006511\_ubiquitin-dependent\_protein\_catabolic\_process | 39 | 0 | 0.000000 | -0.000000 | 1814 | 1615.299678 | 1684.39 | 1753.480322 | 0.928550 |  |
| GO:0006644\_phospholipid\_metabolic\_process | 39 | 0 | 0.000000 | -0.000000 | 1814 | 1615.299678 | 1684.39 | 1753.480322 | 0.928550 |  |
| GO:0021953\_central\_nervous\_system\_neuron\_differentiation | 39 | 0 | 0.000000 | -0.000000 | 1814 | 1615.299678 | 1684.39 | 1753.480322 | 0.928550 |  |
| GO:0031279\_regulation\_of\_cyclase\_activity | 39 | 0 | 0.000000 | -0.000000 | 1814 | 1615.299678 | 1684.39 | 1753.480322 | 0.928550 |  |
| GO:0051339\_regulation\_of\_lyase\_activity | 39 | 0 | 0.000000 | -0.000000 | 1814 | 1615.299678 | 1684.39 | 1753.480322 | 0.928550 |  |
| GO:0001759\_induction\_of\_an\_organ | 15 | 0 | 0.000000 | -0.000000 | 1857 | 1663.897232 | 1730.78 | 1797.662768 | 0.932030 |  |
| GO:0001782\_B\_cell\_homeostasis | 15 | 0 | 0.000000 | -0.000000 | 1857 | 1663.897232 | 1730.78 | 1797.662768 | 0.932030 |  |
| GO:0001964\_startle\_response | 15 | 0 | 0.000000 | -0.000000 | 1857 | 1663.897232 | 1730.78 | 1797.662768 | 0.932030 |  |
| GO:0002286\_T\_cell\_activation\_during\_immune\_response | 15 | 0 | 0.000000 | -0.000000 | 1857 | 1663.897232 | 1730.78 | 1797.662768 | 0.932030 |  |
| GO:0002495\_antigen\_processing\_and\_presentation\_of\_peptide\_antigen\_via\_MHC\_class\_II | 15 | 0 | 0.000000 | -0.000000 | 1857 | 1663.897232 | 1730.78 | 1797.662768 | 0.932030 |  |
| GO:0002504\_antigen\_processing\_and\_presentation\_of\_peptide\_or\_polysaccharide\_antigen\_via\_MHC\_class\_II | 15 | 0 | 0.000000 | -0.000000 | 1857 | 1663.897232 | 1730.78 | 1797.662768 | 0.932030 |  |
| GO:0002709\_regulation\_of\_T\_cell\_mediated\_immunity | 15 | 0 | 0.000000 | -0.000000 | 1857 | 1663.897232 | 1730.78 | 1797.662768 | 0.932030 |  |
| GO:0006473\_protein\_amino\_acid\_acetylation | 15 | 0 | 0.000000 | -0.000000 | 1857 | 1663.897232 | 1730.78 | 1797.662768 | 0.932030 |  |
| GO:0006487\_protein\_amino\_acid\_N-linked\_glycosylation | 15 | 0 | 0.000000 | -0.000000 | 1857 | 1663.897232 | 1730.78 | 1797.662768 | 0.932030 |  |
| GO:0006749\_glutathione\_metabolic\_process | 15 | 0 | 0.000000 | -0.000000 | 1857 | 1663.897232 | 1730.78 | 1797.662768 | 0.932030 |  |
| GO:0006885\_regulation\_of\_pH | 15 | 0 | 0.000000 | -0.000000 | 1857 | 1663.897232 | 1730.78 | 1797.662768 | 0.932030 |  |
| GO:0007040\_lysosome\_organization | 15 | 0 | 0.000000 | -0.000000 | 1857 | 1663.897232 | 1730.78 | 1797.662768 | 0.932030 |  |
| GO:0007173\_epidermal\_growth\_factor\_receptor\_signaling\_pathway | 15 | 0 | 0.000000 | -0.000000 | 1857 | 1663.897232 | 1730.78 | 1797.662768 | 0.932030 |  |
| GO:0007200\_activation\_of\_phospholipase\_C\_activity\_by\_G-protein\_coupled\_receptor\_protein\_signaling\_pathway\_coupled\_to\_IP3\_second\_messenger | 15 | 0 | 0.000000 | -0.000000 | 1857 | 1663.897232 | 1730.78 | 1797.662768 | 0.932030 |  |
| GO:0007202\_activation\_of\_phospholipase\_C\_activity | 15 | 0 | 0.000000 | -0.000000 | 1857 | 1663.897232 | 1730.78 | 1797.662768 | 0.932030 |  |
| GO:0007218\_neuropeptide\_signaling\_pathway | 15 | 0 | 0.000000 | -0.000000 | 1857 | 1663.897232 | 1730.78 | 1797.662768 | 0.932030 |  |
| GO:0007588\_excretion | 15 | 0 | 0.000000 | -0.000000 | 1857 | 1663.897232 | 1730.78 | 1797.662768 | 0.932030 |  |
| GO:0007618\_mating | 15 | 0 | 0.000000 | -0.000000 | 1857 | 1663.897232 | 1730.78 | 1797.662768 | 0.932030 |  |
| GO:0008543\_fibroblast\_growth\_factor\_receptor\_signaling\_pathway | 15 | 0 | 0.000000 | -0.000000 | 1857 | 1663.897232 | 1730.78 | 1797.662768 | 0.932030 |  |
| GO:0009062\_fatty\_acid\_catabolic\_process | 15 | 0 | 0.000000 | -0.000000 | 1857 | 1663.897232 | 1730.78 | 1797.662768 | 0.932030 |  |
| GO:0009116\_nucleoside\_metabolic\_process | 15 | 0 | 0.000000 | -0.000000 | 1857 | 1663.897232 | 1730.78 | 1797.662768 | 0.932030 |  |
| GO:0010092\_specification\_of\_organ\_identity | 15 | 0 | 0.000000 | -0.000000 | 1857 | 1663.897232 | 1730.78 | 1797.662768 | 0.932030 |  |
| GO:0010518\_positive\_regulation\_of\_phospholipase\_activity | 15 | 0 | 0.000000 | -0.000000 | 1857 | 1663.897232 | 1730.78 | 1797.662768 | 0.932030 |  |
| GO:0010863\_positive\_regulation\_of\_phospholipase\_C\_activity | 15 | 0 | 0.000000 | -0.000000 | 1857 | 1663.897232 | 1730.78 | 1797.662768 | 0.932030 |  |
| GO:0015931\_nucleobase\_\_nucleoside\_\_nucleotide\_and\_nucleic\_acid\_transport | 15 | 0 | 0.000000 | -0.000000 | 1857 | 1663.897232 | 1730.78 | 1797.662768 | 0.932030 |  |
| GO:0019886\_antigen\_processing\_and\_presentation\_of\_exogenous\_peptide\_antigen\_via\_MHC\_class\_II | 15 | 0 | 0.000000 | -0.000000 | 1857 | 1663.897232 | 1730.78 | 1797.662768 | 0.932030 |  |
| GO:0021872\_generation\_of\_neurons\_in\_the\_forebrain | 15 | 0 | 0.000000 | -0.000000 | 1857 | 1663.897232 | 1730.78 | 1797.662768 | 0.932030 |  |
| GO:0022600\_digestive\_system\_process | 15 | 0 | 0.000000 | -0.000000 | 1857 | 1663.897232 | 1730.78 | 1797.662768 | 0.932030 |  |
| GO:0030041\_actin\_filament\_polymerization | 15 | 0 | 0.000000 | -0.000000 | 1857 | 1663.897232 | 1730.78 | 1797.662768 | 0.932030 |  |
| GO:0031329\_regulation\_of\_cellular\_catabolic\_process | 15 | 0 | 0.000000 | -0.000000 | 1857 | 1663.897232 | 1730.78 | 1797.662768 | 0.932030 |  |
| GO:0035249\_synaptic\_transmission\_\_glutamatergic | 15 | 0 | 0.000000 | -0.000000 | 1857 | 1663.897232 | 1730.78 | 1797.662768 | 0.932030 |  |
| GO:0042306\_regulation\_of\_protein\_import\_into\_nucleus | 15 | 0 | 0.000000 | -0.000000 | 1857 | 1663.897232 | 1730.78 | 1797.662768 | 0.932030 |  |
| GO:0045666\_positive\_regulation\_of\_neuron\_differentiation | 15 | 0 | 0.000000 | -0.000000 | 1857 | 1663.897232 | 1730.78 | 1797.662768 | 0.932030 |  |
| GO:0046164\_alcohol\_catabolic\_process | 15 | 0 | 0.000000 | -0.000000 | 1857 | 1663.897232 | 1730.78 | 1797.662768 | 0.932030 |  |
| GO:0046638\_positive\_regulation\_of\_alpha-beta\_T\_cell\_differentiation | 15 | 0 | 0.000000 | -0.000000 | 1857 | 1663.897232 | 1730.78 | 1797.662768 | 0.932030 |  |
| GO:0048709\_oligodendrocyte\_differentiation | 15 | 0 | 0.000000 | -0.000000 | 1857 | 1663.897232 | 1730.78 | 1797.662768 | 0.932030 |  |
| GO:0050729\_positive\_regulation\_of\_inflammatory\_response | 15 | 0 | 0.000000 | -0.000000 | 1857 | 1663.897232 | 1730.78 | 1797.662768 | 0.932030 |  |
| GO:0050796\_regulation\_of\_insulin\_secretion | 15 | 0 | 0.000000 | -0.000000 | 1857 | 1663.897232 | 1730.78 | 1797.662768 | 0.932030 |  |
| GO:0050798\_activated\_T\_cell\_proliferation | 15 | 0 | 0.000000 | -0.000000 | 1857 | 1663.897232 | 1730.78 | 1797.662768 | 0.932030 |  |
| GO:0060425\_lung\_morphogenesis | 15 | 0 | 0.000000 | -0.000000 | 1857 | 1663.897232 | 1730.78 | 1797.662768 | 0.932030 |  |
| GO:0060442\_branching\_involved\_in\_prostate\_gland\_morphogenesis | 15 | 0 | 0.000000 | -0.000000 | 1857 | 1663.897232 | 1730.78 | 1797.662768 | 0.932030 |  |
| GO:0060749\_mammary\_gland\_alveolus\_development | 15 | 0 | 0.000000 | -0.000000 | 1857 | 1663.897232 | 1730.78 | 1797.662768 | 0.932030 |  |
| GO:0070507\_regulation\_of\_microtubule\_cytoskeleton\_organization | 15 | 0 | 0.000000 | -0.000000 | 1857 | 1663.897232 | 1730.78 | 1797.662768 | 0.932030 |  |
| GO:0051270\_regulation\_of\_cell\_motion | 73 | 0 | 0.000000 | -0.000000 | 1858 | 1666.214799 | 1732.85 | 1799.485201 | 0.932643 |  |
| GO:0019932\_second-messenger-mediated\_signaling | 68 | 0 | 0.000000 | -0.000000 | 1860 | 1668.382433 | 1734.67 | 1800.957567 | 0.932618 |  |
| GO:0034962\_cellular\_biopolymer\_catabolic\_process | 68 | 0 | 0.000000 | -0.000000 | 1860 | 1668.382433 | 1734.67 | 1800.957567 | 0.932618 |  |
| GO:0000375\_RNA\_splicing\_\_via\_transesterification\_reactions | 12 | 0 | 0.000000 | -0.000000 | 1925 | 1742.951773 | 1806.53 | 1870.108227 | 0.938457 |  |
| GO:0000377\_RNA\_splicing\_\_via\_transesterification\_reactions\_with\_bulged\_adenosine\_as\_nucleophile | 12 | 0 | 0.000000 | -0.000000 | 1925 | 1742.951773 | 1806.53 | 1870.108227 | 0.938457 |  |
| GO:0000398\_nuclear\_mRNA\_splicing\_\_via\_spliceosome | 12 | 0 | 0.000000 | -0.000000 | 1925 | 1742.951773 | 1806.53 | 1870.108227 | 0.938457 |  |
| GO:0001662\_behavioral\_fear\_response | 12 | 0 | 0.000000 | -0.000000 | 1925 | 1742.951773 | 1806.53 | 1870.108227 | 0.938457 |  |
| GO:0001738\_morphogenesis\_of\_a\_polarized\_epithelium | 12 | 0 | 0.000000 | -0.000000 | 1925 | 1742.951773 | 1806.53 | 1870.108227 | 0.938457 |  |
| GO:0002026\_regulation\_of\_the\_force\_of\_heart\_contraction | 12 | 0 | 0.000000 | -0.000000 | 1925 | 1742.951773 | 1806.53 | 1870.108227 | 0.938457 |  |
| GO:0002209\_behavioral\_defense\_response | 12 | 0 | 0.000000 | -0.000000 | 1925 | 1742.951773 | 1806.53 | 1870.108227 | 0.938457 |  |
| GO:0002218\_activation\_of\_innate\_immune\_response | 12 | 0 | 0.000000 | -0.000000 | 1925 | 1742.951773 | 1806.53 | 1870.108227 | 0.938457 |  |
| GO:0002437\_inflammatory\_response\_to\_antigenic\_stimulus | 12 | 0 | 0.000000 | -0.000000 | 1925 | 1742.951773 | 1806.53 | 1870.108227 | 0.938457 |  |
| GO:0002763\_positive\_regulation\_of\_myeloid\_leukocyte\_differentiation | 12 | 0 | 0.000000 | -0.000000 | 1925 | 1742.951773 | 1806.53 | 1870.108227 | 0.938457 |  |
| GO:0002820\_negative\_regulation\_of\_adaptive\_immune\_response | 12 | 0 | 0.000000 | -0.000000 | 1925 | 1742.951773 | 1806.53 | 1870.108227 | 0.938457 |  |
| GO:0002823\_negative\_regulation\_of\_adaptive\_immune\_response\_based\_on\_somatic\_recombination\_of\_immune\_receptors\_built\_from\_immunoglobulin\_superfamily\_domains | 12 | 0 | 0.000000 | -0.000000 | 1925 | 1742.951773 | 1806.53 | 1870.108227 | 0.938457 |  |
| GO:0002861\_regulation\_of\_inflammatory\_response\_to\_antigenic\_stimulus | 12 | 0 | 0.000000 | -0.000000 | 1925 | 1742.951773 | 1806.53 | 1870.108227 | 0.938457 |  |
| GO:0006308\_DNA\_catabolic\_process | 12 | 0 | 0.000000 | -0.000000 | 1925 | 1742.951773 | 1806.53 | 1870.108227 | 0.938457 |  |
| GO:0006403\_RNA\_localization | 12 | 0 | 0.000000 | -0.000000 | 1925 | 1742.951773 | 1806.53 | 1870.108227 | 0.938457 |  |
| GO:0006413\_translational\_initiation | 12 | 0 | 0.000000 | -0.000000 | 1925 | 1742.951773 | 1806.53 | 1870.108227 | 0.938457 |  |
| GO:0006446\_regulation\_of\_translational\_initiation | 12 | 0 | 0.000000 | -0.000000 | 1925 | 1742.951773 | 1806.53 | 1870.108227 | 0.938457 |  |
| GO:0006839\_mitochondrial\_transport | 12 | 0 | 0.000000 | -0.000000 | 1925 | 1742.951773 | 1806.53 | 1870.108227 | 0.938457 |  |
| GO:0006879\_cellular\_iron\_ion\_homeostasis | 12 | 0 | 0.000000 | -0.000000 | 1925 | 1742.951773 | 1806.53 | 1870.108227 | 0.938457 |  |
| GO:0007143\_female\_meiosis | 12 | 0 | 0.000000 | -0.000000 | 1925 | 1742.951773 | 1806.53 | 1870.108227 | 0.938457 |  |
| GO:0007340\_acrosome\_reaction | 12 | 0 | 0.000000 | -0.000000 | 1925 | 1742.951773 | 1806.53 | 1870.108227 | 0.938457 |  |
| GO:0008038\_neuron\_recognition | 12 | 0 | 0.000000 | -0.000000 | 1925 | 1742.951773 | 1806.53 | 1870.108227 | 0.938457 |  |
| GO:0008045\_motor\_axon\_guidance | 12 | 0 | 0.000000 | -0.000000 | 1925 | 1742.951773 | 1806.53 | 1870.108227 | 0.938457 |  |
| GO:0008277\_regulation\_of\_G-protein\_coupled\_receptor\_protein\_signaling\_pathway | 12 | 0 | 0.000000 | -0.000000 | 1925 | 1742.951773 | 1806.53 | 1870.108227 | 0.938457 |  |
| GO:0009262\_deoxyribonucleotide\_metabolic\_process | 12 | 0 | 0.000000 | -0.000000 | 1925 | 1742.951773 | 1806.53 | 1870.108227 | 0.938457 |  |
| GO:0009886\_post-embryonic\_morphogenesis | 12 | 0 | 0.000000 | -0.000000 | 1925 | 1742.951773 | 1806.53 | 1870.108227 | 0.938457 |  |
| GO:0015872\_dopamine\_transport | 12 | 0 | 0.000000 | -0.000000 | 1925 | 1742.951773 | 1806.53 | 1870.108227 | 0.938457 |  |
| GO:0021513\_spinal\_cord\_dorsal\_ventral\_patterning | 12 | 0 | 0.000000 | -0.000000 | 1925 | 1742.951773 | 1806.53 | 1870.108227 | 0.938457 |  |
| GO:0030010\_establishment\_of\_cell\_polarity | 12 | 0 | 0.000000 | -0.000000 | 1925 | 1742.951773 | 1806.53 | 1870.108227 | 0.938457 |  |
| GO:0030166\_proteoglycan\_biosynthetic\_process | 12 | 0 | 0.000000 | -0.000000 | 1925 | 1742.951773 | 1806.53 | 1870.108227 | 0.938457 |  |
| GO:0030225\_macrophage\_differentiation | 12 | 0 | 0.000000 | -0.000000 | 1925 | 1742.951773 | 1806.53 | 1870.108227 | 0.938457 |  |
| GO:0030262\_apoptotic\_nuclear\_changes | 12 | 0 | 0.000000 | -0.000000 | 1925 | 1742.951773 | 1806.53 | 1870.108227 | 0.938457 |  |
| GO:0030330\_DNA\_damage\_response\_\_signal\_transduction\_by\_p53\_class\_mediator | 12 | 0 | 0.000000 | -0.000000 | 1925 | 1742.951773 | 1806.53 | 1870.108227 | 0.938457 |  |
| GO:0030514\_negative\_regulation\_of\_BMP\_signaling\_pathway | 12 | 0 | 0.000000 | -0.000000 | 1925 | 1742.951773 | 1806.53 | 1870.108227 | 0.938457 |  |
| GO:0031056\_regulation\_of\_histone\_modification | 12 | 0 | 0.000000 | -0.000000 | 1925 | 1742.951773 | 1806.53 | 1870.108227 | 0.938457 |  |
| GO:0031109\_microtubule\_polymerization\_or\_depolymerization | 12 | 0 | 0.000000 | -0.000000 | 1925 | 1742.951773 | 1806.53 | 1870.108227 | 0.938457 |  |
| GO:0032755\_positive\_regulation\_of\_interleukin-6\_production | 12 | 0 | 0.000000 | -0.000000 | 1925 | 1742.951773 | 1806.53 | 1870.108227 | 0.938457 |  |
| GO:0042278\_purine\_nucleoside\_metabolic\_process | 12 | 0 | 0.000000 | -0.000000 | 1925 | 1742.951773 | 1806.53 | 1870.108227 | 0.938457 |  |
| GO:0042446\_hormone\_biosynthetic\_process | 12 | 0 | 0.000000 | -0.000000 | 1925 | 1742.951773 | 1806.53 | 1870.108227 | 0.938457 |  |
| GO:0042743\_hydrogen\_peroxide\_metabolic\_process | 12 | 0 | 0.000000 | -0.000000 | 1925 | 1742.951773 | 1806.53 | 1870.108227 | 0.938457 |  |
| GO:0043331\_response\_to\_dsRNA | 12 | 0 | 0.000000 | -0.000000 | 1925 | 1742.951773 | 1806.53 | 1870.108227 | 0.938457 |  |
| GO:0043392\_negative\_regulation\_of\_DNA\_binding | 12 | 0 | 0.000000 | -0.000000 | 1925 | 1742.951773 | 1806.53 | 1870.108227 | 0.938457 |  |
| GO:0043487\_regulation\_of\_RNA\_stability | 12 | 0 | 0.000000 | -0.000000 | 1925 | 1742.951773 | 1806.53 | 1870.108227 | 0.938457 |  |
| GO:0045076\_regulation\_of\_interleukin-2\_biosynthetic\_process | 12 | 0 | 0.000000 | -0.000000 | 1925 | 1742.951773 | 1806.53 | 1870.108227 | 0.938457 |  |
| GO:0045445\_myoblast\_differentiation | 12 | 0 | 0.000000 | -0.000000 | 1925 | 1742.951773 | 1806.53 | 1870.108227 | 0.938457 |  |
| GO:0045471\_response\_to\_ethanol | 12 | 0 | 0.000000 | -0.000000 | 1925 | 1742.951773 | 1806.53 | 1870.108227 | 0.938457 |  |
| GO:0045792\_negative\_regulation\_of\_cell\_size | 12 | 0 | 0.000000 | -0.000000 | 1925 | 1742.951773 | 1806.53 | 1870.108227 | 0.938457 |  |
| GO:0046128\_purine\_ribonucleoside\_metabolic\_process | 12 | 0 | 0.000000 | -0.000000 | 1925 | 1742.951773 | 1806.53 | 1870.108227 | 0.938457 |  |
| GO:0046330\_positive\_regulation\_of\_JNK\_cascade | 12 | 0 | 0.000000 | -0.000000 | 1925 | 1742.951773 | 1806.53 | 1870.108227 | 0.938457 |  |
| GO:0048169\_regulation\_of\_long-term\_neuronal\_synaptic\_plasticity | 12 | 0 | 0.000000 | -0.000000 | 1925 | 1742.951773 | 1806.53 | 1870.108227 | 0.938457 |  |
| GO:0048820\_hair\_follicle\_maturation | 12 | 0 | 0.000000 | -0.000000 | 1925 | 1742.951773 | 1806.53 | 1870.108227 | 0.938457 |  |
| GO:0050432\_catecholamine\_secretion | 12 | 0 | 0.000000 | -0.000000 | 1925 | 1742.951773 | 1806.53 | 1870.108227 | 0.938457 |  |
| GO:0050795\_regulation\_of\_behavior | 12 | 0 | 0.000000 | -0.000000 | 1925 | 1742.951773 | 1806.53 | 1870.108227 | 0.938457 |  |
| GO:0050803\_regulation\_of\_synapse\_structure\_and\_activity | 12 | 0 | 0.000000 | -0.000000 | 1925 | 1742.951773 | 1806.53 | 1870.108227 | 0.938457 |  |
| GO:0050848\_regulation\_of\_calcium-mediated\_signaling | 12 | 0 | 0.000000 | -0.000000 | 1925 | 1742.951773 | 1806.53 | 1870.108227 | 0.938457 |  |
| GO:0050850\_positive\_regulation\_of\_calcium-mediated\_signaling | 12 | 0 | 0.000000 | -0.000000 | 1925 | 1742.951773 | 1806.53 | 1870.108227 | 0.938457 |  |
| GO:0050853\_B\_cell\_receptor\_signaling\_pathway | 12 | 0 | 0.000000 | -0.000000 | 1925 | 1742.951773 | 1806.53 | 1870.108227 | 0.938457 |  |
| GO:0050869\_negative\_regulation\_of\_B\_cell\_activation | 12 | 0 | 0.000000 | -0.000000 | 1925 | 1742.951773 | 1806.53 | 1870.108227 | 0.938457 |  |
| GO:0051145\_smooth\_muscle\_cell\_differentiation | 12 | 0 | 0.000000 | -0.000000 | 1925 | 1742.951773 | 1806.53 | 1870.108227 | 0.938457 |  |
| GO:0051588\_regulation\_of\_neurotransmitter\_transport | 12 | 0 | 0.000000 | -0.000000 | 1925 | 1742.951773 | 1806.53 | 1870.108227 | 0.938457 |  |
| GO:0055114\_oxidation\_reduction | 12 | 0 | 0.000000 | -0.000000 | 1925 | 1742.951773 | 1806.53 | 1870.108227 | 0.938457 |  |
| GO:0060048\_cardiac\_muscle\_contraction | 12 | 0 | 0.000000 | -0.000000 | 1925 | 1742.951773 | 1806.53 | 1870.108227 | 0.938457 |  |
| GO:0060525\_prostate\_glandular\_acinus\_development | 12 | 0 | 0.000000 | -0.000000 | 1925 | 1742.951773 | 1806.53 | 1870.108227 | 0.938457 |  |
| GO:0060736\_prostate\_gland\_growth | 12 | 0 | 0.000000 | -0.000000 | 1925 | 1742.951773 | 1806.53 | 1870.108227 | 0.938457 |  |
| GO:0070304\_positive\_regulation\_of\_stress-activated\_protein\_kinase\_signaling\_pathway | 12 | 0 | 0.000000 | -0.000000 | 1925 | 1742.951773 | 1806.53 | 1870.108227 | 0.938457 |  |
| GO:0034622\_cellular\_macromolecular\_complex\_assembly | 58 | 0 | 0.000000 | -0.000000 | 1927 | 1745.233781 | 1808.45 | 1871.666219 | 0.938480 |  |
| GO:0050804\_regulation\_of\_synaptic\_transmission | 58 | 0 | 0.000000 | -0.000000 | 1927 | 1745.233781 | 1808.45 | 1871.666219 | 0.938480 |  |
| GO:0002695\_negative\_regulation\_of\_leukocyte\_activation | 38 | 0 | 0.000000 | -0.000000 | 1937 | 1759.143727 | 1821.27 | 1883.396273 | 0.940253 |  |
| GO:0006820\_anion\_transport | 38 | 0 | 0.000000 | -0.000000 | 1937 | 1759.143727 | 1821.27 | 1883.396273 | 0.940253 |  |
| GO:0008016\_regulation\_of\_heart\_contraction | 38 | 0 | 0.000000 | -0.000000 | 1937 | 1759.143727 | 1821.27 | 1883.396273 | 0.940253 |  |
| GO:0010975\_regulation\_of\_neuron\_projection\_development | 38 | 0 | 0.000000 | -0.000000 | 1937 | 1759.143727 | 1821.27 | 1883.396273 | 0.940253 |  |
| GO:0016053\_organic\_acid\_biosynthetic\_process | 38 | 0 | 0.000000 | -0.000000 | 1937 | 1759.143727 | 1821.27 | 1883.396273 | 0.940253 |  |
| GO:0045580\_regulation\_of\_T\_cell\_differentiation | 38 | 0 | 0.000000 | -0.000000 | 1937 | 1759.143727 | 1821.27 | 1883.396273 | 0.940253 |  |
| GO:0046394\_carboxylic\_acid\_biosynthetic\_process | 38 | 0 | 0.000000 | -0.000000 | 1937 | 1759.143727 | 1821.27 | 1883.396273 | 0.940253 |  |
| GO:0050727\_regulation\_of\_inflammatory\_response | 38 | 0 | 0.000000 | -0.000000 | 1937 | 1759.143727 | 1821.27 | 1883.396273 | 0.940253 |  |
| GO:0050866\_negative\_regulation\_of\_cell\_activation | 38 | 0 | 0.000000 | -0.000000 | 1937 | 1759.143727 | 1821.27 | 1883.396273 | 0.940253 |  |
| GO:0051348\_negative\_regulation\_of\_transferase\_activity | 38 | 0 | 0.000000 | -0.000000 | 1937 | 1759.143727 | 1821.27 | 1883.396273 | 0.940253 |  |
| GO:0006171\_cAMP\_biosynthetic\_process | 42 | 0 | 0.000000 | -0.000000 | 1946 | 1772.032345 | 1832.72 | 1893.407655 | 0.941788 |  |
| GO:0010769\_regulation\_of\_cell\_morphogenesis\_involved\_in\_differentiation | 42 | 0 | 0.000000 | -0.000000 | 1946 | 1772.032345 | 1832.72 | 1893.407655 | 0.941788 |  |
| GO:0019221\_cytokine-mediated\_signaling\_pathway | 42 | 0 | 0.000000 | -0.000000 | 1946 | 1772.032345 | 1832.72 | 1893.407655 | 0.941788 |  |
| GO:0019941\_modification-dependent\_protein\_catabolic\_process | 42 | 0 | 0.000000 | -0.000000 | 1946 | 1772.032345 | 1832.72 | 1893.407655 | 0.941788 |  |
| GO:0032946\_positive\_regulation\_of\_mononuclear\_cell\_proliferation | 42 | 0 | 0.000000 | -0.000000 | 1946 | 1772.032345 | 1832.72 | 1893.407655 | 0.941788 |  |
| GO:0043632\_modification-dependent\_macromolecule\_catabolic\_process | 42 | 0 | 0.000000 | -0.000000 | 1946 | 1772.032345 | 1832.72 | 1893.407655 | 0.941788 |  |
| GO:0050671\_positive\_regulation\_of\_lymphocyte\_proliferation | 42 | 0 | 0.000000 | -0.000000 | 1946 | 1772.032345 | 1832.72 | 1893.407655 | 0.941788 |  |
| GO:0051345\_positive\_regulation\_of\_hydrolase\_activity | 42 | 0 | 0.000000 | -0.000000 | 1946 | 1772.032345 | 1832.72 | 1893.407655 | 0.941788 |  |
| GO:0051603\_proteolysis\_involved\_in\_cellular\_protein\_catabolic\_process | 42 | 0 | 0.000000 | -0.000000 | 1946 | 1772.032345 | 1832.72 | 1893.407655 | 0.941788 |  |
| GO:0000209\_protein\_polyubiquitination | 10 | 0 | 0.000000 | -0.000000 | 2047 | 1885.606101 | 1943.26 | 2000.913899 | 0.949321 |  |
| GO:0000724\_double-strand\_break\_repair\_via\_homologous\_recombination | 10 | 0 | 0.000000 | -0.000000 | 2047 | 1885.606101 | 1943.26 | 2000.913899 | 0.949321 |  |
| GO:0000725\_recombinational\_repair | 10 | 0 | 0.000000 | -0.000000 | 2047 | 1885.606101 | 1943.26 | 2000.913899 | 0.949321 |  |
| GO:0001578\_microtubule\_bundle\_formation | 10 | 0 | 0.000000 | -0.000000 | 2047 | 1885.606101 | 1943.26 | 2000.913899 | 0.949321 |  |
| GO:0001659\_temperature\_homeostasis | 10 | 0 | 0.000000 | -0.000000 | 2047 | 1885.606101 | 1943.26 | 2000.913899 | 0.949321 |  |
| GO:0001773\_myeloid\_dendritic\_cell\_activation | 10 | 0 | 0.000000 | -0.000000 | 2047 | 1885.606101 | 1943.26 | 2000.913899 | 0.949321 |  |
| GO:0001832\_blastocyst\_growth | 10 | 0 | 0.000000 | -0.000000 | 2047 | 1885.606101 | 1943.26 | 2000.913899 | 0.949321 |  |
| GO:0001914\_regulation\_of\_T\_cell\_mediated\_cytotoxicity | 10 | 0 | 0.000000 | -0.000000 | 2047 | 1885.606101 | 1943.26 | 2000.913899 | 0.949321 |  |
| GO:0001990\_regulation\_of\_systemic\_arterial\_blood\_pressure\_by\_hormone | 10 | 0 | 0.000000 | -0.000000 | 2047 | 1885.606101 | 1943.26 | 2000.913899 | 0.949321 |  |
| GO:0002070\_epithelial\_cell\_maturation | 10 | 0 | 0.000000 | -0.000000 | 2047 | 1885.606101 | 1943.26 | 2000.913899 | 0.949321 |  |
| GO:0002673\_regulation\_of\_acute\_inflammatory\_response | 10 | 0 | 0.000000 | -0.000000 | 2047 | 1885.606101 | 1943.26 | 2000.913899 | 0.949321 |  |
| GO:0002711\_positive\_regulation\_of\_T\_cell\_mediated\_immunity | 10 | 0 | 0.000000 | -0.000000 | 2047 | 1885.606101 | 1943.26 | 2000.913899 | 0.949321 |  |
| GO:0006040\_amino\_sugar\_metabolic\_process | 10 | 0 | 0.000000 | -0.000000 | 2047 | 1885.606101 | 1943.26 | 2000.913899 | 0.949321 |  |
| GO:0006109\_regulation\_of\_carbohydrate\_metabolic\_process | 10 | 0 | 0.000000 | -0.000000 | 2047 | 1885.606101 | 1943.26 | 2000.913899 | 0.949321 |  |
| GO:0006289\_nucleotide-excision\_repair | 10 | 0 | 0.000000 | -0.000000 | 2047 | 1885.606101 | 1943.26 | 2000.913899 | 0.949321 |  |
| GO:0006342\_chromatin\_silencing | 10 | 0 | 0.000000 | -0.000000 | 2047 | 1885.606101 | 1943.26 | 2000.913899 | 0.949321 |  |
| GO:0006405\_RNA\_export\_from\_nucleus | 10 | 0 | 0.000000 | -0.000000 | 2047 | 1885.606101 | 1943.26 | 2000.913899 | 0.949321 |  |
| GO:0006801\_superoxide\_metabolic\_process | 10 | 0 | 0.000000 | -0.000000 | 2047 | 1885.606101 | 1943.26 | 2000.913899 | 0.949321 |  |
| GO:0006805\_xenobiotic\_metabolic\_process | 10 | 0 | 0.000000 | -0.000000 | 2047 | 1885.606101 | 1943.26 | 2000.913899 | 0.949321 |  |
| GO:0006826\_iron\_ion\_transport | 10 | 0 | 0.000000 | -0.000000 | 2047 | 1885.606101 | 1943.26 | 2000.913899 | 0.949321 |  |
| GO:0006921\_cell\_structure\_disassembly\_during\_apoptosis | 10 | 0 | 0.000000 | -0.000000 | 2047 | 1885.606101 | 1943.26 | 2000.913899 | 0.949321 |  |
| GO:0006968\_cellular\_defense\_response | 10 | 0 | 0.000000 | -0.000000 | 2047 | 1885.606101 | 1943.26 | 2000.913899 | 0.949321 |  |
| GO:0007006\_mitochondrial\_membrane\_organization | 10 | 0 | 0.000000 | -0.000000 | 2047 | 1885.606101 | 1943.26 | 2000.913899 | 0.949321 |  |
| GO:0007044\_cell-substrate\_junction\_assembly | 10 | 0 | 0.000000 | -0.000000 | 2047 | 1885.606101 | 1943.26 | 2000.913899 | 0.949321 |  |
| GO:0007093\_mitotic\_cell\_cycle\_checkpoint | 10 | 0 | 0.000000 | -0.000000 | 2047 | 1885.606101 | 1943.26 | 2000.913899 | 0.949321 |  |
| GO:0007172\_signal\_complex\_assembly | 10 | 0 | 0.000000 | -0.000000 | 2047 | 1885.606101 | 1943.26 | 2000.913899 | 0.949321 |  |
| GO:0007194\_negative\_regulation\_of\_adenylate\_cyclase\_activity | 10 | 0 | 0.000000 | -0.000000 | 2047 | 1885.606101 | 1943.26 | 2000.913899 | 0.949321 |  |
| GO:0008088\_axon\_cargo\_transport | 10 | 0 | 0.000000 | -0.000000 | 2047 | 1885.606101 | 1943.26 | 2000.913899 | 0.949321 |  |
| GO:0008206\_bile\_acid\_metabolic\_process | 10 | 0 | 0.000000 | -0.000000 | 2047 | 1885.606101 | 1943.26 | 2000.913899 | 0.949321 |  |
| GO:0008211\_glucocorticoid\_metabolic\_process | 10 | 0 | 0.000000 | -0.000000 | 2047 | 1885.606101 | 1943.26 | 2000.913899 | 0.949321 |  |
| GO:0009066\_aspartate\_family\_amino\_acid\_metabolic\_process | 10 | 0 | 0.000000 | -0.000000 | 2047 | 1885.606101 | 1943.26 | 2000.913899 | 0.949321 |  |
| GO:0009110\_vitamin\_biosynthetic\_process | 10 | 0 | 0.000000 | -0.000000 | 2047 | 1885.606101 | 1943.26 | 2000.913899 | 0.949321 |  |
| GO:0009620\_response\_to\_fungus | 10 | 0 | 0.000000 | -0.000000 | 2047 | 1885.606101 | 1943.26 | 2000.913899 | 0.949321 |  |
| GO:0009743\_response\_to\_carbohydrate\_stimulus | 10 | 0 | 0.000000 | -0.000000 | 2047 | 1885.606101 | 1943.26 | 2000.913899 | 0.949321 |  |
| GO:0010827\_regulation\_of\_glucose\_transport | 10 | 0 | 0.000000 | -0.000000 | 2047 | 1885.606101 | 1943.26 | 2000.913899 | 0.949321 |  |
| GO:0015718\_monocarboxylic\_acid\_transport | 10 | 0 | 0.000000 | -0.000000 | 2047 | 1885.606101 | 1943.26 | 2000.913899 | 0.949321 |  |
| GO:0016197\_endosome\_transport | 10 | 0 | 0.000000 | -0.000000 | 2047 | 1885.606101 | 1943.26 | 2000.913899 | 0.949321 |  |
| GO:0016486\_peptide\_hormone\_processing | 10 | 0 | 0.000000 | -0.000000 | 2047 | 1885.606101 | 1943.26 | 2000.913899 | 0.949321 |  |
| GO:0017156\_calcium\_ion-dependent\_exocytosis | 10 | 0 | 0.000000 | -0.000000 | 2047 | 1885.606101 | 1943.26 | 2000.913899 | 0.949321 |  |
| GO:0019321\_pentose\_metabolic\_process | 10 | 0 | 0.000000 | -0.000000 | 2047 | 1885.606101 | 1943.26 | 2000.913899 | 0.949321 |  |
| GO:0021534\_cell\_proliferation\_in\_hindbrain | 10 | 0 | 0.000000 | -0.000000 | 2047 | 1885.606101 | 1943.26 | 2000.913899 | 0.949321 |  |
| GO:0021871\_forebrain\_regionalization | 10 | 0 | 0.000000 | -0.000000 | 2047 | 1885.606101 | 1943.26 | 2000.913899 | 0.949321 |  |
| GO:0021895\_cerebral\_cortex\_neuron\_differentiation | 10 | 0 | 0.000000 | -0.000000 | 2047 | 1885.606101 | 1943.26 | 2000.913899 | 0.949321 |  |
| GO:0021924\_cell\_proliferation\_in\_the\_external\_granule\_layer | 10 | 0 | 0.000000 | -0.000000 | 2047 | 1885.606101 | 1943.26 | 2000.913899 | 0.949321 |  |
| GO:0021930\_granule\_cell\_precursor\_proliferation | 10 | 0 | 0.000000 | -0.000000 | 2047 | 1885.606101 | 1943.26 | 2000.913899 | 0.949321 |  |
| GO:0021952\_central\_nervous\_system\_projection\_neuron\_axonogenesis | 10 | 0 | 0.000000 | -0.000000 | 2047 | 1885.606101 | 1943.26 | 2000.913899 | 0.949321 |  |
| GO:0022900\_electron\_transport\_chain | 10 | 0 | 0.000000 | -0.000000 | 2047 | 1885.606101 | 1943.26 | 2000.913899 | 0.949321 |  |
| GO:0022904\_respiratory\_electron\_transport\_chain | 10 | 0 | 0.000000 | -0.000000 | 2047 | 1885.606101 | 1943.26 | 2000.913899 | 0.949321 |  |
| GO:0030833\_regulation\_of\_actin\_filament\_polymerization | 10 | 0 | 0.000000 | -0.000000 | 2047 | 1885.606101 | 1943.26 | 2000.913899 | 0.949321 |  |
| GO:0031280\_negative\_regulation\_of\_cyclase\_activity | 10 | 0 | 0.000000 | -0.000000 | 2047 | 1885.606101 | 1943.26 | 2000.913899 | 0.949321 |  |
| GO:0031331\_positive\_regulation\_of\_cellular\_catabolic\_process | 10 | 0 | 0.000000 | -0.000000 | 2047 | 1885.606101 | 1943.26 | 2000.913899 | 0.949321 |  |
| GO:0031645\_negative\_regulation\_of\_neurological\_system\_process | 10 | 0 | 0.000000 | -0.000000 | 2047 | 1885.606101 | 1943.26 | 2000.913899 | 0.949321 |  |
| GO:0032318\_regulation\_of\_Ras\_GTPase\_activity | 10 | 0 | 0.000000 | -0.000000 | 2047 | 1885.606101 | 1943.26 | 2000.913899 | 0.949321 |  |
| GO:0032602\_chemokine\_production | 10 | 0 | 0.000000 | -0.000000 | 2047 | 1885.606101 | 1943.26 | 2000.913899 | 0.949321 |  |
| GO:0032633\_interleukin-4\_production | 10 | 0 | 0.000000 | -0.000000 | 2047 | 1885.606101 | 1943.26 | 2000.913899 | 0.949321 |  |
| GO:0032642\_regulation\_of\_chemokine\_production | 10 | 0 | 0.000000 | -0.000000 | 2047 | 1885.606101 | 1943.26 | 2000.913899 | 0.949321 |  |
| GO:0032673\_regulation\_of\_interleukin-4\_production | 10 | 0 | 0.000000 | -0.000000 | 2047 | 1885.606101 | 1943.26 | 2000.913899 | 0.949321 |  |
| GO:0032760\_positive\_regulation\_of\_tumor\_necrosis\_factor\_production | 10 | 0 | 0.000000 | -0.000000 | 2047 | 1885.606101 | 1943.26 | 2000.913899 | 0.949321 |  |
| GO:0033081\_regulation\_of\_T\_cell\_differentiation\_in\_the\_thymus | 10 | 0 | 0.000000 | -0.000000 | 2047 | 1885.606101 | 1943.26 | 2000.913899 | 0.949321 |  |
| GO:0034637\_cellular\_carbohydrate\_biosynthetic\_process | 10 | 0 | 0.000000 | -0.000000 | 2047 | 1885.606101 | 1943.26 | 2000.913899 | 0.949321 |  |
| GO:0040015\_negative\_regulation\_of\_multicellular\_organism\_growth | 10 | 0 | 0.000000 | -0.000000 | 2047 | 1885.606101 | 1943.26 | 2000.913899 | 0.949321 |  |
| GO:0042088\_T-helper\_1\_type\_immune\_response | 10 | 0 | 0.000000 | -0.000000 | 2047 | 1885.606101 | 1943.26 | 2000.913899 | 0.949321 |  |
| GO:0042177\_negative\_regulation\_of\_protein\_catabolic\_process | 10 | 0 | 0.000000 | -0.000000 | 2047 | 1885.606101 | 1943.26 | 2000.913899 | 0.949321 |  |
| GO:0042755\_eating\_behavior | 10 | 0 | 0.000000 | -0.000000 | 2047 | 1885.606101 | 1943.26 | 2000.913899 | 0.949321 |  |
| GO:0043113\_receptor\_clustering | 10 | 0 | 0.000000 | -0.000000 | 2047 | 1885.606101 | 1943.26 | 2000.913899 | 0.949321 |  |
| GO:0043330\_response\_to\_exogenous\_dsRNA | 10 | 0 | 0.000000 | -0.000000 | 2047 | 1885.606101 | 1943.26 | 2000.913899 | 0.949321 |  |
| GO:0043488\_regulation\_of\_mRNA\_stability | 10 | 0 | 0.000000 | -0.000000 | 2047 | 1885.606101 | 1943.26 | 2000.913899 | 0.949321 |  |
| GO:0043525\_positive\_regulation\_of\_neuron\_apoptosis | 10 | 0 | 0.000000 | -0.000000 | 2047 | 1885.606101 | 1943.26 | 2000.913899 | 0.949321 |  |
| GO:0044259\_multicellular\_organismal\_macromolecule\_metabolic\_process | 10 | 0 | 0.000000 | -0.000000 | 2047 | 1885.606101 | 1943.26 | 2000.913899 | 0.949321 |  |
| GO:0045132\_meiotic\_chromosome\_segregation | 10 | 0 | 0.000000 | -0.000000 | 2047 | 1885.606101 | 1943.26 | 2000.913899 | 0.949321 |  |
| GO:0045576\_mast\_cell\_activation | 10 | 0 | 0.000000 | -0.000000 | 2047 | 1885.606101 | 1943.26 | 2000.913899 | 0.949321 |  |
| GO:0045776\_negative\_regulation\_of\_blood\_pressure | 10 | 0 | 0.000000 | -0.000000 | 2047 | 1885.606101 | 1943.26 | 2000.913899 | 0.949321 |  |
| GO:0045777\_positive\_regulation\_of\_blood\_pressure | 10 | 0 | 0.000000 | -0.000000 | 2047 | 1885.606101 | 1943.26 | 2000.913899 | 0.949321 |  |
| GO:0045814\_negative\_regulation\_of\_gene\_expression\_\_epigenetic | 10 | 0 | 0.000000 | -0.000000 | 2047 | 1885.606101 | 1943.26 | 2000.913899 | 0.949321 |  |
| GO:0045911\_positive\_regulation\_of\_DNA\_recombination | 10 | 0 | 0.000000 | -0.000000 | 2047 | 1885.606101 | 1943.26 | 2000.913899 | 0.949321 |  |
| GO:0046887\_positive\_regulation\_of\_hormone\_secretion | 10 | 0 | 0.000000 | -0.000000 | 2047 | 1885.606101 | 1943.26 | 2000.913899 | 0.949321 |  |
| GO:0048291\_isotype\_switching\_to\_IgG\_isotypes | 10 | 0 | 0.000000 | -0.000000 | 2047 | 1885.606101 | 1943.26 | 2000.913899 | 0.949321 |  |
| GO:0048302\_regulation\_of\_isotype\_switching\_to\_IgG\_isotypes | 10 | 0 | 0.000000 | -0.000000 | 2047 | 1885.606101 | 1943.26 | 2000.913899 | 0.949321 |  |
| GO:0048384\_retinoic\_acid\_receptor\_signaling\_pathway | 10 | 0 | 0.000000 | -0.000000 | 2047 | 1885.606101 | 1943.26 | 2000.913899 | 0.949321 |  |
| GO:0048641\_regulation\_of\_skeletal\_muscle\_tissue\_development | 10 | 0 | 0.000000 | -0.000000 | 2047 | 1885.606101 | 1943.26 | 2000.913899 | 0.949321 |  |
| GO:0050657\_nucleic\_acid\_transport | 10 | 0 | 0.000000 | -0.000000 | 2047 | 1885.606101 | 1943.26 | 2000.913899 | 0.949321 |  |
| GO:0050658\_RNA\_transport | 10 | 0 | 0.000000 | -0.000000 | 2047 | 1885.606101 | 1943.26 | 2000.913899 | 0.949321 |  |
| GO:0050663\_cytokine\_secretion | 10 | 0 | 0.000000 | -0.000000 | 2047 | 1885.606101 | 1943.26 | 2000.913899 | 0.949321 |  |
| GO:0050879\_multicellular\_organismal\_movement | 10 | 0 | 0.000000 | -0.000000 | 2047 | 1885.606101 | 1943.26 | 2000.913899 | 0.949321 |  |
| GO:0050881\_musculoskeletal\_movement | 10 | 0 | 0.000000 | -0.000000 | 2047 | 1885.606101 | 1943.26 | 2000.913899 | 0.949321 |  |
| GO:0050886\_endocrine\_process | 10 | 0 | 0.000000 | -0.000000 | 2047 | 1885.606101 | 1943.26 | 2000.913899 | 0.949321 |  |
| GO:0050892\_intestinal\_absorption | 10 | 0 | 0.000000 | -0.000000 | 2047 | 1885.606101 | 1943.26 | 2000.913899 | 0.949321 |  |
| GO:0051208\_sequestering\_of\_calcium\_ion | 10 | 0 | 0.000000 | -0.000000 | 2047 | 1885.606101 | 1943.26 | 2000.913899 | 0.949321 |  |
| GO:0051209\_release\_of\_sequestered\_calcium\_ion\_into\_cytosol | 10 | 0 | 0.000000 | -0.000000 | 2047 | 1885.606101 | 1943.26 | 2000.913899 | 0.949321 |  |
| GO:0051224\_negative\_regulation\_of\_protein\_transport | 10 | 0 | 0.000000 | -0.000000 | 2047 | 1885.606101 | 1943.26 | 2000.913899 | 0.949321 |  |
| GO:0051236\_establishment\_of\_RNA\_localization | 10 | 0 | 0.000000 | -0.000000 | 2047 | 1885.606101 | 1943.26 | 2000.913899 | 0.949321 |  |
| GO:0051238\_sequestering\_of\_metal\_ion | 10 | 0 | 0.000000 | -0.000000 | 2047 | 1885.606101 | 1943.26 | 2000.913899 | 0.949321 |  |
| GO:0051262\_protein\_tetramerization | 10 | 0 | 0.000000 | -0.000000 | 2047 | 1885.606101 | 1943.26 | 2000.913899 | 0.949321 |  |
| GO:0051282\_regulation\_of\_sequestering\_of\_calcium\_ion | 10 | 0 | 0.000000 | -0.000000 | 2047 | 1885.606101 | 1943.26 | 2000.913899 | 0.949321 |  |
| GO:0051283\_negative\_regulation\_of\_sequestering\_of\_calcium\_ion | 10 | 0 | 0.000000 | -0.000000 | 2047 | 1885.606101 | 1943.26 | 2000.913899 | 0.949321 |  |
| GO:0051350\_negative\_regulation\_of\_lyase\_activity | 10 | 0 | 0.000000 | -0.000000 | 2047 | 1885.606101 | 1943.26 | 2000.913899 | 0.949321 |  |
| GO:0051445\_regulation\_of\_meiotic\_cell\_cycle | 10 | 0 | 0.000000 | -0.000000 | 2047 | 1885.606101 | 1943.26 | 2000.913899 | 0.949321 |  |
| GO:0051650\_establishment\_of\_vesicle\_localization | 10 | 0 | 0.000000 | -0.000000 | 2047 | 1885.606101 | 1943.26 | 2000.913899 | 0.949321 |  |
| GO:0060135\_maternal\_process\_involved\_in\_female\_pregnancy | 10 | 0 | 0.000000 | -0.000000 | 2047 | 1885.606101 | 1943.26 | 2000.913899 | 0.949321 |  |
| GO:0060216\_definitive\_hemopoiesis | 10 | 0 | 0.000000 | -0.000000 | 2047 | 1885.606101 | 1943.26 | 2000.913899 | 0.949321 |  |
| GO:0060706\_cell\_differentiation\_involved\_in\_embryonic\_placenta\_development | 10 | 0 | 0.000000 | -0.000000 | 2047 | 1885.606101 | 1943.26 | 2000.913899 | 0.949321 |  |
| GO:0043086\_negative\_regulation\_of\_catalytic\_activity | 65 | 0 | 0.000000 | -0.000000 | 2048 | 1887.058253 | 1944.56 | 2002.061747 | 0.949492 |  |
| GO:0002705\_positive\_regulation\_of\_leukocyte\_mediated\_immunity | 28 | 0 | 0.000000 | -0.000000 | 2060 | 1901.405284 | 1957.67 | 2013.934716 | 0.950325 |  |
| GO:0002708\_positive\_regulation\_of\_lymphocyte\_mediated\_immunity | 28 | 0 | 0.000000 | -0.000000 | 2060 | 1901.405284 | 1957.67 | 2013.934716 | 0.950325 |  |
| GO:0006470\_protein\_amino\_acid\_dephosphorylation | 28 | 0 | 0.000000 | -0.000000 | 2060 | 1901.405284 | 1957.67 | 2013.934716 | 0.950325 |  |
| GO:0006997\_nucleus\_organization | 28 | 0 | 0.000000 | -0.000000 | 2060 | 1901.405284 | 1957.67 | 2013.934716 | 0.950325 |  |
| GO:0007127\_meiosis\_I | 28 | 0 | 0.000000 | -0.000000 | 2060 | 1901.405284 | 1957.67 | 2013.934716 | 0.950325 |  |
| GO:0007585\_respiratory\_gaseous\_exchange | 28 | 0 | 0.000000 | -0.000000 | 2060 | 1901.405284 | 1957.67 | 2013.934716 | 0.950325 |  |
| GO:0030073\_insulin\_secretion | 28 | 0 | 0.000000 | -0.000000 | 2060 | 1901.405284 | 1957.67 | 2013.934716 | 0.950325 |  |
| GO:0042100\_B\_cell\_proliferation | 28 | 0 | 0.000000 | -0.000000 | 2060 | 1901.405284 | 1957.67 | 2013.934716 | 0.950325 |  |
| GO:0045926\_negative\_regulation\_of\_growth | 28 | 0 | 0.000000 | -0.000000 | 2060 | 1901.405284 | 1957.67 | 2013.934716 | 0.950325 |  |
| GO:0048863\_stem\_cell\_differentiation | 28 | 0 | 0.000000 | -0.000000 | 2060 | 1901.405284 | 1957.67 | 2013.934716 | 0.950325 |  |
| GO:0050871\_positive\_regulation\_of\_B\_cell\_activation | 28 | 0 | 0.000000 | -0.000000 | 2060 | 1901.405284 | 1957.67 | 2013.934716 | 0.950325 |  |
| GO:0051188\_cofactor\_biosynthetic\_process | 28 | 0 | 0.000000 | -0.000000 | 2060 | 1901.405284 | 1957.67 | 2013.934716 | 0.950325 |  |
| GO:0006486\_protein\_amino\_acid\_glycosylation | 31 | 0 | 0.000000 | -0.000000 | 2079 | 1925.505638 | 1979.74 | 2033.974362 | 0.952256 |  |
| GO:0006639\_acylglycerol\_metabolic\_process | 31 | 0 | 0.000000 | -0.000000 | 2079 | 1925.505638 | 1979.74 | 2033.974362 | 0.952256 |  |
| GO:0006665\_sphingolipid\_metabolic\_process | 31 | 0 | 0.000000 | -0.000000 | 2079 | 1925.505638 | 1979.74 | 2033.974362 | 0.952256 |  |
| GO:0008645\_hexose\_transport | 31 | 0 | 0.000000 | -0.000000 | 2079 | 1925.505638 | 1979.74 | 2033.974362 | 0.952256 |  |
| GO:0015749\_monosaccharide\_transport | 31 | 0 | 0.000000 | -0.000000 | 2079 | 1925.505638 | 1979.74 | 2033.974362 | 0.952256 |  |
| GO:0015758\_glucose\_transport | 31 | 0 | 0.000000 | -0.000000 | 2079 | 1925.505638 | 1979.74 | 2033.974362 | 0.952256 |  |
| GO:0016049\_cell\_growth | 31 | 0 | 0.000000 | -0.000000 | 2079 | 1925.505638 | 1979.74 | 2033.974362 | 0.952256 |  |
| GO:0016311\_dephosphorylation | 31 | 0 | 0.000000 | -0.000000 | 2079 | 1925.505638 | 1979.74 | 2033.974362 | 0.952256 |  |
| GO:0021954\_central\_nervous\_system\_neuron\_development | 31 | 0 | 0.000000 | -0.000000 | 2079 | 1925.505638 | 1979.74 | 2033.974362 | 0.952256 |  |
| GO:0033555\_multicellular\_organismal\_response\_to\_stress | 31 | 0 | 0.000000 | -0.000000 | 2079 | 1925.505638 | 1979.74 | 2033.974362 | 0.952256 |  |
| GO:0043269\_regulation\_of\_ion\_transport | 31 | 0 | 0.000000 | -0.000000 | 2079 | 1925.505638 | 1979.74 | 2033.974362 | 0.952256 |  |
| GO:0043413\_biopolymer\_glycosylation | 31 | 0 | 0.000000 | -0.000000 | 2079 | 1925.505638 | 1979.74 | 2033.974362 | 0.952256 |  |
| GO:0045088\_regulation\_of\_innate\_immune\_response | 31 | 0 | 0.000000 | -0.000000 | 2079 | 1925.505638 | 1979.74 | 2033.974362 | 0.952256 |  |
| GO:0046632\_alpha-beta\_T\_cell\_differentiation | 31 | 0 | 0.000000 | -0.000000 | 2079 | 1925.505638 | 1979.74 | 2033.974362 | 0.952256 |  |
| GO:0048167\_regulation\_of\_synaptic\_plasticity | 31 | 0 | 0.000000 | -0.000000 | 2079 | 1925.505638 | 1979.74 | 2033.974362 | 0.952256 |  |
| GO:0050868\_negative\_regulation\_of\_T\_cell\_activation | 31 | 0 | 0.000000 | -0.000000 | 2079 | 1925.505638 | 1979.74 | 2033.974362 | 0.952256 |  |
| GO:0051640\_organelle\_localization | 31 | 0 | 0.000000 | -0.000000 | 2079 | 1925.505638 | 1979.74 | 2033.974362 | 0.952256 |  |
| GO:0051899\_membrane\_depolarization | 31 | 0 | 0.000000 | -0.000000 | 2079 | 1925.505638 | 1979.74 | 2033.974362 | 0.952256 |  |
| GO:0070085\_glycosylation | 31 | 0 | 0.000000 | -0.000000 | 2079 | 1925.505638 | 1979.74 | 2033.974362 | 0.952256 |  |
| GO:0001819\_positive\_regulation\_of\_cytokine\_production | 36 | 0 | 0.000000 | -0.000000 | 2090 | 1938.381407 | 1991.37 | 2044.358593 | 0.952809 |  |
| GO:0006469\_negative\_regulation\_of\_protein\_kinase\_activity | 36 | 0 | 0.000000 | -0.000000 | 2090 | 1938.381407 | 1991.37 | 2044.358593 | 0.952809 |  |
| GO:0007187\_G-protein\_signaling\_\_coupled\_to\_cyclic\_nucleotide\_second\_messenger | 36 | 0 | 0.000000 | -0.000000 | 2090 | 1938.381407 | 1991.37 | 2044.358593 | 0.952809 |  |
| GO:0007631\_feeding\_behavior | 36 | 0 | 0.000000 | -0.000000 | 2090 | 1938.381407 | 1991.37 | 2044.358593 | 0.952809 |  |
| GO:0019228\_regulation\_of\_action\_potential\_in\_neuron | 36 | 0 | 0.000000 | -0.000000 | 2090 | 1938.381407 | 1991.37 | 2044.358593 | 0.952809 |  |
| GO:0021510\_spinal\_cord\_development | 36 | 0 | 0.000000 | -0.000000 | 2090 | 1938.381407 | 1991.37 | 2044.358593 | 0.952809 |  |
| GO:0030072\_peptide\_hormone\_secretion | 36 | 0 | 0.000000 | -0.000000 | 2090 | 1938.381407 | 1991.37 | 2044.358593 | 0.952809 |  |
| GO:0033673\_negative\_regulation\_of\_kinase\_activity | 36 | 0 | 0.000000 | -0.000000 | 2090 | 1938.381407 | 1991.37 | 2044.358593 | 0.952809 |  |
| GO:0042742\_defense\_response\_to\_bacterium | 36 | 0 | 0.000000 | -0.000000 | 2090 | 1938.381407 | 1991.37 | 2044.358593 | 0.952809 |  |
| GO:0050851\_antigen\_receptor-mediated\_signaling\_pathway | 36 | 0 | 0.000000 | -0.000000 | 2090 | 1938.381407 | 1991.37 | 2044.358593 | 0.952809 |  |
| GO:0050900\_leukocyte\_migration | 36 | 0 | 0.000000 | -0.000000 | 2090 | 1938.381407 | 1991.37 | 2044.358593 | 0.952809 |  |
| GO:0000302\_response\_to\_reactive\_oxygen\_species | 16 | 0 | 0.000000 | -0.000000 | 2128 | 1979.953551 | 2031.18 | 2082.406449 | 0.954502 |  |
| GO:0003044\_regulation\_of\_systemic\_arterial\_blood\_pressure\_mediated\_by\_a\_chemical\_signal | 16 | 0 | 0.000000 | -0.000000 | 2128 | 1979.953551 | 2031.18 | 2082.406449 | 0.954502 |  |
| GO:0006664\_glycolipid\_metabolic\_process | 16 | 0 | 0.000000 | -0.000000 | 2128 | 1979.953551 | 2031.18 | 2082.406449 | 0.954502 |  |
| GO:0006821\_chloride\_transport | 16 | 0 | 0.000000 | -0.000000 | 2128 | 1979.953551 | 2031.18 | 2082.406449 | 0.954502 |  |
| GO:0007033\_vacuole\_organization | 16 | 0 | 0.000000 | -0.000000 | 2128 | 1979.953551 | 2031.18 | 2082.406449 | 0.954502 |  |
| GO:0007156\_homophilic\_cell\_adhesion | 16 | 0 | 0.000000 | -0.000000 | 2128 | 1979.953551 | 2031.18 | 2082.406449 | 0.954502 |  |
| GO:0007602\_phototransduction | 16 | 0 | 0.000000 | -0.000000 | 2128 | 1979.953551 | 2031.18 | 2082.406449 | 0.954502 |  |
| GO:0008654\_phospholipid\_biosynthetic\_process | 16 | 0 | 0.000000 | -0.000000 | 2128 | 1979.953551 | 2031.18 | 2082.406449 | 0.954502 |  |
| GO:0010038\_response\_to\_metal\_ion | 16 | 0 | 0.000000 | -0.000000 | 2128 | 1979.953551 | 2031.18 | 2082.406449 | 0.954502 |  |
| GO:0010243\_response\_to\_organic\_nitrogen | 16 | 0 | 0.000000 | -0.000000 | 2128 | 1979.953551 | 2031.18 | 2082.406449 | 0.954502 |  |
| GO:0010876\_lipid\_localization | 16 | 0 | 0.000000 | -0.000000 | 2128 | 1979.953551 | 2031.18 | 2082.406449 | 0.954502 |  |
| GO:0014075\_response\_to\_amine\_stimulus | 16 | 0 | 0.000000 | -0.000000 | 2128 | 1979.953551 | 2031.18 | 2082.406449 | 0.954502 |  |
| GO:0019722\_calcium-mediated\_signaling | 16 | 0 | 0.000000 | -0.000000 | 2128 | 1979.953551 | 2031.18 | 2082.406449 | 0.954502 |  |
| GO:0019751\_polyol\_metabolic\_process | 16 | 0 | 0.000000 | -0.000000 | 2128 | 1979.953551 | 2031.18 | 2082.406449 | 0.954502 |  |
| GO:0019915\_lipid\_storage | 16 | 0 | 0.000000 | -0.000000 | 2128 | 1979.953551 | 2031.18 | 2082.406449 | 0.954502 |  |
| GO:0021522\_spinal\_cord\_motor\_neuron\_differentiation | 16 | 0 | 0.000000 | -0.000000 | 2128 | 1979.953551 | 2031.18 | 2082.406449 | 0.954502 |  |
| GO:0030890\_positive\_regulation\_of\_B\_cell\_proliferation | 16 | 0 | 0.000000 | -0.000000 | 2128 | 1979.953551 | 2031.18 | 2082.406449 | 0.954502 |  |
| GO:0031345\_negative\_regulation\_of\_cell\_projection\_organization | 16 | 0 | 0.000000 | -0.000000 | 2128 | 1979.953551 | 2031.18 | 2082.406449 | 0.954502 |  |
| GO:0031570\_DNA\_integrity\_checkpoint | 16 | 0 | 0.000000 | -0.000000 | 2128 | 1979.953551 | 2031.18 | 2082.406449 | 0.954502 |  |
| GO:0031669\_cellular\_response\_to\_nutrient\_levels | 16 | 0 | 0.000000 | -0.000000 | 2128 | 1979.953551 | 2031.18 | 2082.406449 | 0.954502 |  |
| GO:0032663\_regulation\_of\_interleukin-2\_production | 16 | 0 | 0.000000 | -0.000000 | 2128 | 1979.953551 | 2031.18 | 2082.406449 | 0.954502 |  |
| GO:0034976\_response\_to\_endoplasmic\_reticulum\_stress | 16 | 0 | 0.000000 | -0.000000 | 2128 | 1979.953551 | 2031.18 | 2082.406449 | 0.954502 |  |
| GO:0042594\_response\_to\_starvation | 16 | 0 | 0.000000 | -0.000000 | 2128 | 1979.953551 | 2031.18 | 2082.406449 | 0.954502 |  |
| GO:0042596\_fear\_response | 16 | 0 | 0.000000 | -0.000000 | 2128 | 1979.953551 | 2031.18 | 2082.406449 | 0.954502 |  |
| GO:0043122\_regulation\_of\_I-kappaB\_kinase\_NF-kappaB\_cascade | 16 | 0 | 0.000000 | -0.000000 | 2128 | 1979.953551 | 2031.18 | 2082.406449 | 0.954502 |  |
| GO:0043367\_CD4-positive\_\_alpha\_beta\_T\_cell\_differentiation | 16 | 0 | 0.000000 | -0.000000 | 2128 | 1979.953551 | 2031.18 | 2082.406449 | 0.954502 |  |
| GO:0045104\_intermediate\_filament\_cytoskeleton\_organization | 16 | 0 | 0.000000 | -0.000000 | 2128 | 1979.953551 | 2031.18 | 2082.406449 | 0.954502 |  |
| GO:0046148\_pigment\_biosynthetic\_process | 16 | 0 | 0.000000 | -0.000000 | 2128 | 1979.953551 | 2031.18 | 2082.406449 | 0.954502 |  |
| GO:0046467\_membrane\_lipid\_biosynthetic\_process | 16 | 0 | 0.000000 | -0.000000 | 2128 | 1979.953551 | 2031.18 | 2082.406449 | 0.954502 |  |
| GO:0046633\_alpha-beta\_T\_cell\_proliferation | 16 | 0 | 0.000000 | -0.000000 | 2128 | 1979.953551 | 2031.18 | 2082.406449 | 0.954502 |  |
| GO:0046700\_heterocycle\_catabolic\_process | 16 | 0 | 0.000000 | -0.000000 | 2128 | 1979.953551 | 2031.18 | 2082.406449 | 0.954502 |  |
| GO:0048015\_phosphoinositide-mediated\_signaling | 16 | 0 | 0.000000 | -0.000000 | 2128 | 1979.953551 | 2031.18 | 2082.406449 | 0.954502 |  |
| GO:0048286\_lung\_alveolus\_development | 16 | 0 | 0.000000 | -0.000000 | 2128 | 1979.953551 | 2031.18 | 2082.406449 | 0.954502 |  |
| GO:0048483\_autonomic\_nervous\_system\_development | 16 | 0 | 0.000000 | -0.000000 | 2128 | 1979.953551 | 2031.18 | 2082.406449 | 0.954502 |  |
| GO:0050974\_detection\_of\_mechanical\_stimulus\_involved\_in\_sensory\_perception | 16 | 0 | 0.000000 | -0.000000 | 2128 | 1979.953551 | 2031.18 | 2082.406449 | 0.954502 |  |
| GO:0051048\_negative\_regulation\_of\_secretion | 16 | 0 | 0.000000 | -0.000000 | 2128 | 1979.953551 | 2031.18 | 2082.406449 | 0.954502 |  |
| GO:0051937\_catecholamine\_transport | 16 | 0 | 0.000000 | -0.000000 | 2128 | 1979.953551 | 2031.18 | 2082.406449 | 0.954502 |  |
| GO:0060193\_positive\_regulation\_of\_lipase\_activity | 16 | 0 | 0.000000 | -0.000000 | 2128 | 1979.953551 | 2031.18 | 2082.406449 | 0.954502 |  |
| GO:0006338\_chromatin\_remodeling | 19 | 0 | 0.000000 | -0.000000 | 2146 | 2008.472447 | 2058.26 | 2108.047553 | 0.959115 |  |
| GO:0006497\_protein\_amino\_acid\_lipidation | 19 | 0 | 0.000000 | -0.000000 | 2146 | 2008.472447 | 2058.26 | 2108.047553 | 0.959115 |  |
| GO:0006672\_ceramide\_metabolic\_process | 19 | 0 | 0.000000 | -0.000000 | 2146 | 2008.472447 | 2058.26 | 2108.047553 | 0.959115 |  |
| GO:0007569\_cell\_aging | 19 | 0 | 0.000000 | -0.000000 | 2146 | 2008.472447 | 2058.26 | 2108.047553 | 0.959115 |  |
| GO:0007595\_lactation | 19 | 0 | 0.000000 | -0.000000 | 2146 | 2008.472447 | 2058.26 | 2108.047553 | 0.959115 |  |
| GO:0010639\_negative\_regulation\_of\_organelle\_organization | 19 | 0 | 0.000000 | -0.000000 | 2146 | 2008.472447 | 2058.26 | 2108.047553 | 0.959115 |  |
| GO:0010952\_positive\_regulation\_of\_peptidase\_activity | 19 | 0 | 0.000000 | -0.000000 | 2146 | 2008.472447 | 2058.26 | 2108.047553 | 0.959115 |  |
| GO:0030518\_steroid\_hormone\_receptor\_signaling\_pathway | 19 | 0 | 0.000000 | -0.000000 | 2146 | 2008.472447 | 2058.26 | 2108.047553 | 0.959115 |  |
| GO:0030595\_leukocyte\_chemotaxis | 19 | 0 | 0.000000 | -0.000000 | 2146 | 2008.472447 | 2058.26 | 2108.047553 | 0.959115 |  |
| GO:0042462\_eye\_photoreceptor\_cell\_development | 19 | 0 | 0.000000 | -0.000000 | 2146 | 2008.472447 | 2058.26 | 2108.047553 | 0.959115 |  |
| GO:0043280\_positive\_regulation\_of\_caspase\_activity | 19 | 0 | 0.000000 | -0.000000 | 2146 | 2008.472447 | 2058.26 | 2108.047553 | 0.959115 |  |
| GO:0046890\_regulation\_of\_lipid\_biosynthetic\_process | 19 | 0 | 0.000000 | -0.000000 | 2146 | 2008.472447 | 2058.26 | 2108.047553 | 0.959115 |  |
| GO:0048536\_spleen\_development | 19 | 0 | 0.000000 | -0.000000 | 2146 | 2008.472447 | 2058.26 | 2108.047553 | 0.959115 |  |
| GO:0050728\_negative\_regulation\_of\_inflammatory\_response | 19 | 0 | 0.000000 | -0.000000 | 2146 | 2008.472447 | 2058.26 | 2108.047553 | 0.959115 |  |
| GO:0050931\_pigment\_cell\_differentiation | 19 | 0 | 0.000000 | -0.000000 | 2146 | 2008.472447 | 2058.26 | 2108.047553 | 0.959115 |  |
| GO:0051056\_regulation\_of\_small\_GTPase\_mediated\_signal\_transduction | 19 | 0 | 0.000000 | -0.000000 | 2146 | 2008.472447 | 2058.26 | 2108.047553 | 0.959115 |  |
| GO:0060079\_regulation\_of\_excitatory\_postsynaptic\_membrane\_potential | 19 | 0 | 0.000000 | -0.000000 | 2146 | 2008.472447 | 2058.26 | 2108.047553 | 0.959115 |  |
| GO:0060326\_cell\_chemotaxis | 19 | 0 | 0.000000 | -0.000000 | 2146 | 2008.472447 | 2058.26 | 2108.047553 | 0.959115 |  |
| GO:0002696\_positive\_regulation\_of\_leukocyte\_activation | 82 | 0 | 0.000000 | -0.000000 | 2147 | 2010.809221 | 2060.08 | 2109.350779 | 0.959516 |  |
| GO:0007611\_learning\_or\_memory | 70 | 0 | 0.000000 | -0.000000 | 2150 | 2013.322642 | 2062.25 | 2111.177358 | 0.959186 |  |
| GO:0009617\_response\_to\_bacterium | 70 | 0 | 0.000000 | -0.000000 | 2150 | 2013.322642 | 2062.25 | 2111.177358 | 0.959186 |  |
| GO:0070838\_divalent\_metal\_ion\_transport | 70 | 0 | 0.000000 | -0.000000 | 2150 | 2013.322642 | 2062.25 | 2111.177358 | 0.959186 |  |
| GO:0006325\_chromatin\_organization | 83 | 0 | 0.000000 | -0.000000 | 2153 | 2016.799029 | 2065.21 | 2113.620971 | 0.959224 |  |
| GO:0007017\_microtubule-based\_process | 83 | 0 | 0.000000 | -0.000000 | 2153 | 2016.799029 | 2065.21 | 2113.620971 | 0.959224 |  |
| GO:0030534\_adult\_behavior | 83 | 0 | 0.000000 | -0.000000 | 2153 | 2016.799029 | 2065.21 | 2113.620971 | 0.959224 |  |
| GO:0000187\_activation\_of\_MAPK\_activity | 30 | 0 | 0.000000 | -0.000000 | 2165 | 2034.970984 | 2082.13 | 2129.289016 | 0.961723 |  |
| GO:0007435\_salivary\_gland\_morphogenesis | 30 | 0 | 0.000000 | -0.000000 | 2165 | 2034.970984 | 2082.13 | 2129.289016 | 0.961723 |  |
| GO:0030522\_intracellular\_receptor-mediated\_signaling\_pathway | 30 | 0 | 0.000000 | -0.000000 | 2165 | 2034.970984 | 2082.13 | 2129.289016 | 0.961723 |  |
| GO:0031281\_positive\_regulation\_of\_cyclase\_activity | 30 | 0 | 0.000000 | -0.000000 | 2165 | 2034.970984 | 2082.13 | 2129.289016 | 0.961723 |  |
| GO:0031668\_cellular\_response\_to\_extracellular\_stimulus | 30 | 0 | 0.000000 | -0.000000 | 2165 | 2034.970984 | 2082.13 | 2129.289016 | 0.961723 |  |
| GO:0032102\_negative\_regulation\_of\_response\_to\_external\_stimulus | 30 | 0 | 0.000000 | -0.000000 | 2165 | 2034.970984 | 2082.13 | 2129.289016 | 0.961723 |  |
| GO:0033500\_carbohydrate\_homeostasis | 30 | 0 | 0.000000 | -0.000000 | 2165 | 2034.970984 | 2082.13 | 2129.289016 | 0.961723 |  |
| GO:0042552\_myelination | 30 | 0 | 0.000000 | -0.000000 | 2165 | 2034.970984 | 2082.13 | 2129.289016 | 0.961723 |  |
| GO:0042593\_glucose\_homeostasis | 30 | 0 | 0.000000 | -0.000000 | 2165 | 2034.970984 | 2082.13 | 2129.289016 | 0.961723 |  |
| GO:0045762\_positive\_regulation\_of\_adenylate\_cyclase\_activity | 30 | 0 | 0.000000 | -0.000000 | 2165 | 2034.970984 | 2082.13 | 2129.289016 | 0.961723 |  |
| GO:0051349\_positive\_regulation\_of\_lyase\_activity | 30 | 0 | 0.000000 | -0.000000 | 2165 | 2034.970984 | 2082.13 | 2129.289016 | 0.961723 |  |
| GO:0052547\_regulation\_of\_peptidase\_activity | 30 | 0 | 0.000000 | -0.000000 | 2165 | 2034.970984 | 2082.13 | 2129.289016 | 0.961723 |  |
| GO:0001508\_regulation\_of\_action\_potential | 43 | 0 | 0.000000 | -0.000000 | 2173 | 2047.598470 | 2093.51 | 2139.421530 | 0.963419 |  |
| GO:0002819\_regulation\_of\_adaptive\_immune\_response | 43 | 0 | 0.000000 | -0.000000 | 2173 | 2047.598470 | 2093.51 | 2139.421530 | 0.963419 |  |
| GO:0002822\_regulation\_of\_adaptive\_immune\_response\_based\_on\_somatic\_recombination\_of\_immune\_receptors\_built\_from\_immunoglobulin\_superfamily\_domains | 43 | 0 | 0.000000 | -0.000000 | 2173 | 2047.598470 | 2093.51 | 2139.421530 | 0.963419 |  |
| GO:0019637\_organophosphate\_metabolic\_process | 43 | 0 | 0.000000 | -0.000000 | 2173 | 2047.598470 | 2093.51 | 2139.421530 | 0.963419 |  |
| GO:0030814\_regulation\_of\_cAMP\_metabolic\_process | 43 | 0 | 0.000000 | -0.000000 | 2173 | 2047.598470 | 2093.51 | 2139.421530 | 0.963419 |  |
| GO:0032446\_protein\_modification\_by\_small\_protein\_conjugation | 43 | 0 | 0.000000 | -0.000000 | 2173 | 2047.598470 | 2093.51 | 2139.421530 | 0.963419 |  |
| GO:0046879\_hormone\_secretion | 43 | 0 | 0.000000 | -0.000000 | 2173 | 2047.598470 | 2093.51 | 2139.421530 | 0.963419 |  |
| GO:0051604\_protein\_maturation | 43 | 0 | 0.000000 | -0.000000 | 2173 | 2047.598470 | 2093.51 | 2139.421530 | 0.963419 |  |
| GO:0051969\_regulation\_of\_transmission\_of\_nerve\_impulse | 61 | 0 | 0.000000 | -0.000000 | 2174 | 2050.582601 | 2095.9 | 2141.217399 | 0.964075 |  |
| GO:0000096\_sulfur\_amino\_acid\_metabolic\_process | 11 | 0 | 0.000000 | -0.000000 | 2247 | 2126.468971 | 2170.1 | 2213.731029 | 0.965777 |  |
| GO:0000271\_polysaccharide\_biosynthetic\_process | 11 | 0 | 0.000000 | -0.000000 | 2247 | 2126.468971 | 2170.1 | 2213.731029 | 0.965777 |  |
| GO:0000737\_DNA\_catabolic\_process\_\_endonucleolytic | 11 | 0 | 0.000000 | -0.000000 | 2247 | 2126.468971 | 2170.1 | 2213.731029 | 0.965777 |  |
| GO:0001101\_response\_to\_acid | 11 | 0 | 0.000000 | -0.000000 | 2247 | 2126.468971 | 2170.1 | 2213.731029 | 0.965777 |  |
| GO:0001837\_epithelial\_to\_mesenchymal\_transition | 11 | 0 | 0.000000 | -0.000000 | 2247 | 2126.468971 | 2170.1 | 2213.731029 | 0.965777 |  |
| GO:0001913\_T\_cell\_mediated\_cytotoxicity | 11 | 0 | 0.000000 | -0.000000 | 2247 | 2126.468971 | 2170.1 | 2213.731029 | 0.965777 |  |
| GO:0001952\_regulation\_of\_cell-matrix\_adhesion | 11 | 0 | 0.000000 | -0.000000 | 2247 | 2126.468971 | 2170.1 | 2213.731029 | 0.965777 |  |
| GO:0001963\_synaptic\_transmission\_\_dopaminergic | 11 | 0 | 0.000000 | -0.000000 | 2247 | 2126.468971 | 2170.1 | 2213.731029 | 0.965777 |  |
| GO:0002444\_myeloid\_leukocyte\_mediated\_immunity | 11 | 0 | 0.000000 | -0.000000 | 2247 | 2126.468971 | 2170.1 | 2213.731029 | 0.965777 |  |
| GO:0002467\_germinal\_center\_formation | 11 | 0 | 0.000000 | -0.000000 | 2247 | 2126.468971 | 2170.1 | 2213.731029 | 0.965777 |  |
| GO:0002758\_innate\_immune\_response-activating\_signal\_transduction | 11 | 0 | 0.000000 | -0.000000 | 2247 | 2126.468971 | 2170.1 | 2213.731029 | 0.965777 |  |
| GO:0006333\_chromatin\_assembly\_or\_disassembly | 11 | 0 | 0.000000 | -0.000000 | 2247 | 2126.468971 | 2170.1 | 2213.731029 | 0.965777 |  |
| GO:0006635\_fatty\_acid\_beta-oxidation | 11 | 0 | 0.000000 | -0.000000 | 2247 | 2126.468971 | 2170.1 | 2213.731029 | 0.965777 |  |
| GO:0006637\_acyl-CoA\_metabolic\_process | 11 | 0 | 0.000000 | -0.000000 | 2247 | 2126.468971 | 2170.1 | 2213.731029 | 0.965777 |  |
| GO:0006690\_icosanoid\_metabolic\_process | 11 | 0 | 0.000000 | -0.000000 | 2247 | 2126.468971 | 2170.1 | 2213.731029 | 0.965777 |  |
| GO:0006779\_porphyrin\_biosynthetic\_process | 11 | 0 | 0.000000 | -0.000000 | 2247 | 2126.468971 | 2170.1 | 2213.731029 | 0.965777 |  |
| GO:0007051\_spindle\_organization | 11 | 0 | 0.000000 | -0.000000 | 2247 | 2126.468971 | 2170.1 | 2213.731029 | 0.965777 |  |
| GO:0007088\_regulation\_of\_mitosis | 11 | 0 | 0.000000 | -0.000000 | 2247 | 2126.468971 | 2170.1 | 2213.731029 | 0.965777 |  |
| GO:0007159\_leukocyte\_adhesion | 11 | 0 | 0.000000 | -0.000000 | 2247 | 2126.468971 | 2170.1 | 2213.731029 | 0.965777 |  |
| GO:0007162\_negative\_regulation\_of\_cell\_adhesion | 11 | 0 | 0.000000 | -0.000000 | 2247 | 2126.468971 | 2170.1 | 2213.731029 | 0.965777 |  |
| GO:0007215\_glutamate\_signaling\_pathway | 11 | 0 | 0.000000 | -0.000000 | 2247 | 2126.468971 | 2170.1 | 2213.731029 | 0.965777 |  |
| GO:0007260\_tyrosine\_phosphorylation\_of\_STAT\_protein | 11 | 0 | 0.000000 | -0.000000 | 2247 | 2126.468971 | 2170.1 | 2213.731029 | 0.965777 |  |
| GO:0008652\_cellular\_amino\_acid\_biosynthetic\_process | 11 | 0 | 0.000000 | -0.000000 | 2247 | 2126.468971 | 2170.1 | 2213.731029 | 0.965777 |  |
| GO:0009064\_glutamine\_family\_amino\_acid\_metabolic\_process | 11 | 0 | 0.000000 | -0.000000 | 2247 | 2126.468971 | 2170.1 | 2213.731029 | 0.965777 |  |
| GO:0009141\_nucleoside\_triphosphate\_metabolic\_process | 11 | 0 | 0.000000 | -0.000000 | 2247 | 2126.468971 | 2170.1 | 2213.731029 | 0.965777 |  |
| GO:0009166\_nucleotide\_catabolic\_process | 11 | 0 | 0.000000 | -0.000000 | 2247 | 2126.468971 | 2170.1 | 2213.731029 | 0.965777 |  |
| GO:0009409\_response\_to\_cold | 11 | 0 | 0.000000 | -0.000000 | 2247 | 2126.468971 | 2170.1 | 2213.731029 | 0.965777 |  |
| GO:0010259\_multicellular\_organismal\_aging | 11 | 0 | 0.000000 | -0.000000 | 2247 | 2126.468971 | 2170.1 | 2213.731029 | 0.965777 |  |
| GO:0014902\_myotube\_differentiation | 11 | 0 | 0.000000 | -0.000000 | 2247 | 2126.468971 | 2170.1 | 2213.731029 | 0.965777 |  |
| GO:0016079\_synaptic\_vesicle\_exocytosis | 11 | 0 | 0.000000 | -0.000000 | 2247 | 2126.468971 | 2170.1 | 2213.731029 | 0.965777 |  |
| GO:0021602\_cranial\_nerve\_morphogenesis | 11 | 0 | 0.000000 | -0.000000 | 2247 | 2126.468971 | 2170.1 | 2213.731029 | 0.965777 |  |
| GO:0030238\_male\_sex\_determination | 11 | 0 | 0.000000 | -0.000000 | 2247 | 2126.468971 | 2170.1 | 2213.731029 | 0.965777 |  |
| GO:0030308\_negative\_regulation\_of\_cell\_growth | 11 | 0 | 0.000000 | -0.000000 | 2247 | 2126.468971 | 2170.1 | 2213.731029 | 0.965777 |  |
| GO:0030593\_neutrophil\_chemotaxis | 11 | 0 | 0.000000 | -0.000000 | 2247 | 2126.468971 | 2170.1 | 2213.731029 | 0.965777 |  |
| GO:0030878\_thyroid\_gland\_development | 11 | 0 | 0.000000 | -0.000000 | 2247 | 2126.468971 | 2170.1 | 2213.731029 | 0.965777 |  |
| GO:0030968\_endoplasmic\_reticulum\_unfolded\_protein\_response | 11 | 0 | 0.000000 | -0.000000 | 2247 | 2126.468971 | 2170.1 | 2213.731029 | 0.965777 |  |
| GO:0031110\_regulation\_of\_microtubule\_polymerization\_or\_depolymerization | 11 | 0 | 0.000000 | -0.000000 | 2247 | 2126.468971 | 2170.1 | 2213.731029 | 0.965777 |  |
| GO:0031646\_positive\_regulation\_of\_neurological\_system\_process | 11 | 0 | 0.000000 | -0.000000 | 2247 | 2126.468971 | 2170.1 | 2213.731029 | 0.965777 |  |
| GO:0031647\_regulation\_of\_protein\_stability | 11 | 0 | 0.000000 | -0.000000 | 2247 | 2126.468971 | 2170.1 | 2213.731029 | 0.965777 |  |
| GO:0032655\_regulation\_of\_interleukin-12\_production | 11 | 0 | 0.000000 | -0.000000 | 2247 | 2126.468971 | 2170.1 | 2213.731029 | 0.965777 |  |
| GO:0033014\_tetrapyrrole\_biosynthetic\_process | 11 | 0 | 0.000000 | -0.000000 | 2247 | 2126.468971 | 2170.1 | 2213.731029 | 0.965777 |  |
| GO:0033059\_cellular\_pigmentation | 11 | 0 | 0.000000 | -0.000000 | 2247 | 2126.468971 | 2170.1 | 2213.731029 | 0.965777 |  |
| GO:0033559\_unsaturated\_fatty\_acid\_metabolic\_process | 11 | 0 | 0.000000 | -0.000000 | 2247 | 2126.468971 | 2170.1 | 2213.731029 | 0.965777 |  |
| GO:0034620\_cellular\_response\_to\_unfolded\_protein | 11 | 0 | 0.000000 | -0.000000 | 2247 | 2126.468971 | 2170.1 | 2213.731029 | 0.965777 |  |
| GO:0034762\_regulation\_of\_transmembrane\_transport | 11 | 0 | 0.000000 | -0.000000 | 2247 | 2126.468971 | 2170.1 | 2213.731029 | 0.965777 |  |
| GO:0035176\_social\_behavior | 11 | 0 | 0.000000 | -0.000000 | 2247 | 2126.468971 | 2170.1 | 2213.731029 | 0.965777 |  |
| GO:0042036\_negative\_regulation\_of\_cytokine\_biosynthetic\_process | 11 | 0 | 0.000000 | -0.000000 | 2247 | 2126.468971 | 2170.1 | 2213.731029 | 0.965777 |  |
| GO:0042219\_cellular\_amino\_acid\_derivative\_catabolic\_process | 11 | 0 | 0.000000 | -0.000000 | 2247 | 2126.468971 | 2170.1 | 2213.731029 | 0.965777 |  |
| GO:0042439\_ethanolamine\_and\_derivative\_metabolic\_process | 11 | 0 | 0.000000 | -0.000000 | 2247 | 2126.468971 | 2170.1 | 2213.731029 | 0.965777 |  |
| GO:0042542\_response\_to\_hydrogen\_peroxide | 11 | 0 | 0.000000 | -0.000000 | 2247 | 2126.468971 | 2170.1 | 2213.731029 | 0.965777 |  |
| GO:0042551\_neuron\_maturation | 11 | 0 | 0.000000 | -0.000000 | 2247 | 2126.468971 | 2170.1 | 2213.731029 | 0.965777 |  |
| GO:0043270\_positive\_regulation\_of\_ion\_transport | 11 | 0 | 0.000000 | -0.000000 | 2247 | 2126.468971 | 2170.1 | 2213.731029 | 0.965777 |  |
| GO:0043576\_regulation\_of\_respiratory\_gaseous\_exchange | 11 | 0 | 0.000000 | -0.000000 | 2247 | 2126.468971 | 2170.1 | 2213.731029 | 0.965777 |  |
| GO:0045026\_plasma\_membrane\_fusion | 11 | 0 | 0.000000 | -0.000000 | 2247 | 2126.468971 | 2170.1 | 2213.731029 | 0.965777 |  |
| GO:0045055\_regulated\_secretory\_pathway | 11 | 0 | 0.000000 | -0.000000 | 2247 | 2126.468971 | 2170.1 | 2213.731029 | 0.965777 |  |
| GO:0045216\_cell-cell\_junction\_organization | 11 | 0 | 0.000000 | -0.000000 | 2247 | 2126.468971 | 2170.1 | 2213.731029 | 0.965777 |  |
| GO:0046323\_glucose\_import | 11 | 0 | 0.000000 | -0.000000 | 2247 | 2126.468971 | 2170.1 | 2213.731029 | 0.965777 |  |
| GO:0046425\_regulation\_of\_JAK-STAT\_cascade | 11 | 0 | 0.000000 | -0.000000 | 2247 | 2126.468971 | 2170.1 | 2213.731029 | 0.965777 |  |
| GO:0046716\_muscle\_maintenance | 11 | 0 | 0.000000 | -0.000000 | 2247 | 2126.468971 | 2170.1 | 2213.731029 | 0.965777 |  |
| GO:0046928\_regulation\_of\_neurotransmitter\_secretion | 11 | 0 | 0.000000 | -0.000000 | 2247 | 2126.468971 | 2170.1 | 2213.731029 | 0.965777 |  |
| GO:0048009\_insulin-like\_growth\_factor\_receptor\_signaling\_pathway | 11 | 0 | 0.000000 | -0.000000 | 2247 | 2126.468971 | 2170.1 | 2213.731029 | 0.965777 |  |
| GO:0048193\_Golgi\_vesicle\_transport | 11 | 0 | 0.000000 | -0.000000 | 2247 | 2126.468971 | 2170.1 | 2213.731029 | 0.965777 |  |
| GO:0050772\_positive\_regulation\_of\_axonogenesis | 11 | 0 | 0.000000 | -0.000000 | 2247 | 2126.468971 | 2170.1 | 2213.731029 | 0.965777 |  |
| GO:0050806\_positive\_regulation\_of\_synaptic\_transmission | 11 | 0 | 0.000000 | -0.000000 | 2247 | 2126.468971 | 2170.1 | 2213.731029 | 0.965777 |  |
| GO:0050807\_regulation\_of\_synapse\_organization | 11 | 0 | 0.000000 | -0.000000 | 2247 | 2126.468971 | 2170.1 | 2213.731029 | 0.965777 |  |
| GO:0050830\_defense\_response\_to\_Gram-positive\_bacterium | 11 | 0 | 0.000000 | -0.000000 | 2247 | 2126.468971 | 2170.1 | 2213.731029 | 0.965777 |  |
| GO:0050854\_regulation\_of\_antigen\_receptor-mediated\_signaling\_pathway | 11 | 0 | 0.000000 | -0.000000 | 2247 | 2126.468971 | 2170.1 | 2213.731029 | 0.965777 |  |
| GO:0051092\_positive\_regulation\_of\_NF-kappaB\_transcription\_factor\_activity | 11 | 0 | 0.000000 | -0.000000 | 2247 | 2126.468971 | 2170.1 | 2213.731029 | 0.965777 |  |
| GO:0051494\_negative\_regulation\_of\_cytoskeleton\_organization | 11 | 0 | 0.000000 | -0.000000 | 2247 | 2126.468971 | 2170.1 | 2213.731029 | 0.965777 |  |
| GO:0051648\_vesicle\_localization | 11 | 0 | 0.000000 | -0.000000 | 2247 | 2126.468971 | 2170.1 | 2213.731029 | 0.965777 |  |
| GO:0051783\_regulation\_of\_nuclear\_division | 11 | 0 | 0.000000 | -0.000000 | 2247 | 2126.468971 | 2170.1 | 2213.731029 | 0.965777 |  |
| GO:0051971\_positive\_regulation\_of\_transmission\_of\_nerve\_impulse | 11 | 0 | 0.000000 | -0.000000 | 2247 | 2126.468971 | 2170.1 | 2213.731029 | 0.965777 |  |
| GO:0060004\_reflex | 11 | 0 | 0.000000 | -0.000000 | 2247 | 2126.468971 | 2170.1 | 2213.731029 | 0.965777 |  |
| GO:0006461\_protein\_complex\_assembly | 78 | 0 | 0.000000 | -0.000000 | 2252 | 2129.949471 | 2172.86 | 2215.770529 | 0.964858 |  |
| GO:0007519\_skeletal\_muscle\_tissue\_development | 78 | 0 | 0.000000 | -0.000000 | 2252 | 2129.949471 | 2172.86 | 2215.770529 | 0.964858 |  |
| GO:0051251\_positive\_regulation\_of\_lymphocyte\_activation | 78 | 0 | 0.000000 | -0.000000 | 2252 | 2129.949471 | 2172.86 | 2215.770529 | 0.964858 |  |
| GO:0060538\_skeletal\_muscle\_organ\_development | 78 | 0 | 0.000000 | -0.000000 | 2252 | 2129.949471 | 2172.86 | 2215.770529 | 0.964858 |  |
| GO:0070271\_protein\_complex\_biogenesis | 78 | 0 | 0.000000 | -0.000000 | 2252 | 2129.949471 | 2172.86 | 2215.770529 | 0.964858 |  |
| GO:0031644\_regulation\_of\_neurological\_system\_process | 64 | 0 | 0.000000 | -0.000000 | 2254 | 2132.775027 | 2175.08 | 2217.384973 | 0.964987 |  |
| GO:0070663\_regulation\_of\_leukocyte\_proliferation | 64 | 0 | 0.000000 | -0.000000 | 2254 | 2132.775027 | 2175.08 | 2217.384973 | 0.964987 |  |
| GO:0002706\_regulation\_of\_lymphocyte\_mediated\_immunity | 52 | 0 | 0.000000 | -0.000000 | 2258 | 2135.991552 | 2177.93 | 2219.868448 | 0.964539 |  |
| GO:0009124\_nucleoside\_monophosphate\_biosynthetic\_process | 52 | 0 | 0.000000 | -0.000000 | 2258 | 2135.991552 | 2177.93 | 2219.868448 | 0.964539 |  |
| GO:0010608\_posttranscriptional\_regulation\_of\_gene\_expression | 52 | 0 | 0.000000 | -0.000000 | 2258 | 2135.991552 | 2177.93 | 2219.868448 | 0.964539 |  |
| GO:0048585\_negative\_regulation\_of\_response\_to\_stimulus | 52 | 0 | 0.000000 | -0.000000 | 2258 | 2135.991552 | 2177.93 | 2219.868448 | 0.964539 |  |
| GO:0006508\_proteolysis | 76 | 0 | 0.000000 | -0.000000 | 2259 | 2138.971677 | 2180.3 | 2221.628323 | 0.965162 |  |
| GO:0006732\_coenzyme\_metabolic\_process | 46 | 0 | 0.000000 | -0.000000 | 2263 | 2145.505209 | 2186.12 | 2226.734791 | 0.966027 |  |
| GO:0007612\_learning | 46 | 0 | 0.000000 | -0.000000 | 2263 | 2145.505209 | 2186.12 | 2226.734791 | 0.966027 |  |
| GO:0046631\_alpha-beta\_T\_cell\_activation | 46 | 0 | 0.000000 | -0.000000 | 2263 | 2145.505209 | 2186.12 | 2226.734791 | 0.966027 |  |
| GO:0051098\_regulation\_of\_binding | 46 | 0 | 0.000000 | -0.000000 | 2263 | 2145.505209 | 2186.12 | 2226.734791 | 0.966027 |  |
| GO:0032870\_cellular\_response\_to\_hormone\_stimulus | 45 | 0 | 0.000000 | -0.000000 | 2266 | 2149.473872 | 2189.76 | 2230.046128 | 0.966355 |  |
| GO:0043623\_cellular\_protein\_complex\_assembly | 45 | 0 | 0.000000 | -0.000000 | 2266 | 2149.473872 | 2189.76 | 2230.046128 | 0.966355 |  |
| GO:0046058\_cAMP\_metabolic\_process | 45 | 0 | 0.000000 | -0.000000 | 2266 | 2149.473872 | 2189.76 | 2230.046128 | 0.966355 |  |
| GO:0000045\_autophagic\_vacuole\_formation | 5 | 0 | 0.000000 | -0.000000 | 2521 | 2425.543492 | 2461.58 | 2497.616508 | 0.976430 |  |
| GO:0000070\_mitotic\_sister\_chromatid\_segregation | 5 | 0 | 0.000000 | -0.000000 | 2521 | 2425.543492 | 2461.58 | 2497.616508 | 0.976430 |  |
| GO:0000097\_sulfur\_amino\_acid\_biosynthetic\_process | 5 | 0 | 0.000000 | -0.000000 | 2521 | 2425.543492 | 2461.58 | 2497.616508 | 0.976430 |  |
| GO:0000239\_pachytene | 5 | 0 | 0.000000 | -0.000000 | 2521 | 2425.543492 | 2461.58 | 2497.616508 | 0.976430 |  |
| GO:0000272\_polysaccharide\_catabolic\_process | 5 | 0 | 0.000000 | -0.000000 | 2521 | 2425.543492 | 2461.58 | 2497.616508 | 0.976430 |  |
| GO:0000288\_nuclear-transcribed\_mRNA\_catabolic\_process\_\_deadenylation-dependent\_decay | 5 | 0 | 0.000000 | -0.000000 | 2521 | 2425.543492 | 2461.58 | 2497.616508 | 0.976430 |  |
| GO:0000578\_embryonic\_axis\_specification | 5 | 0 | 0.000000 | -0.000000 | 2521 | 2425.543492 | 2461.58 | 2497.616508 | 0.976430 |  |
| GO:0000819\_sister\_chromatid\_segregation | 5 | 0 | 0.000000 | -0.000000 | 2521 | 2425.543492 | 2461.58 | 2497.616508 | 0.976430 |  |
| GO:0000956\_nuclear-transcribed\_mRNA\_catabolic\_process | 5 | 0 | 0.000000 | -0.000000 | 2521 | 2425.543492 | 2461.58 | 2497.616508 | 0.976430 |  |
| GO:0001562\_response\_to\_protozoan | 5 | 0 | 0.000000 | -0.000000 | 2521 | 2425.543492 | 2461.58 | 2497.616508 | 0.976430 |  |
| GO:0001865\_NK\_T\_cell\_differentiation | 5 | 0 | 0.000000 | -0.000000 | 2521 | 2425.543492 | 2461.58 | 2497.616508 | 0.976430 |  |
| GO:0001957\_intramembranous\_ossification | 5 | 0 | 0.000000 | -0.000000 | 2521 | 2425.543492 | 2461.58 | 2497.616508 | 0.976430 |  |
| GO:0001959\_regulation\_of\_cytokine-mediated\_signaling\_pathway | 5 | 0 | 0.000000 | -0.000000 | 2521 | 2425.543492 | 2461.58 | 2497.616508 | 0.976430 |  |
| GO:0001991\_regulation\_of\_systemic\_arterial\_blood\_pressure\_by\_circulatory\_renin-angiotensin | 5 | 0 | 0.000000 | -0.000000 | 2521 | 2425.543492 | 2461.58 | 2497.616508 | 0.976430 |  |
| GO:0001993\_regulation\_of\_systemic\_arterial\_blood\_pressure\_by\_norepinephrine-epinephrine | 5 | 0 | 0.000000 | -0.000000 | 2521 | 2425.543492 | 2461.58 | 2497.616508 | 0.976430 |  |
| GO:0002448\_mast\_cell\_mediated\_immunity | 5 | 0 | 0.000000 | -0.000000 | 2521 | 2425.543492 | 2461.58 | 2497.616508 | 0.976430 |  |
| GO:0002517\_T\_cell\_tolerance\_induction | 5 | 0 | 0.000000 | -0.000000 | 2521 | 2425.543492 | 2461.58 | 2497.616508 | 0.976430 |  |
| GO:0002634\_regulation\_of\_germinal\_center\_formation | 5 | 0 | 0.000000 | -0.000000 | 2521 | 2425.543492 | 2461.58 | 2497.616508 | 0.976430 |  |
| GO:0002664\_regulation\_of\_T\_cell\_tolerance\_induction | 5 | 0 | 0.000000 | -0.000000 | 2521 | 2425.543492 | 2461.58 | 2497.616508 | 0.976430 |  |
| GO:0002666\_positive\_regulation\_of\_T\_cell\_tolerance\_induction | 5 | 0 | 0.000000 | -0.000000 | 2521 | 2425.543492 | 2461.58 | 2497.616508 | 0.976430 |  |
| GO:0002710\_negative\_regulation\_of\_T\_cell\_mediated\_immunity | 5 | 0 | 0.000000 | -0.000000 | 2521 | 2425.543492 | 2461.58 | 2497.616508 | 0.976430 |  |
| GO:0002716\_negative\_regulation\_of\_natural\_killer\_cell\_mediated\_immunity | 5 | 0 | 0.000000 | -0.000000 | 2521 | 2425.543492 | 2461.58 | 2497.616508 | 0.976430 |  |
| GO:0002825\_regulation\_of\_T-helper\_1\_type\_immune\_response | 5 | 0 | 0.000000 | -0.000000 | 2521 | 2425.543492 | 2461.58 | 2497.616508 | 0.976430 |  |
| GO:0002862\_negative\_regulation\_of\_inflammatory\_response\_to\_antigenic\_stimulus | 5 | 0 | 0.000000 | -0.000000 | 2521 | 2425.543492 | 2461.58 | 2497.616508 | 0.976430 |  |
| GO:0002866\_positive\_regulation\_of\_acute\_inflammatory\_response\_to\_antigenic\_stimulus | 5 | 0 | 0.000000 | -0.000000 | 2521 | 2425.543492 | 2461.58 | 2497.616508 | 0.976430 |  |
| GO:0002885\_positive\_regulation\_of\_hypersensitivity | 5 | 0 | 0.000000 | -0.000000 | 2521 | 2425.543492 | 2461.58 | 2497.616508 | 0.976430 |  |
| GO:0002902\_regulation\_of\_B\_cell\_apoptosis | 5 | 0 | 0.000000 | -0.000000 | 2521 | 2425.543492 | 2461.58 | 2497.616508 | 0.976430 |  |
| GO:0003071\_renal\_system\_process\_involved\_in\_regulation\_of\_systemic\_arterial\_blood\_pressure | 5 | 0 | 0.000000 | -0.000000 | 2521 | 2425.543492 | 2461.58 | 2497.616508 | 0.976430 |  |
| GO:0005980\_glycogen\_catabolic\_process | 5 | 0 | 0.000000 | -0.000000 | 2521 | 2425.543492 | 2461.58 | 2497.616508 | 0.976430 |  |
| GO:0006023\_aminoglycan\_biosynthetic\_process | 5 | 0 | 0.000000 | -0.000000 | 2521 | 2425.543492 | 2461.58 | 2497.616508 | 0.976430 |  |
| GO:0006024\_glycosaminoglycan\_biosynthetic\_process | 5 | 0 | 0.000000 | -0.000000 | 2521 | 2425.543492 | 2461.58 | 2497.616508 | 0.976430 |  |
| GO:0006268\_DNA\_unwinding\_during\_replication | 5 | 0 | 0.000000 | -0.000000 | 2521 | 2425.543492 | 2461.58 | 2497.616508 | 0.976430 |  |
| GO:0006270\_DNA\_replication\_initiation | 5 | 0 | 0.000000 | -0.000000 | 2521 | 2425.543492 | 2461.58 | 2497.616508 | 0.976430 |  |
| GO:0006346\_methylation-dependent\_chromatin\_silencing | 5 | 0 | 0.000000 | -0.000000 | 2521 | 2425.543492 | 2461.58 | 2497.616508 | 0.976430 |  |
| GO:0006376\_mRNA\_splice\_site\_selection | 5 | 0 | 0.000000 | -0.000000 | 2521 | 2425.543492 | 2461.58 | 2497.616508 | 0.976430 |  |
| GO:0006378\_mRNA\_polyadenylation | 5 | 0 | 0.000000 | -0.000000 | 2521 | 2425.543492 | 2461.58 | 2497.616508 | 0.976430 |  |
| GO:0006400\_tRNA\_modification | 5 | 0 | 0.000000 | -0.000000 | 2521 | 2425.543492 | 2461.58 | 2497.616508 | 0.976430 |  |
| GO:0006506\_GPI\_anchor\_biosynthetic\_process | 5 | 0 | 0.000000 | -0.000000 | 2521 | 2425.543492 | 2461.58 | 2497.616508 | 0.976430 |  |
| GO:0006509\_membrane\_protein\_ectodomain\_proteolysis | 5 | 0 | 0.000000 | -0.000000 | 2521 | 2425.543492 | 2461.58 | 2497.616508 | 0.976430 |  |
| GO:0006515\_misfolded\_or\_incompletely\_synthesized\_protein\_catabolic\_process | 5 | 0 | 0.000000 | -0.000000 | 2521 | 2425.543492 | 2461.58 | 2497.616508 | 0.976430 |  |
| GO:0006541\_glutamine\_metabolic\_process | 5 | 0 | 0.000000 | -0.000000 | 2521 | 2425.543492 | 2461.58 | 2497.616508 | 0.976430 |  |
| GO:0006570\_tyrosine\_metabolic\_process | 5 | 0 | 0.000000 | -0.000000 | 2521 | 2425.543492 | 2461.58 | 2497.616508 | 0.976430 |  |
| GO:0006577\_betaine\_metabolic\_process | 5 | 0 | 0.000000 | -0.000000 | 2521 | 2425.543492 | 2461.58 | 2497.616508 | 0.976430 |  |
| GO:0006586\_indolalkylamine\_metabolic\_process | 5 | 0 | 0.000000 | -0.000000 | 2521 | 2425.543492 | 2461.58 | 2497.616508 | 0.976430 |  |
| GO:0006636\_unsaturated\_fatty\_acid\_biosynthetic\_process | 5 | 0 | 0.000000 | -0.000000 | 2521 | 2425.543492 | 2461.58 | 2497.616508 | 0.976430 |  |
| GO:0006691\_leukotriene\_metabolic\_process | 5 | 0 | 0.000000 | -0.000000 | 2521 | 2425.543492 | 2461.58 | 2497.616508 | 0.976430 |  |
| GO:0006704\_glucocorticoid\_biosynthetic\_process | 5 | 0 | 0.000000 | -0.000000 | 2521 | 2425.543492 | 2461.58 | 2497.616508 | 0.976430 |  |
| GO:0006903\_vesicle\_targeting | 5 | 0 | 0.000000 | -0.000000 | 2521 | 2425.543492 | 2461.58 | 2497.616508 | 0.976430 |  |
| GO:0006929\_substrate-bound\_cell\_migration | 5 | 0 | 0.000000 | -0.000000 | 2521 | 2425.543492 | 2461.58 | 2497.616508 | 0.976430 |  |
| GO:0006983\_ER\_overload\_response | 5 | 0 | 0.000000 | -0.000000 | 2521 | 2425.543492 | 2461.58 | 2497.616508 | 0.976430 |  |
| GO:0007029\_endoplasmic\_reticulum\_organization | 5 | 0 | 0.000000 | -0.000000 | 2521 | 2425.543492 | 2461.58 | 2497.616508 | 0.976430 |  |
| GO:0007091\_mitotic\_metaphase\_anaphase\_transition | 5 | 0 | 0.000000 | -0.000000 | 2521 | 2425.543492 | 2461.58 | 2497.616508 | 0.976430 |  |
| GO:0007320\_insemination | 5 | 0 | 0.000000 | -0.000000 | 2521 | 2425.543492 | 2461.58 | 2497.616508 | 0.976430 |  |
| GO:0007622\_rhythmic\_behavior | 5 | 0 | 0.000000 | -0.000000 | 2521 | 2425.543492 | 2461.58 | 2497.616508 | 0.976430 |  |
| GO:0007638\_mechanosensory\_behavior | 5 | 0 | 0.000000 | -0.000000 | 2521 | 2425.543492 | 2461.58 | 2497.616508 | 0.976430 |  |
| GO:0008343\_adult\_feeding\_behavior | 5 | 0 | 0.000000 | -0.000000 | 2521 | 2425.543492 | 2461.58 | 2497.616508 | 0.976430 |  |
| GO:0008631\_induction\_of\_apoptosis\_by\_oxidative\_stress | 5 | 0 | 0.000000 | -0.000000 | 2521 | 2425.543492 | 2461.58 | 2497.616508 | 0.976430 |  |
| GO:0009132\_nucleoside\_diphosphate\_metabolic\_process | 5 | 0 | 0.000000 | -0.000000 | 2521 | 2425.543492 | 2461.58 | 2497.616508 | 0.976430 |  |
| GO:0009146\_purine\_nucleoside\_triphosphate\_catabolic\_process | 5 | 0 | 0.000000 | -0.000000 | 2521 | 2425.543492 | 2461.58 | 2497.616508 | 0.976430 |  |
| GO:0009151\_purine\_deoxyribonucleotide\_metabolic\_process | 5 | 0 | 0.000000 | -0.000000 | 2521 | 2425.543492 | 2461.58 | 2497.616508 | 0.976430 |  |
| GO:0009154\_purine\_ribonucleotide\_catabolic\_process | 5 | 0 | 0.000000 | -0.000000 | 2521 | 2425.543492 | 2461.58 | 2497.616508 | 0.976430 |  |
| GO:0009215\_purine\_deoxyribonucleoside\_triphosphate\_metabolic\_process | 5 | 0 | 0.000000 | -0.000000 | 2521 | 2425.543492 | 2461.58 | 2497.616508 | 0.976430 |  |
| GO:0009251\_glucan\_catabolic\_process | 5 | 0 | 0.000000 | -0.000000 | 2521 | 2425.543492 | 2461.58 | 2497.616508 | 0.976430 |  |
| GO:0009261\_ribonucleotide\_catabolic\_process | 5 | 0 | 0.000000 | -0.000000 | 2521 | 2425.543492 | 2461.58 | 2497.616508 | 0.976430 |  |
| GO:0009263\_deoxyribonucleotide\_biosynthetic\_process | 5 | 0 | 0.000000 | -0.000000 | 2521 | 2425.543492 | 2461.58 | 2497.616508 | 0.976430 |  |
| GO:0009264\_deoxyribonucleotide\_catabolic\_process | 5 | 0 | 0.000000 | -0.000000 | 2521 | 2425.543492 | 2461.58 | 2497.616508 | 0.976430 |  |
| GO:0009296\_flagellum\_assembly | 5 | 0 | 0.000000 | -0.000000 | 2521 | 2425.543492 | 2461.58 | 2497.616508 | 0.976430 |  |
| GO:0009303\_rRNA\_transcription | 5 | 0 | 0.000000 | -0.000000 | 2521 | 2425.543492 | 2461.58 | 2497.616508 | 0.976430 |  |
| GO:0009312\_oligosaccharide\_biosynthetic\_process | 5 | 0 | 0.000000 | -0.000000 | 2521 | 2425.543492 | 2461.58 | 2497.616508 | 0.976430 |  |
| GO:0009437\_carnitine\_metabolic\_process | 5 | 0 | 0.000000 | -0.000000 | 2521 | 2425.543492 | 2461.58 | 2497.616508 | 0.976430 |  |
| GO:0010460\_positive\_regulation\_of\_heart\_rate | 5 | 0 | 0.000000 | -0.000000 | 2521 | 2425.543492 | 2461.58 | 2497.616508 | 0.976430 |  |
| GO:0010522\_regulation\_of\_calcium\_ion\_transport\_into\_cytosol | 5 | 0 | 0.000000 | -0.000000 | 2521 | 2425.543492 | 2461.58 | 2497.616508 | 0.976430 |  |
| GO:0010676\_positive\_regulation\_of\_cellular\_carbohydrate\_metabolic\_process | 5 | 0 | 0.000000 | -0.000000 | 2521 | 2425.543492 | 2461.58 | 2497.616508 | 0.976430 |  |
| GO:0010828\_positive\_regulation\_of\_glucose\_transport | 5 | 0 | 0.000000 | -0.000000 | 2521 | 2425.543492 | 2461.58 | 2497.616508 | 0.976430 |  |
| GO:0014044\_Schwann\_cell\_development | 5 | 0 | 0.000000 | -0.000000 | 2521 | 2425.543492 | 2461.58 | 2497.616508 | 0.976430 |  |
| GO:0014068\_positive\_regulation\_of\_phosphoinositide\_3-kinase\_cascade | 5 | 0 | 0.000000 | -0.000000 | 2521 | 2425.543492 | 2461.58 | 2497.616508 | 0.976430 |  |
| GO:0015802\_basic\_amino\_acid\_transport | 5 | 0 | 0.000000 | -0.000000 | 2521 | 2425.543492 | 2461.58 | 2497.616508 | 0.976430 |  |
| GO:0016048\_detection\_of\_temperature\_stimulus | 5 | 0 | 0.000000 | -0.000000 | 2521 | 2425.543492 | 2461.58 | 2497.616508 | 0.976430 |  |
| GO:0016188\_synaptic\_vesicle\_maturation | 5 | 0 | 0.000000 | -0.000000 | 2521 | 2425.543492 | 2461.58 | 2497.616508 | 0.976430 |  |
| GO:0016579\_protein\_deubiquitination | 5 | 0 | 0.000000 | -0.000000 | 2521 | 2425.543492 | 2461.58 | 2497.616508 | 0.976430 |  |
| GO:0017148\_negative\_regulation\_of\_translation | 5 | 0 | 0.000000 | -0.000000 | 2521 | 2425.543492 | 2461.58 | 2497.616508 | 0.976430 |  |
| GO:0017158\_regulation\_of\_calcium\_ion-dependent\_exocytosis | 5 | 0 | 0.000000 | -0.000000 | 2521 | 2425.543492 | 2461.58 | 2497.616508 | 0.976430 |  |
| GO:0019227\_neuronal\_action\_potential\_propagation | 5 | 0 | 0.000000 | -0.000000 | 2521 | 2425.543492 | 2461.58 | 2497.616508 | 0.976430 |  |
| GO:0019430\_removal\_of\_superoxide\_radicals | 5 | 0 | 0.000000 | -0.000000 | 2521 | 2425.543492 | 2461.58 | 2497.616508 | 0.976430 |  |
| GO:0021554\_optic\_nerve\_development | 5 | 0 | 0.000000 | -0.000000 | 2521 | 2425.543492 | 2461.58 | 2497.616508 | 0.976430 |  |
| GO:0021772\_olfactory\_bulb\_development | 5 | 0 | 0.000000 | -0.000000 | 2521 | 2425.543492 | 2461.58 | 2497.616508 | 0.976430 |  |
| GO:0021826\_substrate-independent\_telencephalic\_tangential\_migration | 5 | 0 | 0.000000 | -0.000000 | 2521 | 2425.543492 | 2461.58 | 2497.616508 | 0.976430 |  |
| GO:0021843\_substrate-independent\_telencephalic\_tangential\_interneuron\_migration | 5 | 0 | 0.000000 | -0.000000 | 2521 | 2425.543492 | 2461.58 | 2497.616508 | 0.976430 |  |
| GO:0021854\_hypothalamus\_development | 5 | 0 | 0.000000 | -0.000000 | 2521 | 2425.543492 | 2461.58 | 2497.616508 | 0.976430 |  |
| GO:0021988\_olfactory\_lobe\_development | 5 | 0 | 0.000000 | -0.000000 | 2521 | 2425.543492 | 2461.58 | 2497.616508 | 0.976430 |  |
| GO:0022415\_viral\_reproductive\_process | 5 | 0 | 0.000000 | -0.000000 | 2521 | 2425.543492 | 2461.58 | 2497.616508 | 0.976430 |  |
| GO:0030004\_cellular\_monovalent\_inorganic\_cation\_homeostasis | 5 | 0 | 0.000000 | -0.000000 | 2521 | 2425.543492 | 2461.58 | 2497.616508 | 0.976430 |  |
| GO:0030261\_chromosome\_condensation | 5 | 0 | 0.000000 | -0.000000 | 2521 | 2425.543492 | 2461.58 | 2497.616508 | 0.976430 |  |
| GO:0030299\_intestinal\_cholesterol\_absorption | 5 | 0 | 0.000000 | -0.000000 | 2521 | 2425.543492 | 2461.58 | 2497.616508 | 0.976430 |  |
| GO:0030431\_sleep | 5 | 0 | 0.000000 | -0.000000 | 2521 | 2425.543492 | 2461.58 | 2497.616508 | 0.976430 |  |
| GO:0030433\_ER-associated\_protein\_catabolic\_process | 5 | 0 | 0.000000 | -0.000000 | 2521 | 2425.543492 | 2461.58 | 2497.616508 | 0.976430 |  |
| GO:0030641\_regulation\_of\_cellular\_pH | 5 | 0 | 0.000000 | -0.000000 | 2521 | 2425.543492 | 2461.58 | 2497.616508 | 0.976430 |  |
| GO:0030801\_positive\_regulation\_of\_cyclic\_nucleotide\_metabolic\_process | 5 | 0 | 0.000000 | -0.000000 | 2521 | 2425.543492 | 2461.58 | 2497.616508 | 0.976430 |  |
| GO:0030804\_positive\_regulation\_of\_cyclic\_nucleotide\_biosynthetic\_process | 5 | 0 | 0.000000 | -0.000000 | 2521 | 2425.543492 | 2461.58 | 2497.616508 | 0.976430 |  |
| GO:0030810\_positive\_regulation\_of\_nucleotide\_biosynthetic\_process | 5 | 0 | 0.000000 | -0.000000 | 2521 | 2425.543492 | 2461.58 | 2497.616508 | 0.976430 |  |
| GO:0030823\_regulation\_of\_cGMP\_metabolic\_process | 5 | 0 | 0.000000 | -0.000000 | 2521 | 2425.543492 | 2461.58 | 2497.616508 | 0.976430 |  |
| GO:0030838\_positive\_regulation\_of\_actin\_filament\_polymerization | 5 | 0 | 0.000000 | -0.000000 | 2521 | 2425.543492 | 2461.58 | 2497.616508 | 0.976430 |  |
| GO:0030851\_granulocyte\_differentiation | 5 | 0 | 0.000000 | -0.000000 | 2521 | 2425.543492 | 2461.58 | 2497.616508 | 0.976430 |  |
| GO:0030866\_cortical\_actin\_cytoskeleton\_organization | 5 | 0 | 0.000000 | -0.000000 | 2521 | 2425.543492 | 2461.58 | 2497.616508 | 0.976430 |  |
| GO:0030889\_negative\_regulation\_of\_B\_cell\_proliferation | 5 | 0 | 0.000000 | -0.000000 | 2521 | 2425.543492 | 2461.58 | 2497.616508 | 0.976430 |  |
| GO:0030917\_midbrain-hindbrain\_boundary\_development | 5 | 0 | 0.000000 | -0.000000 | 2521 | 2425.543492 | 2461.58 | 2497.616508 | 0.976430 |  |
| GO:0031047\_gene\_silencing\_by\_RNA | 5 | 0 | 0.000000 | -0.000000 | 2521 | 2425.543492 | 2461.58 | 2497.616508 | 0.976430 |  |
| GO:0031057\_negative\_regulation\_of\_histone\_modification | 5 | 0 | 0.000000 | -0.000000 | 2521 | 2425.543492 | 2461.58 | 2497.616508 | 0.976430 |  |
| GO:0031058\_positive\_regulation\_of\_histone\_modification | 5 | 0 | 0.000000 | -0.000000 | 2521 | 2425.543492 | 2461.58 | 2497.616508 | 0.976430 |  |
| GO:0031060\_regulation\_of\_histone\_methylation | 5 | 0 | 0.000000 | -0.000000 | 2521 | 2425.543492 | 2461.58 | 2497.616508 | 0.976430 |  |
| GO:0031122\_cytoplasmic\_microtubule\_organization | 5 | 0 | 0.000000 | -0.000000 | 2521 | 2425.543492 | 2461.58 | 2497.616508 | 0.976430 |  |
| GO:0031532\_actin\_cytoskeleton\_reorganization | 5 | 0 | 0.000000 | -0.000000 | 2521 | 2425.543492 | 2461.58 | 2497.616508 | 0.976430 |  |
| GO:0032024\_positive\_regulation\_of\_insulin\_secretion | 5 | 0 | 0.000000 | -0.000000 | 2521 | 2425.543492 | 2461.58 | 2497.616508 | 0.976430 |  |
| GO:0032320\_positive\_regulation\_of\_Ras\_GTPase\_activity | 5 | 0 | 0.000000 | -0.000000 | 2521 | 2425.543492 | 2461.58 | 2497.616508 | 0.976430 |  |
| GO:0032332\_positive\_regulation\_of\_chondrocyte\_differentiation | 5 | 0 | 0.000000 | -0.000000 | 2521 | 2425.543492 | 2461.58 | 2497.616508 | 0.976430 |  |
| GO:0032350\_regulation\_of\_hormone\_metabolic\_process | 5 | 0 | 0.000000 | -0.000000 | 2521 | 2425.543492 | 2461.58 | 2497.616508 | 0.976430 |  |
| GO:0032368\_regulation\_of\_lipid\_transport | 5 | 0 | 0.000000 | -0.000000 | 2521 | 2425.543492 | 2461.58 | 2497.616508 | 0.976430 |  |
| GO:0032400\_melanosome\_localization | 5 | 0 | 0.000000 | -0.000000 | 2521 | 2425.543492 | 2461.58 | 2497.616508 | 0.976430 |  |
| GO:0032434\_regulation\_of\_proteasomal\_ubiquitin-dependent\_protein\_catabolic\_process | 5 | 0 | 0.000000 | -0.000000 | 2521 | 2425.543492 | 2461.58 | 2497.616508 | 0.976430 |  |
| GO:0032479\_regulation\_of\_type\_I\_interferon\_production | 5 | 0 | 0.000000 | -0.000000 | 2521 | 2425.543492 | 2461.58 | 2497.616508 | 0.976430 |  |
| GO:0032494\_response\_to\_peptidoglycan | 5 | 0 | 0.000000 | -0.000000 | 2521 | 2425.543492 | 2461.58 | 2497.616508 | 0.976430 |  |
| GO:0032508\_DNA\_duplex\_unwinding | 5 | 0 | 0.000000 | -0.000000 | 2521 | 2425.543492 | 2461.58 | 2497.616508 | 0.976430 |  |
| GO:0032720\_negative\_regulation\_of\_tumor\_necrosis\_factor\_production | 5 | 0 | 0.000000 | -0.000000 | 2521 | 2425.543492 | 2461.58 | 2497.616508 | 0.976430 |  |
| GO:0032845\_negative\_regulation\_of\_homeostatic\_process | 5 | 0 | 0.000000 | -0.000000 | 2521 | 2425.543492 | 2461.58 | 2497.616508 | 0.976430 |  |
| GO:0032846\_positive\_regulation\_of\_homeostatic\_process | 5 | 0 | 0.000000 | -0.000000 | 2521 | 2425.543492 | 2461.58 | 2497.616508 | 0.976430 |  |
| GO:0033003\_regulation\_of\_mast\_cell\_activation | 5 | 0 | 0.000000 | -0.000000 | 2521 | 2425.543492 | 2461.58 | 2497.616508 | 0.976430 |  |
| GO:0033005\_positive\_regulation\_of\_mast\_cell\_activation | 5 | 0 | 0.000000 | -0.000000 | 2521 | 2425.543492 | 2461.58 | 2497.616508 | 0.976430 |  |
| GO:0033023\_mast\_cell\_homeostasis | 5 | 0 | 0.000000 | -0.000000 | 2521 | 2425.543492 | 2461.58 | 2497.616508 | 0.976430 |  |
| GO:0033024\_mast\_cell\_apoptosis | 5 | 0 | 0.000000 | -0.000000 | 2521 | 2425.543492 | 2461.58 | 2497.616508 | 0.976430 |  |
| GO:0033025\_regulation\_of\_mast\_cell\_apoptosis | 5 | 0 | 0.000000 | -0.000000 | 2521 | 2425.543492 | 2461.58 | 2497.616508 | 0.976430 |  |
| GO:0033033\_negative\_regulation\_of\_myeloid\_cell\_apoptosis | 5 | 0 | 0.000000 | -0.000000 | 2521 | 2425.543492 | 2461.58 | 2497.616508 | 0.976430 |  |
| GO:0033144\_negative\_regulation\_of\_steroid\_hormone\_receptor\_signaling\_pathway | 5 | 0 | 0.000000 | -0.000000 | 2521 | 2425.543492 | 2461.58 | 2497.616508 | 0.976430 |  |
| GO:0033146\_regulation\_of\_estrogen\_receptor\_signaling\_pathway | 5 | 0 | 0.000000 | -0.000000 | 2521 | 2425.543492 | 2461.58 | 2497.616508 | 0.976430 |  |
| GO:0033205\_cytokinesis\_during\_cell\_cycle | 5 | 0 | 0.000000 | -0.000000 | 2521 | 2425.543492 | 2461.58 | 2497.616508 | 0.976430 |  |
| GO:0033619\_membrane\_protein\_proteolysis | 5 | 0 | 0.000000 | -0.000000 | 2521 | 2425.543492 | 2461.58 | 2497.616508 | 0.976430 |  |
| GO:0033627\_cell\_adhesion\_mediated\_by\_integrin | 5 | 0 | 0.000000 | -0.000000 | 2521 | 2425.543492 | 2461.58 | 2497.616508 | 0.976430 |  |
| GO:0033692\_cellular\_polysaccharide\_biosynthetic\_process | 5 | 0 | 0.000000 | -0.000000 | 2521 | 2425.543492 | 2461.58 | 2497.616508 | 0.976430 |  |
| GO:0035058\_sensory\_cilium\_assembly | 5 | 0 | 0.000000 | -0.000000 | 2521 | 2425.543492 | 2461.58 | 2497.616508 | 0.976430 |  |
| GO:0035065\_regulation\_of\_histone\_acetylation | 5 | 0 | 0.000000 | -0.000000 | 2521 | 2425.543492 | 2461.58 | 2497.616508 | 0.976430 |  |
| GO:0035089\_establishment\_of\_apical\_basal\_cell\_polarity | 5 | 0 | 0.000000 | -0.000000 | 2521 | 2425.543492 | 2461.58 | 2497.616508 | 0.976430 |  |
| GO:0035095\_behavioral\_response\_to\_nicotine | 5 | 0 | 0.000000 | -0.000000 | 2521 | 2425.543492 | 2461.58 | 2497.616508 | 0.976430 |  |
| GO:0035234\_germ\_cell\_programmed\_cell\_death | 5 | 0 | 0.000000 | -0.000000 | 2521 | 2425.543492 | 2461.58 | 2497.616508 | 0.976430 |  |
| GO:0035238\_vitamin\_A\_biosynthetic\_process | 5 | 0 | 0.000000 | -0.000000 | 2521 | 2425.543492 | 2461.58 | 2497.616508 | 0.976430 |  |
| GO:0042362\_fat-soluble\_vitamin\_biosynthetic\_process | 5 | 0 | 0.000000 | -0.000000 | 2521 | 2425.543492 | 2461.58 | 2497.616508 | 0.976430 |  |
| GO:0042364\_water-soluble\_vitamin\_biosynthetic\_process | 5 | 0 | 0.000000 | -0.000000 | 2521 | 2425.543492 | 2461.58 | 2497.616508 | 0.976430 |  |
| GO:0042430\_indole\_and\_derivative\_metabolic\_process | 5 | 0 | 0.000000 | -0.000000 | 2521 | 2425.543492 | 2461.58 | 2497.616508 | 0.976430 |  |
| GO:0042434\_indole\_derivative\_metabolic\_process | 5 | 0 | 0.000000 | -0.000000 | 2521 | 2425.543492 | 2461.58 | 2497.616508 | 0.976430 |  |
| GO:0042487\_regulation\_of\_odontogenesis\_of\_dentine-containing\_tooth | 5 | 0 | 0.000000 | -0.000000 | 2521 | 2425.543492 | 2461.58 | 2497.616508 | 0.976430 |  |
| GO:0042506\_tyrosine\_phosphorylation\_of\_Stat5\_protein | 5 | 0 | 0.000000 | -0.000000 | 2521 | 2425.543492 | 2461.58 | 2497.616508 | 0.976430 |  |
| GO:0042516\_regulation\_of\_tyrosine\_phosphorylation\_of\_Stat3\_protein | 5 | 0 | 0.000000 | -0.000000 | 2521 | 2425.543492 | 2461.58 | 2497.616508 | 0.976430 |  |
| GO:0042531\_positive\_regulation\_of\_tyrosine\_phosphorylation\_of\_STAT\_protein | 5 | 0 | 0.000000 | -0.000000 | 2521 | 2425.543492 | 2461.58 | 2497.616508 | 0.976430 |  |
| GO:0042533\_tumor\_necrosis\_factor\_biosynthetic\_process | 5 | 0 | 0.000000 | -0.000000 | 2521 | 2425.543492 | 2461.58 | 2497.616508 | 0.976430 |  |
| GO:0042534\_regulation\_of\_tumor\_necrosis\_factor\_biosynthetic\_process | 5 | 0 | 0.000000 | -0.000000 | 2521 | 2425.543492 | 2461.58 | 2497.616508 | 0.976430 |  |
| GO:0042537\_benzene\_and\_derivative\_metabolic\_process | 5 | 0 | 0.000000 | -0.000000 | 2521 | 2425.543492 | 2461.58 | 2497.616508 | 0.976430 |  |
| GO:0042554\_superoxide\_anion\_generation | 5 | 0 | 0.000000 | -0.000000 | 2521 | 2425.543492 | 2461.58 | 2497.616508 | 0.976430 |  |
| GO:0042590\_antigen\_processing\_and\_presentation\_of\_exogenous\_peptide\_antigen\_via\_MHC\_class\_I | 5 | 0 | 0.000000 | -0.000000 | 2521 | 2425.543492 | 2461.58 | 2497.616508 | 0.976430 |  |
| GO:0042752\_regulation\_of\_circadian\_rhythm | 5 | 0 | 0.000000 | -0.000000 | 2521 | 2425.543492 | 2461.58 | 2497.616508 | 0.976430 |  |
| GO:0042756\_drinking\_behavior | 5 | 0 | 0.000000 | -0.000000 | 2521 | 2425.543492 | 2461.58 | 2497.616508 | 0.976430 |  |
| GO:0042904\_9-cis-retinoic\_acid\_biosynthetic\_process | 5 | 0 | 0.000000 | -0.000000 | 2521 | 2425.543492 | 2461.58 | 2497.616508 | 0.976430 |  |
| GO:0042905\_9-cis-retinoic\_acid\_metabolic\_process | 5 | 0 | 0.000000 | -0.000000 | 2521 | 2425.543492 | 2461.58 | 2497.616508 | 0.976430 |  |
| GO:0043046\_DNA\_methylation\_during\_gametogenesis | 5 | 0 | 0.000000 | -0.000000 | 2521 | 2425.543492 | 2461.58 | 2497.616508 | 0.976430 |  |
| GO:0043266\_regulation\_of\_potassium\_ion\_transport | 5 | 0 | 0.000000 | -0.000000 | 2521 | 2425.543492 | 2461.58 | 2497.616508 | 0.976430 |  |
| GO:0043277\_apoptotic\_cell\_clearance | 5 | 0 | 0.000000 | -0.000000 | 2521 | 2425.543492 | 2461.58 | 2497.616508 | 0.976430 |  |
| GO:0043302\_positive\_regulation\_of\_leukocyte\_degranulation | 5 | 0 | 0.000000 | -0.000000 | 2521 | 2425.543492 | 2461.58 | 2497.616508 | 0.976430 |  |
| GO:0043303\_mast\_cell\_degranulation | 5 | 0 | 0.000000 | -0.000000 | 2521 | 2425.543492 | 2461.58 | 2497.616508 | 0.976430 |  |
| GO:0043304\_regulation\_of\_mast\_cell\_degranulation | 5 | 0 | 0.000000 | -0.000000 | 2521 | 2425.543492 | 2461.58 | 2497.616508 | 0.976430 |  |
| GO:0043306\_positive\_regulation\_of\_mast\_cell\_degranulation | 5 | 0 | 0.000000 | -0.000000 | 2521 | 2425.543492 | 2461.58 | 2497.616508 | 0.976430 |  |
| GO:0043393\_regulation\_of\_protein\_binding | 5 | 0 | 0.000000 | -0.000000 | 2521 | 2425.543492 | 2461.58 | 2497.616508 | 0.976430 |  |
| GO:0043403\_skeletal\_muscle\_regeneration | 5 | 0 | 0.000000 | -0.000000 | 2521 | 2425.543492 | 2461.58 | 2497.616508 | 0.976430 |  |
| GO:0043489\_RNA\_stabilization | 5 | 0 | 0.000000 | -0.000000 | 2521 | 2425.543492 | 2461.58 | 2497.616508 | 0.976430 |  |
| GO:0043526\_neuroprotection | 5 | 0 | 0.000000 | -0.000000 | 2521 | 2425.543492 | 2461.58 | 2497.616508 | 0.976430 |  |
| GO:0043555\_regulation\_of\_translation\_in\_response\_to\_stress | 5 | 0 | 0.000000 | -0.000000 | 2521 | 2425.543492 | 2461.58 | 2497.616508 | 0.976430 |  |
| GO:0043558\_regulation\_of\_translational\_initiation\_in\_response\_to\_stress | 5 | 0 | 0.000000 | -0.000000 | 2521 | 2425.543492 | 2461.58 | 2497.616508 | 0.976430 |  |
| GO:0043631\_RNA\_polyadenylation | 5 | 0 | 0.000000 | -0.000000 | 2521 | 2425.543492 | 2461.58 | 2497.616508 | 0.976430 |  |
| GO:0043648\_dicarboxylic\_acid\_metabolic\_process | 5 | 0 | 0.000000 | -0.000000 | 2521 | 2425.543492 | 2461.58 | 2497.616508 | 0.976430 |  |
| GO:0044058\_regulation\_of\_digestive\_system\_process | 5 | 0 | 0.000000 | -0.000000 | 2521 | 2425.543492 | 2461.58 | 2497.616508 | 0.976430 |  |
| GO:0044241\_lipid\_digestion | 5 | 0 | 0.000000 | -0.000000 | 2521 | 2425.543492 | 2461.58 | 2497.616508 | 0.976430 |  |
| GO:0044247\_cellular\_polysaccharide\_catabolic\_process | 5 | 0 | 0.000000 | -0.000000 | 2521 | 2425.543492 | 2461.58 | 2497.616508 | 0.976430 |  |
| GO:0045063\_T-helper\_1\_cell\_differentiation | 5 | 0 | 0.000000 | -0.000000 | 2521 | 2425.543492 | 2461.58 | 2497.616508 | 0.976430 |  |
| GO:0045064\_T-helper\_2\_cell\_differentiation | 5 | 0 | 0.000000 | -0.000000 | 2521 | 2425.543492 | 2461.58 | 2497.616508 | 0.976430 |  |
| GO:0045080\_positive\_regulation\_of\_chemokine\_biosynthetic\_process | 5 | 0 | 0.000000 | -0.000000 | 2521 | 2425.543492 | 2461.58 | 2497.616508 | 0.976430 |  |
| GO:0045123\_cellular\_extravasation | 5 | 0 | 0.000000 | -0.000000 | 2521 | 2425.543492 | 2461.58 | 2497.616508 | 0.976430 |  |
| GO:0045197\_establishment\_or\_maintenance\_of\_epithelial\_cell\_apical\_basal\_polarity | 5 | 0 | 0.000000 | -0.000000 | 2521 | 2425.543492 | 2461.58 | 2497.616508 | 0.976430 |  |
| GO:0045213\_neurotransmitter\_receptor\_metabolic\_process | 5 | 0 | 0.000000 | -0.000000 | 2521 | 2425.543492 | 2461.58 | 2497.616508 | 0.976430 |  |
| GO:0045342\_MHC\_class\_II\_biosynthetic\_process | 5 | 0 | 0.000000 | -0.000000 | 2521 | 2425.543492 | 2461.58 | 2497.616508 | 0.976430 |  |
| GO:0045410\_positive\_regulation\_of\_interleukin-6\_biosynthetic\_process | 5 | 0 | 0.000000 | -0.000000 | 2521 | 2425.543492 | 2461.58 | 2497.616508 | 0.976430 |  |
| GO:0045586\_regulation\_of\_gamma-delta\_T\_cell\_differentiation | 5 | 0 | 0.000000 | -0.000000 | 2521 | 2425.543492 | 2461.58 | 2497.616508 | 0.976430 |  |
| GO:0045588\_positive\_regulation\_of\_gamma-delta\_T\_cell\_differentiation | 5 | 0 | 0.000000 | -0.000000 | 2521 | 2425.543492 | 2461.58 | 2497.616508 | 0.976430 |  |
| GO:0045622\_regulation\_of\_T-helper\_cell\_differentiation | 5 | 0 | 0.000000 | -0.000000 | 2521 | 2425.543492 | 2461.58 | 2497.616508 | 0.976430 |  |
| GO:0045648\_positive\_regulation\_of\_erythrocyte\_differentiation | 5 | 0 | 0.000000 | -0.000000 | 2521 | 2425.543492 | 2461.58 | 2497.616508 | 0.976430 |  |
| GO:0045651\_positive\_regulation\_of\_macrophage\_differentiation | 5 | 0 | 0.000000 | -0.000000 | 2521 | 2425.543492 | 2461.58 | 2497.616508 | 0.976430 |  |
| GO:0045661\_regulation\_of\_myoblast\_differentiation | 5 | 0 | 0.000000 | -0.000000 | 2521 | 2425.543492 | 2461.58 | 2497.616508 | 0.976430 |  |
| GO:0045730\_respiratory\_burst | 5 | 0 | 0.000000 | -0.000000 | 2521 | 2425.543492 | 2461.58 | 2497.616508 | 0.976430 |  |
| GO:0045744\_negative\_regulation\_of\_G-protein\_coupled\_receptor\_protein\_signaling\_pathway | 5 | 0 | 0.000000 | -0.000000 | 2521 | 2425.543492 | 2461.58 | 2497.616508 | 0.976430 |  |
| GO:0045745\_positive\_regulation\_of\_G-protein\_coupled\_receptor\_protein\_signaling\_pathway | 5 | 0 | 0.000000 | -0.000000 | 2521 | 2425.543492 | 2461.58 | 2497.616508 | 0.976430 |  |
| GO:0045773\_positive\_regulation\_of\_axon\_extension | 5 | 0 | 0.000000 | -0.000000 | 2521 | 2425.543492 | 2461.58 | 2497.616508 | 0.976430 |  |
| GO:0045851\_pH\_reduction | 5 | 0 | 0.000000 | -0.000000 | 2521 | 2425.543492 | 2461.58 | 2497.616508 | 0.976430 |  |
| GO:0045885\_positive\_regulation\_of\_survival\_gene\_product\_expression | 5 | 0 | 0.000000 | -0.000000 | 2521 | 2425.543492 | 2461.58 | 2497.616508 | 0.976430 |  |
| GO:0045953\_negative\_regulation\_of\_natural\_killer\_cell\_mediated\_cytotoxicity | 5 | 0 | 0.000000 | -0.000000 | 2521 | 2425.543492 | 2461.58 | 2497.616508 | 0.976430 |  |
| GO:0045981\_positive\_regulation\_of\_nucleotide\_metabolic\_process | 5 | 0 | 0.000000 | -0.000000 | 2521 | 2425.543492 | 2461.58 | 2497.616508 | 0.976430 |  |
| GO:0045987\_positive\_regulation\_of\_smooth\_muscle\_contraction | 5 | 0 | 0.000000 | -0.000000 | 2521 | 2425.543492 | 2461.58 | 2497.616508 | 0.976430 |  |
| GO:0046326\_positive\_regulation\_of\_glucose\_import | 5 | 0 | 0.000000 | -0.000000 | 2521 | 2425.543492 | 2461.58 | 2497.616508 | 0.976430 |  |
| GO:0046339\_diacylglycerol\_metabolic\_process | 5 | 0 | 0.000000 | -0.000000 | 2521 | 2425.543492 | 2461.58 | 2497.616508 | 0.976430 |  |
| GO:0046456\_icosanoid\_biosynthetic\_process | 5 | 0 | 0.000000 | -0.000000 | 2521 | 2425.543492 | 2461.58 | 2497.616508 | 0.976430 |  |
| GO:0046459\_short-chain\_fatty\_acid\_metabolic\_process | 5 | 0 | 0.000000 | -0.000000 | 2521 | 2425.543492 | 2461.58 | 2497.616508 | 0.976430 |  |
| GO:0046489\_phosphoinositide\_biosynthetic\_process | 5 | 0 | 0.000000 | -0.000000 | 2521 | 2425.543492 | 2461.58 | 2497.616508 | 0.976430 |  |
| GO:0046621\_negative\_regulation\_of\_organ\_growth | 5 | 0 | 0.000000 | -0.000000 | 2521 | 2425.543492 | 2461.58 | 2497.616508 | 0.976430 |  |
| GO:0046643\_regulation\_of\_gamma-delta\_T\_cell\_activation | 5 | 0 | 0.000000 | -0.000000 | 2521 | 2425.543492 | 2461.58 | 2497.616508 | 0.976430 |  |
| GO:0046645\_positive\_regulation\_of\_gamma-delta\_T\_cell\_activation | 5 | 0 | 0.000000 | -0.000000 | 2521 | 2425.543492 | 2461.58 | 2497.616508 | 0.976430 |  |
| GO:0046785\_microtubule\_polymerization | 5 | 0 | 0.000000 | -0.000000 | 2521 | 2425.543492 | 2461.58 | 2497.616508 | 0.976430 |  |
| GO:0048013\_ephrin\_receptor\_signaling\_pathway | 5 | 0 | 0.000000 | -0.000000 | 2521 | 2425.543492 | 2461.58 | 2497.616508 | 0.976430 |  |
| GO:0048149\_behavioral\_response\_to\_ethanol | 5 | 0 | 0.000000 | -0.000000 | 2521 | 2425.543492 | 2461.58 | 2497.616508 | 0.976430 |  |
| GO:0048255\_mRNA\_stabilization | 5 | 0 | 0.000000 | -0.000000 | 2521 | 2425.543492 | 2461.58 | 2497.616508 | 0.976430 |  |
| GO:0048289\_isotype\_switching\_to\_IgE\_isotypes | 5 | 0 | 0.000000 | -0.000000 | 2521 | 2425.543492 | 2461.58 | 2497.616508 | 0.976430 |  |
| GO:0048293\_regulation\_of\_isotype\_switching\_to\_IgE\_isotypes | 5 | 0 | 0.000000 | -0.000000 | 2521 | 2425.543492 | 2461.58 | 2497.616508 | 0.976430 |  |
| GO:0048485\_sympathetic\_nervous\_system\_development | 5 | 0 | 0.000000 | -0.000000 | 2521 | 2425.543492 | 2461.58 | 2497.616508 | 0.976430 |  |
| GO:0048532\_anatomical\_structure\_arrangement | 5 | 0 | 0.000000 | -0.000000 | 2521 | 2425.543492 | 2461.58 | 2497.616508 | 0.976430 |  |
| GO:0048570\_notochord\_morphogenesis | 5 | 0 | 0.000000 | -0.000000 | 2521 | 2425.543492 | 2461.58 | 2497.616508 | 0.976430 |  |
| GO:0048659\_smooth\_muscle\_cell\_proliferation | 5 | 0 | 0.000000 | -0.000000 | 2521 | 2425.543492 | 2461.58 | 2497.616508 | 0.976430 |  |
| GO:0048664\_neuron\_fate\_determination | 5 | 0 | 0.000000 | -0.000000 | 2521 | 2425.543492 | 2461.58 | 2497.616508 | 0.976430 |  |
| GO:0048715\_negative\_regulation\_of\_oligodendrocyte\_differentiation | 5 | 0 | 0.000000 | -0.000000 | 2521 | 2425.543492 | 2461.58 | 2497.616508 | 0.976430 |  |
| GO:0048934\_peripheral\_nervous\_system\_neuron\_differentiation | 5 | 0 | 0.000000 | -0.000000 | 2521 | 2425.543492 | 2461.58 | 2497.616508 | 0.976430 |  |
| GO:0050665\_hydrogen\_peroxide\_biosynthetic\_process | 5 | 0 | 0.000000 | -0.000000 | 2521 | 2425.543492 | 2461.58 | 2497.616508 | 0.976430 |  |
| GO:0050701\_interleukin-1\_secretion | 5 | 0 | 0.000000 | -0.000000 | 2521 | 2425.543492 | 2461.58 | 2497.616508 | 0.976430 |  |
| GO:0050715\_positive\_regulation\_of\_cytokine\_secretion | 5 | 0 | 0.000000 | -0.000000 | 2521 | 2425.543492 | 2461.58 | 2497.616508 | 0.976430 |  |
| GO:0050819\_negative\_regulation\_of\_coagulation | 5 | 0 | 0.000000 | -0.000000 | 2521 | 2425.543492 | 2461.58 | 2497.616508 | 0.976430 |  |
| GO:0050858\_negative\_regulation\_of\_antigen\_receptor-mediated\_signaling\_pathway | 5 | 0 | 0.000000 | -0.000000 | 2521 | 2425.543492 | 2461.58 | 2497.616508 | 0.976430 |  |
| GO:0050860\_negative\_regulation\_of\_T\_cell\_receptor\_signaling\_pathway | 5 | 0 | 0.000000 | -0.000000 | 2521 | 2425.543492 | 2461.58 | 2497.616508 | 0.976430 |  |
| GO:0051057\_positive\_regulation\_of\_small\_GTPase\_mediated\_signal\_transduction | 5 | 0 | 0.000000 | -0.000000 | 2521 | 2425.543492 | 2461.58 | 2497.616508 | 0.976430 |  |
| GO:0051136\_regulation\_of\_NK\_T\_cell\_differentiation | 5 | 0 | 0.000000 | -0.000000 | 2521 | 2425.543492 | 2461.58 | 2497.616508 | 0.976430 |  |
| GO:0051138\_positive\_regulation\_of\_NK\_T\_cell\_differentiation | 5 | 0 | 0.000000 | -0.000000 | 2521 | 2425.543492 | 2461.58 | 2497.616508 | 0.976430 |  |
| GO:0051177\_meiotic\_sister\_chromatid\_cohesion | 5 | 0 | 0.000000 | -0.000000 | 2521 | 2425.543492 | 2461.58 | 2497.616508 | 0.976430 |  |
| GO:0051289\_protein\_homotetramerization | 5 | 0 | 0.000000 | -0.000000 | 2521 | 2425.543492 | 2461.58 | 2497.616508 | 0.976430 |  |
| GO:0051298\_centrosome\_duplication | 5 | 0 | 0.000000 | -0.000000 | 2521 | 2425.543492 | 2461.58 | 2497.616508 | 0.976430 |  |
| GO:0051323\_metaphase | 5 | 0 | 0.000000 | -0.000000 | 2521 | 2425.543492 | 2461.58 | 2497.616508 | 0.976430 |  |
| GO:0051403\_stress-activated\_MAPK\_cascade | 5 | 0 | 0.000000 | -0.000000 | 2521 | 2425.543492 | 2461.58 | 2497.616508 | 0.976430 |  |
| GO:0051453\_regulation\_of\_intracellular\_pH | 5 | 0 | 0.000000 | -0.000000 | 2521 | 2425.543492 | 2461.58 | 2497.616508 | 0.976430 |  |
| GO:0051668\_localization\_within\_membrane | 5 | 0 | 0.000000 | -0.000000 | 2521 | 2425.543492 | 2461.58 | 2497.616508 | 0.976430 |  |
| GO:0051898\_negative\_regulation\_of\_protein\_kinase\_B\_signaling\_cascade | 5 | 0 | 0.000000 | -0.000000 | 2521 | 2425.543492 | 2461.58 | 2497.616508 | 0.976430 |  |
| GO:0051905\_establishment\_of\_pigment\_granule\_localization | 5 | 0 | 0.000000 | -0.000000 | 2521 | 2425.543492 | 2461.58 | 2497.616508 | 0.976430 |  |
| GO:0051954\_positive\_regulation\_of\_amine\_transport | 5 | 0 | 0.000000 | -0.000000 | 2521 | 2425.543492 | 2461.58 | 2497.616508 | 0.976430 |  |
| GO:0051966\_regulation\_of\_synaptic\_transmission\_\_glutamatergic | 5 | 0 | 0.000000 | -0.000000 | 2521 | 2425.543492 | 2461.58 | 2497.616508 | 0.976430 |  |
| GO:0060045\_positive\_regulation\_of\_cardiac\_muscle\_cell\_proliferation | 5 | 0 | 0.000000 | -0.000000 | 2521 | 2425.543492 | 2461.58 | 2497.616508 | 0.976430 |  |
| GO:0060073\_micturition | 5 | 0 | 0.000000 | -0.000000 | 2521 | 2425.543492 | 2461.58 | 2497.616508 | 0.976430 |  |
| GO:0060351\_cartilage\_development\_involved\_in\_endochondral\_bone\_morphogenesis | 5 | 0 | 0.000000 | -0.000000 | 2521 | 2425.543492 | 2461.58 | 2497.616508 | 0.976430 |  |
| GO:0060438\_trachea\_development | 5 | 0 | 0.000000 | -0.000000 | 2521 | 2425.543492 | 2461.58 | 2497.616508 | 0.976430 |  |
| GO:0060600\_dichotomous\_subdivision\_of\_an\_epithelial\_terminal\_unit | 5 | 0 | 0.000000 | -0.000000 | 2521 | 2425.543492 | 2461.58 | 2497.616508 | 0.976430 |  |
| GO:0060605\_tube\_lumen\_cavitation | 5 | 0 | 0.000000 | -0.000000 | 2521 | 2425.543492 | 2461.58 | 2497.616508 | 0.976430 |  |
| GO:0060662\_salivary\_gland\_cavitation | 5 | 0 | 0.000000 | -0.000000 | 2521 | 2425.543492 | 2461.58 | 2497.616508 | 0.976430 |  |
| GO:0060665\_regulation\_of\_branching\_involved\_in\_salivary\_gland\_morphogenesis\_by\_mesenchymal-epithelial\_signaling | 5 | 0 | 0.000000 | -0.000000 | 2521 | 2425.543492 | 2461.58 | 2497.616508 | 0.976430 |  |
| GO:0060707\_trophoblast\_giant\_cell\_differentiation | 5 | 0 | 0.000000 | -0.000000 | 2521 | 2425.543492 | 2461.58 | 2497.616508 | 0.976430 |  |
| GO:0060745\_mammary\_gland\_branching\_involved\_in\_pregnancy | 5 | 0 | 0.000000 | -0.000000 | 2521 | 2425.543492 | 2461.58 | 2497.616508 | 0.976430 |  |
| GO:0070371\_ERK1\_and\_ERK2\_cascade | 5 | 0 | 0.000000 | -0.000000 | 2521 | 2425.543492 | 2461.58 | 2497.616508 | 0.976430 |  |
| GO:0070372\_regulation\_of\_ERK1\_and\_ERK2\_cascade | 5 | 0 | 0.000000 | -0.000000 | 2521 | 2425.543492 | 2461.58 | 2497.616508 | 0.976430 |  |
| GO:0070374\_positive\_regulation\_of\_ERK1\_and\_ERK2\_cascade | 5 | 0 | 0.000000 | -0.000000 | 2521 | 2425.543492 | 2461.58 | 2497.616508 | 0.976430 |  |
| GO:0030334\_regulation\_of\_cell\_migration | 59 | 0 | 0.000000 | -0.000000 | 2523 | 2428.645038 | 2464.09 | 2499.534962 | 0.976651 |  |
| GO:0050870\_positive\_regulation\_of\_T\_cell\_activation | 59 | 0 | 0.000000 | -0.000000 | 2523 | 2428.645038 | 2464.09 | 2499.534962 | 0.976651 |  |
| GO:0032944\_regulation\_of\_mononuclear\_cell\_proliferation | 62 | 0 | 0.000000 | -0.000000 | 2526 | 2433.766441 | 2468.54 | 2503.313559 | 0.977253 |  |
| GO:0050670\_regulation\_of\_lymphocyte\_proliferation | 62 | 0 | 0.000000 | -0.000000 | 2526 | 2433.766441 | 2468.54 | 2503.313559 | 0.977253 |  |
| GO:0050954\_sensory\_perception\_of\_mechanical\_stimulus | 62 | 0 | 0.000000 | -0.000000 | 2526 | 2433.766441 | 2468.54 | 2503.313559 | 0.977253 |  |
| GO:0007126\_meiosis | 55 | 0 | 0.000000 | -0.000000 | 2529 | 2438.190649 | 2472.73 | 2507.269351 | 0.977750 |  |
| GO:0007605\_sensory\_perception\_of\_sound | 55 | 0 | 0.000000 | -0.000000 | 2529 | 2438.190649 | 2472.73 | 2507.269351 | 0.977750 |  |
| GO:0051327\_M\_phase\_of\_meiotic\_cell\_cycle | 55 | 0 | 0.000000 | -0.000000 | 2529 | 2438.190649 | 2472.73 | 2507.269351 | 0.977750 |  |
| GO:0001912\_positive\_regulation\_of\_leukocyte\_mediated\_cytotoxicity | 20 | 0 | 0.000000 | -0.000000 | 2549 | 2463.978859 | 2497.27 | 2530.561141 | 0.979706 |  |
| GO:0005977\_glycogen\_metabolic\_process | 20 | 0 | 0.000000 | -0.000000 | 2549 | 2463.978859 | 2497.27 | 2530.561141 | 0.979706 |  |
| GO:0006073\_cellular\_glucan\_metabolic\_process | 20 | 0 | 0.000000 | -0.000000 | 2549 | 2463.978859 | 2497.27 | 2530.561141 | 0.979706 |  |
| GO:0006518\_peptide\_metabolic\_process | 20 | 0 | 0.000000 | -0.000000 | 2549 | 2463.978859 | 2497.27 | 2530.561141 | 0.979706 |  |
| GO:0007416\_synaptogenesis | 20 | 0 | 0.000000 | -0.000000 | 2549 | 2463.978859 | 2497.27 | 2530.561141 | 0.979706 |  |
| GO:0007528\_neuromuscular\_junction\_development | 20 | 0 | 0.000000 | -0.000000 | 2549 | 2463.978859 | 2497.27 | 2530.561141 | 0.979706 |  |
| GO:0007586\_digestion | 20 | 0 | 0.000000 | -0.000000 | 2549 | 2463.978859 | 2497.27 | 2530.561141 | 0.979706 |  |
| GO:0009615\_response\_to\_virus | 20 | 0 | 0.000000 | -0.000000 | 2549 | 2463.978859 | 2497.27 | 2530.561141 | 0.979706 |  |
| GO:0016571\_histone\_methylation | 20 | 0 | 0.000000 | -0.000000 | 2549 | 2463.978859 | 2497.27 | 2530.561141 | 0.979706 |  |
| GO:0031128\_developmental\_induction | 20 | 0 | 0.000000 | -0.000000 | 2549 | 2463.978859 | 2497.27 | 2530.561141 | 0.979706 |  |
| GO:0031343\_positive\_regulation\_of\_cell\_killing | 20 | 0 | 0.000000 | -0.000000 | 2549 | 2463.978859 | 2497.27 | 2530.561141 | 0.979706 |  |
| GO:0032582\_negative\_regulation\_of\_gene-specific\_transcription | 20 | 0 | 0.000000 | -0.000000 | 2549 | 2463.978859 | 2497.27 | 2530.561141 | 0.979706 |  |
| GO:0032640\_tumor\_necrosis\_factor\_production | 20 | 0 | 0.000000 | -0.000000 | 2549 | 2463.978859 | 2497.27 | 2530.561141 | 0.979706 |  |
| GO:0032680\_regulation\_of\_tumor\_necrosis\_factor\_production | 20 | 0 | 0.000000 | -0.000000 | 2549 | 2463.978859 | 2497.27 | 2530.561141 | 0.979706 |  |
| GO:0044042\_glucan\_metabolic\_process | 20 | 0 | 0.000000 | -0.000000 | 2549 | 2463.978859 | 2497.27 | 2530.561141 | 0.979706 |  |
| GO:0045017\_glycerolipid\_biosynthetic\_process | 20 | 0 | 0.000000 | -0.000000 | 2549 | 2463.978859 | 2497.27 | 2530.561141 | 0.979706 |  |
| GO:0045168\_cell-cell\_signaling\_involved\_in\_cell\_fate\_specification | 20 | 0 | 0.000000 | -0.000000 | 2549 | 2463.978859 | 2497.27 | 2530.561141 | 0.979706 |  |
| GO:0045639\_positive\_regulation\_of\_myeloid\_cell\_differentiation | 20 | 0 | 0.000000 | -0.000000 | 2549 | 2463.978859 | 2497.27 | 2530.561141 | 0.979706 |  |
| GO:0046822\_regulation\_of\_nucleocytoplasmic\_transport | 20 | 0 | 0.000000 | -0.000000 | 2549 | 2463.978859 | 2497.27 | 2530.561141 | 0.979706 |  |
| GO:0060191\_regulation\_of\_lipase\_activity | 20 | 0 | 0.000000 | -0.000000 | 2549 | 2463.978859 | 2497.27 | 2530.561141 | 0.979706 |  |
| GO:0006638\_neutral\_lipid\_metabolic\_process | 32 | 0 | 0.000000 | -0.000000 | 2560 | 2475.041930 | 2507.37 | 2539.698070 | 0.979441 |  |
| GO:0006662\_glycerol\_ether\_metabolic\_process | 32 | 0 | 0.000000 | -0.000000 | 2560 | 2475.041930 | 2507.37 | 2539.698070 | 0.979441 |  |
| GO:0006937\_regulation\_of\_muscle\_contraction | 32 | 0 | 0.000000 | -0.000000 | 2560 | 2475.041930 | 2507.37 | 2539.698070 | 0.979441 |  |
| GO:0007249\_I-kappaB\_kinase\_NF-kappaB\_cascade | 32 | 0 | 0.000000 | -0.000000 | 2560 | 2475.041930 | 2507.37 | 2539.698070 | 0.979441 |  |
| GO:0007272\_ensheathment\_of\_neurons | 32 | 0 | 0.000000 | -0.000000 | 2560 | 2475.041930 | 2507.37 | 2539.698070 | 0.979441 |  |
| GO:0008366\_axon\_ensheathment | 32 | 0 | 0.000000 | -0.000000 | 2560 | 2475.041930 | 2507.37 | 2539.698070 | 0.979441 |  |
| GO:0042102\_positive\_regulation\_of\_T\_cell\_proliferation | 32 | 0 | 0.000000 | -0.000000 | 2560 | 2475.041930 | 2507.37 | 2539.698070 | 0.979441 |  |
| GO:0050768\_negative\_regulation\_of\_neurogenesis | 32 | 0 | 0.000000 | -0.000000 | 2560 | 2475.041930 | 2507.37 | 2539.698070 | 0.979441 |  |
| GO:0050770\_regulation\_of\_axonogenesis | 32 | 0 | 0.000000 | -0.000000 | 2560 | 2475.041930 | 2507.37 | 2539.698070 | 0.979441 |  |
| GO:0050885\_neuromuscular\_process\_controlling\_balance | 32 | 0 | 0.000000 | -0.000000 | 2560 | 2475.041930 | 2507.37 | 2539.698070 | 0.979441 |  |
| GO:0051259\_protein\_oligomerization | 32 | 0 | 0.000000 | -0.000000 | 2560 | 2475.041930 | 2507.37 | 2539.698070 | 0.979441 |  |
| GO:0001910\_regulation\_of\_leukocyte\_mediated\_cytotoxicity | 27 | 0 | 0.000000 | -0.000000 | 2572 | 2489.241743 | 2520.57 | 2551.898257 | 0.980004 |  |
| GO:0007422\_peripheral\_nervous\_system\_development | 27 | 0 | 0.000000 | -0.000000 | 2572 | 2489.241743 | 2520.57 | 2551.898257 | 0.980004 |  |
| GO:0008286\_insulin\_receptor\_signaling\_pathway | 27 | 0 | 0.000000 | -0.000000 | 2572 | 2489.241743 | 2520.57 | 2551.898257 | 0.980004 |  |
| GO:0009411\_response\_to\_UV | 27 | 0 | 0.000000 | -0.000000 | 2572 | 2489.241743 | 2520.57 | 2551.898257 | 0.980004 |  |
| GO:0009913\_epidermal\_cell\_differentiation | 27 | 0 | 0.000000 | -0.000000 | 2572 | 2489.241743 | 2520.57 | 2551.898257 | 0.980004 |  |
| GO:0019884\_antigen\_processing\_and\_presentation\_of\_exogenous\_antigen | 27 | 0 | 0.000000 | -0.000000 | 2572 | 2489.241743 | 2520.57 | 2551.898257 | 0.980004 |  |
| GO:0030100\_regulation\_of\_endocytosis | 27 | 0 | 0.000000 | -0.000000 | 2572 | 2489.241743 | 2520.57 | 2551.898257 | 0.980004 |  |
| GO:0031341\_regulation\_of\_cell\_killing | 27 | 0 | 0.000000 | -0.000000 | 2572 | 2489.241743 | 2520.57 | 2551.898257 | 0.980004 |  |
| GO:0032496\_response\_to\_lipopolysaccharide | 27 | 0 | 0.000000 | -0.000000 | 2572 | 2489.241743 | 2520.57 | 2551.898257 | 0.980004 |  |
| GO:0045582\_positive\_regulation\_of\_T\_cell\_differentiation | 27 | 0 | 0.000000 | -0.000000 | 2572 | 2489.241743 | 2520.57 | 2551.898257 | 0.980004 |  |
| GO:0051272\_positive\_regulation\_of\_cell\_motion | 27 | 0 | 0.000000 | -0.000000 | 2572 | 2489.241743 | 2520.57 | 2551.898257 | 0.980004 |  |
| GO:0070482\_response\_to\_oxygen\_levels | 27 | 0 | 0.000000 | -0.000000 | 2572 | 2489.241743 | 2520.57 | 2551.898257 | 0.980004 |  |
| GO:0002791\_regulation\_of\_peptide\_secretion | 17 | 0 | 0.000000 | -0.000000 | 2608 | 2528.269497 | 2558.6 | 2588.930503 | 0.981058 |  |
| GO:0006261\_DNA-dependent\_DNA\_replication | 17 | 0 | 0.000000 | -0.000000 | 2608 | 2528.269497 | 2558.6 | 2588.930503 | 0.981058 |  |
| GO:0006323\_DNA\_packaging | 17 | 0 | 0.000000 | -0.000000 | 2608 | 2528.269497 | 2558.6 | 2588.930503 | 0.981058 |  |
| GO:0006767\_water-soluble\_vitamin\_metabolic\_process | 17 | 0 | 0.000000 | -0.000000 | 2608 | 2528.269497 | 2558.6 | 2588.930503 | 0.981058 |  |
| GO:0006813\_potassium\_ion\_transport | 17 | 0 | 0.000000 | -0.000000 | 2608 | 2528.269497 | 2558.6 | 2588.930503 | 0.981058 |  |
| GO:0006919\_activation\_of\_caspase\_activity | 17 | 0 | 0.000000 | -0.000000 | 2608 | 2528.269497 | 2558.6 | 2588.930503 | 0.981058 |  |
| GO:0006984\_ER-nuclear\_signaling\_pathway | 17 | 0 | 0.000000 | -0.000000 | 2608 | 2528.269497 | 2558.6 | 2588.930503 | 0.981058 |  |
| GO:0008380\_RNA\_splicing | 17 | 0 | 0.000000 | -0.000000 | 2608 | 2528.269497 | 2558.6 | 2588.930503 | 0.981058 |  |
| GO:0010035\_response\_to\_inorganic\_substance | 17 | 0 | 0.000000 | -0.000000 | 2608 | 2528.269497 | 2558.6 | 2588.930503 | 0.981058 |  |
| GO:0010517\_regulation\_of\_phospholipase\_activity | 17 | 0 | 0.000000 | -0.000000 | 2608 | 2528.269497 | 2558.6 | 2588.930503 | 0.981058 |  |
| GO:0010565\_regulation\_of\_cellular\_ketone\_metabolic\_process | 17 | 0 | 0.000000 | -0.000000 | 2608 | 2528.269497 | 2558.6 | 2588.930503 | 0.981058 |  |
| GO:0010741\_negative\_regulation\_of\_protein\_kinase\_cascade | 17 | 0 | 0.000000 | -0.000000 | 2608 | 2528.269497 | 2558.6 | 2588.930503 | 0.981058 |  |
| GO:0015807\_L-amino\_acid\_transport | 17 | 0 | 0.000000 | -0.000000 | 2608 | 2528.269497 | 2558.6 | 2588.930503 | 0.981058 |  |
| GO:0016052\_carbohydrate\_catabolic\_process | 17 | 0 | 0.000000 | -0.000000 | 2608 | 2528.269497 | 2558.6 | 2588.930503 | 0.981058 |  |
| GO:0019395\_fatty\_acid\_oxidation | 17 | 0 | 0.000000 | -0.000000 | 2608 | 2528.269497 | 2558.6 | 2588.930503 | 0.981058 |  |
| GO:0021545\_cranial\_nerve\_development | 17 | 0 | 0.000000 | -0.000000 | 2608 | 2528.269497 | 2558.6 | 2588.930503 | 0.981058 |  |
| GO:0030101\_natural\_killer\_cell\_activation | 17 | 0 | 0.000000 | -0.000000 | 2608 | 2528.269497 | 2558.6 | 2588.930503 | 0.981058 |  |
| GO:0030317\_sperm\_motility | 17 | 0 | 0.000000 | -0.000000 | 2608 | 2528.269497 | 2558.6 | 2588.930503 | 0.981058 |  |
| GO:0034440\_lipid\_oxidation | 17 | 0 | 0.000000 | -0.000000 | 2608 | 2528.269497 | 2558.6 | 2588.930503 | 0.981058 |  |
| GO:0034470\_ncRNA\_processing | 17 | 0 | 0.000000 | -0.000000 | 2608 | 2528.269497 | 2558.6 | 2588.930503 | 0.981058 |  |
| GO:0042254\_ribosome\_biogenesis | 17 | 0 | 0.000000 | -0.000000 | 2608 | 2528.269497 | 2558.6 | 2588.930503 | 0.981058 |  |
| GO:0042384\_cilium\_assembly | 17 | 0 | 0.000000 | -0.000000 | 2608 | 2528.269497 | 2558.6 | 2588.930503 | 0.981058 |  |
| GO:0042440\_pigment\_metabolic\_process | 17 | 0 | 0.000000 | -0.000000 | 2608 | 2528.269497 | 2558.6 | 2588.930503 | 0.981058 |  |
| GO:0043407\_negative\_regulation\_of\_MAP\_kinase\_activity | 17 | 0 | 0.000000 | -0.000000 | 2608 | 2528.269497 | 2558.6 | 2588.930503 | 0.981058 |  |
| GO:0045333\_cellular\_respiration | 17 | 0 | 0.000000 | -0.000000 | 2608 | 2528.269497 | 2558.6 | 2588.930503 | 0.981058 |  |
| GO:0045786\_negative\_regulation\_of\_cell\_cycle | 17 | 0 | 0.000000 | -0.000000 | 2608 | 2528.269497 | 2558.6 | 2588.930503 | 0.981058 |  |
| GO:0046637\_regulation\_of\_alpha-beta\_T\_cell\_differentiation | 17 | 0 | 0.000000 | -0.000000 | 2608 | 2528.269497 | 2558.6 | 2588.930503 | 0.981058 |  |
| GO:0048168\_regulation\_of\_neuronal\_synaptic\_plasticity | 17 | 0 | 0.000000 | -0.000000 | 2608 | 2528.269497 | 2558.6 | 2588.930503 | 0.981058 |  |
| GO:0048265\_response\_to\_pain | 17 | 0 | 0.000000 | -0.000000 | 2608 | 2528.269497 | 2558.6 | 2588.930503 | 0.981058 |  |
| GO:0048873\_homeostasis\_of\_number\_of\_cells\_within\_a\_tissue | 17 | 0 | 0.000000 | -0.000000 | 2608 | 2528.269497 | 2558.6 | 2588.930503 | 0.981058 |  |
| GO:0051091\_positive\_regulation\_of\_transcription\_factor\_activity | 17 | 0 | 0.000000 | -0.000000 | 2608 | 2528.269497 | 2558.6 | 2588.930503 | 0.981058 |  |
| GO:0051605\_protein\_maturation\_by\_peptide\_bond\_cleavage | 17 | 0 | 0.000000 | -0.000000 | 2608 | 2528.269497 | 2558.6 | 2588.930503 | 0.981058 |  |
| GO:0055067\_monovalent\_inorganic\_cation\_homeostasis | 17 | 0 | 0.000000 | -0.000000 | 2608 | 2528.269497 | 2558.6 | 2588.930503 | 0.981058 |  |
| GO:0055072\_iron\_ion\_homeostasis | 17 | 0 | 0.000000 | -0.000000 | 2608 | 2528.269497 | 2558.6 | 2588.930503 | 0.981058 |  |
| GO:0060350\_endochondral\_bone\_morphogenesis | 17 | 0 | 0.000000 | -0.000000 | 2608 | 2528.269497 | 2558.6 | 2588.930503 | 0.981058 |  |
| GO:0090047\_positive\_regulation\_of\_transcription\_regulator\_activity | 17 | 0 | 0.000000 | -0.000000 | 2608 | 2528.269497 | 2558.6 | 2588.930503 | 0.981058 |  |
| GO:0001906\_cell\_killing | 35 | 0 | 0.000000 | -0.000000 | 2616 | 2537.879180 | 2567.83 | 2597.780820 | 0.981586 |  |
| GO:0001909\_leukocyte\_mediated\_cytotoxicity | 35 | 0 | 0.000000 | -0.000000 | 2616 | 2537.879180 | 2567.83 | 2597.780820 | 0.981586 |  |
| GO:0007292\_female\_gamete\_generation | 35 | 0 | 0.000000 | -0.000000 | 2616 | 2537.879180 | 2567.83 | 2597.780820 | 0.981586 |  |
| GO:0016567\_protein\_ubiquitination | 35 | 0 | 0.000000 | -0.000000 | 2616 | 2537.879180 | 2567.83 | 2597.780820 | 0.981586 |  |
| GO:0018904\_organic\_ether\_metabolic\_process | 35 | 0 | 0.000000 | -0.000000 | 2616 | 2537.879180 | 2567.83 | 2597.780820 | 0.981586 |  |
| GO:0051051\_negative\_regulation\_of\_transport | 35 | 0 | 0.000000 | -0.000000 | 2616 | 2537.879180 | 2567.83 | 2597.780820 | 0.981586 |  |
| GO:0051325\_interphase | 35 | 0 | 0.000000 | -0.000000 | 2616 | 2537.879180 | 2567.83 | 2597.780820 | 0.981586 |  |
| GO:0051329\_interphase\_of\_mitotic\_cell\_cycle | 35 | 0 | 0.000000 | -0.000000 | 2616 | 2537.879180 | 2567.83 | 2597.780820 | 0.981586 |  |
| GO:0006913\_nucleocytoplasmic\_transport | 71 | 0 | 0.000000 | -0.000000 | 2617 | 2540.360023 | 2570.01 | 2599.659977 | 0.982044 |  |
| GO:0000018\_regulation\_of\_DNA\_recombination | 21 | 0 | 0.000000 | -0.000000 | 2644 | 2575.074658 | 2602.26 | 2629.445342 | 0.984213 |  |
| GO:0000075\_cell\_cycle\_checkpoint | 21 | 0 | 0.000000 | -0.000000 | 2644 | 2575.074658 | 2602.26 | 2629.445342 | 0.984213 |  |
| GO:0001754\_eye\_photoreceptor\_cell\_differentiation | 21 | 0 | 0.000000 | -0.000000 | 2644 | 2575.074658 | 2602.26 | 2629.445342 | 0.984213 |  |
| GO:0002263\_cell\_activation\_during\_immune\_response | 21 | 0 | 0.000000 | -0.000000 | 2644 | 2575.074658 | 2602.26 | 2629.445342 | 0.984213 |  |
| GO:0002366\_leukocyte\_activation\_during\_immune\_response | 21 | 0 | 0.000000 | -0.000000 | 2644 | 2575.074658 | 2602.26 | 2629.445342 | 0.984213 |  |
| GO:0002456\_T\_cell\_mediated\_immunity | 21 | 0 | 0.000000 | -0.000000 | 2644 | 2575.074658 | 2602.26 | 2629.445342 | 0.984213 |  |
| GO:0006633\_fatty\_acid\_biosynthetic\_process | 21 | 0 | 0.000000 | -0.000000 | 2644 | 2575.074658 | 2602.26 | 2629.445342 | 0.984213 |  |
| GO:0006814\_sodium\_ion\_transport | 21 | 0 | 0.000000 | -0.000000 | 2644 | 2575.074658 | 2602.26 | 2629.445342 | 0.984213 |  |
| GO:0007189\_activation\_of\_adenylate\_cyclase\_activity\_by\_G-protein\_signaling\_pathway | 21 | 0 | 0.000000 | -0.000000 | 2644 | 2575.074658 | 2602.26 | 2629.445342 | 0.984213 |  |
| GO:0008637\_apoptotic\_mitochondrial\_changes | 21 | 0 | 0.000000 | -0.000000 | 2644 | 2575.074658 | 2602.26 | 2629.445342 | 0.984213 |  |
| GO:0010578\_regulation\_of\_adenylate\_cyclase\_activity\_involved\_in\_G-protein\_signaling | 21 | 0 | 0.000000 | -0.000000 | 2644 | 2575.074658 | 2602.26 | 2629.445342 | 0.984213 |  |
| GO:0010579\_positive\_regulation\_of\_adenylate\_cyclase\_activity\_by\_G-protein\_signaling\_pathway | 21 | 0 | 0.000000 | -0.000000 | 2644 | 2575.074658 | 2602.26 | 2629.445342 | 0.984213 |  |
| GO:0015844\_monoamine\_transport | 21 | 0 | 0.000000 | -0.000000 | 2644 | 2575.074658 | 2602.26 | 2629.445342 | 0.984213 |  |
| GO:0017157\_regulation\_of\_exocytosis | 21 | 0 | 0.000000 | -0.000000 | 2644 | 2575.074658 | 2602.26 | 2629.445342 | 0.984213 |  |
| GO:0019827\_stem\_cell\_maintenance | 21 | 0 | 0.000000 | -0.000000 | 2644 | 2575.074658 | 2602.26 | 2629.445342 | 0.984213 |  |
| GO:0021532\_neural\_tube\_patterning | 21 | 0 | 0.000000 | -0.000000 | 2644 | 2575.074658 | 2602.26 | 2629.445342 | 0.984213 |  |
| GO:0030216\_keratinocyte\_differentiation | 21 | 0 | 0.000000 | -0.000000 | 2644 | 2575.074658 | 2602.26 | 2629.445342 | 0.984213 |  |
| GO:0030888\_regulation\_of\_B\_cell\_proliferation | 21 | 0 | 0.000000 | -0.000000 | 2644 | 2575.074658 | 2602.26 | 2629.445342 | 0.984213 |  |
| GO:0032147\_activation\_of\_protein\_kinase\_activity | 21 | 0 | 0.000000 | -0.000000 | 2644 | 2575.074658 | 2602.26 | 2629.445342 | 0.984213 |  |
| GO:0034330\_cell\_junction\_organization | 21 | 0 | 0.000000 | -0.000000 | 2644 | 2575.074658 | 2602.26 | 2629.445342 | 0.984213 |  |
| GO:0043279\_response\_to\_alkaloid | 21 | 0 | 0.000000 | -0.000000 | 2644 | 2575.074658 | 2602.26 | 2629.445342 | 0.984213 |  |
| GO:0046519\_sphingoid\_metabolic\_process | 21 | 0 | 0.000000 | -0.000000 | 2644 | 2575.074658 | 2602.26 | 2629.445342 | 0.984213 |  |
| GO:0048675\_axon\_extension | 21 | 0 | 0.000000 | -0.000000 | 2644 | 2575.074658 | 2602.26 | 2629.445342 | 0.984213 |  |
| GO:0050777\_negative\_regulation\_of\_immune\_response | 21 | 0 | 0.000000 | -0.000000 | 2644 | 2575.074658 | 2602.26 | 2629.445342 | 0.984213 |  |
| GO:0051258\_protein\_polymerization | 21 | 0 | 0.000000 | -0.000000 | 2644 | 2575.074658 | 2602.26 | 2629.445342 | 0.984213 |  |
| GO:0051271\_negative\_regulation\_of\_cell\_motion | 21 | 0 | 0.000000 | -0.000000 | 2644 | 2575.074658 | 2602.26 | 2629.445342 | 0.984213 |  |
| GO:0051656\_establishment\_of\_organelle\_localization | 21 | 0 | 0.000000 | -0.000000 | 2644 | 2575.074658 | 2602.26 | 2629.445342 | 0.984213 |  |
| GO:0031347\_regulation\_of\_defense\_response | 67 | 0 | 0.000000 | -0.000000 | 2645 | 2577.798089 | 2604.53 | 2631.261911 | 0.984699 |  |
| GO:0002790\_peptide\_secretion | 37 | 0 | 0.000000 | -0.000000 | 2652 | 2586.166768 | 2612.31 | 2638.453232 | 0.985034 |  |
| GO:0019933\_cAMP-mediated\_signaling | 37 | 0 | 0.000000 | -0.000000 | 2652 | 2586.166768 | 2612.31 | 2638.453232 | 0.985034 |  |
| GO:0031349\_positive\_regulation\_of\_defense\_response | 37 | 0 | 0.000000 | -0.000000 | 2652 | 2586.166768 | 2612.31 | 2638.453232 | 0.985034 |  |
| GO:0032869\_cellular\_response\_to\_insulin\_stimulus | 37 | 0 | 0.000000 | -0.000000 | 2652 | 2586.166768 | 2612.31 | 2638.453232 | 0.985034 |  |
| GO:0045761\_regulation\_of\_adenylate\_cyclase\_activity | 37 | 0 | 0.000000 | -0.000000 | 2652 | 2586.166768 | 2612.31 | 2638.453232 | 0.985034 |  |
| GO:0051101\_regulation\_of\_DNA\_binding | 37 | 0 | 0.000000 | -0.000000 | 2652 | 2586.166768 | 2612.31 | 2638.453232 | 0.985034 |  |
| GO:0051250\_negative\_regulation\_of\_lymphocyte\_activation | 37 | 0 | 0.000000 | -0.000000 | 2652 | 2586.166768 | 2612.31 | 2638.453232 | 0.985034 |  |
| GO:0000087\_M\_phase\_of\_mitotic\_cell\_cycle | 25 | 0 | 0.000000 | -0.000000 | 2666 | 2606.065863 | 2630.48 | 2654.894137 | 0.986677 |  |
| GO:0002526\_acute\_inflammatory\_response | 25 | 0 | 0.000000 | -0.000000 | 2666 | 2606.065863 | 2630.48 | 2654.894137 | 0.986677 |  |
| GO:0007628\_adult\_walking\_behavior | 25 | 0 | 0.000000 | -0.000000 | 2666 | 2606.065863 | 2630.48 | 2654.894137 | 0.986677 |  |
| GO:0031348\_negative\_regulation\_of\_defense\_response | 25 | 0 | 0.000000 | -0.000000 | 2666 | 2606.065863 | 2630.48 | 2654.894137 | 0.986677 |  |
| GO:0032103\_positive\_regulation\_of\_response\_to\_external\_stimulus | 25 | 0 | 0.000000 | -0.000000 | 2666 | 2606.065863 | 2630.48 | 2654.894137 | 0.986677 |  |
| GO:0032945\_negative\_regulation\_of\_mononuclear\_cell\_proliferation | 25 | 0 | 0.000000 | -0.000000 | 2666 | 2606.065863 | 2630.48 | 2654.894137 | 0.986677 |  |
| GO:0043543\_protein\_amino\_acid\_acylation | 25 | 0 | 0.000000 | -0.000000 | 2666 | 2606.065863 | 2630.48 | 2654.894137 | 0.986677 |  |
| GO:0045089\_positive\_regulation\_of\_innate\_immune\_response | 25 | 0 | 0.000000 | -0.000000 | 2666 | 2606.065863 | 2630.48 | 2654.894137 | 0.986677 |  |
| GO:0048285\_organelle\_fission | 25 | 0 | 0.000000 | -0.000000 | 2666 | 2606.065863 | 2630.48 | 2654.894137 | 0.986677 |  |
| GO:0050672\_negative\_regulation\_of\_lymphocyte\_proliferation | 25 | 0 | 0.000000 | -0.000000 | 2666 | 2606.065863 | 2630.48 | 2654.894137 | 0.986677 |  |
| GO:0050852\_T\_cell\_receptor\_signaling\_pathway | 25 | 0 | 0.000000 | -0.000000 | 2666 | 2606.065863 | 2630.48 | 2654.894137 | 0.986677 |  |
| GO:0051090\_regulation\_of\_transcription\_factor\_activity | 25 | 0 | 0.000000 | -0.000000 | 2666 | 2606.065863 | 2630.48 | 2654.894137 | 0.986677 |  |
| GO:0070664\_negative\_regulation\_of\_leukocyte\_proliferation | 25 | 0 | 0.000000 | -0.000000 | 2666 | 2606.065863 | 2630.48 | 2654.894137 | 0.986677 |  |
| GO:0090046\_regulation\_of\_transcription\_regulator\_activity | 25 | 0 | 0.000000 | -0.000000 | 2666 | 2606.065863 | 2630.48 | 2654.894137 | 0.986677 |  |
| GO:0002768\_immune\_response-regulating\_cell\_surface\_receptor\_signaling\_pathway | 44 | 0 | 0.000000 | -0.000000 | 2675 | 2620.395647 | 2643.05 | 2665.704353 | 0.988056 |  |
| GO:0006606\_protein\_import\_into\_nucleus | 44 | 0 | 0.000000 | -0.000000 | 2675 | 2620.395647 | 2643.05 | 2665.704353 | 0.988056 |  |
| GO:0009914\_hormone\_transport | 44 | 0 | 0.000000 | -0.000000 | 2675 | 2620.395647 | 2643.05 | 2665.704353 | 0.988056 |  |
| GO:0030802\_regulation\_of\_cyclic\_nucleotide\_biosynthetic\_process | 44 | 0 | 0.000000 | -0.000000 | 2675 | 2620.395647 | 2643.05 | 2665.704353 | 0.988056 |  |
| GO:0030808\_regulation\_of\_nucleotide\_biosynthetic\_process | 44 | 0 | 0.000000 | -0.000000 | 2675 | 2620.395647 | 2643.05 | 2665.704353 | 0.988056 |  |
| GO:0044257\_cellular\_protein\_catabolic\_process | 44 | 0 | 0.000000 | -0.000000 | 2675 | 2620.395647 | 2643.05 | 2665.704353 | 0.988056 |  |
| GO:0051170\_nuclear\_import | 44 | 0 | 0.000000 | -0.000000 | 2675 | 2620.395647 | 2643.05 | 2665.704353 | 0.988056 |  |
| GO:0070665\_positive\_regulation\_of\_leukocyte\_proliferation | 44 | 0 | 0.000000 | -0.000000 | 2675 | 2620.395647 | 2643.05 | 2665.704353 | 0.988056 |  |
| GO:0070668\_positive\_regulation\_of\_mast\_cell\_proliferation | 44 | 0 | 0.000000 | -0.000000 | 2675 | 2620.395647 | 2643.05 | 2665.704353 | 0.988056 |  |
| GO:0015837\_amine\_transport | 66 | 0 | 0.000000 | -0.000000 | 2676 | 2623.661272 | 2645.72 | 2667.778728 | 0.988685 |  |
| GO:0000084\_S\_phase\_of\_mitotic\_cell\_cycle | 3 | 0 |  |  |  |  |  |  |  |  |
| GO:0000089\_mitotic\_metaphase | 3 | 0 |  |  |  |  |  |  |  |  |
| GO:0000098\_sulfur\_amino\_acid\_catabolic\_process | 3 | 0 |  |  |  |  |  |  |  |  |
| GO:0000103\_sulfate\_assimilation | 3 | 0 |  |  |  |  |  |  |  |  |
| GO:0000212\_meiotic\_spindle\_organization | 3 | 0 |  |  |  |  |  |  |  |  |
| GO:0000281\_cytokinesis\_after\_mitosis | 3 | 0 |  |  |  |  |  |  |  |  |
| GO:0000303\_response\_to\_superoxide | 3 | 0 |  |  |  |  |  |  |  |  |
| GO:0000320\_re-entry\_into\_mitotic\_cell\_cycle | 3 | 0 |  |  |  |  |  |  |  |  |
| GO:0000380\_alternative\_nuclear\_mRNA\_splicing\_\_via\_spliceosome | 3 | 0 |  |  |  |  |  |  |  |  |
| GO:0001516\_prostaglandin\_biosynthetic\_process | 3 | 0 |  |  |  |  |  |  |  |  |
| GO:0001553\_luteinization | 3 | 0 |  |  |  |  |  |  |  |  |
| GO:0001574\_ganglioside\_biosynthetic\_process | 3 | 0 |  |  |  |  |  |  |  |  |
| GO:0001705\_ectoderm\_formation | 3 | 0 |  |  |  |  |  |  |  |  |
| GO:0001778\_plasma\_membrane\_repair | 3 | 0 |  |  |  |  |  |  |  |  |
| GO:0001780\_neutrophil\_homeostasis | 3 | 0 |  |  |  |  |  |  |  |  |
| GO:0001802\_type\_III\_hypersensitivity | 3 | 0 |  |  |  |  |  |  |  |  |
| GO:0001803\_regulation\_of\_type\_III\_hypersensitivity | 3 | 0 |  |  |  |  |  |  |  |  |
| GO:0001805\_positive\_regulation\_of\_type\_III\_hypersensitivity | 3 | 0 |  |  |  |  |  |  |  |  |
| GO:0001812\_positive\_regulation\_of\_type\_I\_hypersensitivity | 3 | 0 |  |  |  |  |  |  |  |  |
| GO:0001831\_trophectodermal\_cellular\_morphogenesis | 3 | 0 |  |  |  |  |  |  |  |  |
| GO:0001844\_protein\_insertion\_into\_mitochondrial\_membrane\_during\_induction\_of\_apoptosis | 3 | 0 |  |  |  |  |  |  |  |  |
| GO:0001878\_response\_to\_yeast | 3 | 0 |  |  |  |  |  |  |  |  |
| GO:0001895\_retina\_homeostasis | 3 | 0 |  |  |  |  |  |  |  |  |
| GO:0001915\_negative\_regulation\_of\_T\_cell\_mediated\_cytotoxicity | 3 | 0 |  |  |  |  |  |  |  |  |
| GO:0001937\_negative\_regulation\_of\_endothelial\_cell\_proliferation | 3 | 0 |  |  |  |  |  |  |  |  |
| GO:0001953\_negative\_regulation\_of\_cell-matrix\_adhesion | 3 | 0 |  |  |  |  |  |  |  |  |
| GO:0001955\_blood\_vessel\_maturation | 3 | 0 |  |  |  |  |  |  |  |  |
| GO:0001960\_negative\_regulation\_of\_cytokine-mediated\_signaling\_pathway | 3 | 0 |  |  |  |  |  |  |  |  |
| GO:0001973\_adenosine\_receptor\_signaling\_pathway | 3 | 0 |  |  |  |  |  |  |  |  |
| GO:0001996\_positive\_regulation\_of\_heart\_rate\_by\_epinephrine-norepinephrine | 3 | 0 |  |  |  |  |  |  |  |  |
| GO:0002034\_regulation\_of\_blood\_vessel\_size\_by\_renin-angiotensin | 3 | 0 |  |  |  |  |  |  |  |  |
| GO:0002238\_response\_to\_molecule\_of\_fungal\_origin | 3 | 0 |  |  |  |  |  |  |  |  |
| GO:0002275\_myeloid\_cell\_activation\_during\_immune\_response | 3 | 0 |  |  |  |  |  |  |  |  |
| GO:0002281\_macrophage\_activation\_during\_immune\_response | 3 | 0 |  |  |  |  |  |  |  |  |
| GO:0002309\_T\_cell\_proliferation\_during\_immune\_response | 3 | 0 |  |  |  |  |  |  |  |  |
| GO:0002361\_CD4-positive\_\_CD25-positive\_\_alpha-beta\_regulatory\_T\_cell\_differentiation | 3 | 0 |  |  |  |  |  |  |  |  |
| GO:0002369\_T\_cell\_cytokine\_production | 3 | 0 |  |  |  |  |  |  |  |  |
| GO:0002428\_antigen\_processing\_and\_presentation\_of\_peptide\_antigen\_via\_MHC\_class\_Ib | 3 | 0 |  |  |  |  |  |  |  |  |
| GO:0002446\_neutrophil\_mediated\_immunity | 3 | 0 |  |  |  |  |  |  |  |  |
| GO:0002477\_antigen\_processing\_and\_presentation\_of\_exogenous\_peptide\_antigen\_via\_MHC\_class\_Ib | 3 | 0 |  |  |  |  |  |  |  |  |
| GO:0002481\_antigen\_processing\_and\_presentation\_of\_exogenous\_protein\_antigen\_via\_MHC\_class\_Ib\_\_TAP-dependent | 3 | 0 |  |  |  |  |  |  |  |  |
| GO:0002513\_tolerance\_induction\_to\_self\_antigen | 3 | 0 |  |  |  |  |  |  |  |  |
| GO:0002568\_somatic\_diversification\_of\_T\_cell\_receptor\_genes | 3 | 0 |  |  |  |  |  |  |  |  |
| GO:0002674\_negative\_regulation\_of\_acute\_inflammatory\_response | 3 | 0 |  |  |  |  |  |  |  |  |
| GO:0002681\_somatic\_recombination\_of\_T\_cell\_receptor\_gene\_segments | 3 | 0 |  |  |  |  |  |  |  |  |
| GO:0002713\_negative\_regulation\_of\_B\_cell\_mediated\_immunity | 3 | 0 |  |  |  |  |  |  |  |  |
| GO:0002827\_positive\_regulation\_of\_T-helper\_1\_type\_immune\_response | 3 | 0 |  |  |  |  |  |  |  |  |
| GO:0002865\_negative\_regulation\_of\_acute\_inflammatory\_response\_to\_antigenic\_stimulus | 3 | 0 |  |  |  |  |  |  |  |  |
| GO:0002884\_negative\_regulation\_of\_hypersensitivity | 3 | 0 |  |  |  |  |  |  |  |  |
| GO:0002890\_negative\_regulation\_of\_immunoglobulin\_mediated\_immune\_response | 3 | 0 |  |  |  |  |  |  |  |  |
| GO:0002904\_positive\_regulation\_of\_B\_cell\_apoptosis | 3 | 0 |  |  |  |  |  |  |  |  |
| GO:0003009\_skeletal\_muscle\_contraction | 3 | 0 |  |  |  |  |  |  |  |  |
| GO:0003072\_renal\_control\_of\_peripheral\_vascular\_resistance\_involved\_in\_regulation\_of\_systemic\_arterial\_blood\_pressure | 3 | 0 |  |  |  |  |  |  |  |  |
| GO:0006047\_UDP-N-acetylglucosamine\_metabolic\_process | 3 | 0 |  |  |  |  |  |  |  |  |
| GO:0006067\_ethanol\_metabolic\_process | 3 | 0 |  |  |  |  |  |  |  |  |
| GO:0006072\_glycerol-3-phosphate\_metabolic\_process | 3 | 0 |  |  |  |  |  |  |  |  |
| GO:0006103\_2-oxoglutarate\_metabolic\_process | 3 | 0 |  |  |  |  |  |  |  |  |
| GO:0006107\_oxaloacetate\_metabolic\_process | 3 | 0 |  |  |  |  |  |  |  |  |
| GO:0006166\_purine\_ribonucleoside\_salvage | 3 | 0 |  |  |  |  |  |  |  |  |
| GO:0006220\_pyrimidine\_nucleotide\_metabolic\_process | 3 | 0 |  |  |  |  |  |  |  |  |
| GO:0006282\_regulation\_of\_DNA\_repair | 3 | 0 |  |  |  |  |  |  |  |  |
| GO:0006287\_base-excision\_repair\_\_gap-filling | 3 | 0 |  |  |  |  |  |  |  |  |
| GO:0006301\_postreplication\_repair | 3 | 0 |  |  |  |  |  |  |  |  |
| GO:0006361\_transcription\_initiation\_from\_RNA\_polymerase\_I\_promoter | 3 | 0 |  |  |  |  |  |  |  |  |
| GO:0006367\_transcription\_initiation\_from\_RNA\_polymerase\_II\_promoter | 3 | 0 |  |  |  |  |  |  |  |  |
| GO:0006414\_translational\_elongation | 3 | 0 |  |  |  |  |  |  |  |  |
| GO:0006491\_N-glycan\_processing | 3 | 0 |  |  |  |  |  |  |  |  |
| GO:0006498\_N-terminal\_protein\_lipidation | 3 | 0 |  |  |  |  |  |  |  |  |
| GO:0006531\_aspartate\_metabolic\_process | 3 | 0 |  |  |  |  |  |  |  |  |
| GO:0006598\_polyamine\_catabolic\_process | 3 | 0 |  |  |  |  |  |  |  |  |
| GO:0006620\_posttranslational\_protein\_targeting\_to\_membrane | 3 | 0 |  |  |  |  |  |  |  |  |
| GO:0006625\_protein\_targeting\_to\_peroxisome | 3 | 0 |  |  |  |  |  |  |  |  |
| GO:0006651\_diacylglycerol\_biosynthetic\_process | 3 | 0 |  |  |  |  |  |  |  |  |
| GO:0006670\_sphingosine\_metabolic\_process | 3 | 0 |  |  |  |  |  |  |  |  |
| GO:0006677\_glycosylceramide\_metabolic\_process | 3 | 0 |  |  |  |  |  |  |  |  |
| GO:0006689\_ganglioside\_catabolic\_process | 3 | 0 |  |  |  |  |  |  |  |  |
| GO:0006699\_bile\_acid\_biosynthetic\_process | 3 | 0 |  |  |  |  |  |  |  |  |
| GO:0006791\_sulfur\_utilization | 3 | 0 |  |  |  |  |  |  |  |  |
| GO:0006817\_phosphate\_transport | 3 | 0 |  |  |  |  |  |  |  |  |
| GO:0006825\_copper\_ion\_transport | 3 | 0 |  |  |  |  |  |  |  |  |
| GO:0006828\_manganese\_ion\_transport | 3 | 0 |  |  |  |  |  |  |  |  |
| GO:0006857\_oligopeptide\_transport | 3 | 0 |  |  |  |  |  |  |  |  |
| GO:0006892\_post-Golgi\_vesicle-mediated\_transport | 3 | 0 |  |  |  |  |  |  |  |  |
| GO:0006926\_virus-infected\_cell\_apoptosis | 3 | 0 |  |  |  |  |  |  |  |  |
| GO:0006953\_acute-phase\_response | 3 | 0 |  |  |  |  |  |  |  |  |
| GO:0007000\_nucleolus\_organization | 3 | 0 |  |  |  |  |  |  |  |  |
| GO:0007041\_lysosomal\_transport | 3 | 0 |  |  |  |  |  |  |  |  |
| GO:0007043\_cell-cell\_junction\_assembly | 3 | 0 |  |  |  |  |  |  |  |  |
| GO:0007090\_regulation\_of\_S\_phase\_of\_mitotic\_cell\_cycle | 3 | 0 |  |  |  |  |  |  |  |  |
| GO:0007195\_inhibition\_of\_adenylate\_cyclase\_activity\_by\_dopamine\_receptor\_signaling\_pathway | 3 | 0 |  |  |  |  |  |  |  |  |
| GO:0007199\_G-protein\_signaling\_\_coupled\_to\_cGMP\_nucleotide\_second\_messenger | 3 | 0 |  |  |  |  |  |  |  |  |
| GO:0007213\_muscarinic\_acetylcholine\_receptor\_signaling\_pathway | 3 | 0 |  |  |  |  |  |  |  |  |
| GO:0007250\_activation\_of\_NF-kappaB-inducing\_kinase\_activity | 3 | 0 |  |  |  |  |  |  |  |  |
| GO:0007252\_I-kappaB\_phosphorylation | 3 | 0 |  |  |  |  |  |  |  |  |
| GO:0007262\_STAT\_protein\_nuclear\_translocation | 3 | 0 |  |  |  |  |  |  |  |  |
| GO:0007288\_sperm\_axoneme\_assembly | 3 | 0 |  |  |  |  |  |  |  |  |
| GO:0007350\_blastoderm\_segmentation | 3 | 0 |  |  |  |  |  |  |  |  |
| GO:0007412\_axon\_target\_recognition | 3 | 0 |  |  |  |  |  |  |  |  |
| GO:0007468\_regulation\_of\_rhodopsin\_gene\_expression | 3 | 0 |  |  |  |  |  |  |  |  |
| GO:0007525\_somatic\_muscle\_development | 3 | 0 |  |  |  |  |  |  |  |  |
| GO:0007635\_chemosensory\_behavior | 3 | 0 |  |  |  |  |  |  |  |  |
| GO:0008090\_retrograde\_axon\_cargo\_transport | 3 | 0 |  |  |  |  |  |  |  |  |
| GO:0008347\_glial\_cell\_migration | 3 | 0 |  |  |  |  |  |  |  |  |
| GO:0008635\_activation\_of\_caspase\_activity\_by\_cytochrome\_c | 3 | 0 |  |  |  |  |  |  |  |  |
| GO:0009060\_aerobic\_respiration | 3 | 0 |  |  |  |  |  |  |  |  |
| GO:0009081\_branched\_chain\_family\_amino\_acid\_metabolic\_process | 3 | 0 |  |  |  |  |  |  |  |  |
| GO:0009086\_methionine\_biosynthetic\_process | 3 | 0 |  |  |  |  |  |  |  |  |
| GO:0009135\_purine\_nucleoside\_diphosphate\_metabolic\_process | 3 | 0 |  |  |  |  |  |  |  |  |
| GO:0009137\_purine\_nucleoside\_diphosphate\_catabolic\_process | 3 | 0 |  |  |  |  |  |  |  |  |
| GO:0009155\_purine\_deoxyribonucleotide\_catabolic\_process | 3 | 0 |  |  |  |  |  |  |  |  |
| GO:0009179\_purine\_ribonucleoside\_diphosphate\_metabolic\_process | 3 | 0 |  |  |  |  |  |  |  |  |
| GO:0009181\_purine\_ribonucleoside\_diphosphate\_catabolic\_process | 3 | 0 |  |  |  |  |  |  |  |  |
| GO:0009185\_ribonucleoside\_diphosphate\_metabolic\_process | 3 | 0 |  |  |  |  |  |  |  |  |
| GO:0009191\_ribonucleoside\_diphosphate\_catabolic\_process | 3 | 0 |  |  |  |  |  |  |  |  |
| GO:0009199\_ribonucleoside\_triphosphate\_metabolic\_process | 3 | 0 |  |  |  |  |  |  |  |  |
| GO:0009204\_deoxyribonucleoside\_triphosphate\_catabolic\_process | 3 | 0 |  |  |  |  |  |  |  |  |
| GO:0009205\_purine\_ribonucleoside\_triphosphate\_metabolic\_process | 3 | 0 |  |  |  |  |  |  |  |  |
| GO:0009217\_purine\_deoxyribonucleoside\_triphosphate\_catabolic\_process | 3 | 0 |  |  |  |  |  |  |  |  |
| GO:0009448\_gamma-aminobutyric\_acid\_metabolic\_process | 3 | 0 |  |  |  |  |  |  |  |  |
| GO:0010043\_response\_to\_zinc\_ion | 3 | 0 |  |  |  |  |  |  |  |  |
| GO:0010159\_specification\_of\_organ\_position | 3 | 0 |  |  |  |  |  |  |  |  |
| GO:0010172\_embryonic\_body\_morphogenesis | 3 | 0 |  |  |  |  |  |  |  |  |
| GO:0010216\_maintenance\_of\_DNA\_methylation | 3 | 0 |  |  |  |  |  |  |  |  |
| GO:0010273\_detoxification\_of\_copper\_ion | 3 | 0 |  |  |  |  |  |  |  |  |
| GO:0010454\_negative\_regulation\_of\_cell\_fate\_commitment | 3 | 0 |  |  |  |  |  |  |  |  |
| GO:0010507\_negative\_regulation\_of\_autophagy | 3 | 0 |  |  |  |  |  |  |  |  |
| GO:0010524\_positive\_regulation\_of\_calcium\_ion\_transport\_into\_cytosol | 3 | 0 |  |  |  |  |  |  |  |  |
| GO:0010573\_vascular\_endothelial\_growth\_factor\_production | 3 | 0 |  |  |  |  |  |  |  |  |
| GO:0010574\_regulation\_of\_vascular\_endothelial\_growth\_factor\_production | 3 | 0 |  |  |  |  |  |  |  |  |
| GO:0010575\_positive\_regulation\_vascular\_endothelial\_growth\_factor\_production | 3 | 0 |  |  |  |  |  |  |  |  |
| GO:0010632\_regulation\_of\_epithelial\_cell\_migration | 3 | 0 |  |  |  |  |  |  |  |  |
| GO:0010717\_regulation\_of\_epithelial\_to\_mesenchymal\_transition | 3 | 0 |  |  |  |  |  |  |  |  |
| GO:0010884\_positive\_regulation\_of\_lipid\_storage | 3 | 0 |  |  |  |  |  |  |  |  |
| GO:0010888\_negative\_regulation\_of\_lipid\_storage | 3 | 0 |  |  |  |  |  |  |  |  |
| GO:0010889\_regulation\_of\_sequestering\_of\_triglyceride | 3 | 0 |  |  |  |  |  |  |  |  |
| GO:0010893\_positive\_regulation\_of\_steroid\_biosynthetic\_process | 3 | 0 |  |  |  |  |  |  |  |  |
| GO:0010894\_negative\_regulation\_of\_steroid\_biosynthetic\_process | 3 | 0 |  |  |  |  |  |  |  |  |
| GO:0010998\_regulation\_of\_translational\_initiation\_by\_eIF2\_alpha\_phosphorylation | 3 | 0 |  |  |  |  |  |  |  |  |
| GO:0010999\_regulation\_of\_eIF2\_alpha\_phosphorylation\_by\_heme | 3 | 0 |  |  |  |  |  |  |  |  |
| GO:0014074\_response\_to\_purine | 3 | 0 |  |  |  |  |  |  |  |  |
| GO:0014909\_smooth\_muscle\_cell\_migration | 3 | 0 |  |  |  |  |  |  |  |  |
| GO:0015669\_gas\_transport | 3 | 0 |  |  |  |  |  |  |  |  |
| GO:0015760\_glucose-6-phosphate\_transport | 3 | 0 |  |  |  |  |  |  |  |  |
| GO:0015816\_glycine\_transport | 3 | 0 |  |  |  |  |  |  |  |  |
| GO:0015838\_betaine\_transport | 3 | 0 |  |  |  |  |  |  |  |  |
| GO:0015871\_choline\_transport | 3 | 0 |  |  |  |  |  |  |  |  |
| GO:0015879\_carnitine\_transport | 3 | 0 |  |  |  |  |  |  |  |  |
| GO:0015893\_drug\_transport | 3 | 0 |  |  |  |  |  |  |  |  |
| GO:0015909\_long-chain\_fatty\_acid\_transport | 3 | 0 |  |  |  |  |  |  |  |  |
| GO:0015936\_coenzyme\_A\_metabolic\_process | 3 | 0 |  |  |  |  |  |  |  |  |
| GO:0016241\_regulation\_of\_macroautophagy | 3 | 0 |  |  |  |  |  |  |  |  |
| GO:0016322\_neuron\_remodeling | 3 | 0 |  |  |  |  |  |  |  |  |
| GO:0016556\_mRNA\_modification | 3 | 0 |  |  |  |  |  |  |  |  |
| GO:0016973\_poly(A)+\_mRNA\_export\_from\_nucleus | 3 | 0 |  |  |  |  |  |  |  |  |
| GO:0018196\_peptidyl-asparagine\_modification | 3 | 0 |  |  |  |  |  |  |  |  |
| GO:0018208\_peptidyl-proline\_modification | 3 | 0 |  |  |  |  |  |  |  |  |
| GO:0018279\_protein\_amino\_acid\_N-linked\_glycosylation\_via\_asparagine | 3 | 0 |  |  |  |  |  |  |  |  |
| GO:0018894\_dibenzo-p-dioxin\_metabolic\_process | 3 | 0 |  |  |  |  |  |  |  |  |
| GO:0019058\_viral\_infectious\_cycle | 3 | 0 |  |  |  |  |  |  |  |  |
| GO:0019230\_proprioception | 3 | 0 |  |  |  |  |  |  |  |  |
| GO:0019236\_response\_to\_pheromone | 3 | 0 |  |  |  |  |  |  |  |  |
| GO:0019359\_nicotinamide\_nucleotide\_biosynthetic\_process | 3 | 0 |  |  |  |  |  |  |  |  |
| GO:0019363\_pyridine\_nucleotide\_biosynthetic\_process | 3 | 0 |  |  |  |  |  |  |  |  |
| GO:0019438\_aromatic\_compound\_biosynthetic\_process | 3 | 0 |  |  |  |  |  |  |  |  |
| GO:0019439\_aromatic\_compound\_catabolic\_process | 3 | 0 |  |  |  |  |  |  |  |  |
| GO:0019605\_butyrate\_metabolic\_process | 3 | 0 |  |  |  |  |  |  |  |  |
| GO:0019614\_catechol\_catabolic\_process | 3 | 0 |  |  |  |  |  |  |  |  |
| GO:0019674\_NAD\_metabolic\_process | 3 | 0 |  |  |  |  |  |  |  |  |
| GO:0019852\_L-ascorbic\_acid\_metabolic\_process | 3 | 0 |  |  |  |  |  |  |  |  |
| GO:0019934\_cGMP-mediated\_signaling | 3 | 0 |  |  |  |  |  |  |  |  |
| GO:0019987\_negative\_regulation\_of\_anti-apoptosis | 3 | 0 |  |  |  |  |  |  |  |  |
| GO:0021527\_spinal\_cord\_association\_neuron\_differentiation | 3 | 0 |  |  |  |  |  |  |  |  |
| GO:0021529\_spinal\_cord\_oligodendrocyte\_cell\_differentiation | 3 | 0 |  |  |  |  |  |  |  |  |
| GO:0021530\_spinal\_cord\_oligodendrocyte\_cell\_fate\_specification | 3 | 0 |  |  |  |  |  |  |  |  |
| GO:0021555\_midbrain-hindbrain\_boundary\_morphogenesis | 3 | 0 |  |  |  |  |  |  |  |  |
| GO:0021563\_glossopharyngeal\_nerve\_development | 3 | 0 |  |  |  |  |  |  |  |  |
| GO:0021570\_rhombomere\_4\_development | 3 | 0 |  |  |  |  |  |  |  |  |
| GO:0021591\_ventricular\_system\_development | 3 | 0 |  |  |  |  |  |  |  |  |
| GO:0021615\_glossopharyngeal\_nerve\_morphogenesis | 3 | 0 |  |  |  |  |  |  |  |  |
| GO:0021794\_thalamus\_development | 3 | 0 |  |  |  |  |  |  |  |  |
| GO:0021797\_forebrain\_anterior\_posterior\_pattern\_formation | 3 | 0 |  |  |  |  |  |  |  |  |
| GO:0021798\_forebrain\_dorsal\_ventral\_pattern\_formation | 3 | 0 |  |  |  |  |  |  |  |  |
| GO:0021800\_cerebral\_cortex\_tangential\_migration | 3 | 0 |  |  |  |  |  |  |  |  |
| GO:0021859\_pyramidal\_neuron\_differentiation | 3 | 0 |  |  |  |  |  |  |  |  |
| GO:0021860\_pyramidal\_neuron\_development | 3 | 0 |  |  |  |  |  |  |  |  |
| GO:0021889\_olfactory\_bulb\_interneuron\_differentiation | 3 | 0 |  |  |  |  |  |  |  |  |
| GO:0021891\_olfactory\_bulb\_interneuron\_development | 3 | 0 |  |  |  |  |  |  |  |  |
| GO:0021912\_regulation\_of\_transcription\_from\_RNA\_polymerase\_II\_promoter\_involved\_in\_spinal\_cord\_motor\_neuron\_fate\_specification | 3 | 0 |  |  |  |  |  |  |  |  |
| GO:0021979\_hypothalamus\_cell\_differentiation | 3 | 0 |  |  |  |  |  |  |  |  |
| GO:0022010\_myelination\_in\_the\_central\_nervous\_system | 3 | 0 |  |  |  |  |  |  |  |  |
| GO:0022027\_interkinetic\_nuclear\_migration | 3 | 0 |  |  |  |  |  |  |  |  |
| GO:0030033\_microvillus\_assembly | 3 | 0 |  |  |  |  |  |  |  |  |
| GO:0030091\_protein\_repair | 3 | 0 |  |  |  |  |  |  |  |  |
| GO:0030195\_negative\_regulation\_of\_blood\_coagulation | 3 | 0 |  |  |  |  |  |  |  |  |
| GO:0030224\_monocyte\_differentiation | 3 | 0 |  |  |  |  |  |  |  |  |
| GO:0030307\_positive\_regulation\_of\_cell\_growth | 3 | 0 |  |  |  |  |  |  |  |  |
| GO:0030319\_cellular\_di-\_\_tri-valent\_inorganic\_anion\_homeostasis | 3 | 0 |  |  |  |  |  |  |  |  |
| GO:0030320\_cellular\_monovalent\_inorganic\_anion\_homeostasis | 3 | 0 |  |  |  |  |  |  |  |  |
| GO:0030321\_transepithelial\_chloride\_transport | 3 | 0 |  |  |  |  |  |  |  |  |
| GO:0030501\_positive\_regulation\_of\_bone\_mineralization | 3 | 0 |  |  |  |  |  |  |  |  |
| GO:0030513\_positive\_regulation\_of\_BMP\_signaling\_pathway | 3 | 0 |  |  |  |  |  |  |  |  |
| GO:0030538\_embryonic\_genitalia\_morphogenesis | 3 | 0 |  |  |  |  |  |  |  |  |
| GO:0030574\_collagen\_catabolic\_process | 3 | 0 |  |  |  |  |  |  |  |  |
| GO:0030643\_cellular\_phosphate\_ion\_homeostasis | 3 | 0 |  |  |  |  |  |  |  |  |
| GO:0030718\_germ-line\_stem\_cell\_maintenance | 3 | 0 |  |  |  |  |  |  |  |  |
| GO:0030730\_sequestering\_of\_triglyceride | 3 | 0 |  |  |  |  |  |  |  |  |
| GO:0030857\_negative\_regulation\_of\_epithelial\_cell\_differentiation | 3 | 0 |  |  |  |  |  |  |  |  |
| GO:0030916\_otic\_vesicle\_formation | 3 | 0 |  |  |  |  |  |  |  |  |
| GO:0031000\_response\_to\_caffeine | 3 | 0 |  |  |  |  |  |  |  |  |
| GO:0031063\_regulation\_of\_histone\_deacetylation | 3 | 0 |  |  |  |  |  |  |  |  |
| GO:0031065\_positive\_regulation\_of\_histone\_deacetylation | 3 | 0 |  |  |  |  |  |  |  |  |
| GO:0031112\_positive\_regulation\_of\_microtubule\_polymerization\_or\_depolymerization | 3 | 0 |  |  |  |  |  |  |  |  |
| GO:0031116\_positive\_regulation\_of\_microtubule\_polymerization | 3 | 0 |  |  |  |  |  |  |  |  |
| GO:0031133\_regulation\_of\_axon\_diameter | 3 | 0 |  |  |  |  |  |  |  |  |
| GO:0031282\_regulation\_of\_guanylate\_cyclase\_activity | 3 | 0 |  |  |  |  |  |  |  |  |
| GO:0031333\_negative\_regulation\_of\_protein\_complex\_assembly | 3 | 0 |  |  |  |  |  |  |  |  |
| GO:0031397\_negative\_regulation\_of\_protein\_ubiquitination | 3 | 0 |  |  |  |  |  |  |  |  |
| GO:0031398\_positive\_regulation\_of\_protein\_ubiquitination | 3 | 0 |  |  |  |  |  |  |  |  |
| GO:0031503\_protein\_complex\_localization | 3 | 0 |  |  |  |  |  |  |  |  |
| GO:0031571\_G1\_DNA\_damage\_checkpoint | 3 | 0 |  |  |  |  |  |  |  |  |
| GO:0031579\_membrane\_raft\_organization | 3 | 0 |  |  |  |  |  |  |  |  |
| GO:0031638\_zymogen\_activation | 3 | 0 |  |  |  |  |  |  |  |  |
| GO:0031641\_regulation\_of\_myelination | 3 | 0 |  |  |  |  |  |  |  |  |
| GO:0031642\_negative\_regulation\_of\_myelination | 3 | 0 |  |  |  |  |  |  |  |  |
| GO:0031649\_heat\_generation | 3 | 0 |  |  |  |  |  |  |  |  |
| GO:0031943\_regulation\_of\_glucocorticoid\_metabolic\_process | 3 | 0 |  |  |  |  |  |  |  |  |
| GO:0032020\_ISG15-protein\_conjugation | 3 | 0 |  |  |  |  |  |  |  |  |
| GO:0032060\_bleb\_formation | 3 | 0 |  |  |  |  |  |  |  |  |
| GO:0032095\_regulation\_of\_response\_to\_food | 3 | 0 |  |  |  |  |  |  |  |  |
| GO:0032272\_negative\_regulation\_of\_protein\_polymerization | 3 | 0 |  |  |  |  |  |  |  |  |
| GO:0032288\_myelin\_assembly | 3 | 0 |  |  |  |  |  |  |  |  |
| GO:0032291\_ensheathment\_of\_axons\_in\_the\_central\_nervous\_system | 3 | 0 |  |  |  |  |  |  |  |  |
| GO:0032355\_response\_to\_estradiol\_stimulus | 3 | 0 |  |  |  |  |  |  |  |  |
| GO:0032402\_melanosome\_transport | 3 | 0 |  |  |  |  |  |  |  |  |
| GO:0032411\_positive\_regulation\_of\_transporter\_activity | 3 | 0 |  |  |  |  |  |  |  |  |
| GO:0032414\_positive\_regulation\_of\_ion\_transmembrane\_transporter\_activity | 3 | 0 |  |  |  |  |  |  |  |  |
| GO:0032436\_positive\_regulation\_of\_proteasomal\_ubiquitin-dependent\_protein\_catabolic\_process | 3 | 0 |  |  |  |  |  |  |  |  |
| GO:0032528\_microvillus\_organization | 3 | 0 |  |  |  |  |  |  |  |  |
| GO:0032536\_regulation\_of\_cell\_projection\_size | 3 | 0 |  |  |  |  |  |  |  |  |
| GO:0032632\_interleukin-3\_production | 3 | 0 |  |  |  |  |  |  |  |  |
| GO:0032634\_interleukin-5\_production | 3 | 0 |  |  |  |  |  |  |  |  |
| GO:0032674\_regulation\_of\_interleukin-5\_production | 3 | 0 |  |  |  |  |  |  |  |  |
| GO:0032703\_negative\_regulation\_of\_interleukin-2\_production | 3 | 0 |  |  |  |  |  |  |  |  |
| GO:0032753\_positive\_regulation\_of\_interleukin-4\_production | 3 | 0 |  |  |  |  |  |  |  |  |
| GO:0032823\_regulation\_of\_natural\_killer\_cell\_differentiation | 3 | 0 |  |  |  |  |  |  |  |  |
| GO:0032825\_positive\_regulation\_of\_natural\_killer\_cell\_differentiation | 3 | 0 |  |  |  |  |  |  |  |  |
| GO:0032856\_activation\_of\_Ras\_GTPase\_activity | 3 | 0 |  |  |  |  |  |  |  |  |
| GO:0032862\_activation\_of\_Rho\_GTPase\_activity | 3 | 0 |  |  |  |  |  |  |  |  |
| GO:0032874\_positive\_regulation\_of\_stress-activated\_MAPK\_cascade | 3 | 0 |  |  |  |  |  |  |  |  |
| GO:0032881\_regulation\_of\_polysaccharide\_metabolic\_process | 3 | 0 |  |  |  |  |  |  |  |  |
| GO:0032890\_regulation\_of\_organic\_acid\_transport | 3 | 0 |  |  |  |  |  |  |  |  |
| GO:0033058\_directional\_locomotion | 3 | 0 |  |  |  |  |  |  |  |  |
| GO:0033080\_immature\_T\_cell\_proliferation\_in\_the\_thymus | 3 | 0 |  |  |  |  |  |  |  |  |
| GO:0033084\_regulation\_of\_immature\_T\_cell\_proliferation\_in\_the\_thymus | 3 | 0 |  |  |  |  |  |  |  |  |
| GO:0033091\_positive\_regulation\_of\_immature\_T\_cell\_proliferation | 3 | 0 |  |  |  |  |  |  |  |  |
| GO:0033137\_negative\_regulation\_of\_peptidyl-serine\_phosphorylation | 3 | 0 |  |  |  |  |  |  |  |  |
| GO:0033153\_T\_cell\_receptor\_V(D)J\_recombination | 3 | 0 |  |  |  |  |  |  |  |  |
| GO:0033209\_tumor\_necrosis\_factor-mediated\_signaling\_pathway | 3 | 0 |  |  |  |  |  |  |  |  |
| GO:0033261\_regulation\_of\_S\_phase | 3 | 0 |  |  |  |  |  |  |  |  |
| GO:0033600\_negative\_regulation\_of\_mammary\_gland\_epithelial\_cell\_proliferation | 3 | 0 |  |  |  |  |  |  |  |  |
| GO:0033631\_cell-cell\_adhesion\_mediated\_by\_integrin | 3 | 0 |  |  |  |  |  |  |  |  |
| GO:0033993\_response\_to\_lipid | 3 | 0 |  |  |  |  |  |  |  |  |
| GO:0034220\_ion\_transmembrane\_transport | 3 | 0 |  |  |  |  |  |  |  |  |
| GO:0034308\_monohydric\_alcohol\_metabolic\_process | 3 | 0 |  |  |  |  |  |  |  |  |
| GO:0034313\_diol\_catabolic\_process | 3 | 0 |  |  |  |  |  |  |  |  |
| GO:0034331\_cell\_junction\_maintenance | 3 | 0 |  |  |  |  |  |  |  |  |
| GO:0034332\_adherens\_junction\_organization | 3 | 0 |  |  |  |  |  |  |  |  |
| GO:0034375\_high-density\_lipoprotein\_particle\_remodeling | 3 | 0 |  |  |  |  |  |  |  |  |
| GO:0034381\_lipoprotein\_particle\_clearance | 3 | 0 |  |  |  |  |  |  |  |  |
| GO:0034612\_response\_to\_tumor\_necrosis\_factor | 3 | 0 |  |  |  |  |  |  |  |  |
| GO:0034655\_nucleobase\_\_nucleoside\_\_nucleotide\_and\_nucleic\_acid\_catabolic\_process | 3 | 0 |  |  |  |  |  |  |  |  |
| GO:0034656\_nucleobase\_\_nucleoside\_and\_nucleotide\_catabolic\_process | 3 | 0 |  |  |  |  |  |  |  |  |
| GO:0035067\_negative\_regulation\_of\_histone\_acetylation | 3 | 0 |  |  |  |  |  |  |  |  |
| GO:0035084\_flagellar\_axoneme\_assembly | 3 | 0 |  |  |  |  |  |  |  |  |
| GO:0035166\_post-embryonic\_hemopoiesis | 3 | 0 |  |  |  |  |  |  |  |  |
| GO:0035283\_central\_nervous\_system\_segmentation | 3 | 0 |  |  |  |  |  |  |  |  |
| GO:0035284\_brain\_segmentation | 3 | 0 |  |  |  |  |  |  |  |  |
| GO:0042097\_interleukin-4\_biosynthetic\_process | 3 | 0 |  |  |  |  |  |  |  |  |
| GO:0042135\_neurotransmitter\_catabolic\_process | 3 | 0 |  |  |  |  |  |  |  |  |
| GO:0042271\_susceptibility\_to\_natural\_killer\_cell\_mediated\_cytotoxicity | 3 | 0 |  |  |  |  |  |  |  |  |
| GO:0042273\_ribosomal\_large\_subunit\_biogenesis | 3 | 0 |  |  |  |  |  |  |  |  |
| GO:0042375\_quinone\_cofactor\_metabolic\_process | 3 | 0 |  |  |  |  |  |  |  |  |
| GO:0042420\_dopamine\_catabolic\_process | 3 | 0 |  |  |  |  |  |  |  |  |
| GO:0042421\_norepinephrine\_biosynthetic\_process | 3 | 0 |  |  |  |  |  |  |  |  |
| GO:0042424\_catecholamine\_catabolic\_process | 3 | 0 |  |  |  |  |  |  |  |  |
| GO:0042447\_hormone\_catabolic\_process | 3 | 0 |  |  |  |  |  |  |  |  |
| GO:0042448\_progesterone\_metabolic\_process | 3 | 0 |  |  |  |  |  |  |  |  |
| GO:0042523\_positive\_regulation\_of\_tyrosine\_phosphorylation\_of\_Stat5\_protein | 3 | 0 |  |  |  |  |  |  |  |  |
| GO:0042659\_regulation\_of\_cell\_fate\_specification | 3 | 0 |  |  |  |  |  |  |  |  |
| GO:0042668\_auditory\_receptor\_cell\_fate\_determination | 3 | 0 |  |  |  |  |  |  |  |  |
| GO:0042670\_retinal\_cone\_cell\_differentiation | 3 | 0 |  |  |  |  |  |  |  |  |
| GO:0042693\_muscle\_cell\_fate\_commitment | 3 | 0 |  |  |  |  |  |  |  |  |
| GO:0042711\_maternal\_behavior | 3 | 0 |  |  |  |  |  |  |  |  |
| GO:0042745\_circadian\_sleep\_wake\_cycle | 3 | 0 |  |  |  |  |  |  |  |  |
| GO:0042759\_long-chain\_fatty\_acid\_biosynthetic\_process | 3 | 0 |  |  |  |  |  |  |  |  |
| GO:0042787\_protein\_ubiquitination\_during\_ubiquitin-dependent\_protein\_catabolic\_process | 3 | 0 |  |  |  |  |  |  |  |  |
| GO:0043045\_DNA\_methylation\_during\_embryonic\_development | 3 | 0 |  |  |  |  |  |  |  |  |
| GO:0043090\_amino\_acid\_import | 3 | 0 |  |  |  |  |  |  |  |  |
| GO:0043092\_L-amino\_acid\_import | 3 | 0 |  |  |  |  |  |  |  |  |
| GO:0043094\_cellular\_metabolic\_compound\_salvage | 3 | 0 |  |  |  |  |  |  |  |  |
| GO:0043101\_purine\_salvage | 3 | 0 |  |  |  |  |  |  |  |  |
| GO:0043149\_stress\_fiber\_formation | 3 | 0 |  |  |  |  |  |  |  |  |
| GO:0043174\_nucleoside\_salvage | 3 | 0 |  |  |  |  |  |  |  |  |
| GO:0043200\_response\_to\_amino\_acid\_stimulus | 3 | 0 |  |  |  |  |  |  |  |  |
| GO:0043267\_negative\_regulation\_of\_potassium\_ion\_transport | 3 | 0 |  |  |  |  |  |  |  |  |
| GO:0043371\_negative\_regulation\_of\_CD4-positive\_\_alpha\_beta\_T\_cell\_differentiation | 3 | 0 |  |  |  |  |  |  |  |  |
| GO:0043462\_regulation\_of\_ATPase\_activity | 3 | 0 |  |  |  |  |  |  |  |  |
| GO:0043569\_negative\_regulation\_of\_insulin-like\_growth\_factor\_receptor\_signaling\_pathway | 3 | 0 |  |  |  |  |  |  |  |  |
| GO:0043574\_peroxisomal\_transport | 3 | 0 |  |  |  |  |  |  |  |  |
| GO:0043586\_tongue\_development | 3 | 0 |  |  |  |  |  |  |  |  |
| GO:0043900\_regulation\_of\_multi-organism\_process | 3 | 0 |  |  |  |  |  |  |  |  |
| GO:0043954\_cellular\_component\_maintenance | 3 | 0 |  |  |  |  |  |  |  |  |
| GO:0044030\_regulation\_of\_DNA\_methylation | 3 | 0 |  |  |  |  |  |  |  |  |
| GO:0044089\_positive\_regulation\_of\_cellular\_component\_biogenesis | 3 | 0 |  |  |  |  |  |  |  |  |
| GO:0044273\_sulfur\_compound\_catabolic\_process | 3 | 0 |  |  |  |  |  |  |  |  |
| GO:0045047\_protein\_targeting\_to\_ER | 3 | 0 |  |  |  |  |  |  |  |  |
| GO:0045085\_negative\_regulation\_of\_interleukin-2\_biosynthetic\_process | 3 | 0 |  |  |  |  |  |  |  |  |
| GO:0045110\_intermediate\_filament\_bundle\_assembly | 3 | 0 |  |  |  |  |  |  |  |  |
| GO:0045143\_homologous\_chromosome\_segregation | 3 | 0 |  |  |  |  |  |  |  |  |
| GO:0045198\_establishment\_of\_epithelial\_cell\_apical\_basal\_polarity | 3 | 0 |  |  |  |  |  |  |  |  |
| GO:0045217\_cell-cell\_junction\_maintenance | 3 | 0 |  |  |  |  |  |  |  |  |
| GO:0045348\_positive\_regulation\_of\_MHC\_class\_II\_biosynthetic\_process | 3 | 0 |  |  |  |  |  |  |  |  |
| GO:0045402\_regulation\_of\_interleukin-4\_biosynthetic\_process | 3 | 0 |  |  |  |  |  |  |  |  |
| GO:0045404\_positive\_regulation\_of\_interleukin-4\_biosynthetic\_process | 3 | 0 |  |  |  |  |  |  |  |  |
| GO:0045542\_positive\_regulation\_of\_cholesterol\_biosynthetic\_process | 3 | 0 |  |  |  |  |  |  |  |  |
| GO:0045623\_negative\_regulation\_of\_T-helper\_cell\_differentiation | 3 | 0 |  |  |  |  |  |  |  |  |
| GO:0045625\_regulation\_of\_T-helper\_1\_cell\_differentiation | 3 | 0 |  |  |  |  |  |  |  |  |
| GO:0045717\_negative\_regulation\_of\_fatty\_acid\_biosynthetic\_process | 3 | 0 |  |  |  |  |  |  |  |  |
| GO:0045723\_positive\_regulation\_of\_fatty\_acid\_biosynthetic\_process | 3 | 0 |  |  |  |  |  |  |  |  |
| GO:0045746\_negative\_regulation\_of\_Notch\_signaling\_pathway | 3 | 0 |  |  |  |  |  |  |  |  |
| GO:0045806\_negative\_regulation\_of\_endocytosis | 3 | 0 |  |  |  |  |  |  |  |  |
| GO:0045829\_negative\_regulation\_of\_isotype\_switching | 3 | 0 |  |  |  |  |  |  |  |  |
| GO:0045844\_positive\_regulation\_of\_striated\_muscle\_development | 3 | 0 |  |  |  |  |  |  |  |  |
| GO:0045907\_positive\_regulation\_of\_vasoconstriction | 3 | 0 |  |  |  |  |  |  |  |  |
| GO:0045922\_negative\_regulation\_of\_fatty\_acid\_metabolic\_process | 3 | 0 |  |  |  |  |  |  |  |  |
| GO:0045939\_negative\_regulation\_of\_steroid\_metabolic\_process | 3 | 0 |  |  |  |  |  |  |  |  |
| GO:0046013\_regulation\_of\_T\_cell\_homeostatic\_proliferation | 3 | 0 |  |  |  |  |  |  |  |  |
| GO:0046034\_ATP\_metabolic\_process | 3 | 0 |  |  |  |  |  |  |  |  |
| GO:0046325\_negative\_regulation\_of\_glucose\_import | 3 | 0 |  |  |  |  |  |  |  |  |
| GO:0046426\_negative\_regulation\_of\_JAK-STAT\_cascade | 3 | 0 |  |  |  |  |  |  |  |  |
| GO:0046457\_prostanoid\_biosynthetic\_process | 3 | 0 |  |  |  |  |  |  |  |  |
| GO:0046479\_glycosphingolipid\_catabolic\_process | 3 | 0 |  |  |  |  |  |  |  |  |
| GO:0046488\_phosphatidylinositol\_metabolic\_process | 3 | 0 |  |  |  |  |  |  |  |  |
| GO:0046549\_retinal\_cone\_cell\_development | 3 | 0 |  |  |  |  |  |  |  |  |
| GO:0046605\_regulation\_of\_centrosome\_cycle | 3 | 0 |  |  |  |  |  |  |  |  |
| GO:0046688\_response\_to\_copper\_ion | 3 | 0 |  |  |  |  |  |  |  |  |
| GO:0046717\_acid\_secretion | 3 | 0 |  |  |  |  |  |  |  |  |
| GO:0046825\_regulation\_of\_protein\_export\_from\_nucleus | 3 | 0 |  |  |  |  |  |  |  |  |
| GO:0046885\_regulation\_of\_hormone\_biosynthetic\_process | 3 | 0 |  |  |  |  |  |  |  |  |
| GO:0048003\_antigen\_processing\_and\_presentation\_of\_lipid\_antigen\_via\_MHC\_class\_Ib | 3 | 0 |  |  |  |  |  |  |  |  |
| GO:0048007\_antigen\_processing\_and\_presentation\_\_exogenous\_lipid\_antigen\_via\_MHC\_class\_Ib | 3 | 0 |  |  |  |  |  |  |  |  |
| GO:0048012\_hepatocyte\_growth\_factor\_receptor\_signaling\_pathway | 3 | 0 |  |  |  |  |  |  |  |  |
| GO:0048050\_post-embryonic\_eye\_morphogenesis | 3 | 0 |  |  |  |  |  |  |  |  |
| GO:0048087\_positive\_regulation\_of\_pigmentation\_during\_development | 3 | 0 |  |  |  |  |  |  |  |  |
| GO:0048246\_macrophage\_chemotaxis | 3 | 0 |  |  |  |  |  |  |  |  |
| GO:0048251\_elastic\_fiber\_assembly | 3 | 0 |  |  |  |  |  |  |  |  |
| GO:0048294\_negative\_regulation\_of\_isotype\_switching\_to\_IgE\_isotypes | 3 | 0 |  |  |  |  |  |  |  |  |
| GO:0048318\_axial\_mesoderm\_development | 3 | 0 |  |  |  |  |  |  |  |  |
| GO:0048597\_post-embryonic\_camera-type\_eye\_morphogenesis | 3 | 0 |  |  |  |  |  |  |  |  |
| GO:0048636\_positive\_regulation\_of\_muscle\_development | 3 | 0 |  |  |  |  |  |  |  |  |
| GO:0048660\_regulation\_of\_smooth\_muscle\_cell\_proliferation | 3 | 0 |  |  |  |  |  |  |  |  |
| GO:0048668\_collateral\_sprouting | 3 | 0 |  |  |  |  |  |  |  |  |
| GO:0048676\_axon\_extension\_involved\_in\_development | 3 | 0 |  |  |  |  |  |  |  |  |
| GO:0048755\_branching\_morphogenesis\_of\_a\_nerve | 3 | 0 |  |  |  |  |  |  |  |  |
| GO:0048859\_formation\_of\_anatomical\_boundary | 3 | 0 |  |  |  |  |  |  |  |  |
| GO:0048865\_stem\_cell\_fate\_commitment | 3 | 0 |  |  |  |  |  |  |  |  |
| GO:0050435\_beta-amyloid\_metabolic\_process | 3 | 0 |  |  |  |  |  |  |  |  |
| GO:0050650\_chondroitin\_sulfate\_proteoglycan\_biosynthetic\_process | 3 | 0 |  |  |  |  |  |  |  |  |
| GO:0050703\_interleukin-1\_alpha\_secretion | 3 | 0 |  |  |  |  |  |  |  |  |
| GO:0050705\_regulation\_of\_interleukin-1\_alpha\_secretion | 3 | 0 |  |  |  |  |  |  |  |  |
| GO:0050709\_negative\_regulation\_of\_protein\_secretion | 3 | 0 |  |  |  |  |  |  |  |  |
| GO:0050710\_negative\_regulation\_of\_cytokine\_secretion | 3 | 0 |  |  |  |  |  |  |  |  |
| GO:0050717\_positive\_regulation\_of\_interleukin-1\_alpha\_secretion | 3 | 0 |  |  |  |  |  |  |  |  |
| GO:0050774\_negative\_regulation\_of\_dendrite\_morphogenesis | 3 | 0 |  |  |  |  |  |  |  |  |
| GO:0050857\_positive\_regulation\_of\_antigen\_receptor-mediated\_signaling\_pathway | 3 | 0 |  |  |  |  |  |  |  |  |
| GO:0050882\_voluntary\_musculoskeletal\_movement | 3 | 0 |  |  |  |  |  |  |  |  |
| GO:0050913\_sensory\_perception\_of\_bitter\_taste | 3 | 0 |  |  |  |  |  |  |  |  |
| GO:0050957\_equilibrioception | 3 | 0 |  |  |  |  |  |  |  |  |
| GO:0050996\_positive\_regulation\_of\_lipid\_catabolic\_process | 3 | 0 |  |  |  |  |  |  |  |  |
| GO:0051149\_positive\_regulation\_of\_muscle\_cell\_differentiation | 3 | 0 |  |  |  |  |  |  |  |  |
| GO:0051204\_protein\_insertion\_into\_mitochondrial\_membrane | 3 | 0 |  |  |  |  |  |  |  |  |
| GO:0051291\_protein\_heterooligomerization | 3 | 0 |  |  |  |  |  |  |  |  |
| GO:0051302\_regulation\_of\_cell\_division | 3 | 0 |  |  |  |  |  |  |  |  |
| GO:0051320\_S\_phase | 3 | 0 |  |  |  |  |  |  |  |  |
| GO:0051450\_myoblast\_proliferation | 3 | 0 |  |  |  |  |  |  |  |  |
| GO:0051583\_dopamine\_uptake | 3 | 0 |  |  |  |  |  |  |  |  |
| GO:0051798\_positive\_regulation\_of\_hair\_follicle\_development | 3 | 0 |  |  |  |  |  |  |  |  |
| GO:0051882\_mitochondrial\_depolarization | 3 | 0 |  |  |  |  |  |  |  |  |
| GO:0051900\_regulation\_of\_mitochondrial\_depolarization | 3 | 0 |  |  |  |  |  |  |  |  |
| GO:0051925\_regulation\_of\_calcium\_ion\_transport\_via\_voltage-gated\_calcium\_channel\_activity | 3 | 0 |  |  |  |  |  |  |  |  |
| GO:0051926\_negative\_regulation\_of\_calcium\_ion\_transport | 3 | 0 |  |  |  |  |  |  |  |  |
| GO:0051930\_regulation\_of\_sensory\_perception\_of\_pain | 3 | 0 |  |  |  |  |  |  |  |  |
| GO:0051931\_regulation\_of\_sensory\_perception | 3 | 0 |  |  |  |  |  |  |  |  |
| GO:0051934\_catecholamine\_uptake\_during\_transmission\_of\_nerve\_impulse | 3 | 0 |  |  |  |  |  |  |  |  |
| GO:0051955\_regulation\_of\_amino\_acid\_transport | 3 | 0 |  |  |  |  |  |  |  |  |
| GO:0051962\_positive\_regulation\_of\_nervous\_system\_development | 3 | 0 |  |  |  |  |  |  |  |  |
| GO:0051965\_positive\_regulation\_of\_synaptogenesis | 3 | 0 |  |  |  |  |  |  |  |  |
| GO:0051967\_negative\_regulation\_of\_synaptic\_transmission\_\_glutamatergic | 3 | 0 |  |  |  |  |  |  |  |  |
| GO:0051983\_regulation\_of\_chromosome\_segregation | 3 | 0 |  |  |  |  |  |  |  |  |
| GO:0055061\_di-\_\_tri-valent\_inorganic\_anion\_homeostasis | 3 | 0 |  |  |  |  |  |  |  |  |
| GO:0055062\_phosphate\_ion\_homeostasis | 3 | 0 |  |  |  |  |  |  |  |  |
| GO:0055083\_monovalent\_inorganic\_anion\_homeostasis | 3 | 0 |  |  |  |  |  |  |  |  |
| GO:0055117\_regulation\_of\_cardiac\_muscle\_contraction | 3 | 0 |  |  |  |  |  |  |  |  |
| GO:0060009\_Sertoli\_cell\_development | 3 | 0 |  |  |  |  |  |  |  |  |
| GO:0060024\_rhythmic\_synaptic\_transmission | 3 | 0 |  |  |  |  |  |  |  |  |
| GO:0060033\_anatomical\_structure\_regression | 3 | 0 |  |  |  |  |  |  |  |  |
| GO:0060040\_retinal\_bipolar\_neuron\_differentiation | 3 | 0 |  |  |  |  |  |  |  |  |
| GO:0060055\_angiogenesis\_involved\_in\_wound\_healing | 3 | 0 |  |  |  |  |  |  |  |  |
| GO:0060084\_synaptic\_transmission\_involved\_in\_micturition | 3 | 0 |  |  |  |  |  |  |  |  |
| GO:0060123\_regulation\_of\_growth\_hormone\_secretion | 3 | 0 |  |  |  |  |  |  |  |  |
| GO:0060126\_somatotropin\_secreting\_cell\_differentiation | 3 | 0 |  |  |  |  |  |  |  |  |
| GO:0060192\_negative\_regulation\_of\_lipase\_activity | 3 | 0 |  |  |  |  |  |  |  |  |
| GO:0060219\_camera-type\_eye\_photoreceptor\_cell\_differentiation | 3 | 0 |  |  |  |  |  |  |  |  |
| GO:0060285\_ciliary\_cell\_motility | 3 | 0 |  |  |  |  |  |  |  |  |
| GO:0060294\_cilium\_movement\_involved\_in\_ciliary\_motility | 3 | 0 |  |  |  |  |  |  |  |  |
| GO:0060295\_regulation\_of\_cilium\_movement\_involved\_in\_ciliary\_motility | 3 | 0 |  |  |  |  |  |  |  |  |
| GO:0060296\_regulation\_of\_cilium\_beat\_frequency\_involved\_in\_ciliary\_motility | 3 | 0 |  |  |  |  |  |  |  |  |
| GO:0060314\_regulation\_of\_ryanodine-sensitive\_calcium-release\_channel\_activity | 3 | 0 |  |  |  |  |  |  |  |  |
| GO:0060396\_growth\_hormone\_receptor\_signaling\_pathway | 3 | 0 |  |  |  |  |  |  |  |  |
| GO:0060416\_response\_to\_growth\_hormone\_stimulus | 3 | 0 |  |  |  |  |  |  |  |  |
| GO:0060428\_lung\_epithelium\_development | 3 | 0 |  |  |  |  |  |  |  |  |
| GO:0060433\_bronchus\_development | 3 | 0 |  |  |  |  |  |  |  |  |
| GO:0060435\_bronchiole\_development | 3 | 0 |  |  |  |  |  |  |  |  |
| GO:0060460\_left\_lung\_morphogenesis | 3 | 0 |  |  |  |  |  |  |  |  |
| GO:0060491\_regulation\_of\_cell\_projection\_assembly | 3 | 0 |  |  |  |  |  |  |  |  |
| GO:0060523\_prostate\_epithelial\_cord\_elongation | 3 | 0 |  |  |  |  |  |  |  |  |
| GO:0060586\_multicellular\_organismal\_iron\_ion\_homeostasis | 3 | 0 |  |  |  |  |  |  |  |  |
| GO:0060596\_mammary\_placode\_formation | 3 | 0 |  |  |  |  |  |  |  |  |
| GO:0060632\_regulation\_of\_microtubule-based\_movement | 3 | 0 |  |  |  |  |  |  |  |  |
| GO:0060648\_mammary\_gland\_bud\_morphogenesis | 3 | 0 |  |  |  |  |  |  |  |  |
| GO:0060684\_epithelial-mesenchymal\_cell\_signaling | 3 | 0 |  |  |  |  |  |  |  |  |
| GO:0060689\_cell\_differentiation\_involved\_in\_salivary\_gland\_development | 3 | 0 |  |  |  |  |  |  |  |  |
| GO:0060708\_spongiotrophoblast\_differentiation | 3 | 0 |  |  |  |  |  |  |  |  |
| GO:0060746\_parental\_behavior | 3 | 0 |  |  |  |  |  |  |  |  |
| GO:0060748\_tertiary\_branching\_involved\_in\_mammary\_gland\_duct\_morphogenesis | 3 | 0 |  |  |  |  |  |  |  |  |
| GO:0070102\_interleukin-6-mediated\_signaling\_pathway | 3 | 0 |  |  |  |  |  |  |  |  |
| GO:0070169\_positive\_regulation\_of\_biomineral\_formation | 3 | 0 |  |  |  |  |  |  |  |  |
| GO:0070206\_protein\_trimerization | 3 | 0 |  |  |  |  |  |  |  |  |
| GO:0070207\_protein\_homotrimerization | 3 | 0 |  |  |  |  |  |  |  |  |
| GO:0070229\_negative\_regulation\_of\_lymphocyte\_apoptosis | 3 | 0 |  |  |  |  |  |  |  |  |
| GO:0070230\_positive\_regulation\_of\_lymphocyte\_apoptosis | 3 | 0 |  |  |  |  |  |  |  |  |
| GO:0070232\_regulation\_of\_T\_cell\_apoptosis | 3 | 0 |  |  |  |  |  |  |  |  |
| GO:0070233\_negative\_regulation\_of\_T\_cell\_apoptosis | 3 | 0 |  |  |  |  |  |  |  |  |
| GO:0070242\_thymocyte\_apoptosis | 3 | 0 |  |  |  |  |  |  |  |  |
| GO:0070243\_regulation\_of\_thymocyte\_apoptosis | 3 | 0 |  |  |  |  |  |  |  |  |
| GO:0070244\_negative\_regulation\_of\_thymocyte\_apoptosis | 3 | 0 |  |  |  |  |  |  |  |  |
| GO:0070423\_nucleotide-binding\_oligomerization\_domain\_containing\_signaling\_pathway | 3 | 0 |  |  |  |  |  |  |  |  |
| GO:0070427\_nucleotide-binding\_oligomerization\_domain\_containing\_1\_signaling\_pathway | 3 | 0 |  |  |  |  |  |  |  |  |
| GO:0070431\_nucleotide-binding\_oligomerization\_domain\_containing\_2\_signaling\_pathway | 3 | 0 |  |  |  |  |  |  |  |  |
| GO:0070633\_transepithelial\_transport | 3 | 0 |  |  |  |  |  |  |  |  |
| GO:0070846\_Hsp90\_deacetylation | 3 | 0 |  |  |  |  |  |  |  |  |
| GO:0070873\_regulation\_of\_glycogen\_metabolic\_process | 3 | 0 |  |  |  |  |  |  |  |  |
| GO:0070875\_positive\_regulation\_of\_glycogen\_metabolic\_process | 3 | 0 |  |  |  |  |  |  |  |  |
| GO:0001666\_response\_to\_hypoxia | 26 | 0 | 0.000000 | -0.000000 | 2691 | 2642.594794 | 2663.37 | 2684.145206 | 0.989732 |  |
| GO:0006800\_oxygen\_and\_reactive\_oxygen\_species\_metabolic\_process | 26 | 0 | 0.000000 | -0.000000 | 2691 | 2642.594794 | 2663.37 | 2684.145206 | 0.989732 |  |
| GO:0007405\_neuroblast\_proliferation | 26 | 0 | 0.000000 | -0.000000 | 2691 | 2642.594794 | 2663.37 | 2684.145206 | 0.989732 |  |
| GO:0007613\_memory | 26 | 0 | 0.000000 | -0.000000 | 2691 | 2642.594794 | 2663.37 | 2684.145206 | 0.989732 |  |
| GO:0007623\_circadian\_rhythm | 26 | 0 | 0.000000 | -0.000000 | 2691 | 2642.594794 | 2663.37 | 2684.145206 | 0.989732 |  |
| GO:0009310\_amine\_catabolic\_process | 26 | 0 | 0.000000 | -0.000000 | 2691 | 2642.594794 | 2663.37 | 2684.145206 | 0.989732 |  |
| GO:0009636\_response\_to\_toxin | 26 | 0 | 0.000000 | -0.000000 | 2691 | 2642.594794 | 2663.37 | 2684.145206 | 0.989732 |  |
| GO:0010959\_regulation\_of\_metal\_ion\_transport | 26 | 0 | 0.000000 | -0.000000 | 2691 | 2642.594794 | 2663.37 | 2684.145206 | 0.989732 |  |
| GO:0019233\_sensory\_perception\_of\_pain | 26 | 0 | 0.000000 | -0.000000 | 2691 | 2642.594794 | 2663.37 | 2684.145206 | 0.989732 |  |
| GO:0032609\_interferon-gamma\_production | 26 | 0 | 0.000000 | -0.000000 | 2691 | 2642.594794 | 2663.37 | 2684.145206 | 0.989732 |  |
| GO:0040029\_regulation\_of\_gene\_expression\_\_epigenetic | 26 | 0 | 0.000000 | -0.000000 | 2691 | 2642.594794 | 2663.37 | 2684.145206 | 0.989732 |  |
| GO:0045765\_regulation\_of\_angiogenesis | 26 | 0 | 0.000000 | -0.000000 | 2691 | 2642.594794 | 2663.37 | 2684.145206 | 0.989732 |  |
| GO:0046530\_photoreceptor\_cell\_differentiation | 26 | 0 | 0.000000 | -0.000000 | 2691 | 2642.594794 | 2663.37 | 2684.145206 | 0.989732 |  |
| GO:0050873\_brown\_fat\_cell\_differentiation | 26 | 0 | 0.000000 | -0.000000 | 2691 | 2642.594794 | 2663.37 | 2684.145206 | 0.989732 |  |
| GO:0051480\_cytosolic\_calcium\_ion\_homeostasis | 26 | 0 | 0.000000 | -0.000000 | 2691 | 2642.594794 | 2663.37 | 2684.145206 | 0.989732 |  |
| GO:0000041\_transition\_metal\_ion\_transport | 22 | 0 | 0.000000 | -0.000000 | 2718 | 2680.541407 | 2699.33 | 2718.118593 | 0.993131 |  |
| GO:0001558\_regulation\_of\_cell\_growth | 22 | 0 | 0.000000 | -0.000000 | 2718 | 2680.541407 | 2699.33 | 2718.118593 | 0.993131 |  |
| GO:0001947\_heart\_looping | 22 | 0 | 0.000000 | -0.000000 | 2718 | 2680.541407 | 2699.33 | 2718.118593 | 0.993131 |  |
| GO:0002478\_antigen\_processing\_and\_presentation\_of\_exogenous\_peptide\_antigen | 22 | 0 | 0.000000 | -0.000000 | 2718 | 2680.541407 | 2699.33 | 2718.118593 | 0.993131 |  |
| GO:0002712\_regulation\_of\_B\_cell\_mediated\_immunity | 22 | 0 | 0.000000 | -0.000000 | 2718 | 2680.541407 | 2699.33 | 2718.118593 | 0.993131 |  |
| GO:0002889\_regulation\_of\_immunoglobulin\_mediated\_immune\_response | 22 | 0 | 0.000000 | -0.000000 | 2718 | 2680.541407 | 2699.33 | 2718.118593 | 0.993131 |  |
| GO:0006112\_energy\_reserve\_metabolic\_process | 22 | 0 | 0.000000 | -0.000000 | 2718 | 2680.541407 | 2699.33 | 2718.118593 | 0.993131 |  |
| GO:0007059\_chromosome\_segregation | 22 | 0 | 0.000000 | -0.000000 | 2718 | 2680.541407 | 2699.33 | 2718.118593 | 0.993131 |  |
| GO:0008624\_induction\_of\_apoptosis\_by\_extracellular\_signals | 22 | 0 | 0.000000 | -0.000000 | 2718 | 2680.541407 | 2699.33 | 2718.118593 | 0.993131 |  |
| GO:0009896\_positive\_regulation\_of\_catabolic\_process | 22 | 0 | 0.000000 | -0.000000 | 2718 | 2680.541407 | 2699.33 | 2718.118593 | 0.993131 |  |
| GO:0021675\_nerve\_development | 22 | 0 | 0.000000 | -0.000000 | 2718 | 2680.541407 | 2699.33 | 2718.118593 | 0.993131 |  |
| GO:0030335\_positive\_regulation\_of\_cell\_migration | 22 | 0 | 0.000000 | -0.000000 | 2718 | 2680.541407 | 2699.33 | 2718.118593 | 0.993131 |  |
| GO:0030705\_cytoskeleton-dependent\_intracellular\_transport | 22 | 0 | 0.000000 | -0.000000 | 2718 | 2680.541407 | 2699.33 | 2718.118593 | 0.993131 |  |
| GO:0032649\_regulation\_of\_interferon-gamma\_production | 22 | 0 | 0.000000 | -0.000000 | 2718 | 2680.541407 | 2699.33 | 2718.118593 | 0.993131 |  |
| GO:0032886\_regulation\_of\_microtubule-based\_process | 22 | 0 | 0.000000 | -0.000000 | 2718 | 2680.541407 | 2699.33 | 2718.118593 | 0.993131 |  |
| GO:0034097\_response\_to\_cytokine\_stimulus | 22 | 0 | 0.000000 | -0.000000 | 2718 | 2680.541407 | 2699.33 | 2718.118593 | 0.993131 |  |
| GO:0034660\_ncRNA\_metabolic\_process | 22 | 0 | 0.000000 | -0.000000 | 2718 | 2680.541407 | 2699.33 | 2718.118593 | 0.993131 |  |
| GO:0040018\_positive\_regulation\_of\_multicellular\_organism\_growth | 22 | 0 | 0.000000 | -0.000000 | 2718 | 2680.541407 | 2699.33 | 2718.118593 | 0.993131 |  |
| GO:0042130\_negative\_regulation\_of\_T\_cell\_proliferation | 22 | 0 | 0.000000 | -0.000000 | 2718 | 2680.541407 | 2699.33 | 2718.118593 | 0.993131 |  |
| GO:0042461\_photoreceptor\_cell\_development | 22 | 0 | 0.000000 | -0.000000 | 2718 | 2680.541407 | 2699.33 | 2718.118593 | 0.993131 |  |
| GO:0043112\_receptor\_metabolic\_process | 22 | 0 | 0.000000 | -0.000000 | 2718 | 2680.541407 | 2699.33 | 2718.118593 | 0.993131 |  |
| GO:0044264\_cellular\_polysaccharide\_metabolic\_process | 22 | 0 | 0.000000 | -0.000000 | 2718 | 2680.541407 | 2699.33 | 2718.118593 | 0.993131 |  |
| GO:0046635\_positive\_regulation\_of\_alpha-beta\_T\_cell\_activation | 22 | 0 | 0.000000 | -0.000000 | 2718 | 2680.541407 | 2699.33 | 2718.118593 | 0.993131 |  |
| GO:0046883\_regulation\_of\_hormone\_secretion | 22 | 0 | 0.000000 | -0.000000 | 2718 | 2680.541407 | 2699.33 | 2718.118593 | 0.993131 |  |
| GO:0048477\_oogenesis | 22 | 0 | 0.000000 | -0.000000 | 2718 | 2680.541407 | 2699.33 | 2718.118593 | 0.993131 |  |
| GO:0048864\_stem\_cell\_development | 22 | 0 | 0.000000 | -0.000000 | 2718 | 2680.541407 | 2699.33 | 2718.118593 | 0.993131 |  |
| GO:0051260\_protein\_homooligomerization | 22 | 0 | 0.000000 | -0.000000 | 2718 | 2680.541407 | 2699.33 | 2718.118593 | 0.993131 |  |
| GO:0000279\_M\_phase | 85 | 0 | 0.000000 | 0.000000 | 2749 | 2746.878917 | 2748.64 | 2750.401083 | 0.999869 |  |
| GO:0001816\_cytokine\_production | 122 | 0 | 0.000000 | 0.000000 | 2749 | 2746.878917 | 2748.64 | 2750.401083 | 0.999869 |  |
| GO:0001817\_regulation\_of\_cytokine\_production | 99 | 0 | 0.000000 | 0.000000 | 2749 | 2746.878917 | 2748.64 | 2750.401083 | 0.999869 |  |
| GO:0002684\_positive\_regulation\_of\_immune\_system\_process | 148 | 0 | 0.000000 | 0.000000 | 2749 | 2746.878917 | 2748.64 | 2750.401083 | 0.999869 |  |
| GO:0003001\_generation\_of\_a\_signal\_involved\_in\_cell-cell\_signaling | 87 | 0 | 0.000000 | 0.000000 | 2749 | 2746.878917 | 2748.64 | 2750.401083 | 0.999869 |  |
| GO:0006605\_protein\_targeting | 86 | 0 | 0.000000 | 0.000000 | 2749 | 2746.878917 | 2748.64 | 2750.401083 | 0.999869 |  |
| GO:0006886\_intracellular\_protein\_transport | 122 | 0 | 0.000000 | 0.000000 | 2749 | 2746.878917 | 2748.64 | 2750.401083 | 0.999869 |  |
| GO:0006897\_endocytosis | 86 | 0 | 0.000000 | 0.000000 | 2749 | 2746.878917 | 2748.64 | 2750.401083 | 0.999869 |  |
| GO:0008150\_biological\_process | 4605 | 53 | 1.000000 | 0.000000 | 2749 | 2746.878917 | 2748.64 | 2750.401083 | 0.999869 |  |
| GO:0009607\_response\_to\_biotic\_stimulus | 114 | 0 | 0.000000 | 0.000000 | 2749 | 2746.878917 | 2748.64 | 2750.401083 | 0.999869 |  |
| GO:0009968\_negative\_regulation\_of\_signal\_transduction | 103 | 0 | 0.000000 | 0.000000 | 2749 | 2746.878917 | 2748.64 | 2750.401083 | 0.999869 |  |
| GO:0010324\_membrane\_invagination | 86 | 0 | 0.000000 | 0.000000 | 2749 | 2746.878917 | 2748.64 | 2750.401083 | 0.999869 |  |
| GO:0010648\_negative\_regulation\_of\_cell\_communication | 110 | 0 | 0.000000 | 0.000000 | 2749 | 2746.878917 | 2748.64 | 2750.401083 | 0.999869 |  |
| GO:0022402\_cell\_cycle\_process | 155 | 0 | 0.000000 | 0.000000 | 2749 | 2746.878917 | 2748.64 | 2750.401083 | 0.999869 |  |
| GO:0022403\_cell\_cycle\_phase | 119 | 0 | 0.000000 | 0.000000 | 2749 | 2746.878917 | 2748.64 | 2750.401083 | 0.999869 |  |
| GO:0030001\_metal\_ion\_transport | 122 | 0 | 0.000000 | 0.000000 | 2749 | 2746.878917 | 2748.64 | 2750.401083 | 0.999869 |  |
| GO:0030163\_protein\_catabolic\_process | 101 | 0 | 0.000000 | 0.000000 | 2749 | 2746.878917 | 2748.64 | 2750.401083 | 0.999869 |  |
| GO:0032943\_mononuclear\_cell\_proliferation | 94 | 0 | 0.000000 | 0.000000 | 2749 | 2746.878917 | 2748.64 | 2750.401083 | 0.999869 |  |
| GO:0042391\_regulation\_of\_membrane\_potential | 95 | 0 | 0.000000 | 0.000000 | 2749 | 2746.878917 | 2748.64 | 2750.401083 | 0.999869 |  |
| GO:0043285\_biopolymer\_catabolic\_process | 129 | 0 | 0.000000 | 0.000000 | 2749 | 2746.878917 | 2748.64 | 2750.401083 | 0.999869 |  |
| GO:0046651\_lymphocyte\_proliferation | 94 | 0 | 0.000000 | 0.000000 | 2749 | 2746.878917 | 2748.64 | 2750.401083 | 0.999869 |  |
| GO:0046907\_intracellular\_transport | 194 | 0 | 0.000000 | 0.000000 | 2749 | 2746.878917 | 2748.64 | 2750.401083 | 0.999869 |  |
| GO:0048584\_positive\_regulation\_of\_response\_to\_stimulus | 115 | 0 | 0.000000 | 0.000000 | 2749 | 2746.878917 | 2748.64 | 2750.401083 | 0.999869 |  |
| GO:0050778\_positive\_regulation\_of\_immune\_response | 87 | 0 | 0.000000 | 0.000000 | 2749 | 2746.878917 | 2748.64 | 2750.401083 | 0.999869 |  |
| GO:0050863\_regulation\_of\_T\_cell\_activation | 88 | 0 | 0.000000 | 0.000000 | 2749 | 2746.878917 | 2748.64 | 2750.401083 | 0.999869 |  |
| GO:0051249\_regulation\_of\_lymphocyte\_activation | 112 | 0 | 0.000000 | 0.000000 | 2749 | 2746.878917 | 2748.64 | 2750.401083 | 0.999869 |  |
| GO:0051276\_chromosome\_organization | 129 | 0 | 0.000000 | 0.000000 | 2749 | 2746.878917 | 2748.64 | 2750.401083 | 0.999869 |  |
| GO:0051707\_response\_to\_other\_organism | 95 | 0 | 0.000000 | 0.000000 | 2749 | 2746.878917 | 2748.64 | 2750.401083 | 0.999869 |  |
| GO:0065003\_macromolecular\_complex\_assembly | 93 | 0 | 0.000000 | 0.000000 | 2749 | 2746.878917 | 2748.64 | 2750.401083 | 0.999869 |  |
| GO:0070661\_leukocyte\_proliferation | 96 | 0 | 0.000000 | 0.000000 | 2749 | 2746.878917 | 2748.64 | 2750.401083 | 0.999869 |  |
| GO:0070887\_cellular\_response\_to\_chemical\_stimulus | 85 | 0 | 0.000000 | 0.000000 | 2749 | 2746.878917 | 2748.64 | 2750.401083 | 0.999869 |  |
| GO:0000226\_microtubule\_cytoskeleton\_organization | 57 | 0 | 0.000000 | 0.000000 | 2755 | 2755.000000 | 2755.0 | 2755.000000 | 1.000000 |  |
| GO:0008344\_adult\_locomotory\_behavior | 57 | 0 | 0.000000 | 0.000000 | 2755 | 2755.000000 | 2755.0 | 2755.000000 | 1.000000 |  |
| GO:0018108\_peptidyl-tyrosine\_phosphorylation | 57 | 0 | 0.000000 | 0.000000 | 2755 | 2755.000000 | 2755.0 | 2755.000000 | 1.000000 |  |
| GO:0018212\_peptidyl-tyrosine\_modification | 57 | 0 | 0.000000 | 0.000000 | 2755 | 2755.000000 | 2755.0 | 2755.000000 | 1.000000 |  |
| GO:0033365\_protein\_localization\_in\_organelle | 57 | 0 | 0.000000 | 0.000000 | 2755 | 2755.000000 | 2755.0 | 2755.000000 | 1.000000 |  |
| GO:0045444\_fat\_cell\_differentiation | 57 | 0 | 0.000000 | 0.000000 | 2755 | 2755.000000 | 2755.0 | 2755.000000 | 1.000000 |  |
