# Supplementary material for: RedundancyMiner: De-replication of redundant GO categories in microarray and proteomics analysis
Source: BMC Bioinformatics. 2011 Feb 10;12:52. doi: 10.1186/1471-2105-12-52 (PMC3223614; doi:10.1186/1471-2105-12-52)
Supplement: Additional file 8 — Retinal development HTGM download. compressed package of the results of running HTGM on the retinal development genes list. [file 1471-2105-12-52-S8.ZIP › SCENARIO_2_MODIFIED/total.txt.total.txt.dir/Exp1_BestClusterMap_LEIGS_KM_24.csv.join.9.txt.dir/Exp1_BestClusterMap_LEIGS_KM_24.csv.join.9.txt.change.gce.html]

Gene Category Report for Exp1\_BestClusterMap\_LEIGS\_KM\_24.csv.join.9.txt

# Gene Category Report for Exp1\_BestClusterMap\_LEIGS\_KM\_24.csv.join.9.txt

| HYPERLINKED GO CATEGORY | HYPERLINKED GENE NAME | TOTAL GENES | CHANGED GENES | ENRICHMENT | LOG10(p) | CUMULATIVE NUMBER OF CATEGORIES | CUMULATIVE RANDOMS MEAN | FALSE DISCOVERY RATE |  |
| --- | --- | --- | --- | --- | --- | --- | --- | --- | --- |
| GO:0000280\_nuclear\_division | NEK2 | 24 | 2 | 21.319444 | -2.421871 | 2 | 2.28 | 1.140000 |  |
| GO:0000280\_nuclear\_division | PES1 | 24 | 2 | 21.319444 | -2.421871 | 2 | 2.28 | 1.140000 |  |
| GO:0007067\_mitosis | NEK2 | 24 | 2 | 21.319444 | -2.421871 | 2 | 2.28 | 1.140000 |  |
| GO:0007067\_mitosis | PES1 | 24 | 2 | 21.319444 | -2.421871 | 2 | 2.28 | 1.140000 |  |
| GO:0006613\_cotranslational\_protein\_targeting\_to\_membrane | ARL6IP1 | 1 | 1 |  |  |  |  |  |  |
| GO:0045454\_cell\_redox\_homeostasis | APEX1 | 1 | 1 |  |  |  |  |  |  |
| GO:0000087\_M\_phase\_of\_mitotic\_cell\_cycle | NEK2 | 25 | 2 | 20.466667 | -2.386663 | 4 | 2.49 | 0.622500 |  |
| GO:0000087\_M\_phase\_of\_mitotic\_cell\_cycle | PES1 | 25 | 2 | 20.466667 | -2.386663 | 4 | 2.49 | 0.622500 |  |
| GO:0048285\_organelle\_fission | NEK2 | 25 | 2 | 20.466667 | -2.386663 | 4 | 2.49 | 0.622500 |  |
| GO:0048285\_organelle\_fission | PES1 | 25 | 2 | 20.466667 | -2.386663 | 4 | 2.49 | 0.622500 |  |
| GO:0006398\_histone\_mRNA\_3'-end\_processing | SLBP | 2 | 1 |  |  |  |  |  |  |
| GO:0008334\_histone\_mRNA\_metabolic\_process | SLBP | 2 | 1 |  |  |  |  |  |  |
| GO:0000089\_mitotic\_metaphase | PES1 | 3 | 1 |  |  |  |  |  |  |
| GO:0007000\_nucleolus\_organization | PES1 | 3 | 1 |  |  |  |  |  |  |
| GO:0048246\_macrophage\_chemotaxis | NUP85 | 3 | 1 |  |  |  |  |  |  |
| GO:0000070\_mitotic\_sister\_chromatid\_segregation | NEK2 | 5 | 1 | 51.166667 | -1.712195 | 7 | 14.76 | 2.108571 |  |
| GO:0000819\_sister\_chromatid\_segregation | NEK2 | 5 | 1 | 51.166667 | -1.712195 | 7 | 14.76 | 2.108571 |  |
| GO:0051323\_metaphase | PES1 | 5 | 1 | 51.166667 | -1.712195 | 7 | 14.76 | 2.108571 |  |
| GO:0060710\_chorio-allantoic\_fusion | DNAJB6 | 6 | 1 | 42.638889 | -1.633815 | 8 | 20.25 | 2.531250 |  |
| GO:0006612\_protein\_targeting\_to\_membrane | ARL6IP1 | 7 | 1 | 36.547619 | -1.567669 | 11 | 26.01 | 2.364545 |  |
| GO:0031017\_exocrine\_pancreas\_development | IGF2 | 7 | 1 | 36.547619 | -1.567669 | 11 | 26.01 | 2.364545 |  |
| GO:0031124\_mRNA\_3'-end\_processing | SLBP | 7 | 1 | 36.547619 | -1.567669 | 11 | 26.01 | 2.364545 |  |
| GO:0031123\_RNA\_3'-end\_processing | SLBP | 8 | 1 | 31.979167 | -1.510477 | 12 | 31.08 | 2.590000 |  |
| GO:0000278\_mitotic\_cell\_cycle | NEK2 | 80 | 2 | 6.395833 | -1.419137 | 13 | 37.38 | 2.875385 |  |
| GO:0000278\_mitotic\_cell\_cycle | PES1 | 80 | 2 | 6.395833 | -1.419137 | 13 | 37.38 | 2.875385 |  |
| GO:0006826\_iron\_ion\_transport | SFXN1 | 10 | 1 | 25.583333 | -1.415166 | 14 | 42.03 | 3.002143 |  |
| GO:0030308\_negative\_regulation\_of\_cell\_growth | TRO | 11 | 1 | 23.257576 | -1.374573 | 16 | 46.74 | 2.921250 |  |
| GO:0042551\_neuron\_maturation | CDKN1C | 11 | 1 | 23.257576 | -1.374573 | 16 | 46.74 | 2.921250 |  |
| GO:0000279\_M\_phase | NEK2 | 85 | 2 | 6.019608 | -1.371137 | 17 | 46.88 | 2.757647 |  |
| GO:0000279\_M\_phase | PES1 | 85 | 2 | 6.019608 | -1.371137 | 17 | 46.88 | 2.757647 |  |
| GO:0006605\_protein\_targeting | ARL6IP1 | 86 | 2 | 5.949612 | -1.361914 | 18 | 47.34 | 2.630000 |  |
| GO:0006605\_protein\_targeting | KPNA4 | 86 | 2 | 5.949612 | -1.361914 | 18 | 47.34 | 2.630000 |  |
| GO:0045792\_negative\_regulation\_of\_cell\_size | TRO | 12 | 1 | 21.319444 | -1.337583 | 19 | 51.49 | 2.710000 |  |
| GO:0006898\_receptor-mediated\_endocytosis | PICALM | 13 | 1 | 19.679487 | -1.303619 | 20 | 55.74 | 2.787000 |  |
| GO:0030032\_lamellipodium\_assembly | NUP85 | 14 | 1 | 18.273810 | -1.272233 | 21 | 60.52 | 2.881905 |  |
| GO:0060713\_labyrinthine\_layer\_morphogenesis | DNAJB6 | 16 | 1 | 15.989583 | -1.215835 | 22 | 68.41 | 3.109545 |  |
| GO:0042254\_ribosome\_biogenesis | PES1 | 17 | 1 | 15.049020 | -1.190303 | 23 | 72.33 | 3.144783 |  |
| GO:0030595\_leukocyte\_chemotaxis | NUP85 | 19 | 1 | 13.464912 | -1.143591 | 25 | 79.72 | 3.188800 |  |
| GO:0060326\_cell\_chemotaxis | NUP85 | 19 | 1 | 13.464912 | -1.143591 | 25 | 79.72 | 3.188800 |  |
| GO:0016571\_histone\_methylation | MLL3 | 20 | 1 | 12.791667 | -1.122110 | 27 | 83.35 | 3.087037 |  |
| GO:0042326\_negative\_regulation\_of\_phosphorylation | CDKN1C | 20 | 1 | 12.791667 | -1.122110 | 27 | 83.35 | 3.087037 |  |
| GO:0022403\_cell\_cycle\_phase | NEK2 | 119 | 2 | 4.299720 | -1.111176 | 28 | 83.62 | 2.986429 |  |
| GO:0022403\_cell\_cycle\_phase | PES1 | 119 | 2 | 4.299720 | -1.111176 | 28 | 83.62 | 2.986429 |  |
| GO:0010563\_negative\_regulation\_of\_phosphorus\_metabolic\_process | CDKN1C | 21 | 1 | 12.182540 | -1.101716 | 30 | 87.32 | 2.910667 |  |
| GO:0045936\_negative\_regulation\_of\_phosphate\_metabolic\_process | CDKN1C | 21 | 1 | 12.182540 | -1.101716 | 30 | 87.32 | 2.910667 |  |
| GO:0006886\_intracellular\_protein\_transport | ARL6IP1 | 122 | 2 | 4.193989 | -1.092430 | 31 | 88.33 | 2.849355 |  |
| GO:0006886\_intracellular\_protein\_transport | KPNA4 | 122 | 2 | 4.193989 | -1.092430 | 31 | 88.33 | 2.849355 |  |
| GO:0000041\_transition\_metal\_ion\_transport | SFXN1 | 22 | 1 | 11.628788 | -1.082308 | 34 | 92.51 | 2.720882 |  |
| GO:0001558\_regulation\_of\_cell\_growth | TRO | 22 | 1 | 11.628788 | -1.082308 | 34 | 92.51 | 2.720882 |  |
| GO:0007059\_chromosome\_segregation | NEK2 | 22 | 1 | 11.628788 | -1.082308 | 34 | 92.51 | 2.720882 |  |
| GO:0006397\_mRNA\_processing | SLBP | 23 | 1 | 11.123188 | -1.063797 | 36 | 95.43 | 2.650833 |  |
| GO:0022613\_ribonucleoprotein\_complex\_biogenesis | PES1 | 23 | 1 | 11.123188 | -1.063797 | 36 | 95.43 | 2.650833 |  |
| GO:0051276\_chromosome\_organization | NEK2 | 129 | 2 | 3.966408 | -1.050702 | 37 | 96.3 | 2.602703 |  |
| GO:0051276\_chromosome\_organization | MLL3 | 129 | 2 | 3.966408 | -1.050702 | 37 | 96.3 | 2.602703 |  |
| GO:0006479\_protein\_amino\_acid\_methylation | MLL3 | 27 | 1 | 9.475309 | -0.997336 | 40 | 107.5 | 2.687500 |  |
| GO:0008213\_protein\_amino\_acid\_alkylation | MLL3 | 27 | 1 | 9.475309 | -0.997336 | 40 | 107.5 | 2.687500 |  |
| GO:0031016\_pancreas\_development | IGF2 | 27 | 1 | 9.475309 | -0.997336 | 40 | 107.5 | 2.687500 |  |
| GO:0034613\_cellular\_protein\_localization | ARL6IP1 | 139 | 2 | 3.681055 | -0.995487 | 41 | 107.78 | 2.628780 |  |
| GO:0034613\_cellular\_protein\_localization | KPNA4 | 139 | 2 | 3.681055 | -0.995487 | 41 | 107.78 | 2.628780 |  |
| GO:0070727\_cellular\_macromolecule\_localization | ARL6IP1 | 141 | 2 | 3.628842 | -0.985007 | 42 | 108.16 | 2.575238 |  |
| GO:0070727\_cellular\_macromolecule\_localization | KPNA4 | 141 | 2 | 3.628842 | -0.985007 | 42 | 108.16 | 2.575238 |  |
| GO:0006997\_nucleus\_organization | PES1 | 28 | 1 | 9.136905 | -0.982334 | 44 | 110.13 | 2.502955 |  |
| GO:0045926\_negative\_regulation\_of\_growth | TRO | 28 | 1 | 9.136905 | -0.982334 | 44 | 110.13 | 2.502955 |  |
| GO:0016049\_cell\_growth | TRO | 31 | 1 | 8.252688 | -0.940506 | 45 | 120.28 | 2.672889 |  |
| GO:0022402\_cell\_cycle\_process | NEK2 | 155 | 2 | 3.301075 | -0.916300 | 46 | 122.9 | 2.671739 |  |
| GO:0022402\_cell\_cycle\_process | PES1 | 155 | 2 | 3.301075 | -0.916300 | 46 | 122.9 | 2.671739 |  |
| GO:0060711\_labyrinthine\_layer\_development | DNAJB6 | 34 | 1 | 7.524510 | -0.902760 | 47 | 127.09 | 2.704043 |  |
| GO:0050900\_leukocyte\_migration | NUP85 | 36 | 1 | 7.106481 | -0.879516 | 48 | 132.19 | 2.753958 |  |
| GO:0032259\_methylation | MLL3 | 38 | 1 | 6.732456 | -0.857613 | 50 | 138.3 | 2.766000 |  |
| GO:0043414\_biopolymer\_methylation | MLL3 | 38 | 1 | 6.732456 | -0.857613 | 50 | 138.3 | 2.766000 |  |
| GO:0006730\_one-carbon\_metabolic\_process | MLL3 | 39 | 1 | 6.559829 | -0.847120 | 51 | 141.26 | 2.769804 |  |
| GO:0008203\_cholesterol\_metabolic\_process | SOAT1 | 40 | 1 | 6.395833 | -0.836913 | 54 | 143.78 | 2.662593 |  |
| GO:0016071\_mRNA\_metabolic\_process | SLBP | 40 | 1 | 6.395833 | -0.836913 | 54 | 143.78 | 2.662593 |  |
| GO:0035272\_exocrine\_system\_development | IGF2 | 40 | 1 | 6.395833 | -0.836913 | 54 | 143.78 | 2.662593 |  |
| GO:0015031\_protein\_transport | ARL6IP1 | 175 | 2 | 2.923810 | -0.830215 | 55 | 144.12 | 2.620364 |  |
| GO:0015031\_protein\_transport | KPNA4 | 175 | 2 | 2.923810 | -0.830215 | 55 | 144.12 | 2.620364 |  |
| GO:0008361\_regulation\_of\_cell\_size | TRO | 42 | 1 | 6.091270 | -0.817298 | 57 | 150.35 | 2.637719 |  |
| GO:0016125\_sterol\_metabolic\_process | SOAT1 | 42 | 1 | 6.091270 | -0.817298 | 57 | 150.35 | 2.637719 |  |
| GO:0045184\_establishment\_of\_protein\_localization | ARL6IP1 | 180 | 2 | 2.842593 | -0.810578 | 58 | 151.13 | 2.605690 |  |
| GO:0045184\_establishment\_of\_protein\_localization | KPNA4 | 180 | 2 | 2.842593 | -0.810578 | 58 | 151.13 | 2.605690 |  |
| GO:0006606\_protein\_import\_into\_nucleus | KPNA4 | 44 | 1 | 5.814394 | -0.798668 | 60 | 158.22 | 2.637000 |  |
| GO:0051170\_nuclear\_import | KPNA4 | 44 | 1 | 5.814394 | -0.798668 | 60 | 158.22 | 2.637000 |  |
| GO:0007155\_cell\_adhesion | TRO | 186 | 2 | 2.750896 | -0.787894 | 62 | 160.39 | 2.586935 |  |
| GO:0007155\_cell\_adhesion | DNAJB6 | 186 | 2 | 2.750896 | -0.787894 | 62 | 160.39 | 2.586935 |  |
| GO:0022610\_biological\_adhesion | TRO | 186 | 2 | 2.750896 | -0.787894 | 62 | 160.39 | 2.586935 |  |
| GO:0022610\_biological\_adhesion | DNAJB6 | 186 | 2 | 2.750896 | -0.787894 | 62 | 160.39 | 2.586935 |  |
| GO:0030218\_erythrocyte\_differentiation | SFXN1 | 46 | 1 | 5.561594 | -0.780935 | 63 | 161.98 | 2.571111 |  |
| GO:0006396\_RNA\_processing | SLBP | 47 | 1 | 5.443262 | -0.772380 | 65 | 164.7 | 2.533846 |  |
| GO:0016570\_histone\_modification | MLL3 | 47 | 1 | 5.443262 | -0.772380 | 65 | 164.7 | 2.533846 |  |
| GO:0034504\_protein\_localization\_in\_nucleus | KPNA4 | 48 | 1 | 5.329861 | -0.764021 | 66 | 165.85 | 2.512879 |  |
| GO:0046907\_intracellular\_transport | ARL6IP1 | 194 | 2 | 2.637457 | -0.759043 | 67 | 166.33 | 2.482537 |  |
| GO:0046907\_intracellular\_transport | KPNA4 | 194 | 2 | 2.637457 | -0.759043 | 67 | 166.33 | 2.482537 |  |
| GO:0034101\_erythrocyte\_homeostasis | SFXN1 | 49 | 1 | 5.221088 | -0.755851 | 68 | 168.75 | 2.481618 |  |
| GO:0017038\_protein\_import | KPNA4 | 50 | 1 | 5.116667 | -0.747861 | 69 | 171.33 | 2.483043 |  |
| GO:0016569\_covalent\_chromatin\_modification | MLL3 | 51 | 1 | 5.016340 | -0.740044 | 70 | 173.98 | 2.485429 |  |
| GO:0006935\_chemotaxis | NUP85 | 53 | 1 | 4.827044 | -0.724905 | 73 | 176.91 | 2.423425 |  |
| GO:0030031\_cell\_projection\_assembly | NUP85 | 53 | 1 | 4.827044 | -0.724905 | 73 | 176.91 | 2.423425 |  |
| GO:0042330\_taxis | NUP85 | 53 | 1 | 4.827044 | -0.724905 | 73 | 176.91 | 2.423425 |  |
| GO:0009987\_cellular\_process | ARL6IP1 | 3868 | 17 | 1.124397 | -0.718280 | 74 | 177.69 | 2.401216 |  |
| GO:0009987\_cellular\_process | SOAT1 | 3868 | 17 | 1.124397 | -0.718280 | 74 | 177.69 | 2.401216 |  |
| GO:0009987\_cellular\_process | WNT5B | 3868 | 17 | 1.124397 | -0.718280 | 74 | 177.69 | 2.401216 |  |
| GO:0009987\_cellular\_process | NEK2 | 3868 | 17 | 1.124397 | -0.718280 | 74 | 177.69 | 2.401216 |  |
| GO:0009987\_cellular\_process | E2F8 | 3868 | 17 | 1.124397 | -0.718280 | 74 | 177.69 | 2.401216 |  |
| GO:0009987\_cellular\_process | NUDT5 | 3868 | 17 | 1.124397 | -0.718280 | 74 | 177.69 | 2.401216 |  |
| GO:0009987\_cellular\_process | SFXN1 | 3868 | 17 | 1.124397 | -0.718280 | 74 | 177.69 | 2.401216 |  |
| GO:0009987\_cellular\_process | NUP85 | 3868 | 17 | 1.124397 | -0.718280 | 74 | 177.69 | 2.401216 |  |
| GO:0009987\_cellular\_process | SLBP | 3868 | 17 | 1.124397 | -0.718280 | 74 | 177.69 | 2.401216 |  |
| GO:0009987\_cellular\_process | CDKN1C | 3868 | 17 | 1.124397 | -0.718280 | 74 | 177.69 | 2.401216 |  |
| GO:0009987\_cellular\_process | PICALM | 3868 | 17 | 1.124397 | -0.718280 | 74 | 177.69 | 2.401216 |  |
| GO:0009987\_cellular\_process | TRO | 3868 | 17 | 1.124397 | -0.718280 | 74 | 177.69 | 2.401216 |  |
| GO:0009987\_cellular\_process | KPNA4 | 3868 | 17 | 1.124397 | -0.718280 | 74 | 177.69 | 2.401216 |  |
| GO:0009987\_cellular\_process | MLL3 | 3868 | 17 | 1.124397 | -0.718280 | 74 | 177.69 | 2.401216 |  |
| GO:0009987\_cellular\_process | PES1 | 3868 | 17 | 1.124397 | -0.718280 | 74 | 177.69 | 2.401216 |  |
| GO:0009987\_cellular\_process | APEX1 | 3868 | 17 | 1.124397 | -0.718280 | 74 | 177.69 | 2.401216 |  |
| GO:0009987\_cellular\_process | DNAJB6 | 3868 | 17 | 1.124397 | -0.718280 | 74 | 177.69 | 2.401216 |  |
| GO:0001892\_embryonic\_placenta\_development | DNAJB6 | 57 | 1 | 4.488304 | -0.696433 | 76 | 186.49 | 2.453816 |  |
| GO:0033365\_protein\_localization\_in\_organelle | KPNA4 | 57 | 1 | 4.488304 | -0.696433 | 76 | 186.49 | 2.453816 |  |
| GO:0048469\_cell\_maturation | CDKN1C | 59 | 1 | 4.336158 | -0.683018 | 77 | 188.4 | 2.446753 |  |
| GO:0045892\_negative\_regulation\_of\_transcription\_\_DNA-dependent | CDKN1C | 218 | 2 | 2.347095 | -0.680882 | 78 | 188.53 | 2.417051 |  |
| GO:0045892\_negative\_regulation\_of\_transcription\_\_DNA-dependent | DNAJB6 | 218 | 2 | 2.347095 | -0.680882 | 78 | 188.53 | 2.417051 |  |
| GO:0051253\_negative\_regulation\_of\_RNA\_metabolic\_process | CDKN1C | 220 | 2 | 2.325758 | -0.674876 | 79 | 189.33 | 2.396582 |  |
| GO:0051253\_negative\_regulation\_of\_RNA\_metabolic\_process | DNAJB6 | 220 | 2 | 2.325758 | -0.674876 | 79 | 189.33 | 2.396582 |  |
| GO:0044085\_cellular\_component\_biogenesis | NUP85 | 237 | 2 | 2.158931 | -0.626579 | 80 | 202.03 | 2.525375 |  |
| GO:0044085\_cellular\_component\_biogenesis | PES1 | 237 | 2 | 2.158931 | -0.626579 | 80 | 202.03 | 2.525375 |  |
| GO:0007049\_cell\_cycle | NEK2 | 238 | 2 | 2.149860 | -0.623882 | 81 | 202.27 | 2.497160 |  |
| GO:0007049\_cell\_cycle | PES1 | 238 | 2 | 2.149860 | -0.623882 | 81 | 202.27 | 2.497160 |  |
| GO:0006913\_nucleocytoplasmic\_transport | KPNA4 | 71 | 1 | 3.603286 | -0.611946 | 82 | 205.95 | 2.511585 |  |
| GO:0016568\_chromatin\_modification | MLL3 | 72 | 1 | 3.553241 | -0.606648 | 84 | 209.16 | 2.490000 |  |
| GO:0051169\_nuclear\_transport | KPNA4 | 72 | 1 | 3.553241 | -0.606648 | 84 | 209.16 | 2.490000 |  |
| GO:0006996\_organelle\_organization | NEK2 | 449 | 3 | 1.709354 | -0.596047 | 85 | 212.06 | 2.494824 |  |
| GO:0006996\_organelle\_organization | PES1 | 449 | 3 | 1.709354 | -0.596047 | 85 | 212.06 | 2.494824 |  |
| GO:0006996\_organelle\_organization | MLL3 | 449 | 3 | 1.709354 | -0.596047 | 85 | 212.06 | 2.494824 |  |
| GO:0008104\_protein\_localization | ARL6IP1 | 251 | 2 | 2.038513 | -0.590167 | 86 | 213.56 | 2.483256 |  |
| GO:0008104\_protein\_localization | KPNA4 | 251 | 2 | 2.038513 | -0.590167 | 86 | 213.56 | 2.483256 |  |
| GO:0016481\_negative\_regulation\_of\_transcription | CDKN1C | 253 | 2 | 2.022398 | -0.585191 | 88 | 214.82 | 2.441136 |  |
| GO:0016481\_negative\_regulation\_of\_transcription | DNAJB6 | 253 | 2 | 2.022398 | -0.585191 | 88 | 214.82 | 2.441136 |  |
| GO:0030097\_hemopoiesis | PICALM | 253 | 2 | 2.022398 | -0.585191 | 88 | 214.82 | 2.441136 |  |
| GO:0030097\_hemopoiesis | SFXN1 | 253 | 2 | 2.022398 | -0.585191 | 88 | 214.82 | 2.441136 |  |
| GO:0001890\_placenta\_development | DNAJB6 | 77 | 1 | 3.322511 | -0.581360 | 89 | 215.64 | 2.422921 |  |
| GO:0015674\_di-\_\_tri-valent\_inorganic\_cation\_transport | SFXN1 | 79 | 1 | 3.238397 | -0.571769 | 90 | 219.12 | 2.434667 |  |
| GO:0010629\_negative\_regulation\_of\_gene\_expression | CDKN1C | 262 | 2 | 1.952926 | -0.563450 | 91 | 221.28 | 2.431648 |  |
| GO:0010629\_negative\_regulation\_of\_gene\_expression | DNAJB6 | 262 | 2 | 1.952926 | -0.563450 | 91 | 221.28 | 2.431648 |  |
| GO:0021700\_developmental\_maturation | CDKN1C | 81 | 1 | 3.158436 | -0.562455 | 92 | 221.5 | 2.407609 |  |
| GO:0008202\_steroid\_metabolic\_process | SOAT1 | 82 | 1 | 3.119919 | -0.557898 | 93 | 223.27 | 2.400753 |  |
| GO:0006325\_chromatin\_organization | MLL3 | 83 | 1 | 3.082329 | -0.553405 | 94 | 225.03 | 2.393936 |  |
| GO:0045934\_negative\_regulation\_of\_nucleobase\_\_nucleoside\_\_nucleotide\_and\_nucleic\_acid\_metabolic\_process | CDKN1C | 270 | 2 | 1.895062 | -0.544976 | 95 | 226.05 | 2.379474 |  |
| GO:0045934\_negative\_regulation\_of\_nucleobase\_\_nucleoside\_\_nucleotide\_and\_nucleic\_acid\_metabolic\_process | DNAJB6 | 270 | 2 | 1.895062 | -0.544976 | 95 | 226.05 | 2.379474 |  |
| GO:0070887\_cellular\_response\_to\_chemical\_stimulus | NUP85 | 85 | 1 | 3.009804 | -0.544604 | 96 | 226.75 | 2.361979 |  |
| GO:0051172\_negative\_regulation\_of\_nitrogen\_compound\_metabolic\_process | CDKN1C | 271 | 2 | 1.888069 | -0.542721 | 97 | 226.86 | 2.338763 |  |
| GO:0051172\_negative\_regulation\_of\_nitrogen\_compound\_metabolic\_process | DNAJB6 | 271 | 2 | 1.888069 | -0.542721 | 97 | 226.86 | 2.338763 |  |
| GO:0006897\_endocytosis | PICALM | 86 | 1 | 2.974806 | -0.540295 | 99 | 228.54 | 2.308485 |  |
| GO:0010324\_membrane\_invagination | PICALM | 86 | 1 | 2.974806 | -0.540295 | 99 | 228.54 | 2.308485 |  |
| GO:0016337\_cell-cell\_adhesion | DNAJB6 | 87 | 1 | 2.940613 | -0.536043 | 100 | 231.14 | 2.311400 |  |
| GO:0010558\_negative\_regulation\_of\_macromolecule\_biosynthetic\_process | CDKN1C | 274 | 2 | 1.867397 | -0.536025 | 102 | 231.47 | 2.269314 |  |
| GO:0010558\_negative\_regulation\_of\_macromolecule\_biosynthetic\_process | DNAJB6 | 274 | 2 | 1.867397 | -0.536025 | 102 | 231.47 | 2.269314 |  |
| GO:0033036\_macromolecule\_localization | ARL6IP1 | 274 | 2 | 1.867397 | -0.536025 | 102 | 231.47 | 2.269314 |  |
| GO:0033036\_macromolecule\_localization | KPNA4 | 274 | 2 | 1.867397 | -0.536025 | 102 | 231.47 | 2.269314 |  |
| GO:0048534\_hemopoietic\_or\_lymphoid\_organ\_development | PICALM | 277 | 2 | 1.847172 | -0.529431 | 103 | 232.59 | 2.258155 |  |
| GO:0048534\_hemopoietic\_or\_lymphoid\_organ\_development | SFXN1 | 277 | 2 | 1.847172 | -0.529431 | 103 | 232.59 | 2.258155 |  |
| GO:0031327\_negative\_regulation\_of\_cellular\_biosynthetic\_process | CDKN1C | 282 | 2 | 1.814421 | -0.518663 | 104 | 235.21 | 2.261635 |  |
| GO:0031327\_negative\_regulation\_of\_cellular\_biosynthetic\_process | DNAJB6 | 282 | 2 | 1.814421 | -0.518663 | 104 | 235.21 | 2.261635 |  |
| GO:0006810\_transport | ARL6IP1 | 718 | 4 | 1.425255 | -0.515752 | 105 | 235.73 | 2.245048 |  |
| GO:0006810\_transport | PICALM | 718 | 4 | 1.425255 | -0.515752 | 105 | 235.73 | 2.245048 |  |
| GO:0006810\_transport | SFXN1 | 718 | 4 | 1.425255 | -0.515752 | 105 | 235.73 | 2.245048 |  |
| GO:0006810\_transport | KPNA4 | 718 | 4 | 1.425255 | -0.515752 | 105 | 235.73 | 2.245048 |  |
| GO:0009890\_negative\_regulation\_of\_biosynthetic\_process | CDKN1C | 284 | 2 | 1.801643 | -0.514431 | 106 | 236.67 | 2.232736 |  |
| GO:0009890\_negative\_regulation\_of\_biosynthetic\_process | DNAJB6 | 284 | 2 | 1.801643 | -0.514431 | 106 | 236.67 | 2.232736 |  |
| GO:0051234\_establishment\_of\_localization | ARL6IP1 | 729 | 4 | 1.403749 | -0.501219 | 107 | 240.74 | 2.249907 |  |
| GO:0051234\_establishment\_of\_localization | PICALM | 729 | 4 | 1.403749 | -0.501219 | 107 | 240.74 | 2.249907 |  |
| GO:0051234\_establishment\_of\_localization | SFXN1 | 729 | 4 | 1.403749 | -0.501219 | 107 | 240.74 | 2.249907 |  |
| GO:0051234\_establishment\_of\_localization | KPNA4 | 729 | 4 | 1.403749 | -0.501219 | 107 | 240.74 | 2.249907 |  |
| GO:0002376\_immune\_system\_process | PICALM | 505 | 3 | 1.519802 | -0.501050 | 108 | 240.91 | 2.230648 |  |
| GO:0002376\_immune\_system\_process | NUP85 | 505 | 3 | 1.519802 | -0.501050 | 108 | 240.91 | 2.230648 |  |
| GO:0002376\_immune\_system\_process | SFXN1 | 505 | 3 | 1.519802 | -0.501050 | 108 | 240.91 | 2.230648 |  |
| GO:0016043\_cellular\_component\_organization | PICALM | 964 | 5 | 1.326936 | -0.495927 | 109 | 243.04 | 2.229725 |  |
| GO:0016043\_cellular\_component\_organization | NEK2 | 964 | 5 | 1.326936 | -0.495927 | 109 | 243.04 | 2.229725 |  |
| GO:0016043\_cellular\_component\_organization | NUP85 | 964 | 5 | 1.326936 | -0.495927 | 109 | 243.04 | 2.229725 |  |
| GO:0016043\_cellular\_component\_organization | PES1 | 964 | 5 | 1.326936 | -0.495927 | 109 | 243.04 | 2.229725 |  |
| GO:0016043\_cellular\_component\_organization | MLL3 | 964 | 5 | 1.326936 | -0.495927 | 109 | 243.04 | 2.229725 |  |
| GO:0002520\_immune\_system\_development | PICALM | 295 | 2 | 1.734463 | -0.491899 | 110 | 244.76 | 2.225091 |  |
| GO:0002520\_immune\_system\_development | SFXN1 | 295 | 2 | 1.734463 | -0.491899 | 110 | 244.76 | 2.225091 |  |
| GO:0048872\_homeostasis\_of\_number\_of\_cells | SFXN1 | 105 | 1 | 2.436508 | -0.468153 | 111 | 250.57 | 2.257387 |  |
| GO:0030099\_myeloid\_cell\_differentiation | SFXN1 | 108 | 1 | 2.368827 | -0.458203 | 112 | 252.45 | 2.254018 |  |
| GO:0040008\_regulation\_of\_growth | TRO | 113 | 1 | 2.264012 | -0.442346 | 113 | 256.27 | 2.267876 |  |
| GO:0010605\_negative\_regulation\_of\_macromolecule\_metabolic\_process | CDKN1C | 331 | 2 | 1.545821 | -0.426006 | 114 | 260.96 | 2.289123 |  |
| GO:0010605\_negative\_regulation\_of\_macromolecule\_metabolic\_process | DNAJB6 | 331 | 2 | 1.545821 | -0.426006 | 114 | 260.96 | 2.289123 |  |
| GO:0031324\_negative\_regulation\_of\_cellular\_metabolic\_process | CDKN1C | 332 | 2 | 1.541165 | -0.424329 | 115 | 261.74 | 2.276000 |  |
| GO:0031324\_negative\_regulation\_of\_cellular\_metabolic\_process | DNAJB6 | 332 | 2 | 1.541165 | -0.424329 | 115 | 261.74 | 2.276000 |  |
| GO:0030001\_metal\_ion\_transport | SFXN1 | 122 | 1 | 2.096995 | -0.415877 | 116 | 265.03 | 2.284741 |  |
| GO:0051649\_establishment\_of\_localization\_in\_cell | ARL6IP1 | 342 | 2 | 1.496101 | -0.407981 | 117 | 267.47 | 2.286068 |  |
| GO:0051649\_establishment\_of\_localization\_in\_cell | KPNA4 | 342 | 2 | 1.496101 | -0.407981 | 117 | 267.47 | 2.286068 |  |
| GO:0009892\_negative\_regulation\_of\_metabolic\_process | CDKN1C | 348 | 2 | 1.470307 | -0.398521 | 118 | 269.9 | 2.287288 |  |
| GO:0009892\_negative\_regulation\_of\_metabolic\_process | DNAJB6 | 348 | 2 | 1.470307 | -0.398521 | 118 | 269.9 | 2.287288 |  |
| GO:0051179\_localization | ARL6IP1 | 1058 | 5 | 1.209042 | -0.397737 | 119 | 270.05 | 2.269328 |  |
| GO:0051179\_localization | PICALM | 1058 | 5 | 1.209042 | -0.397737 | 119 | 270.05 | 2.269328 |  |
| GO:0051179\_localization | NUP85 | 1058 | 5 | 1.209042 | -0.397737 | 119 | 270.05 | 2.269328 |  |
| GO:0051179\_localization | SFXN1 | 1058 | 5 | 1.209042 | -0.397737 | 119 | 270.05 | 2.269328 |  |
| GO:0051179\_localization | KPNA4 | 1058 | 5 | 1.209042 | -0.397737 | 119 | 270.05 | 2.269328 |  |
| GO:0016044\_membrane\_organization | PICALM | 140 | 1 | 1.827381 | -0.369632 | 120 | 277.57 | 2.313083 |  |
| GO:0051641\_cellular\_localization | ARL6IP1 | 370 | 2 | 1.382883 | -0.365912 | 121 | 278.59 | 2.302397 |  |
| GO:0051641\_cellular\_localization | KPNA4 | 370 | 2 | 1.382883 | -0.365912 | 121 | 278.59 | 2.302397 |  |
| GO:0006812\_cation\_transport | SFXN1 | 146 | 1 | 1.752283 | -0.355886 | 122 | 281.77 | 2.309590 |  |
| GO:0006066\_alcohol\_metabolic\_process | SOAT1 | 158 | 1 | 1.619198 | -0.330493 | 123 | 289.02 | 2.349756 |  |
| GO:0007626\_locomotory\_behavior | NUP85 | 163 | 1 | 1.569530 | -0.320659 | 124 | 291.59 | 2.351532 |  |
| GO:0042325\_regulation\_of\_phosphorylation | CDKN1C | 164 | 1 | 1.559959 | -0.318740 | 125 | 291.97 | 2.335760 |  |
| GO:0019220\_regulation\_of\_phosphate\_metabolic\_process | CDKN1C | 165 | 1 | 1.550505 | -0.316838 | 127 | 293.81 | 2.313465 |  |
| GO:0051174\_regulation\_of\_phosphorus\_metabolic\_process | CDKN1C | 165 | 1 | 1.550505 | -0.316838 | 127 | 293.81 | 2.313465 |  |
| GO:0016070\_RNA\_metabolic\_process | CDKN1C | 658 | 3 | 1.166413 | -0.313495 | 128 | 295.25 | 2.306641 |  |
| GO:0016070\_RNA\_metabolic\_process | DNAJB6 | 658 | 3 | 1.166413 | -0.313495 | 128 | 295.25 | 2.306641 |  |
| GO:0016070\_RNA\_metabolic\_process | SLBP | 658 | 3 | 1.166413 | -0.313495 | 128 | 295.25 | 2.306641 |  |
| GO:0042592\_homeostatic\_process | SFXN1 | 419 | 2 | 1.221161 | -0.303440 | 129 | 298.66 | 2.315194 |  |
| GO:0042592\_homeostatic\_process | APEX1 | 419 | 2 | 1.221161 | -0.303440 | 129 | 298.66 | 2.315194 |  |
| GO:0000122\_negative\_regulation\_of\_transcription\_from\_RNA\_polymerase\_II\_promoter | CDKN1C | 175 | 1 | 1.461905 | -0.298635 | 130 | 301.17 | 2.316692 |  |
| GO:0016192\_vesicle-mediated\_transport | PICALM | 184 | 1 | 1.390399 | -0.283437 | 131 | 308.83 | 2.357481 |  |
| GO:0065008\_regulation\_of\_biological\_quality | TRO | 693 | 3 | 1.107504 | -0.281517 | 132 | 309.31 | 2.343258 |  |
| GO:0065008\_regulation\_of\_biological\_quality | SFXN1 | 693 | 3 | 1.107504 | -0.281517 | 132 | 309.31 | 2.343258 |  |
| GO:0065008\_regulation\_of\_biological\_quality | APEX1 | 693 | 3 | 1.107504 | -0.281517 | 132 | 309.31 | 2.343258 |  |
| GO:0006811\_ion\_transport | SFXN1 | 186 | 1 | 1.375448 | -0.280199 | 133 | 310.8 | 2.336842 |  |
| GO:0019725\_cellular\_homeostasis | APEX1 | 195 | 1 | 1.311966 | -0.266217 | 134 | 315.76 | 2.356418 |  |
| GO:0022607\_cellular\_component\_assembly | NUP85 | 204 | 1 | 1.254085 | -0.253127 | 135 | 319.76 | 2.368593 |  |
| GO:0006139\_nucleobase\_\_nucleoside\_\_nucleotide\_and\_nucleic\_acid\_metabolic\_process | CDKN1C | 1002 | 4 | 1.021291 | -0.241816 | 136 | 322.65 | 2.372426 |  |
| GO:0006139\_nucleobase\_\_nucleoside\_\_nucleotide\_and\_nucleic\_acid\_metabolic\_process | NUDT5 | 1002 | 4 | 1.021291 | -0.241816 | 136 | 322.65 | 2.372426 |  |
| GO:0006139\_nucleobase\_\_nucleoside\_\_nucleotide\_and\_nucleic\_acid\_metabolic\_process | DNAJB6 | 1002 | 4 | 1.021291 | -0.241816 | 136 | 322.65 | 2.372426 |  |
| GO:0006139\_nucleobase\_\_nucleoside\_\_nucleotide\_and\_nucleic\_acid\_metabolic\_process | SLBP | 1002 | 4 | 1.021291 | -0.241816 | 136 | 322.65 | 2.372426 |  |
| GO:0040007\_growth | TRO | 217 | 1 | 1.178955 | -0.235634 | 137 | 324.07 | 2.365474 |  |
| GO:0001701\_in\_utero\_embryonic\_development | DNAJB6 | 221 | 1 | 1.157617 | -0.230560 | 138 | 326.36 | 2.364928 |  |
| GO:0048523\_negative\_regulation\_of\_cellular\_process | CDKN1C | 774 | 3 | 0.991602 | -0.218994 | 139 | 328.87 | 2.365971 |  |
| GO:0048523\_negative\_regulation\_of\_cellular\_process | TRO | 774 | 3 | 0.991602 | -0.218994 | 139 | 328.87 | 2.365971 |  |
| GO:0048523\_negative\_regulation\_of\_cellular\_process | DNAJB6 | 774 | 3 | 0.991602 | -0.218994 | 139 | 328.87 | 2.365971 |  |
| GO:0016477\_cell\_migration | NUP85 | 234 | 1 | 1.093305 | -0.214979 | 140 | 330.69 | 2.362071 |  |
| GO:0007267\_cell-cell\_signaling | WNT5B | 252 | 1 | 1.015212 | -0.195473 | 141 | 336.56 | 2.386950 |  |
| GO:0048731\_system\_development | CDKN1C | 1609 | 6 | 0.954009 | -0.191549 | 142 | 338.89 | 2.386549 |  |
| GO:0048731\_system\_development | WNT5B | 1609 | 6 | 0.954009 | -0.191549 | 142 | 338.89 | 2.386549 |  |
| GO:0048731\_system\_development | PICALM | 1609 | 6 | 0.954009 | -0.191549 | 142 | 338.89 | 2.386549 |  |
| GO:0048731\_system\_development | IGF2 | 1609 | 6 | 0.954009 | -0.191549 | 142 | 338.89 | 2.386549 |  |
| GO:0048731\_system\_development | SFXN1 | 1609 | 6 | 0.954009 | -0.191549 | 142 | 338.89 | 2.386549 |  |
| GO:0048731\_system\_development | DNAJB6 | 1609 | 6 | 0.954009 | -0.191549 | 142 | 338.89 | 2.386549 |  |
| GO:0048870\_cell\_motility | NUP85 | 257 | 1 | 0.995460 | -0.190437 | 143 | 339.67 | 2.375315 |  |
| GO:0048666\_neuron\_development | CDKN1C | 262 | 1 | 0.976463 | -0.185556 | 144 | 342.27 | 2.376875 |  |
| GO:0030030\_cell\_projection\_organization | NUP85 | 263 | 1 | 0.972750 | -0.184597 | 145 | 342.56 | 2.362483 |  |
| GO:0044255\_cellular\_lipid\_metabolic\_process | SOAT1 | 264 | 1 | 0.969066 | -0.183645 | 146 | 342.91 | 2.348699 |  |
| GO:0048513\_organ\_development | PICALM | 1365 | 5 | 0.937118 | -0.183378 | 147 | 343.1 | 2.334014 |  |
| GO:0048513\_organ\_development | WNT5B | 1365 | 5 | 0.937118 | -0.183378 | 147 | 343.1 | 2.334014 |  |
| GO:0048513\_organ\_development | SFXN1 | 1365 | 5 | 0.937118 | -0.183378 | 147 | 343.1 | 2.334014 |  |
| GO:0048513\_organ\_development | IGF2 | 1365 | 5 | 0.937118 | -0.183378 | 147 | 343.1 | 2.334014 |  |
| GO:0048513\_organ\_development | DNAJB6 | 1365 | 5 | 0.937118 | -0.183378 | 147 | 343.1 | 2.334014 |  |
| GO:0051716\_cellular\_response\_to\_stimulus | NUP85 | 273 | 1 | 0.937118 | -0.175327 | 148 | 345.46 | 2.334189 |  |
| GO:0007610\_behavior | NUP85 | 279 | 1 | 0.916965 | -0.170027 | 149 | 348.29 | 2.337517 |  |
| GO:0006355\_regulation\_of\_transcription\_\_DNA-dependent | CDKN1C | 575 | 2 | 0.889855 | -0.169348 | 150 | 348.87 | 2.325800 |  |
| GO:0006355\_regulation\_of\_transcription\_\_DNA-dependent | DNAJB6 | 575 | 2 | 0.889855 | -0.169348 | 150 | 348.87 | 2.325800 |  |
| GO:0048519\_negative\_regulation\_of\_biological\_process | CDKN1C | 859 | 3 | 0.893481 | -0.167469 | 151 | 349.36 | 2.313642 |  |
| GO:0048519\_negative\_regulation\_of\_biological\_process | TRO | 859 | 3 | 0.893481 | -0.167469 | 151 | 349.36 | 2.313642 |  |
| GO:0048519\_negative\_regulation\_of\_biological\_process | DNAJB6 | 859 | 3 | 0.893481 | -0.167469 | 151 | 349.36 | 2.313642 |  |
| GO:0006629\_lipid\_metabolic\_process | SOAT1 | 285 | 1 | 0.897661 | -0.164913 | 152 | 350.92 | 2.308684 |  |
| GO:0051252\_regulation\_of\_RNA\_metabolic\_process | CDKN1C | 590 | 2 | 0.867232 | -0.160150 | 153 | 352.24 | 2.302222 |  |
| GO:0051252\_regulation\_of\_RNA\_metabolic\_process | DNAJB6 | 590 | 2 | 0.867232 | -0.160150 | 153 | 352.24 | 2.302222 |  |
| GO:0006807\_nitrogen\_compound\_metabolic\_process | CDKN1C | 1147 | 4 | 0.892183 | -0.160135 | 154 | 352.53 | 2.289156 |  |
| GO:0006807\_nitrogen\_compound\_metabolic\_process | NUDT5 | 1147 | 4 | 0.892183 | -0.160135 | 154 | 352.53 | 2.289156 |  |
| GO:0006807\_nitrogen\_compound\_metabolic\_process | DNAJB6 | 1147 | 4 | 0.892183 | -0.160135 | 154 | 352.53 | 2.289156 |  |
| GO:0006807\_nitrogen\_compound\_metabolic\_process | SLBP | 1147 | 4 | 0.892183 | -0.160135 | 154 | 352.53 | 2.289156 |  |
| GO:0006351\_transcription\_\_DNA-dependent | CDKN1C | 594 | 2 | 0.861392 | -0.157782 | 155 | 353.07 | 2.277871 |  |
| GO:0006351\_transcription\_\_DNA-dependent | DNAJB6 | 594 | 2 | 0.861392 | -0.157782 | 155 | 353.07 | 2.277871 |  |
| GO:0032774\_RNA\_biosynthetic\_process | CDKN1C | 595 | 2 | 0.859944 | -0.157195 | 156 | 353.39 | 2.265321 |  |
| GO:0032774\_RNA\_biosynthetic\_process | DNAJB6 | 595 | 2 | 0.859944 | -0.157195 | 156 | 353.39 | 2.265321 |  |
| GO:0040011\_locomotion | NUP85 | 295 | 1 | 0.867232 | -0.156778 | 157 | 354.58 | 2.258471 |  |
| GO:0048856\_anatomical\_structure\_development | CDKN1C | 1688 | 6 | 0.909360 | -0.155841 | 158 | 355.01 | 2.246899 |  |
| GO:0048856\_anatomical\_structure\_development | PICALM | 1688 | 6 | 0.909360 | -0.155841 | 158 | 355.01 | 2.246899 |  |
| GO:0048856\_anatomical\_structure\_development | WNT5B | 1688 | 6 | 0.909360 | -0.155841 | 158 | 355.01 | 2.246899 |  |
| GO:0048856\_anatomical\_structure\_development | SFXN1 | 1688 | 6 | 0.909360 | -0.155841 | 158 | 355.01 | 2.246899 |  |
| GO:0048856\_anatomical\_structure\_development | IGF2 | 1688 | 6 | 0.909360 | -0.155841 | 158 | 355.01 | 2.246899 |  |
| GO:0048856\_anatomical\_structure\_development | DNAJB6 | 1688 | 6 | 0.909360 | -0.155841 | 158 | 355.01 | 2.246899 |  |
| GO:0016310\_phosphorylation | CDKN1C | 309 | 1 | 0.827940 | -0.146148 | 159 | 358.13 | 2.252390 |  |
| GO:0010467\_gene\_expression | CDKN1C | 905 | 3 | 0.848066 | -0.144467 | 160 | 358.58 | 2.241125 |  |
| GO:0010467\_gene\_expression | DNAJB6 | 905 | 3 | 0.848066 | -0.144467 | 160 | 358.58 | 2.241125 |  |
| GO:0010467\_gene\_expression | SLBP | 905 | 3 | 0.848066 | -0.144467 | 160 | 358.58 | 2.241125 |  |
| GO:0009887\_organ\_morphogenesis | WNT5B | 642 | 2 | 0.796989 | -0.131903 | 161 | 359.93 | 2.235590 |  |
| GO:0009887\_organ\_morphogenesis | IGF2 | 642 | 2 | 0.796989 | -0.131903 | 161 | 359.93 | 2.235590 |  |
| GO:0006928\_cell\_motion | NUP85 | 330 | 1 | 0.775253 | -0.131695 | 163 | 361.05 | 2.215031 |  |
| GO:0051674\_localization\_of\_cell | NUP85 | 330 | 1 | 0.775253 | -0.131695 | 163 | 361.05 | 2.215031 |  |
| GO:0007275\_multicellular\_organismal\_development | CDKN1C | 1760 | 6 | 0.872159 | -0.128096 | 164 | 362.92 | 2.212927 |  |
| GO:0007275\_multicellular\_organismal\_development | WNT5B | 1760 | 6 | 0.872159 | -0.128096 | 164 | 362.92 | 2.212927 |  |
| GO:0007275\_multicellular\_organismal\_development | PICALM | 1760 | 6 | 0.872159 | -0.128096 | 164 | 362.92 | 2.212927 |  |
| GO:0007275\_multicellular\_organismal\_development | IGF2 | 1760 | 6 | 0.872159 | -0.128096 | 164 | 362.92 | 2.212927 |  |
| GO:0007275\_multicellular\_organismal\_development | SFXN1 | 1760 | 6 | 0.872159 | -0.128096 | 164 | 362.92 | 2.212927 |  |
| GO:0007275\_multicellular\_organismal\_development | DNAJB6 | 1760 | 6 | 0.872159 | -0.128096 | 164 | 362.92 | 2.212927 |  |
| GO:0009605\_response\_to\_external\_stimulus | NUP85 | 339 | 1 | 0.754671 | -0.125995 | 165 | 363.74 | 2.204485 |  |
| GO:0006793\_phosphorus\_metabolic\_process | CDKN1C | 340 | 1 | 0.752451 | -0.125378 | 167 | 364.84 | 2.184671 |  |
| GO:0006796\_phosphate\_metabolic\_process | CDKN1C | 340 | 1 | 0.752451 | -0.125378 | 167 | 364.84 | 2.184671 |  |
| GO:0009653\_anatomical\_structure\_morphogenesis | WNT5B | 958 | 3 | 0.801148 | -0.121533 | 168 | 366.01 | 2.178631 |  |
| GO:0009653\_anatomical\_structure\_morphogenesis | IGF2 | 958 | 3 | 0.801148 | -0.121533 | 168 | 366.01 | 2.178631 |  |
| GO:0009653\_anatomical\_structure\_morphogenesis | DNAJB6 | 958 | 3 | 0.801148 | -0.121533 | 168 | 366.01 | 2.178631 |  |
| GO:0045449\_regulation\_of\_transcription | CDKN1C | 676 | 2 | 0.756903 | -0.116115 | 169 | 367.24 | 2.173018 |  |
| GO:0045449\_regulation\_of\_transcription | DNAJB6 | 676 | 2 | 0.756903 | -0.116115 | 169 | 367.24 | 2.173018 |  |
| GO:0030182\_neuron\_differentiation | CDKN1C | 356 | 1 | 0.718633 | -0.115955 | 170 | 367.53 | 2.161941 |  |
| GO:0043009\_chordate\_embryonic\_development | DNAJB6 | 365 | 1 | 0.700913 | -0.110996 | 171 | 369.44 | 2.160468 |  |
| GO:0009792\_embryonic\_development\_ending\_in\_birth\_or\_egg\_hatching | DNAJB6 | 368 | 1 | 0.695199 | -0.109395 | 172 | 369.8 | 2.150000 |  |
| GO:0006350\_transcription | CDKN1C | 701 | 2 | 0.729910 | -0.105682 | 173 | 371.37 | 2.146647 |  |
| GO:0006350\_transcription | DNAJB6 | 701 | 2 | 0.729910 | -0.105682 | 173 | 371.37 | 2.146647 |  |
| GO:0043687\_post-translational\_protein\_modification | MLL3 | 384 | 1 | 0.666233 | -0.101265 | 174 | 373.0 | 2.143678 |  |
| GO:0048699\_generation\_of\_neurons | CDKN1C | 396 | 1 | 0.646044 | -0.095594 | 175 | 375.12 | 2.143543 |  |
| GO:0042221\_response\_to\_chemical\_stimulus | NUP85 | 409 | 1 | 0.625509 | -0.089830 | 176 | 375.89 | 2.135739 |  |
| GO:0010556\_regulation\_of\_macromolecule\_biosynthetic\_process | CDKN1C | 745 | 2 | 0.686801 | -0.089450 | 177 | 376.15 | 2.125141 |  |
| GO:0010556\_regulation\_of\_macromolecule\_biosynthetic\_process | DNAJB6 | 745 | 2 | 0.686801 | -0.089450 | 177 | 376.15 | 2.125141 |  |
| GO:0019219\_regulation\_of\_nucleobase\_\_nucleoside\_\_nucleotide\_and\_nucleic\_acid\_metabolic\_process | CDKN1C | 757 | 2 | 0.675914 | -0.085451 | 178 | 377.48 | 2.120674 |  |
| GO:0019219\_regulation\_of\_nucleobase\_\_nucleoside\_\_nucleotide\_and\_nucleic\_acid\_metabolic\_process | DNAJB6 | 757 | 2 | 0.675914 | -0.085451 | 178 | 377.48 | 2.120674 |  |
| GO:0044238\_primary\_metabolic\_process | SOAT1 | 1905 | 6 | 0.805774 | -0.084098 | 179 | 377.61 | 2.109553 |  |
| GO:0044238\_primary\_metabolic\_process | CDKN1C | 1905 | 6 | 0.805774 | -0.084098 | 179 | 377.61 | 2.109553 |  |
| GO:0044238\_primary\_metabolic\_process | NUDT5 | 1905 | 6 | 0.805774 | -0.084098 | 179 | 377.61 | 2.109553 |  |
| GO:0044238\_primary\_metabolic\_process | MLL3 | 1905 | 6 | 0.805774 | -0.084098 | 179 | 377.61 | 2.109553 |  |
| GO:0044238\_primary\_metabolic\_process | DNAJB6 | 1905 | 6 | 0.805774 | -0.084098 | 179 | 377.61 | 2.109553 |  |
| GO:0044238\_primary\_metabolic\_process | SLBP | 1905 | 6 | 0.805774 | -0.084098 | 179 | 377.61 | 2.109553 |  |
| GO:0022008\_neurogenesis | CDKN1C | 423 | 1 | 0.604807 | -0.084031 | 180 | 377.83 | 2.099056 |  |
| GO:0051171\_regulation\_of\_nitrogen\_compound\_metabolic\_process | CDKN1C | 771 | 2 | 0.663640 | -0.080999 | 181 | 378.58 | 2.091602 |  |
| GO:0051171\_regulation\_of\_nitrogen\_compound\_metabolic\_process | DNAJB6 | 771 | 2 | 0.663640 | -0.080999 | 181 | 378.58 | 2.091602 |  |
| GO:0006357\_regulation\_of\_transcription\_from\_RNA\_polymerase\_II\_promoter | CDKN1C | 435 | 1 | 0.588123 | -0.079373 | 182 | 379.98 | 2.087802 |  |
| GO:0010468\_regulation\_of\_gene\_expression | CDKN1C | 778 | 2 | 0.657669 | -0.078855 | 183 | 380.26 | 2.077923 |  |
| GO:0010468\_regulation\_of\_gene\_expression | DNAJB6 | 778 | 2 | 0.657669 | -0.078855 | 183 | 380.26 | 2.077923 |  |
| GO:0006464\_protein\_modification\_process | MLL3 | 439 | 1 | 0.582764 | -0.077881 | 184 | 380.75 | 2.069293 |  |
| GO:0006366\_transcription\_from\_RNA\_polymerase\_II\_promoter | CDKN1C | 444 | 1 | 0.576201 | -0.076057 | 185 | 381.99 | 2.064811 |  |
| GO:0010926\_anatomical\_structure\_formation | NUP85 | 447 | 1 | 0.572334 | -0.074984 | 186 | 382.32 | 2.055484 |  |
| GO:0034960\_cellular\_biopolymer\_metabolic\_process | CDKN1C | 1395 | 4 | 0.733572 | -0.074465 | 187 | 382.44 | 2.045134 |  |
| GO:0034960\_cellular\_biopolymer\_metabolic\_process | MLL3 | 1395 | 4 | 0.733572 | -0.074465 | 187 | 382.44 | 2.045134 |  |
| GO:0034960\_cellular\_biopolymer\_metabolic\_process | DNAJB6 | 1395 | 4 | 0.733572 | -0.074465 | 187 | 382.44 | 2.045134 |  |
| GO:0034960\_cellular\_biopolymer\_metabolic\_process | SLBP | 1395 | 4 | 0.733572 | -0.074465 | 187 | 382.44 | 2.045134 |  |
| GO:0034961\_cellular\_biopolymer\_biosynthetic\_process | CDKN1C | 804 | 2 | 0.636401 | -0.071350 | 188 | 383.71 | 2.041011 |  |
| GO:0034961\_cellular\_biopolymer\_biosynthetic\_process | DNAJB6 | 804 | 2 | 0.636401 | -0.071350 | 188 | 383.71 | 2.041011 |  |
| GO:0043412\_biopolymer\_modification | MLL3 | 458 | 1 | 0.558588 | -0.071182 | 189 | 384.31 | 2.033386 |  |
| GO:0043284\_biopolymer\_biosynthetic\_process | CDKN1C | 807 | 2 | 0.634036 | -0.070528 | 190 | 384.55 | 2.023947 |  |
| GO:0043284\_biopolymer\_biosynthetic\_process | DNAJB6 | 807 | 2 | 0.634036 | -0.070528 | 190 | 384.55 | 2.023947 |  |
| GO:0031326\_regulation\_of\_cellular\_biosynthetic\_process | CDKN1C | 812 | 2 | 0.630131 | -0.069179 | 191 | 384.91 | 2.015236 |  |
| GO:0031326\_regulation\_of\_cellular\_biosynthetic\_process | DNAJB6 | 812 | 2 | 0.630131 | -0.069179 | 191 | 384.91 | 2.015236 |  |
| GO:0009889\_regulation\_of\_biosynthetic\_process | CDKN1C | 815 | 2 | 0.627812 | -0.068380 | 192 | 385.11 | 2.005781 |  |
| GO:0009889\_regulation\_of\_biosynthetic\_process | DNAJB6 | 815 | 2 | 0.627812 | -0.068380 | 192 | 385.11 | 2.005781 |  |
| GO:0044237\_cellular\_metabolic\_process | SOAT1 | 1974 | 6 | 0.777609 | -0.067915 | 193 | 385.27 | 1.996218 |  |
| GO:0044237\_cellular\_metabolic\_process | CDKN1C | 1974 | 6 | 0.777609 | -0.067915 | 193 | 385.27 | 1.996218 |  |
| GO:0044237\_cellular\_metabolic\_process | NUDT5 | 1974 | 6 | 0.777609 | -0.067915 | 193 | 385.27 | 1.996218 |  |
| GO:0044237\_cellular\_metabolic\_process | MLL3 | 1974 | 6 | 0.777609 | -0.067915 | 193 | 385.27 | 1.996218 |  |
| GO:0044237\_cellular\_metabolic\_process | DNAJB6 | 1974 | 6 | 0.777609 | -0.067915 | 193 | 385.27 | 1.996218 |  |
| GO:0044237\_cellular\_metabolic\_process | SLBP | 1974 | 6 | 0.777609 | -0.067915 | 193 | 385.27 | 1.996218 |  |
| GO:0044249\_cellular\_biosynthetic\_process | CDKN1C | 1150 | 3 | 0.667391 | -0.063165 | 194 | 385.74 | 1.988351 |  |
| GO:0044249\_cellular\_biosynthetic\_process | SOAT1 | 1150 | 3 | 0.667391 | -0.063165 | 194 | 385.74 | 1.988351 |  |
| GO:0044249\_cellular\_biosynthetic\_process | DNAJB6 | 1150 | 3 | 0.667391 | -0.063165 | 194 | 385.74 | 1.988351 |  |
| GO:0044260\_cellular\_macromolecule\_metabolic\_process | CDKN1C | 1447 | 4 | 0.707210 | -0.062683 | 195 | 385.86 | 1.978769 |  |
| GO:0044260\_cellular\_macromolecule\_metabolic\_process | MLL3 | 1447 | 4 | 0.707210 | -0.062683 | 195 | 385.86 | 1.978769 |  |
| GO:0044260\_cellular\_macromolecule\_metabolic\_process | DNAJB6 | 1447 | 4 | 0.707210 | -0.062683 | 195 | 385.86 | 1.978769 |  |
| GO:0044260\_cellular\_macromolecule\_metabolic\_process | SLBP | 1447 | 4 | 0.707210 | -0.062683 | 195 | 385.86 | 1.978769 |  |
| GO:0009058\_biosynthetic\_process | SOAT1 | 1175 | 3 | 0.653191 | -0.057791 | 196 | 386.08 | 1.969796 |  |
| GO:0009058\_biosynthetic\_process | CDKN1C | 1175 | 3 | 0.653191 | -0.057791 | 196 | 386.08 | 1.969796 |  |
| GO:0009058\_biosynthetic\_process | DNAJB6 | 1175 | 3 | 0.653191 | -0.057791 | 196 | 386.08 | 1.969796 |  |
| GO:0043283\_biopolymer\_metabolic\_process | CDKN1C | 1490 | 4 | 0.686801 | -0.054175 | 197 | 386.77 | 1.963299 |  |
| GO:0043283\_biopolymer\_metabolic\_process | MLL3 | 1490 | 4 | 0.686801 | -0.054175 | 197 | 386.77 | 1.963299 |  |
| GO:0043283\_biopolymer\_metabolic\_process | DNAJB6 | 1490 | 4 | 0.686801 | -0.054175 | 197 | 386.77 | 1.963299 |  |
| GO:0043283\_biopolymer\_metabolic\_process | SLBP | 1490 | 4 | 0.686801 | -0.054175 | 197 | 386.77 | 1.963299 |  |
| GO:0032502\_developmental\_process | CDKN1C | 2060 | 6 | 0.745146 | -0.051336 | 198 | 387.43 | 1.956717 |  |
| GO:0032502\_developmental\_process | WNT5B | 2060 | 6 | 0.745146 | -0.051336 | 198 | 387.43 | 1.956717 |  |
| GO:0032502\_developmental\_process | PICALM | 2060 | 6 | 0.745146 | -0.051336 | 198 | 387.43 | 1.956717 |  |
| GO:0032502\_developmental\_process | IGF2 | 2060 | 6 | 0.745146 | -0.051336 | 198 | 387.43 | 1.956717 |  |
| GO:0032502\_developmental\_process | SFXN1 | 2060 | 6 | 0.745146 | -0.051336 | 198 | 387.43 | 1.956717 |  |
| GO:0032502\_developmental\_process | DNAJB6 | 2060 | 6 | 0.745146 | -0.051336 | 198 | 387.43 | 1.956717 |  |
| GO:0034645\_cellular\_macromolecule\_biosynthetic\_process | CDKN1C | 901 | 2 | 0.567888 | -0.048830 | 199 | 387.76 | 1.948543 |  |
| GO:0034645\_cellular\_macromolecule\_biosynthetic\_process | DNAJB6 | 901 | 2 | 0.567888 | -0.048830 | 199 | 387.76 | 1.948543 |  |
| GO:0008283\_cell\_proliferation | E2F8 | 544 | 1 | 0.470282 | -0.047494 | 200 | 388.31 | 1.941550 |  |
| GO:0009059\_macromolecule\_biosynthetic\_process | CDKN1C | 910 | 2 | 0.562271 | -0.047116 | 201 | 388.51 | 1.932886 |  |
| GO:0009059\_macromolecule\_biosynthetic\_process | DNAJB6 | 910 | 2 | 0.562271 | -0.047116 | 201 | 388.51 | 1.932886 |  |
| GO:0044267\_cellular\_protein\_metabolic\_process | MLL3 | 559 | 1 | 0.457662 | -0.044265 | 202 | 389.13 | 1.926386 |  |
| GO:0080090\_regulation\_of\_primary\_metabolic\_process | CDKN1C | 926 | 2 | 0.552556 | -0.044206 | 203 | 389.29 | 1.917685 |  |
| GO:0080090\_regulation\_of\_primary\_metabolic\_process | DNAJB6 | 926 | 2 | 0.552556 | -0.044206 | 203 | 389.29 | 1.917685 |  |
| GO:0009790\_embryonic\_development | DNAJB6 | 567 | 1 | 0.451205 | -0.042634 | 204 | 389.5 | 1.909314 |  |
| GO:0060255\_regulation\_of\_macromolecule\_metabolic\_process | CDKN1C | 936 | 2 | 0.546652 | -0.042472 | 205 | 389.71 | 1.901024 |  |
| GO:0060255\_regulation\_of\_macromolecule\_metabolic\_process | DNAJB6 | 936 | 2 | 0.546652 | -0.042472 | 205 | 389.71 | 1.901024 |  |
| GO:0043170\_macromolecule\_metabolic\_process | CDKN1C | 1576 | 4 | 0.649323 | -0.040070 | 206 | 390.08 | 1.893592 |  |
| GO:0043170\_macromolecule\_metabolic\_process | MLL3 | 1576 | 4 | 0.649323 | -0.040070 | 206 | 390.08 | 1.893592 |  |
| GO:0043170\_macromolecule\_metabolic\_process | DNAJB6 | 1576 | 4 | 0.649323 | -0.040070 | 206 | 390.08 | 1.893592 |  |
| GO:0043170\_macromolecule\_metabolic\_process | SLBP | 1576 | 4 | 0.649323 | -0.040070 | 206 | 390.08 | 1.893592 |  |
| GO:0008152\_metabolic\_process | CDKN1C | 2133 | 6 | 0.719644 | -0.039973 | 207 | 390.18 | 1.884928 |  |
| GO:0008152\_metabolic\_process | SOAT1 | 2133 | 6 | 0.719644 | -0.039973 | 207 | 390.18 | 1.884928 |  |
| GO:0008152\_metabolic\_process | NUDT5 | 2133 | 6 | 0.719644 | -0.039973 | 207 | 390.18 | 1.884928 |  |
| GO:0008152\_metabolic\_process | MLL3 | 2133 | 6 | 0.719644 | -0.039973 | 207 | 390.18 | 1.884928 |  |
| GO:0008152\_metabolic\_process | DNAJB6 | 2133 | 6 | 0.719644 | -0.039973 | 207 | 390.18 | 1.884928 |  |
| GO:0008152\_metabolic\_process | SLBP | 2133 | 6 | 0.719644 | -0.039973 | 207 | 390.18 | 1.884928 |  |
| GO:0032501\_multicellular\_organismal\_process | CDKN1C | 2183 | 6 | 0.703161 | -0.033437 | 208 | 392.09 | 1.885048 |  |
| GO:0032501\_multicellular\_organismal\_process | WNT5B | 2183 | 6 | 0.703161 | -0.033437 | 208 | 392.09 | 1.885048 |  |
| GO:0032501\_multicellular\_organismal\_process | PICALM | 2183 | 6 | 0.703161 | -0.033437 | 208 | 392.09 | 1.885048 |  |
| GO:0032501\_multicellular\_organismal\_process | IGF2 | 2183 | 6 | 0.703161 | -0.033437 | 208 | 392.09 | 1.885048 |  |
| GO:0032501\_multicellular\_organismal\_process | SFXN1 | 2183 | 6 | 0.703161 | -0.033437 | 208 | 392.09 | 1.885048 |  |
| GO:0032501\_multicellular\_organismal\_process | DNAJB6 | 2183 | 6 | 0.703161 | -0.033437 | 208 | 392.09 | 1.885048 |  |
| GO:0007399\_nervous\_system\_development | CDKN1C | 621 | 1 | 0.411970 | -0.033081 | 209 | 392.29 | 1.876986 |  |
| GO:0031323\_regulation\_of\_cellular\_metabolic\_process | CDKN1C | 1015 | 2 | 0.504105 | -0.030822 | 210 | 392.68 | 1.869905 |  |
| GO:0031323\_regulation\_of\_cellular\_metabolic\_process | DNAJB6 | 1015 | 2 | 0.504105 | -0.030822 | 210 | 392.68 | 1.869905 |  |
| GO:0048468\_cell\_development | CDKN1C | 654 | 1 | 0.391182 | -0.028318 | 211 | 393.14 | 1.863223 |  |
| GO:0019538\_protein\_metabolic\_process | MLL3 | 655 | 1 | 0.390585 | -0.028185 | 212 | 393.28 | 1.855094 |  |
| GO:0030154\_cell\_differentiation | CDKN1C | 1060 | 2 | 0.482704 | -0.025579 | 213 | 393.84 | 1.849014 |  |
| GO:0030154\_cell\_differentiation | SFXN1 | 1060 | 2 | 0.482704 | -0.025579 | 213 | 393.84 | 1.849014 |  |
| GO:0019222\_regulation\_of\_metabolic\_process | CDKN1C | 1088 | 2 | 0.470282 | -0.022743 | 214 | 394.67 | 1.844252 |  |
| GO:0019222\_regulation\_of\_metabolic\_process | DNAJB6 | 1088 | 2 | 0.470282 | -0.022743 | 214 | 394.67 | 1.844252 |  |
| GO:0048869\_cellular\_developmental\_process | CDKN1C | 1113 | 2 | 0.459718 | -0.020457 | 215 | 395.63 | 1.840140 |  |
| GO:0048869\_cellular\_developmental\_process | SFXN1 | 1113 | 2 | 0.459718 | -0.020457 | 215 | 395.63 | 1.840140 |  |
| GO:0050794\_regulation\_of\_cellular\_process | CDKN1C | 2190 | 5 | 0.584094 | -0.011074 | 216 | 398.02 | 1.842685 |  |
| GO:0050794\_regulation\_of\_cellular\_process | WNT5B | 2190 | 5 | 0.584094 | -0.011074 | 216 | 398.02 | 1.842685 |  |
| GO:0050794\_regulation\_of\_cellular\_process | TRO | 2190 | 5 | 0.584094 | -0.011074 | 216 | 398.02 | 1.842685 |  |
| GO:0050794\_regulation\_of\_cellular\_process | APEX1 | 2190 | 5 | 0.584094 | -0.011074 | 216 | 398.02 | 1.842685 |  |
| GO:0050794\_regulation\_of\_cellular\_process | DNAJB6 | 2190 | 5 | 0.584094 | -0.011074 | 216 | 398.02 | 1.842685 |  |
| GO:0007165\_signal\_transduction | WNT5B | 915 | 1 | 0.279599 | -0.008064 | 217 | 398.76 | 1.837604 |  |
| GO:0065007\_biological\_regulation | CDKN1C | 2593 | 6 | 0.591978 | -0.005959 | 218 | 399.23 | 1.831330 |  |
| GO:0065007\_biological\_regulation | WNT5B | 2593 | 6 | 0.591978 | -0.005959 | 218 | 399.23 | 1.831330 |  |
| GO:0065007\_biological\_regulation | TRO | 2593 | 6 | 0.591978 | -0.005959 | 218 | 399.23 | 1.831330 |  |
| GO:0065007\_biological\_regulation | SFXN1 | 2593 | 6 | 0.591978 | -0.005959 | 218 | 399.23 | 1.831330 |  |
| GO:0065007\_biological\_regulation | APEX1 | 2593 | 6 | 0.591978 | -0.005959 | 218 | 399.23 | 1.831330 |  |
| GO:0065007\_biological\_regulation | DNAJB6 | 2593 | 6 | 0.591978 | -0.005959 | 218 | 399.23 | 1.831330 |  |
| GO:0050789\_regulation\_of\_biological\_process | CDKN1C | 2357 | 5 | 0.542710 | -0.005168 | 219 | 399.49 | 1.824155 |  |
| GO:0050789\_regulation\_of\_biological\_process | WNT5B | 2357 | 5 | 0.542710 | -0.005168 | 219 | 399.49 | 1.824155 |  |
| GO:0050789\_regulation\_of\_biological\_process | TRO | 2357 | 5 | 0.542710 | -0.005168 | 219 | 399.49 | 1.824155 |  |
| GO:0050789\_regulation\_of\_biological\_process | APEX1 | 2357 | 5 | 0.542710 | -0.005168 | 219 | 399.49 | 1.824155 |  |
| GO:0050789\_regulation\_of\_biological\_process | DNAJB6 | 2357 | 5 | 0.542710 | -0.005168 | 219 | 399.49 | 1.824155 |  |
| GO:0007154\_cell\_communication | WNT5B | 1096 | 1 | 0.233425 | -0.003236 | 220 | 399.85 | 1.817500 |  |
| GO:0050896\_response\_to\_stimulus | NUP85 | 1107 | 1 | 0.231105 | -0.003057 | 221 | 399.89 | 1.809457 |  |
| GO:0008150\_biological\_process | ARL6IP1 | 4605 | 18 | 1.000000 | 0.000000 | 1856 | 1898.19 | 1.022732 |  |
| GO:0008150\_biological\_process | SOAT1 | 4605 | 18 | 1.000000 | 0.000000 | 1856 | 1898.19 | 1.022732 |  |
| GO:0008150\_biological\_process | WNT5B | 4605 | 18 | 1.000000 | 0.000000 | 1856 | 1898.19 | 1.022732 |  |
| GO:0008150\_biological\_process | NEK2 | 4605 | 18 | 1.000000 | 0.000000 | 1856 | 1898.19 | 1.022732 |  |
| GO:0008150\_biological\_process | E2F8 | 4605 | 18 | 1.000000 | 0.000000 | 1856 | 1898.19 | 1.022732 |  |
| GO:0008150\_biological\_process | NUDT5 | 4605 | 18 | 1.000000 | 0.000000 | 1856 | 1898.19 | 1.022732 |  |
| GO:0008150\_biological\_process | IGF2 | 4605 | 18 | 1.000000 | 0.000000 | 1856 | 1898.19 | 1.022732 |  |
| GO:0008150\_biological\_process | SFXN1 | 4605 | 18 | 1.000000 | 0.000000 | 1856 | 1898.19 | 1.022732 |  |
| GO:0008150\_biological\_process | NUP85 | 4605 | 18 | 1.000000 | 0.000000 | 1856 | 1898.19 | 1.022732 |  |
| GO:0008150\_biological\_process | SLBP | 4605 | 18 | 1.000000 | 0.000000 | 1856 | 1898.19 | 1.022732 |  |
| GO:0008150\_biological\_process | CDKN1C | 4605 | 18 | 1.000000 | 0.000000 | 1856 | 1898.19 | 1.022732 |  |
| GO:0008150\_biological\_process | PICALM | 4605 | 18 | 1.000000 | 0.000000 | 1856 | 1898.19 | 1.022732 |  |
| GO:0008150\_biological\_process | TRO | 4605 | 18 | 1.000000 | 0.000000 | 1856 | 1898.19 | 1.022732 |  |
| GO:0008150\_biological\_process | KPNA4 | 4605 | 18 | 1.000000 | 0.000000 | 1856 | 1898.19 | 1.022732 |  |
| GO:0008150\_biological\_process | MLL3 | 4605 | 18 | 1.000000 | 0.000000 | 1856 | 1898.19 | 1.022732 |  |
| GO:0008150\_biological\_process | PES1 | 4605 | 18 | 1.000000 | 0.000000 | 1856 | 1898.19 | 1.022732 |  |
| GO:0008150\_biological\_process | APEX1 | 4605 | 18 | 1.000000 | 0.000000 | 1856 | 1898.19 | 1.022732 |  |
| GO:0008150\_biological\_process | DNAJB6 | 4605 | 18 | 1.000000 | 0.000000 | 1856 | 1898.19 | 1.022732 |  |
