# Supplementary material for: RedundancyMiner: De-replication of redundant GO categories in microarray and proteomics analysis
Source: BMC Bioinformatics. 2011 Feb 10;12:52. doi: 10.1186/1471-2105-12-52 (PMC3223614; doi:10.1186/1471-2105-12-52)
Supplement: Additional file 8 — Retinal development HTGM download. compressed package of the results of running HTGM on the retinal development genes list. [file 1471-2105-12-52-S8.ZIP › SCENARIO_2_MODIFIED/total.txt.total.txt.dir/index.html]

Results - total.txt.total.txt

# Results - total.txt.total.txt

| Integrative Results | | |
| --- | --- | --- |
| Type | File | Description |
| Integrative Category Report | total.txt.total.txt.report | List of the number of significantly changed categories in each changed genes file |
| Total Versus Total Report | total.txt.total.txt.total.tvt | GO category mapping of all genes in total file |
| Clustered Image Map(CIM) data: Significant categories versus genes in those categories | | || Integrative CIM Data | total.txt.total.txt.change.series.CIM | Matrix of significant categories for changed genes against those genes |
| CIM images clustered by category and by experiment (does not preserve original experiment ordering) | | || Integrative CIM Image | total.txt.total.txt.change.series.CIM.html | Significant categories for changed genes |
| CIM images clustered by category only (use if input files are already ordered, e.g. time course): | | || Integrative CIM Image | total.txt.total.txt.change.series.CIM.html | Significant categories for changed genes |
| Instructions for tuning color on Integrative CIM Images: | | || The default color scheme and binning approach for the above images may not be ideal. We suggest after viewing the CIM Image, select the Color button, and set the color range described here, and select Asymetric interval for the binning method.  You can read more about tuning the colors of the CIM Images for the HTGM result files.  The re-coloring features are not currently operational if you are viewing the CIM Image from the downloaded archive. If you want to do this color tuning step, you can either do so from the original online URL if it is still available, or regenerate the CIM your self by submitting the CIM Data file, and follow these generation instructions. | | || --- | | ||| Results for Each Changed File | | |
| --- | --- | --- |
| Exp1\_BestClusterMap\_LEIGS\_KM\_24.csv.join.1.txt.dir/ | | |  |
| Exp1\_BestClusterMap\_LEIGS\_KM\_24.csv.join.2.txt.dir/ | | |  |
| Exp1\_BestClusterMap\_LEIGS\_KM\_24.csv.join.3.txt.dir/ | | |  |
| Exp1\_BestClusterMap\_LEIGS\_KM\_24.csv.join.4.txt.dir/ | | |  |
| Exp1\_BestClusterMap\_LEIGS\_KM\_24.csv.join.5.txt.dir/ | | |  |
| Exp1\_BestClusterMap\_LEIGS\_KM\_24.csv.join.6.txt.dir/ | | |  |
| Exp1\_BestClusterMap\_LEIGS\_KM\_24.csv.join.7.txt.dir/ | | |  |
| Exp1\_BestClusterMap\_LEIGS\_KM\_24.csv.join.8.txt.dir/ | | |  |
| Exp1\_BestClusterMap\_LEIGS\_KM\_24.csv.join.9.txt.dir/ | | |  |
| Exp1\_BestClusterMap\_LEIGS\_KM\_24.csv.join.10.txt.dir/ | | |  |
| Exp1\_BestClusterMap\_LEIGS\_KM\_24.csv.join.11.txt.dir/ | | |  |
| Exp1\_BestClusterMap\_LEIGS\_KM\_24.csv.join.12.txt.dir/ | | |  |
| Exp1\_BestClusterMap\_LEIGS\_KM\_24.csv.join.14.txt.dir/ | | |  |
| Exp1\_BestClusterMap\_LEIGS\_KM\_24.csv.join.15.txt.dir/ | | |  |
| Exp1\_BestClusterMap\_LEIGS\_KM\_24.csv.join.16.txt.dir/ | | |  |
| Exp1\_BestClusterMap\_LEIGS\_KM\_24.csv.join.17.txt.dir/ | | |  |
| Exp1\_BestClusterMap\_LEIGS\_KM\_24.csv.join.18.txt.dir/ | | |  |
| Exp1\_BestClusterMap\_LEIGS\_KM\_24.csv.join.19.txt.dir/ | | |  |
| Exp1\_BestClusterMap\_LEIGS\_KM\_24.csv.join.20.txt.dir/ | | |  |
| Exp1\_BestClusterMap\_LEIGS\_KM\_24.csv.join.21.txt.dir/ | | |  |
| Exp1\_BestClusterMap\_LEIGS\_KM\_24.csv.join.22.txt.dir/ | | |  |
| Exp1\_BestClusterMap\_LEIGS\_KM\_24.csv.join.23.txt.dir/ | | |  |
| Exp1\_BestClusterMap\_LEIGS\_KM\_24.csv.join.24.txt.dir/ | | |  |
| --- | | ||| Navigation | | |
| --- | --- | --- |
| Up to Parent(HTGM Job Summary) | | |
