# Supplementary material for: RedundancyMiner: De-replication of redundant GO categories in microarray and proteomics analysis
Source: BMC Bioinformatics. 2011 Feb 10;12:52. doi: 10.1186/1471-2105-12-52 (PMC3223614; doi:10.1186/1471-2105-12-52)
Supplement: Additional file 8 — Retinal development HTGM download. compressed package of the results of running HTGM on the retinal development genes list. [file 1471-2105-12-52-S8.ZIP › SCENARIO_2_MODIFIED/total.txt.total.txt.dir/total.txt.total.txt.change.series.CIM.1.dir/cgi_user_x.html]

**X-axis Names**   
Cluster is based on euclidean distance  
Cluster method is: average  
plclust  
height plot  

1.GO:0007417\_central\_nervous\_system\_development

2.GO:0007507\_heart\_development

3.GO:0060840\_artery\_development

4.GO:0048844\_artery\_morphogenesis

5.GO:0003018\_vascular\_process\_in\_circulatory\_system

6.GO:0035150\_regulation\_of\_tube\_size

7.GO:0050880\_regulation\_of\_blood\_vessel\_size

8.GO:0007420\_brain\_development

9.GO:0045785\_positive\_regulation\_of\_cell\_adhesion

10.GO:0006022\_aminoglycan\_metabolic\_process

11.GO:0030203\_glycosaminoglycan\_metabolic\_process

12.GO:0006887\_exocytosis

13.GO:0043583\_ear\_development

14.GO:0060113\_inner\_ear\_receptor\_cell\_differentiation

15.GO:0002011\_morphogenesis\_of\_an\_epithelial\_sheet

16.GO:0048839\_inner\_ear\_development

17.GO:0031122\_cytoplasmic\_microtubule\_organization

18.GO:0048489\_synaptic\_vesicle\_transport

19.GO:0030030\_cell\_projection\_organization

20.GO:0007411\_axon\_guidance

21.GO:0048666\_neuron\_development

22.GO:0010623\_developmental\_programmed\_cell\_death

23.GO:0040012\_regulation\_of\_locomotion

24.GO:0014047\_glutamate\_secretion

25.GO:0051272\_positive\_regulation\_of\_cell\_motion

26.GO:0001654\_eye\_development

27.GO:0048070\_regulation\_of\_pigmentation\_during\_development

28.GO:0048592\_eye\_morphogenesis

29.GO:0030335\_positive\_regulation\_of\_cell\_migration

30.GO:0030334\_regulation\_of\_cell\_migration

31.GO:0033033\_negative\_regulation\_of\_myeloid\_cell\_apoptosis

32.GO:0051402\_neuron\_apoptosis

33.GO:0042462\_eye\_photoreceptor\_cell\_development

34.GO:0050931\_pigment\_cell\_differentiation

35.GO:0001657\_ureteric\_bud\_development

36.GO:0030318\_melanocyte\_differentiation

37.GO:0040011\_locomotion

38.GO:0043524\_negative\_regulation\_of\_neuron\_apoptosis

39.GO:0046666\_retinal\_cell\_programmed\_cell\_death

40.GO:0051384\_response\_to\_glucocorticoid\_stimulus

41.GO:0031960\_response\_to\_corticosteroid\_stimulus

42.GO:0042461\_photoreceptor\_cell\_development

43.GO:0010464\_regulation\_of\_mesenchymal\_cell\_proliferation

44.GO:0010463\_mesenchymal\_cell\_proliferation

45.GO:0001569\_patterning\_of\_blood\_vessels

46.GO:0000082\_G1\_S\_transition\_of\_mitotic\_cell\_cycle

47.GO:0051270\_regulation\_of\_cell\_motion

48.GO:0001754\_eye\_photoreceptor\_cell\_differentiation

49.GO:0002053\_positive\_regulation\_of\_mesenchymal\_cell\_proliferation

50.GO:0046530\_photoreceptor\_cell\_differentiation

51.GO:0031102\_neuron\_projection\_regeneration

52.GO:0031103\_axon\_regeneration

53.GO:0007409\_axonogenesis

54.GO:0033032\_regulation\_of\_myeloid\_cell\_apoptosis

55.GO:0016477\_cell\_migration

56.GO:0001656\_metanephros\_development

57.GO:0045664\_regulation\_of\_neuron\_differentiation

58.GO:0031175\_neuron\_projection\_development

59.GO:0006275\_regulation\_of\_DNA\_replication

60.GO:0001945\_lymph\_vessel\_development

61.GO:0048598\_embryonic\_morphogenesis

62.GO:0048729\_tissue\_morphogenesis

63.GO:0022610\_biological\_adhesion

64.GO:0007155\_cell\_adhesion

65.GO:0048514\_blood\_vessel\_morphogenesis

66.GO:0045787\_positive\_regulation\_of\_cell\_cycle

67.GO:0001655\_urogenital\_system\_development

68.GO:0018149\_peptide\_cross-linking

69.GO:0045669\_positive\_regulation\_of\_osteoblast\_differentiation

70.GO:0050654\_chondroitin\_sulfate\_proteoglycan\_metabolic\_process

71.GO:0019800\_peptide\_cross-linking\_via\_chondroitin\_4-sulfate\_glycosaminoglycan

72.GO:0001501\_skeletal\_system\_development

73.GO:0030155\_regulation\_of\_cell\_adhesion

74.GO:0006916\_anti-apoptosis

75.GO:0010721\_negative\_regulation\_of\_cell\_development

76.GO:0045860\_positive\_regulation\_of\_protein\_kinase\_activity

77.GO:0007179\_transforming\_growth\_factor\_beta\_receptor\_signaling\_pathway

78.GO:0050767\_regulation\_of\_neurogenesis

79.GO:0046394\_carboxylic\_acid\_biosynthetic\_process

80.GO:0042493\_response\_to\_drug

81.GO:0016053\_organic\_acid\_biosynthetic\_process

82.GO:0031401\_positive\_regulation\_of\_protein\_modification\_process

83.GO:0048870\_cell\_motility

84.GO:0043523\_regulation\_of\_neuron\_apoptosis

85.GO:0051174\_regulation\_of\_phosphorus\_metabolic\_process

86.GO:0019220\_regulation\_of\_phosphate\_metabolic\_process

87.GO:0048812\_neuron\_projection\_morphogenesis

88.GO:0001934\_positive\_regulation\_of\_protein\_amino\_acid\_phosphorylation

89.GO:0048066\_pigmentation\_during\_development

90.GO:0048469\_cell\_maturation

91.GO:0042116\_macrophage\_activation

92.GO:0007006\_mitochondrial\_membrane\_organization

93.GO:0042325\_regulation\_of\_phosphorylation

94.GO:0042551\_neuron\_maturation

95.GO:0006637\_acyl-CoA\_metabolic\_process

96.GO:0048678\_response\_to\_axon\_injury

97.GO:0001952\_regulation\_of\_cell-matrix\_adhesion

98.GO:0042327\_positive\_regulation\_of\_phosphorylation

99.GO:0045937\_positive\_regulation\_of\_phosphate\_metabolic\_process

100.GO:0010562\_positive\_regulation\_of\_phosphorus\_metabolic\_process

101.GO:0048667\_cell\_morphogenesis\_involved\_in\_neuron\_differentiation

102.GO:0048858\_cell\_projection\_morphogenesis

103.GO:0033028\_myeloid\_cell\_apoptosis

104.GO:0007423\_sensory\_organ\_development

105.GO:0050768\_negative\_regulation\_of\_neurogenesis

106.GO:0042490\_mechanoreceptor\_differentiation

107.GO:0050769\_positive\_regulation\_of\_neurogenesis

108.GO:0008156\_negative\_regulation\_of\_DNA\_replication

109.GO:0060284\_regulation\_of\_cell\_development
