# Supplementary material for: RedundancyMiner: De-replication of redundant GO categories in microarray and proteomics analysis
Source: BMC Bioinformatics. 2011 Feb 10;12:52. doi: 10.1186/1471-2105-12-52 (PMC3223614; doi:10.1186/1471-2105-12-52)
Supplement: Additional file 8 — Retinal development HTGM download. compressed package of the results of running HTGM on the retinal development genes list. [file 1471-2105-12-52-S8.ZIP › SCENARIO_2_MODIFIED/total.txt.total.txt.dir/total.txt.total.txt.change.series.CIM.2.dir/cgi_user_y.html]

**Y-axis Names**   
Algorithm: none   

1.Exp1\_BestClusterMap\_LEIGS\_KM\_24.csv.join.1.txt

2.Exp1\_BestClusterMap\_LEIGS\_KM\_24.csv.join.2.txt

3.Exp1\_BestClusterMap\_LEIGS\_KM\_24.csv.join.3.txt

4.Exp1\_BestClusterMap\_LEIGS\_KM\_24.csv.join.4.txt

5.Exp1\_BestClusterMap\_LEIGS\_KM\_24.csv.join.5.txt

6.Exp1\_BestClusterMap\_LEIGS\_KM\_24.csv.join.6.txt

7.Exp1\_BestClusterMap\_LEIGS\_KM\_24.csv.join.7.txt

8.Exp1\_BestClusterMap\_LEIGS\_KM\_24.csv.join.8.txt

9.Exp1\_BestClusterMap\_LEIGS\_KM\_24.csv.join.9.txt

10.Exp1\_BestClusterMap\_LEIGS\_KM\_24.csv.join.10.txt

11.Exp1\_BestClusterMap\_LEIGS\_KM\_24.csv.join.11.txt

12.Exp1\_BestClusterMap\_LEIGS\_KM\_24.csv.join.12.txt

13.Exp1\_BestClusterMap\_LEIGS\_KM\_24.csv.join.14.txt

14.Exp1\_BestClusterMap\_LEIGS\_KM\_24.csv.join.15.txt

15.Exp1\_BestClusterMap\_LEIGS\_KM\_24.csv.join.16.txt

16.Exp1\_BestClusterMap\_LEIGS\_KM\_24.csv.join.17.txt

17.Exp1\_BestClusterMap\_LEIGS\_KM\_24.csv.join.18.txt

18.Exp1\_BestClusterMap\_LEIGS\_KM\_24.csv.join.19.txt

19.Exp1\_BestClusterMap\_LEIGS\_KM\_24.csv.join.20.txt

20.Exp1\_BestClusterMap\_LEIGS\_KM\_24.csv.join.21.txt

21.Exp1\_BestClusterMap\_LEIGS\_KM\_24.csv.join.22.txt

22.Exp1\_BestClusterMap\_LEIGS\_KM\_24.csv.join.23.txt

23.Exp1\_BestClusterMap\_LEIGS\_KM\_24.csv.join.24.txt
